# Genome-Wide Analyses Suggest Mechanisms Involving Early B-Cell Development in Canine IgA Deficiency

**DOI:** 10.1371/journal.pone.0133844

**Published:** 2015-07-30

**Authors:** Mia Olsson, Katarina Tengvall, Marcel Frankowiack, Marcin Kierczak, Kerstin Bergvall, Erik Axelsson, Linda Tintle, Eliane Marti, Petra Roosje, Tosso Leeb, Åke Hedhammar, Lennart Hammarström, Kerstin Lindblad-Toh

**Affiliations:** 1 Department of Laboratory Medicine, Division of Clinical Immunology, Karolinska Institute at Karolinska University Hospital, Huddinge, Sweden; 2 Science for Life Laboratory, Department of Medical Biochemistry and Microbiology, Uppsala University, Uppsala, Sweden; 3 Department of Clinical Sciences, Swedish University of Agricultural Sciences, Uppsala, Sweden; 4 Wurtsboro Veterinary Clinic, Wurtsboro, New York, United States of America; 5 Department of Clinical Veterinary Medicine, Division of Clinical Dermatology, Vetsuisse Faculty, University of Bern, Bern, Switzerland; 6 Dermfocus, Vetsuisse Faculty, University of Bern, Bern Switzerland; 7 Department of Clinical Research and Veterinary Public Health, Vetsuisse Faculty, University of Bern, Bern, Switzerland; 8 Institute of Genetics, Vetsuisse Faculty, University of Bern, Bern, Switzerland; 9 Broad Institute of MIT and Harvard, Cambridge, Massachusetts, United States of America; Stanford University School of Medicine, UNITED STATES

## Abstract

Immunoglobulin A deficiency (IgAD) is the most common primary immune deficiency disorder in both humans and dogs, characterized by recurrent mucosal tract infections and a predisposition for allergic and other immune mediated diseases. In several dog breeds, low IgA levels have been observed at a high frequency and with a clinical resemblance to human IgAD. In this study, we used genome-wide association studies (GWAS) to identify genomic regions associated with low IgA levels in dogs as a comparative model for human IgAD. We used a novel percentile groups-approach to establish breed-specific cut-offs and to perform analyses in a close to continuous manner. GWAS performed in four breeds prone to low IgA levels (German shepherd, Golden retriever, Labrador retriever and Shar-Pei) identified 35 genomic loci suggestively associated (p <0.0005) to IgA levels. In German shepherd, three genomic regions (candidate genes include *KIRREL3 *and *SERPINA9*) were genome-wide significantly associated (p <0.0002) with IgA levels. A ~20kb long haplotype on CFA28, significantly associated (p = 0.0005) to IgA levels in Shar-Pei, was positioned within the first intron of the gene *SLIT1*. Both *KIRREL3 *and *SLIT1* are highly expressed in the central nervous system and in bone marrow and are potentially important during B-cell development. *SERPINA9* expression is restricted to B-cells and peaks at the time-point when B-cells proliferate into antibody-producing plasma cells. The suggestively associated regions were enriched for genes in Gene Ontology gene sets involving inflammation and early immune cell development.

## Introduction

Immunoglobulin A (IgA) is the second most abundant antibody in human serum, a key immunoglobulin in mucosal defence (secretory IgA) and a trigger of effector functions (serum IgA) of the immune system [1, 2]. In Caucasians, IgA deficiency (IgAD) is the most common primary immunodeficiency disorder and affects about 1 in 600 individuals in the general population [3]. Many individuals with IgAD are asymptomatic but approximately 1/3 suffer from recurrent infections at mucosal sites as well as from autoimmune diseases [4–6]. Human IgAD is a complex disorder presumably influenced by several, yet unknown, genetic alterations. The α1 and α2 genes, encoding the heavy and the light chains of IgA, are expressed and functional in IgAD patients. No mutations have been identified within the coding regions of these genes [7], suggesting a regulatory defect in IgA production or secretion. Similar to many other immune-mediated diseases, there is a strong correlation between IgAD and genes of the MHC region [8]. Also non-MHC genes have previously been shown to be associated with human IgAD including the interferon induced with helicase C domain 1 (*IFIH1*) and C-type lectin domain family 10, member A (*CLEC16A*) genes [9], but no causative mutations have been identified.

Although a number of murine models for IgAD exist where the phenotype is genetically or experimentally induced, none of them resemble the human disease [3]. It is recognized that low concentrations, or even overt deficiency of IgA is present in several dog breeds including the Shar-Pei [10, 11], selected populations of Beagles [12, 13] and German Shepherds [14]. Just like humans, dogs with low IgA levels suffer from recurrent infections and immune-related/mediated health problems [13, 15–17].

Apart from humans, the domestic dog is the species with the largest characterized collection of naturally occurring genetic diseases, of which more than 350 have been described to clinically resemble the corresponding human disease [18]. Gene mapping in dogs has proven to be very successful and the list of disease-causing genes that have been identified in dogs is constantly growing (some are reviewed in [19]). Complex diseases are problematic to study even in dogs. However, a recent study of canine osteosarcoma presented novel approaches by using genome-wide analyses to unravel pathways and genes involved in this highly polygenic disease [20].

Although some breeds show low IgA levels, no underlying genetic risk factor has as yet been described in dogs. A challenge when studying canine IgAD is the lack of an accepted cut-off value for defining abnormally low serum IgA levels. In a previous study, we performed the largest serum IgA screen in dogs to date (~1300 dogs) representing 22 breeds [15, 17]. In that study we observed that the IgA ranges varied extensively between the different breeds, which could partly explain the failure to establish a general canine IgAD cut-off. In the present study, we used genome-wide SNP data to perform genome-wide association studies (GWAS) in four breeds prone to low IgA levels according to our previous study [15]; German shepherd (GSD), Golden retriever (GR), Labrador retriever (LR) and Shar-Pei (SP), aiming to identify new loci involved in IgAD. In order to handle a phenotype based solely on a continuous and fluctuating variable with undefined cut-offs in GWAS, we formulated a sliding window-approach using percentile groups. Following GWAS, we used IgA associated regions from all four breeds and performed pathway analyses to identify biological pathways and genes of importance for IgAD. We identified promising candidate genes and pathways to explain IgAD in dogs, involving early B-cell development, proliferation, class-switching and inflammation.

## Results

### IgA levels correlate with age in all breeds and with canine atopic dermatitis in German shepherd

**Fig 1:**
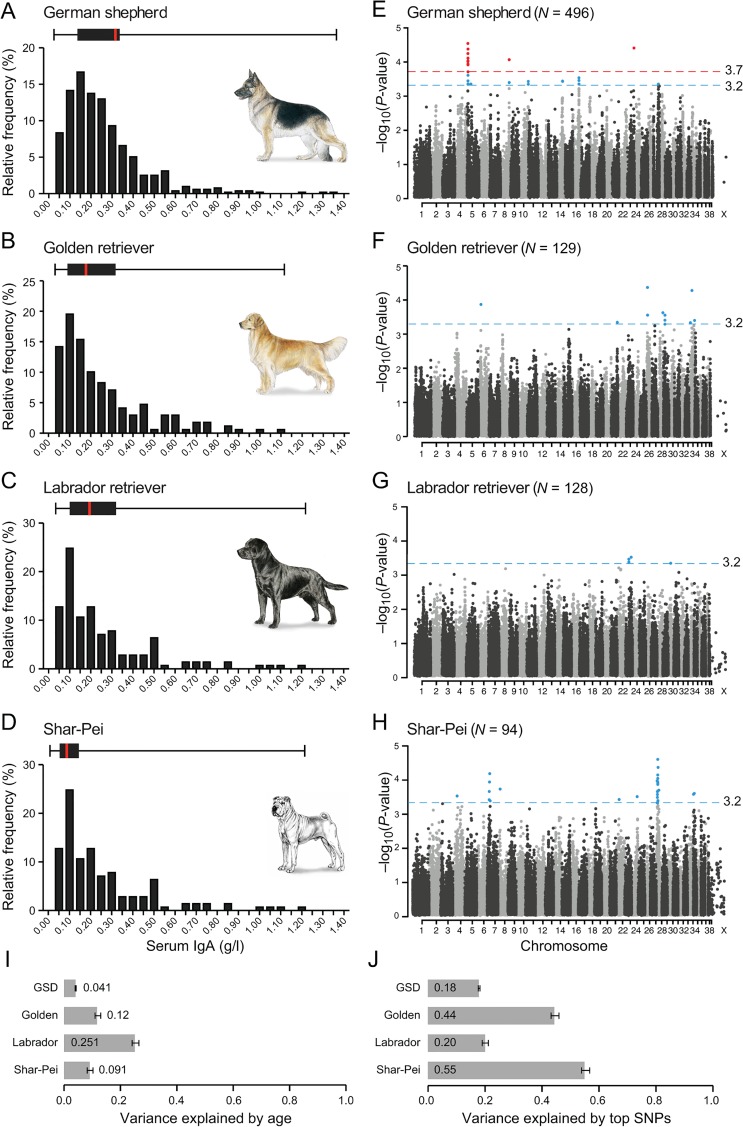
A combined GWAS in four breeds identifies 35 loci associated with IgA levels. (A-D) The distribution of IgA levels (0–1.40 g/l) clearly varied between the four breeds, here presented as relative frequency (%) and as box plots with the black box marking percentile 25 to 75 and red bar the median. The combined GWAS analyses from four runs (IgA levels divided into 2, 3, 4 and 5 groups) are presented in panel E-H, with the nominal significance defined at-log10p of 3.2 (grey line). In GSD (E), one region (14 SNPs) on CFA5 and single SNPs on CFA8 and CFA23 showed genome wide significance (red line at-log10p of 3.7) based on 1,000 permutations in five groups. In total, 35 suggestively associated loci (8 in GSD, 8 in GR, 3 in LR and 16 in SP) were defined based on LD of r^2^ >0.8 within 0.5 Mb of the top SNP. The fraction of phenotypic variance explained by (I) phenotypic variance explained by age was the lowest in GSD (4%) and remarkably high in LR (25%) and (J) the top SNP in each of the suggestively associated loci varied from 18% in GSD to 55% in SP.

We first evaluated possible factors acting on IgA levels. We observed no sex-related differences in IgA levels but a positive correlation with age (S1 Table). The phenotypic variance explained by age (in dogs older than 1 year) ranged from 4% in German shepherd (GSD) to 25% in Labrador retriever (LR) (Fig 1). In our previous studies [15, 17], we reported significantly lower IgA levels in GSDs affected by canine atopic dermatitis (CAD) compared to healthy GSDs. In the present study we used partially overlapping GSD samples and again observed significantly lower IgA levels in CAD cases compared to controls (N_cases_ = 101, N_controls_ = 130, p = 8.5 x 10^−8^). We did not observe any differences in IgA levels between CAD cases and controls in LR (N_cases_ = 67, N_controls_ = 74, p = 0.60) or GR (N_cases_ = 84, N_controls_ = 85, p = 0.72), although these two breeds are also predisposed to CAD.

### Thirty-five GWAS regions nominally associated with IgA levels

We identified three loci significantly associated with IgA levels in GSD and in total 35 regions nominally associated in the four breeds. GWAS was performed using the 170K Illumina canine HD array with 496 GSDs (88,704 markers), 129 GRs (100,680 markers), 128 LRs (112,428 markers) and 94 SPs (106,662 markers) passing quality control (Material and Methods and S2 Table) Age was included as a covariate and we controlled for both cryptic relatedness and population sub-structure by using a mixed model approach [21]. In GSD we also took the population structure into account by using subpopulation assignment as a covariate (S1 Table). To remedy potential bias introduced by having no cut-off value and a fluctuating phenotype, we combined p-values from four different GWAS runs of each breed; three runs were based on percentile groups of the IgA concentration and analysed as a continuous trait and the fourth run was a case-control analysis of extreme values (described in detail in Materials and Methods). Results from each run and quantile-quantile plots are presented in S1–S4 Figs. We set the nominal significance threshold at p = 0.0005 and SNPs with p-values below this threshold were considered suggestively associated. In addition, IgA associated regions were defined by linkage disequilibrium of r^2^ >0.8 within 1 Mb of the suggestively associated SNPs (Fig 1, S5 Fig and Table 1). The phenotypic variance explained by the top SNPs in the suggestively associated regions (i.e. one SNP representing each region) ranged from 18% in GSDs to 55% in SP (Fig 1). SNPs exceeding 97.5% the upper confidence interval (CI), defined empirically using 1,000 permutations, were considered genome-wide significant. Both approaches were consistent with methods described in Karlsson et al., 2013 [20].

**Table 1:** Genomic loci nominally associated with IgA levels.

SNP	Chr	Postion	p^a^	Alleles	Risk allele	Region start-end	Size (kb)	Genes in region	Closest gene to SNP
**German shepherd**								* *	* *
BICF2P417629	5	7,009,995	3.0E-05	G/A	A	6,498,684–8,172,621	1,674	*KIRREL3*, *ST3GAL4*, *DCP*	*KIRREL3* (upstream of)
BICF2P853011	5	29,238,199	4.6E-04	T/C	C	29,188,112–29,288,253	100	*TMEM123*, *MMP7*	*TMEM123* (intron)
TIGRP2P118956	8	63,777,575	8.9E-05	A/G	G	63,211,755–63,827,575	616	*PP4R4*, *RETNLB*, *GSC*, *SERPINA1*,*2*,*3–6*,*9–13*	*GSC* (upstream of)
BICF2S23435776	10	69,122,184	3.9E-04	A/C	C	69,072,184–69,174,348	102	*ADD2*, *CLEC4F*, *FIGLA*	*ADD2* (upstream of)
BICF2G630530840	14	42,224,407	3.8E-04	A/C	A	42,174,407–42,291,282	117	*CHN2*	*CHN2* (intronic)
BICF2P168717	16	46,117,662	3.0E-04	A/C	A	45,960,356–46,377,153	417	*IRF2*, *ENPP6*	*IRF2* (intronic)
BICF2G630365408	23	48,489,474	4.1E-05	C/A	C	48,439,474–48,539,474	100	*DHX36*, *GPR149*	*GPR149* (intronic)
BICF2P247941	27	37,404,652	4.6E-04	T/C	T	37,248,047–37,454,652	207	*SLC2A4*,*14*, *NANOG*, *FOXJ2*, *C3AR1*	*FOXJ2* (upstream of)
**Golden retriever**								* *	
BICF2P856602	6	18,062,312	1.6E-04	C/T	T	18,011,946–18,112,312	100	*Many*!	*PPP2CB* (intronic)
BICF2P442233	21	45,973,013	4.4E-04	T/C	T	45,923,013–46,023,013	100	*LUZP2*	*LUZP2* (intronic)
BICF2P261279	26	7,164,610	3.9E-05	G/A	A	7,114,611–7,846,707	732	*Many*!	*Lrrc43* (intronic)
BICF2G630261705	28	37,236,337	2.5E-04	T/C	T	37,228,122–37,362,478	134	*none*	*MKI67* (upstream of)
BICF2S232271	29	11,752,985	3.2E-04	A/G	G	10,808,328–11,803,162	995	*LINC01301*, *CLVS1*, *YWHAZ*, *RAB2A*, *CHD6*, *CHD7*, *CLVS1*	*CLVS1* (intronic)
BICF2P877790	34	10,311,397	4.4E-04	C/T	T	10,261,397–10,685,205	424	*IRX2*, *C5orf38*	*IRX2* (downstream of)
BICF2P963046	34	21,820,555	4.7E-05	G/T	G	21,406,221–22,589,723	1183	*TPRG1*, *TP63*, *LEPREL1*, *CLDN1*, *THEM207*, *IL1RAD*, *CLDN16*	*TP63* (upstream of)
BICF2P1301719	34	41,393,801	4.4E-04	C/T	C	41,343,801–41,751,420	408	*LRMP*	*LRMP* (upstream of)
**Labrador retriever**								* *	
BICF2G630378390	23	24,721,148	3.8E-04	G/A	G	24,665,511–24,771,148	106	*SATB1*, *LOC339862*	*SATB1* (downstream of)
TIGRP2P306755	23	42,076,158	3.3E-04	C/T	C	42,026,107–42,142,835	117	*None*	*PLSCR5* (upstream)
BICF2P227171	30	17,867,239	4.9E-04	T/C	T	17,817,239–17,917,239	100	*GNB5*, *MYO5C*	*GNB5* (intronic)
**Shar-Pei**								* *	
BICF2S23136666	4	31,168,913	3.2E-04	C/A	A	31,118,913–31,218,913	100	*NRG3*	*NRG3* (intronic)
BICF2G630552985	7	15,555,664	4.1E-04	G/A	A	15505664–15,605,664	100	*None*	*ZNF648* (upstream of)
BICF2S23410286	7	16,196,250	2.4E-04	C/T	T	16,146,250–16,246,197	100	*NPL*, *DHX9*	*NPL* (intronic)
BICF2P1401994	7	17,786,665	1.3E-04	C/T	T	17,736,665–17,836,665	100	*C1orf21*	*C1orf21* (intronic)
BICF2S23136652	7	19,673,829	7.1E-05	T/C	C	19,623,829–19,723,829	100	*MTFR1*, *PTGS2*	*PTGS2* (intronic)
BICF2P1436700	7	22,764,328	4.4E-04	A/G	G	22,714,328–22,814,328	100	*ASTN1*, *PAPPA2*	*ASTN1 & PAPPA2* (downstream of)
BICF2P215640	8	16,058,011	2.0E-04	A/G	G	16,008,011–16,108,075	100	*MIPOL1*, *FOXA1*	*MIPOL1 & FOXA1* (downstream of)
BICF2S2361733	22	20,899,197	4.1E-04	T/G	G	20,848,859–20,949,206	100	*none*	none, closest gene is *PCDH9*
TIGRP2P320304	24	41,301,998	3.3E-04	T/C	C	41,251,998–41,351,998	100	*CBLN4*	downstream of *CBLN4*
BICF2S23711428	28	5,983,856	1.2E-04	G/T	T	5,933,856–6,033,856	100	*HECTD2*	*HECTD2 (upstream of)*
BICF2G630276743	28	7,733,212	2.4E-04	T/C	T	7,683,212–7,803,352	120	*MYOF*, *CEP55*, *FFAR4*	*MYOF* (intronic)
BICF2G630276012	28	9,909,408	1.2E-04	A/G	G	9,859,408–9,994,305	135	*TLL2*, *TM9SF3*, *PIK3AP1*	*TM9SF3* (intronic)
BICF2G630275788	28	10,515,232	2.7E-05	G/A	G	10,446,800–13,077,479	2,631	*Many*!	*SLIT1* (intronic)
BICF2P677609	28	18,823,291	2.2E-04	A/G	G	18,773,291–18,873,291	100	*SORCS1*	*SORCS1* (intronic)
TIGRP2P404896	35	1,981,709	2.9E-04	C/T	T	1,931,709–2,031,709	100	*FOXC1*	*FOXC1 (intronic)*
BICF2G630772654	35	8,300,115	2.7E-04	G/A	A	8,250,009–8,350,115	100	*SLC35B3*	*SLC35B3* (downstream of)

A total of 35 IgA associated regions were defined across four dog breeds based on SNPs in strong linkage disequilibrium (LD, r^2^ >0.8) and within 1 Mb of the top SNP (plus the flanking 50 kb). All regions except four, contained one or more protein-coding genes. Displayed in the table are the genomic positions (Canfam 3.1) of the associated regions, information about the SNPs including p values (^a^ P1df) and gene content.

#### A locus on chromosome 5 associated to IgA levels in German shepherds

**Fig 2:**
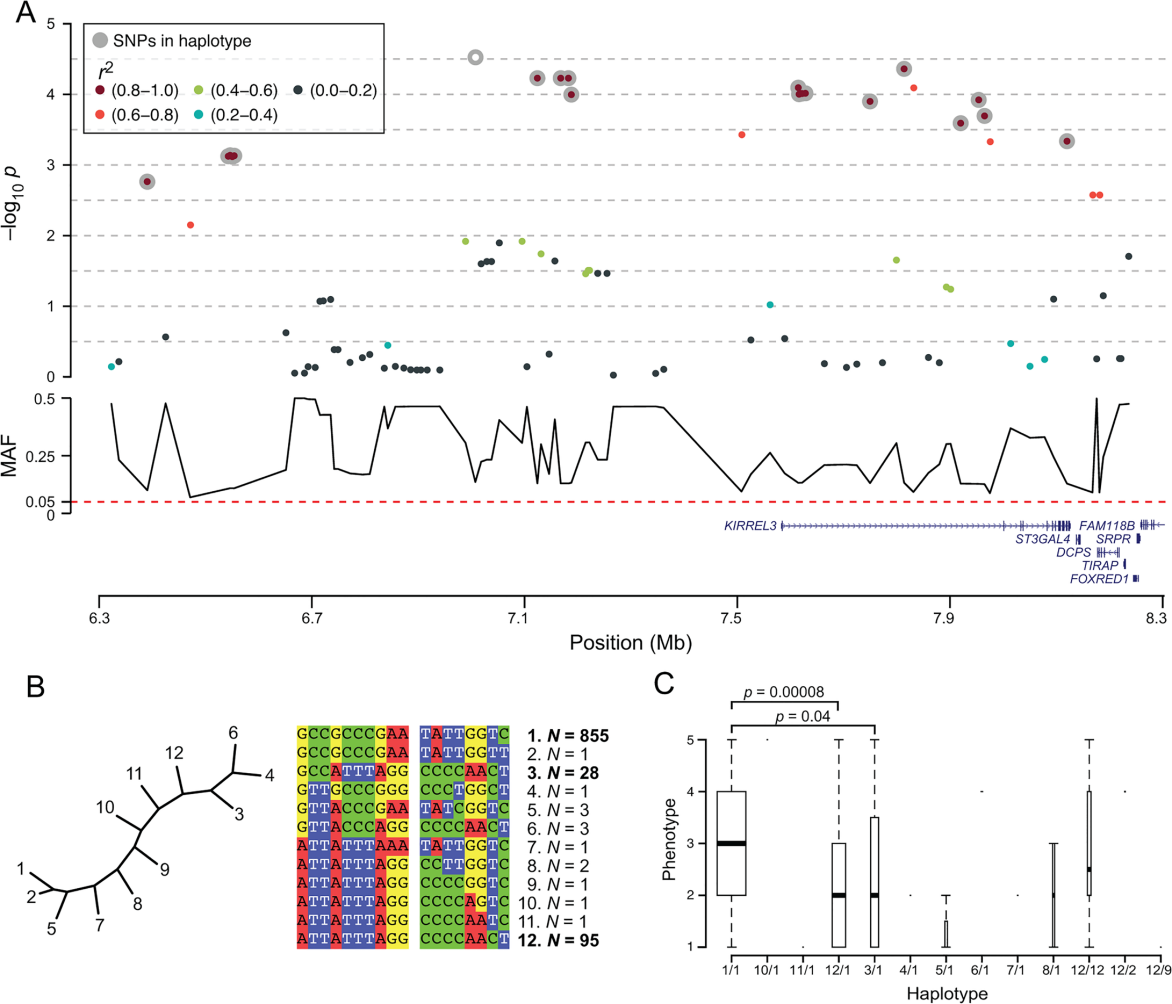
Two risk haplotypes at the German shepherd CFA5 locus results in lower IgA levels. (A) The genome wide significant locus on CFA5 consisted of 17 SNPs (grey circles) in LD (r2 >0.8) with the top SNP (white), with only the *KIRREL3* gene within the associated region and six genes adjacent. (B) The 18 SNPs were phased into 12 different haplotypes, of which nine were rare (N <3). Haplotype 1 was the most common (N = 855) and the remaining two haplotypes; 12 and 3 were more similar to each other than to haplotype 1. (C) Dogs homozygous for haplotype 1 (1/1) represented all groups of IgA evenly, whereas dogs heterozygous 1/12 and 1/3 had significantly lower IgA levels compared to 1/1 (p = 0.04 and 0.0008, respectively).

In GSD, a ~1 Mb long locus consisting of 14 SNPs on canine chromosome 5 (CFA5) 7,009,995–7,967,689 bp (CanFam3.1), with p-values ranging from 3.0 x 10^−5^ to 2.0 x 10^−4^, passed the threshold of genome-wide significant association. Based on SNPs in LD (r^2^ >0.8) with the top SNP, we phased a ~1.7 Mb long region (18 SNPs stretching from 6,392,996–8,122,621), which resulted in 12 different haplotypes. We defined two risk haplotypes; haplotype 3 and 12 (N = 28 and N = 95, respectively), one (haplotype 1) as the most common (N = 855) and present in all groups of dogs independent of IgA levels, and nine rare haplotypes (N ≤3). Heterozygous dogs carrying haplotypes 1 and 3, and haplotypes 1 and 12 had significantly lower IgA levels compared to dogs homozygous for haplotype 1 (p = 0.04 and p = 0.00008, respectively) (Fig 2). Despite the large size of the haplotype it harboured only one gene, *KIRREL3* (alias *NEPH1*), a transmembrane protein implicated in development, both in synapse formation and as a regulator of hematopoiesis [22, 23].

In addition to the CFA5 locus, two single SNPs on CFA8 and CFA23 passed the threshold of genome-wide significance (Fig 1 and S6 Fig). The CFA23 top SNP (CFA23: 48,489,474), not in LD with any other SNP, was located in the intron of *GPR149* (G protein-coupled receptor 149), one of the orphan GPRs with unknown ligands and highly expressed in oocytes (with important functions for fertility) but also in the brain [24, 25]. The top SNP on CFA8 was in high LD (r^2^ >0.8) with three SNPs spanning ~0.5 Mb (CFA8: 63,261,755–63,777,575), but none of the phased 11 haplotypes could be defined as risk or protective (data not shown). This region contained 13 genes, including *GSC* (a transcription factor that defines neural-crest cell-fate specification and contributes to dorsal—ventral patterning [26]), *RETNLB* (resistin-like beta, associated with insulin resistance), *PPP4R4* (Protein Phosphatase 4, Regulatory Subunit 4, a putative regulatory subunit of serine/threonine-protein phosphatase 4) and 10 genes in the SERPINA family (Serpin Peptidase Inhibitor, Clade A (Alpha-1 Antiproteinase), members *1*,*2*,*3–6*,*9–13*). SERPINAs are protease inhibitors with multiple immune functions. *SERPINA9* has a role in naïve B-cell maintenance and its expression is restricted to the germinal centre of secondary lymphoid organs where B-cells proliferate [27].

#### Risk haplotype on chromosome 28 in Shar-Pei

**Fig 3:**
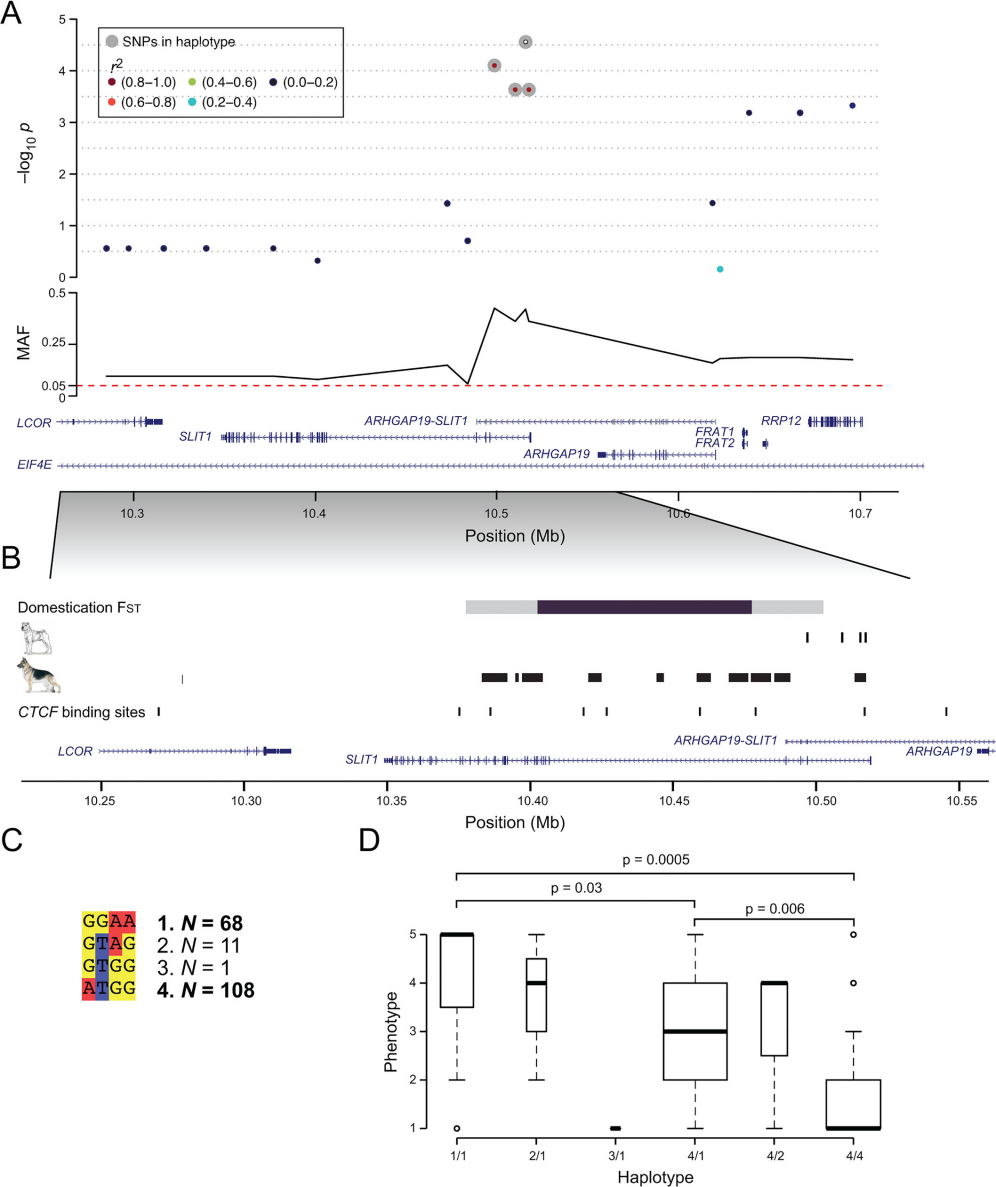
The *SLIT1* gene harbours an associated haplotype in Shar-Pei and fixed blocks in German shepherd. (A) In SP, four SNPs (grey circles) suggestively associated to IgA levels, were located within the first intron of *SLIT1*. (B) A distinct increase in the degree of genetic differentiation (F_ST_) between dogs and wolves span a 75 kb region (windows with F_ST_ >0.67 are coloured in black) within the *SLIT1* locus, with F_ST_ values of two consecutive 50 kb windows reaching 0.68 and 0.67, respectively (windows with F_ST_ >0.43 are coloured in grey). More extreme genetic differentiation was only seen in 7% of the whole dog genome, potentially indicating that IgA levels may have been affected in a pleiotropic manner by primary selection affecting another primary target (such as brain function) during dog domestication. Blocks of fixation were identified in GSD, spanning several regulatory sites including binding sites for the transcription factor CTCF. (C) The top SNPs in SP were in high LD (r^2^ >0.8) and phased into four haplotypes where two were common (1 and 4) and two were rare (2 and 3). Dogs homozygous for 1/1 had significantly higher IgA levels compared to dogs homozygous for 4 (4/4) and heterozygous 4/1 (p = 0.0005 and p = 0.03, respectively). Additionally, homozygous 4/4 had significantly lower IgA levels than heterozygous 4/1 (p = 0.006) indicating an additive effect of the risk and/or protective haplotype.

The strongest signal of association to IgA levels in SP was a region on CFA28 with the top SNP at position 13,512,782 bp. At this locus, we identified a ~20 kb haplotype (CFA28: 10,496,764–10,517,160) based on 4 SNPs in high LD (r^2^ >0.8) with p-values ranging from 2.7 x 10^−5^ to 2.3 x 10^−4^. In total two rare (N ≤11) and two common haplotypes were identified. Haplotype 4 was defined as the risk (N = 108) and haplotype 1 as the protective haplotype (N = 68), as we discovered a significant difference (p = 0.0005) in IgA levels between dogs homozygous for the risk haplotype (4/4) vs. dogs homozygous for the control haplotype (1/1). Heterozygous dogs (1/4) had intermediate IgA levels, significantly different both compared to 1/1 (p = 0.03) and to 4/4 (p = 0.006) (Fig 3) suggesting an additive nature of the effect. This short haplotype spanned the first intron of the gene *SLIT1*, a large extracellular matrix-secreted glycoprotein that functions as a ligand to the repulsive guidance receptors (Robo) family [28].

#### Fixation of regulatory sites within *SLIT1* in German shepherd

The SP risk allele at the top SNP (position CFA28: 10,515,232) was completely fixed in both GSD and LR (frequency = 1.0) and had a frequency of 0.90 in other breeds serving as controls (N_dogs_ = 350) (S3 Table). We therefore studied this region (expanding it to ~3 Mb; CFA28: 9,000,091–11,999,424) in more detail by utilizing allele frequencies for 13,004 variants (9486 SNPs and 3518 indels) from an additional 20 GSDs and 20 LRs (S4 and S5 Tables, respectively). We defined regions of fixation in windows of 11 variants (1 variant overlap) and where the proportion of fixed variants was 1. We identified 79 fixed regions in LR and 356 in GSD within the ~3 Mb region (S6 and S7 Tables, respectively). Next we focused on a closer region around *SLIT1*, in total ~400 kb (CFA28: 10,201,240–10,600,160), and identified 169 fixed regions in GSD forming 14 blocks of fixation out of which four small blocks (130–220 bp) were located downstream of *SLIT1* and 10 longer blocks (1–9 kb) spanned 134 kb within *SLIT1*. The block located furthest upstream of *SLIT1* harboured two of the four top IgA associated SNPs in SP, including the top SNP (CFA28: 10,515,232). Through comparison to human (hg19), we noted a high regulatory potential within the 134 kb region. Specifically, there were predicted binding sites for the transcription factor CTCF within four fixed blocks in GSD including the block with the SP top SNPs (Fig 3 and S7 Fig). In LR, we identified 37 fixed regions (within the ~400 kb) forming four blocks (ranging from 130 to 242 bp in size) but none were located within or near *SLIT1*.

#### Domestication sweep within *SLIT1*

To test whether the increased degree of fixation in this region may be associated with selection during initial dog domestication, we analysed our previously published pooled whole-genome resequencing data representing 12 wolves and 60 dogs from 14 diverse breeds [29]. We noted a distinct increase in Fixation Index F_ST_ (F_ST_ = 0.68 and 0.67; Z(F_ST_) >4) and a decrease in dog heterozygosity H_P_ (H_P_ = 0.12 and 0.14; Z(H_P_) <-2.6) in two consecutive windows (spanning CFA28: 10,403,080–10,478,044) within the *SLIT1* gene, 37 kb downstream from the top associated SNP and 19 kb from the end of the associated haplotype in SP (Fig 3, S7 Fig and S8 and S9 Tables).

### Pathway analyses

#### Genes involved in inflammation dominate pathways of IgA associated regions

While the different breeds had no overlapping association signal, we wanted to explore if common pathways could be found among the different risk loci. We identified significant connectivity between genes in the IgA associated regions across three breeds (GSD, GR and SP but not LR) using the PubMed-based program GRAIL [30]. The genes were connected by pathway key terms (S10 Table). We aimed at defining common pathways across many of our regions and found that the pathways representing numerous (7 or 8) regions were ‘serum’ (rank #7), ‘insulin’ (#8), ‘matrix’ (#10), ‘carcinoma’ (#13) and ‘complement’ (#16) (Table 2). Four out of nine genes with significant GRAIL p-value were clearly involved in inflammation such as two SERPINA genes (*SERPINA9* and *SERPINA12*, inhibits proteases), *C3AR1* (Complement Component 3a Receptor, a major player in the complement system) and *IL31* (Interleukin-31, a T-cell derived cytokine associated with chronic skin inflammation and pruritus). Additional genes with GRAIL p <0.05 were *GRP149* and four different enzymes: *PTGS2* (prostaglandin-endoperoxide synthase 2, the key enzyme in prostaglandin biosynthesis), *HOGA1* (4-hydroxy-2-oxoglutarate aldolase 1, a mitochondrial enzyme), *ALDOA* (aldolase A, a glycolytic enzyme) and *NPL* (N-acetylneuraminate pyruvate lyase, an enzyme which catalyzes sialic acid) (Table 2 and S10 Table).

**Table 2:** GRAIL pathways of the IgA associated regions.

Chr	Region start-end	Genes	GRAIL p	'serum'	'insulin'	'matrix'	'carcinoma'	'complement'
**German shepherd**								
8	63,211,755–63,827,575	*SERPINA1*	0.13	•		•	•	•
8	63,211,755–63,827,575	*SERPINA3*	0.064	•		•	•	•
8	63,211,755–63,827,575	*SERPINA4*	0.17	•			•	
8	63,211,755–63,827,575	*SERPINA6*	0.19	•	•			
8	63,211,755–63,827,575	SERPINA9*	**0.037**			•		
8	63,211,755–63,827,575	SERPINA12*	**0.019**			•		
10	69,072,184–69,174,348	*FIGLA*	0.12	•	•	•		•
23	48,439,474–48,539,474	***GPR149***	**0.0018**			•		
27	37,248,047–37,454,652	C3AR1*	**0.014**	•	•			•
27	37,248,047–37,454,652	*SLC2A3*	0.16		•		•	
5	29,188,112–29,288,253	*MMP7*	0.18	•	•		•	
**Golden retriever**								
6	18,011,946–18,112,312	***ALDOA***	**0.048**					•
6	18,011,946–18,112,312	*TBX6*	0.12		•			
26	7,114,611–7,846,707	*DIABLO*	0.15				•	
26	7,114,611–7,846,707	IL31*	**0.0056**	•		•	•	
**Shar-Pei**								
28	10,446,800–13,077,479	*MORN4 / C10orf83*	0.075					•
28	10,446,800–13,077,479	***HOGA1 /C10orf65/NPL2***	**0.013**	•				
28	10,446,800–13,077,479	*CHUK*	0.16		•		•	
28	10,446,800–13,077,479	*CPN1*	0.10	•				•
28	10,446,800–13,077,479	*HPSE2*	0.11			•	•	
28	10,446,800–13,077,479	*NKX2-3*	0.10	•	•			•
28	10,446,800–13,077,479	*SFRP5*	0.17				•	
7	19,623,829–19,723,829	***PTGS2***	**0.049**	•		•	•	
7	22,714,328–22,814,328	*ASTN1*	0.14	•				
7	22,714,328–22,814,328	*PAPPA2*	0.071	•	•	•	•	•
7	16,146,250–16,246,197	*DHX9*	0.17					•
		Total number of genes		**14**	**9**	**10**	**12**	**10**
		Total number of regions		**8**	**7**	**7**	**7**	**7**

Genes in the 35 IgA associated regions across four breeds were analysed for their connectivity using the web-based software GRAIL. This table display the five pathways represented by multiple regions (7 or 8) and breeds (GSD, GR and SP). Out of the eight genes (bold) that were assigned a significant GRAIL p (<0.05) based on the connectivity to genes in other IgA associated regions, half were involved in inflammation (marked with an asterisk*).

#### Gene sets enriched in IgA associated regions

We also used INRICH to search for gene set enrichment based on the Gene Ontology (GO) database [31]. In total 51 gene sets were significantly (p ≤0.05) enriched in our regions and the top nine also reached significance after correction for multiple testing (p_corrected_ ≤0.05) (S11 and S12 Tables). There were 11 gene sets (four with p_corrected_ ≤0.05) relating directly to transcriptional activity, including the top term (GO:0003700, p = 3.0 x 10^−6^). The other top gene set was response to estradiol stimulus (GO:0032355, p = 3.0 x 10^−6^). We also hit gene sets related to haematopoiesis (GO:0030097, p = 8.0 x 10^−4^) and platelets (GO:0031093 with p_corrected_ = 0.03, GO:0002576, GO:0030168 and GO:0007596; p ranging from 8.5 x 10^−5^ to 1.1 x 10^−3^). We detected gene sets related to cytokine responses (GO:0034097, p = 1.5 x 10^−4^, p_corrected_ = 0.05) and lipopolysaccharide (GO:0032496, p = 6.0 x 10^−4^). The additional gene sets significant after correction were regulation of cell growth (GO:0001558, p = 1.0 x 10^−5^) and actin filament organization (GO:0007015, p = 8.3 x 10^−5^).

## Discussion

Despite the attention IgA levels in dogs have received in the past, the normal range of serum IgA as well as a generally accepted cut-off value for IgAD has not yet been established. In a previous study [15], we found that the IgA levels vary extensively between breeds, which could explain the lack of an accepted cut-off. The literature often reports breed-specific IgA values and suggested cut-offs for canine IgAD mark the lower limit for the 95% confidence interval for the mean in the studied populations [11, 13, 32]. However, this does not mark a physiologically proven threshold to distinguish between low levels of IgA and IgAD in dogs.

### Combining association p-values distinguish true signals

In this study we did not set out to define a cut-off that determines IgAD. Instead, we used four breeds, identified from our earlier study as breeds with generally low serum IgA levels, in GWAS to identify genes and pathways that could be of relevance for both canine and human IgAD. We also suggest a novel approach to perform genome-wide association mapping of complex continuous traits using multiple runs of groups based on percentile intervals instead of the actual value. The rationale behind this approach originates from several factors complicating the interpretation. First, the lack of a generally accepted cut-off value to distinguish normal IgA levels from deficient in dogs hinders an appropriate case-control association study. Second, IgA levels are known to fluctuate depending on the environmental exposure at the time-point of sampling. Third, the IgA ranges (as well as mean and median values) vary widely between breeds and [15] therefore an approach that takes breed differences into account is proposed. The rationale behind *combining* p-values from three different percentile series is to remove the strict cut-offs created between percentile groups. In this way we also dilute false positives and increase the chance to distinguish the true signals. To the three series of percentile groups, we also added a robust case and control analysis with the necessary removal of dogs with intermediate IgA levels (cases<25% percentile, controls >75% percentile) in order to cover the possibility that extreme values would detect signals. Thus, the statistical model we used for identifying loci associated with IgAD are based on a continuous analysis but developed to fit the actual phenotype. In humans, IgA levels are normally higher in males [33, 34] whereas in dogs the reports are conflicting [12, 35, 36]. In our large set of samples we could not detect any difference in IgA levels between the sexes.

#### IgA associated regions

We identified 35 loci nominally associated with IgA levels in the four different dog breeds. Interestingly, none resided within the MHC gene region. In GSD we identified genome-wide significantly associated SNPs possibly due to GSDs having strong risk factors or simply because the sample set (~500) was large enough to reach significance. Although the number of SPs was much lower (~100), we detected one haplotype (within *SLIT1*) significantly associated to IgA levels that is likely to contribute to the large proportion of the phenotypic variance explained (55% by top SNPs). The smaller sample sets in combination with many risk factors with small effects are likely the reason for the lack of significant results in the other breeds. GSD and SP are breeds that have been repeatedly identified as ‘high-risk IgAD breeds’ by us and by others [10, 11, 14, 15, 36–38]. Our genetic results are in concordance with these previous reports, as GSD and SP appear to carry the strongest risk factors for low IgA levels.

### Candidate genes implicated in early B-cell development

Human IgAD is not associated with an absence or decrease in the B-cells themselves, but a failure of B-cells to differentiate into mature IgA-secreting plasma cells [39]. In dogs, less is known about the disease mechanism but clinically it appears similar to human IgAD. The development of B-cells begins in the bone marrow with the formation of blood compartments from hematopoietic stem cells (hematopoiesis). With regards to B-cells it also includes the genetic recombination of heavy and variable chains (referred to as V(D)J-joining). Interestingly, IgAD in humans also seem to involve stem cells as the phenotype can be transferred by bone marrow transplantation [40]. In our study we identified two significantly associated regions harbouring the novel candidate genes *KIRREL3* and *SLIT1* with documented roles in hematopoiesis.

*KIRREL3* was the only gene within the long-range (~1.7 Mb) haplotype on CFA5, significantly associated with IgA levels in the GSDs. This transmembrane protein is widely expressed in the nervous system [41] where it has been implicated in synapse formation and abnormal brain function [22, 42] and hence is an important developmental gene. Moreover, a homolog of *KIRREL3* (mKirre) has been reported as one of the genes needed to support and regulate hematopoiesis in bone marrow, which suggest that its developmental function also covers the formation of lymphocytes (including B- cells)[23].

The significantly associated haplotype in SP was located in the first intron of the *SLIT1* gene. The haplotype analysis revealed an additive effect where dogs homozygous for either the risk or protective haplotype were significantly different from the heterozygous dogs, which strengthens the possibility that it is functional. The SLIT proteins and their Robo receptors form complexes with conserved guidance cues for repulsion, attraction or branching that influences cell migration and proliferation of cells in the central neural system as well as immune cells [28, 43–45]. Robo/SLIT has been demonstrated to modulate the chemo attractant-induced migration of mature leukocytes through inflammation as well as axon migration and branching during development [46, 47]. A similar cue mechanism of Robo/SLIT appears to regulate homing, self-renewal, migration and proliferation of hematopoietic stem and progenitor cells in the bone marrow [48–50]. In addition, missense mutations in *SLIT1* have been connected to aplastic anemia (AA), a rare but life-threatening bone marrow failure syndrome [51], which also support the role of *SLIT1* in early hematopoiesis.

#### Signals of selection in *SLIT1* indicate pleiotropic effects

We found that GSDs were fixed for regions within *SLIT1* with high regulatory potential and binding sites dominated by the transcription factor CTCF, a highly conserved zinc finger protein with various genomic regulatory functions including transcriptional activation/repression and with a critical role in coordinating DNA methylation and higher-order chromatin loops [52]. Moreover, CTCF has a known role in V(D)J recombination in B and T cells [53]. When comparing wolves to dogs and evaluating the F_ST_, we identified a 75 kb domestication sweep signal, based on two consecutive windows with F_ST_ = 0.68 and 0.67. This degree of fixation deviates from the average F_ST_ between dog and wolf (mean F_ST_ = 0.33) by more than 4 standard deviations and a similar or more extreme differentiation is only observed in 7% of the dog autosomal genome, indicating that selection at *SLIT1* may have contributed to important dog domestication features, which typically affect brain function [29].

We find it interesting that one of our candidate genes are restricted to B-cells (*SERPINA9*) and the other two (*KIRREL3* and *SLIT1)* share the features of being essential in both brain and immune cell development. Both *KIRREL3* and *SLIT1* are recognized for their function in the nervous system, and only recently it has become evident that Robo/SLIT signalling as well as *KIRREL3* are also key players in immune cell development. Moreover, *KIRREL3* knock-out mice display a loss of male-male aggression [54] implicating changes in behavior connected to this gene. As dog selection often target behavioural traits we speculate that a selective pressure on these two genes could also have enriched for immune defects in a pleiotropic manner.

Altogether, the two genes (*KIRREL3* and *SLIT1*) suggest an alteration in early B-cell development in canine IgAD. As the generation of circulating B-cells, their migration and proliferation pattern are not known in dogs, defects at the hematopoietic level cannot be excluded. Interestingly, hematopoiesis was also highlighted in our gene set enrichment analysis. Furthermore, early B-cell development defects characterize a variety of immunodeficiency disorders in humans [55].

### Genes potentially involved in B-cell proliferation

Class-switch recombination (CSR), is a process involved in the late proliferation steps before B-cells differentiate into plasma cells and occurs in the germinal centres of secondary lymphoid organs. Similar to V(D)J joining in early B-cell development, class-switching also involves DNA recombination, and represents the process in which the immune system produces antibodies of different isotypes. Two important signals to initiate germinal centre reactions, such as CSR, is the engagement of the B-cell receptor complex (by antigen) or CD40 (by CD40L). An interesting gene for CSR is *SERPINA9*, located within the genome-wide significant association signal (GSD CFA8) and with a significant GRAIL p-value. The expression of *SERPINA9* is restricted to B-cells in germinal centres of secondary lymphoid organs where it inhibits trypsin-like proteases. Interestingly, the expression of *SERPINA9* is enhanced in vitro by CD40/CD40L signaling and down regulated once these cells are differentiating into circulating plasma- or memory cells [27]. Thus, this gene is not only highly interesting for IgAD due to its restricted expression pattern, but also as it appears to play an important role in the complex array of events leading to specific antibody responses.

### Pathways implicate genes involved in inflammation

It is not known whether IgAD actually originates from B-cells themselves, or if the phenotype reflects impairments in T-helper cells, of antigen-presenting cells (which both stimulate the differentiation and proliferation of B-cells), or even abnormalities in the cytokine network [6]. Both programs used for the pathway analyses detected pathways related to the immune system; GRAIL specifying genes predominantly in inflammation (*SERPINA9/12*, *C3AR1*, *and IL31*) and INRICH gene sets involving early development of immune cells, cytokine responses and platelet activation.

#### A potential bias towards inflammation

The majority of the sample set for this study was initially collected as cases and controls of other immune-related disease phenotypes such as CAD (GSD, GR, LR), pancreatic acinar atrophy (GSD) and Shar-Pei autoinflammatory disease (SP). Consequently, the breeds in the study are predisposed for these disease phenotypes (in addition to low IgA levels) and the dogs sampled were specifically selected, the incidence of disease within the sample cohort is probably not representative for the whole breed. If we also consider the poorly understood connection between IgAD levels and autoimmunity, asthma and allergy [6], genetic risk factors of immune-mediated diseases (predominantly inflammatory diseases in our cohort) may theoretically affect the low IgA dogs in our sample set and introduce a bias leading to the association to genomic regions involved in the various immune diseases and not primarily to low IgA levels. However, in our previous paper [15] where we performed serum IgA screening in ~1300 dogs (22 breeds), we also performed correlation analyses between IgA levels and some breed’s specific immune disease. The breeds and diseases studied included GSD (CAD and pancreatic acinar atrophy), LR (CAD), GR (CAD), SP (Shar-Pei autoinflammatory disease), Standard poodle (Addison’s disease), Bearded collie (Addison’s disease), Nova Scotia duck tolling retriever (steroid responsive meningitis arteritis), Jämthund (diabetes mellitus) and Giant Schnauzer (lymphocytic thyroiditis). Remarkably, IgA levels did only show correlation to CAD (p <0.0001) and pancreatic acinar atrophy (p = 0.04) and to no other diseases in any other breed.

#### Low IgA levels correlated to CAD in German shepherd

Possibly, there is an IgA dependent CAD and an IgA independent CAD since CAD clearly correlates to low IgA levels in GSD but not in GR and LR. In our previous study [17] we detected a genome-wide significant signal associated with CAD in GSDs when the effect of IgA levels was taken into account. Without the IgA levels the signal was absent suggesting that the CAD phenotype in GSD is partly caused by low IgA levels rather than the opposite scenario; that CAD would cause low IgA levels in the dogs as a secondary effect. In this present study, several genes, with possible functions in CAD, were significant in the GRAIL pathway analyses; *C3AR1* has been associated with childhood bronchial asthma, a common chronic inflammatory disease characterized by hyperresponsive airways, excess mucus production, eosinophil activation, and the production of IgE [56]; *IL31* involved in skin inflammation and pruritus [57] and in inflammatory bowel disease; and the *SERPINA* gene family with its anti-inflammatory properties are interesting for inflammatory disease. Hence, the genes influencing IgA levels could possibly predispose to CAD directly, or indirectly through the effect of having low IgA levels.

## Conclusion

IgAD is a complex disease phenotype influenced by multiple unknown genetic factors. In this comparative study, we took advantage of the beneficial genome structure in dog breeds, its resemblance to the human disease phenotype and used a new approach to handle a continuous variable. This resulted in the successful mapping of four genomic regions significantly associated to IgA levels in dogs with suggested disease mechanisms involving early B-cell development (candidate genes *KIRREL3* and *SLIT1*) and proliferation of B-cells (candidate gene *SERPINA9*). In addition, pathway analyses of 35 nominally associated regions also implicated inflammatory routes, which suggest an aetiology of IgAD to not be restricted to the humoral immune response but also to the innate immune system and inflammatory responses.

## Materials and Methods

### Samples

Blood samples, EDTA blood for DNA extraction and serum for IgA quantification, were collected from 1101 privately owned, purebred dogs including 516 German shepherds (GSD), 187 Golden retrievers (GR), 302 Labrador retrievers (LR) and 96 Shar-Pei (SP) in collaboration with veterinarians in the United States, Sweden and Switzerland. Owner consent was collected for each dog. Ethical approvals were granted by the Swedish Animal Ethical Committee C139/9 and C2/12 (the Swedish animal Welfare Agency no. 31-4714/09 and 31-998/12, respectively). The Broad Institute: Lindblad-Toh 0910-074-13 and the canton of Bern: Tosso Leeb 23/10. Serum was separated from the red blood cells by centrifugation and stored at -20°C until used. Genomic DNA was extracted from EDTA blood samples using the Qiagen midi prep extraction kit (Qiagen, Hilden, Germany), diluted in de-ionized water and stored at -20°C until used.

### Quantification of Immunoglobulins using Canine IgA ELISA

Serum IgA levels were quantified by using a capture enzyme-linked immunosorbent assay (ELISA) protocol described previously [15, 17, 58] and all samples were quantified as part of previous study [15]; except for 191 GSDs already quantified as part of our previous CAD GWAS [15, 17, 58] and for the addition of serum samples from 197 GSDs for the current study. All samples were quantified at least four times. Samples showing strong variation between duplicates were run up to six times and potential outliers were subsequently excluded. Two samples (both LR) were also excluded for being potential outliers due to very high IgA concentration (1.98 g/l and 1.88 g/l). In short, serum samples were measured for their serum IgA concentration using polyclonal affinity purified goat anti-dog IgA antibodies (AbD Serotec, Oxford, UK) and alkaline phosphatase-conjugated polyclonal goat anti-dog IgA (Bethyl Laboratories, TX, USA). The antibodies were diluted 1:2,000 with carbonate-bicarbonate buffer (0.05 M, pH 9.6) respectively, Tris-buffered saline and Tween (TBST). Samples were diluted 1:25,000; 1:50,000 and 1:100,000 with phosphate-buffered saline with Tween (PBST). Para-Nitrophenylphosphate (PNPP) was used as substrate. Serum samples from dogs, kindly provided by Professor M.J. Day (Bristol University, England) with previously determined IgA concentrations were used as controls for each individual measurement. The quantification of serum IgA in the sample set has been described in our previous study [15] and the distribution is presented for the current data set in Fig 1 and S13 Table.

### SNP genotyping and quality control

The initial data set for GWAS consisted of 1101 dogs genotyped by the 170K Canine HD Bead-Chip (Illumina San Diego, CA, USA) as part of various other dog projects within our research group. The four breeds were analysed separately. An iterative quality control was performed of the 170K markers prior to further analyses in order to remove non-informative markers (SNPs with a minor allele frequency below 5%) and poorly genotyped markers (call rate ≤0.95). Dogs were excluded due to trait or covariate missing and with an age below one year, too high identity by state (IBS ≤0.95), low call rate (≤0.95) or if showing too high autosomal heterozygosity (≤95%). The breakdown of the dataset and the quality control are shown in S2 Table and the final dataset used in GWAS consisted of 496 GSDs (88704 markers), 129 GRs (100680 markers), 128 LRs (112428 markers) and 94 SPs (106662 markers). All coordinates presented in the results are from the CanFam3.1 genome assembly (Sept. 2011).

### Correlation to fixed effects

We used Pearson's product moment correlation coefficient to test if the variables age, sex, subpopulations (if present) and canine atopic dermatitis (CAD, in GSD, GR and LR) were correlated with IgA levels.

### Genome-wide association studies

We tested for association between IgA levels and genetic markers in all four breeds separately and used the age in years at sampling as a covariate. When analysing GSDs, we also included subpopulation assignment as a covariate. Cryptic relatedness and population sub-structure were controlled through a mixed model approach [21] and the GenABEL package ver. 1.7–0 [59], part of the R statistical software ver.2.14.2 [60], was used.

Four different GWAS runs were performed in each breed separately. The IgA levels were divided into groups of percentile intervals; the 20% percentile creating five groups (0–20, 21–40, 41–60, 61–80, 81–90), the 25% percentile creating four groups (0–25, 26–50, 51–75, 76–100) and the 33,3% percentile creating three groups (0–33, 34–67, 68–100). The percentile groups formed in each breed as well as the number of dogs within each group are presented in S14 Table. The analyses were performed using the group number as its phenotype label (in a close to continuous analysis manner). The fourth GWAS was formed with cases and controls; dogs with IgA levels lower than the 25% percentile as cases and levels higher than the 75% percentile as controls, thus the middle group was excluded resulting in a lower number of dogs compared to the other GWAS datasets. The p-values from these four runs were then combined (for each breed separately) using the following formula:

p^=(∏i=1npi¯)1n,

where $\hat{p}$ is a vector of merged p-values, $\bar{p}$_i is a vector of i-th run of association study and n is the number of runs. We then used $\hat{p}$ values for defining associated SNPs at the final stage of the analyses.

### Threshold for associated SNPs and regions

Similarly to Karlsson et al. [20], we defined genome-wide significance using 95% CIs calculated from the empirical distribution of p-values observed by rerunning the GWAS with randomly permuted phenotypes 1,000 times. For each breed separately, SNPs exceeding the 97.5% upper empirical CI were defined as genome-wide significant in that breed. The permutations were performed in each percentile group independently (i.e. four separate runs per breed**,** S8 and S9 Figs) and if more than one run resulted in SNPs crossing the 97.5% upper CI the strictest threshold was used to define the genome-wide significant SNPs in the combined analysis. SNPs were considered suggestively associated if p <0.0005, using the uncorrected p-value (P1df). We used uncorrected p-values (no genomic control), since the mixed model framework already sufficiently corrected for population structure. IgA associated regions were based on the latter threshold and defined using strong linkage disequilibrium (LD) of r^2^ >0.8 within 1 Mb of top SNP (according to methods described in Karlsson et al. [20]).

### Variance explained by top loci and fixed effects

We calculated the phenotypic variance explained by fixed effects (age) and by the top SNPs in the IgA associated regions. We used five percentile groups as the phenotype and calculated variance explained by comparing the original phenotypic variance with the residual variance of a linear mixed model with appropriate fixed effects included (age in our case). We used standard jackknife resampling to determine the variance estimation error.

### Haplotype analysis

We used PHASE 2.1.1 [61] to phase associated and linked SNPs into haplotypes and GenABEL to define associated SNPs, SNPs in LD (>0.8) and to plot haplotype distributions. In addition, we used the Welsh two-sample t-test to detect significant differences between haplotypes in dogs with different IgA levels using five percentile groups.

### Fixation of associated SNPs

We calculated risk allele frequencies of the top SNPs in the IgA associated regions to evaluate if associated SNPs in one breed were fixed (frequency >0.95) in any of the other breeds (S3 Table). In addition, we calculated the frequencies in a group of 350 dogs representing 25 breeds (S15 Table) part of the dataset used in a previous study by Vaysse et. al., 2011 [62]). Breeds with a suspected predisposition to low IgA levels were excluded from the initial dataset. Poorly genotyped markers (call rate ≤0.95) were excluded prior to the analysis.

### Allele frequencies from eight dog breeds

To study detailed patterns of genetic variation in a ~3 Mb region spanning the *SLIT1* locus (CFA28: 9,000,091–11,999,424) we analyzed sequence data from 160 dogs from eight breeds (20 dogs per breed, none of which were included in the GWAS) (S16 Table). Briefly, we used Burrows-Wheeler Aligner, BWA [63] and GATK [64] following the best practice described by GATK (https://www.broadinstitute.org/gatk/) to map sequence reads to the reference genome and call variants. In total we characterized 13,004 variants (9486 SNPs and 3518 indels) in the 3 Mb region. Based on allele frequencies of the called variants, we then searched for regions of fixation in the 20 GSDs and 20 LRs (S4 and S5 Tables, respectively). We calculated the proportion of fixed variants in sliding windows of 11 variants (to obtain one centred variant and five on each side) using one variant overlap and defined regions of fixation where the proportion was set to 1 (i.e. all variants fixed within a window). We defined blocks of fixation by combining adjacent windows plus adding five variants (i.e. fixed variants based on the definition of fixed regions) on each side, within a ~400 kb region (10,201,240–10,600,160), including *SLIT1*. We also compared this region to human (hg19) to evaluate regulatory potential and transcription factor binding sites. For comparison we also tried 25 variants per windows (one variant overlap) resulting in a very similar pattern (S10 Fig). Thus, the results presented in this paper (from 11 variant-windows) appear robust.

### Fixation index for wolf versus dog

We utilized a previously published whole-genome resequencing data set [29], to investigate the degree of genetic differentiation between dog and wolf in the CFA28 region that includes the *SLIT1* gene. To this end we first estimated allele frequencies throughout the autosomal part of the genome in the dog and wolf population by counting allele specific sequencing reads in the single wolf pool (12 wolves) and all five dog pools (60 dogs from 14 breeds) combined, respectively. We then calculated Weir and Cockerhams (1984) version of the fixation index (F_ST_) for all SNPs. Given a minimum of 10 segregating sites per window we averaged F_ST_ values across 50 kb windows sliding 25 kb at a time and Z-transformed the resultant distribution. Next, we used the same approach to calculate the average heterozygosity for the dog pools (H_P_) and Z-transformed the distribution. S8 and S9 Tables present the F_ST_ and H_P_, respectively, for the studied region (CFA28: 10,053,171–10,978,044).

### Pathway analyses

#### GRAIL

Regions of association within each breed were lifted over to the human genome hg18 coordinates (genome.ucsc.edu/cgi-bin/hgLiftOver) with 50kb flanks on each side (S17 Table). Pathway analyses were performed with the web-based program GRAIL [30] using the PubMed text (Aug 2012) database on the genomic regions with gene size correction turned on. Connectivity between loci was tested in all breeds combined for IgA associated regions. The key terms were presented in a ranking order based on both the uniqueness and specificity of the term itself together with the number of genes in the regions involved in the particular pathway. In addition, each gene was given a raw GRAIL p-value and the gene within each region with the best connectivity (i.e. lowest raw GRAIL p-value) was also given a corrected GRAIL p-value. Since only a subset of the genes had a corrected p-value, we considered a raw GRAIL p < 0.05 significant.

#### INRICH

Gene set enrichment testing was performed with INRICH (INterval enRICHment analysis), using 1,000,000 permutations to test the IgA associated regions for enrichments in gene sets from the GO catalogue (downloaded from the INRICH website on 14 October 2014)[65]. We restricted the gene sets tested to between 5 and 1,000 genes in order to exclude the widest GO terms. We used a reference map file of 17,099 genes lifted over to Canfam3.1 from the hg19 RefSeq Gene catalogue using the Liftover utility (downloaded from http://hgdownload.cse.ucsc.edu/admin/exe/) with minMatch set to 0.45. We used the SNP map file before QC (Canfam3.1: 172,950 markers).

## Supporting Information

S1 Fig Results from each of the four GWAS runs in GSD. Panel A-D presents the GWAS results and quantile-quantile plots from GSD in 2, 3, 4, 5 percentile groups, respectively.
(PDF)

S2 Fig Results from each of the four GWAS runs in GR. Panel A-D presents the GWAS results and quantile-quantile plots from GR in 2, 3, 4, 5 percentile groups, respectively.
(PDF)

S3 Fig Results from each of the four GWAS runs in LR. Panel A-D presents the GWAS results and quantile-quantile plots from LR in 2, 3, 4, 5 percentile groups, respectively.
(PDF)

S4 Fig Results from each of the four GWAS runs in SP. Panel A-D presents the GWAS results and quantile-quantile plots from SP in 2, 3, 4, 5 percentile groups, respectively.
(PDF)

S5 Fig The IgA level distribution and the combined GWAS results for each breed (same as Fig 1) including quantile-quantile plots. Panel A-D presents the distribution of IgA levels (0–1.40 g/l) as relative frequency (%) and as box plots with the black box marking percentile 25 to 75 and red bar the median, in GSD, GR, LR and SP respectively. The combined GWAS analyses from four runs (IgA levels divided into 2, 3, 4 and 5 groups) are presented in panel E-H (in GSD, GR, LR and SP, respectively. Panel I-L shows the combined quantile-quantile plots for GSD, GR, LR and SP, respectively.
(PDF)

S6 Fig The genome-wide significantly associated regions on CFA8 and CFA23 in GSD. Zooming in on the significantly associated regions (significant SNP in white) including the genes from the UCSC browser on CFA8 (A) and CFA23 (B).
(PDF)

S7 Fig The region on CFA28 including full-fixation tracks in GSD and human (hg19) liftover. The proportion of variants with variation in 20 GSDs in sliding-windows (11 variants per window, 1 variant overlap) is presented in A. Panel B shows a distinct increase in the degree of genetic differentiation (F_ST_) between dogs and wolves spanning a 75-kb region (windows with F_ST_ > 0.67 are coloured in black) within the *SLIT1* locus. F_ST_ values of two consecutive 50-kb windows reached 0.68 and 0.67, respectively (windows with F_ST_ > 0.43 are coloured in grey). Additionally, panel B presents the positions of the top-associated SNPs in SP, black bars representing blocks of fixed variants in GSD (based on panel A) and the positions of CTCF transcription factor binding sites based on the human (hg19) Transcription Factor ChIP-seq from ENCODE V2 and the genes in the region. The corresponding region in human hg19 with the full track from Transcription Factor ChIP-seq from ENCODE V2 is presented in panel C.
(PDF)

S8 Fig Quantile-quantile plots after 1,000 permutations in GSD and GR. Panel A-D presents the results for GSD and panel E-H for GR from 1,000 permutations in 2, 3, 4, 5 percentile groups, respectively.
(PDF)

S9 Fig Quantile-quantile plots after 1,000 permutations in LR and SP. Panel A-D presents the results for LR and panel E-H for SP from 1,000 permutations in 2, 3, 4, 5 percentile groups, respectively.
(PDF)

S10 Fig The proportion of variants with variation in GSD and LR within the SP associated region on CFA28. By using a sliding window of 11 and 25 (for comparison) variants in LR (A-B) and GSD (C-D), across 400 kb on CFA28, the proportion of variants with variation was measured. Each bar representing one variant; 0 means no variation and 1 that all variants in the window are polymorphic. Panel E shows the location of the top four SNPs in SP and the genes in the UCSC browser.
(PDF)

S1 Table Age and sex correlation in four breeds and subpopulations in GSD. (PDF)

S2 Table Breakdown of samples and markers in GWAS. (PDF)

S3 Table Frequencies of the top SNPs in the IgA associated regions. (XLSX)

S4 Table Allele frequencies based on 20 GSDs across CFA28: 9,000,091–11,999,424. (PDF)

S5 Table Allele frequencies based on 20 LRs across CFA28: 9,000,091–11,999,424. (PDF)

S6 Table Fixation using 11 variants per windows (1 variant overlap) in 20 GSDs across CFA28: 9,000,091–11,999,424. (XLSX)

S7 Table Fixation using 11 variants per windows (1 variant overlap) in 20 LRs across CFA28: 9,000,091–11,999,424. (XLSX)

S8 Table F_ST_ values comparing dog and wolf pools in the CFA28 region. (PDF)

S9 Table Z-transformed average pooled heterozygosity in dog in the CFA28 region. (PDF)

S10 Table Complete GRAIL pathway results from IgA associated regions. (PDF)

S11 Table INRICH results. (PDF)

S12 Table The complete output from INRICH. (PDF)

S13 Table Descriptive statistics for IgA in the four breeds. (PDF)

S14 Table IgA intervals (and number of individuals) used in GWAS. (PDF)

S15 Table The complete set of control breeds. (PDF)

S16 Table Allele frequencies based on eight breeds (160 dogs) across CFA28: 9,000,091–11,999,424. (PDF)

S17 Table Coordinates in canfam2.0, hg18 and canfam3.0 for all IgA associated regions for GRAIL and INRICH analyses. (PDF)
