# Correction: Genome-Wide Analyses Suggest Mechanisms Involving Early B-Cell Development in Canine IgA Deficiency

**DOI:** 10.1371/journal.pone.0138405

**Published:** 2015-09-14

**Authors:** Mia Olsson, Katarina Tengvall, Marcel Frankowiack, Marcin Kierczak, Kerstin Bergvall, Erik Axelsson, Linda Tintle, Eliane Marti, Petra Roosje, Tosso Leeb, Åke Hedhammar, Lennart Hammarström, Kerstin Lindblad-Toh

The Data Availability statement for this paper is incorrect. The correct statement is: All genotypes and phenotypes are available from the Dryad Digital Repository: http://dx.doi.org/10.5061/dryad.pm7mt
