# Supplementary figures and images for: Genome-Wide Analyses Suggest Mechanisms Involving Early B-Cell Development in Canine IgA Deficiency
Source: PLoS One. 2015 Jul 30;10(7):e0133844. doi: 10.1371/journal.pone.0133844 (PMC4520476; doi:10.1371/journal.pone.0133844)

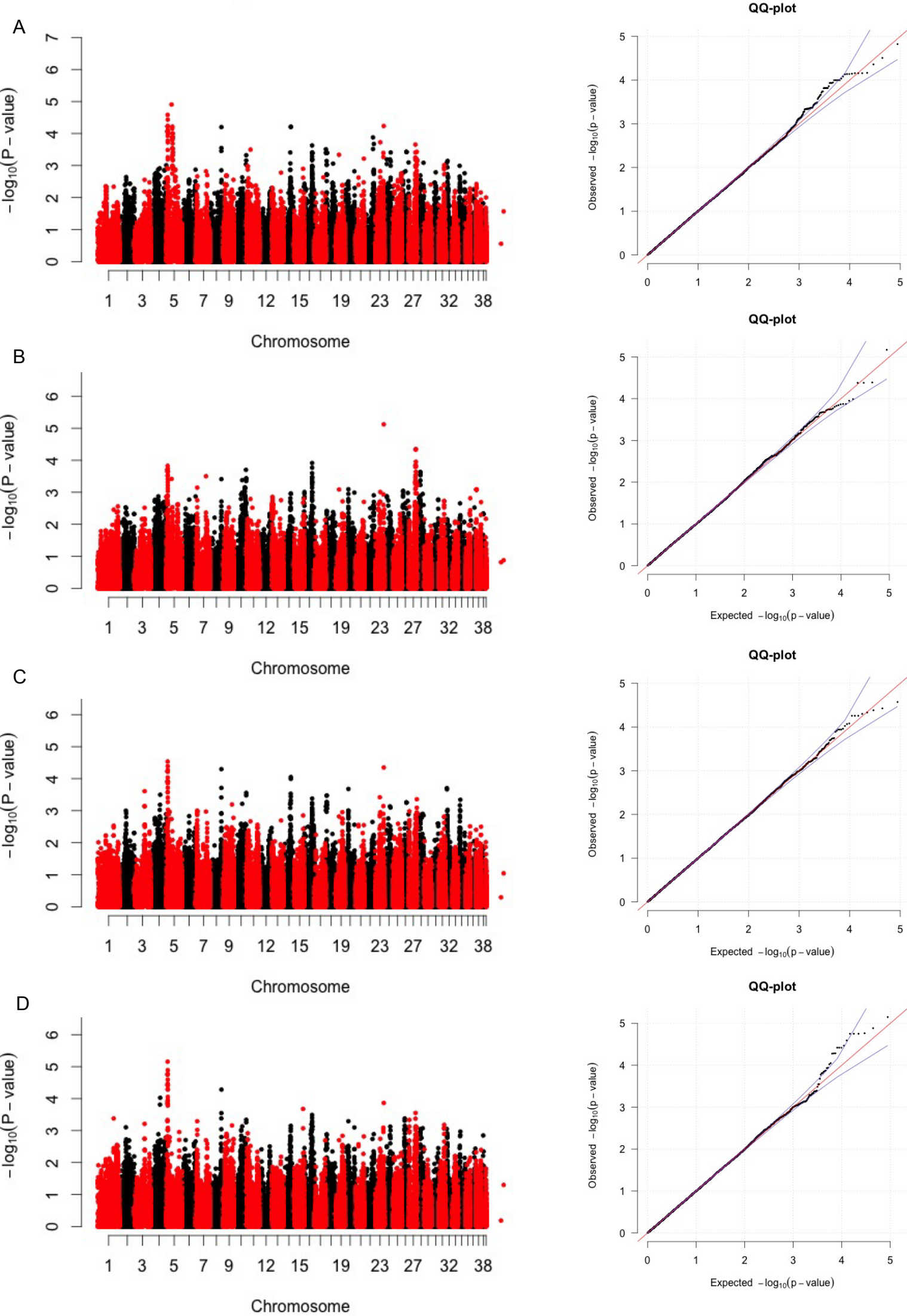

Supplement: S1 Fig — Panel A-D presents the GWAS results and quantile-quantile plots from GSD in 2, 3, 4, 5 percentile groups, respectively. (PDF) [file pone.0133844.s001.pdf]

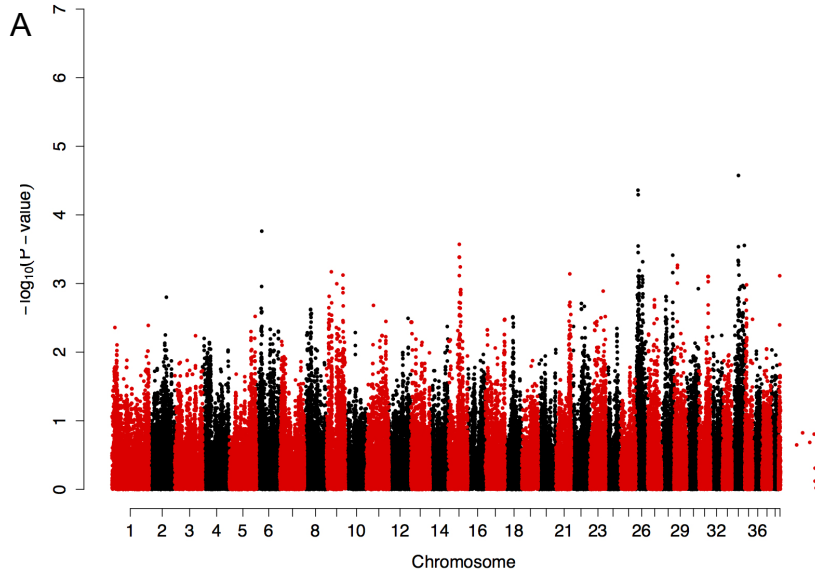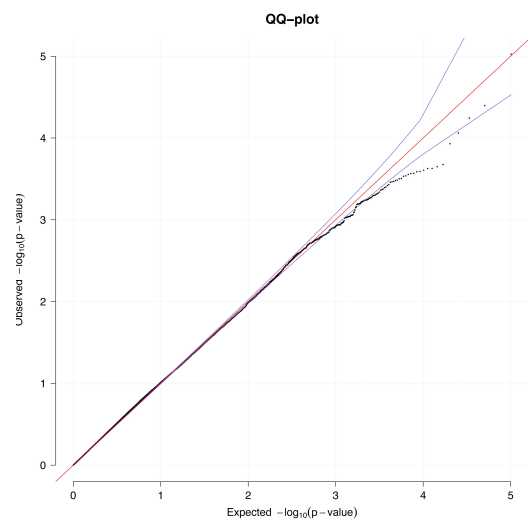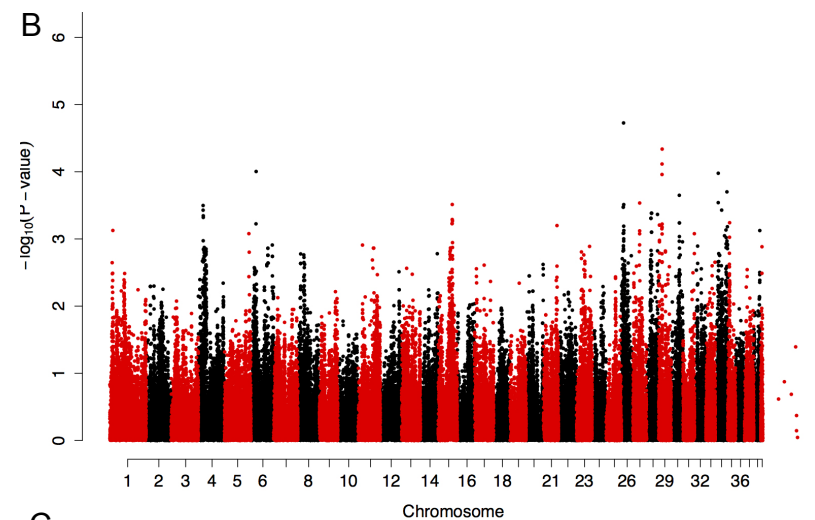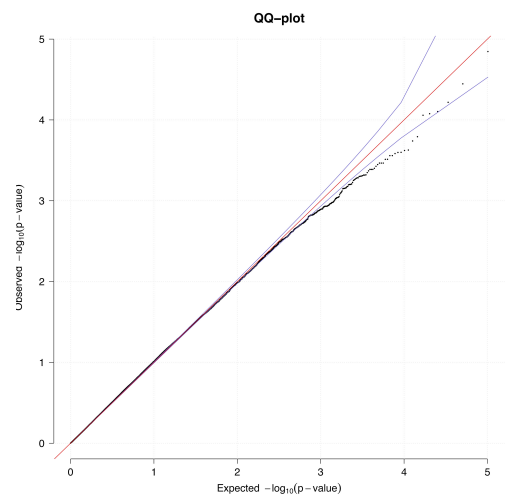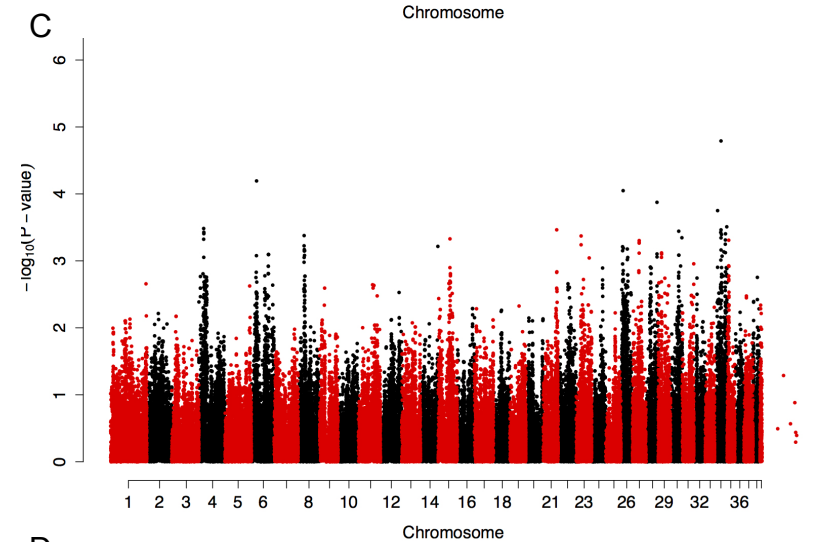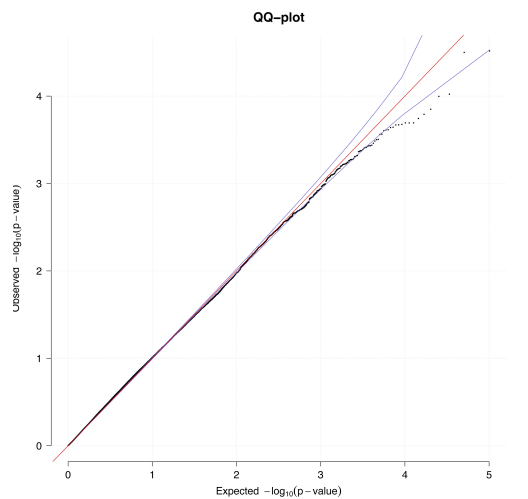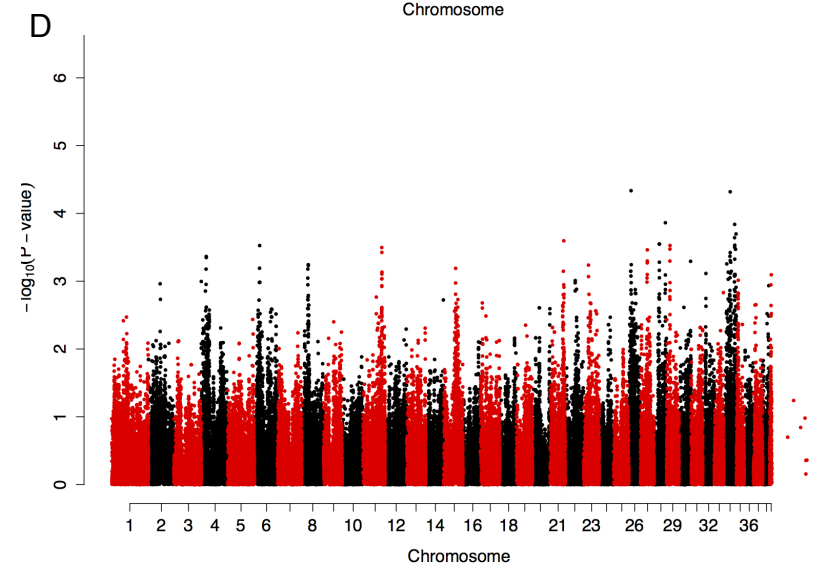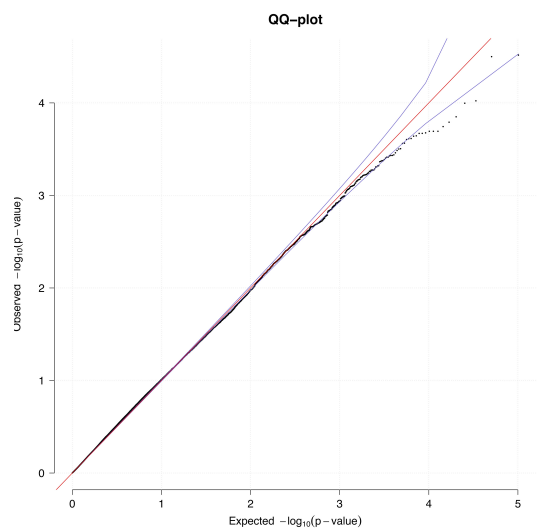

Supplement: S2 Fig — Panel A-D presents the GWAS results and quantile-quantile plots from GR in 2, 3, 4, 5 percentile groups, respectively. (PDF) [file pone.0133844.s002.pdf]

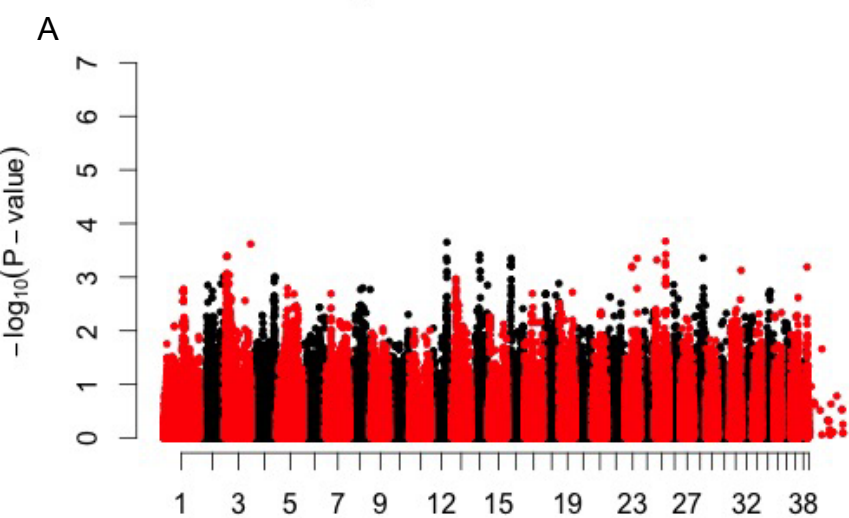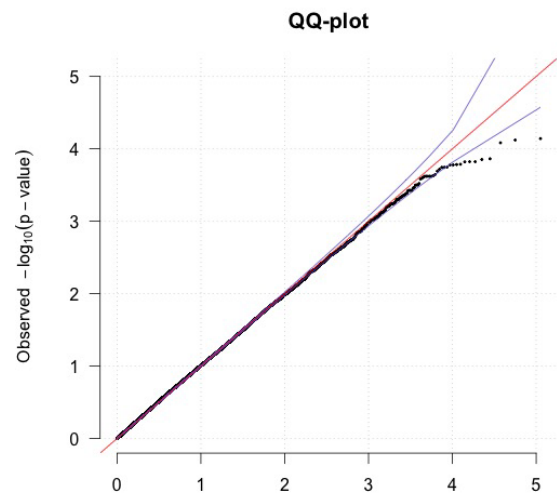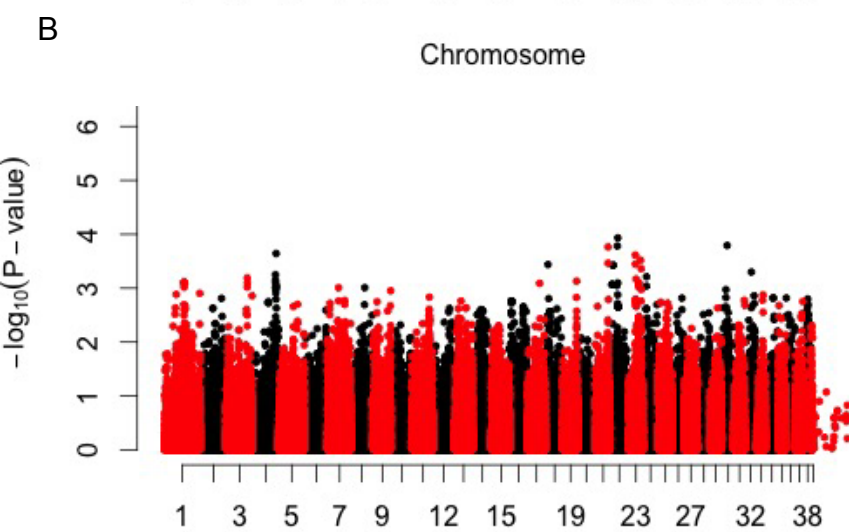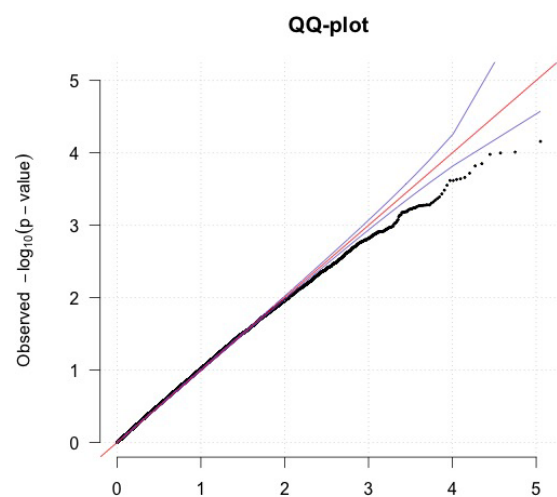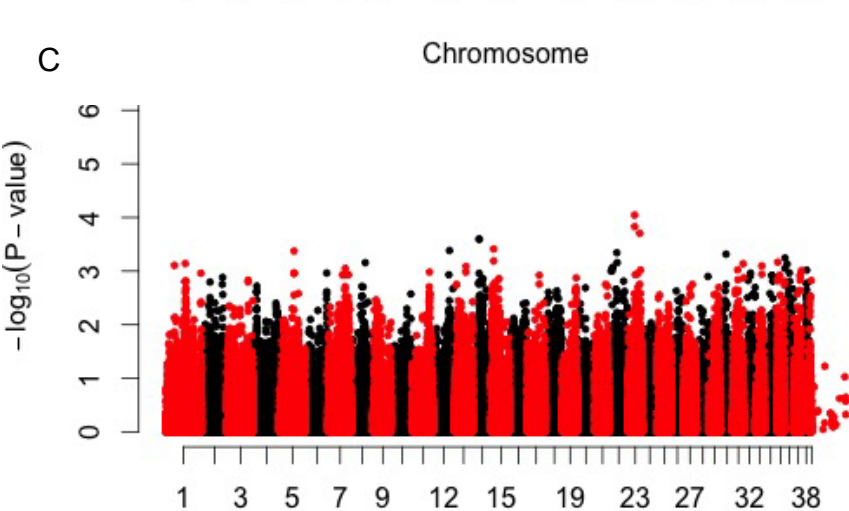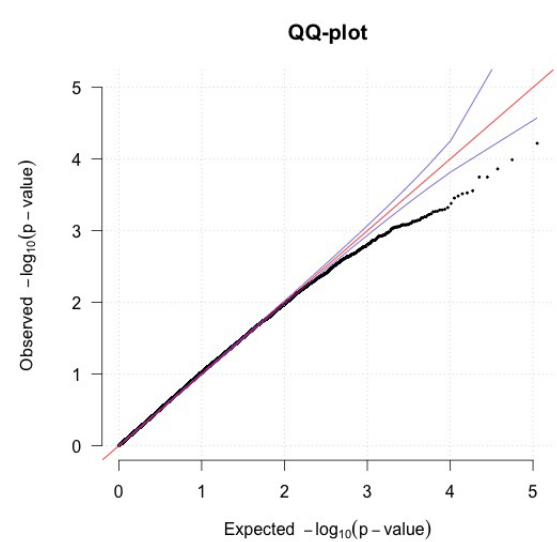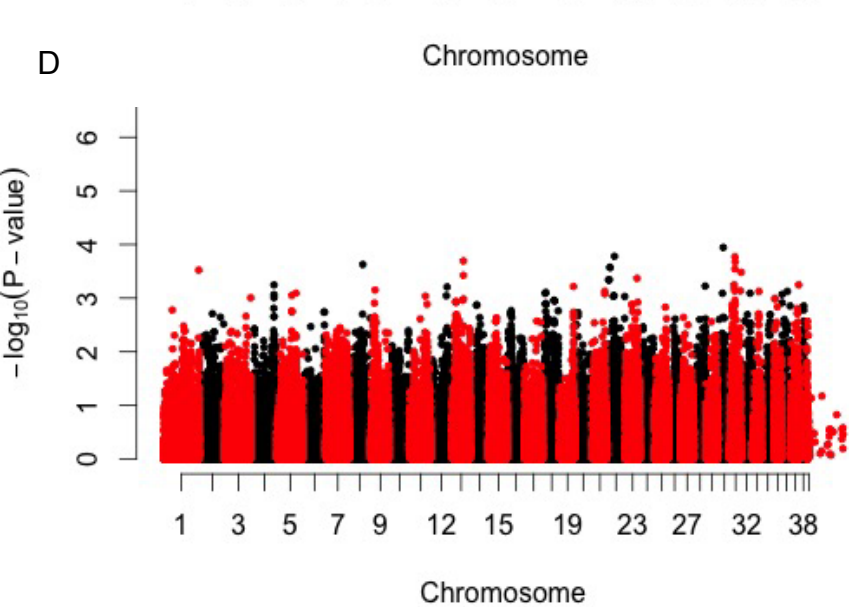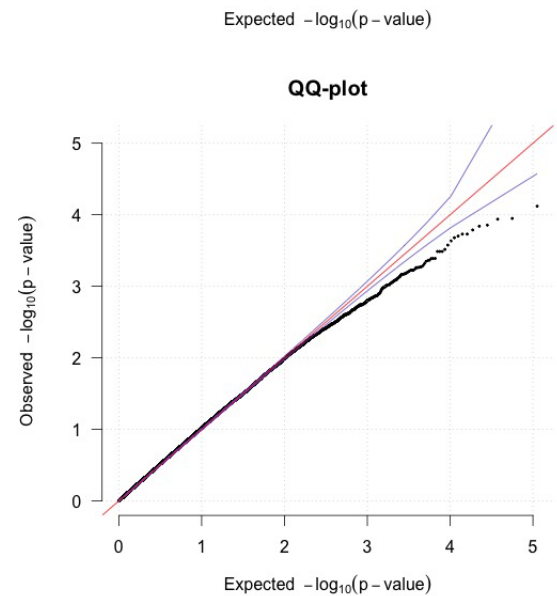

Supplement: S3 Fig — Panel A-D presents the GWAS results and quantile-quantile plots from LR in 2, 3, 4, 5 percentile groups, respectively. (PDF) [file pone.0133844.s003.pdf]

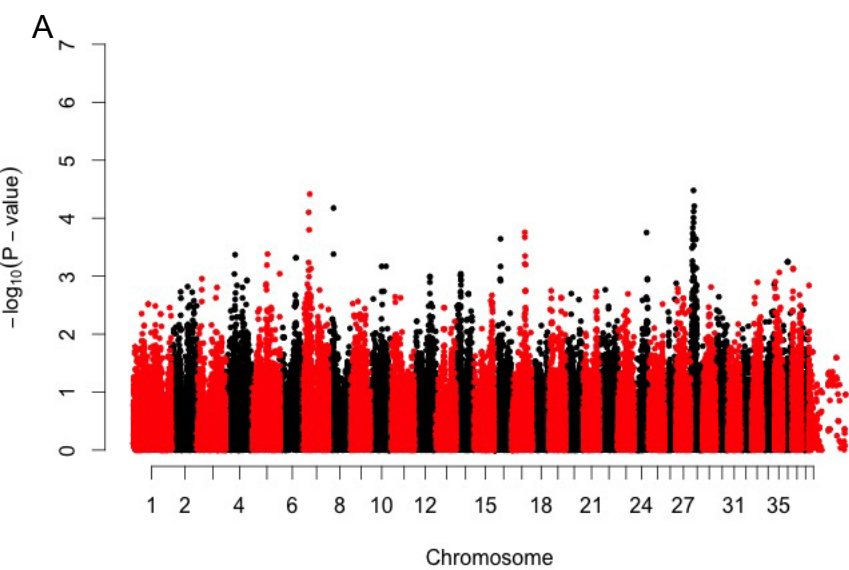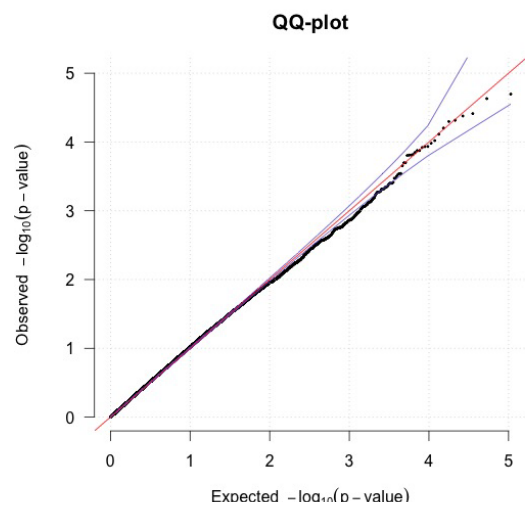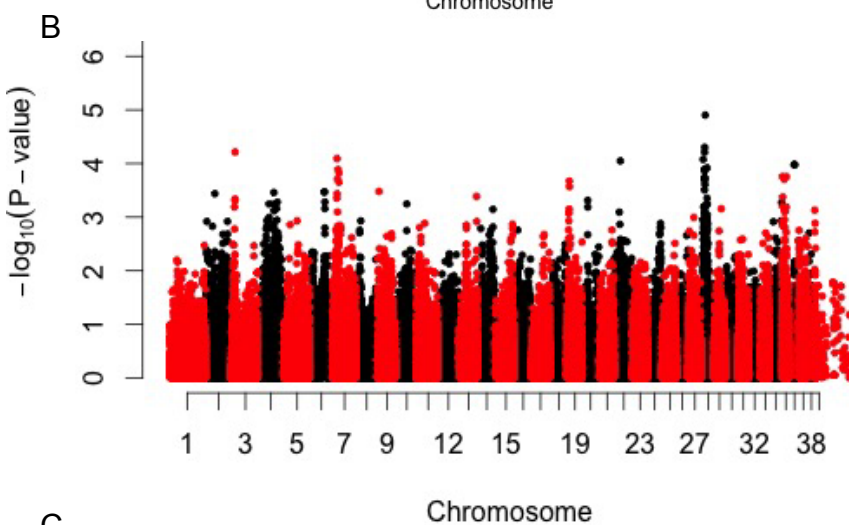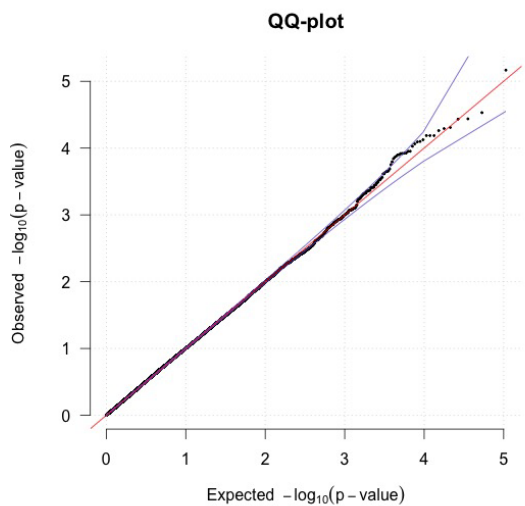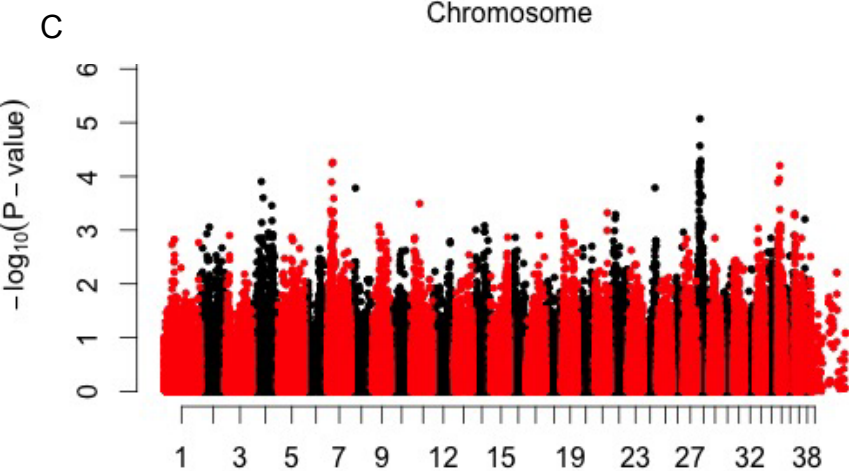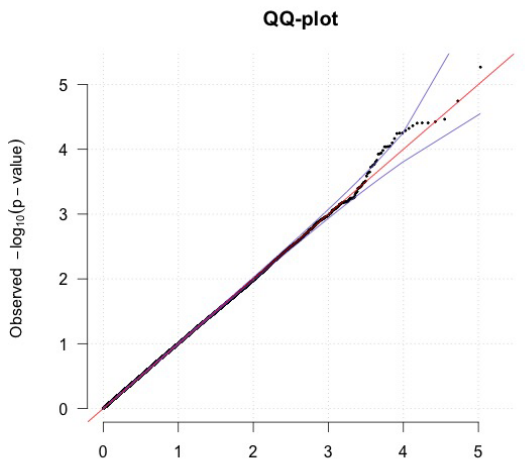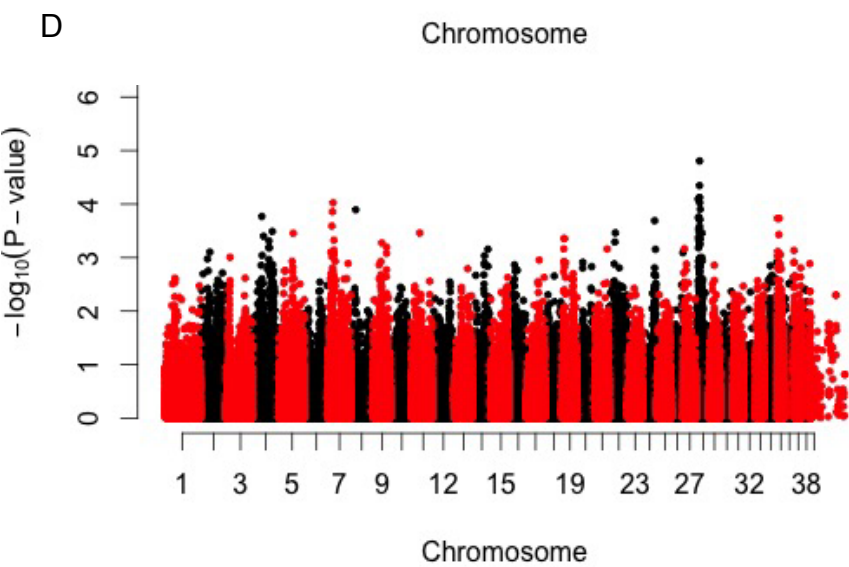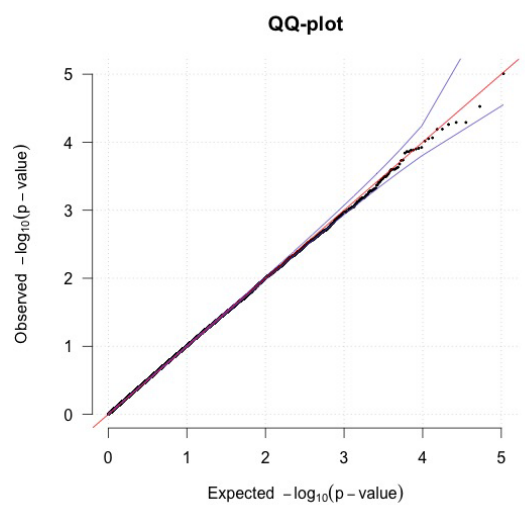

Supplement: S4 Fig — Panel A-D presents the GWAS results and quantile-quantile plots from SP in 2, 3, 4, 5 percentile groups, respectively. (PDF) [file pone.0133844.s004.pdf]

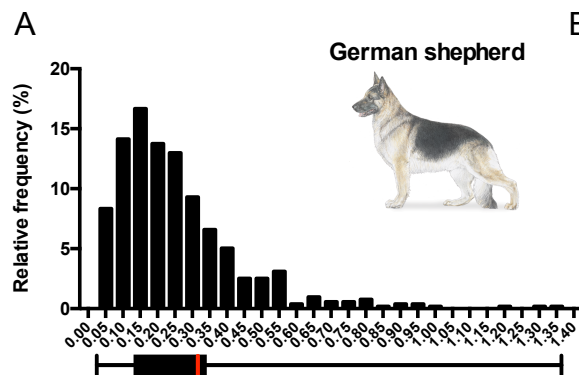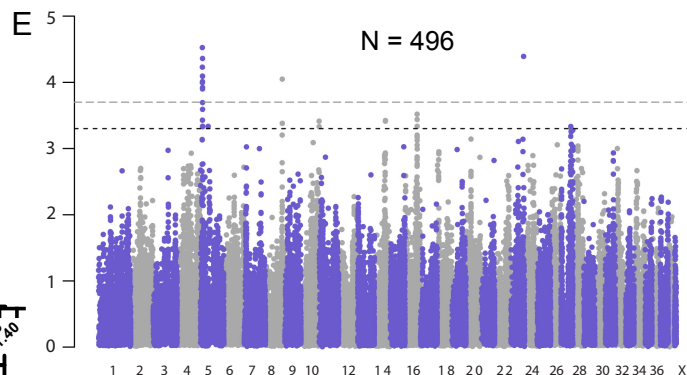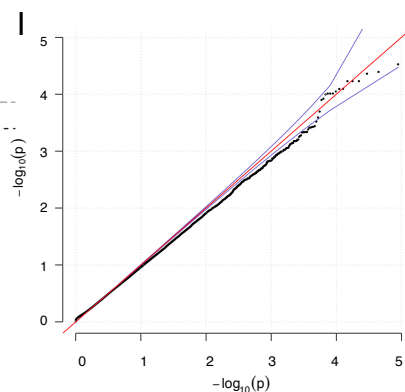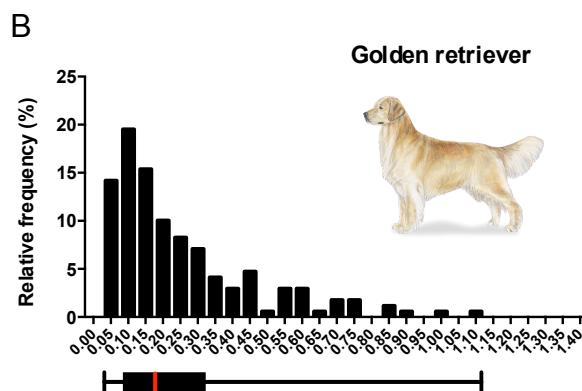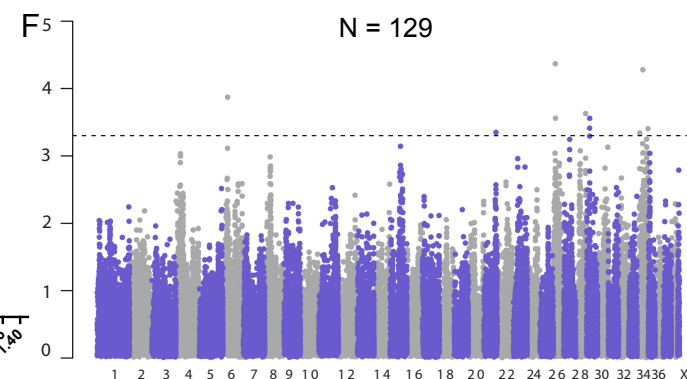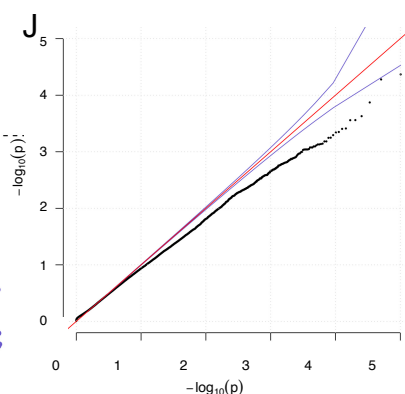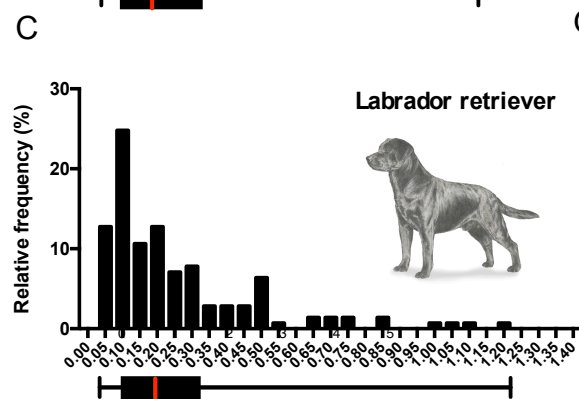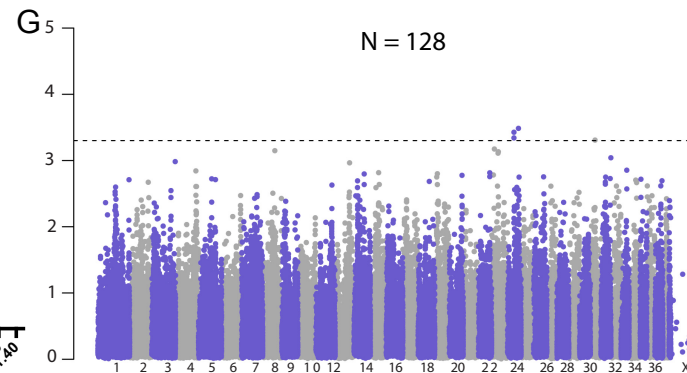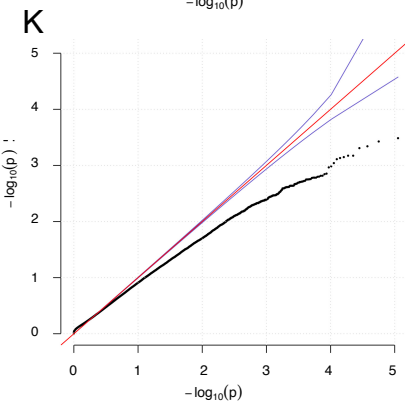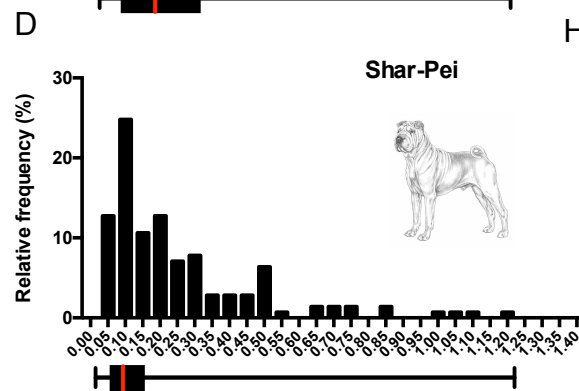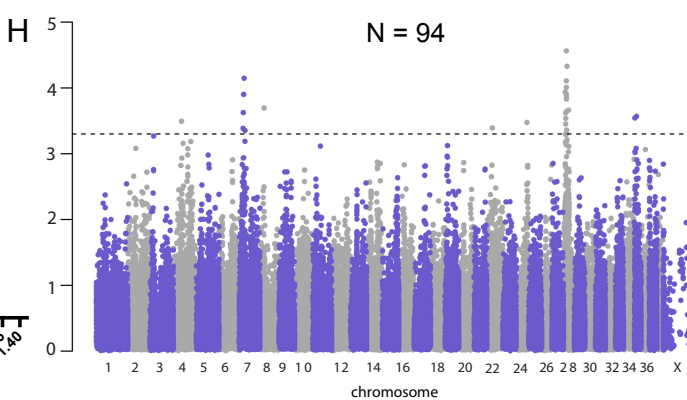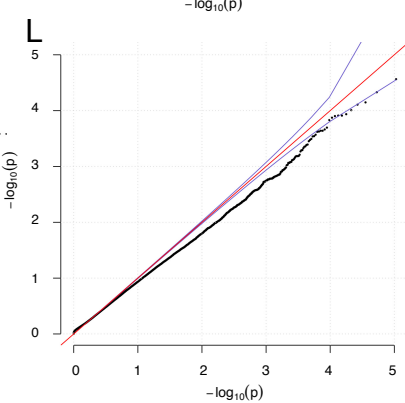

Supplement: S5 Fig — Panel A-D presents the distribution of IgA levels (0–1.40 g/l) as relative frequency (%) and as box plots with the black box marking percentile 25 to 75 and red bar the median, in GSD, GR, LR and SP respectively. The combined GWAS analyses from four runs (IgA levels divided into 2, 3, 4 and 5 groups) are presented in panel E-H (in GSD, GR, LR and SP, respectively. Panel I-L shows the combined quantile-quantile plots for GSD, GR, LR and SP, respectively. (PDF) [file pone.0133844.s005.pdf]

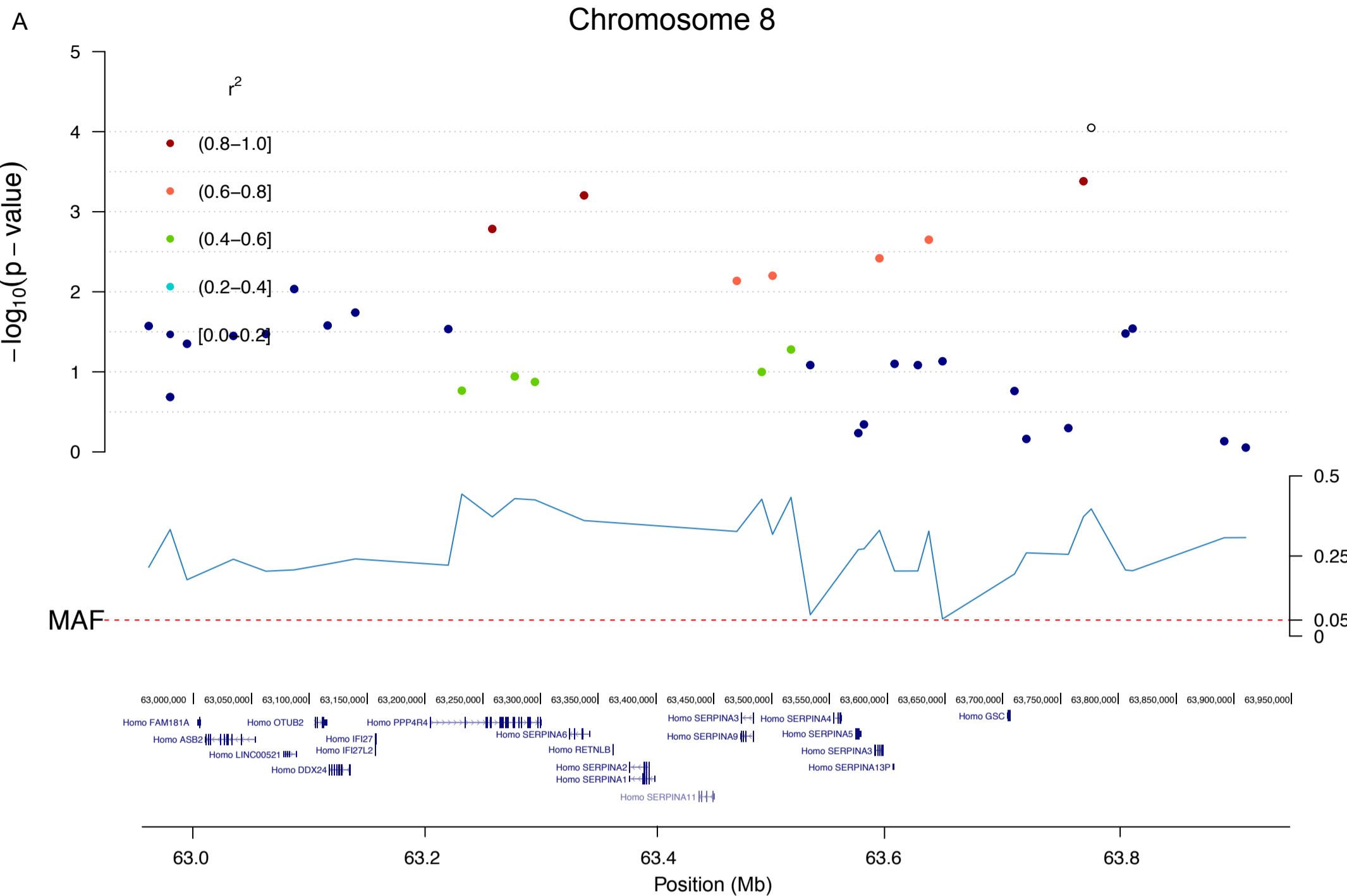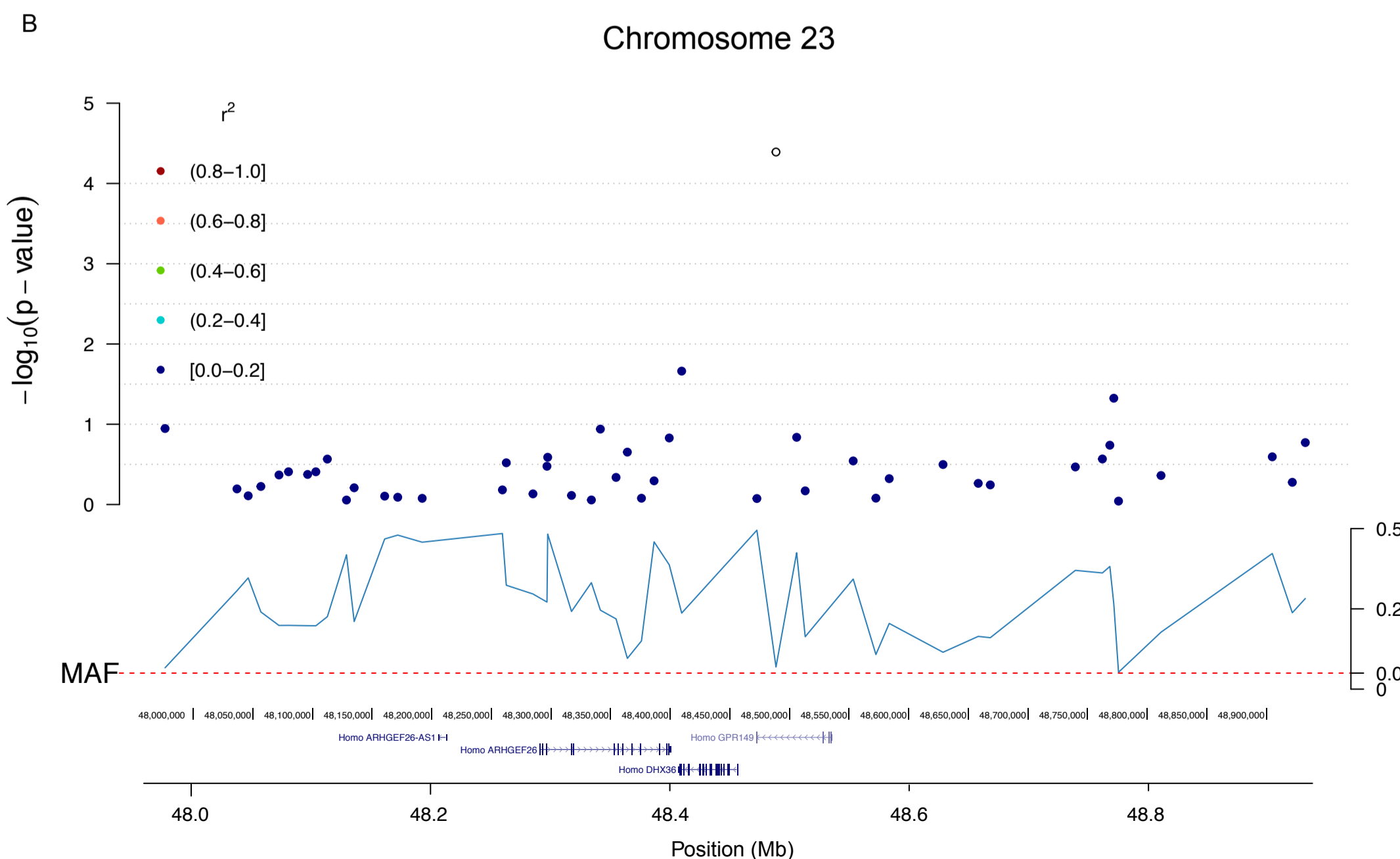

Supplement: S6 Fig — Zooming in on the significantly associated regions (significant SNP in white) including the genes from the UCSC browser on CFA8 (A) and CFA23 (B). (PDF) [file pone.0133844.s006.pdf]

A

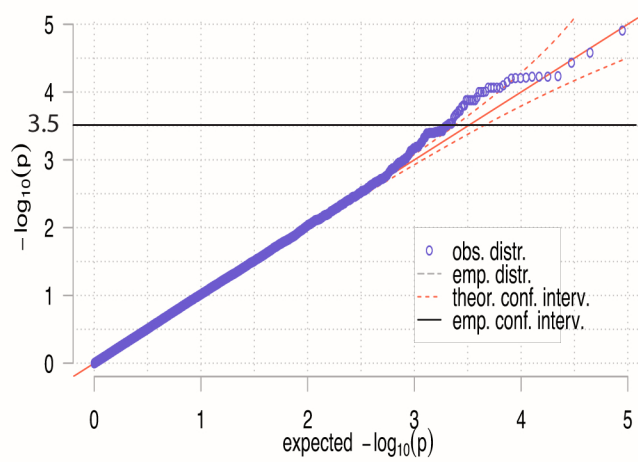

B

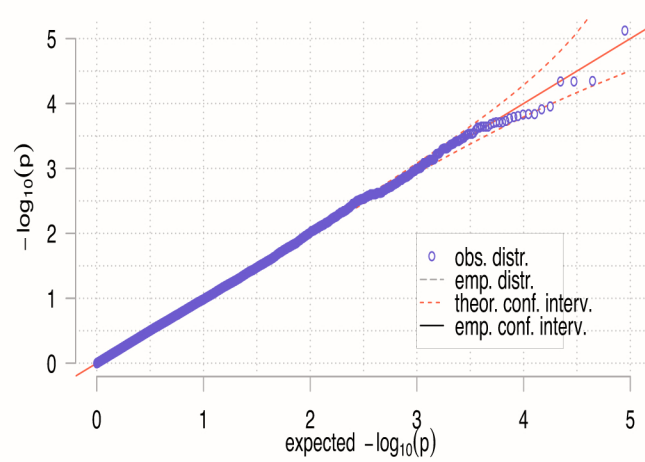

C

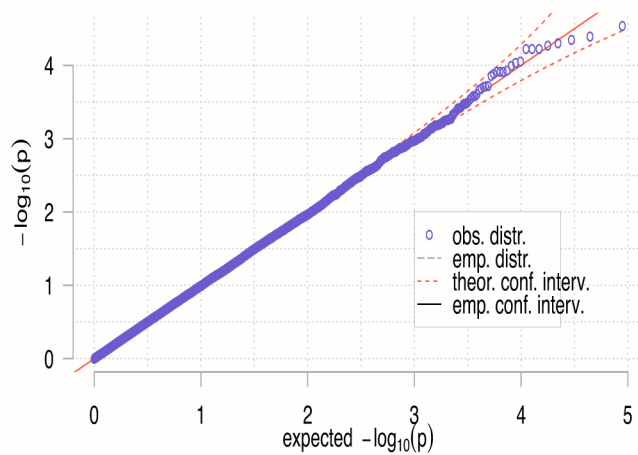

D

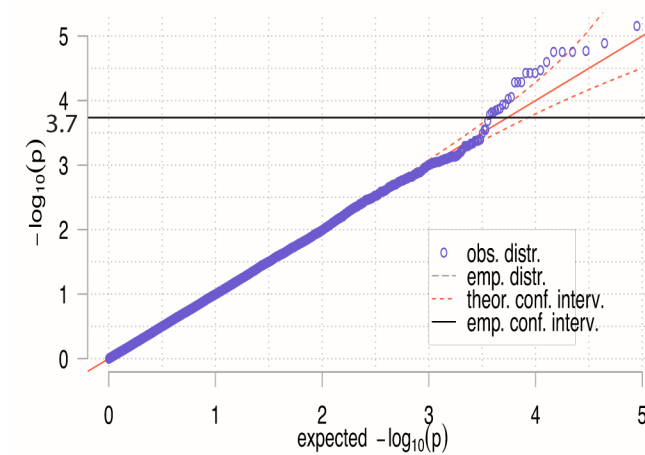

E

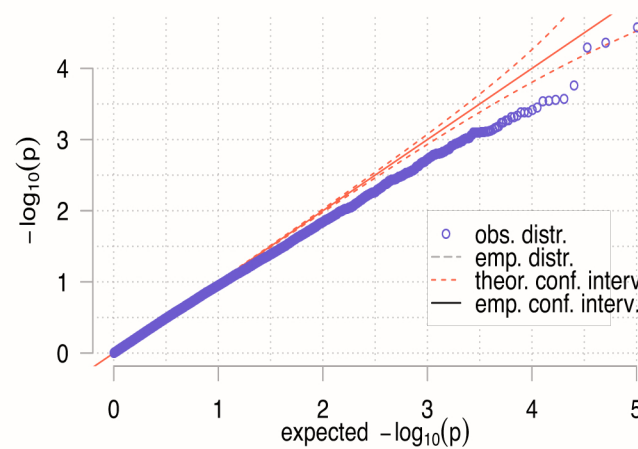

F

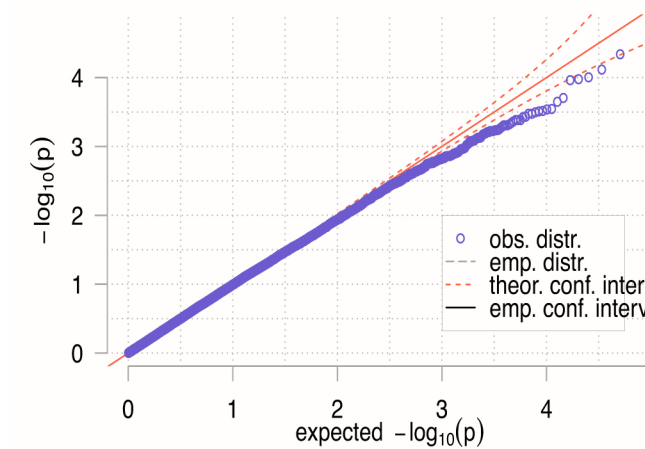

G

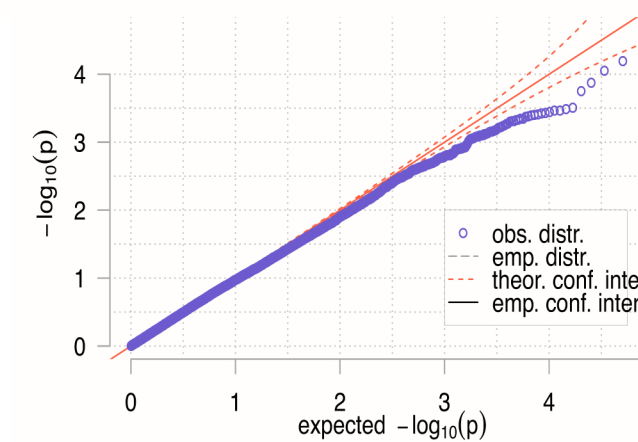

H

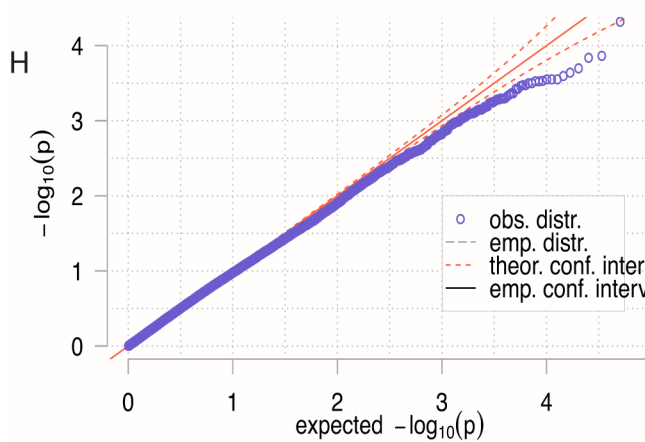

Supplement: S8 Fig — Panel A-D presents the results for GSD and panel E-H for GR from 1,000 permutations in 2, 3, 4, 5 percentile groups, respectively. (PDF) [file pone.0133844.s008.pdf]

A

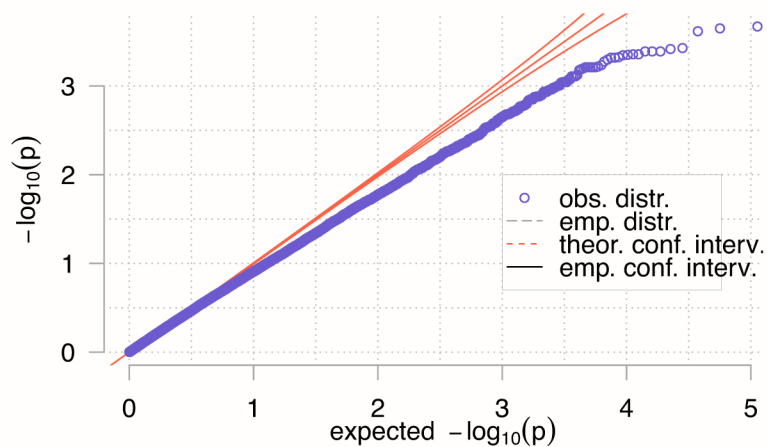

B

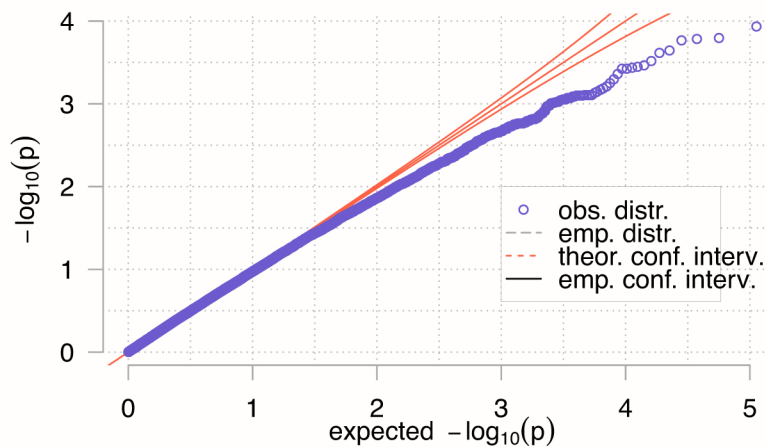

C

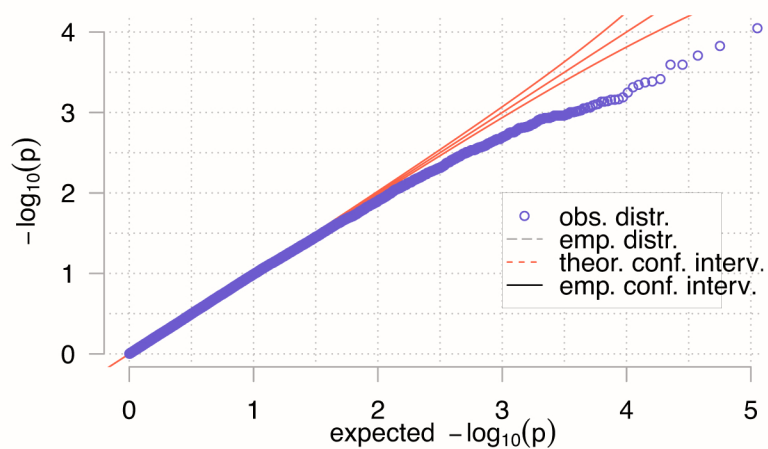

D

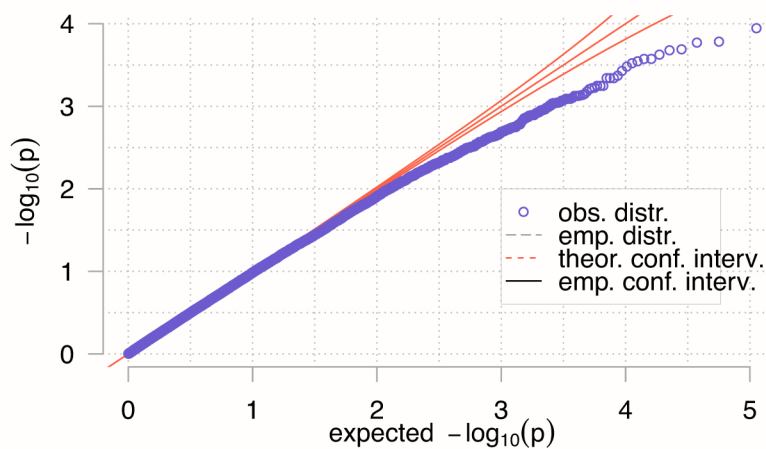

E

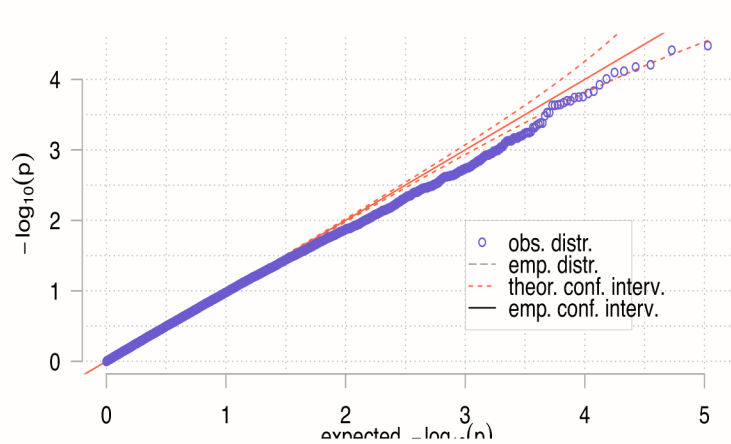

F

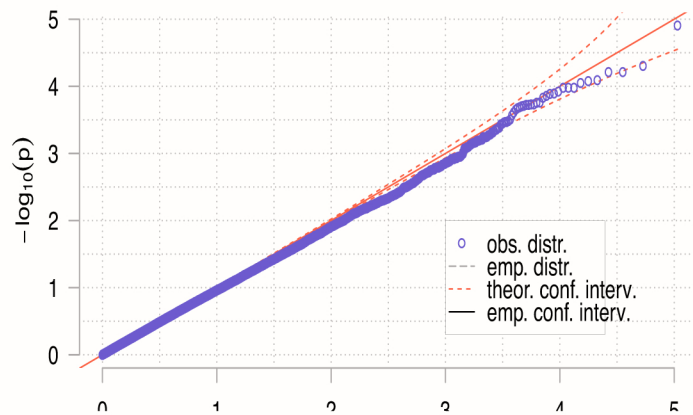

G

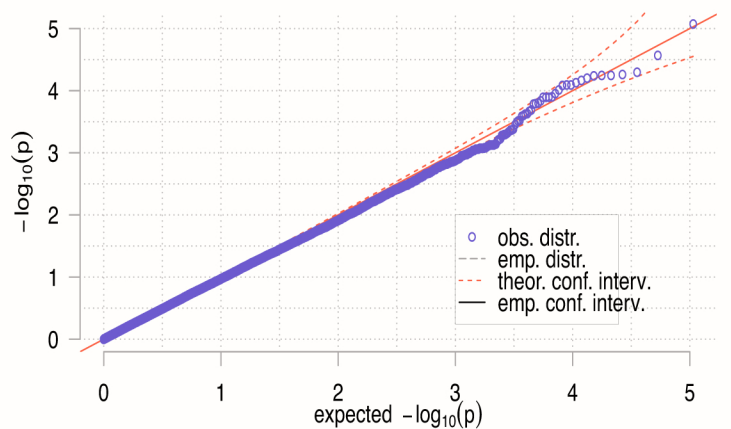

H

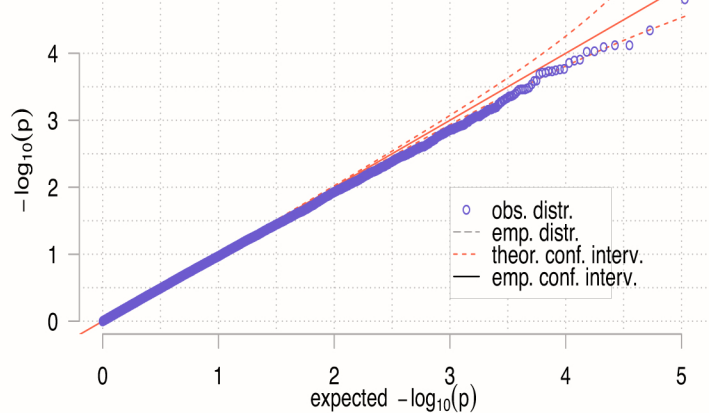

Supplement: S9 Fig — Panel A-D presents the results for LR and panel E-H for SP from 1,000 permutations in 2, 3, 4, 5 percentile groups, respectively. (PDF) [file pone.0133844.s009.pdf]

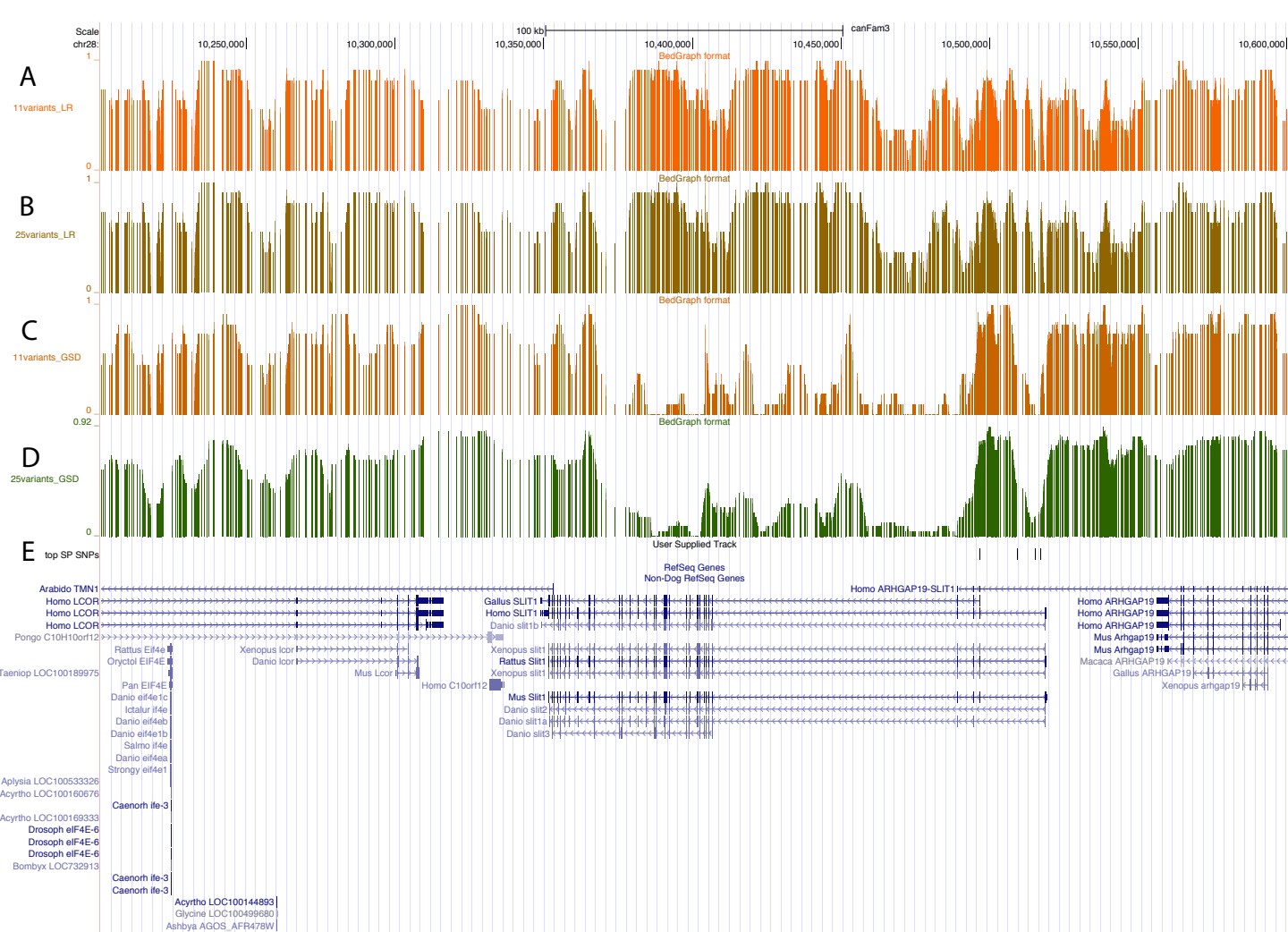

Supplement: S10 Fig — By using a sliding window of 11 and 25 (for comparison) variants in LR (A-B) and GSD (C-D), across 400 kb on CFA28, the proportion of variants with variation was measured. Each bar representing one variant; 0 means no variation and 1 that all variants in the window are polymorphic. Panel E shows the location of the top four SNPs in SP and the genes in the UCSC browser. (PDF) [file pone.0133844.s010.pdf]
