# Supplementary material for: Genome-Wide Analyses Suggest Mechanisms Involving Early B-Cell Development in Canine IgA Deficiency
Source: PLoS One. 2015 Jul 30;10(7):e0133844. doi: 10.1371/journal.pone.0133844 (PMC4520476; doi:10.1371/journal.pone.0133844)
Supplement: S1 Table — (PDF) [file pone.0133844.s011.pdf]

Table S1. Age and sex correlation in four breeds and subpopulations in German shepherd

	Pearson's product-moment correlation				Welch Two sample t-test		
	Age correlation	Age p-value	Sex correlation	Sex p-value	subpop1 mean	subpop2 mean	p-value (difference)
**GSD**							
Five percentile groups	0.2024165	5.526e-06	0.02855347	0.5258	3.071942	2.802752	0.03555
Four percentile groups	0.1915557	1.745e-05	0.008781387	0.8453	2.553957	2.362385	0.06232
Three percentile groups	0.1800242	5.526e-05	0.005338612	0.9056	2.043165	1.908257	0.06754
Two percentile groups	0.2525495	3.541e-05	0.007779909	0.9003	0.5395683	0.4390244	0.105
**GR**					no subpop		
Five percentile groups	0.3568886	3.302e-05	0.01597527	0.8367			
Four percentile groups	0.3637513	2.262e-05	-0.01893164	0.807			
Three percentile groups	0.3552561	3.608e-05	-0.03545191	0.6472			
Two percentile groups	0.4542228	5.426e-05	-0.01550264	0.8802	no subpop		
**LR**							
Five percentile groups	0.5008708	1.732e-09	0.1129993	0.1822			
Four percentile groups	0.5081548	9.182e-10	0.109205	0.1974			
Three percentile groups	0.4356144	2.761e-07	0.07357515	0.3859			
Two percentile groups	0.6270826	8.165e-09	0.1323422	0.2512			
**SP**					no subpop		
Five percentile groups	0.3016215	0.003133	0.03741546	0.7203			
Four percentile groups	0.3035804	0.002935	0.006011288	0.9541			
Three percentile groups	0.2745766	0.007402	0.006632365	0.9494			
Two percentile groups	0.3720542	0.006083	-0.06609195	0.6382			
