# Supplementary material for: Genome-Wide Analyses Suggest Mechanisms Involving Early B-Cell Development in Canine IgA Deficiency
Source: PLoS One. 2015 Jul 30;10(7):e0133844. doi: 10.1371/journal.pone.0133844 (PMC4520476; doi:10.1371/journal.pone.0133844)
Supplement: S2 Table — (PDF) [file pone.0133844.s012.pdf]

Table S2. Breakdown of sample and markers in GWAS

		SNPs and individuals genotyped	SNPs and individuals removed due to low call rate (<95%)	SNPs removed due to low MAF (<0.05)	SNP removed due to deviation from HWE (p<1e-06%)	Individuals removed due to FDR**<1%	Individuals removed due to IBS*** >=0.95	Individuals removed due to trait or covariate missing	**Final nr. of SNPs and individuals in GWAS analyses - 3, 4, 5 groups analyses**	Final nr. of SNPs and individuals in GWAS analyses - cases vs controls
GSD	markers	173662	10298	70127	7355	-	-	-	**88704 (51%)**	88704
	dogs	516	14	-	-	1	6	0	**496**	262
GR	markers	174376	4021	66538	4719	-	-	-	**100680 (58%)**	100680
	dogs	187	0	-	-		2	58	**129**	73
LR	markers	174376	3197	54937	4794	-	-	-	**112428 (64%)**	112428
	dogs	141*	0	-	-	0	0	13	**128**	69
SP	markers	173662	9631	55678	3116	-	-	-	**106622 (61%)**	106622
	dogs	96	2	-	-	1	0	0	**94**	53

**Out of 302 genotyped individuals 141 were analyzed for IgA, thus only these were included in the QC*

***Too high autosomal heterozygosity*

****Mean IBS based on 2000 autosomal markers*

Note: SNPs and individuals left may be higher than the subtraction due to group overlap between exclusion criteria
