# Supplementary material for: Genome-Wide Analyses Suggest Mechanisms Involving Early B-Cell Development in Canine IgA Deficiency
Source: PLoS One. 2015 Jul 30;10(7):e0133844. doi: 10.1371/journal.pone.0133844 (PMC4520476; doi:10.1371/journal.pone.0133844)
Supplement: S4 Table — (PDF) [file pone.0133844.s014.pdf]

Table S4. Allele frequencies based on 20 German shepherds across chr28: 9000091-11999424 (canfam3)

CHROM	POS	N_ALLELES	N_CHR	{ALLELE:FREQ}				
chr28	9000091	2	38	T:1	C:0			
chr28	9000246	2	36	T:1	TCAA:0			
chr28	9000473	2	38	A:1	G:0			
chr28	9000522	2	40	T:1	C:0			
chr28	9000657	2	36	C:1	CTTTAT:0			
chr28	9000789	2	38	T:1	C:0			
chr28	9000939	2	40	CGG:1	C:0			
chr28	9001032	2	40	C:1	G:0			
chr28	9001109	2	36	C:1	G:0			
chr28	9001229	5	40	GAGAGAAGAGA:0.075	G:0.125	GAGAGA:0.225	GAGAGAAGAGAAGAGA:0.425	GAGAGAAGAGAAGAGAAGAGAAGAGAAGAGA:0.15
chr28	9001674	2	40	G:1	A:0			
chr28	9001787	2	36	CA:1	C:0			
chr28	9001833	2	40	A:1	G:0			
chr28	9001982	2	36	AT:1	A:0			
chr28	9002236	2	36	ATCCAGGAC:1	A:0			
chr28	9002267	2	36	C:1	T:0			
chr28	9002509	2	36	C:0.833333	T:0.166667			
chr28	9002784	2	38	A:1	G:0			
chr28	9002831	2	38	C:1	T:0			
chr28	9003437	2	38	T:1	C:0			
chr28	9003632	2	36	G:1	GCTCT:0			
chr28	9003633	2	36	C:1	CTCTT:0			
chr28	9003904	2	38	C:1	T:0			
chr28	9004320	2	38	A:1	C:0			
chr28	9004520	2	40	A:1	C:0			
chr28	9004564	2	38	A:1	G:0			
chr28	9004919	2	32	T:1	C:0			
chr28	9005309	2	38	A:1	G:0			
chr28	9005338	2	36	T:1	C:0			
chr28	9005502	2	38	G:1	C:0			
chr28	9005638	2	40	G:1	A:0			
chr28	9005688	2	40	A:1	C:0			
chr28	9006019	2	36	A:1	AC:0			
chr28	9006568	2	36	T:0.833333	TA:0.166667			
chr28	9007042	2	32	A:1	C:0			
chr28	9007529	2	40	CA:1	C:0			
chr28	9008103	2	36	T:1	C:0			
chr28	9008419	2	38	C:1	T:0			
chr28	9008506	2	36	CAGAG:1	C:0			
chr28	9008594	2	32	C:1	T:0			
chr28	9008801	2	38	C:1	CA:0			
chr28	9008900	2	38	T:0.973684	TA:0.0263158			
chr28	9009257	2	36	A:0.944444	G:0.0555556			
chr28	9009318	2	36	G:1	A:0			
chr28	9009369	2	38	AAC:1	A:0			
chr28	9009943	2	34	C:1	T:0			
chr28	9010058	2	38	GTT:1	G:0			
chr28	9010471	2	32	G:0.96875	A:0.03125			
chr28	9011133	2	38	A:1	C:0			

chr28	9011297	2	38	G:1	A:0			
chr28	9011572	2	38	A:1	T:0			
chr28	9011586	2	36	TGTA:1	T:0			
chr28	9012020	2	40	CCTTTCTTTCT:0.9	C:0.1			
chr28	9012155	2	38	C:1	T:0			
chr28	9012207	2	38	GCTC:1	G:0			
chr28	9012229	2	38	G:1	T:0			
chr28	9012232	2	38	A:1	C:0			
chr28	9012244	2	38	C:1	T:0			
chr28	9012364	2	40	G:1	A:0			
chr28	9012499	2	34	C:1	T:0			
chr28	9012965	2	40	G:1	A:0			
chr28	9013095	2	38	C:1	G:0			
chr28	9013098	2	38	A:1	G:0			
chr28	9013127	2	38	T:1	C:0			
chr28	9013382	2	36	T:1	C:0			
chr28	9013443	2	38	G:1	A:0			
chr28	9013453	2	38	G:1	A:0			
chr28	9013626	2	40	C:1	T:0			
chr28	9013745	2	40	T:1	C:0			
chr28	9013848	2	40	C:1	T:0			
chr28	9013894	2	40	C:1	T:0			
chr28	9013951	2	34	C:1	G:0			
chr28	9014048	2	40	G:1	A:0			
chr28	9014089	2	38	A:1	C:0			
chr28	9014093	2	36	C:0.972222	CA:0.0277778			
chr28	9014112	2	34	T:1	C:0			
chr28	9014138	2	40	T:1	G:0			
chr28	9014282	2	38	C:1	T:0			
chr28	9014788	2	38	G:1	GTCA:0			
chr28	9014789	2	38	G:1	GGATCCTGATC:0			
chr28	9014793	2	38	C:1	T:0			
chr28	9014795	2	38	CG:1	C:0			
chr28	9014797	2	38	G:1	T:0			
chr28	9014841	2	38	C:1	T:0			
chr28	9014988	2	40	G:1	T:0			
chr28	9015209	2	36	CAT:1	C:0			
chr28	9015491	2	38	A:1	G:0			
chr28	9015823	2	40	T:1	A:0			
chr28	9016088	2	40	C:1	G:0			
chr28	9016103	2	40	A:1	G:0			
chr28	9016322	2	38	A:1	AAT:0			
chr28	9016326	2	36	CACAT:1	C:0			
chr28	9016330	5	38	T:0.421053	C:0.0263158	TAC:0.105263	TACAC:0.368421	TACACAC:0.0789474
chr28	9016360	3	38	C:1	G:0	CACAG:0		
chr28	9016682	2	34	G:1	GA:0			
chr28	9016800	2	40	T:1	A:0			
chr28	9016982	2	38	A:1	G:0			
chr28	9017130	3	40	GAA:0.9	G:0.025	GA:0.075		
chr28	9017172	2	36	A:1	AAAGC:0			
chr28	9017284	2	36	C:1	T:0			
chr28	9017304	2	36	T:1	G:0			
chr28	9017446	2	34	C:1	T:0			

chr28 9017524 2 30 TCTCTCC:0.766667 T:0.233333
chr28 9017528 2 30 TCC:0.433333 T:0.566667
chr28 9017953 2 34 G:1 A:0
chr28 9018548 2 40 T:0.1 G:0.9
chr28 9018662 2 36 C:1 G:0
chr28 9018674 2 32 TA:1 T:0
chr28 9018856 5 38 CAAATAAATAAAT:0.552632 C:
0.210526 CTAAAT:0 CTAAATAAAT:0 CAAATAAATAAATAAAT:0.236842
chr28 9018860 2 38 T:1 TCTC:0
chr28 9018864 2 38 T:1 TCTC:0
chr28 9019603 2 36 C:1 T:0
chr28 9019751 2 34 A:1 AT:0
chr28 9020694 2 40 C:1 CAAAT:0
chr28 9020794 2 34 A:1 G:0
chr28 9020798 2 34 A:1 G:0
chr28 9021002 2 36 A:1 T:0
chr28 9022289 2 40 C:1 T:0
chr28 9022290 2 40 A:1 G:0
chr28 9022351 2 36 C:1 T:0
chr28 9022352 2 36 G:1 A:0
chr28 9022420 2 40 G:0.825 T:0.175
chr28 9022668 2 32 AG:1 A:0
chr28 9022984 2 24 C:0.875 CT:0.125
chr28 9023016 2 26 G:1 T:0
chr28 9023157 2 36 C:1 CTGTGTG:0
chr28 9023159 3 36 CTG:0.944444 C:0.0555556
GTG:0
chr28 9023202 2 30 T:1 TA:0
chr28 9023408 2 32 G:1 GTAAA:0
chr28 9023450 3 36 TGA:0.611111 T:0.0833333
TGAGA:0.305556
chr28 9023552 2 30 G:1 GATC:0
chr28 9024684 2 38 A:1 G:0
chr28 9024720 4 32 T:0.09375 TA:0.53125
TAA:0.25 TAAA:0.125
chr28 9024730 2 36 A:1 AAAAAAAAAAAG:0
chr28 9024778 2 36 C:1 T:0
chr28 9025290 2 40 C:1 T:0
chr28 9025339 2 38 T:1 TG:0
chr28 9025345 2 38 A:1 T:0
chr28 9025361 2 36 T:1 A:0
chr28 9025362 2 36 A:1 T:0
chr28 9025730 2 26 AAT:1 A:0
chr28 9025731 2 26 AT:0 A:1
chr28 9025899 2 32 C:1 T:0
chr28 9026013 2 36 TG:1 T:0
chr28 9026014 2 36 G:1 T:0
chr28 9026018 2 36 T:1 A:0
chr28 9026023 2 36 T:1 A:0
chr28 9026048 2 36 C:1 T:0
chr28 9026069 2 36 G:1 C:0
chr28 9026275 3 34 TAA:0.735294 T:0.0882353
TA:0.176471
chr28 9026405 2 34 G:1 A:0

chr28	9026635	2	38	A:1	G:0	
chr28	9026679	2	38	T:1	C:0	
chr28	9026960	2	38	A:1	G:0	
chr28	9027069	2	34	C:1	A:0	
chr28	9028226	2	40	C:1	T:0	
chr28	9028471	2	40	C:1	T:0	
chr28	9029031	2	36	C:0.638889	CA:0.361111	
chr28	9029032	2	40	A:1	AAAAAAAAAATAAAAAT:0	
chr28	9029064	2	38	T:1	C:0	
chr28	9029759	2	26	C:0.961538	CT:0.0384615	
chr28	9029808	2	40	CACAGAGAGAGAGAGAG:0.925	C:0.075	
chr28	9029810	3	40	CAG:0.275	C:0.55	CAGAG: 0.175
chr28	9029956	2	34	G:1	GAGCC:0	
chr28	9030144	2	36	A:0.861111	G:0.138889	
chr28	9030185	2	36	T:1	A:0	
chr28	9030186	2	36	T:1	A:0	
chr28	9030657	2	36	G:1	A:0	
chr28	9030699	2	36	C:0.833333	T:0.166667	
chr28	9031234	2	34	G:1	A:0	
chr28	9031701	2	36	C:0.666667	T:0.333333	
chr28	9031738	2	36	T:1	C:0	
chr28	9031741	3	30	GT:0.766667	G:0.0333333	GTT:0.2
chr28	9032465	2	38	A:1	G:0	
chr28	9033585	2	34	T:1	A:0	
chr28	9033586	2	34	T:1	C:0	
chr28	9033837	2	28	TA:0.821429	T:0.178571	
chr28	9034448	2	40	G:1	A:0	
chr28	9035127	2	36	C:1	CTTAT:0	
chr28	9035331	2	36	T:1	TAATA:0	
chr28	9036932	2	40	T:1	C:0	
chr28	9037083	2	36	T:0.222222	C:0.777778	
chr28	9037089	2	36	T:1	C:0	
chr28	9037329	2	34	G:1	A:0	
chr28	9037585	2	34	G:1	GA:0	
chr28	9037594	2	34	C:1	A:0	
chr28	9037846	2	36	CTG:1	C:0	
chr28	9038349	2	38	A:1	G:0	
chr28	9038962	2	36	G:1	A:0	
chr28	9039044	2	36	T:0.833333	G:0.166667	
chr28	9039059	2	38	C:1	CCTT:0	
chr28	9039125	2	34	C:1	T:0	
chr28	9039672	2	36	G:1	A:0	
chr28	9039766	2	36	G:1	C:0	
chr28	9040250	2	36	T:0.861111	C:0.138889	
chr28	9040258	2	36	GC:0.416667	G:0.583333	
chr28	9040648	2	38	G:1	C:0	
chr28	9040777	2	38	C:1	CACTA:0	
chr28	9040794	2	40	A:1	G:0	
chr28	9040979	2	34	T:1	C:0	
chr28	9040991	2	36	ACT:1	A:0	
chr28	9041256	2	38	G:1	A:0	
chr28	9041508	2	34	A:1	C:0	

chr28	9041559	2	34	G:1	A:0		
chr28	9041584	2	36	CTT:1	C:0		
chr28	9042088	2	34	G:1	A:0		
chr28	9042090	2	34	A:1	T:0		
chr28	9042656	2	36	T:1	G:0		
chr28	9042713	2	38	C:1	T:0		
chr28	9043052	2	38	GC:1	G:0		
chr28	9046466	2	36	G:1	A:0		
chr28	9046907	2	38	A:1	AAC:0		
chr28	9047244	2	36	A:0	G:1		
chr28	9047405	2	38	G:1	A:0		
chr28	9047791	2	36	T:0	TG:1		
chr28	9048050	2	36	T:1	C:0		
chr28	9048208	2	22	AT:0	A:1		
chr28	9048224	2	18	T:1	C:0		
chr28	9048896	2	30	GC:1	G:0		
chr28	9049363	2	40	AAAG:1	A:0		
chr28	9049366	3	40	GAAGAAAGAAAGAAAGA:0.275	G:0.625	GAAGA:0.1	
chr28	9049429	2	40	G:1	GA:0		
chr28	9050173	2	34	TG:1	T:0		
chr28	9050240	2	38	A:1	C:0		
chr28	9051784	2	36	G:1	C:0		
chr28	9051823	2	36	C:1	T:0		
chr28	9051826	2	36	C:1	T:0		
chr28	9052674	2	38	A:1	T:0		
chr28	9052923	2	36	G:1	A:0		
chr28	9052947	4	38	AACACACAC:0.184211	A:0.157895	AAC:0.552632	AACAC:0.105263
chr28	9053125	2	38	G:1	A:0		
chr28	9053140	2	40	A:1	G:0		
chr28	9053532	2	40	G:1	C:0		
chr28	9055264	2	38	GT:0.263158	G:0.736842		
chr28	9056134	2	38	GGCAGCCCCCGTGGT:1	G:0		
chr28	9056143	2	38	C:1	G:0		
chr28	9056457	2	38	A:1	G:0		
chr28	9057481	2	40	T:1	A:0		
chr28	9058292	2	36	T:1	TA:0		
chr28	9059203	2	36	ATTTCTT:1	A:0		
chr28	9059843	2	40	G:1	C:0		
chr28	9060075	4	40	TACAAA:0.925	T:0.075	TACAAAACAAA:0	TACAAAACAAAACAAAACAAA:0
chr28	9060852	2	38	C:0.315789	T:0.684211		
chr28	9061609	2	40	A:1	C:0		
chr28	9061679	2	36	A:1	G:0		
chr28	9061900	2	40	C:1	T:0		
chr28	9062033	2	36	TCGCTCACA:1	T:0		
chr28	9062147	2	38	G:1	A:0		
chr28	9062361	3	30	TGG:0.833333	T:0	TG:0.166667	
chr28	9063282	2	16	AC:0.9375	A:0.0625		
chr28	9063400	2	26	G:0.153846	C:0.846154		
chr28	9064386	2	34	C:0.941176	CTT:0.0588235		
chr28	9064472	2	38	G:0.868421	GC:0.131579		

chr28	9064477	2	38	G:0.868421	C:0.131579	
chr28	9064668	2	38	G:1	T:0	
chr28	9065709	2	38	GA:0.973684	G:0.0263158	
chr28	9066664	2	36	G:0.805556	A:0.194444	
chr28	9066770	2	38	A:1	G:0	
chr28	9067055	2	38	G:1	A:0	
chr28	9068116	2	38	A:0.368421	AAAAG:0.631579	
chr28	9068122	2	38	A:1	AAGAAAG:0	
chr28	9068140	2	38	G:1	A:0	
chr28	9068147	2	38	A:1	AAAG:0	
chr28	9068158	3	38	G:0.526316	GAAGA:0.236842	GGAAGAAAGAAAGAAAGGAAGGAAGAAAGAAAGAAAGA:0.236842
chr28	9068456	2	38	C:1	T:0	
chr28	9068849	2	40	T:0.975	TAAAAAA:0.025	
chr28	9068872	2	38	GGAATA:1	G:0	
chr28	9069173	2	36	G:1	A:0	
chr28	9069266	2	36	A:1	G:0	
chr28	9069399	2	28	C:0.785714	CA:0.214286	
chr28	9069746	2	34	T:1	C:0	
chr28	9070332	2	36	A:1	AGT:0	
chr28	9071571	2	38	G:1	T:0	
chr28	9071574	2	38	G:1	T:0	
chr28	9071694	2	36	A:1	G:0	
chr28	9071730	2	34	G:0.441176	T:0.558824	
chr28	9072256	2	34	T:1	A:0	
chr28	9073177	3	40	TAA:0.225	T:0	TA:0.775
chr28	9073184	2	40	A:0.9	T:0.1	
chr28	9073654	2	38	T:1	G:0	
chr28	9073869	2	34	T:1	C:0	
chr28	9073993	2	36	A:0.777778	AT:0.222222	
chr28	9073995	2	36	A:0.777778	T:0.222222	
chr28	9074033	2	38	CAGATAGAG:0.789474	C: 0.210526	
chr28	9074037	3	38	TAGAGAG:0.815789	T:0	TAGAG: 0.184211
chr28	9074750	2	38	TA:0.789474	T:0.210526	
chr28	9075130	2	36	C:0.75	T:0.25	
chr28	9075511	2	40	CT:0.775	C:0.225	
chr28	9075676	2	38	A:0.815789	G:0.184211	
chr28	9075684	2	38	T:0.815789	A:0.184211	
chr28	9076002	2	32	C:1	T:0	
chr28	9076092	2	38	G:0.736842	C:0.263158	
chr28	9076158	2	36	T:0.805556	C:0.194444	
chr28	9076182	2	36	T:0.805556	C:0.194444	
chr28	9076673	2	40	CA:0.775	C:0.225	
chr28	9076764	2	38	T:0.5	C:0.5	
chr28	9076812	2	32	G:0.75	T:0.25	
chr28	9076821	2	32	T:0.75	G:0.25	
chr28	9076835	2	32	G:0.75	T:0.25	
chr28	9076846	2	32	C:0.75	T:0.25	
chr28	9076857	2	32	A:0.75	T:0.25	
chr28	9076902	2	34	T:0.764706	C:0.235294	
chr28	9076908	2	34	T:0.764706	C:0.235294	
chr28	9076958	3	34	TA:0.588235	T:0.176471	

TAA:0.235294

chr28	9077225	2	36	C:0.805556	T:0.194444	
chr28	9077262	2	34	T:0.823529	A:0.176471	
chr28	9077365	2	38	A:0.710526	G:0.289474	
chr28	9077504	2	40	C:0.825	T:0.175	
chr28	9077715	2	38	T:0.868421	C:0.131579	
chr28	9077725	3	38	C:0.868421	CTTT:0.105263	CTTTT:0.0263158
chr28	9078016	2	36	GCAGT:0.888889	G:0.111111	
chr28	9078201	2	38	C:0.789474	T:0.210526	
chr28	9078207	2	38	A:0.789474	G:0.210526	
chr28	9078254	2	38	T:0.815789	G:0.184211	
chr28	9078267	2	38	C:0.789474	A:0.210526	
chr28	9078294	2	38	A:0.763158	G:0.236842	
chr28	9078307	2	38	T:0.763158	TG:0.236842	
chr28	9078314	2	38	G:0.763158	A:0.236842	
chr28	9078760	2	34	C:0.235294	CT:0.764706	
chr28	9079172	2	38	A:0.815789	G:0.184211	
chr28	9079178	2	38	G:0.815789	A:0.184211	
chr28	9079196	2	38	A:0.815789	G:0.184211	
chr28	9079218	2	38	A:0.815789	G:0.184211	
chr28	9079312	2	36	C:0.75	T:0.25	
chr28	9079313	2	36	CT:0.75	C:0.25	
chr28	9079508	2	34	C:0.941176	T:0.0588235	
chr28	9079509	2	34	A:0.941176	G:0.0588235	
chr28	9079784	2	38	C:1	CT:0	
chr28	9080212	2	36	G:1	GCC:0	
chr28	9080216	2	36	C:1	CGA:0	
chr28	9080219	2	36	T:0.833333	G:0.166667	
chr28	9080220	2	36	C:0.833333	A:0.166667	
chr28	9080303	2	38	G:0.815789	A:0.184211	
chr28	9080344	2	34	C:1	T:0	
chr28	9080453	2	36	T:0.805556	C:0.194444	
chr28	9080484	2	32	T:0.875	TA:0.125	
chr28	9080539	2	36	G:0.777778	A:0.222222	
chr28	9081156	2	26	C:0.692308	CT:0.307692	
chr28	9081540	2	40	A:0.75	C:0.25	
chr28	9081732	2	34	C:0.911765	A:0.0882353	
chr28	9082462	2	34	C:0.735294	CT:0.264706	
chr28	9082581	2	38	G:0.736842	A:0.263158	
chr28	9082712	2	36	CAA:0.805556	C:0.194444	
chr28	9082716	2	36	T:0.805556	C:0.194444	
chr28	9082732	2	36	C:0.805556	CTT:0.194444	
chr28	9082740	2	36	C:0.0277778	T:0.972222	
chr28	9083013	2	34	A:0.735294	G:0.264706	
chr28	9083057	2	36	T:0.777778	C:0.222222	
chr28	9083137	2	36	C:0.805556	T:0.194444	
chr28	9083675	2	38	CT:0.973684	C:0.0263158	
chr28	9084375	2	38	CCT:0.789474	C:0.210526	
chr28	9084629	2	36	G:0.805556	C:0.194444	
chr28	9084938	2	36	G:0.833333	A:0.166667	
chr28	9085196	2	36	G:0.805556	T:0.194444	
chr28	9085578	2	34	G:0.852941	GCTCTCT:0.147059	
chr28	9085618	2	36	T:0.861111	A:0.138889	

chr28	9086088	2	34	CTT:0.794118	C:0.205882	
chr28	9086258	2	36	G:0.777778	C:0.222222	
chr28	9086297	2	36	CTTTATA:0.75	C:0.25	
chr28	9086512	2	40	T:1	C:0	
chr28	9086603	2	40	T:1	C:0	
chr28	9086707	2	38	C:0.736842	G:0.263158	
chr28	9086913	2	34	T:0.852941	C:0.147059	
chr28	9087018	2	36	A:0.0277778	C:0.972222	
chr28	9087957	2	38	A:0.815789	G:0.184211	
chr28	9088172	3	34	TA:0.764706	T:0.147059	TAA:0.0882353
chr28	9088444	2	38	TAGCTA:0.815789	T:0.184211	
chr28	9088609	2	34	T:0.735294	TAA:0.264706	
chr28	9088684	2	36	T:0.805556	C:0.194444	
chr28	9088799	2	32	T:0.71875	TAA:0.28125	
chr28	9088927	2	34	C:1	G:0	
chr28	9089022	2	28	C:0.75	A:0.25	
chr28	9089105	2	38	C:1	T:0	
chr28	9089203	2	38	T:0.815789	A:0.184211	
chr28	9089232	2	38	C:1	CAGAGAG:0	
chr28	9089415	2	38	C:1	T:0	
chr28	9090136	2	40	G:1	A:0	
chr28	9090764	2	34	A:1	C:0	
chr28	9090988	2	34	A:0.705882	G:0.294118	
chr28	9091191	2	38	C:1	T:0	
chr28	9091307	2	36	C:1	T:0	
chr28	9091652	2	34	C:0.852941	T:0.147059	
chr28	9091771	2	40	GT:1	G:0	
chr28	9091923	2	38	G:0.710526	A:0.289474	
chr28	9092080	2	40	C:1	G:0	
chr28	9092223	2	38	C:0.815789	T:0.184211	
chr28	9092909	2	32	CT:0.8125	C:0.1875	
chr28	9093041	2	40	C:0.8	T:0.2	
chr28	9093112	2	32	G:0.75	A:0.25	
chr28	9093187	2	38	CCT:0.736842	C:0.263158	
chr28	9093590	2	32	CCT:0.71875	C:0.28125	
chr28	9093627	2	30	TAAAA:0.966667	T:0.0333333	
chr28	9093630	2	34	A:1	AT:0	
chr28	9093633	2	34	A:1	AT:0	
chr28	9093635	2	30	A:0.966667	T:0.0333333	
chr28	9093641	2	34	A:1	T:0	
chr28	9094162	2	40	A:0.825	G:0.175	
chr28	9094920	2	36	T:0.722222	G:0.277778	
chr28	9095328	2	32	G:1	A:0	
chr28	9095562	2	40	CTCTG:0.75	C:0.25	
chr28	9095605	3	40	AG:0.725	A:0.275	AGG:0
chr28	9095719	2	28	G:1	A:0	
chr28	9095822	2	38	G:0.868421	T:0.131579	
chr28	9096051	2	38	A:0.842105	G:0.157895	
chr28	9096278	2	34	T:1	G:0	
chr28	9096366	2	36	TAC:0.833333	T:0.166667	
chr28	9096449	2	36	C:1	G:0	
chr28	9096503	2	40	C:0.6	CTTTTTTTTTTTT:0.4	
chr28	9096636	2	36	T:1	C:0	

chr28 9097105 2 40 C:0.825 T:0.175
chr28 9097982 2 40 C:0.825 T:0.175
chr28 9098466 2 38 T:1 A:0
chr28 9098761 2 38 AC:0.815789 A:0.184211
chr28 9099002 2 38 C:1 T:0
chr28 9099249 2 38 T:0.763158 C:0.236842
chr28 9099266 2 32 G:1 T:0
chr28 9099550 2 34 T:1 TAA:0
chr28 9099640 2 30 A:0.966667 C:0.0333333
chr28 9100002 2 36 G:1 A:0
chr28 9100006 2 36 C:1 T:0
chr28 9100067 2 36 TTCTC:0.916667 T:0.0833333
chr28 9100103 2 36 GA:1 G:0
chr28 9100185 2 40 G:0.825 C:0.175
chr28 9100333 2 30 T:1 C:0
chr28 9100450 3 40 C:0.825 CTCTG:0.175
CTCTCTCTCTCTCTG:0
chr28 9100716 2 36 C:1 A:0
chr28 9100869 2 34 C:1 T:0
chr28 9101114 2 36 T:1 C:0
chr28 9101296 2 36 C:0.694444 G:0.305556
chr28 9102150 2 34 A:1 G:0
chr28 9102269 2 38 G:1 C:0
chr28 9102282 2 36 A:1 G:0
chr28 9102285 2 36 G:1 C:0
chr28 9102675 2 32 CCTGT:1 C:0
chr28 9103023 2 32 C:1 T:0
chr28 9103084 2 38 T:0.815789 A:0.184211
chr28 9103085 2 38 T:0.815789 A:0.184211
chr28 9103207 2 34 T:1 C:0
chr28 9103230 2 32 T:0 C:1
chr28 9103393 2 40 T:1 C:0
chr28 9103559 2 32 G:1 A:0
chr28 9104029 2 38 A:1 G:0
chr28 9104106 2 34 C:1 G:0
chr28 9104152 2 38 TAAC:0.789474 T:0.210526
chr28 9104461 2 36 T:0.972222 TA:0.0277778
chr28 9104565 2 34 CAATT:0.970588 C:0.0294118
chr28 9104842 2 36 C:1 CTTTCTTTTCT:0
chr28 9104935 2 38 A:1 G:0
chr28 9105010 2 38 CTTTTTTTTGTTTT:0.736842 C:
0.263158
chr28 9105019 2 38 GT:0.815789 G:0.184211
chr28 9105026 2 38 T:1 G:0
chr28 9105332 2 38 C:1 G:0
chr28 9105405 2 38 G:0.763158 A:0.236842
chr28 9105780 2 36 G:1 A:0
chr28 9106125 2 38 TCTTTCCC:0.921053 T:
0.0789474
chr28 9106133 2 38 A:0.921053 AGGT:0.0789474
chr28 9106477 2 36 A:1 G:0
chr28 9106924 2 36 G:0.777778 A:0.222222
chr28 9107300 2 36 G:1 A:0
chr28 9107320 2 34 C:1 T:0

chr28	9107462	2	38	G:0.868421	C:0.131579	
chr28	9107744	2	40	C:0.85	T:0.15	
chr28	9108048	2	36	A:1	G:0	
chr28	9108085	2	36	G:0.805556	A:0.194444	
chr28	9108126	2	40	T:1	C:0	
chr28	9108519	2	32	CTTT:1	C:0	
chr28	9108535	2	32	T:0.96875	A:0.03125	
chr28	9108685	2	40	GAA:1	G:0	
chr28	9108720	2	40	T:1	C:0	
chr28	9108873	2	36	C:1	T:0	
chr28	9108950	2	32	C:1	A:0	
chr28	9108980	2	38	AAAAAG:0.947368	A:0.0526316	
chr28	9109317	2	36	T:1	A:0	
chr28	9109371	2	36	A:1	G:0	
chr28	9109634	2	36	A:1	G:0	
chr28	9109805	2	36	C:1	T:0	
chr28	9110262	2	38	A:1	G:0	
chr28	9110338	2	38	G:1	A:0	
chr28	9110411	2	36	G:1	A:0	
chr28	9110488	2	38	T:1	C:0	
chr28	9110514	3	36	TA:0.833333	T:0	TAA:0.166667
chr28	9110601	2	34	GA:1	G:0	
chr28	9110604	2	34	GT:1	G:0	
chr28	9110928	2	38	A:0.815789	G:0.184211	
chr28	9111013	2	40	G:1	A:0	
chr28	9111170	2	38	A:1	C:0	
chr28	9111254	2	36	G:1	GT:0	
chr28	9111556	2	38	C:0.815789	G:0.184211	
chr28	9111695	2	34	A:1	G:0	
chr28	9112237	2	40	G:1	A:0	
chr28	9113042	2	36	G:1	A:0	
chr28	9113206	2	38	C:0.815789	G:0.184211	
chr28	9113447	2	40	TTTA:1	T:0	
chr28	9113448	2	40	TTA:1	T:0	
chr28	9113458	2	40	TTTTA:1	T:0	
chr28	9113486	2	34	C:1	CAG:0	
chr28	9113601	2	34	C:1	A:0	
chr28	9113608	2	34	T:0.852941	C:0.147059	
chr28	9113777	2	38	TG:1	T:0	
chr28	9113925	2	32	TA:1	T:0	
chr28	9114091	2	36	G:0.805556	C:0.194444	
chr28	9114192	2	40	C:1	A:0	
chr28	9114394	2	38	T:0.789474	A:0.210526	
chr28	9114401	2	38	C:1	CAA:0	
chr28	9114999	2	34	C:1	T:0	
chr28	9115428	2	34	C:1	T:0	
chr28	9115456	2	38	A:1	G:0	
chr28	9115654	2	40	A:1	G:0	
chr28	9115933	2	36	G:0.805556	GTTGT:0.194444	
chr28	9116460	2	38	C:1	T:0	
chr28	9117028	2	36	T:0.777778	A:0.222222	
chr28	9117210	2	38	T:0.789474	C:0.210526	
chr28	9117252	2	40	A:0.775	G:0.225	

chr28 9117980 2 38 A:1 T:0
chr28 9117981 3 38 AAG:1 A:0 GAG:0
chr28 9118004 2 38 C:1 T:0
chr28 9118072 2 34 GAAA:1 G:0
chr28 9118145 2 40 C:1 T:0
chr28 9118226 2 40 T:1 C:0
chr28 9118408 2 36 T:0.861111 C:0.138889
chr28 9118672 2 40 T:1 A:0
chr28 9118674 2 40 C:1 A:0
chr28 9118781 3 36 CT:0.472222 C:0.277778
CTT:0.25
chr28 9118803 2 36 TTTTA:0.805556 T:0.194444
chr28 9119002 2 36 G:1 T:0
chr28 9119203 2 36 G:1 A:0
chr28 9119345 3 32 TA:0.4375 T:0.34375
TAAA:0.21875
chr28 9119362 2 36 A:1 AT:0
chr28 9119470 2 40 T:1 A:0
chr28 9119870 3 40 AT:0.825 A:0.025 ATGTTTT:0.15
chr28 9120006 2 34 G:1 A:0
chr28 9120038 2 38 A:0.789474 G:0.210526
chr28 9120081 2 38 C:1 T:0
chr28 9120109 2 38 C:0.815789 T:0.184211
chr28 9120303 2 32 C:1 T:0
chr28 9120801 2 36 C:0.722222 T:0.277778
chr28 9121069 2 36 G:1 A:0
chr28 9121180 2 36 C:0.972222 CT:0.0277778
chr28 9121516 2 40 A:0.75 G:0.25
chr28 9122021 2 40 AGGACATTTGGGCAGTGATGTTTTT:0.75
A:0.25
chr28 9122067 2 36 T:1 TA:0
chr28 9122560 2 32 C:0.75 T:0.25
chr28 9122818 2 32 G:1 A:0
chr28 9122925 2 40 A:1 C:0
chr28 9123875 2 40 C:1 T:0
chr28 9123928 2 36 C:1 T:0
chr28 9123957 2 38 T:0.763158 C:0.236842
chr28 9124006 2 38 CCTCTCTCT:0.947368 C:
0.0526316
chr28 9124042 2 38 A:1 T:0
chr28 9124060 2 38 A:1 C:0
chr28 9124061 2 38 C:1 A:0
chr28 9124281 2 34 T:1 C:0
chr28 9124572 2 34 T:1 TC:0
chr28 9124752 2 34 T:1 C:0
chr28 9124828 3 26 CT:0.384615 C:0.0384615
CTT:0.576923
chr28 9124843 2 32 T:1 A:0
chr28 9124899 2 28 C:1 G:0
chr28 9124901 2 28 C:1 G:0
chr28 9124972 2 36 G:1 A:0
chr28 9124982 2 36 G:1 A:0
chr28 9125096 2 38 A:1 G:0
chr28 9126738 2 36 T:1 C:0

chr28	9127083	2	38	C:1	T:0	
chr28	9127129	2	38	CCTCT:1	C:0	
chr28	9127181	2	34	TTAAAA:0.911765	T:0.0882353	
chr28	9127226	2	38	A:1	C:0	
chr28	9127250	2	38	G:1	GA:0	
chr28	9127363	2	38	T:1	A:0	
chr28	9127803	2	36	T:1	TA:0	
chr28	9127952	2	38	A:1	G:0	
chr28	9128086	2	38	T:0.842105	C:0.157895	
chr28	9128585	2	34	G:1	T:0	
chr28	9128938	2	34	T:1	C:0	
chr28	9129276	2	34	C:1	T:0	
chr28	9129297	2	36	C:1	A:0	
chr28	9129460	2	38	A:1	G:0	
chr28	9129912	2	34	T:1	C:0	
chr28	9129963	2	38	C:0.763158	T:0.236842	
chr28	9129964	2	38	A:0.763158	G:0.236842	
chr28	9130261	2	38	T:1	C:0	
chr28	9130795	2	36	C:0.777778	T:0.222222	
chr28	9130813	2	38	T:0.815789	C:0.184211	
chr28	9130936	2	38	T:1	C:0	
chr28	9130939	2	38	A:1	G:0	
chr28	9131645	2	18	G:0.388889	A:0.611111	
chr28	9132326	2	24	T:0.75	C:0.25	
chr28	9132540	2	34	T:0.794118	C:0.205882	
chr28	9134968	2	34	C:1	T:0	
chr28	9135012	2	22	TA:0.772727	T:0.227273	
chr28	9135153	2	34	C:1	T:0	
chr28	9135492	3	30	TA:0.533333	T:0.266667	TAAA:0.2
chr28	9135625	2	40	T:1	A:0	
chr28	9136723	2	38	CAT:1	C:0	
chr28	9136768	2	36	T:1	G:0	
chr28	9136846	2	36	A:1	T:0	
chr28	9136848	2	36	G:1	T:0	
chr28	9136969	2	38	G:1	A:0	
chr28	9137075	2	34	CT:0.941176	C:0.0588235	
chr28	9137096	2	38	TTTTA:0.736842	T:0.263158	
chr28	9137144	2	34	T:1	C:0	
chr28	9137614	2	38	A:1	C:0	
chr28	9138170	3	38	C:0.894737	CCTCT:0.105263	CCTCTCTCTCTCTCTCT:0
chr28	9138203	2	34	GAATA:0.941176	G:0.0588235	
chr28	9138235	2	32	T:0.09375	TA:0.90625	
chr28	9139044	2	36	A:1	G:0	
chr28	9139062	2	38	C:0.789474	CTTCT:0.210526	
chr28	9139324	2	34	C:1	CACAGAGAG:0	
chr28	9139613	2	32	G:1	C:0	
chr28	9139644	2	32	G:1	T:0	
chr28	9139804	2	32	G:0.84375	T:0.15625	
chr28	9139909	2	38	C:1	G:0	
chr28	9140077	2	36	A:1	T:0	
chr28	9140530	2	38	G:0.710526	C:0.289474	
chr28	9140720	2	36	G:1	A:0	

chr28 9140804 2 34 C:1 CTG:0
chr28 9141197 2 38 T:1 TA:0
chr28 9141271 2 36 A:1 T:0
chr28 9141396 2 36 G:1 C:0
chr28 9142392 2 36 G:1 A:0
chr28 9142600 2 34 A:1 T:0
chr28 9142635 2 36 A:0.777778 AT:0.222222
chr28 9142668 2 38 TAGAC:0.973684 T:0.0263158
chr28 9142680 3 40 AAGAGAGAG:0.65 A:0.025
AAGAGAGAGAG:0.325
chr28 9142841 3 30 TCC:0.7 T:0.3 TC:0
chr28 9142881 2 34 T:0.941176 TA:0.0588235
chr28 9143006 2 36 C:0.805556 T:0.194444
chr28 9143377 3 34 AT:0.705882 A:0.117647
ATT:0.176471
chr28 9143426 2 32 CAGAGAGAG:0.90625 C:
0.09375
chr28 9143525 2 38 A:0.789474 G:0.210526
chr28 9143900 2 38 TA:0.973684 T:0.0263158
chr28 9144440 2 38 G:0.815789 T:0.184211
chr28 9144446 2 40 G:1 A:0
chr28 9144686 2 36 A:1 T:0
chr28 9145028 2 34 TGAAATATCTGGAAAAAAAAAAAAAAA:
0.823529 T:0.176471
chr28 9145032 2 34 ATATC:1 A:0
chr28 9145038 2 34 GGAAAAAAAAAAAAAAAGAAATA:1 G:0
chr28 9145148 2 30 CT:0.5 C:0.5
chr28 9145161 2 34 T:0.735294 A:0.264706
chr28 9145164 4 40 GATTTATTT:0.65 G:0.2 GATTT:0
GATTTATTTATTT:0.15
chr28 9145226 2 40 T:1 TGAGA:0
chr28 9145643 2 38 C:1 CAAGAT:0
chr28 9145790 2 40 CTCT:1 C:0
chr28 9145810 2 40 T:1 A:0
chr28 9145957 2 38 CACG:0.763158 C:0.236842
chr28 9146538 2 36 G:0.833333 A:0.166667
chr28 9146594 2 38 ATC:0.868421 A:0.131579
chr28 9146977 2 28 T:1 A:0
chr28 9147023 2 34 G:1 T:0
chr28 9147135 2 38 TC:0.789474 T:0.210526
chr28 9147162 2 38 T:1 C:0
chr28 9147164 2 38 T:1 C:0
chr28 9147406 2 22 A:1 G:0
chr28 9149465 2 28 TA:0.5 T:0.5
chr28 9150836 2 32 GT:0.875 G:0.125
chr28 9153248 2 36 C:1 T:0
chr28 9157090 2 38 GT:0.842105 G:0.157895
chr28 9157133 2 34 CAGAG:0.970588 C:0.0294118
chr28 9158152 2 32 AT:1 A:0
chr28 9158201 2 30 GGA:0.933333 G:0.0666667
chr28 9158997 2 34 TA:0.941176 T:0.0588235
chr28 9159272 3 34 AAG:0.941176 A:0.0588235
AAGAGAG:0
chr28 9160165 2 36 T:1 TA:0

chr28	9160327	2	30	CTG:0.3	C:0.7
chr28	9160429	2	38	C:0.789474	T:0.210526
chr28	9160757	2	36	AC:1	A:0
chr28	9161374	2	34	T:1	A:0
chr28	9161866	2	38	C:1	T:0
chr28	9162302	2	38	GGA:0.894737	G:0.105263
chr28	9163197	2	36	GA:0	G:1
chr28	9163247	2	32	A:1	G:0
chr28	9164020	2	40	C:1
CTTTGTTCTTCTTTTTTTTTTTTTT:0
chr28	9164559	2	38	G:1	GT:0
chr28	9164798	2	36	TA:0.722222	T:0.277778
chr28	9165080	3	38	TA:0.868421	T:0.131579
AA:0
chr28	9165804	2	36	T:0.777778	C:0.222222
chr28	9166894	2	36	C:0.833333	CT:0.166667
chr28	9167364	2	32	C:0.46875	CT:0.53125
chr28	9167454	2	32	C:1	T:0
chr28	9168441	2	26	CAG:0.692308	C:0.307692
chr28	9168549	2	30	A:0.633333	G:0.366667
chr28	9169426	2	40	A:0.675	G:0.325
chr28	9170031	2	38	G:0.789474	A:0.210526
chr28	9170724	2	36	C:0.138889	CAGAG:0.861111
chr28	9171159	2	40	C:1	T:0
chr28	9175560	2	36	T:1	C:0
chr28	9175795	2	32	CT:0.96875	C:0.03125
chr28	9178631	2	34	T:0.911765	C:0.0882353
chr28	9180681	2	32	A:1	G:0
chr28	9180806	2	40	C:1	T:0
chr28	9180948	2	32	T:1	G:0
chr28	9181107	2	40	C:0.85	T:0.15
chr28	9181265	2	36	C:1	T:0
chr28	9184306	3	38	CAG:0.684211	C:0.0789474
CAGAG:0.236842
chr28	9187030	2	36	CAAAT:0.527778	C:0.472222
chr28	9187804	2	34	G:0.794118	A:0.205882
chr28	9188964	2	36	T:1	C:0
chr28	9190252	2	34	CT:0.941176	C:0.0588235
chr28	9190810	2	36	G:0.805556	A:0.194444
chr28	9191746	2	36	G:0	T:1
chr28	9194302	2	36	C:0.972222	T:0.0277778
chr28	9195381	2	38	T:1	G:0
chr28	9195814	2	38	G:1	A:0
chr28	9197247	2	38	G:1	A:0
chr28	9200444	2	40	G:1	A:0
chr28	9200919	2	32	CT:0.6875	C:0.3125
chr28	9202338	2	34	C:0.676471	T:0.323529
chr28	9202393	2	26	AT:0.769231	A:0.230769
chr28	9203008	4	40	CTTCTTTCT:0.2	C:0.175	CTTCT:
0.2	CTTCTTTCTTTCT:0.425
chr28	9203378	2	32	GT:0.875	G:0.125
chr28	9204202	2	34	GAC:0.823529	G:0.176471
chr28	9205805	2	40	CTTCTTCTTCTTCTCT:0.975	C:0.025
chr28	9205814	2	38	CTTCTCT:0.973684	C:0.0263158

chr28 9208416 2 36 T:1 A:0
chr28 9208559 2 28 C:1 T:0
chr28 9208833 2 36 C:1 T:0
chr28 9210336 2 34 C:1 T:0
chr28 9211793 2 24 AC:0 A:1
chr28 9214201 2 38 G:0.684211 A:0.315789
chr28 9218725 2 36 C:0.972222 T:0.0277778
chr28 9220104 2 32 A:0.625 AATTC:0.375
chr28 9221330 2 26 T:0.423077 C:0.576923
chr28 9222795 2 38 C:1 T:0
chr28 9224068 2 38 G:0 A:1
chr28 9224369 2 40 A:0.675 G:0.325
chr28 9225820 2 36 C:0.916667 G:0.0833333
chr28 9225982 2 36 T:0.972222 G:0.0277778
chr28 9226256 2 34 TCAAA:0.705882 T:0.294118
chr28 9226379 2 38 G:1 A:0
chr28 9226462 2 36 C:1 T:0
chr28 9229209 2 32 C:1 T:0
chr28 9231342 3 34 C:0.735294 CT:0.264706
CTT:0
chr28 9234080 2 36 C:1 T:0
chr28 9235371 2 36 G:0.694444 A:0.305556
chr28 9235627 2 38 G:1 C:0
chr28 9236536 3 34 T:0.147059 TTG:0.411765
TTGTG:0.441176
chr28 9241046 2 28 T:0.964286 TAC:0.0357143
chr28 9241915 2 38 G:1 T:0
chr28 9242810 2 40 C:1 T:0
chr28 9245246 2 38 G:1 A:0
chr28 9245562 2 36 C:0.972222 CAG:0.0277778
chr28 9245594 2 38 GAAACATAGGC:0.921053 G:
0.0789474
chr28 9245609 2 38 G:0.921053 A:0.0789474
chr28 9246068 3 28 CT:0.678571 C:0.142857
CTT:0.178571
chr28 9249971 2 36 GA:0.833333 G:0.166667
chr28 9252355 2 32 G:0.875 GC:0.125
chr28 9255857 2 36 A:1 G:0
chr28 9256832 2 28 T:1 C:0
chr28 9258279 2 32 T:1 TTTTC:0
chr28 9260451 2 36 TA:0.805556 T:0.194444
chr28 9262984 2 36 C:1 A:0
chr28 9263081 2 38 T:0.973684 A:0.0263158
chr28 9263695 2 38 C:0 A:1
chr28 9266238 4 40 TAAATAAAATA:0.1 T:0.5 TAAATA:
0.2 TAAATAAAATAAAATA:0.2
chr28 9266559 2 36 C:1 T:0
chr28 9268562 3 34 T:0.882353 TCA:0.117647
TCACA:0
chr28 9270413 2 34 T:0.794118 TA:0.205882
chr28 9270532 2 34 C:1 G:0
chr28 9271166 3 40 AAG:0.925 A:0.075 AAGAGAG:
0
chr28 9274164 2 38 A:1 T:0

chr28 9274623 2 36 G:1 A:0
chr28 9274782 3 34 TTTTA:0.352941 T:0.0588235
TTTTATTTA:0.588235
chr28 9276305 2 36 G:1 A:0
chr28 9279792 3 36 TTCTCTCTCTCTC:0.694444 T:
0.138889 TTCTCTC:0.166667
chr28 9279868 2 32 A:1 T:0
chr28 9279890 2 34 T:0.441176 TA:0.558824
chr28 9282265 2 38 C:1 T:0
chr28 9286422 2 36 A:0.861111 G:0.138889
chr28 9289479 2 36 G:0 GT:1
chr28 9289501 2 36 AG:0 A:1
chr28 9289508 2 36 C:0 CA:1
chr28 9289522 2 34 CT:0 C:1
chr28 9289527 2 34 C:0 CA:1
chr28 9289532 2 34 AT:0 A:1
chr28 9289540 2 32 A:0 AC:1
chr28 9289550 2 32 G:0 C:1
chr28 9289551 2 32 C:0 T:1
chr28 9289563 2 32 A:0 AT:1
chr28 9289582 2 34 T:0 TTG:1
chr28 9289586 2 34 A:0 AG:1
chr28 9289594 2 36 A:0 AT:1
chr28 9289602 2 36 C:0 CT:1
chr28 9289611 2 38 C:0 CT:1
chr28 9289613 2 38 A:0 T:1
chr28 9289628 2 38 T:0 C:1
chr28 9289678 2 34 G:0 GA:1
chr28 9289765 3 36 GT:0.805556 G:0.0833333
GTT:0.111111
chr28 9291028 2 32 G:0.84375 GA:0.15625
chr28 9292480 2 40 A:0.875 G:0.125
chr28 9292702 2 36 A:1 AT:0
chr28 9293543 2 40 A:1 T:0
chr28 9294590 2 36 CT:0.805556 C:0.194444
chr28 9295280 2 32 C:1 CTGTG:0
chr28 9296459 2 38 C:0.342105 A:0.657895
chr28 9297124 2 38 TG:0 T:1
chr28 9297705 2 32 CT:0.71875 C:0.28125
chr28 9297733 2 32 T:0.78125 A:0.21875
chr28 9297746 2 32 T:1 G:0
chr28 9299280 2 28 T:0.821429 TA:0.178571
chr28 9302028 2 36 G:1 A:0
chr28 9303306 2 38 C:1 T:0
chr28 9307062 3 26 TA:0.538462 T:0.153846
TAA:0.307692
chr28 9307815 2 38 C:1 T:0
chr28 9310023 3 38 TA:0.921053 T:0.0789474
TAA:0
chr28 9310572 2 40 G:1 A:0
chr28 9313118 2 36 C:1 T:0
chr28 9313791 2 32 TA:0.9375 T:0.0625
chr28 9314189 2 34 A:1 T:0
chr28 9314438 2 28 CATATATATAT:0.285714 C:

0.714286
chr28	9315188	2	36	AC:0.916667	A:0.0833333
chr28	9315208	2	38	A:1	G:0
chr28	9318528	2	24	TA:0.75	T:0.25
chr28	9320292	2	34	G:1	A:0
chr28	9320305	2	38	TG:0.973684	T:0.0263158
chr28	9320833	2	28	G:0.964286	GCA:0.0357143
chr28	9322001	2	38	A:1	G:0
chr28	9323951	2	36	G:0	A:1
chr28	9325691	2	36	A:1	G:0
chr28	9326224	2	40	C:1	T:0
chr28	9330049	2	30	CCT:0.933333	C:0.0666667
chr28	9330093	2	36	TA:0.944444	T:0.0555556
chr28	9338162	2	36	CT:0.75	C:0.25
chr28	9338671	2	36	T:1	C:0
chr28	9339342	2	30	T:0.366667	C:0.633333
chr28	9340687	2	40	A:1	C:0
chr28	9342346	2	36	CAGAG:1	C:0
chr28	9342381	2	36	G:0.611111	A:0.388889
chr28	9344449	2	34	T:1	G:0
chr28	9346223	2	36	C:0.944444	CAATA:0.0555556
chr28	9346437	2	36	GA:1	G:0
chr28	9347895	3	34	T:0.176471	TTTTA:0.705882
TTTTATTTA:0.117647
chr28	9349792	2	38	T:1	C:0
chr28	9350405	2	38	A:1	G:0
chr28	9351340	4	40	TATAAATAA:0.1	T:0.525	TATAA:
0.375	TATAAATAAATAA:0
chr28	9352037	2	36	G:1	C:0
chr28	9355014	2	34	CCT:0.970588	C:0.0294118
chr28	9356156	2	28	TA:0.428571	T:0.571429
chr28	9356483	2	34	CT:0.735294	C:0.264706
chr28	9357139	3	32	AT:1	A:0	ATT:0
chr28	9357371	2	38	C:0	A:1
chr28	9357778	2	36	T:1	TA:0
chr28	9359170	2	38	A:1	T:0
chr28	9359227	2	38	GGCAGAGGGAGAA:0.947368	G:
0.0526316
chr28	9359764	2	34	TA:0.882353	T:0.117647
chr28	9360813	2	40	C:0.8	T:0.2
chr28	9361289	2	34	T:0.323529	C:0.676471
chr28	9361760	2	36	CTG:0.944444	C:0.0555556
chr28	9361762	2	36	G:0.583333	C:0.416667
chr28	9362169	2	38	G:1	A:0
chr28	9363389	2	38	T:0.973684	G:0.0263158
chr28	9363703	2	38	AAAAGAAAG:1	A:0
chr28	9363721	3	40	AAGAAAGAAAAAG:0.8	A:0
AAAAGAAAAAG:0.2
chr28	9363727	2	40	GAA:0.975	G:0.025
chr28	9363729	2	40	A:0.45	AAG:0.55
chr28	9364175	2	34	T:1	C:0
chr28	9364724	2	38	G:1	A:0
chr28	9365482	2	40	CAA:1	C:0
chr28	9365955	2	36	C:0	T:1

chr28	9366573	2	38	T:1	A:0	
chr28	9367828	2	36	A:0	T:1	
chr28	9367875	2	36	TA:1	T:0	
chr28	9367881	2	36	G:1	C:0	
chr28	9368763	2	16	TA:0.8125		T:0.1875
chr28	9370512	2	30	A:0.9	G:0.1	
chr28	9371129	2	24	G:0.291667		C:0.708333
chr28	9371139	2	24	TCCGCGCCGCCCGCGCCGC:0.333333		T:0.666667
chr28	9371311	2	24	C:0.416667		G:0.583333
chr28	9371383	2	22	G:0.136364		T:0.863636
chr28	9371461	2	26	C:0.884615		G:0.115385
chr28	9372170	2	38	A:1	G:0	
chr28	9372185	2	38	A:1	G:0	
chr28	9372781	2	34	CT:0.441176		C:0.558824
chr28	9374018	2	34	G:0.647059		C:0.352941
chr28	9374197	2	36	T:1	C:0	
chr28	9374225	2	34	T:1	TTC:0	
chr28	9374408	2	38	C:1	T:0	
chr28	9375292	2	38	A:0	C:1	
chr28	9375559	2	38	C:1	CCTCT:0	
chr28	9376063	2	36	A:1	T:0	
chr28	9376620	2	36	A:1	T:0	
chr28	9376724	2	38	A:1	G:0	
chr28	9377246	2	40	TCA:0.725		T:0.275
chr28	9377362	2	34	T:1	A:0	
chr28	9377406	2	34	G:1	A:0	
chr28	9378066	2	36	TA:0.777778		T:0.222222
chr28	9378352	2	40	T:1	C:0	
chr28	9379155	2	34	CT:0.941176		C:0.0588235
chr28	9379714	2	38	AT:0.578947		A:0.421053
chr28	9379904	2	36	G:1	A:0	
chr28	9379915	2	40	G:1	A:0	
chr28	9381902	2	38	G:1	A:0	
chr28	9382377	2	30	C:1	T:0	
chr28	9382570	2	34	T:1	C:0	
chr28	9382869	2	34	A:1	G:0	
chr28	9383106	2	30	T:1	C:0	
chr28	9383420	2	38	G:1	T:0	
chr28	9383516	2	36	G:1	A:0	
chr28	9383681	2	36	G:0.972222		A:0.0277778
chr28	9384144	2	38	G:0.973684		T:0.0263158
chr28	9384153	2	38	T:0.973684		C:0.0263158
chr28	9384469	2	36	C:1	CT:0	
chr28	9384854	2	34	C:1	G:0	
chr28	9384896	2	32	CT:1	C:0	
chr28	9385165	2	34	A:1	G:0	
chr28	9385324	2	36	TG:1	T:0	
chr28	9385492	2	38	G:0.947368		GT:0.0526316
chr28	9385817	2	38	TAA:0.5	T:0.5	
chr28	9385948	2	36	A:1	G:0	
chr28	9386281	2	36	AGCT:1	A:0	
chr28	9386287	2	36	T:1	A:0	
chr28	9387287	2	36	C:0.972222		T:0.0277778

chr28	9387331	2	36	C:1	T:0	
chr28	9387708	2	36	A:1	T:0	
chr28	9387720	2	36	A:1	T:0	
chr28	9387722	2	36	T:1	TC:0	
chr28	9388020	2	34	T:1	A:0	
chr28	9388110	2	38	C:1	T:0	
chr28	9388356	2	30	C:1	A:0	
chr28	9388423	2	38	T:1	A:0	
chr28	9388498	2	34	AT:0.970588	A:0.0294118	
chr28	9388688	2	36	G:1	A:0	
chr28	9388702	2	36	A:1	G:0	
chr28	9388808	2	40	CTTCTG:1	C:0	
chr28	9388923	2	34	G:1	A:0	
chr28	9389027	2	36	C:1	T:0	
chr28	9389031	2	36	A:1	G:0	
chr28	9389127	2	38	T:1	G:0	
chr28	9389456	2	34	TA:0.970588	T:0.0294118	
chr28	9389930	2	36	G:1	A:0	
chr28	9390113	2	32	C:1	G:0	
chr28	9390141	2	40	GTCTCTGCCTCTCTCTCTC:0.825	G:0.175	
chr28	9390148	2	40	C:1	CCT:0	
chr28	9390206	2	34	AT:1	A:0	
chr28	9390620	3	30	CTTT:0.166667	C:0	CTTTT: 0.833333
chr28	9390863	2	36	G:1	A:0	
chr28	9390877	2	36	A:1	G:0	
chr28	9391347	2	34	G:1	T:0	
chr28	9391436	2	38	A:1	G:0	
chr28	9391460	2	40	C:1	A:0	
chr28	9391949	2	34	TAA:0.823529	T:0.176471	
chr28	9392036	2	38	T:1	C:0	
chr28	9392240	2	38	T:1	C:0	
chr28	9392571	2	32	G:1	A:0	
chr28	9392725	2	38	A:1	G:0	
chr28	9392832	2	40	G:1	T:0	
chr28	9393098	2	38	CGTTT:1	C:0	
chr28	9393371	2	36	G:1	A:0	
chr28	9393865	2	34	A:1	AAT:0	
chr28	9393906	2	38	A:1	AC:0	
chr28	9394201	2	32	C:1	T:0	
chr28	9394334	2	40	T:1	TA:0	
chr28	9394397	2	40	C:0.975	CT:0.025	
chr28	9394399	2	40	GCCCAGTTAA:0.975	G:0.025	
chr28	9394573	2	38	G:1	A:0	
chr28	9394893	2	34	C:1	G:0	
chr28	9394988	2	34	C:0.735294	CT:0.264706	
chr28	9395160	2	36	T:1	A:0	
chr28	9395301	2	40	G:1	T:0	
chr28	9395358	2	38	G:1	T:0	
chr28	9397180	2	32	TA:1	T:0	
chr28	9397221	2	38	T:1	TG:0	
chr28	9397328	2	34	C:1	A:0	
chr28	9397335	2	34	T:1	A:0	
chr28	9397356	2	34	CT:1	C:0	

chr28	9397587	2	38	C:1	T:0	
chr28	9397666	2	38	A:1	T:0	
chr28	9398030	2	34	A:1	G:0	
chr28	9398401	2	40	TA:1	T:0	
chr28	9398419	2	40	T:1	TA:0	
chr28	9398455	2	40	T:1	G:0	
chr28	9398642	2	30	A:1	T:0	
chr28	9398643	2	30	A:1	C:0	
chr28	9398660	2	30	A:1	G:0	
chr28	9398733	2	34	C:1	T:0	
chr28	9399036	2	30	G:1	A:0	
chr28	9399350	2	34	CCT:0.147059	C:0.852941	
chr28	9399378	2	34	AAATT:0.735294	A:0.264706	
chr28	9399604	2	34	T:1	C:0	
chr28	9399605	2	34	G:1	A:0	
chr28	9399623	2	34	G:1	C:0	
chr28	9399641	2	34	T:1	C:0	
chr28	9399673	2	38	G:1	A:0	
chr28	9399755	3	34	TA:0.588235	T:0.352941	TAA:0.0588235
chr28	9399791	2	36	A:1	C:0	
chr28	9399927	2	38	A:1	C:0	
chr28	9400413	2	30	C:1	T:0	
chr28	9400472	2	38	T:1	TG:0	
chr28	9400654	2	36	G:1	A:0	
chr28	9400813	2	36	GA:0.972222	G:0.0277778	
chr28	9400892	2	38	C:1	T:0	
chr28	9401088	2	38	C:1	A:0	
chr28	9401781	2	38	A:1	ATGTGTGTGTG:0	
chr28	9401846	2	36	G:1	A:0	
chr28	9401855	2	36	G:1	A:0	
chr28	9402130	2	36	A:1	AT:0	
chr28	9402175	3	32	CT:0.75	C:0.09375	CTT: 0.15625
chr28	9402275	2	38	T:1	C:0	
chr28	9402316	2	38	T:1	C:0	
chr28	9402515	3	32	ATC:0.875	A:0.125	ATTCTC:0
chr28	9402517	2	32	C:0.90625	T:0.09375	
chr28	9403020	2	38	A:1	AT:0	
chr28	9403055	2	40	AAC:1	A:0	
chr28	9403414	2	38	T:1	C:0	
chr28	9403486	2	38	T:1	C:0	
chr28	9403716	2	38	A:1	G:0	
chr28	9403888	2	32	C:1	T:0	
chr28	9403982	2	38	G:1	C:0	
chr28	9404223	2	38	AAT:0.736842	A:0.263158	
chr28	9404561	2	38	G:1	A:0	
chr28	9404634	2	34	C:1	T:0	
chr28	9404838	2	38	T:1	C:0	
chr28	9404885	2	40	T:1	G:0	
chr28	9405333	2	38	A:1	G:0	
chr28	9405405	2	32	C:1	A:0	
chr28	9405444	2	34	G:1	T:0	
chr28	9405823	2	34	A:1	G:0	

chr28	9405940	2	32	G:1	GA:0
chr28	9406050	2	38	CT:1	C:0
chr28	9406264	2	40	G:1	A:0
chr28	9406371	2	38	C:1	G:0
chr28	9406496	2	36	T:1	G:0
chr28	9406604	2	36	A:1	G:0
chr28	9406877	2	36	C:1	T:0
chr28	9406943	2	36	G:1	A:0
chr28	9407001	2	38	GTC:0.947368	G:0.0526316
chr28	9407048	2	34	TA:0.647059	T:0.352941
chr28	9407144	2	36	G:1	A:0
chr28	9407251	2	36	G:1	A:0
chr28	9407317	2	36	CT:0.944444	C:0.0555556
chr28	9407363	2	32	C:1	CAGAG:0
chr28	9407435	2	34	T:1	C:0
chr28	9407708	2	40	A:1	G:0
chr28	9408117	2	38	A:1	G:0
chr28	9408844	2	38	T:1	C:0
chr28	9409094	5	40	TATAA:0.3	T:0.375
TATAAATAA:0.3	TATAAATAAATAA:0.025	TATAAATAAATAAATAA:0
chr28	9409136	2	40	T:0.975	TAAATAAAA:0.025
chr28	9409832	2	36	A:1	G:0
chr28	9412296	2	40	G:1	A:0
chr28	9412365	2	36	C:1	T:0
chr28	9412764	2	36	T:1	A:0
chr28	9413209	2	30	C:0.933333	CCT:0.0666667
chr28	9414925	2	30	TA:0.933333	T:0.0666667
chr28	9414938	2	32	TTAA:1	T:0
chr28	9414939	2	30	TA:0	T:1
chr28	9414983	2	34	A:0	G:1
chr28	9415014	2	34	A:0	G:1
chr28	9415036	2	34	A:0	G:1
chr28	9415125	2	32	A:0	C:1
chr28	9415544	2	34	T:1	G:0
chr28	9416238	2	40	C:0.375	CA:0.625
chr28	9416347	2	40	C:0.375	CA:0.625
chr28	9416349	2	40	C:0.375	CA:0.625
chr28	9416351	2	40	G:0.375	GA:0.625
chr28	9416355	2	40	C:0.375	CT:0.625
chr28	9417023	2	36	GTTA:0.972222	G:0.0277778
chr28	9417266	2	38	G:1	A:0
chr28	9417400	2	36	C:1	T:0
chr28	9417497	2	34	C:1	G:0
chr28	9418472	2	38	A:1	T:0
chr28	9418536	2	34	A:1	G:0
chr28	9419145	2	32	T:1	C:0
chr28	9419440	3	36	CATATAT:0.194444	C:0.138889
CATAT:0.666667
chr28	9420740	2	36	T:0.944444	A:0.0555556
chr28	9422117	3	32	TAA:0.09375	T:0.40625
TA:0.5
chr28	9422780	2	38	GTTTGTT:0.973684	G:0.0263158
chr28	9423180	2	36	T:1	A:0
chr28	9423467	2	40	A:1	G:0

chr28	9423648	2	36	C:1	T:0	
chr28	9423833	2	38	C:1	CT:0	
chr28	9423839	2	38	T:0.684211		TG:0.315789
chr28	9423840	2	36	G:0.638889		T:0.361111
chr28	9425194	2	40	T:1	G:0	
chr28	9425684	2	36	C:1	T:0	
chr28	9425738	2	34	C:1	A:0	
chr28	9425879	2	36	TA:1	T:0	
chr28	9425882	2	36	T:1	C:0	
chr28	9425956	2	38	G:1	T:0	
chr28	9426387	2	36	A:1	G:0	
chr28	9426951	2	34	T:0.852941		A:0.147059
chr28	9427212	2	40	C:1	T:0	
chr28	9427415	2	36	A:1	C:0	
chr28	9427767	2	38	A:1	T:0	
chr28	9428233	2	40	T:1	C:0	
chr28	9428369	2	34	G:1	A:0	
chr28	9428449	2	34	G:1	C:0	
chr28	9428465	2	32	C:1	T:0	
chr28	9428611	2	40	ATCTCCCTGGTG:0		A:1
chr28	9428625	2	40	TATCTTTC:0		T:1
chr28	9428633	2	40	C:0	CAAAAAAAAAAAAAAAAAA:1	
chr28	9428636	2	40	C:0	A:1	
chr28	9428727	2	40	C:0	A:1	
chr28	9428782	2	36	G:0	A:1	
chr28	9428833	2	38	C:1	T:0	
chr28	9428852	2	36	C:0	CA:1	
chr28	9428890	2	38	G:1	C:0	
chr28	9428914	2	38	T:1	C:0	
chr28	9428931	2	38	T:0	A:1	
chr28	9429394	2	40	A:1	T:0	
chr28	9429401	2	40	A:1	ATAAATAAATAAATAAATAAATAAATAAATAAAT:0	
chr28	9429409	2	40	G:1	T:0	
chr28	9429422	2	40	C:1	G:0	
chr28	9429460	2	40	G:1	A:0	
chr28	9429511	2	36	G:1	C:0	
chr28	9429518	2	38	A:1	G:0	
chr28	9429977	2	36	C:1	T:0	
chr28	9430651	2	30	TA:1	T:0	
chr28	9431418	2	38	T:1	G:0	
chr28	9431600	2	36	T:1	C:0	
chr28	9431728	2	36	TG:1	T:0	
chr28	9431746	2	38	G:1	T:0	
chr28	9431863	2	38	C:1	T:0	
chr28	9431864	2	38	C:1	G:0	
chr28	9431869	2	38	G:1	T:0	
chr28	9432029	2	34	G:1	GAC:0	
chr28	9432086	2	36	G:1	C:0	
chr28	9432105	2	36	C:1	T:0	
chr28	9432107	2	36	T:1	A:0	
chr28	9432244	2	40	C:1	T:0	
chr28	9432247	2	40	C:1	CGGGATCCCTGGGTGGCGCAGCGGTTT:0	

chr28	9432303	2	36	T:1	C:0	
chr28	9432351	2	38	A:1	G:0	
chr28	9432764	2	40	A:1	T:0	
chr28	9432963	2	38	G:1	C:0	
chr28	9433378	2	36	CAAAT:1	C:0	
chr28	9433708	2	38	T:1	C:0	
chr28	9433904	2	38	C:1	T:0	
chr28	9434848	2	34	C:1	T:0	
chr28	9435007	2	38	G:1	A:0	
chr28	9435282	2	36	A:1	T:0	
chr28	9435329	2	38	G:1	A:0	
chr28	9435369	2	38	G:1	T:0	
chr28	9435417	2	34	A:1	C:0	
chr28	9435633	2	38	G:1	A:0	
chr28	9435984	2	32	G:0.84375	GTT:0.15625	
chr28	9436029	2	38	T:1	C:0	
chr28	9436182	2	32	C:1	T:0	
chr28	9436385	2	38	A:1	G:0	
chr28	9436531	2	32	C:1	T:0	
chr28	9437044	2	40	GAAAAAAAGA:1	G:0	
chr28	9437167	3	38	G:0.868421	GAAGA:0.131579	GAAGAAAGAAAGAAAGA:0
chr28	9437220	2	38	AAG:0.973684	A:0.0263158	
chr28	9438308	2	38	T:1	C:0	
chr28	9438570	2	38	A:1	G:0	
chr28	9438598	2	36	A:1	G:0	
chr28	9439112	2	38	G:1	A:0	
chr28	9439383	2	40	A:1	C:0	
chr28	9439813	2	38	C:1	T:0	
chr28	9439816	2	38	C:1	T:0	
chr28	9439872	2	40	G:1	A:0	
chr28	9440086	2	40	G:1	A:0	
chr28	9440269	2	36	T:1	C:0	
chr28	9440301	2	40	C:1	T:0	
chr28	9440404	2	34	T:0.882353	TA:0.117647	
chr28	9441330	2	36	T:1	C:0	
chr28	9441547	3	36	ATT:0.888889	A:0	AT:0.111111
chr28	9441578	2	38	A:1	T:0	
chr28	9441710	2	36	CAG:0.916667	C:0.0833333	
chr28	9441745	2	38	G:1	T:0	
chr28	9441756	2	38	C:1	T:0	
chr28	9442441	2	10	G:0	GC:1	
chr28	9442473	2	30	A:0	AG:1	
chr28	9442561	2	36	A:0	C:1	
chr28	9442666	2	34	G:1	A:0	
chr28	9442971	2	34	C:1	T:0	
chr28	9443556	2	36	CTTTT:1	C:0	
chr28	9443584	2	36	C:1	T:0	
chr28	9443764	2	36	A:1	G:0	
chr28	9443880	2	38	A:1	T:0	
chr28	9444011	2	36	G:1	A:0	
chr28	9444061	2	32	ATGT:0.96875	A:0.03125	
chr28	9444079	2	32	T:0.96875	C:0.03125	

chr28	9444233	2	38	T:1	A:0	
chr28	9444234	2	38	T:0.947368	A:0.0526316	
chr28	9444364	2	34	A:1	T:0	
chr28	9444470	2	40	C:1	G:0	
chr28	9445035	2	38	G:1	A:0	
chr28	9445149	2	36	T:1	G:0	
chr28	9445583	2	40	GTTCATTTCTCC:0.525	G:0.475	
chr28	9445639	2	40	C:0	CA:1	
chr28	9445772	2	38	T:1	G:0	
chr28	9445921	2	36	C:1	G:0	
chr28	9445978	2	36	GACAA:1	G:0	
chr28	9445984	2	36	C:1	T:0	
chr28	9446204	2	36	A:1	G:0	
chr28	9446344	2	38	A:1	T:0	
chr28	9446623	3	30	GACAC:0.2	G:0.766667	GAC:0.0333333
chr28	9447360	2	38	T:1	C:0	
chr28	9448027	2	38	C:0	CT:1	
chr28	9448308	2	38	T:1	C:0	
chr28	9448777	2	36	A:1	G:0	
chr28	9448821	2	34	A:1	T:0	
chr28	9448828	2	34	G:1	A:0	
chr28	9449544	2	38	A:1	G:0	
chr28	9449897	2	40	G:1	A:0	
chr28	9449905	2	40	A:1	G:0	
chr28	9450169	2	36	A:1	G:0	
chr28	9450262	2	38	A:1	G:0	
chr28	9450367	2	38	C:1	T:0	
chr28	9450609	2	38	G:1	A:0	
chr28	9450610	2	38	T:1	A:0	
chr28	9450900	2	40	G:1	A:0	
chr28	9451000	2	32	T:1	G:0	
chr28	9451020	2	32	A:1	G:0	
chr28	9451201	2	36	T:1	C:0	
chr28	9451362	2	30	T:1	C:0	
chr28	9451900	2	36	G:1	T:0	
chr28	9452012	2	40	G:1	A:0	
chr28	9452028	2	40	T:1	A:0	
chr28	9452129	2	34	T:1	C:0	
chr28	9452233	2	38	A:1	G:0	
chr28	9452270	2	36	G:1	A:0	
chr28	9452314	2	30	TG:0.8	T:0.2	
chr28	9452427	2	36	G:1	A:0	
chr28	9452712	2	34	A:1	G:0	
chr28	9452734	2	34	T:1	G:0	
chr28	9453163	3	38	C:0.236842	CA:0.736842	CAA:0.0263158
chr28	9453932	2	40	TTATCTCTAAAA:0.9	T:0.1	
chr28	9454106	2	34	A:1	T:0	
chr28	9454107	2	34	A:1	T:0	
chr28	9454196	2	40	C:1	T:0	
chr28	9454395	2	38	T:1	TA:0	
chr28	9454706	2	36	TC:1	T:0	
chr28	9454805	2	36	C:1	T:0	

chr28	9455025	2	40	A:0.975	C:0.025
chr28	9455167	2	36	T:1	C:0
chr28	9455343	2	38	C:1	T:0
chr28	9455449	2	38	A:1	G:0
chr28	9455723	2	38	A:1	G:0
chr28	9455757	2	36	T:1	C:0
chr28	9455869	2	40	T:1	C:0
chr28	9455902	2	36	G:1	A:0
chr28	9456042	2	28	C:1	A:0
chr28	9456143	2	38	C:0.947368	A:0.0526316
chr28	9456168	2	40	C:1	CTGTT:0
chr28	9456189	2	40	C:1	T:0
chr28	9456651	2	40	C:1	A:0
chr28	9456873	2	38	A:1	T:0
chr28	9456996	2	40	A:1	AGTCAGAATTG:0
chr28	9457229	2	34	C:1	T:0
chr28	9457587	2	36	A:1	ATACT:0
chr28	9457713	2	38	C:1	T:0
chr28	9457811	2	40	T:1
TAGAAACTTACTGTTTTAAAAAAAATAAATAAATAAAATAAAATAAAAAAAAAA:0
chr28	9457928	2	38	C:1	G:0
chr28	9457936	2	38	A:1	G:0
chr28	9458001	2	34	T:1	A:0
chr28	9458292	2	36	A:1	G:0
chr28	9458778	2	32	G:0.96875	T:0.03125
chr28	9458795	2	30	T:1	C:0
chr28	9459245	2	40	G:1	T:0
chr28	9459498	2	36	CA:1	C:0
chr28	9459828	2	40	A:1	G:0
chr28	9459891	2	40	A:1	C:0
chr28	9460192	2	36	T:1	C:0
chr28	9461215	2	38	A:1	G:0
chr28	9461439	2	36	A:1	G:0
chr28	9462044	2	36	A:0.833333	T:0.166667
chr28	9462052	3	38	A:0.973684	T:0	AT:
0.0263158
chr28	9462054	3	38	T:0.0263158	A:0.0526316
TA:0.921053
chr28	9462408	2	38	G:1	A:0
chr28	9462535	2	38	A:1	G:0
chr28	9462713	2	40	G:1	T:0
chr28	9462752	2	40	G:1	T:0
chr28	9462942	2	36	A:1	T:0
chr28	9463848	2	36	G:1	C:0
chr28	9463906	2	34	CCT:0.882353	C:0.117647
chr28	9463999	2	38	T:1	A:0
chr28	9464086	2	38	CAA:1	C:0
chr28	9464290	2	34	C:1	CAAT:0
chr28	9464350	2	36	G:1	A:0
chr28	9464386	3	36	GATAT:0.777778	G:0	GAT:
0.222222
chr28	9465510	2	34	C:1	A:0
chr28	9465524	3	32	G:0.71875	GATTT:0.09375
GATTTATTT:0.1875

chr28	9465616	2	38	G:1	A:0	
chr28	9465675	2	30	G:1	A:0	
chr28	9466782	2	38	T:1	C:0	
chr28	9466972	2	36	G:1	A:0	
chr28	9467047	2	38	G:1	A:0	
chr28	9467215	2	34	AG:1	A:0	
chr28	9467298	2	36	C:1	T:0	
chr28	9467639	2	38	G:1	T:0	
chr28	9467695	2	38	G:1	A:0	
chr28	9467980	2	34	C:1	T:0	
chr28	9468105	2	34	T:1	C:0	
chr28	9468372	2	30	C:0	A:1	
chr28	9468424	2	36	G:0	T:1	
chr28	9468495	2	30	G:0.833333	T:0.166667	
chr28	9468515	2	26	GC:0.653846	G:0.346154	
chr28	9468894	2	38	A:1	G:0	
chr28	9468919	2	38	T:1	G:0	
chr28	9469082	2	38	A:1	C:0	
chr28	9469240	2	32	T:1	C:0	
chr28	9469513	2	38	C:1	T:0	
chr28	9469663	2	36	G:1	A:0	
chr28	9469964	2	38	A:1	C:0	
chr28	9470952	2	34	A:1	G:0	
chr28	9471337	2	38	T:0.710526	C:0.289474	
chr28	9471374	2	36	A:1	T:0	
chr28	9471647	2	36	T:1	C:0	
chr28	9471683	2	34	C:1	T:0	
chr28	9471820	3	38	TAAA:0.421053	T:0	TAA: 0.578947
chr28	9471824	2	38	A:1	T:0	
chr28	9471834	2	38	A:1	T:0	
chr28	9471878	2	26	TA:0.884615	T:0.115385	
chr28	9472139	2	36	G:1	GAATA:0	
chr28	9472160	2	36	A:1	C:0	
chr28	9472164	3	36	TA:0.916667	T:0.0833333	TAA:0
chr28	9472507	2	34	A:1	T:0	
chr28	9472509	2	34	A:1	ATT:0	
chr28	9472687	2	34	TAA:1	T:0	
chr28	9472771	2	34	T:1	C:0	
chr28	9472811	2	34	T:1	C:0	
chr28	9472976	2	36	C:0.972222	T:0.0277778	
chr28	9473107	2	36	G:0.611111	T:0.388889	
chr28	9473232	2	36	T:0	C:1	
chr28	9473467	2	36	C:1	G:0	
chr28	9473630	2	36	G:1	A:0	
chr28	9473921	2	38	C:1	T:0	
chr28	9474078	2	38	A:1	T:0	
chr28	9474098	2	38	GT:0.947368	G:0.0526316	
chr28	9474243	2	36	G:1	A:0	
chr28	9474274	2	34	A:0.970588	G:0.0294118	
chr28	9474534	2	38	C:1	G:0	
chr28	9475305	2	36	C:1	A:0	
chr28	9475872	2	40	T:0.825	TA:0.175	

chr28	9475873	2	40	A:1	T:0		
chr28	9477403	2	40	C:1	G:0		
chr28	9477706	2	38	C:1	A:0		
chr28	9480667	2	36	T:1	C:0		
chr28	9481732	2	40	G:1	T:0		
chr28	9484016	2	34	T:1	C:0		
chr28	9484192	2	32	G:1	GTA:0		
chr28	9484730	2	38	T:1	C:0		
chr28	9485452	2	38	GTA:0.947368	G:0.0526316		
chr28	9485454	2	40	A:1	G:0		
chr28	9485472	2	38	C:0.947368	T:0.0526316		
chr28	9486159	4	36	ATATTTATT:0.222222	A:0.333333	ATATT:0.444444	ATATTTATTTATT:0
chr28	9486719	3	38	TGA:0.973684	T:0.0263158	TGAGA:0	
chr28	9487304	2	36	A:1	C:0		
chr28	9488801	2	38	AAG:1	A:0		
chr28	9489052	2	20	AT:0.75	A:0.25		
chr28	9489128	2	36	C:1	T:0		
chr28	9489474	2	30	T:0	A:1		
chr28	9490421	2	40	C:1	T:0		
chr28	9492278	2	40	G:0.925	T:0.075		
chr28	9492364	2	36	GC:0.833333	G:0.166667		
chr28	9492496	2	28	TG:0.535714	T:0.464286		
chr28	9492789	2	34	AT:0.0588235	A:0.941176		
chr28	9492936	2	36	C:0.888889	A:0.111111		
chr28	9493004	2	40	G:1	T:0		
chr28	9493238	3	40	TTTTATTTA:0.725	T:0.275	TTTTA:0	
chr28	9493368	2	38	G:0.526316	A:0.473684		
chr28	9493572	2	38	C:0.552632	T:0.447368		
chr28	9493903	2	38	C:0.578947	T:0.421053		
chr28	9494588	2	40	C:0.85	T:0.15		
chr28	9494885	2	38	CTATGTTTCCTAGT:0.447368	C:0.552632		
chr28	9494918	2	38	C:1	T:0		
chr28	9495016	2	36	C:0.805556	G:0.194444		
chr28	9495288	2	30	G:0.466667	T:0.533333		
chr28	9495331	2	32	A:0.46875	AT:0.53125		
chr28	9495351	2	34	TTTTAAATTA:0.941176	T:0.0588235		
chr28	9495479	2	36	T:0.611111	A:0.388889		
chr28	9495505	2	36	C:0.444444	A:0.555556		
chr28	9495512	2	36	C:0.444444	A:0.555556		
chr28	9495626	2	40	C:0.475	CACAG:0.525		
chr28	9495731	2	36	A:0	G:1		
chr28	9495755	2	36	G:0.583333	A:0.416667		
chr28	9495778	2	38	G:0.578947	A:0.421053		
chr28	9496008	2	38	T:0.657895	TATG:0.342105		
chr28	9496100	2	30	C:0.6	T:0.4		
chr28	9496179	2	40	GATT:0.9	G:0.1		
chr28	9496208	2	38	AG:0.526316	A:0.473684		
chr28	9496209	2	38	GGAGA:0.5	G:0.5		
chr28	9496213	2	38	A:0.526316	AG:0.473684		
chr28	9496260	2	36	G:0.472222	T:0.527778		

chr28	9496284	3	36	T:0.472222	C:0.444444
G:0.0833333
chr28	9496489	2	34	CT:0.529412	C:0.470588
chr28	9496593	2	38	A:0.578947	G:0.421053
chr28	9498183	2	38	A:0.526316	G:0.473684
chr28	9498273	2	32	A:0.59375	T:0.40625
chr28	9498387	2	36	A:0.583333	G:0.416667
chr28	9498587	3	36	A:0.361111	AT:0.527778
ATT:0.111111
chr28	9498665	2	36	A:0.555556	G:0.444444
chr28	9498680	2	36	G:0.722222	A:0.277778
chr28	9498726	2	36	T:0.444444	G:0.555556
chr28	9498764	2	34	G:0.617647	A:0.382353
chr28	9498915	3	30	GT:0.433333	G:0.166667
GTT:0.4
chr28	9499084	2	38	A:0.552632	AGTTGTT:0.447368
chr28	9499151	2	36	C:1	T:0
chr28	9499210	2	34	G:0.441176	GAA:0.558824
chr28	9499407	2	32	GA:1	G:0
chr28	9499490	2	36	G:0.638889	T:0.361111
chr28	9499610	2	36	C:0.416667	T:0.583333
chr28	9499889	2	36	T:0.361111	G:0.638889
chr28	9499975	2	38	C:0.394737	A:0.605263
chr28	9500610	2	36	A:0.527778	G:0.472222
chr28	9500809	2	40	A:0.625	G:0.375
chr28	9501246	2	34	C:0.588235	T:0.411765
chr28	9501285	2	34	T:0.617647	C:0.382353
chr28	9501415	3	36	T:0.416667	TTA:0.333333
TTTA:0.25
chr28	9501431	2	36	AT:0.416667	A:0.583333
chr28	9501440	2	36	T:0.416667	A:0.583333
chr28	9501715	2	38	CT:0.394737	C:0.605263
chr28	9501902	2	38	T:0.421053	C:0.578947
chr28	9502402	2	38	G:0.447368	A:0.552632
chr28	9502449	2	38	C:0.842105	T:0.157895
chr28	9503233	2	38	T:0.342105	C:0.657895
chr28	9503303	2	38	A:0.815789	G:0.184211
chr28	9503721	2	32	C:0.9375	T:0.0625
chr28	9503885	2	40	T:0.525	G:0.475
chr28	9504296	2	38	T:0.578947	C:0.421053
chr28	9504525	2	36	A:0.583333	C:0.416667
chr28	9505005	2	36	T:0.5	C:0.5
chr28	9505226	2	38	A:0.605263	G:0.394737
chr28	9505395	2	36	A:0.638889	AAAT:0.361111
chr28	9505549	2	40	CTCCTTCCTTTCCTGTATCTTTATGCT:0.5
C:0.5
chr28	9505860	2	34	T:0.470588	C:0.529412
chr28	9506135	2	34	T:0.588235	A:0.411765
chr28	9506136	2	34	C:0.588235	A:0.411765
chr28	9506513	2	40	T:0.4	A:0.6
chr28	9506791	2	38	G:0.342105	A:0.657895
chr28	9507084	2	40	A:0.625	G:0.375
chr28	9507340	2	40	T:0.625	TTCTCTCTCTC:0.375
chr28	9508167	2	34	T:0.705882	C:0.294118

chr28 9508173 2 34 T:1 G:0
chr28 9508449 2 36 A:0.583333 G:0.416667
chr28 9508663 2 40 G:1 A:0
chr28 9508770 2 38 G:0.631579 A:0.368421
chr28 9509126 2 38 G:0.5 A:0.5
chr28 9509282 2 36 T:0.444444 C:0.555556
chr28 9509465 2 36 C:0.555556 T:0.444444
chr28 9509494 2 38 T:0.368421 A:0.631579
chr28 9510303 2 38 C:0.368421 CT:0.631579
chr28 9510340 2 30 GTTTT:0.333333 G:0.666667
chr28 9510675 3 38 TAA:0.631579 T:0.289474
TA:0.0789474
chr28 9511120 2 26 AT:0.846154 A:0.153846
chr28 9511125 3 26 T:0.153846 G:0.423077
TG:0.423077
chr28 9511342 2 26 CTTT:0.538462 C:0.461538
chr28 9511791 2 36 T:0.777778 C:0.222222
chr28 9512450 3 38 CCTCTCT:0.578947 C:0.342105
CCTCT:0.0789474
chr28 9512527 2 38 A:0.473684 C:0.526316
chr28 9512624 2 36 A:0.416667 C:0.583333
chr28 9512645 2 36 TG:0.694444 T:0.305556
chr28 9513488 5 40 GGT:0.425 G:0.1 GGTGT:
0.1 GGTGTGTGT:0.05 GGTGTGTGTGT:0.325
chr28 9513529 2 40 TTGATTTGCATTTCTCTGA:0.875 T:0.125
chr28 9513642 2 24 CTT:0.625 C:0.375
chr28 9513673 2 32 T:0.5 C:0.5
chr28 9513850 2 36 A:0.361111 G:0.638889
chr28 9513900 2 34 C:0.676471 CTT:0.323529
chr28 9514092 2 34 C:1 T:0
chr28 9514247 2 40 A:0.875 G:0.125
chr28 9514276 2 40 C:0.6 T:0.4
chr28 9514391 3 34 CCT:0.676471 C:0.294118
CCTCT:0.0294118
chr28 9515554 2 40 C:0.575 T:0.425
chr28 9516333 2 34 A:0.441176 ATAG:0.558824
chr28 9517458 2 38 G:0.526316 T:0.473684
chr28 9517894 2 30 GA:0.933333 G:0.0666667
chr28 9517899 2 34 GAAAAGAAAAGAAAAGA:0.823529
G:0.176471
chr28 9517904 2 34 GAAAAGAAAAGA:0.911765 G:
0.0882353
chr28 9517909 2 40 GAAAAGA:0.9 G:0.1
chr28 9517914 2 40 GA:0.875 G:0.125
chr28 9517928 3 40 AAAAAGAAAAG:0.4 A:0.6 AAAAAG:0
chr28 9518095 2 32 GAGAA:0.78125 G:0.21875
chr28 9518139 2 36 AAAAGAAAGAAAG:0.805556 A:
0.194444
chr28 9518438 2 34 G:0.735294 T:0.264706
chr28 9518562 2 36 C:0.638889 CT:0.361111
chr28 9518567 2 36 T:0.388889 TA:0.611111
chr28 9518776 2 28 G:0.5 C:0.5
chr28 9518823 4 30 TAAAA:0.433333 T:0.0666667
TAA:0.133333 TAAA:0.366667

chr28	9518931	2	36	AC:0.527778	A:0.472222		
chr28	9518934	2	36	TGGA:0.527778	T:0.472222		
chr28	9520117	2	38	C:1	T:0		
chr28	9520496	2	38	C:0.526316	G:0.473684		
chr28	9520512	2	38	G:0.526316	A:0.473684		
chr28	9520561	2	34	A:0.411765	T:0.588235		
chr28	9520834	2	38	CA:0.421053	C:0.578947		
chr28	9520922	2	34	G:0.529412	T:0.470588		
chr28	9520997	2	32	G:0.875	A:0.125		
chr28	9521353	2	38	T:0.894737	A:0.105263		
chr28	9521380	2	38	G:0.578947	A:0.421053		
chr28	9521388	2	40	C:0.55	T:0.45		
chr28	9522025	2	38	C:0.842105	T:0.157895		
chr28	9522046	3	34	G:0.852941	GAA:0.147059	GAAA:0	
chr28	9522056	2	34	AG:0.617647	A:0.382353		
chr28	9522057	2	34	G:0.852941	A:0.147059		
chr28	9522239	2	38	A:0.894737	T:0.105263		
chr28	9522566	2	40	G:0.825	T:0.175		
chr28	9522631	2	38	C:0.868421	T:0.131579		
chr28	9522639	2	40	A:1	G:0		
chr28	9522821	2	32	C:0.375	CCTCTGT:0.625		
chr28	9523058	2	34	T:0.558824	G:0.441176		
chr28	9523213	2	28	C:0.892857	T:0.107143		
chr28	9524088	2	38	G:0.842105	A:0.157895		
chr28	9524160	2	38	T:0.552632	TC:0.447368		
chr28	9524162	2	38	T:0.552632	C:0.447368		
chr28	9524196	2	36	T:0.416667	A:0.583333		
chr28	9525081	2	36	G:0.444444	A:0.555556		
chr28	9525142	2	40	G:0.675	A:0.325		
chr28	9525692	2	40	G:0.875	C:0.125		
chr28	9525696	2	40	C:0.45	T:0.55		
chr28	9526115	2	38	T:0.578947	TA:0.421053		
chr28	9526276	2	40	G:1	A:0		
chr28	9526780	2	34	G:0.617647	C:0.382353		
chr28	9527150	2	36	G:0.5	T:0.5		
chr28	9527488	2	26	A:0.153846	C:0.846154		
chr28	9527492	2	26	C:0.153846	A:0.846154		
chr28	9527499	2	32	A:0.125	AC:0.875		
chr28	9527529	4	40	GAAGAAAGA:0.825	G:0.15	GAAGA:0.025	GAAGAAAGAAAGA:0
chr28	9527537	2	40	A:1	AAAG:0		
chr28	9527764	2	34	A:0.588235	G:0.411765		
chr28	9528033	2	38	G:0.684211	T:0.315789		
chr28	9528160	2	36	G:0.861111	C:0.138889		
chr28	9528182	3	36	T:0.666667	TA:0.333333	TAA:0	
chr28	9528207	2	38	A:0.868421	AAC:0.131579		
chr28	9528208	2	38	A:0.710526	AC:0.289474		
chr28	9528209	2	38	A:0.710526	AAAC:0.289474		
chr28	9528216	2	38	A:1	AC:0		
chr28	9528253	2	38	T:0.684211	C:0.315789		
chr28	9528902	2	36	A:0.5	G:0.5		
chr28	9529067	2	36	C:0.388889	T:0.611111		

chr28	9529360	2	38	C:0.736842	CACAGAGAAAGAGAGAGAGAGAGAGAGAGGCAGAGAGACA:0.263158	
chr28	9529491	2	30	G:0.5	GT:0.5	
chr28	9529511	2	30	CTTATATTTATTTT:0.833333	C:0.166667	
chr28	9529577	2	16	A:0.8125	AAG:0.1875	
chr28	9529688	2	38	G:0.552632	T:0.447368	
chr28	9529766	2	32	A:0.8125	ACC:0.1875	
chr28	9529773	2	32	T:0.46875	C:0.53125	
chr28	9530088	2	38	A:0.842105	G:0.157895	
chr28	9531740	2	36	T:0.583333	C:0.416667	
chr28	9532276	2	40	A:0.825	G:0.175	
chr28	9533095	2	38	A:0.473684	G:0.526316	
chr28	9533300	2	32	T:0.59375	G:0.40625	
chr28	9533491	2	38	A:0.868421	T:0.131579	
chr28	9533685	2	34	G:0.764706	T:0.235294	
chr28	9533715	2	36	T:1	C:0	
chr28	9533792	2	40	G:0.475	GT:0.525	
chr28	9533874	2	38	CT:0.605263	C:0.394737	
chr28	9534245	2	40	T:0.95	TA:0.05	
chr28	9534425	2	36	T:0.583333	C:0.416667	
chr28	9534450	3	38	CTTTTATTTTA:0.526316	C:0.447368	CTTTTA:0.0263158
chr28	9534493	2	30	TTTTA:0.8	T:0.2	
chr28	9534662	2	32	C:0.625	T:0.375	
chr28	9534670	2	30	TTC:0.833333	T:0.166667	
chr28	9534691	2	26	T:0.538462	TTTTA:0.461538	
chr28	9534741	2	26	G:0.461538	C:0.538462	
chr28	9534747	2	28	G:0.785714	A:0.214286	
chr28	9535206	2	38	C:0.868421	T:0.131579	
chr28	9535650	2	34	A:0.529412	G:0.470588	
chr28	9535696	2	38	C:0.421053	T:0.578947	
chr28	9535967	2	34	T:0.529412	C:0.470588	
chr28	9536027	2	38	TC:1	T:0	
chr28	9536028	3	38	C:0.447368	CTT:0.105263	CTTT:0.447368
chr28	9536056	2	36	T:0.611111	G:0.388889	
chr28	9536195	2	30	C:0.433333	T:0.566667	
chr28	9536362	2	34	T:0.441176	C:0.558824	
chr28	9536624	3	34	TA:0.705882	T:0.0588235	TAA:0.235294
chr28	9537154	2	36	A:0.833333	AT:0.166667	
chr28	9537172	2	36	AAC:1	A:0	
chr28	9537354	2	34	T:1	A:0	
chr28	9537511	2	34	TA:0.911765	T:0.0882353	
chr28	9537555	2	36	A:0.888889	T:0.111111	
chr28	9537556	2	36	T:0.888889	G:0.111111	
chr28	9537565	2	36	A:0.888889	C:0.111111	
chr28	9537697	2	40	G:1	C:0	
chr28	9537699	2	40	G:0.525	A:0.475	
chr28	9537780	2	38	C:1	T:0	
chr28	9538134	2	40	T:0.875	TAAAAAAAC:0.125	
chr28	9538161	2	40	TCA:0.875	T:0.125	
chr28	9538230	2	38	C:0.868421	G:0.131579	

chr28	9538320	2	32	A:0.90625	AG:0.09375	
chr28	9538380	2	26	G:0.961538	C:0.0384615	
chr28	9538530	2	36	A:0.805556	G:0.194444	
chr28	9538894	2	28	CG:0.821429	C:0.178571	
chr28	9539003	2	34	C:0.911765	G:0.0882353	
chr28	9539029	2	34	G:0.882353	T:0.117647	
chr28	9539131	2	40	G:0.825	C:0.175	
chr28	9539496	2	38	A:0.973684	G:0.0263158	
chr28	9539650	2	40	C:1	CTCTA:0	
chr28	9539693	2	34	A:0.882353	T:0.117647	
chr28	9539856	2	32	A:0.90625	G:0.09375	
chr28	9539946	2	36	C:0.805556	T:0.194444	
chr28	9540151	2	36	G:0.833333	A:0.166667	
chr28	9540383	2	36	T:0.888889	TA:0.111111	
chr28	9540981	2	40	C:1	T:0	
chr28	9542285	2	40	G:0.875	C:0.125	
chr28	9542524	2	38	C:1	G:0	
chr28	9542826	2	36	G:0.805556	C:0.194444	
chr28	9543002	2	38	G:0.710526	T:0.289474	
chr28	9543081	2	38	G:0.710526	C:0.289474	
chr28	9543089	2	38	T:1	TGTG:0	
chr28	9543110	2	36	GGT:0.888889	G:0.111111	
chr28	9543112	2	40	T:0.8	TGGTG:0.2	
chr28	9543441	2	38	CCCTCTA:0.842105	C:0.157895	
chr28	9543447	2	38	A:0.842105	C:0.157895	
chr28	9543483	2	36	TCTCA:0.805556	T:0.194444	
chr28	9543487	2	36	A:0.861111	T:0.138889	
chr28	9543735	2	38	C:0.736842	T:0.263158	
chr28	9543860	3	38	AACACACACAC:0.842105	A:0.131579	AACACACACACAC:0.0263158
chr28	9544056	2	36	A:0.666667	AT:0.333333	
chr28	9544375	2	38	G:0.868421	C:0.131579	
chr28	9544725	2	34	C:0.764706	CT:0.235294	
chr28	9544848	2	38	G:1	GTCA:0	
chr28	9545657	2	32	G:0.75	GC:0.25	
chr28	9545740	2	36	G:0.861111	A:0.138889	
chr28	9546080	2	36	C:0.75	CTAAA:0.25	
chr28	9546249	2	36	T:0.861111	A:0.138889	
chr28	9546401	2	38	G:0.684211	T:0.315789	
chr28	9547729	2	34	G:1	A:0	
chr28	9547754	2	38	G:0.631579	GAAACTT:0.368421	
chr28	9547967	2	36	T:0.888889	C:0.111111	
chr28	9548836	2	38	G:1	T:0	
chr28	9548879	2	38	C:0.973684	CCT:0.0263158	
chr28	9548927	2	34	T:0.764706	TA:0.235294	
chr28	9549186	2	38	TAA:0.763158	T:0.236842	
chr28	9549260	2	36	C:1	T:0	
chr28	9549466	2	40	T:0.725	G:0.275	
chr28	9549533	2	36	A:0.833333	G:0.166667	
chr28	9549617	2	38	T:0.868421	G:0.131579	
chr28	9550879	3	32	GAA:0.78125	G:0.125	GA:0.09375
chr28	9550955	2	34	T:0.764706	G:0.235294	
chr28	9551321	2	36	AT:0.916667	A:0.0833333	

chr28	9551324	2	36	T:0.0833333	A:0.916667
chr28	9551336	2	36	C:0.916667	T:0.0833333
chr28	9551751	2	36	A:0.861111	G:0.138889
chr28	9552058	2	38	G:1	A:0
chr28	9552210	2	36	A:1	G:0
chr28	9552368	2	40	A:0.9	G:0.1
chr28	9552896	2	38	GA:0.894737	G:0.105263
chr28	9552963	2	36	T:0.722222	C:0.277778
chr28	9553043	2	38	C:0.842105	CCTCT:0.157895
chr28	9553084	2	36	T:0.916667	TA:0.0833333
chr28	9553085	2	36	A:1	T:0
chr28	9553087	2	36	A:1	AT:0
chr28	9553185	2	38	ATTCTT:0.868421	A:0.131579
chr28	9553297	2	38	CAT:1	C:0
chr28	9553608	2	38	G:0.710526	A:0.289474
chr28	9553743	2	38	TA:0.815789	T:0.184211
chr28	9554716	2	38	T:1	G:0
chr28	9555719	2	36	A:0.861111	C:0.138889
chr28	9556220	3	36	TA:0.888889	T:0.0555556
TAA:0.0555556
chr28	9556926	2	34	C:0.911765	G:0.0882353
chr28	9557108	2	38	A:1	T:0
chr28	9557190	2	40	T:0.4	C:0.6
chr28	9557298	2	38	A:0.894737	T:0.105263
chr28	9557305	2	38	T:0.894737	A:0.105263
chr28	9557451	2	40	C:1	T:0
chr28	9558305	2	36	AAGAT:1	A:0
chr28	9558311	3	40	G:0.275	T:0.25
GATTTATTTATTTATTT:0.475
chr28	9558838	2	36	T:0.805556	C:0.194444
chr28	9559014	2	34	G:0.823529	GA:0.176471
chr28	9559483	2	34	G:0.441176	A:0.558824
chr28	9559536	6	38
ATTTCTTTTCTTTTCTTTTCTTTTCTTTTCTTTTCT:0.342105	A:0.157895
ATTTCT:0	ATTTCTTTTCTTTTCT:0.0789474	ATTTCTTTTCTTTTCTTTTCT:
0.157895	ATTTCTTTTCTTTTCTTTTCTTTTCT:0.263158
chr28	9559552	2	38	TTTCTTTTCTTTTCTTTTCTTTTCTTTTC:1
T:0
chr28	9560695	2	38	A:0.815789	G:0.184211
chr28	9560734	2	30	C:0.9	T:0.1
chr28	9561005	2	38	G:0.473684	GAA:0.526316
chr28	9561271	2	34	T:0.529412	C:0.470588
chr28	9561290	2	38	G:0.842105	A:0.157895
chr28	9561550	2	40	G:0.825	A:0.175
chr28	9561952	2	36	C:0.416667	CCT:0.583333
chr28	9561991	2	34	T:0.529412	A:0.470588
chr28	9561992	2	34	T:0.529412	A:0.470588
chr28	9562048	2	40	T:0.8
TAAGCGTCTGACTCTTAAAAATAAAAAAAAAAATAAATAAATAAAAAAAAAA:0.2
chr28	9562139	3	32	TAAAAA:0.40625	T:0.40625
TAAAA:0.1875
chr28	9562141	2	30	A:0.8	T:0.2
chr28	9562226	2	40	C:0.925	T:0.075
chr28	9562307	2	36	A:0.861111	G:0.138889

chr28 9562854 2 24 GTC:1 G:0
chr28 9562963 2 34 C:0.470588 T:0.529412
chr28 9563089 2 36 ACTTC:1 A:0
chr28 9563094 2 36 T:1 G:0
chr28 9563687 2 36 T:0.333333 C:0.666667
chr28 9563689 2 36 T:1 C:0
chr28 9563728 2 38 G:1 A:0
chr28 9563806 2 38 C:0.342105 T:0.657895
chr28 9563872 2 34 A:0.294118 G:0.705882
chr28 9563966 2 40 T:0.4 C:0.6
chr28 9564087 2 32 T:0.25 A:0.75
chr28 9564157 4 38 CATATATATATATATATAT:0.263158
C:0.447368 CAT:0.210526 CATATATATATATATAT:0.0789474
chr28 9564232 2 40 A:0.35 G:0.65
chr28 9564455 2 40 C:0.375 A:0.625
chr28 9564517 2 40 C:0.375 T:0.625
chr28 9564662 2 38 ATACC:0.447368 A:0.552632
chr28 9564839 2 38 A:0.710526 ATCTC:0.289474
chr28 9564860 2 38 T:0.684211 TC:0.315789
chr28 9564863 2 38 T:0.684211 TTC:0.315789
chr28 9564880 2 36 C:0.722222 T:0.277778
chr28 9564988 2 34 C:0.382353 T:0.617647
chr28 9565137 2 40 G:0.375 A:0.625
chr28 9565255 2 40 T:1 G:0
chr28 9565492 2 40 T:0.4 A:0.6
chr28 9565976 2 36 CT:0.666667 C:0.333333
chr28 9566010 2 40 C:0.45 T:0.55
chr28 9566172 2 36 CTTCCTAT:0.722222 C:
0.277778
chr28 9566326 2 40 T:0.675 C:0.325
chr28 9566408 2 36 T:0.333333 C:0.666667
chr28 9566466 2 36 T:1 C:0
chr28 9566635 2 30 CT:0.733333 C:0.266667
chr28 9566638 2 34 T:0.794118 C:0.205882
chr28 9566641 3 34 T:0.676471 TTTTC:0.323529
TTTTTC:0
chr28 9566845 2 34 A:0.441176 T:0.558824
chr28 9566980 3 36 CAA:0.388889 C:0.222222
CA:0.388889
chr28 9567018 2 36 C:0.361111 T:0.638889
chr28 9567089 2 36 A:0.305556 G:0.694444
chr28 9567242 2 38 C:0.394737 T:0.605263
chr28 9567334 2 36 T:0.388889 G:0.611111
chr28 9567374 2 34 T:0.441176 TAAAAAA:0.558824
chr28 9567491 2 34 A:0.294118 T:0.705882
chr28 9567567 2 40 A:0.375 G:0.625
chr28 9567838 2 40 T:0.625 C:0.375
chr28 9568410 2 40 C:0.65 T:0.35
chr28 9568453 2 38 A:0.5 C:0.5
chr28 9568758 2 34 T:0.705882 G:0.294118
chr28 9568823 2 36 T:0.444444 C:0.555556
chr28 9568867 2 32 T:0.96875 A:0.03125
chr28 9568868 2 34 A:0.676471 T:0.323529
chr28 9568876 2 34 A:0.676471 T:0.323529

chr28 9568938 2 38 TAC:0.605263 T:0.394737
chr28 9569039 2 32 C:0.6875 CAT:0.3125
chr28 9569139 2 38 A:1 G:0
chr28 9569192 2 34 A:0.705882 AG:0.294118
chr28 9569449 2 38 T:0.578947 C:0.421053
chr28 9569456 2 38 T:0.578947 C:0.421053
chr28 9569549 2 38 A:0.631579 T:0.368421
chr28 9569567 2 38 A:0.473684 G:0.526316
chr28 9569730 2 38 A:0.605263 G:0.394737
chr28 9569792 2 32 C:0.71875 T:0.28125
chr28 9569865 2 34 T:1 TA:0
chr28 9569873 3 38 A:0.473684 AAAG:0.342105
AAAAAAG:0.184211
chr28 9569932 2 38 T:0.684211 G:0.315789
chr28 9569936 2 38 T:0.684211 C:0.315789
chr28 9570043 2 36 T:0.722222 A:0.277778
chr28 9570739 2 32 T:0.40625 TA:0.59375
chr28 9570895 2 36 C:0.583333 T:0.416667
chr28 9571027 2 38 T:0.710526 A:0.289474
chr28 9571090 2 38 A:0.684211 T:0.315789
chr28 9571210 2 36 C:0.638889 T:0.361111
chr28 9571231 2 36 TA:0.694444 T:0.305556
chr28 9571414 2 36 T:0.444444 G:0.555556
chr28 9571507 2 38 A:0.473684 G:0.526316
chr28 9571879 2 30 A:0.466667 T:0.533333
chr28 9572284 2 36 TCAG:0.75 T:0.25
chr28 9572372 4 26 AG:0.423077 A:0.423077
AGG:0.0384615 AGGG:0.115385
chr28 9574033 2 36 G:0.666667 T:0.333333
chr28 9574255 2 36 G:0.611111 T:0.388889
chr28 9574382 2 40 G:0.675 C:0.325
chr28 9574497 2 38 G:0.605263 GT:0.394737
chr28 9574508 2 38 T:0.605263 TTTA:0.394737
chr28 9574595 2 38 C:0.710526 T:0.289474
chr28 9574685 2 38 C:0.578947 T:0.421053
chr28 9574911 2 36 G:0.638889 T:0.361111
chr28 9575111 2 40 T:0.125 TG:0.875
chr28 9575539 2 40 T:0.65 TC:0.35
chr28 9575563 2 40 G:0.65 T:0.35
chr28 9575754 2 36 A:1 G:0
chr28 9575783 2 38 A:0.973684 T:0.0263158
chr28 9576019 2 38 A:0.684211 T:0.315789
chr28 9576029 2 38 A:0.684211 T:0.315789
chr28 9576357 2 36 T:0.694444 G:0.305556
chr28 9576358 2 36 T:0.694444 C:0.305556
chr28 9576589 2 38 G:0.657895 A:0.342105
chr28 9576677 2 40 C:0.475 G:0.525
chr28 9577179 2 34 ATAAAG:0.588235 A:0.411765
chr28 9577329 2 20 TG:0.25 T:0.75
chr28 9577593 2 38 A:1 G:0
chr28 9577819 2 38 A:1 G:0
chr28 9577997 2 34 C:0.529412 T:0.470588
chr28 9578021 2 38 C:0.921053 CGGG:0.0789474
chr28 9578022 2 38 A:0.921053 ATCCCTGGGTGGCGC:

0.0789474
chr28	9578024	2	38	G:0.921053
GCGGTTTGGCGCCTGCCT:0.0789474
chr28	9578050	2	34	G:0.705882	A:0.294118
chr28	9578247	2	34	A:0.735294	G:0.264706
chr28	9578336	2	40	A:0.675
ATTATCTTTATTATTTTCTTTTTTTTTTTTT:0.325
chr28	9578932	2	34	T:0.705882	G:0.294118
chr28	9579011	2	40	A:0.7	ATATAGAATATATGTCAT:0.3
chr28	9579015	2	40	A:0.7	ATAT:0.3
chr28	9579016	2	40	C:0.7	G:0.3
chr28	9579207	2	36	G:0.611111	A:0.388889
chr28	9579410	2	36	C:1	T:0
chr28	9579440	2	36	T:0.722222	C:0.277778
chr28	9579871	2	32	G:0.75	T:0.25
chr28	9580107	2	40	A:0.775	G:0.225
chr28	9580128	2	40	C:0.5	T:0.5
chr28	9580216	2	38	G:0.421053	A:0.578947
chr28	9580396	2	38	T:0.736842	G:0.263158
chr28	9580457	2	38	G:0.657895	A:0.342105
chr28	9580922	2	36	A:0.638889	AT:0.361111
chr28	9580931	2	38	C:0.684211	CCA:0.315789
chr28	9581200	2	32	TA:0.65625	T:0.34375
chr28	9581214	2	36	AT:1	A:0
chr28	9581215	2	36	T:0.861111	A:0.138889
chr28	9581374	2	36	G:0.638889	A:0.361111
chr28	9581495	2	32	G:1	A:0
chr28	9581533	2	34	G:0.705882	A:0.294118
chr28	9581568	2	32	G:0.78125	A:0.21875
chr28	9581640	2	34	T:0.558824	C:0.441176
chr28	9581651	2	36	C:0.0555556	T:0.944444
chr28	9581821	2	32	G:0.71875	A:0.28125
chr28	9581918	2	38	A:0.605263	G:0.394737
chr28	9582135	2	32	T:0.65625	C:0.34375
chr28	9582608	2	36	A:0.611111	G:0.388889
chr28	9582966	2	38	A:0.684211	AT:0.315789
chr28	9582975	2	38	G:0.684211	A:0.315789
chr28	9582976	2	38	C:0.842105	CT:0.157895
chr28	9582986	2	38	T:0.684211	TA:0.315789
chr28	9583014	2	38	TGAGA:0.842105	T:0.157895
chr28	9583915	2	36	A:0.888889	AGT:0.111111
chr28	9584159	2	36	G:0.916667	GC:0.0833333
chr28	9584161	2	36	G:0.916667	A:0.0833333
chr28	9584202	2	36	A:0.25	G:0.75
chr28	9584260	2	28	G:1	GTC:0
chr28	9584262	2	34	C:0.764706	CTA:0.235294
chr28	9585083	2	36	G:0.666667	A:0.333333
chr28	9585095	2	36	A:0.666667	G:0.333333
chr28	9585230	2	38	C:0.131579	T:0.868421
chr28	9585360	2	34	G:0.588235	GT:0.411765
chr28	9586291	2	40	A:0.7	G:0.3
chr28	9586670	2	36	G:1	A:0
chr28	9587213	2	38	C:0.710526	CT:0.289474
chr28	9587279	2	38	T:0.736842	C:0.263158

chr28	9587607	2	38	C:0.631579	G:0.368421	
chr28	9587634	2	38	T:0.868421	C:0.131579	
chr28	9587706	2	36	G:0.583333	GT:0.416667	
chr28	9588002	2	34	C:0.647059	T:0.352941	
chr28	9588003	2	34	A:0.647059	G:0.352941	
chr28	9589333	2	36	G:0.638889	A:0.361111	
chr28	9590225	2	36	G:0.472222	A:0.527778	
chr28	9590592	2	38	G:0.710526	T:0.289474	
chr28	9590966	2	38	A:0.473684	G:0.526316	
chr28	9591226	3	34	GTA:0.5	G:0.147059	GTATATA:0.352941
chr28	9591411	2	32	A:0.4375	T:0.5625	
chr28	9591551	2	38	C:0.710526	T:0.289474	
chr28	9591697	2	40	A:0.675	C:0.325	
chr28	9591720	2	38	C:0.868421	T:0.131579	
chr28	9591912	2	40	A:0.7	AG:0.3	
chr28	9591913	3	40	AT:0.375	A:0.325	TT:0.3
chr28	9592007	2	38	G:0.815789	A:0.184211	
chr28	9592121	2	36	C:0.805556	T:0.194444	
chr28	9592682	2	38	G:0.684211	GT:0.315789	
chr28	9593230	2	40	T:0.425	C:0.575	
chr28	9593310	2	36	C:0.694444	T:0.305556	
chr28	9593919	2	38	C:0.605263	G:0.394737	
chr28	9593973	2	40	T:1	C:0	
chr28	9594038	2	36	G:0.666667	A:0.333333	
chr28	9595125	2	36	T:0.722222	C:0.277778	
chr28	9595450	2	38	G:1	T:0	
chr28	9595656	2	36	T:0.611111	C:0.388889	
chr28	9596638	2	40	T:0.475	C:0.525	
chr28	9596694	2	36	G:0.972222	A:0.0277778	
chr28	9597063	2	40	T:1	C:0	
chr28	9599398	2	32	A:0.59375	AT:0.40625	
chr28	9599515	2	38	C:0.526316	T:0.473684	
chr28	9599719	2	36	C:0.888889	T:0.111111	
chr28	9599929	2	34	G:1	A:0	
chr28	9599962	3	30	CT:0.3	C:0.266667	CTT:0.433333
chr28	9600142	2	36	G:0.638889	GAA:0.361111	
chr28	9600972	2	38	G:0.789474	A:0.210526	
chr28	9601336	2	36	T:0.777778	C:0.222222	
chr28	9601346	2	34	G:0.382353	GTTT:0.617647	
chr28	9601688	2	38	A:1	T:0	
chr28	9601819	2	40	C:0.725	CT:0.275	
chr28	9602071	2	36	G:0.888889	T:0.111111	
chr28	9602515	2	38	C:0.210526	CAGAG:0.789474	
chr28	9602556	2	36	T:0.138889	C:0.861111	
chr28	9602709	2	36	G:0.694444	T:0.305556	
chr28	9602781	2	40	G:0.65	A:0.35	
chr28	9603071	2	40	G:0.725	A:0.275	
chr28	9603595	2	32	C:0.78125	T:0.21875	
chr28	9603705	2	40	CAAATGT:0.675	C:0.325	
chr28	9603737	2	40	G:0.125	A:0.875	
chr28	9603866	2	30	A:0.6	G:0.4	
chr28	9604113	2	34	C:0.676471	CAAT:0.323529	

chr28	9604502	2	38	C:0.657895	T:0.342105	
chr28	9604526	2	36	C:0.555556	G:0.444444	
chr28	9604595	2	36	A:0.861111	AT:0.138889	
chr28	9604900	2	40	C:0.075	T:0.925	
chr28	9604964	2	36	A:0.166667	AATT:0.833333	
chr28	9605189	2	36	T:0.666667	C:0.333333	
chr28	9605274	2	38	C:0.631579	T:0.368421	
chr28	9605900	2	38	A:1	G:0	
chr28	9606015	2	38	A:1	G:0	
chr28	9606378	2	38	A:0.868421	C:0.131579	
chr28	9607071	2	40	A:0.15	G:0.85	
chr28	9607195	2	38	G:0.684211	A:0.315789	
chr28	9607695	2	40	T:0.925	TG:0.075	
chr28	9608901	2	40	C:0.6	T:0.4	
chr28	9609333	2	36	C:0.888889	T:0.111111	
chr28	9609384	2	40	A:0.9	G:0.1	
chr28	9609453	2	38	AG:0.894737	A:0.105263	
chr28	9609874	2	38	T:0.631579	C:0.368421	
chr28	9609894	2	36	G:0.694444	A:0.305556	
chr28	9609925	2	38	C:0.131579	CAAAT:0.868421	
chr28	9610424	2	36	C:0.638889	G:0.361111	
chr28	9610717	2	36	T:0.583333	A:0.416667	
chr28	9610718	3	36	T:0.416667	A:0.416667	TA:0.166667
chr28	9610948	2	34	A:0.176471	T:0.823529	
chr28	9611116	2	26	C:0.115385	CTT:0.884615	
chr28	9612034	2	38	G:0.710526	A:0.289474	
chr28	9612691	2	36	G:0.638889	A:0.361111	
chr28	9612988	2	34	G:0.852941	A:0.147059	
chr28	9613566	2	36	C:0.583333	CT:0.416667	
chr28	9614922	2	40	ACTTT:0.55	A:0.45	
chr28	9615103	2	36	C:0.555556	G:0.444444	
chr28	9615195	3	38	G:0.684211	GA:0.131579	GAA:0.184211
chr28	9615206	2	38	C:0.710526	A:0.289474	
chr28	9615427	2	36	T:0.888889	C:0.111111	
chr28	9615564	2	38	T:0.842105	G:0.157895	
chr28	9616775	2	38	A:0.789474	C:0.210526	
chr28	9616846	2	40	A:0.975	C:0.025	
chr28	9616922	2	28	TA:0.392857	T:0.607143	
chr28	9616934	2	28	A:0.392857	C:0.607143	
chr28	9617161	2	34	T:0.558824	C:0.441176	
chr28	9617887	2	34	C:0.823529	T:0.176471	
chr28	9618073	2	38	A:0.526316	T:0.473684	
chr28	9619536	2	38	A:0.631579	G:0.368421	
chr28	9620179	2	40	AG:0.15	A:0.85	
chr28	9620202	2	40	G:0.975	A:0.025	
chr28	9620729	2	38	A:0.447368	T:0.552632	
chr28	9620950	2	36	T:0.138889	C:0.861111	
chr28	9621608	2	38	C:1	T:0	
chr28	9622738	2	40	ATT:1	A:0	
chr28	9623784	2	40	A:0.425	G:0.575	
chr28	9623870	2	38	A:1	G:0	
chr28	9624327	2	36	C:0.361111	T:0.638889	

chr28	9624715	2	40	A:0.825	G:0.175		
chr28	9625347	2	38	T:0.131579	C:0.868421		
chr28	9625621	2	34	A:0.558824	G:0.441176		
chr28	9626145	2	34	TG:1	T:0		
chr28	9626211	2	34	T:1	C:0		
chr28	9627016	2	38	A:1	C:0		
chr28	9627355	2	38	C:0.210526	A:0.789474		
chr28	9628345	2	32	C:0.9375	T:0.0625		
chr28	9629034	2	38	A:0.131579	T:0.868421		
chr28	9630247	2	40	A:0.65	AAG:0.35		
chr28	9630576	2	32	C:0.1875	CAT:0.8125		
chr28	9630904	2	38	A:0.421053	G:0.578947		
chr28	9630930	2	38	C:1	T:0		
chr28	9630936	2	38	A:0.868421	T:0.131579		
chr28	9631549	2	40	A:0.15	T:0.85		
chr28	9632064	2	40	CT:1	C:0		
chr28	9632137	4	40	G:0.475	GCATT:0.05	GCATTCATT:0.25	GCATTCATTCATT:0.225
chr28	9632654	2	38	G:0.605263	A:0.394737		
chr28	9632733	2	36	A:0.194444	ATCTG:0.805556		
chr28	9632756	2	38	TGGCTACAG:0.763158	T: 0.236842		
chr28	9632783	2	36	G:1	A:0		
chr28	9632961	2	36	T:1	TTTTG:0		
chr28	9633726	2	36	C:0.138889	T:0.861111		
chr28	9633821	2	30	GATTT:0.8	G:0.2		
chr28	9634589	2	32	C:0.46875	CT:0.53125		
chr28	9634607	2	38	T:0.789474	G:0.210526		
chr28	9634619	2	40	TTTTG:0.8	T:0.2		
chr28	9634690	2	36	G:0.527778	A:0.472222		
chr28	9634767	2	34	G:0.882353	A:0.117647		
chr28	9635248	2	40	G:1	T:0		
chr28	9635437	2	36	G:1	A:0		
chr28	9635479	2	34	G:1	T:0		
chr28	9635722	2	34	C:0.882353	T:0.117647		
chr28	9637485	2	38	G:0.184211	A:0.815789		
chr28	9637689	2	36	G:0.25	A:0.75		
chr28	9638048	2	38	A:0	AT:1		
chr28	9638178	3	40	TAA:0.575	T:0.1	TA:0.325	
chr28	9638628	2	34	C:0.588235	T:0.411765		
chr28	9638765	2	38	G:0.815789	A:0.184211		
chr28	9638891	2	36	AT:0.75	A:0.25		
chr28	9638897	2	36	T:0.75	A:0.25		
chr28	9638903	2	34	TTTTTA:0.911765	T:0.0882353		
chr28	9638904	2	34	TTTTA:0.882353	T:0.117647		
chr28	9639007	2	38	T:0.684211	C:0.315789		
chr28	9639582	2	36	G:1	A:0		
chr28	9640520	2	36	T:0.944444	C:0.0555556		
chr28	9640812	2	34	C:0.205882	T:0.794118		
chr28	9640917	2	34	A:1	T:0		
chr28	9641210	2	36	C:0.638889	T:0.361111		
chr28	9641320	3	30	TAAAAAAA:0.733333	T: 0.133333	TA:0.133333	
chr28	9641432	2	32	GAT:0.84375	G:0.15625		

chr28 9641446 4 40 T:0.85
TATATATATATGATGTCATATATATATGATGTCATC:0.1
TATATATATATATGATGTCATATATATATGATGTCATC:0.05
TATATATATATATATGATGTCATATATATATGATGTCATC:0
chr28 9641936 2 40 A:0.675 G:0.325
chr28 9642104 2 38 C:0.684211 G:0.315789
chr28 9642174 2 32 G:0.84375 T:0.15625
chr28 9643130 2 38 GA:0.684211 G:0.315789
chr28 9646093 2 40 A:0.275 G:0.725
chr28 9646782 2 32 C:0.78125 CT:0.21875
chr28 9647574 2 36 T:0.777778 TA:0.222222
chr28 9647680 3 40 A:0.375 AGTGTGTGT:0.3
AGTGTGTGTGT:0.325
chr28 9647845 2 40 T:0.125 C:0.875
chr28 9648320 2 36 G:1 A:0
chr28 9648412 2 38 C:0.842105 T:0.157895
chr28 9648490 2 40 C:1 T:0
chr28 9648986 2 38 A:1 G:0
chr28 9649214 2 40 A:0.45 G:0.55
chr28 9649446 2 38 A:0.552632 C:0.447368
chr28 9649505 2 40 A:0.55 G:0.45
chr28 9650323 3 30 G:0.3 GTT:0.266667 GTTT:
0.433333
chr28 9650634 2 36 C:0.555556 A:0.444444
chr28 9650639 2 36 T:1 C:0
chr28 9651038 2 38 T:0.5 C:0.5
chr28 9651083 2 38 CTT:0.578947 C:0.421053
chr28 9651369 2 34 A:0.882353 G:0.117647
chr28 9651668 2 40 T:0.625 C:0.375
chr28 9651680 2 40 G:0.475 A:0.525
chr28 9652019 2 38 TGTCA:0.421053 T:0.578947
chr28 9652056 2 38 G:0.684211 C:0.315789
chr28 9652957 2 36 T:0.527778 C:0.472222
chr28 9653028 2 36 T:0.444444 C:0.555556
chr28 9653703 2 38 T:0.657895 C:0.342105
chr28 9653750 2 36 A:0.861111 G:0.138889
chr28 9653818 2 38 T:0.526316 TTAA:0.473684
chr28 9654143 2 32 A:0.65625 G:0.34375
chr28 9654174 2 30 C:0.733333 T:0.266667
chr28 9654445 2 20 G:0.65 GC:0.35
chr28 9654971 2 36 A:0.666667 G:0.333333
chr28 9655006 2 38 C:0.473684 T:0.526316
chr28 9655026 2 40 T:0.35 C:0.65
chr28 9655278 2 38 T:0.894737
TGAGTTACCCCTCACTG:0.105263
chr28 9655280 2 38 A:0.631579
AGTTACCCCTCACTGGG:0.368421
chr28 9655689 2 38 C:0.684211 T:0.315789
chr28 9655751 2 30 C:0.9 T:0.1
chr28 9655846 2 36 G:0.611111 A:0.388889
chr28 9656111 2 40 A:0.95 G:0.05
chr28 9656180 2 34 T:0.441176 G:0.558824
chr28 9656183 2 34 C:0.794118 CA:0.205882
chr28 9656400 2 38 G:0.842105 A:0.157895

chr28	9656541	2	38	A:0.421053	C:0.578947
chr28	9656548	2	38	G:0.815789	A:0.184211
chr28	9656876	2	34	A:0.852941	T:0.147059
chr28	9657139	2	22	T:0.318182	C:0.681818
chr28	9657309	2	40	GT:0.55	G:0.45
chr28	9657397	2	36	G:0.861111	A:0.138889
chr28	9657624	2	34	T:0.323529	A:0.676471
chr28	9657795	2	24	C:0.666667	A:0.333333
chr28	9657904	2	26	G:0.884615	A:0.115385
chr28	9657940	2	34	A:0.588235	AGCATGCAGT:0.411765
chr28	9658171	2	34	TC:0.852941	T:0.147059
chr28	9658397	2	40	T:0.45	C:0.55
chr28	9658782	2	38	C:0.710526	T:0.289474
chr28	9658807	2	38	A:0.552632	T:0.447368
chr28	9659055	2	36	A:0.444444	AGC:0.555556
chr28	9659105	2	38	T:0.552632	TGCTTCCGCTGTGTCAG:0.447368
chr28	9659176	2	38	G:0.421053	GA:0.578947
chr28	9659199	2	38	A:0.605263	G:0.394737
chr28	9659205	2	38	G:0.605263	A:0.394737
chr28	9659489	2	40	CAA:0.85	C:0.15
chr28	9659589	2	36	C:0.583333	T:0.416667
chr28	9659647	2	36	T:0.333333	C:0.666667
chr28	9659741	2	38	G:0.578947	A:0.421053
chr28	9659752	2	38	T:0.736842	A:0.263158
chr28	9659891	2	32	G:0.5	GC:0.5
chr28	9660193	2	40	G:0.825	T:0.175
chr28	9660414	2	40	C:0.7	T:0.3
chr28	9660529	2	36	C:0.833333	T:0.166667
chr28	9660628	2	40	T:0.575	G:0.425
chr28	9660887	2	36	T:0.166667	C:0.833333
chr28	9661344	2	36	G:0.916667	T:0.0833333
chr28	9661812	2	34	C:0.264706	G:0.735294
chr28	9661982	2	32	TAA:0.84375	T:0.15625
chr28	9661983	2	32	A:0.9375	T:0.0625
chr28	9662011	2	34	C:0.617647	CAG:0.382353
chr28	9662101	2	32	A:0.1875	T:0.8125
chr28	9662113	2	32	C:0.84375	T:0.15625
chr28	9662146	2	36	T:0.305556	C:0.694444
chr28	9662157	2	36	G:0.583333	A:0.416667
chr28	9662260	2	38	C:0.657895	T:0.342105
chr28	9662584	2	36	A:0.75	T:0.25
chr28	9662786	2	36	T:0.861111	C:0.138889
chr28	9662830	2	36	C:0.916667	T:0.0833333
chr28	9663399	2	40	C:0.8	T:0.2
chr28	9663510	2	38	A:0.473684	G:0.526316
chr28	9663899	2	38	G:0.552632	A:0.447368
chr28	9664071	2	36	T:0.444444	C:0.555556
chr28	9664231	2	40	T:0.675	A:0.325
chr28	9664387	2	38	G:0.473684	A:0.526316
chr28	9664447	2	40	G:0.65	A:0.35
chr28	9664638	2	40	A:0.375	G:0.625
chr28	9665033	2	36	T:0.805556	C:0.194444

chr28	9665243	2	36	G:0.694444	A:0.305556	
chr28	9665256	2	36	C:0.555556	G:0.444444	
chr28	9665461	2	36	G:0.666667	A:0.333333	
chr28	9665513	2	34	C:1	T:0	
chr28	9666121	2	32	A:0.375	T:0.625	
chr28	9666214	2	40	G:0.675	A:0.325	
chr28	9666242	2	38	A:0.447368	G:0.552632	
chr28	9666302	2	40	T:0.525	G:0.475	
chr28	9666472	2	40	G:0.825	C:0.175	
chr28	9666500	2	36	G:1	A:0	
chr28	9666626	2	26	C:0.576923	CT:0.423077	
chr28	9666723	2	40	C:0.9	T:0.1	
chr28	9666783	2	38	C:0.815789	G:0.184211	
chr28	9666948	2	36	G:0.5	T:0.5	
chr28	9667079	2	38	T:0.631579	C:0.368421	
chr28	9667086	2	38	C:0.894737	CCG:0.105263	
chr28	9667231	2	38	C:0.473684	A:0.526316	
chr28	9667505	2	38	C:0.657895	T:0.342105	
chr28	9667701	2	34	C:0.823529	T:0.176471	
chr28	9667996	2	36	A:0.555556	G:0.444444	
chr28	9668104	2	34	C:0.558824	T:0.441176	
chr28	9668263	2	38	A:0.815789	G:0.184211	
chr28	9668410	2	36	C:0	CA:1	
chr28	9668421	2	36	TG:0	T:1	
chr28	9668423	2	36	T:0	A:1	
chr28	9668439	2	38	C:0.921053	T:0.0789474	
chr28	9668995	2	40	G:0.6	A:0.4	
chr28	9669489	2	36	G:0.805556	A:0.194444	
chr28	9669533	2	38	T:0.815789	C:0.184211	
chr28	9669608	2	40	T:0.875	C:0.125	
chr28	9669803	2	36	T:0.0555556	C:0.944444	
chr28	9669900	2	40	T:0.625	TCC:0.375	
chr28	9669906	3	40	A:0.6	AAAAAC:0.1	AAAAC:0.3
chr28	9670366	2	32	A:0	G:1	
chr28	9671318	2	36	G:0.694444	A:0.305556	
chr28	9671397	2	38	C:0.315789	T:0.684211	
chr28	9671732	2	38	A:0.842105	G:0.157895	
chr28	9671764	2	38	C:0.736842	T:0.263158	
chr28	9671862	2	38	T:0.421053	C:0.578947	
chr28	9671913	2	38	A:0.921053	G:0.0789474	
chr28	9671943	2	40	A:0.45	ACTGT:0.55	
chr28	9672157	2	38	C:0.631579	CT:0.368421	
chr28	9672165	2	38	T:0.921053	A:0.0789474	
chr28	9672255	2	38	T:0.210526	G:0.789474	
chr28	9672418	2	38	A:0.605263	G:0.394737	
chr28	9672819	2	36	C:1	CT:0	
chr28	9672838	2	38	C:0.526316	T:0.473684	
chr28	9672902	2	36	A:0.472222	G:0.527778	
chr28	9672992	2	28	T:1	C:0	
chr28	9673011	2	28	A:1	C:0	
chr28	9673012	2	28	T:1	C:0	
chr28	9673119	2	16	C:1	T:0	
chr28	9673653	2	30	CA:0.8	C:0.2	

chr28	9673654	2	30	A:0.9	C:0.1		
chr28	9673757	2	36	C:0.777778	T:0.222222		
chr28	9674395	2	22	C:0.454545	T:0.545455		
chr28	9674746	2	40	G:1	T:0		
chr28	9674957	2	30	T:0.833333	TA:0.166667		
chr28	9675054	2	40	G:0.55	GA:0.45		
chr28	9675246	2	38	C:0.815789	CT:0.184211		
chr28	9675392	2	38	G:0.973684	A:0.0263158		
chr28	9675737	2	34	C:0.117647	T:0.882353		
chr28	9676395	2	38	C:0.842105	T:0.157895		
chr28	9676547	2	34	C:0.823529	T:0.176471		
chr28	9676732	2	38	G:0.894737	A:0.105263		
chr28	9676832	2	36	T:0.861111	C:0.138889		
chr28	9677014	2	36	A:0.833333	G:0.166667		
chr28	9677144	2	36	A:0.861111	T:0.138889		
chr28	9677639	2	38	A:0.894737	ATTG:0.105263		
chr28	9677825	2	38	G:0.0789474	A:0.921053		
chr28	9677987	2	38	G:1	C:0		
chr28	9678142	2	38	A:0.157895	C:0.842105		
chr28	9678350	2	40	C:0.075	A:0.925		
chr28	9678399	2	36	G:0.166667	A:0.833333		
chr28	9678745	2	38	G:0.815789	T:0.184211		
chr28	9679435	2	38	A:0.763158	G:0.236842		
chr28	9679624	2	34	A:1	C:0		
chr28	9679710	2	36	A:0.805556	G:0.194444		
chr28	9680298	2	36	C:0.833333	T:0.166667		
chr28	9680322	2	36	T:0.833333	C:0.166667		
chr28	9680453	2	40	C:0.35	T:0.65		
chr28	9680576	2	40	C:0.7	T:0.3		
chr28	9680940	2	34	A:0.117647	G:0.882353		
chr28	9681149	2	38	G:0.842105	A:0.157895		
chr28	9681230	2	38	T:0.105263	C:0.894737		
chr28	9681350	2	34	C:0.852941	T:0.147059		
chr28	9681525	2	38	C:0.736842	T:0.263158		
chr28	9681616	2	36	G:0.166667	A:0.833333		
chr28	9682796	2	40	A:0.925	T:0.075		
chr28	9684073	2	26	A:0.846154	G:0.153846		
chr28	9684650	2	36	G:0.222222	GTA:0.777778		
chr28	9684944	2	34	T:0.411765	C:0.588235		
chr28	9685059	2	32	C:0.71875	CCTCTCT:0.28125		
chr28	9685108	4	32	TAA:0.28125	T:0.4375	TA:0.21875	TAAA:0.0625
chr28	9686194	2	34	G:0.882353	A:0.117647		
chr28	9687510	2	36	G:1	A:0		
chr28	9687764	2	38	A:0.368421	C:0.631579		
chr28	9688323	2	36	C:0.416667	G:0.583333		
chr28	9688775	2	34	A:0.852941	G:0.147059		
chr28	9690095	2	38	A:0.842105	AAAT:0.157895		
chr28	9690750	2	36	C:0.888889	CAG:0.111111		
chr28	9691046	2	38	T:0.868421	TAA:0.131579		
chr28	9691065	2	36	AAAT:0.833333	A:0.166667		
chr28	9691066	2	34	AAT:0.647059	A:0.352941		
chr28	9691068	3	38	TAA:1	T:0	TA:0	
chr28	9691517	2	34	T:0.882353	A:0.117647		

chr28	9692141	2	36	A:0.861111	T:0.138889		
chr28	9692875	2	34	C:0.617647	T:0.382353		
chr28	9693030	2	36	C:0.833333	CG:0.166667		
chr28	9693291	2	32	G:0.1875	A:0.8125		
chr28	9693366	2	34	A:0.147059	G:0.852941		
chr28	9694301	3	34	ACT:0.852941	A:0	ACTCT:0.147059	
chr28	9694803	2	36	A:0.888889	C:0.111111		
chr28	9695387	2	8	TGGGGG:0.5	T:0.5		
chr28	9696099	2	38	G:0.184211	T:0.815789		
chr28	9696868	2	38	T:0.684211	C:0.315789		
chr28	9696922	2	38	C:0.684211	T:0.315789		
chr28	9697041	3	34	A:0.176471	ATTTCTTTC:0.558824	ATTTCTTTCTTTC:0.264706	
chr28	9697490	2	38	G:1	A:0		
chr28	9697717	2	34	A:0.852941	T:0.147059		
chr28	9697967	2	28	A:0.357143	AT:0.642857		
chr28	9698894	2	38	CTTTTTTTTTTTT:0.736842	C:0.263158		
chr28	9699500	3	32	G:0.46875	GTT:0.40625	GTTT:0.125	
chr28	9701740	2	38	G:0.842105	T:0.157895		
chr28	9703418	2	38	C:0.578947	T:0.421053		
chr28	9703677	2	34	T:1	A:0		
chr28	9704056	2	38	T:0.973684	C:0.0263158		
chr28	9704171	2	36	G:0.833333	A:0.166667		
chr28	9704381	2	36	C:0.694444	T:0.305556		
chr28	9704426	2	38	T:0.105263	C:0.894737		
chr28	9704625	2	36	C:0.638889	T:0.361111		
chr28	9704945	2	36	G:0.166667	A:0.833333		
chr28	9706082	2	40	T:0.475	C:0.525		
chr28	9706194	2	34	G:0.0882353	T:0.911765		
chr28	9706698	2	40	C:0.15	T:0.85		
chr28	9707018	2	38	C:0.868421	CAG:0.131579		
chr28	9707475	2	38	T:0.842105	C:0.157895		
chr28	9708142	2	38	G:0.868421	A:0.131579		
chr28	9708223	4	40	TAATAAAATAAAATAA:0.35	T:0.375	TAATAAAATAA:0.05	TAATAAAATAAAATAAAATAA:0.225
chr28	9710033	2	36	A:0	G:1		
chr28	9710282	3	40	CATAGAT:0.6	C:0.35	CATAGATATAGAT:0.05	
chr28	9710299	2	40	A:1	ATC:0		
chr28	9710301	2	40	A:1	AGATCTAGATC:0		
chr28	9710322	2	40	G:1	GATATAGAT:0		
chr28	9710326	2	40	G:1	T:0		
chr28	9710328	3	40	T:1	G:0	TATAG:0	
chr28	9711031	2	36	AGTGT:0.833333	A:0.166667		
chr28	9711258	2	34	G:0.676471	A:0.323529		
chr28	9712579	2	36	T:1	TA:0		
chr28	9713224	2	38	C:1	G:0		
chr28	9713970	2	36	A:0.833333	G:0.166667		
chr28	9714649	2	38	CAATTTAATTACTTCAATTAAAAAA:0	C:1		
chr28	9714674	2	38	A:0	AGG:1		

chr28 9715061 2 34 T:1 TA:0
chr28 9715135 2 38 T:0.973684 A:0.0263158
chr28 9715311 2 36 C:1 T:0
chr28 9715329 3 36 TA:0.694444 T:0.111111
TAA:0.194444
chr28 9715348 2 36 T:0.138889 C:0.861111
chr28 9715604 2 40 G:0.15 T:0.85
chr28 9716009 2 40 G:0.85 A:0.15
chr28 9716417 2 38 CT:0.736842 C:0.263158
chr28 9717522 2 40 G:0.675 A:0.325
chr28 9717530 5 40 ATGTG:0 A:0.15 ATG:0
ATGTGTGTGTG:0.275 ATGTGTGTGTGTG:0.575
chr28 9717732 2 38 C:1 T:0
chr28 9717766 2 38 ACT:0.105263 A:0.894737
chr28 9718146 2 34 T:0.852941 C:0.147059
chr28 9718514 2 38 A:0.184211 G:0.815789
chr28 9718616 2 32 TA:0.875 T:0.125
chr28 9718710 2 36 A:0.111111 C:0.888889
chr28 9719127 2 34 G:0.852941 T:0.147059
chr28 9719292 2 36 C:0.111111 T:0.888889
chr28 9719408 2 34 G:0.705882 A:0.294118
chr28 9719755 2 34 A:0.911765 C:0.0882353
chr28 9720134 2 40 C:0.725 T:0.275
chr28 9721639 2 36 T:0.194444 C:0.805556
chr28 9721686 2 40 C:1 T:0
chr28 9721729 2 38 C:0.789474 T:0.210526
chr28 9722300 2 38 C:0.131579 T:0.868421
chr28 9722335 2 36 C:0.861111 T:0.138889
chr28 9722472 2 40 G:0.975 A:0.025
chr28 9722667 2 34 A:0.794118 C:0.205882
chr28 9722668 2 34 GT:0.794118 G:0.205882
chr28 9722959 2 38 C:1 T:0
chr28 9723206 2 34 A:1 G:0
chr28 9723630 2 32 G:0.125 A:0.875
chr28 9723919 2 36 C:0.861111 G:0.138889
chr28 9723997 2 36 G:0.861111 A:0.138889
chr28 9724006 2 40 A:1 G:0
chr28 9724574 2 36 G:1 C:0
chr28 9724633 2 36 T:1 C:0
chr28 9724744 2 38 C:1 T:0
chr28 9724827 2 36 A:1 G:0
chr28 9724894 2 36 T:1 C:0
chr28 9724895 2 36 T:1 C:0
chr28 9725235 2 40 A:1 AC:0
chr28 9725236 2 40 A:1 T:0
chr28 9725255 2 40 T:1 C:0
chr28 9725275 2 40 T:1 C:0
chr28 9725447 2 38 T:1 G:0
chr28 9725570 2 36 G:0.722222 A:0.277778
chr28 9725663 2 34 G:1 A:0
chr28 9725823 2 36 T:1 C:0
chr28 9725868 2 36 TG:0.861111 T:0.138889
chr28 9725883 2 34 A:1 C:0
chr28 9725982 2 38 T:1 C:0

chr28	9726140	2	38	CT:1	C:0		
chr28	9726488	2	38	T:0.631579	C:0.368421		
chr28	9726505	2	40	TA:0.15	T:0.85		
chr28	9727078	2	40	T:0.125	C:0.875		
chr28	9727823	2	40	C:1	T:0		
chr28	9727831	2	40	AAAACGT:0.475	A:0.525		
chr28	9728288	2	36	T:0.805556	C:0.194444		
chr28	9728361	2	38	A:1	T:0		
chr28	9728491	2	36	T:0.0833333	G:0.916667		
chr28	9729023	2	40	G:0.875	A:0.125		
chr28	9729591	4	40	TAATGAATG:0.4	T:0.25	TAATG:0.35	TAATGAATGAATG:0
chr28	9730419	2	38	T:0.868421	A:0.131579		
chr28	9730820	2	38	T:0	C:1		
chr28	9730882	2	38	T:1	C:0		
chr28	9731081	2	32	C:0.875	T:0.125		
chr28	9731210	2	38	A:1	AT:0		
chr28	9731212	2	38	A:1	T:0		
chr28	9731223	2	38	GAT:0.157895	G:0.842105		
chr28	9731276	2	38	C:0.894737	T:0.105263		
chr28	9731289	2	38	T:0.894737	G:0.105263		
chr28	9731315	2	38	CAAT:0.868421	C:0.131579		
chr28	9731461	2	36	C:0.833333	T:0.166667		
chr28	9731643	2	38	A:0.842105	G:0.157895		
chr28	9731646	2	38	G:0.657895	A:0.342105		
chr28	9732250	2	36	C:0.916667	G:0.0833333		
chr28	9732289	2	36	TCC:0.861111	T:0.138889		
chr28	9732713	2	34	A:0.823529	C:0.176471		
chr28	9732824	2	40	C:1	CTATATATATAACTATA:0		
chr28	9733050	2	38	C:1	T:0		
chr28	9733070	2	36	A:0.861111	G:0.138889		
chr28	9733084	2	36	C:1	T:0		
chr28	9733147	2	38	T:1	A:0		
chr28	9733150	2	38	C:1	T:0		
chr28	9733564	2	36	G:0.833333	A:0.166667		
chr28	9733818	2	36	CAGAA:0.861111	C:0.138889		
chr28	9733823	2	36	A:0.861111	C:0.138889		
chr28	9733861	2	38	A:1	G:0		
chr28	9734028	2	32	T:0.90625	C:0.09375		
chr28	9734239	2	38	T:0.447368	C:0.552632		
chr28	9734628	3	36	G:0.0555556	GTC:0.25	GTCTC:0.694444	
chr28	9734706	2	40	A:0.85	T:0.15		
chr28	9734721	3	36	CAA:0.722222	C:0.166667	CAAA:0.111111	
chr28	9734868	2	36	G:0.861111	C:0.138889		
chr28	9736579	2	34	A:0.588235	G:0.411765		
chr28	9736608	2	36	A:1	T:0		
chr28	9736672	2	34	T:0.558824	TAAG:0.441176		
chr28	9736841	2	36	A:0.472222	G:0.527778		
chr28	9737006	4	36	A:0.194444	ATG:0.444444	ATGTGTGTGTGTGTGTG:0.361111	ATGTGTGTGTGTGTGTGTG:0
chr28	9737028	2	30	A:0.566667	ATT:0.433333		
chr28	9737409	2	38	T:0.368421	C:0.631579		

chr28	9737548	3	40	GT:0.125	G:0.6	GTT:0.275	
chr28	9737645	2	38	A:0.421053	C:0.578947		
chr28	9737667	2	36	C:0.611111	T:0.388889		
chr28	9737805	2	36	A:0.416667	G:0.583333		
chr28	9737930	2	38	T:0.894737	C:0.105263		
chr28	9738289	2	34	CA:0.676471	C:0.323529		
chr28	9738986	2	34	T:0.705882	C:0.294118		
chr28	9739027	2	32	G:0.90625	A:0.09375		
chr28	9739349	2	38	G:0.894737	A:0.105263		
chr28	9739370	2	38	T:0.894737	C:0.105263		
chr28	9739420	2	40	C:0.85	T:0.15		
chr28	9739441	2	40	T:0.85	C:0.15		
chr28	9739464	2	40	T:0.85	C:0.15		
chr28	9739475	2	40	G:0.85	A:0.15		
chr28	9739585	2	38	C:0.842105	T:0.157895		
chr28	9739593	2	38	G:0.657895	A:0.342105		
chr28	9739661	2	38	C:0.578947	T:0.421053		
chr28	9739683	2	38	G:0.973684	T:0.0263158		
chr28	9739715	3	32	A:0.71875	C:0	T:0.28125	
chr28	9739815	2	40	G:0.925	A:0.075		
chr28	9739860	2	38	C:0.894737	T:0.105263		
chr28	9739865	2	38	C:0.894737	G:0.105263		
chr28	9739866	2	38	A:1	G:0		
chr28	9739947	2	36	G:0.972222	C:0.0277778		
chr28	9740055	2	36	G:0.361111	A:0.638889		
chr28	9740120	2	38	C:0.894737	T:0.105263		
chr28	9740234	2	36	G:0.444444	A:0.555556		
chr28	9740295	2	36	C:0.666667	T:0.333333		
chr28	9740534	2	40	G:0.6	A:0.4		
chr28	9740867	2	32	G:0.40625	A:0.59375		
chr28	9740886	2	36	C:0.861111	T:0.138889		
chr28	9740999	2	40	CT:0.7	C:0.3		
chr28	9741008	4	38	GT:0.315789	G:0.578947	GTT:0	GTTT:0.105263
chr28	9741185	2	40	TCTC:0.825	T:0.175		
chr28	9741427	2	40	TTC:0.875	T:0.125		
chr28	9741441	2	40	T:0.875	C:0.125		
chr28	9741450	2	40	T:0.875	C:0.125		
chr28	9741626	2	34	A:0.911765	C:0.0882353		
chr28	9741663	2	34	G:0.911765	A:0.0882353		
chr28	9741924	2	38	G:0.921053	A:0.0789474		
chr28	9741942	2	38	AATTG:0.921053	A:0.0789474		
chr28	9742027	2	30	T:0	C:1		
chr28	9742045	2	34	C:0.911765	T:0.0882353		
chr28	9742095	2	36	A:0.972222	AT:0.0277778		
chr28	9742175	2	40	G:0	A:1		
chr28	9742286	2	36	C:0.916667	T:0.0833333		
chr28	9742324	2	36	C:0.888889	T:0.111111		
chr28	9742332	2	38	A:0.710526	G:0.289474		
chr28	9742394	2	36	G:0.888889	A:0.111111		
chr28	9742571	2	38	G:1	A:0		
chr28	9742760	2	38	T:0.578947	G:0.421053		
chr28	9742857	2	38	G:0.842105	C:0.157895		

chr28	9743064	2	40	T:0.875	A:0.125		
chr28	9743116	2	40	T:0.9	TA:0.1		
chr28	9743160	2	36	C:0.916667	A:0.0833333		
chr28	9743176	2	36	A:0.916667	C:0.0833333		
chr28	9743185	2	36	G:0.583333	A:0.416667		
chr28	9743202	2	40	A:0.875	G:0.125		
chr28	9743328	2	38	T:0.868421	C:0.131579		
chr28	9743590	2	40	G:0.925	GGGAT:0.075		
chr28	9743591	4	40	GGATAGATA:0.45	G:0.15	GGATA:0.4	GGATAGATAGATA:0
chr28	9743595	2	40	A:1	G:0		
chr28	9743634	3	40	T:0.9	TAGATAG:0.025	TGATAGATAGATAG:0.075	
chr28	9743837	2	34	G:0.647059	C:0.352941		
chr28	9743838	2	34	T:0.588235	C:0.411765		
chr28	9743960	2	36	A:0.194444	T:0.805556		
chr28	9744247	2	40	C:0.9	A:0.1		
chr28	9744622	2	38	A:0.947368	G:0.0526316		
chr28	9744849	2	40	G:0.8	A:0.2		
chr28	9745014	2	36	G:0.888889	T:0.111111		
chr28	9745028	2	38	T:0.657895	TA:0.342105		
chr28	9745205	2	34	C:1	T:0		
chr28	9745251	2	38	T:0.842105	C:0.157895		
chr28	9745261	2	38	G:0.842105	A:0.157895		
chr28	9745314	2	38	G:0.736842	A:0.263158		
chr28	9746163	2	38	T:0.842105	G:0.157895		
chr28	9746181	2	38	C:0.868421	T:0.131579		
chr28	9746220	2	38	C:0.842105	T:0.157895		
chr28	9746611	2	36	TC:0.638889	T:0.361111		
chr28	9746612	2	36	C:0.888889	T:0.111111		
chr28	9746758	2	36	T:0.666667	C:0.333333		
chr28	9747095	2	38	T:0.578947	TAC:0.421053		
chr28	9747240	2	40	TC:0.95	T:0.05		
chr28	9747525	2	38	A:0.842105	T:0.157895		
chr28	9747633	2	34	C:0.735294	T:0.264706		
chr28	9747866	2	36	C:0.527778	T:0.472222		
chr28	9747984	2	38	A:0	G:1		
chr28	9748265	2	38	G:0.947368	A:0.0526316		
chr28	9748530	2	38	G:0.894737	A:0.105263		
chr28	9749203	2	38	A:0.842105	G:0.157895		
chr28	9749315	2	30	C:0.766667	CT:0.233333		
chr28	9749494	2	38	A:0.394737	G:0.605263		
chr28	9749795	2	38	A:0.368421	G:0.631579		
chr28	9749831	2	40	GC:0.575	G:0.425		
chr28	9750270	2	36	G:0.666667	C:0.333333		
chr28	9750347	2	38	A:0	C:1		
chr28	9750948	2	34	A:0.0294118	G:0.970588		
chr28	9751061	2	32	C:0.875	G:0.125		
chr28	9751265	2	32	C:0.59375	T:0.40625		
chr28	9751372	2	36	C:0.75	T:0.25		
chr28	9751465	4	32	AT:0.5625	A:0.15625	ATT:0.28125	ATTTT:0
chr28	9752140	3	38	C:0.368421	CTG:0.631579	CTGTG:0	

chr28 9752675 2 38 G:0.842105 A:0.157895
chr28 9752676 2 38 A:0.842105 G:0.157895
chr28 9753535 2 32 C:0.875 A:0.125
chr28 9753816 2 36 G:0.555556 A:0.444444
chr28 9754192 2 34 T:0.441176 C:0.558824
chr28 9754329 2 40 T:0.45 C:0.55
chr28 9754954 2 40 T:0.9 TTTTTTA:0.1
chr28 9754961 2 40 A:0.9 ATTTATTTTTTTTTAAATTT:0.1
chr28 9755512 2 34 G:1 A:0
chr28 9755568 2 40 C:0.55 A:0.45
chr28 9755764 2 30 G:0.4 T:0.6
chr28 9755816 2 38 A:0.5 G:0.5
chr28 9755909 2 38 CAAATAATAATAATAATAA:0.894737
C:0.105263
chr28 9755910 6 38 AAATAATAATAATAAT:0.473684 A:
0.0263158 AAAT:0 AAATAAT:0.0526316 AAATAATAAT:
0.157895 AAATAATAATAAT:0.289474
chr28 9756064 2 38 A:0.5 G:0.5
chr28 9756106 2 36 C:0.527778 G:0.472222
chr28 9756139 2 38 T:0.921053 TA:0.0789474
chr28 9756756 2 32 A:0.84375 G:0.15625
chr28 9756881 2 38 A:0.605263 G:0.394737
chr28 9757016 2 38 C:0.394737 G:0.605263
chr28 9757207 2 38 T:0.842105 A:0.157895
chr28 9757230 2 40 C:1 T:0
chr28 9757270 2 40 TCCTTCTCTCC:0.85 T:0.15
chr28 9757282 2 40 T:0.85 G:0.15
chr28 9757346 2 40 TTCTCTCCATAAGTAATGATC:0.85
T:0.15
chr28 9757454 2 36 C:0.527778 T:0.472222
chr28 9757710 2 38 A:0.842105 G:0.157895
chr28 9758156 2 36 C:0.694444 CTTTTTTTT:
0.305556
chr28 9758433 2 38 TA:0.394737 T:0.605263
chr28 9758746 2 36 T:0.805556 A:0.194444
chr28 9758811 2 34 C:1 T:0
chr28 9759090 2 40 GA:0.8 G:0.2
chr28 9759127 2 38 G:0.631579 GAA:0.368421
chr28 9759435 2 36 G:0.75 C:0.25
chr28 9759494 2 30 T:0.1 TAA:0.9
chr28 9759580 2 40 C:0.6 A:0.4
chr28 9759649 2 36 T:0.694444 C:0.305556
chr28 9759874 2 32 T:0.78125 C:0.21875
chr28 9759938 2 36 A:0.305556 G:0.694444
chr28 9759994 2 36 G:0.777778 A:0.222222
chr28 9760373 2 38 T:0.710526 C:0.289474
chr28 9760542 2 34 G:0.647059 A:0.352941
chr28 9760660 2 38 C:0.789474 T:0.210526
chr28 9761086 2 38 GA:0.815789 G:0.184211
chr28 9761098 2 38 A:0.657895 C:0.342105
chr28 9761245 2 36 C:0.583333 CCT:0.416667
chr28 9761552 2 40 T:0.7 G:0.3
chr28 9761728 2 38 G:0.842105 GT:0.157895
chr28 9762479 2 38 TG:0.763158 T:0.236842

chr28	9762767	2	38	AAG:0.947368	A:0.0526316
chr28	9763333	2	38	A:0.921053	T:0.0789474
chr28	9763478	2	36	C:0.25	T:0.75
chr28	9763486	2	38	T:0	C:1
chr28	9764176	2	40	C:0.325	G:0.675
chr28	9764669	2	36	G:0.361111	C:0.638889
chr28	9764759	2	38	G:0.657895
GAAATACAGATGTCAAAAAAAAAAAAA:0.342105
chr28	9765181	2	40	C:1	T:0
chr28	9765523	2	36	T:0	G:1
chr28	9765634	2	40	C:0.6	G:0.4
chr28	9765903	2	36	C:0.805556	T:0.194444
chr28	9766373	2	24	T:0.541667	C:0.458333
chr28	9766593	2	34	GAGAAAGAAAAGAAGAAAGAAAGAAGA:
0.382353	G:0.617647
chr28	9766676	2	34	AAG:0.411765	A:0.588235
chr28	9766683	2	34	G:0.411765	GA:0.588235
chr28	9766993	2	36	G:0.388889	A:0.611111
chr28	9767153	2	38	C:0.842105	T:0.157895
chr28	9767192	2	36	T:1	C:0
chr28	9767386	2	38	T:0.657895	TTAGA:0.342105
chr28	9767447	2	40	T:0.35	C:0.65
chr28	9767480	2	40	G:0.625	C:0.375
chr28	9767673	2	40	G:0.675	A:0.325
chr28	9768353	2	40	A:0.35	G:0.65
chr28	9768697	2	38	C:0.789474	T:0.210526
chr28	9769049	2	40	A:0.85	AT:0.15
chr28	9769050	2	40	T:0.875	TA:0.125
chr28	9770759	2	36	G:0.833333	C:0.166667
chr28	9771167	2	38	C:0.710526	T:0.289474
chr28	9771342	2	34	C:0.411765	T:0.588235
chr28	9771538	2	38	T:0.815789	TA:0.184211
chr28	9771702	2	38	C:0.921053	T:0.0789474
chr28	9771730	2	38	A:0.421053	G:0.578947
chr28	9772152	2	36	G:0.722222	T:0.277778
chr28	9772348	2	38	A:0.763158	G:0.236842
chr28	9772580	2	36	A:0.555556	G:0.444444
chr28	9772908	2	38	T:0.710526	C:0.289474
chr28	9772982	2	40	ATG:0.725	A:0.275
chr28	9772985	2	40	CCAGTGTTAA:0.725	C:0.275
chr28	9773464	2	36	C:0.916667	T:0.0833333
chr28	9773787	2	34	A:0.764706	G:0.235294
chr28	9773853	2	36	A:0.583333	G:0.416667
chr28	9773867	2	36	C:0.75	T:0.25
chr28	9774139	2	36	G:0.777778	A:0.222222
chr28	9774505	2	36	A:0.888889	G:0.111111
chr28	9774726	2	38	C:0.394737	T:0.605263
chr28	9774815	2	36	C:0.638889	T:0.361111
chr28	9775141	2	38	G:0.631579	A:0.368421
chr28	9775184	2	38	T:0.894737	TC:0.105263
chr28	9775707	2	36	C:0.777778	T:0.222222
chr28	9775881	2	38	G:0.710526	GAA:0.289474
chr28	9776378	2	32	T:0.375	C:0.625
chr28	9776533	2	36	C:0.555556	T:0.444444

chr28	9776550	2	34	T:0.705882	TC:0.294118
chr28	9777051	2	38	G:0.631579	A:0.368421
chr28	9777185	2	40	G:0.725	C:0.275
chr28	9777308	2	38	T:0.789474	C:0.210526
chr28	9777424	2	34	T:0.764706	C:0.235294
chr28	9777623	2	34	T:0.676471	C:0.323529
chr28	9777957	2	38	C:0.763158	A:0.236842
chr28	9777990	2	38	T:0.578947	A:0.421053
chr28	9778333	2	40	C:1	T:0
chr28	9778511	2	38	C:0.789474	T:0.210526
chr28	9778650	2	38	CT:0.736842	C:0.263158
chr28	9778713	2	36	T:0.333333	G:0.666667
chr28	9779028	2	36	A:0.333333	G:0.666667
chr28	9779184	2	34	A:0	G:1
chr28	9779188	2	34	A:0.823529	G:0.176471
chr28	9779472	2	36	C:1	T:0
chr28	9779502	2	36	G:0.25	A:0.75
chr28	9779585	2	36	G:0.861111	A:0.138889
chr28	9779744	2	36	G:0.722222	A:0.277778
chr28	9779765	2	36	C:0.611111	T:0.388889
chr28	9779849	2	38	G:0	C:1
chr28	9779986	2	38	C:0.842105	G:0.157895
chr28	9780107	2	38	T:0.842105	C:0.157895
chr28	9780582	2	22	GAAAA:0	G:1
chr28	9780586	2	22	A:0	AGGCGC:1
chr28	9781109	2	28	CT:0.428571	C:0.571429
chr28	9781370	2	32	G:0.59375	A:0.40625
chr28	9781580	2	36	T:0.472222	C:0.527778
chr28	9781610	2	36	A:0.5	G:0.5
chr28	9781893	2	36	C:0.583333	T:0.416667
chr28	9782443	2	34	T:0.647059	G:0.352941
chr28	9782499	2	36	G:0.916667	A:0.0833333
chr28	9782808	2	22	A:0.954545	T:0.0454545
chr28	9782809	2	22	C:0.954545	A:0.0454545
chr28	9782819	2	20	G:0.65	T:0.35
chr28	9782979	2	30	C:1	T:0
chr28	9783183	2	32	C:0	G:1
chr28	9783199	2	36	T:0.944444	C:0.0555556
chr28	9783448	2	32	T:0.59375	C:0.40625
chr28	9783582	2	36	C:0.861111	A:0.138889
chr28	9783784	2	38	A:0.842105	G:0.157895
chr28	9783843	2	40	G:0.475	T:0.525
chr28	9783881	2	40	T:0.475	G:0.525
chr28	9784009	2	40	G:0.45	C:0.55
chr28	9784056	2	40	G:0.65	A:0.35
chr28	9784260	2	36	C:0.638889	T:0.361111
chr28	9784474	2	36	T:0.916667	C:0.0833333
chr28	9784506	2	34	C:0.588235	T:0.411765
chr28	9784586	2	36	C:1	G:0
chr28	9784588	2	36	G:0.694444	A:0.305556
chr28	9784622	2	36	T:0.888889	C:0.111111
chr28	9784780	2	40	T:0.925	G:0.075
chr28	9785095	2	34	C:0.911765	A:0.0882353
chr28	9785624	2	38	C:0.894737	T:0.105263

chr28	9785777	2	40	T:0.875	C:0.125
chr28	9785850	2	40	G:0.9	A:0.1
chr28	9785965	2	36	T:0.916667	G:0.0833333
chr28	9786023	2	38	G:1	A:0
chr28	9786138	2	32	A:0.875	G:0.125
chr28	9786603	2	36	G:0.916667	T:0.0833333
chr28	9786794	2	36	G:0.972222	A:0.0277778
chr28	9786881	2	34	C:0.882353	T:0.117647
chr28	9786990	2	38	C:0.710526	T:0.289474
chr28	9787175	2	32	A:0.875	G:0.125
chr28	9787339	2	40	C:0.875	T:0.125
chr28	9787422	2	36	T:0.861111	C:0.138889
chr28	9787520	2	36	T:0.888889	G:0.111111
chr28	9787968	2	40	A:0.9	G:0.1
chr28	9788045	2	36	G:0.833333	A:0.166667
chr28	9788135	2	36	T:0.888889	A:0.111111
chr28	9788454	2	40	G:0.825	A:0.175
chr28	9788807	2	34	G:0.852941	A:0.147059
chr28	9788899	2	38	T:0.894737	A:0.105263
chr28	9789035	2	34	CT:0.941176	C:0.0588235
chr28	9789068	2	38	C:0.894737	CAAAT:0.105263
chr28	9789180	2	32	C:0.875	T:0.125
chr28	9789612	2	34	T:0.882353	C:0.117647
chr28	9789645	2	36	G:0	A:1
chr28	9789859	2	34	C:0.882353	A:0.117647
chr28	9790016	2	34	T:0.911765	TAGCC:0.0882353
chr28	9790026	2	34	TA:0.941176	T:0.0588235
chr28	9790074	2	32	C:0.90625	T:0.09375
chr28	9790405	2	36	C:1	T:0
chr28	9790419	2	36	G:0.916667	A:0.0833333
chr28	9790455	2	34	T:0.382353	C:0.617647
chr28	9790526	2	36	A:0.833333	G:0.166667
chr28	9790662	2	38	C:0.868421	T:0.131579
chr28	9791240	2	38	A:0.921053	AAG:0.0789474
chr28	9791601	2	40	G:0.9	A:0.1
chr28	9791611	2	40	C:0.9	T:0.1
chr28	9792278	2	32	T:0.59375	TA:0.40625
chr28	9792359	2	38	G:0.842105	T:0.157895
chr28	9792736	2	38	C:1	T:0
chr28	9792838	2	38	G:0.921053	C:0.0789474
chr28	9792888	2	38	AC:0.894737	A:0.105263
chr28	9793291	2	40	G:1	A:0
chr28	9793351	2	38	C:1	T:0
chr28	9793488	2	36	G:1	A:0
chr28	9793596	2	38	C:0.894737	T:0.105263
chr28	9793667	2	40	G:0.95	A:0.05
chr28	9793740	2	38	G:0.894737	T:0.105263
chr28	9793897	2	38	G:0.894737	A:0.105263
chr28	9793969	2	36	A:0.916667	G:0.0833333
chr28	9793999	2	38	G:0.631579	GA:0.368421
chr28	9794014	2	40	AT:0.85	A:0.15
chr28	9794053	2	32	A:0.84375	G:0.15625
chr28	9794505	2	38	A:1	G:0
chr28	9794625	2	38	A:1	G:0

```
chr28	9795001	2	34	C:1	T:0
chr28	9795058	2	36	C:0.861111	G:0.138889
chr28	9795444	2	38	A:0	G:1
chr28	9795515	2	34	T:0	C:1
chr28	9795678	2	36	A:0.861111	G:0.138889
chr28	9795826	2	40	CT:1	C:0
chr28	9795946	2	36	A:0	G:1
chr28	9795986	2	36	T:0.861111	C:0.138889
chr28	9796648	2	34	C:0	T:1
chr28	9796896	2	36	T:0.944444	C:0.0555556
chr28	9797057	2	40	C:0.875	A:0.125
chr28	9797235	2	36	T:1	C:0
chr28	9797265	2	40	T:0.9	C:0.1
chr28	9797266	2	40	G:0.9	A:0.1
chr28	9797282	2	40	C:1	T:0
chr28	9797798	2	40	G:1	A:0
chr28	9798027	2	36	C:0.5	T:0.5
chr28	9798108	2	34	G:1	A:0
chr28	9798234	2	38	T:1	C:0
chr28	9800437	2	40	A:0	G:1
chr28	9800547	5	38	C:0.157895	CA:0.0789474
CAA:0.0263158	CAAAAA:0.552632	CAAAAAAA:0.184211
chr28	9801138	2	38	C:1	T:0
chr28	9801139	2	38	A:1	G:0
chr28	9801402	2	32	G:1	T:0
chr28	9801683	2	34	C:1	T:0
chr28	9802136	2	34	C:1	G:0
chr28	9802306	2	32	C:1	T:0
chr28	9802363	4	34	C:0.0588235	CA:0	CAAAA:
0.529412	CAAAAA:0.411765
chr28	9802788	2	36	G:0.0277778	A:0.972222
chr28	9802920	3	32	GAA:0.09375	G:0.1875	GA:
0.71875
chr28	9803120	2	36	C:0.833333	T:0.166667
chr28	9803834	2	36	G:0.833333	A:0.166667
chr28	9803869	2	36	G:0.888889	A:0.111111
chr28	9804295	2	40	G:0.9	A:0.1
chr28	9804465	2	40	T:0.9	C:0.1
chr28	9804556	2	40	T:0.925	TA:0.075
chr28	9804561	2	40	T:0.925	C:0.075
chr28	9804876	2	38	C:0.894737	T:0.105263
chr28	9805551	2	38	T:0.789474	C:0.210526
chr28	9805552	2	38	G:0.789474	C:0.210526
chr28	9805581	2	40	A:0	ACT:1
chr28	9806859	2	34	A:1	G:0
chr28	9807002	4	38	AAC:0.210526	A:0.289474
AACAC:0.0789474	AACACACACACAC:0.421053
chr28	9807618	2	36	A:0.638889	G:0.361111
chr28	9808169	2	38	A:0.815789	C:0.184211
chr28	9808283	2	38	A:0.105263	G:0.894737
chr28	9808530	2	36	G:0.666667	A:0.333333
chr28	9809053	2	36	G:0.611111	A:0.388889
chr28	9809286	2	36	G:0.388889	T:0.611111
chr28	9809566	2	38	C:0.631579	T:0.368421
```

chr28 9810156 2 38 C:0.631579 G:0.368421
chr28 9810157 2 38 A:0 G:1
chr28 9810435 2 34 G:0.647059 A:0.352941
chr28 9810671 2 34 GAGA:0.352941 G:0.647059
chr28 9810895 2 36 A:0.666667 G:0.333333
chr28 9811110 2 38 G:0.578947 A:0.421053
chr28 9811232 2 34 G:0.558824 C:0.441176
chr28 9811910 2 40 C:0.625 T:0.375
chr28 9812192 2 38 C:0.5 T:0.5
chr28 9812358 2 36 G:0.555556 A:0.444444
chr28 9812908 2 38 T:0.631579 A:0.368421
chr28 9812918 3 38 AGTGTGTGTGTGTGT:0.0263158 A:
0.421053 AGTGTGTGTGT:0.552632
chr28 9813361 2 30 T:0 G:1
chr28 9813377 2 32 G:0.53125 A:0.46875
chr28 9813701 2 36 C:0.527778 A:0.472222
chr28 9814165 2 38 C:0.447368 T:0.552632
chr28 9814419 3 40 GAA:0.45 G:0.225 GAAAA:0.325
chr28 9814618 2 38 G:0.631579 T:0.368421
chr28 9814674 2 38 A:0.0526316 G:0.947368
chr28 9814903 2 38 C:0.552632 T:0.447368
chr28 9814907 2 38 T:1 A:0
chr28 9815148 2 38 A:0.0263158 G:0.973684
chr28 9815254 2 36 T:0.694444 TTAATAA:0.305556
chr28 9815653 2 32 G:0.03125 A:0.96875
chr28 9815684 2 38 G:1 A:0
chr28 9815850 2 38 G:0.578947 A:0.421053
chr28 9816208 2 40 T:0.025 C:0.975
chr28 9816217 2 40 C:0.025 A:0.975
chr28 9817044 2 36 A:0.555556 G:0.444444
chr28 9817417 2 40 G:0.15 A:0.85
chr28 9817556 2 34 T:0.588235 C:0.411765
chr28 9818005 3 38 A:0.105263 G:0.5 T:
0.394737
chr28 9818139 2 38 G:0.131579 A:0.868421
chr28 9818302 2 38 C:1 T:0
chr28 9818360 2 38 GGTGA:0.894737 G:0.105263
chr28 9818551 2 38 T:0.210526 C:0.789474
chr28 9818586 2 32 G:0.1875 A:0.8125
chr28 9818719 2 40 C:0.15 T:0.85
chr28 9818756 2 40 C:0.175 T:0.825
chr28 9819007 2 38 T:0.184211 C:0.815789
chr28 9820122 2 38 T:1 A:0
chr28 9820376 2 38 G:0.0263158 C:0.973684
chr28 9821600 2 34 T:1 C:0
chr28 9821617 2 32 A:0.90625 AAAAC:0.09375
chr28 9821736 2 36 G:0.888889 A:0.111111
chr28 9822122 2 38 GAA:1 G:0
chr28 9822210 2 38 C:0.0263158 T:0.973684
chr28 9822230 2 36 A:0.0277778 C:0.972222
chr28 9822659 2 38 G:0.0263158 A:0.973684
chr28 9822684 2 38 C:0.157895 T:0.842105
chr28 9822815 2 38 T:0.0263158 C:0.973684
chr28 9822998 2 38 C:0.973684 CA:0.0263158

chr28	9823379	2	36	G:0.833333	T:0.166667		
chr28	9823507	2	34	T:0.882353	C:0.117647		
chr28	9823706	2	38	G:0.578947	A:0.421053		
chr28	9824113	2	34	A:0.411765	G:0.588235		
chr28	9824436	2	38	T:0.921053	G:0.0789474		
chr28	9824554	2	36	C:0.555556	T:0.444444		
chr28	9824573	2	36	C:0.611111	A:0.388889		
chr28	9824820	2	22	A:0.363636	T:0.636364		
chr28	9824902	2	38	G:0.0263158	A:0.973684		
chr28	9825043	2	36	A:1	G:0		
chr28	9825151	2	40	CTTTTTTTTTT:0.65	C:0.35		
chr28	9825239	2	38	T:0.921053	C:0.0789474		
chr28	9825274	2	40	T:0.5	C:0.5		
chr28	9825381	2	32	T:0.5	C:0.5		
chr28	9825714	2	38	C:1	T:0		
chr28	9825812	2	40	T:0.425	C:0.575		
chr28	9826285	2	34	G:1	A:0		
chr28	9826483	2	36	GAAGT:0.611111	G:0.388889		
chr28	9826672	2	38	C:0.473684	T:0.526316		
chr28	9827433	2	36	TTTTTC:0.861111	T:0.138889		
chr28	9827734	2	38	A:0.473684	C:0.526316		
chr28	9827865	2	40	C:0.5	T:0.5		
chr28	9828526	2	38	C:0.473684	A:0.526316		
chr28	9828545	2	36	TACACACACAA:1	T:0		
chr28	9828555	4	40	AACACACACACACAC:0.325	A:0.125	AACAC:0.55	CACACACACACACAC:0
chr28	9828679	2	36	C:0.5	A:0.5		
chr28	9828690	2	40	TACAA:0.475	T:0.525		
chr28	9828927	2	38	C:0.5	T:0.5		
chr28	9829025	2	38	C:0.789474	T:0.210526		
chr28	9829252	2	38	T:0.421053	C:0.578947		
chr28	9829295	2	38	T:0.473684	A:0.526316		
chr28	9829352	2	38	T:0.526316	G:0.473684		
chr28	9829354	2	38	G:0.526316	A:0.473684		
chr28	9829438	2	36	G:0.583333	A:0.416667		
chr28	9830182	2	38	G:0.447368	T:0.552632		
chr28	9830309	2	38	T:0.5	TA:0.5		
chr28	9830679	2	40	T:0.025	TA:0.975		
chr28	9830811	2	38	C:1	A:0		
chr28	9831040	2	38	G:0.421053	A:0.578947		
chr28	9831068	2	36	C:0.388889	T:0.611111		
chr28	9831687	2	40	T:0.475	C:0.525		
chr28	9831751	2	40	T:0.425	C:0.575		
chr28	9832026	2	36	A:1	G:0		
chr28	9832433	2	38	T:0.5	C:0.5		
chr28	9832538	2	38	A:0.368421	G:0.631579		
chr28	9832873	2	36	CT:0.472222	C:0.527778		
chr28	9833889	2	34	CTT:1	C:0		
chr28	9833997	2	38	T:0.552632	C:0.447368		
chr28	9834032	2	38	G:0.5	C:0.5		
chr28	9834344	2	38	G:0.421053	A:0.578947		
chr28	9834526	2	40	G:0.825	A:0.175		
chr28	9834887	2	36	T:0.416667	G:0.583333		
chr28	9835154	2	38	T:0.921053	C:0.0789474		

chr28	9835283	2	38	T:0.894737	G:0.105263	
chr28	9835327	2	38	T:0.947368	C:0.0526316	
chr28	9836035	2	32	C:0.90625	G:0.09375	
chr28	9836232	2	36	T:0.833333	TGAGAGA:0.166667	
chr28	9836370	2	32	C:0.90625	T:0.09375	
chr28	9836413	2	34	ATGTG:0.5	A:0.5	
chr28	9836440	2	36	TGTGTGC:0.916667	T:0.0833333	
chr28	9836868	2	38	C:0.894737	T:0.105263	
chr28	9836958	2	38	C:1	T:0	
chr28	9837299	2	38	C:0.631579	T:0.368421	
chr28	9837424	2	38	C:0.868421	G:0.131579	
chr28	9837820	2	36	C:0.888889	T:0.111111	
chr28	9838398	2	40	A:0.975	G:0.025	
chr28	9838524	2	38	G:0.947368	A:0.0526316	
chr28	9838729	2	36	G:0.888889	A:0.111111	
chr28	9839753	3	36	G:0.583333	GAT:0.305556	GATAT:0.111111
chr28	9839853	2	28	C:0.142857	CCT:0.857143	
chr28	9840164	2	36	A:0.527778	G:0.472222	
chr28	9840384	2	36	T:0.805556	G:0.194444	
chr28	9840452	2	36	A:0.833333	T:0.166667	
chr28	9840554	2	40	T:0.85	C:0.15	
chr28	9841083	2	38	C:0.631579	G:0.368421	
chr28	9841148	2	38	C:1	T:0	
chr28	9841160	2	38	G:0.894737	A:0.105263	
chr28	9842075	2	36	G:0.833333	A:0.166667	
chr28	9842205	3	38	T:0.868421	TAA:0.0789474	TAAA:0.0526316
chr28	9842215	3	38	TA:0.5	T:0.368421	AA:0.131579
chr28	9842217	2	36	A:0.916667	T:0.0833333	
chr28	9842220	2	36	A:0.583333	T:0.416667	
chr28	9842238	3	38	A:0.0526316	AT:0.815789	AAT:0.131579
chr28	9842322	2	38	C:0.868421	T:0.131579	
chr28	9842442	2	36	G:0.861111	C:0.138889	
chr28	9842543	2	38	A:0.842105	T:0.157895	
chr28	9842745	2	38	C:0.842105	T:0.157895	
chr28	9843409	2	32	T:0.84375	C:0.15625	
chr28	9843430	2	32	T:0.84375	C:0.15625	
chr28	9843567	2	38	T:0.815789	TAA:0.184211	
chr28	9843654	2	34	G:0.558824	C:0.441176	
chr28	9843795	2	0	C:-nan	T:-nan	
chr28	9843984	2	36	T:0.916667	C:0.0833333	
chr28	9843998	2	38	G:0.447368	A:0.552632	
chr28	9844064	2	38	G:1	GAT:0	
chr28	9844773	2	34	C:1	T:0	
chr28	9844863	3	36	CA:0.638889	C:0.0833333	CAA:0.277778
chr28	9844902	2	38	C:0.921053	T:0.0789474	
chr28	9845152	2	38	G:0.894737	A:0.105263	
chr28	9845180	2	38	A:0.894737	T:0.105263	
chr28	9845491	2	32	A:0.90625	G:0.09375	
chr28	9845837	2	38	TA:0.815789	T:0.184211	

chr28	9845981	2	30	A:0.9	G:0.1
chr28	9846137	2	34	A:0.882353	G:0.117647
chr28	9846192	3	38	CAGAG:0.578947	C:0.105263
CAGAGAG:0.315789
chr28	9846229	2	38	G:0.894737	C:0.105263
chr28	9846345	2	34	TC:0.852941	T:0.147059
chr28	9846363	3	38	T:0.210526	TA:0.421053
TAA:0.368421
chr28	9846436	2	40	G:1	A:0
chr28	9846461	2	38	C:0.894737	T:0.105263
chr28	9846494	2	34	T:0.911765	C:0.0882353
chr28	9846687	2	32	C:0.875	CCT:0.125
chr28	9846729	2	32	AT:0.59375	A:0.40625
chr28	9846731	2	32	TTA:0.9375	T:0.0625
chr28	9846732	2	32	TA:0.46875	T:0.53125
chr28	9847396	2	36	C:0.861111	T:0.138889
chr28	9847506	2	36	T:0.0277778	C:0.972222
chr28	9847533	2	34	A:0.705882	C:0.294118
chr28	9848139	2	34	T:0.882353	C:0.117647
chr28	9848246	2	34	G:1	A:0
chr28	9848298	2	36	A:0.0277778	G:0.972222
chr28	9849306	2	34	C:0.0882353	T:0.911765
chr28	9849486	2	36	A:0.888889	AC:0.111111
chr28	9849721	2	38	A:0.157895	C:0.842105
chr28	9849820	2	38	CACA:0.210526	C:0.789474
chr28	9849928	2	34	A:0.147059	T:0.852941
chr28	9850403	2	40
CTAGTAGCACCTAGTAGGTGGGAGAGTGAAGCGGGGGCGAAG:0.025	C:0.975
chr28	9850627	2	38	A:0.947368	G:0.0526316
chr28	9850811	2	38	AT:0.578947	A:0.421053
chr28	9850888	2	36	C:0.138889	CT:0.861111
chr28	9850891	2	36	A:0.138889	T:0.861111
chr28	9851276	2	40	A:0.025	C:0.975
chr28	9851497	2	38	A:0.868421	G:0.131579
chr28	9851627	2	38	G:0.578947	A:0.421053
chr28	9851629	2	36	TC:1	T:0
chr28	9851788	2	36	C:0.583333	T:0.416667
chr28	9851888	2	34	A:0.882353	T:0.117647
chr28	9852068	2	32	TGGG:0.09375	T:0.90625
chr28	9852170	2	32	ACCT:0.40625	A:0.59375
chr28	9852576	2	38	A:0.868421	G:0.131579
chr28	9852994	2	38	A:0.868421	T:0.131579
chr28	9853053	2	36	G:0.527778	A:0.472222
chr28	9853304	2	34	C:0.0294118	T:0.970588
chr28	9853494	2	34	A:0.117647	G:0.882353
chr28	9853508	2	34	A:0.0294118	G:0.970588
chr28	9853632	2	38	C:0.184211	CGAGG:0.815789
chr28	9853923	2	34	C:1	A:0
chr28	9854234	2	40	C:0.175	T:0.825
chr28	9854412	2	36	A:0.166667	T:0.833333
chr28	9854497	2	36	AAC:1	A:0
chr28	9854514	2	38	C:0.0526316	G:0.947368
chr28	9854797	2	38	G:0.131579	A:0.868421
chr28	9855172	2	40	A:0.15	G:0.85

chr28	9855239	3	34	C:0.0294118	CT:0.0882353
CTT:0.882353
chr28	9855333	2	38	A:0.131579	C:0.868421
chr28	9855871	2	36	G:0.0833333	A:0.916667
chr28	9856031	2	38	A:0.105263	AG:0.894737
chr28	9856260	2	38	G:0.105263	A:0.894737
chr28	9856378	2	32	G:0.09375	C:0.90625
chr28	9856454	2	38	CA:1	C:0
chr28	9856687	2	34	A:0.0294118	G:0.970588
chr28	9856755	2	36	C:0.111111	T:0.888889
chr28	9856858	2	36	C:0.888889	T:0.111111
chr28	9857134	2	34	T:0.205882	C:0.794118
chr28	9858708	2	36	C:0.638889	T:0.361111
chr28	9859278	2	36	C:0.583333	T:0.416667
chr28	9861136	4	34	CCT:0.529412	C:0.470588
CCTCT:0	CCTCTCT:0
chr28	9861190	3	28	TA:0.5	T:0.0357143	TAA:
0.464286
chr28	9861661	2	40	C:0.575	T:0.425
chr28	9862142	2	40	C:0.625	T:0.375
chr28	9862228	2	26	GGAGAGA:0.692308	G:0.307692
chr28	9862257	2	30	GAGAGA:0.866667	G:0.133333
chr28	9862263	4	36	GGC:0.722222	GCGC:0.25
G:0	GGCGC:0.0277778
chr28	9862625	2	36	T:1	C:0
chr28	9862626	2	36	C:1	T:0
chr28	9862661	2	38	C:1	T:0
chr28	9862727	2	38	C:1	G:0
chr28	9862798	2	34	G:0.558824	A:0.441176
chr28	9863474	2	40	C:1	A:0
chr28	9863582	2	32	A:1	ATG:0
chr28	9863699	2	38	G:0.684211	A:0.315789
chr28	9863896	2	34	C:1	T:0
chr28	9864348	2	38	G:1	A:0
chr28	9864350	2	38	T:1	TA:0
chr28	9864458	2	40	C:1	T:0
chr28	9864476	2	40	A:0.025	G:0.975
chr28	9864707	2	38	T:1	A:0
chr28	9864884	2	36	G:0.611111	A:0.388889
chr28	9865059	2	40	A:1	G:0
chr28	9865602	2	38	C:1	A:0
chr28	9865684	2	38	A:0.157895	G:0.842105
chr28	9865692	2	38	G:0.657895	T:0.342105
chr28	9865757	2	34	C:1	A:0
chr28	9865970	2	34	C:1	T:0
chr28	9866030	2	38	C:1	T:0
chr28	9866648	4	38	TTTTA:0.315789	T:0.0263158
TTTTATTTA:0.552632	TTTTATTTATTTA:0.105263
chr28	9866773	2	36	C:1	T:0
chr28	9867334	2	38	G:1	A:0
chr28	9867590	2	36	G:1	A:0
chr28	9867754	2	38	T:0.105263	C:0.894737
chr28	9867842	2	34	C:0.647059	G:0.352941
chr28	9867917	2	36	C:1	G:0

chr28	9868206	2	38	A:0.973684	T:0.0263158
chr28	9868291	2	38	T:1	G:0
chr28	9868294	2	38	T:0.184211	C:0.815789
chr28	9868531	2	36	A:0.361111	C:0.638889
chr28	9868569	2	36	T:0.555556	C:0.444444
chr28	9868623	2	36	A:0.416667	G:0.583333
chr28	9868641	2	38	G:0.947368	A:0.0526316
chr28	9868719	2	34	G:0.5	T:0.5
chr28	9868812	2	40	C:0.975	T:0.025
chr28	9868859	2	38	A:1	C:0
chr28	9868965	2	38	TG:0.552632	T:0.447368
chr28	9869492	2	32	T:0.5625	TA:0.4375
chr28	9869832	2	38	C:0.868421	T:0.131579
chr28	9869902	2	34	C:0.882353	T:0.117647
chr28	9870157	2	40	ATTAGT:0.975	A:0.025
chr28	9871663	2	38	A:0.394737	G:0.605263
chr28	9872139	2	40	T:0.575	C:0.425
chr28	9872724	2	40	T:0.375	C:0.625
chr28	9872888	2	36	T:0.583333	A:0.416667
chr28	9872930	2	38	G:0.657895	T:0.342105
chr28	9873655	2	40	G:0.925	T:0.075
chr28	9874827	2	40	G:0.375	C:0.625
chr28	9875173	2	36	A:1	G:0
chr28	9875181	2	38	G:0.631579	T:0.368421
chr28	9875347	2	30	G:0.366667	A:0.633333
chr28	9875558	2	36	T:0.416667	C:0.583333
chr28	9875957	2	38	GA:0.421053	G:0.578947
chr28	9876029	2	38	C:0.789474	T:0.210526
chr28	9876312	2	34	A:1	G:0
chr28	9876381	2	40	T:1	TGGAGGGCCAC:0
chr28	9876550	2	36	G:0.472222	T:0.527778
chr28	9877259	2	38	T:0.421053	C:0.578947
chr28	9877592	2	40	T:0.4	G:0.6
chr28	9877635	2	40	G:0.625	GA:0.375
chr28	9877656	2	40	T:0.475	A:0.525
chr28	9877730	2	40	A:0.475	G:0.525
chr28	9878408	2	36	C:0.416667	G:0.583333
chr28	9878831	2	36	G:0.944444	A:0.0555556
chr28	9878967	2	34	G:0.529412	A:0.470588
chr28	9879096	2	40	T:0.875	A:0.125
chr28	9879617	2	36	G:1	T:0
chr28	9880012	2	34	C:0.294118	T:0.705882
chr28	9880112	2	32	C:0.375	T:0.625
chr28	9880193	2	32	G:0.875	A:0.125
chr28	9880387	2	32	C:0.59375	G:0.40625
chr28	9880464	2	36	CCA:0.944444	C:0.0555556
chr28	9880508	2	36	G:0.416667	A:0.583333
chr28	9880510	2	36	A:0.0833333	G:0.916667
chr28	9880537	2	30	A:0.4	G:0.6
chr28	9880570	2	30	G:0.833333	A:0.166667
chr28	9880870	2	36	T:0.0833333	TC:0.916667
chr28	9880874	2	36	T:0.0833333	TCTACCA:0.916667
chr28	9880964	2	36	A:0.277778	G:0.722222
chr28	9881334	2	30	C:0.733333	G:0.266667

chr28	9881668	2	30	C:0.8	T:0.2		
chr28	9881779	2	40	G:0.825	T:0.175		
chr28	9881780	3	38	AT:0.657895	A:0.236842	ATT:0.105263	
chr28	9881785	2	40	T:0.825	A:0.175		
chr28	9882122	2	38	A:0.815789	G:0.184211		
chr28	9882387	2	38	C:0.710526	A:0.289474		
chr28	9882497	2	40	T:0.85	C:0.15		
chr28	9882690	2	40	T:1	A:0		
chr28	9882745	2	38	G:0.710526	A:0.289474		
chr28	9882816	2	40	G:0.8	A:0.2		
chr28	9882890	2	36	T:0	C:1		
chr28	9882931	2	36	G:1	A:0		
chr28	9882979	2	40	G:1	A:0		
chr28	9883080	2	34	G:0.852941	C:0.147059		
chr28	9883168	2	38	CAG:0.289474	C:0.710526		
chr28	9883170	2	36	GA:0.916667	G:0.0833333		
chr28	9883353	2	40	T:0.1	C:0.9		
chr28	9883357	2	40	C:1	T:0		
chr28	9883364	2	36	A:0.0833333	G:0.916667		
chr28	9883521	2	36	C:0.138889	T:0.861111		
chr28	9883583	2	34	C:0.205882	CCG:0.794118		
chr28	9883586	2	34	A:0.205882	C:0.794118		
chr28	9883756	2	32	AT:0.125	A:0.875		
chr28	9883762	2	34	G:0.941176	C:0.0588235		
chr28	9884133	2	38	G:0.894737	A:0.105263		
chr28	9884153	2	36	TA:1	T:0		
chr28	9884223	2	38	G:0.921053	A:0.0789474		
chr28	9884383	2	40	T:0.1	C:0.9		
chr28	9884555	2	38	T:0.789474	G:0.210526		
chr28	9884681	2	36	A:0.777778	G:0.222222		
chr28	9884689	2	36	G:1	A:0		
chr28	9884923	2	38	A:0.789474	G:0.210526		
chr28	9884992	2	36	A:0.194444	T:0.805556		
chr28	9885099	4	40	T:0.05	TA:0.1	TAA:0.15	TAAAA:0.7
chr28	9885950	2	36	ATCT:0.833333	A:0.166667		
chr28	9886081	2	38	T:1	C:0		
chr28	9886147	2	36	T:0.138889	C:0.861111		
chr28	9886881	2	36	A:1	T:0		
chr28	9887013	2	40	A:0.85	G:0.15		
chr28	9887087	2	30	A:1	C:0		
chr28	9887412	2	36	C:1	T:0		
chr28	9887429	2	34	T:0.882353	G:0.117647		
chr28	9887864	2	36	A:1	G:0		
chr28	9888421	2	32	G:1	A:0		
chr28	9888500	2	38	C:0.921053	A:0.0789474		
chr28	9889061	2	38	C:1	A:0		
chr28	9889248	2	36	A:0.833333	G:0.166667		
chr28	9889768	2	38	TA:0.210526	T:0.789474		
chr28	9889985	2	36	T:1	A:0		
chr28	9890004	2	40	G:0.2	T:0.8		
chr28	9890114	2	36	T:0.861111	G:0.138889		
chr28	9890129	2	34	T:0.852941	A:0.147059		

chr28	9890444	2	40	C:0.9	T:0.1
chr28	9890455	2	40	C:0.9	T:0.1
chr28	9890473	2	40	A:0.9	AC:0.1
chr28	9890512	2	36	G:0.861111	A:0.138889
chr28	9890523	2	36	G:0.861111	T:0.138889
chr28	9890737	2	38	A:0.842105	T:0.157895
chr28	9891212	2	32	G:0.84375	A:0.15625
chr28	9891337	2	34	G:1	T:0
chr28	9891404	2	36	C:0.166667	T:0.833333
chr28	9891610	2	40	C:0.875	CT:0.125
chr28	9891719	2	38	C:0.868421	T:0.131579
chr28	9891795	2	40	TG:0.85	T:0.15
chr28	9892170	2	40	C:0.875	T:0.125
chr28	9892247	2	38	A:0.947368	G:0.0526316
chr28	9892299	2	40	T:0.85	C:0.15
chr28	9892543	2	40	A:0.9	C:0.1
chr28	9892591	2	40	T:0.85	G:0.15
chr28	9892766	2	38	A:0.894737	G:0.105263
chr28	9892877	2	38	C:0.263158	A:0.736842
chr28	9892943	2	36	C:0.888889	T:0.111111
chr28	9893728	2	40	T:0.25	G:0.75
chr28	9893742	2	40	G:0.9	C:0.1
chr28	9893744	2	40	A:0.9	G:0.1
chr28	9893812	2	36	A:1	G:0
chr28	9893869	2	36	C:0.666667	G:0.333333
chr28	9894369	2	34	C:0.941176	CTT:0.0588235
chr28	9894394	2	36	C:0.361111	T:0.638889
chr28	9894917	2	32	A:0.84375	G:0.15625
chr28	9894930	2	32	C:1	T:0
chr28	9895380	2	36	T:1	C:0
chr28	9895399	2	38	G:1	A:0
chr28	9895686	2	38	C:1	T:0
chr28	9895992	2	26	CT:0.423077	C:0.576923
chr28	9897009	2	30	C:0.2	T:0.8
chr28	9897107	2	32	C:1	T:0
chr28	9898734	2	32	G:0.90625	A:0.09375
chr28	9898990	2	38	A:1	T:0
chr28	9899121	2	36	A:0.972222	AT:0.0277778
chr28	9900019	2	34	C:1	T:0
chr28	9900260	2	38	T:0.263158	C:0.736842
chr28	9902271	2	36	C:1	A:0
chr28	9902493	2	34	GTA:0.970588	G:0.0294118
chr28	9902507	2	34	A:0.323529	T:0.676471
chr28	9902509	2	34	A:0.205882	T:0.794118
chr28	9902511	2	34	A:0.205882	T:0.794118
chr28	9902600	2	38	T:0.710526	C:0.289474
chr28	9903002	2	38	C:0.236842	CA:0.763158
chr28	9903712	2	40	C:0.75	T:0.25
chr28	9904554	2	36	G:0.888889	A:0.111111
chr28	9904665	2	36	CT:0.666667	C:0.333333
chr28	9905089	2	34	C:0.382353	A:0.617647
chr28	9905601	2	40	A:0.85	T:0.15
chr28	9905625	2	38	T:0.631579	C:0.368421
chr28	9906398	3	28	C:0.535714	CA:0.178571

CAA:0.285714
chr28	9906895	2	38	T:1	A:0
chr28	9908236	2	36	C:0.972222	T:0.0277778
chr28	9908829	2	34	TAC:1	T:0
chr28	9909116	2	40	A:0.675	T:0.325
chr28	9909253	3	32	TA:0.84375	T:0.15625
TAA:0
chr28	9909408	2	38	G:0.684211	A:0.315789
chr28	9910182	2	38	G:0.815789	A:0.184211
chr28	9910194	2	40	T:1	C:0
chr28	9910897	2	40	C:0.4	CAA:0.6
chr28	9910907	2	40	A:1	C:0
chr28	9910908	2	40	C:0.3	A:0.7
chr28	9911166	2	36	TACAGAC:0.555556	T:0.444444
chr28	9911617	2	36	C:0.277778	A:0.722222
chr28	9912096	2	28	TA:0.678571	T:0.321429
chr28	9912205	2	38	C:0.842105	T:0.157895
chr28	9912296	2	38	G:1	A:0
chr28	9912299	2	38	C:1	A:0
chr28	9912740	2	36	G:1	A:0
chr28	9913290	2	36	G:0.666667	A:0.333333
chr28	9914398	2	36	G:0.972222	GA:0.0277778
chr28	9914533	2	36	C:0.777778	CT:0.222222
chr28	9914702	2	36	C:0.777778	T:0.222222
chr28	9915736	3	38	T:0.315789	TA:0.605263
TAA:0.0789474
chr28	9916602	2	36	C:0.805556	T:0.194444
chr28	9916893	2	40	AGG:0.35	A:0.65
chr28	9916896	2	40	T:0.35	TAG:0.65
chr28	9916898	3	40	AAATGAT:0.125	A:0.6
ATAGTAATGAT:0.275
chr28	9917159	2	38	G:0.631579	A:0.368421
chr28	9917550	2	38	G:0.921053	A:0.0789474
chr28	9917873	2	38	T:0.894737	A:0.105263
chr28	9917902	2	38	C:0.184211	CCA:0.815789
chr28	9917957	2	38	C:0.157895	G:0.842105
chr28	9918034	2	38	C:0.421053	T:0.578947
chr28	9918417	2	40	A:0.275	G:0.725
chr28	9918510	4	40	ATGTGTGTG:0.275	A:0.65	ATGTGTG:
0.075	ATGTGTGTGTGTGTG:0
chr28	9918860	2	36	T:0.388889	C:0.611111
chr28	9918865	2	36	T:0.416667	TA:0.583333
chr28	9920614	2	36	G:1	C:0
chr28	9920691	2	34	A:0.794118	C:0.205882
chr28	9921321	2	36	A:0.388889	G:0.611111
chr28	9921393	2	38	A:1	T:0
chr28	9921431	2	40	T:1	TA:0
chr28	9921727	2	40	A:1	T:0
chr28	9921736	2	38	T:0.184211	TAAAG:0.815789
chr28	9921988	2	40	G:0.35	A:0.65
chr28	9922247	3	40	GAGAAAGAA:0.25	G:0.75	GAGAA:0
chr28	9922309	2	40	GAGA:0.625	G:0.375
chr28	9922312	2	38	AAAAG:0.894737	A:0.105263
chr28	9922350	2	40	AAGAAAGAAAGAAAAAG:0.625	A:0.375

chr28 9922389 3 40 AAAAGAAAGAAAG:0.775 A:0.15
AAAAGAAAG:0.075
chr28 9922484 2 38 A:0.394737 AAACAAC:0.605263
chr28 9922491 2 38 A:0.894737 T:0.105263
chr28 9923102 2 36 C:1 T:0
chr28 9923322 2 36 A:0.333333 G:0.666667
chr28 9923729 2 34 G:1 GA:0
chr28 9924429 2 38 T:0.578947 C:0.421053
chr28 9924670 2 34 G:0.352941 GA:0.647059
chr28 9925035 2 40 T:0.3 C:0.7
chr28 9925087 2 40 C:0.875 T:0.125
chr28 9925180 2 40 A:0.375 T:0.625
chr28 9925490 2 38 G:0.315789 C:0.684211
chr28 9925725 2 36 T:0.222222 C:0.777778
chr28 9927365 2 40 T:0.275 C:0.725
chr28 9927462 2 40 AAT:1 A:0
chr28 9927599 2 36 C:0.666667 T:0.333333
chr28 9928055 2 40 A:0.825 T:0.175
chr28 9928142 2 38 A:1 T:0
chr28 9928654 2 38 C:0.0263158 T:0.973684
chr28 9929062 2 34 CCTCT:0.647059 C:0.352941
chr28 9929534 2 36 CAAAAA:0.916667 C:0.0833333
chr28 9929677 2 16 T:0.875 TA:0.125
chr28 9930040 2 36 TA:0.666667 T:0.333333
chr28 9930276 2 36 TA:0.75 T:0.25
chr28 9931128 2 22 TA:0.636364 T:0.363636
chr28 9932271 2 40 C:0.575 T:0.425
chr28 9932368 2 38 T:0.894737 C:0.105263
chr28 9932584 2 36 T:0.5 C:0.5
chr28 9932724 2 34 C:0.294118 G:0.705882
chr28 9932870 6 38 AATAGATAG:0.342105 A:
0.342105 AATAG:0.0263158 AATAGATAGATAG:0.236842
AATAGATAGATAGATAG:0.0263158 AATAGATAGATAGATAGATAG:0.0263158
chr28 9934201 2 40 G:1 A:0
chr28 9934316 2 38 C:0.868421 T:0.131579
chr28 9934493 2 40 A:0.225 G:0.775
chr28 9934961 2 34 C:0.676471 CA:0.323529
chr28 9935090 2 38 AT:1 A:0
chr28 9935179 2 36 G:0.666667 A:0.333333
chr28 9935476 2 36 T:1 G:0
chr28 9935849 3 38 T:0.684211 TA:0.315789
TAA:0
chr28 9936028 2 40 GA:0 G:1
chr28 9936036 2 40 G:0 GA:1
chr28 9936130 2 36 G:0.305556 A:0.694444
chr28 9938943 3 38 C:0.684211 CA:0.0789474
CAA:0.236842
chr28 9939204 2 34 AAC:0.794118 A:0.205882
chr28 9939206 2 32 C:0.90625 A:0.09375
chr28 9940272 2 36 A:0.805556 G:0.194444
chr28 9940348 2 38 TTTC:0.789474 T:0.210526
chr28 9942369 2 40 GGAGA:1 G:0
chr28 9944305 2 32 T:0.4375 C:0.5625
chr28 9945252 2 36 T:0.777778 C:0.222222

chr28 9945832 2 36 TA:0.666667 T:0.333333
chr28 9947583 2 40 T:1 C:0
chr28 9947736 3 36 A:0.805556 AG:0.194444
AGG:0
chr28 9947747 3 36 A:0.666667 AG:0.333333
G:0
chr28 9947926 2 36 C:1 A:0
chr28 9947989 2 36 C:0.888889 CA:0.111111
chr28 9948544 2 38 A:1 C:0
chr28 9949016 2 32 ACAAT:0.6875 A:0.3125
chr28 9949084 2 34 A:0.705882 AT:0.294118
chr28 9949465 2 32 T:0.875 A:0.125
chr28 9950701 2 32 T:1 C:0
chr28 9951013 2 30 A:0.4 G:0.6
chr28 9951541 2 30 C:0.6 CCTCT:0.4
chr28 9952690 2 40 C:0.85 T:0.15
chr28 9953010 2 38 A:1 T:0
chr28 9953318 2 26 CCT:0.653846 C:0.346154
chr28 9953671 2 40 C:1 T:0
chr28 9953923 3 36 G:0.75 GA:0.222222 GAA:
0.0277778
chr28 9954492 2 38 G:0.342105 GA:0.657895
chr28 9954881 2 36 T:0.388889 G:0.611111
chr28 9954891 2 34 AT:1 A:0
chr28 9954901 2 34 T:0.352941 A:0.647059
chr28 9955119 2 36 T:0.361111 TA:0.638889
chr28 9955544 2 40 G:0.875 T:0.125
chr28 9955881 2 38 C:0.342105 G:0.657895
chr28 9956105 2 38 C:0.921053 T:0.0789474
chr28 9956137 3 36 G:0.555556 GTCTC:0.111111
GTCTCTC:0.333333
chr28 9956173 2 36 TA:0.694444 T:0.305556
chr28 9956424 2 34 A:0.676471 G:0.323529
chr28 9956653 2 32 C:0.375 T:0.625
chr28 9957148 2 38 T:0.684211 C:0.315789
chr28 9957756 2 34 T:0.323529 A:0.676471
chr28 9957779 2 38 T:0.973684 TAAAA:0.0263158
chr28 9957785 2 38 T:0.973684 TAA:0.0263158
chr28 9957787 2 38 A:0.368421 AAT:0.631579
chr28 9957794 3 38 TA:0.289474 AA:0.0789474
T:0.631579
chr28 9957796 2 32 A:0.96875 AT:0.03125
chr28 9957802 2 38 A:0.368421 T:0.631579
chr28 9957821 2 38 T:0.368421 C:0.631579
chr28 9957952 5 36 CCTCTCTCTCTCTCTCTCT:0.166667
C:0.666667 CCTCTCTCTCT:0.0277778 CCTCTCTCTCTCT:0.111111
CCTCTCTCTCTCTCT:0.0277778
chr28 9958001 2 36 CATAA:0.777778 C:0.222222
chr28 9958025 3 38 A:0.710526 C:0.0263158
AATAC:0.263158
chr28 9958242 2 28 CT:0.535714 C:0.464286
chr28 9958526 2 38 A:0.263158 T:0.736842
chr28 9958909 2 40 C:0.925 T:0.075
chr28 9959119 2 32 T:0.25 C:0.75

chr28 9959432 2 34 G:0.764706 A:0.235294
chr28 9959710 2 38 C:1 G:0
chr28 9959963 2 36 T:0.805556 C:0.194444
chr28 9960316 2 40 TA:0.8 T:0.2
chr28 9960317 2 40 A:0.925 T:0.075
chr28 9960471 2 32 TG:1 T:0
chr28 9960605 3 34 TA:0.5 T:0.382353 TAA:
0.117647
chr28 9960622 2 36 T:0.777778 TCTG:0.222222
chr28 9960700 2 34 G:0.941176 A:0.0588235
chr28 9960975 2 36 A:1 T:0
chr28 9961089 2 36 C:0.75 T:0.25
chr28 9961105 2 38 C:0.894737 T:0.105263
chr28 9961338 2 40 A:0.65 AG:0.35
chr28 9961482 2 40 C:0.4 CT:0.6
chr28 9961588 2 40 T:0.675 TC:0.325
chr28 9961876 2 38 A:0.736842 G:0.263158
chr28 9962049 2 40 CAT:0.85 C:0.15
chr28 9962281 2 28 CCT:0.928571 C:0.0714286
chr28 9962470 2 34 T:1 C:0
chr28 9962583 2 40 A:1
AAGACATCACCCAGTCTACGGCCATACCACCCTGAACGCGCCCGATCTCGTCTGATCTCGGAAGCTAA
GCAGGGTCGGGCCTGGTTAGTACTTGGATGGG:0
chr28 9962672 2 38 T:0.789474 C:0.210526
chr28 9963026 2 38 C:0.868421 T:0.131579
chr28 9963417 2 40 C:0.725 T:0.275
chr28 9963449 2 40 G:1 A:0
chr28 9963862 2 36 C:0.222222 T:0.777778
chr28 9964118 2 38 C:0.789474 A:0.210526
chr28 9964363 2 36 T:0.833333 C:0.166667
chr28 9964449 2 40 C:0.875 CCTCT:0.125
chr28 9964485 4 34 T:0.294118 TA:0.558824
TAA:0.147059 TAAA:0
chr28 9964630 2 36 C:0.833333 CCTCT:0.166667
chr28 9964740 2 40 GA:0.175 G:0.825
chr28 9964838 2 40 A:0.15 G:0.85
chr28 9965642 2 38 G:0.894737 A:0.105263
chr28 9965676 2 36 G:0.75 C:0.25
chr28 9967175 2 38 A:1 G:0
chr28 9968483 2 38 A:0.368421 G:0.631579
chr28 9969013 2 38 T:0.605263 C:0.394737
chr28 9969507 2 36 AAAG:1 A:0
chr28 9969512 2 36 A:1 C:0
chr28 9969777 3 40 AAC:0.625 A:0.025 AACACAC:
0.35
chr28 9969871 2 38 G:1 A:0
chr28 9969879 2 38 T:0.263158 C:0.736842
chr28 9970039 2 36 CGGGAGCCCCGCGAT:0.0277778
C:0.972222
chr28 9971287 2 24 G:0.541667 T:0.458333
chr28 9971366 2 28 C:0.714286 A:0.285714
chr28 9971397 2 32 G:0.71875 T:0.28125
chr28 9971574 2 28 C:0.785714 A:0.214286
chr28 9971681 2 38 G:0.736842 C:0.263158

chr28 9971687 2 36 A:0.666667 C:0.333333
chr28 9972155 2 32 C:1 CTTT:0
chr28 9972167 2 30 T:0.766667 TTA:0.233333
chr28 9972180 2 32 C:0.40625 T:0.59375
chr28 9972222 2 30 A:1 T:0
chr28 9972623 2 36 AT:0.916667 A:0.0833333
chr28 9972653 4 38 GT:0.105263 G:0.421053
GTTT:0.263158 GTTTT:0.210526
chr28 9972690 3 36 CAGAG:0.416667 C:0.333333
CAGAGAG:0.25
chr28 9973458 2 38 G:0.763158 A:0.236842
chr28 9973537 2 36 A:0.5 AAC:0.5
chr28 9973915 2 40 C:0.65 CAA:0.35
chr28 9974638 2 40 T:0.3 C:0.7
chr28 9974833 2 38 C:0.447368 T:0.552632
chr28 9974848 2 38 C:1 T:0
chr28 9974904 2 38 A:0.131579 G:0.868421
chr28 9974936 2 36 C:0.333333 T:0.666667
chr28 9975592 3 36 C:0.611111 CACAG:0.111111
CACACAG:0.277778
chr28 9975627 2 32 A:0.4375 G:0.5625
chr28 9975839 2 38 T:0.684211 C:0.315789
chr28 9975887 2 40 T:0.325 C:0.675
chr28 9975979 2 40 A:0.425 C:0.575
chr28 9976067 2 40 A:0.3 T:0.7
chr28 9976079 2 40 C:0.3 T:0.7
chr28 9976406 2 38 T:0.315789 A:0.684211
chr28 9976412 2 38 T:0.315789 C:0.684211
chr28 9976549 2 32 C:0.40625 T:0.59375
chr28 9976570 2 30 GTC:0.466667 G:0.533333
chr28 9976621 2 38 T:0.5 G:0.5
chr28 9976866 2 34 T:0.411765 TAG:0.588235
chr28 9977173 2 30 G:0.566667 A:0.433333
chr28 9977269 2 36 G:0.333333 A:0.666667
chr28 9977368 2 38 G:0.263158 A:0.736842
chr28 9977531 2 38 C:0.394737 CA:0.605263
chr28 9977551 3 40 C:0.625 CA:0 CAA:0.375
chr28 9977615 2 40 T:0.35 C:0.65
chr28 9977652 2 40 G:1 A:0
chr28 9977724 2 38 G:0.473684 A:0.526316
chr28 9977937 2 36 A:0.361111 G:0.638889
chr28 9978248 2 34 A:0.441176 G:0.558824
chr28 9978328 2 38 A:0.710526 G:0.289474
chr28 9978417 2 38 T:0.394737 C:0.605263
chr28 9978479 2 32 A:1 G:0
chr28 9978950 2 36 T:0.444444 C:0.555556
chr28 9979983 2 32 C:1 T:0
chr28 9980549 2 40 C:0.775 T:0.225
chr28 9980829 2 36 T:0.361111 C:0.638889
chr28 9980925 2 38 C:1 T:0
chr28 9981357 2 34 G:1 A:0
chr28 9981483 2 32 G:0.65625 A:0.34375
chr28 9981866 2 38 A:1 ATCCCTGGG:0
chr28 9981870 2 38 C:1 CGCAGCGGT:0

chr28	9981975	2	40	T:0.3	C:0.7	
chr28	9982005	2	38	A:0.368421	G:0.631579	
chr28	9982050	2	38	C:0.973684	T:0.0263158	
chr28	9982104	2	40	C:0.5	T:0.5	
chr28	9982112	2	40	G:0.5	A:0.5	
chr28	9982162	3	40	CCTCTCT:0.275	C:0.65	CCTCT:0.075
chr28	9982193	2	38	C:0.368421	G:0.631579	
chr28	9982831	2	36	C:0.333333	G:0.666667	
chr28	9982863	2	38	C:0.394737	T:0.605263	
chr28	9983073	2	40	C:0.375	T:0.625	
chr28	9983260	2	36	G:0.305556	A:0.694444	
chr28	9983295	2	34	C:0.705882	A:0.294118	
chr28	9983396	2	38	G:1	A:0	
chr28	9983522	2	40	G:0.425	A:0.575	
chr28	9983533	2	40	T:0.425	C:0.575	
chr28	9983570	2	38	C:0.447368	T:0.552632	
chr28	9984009	2	36	T:0.388889	C:0.611111	
chr28	9984025	2	38	G:0.421053	A:0.578947	
chr28	9984223	2	40	C:1	G:0	
chr28	9984291	2	36	C:0.333333	G:0.666667	
chr28	9984452	2	38	T:1	C:0	
chr28	9984534	2	38	C:0.263158	G:0.736842	
chr28	9984633	2	40	G:1	A:0	
chr28	9984635	2	40	G:0.4	C:0.6	
chr28	9984850	2	38	C:1	T:0	
chr28	9984907	2	30	T:0.8	TA:0.2	
chr28	9985389	2	32	A:1	G:0	
chr28	9985655	2	34	G:1	A:0	
chr28	9985722	2	36	T:0.0277778	C:0.972222	
chr28	9985736	2	34	A:0.382353	T:0.617647	
chr28	9985765	2	36	T:1	A:0	
chr28	9985816	2	34	T:1	TA:0	
chr28	9986285	2	40	C:0.675	T:0.325	
chr28	9986551	2	40	T:1	C:0	
chr28	9986841	2	36	G:1	T:0	
chr28	9986915	2	38	C:1	G:0	
chr28	9987186	2	36	T:0.388889	TAG:0.611111	
chr28	9987300	2	38	A:1	T:0	
chr28	9987449	2	38	C:1	T:0	
chr28	9987855	2	38	C:1	A:0	
chr28	9987877	2	36	T:0.444444	TA:0.555556	
chr28	9987927	3	38	AT:0.421053	A:0.342105	ATT:0.236842
chr28	9988108	2	40	A:1	T:0	
chr28	9988315	2	40	T:1	C:0	
chr28	9988511	2	18	C:0.222222	CA:0.777778	
chr28	9989242	2	30	T:1	A:0	
chr28	9990006	2	38	C:1	A:0	
chr28	9990178	2	38	C:0.684211	T:0.315789	
chr28	9990183	2	36	C:0.333333	T:0.666667	
chr28	9990416	2	38	A:1	G:0	
chr28	9990557	2	40	T:1	A:0	
chr28	9990932	2	38	T:1	C:0	

chr28	9990959	2	36	T:1	C:0
chr28	9990970	2	36	C:1	T:0
chr28	9991065	2	38	C:0.631579	T:0.368421
chr28	9991102	2	36	T:0.694444	G:0.305556
chr28	9991563	2	36	A:1	T:0
chr28	9991672	2	34	T:0.676471	C:0.323529
chr28	9991885	2	34	C:1	T:0
chr28	9992325	2	30	C:0.366667	T:0.633333
chr28	9992501	2	22	G:0.863636	A:0.136364
chr28	9992546	2	24	AGGAGG:0.208333	A:0.791667
chr28	9992570	2	24	G:0.208333	A:0.791667
chr28	9992722	2	34	G:0.323529	A:0.676471
chr28	9992731	2	34	C:0.323529	A:0.676471
chr28	9992882	2	36	A:1	G:0
chr28	9992924	2	38	G:0.368421	T:0.631579
chr28	9992928	2	38	A:0.368421	G:0.631579
chr28	9992963	2	38	A:0.368421	G:0.631579
chr28	9993055	2	34	G:0.5	A:0.5
chr28	9993060	2	34	C:0.5	T:0.5
chr28	9993145	2	36	C:0.277778	T:0.722222
chr28	9993279	2	34	G:0.294118	A:0.705882
chr28	9993341	2	36	C:0.333333	T:0.666667
chr28	9993410	2	36	T:0.277778	C:0.722222
chr28	9993413	2	36	T:0.277778	TG:0.722222
chr28	9993962	2	40	C:0.425	T:0.575
chr28	9994647	2	36	C:0.833333	A:0.166667
chr28	9994812	2	32	T:0.40625	C:0.59375
chr28	9995928	2	36	G:0.361111	T:0.638889
chr28	9996072	2	36	G:0.694444	A:0.305556
chr28	9996290	2	36	G:0.694444	A:0.305556
chr28	9996574	2	40	AAGC:0.75	A:0.25
chr28	9996642	2	36	T:0.388889	C:0.611111
chr28	9997195	2	34	G:0.441176	T:0.558824
chr28	9997477	2	34	C:0.764706	G:0.235294
chr28	9997600	4	40	GATTTATTTATTTATTT:0.2	G:0.45
GATTTATTTATTTATTTATTT:0.15	GATTTATTTATTTATTTATTTATTTATTTATTT:
0.2
chr28	9997720	2	34	C:0.647059	T:0.352941
chr28	9997873	4	40	T:0.55	TCACA:0.325	TCACACA:
0.125	TCACACACA:0
chr28	9997890	2	40	C:1	G:0
chr28	9998077	2	40	C:0.725	T:0.275
chr28	9998409	2	36	T:0.722222	C:0.277778
chr28	9998552	2	38	A:0.394737	G:0.605263
chr28	9999121	2	36	C:0.333333	T:0.666667
chr28	10000713	2	36	G:0.888889	A:0.111111
chr28	10001026	2	32	TA:0.5	T:0.5
chr28	10002489	2	38	C:0.447368	T:0.552632
chr28	10002497	2	40	GCTCT:0.725	G:0.275
chr28	10004928	2	38	GA:0.394737	G:0.605263
chr28	10004934	2	38	A:0.394737	G:0.605263
chr28	10004977	2	34	T:0.823529	C:0.176471
chr28	10005188	2	36	C:0.305556	A:0.694444
chr28	10005207	2	38	A:0.315789	G:0.684211

chr28	10005272	2	34	A:0.352941	G:0.647059				
chr28	10005547	2	38	A:0.315789	T:0.684211				
chr28	10005764	2	38	A:0.763158	G:0.236842				
chr28	10006211	2	38	C:0.421053	T:0.578947				
chr28	10006365	2	38	T:0.368421	C:0.631579				
chr28	10006642	2	34	C:0.294118	T:0.705882				
chr28	10006683	2	34	G:0.705882	A:0.294118				
chr28	10006760	2	36	T:0.388889	TAGCCA:0.611111				
chr28	10006950	2	36	G:0.388889	A:0.611111				
chr28	10007006	2	32	G:0.40625	T:0.59375				
chr28	10007231	2	34	C:1	T:0				
chr28	10007530	2	32	G:0.375	A:0.625				
chr28	10007900	2	36	G:0.305556	A:0.694444				
chr28	10008098	2	34	G:0.882353	A:0.117647				
chr28	10008446	2	36	C:0.833333	G:0.166667				
chr28	10009248	2	38	A:0.447368	C:0.552632				
chr28	10009355	2	38	A:0.315789	C:0.684211				
chr28	10009415	2	34	C:0.411765	T:0.588235				
chr28	10009645	2	38	G:0.394737	A:0.605263				
chr28	10009655	2	38	T:0.394737	C:0.605263				
chr28	10010658	2	38	A:0.394737	G:0.605263				
chr28	10010680	2	40	G:0.425	T:0.575				
chr28	10010872	2	34	C:0.352941	T:0.647059				
chr28	10011312	2	36	G:0.305556	C:0.694444				
chr28	10011360	2	36	T:1	TCATC:0				
chr28	10012507	2	34	T:1	C:0				
chr28	10012544	2	36	G:0.666667	A:0.333333				
chr28	10013329	2	38	G:0.763158	T:0.236842				
chr28	10013461	2	40	C:0.375	G:0.625				
chr28	10015175	2	38	AT:0.394737	A:0.605263				
chr28	10015379	2	34	CT:0.617647	C:0.382353				
chr28	10015437	2	34	G:0.676471	A:0.323529				
chr28	10015601	2	36	C:0.916667	CT:0.0833333				
chr28	10015852	2	36	G:0.527778	C:0.472222				
chr28	10015955	2	38	G:0.736842	A:0.263158				
chr28	10015988	2	36	G:0.472222	A:0.527778				
chr28	10016418	2	40	A:0.375	G:0.625				
chr28	10016464	2	38	ACT:0.368421	A:0.631579				
chr28	10016606	2	38	G:0.973684	T:0.0263158				
chr28	10016758	2	40	A:0.675	G:0.325				
chr28	10016842	2	40	T:0.75	C:0.25				
chr28	10017100	2	36	A:0.666667	G:0.333333				
chr28	10018096	2	34	A:1	G:0				
chr28	10018353	2	36	G:0.888889	T:0.111111				
chr28	10020076	2	36	A:0.388889	C:0.611111				
chr28	10020334	6	40	ATTATTTATTTATTTATTTATT:0.3	A:0.425	ATTATTTATTTATTTATTTATTTATT:0.075	ATATTTATTTATTTATTTATTTATTTATT:0.2	ATTATTTATTTATTTATTTATTTATTTATT:0	ATATTTATTTATTTATTTATTTATTTATTTATT:0
chr28	10020378	2	40	T:0.575	C:0.425				
chr28	10020673	2	40	T:0.325	G:0.675				
chr28	10020828	2	36	T:0.305556	C:0.694444				
chr28	10021397	2	40	C:0.65	T:0.35				
chr28	10022282	2	32	A:0.46875	G:0.53125				

chr28	10023320	2	38	G:0.473684	A:0.526316	
chr28	10024839	2	36	T:0.333333	C:0.666667	
chr28	10024912	2	40	C:0.4	T:0.6	
chr28	10025008	2	32	C:1	T:0	
chr28	10025114	2	38	A:0.368421	G:0.631579	
chr28	10025601	2	34	T:0.323529	C:0.676471	
chr28	10026279	2	40	G:0.85	A:0.15	
chr28	10026946	2	38	G:0.447368	C:0.552632	
chr28	10027645	2	36	C:0.972222	T:0.0277778	
chr28	10027876	2	36	T:0.333333	C:0.666667	
chr28	10028796	2	38	CCTTCCA:0.368421	C:0.631579	
chr28	10028803	2	38	T:0.368421	TA:0.631579	
chr28	10029083	2	36	G:0.666667	A:0.333333	
chr28	10029354	2	36	G:0.75	A:0.25	
chr28	10029920	2	40	G:0.725	A:0.275	
chr28	10029933	2	40	GGA:0.975	G:0.025	
chr28	10030068	2	34	G:0.411765	A:0.588235	
chr28	10030773	2	30	T:0.333333	C:0.666667	
chr28	10030790	2	26	C:0.884615	CCT:0.115385	
chr28	10030838	2	34	T:0.735294	TAA:0.264706	
chr28	10030894	2	32	T:0.75	A:0.25	
chr28	10031113	2	36	C:0.611111	T:0.388889	
chr28	10031128	2	34	G:1	A:0	
chr28	10031274	2	30	G:0.4	A:0.6	
chr28	10031469	2	36	CATT:1	C:0	
chr28	10031581	2	36	CA:0.694444	C:0.305556	
chr28	10032164	2	40	C:0.725	CA:0.275	
chr28	10032818	2	38	C:0.394737	T:0.605263	
chr28	10032927	2	34	C:1	A:0	
chr28	10033520	2	34	C:0.764706	T:0.235294	
chr28	10034022	2	34	A:0.617647	T:0.382353	
chr28	10034024	2	34	A:0.323529	G:0.676471	
chr28	10034333	2	36	G:1	A:0	
chr28	10034440	2	36	CCT:0.888889	C:0.111111	
chr28	10034758	2	34	A:0.441176	T:0.558824	
chr28	10035134	2	38	CTCTT:1	C:0	
chr28	10035171	2	36	A:1	G:0	
chr28	10035173	2	36	TA:1	T:0	
chr28	10035182	2	36	A:0.638889	T:0.361111	
chr28	10035532	2	32	G:0.71875	A:0.28125	
chr28	10035735	2	32	G:0.6875	A:0.3125	
chr28	10035823	2	38	AAAAAAT:0.289474	A:0.710526	
chr28	10036775	2	38	T:0.973684	C:0.0263158	
chr28	10037636	2	40	GC:0.9	G:0.1	
chr28	10037685	2	38	T:0.447368	C:0.552632	
chr28	10039396	3	32	TTCTCTC:0.65625	T:0.03125	TTCTCTCTC:0.3125
chr28	10040013	2	36	C:1	G:0	
chr28	10040175	2	36	A:0.361111	T:0.638889	
chr28	10040882	2	38	A:1	C:0	
chr28	10041010	2	36	A:0	G:1	
chr28	10041310	2	40	G:1	A:0	
chr28	10042479	2	40	G:1	A:0	
chr28	10042569	2	40	G:1	A:0	

chr28	10042632	2	38	C:1	T:0	
chr28	10042747	2	40	C:0.4	G:0.6	
chr28	10043976	2	36	A:0.277778	G:0.722222	
chr28	10044855	2	40	A:0.675	T:0.325	
chr28	10044930	2	34	A:0.764706	T:0.235294	
chr28	10044931	2	34	A:0.764706	T:0.235294	
chr28	10044951	2	36	C:0.666667	T:0.333333	
chr28	10045052	2	28	T:0.607143	G:0.392857	
chr28	10045135	2	34	T:0.705882	A:0.294118	
chr28	10045151	2	34	C:0.705882	T:0.294118	
chr28	10045236	2	36	GTAAA:0.694444	G:0.305556	
chr28	10045498	2	32	C:0.59375	T:0.40625	
chr28	10045566	2	38	T:0.631579	A:0.368421	
chr28	10045661	2	36	T:0.583333	C:0.416667	
chr28	10045823	2	38	A:0.710526	G:0.289474	
chr28	10046105	2	36	C:0.972222	T:0.0277778	
chr28	10046511	2	32	C:0.5625	CAA:0.4375	
chr28	10046531	2	28	A:0.571429	G:0.428571	
chr28	10046533	2	30	AC:0.333333	A:0.666667	
chr28	10046770	2	40	C:0.7	T:0.3	
chr28	10046871	2	38	T:0.605263	C:0.394737	
chr28	10046949	2	36	C:0.555556	T:0.444444	
chr28	10047007	2	36	C:0.583333	T:0.416667	
chr28	10047016	2	36	GAA:0.611111	G:0.388889	
chr28	10047087	2	36	T:0.583333	TG:0.416667	
chr28	10047088	2	36	G:0.888889	GT:0.111111	
chr28	10047278	2	40	T:0.4	C:0.6	
chr28	10047881	2	38	G:1	A:0	
chr28	10049508	2	36	G:0.638889	T:0.361111	
chr28	10049599	2	26	CTT:0.807692	C:0.192308	
chr28	10050396	2	38	T:0.631579	C:0.368421	
chr28	10051303	2	38	G:1	A:0	
chr28	10052597	2	34	G:0.735294	T:0.264706	
chr28	10052665	2	36	T:0.833333	G:0.166667	
chr28	10054158	2	28	T:0.821429	TA:0.178571	
chr28	10054168	2	28	C:0.821429	A:0.178571	
chr28	10054326	2	34	A:0.558824	T:0.441176	
chr28	10054423	2	36	C:0.777778	T:0.222222	
chr28	10054475	2	36	A:0.777778	C:0.222222	
chr28	10055778	2	34	A:0.558824	G:0.441176	
chr28	10057013	2	38	G:1	A:0	
chr28	10057327	2	36	C:0.222222	CTG:0.777778	
chr28	10057799	2	36	T:0.583333	G:0.416667	
chr28	10058016	2	32	T:0.46875	C:0.53125	
chr28	10058393	2	32	G:0.6875	A:0.3125	
chr28	10058485	2	40	A:0.5	T:0.5	
chr28	10059372	3	34	C:0.205882	CA:0.382353	CAA:0.411765
chr28	10060023	2	36	G:0.583333	T:0.416667	
chr28	10060123	2	32	T:0.46875	C:0.53125	
chr28	10060126	2	30	AT:0.766667	A:0.233333	
chr28	10060315	2	32	AG:0.6875	A:0.3125	
chr28	10060710	2	40	A:0.2	G:0.8	
chr28	10061091	2	36	G:0.444444	A:0.555556	

chr28	10061284	3	32	TA:0.65625	T:0.34375	TAAA:0
chr28	10061832	2	40	T:0.7	TA:0.3	
chr28	10061836	2	38	AAATTAATT:0.657895	A:0.342105	
chr28	10061840	2	40	T:0.925	A:0.075	
chr28	10061883	2	38	C:0.631579	T:0.368421	
chr28	10062552	2	32	T:0.625	C:0.375	
chr28	10062726	2	36	GT:0.527778	G:0.472222	
chr28	10062863	2	40	T:0	C:1	
chr28	10063276	2	38	A:0.631579	AAT:0.368421	
chr28	10063583	2	40	T:0.8	C:0.2	
chr28	10064160	2	40	GT:0.6	G:0.4	
chr28	10064204	2	36	C:0.527778	CAGAGAG:0.472222	
chr28	10064491	2	36	A:0.527778	G:0.472222	
chr28	10064587	2	40	T:0.65	TA:0.35	
chr28	10064593	2	40	AT:0.525	A:0.475	
chr28	10064594	2	40	T:0.65	A:0.35	
chr28	10065475	2	34	C:0.382353	A:0.617647	
chr28	10065578	2	34	A:0.529412	G:0.470588	
chr28	10065761	2	34	G:1	A:0	
chr28	10065988	2	30	C:0.666667	T:0.333333	
chr28	10066201	2	28	TA:0.607143	T:0.392857	
chr28	10066680	2	38	GT:0.710526	G:0.289474	
chr28	10066743	2	34	A:0.5	G:0.5	
chr28	10066979	2	40	C:0.65	T:0.35	
chr28	10067114	2	34	C:0.352941	T:0.647059	
chr28	10067307	2	38	G:1	A:0	
chr28	10067941	2	34	T:0.617647	G:0.382353	
chr28	10067961	2	34	T:0.617647	A:0.382353	
chr28	10068042	2	38	A:0.394737	G:0.605263	
chr28	10068636	2	34	T:0.117647	C:0.882353	
chr28	10069175	2	34	G:0.764706	GA:0.235294	
chr28	10069892	2	34	T:0.588235	C:0.411765	
chr28	10070151	2	36	C:1	T:0	
chr28	10070483	3	38	CAAAA:0.815789	C:0.0263158	CAAAAA:0.157895
chr28	10070487	2	38	A:0.657895	C:0.342105	
chr28	10070720	2	36	G:0.527778	GT:0.472222	
chr28	10070728	2	36	C:0.527778	CT:0.472222	
chr28	10070746	2	36	A:0.972222	T:0.0277778	
chr28	10070777	2	38	C:0.736842	CAG:0.263158	
chr28	10070843	2	38	G:0.342105	A:0.657895	
chr28	10070878	2	38	GGCTAAAGGCGGC:0.605263	G:0.394737	
chr28	10070947	2	34	G:0.588235	A:0.411765	
chr28	10071041	2	34	G:0.235294	A:0.764706	
chr28	10071202	2	36	G:0.722222	A:0.277778	
chr28	10071317	2	38	GA:0.684211	G:0.315789	
chr28	10071810	2	38	A:0.552632	T:0.447368	
chr28	10072480	2	40	G:0.65	A:0.35	
chr28	10072523	2	38	AT:0.605263	A:0.394737	
chr28	10072534	2	38	AT:0.973684	A:0.0263158	
chr28	10072535	2	38	T:0.605263	A:0.394737	

chr28 10072625 2 38 C:0.631579 G:0.368421
chr28 10072630 2 38 C:0.631579 T:0.368421
chr28 10072761 2 36 A:0.611111 C:0.388889
chr28 10072909 2 40 A:0.975 ATGGG:0.025
chr28 10072913 3 40 GTGGA:0.65 G:0.3
GTGGATGGATGGA:0.05
chr28 10072942 3 40 T:0.575 TGGAAGGAA:0.075
TGGATGGATGGAAGGAA:0.35
chr28 10072989 2 40 C:0.675 T:0.325
chr28 10073256 2 38 C:0.631579 A:0.368421
chr28 10073257 2 38 T:0.631579 TA:0.368421
chr28 10073634 2 24 G:0.666667 A:0.333333
chr28 10073742 2 32 G:0.625 C:0.375
chr28 10074158 2 40 C:0.6 G:0.4
chr28 10074162 2 40 G:0.975 A:0.025
chr28 10074844 2 38 T:0.789474 C:0.210526
chr28 10075230 2 32 C:0.65625 T:0.34375
chr28 10075314 3 32 CTT:0.3125 C:0.15625
CT:0.53125
chr28 10075364 2 26 G:0.615385 GT:0.384615
chr28 10075629 2 32 C:0.65625 A:0.34375
chr28 10075750 2 34 G:0.676471 A:0.323529
chr28 10076043 2 40 C:0.575 T:0.425
chr28 10076045 2 40 T:0.575 A:0.425
chr28 10076129 2 40 T:0.975 C:0.025
chr28 10076485 2 38 T:0.947368 C:0.0526316
chr28 10076686 2 40 G:0.75 A:0.25
chr28 10076703 2 40 G:0.475 A:0.525
chr28 10076850 2 36 C:0.638889 T:0.361111
chr28 10076853 2 36 T:0.638889 C:0.361111
chr28 10076907 2 38 C:0.605263 T:0.394737
chr28 10077169 2 38 C:0.631579 T:0.368421
chr28 10077236 2 36 A:0.944444 AT:0.0555556
chr28 10077237 2 36 T:0.666667 TA:0.333333
chr28 10077324 2 34 TG:0.617647 T:0.382353
chr28 10078138 2 32 C:0.625 T:0.375
chr28 10078199 2 26 G:0.769231 A:0.230769
chr28 10078207 3 34 TAAAAAATAAATAAATA:0.823529
T:0 TAATAAATA:0.176471
chr28 10078210 5 34 AAAATAAATAAATAAAT:0.647059
A:0.176471 AAAATAAAT:0 AAAATAAATAAAT:0.176471
TAAATAAATAAATAAAT:0
chr28 10078261 2 36 AT:0.972222 A:0.0277778
chr28 10078263 2 36 AAAT:0.972222 A:0.0277778
chr28 10078526 2 34 C:1 T:0
chr28 10079795 2 38 T:0.605263 C:0.394737
chr28 10080015 2 38 CTG:0.973684 C:0.0263158
chr28 10080029 2 38 A:0.578947 G:0.421053
chr28 10080379 2 38 TA:0.631579 T:0.368421
chr28 10080459 2 34 CCGA:0.647059 C:0.352941
chr28 10080475 2 32 C:0.71875 T:0.28125
chr28 10080691 2 34 C:1 T:0
chr28 10080746 2 36 G:0.666667 A:0.333333
chr28 10080917 3 34 GCA:0.705882 G:0.0294118

GCACA:0.264706
chr28	10081526	2	38	G:0.157895	A:0.842105
chr28	10081739	2	36	C:0.111111	CTTCTGAA:0.888889
chr28	10081788	2	36	A:0.166667	G:0.833333
chr28	10082157	2	36	CAGAGAGAGAG:0.25	C:0.75
chr28	10082780	3	36	CAAATAAATAAATAAATAAATAAAT:0.194444
	C:0.416667	CAAATAAAT:0.388889
chr28	10082898	2	22	A:0.272727	AGGAG:0.727273
chr28	10084653	2	32	C:1	A:0
chr28	10084683	2	40	C:0.225	G:0.775
chr28	10084892	2	36	G:0.194444	T:0.805556
chr28	10085050	2	40	T:0.275	C:0.725
chr28	10085104	2	36	G:0.222222	A:0.777778
chr28	10085310	2	28	CT:0.607143	C:0.392857
chr28	10085343	2	34	C:0.470588	CT:0.529412
chr28	10086152	2	40	T:0.25	C:0.75
chr28	10086243	2	32	C:0.28125	T:0.71875
chr28	10086541	2	34	C:1	A:0
chr28	10086599	2	36	C:0.444444	CCT:0.555556
chr28	10086657	2	38	T:0.315789	TA:0.684211
chr28	10086958	2	40	C:0.225	G:0.775
chr28	10087380	2	32	CTT:0.71875	C:0.28125
chr28	10087430	2	36	C:0.972222	CAG:0.0277778
chr28	10089009	2	36	GCT:0.222222	G:0.777778
chr28	10089396	3	36	CCTCTCTCT:0.194444	C:
0.361111	CCT:0.444444
chr28	10089464	2	36	A:0.166667	G:0.833333
chr28	10089623	2	14	TA:1	T:0
chr28	10089847	3	36	GTCTC:0.444444	G:0.0833333
GTC:0.472222
chr28	10089900	2	38	A:0.236842	AAAAAG:0.763158
chr28	10089994	2	34	T:0.235294	C:0.764706
chr28	10090186	2	26	G:0.192308	T:0.807692
chr28	10090333	2	28	G:0.5	A:0.5
chr28	10090414	2	22	CCT:0.318182	C:0.681818
chr28	10090458	3	34	A:0.970588	AAAAT:0.0294118
AAAATAAAT:0
chr28	10090502	5	34	T:0.176471	TAAATA:0.205882
TAAATAAATA:0.147059	TAAATAAATAAATA:0.0882353
TAAATAAATAAATAAAATA:0.382353
chr28	10091191	2	32	AAAAAAG:0.65625	A:0.34375
chr28	10091192	2	32	AAAAAG:0.625	A:0.375
chr28	10091349	2	38	C:0.236842	A:0.763158
chr28	10091547	2	32	T:0.3125	TCC:0.6875
chr28	10091937	2	34	TTC:0.205882	T:0.794118
chr28	10092104	2	34	C:0.176471	CA:0.823529
chr28	10092126	2	36	T:0.222222	TAAAATA:0.777778
chr28	10092158	2	20	T:0.9	TA:0.1
chr28	10092160	2	32	A:0.875	AAAATAAAAT:0.125
chr28	10092165	2	20	A:0.9	AAAT:0.1
chr28	10092348	2	30	G:0.7	A:0.3
chr28	10092577	2	34	A:0.294118	ATAAAT:0.705882
chr28	10092836	2	32	T:0.21875	A:0.78125
chr28	10092850	2	32	C:0.21875	G:0.78125

chr28	10093038	2	40	GT:0.2	G:0.8			
chr28	10093112	2	38	A:0.236842	AT:0.763158			
chr28	10093364	2	36	A:0.166667	C:0.833333			
chr28	10093366	2	36	C:0.166667	T:0.833333			
chr28	10093569	2	40	G:0.275	C:0.725			
chr28	10093578	2	40	G:0.275	A:0.725			
chr28	10093621	2	38	G:0.184211	A:0.815789			
chr28	10093670	2	40	T:0.175	C:0.825			
chr28	10093679	2	40	G:1	A:0			
chr28	10093886	2	36	C:0.583333	CGCATT:0.416667			
chr28	10093997	2	36	G:0.611111	A:0.388889			
chr28	10094136	2	34	G:0.588235	A:0.411765			
chr28	10094540	2	36	C:1	G:0			
chr28	10094866	2	34	G:0.970588	GA:0.0294118			
chr28	10095524	2	10	C:0.4	G:0.6			
chr28	10095808	2	30	T:0.733333	A:0.266667			
chr28	10095961	2	36	C:0.583333	A:0.416667			
chr28	10096511	5	40	AAC:0.75	A:0.05	AACAC:0	AACACAC:0.2	AACACACAC:0
chr28	10096535	2	40	C:0.55	CAT:0.45			
chr28	10096621	2	36	A:0.583333	G:0.416667			
chr28	10097262	2	36	CA:1	C:0			
chr28	10098620	2	34	G:0.705882	A:0.294118			
chr28	10098707	2	40	T:0.7	C:0.3			
chr28	10099037	2	36	G:0.638889	C:0.361111			
chr28	10099202	2	38	C:1	T:0			
chr28	10099239	2	36	T:0.694444	TC:0.305556			
chr28	10099738	2	40	G:0.65	T:0.35			
chr28	10100126	2	36	C:0.972222	T:0.0277778			
chr28	10100782	2	40	T:1	C:0			
chr28	10100882	2	38	C:0.605263	T:0.394737			
chr28	10101917	2	36	T:0.694444	TA:0.305556			
chr28	10102255	2	34	C:0.588235	T:0.411765			
chr28	10102743	2	38	T:0.578947	A:0.421053			
chr28	10103171	2	36	G:1	A:0			
chr28	10103202	2	32	TA:0.6875	T:0.3125			
chr28	10103505	2	36	T:0.75	C:0.25			
chr28	10103578	2	38	T:0.684211	A:0.315789			
chr28	10103655	2	34	G:0.588235	A:0.411765			
chr28	10103847	2	38	T:1	C:0			
chr28	10104001	2	36	C:0.75	A:0.25			
chr28	10104218	2	40	T:0.625	C:0.375			
chr28	10105506	2	36	A:0	T:1			
chr28	10105512	2	36	G:0	T:1			
chr28	10105531	2	38	C:0.947368	T:0.0526316			
chr28	10105576	2	38	A:0.736842	AT:0.263158			
chr28	10105579	2	38	A:0.736842	T:0.263158			
chr28	10106044	2	34	T:0.705882	A:0.294118			
chr28	10106203	2	36	ACACCCTGGGC:0.555556	A:0.444444			
chr28	10106407	2	38	T:0.684211	C:0.315789			
chr28	10106817	2	34	T:0.617647	C:0.382353			
chr28	10107030	2	36	T:0.666667	C:0.333333			
chr28	10107056	2	38	G:0.657895	GT:0.342105			

chr28	10107095	2	38	A:0.657895	G:0.342105		
chr28	10107288	2	36	C:0.694444	A:0.305556		
chr28	10107430	2	36	G:0.611111	A:0.388889		
chr28	10107531	2	40	T:0.675	A:0.325		
chr28	10107690	2	40	G:1	A:0		
chr28	10107722	2	38	G:0.736842	A:0.263158		
chr28	10108281	2	36	A:0.583333	C:0.416667		
chr28	10108956	2	36	A:0.861111	T:0.138889		
chr28	10109651	2	40	T:0.725	A:0.275		
chr28	10109700	2	38	C:1	G:0		
chr28	10110817	2	36	C:0.583333	CT:0.416667		
chr28	10111283	2	36	G:0.694444	T:0.305556		
chr28	10111747	2	38	C:0.657895	CT:0.342105		
chr28	10112779	2	38	T:0.526316	A:0.473684		
chr28	10112970	2	38	T:0.552632	C:0.447368		
chr28	10113257	2	36	A:0.75	G:0.25		
chr28	10113277	2	38	G:0.631579	A:0.368421		
chr28	10113438	2	34	T:0.647059	G:0.352941		
chr28	10113915	2	34	A:0.676471	G:0.323529		
chr28	10113965	2	38	T:0.684211	A:0.315789		
chr28	10115385	3	36	TAAAA:0.5	T:0	TAAA:0.5	
chr28	10115597	2	34	G:0.970588	A:0.0294118		
chr28	10115897	2	34	C:1	CGTT:0		
chr28	10116159	2	36	CAG:0.722222	C:0.277778		
chr28	10116680	2	30	G:0.766667	GA:0.233333		
chr28	10117651	2	38	C:0.710526	T:0.289474		
chr28	10117766	2	34	C:0.735294	CT:0.264706		
chr28	10119515	2	36	T:1	C:0		
chr28	10119785	2	36	G:0.694444	A:0.305556		
chr28	10120019	2	36	C:1	T:0		
chr28	10120177	2	38	A:0.631579	G:0.368421		
chr28	10120315	2	36	G:0.638889	A:0.361111		
chr28	10120745	2	36	G:1	A:0		
chr28	10121284	2	40	T:0.7	TTTAC:0.3		
chr28	10121586	2	36	C:0.611111	T:0.388889		
chr28	10122715	2	32	C:0.65625	CT:0.34375		
chr28	10122759	2	38	G:0.578947	C:0.421053		
chr28	10122974	2	40	ATCATGATCCCAGGG:0.6	A:0.4		
chr28	10123077	2	38	T:0.631579	C:0.368421		
chr28	10124051	2	36	A:0.666667	C:0.333333		
chr28	10124215	2	36	C:0.694444	T:0.305556		
chr28	10124244	2	34	A:0.852941	AT:0.147059		
chr28	10124862	2	38	ATT:0.842105	A:0.157895		
chr28	10124867	3	38	T:0.5	TAAA:0.157895	TA:0.342105	
chr28	10124999	4	40	G:0.625	GTCTC:0.05	GTCTCTC:0.05	GTCTCTCTCTCTCTC:0.275
chr28	10125084	2	38	A:0.684211	T:0.315789		
chr28	10125493	2	34	C:0.735294	T:0.264706		
chr28	10125613	2	34	G:0.764706	A:0.235294		
chr28	10125652	2	32	A:0.96875	AT:0.03125		
chr28	10125921	2	38	C:1	G:0		
chr28	10125998	2	40	C:0.975	T:0.025		
chr28	10126072	2	38	AC:0.657895	A:0.342105		

chr28	10126127	2	38	T:0.578947	A:0.421053		
chr28	10126138	2	36	T:0.833333	C:0.166667		
chr28	10126617	2	36	T:0.861111	C:0.138889		
chr28	10126622	2	36	T:1	C:0		
chr28	10126737	2	38	A:0.578947	G:0.421053		
chr28	10127431	2	36	T:0.583333	TA:0.416667		
chr28	10127853	2	36	G:0.972222	A:0.0277778		
chr28	10127978	2	36	C:0.861111	T:0.138889		
chr28	10128193	3	36	T:0.472222	TA:0.527778	TAA:0	
chr28	10128339	2	34	A:0.617647	G:0.382353		
chr28	10128587	2	32	A:0.46875	G:0.53125		
chr28	10128663	2	30	G:0.633333	A:0.366667		
chr28	10128703	2	34	A:0.529412	G:0.470588		
chr28	10128796	2	36	A:0.694444	G:0.305556		
chr28	10128797	2	36	A:0.638889	G:0.361111		
chr28	10129048	2	38	C:0.789474	T:0.210526		
chr28	10129091	2	34	T:0.441176	A:0.558824		
chr28	10129195	2	38	ATCT:0.973684	A:0.0263158		
chr28	10129258	2	36	AG:0.944444	A:0.0555556		
chr28	10129397	2	38	T:0.789474	G:0.210526		
chr28	10129433	2	36	A:0.416667	G:0.583333		
chr28	10129449	2	36	G:0.666667	A:0.333333		
chr28	10129496	2	36	T:0	G:1		
chr28	10129566	2	32	T:0.90625	A:0.09375		
chr28	10129780	4	40	A:0.425	AGTTGTTGTT:0.425	AGTTGTTGTTGTT:0.15	AGTTGTTGTTGTTGTT:0
chr28	10130205	2	40	C:0.975	T:0.025		
chr28	10130209	2	40	T:0.45	C:0.55		
chr28	10130249	2	30	C:0.2	CCT:0.8		
chr28	10130303	2	34	TA:0.735294	T:0.264706		
chr28	10130475	2	38	G:0.947368	A:0.0526316		
chr28	10130573	2	38	G:0.868421	A:0.131579		
chr28	10130657	2	26	C:1	CCT:0		
chr28	10130720	2	30	TAA:0.866667	T:0.133333		
chr28	10130743	2	36	C:0.583333	T:0.416667		
chr28	10130774	2	38	A:0	G:1		
chr28	10130874	2	22	G:0.136364	C:0.863636		
chr28	10131021	2	20	T:0	TA:1		
chr28	10131487	2	32	G:0.96875	T:0.03125		
chr28	10131492	2	36	T:0.361111	G:0.638889		
chr28	10131505	2	36	T:0.361111	C:0.638889		
chr28	10131651	2	36	G:0.833333	GC:0.166667		
chr28	10131678	2	38	C:0.973684	T:0.0263158		
chr28	10131857	2	32	TG:0.78125	T:0.21875		
chr28	10132020	2	40	G:0.825	GT:0.175		
chr28	10132022	3	38	TC:0.657895	T:0	TCC:0.342105	
chr28	10132439	2	38	T:0.736842	C:0.263158		
chr28	10132652	3	36	CT:0.416667	C:0.416667	CTT:0.166667	
chr28	10132900	2	36	C:0.972222	T:0.0277778		
chr28	10133410	2	36	T:0.638889	TC:0.361111		
chr28	10133788	2	40	G:1	GAA:0		

chr28 10133978 2 30 T:0.7 A:0.3
chr28 10134071 2 30 C:0.666667 T:0.333333
chr28 10134365 2 30 A:0.533333 G:0.466667
chr28 10134481 2 34 G:0.588235 A:0.411765
chr28 10134640 2 34 AT:0.529412 A:0.470588
chr28 10134643 2 34 AAT:0.529412 A:0.470588
chr28 10134645 2 32 TA:0.59375 T:0.40625
chr28 10134653 2 34 A:0.529412 T:0.470588
chr28 10134828 2 28 C:0.535714 T:0.464286
chr28 10136462 2 36 C:0.944444 T:0.0555556
chr28 10136854 2 36 T:0.972222 C:0.0277778
chr28 10137070 2 40 GAAAAAGAAAACCAAAAAAAAAAAAAAAAAA:
0.6 G:0.4
chr28 10137137 2 34 G:0.676471 A:0.323529
chr28 10137644 2 34 G:0.588235 A:0.411765
chr28 10137692 2 32 C:0.875 T:0.125
chr28 10137697 2 32 T:0.875 A:0.125
chr28 10137774 2 36 C:1 CCTCT:0
chr28 10137795 2 34 C:0.588235 CTG:0.411765
chr28 10138143 2 30 A:0.6 G:0.4
chr28 10138144 2 30 T:0.6 A:0.4
chr28 10138331 2 30 T:0.633333 C:0.366667
chr28 10138822 2 30 C:0.866667 G:0.133333
chr28 10138986 2 34 G:0.411765 A:0.588235
chr28 10139094 2 38 T:0.605263 A:0.394737
chr28 10139240 2 38 T:0.973684 C:0.0263158
chr28 10139597 2 40 A:0.625 C:0.375
chr28 10139797 2 40 G:1 A:0
chr28 10140077 2 40 C:0 G:1
chr28 10140097 2 40 AG:0 A:1
chr28 10140385 3 36 TA:0.388889 T:0.277778
TAA:0.333333
chr28 10140474 2 32 G:1 A:0
chr28 10140533 2 30 ATC:0.533333 A:0.466667
chr28 10140599 2 40 T:0.2 A:0.8
chr28 10140961 2 36 C:0.861111 T:0.138889
chr28 10141158 2 40 G:0.975 T:0.025
chr28 10141522 2 38 G:1 A:0
chr28 10141979 2 40 G:0.425 A:0.575
chr28 10142270 2 36 G:1 A:0
chr28 10142335 2 38 T:0.973684 C:0.0263158
chr28 10142667 2 34 G:1 A:0
chr28 10142776 2 36 CCT:0.861111 C:0.138889
chr28 10142881 2 36 T:0.944444 G:0.0555556
chr28 10143149 2 38 T:0.868421 A:0.131579
chr28 10143281 2 34 G:0.970588 A:0.0294118
chr28 10143371 2 30 C:1 T:0
chr28 10143432 2 38 A:0.973684 T:0.0263158
chr28 10143856 2 34 A:0.588235 G:0.411765
chr28 10144051 2 34 G:0.941176 A:0.0588235
chr28 10144301 2 36 T:0.888889 C:0.111111
chr28 10144486 2 34 A:0.970588 G:0.0294118
chr28 10144544 2 30 CCT:0.7 C:0.3
chr28 10144548 2 40 T:0.8

TCTCTCTCTCTCTCTCTCTGCTTCTCCCTCTGCCTGTGTCTCTGC:0.2
chr28 10144605 2 38 TA:1 T:0
chr28 10144851 2 38 G:0.973684 A:0.0263158
chr28 10145459 2 36 C:0.388889 CTA:0.611111
chr28 10145579 2 38 T:0.631579 G:0.368421
chr28 10145854 2 40 T:0.3 C:0.7
chr28 10145985 2 36 G:0.972222 T:0.0277778
chr28 10145992 2 36 T:0.972222 C:0.0277778
chr28 10146039 2 38 GGTGT:0.657895 G:0.342105
chr28 10146058 2 38 G:0.842105 GTA:0.157895
chr28 10146103 2 38 T:0.657895 C:0.342105
chr28 10146246 2 38 A:0.842105 G:0.157895
chr28 10146279 2 40 T:0.575 C:0.425
chr28 10147010 2 40 G:0.15 C:0.85
chr28 10147135 2 36 G:0.111111 C:0.888889
chr28 10147191 2 36 T:0.944444 G:0.0555556
chr28 10147472 2 36 A:0.944444 G:0.0555556
chr28 10147807 2 40 T:0 TC:1
chr28 10148232 2 30 GA:0.833333 G:0.166667
chr28 10148242 2 30 AGAG:0.833333 A:0.166667
chr28 10148245 3 38 GAAGA:1 G:0 GAAGAAAGA:0
chr28 10148288 2 36 GAA:1 G:0
chr28 10148294 2 16 AAG:0.6875 A:0.3125
chr28 10148300 2 18 GAA:0.5 G:0.5
chr28 10148412 2 38 C:1 T:0
chr28 10149146 2 36 T:0.138889 G:0.861111
chr28 10149410 2 38 A:0.184211 G:0.815789
chr28 10149449 2 36 G:1 T:0
chr28 10149841 2 36 C:0.888889 CT:0.111111
chr28 10149893 2 34 A:0.941176 T:0.0588235
chr28 10149980 2 36 C:0.916667 T:0.0833333
chr28 10149999 2 34 G:0.970588 C:0.0294118
chr28 10150042 2 36 T:0.944444 C:0.0555556
chr28 10150292 2 36 A:0.194444 T:0.805556
chr28 10150297 2 38 G:0.973684 T:0.0263158
chr28 10150322 2 38 C:0.973684 T:0.0263158
chr28 10150341 2 38 AT:0.921053 A:0.0789474
chr28 10150377 4 38 CAG:0.789474 C:0.131579
CAGAGAGAG:0.0263158 CAGAGAGAGAG:0.0526316
chr28 10150480 2 36 A:0.972222 ACATCCTGAG:
0.0277778
chr28 10150723 2 38 CCTTAT:0.947368 C:0.0526316
chr28 10150822 2 40 C:0.975 A:0.025
chr28 10150902 2 38 A:0.0789474 T:0.921053
chr28 10150992 2 40 C:0.125 A:0.875
chr28 10151037 2 36 G:0.111111 T:0.888889
chr28 10151226 2 32 G:0.1875 A:0.8125
chr28 10151411 2 36 T:0.888889 C:0.111111
chr28 10151543 2 36 AT:0.805556 A:0.194444
chr28 10152372 2 34 C:0.176471 T:0.823529
chr28 10152413 4 30 TAA:0.2 T:0.6 TA:0.2 TAAA:0
chr28 10152907 2 32 GA:0.15625 G:0.84375
chr28 10153343 2 34 G:0.176471 A:0.823529
chr28 10153365 2 36 C:0.972222 T:0.0277778

chr28	10153418	2	36	C:0.138889	A:0.861111
chr28	10153642	2	40	C:0.975	T:0.025
chr28	10153904	2	38	G:0.105263	A:0.894737
chr28	10153945	2	36	GTC:0.888889	G:0.111111
chr28	10153996	2	38	T:0.947368	C:0.0526316
chr28	10154047	2	34	A:0.911765	G:0.0882353
chr28	10154104	2	30	A:0.8	T:0.2
chr28	10154196	2	16	C:0.125	CCT:0.875
chr28	10154306	2	32	C:0.96875	A:0.03125
chr28	10154338	2	38	C:0.210526
CCAAAAAAAAAAACAAAAAAAACAAAAAAAAATAAATTAAAAAAAAAAAAAAAAAAAAAAA:
0.789474
chr28	10154339	2	38	G:0.210526	GAACCTGCCTTCC:
0.789474
chr28	10154461	3	30	T:0.133333	TA:0.466667
TAA:0.4
chr28	10154689	2	40	C:0.35	G:0.65
chr28	10154839	2	28	CCTCT:0.0714286	C:0.928571
chr28	10154898	3	36	CA:0.777778	C:0.166667
CAAA:0.0555556
chr28	10155088	2	34	TAAAAGTTTTTAAAAA:0.911765	T:
0.0882353
chr28	10155093	2	34	GTTTTTAAAAAAAAAGT:0.970588
G:0.0294118
chr28	10155179	2	38	G:0.921053	GA:0.0789474
chr28	10155187	2	38	T:0.552632	A:0.447368
chr28	10155196	2	38	T:0.868421	A:0.131579
chr28	10155249	2	22	T:0.909091	C:0.0909091
chr28	10155371	2	32	G:0.84375	C:0.15625
chr28	10155420	2	38	A:0.789474	T:0.210526
chr28	10155466	2	26	ATC:0.846154	A:0.153846
chr28	10155468	2	28	CTCT:0.928571	C:0.0714286
chr28	10155470	2	26	C:0.230769	T:0.769231
chr28	10155496	2	28	T:0.928571	TGATA:0.0714286
chr28	10155500	2	28	ACT:0.928571	A:0.0714286
chr28	10155509	2	28	GAC:0.928571	G:0.0714286
chr28	10155611	2	40	GC:0	G:1
chr28	10155632	2	30	T:0.0333333	C:0.966667
chr28	10155666	2	32	T:0.90625	TA:0.09375
chr28	10155667	3	32	T:0.125	A:0.09375	TA:
0.78125
chr28	10155678	2	28	T:0	TA:1
chr28	10155975	2	38	C:0.947368	CTAA:0.0526316
chr28	10156113	2	36	C:0.0277778	A:0.972222
chr28	10156169	2	34	AAATT:0.970588	A:0.0294118
chr28	10156261	2	40	T:0.15	C:0.85
chr28	10156606	2	36	G:0.944444	A:0.0555556
chr28	10156658	2	32	C:0.09375	CT:0.90625
chr28	10156835	2	32	G:0.21875	A:0.78125
chr28	10156897	2	34	T:0.147059	C:0.852941
chr28	10157052	2	34	T:0.970588	TA:0.0294118
chr28	10157058	4	34	T:0.852941	TTTTA:0.0588235
TTA:0.0294118	TTTTATTTATTTA:0.0588235
chr28	10157251	2	36	C:0.694444

CTATATGAGTAAAACTTTACAATATTTCTGAAAGATCCAAAAATAGACTGGATCCATCGATAA:
0.305556
chr28	10157831	3	38	TA:0.552632	T:0.447368
TAA:0
chr28	10158165	2	34	GTGTC:0.970588	G:0.0294118
chr28	10158167	3	36	GTCTCTCTC:0.638889	G:
0.0833333	GTCTCTC:0.277778
chr28	10158232	3	36	TAAAAAA:0.722222	TA:0.25	T:
0.0277778
chr28	10158238	2	28	A:0.964286	ATT:0.0357143
chr28	10158290	2	30	A:0.933333	AG:0.0666667
chr28	10158392	2	34	T:0.0882353	C:0.911765
chr28	10158447	2	32	CT:0.96875	C:0.03125
chr28	10158486	2	38	T:0.947368	TAC:0.0526316
chr28	10158490	3	38	C:0.973684	CAGAGAGAGAGAG:
0.0263158	CAGAGAGAGAGAGAG:0
chr28	10158492	2	38	G:0.947368	C:0.0526316
chr28	10158537	2	38	G:0.131579	T:0.868421
chr28	10158667	2	36	A:0.972222	G:0.0277778
chr28	10159009	2	36	G:0.138889	A:0.861111
chr28	10159257	2	34	G:1	GA:0
chr28	10159626	2	36	A:0.916667	T:0.0833333
chr28	10159654	2	38	T:0.894737	A:0.105263
chr28	10159695	2	38	T:0.868421	C:0.131579
chr28	10159868	2	32	C:0.78125	CT:0.21875
chr28	10159995	2	24	C:1	T:0
chr28	10160300	2	28	C:0.892857	T:0.107143
chr28	10160324	2	32	C:0.96875	T:0.03125
chr28	10160325	2	32	G:0.8125	T:0.1875
chr28	10160674	2	34	C:0.0882353	A:0.911765
chr28	10161160	2	38	A:0.947368	G:0.0526316
chr28	10161412	2	38	TAATTTATA:0.894737	T:
0.105263
chr28	10161423	2	38	GA:0.921053	G:0.0789474
chr28	10161772	2	34	TTC:0.941176	T:0.0588235
chr28	10161810	2	40	CTGTGTGTGTGTG:0.15	C:0.85
chr28	10161852	2	34	T:0.852941	A:0.147059
chr28	10161975	2	38	C:0.868421	A:0.131579
chr28	10162099	2	32	TCA:0.1875	T:0.8125
chr28	10162212	2	38	CACTT:0.0789474	C:0.921053
chr28	10162302	4	36	C:0.111111	CT:0.111111
CTT:0.361111	CTTT:0.416667
chr28	10162453	2	30	C:0.166667	T:0.833333
chr28	10162530	2	38	A:0.947368	G:0.0526316
chr28	10162553	2	38	C:0.973684	A:0.0263158
chr28	10162727	2	34	G:1	A:0
chr28	10162764	2	32	A:0.875	G:0.125
chr28	10162876	3	34	T:0.205882	TAAA:0.411765
TAAAA:0.382353
chr28	10162901	2	32	T:0.90625	A:0.09375
chr28	10163470	2	24	G:0.958333	T:0.0416667
chr28	10164070	2	34	GTCTC:1	G:0
chr28	10164099	2	38	TCTG:0.947368	T:0.0526316
chr28	10164101	4	40	T:0.9	TC:0.075	TCTCTC:0

TCTCTCTCTCTCTC:0.025
chr28 10164102 2 40 G:0.9 T:0.1
chr28 10164474 2 32 G:0.1875 A:0.8125
chr28 10164559 2 36 A:0.972222 AGCGCC:0.0277778
chr28 10164587 2 34 C:0.941176 T:0.0588235
chr28 10164611 2 36 C:1 T:0
chr28 10164666 2 32 C:0.15625 G:0.84375
chr28 10164669 2 32 TCTTC:0.96875 T:0.03125
chr28 10164671 3 36 TTCGTTC:0.194444 T:0.75 TGTTC:
0.0555556
chr28 10164675 2 32 T:0.90625 TC:0.09375
chr28 10164677 2 32 C:0.90625 CT:0.09375
chr28 10164702 2 30 A:0 AG:1
chr28 10164787 2 38 C:0.868421 G:0.131579
chr28 10164880 2 40 G:0.175 A:0.825
chr28 10165039 2 30 TCATG:0.233333 T:0.766667
chr28 10165148 3 36 T:0.166667
TTCTCTCTCTCTCTCTC:0.111111 TTCTCTCTCTCTCTCTCTC:0.722222
chr28 10165184 4 34 TAAA:0.264706 T:0.529412
TA:0.205882 TAA:0
chr28 10165347 2 24 C:0.125 CA:0.875
chr28 10165388 2 32 C:1 T:0
chr28 10165697 2 34 G:0.852941 A:0.147059
chr28 10165799 2 38 C:0.815789 A:0.184211
chr28 10165856 2 38 A:0.894737 G:0.105263
chr28 10166000 2 34 C:0.911765 T:0.0882353
chr28 10166114 2 30 T:0.933333 TG:0.0666667
chr28 10166115 3 30 GTTT:0.733333 G:0.0333333
GTT:0.233333
chr28 10166116 2 30 T:0.866667 TGG:0.133333
chr28 10166119 2 30 T:0.966667 G:0.0333333
chr28 10166202 2 40 G:0.9 A:0.1
chr28 10166217 2 40 T:0.9 C:0.1
chr28 10166314 3 36 TGA:0.833333 T:0.166667
TGAGAGA:0
chr28 10166335 2 34 GA:0.970588 G:0.0294118
chr28 10166338 2 34 AGAGGC:0.970588 A:0.0294118
chr28 10166523 2 38 G:0.973684 GC:0.0263158
chr28 10166524 2 36 T:0.0833333 C:0.916667
chr28 10166526 2 38 T:0.973684 C:0.0263158
chr28 10166527 2 36 CCA:0.0833333 C:0.916667
chr28 10166529 2 38 A:0.973684 G:0.0263158
chr28 10166595 2 38 GAGAGAC:0.973684 G:0.0263158
chr28 10166651 2 36 A:0.861111 G:0.138889
chr28 10166815 2 38 C:0.894737 A:0.105263
chr28 10167035 2 24 T:0.916667 TA:0.0833333
chr28 10167173 2 36 AT:0.944444 A:0.0555556
chr28 10167306 2 36 T:0.972222 C:0.0277778
chr28 10167353 2 36 C:0.944444 T:0.0555556
chr28 10167432 2 34 C:0.970588 T:0.0294118
chr28 10167461 2 36 TA:0.972222 T:0.0277778
chr28 10167532 2 36 G:0.916667 A:0.0833333
chr28 10167797 2 36 C:0.888889 T:0.111111
chr28 10167871 2 38 G:0.131579 A:0.868421

chr28	10167923	2	38	A:0.894737	C:0.105263			
chr28	10167993	2	32	A:0.90625	G:0.09375			
chr28	10168029	2	34	G:0.0588235	GT:0.941176			
chr28	10168285	2	38	T:0.0526316	C:0.947368			
chr28	10168409	2	30	TTG:0.9	T:0.1			
chr28	10168410	2	24	TG:0.416667	T:0.583333			
chr28	10168483	2	40	A:0.1	G:0.9			
chr28	10168500	2	40	TA:0.1	T:0.9			
chr28	10168720	2	38	A:0.157895	G:0.842105			
chr28	10169095	2	32	A:0.875	ATT:0.125			
chr28	10169138	2	36	C:1	A:0			
chr28	10169343	2	28	AT:0.821429	A:0.178571			
chr28	10169635	2	38	T:1	A:0			
chr28	10169645	2	38	T:0.868421	C:0.131579			
chr28	10169770	2	40	A:0.925	G:0.075			
chr28	10169804	3	38	CTT:0.842105	C:0.0789474	CT:0.0789474		
chr28	10170040	2	32	T:1	C:0			
chr28	10170089	2	38	G:0.973684	GT:0.0263158			
chr28	10170108	2	34	C:0.882353	T:0.117647			
chr28	10170182	5	38	T:0.921053	TTTTA:0	TTATTTA:0	TTATTTATTTA:0	TTATTTATTTATTTA:0.0789474
chr28	10170238	2	36	A:0.944444	G:0.0555556			
chr28	10170244	2	36	G:0.944444	T:0.0555556			
chr28	10170287	2	36	G:1	A:0			
chr28	10170290	2	36	G:0.944444	A:0.0555556			
chr28	10170527	2	38	T:0.947368	C:0.0526316			
chr28	10170663	2	38	C:0.184211	T:0.815789			
chr28	10170835	2	36	T:0.0833333	A:0.916667			
chr28	10170916	2	36	TA:0.194444	T:0.805556			
chr28	10171137	2	40	G:0.125	A:0.875			
chr28	10171237	2	36	C:0.944444	T:0.0555556			
chr28	10171367	2	40	G:0.2	GA:0.8			
chr28	10171752	2	34	T:0.0294118	C:0.970588			
chr28	10171820	2	34	T:0.0294118	TAAA:0.970588			
chr28	10171859	2	36	G:0.944444	A:0.0555556			
chr28	10171865	2	36	G:0.944444	A:0.0555556			
chr28	10171943	2	40	G:0.95	C:0.05			
chr28	10172177	2	36	C:0.972222	A:0.0277778			
chr28	10172284	2	34	CT:0.941176	C:0.0588235			
chr28	10172425	2	40	A:0.95	C:0.05			
chr28	10172799	2	36	C:0.888889	T:0.111111			
chr28	10173033	2	36	A:0.944444	G:0.0555556			
chr28	10173460	2	34	G:1	C:0			
chr28	10174465	2	34	C:0.117647	T:0.882353			
chr28	10174656	2	36	C:0.888889	T:0.111111			
chr28	10175018	2	30	TA:0.9	T:0.1			
chr28	10175020	2	30	A:0.966667	T:0.0333333			
chr28	10175416	2	34	G:0	C:1			
chr28	10175462	2	32	C:0.96875	T:0.03125			
chr28	10175676	2	36	GCAA:0.944444	G:0.0555556			
chr28	10175995	2	38	A:0.947368	G:0.0526316			
chr28	10176036	2	36	G:0.972222	A:0.0277778			
chr28	10176088	2	38	A:1	T:0			

chr28 10176155 2 40 T:0.95
TAAAATATTTTTGTTGAAAAAAAAAAGAAAAAA:0.05
chr28 10176451 2 36 C:0.944444 T:0.0555556
chr28 10176452 2 36 G:1 A:0
chr28 10176636 2 36 G:0.944444 A:0.0555556
chr28 10177037 2 38 C:0 CA:1
chr28 10177053 2 38 C:0 CA:1
chr28 10177172 2 34 G:0.941176 A:0.0588235
chr28 10177588 2 40 T:0.95 A:0.05
chr28 10177752 2 40 C:0.95 A:0.05
chr28 10177792 2 34 T:0.941176 C:0.0588235
chr28 10177951 2 38 G:0.921053 A:0.0789474
chr28 10177995 2 36 C:0.916667 T:0.0833333
chr28 10178073 2 32 G:0.09375 A:0.90625
chr28 10178127 2 34 T:0.941176 C:0.0588235
chr28 10178215 2 38 T:0.921053 TA:0.0789474
chr28 10178216 3 38 T:0.921053 TA:0 TATA:
0.0789474
chr28 10178259 2 38 G:0.921053 A:0.0789474
chr28 10178587 2 40 CTGGGGAGATGCTT:0.925 C:0.075
chr28 10178669 2 38 G:0.921053 A:0.0789474
chr28 10178780 2 40 CA:0.925 C:0.075
chr28 10178917 2 40 C:0.95 T:0.05
chr28 10178994 2 40 T:0.95 G:0.05
chr28 10179163 2 40 C:1 T:0
chr28 10179338 2 40 T:0.775 C:0.225
chr28 10179482 2 38 A:1 T:0
chr28 10179722 2 34 G:0.647059 GTT:0.352941
chr28 10179732 2 40 T:1 TG:0
chr28 10179736 2 40 T:0.975 TGTTTTTTG:0.025
chr28 10179777 2 36 G:0.972222 A:0.0277778
chr28 10179926 2 32 A:0.96875 T:0.03125
chr28 10180117 2 38 C:0.973684 T:0.0263158
chr28 10180121 2 38 T:0.973684 C:0.0263158
chr28 10180448 2 38 A:0.973684 AGAGAGAGAGAGC:
0.0263158
chr28 10180452 2 34 C:0.117647 T:0.882353
chr28 10180559 2 38 A:0.0526316 G:0.947368
chr28 10180810 2 32 T:0.96875 C:0.03125
chr28 10180918 2 30 TA:0.966667 T:0.0333333
chr28 10180948 2 36 T:0.972222 A:0.0277778
chr28 10181070 2 38 A:0.973684 AT:0.0263158
chr28 10181208 2 40 G:0.125 A:0.875
chr28 10181272 2 40 A:1 C:0
chr28 10181355 2 36 C:1 T:0
chr28 10181433 2 38 T:1 C:0
chr28 10181455 2 38 A:1 ATGCTGC:0
chr28 10181479 2 36 CTTCTGTTT:0.972222 C:
0.0277778
chr28 10181484 2 38 G:0.921053 GT:0.0789474
chr28 10181492 2 34 TTG:1 T:0
chr28 10181494 2 36 G:0.222222 T:0.777778
chr28 10181512 4 34 GTTT:0.147059 G:0.0294118
GTTTT:0 TTTT:0.823529

chr28	10182245	2	40	T:1	C:0		
chr28	10182413	2	36	G:0.972222	T:0.0277778		
chr28	10182486	2	38	T:1	C:0		
chr28	10182646	2	38	G:0.973684	A:0.0263158		
chr28	10182848	4	36	CT:0.138889	C:0.0277778	CTT:0.694444	CTTT:0.138889
chr28	10183221	2	38	G:0.973684	A:0.0263158		
chr28	10183345	2	38	TAGTC:0.973684	T:0.0263158		
chr28	10183420	2	36	T:0.972222	C:0.0277778		
chr28	10183703	2	40	G:0.975	GTTTTTTTTT:0.025		
chr28	10184034	2	28	T:1	TAA:0		
chr28	10184066	2	30	C:0.966667	CT:0.0333333		
chr28	10184150	2	36	A:0.444444	T:0.555556		
chr28	10184152	2	36	ATTTATTT:0.138889	A:0.861111		
chr28	10184489	2	36	A:0.111111	G:0.888889		
chr28	10184650	2	40	CCAAACTTTCTTTTTCTTTTTTTTTTTTTTTTTTTTTTTTTTTTTTTTTT:0	C:1		
chr28	10184933	2	38	T:1	C:0		
chr28	10185046	2	40	TA:0.925	T:0.075		
chr28	10185574	2	34	C:1	T:0		
chr28	10185602	2	38	T:1	A:0		
chr28	10185825	2	34	G:1	A:0		
chr28	10186039	2	32	A:0.90625	AT:0.09375		
chr28	10186510	2	40	G:0.85	A:0.15		
chr28	10187094	2	38	TA:0.131579	T:0.868421		
chr28	10187445	2	36	AAAC:0.916667	A:0.0833333		
chr28	10187515	2	36	G:0.833333	A:0.166667		
chr28	10187527	2	36	G:0.194444	A:0.805556		
chr28	10187572	2	34	C:0.941176	A:0.0588235		
chr28	10187573	2	34	G:0.147059	A:0.852941		
chr28	10187947	2	40	G:0.875	GAAAACATGTCCACACAAAAATGTGTTTAC:0.125		
chr28	10188244	2	36	TC:1	T:0		
chr28	10188277	2	36	C:0.833333	G:0.166667		
chr28	10188361	2	40	T:1	TA:0		
chr28	10188362	2	40	T:0.725	A:0.275		
chr28	10188490	2	36	C:0.888889	T:0.111111		
chr28	10189517	2	28	TC:0.285714	T:0.714286		
chr28	10189654	2	34	G:0.205882	C:0.794118		
chr28	10189689	2	32	C:0.1875	T:0.8125		
chr28	10189692	2	32	T:0.90625	C:0.09375		
chr28	10189913	2	32	A:0.125	G:0.875		
chr28	10190601	2	36	G:1	A:0		
chr28	10190834	2	38	T:0.0526316	A:0.947368		
chr28	10191435	2	38	C:0.842105	G:0.157895		
chr28	10191496	2	36	A:0.888889	G:0.111111		
chr28	10191911	2	34	TA:0.205882	T:0.794118		
chr28	10191933	2	34	G:0.205882	A:0.794118		
chr28	10192231	2	36	C:1	T:0		
chr28	10192255	2	36	C:0.916667	T:0.0833333		
chr28	10192561	2	34	A:0.970588	T:0.0294118		
chr28	10192640	2	34	T:0.117647	A:0.882353		
chr28	10192664	2	32	C:0.03125	G:0.96875		

chr28	10192898	2	22	G:0.227273	C:0.772727		
chr28	10194221	2	28	T:0.214286	C:0.785714		
chr28	10194540	3	28	G:1	GTGC:0	GTGCTGC:0	
chr28	10194740	2	30	A:0.1	G:0.9		
chr28	10195054	2	34	CTT:1	C:0		
chr28	10195593	2	38	GTATT:0.684211	G:0.315789		
chr28	10195621	3	38	GT:0.289474	G:0	GTTTTT:0.710526	
chr28	10196362	2	38	A:0.921053	G:0.0789474		
chr28	10196855	2	30	CTT:1	C:0		
chr28	10196857	4	40	TTTTTC:0.725	T:0	TTTCTTTTC:0.275	TTTTTCTTTTCTTTTCTTTTC:0
chr28	10196894	2	40	T:1	TTTCTTTTTTCTTTTC:0		
chr28	10196944	2	40	C:1	CCTTT:0		
chr28	10196965	2	40	CTTTCTTT:1	C:0		
chr28	10196972	2	40	T:0.8	TC:0.2		
chr28	10196985	2	40	C:0.575	T:0.425		
chr28	10198571	2	36	A:0.555556	AAATT:0.444444		
chr28	10198572	2	38	A:1	AATT:0		
chr28	10198658	4	38	C:0.605263	CATAA:0.315789	CATAAATAA:0.0789474	CATAAATAAATAA:0
chr28	10199197	2	36	C:0.472222	T:0.527778		
chr28	10199651	2	40	C:0.575	CT:0.425		
chr28	10199887	2	38	TTTTA:1	T:0		
chr28	10201240	2	28	CATTT:0	C:1		
chr28	10201918	2	38	C:0.973684	T:0.0263158		
chr28	10202452	2	36	A:0	G:1		
chr28	10204117	2	36	T:0	A:1		
chr28	10204662	2	38	T:0.921053	A:0.0789474		
chr28	10204694	2	32	C:1	CAG:0		
chr28	10204858	2	38	T:0.921053	C:0.0789474		
chr28	10204975	2	40	A:0.9	T:0.1		
chr28	10206008	2	32	C:1	T:0		
chr28	10206285	3	38	TA:0.763158	T:0.210526	TAA:0.0263158	
chr28	10206466	2	36	C:0	T:1		
chr28	10206866	2	24	C:0.333333	CA:0.666667		
chr28	10206960	2	34	A:0.117647	AT:0.882353		
chr28	10209139	2	24	C:0.875	CT:0.125		
chr28	10209599	2	36	C:0.666667	T:0.333333		
chr28	10209824	2	36	T:0.944444	C:0.0555556		
chr28	10210027	2	34	T:0.764706	TA:0.235294		
chr28	10210545	2	36	G:0.0833333	C:0.916667		
chr28	10211524	2	32	CAG:1	C:0		
chr28	10211592	2	34	C:1	T:0		
chr28	10211945	2	38	G:0.947368	A:0.0526316		
chr28	10213084	2	38	A:0.921053	G:0.0789474		
chr28	10214082	2	34	C:1	T:0		
chr28	10215146	2	36	TAA:1	T:0		
chr28	10215148	2	36	A:1	ATTTTTTTTTTTTT:0		
chr28	10215235	2	30	A:0.0666667	G:0.933333		
chr28	10215560	2	24	A:1	C:0		
chr28	10215797	2	28	CT:0.928571	C:0.0714286		
chr28	10216136	2	38	T:0.0789474	G:0.921053		

chr28	10216903	2	36	TTTTA:1	T:0	
chr28	10217303	2	36	C:0.888889		G:0.111111
chr28	10217443	2	38	T:1	G:0	
chr28	10217656	2	34	C:0.0294118		T:0.970588
chr28	10217667	2	34	A:0	T:1	
chr28	10217670	2	34	A:0	T:1	
chr28	10217684	2	30	A:0	G:1	
chr28	10217713	2	34	G:0	C:1	
chr28	10217717	2	34	G:0	C:1	
chr28	10217725	2	34	G:0	A:1	
chr28	10217727	2	34	T:0	C:1	
chr28	10217732	2	34	TG:0	T:1	
chr28	10217750	2	36	G:0	T:1	
chr28	10217757	2	36	A:0	G:1	
chr28	10217769	2	38	C:0	CA:1	
chr28	10217772	2	38	G:0	T:1	
chr28	10217773	2	38	AT:0	A:1	
chr28	10217776	2	38	T:0	C:1	
chr28	10217785	2	38	G:0	GC:1	
chr28	10217791	2	38	TCTAG:0	T:1	
chr28	10217796	2	38	A:0	ATC:1	
chr28	10217805	2	38	A:0	T:1	
chr28	10217814	2	38	A:0	C:1	
chr28	10217815	2	38	C:0	A:1	
chr28	10217816	2	38	T:0	G:1	
chr28	10217887	2	36	G:0	A:1	
chr28	10219877	2	36	A:0.0555556		G:0.944444
chr28	10220208	2	34	G:0	GA:1	
chr28	10220401	2	38	T:0.0789474		TC:0.921053
chr28	10220414	2	38	A:0.973684		T:0.0263158
chr28	10220416	2	38	A:0.0526316		T:0.947368
chr28	10220438	2	34	G:0.941176		GT:0.0588235
chr28	10220523	2	40	G:0.925	A:0.075	
chr28	10220530	2	40	G:1	A:0	
chr28	10220819	2	38	A:1	T:0	
chr28	10220844	2	30	T:0.133333		C:0.866667
chr28	10221024	2	36	A:0	G:1	
chr28	10221026	2	36	A:0	G:1	
chr28	10221031	2	36	AG:0	A:1	
chr28	10221051	2	36	G:0	A:1	
chr28	10221065	2	36	A:0	ATATT:1	
chr28	10221066	2	36	AAGGG:0	A:1	
chr28	10221075	2	36	T:0	A:1	
chr28	10221092	2	36	GAA:0	G:1	
chr28	10221094	2	36	A:0	ATTT:1	
chr28	10221096	2	36	C:0	CT:1	
chr28	10221102	2	36	T:0	TA:1	
chr28	10221130	2	36	G:0	T:1	
chr28	10221169	2	34	A:0	T:1	
chr28	10221188	2	34	G:0	T:1	
chr28	10221251	2	34	C:0.970588		CCT:0.0294118
chr28	10221651	2	34	C:1	CT:0	
chr28	10221784	2	38	TTTCTTTC:0.894737		T: 0.105263

chr28	10221870	2	32	CTCTTTCTTTCTTTCTTTCTTTCTT:0.875
C:0.125
chr28	10222097	2	40
TTCTTTCTTTCTTTCTTCCTTTCTCTCTCTCTCTC:0.075	T:0.925
chr28	10225079	2	36	TCCC:0.916667	T:0.0833333
chr28	10225082	2	36	C:0.0833333	T:0.916667
chr28	10225086	2	30	A:1	ATTC:0
chr28	10225154	2	36	G:1	A:0
chr28	10226261	4	38	CAG:0.447368	C:0.0789474
CAGAG:0.289474	CAGAGAG:0.184211
chr28	10226865	2	40	A:1	C:0
chr28	10227301	2	40	A:0.925	G:0.075
chr28	10227734	3	24	C:0.291667	CT:0.5	CTT:
0.208333
chr28	10227822	2	32	AT:0.9375	A:0.0625
chr28	10228493	2	38	TAAA:1	T:0
chr28	10228498	2	38	A:1	T:0
chr28	10228609	2	36	C:0.944444	T:0.0555556
chr28	10229110	2	38	GA:0.842105	G:0.157895
chr28	10229543	2	36	G:0.694444	GT:0.305556
chr28	10229764	2	38	AT:0.0789474	A:0.921053
chr28	10229828	2	34	C:0.205882	CT:0.794118
chr28	10230082	2	34	C:0.970588	A:0.0294118
chr28	10231615	2	40	G:1	C:0
chr28	10231666	5	40	TTATCTATCTATCTATC:0.3	T:0.175
TTATC:0.1	TTATCTATCTATC:0.15	TTATCTATCTATCTATCTATC:
0.275
chr28	10232630	2	36	T:0	A:1
chr28	10232644	2	36	ATTT:0	A:1
chr28	10232649	2	36	T:0	TGG:1
chr28	10232651	2	36	A:0	G:1
chr28	10232653	2	36	GC:0	G:1
chr28	10232658	2	36	A:0	G:1
chr28	10232662	2	36	C:0	T:1
chr28	10232681	2	36	C:0	A:1
chr28	10232696	2	36	G:0	T:1
chr28	10232705	2	36	C:0	A:1
chr28	10232731	2	38	A:0	T:1
chr28	10232738	2	38	T:0	A:1
chr28	10232760	2	38	C:0	A:1
chr28	10232886	2	38	GT:0.868421	G:0.131579
chr28	10233249	2	36	T:0.916667	A:0.0833333
chr28	10233584	2	36	CT:0.0833333	C:0.916667
chr28	10233628	3	28	A:0.285714	AAG:0.642857
AAGAG:0.0714286
chr28	10234058	2	34	T:0.941176	A:0.0588235
chr28	10234981	2	38	TA:1	T:0
chr28	10236023	2	38	CTGATTGAT:0.973684	C:
0.0263158
chr28	10236871	2	34	C:0.941176	A:0.0588235
chr28	10237348	3	38	T:0.236842	TTTTTA:0.710526
TTTTTATTTTA:0.0526316
chr28	10237407	3	36	CAG:1	C:0	CAGAG:0
chr28	10237409	2	32	G:0	C:1

chr28	10237514	2	34	G:0.0882353	C:0.911765	
chr28	10237537	2	34	G:0.0882353	A:0.911765	
chr28	10239051	2	38	AGT:1	A:0	
chr28	10239081	2	34	T:0.0882353	A:0.911765	
chr28	10239174	2	34	T:0.0882353	A:0.911765	
chr28	10241743	2	38	C:0.894737	T:0.105263	
chr28	10241847	2	40	A:0.95	T:0.05	
chr28	10241959	2	38	C:0.657895	T:0.342105	
chr28	10242101	2	38	A:0.0789474	C:0.921053	
chr28	10242211	2	38	AT:0.131579	A:0.868421	
chr28	10243424	2	40	T:1	C:0	
chr28	10243517	2	40	A:0.925	AAAAAC:0.075	
chr28	10243960	2	38	C:0.921053	CCT:0.0789474	
chr28	10244078	2	34	T:0.911765	G:0.0882353	
chr28	10244190	2	36	C:0.916667	A:0.0833333	
chr28	10244813	2	38	A:0.0789474	G:0.921053	
chr28	10245114	3	30	G:0.9	GT:0.0666667	GTT:0.0333333
chr28	10245632	2	34	C:1	T:0	
chr28	10246538	2	36	C:0.916667	CT:0.0833333	
chr28	10246807	3	30	CTT:0.566667	C:0.133333	CTTT:0.3
chr28	10247331	2	30	TA:0.133333	T:0.866667	
chr28	10247387	2	28	TA:1	T:0	
chr28	10247592	2	24	G:0.5	GT:0.5	
chr28	10247617	2	28	C:0.321429	CT:0.678571	
chr28	10247886	2	36	T:1	C:0	
chr28	10248273	2	34	AC:1	A:0	
chr28	10248337	2	38	T:1	TGAGA:0	
chr28	10250140	2	36	A:0.111111	G:0.888889	
chr28	10250481	2	36	CT:0.805556	C:0.194444	
chr28	10250920	2	36	T:0.0833333	C:0.916667	
chr28	10251016	2	40	T:1	G:0	
chr28	10252020	2	40	G:0.95	A:0.05	
chr28	10254917	2	26	TCA:1	T:0	
chr28	10254924	2	26	C:0.884615	G:0.115385	
chr28	10255526	2	28	G:0	C:1	
chr28	10255597	2	34	C:0	A:1	
chr28	10255637	2	36	G:0	T:1	
chr28	10255654	2	38	T:0	A:1	
chr28	10255824	2	26	CCT:0.269231	C:0.730769	
chr28	10256382	2	36	C:0.0833333	T:0.916667	
chr28	10256928	2	36	C:1	CA:0	
chr28	10257348	2	28	G:0	A:1	
chr28	10257358	2	28	G:0	A:1	
chr28	10258105	2	36	CT:0.805556	C:0.194444	
chr28	10258193	2	30	A:0	G:1	
chr28	10259148	2	26	C:0.961538	CAG:0.0384615	
chr28	10259732	2	34	T:1	TA:0	
chr28	10259775	3	36	ATTTTAT:0.25	A:0.722222	ATTTTATTTTTAT:0.0277778
chr28	10259839	2	28	C:0.785714	CAG:0.214286	
chr28	10261339	2	36	T:1	A:0	
chr28	10261340	2	36	T:0.972222	A:0.0277778	

chr28	10261358	2	32	T:0.9375	A:0.0625	
chr28	10263091	2	40	T:0	A:1	
chr28	10263112	2	40	AGACTGGGAG:0	A:1	
chr28	10263138	2	32	AAAACCAAAAAAAAC:0.0625	A:0.9375	
chr28	10263139	2	32	AAACCAAAAAAAAC:1	A:0	
chr28	10263157	2	40	T:0	A:1	
chr28	10263164	2	40	C:0	A:1	
chr28	10263165	2	40	C:0	T:1	
chr28	10263184	2	40	C:0	T:1	
chr28	10263187	2	40	G:0	T:1	
chr28	10263570	2	40	CCTTTTCCTTTTCCTTTTCCTTTTTCTTTTCTTTCTTTT:0.9	C:0.1	
chr28	10263584	2	40	TTTTCCTTTTTCTTTTCTTTC:1	T:0	
chr28	10263600	2	40	CTTT:1	C:0	
chr28	10263781	2	36	GCCCCCCC:0.0833333	G:0.916667	
chr28	10263940	2	32	G:0.1875	GTTTT:0.8125	
chr28	10264356	2	32	CT:0.84375	C:0.15625	
chr28	10264459	2	32	T:0.9375	A:0.0625	
chr28	10264992	2	38	C:1	G:0	
chr28	10265466	2	36	T:0.888889	A:0.111111	
chr28	10265470	2	36	G:0.0833333	T:0.916667	
chr28	10265967	2	38	AT:0.0789474	A:0.921053	
chr28	10265975	2	38	T:0.0789474	A:0.921053	
chr28	10266146	2	34	G:1	GT:0	
chr28	10267018	2	36	T:0.0833333	C:0.916667	
chr28	10267050	2	38	T:1	A:0	
chr28	10267748	2	38	T:0.552632	A:0.447368	
chr28	10269982	3	36	A:0	T:0.888889	ATT:0.111111
chr28	10269988	2	36	T:1	TTA:0	
chr28	10271879	2	38	C:0.0789474	CT:0.921053	
chr28	10272031	2	34	C:0.970588	CT:0.0294118	
chr28	10272409	2	32	C:0.15625	CT:0.84375	
chr28	10272452	2	34	C:0.970588	CAG:0.0294118	
chr28	10273279	2	34	C:0.911765	T:0.0882353	
chr28	10273590	2	40	A:0	T:1	
chr28	10273628	2	40	G:0	T:1	
chr28	10273855	2	34	C:1	T:0	
chr28	10274988	2	38	C:0.0789474	CT:0.921053	
chr28	10275285	2	38	G:0.0526316	A:0.947368	
chr28	10275539	2	34	A:0.970588	ATATG:0.0294118	
chr28	10275577	2	40	G:1	A:0	
chr28	10275764	2	34	G:0.911765	A:0.0882353	
chr28	10275837	2	36	T:0.888889	A:0.111111	
chr28	10277251	2	36	C:0.111111	A:0.888889	
chr28	10277361	2	38	T:0.210526	TTTTTG:0.789474	
chr28	10277457	2	38	CTG:0.210526	C:0.789474	
chr28	10277913	2	40	A:0.975	G:0.025	
chr28	10278194	2	34	T:0	A:1	
chr28	10278201	2	34	C:0	G:1	
chr28	10278206	2	34	G:0	T:1	
chr28	10278208	2	34	C:0	T:1	
chr28	10278214	2	34	TA:0	T:1	

chr28	10278235	2	36	C:0	A:1
chr28	10278245	2	36	T:0	A:1
chr28	10278248	2	36	C:0	T:1
chr28	10278252	2	36	T:0	A:1
chr28	10278278	2	36	G:0	T:1
chr28	10278281	2	36	A:0	T:1
chr28	10278287	2	36	G:0	T:1
chr28	10278347	2	38	G:0	T:1
chr28	10278366	2	38	T:0	TG:1
chr28	10278374	2	38	GA:0	G:1
chr28	10278377	2	38	A:0	T:1
chr28	10278380	2	38	C:0	T:1
chr28	10278383	2	38	C:0	T:1
chr28	10278384	2	38	G:0	GT:1
chr28	10278389	2	38	A:0	T:1
chr28	10278392	2	38	GA:0	G:1
chr28	10278414	2	36	G:0	T:1
chr28	10278426	2	38	A:0.105263	AG:0.894737
chr28	10278427	2	38	A:0.105263	AT:0.894737
chr28	10278429	2	38	C:0.105263	T:0.894737
chr28	10278431	2	38	T:0.105263	TA:0.894737
chr28	10278436	2	38	C:0.105263	CA:0.894737
chr28	10278862	5	38	T:0.894737	TGAGA:0.0526316
TGAGAGA:0.0526316	TGAGAGAGA:0	TGAGAGAGAGA:0
chr28	10280142	2	40	C:0.05	T:0.95
chr28	10282101	2	40	A:0.925	C:0.075
chr28	10283438	4	40	TATTC:0.4	T:0.125
TATTCATTC:0.225	TATTCATTCATTC:0.25
chr28	10283617	2	34	C:1	T:0
chr28	10283905	4	36	TCACACA:0.75	T:0.222222
TCA:0	TCACA:0.0277778
chr28	10284389	2	40	CT:0.9	C:0.1
chr28	10284402	2	40	T:0.8	A:0.2
chr28	10284429	2	36	C:0.861111	CAG:0.138889
chr28	10284649	2	36	T:0.972222	G:0.0277778
chr28	10286031	2	34	CT:0.794118	C:0.205882
chr28	10286311	2	30	T:1	TA:0
chr28	10286602	2	6	TG:1	T:0
chr28	10286866	2	36	A:1	AT:0
chr28	10288118	2	40	C:0.925	A:0.075
chr28	10289368	2	40	AAATG:0.1	A:0.9
chr28	10290222	2	40	C:0.925	T:0.075
chr28	10291300	2	38	T:1	A:0
chr28	10291599	2	40	T:0.925	G:0.075
chr28	10292188	2	36	G:1	GC:0
chr28	10292196	2	36	C:1	A:0
chr28	10293113	2	34	A:0.294118	AC:0.705882
chr28	10293942	2	38	A:1	G:0
chr28	10293975	2	38	CTG:0.947368	C:0.0526316
chr28	10295027	2	38	G:1	T:0
chr28	10296691	2	38	C:0.921053	A:0.0789474
chr28	10297215	2	34	A:0.823529	AT:0.176471
chr28	10297228	2	32	A:0.375	AT:0.625
chr28	10297234	2	34	T:0.823529	TA:0.176471

chr28	10297242	2	34	A:0.794118	T:0.205882	
chr28	10297584	2	38	T:1	G:0	
chr28	10298321	2	36	T:0.916667	G:0.0833333	
chr28	10299018	2	38	A:0.921053	C:0.0789474	
chr28	10299242	2	36	G:0.972222	GTGATGA:0.0277778	
chr28	10299389	2	28	AG:1	A:0	
chr28	10299571	2	38	CT:0.921053	C:0.0789474	
chr28	10299671	2	36	G:0.0833333	A:0.916667	
chr28	10300921	2	38	T:0.815789	C:0.184211	
chr28	10303275	2	38	T:1	TAATAATTAAATA:0	
chr28	10303278	2	36	TA:1	T:0	
chr28	10303281	2	32	A:0.96875	T:0.03125	
chr28	10303282	2	36	TAAA:1	T:0	
chr28	10303322	2	36	T:0.5	TAAATAAA:0.5	
chr28	10305770	2	38	GT:0.921053	G:0.0789474	
chr28	10306110	2	40	G:1	A:0	
chr28	10306667	3	36	G:0.888889	GT:0.0277778	GTT:0.0833333
chr28	10308168	2	32	CTTTG:0.90625	C:0.09375	
chr28	10308216	2	28	TA:0.642857	T:0.357143	
chr28	10308710	2	34	T:0.882353	C:0.117647	
chr28	10309124	2	40	T:1	TCTTCTCCTTCTC:0	
chr28	10309226	2	36	T:1	A:0	
chr28	10309300	2	38	G:0.105263	A:0.894737	
chr28	10309586	2	36	T:0.888889	TTG:0.111111	
chr28	10309762	2	38	C:0.973684	T:0.0263158	
chr28	10314496	2	34	AG:0.911765	A:0.0882353	
chr28	10318186	2	32	T:0.90625	G:0.09375	
chr28	10320740	2	40	C:0.95	T:0.05	
chr28	10322045	2	38	T:0.684211	A:0.315789	
chr28	10322857	2	36	G:0.972222	A:0.0277778	
chr28	10323774	2	40	A:0.95	G:0.05	
chr28	10324368	2	36	TAC:0.861111	T:0.138889	
chr28	10326115	2	36	A:0.0833333	T:0.916667	
chr28	10326851	2	30	T:0.933333	TA:0.0666667	
chr28	10327782	2	38	TA:0.0789474	T:0.921053	
chr28	10328178	2	38	AG:0.789474	A:0.210526	
chr28	10328491	2	34	C:0.970588	CCTCT:0.0294118	
chr28	10329049	3	36	TA:0.416667	T:0	TAA: 0.583333
chr28	10330111	2	36	G:0.916667	A:0.0833333	
chr28	10330709	2	26	G:0	T:1	
chr28	10330904	2	32	TA:0.09375	T:0.90625	
chr28	10331042	2	38	A:1	T:0	
chr28	10331876	2	38	G:0.842105	A:0.157895	
chr28	10333265	2	36	C:0.0833333	T:0.916667	
chr28	10333520	2	34	G:0.0882353	A:0.911765	
chr28	10335857	2	38	G:1	A:0	
chr28	10336123	2	40	CTTTG:0.225	C:0.775	
chr28	10336126	2	40	T:0.8	G:0.2	
chr28	10336127	2	40	G:0.8	T:0.2	
chr28	10337878	2	38	TTAGCTGCATG:0.894737	T: 0.105263	
chr28	10338084	2	38	A:0.210526	AT:0.789474	

chr28	10338969	2	40	A:0.175	G:0.825		
chr28	10342121	2	38	C:1	G:0		
chr28	10345207	2	36	G:0.833333	T:0.166667		
chr28	10346971	2	32	A:0.21875	AT:0.78125		
chr28	10346982	2	36	AT:1	A:0		
chr28	10346991	2	36	T:1	A:0		
chr28	10347370	2	38	G:1	T:0		
chr28	10347881	2	34	T:0.147059	C:0.852941		
chr28	10348188	2	34	G:0.882353	A:0.117647		
chr28	10348754	2	38	T:0	C:1		
chr28	10350541	2	38	C:1	T:0		
chr28	10352784	2	36	T:0.166667	G:0.833333		
chr28	10353339	2	24	C:0.25	T:0.75		
chr28	10353730	2	36	C:1	A:0		
chr28	10353892	2	32	C:0.96875	T:0.03125		
chr28	10354223	2	34	T:0.294118	C:0.705882		
chr28	10354372	2	34	G:0.205882	GC:0.794118		
chr28	10354374	2	34	G:0.205882	T:0.794118		
chr28	10354908	2	38	C:1	T:0		
chr28	10355356	2	34	C:0.823529	CA:0.176471		
chr28	10355921	2	38	A:0.789474	G:0.210526		
chr28	10356475	2	28	TG:0.75	T:0.25		
chr28	10357042	2	34	T:0.264706	C:0.735294		
chr28	10358217	2	24	TA:0.833333	T:0.166667		
chr28	10358632	2	38	C:0.815789	A:0.184211		
chr28	10358988	2	36	G:1	A:0		
chr28	10359688	2	40	C:1	T:0		
chr28	10360705	2	36	T:0	A:1		
chr28	10360707	2	36	A:0	AT:1		
chr28	10360711	2	36	G:0	T:1		
chr28	10360718	2	36	A:0	AT:1		
chr28	10360739	2	34	T:0	G:1		
chr28	10360895	2	38	A:0.868421	G:0.131579		
chr28	10361065	2	36	G:0.75	A:0.25		
chr28	10361098	2	34	T:0.794118	C:0.205882		
chr28	10362366	2	36	C:0.805556	G:0.194444		
chr28	10363075	3	36	ATG:1	A:0	ATGTG:0	
chr28	10363085	2	36	G:0.972222	GTA:0.0277778		
chr28	10363087	4	36	G:0.805556	A:0.0277778	GTA:0.0277778	GTATA:0.138889
chr28	10363501	2	38	C:1	T:0		
chr28	10363642	2	34	G:0.794118	A:0.205882		
chr28	10363714	2	38	G:0.263158	C:0.736842		
chr28	10363864	2	30	T:0.866667	C:0.133333		
chr28	10364030	2	38	G:0.131579	C:0.868421		
chr28	10364043	2	38	G:0.894737	A:0.105263		
chr28	10365147	2	30	G:0.133333	A:0.866667		
chr28	10365157	2	30	A:0.966667	C:0.0333333		
chr28	10365235	2	36	G:0.194444	GAACGCTGCAGCCC:0.805556		
chr28	10365596	2	36	G:0.25	A:0.75		
chr28	10365841	2	36	CGG:0.861111	C:0.138889		
chr28	10365890	2	34	T:0.823529	C:0.176471		
chr28	10366396	2	38	C:0.657895	T:0.342105		

chr28	10366452	2	38	C:0.631579	T:0.368421
chr28	10366773	2	32	G:1	A:0
chr28	10366873	2	32	G:0.71875	A:0.28125
chr28	10367446	2	40	CCG:0.425	C:0.575
chr28	10367453	2	40	C:0.425	CACACA:0.575
chr28	10367644	2	32	C:0.25	T:0.75
chr28	10367688	2	34	G:0	C:1
chr28	10367732	2	34	G:0.823529	T:0.176471
chr28	10368120	2	36	T:0.722222	C:0.277778
chr28	10369407	2	30	A:1	AGT:0
chr28	10370816	2	36	G:0	A:1
chr28	10370817	2	36	A:0	T:1
chr28	10370858	2	36	T:0	C:1
chr28	10370865	2	36	C:0	G:1
chr28	10370866	2	36	TCCCC:0	T:1
chr28	10370872	2	36	C:0	CTATG:1
chr28	10370927	2	30	T:0	TA:1
chr28	10374078	2	40	C:0.1	T:0.9
chr28	10377572	2	32	C:1	T:0
chr28	10378750	2	28	A:1	G:0
chr28	10378769	2	34	G:1	A:0
chr28	10379163	2	28	C:1	T:0
chr28	10379774	2	34	G:1	C:0
chr28	10379827	2	34	T:1	C:0
chr28	10379987	2	36	A:1	G:0
chr28	10380811	2	40	C:1	A:0
chr28	10381643	2	30	A:1	G:0
chr28	10381703	2	26	C:0.961538	T:0.0384615
chr28	10381792	2	30	G:1	A:0
chr28	10381872	2	38	TCC:0.815789	T:0.184211
chr28	10381875	2	38	TCTCCTCTCCTCTCCTCCCCTCCC:0.815789	T:0.184211
chr28	10382353	2	36	C:1	G:0
chr28	10382580	2	38	C:0.263158	T:0.736842
chr28	10383052	2	38	A:1	T:0
chr28	10383887	2	34	A:1	G:0
chr28	10384028	2	38	A:1	G:0
chr28	10384349	2	38	C:1	T:0
chr28	10384687	2	38	T:1	C:0
chr28	10385385	2	36	G:0	C:1
chr28	10385825	2	38	G:1	A:0
chr28	10385939	2	40	A:1	G:0
chr28	10385971	2	34	G:1	GA:0
chr28	10385974	2	34	G:1	A:0
chr28	10386297	2	36	T:1	C:0
chr28	10386315	2	36	G:1	C:0
chr28	10386328	2	32	A:1	G:0
chr28	10386501	2	32	G:1	GA:0
chr28	10386609	2	34	A:1	T:0
chr28	10386908	2	36	C:1	T:0
chr28	10386918	2	36	T:1	A:0
chr28	10387323	2	40	C:1	T:0
chr28	10387401	2	36	G:1	A:0
chr28	10387594	2	34	T:1	C:0

chr28	10387806	2	22	A:1	G:0	
chr28	10388313	2	32	T:1	C:0	
chr28	10388466	2	40	TG:1	T:0	
chr28	10388628	2	38	T:1	TA:0	
chr28	10388841	2	34	A:1	G:0	
chr28	10389161	2	24	C:1	T:0	
chr28	10389928	2	34	A:1	ATTTCATT:0	
chr28	10390280	2	36	C:1	T:0	
chr28	10390562	2	36	A:1	C:0	
chr28	10390574	2	38	T:1	C:0	
chr28	10391260	2	36	CA:0	C:1	
chr28	10391476	2	36	G:1	C:0	
chr28	10391557	2	38	C:1	T:0	
chr28	10391721	2	32	G:1	A:0	
chr28	10391895	2	34	C:1	T:0	
chr28	10392035	2	34	C:1	T:0	
chr28	10392085	2	38	G:0.631579		T:0.368421
chr28	10392319	2	36	CG:0	C:1	
chr28	10392365	2	34	G:1	A:0	
chr28	10392446	2	40	T:1	C:0	
chr28	10392796	2	26	T:1	C:0	
chr28	10392902	2	32	A:1	G:0	
chr28	10393550	2	32	C:1	G:0	
chr28	10393652	2	32	G:1	GC:0	
chr28	10393861	2	36	C:1	T:0	
chr28	10394024	2	32	T:1	C:0	
chr28	10394271	2	36	AT:1	A:0	
chr28	10394288	2	36	C:0.972222		A:0.0277778
chr28	10394868	2	38	G:1	A:0	
chr28	10394921	2	36	G:1	T:0	
chr28	10395152	2	36	G:1	A:0	
chr28	10395369	2	34	G:1	C:0	
chr28	10395401	2	32	T:1	TA:0	
chr28	10395424	2	32	C:1	CA:0	
chr28	10395599	2	38	G:1	T:0	
chr28	10395865	2	38	A:0	T:1	
chr28	10395870	2	36	C:1	T:0	
chr28	10395873	2	38	C:0	A:1	
chr28	10395874	2	38	A:0	T:1	
chr28	10395884	2	36	G:1	A:0	
chr28	10396284	3	34	A:0.323529		AG:0.0294118
AAGAG:0.647059						
chr28	10396416	2	30	G:1	C:0	
chr28	10396706	2	28	C:0	A:1	
chr28	10396711	2	32	T:1	A:0	
chr28	10396728	2	32	A:0.96875		T:0.03125
chr28	10397205	2	36	T:1	C:0	
chr28	10397433	2	38	T:1	C:0	
chr28	10397476	2	38	T:1	G:0	
chr28	10397487	2	36	G:1	A:0	
chr28	10397646	2	34	GC:1	G:0	
chr28	10397753	2	38	A:1	C:0	
chr28	10398029	2	40	C:1	T:0	
chr28	10398460	2	36	T:1	C:0	

chr28	10398873	2	34	C:1	T:0
chr28	10399254	2	36	G:1	A:0
chr28	10399272	2	40	A:1	AGGAGGAGCAGG:0
chr28	10399274	2	40	CCA:1	C:0
chr28	10399850	2	40	C:1	G:0
chr28	10400340	2	40	C:1	T:0
chr28	10400437	2	34	G:1	A:0
chr28	10400982	2	36	T:1	C:0
chr28	10401034	2	34	T:1	C:0
chr28	10401176	2	34	A:1	T:0
chr28	10401361	2	36	A:1	G:0
chr28	10401442	2	36	C:1	T:0
chr28	10401785	2	40	C:1	T:0
chr28	10401793	2	40	G:1	A:0
chr28	10402031	2	34	G:1	A:0
chr28	10402078	2	32	T:1	C:0
chr28	10402969	2	34	C:1	T:0
chr28	10403312	2	36	A:1	C:0
chr28	10403318	2	36	G:1	A:0
chr28	10403424	2	38	C:1	T:0
chr28	10403627	2	36	T:1	G:0
chr28	10403866	2	38	T:0	C:1
chr28	10403925	2	36	A:1	G:0
chr28	10404228	2	34	A:1	G:0
chr28	10404455	2	26	G:0	GT:1
chr28	10404475	2	26	T:0	TA:1
chr28	10404477	2	26	GA:0	G:1
chr28	10404483	2	26	AC:0	A:1
chr28	10404523	2	32	C:0.40625	CT:0.59375
chr28	10404526	2	32	A:0.40625	G:0.59375
chr28	10404528	2	32	A:0.40625	G:0.59375
chr28	10404530	2	32	CG:0.40625	C:0.59375
chr28	10404532	2	32	CG:0.40625	C:0.59375
chr28	10404536	2	32	C:0.40625	A:0.59375
chr28	10404538	2	32	C:0.40625	A:0.59375
chr28	10404550	2	32	CCCCCCCCCCCTCCCG:0.40625	C:0.59375
chr28	10404948	2	40	T:1	C:0
chr28	10405303	2	40	T:1	C:0
chr28	10405445	2	36	G:0.555556	A:0.444444
chr28	10405479	2	36	T:0	TG:1
chr28	10405525	2	36	G:0	GT:1
chr28	10405526	2	36	A:0	G:1
chr28	10405552	2	36	A:0	AT:1
chr28	10405745	2	34	T:1	C:0
chr28	10405770	2	36	G:1	A:0
chr28	10405847	2	36	G:1	A:0
chr28	10406752	2	34	C:0.882353	T:0.117647
chr28	10407069	2	40	G:1	A:0
chr28	10407082	2	40	G:1	A:0
chr28	10407326	2	38	A:0.973684	AT:0.0263158
chr28	10407690	2	38	T:1	C:0
chr28	10407941	2	40	G:1	A:0
chr28	10407944	2	40	A:1	G:0

chr28	10408200	2	40	T:1	C:0	
chr28	10408224	2	40	G:1	A:0	
chr28	10408225	2	40	C:0.975	A:0.025	
chr28	10408264	2	40	A:0.95	C:0.05	
chr28	10408297	2	38	G:1	A:0	
chr28	10408306	2	38	C:1	T:0	
chr28	10409581	2	38	C:1	T:0	
chr28	10409893	3	30	CCTCT:0.8	C:0.0333333	CCT:0.166667
chr28	10410057	2	38	G:1	A:0	
chr28	10410061	2	38	T:1	C:0	
chr28	10410483	2	36	T:1	C:0	
chr28	10410673	2	32	C:1	A:0	
chr28	10410775	2	38	GACT:0.973684	G:0.0263158	
chr28	10410995	2	36	A:1	T:0	
chr28	10411027	2	34	G:1	A:0	
chr28	10411592	2	36	C:0.944444	CT:0.0555556	
chr28	10411745	2	32	C:1	T:0	
chr28	10411760	2	32	A:1	C:0	
chr28	10411798	2	36	C:1	T:0	
chr28	10411853	2	36	T:1	A:0	
chr28	10412125	2	36	T:1	C:0	
chr28	10412330	2	36	A:1	G:0	
chr28	10412347	2	36	G:1	A:0	
chr28	10412552	2	40	C:0.975	T:0.025	
chr28	10412857	2	36	T:1	TC:0	
chr28	10413499	2	38	C:1	CG:0	
chr28	10413599	2	36	C:1	A:0	
chr28	10413685	2	38	A:1	G:0	
chr28	10414238	2	32	C:1	CT:0	
chr28	10414470	2	32	T:1	C:0	
chr28	10414517	2	34	G:1	A:0	
chr28	10414527	2	34	C:1	T:0	
chr28	10415601	2	34	AAAAAT:1	A:0	
chr28	10415621	2	36	TAAAATAAATA:0.972222	T:0.0277778	
chr28	10415626	2	38	T:0.894737	TAAATA:0.105263	
chr28	10417377	3	36	CGAGAGAGAGAGA:0.777778	C:0.222222	CGAGAGA:0
chr28	10417383	2	38	A:1	ATC:0	
chr28	10417945	2	34	G:1	A:0	
chr28	10418020	2	34	C:0.941176	A:0.0588235	
chr28	10418409	2	32	C:1	T:0	
chr28	10418813	3	38	CAT:0.736842	C:0	CATAT:0.263158
chr28	10418846	2	36	T:0.972222	A:0.0277778	
chr28	10418874	2	36	G:0.972222	GTGTATATA:0.0277778	
chr28	10419697	2	38	A:1	C:0	
chr28	10419784	2	36	T:0.888889	TA:0.111111	
chr28	10420261	2	40	T:1	C:0	
chr28	10420334	2	40	T:1	C:0	
chr28	10420397	2	40	G:1	T:0	
chr28	10420564	2	36	T:1	C:0	

chr28	10420766	2	38	A:1	G:0
chr28	10420781	2	38	A:1	G:0
chr28	10421296	2	38	T:1	G:0
chr28	10421541	2	38	T:1	C:0
chr28	10421665	2	36	A:1	G:0
chr28	10422136	2	38	T:1	TA:0
chr28	10423036	2	38	A:1	G:0
chr28	10423544	2	40	C:1	T:0
chr28	10423594	2	38	G:1	C:0
chr28	10423598	2	38	A:1	C:0
chr28	10423967	2	38	C:1	T:0
chr28	10424083	2	36	A:1	G:0
chr28	10424214	2	40	C:1	T:0
chr28	10424321	2	38	T:1	TG:0
chr28	10424506	2	40	C:1	T:0
chr28	10424645	2	40	A:1	C:0
chr28	10424808	2	40	G:1	A:0
chr28	10424814	2	40	A:1	G:0
chr28	10424852	2	38	C:1	CT:0
chr28	10424875	2	40	A:1	G:0
chr28	10425041	2	36	CGTGTGT:0.805556	C:0.194444
chr28	10425511	2	36	G:1	A:0
chr28	10425606	2	36	T:1	C:0
chr28	10425839	2	36	T:1	A:0
chr28	10425952	2	36	C:1	T:0
chr28	10427022	2	40	GC:1	G:0
chr28	10427176	2	34	G:1	A:0
chr28	10427759	2	34	G:1	A:0
chr28	10427979	2	38	C:1	T:0
chr28	10428209	2	38	C:1	G:0
chr28	10428504	2	38	A:1	G:0
chr28	10428822	2	28	AT:0.642857	A:0.357143
chr28	10428863	2	34	G:1	A:0
chr28	10429206	2	38	C:1	A:0
chr28	10429290	2	38	T:1	C:0
chr28	10429814	2	38	TC:1	T:0
chr28	10430037	2	34	C:1	T:0
chr28	10430172	2	36	C:1	T:0
chr28	10431042	2	30	C:0.933333	CT:0.0666667
chr28	10431233	2	32	G:1	A:0
chr28	10431502	2	38	T:1	C:0
chr28	10432230	2	28	G:1	T:0
chr28	10432245	2	30	G:1	A:0
chr28	10432448	2	36	T:1	A:0
chr28	10432673	2	36	C:0.972222	T:0.0277778
chr28	10433001	2	24	AGAGAGAGGAG:0.958333	A: 0.0416667
chr28	10433005	2	24	AGAGGAG:0.833333	A:0.166667
chr28	10433007	2	24	AGGAG:0.25	A:0.75
chr28	10434312	2	34	A:1	G:0
chr28	10436203	2	36	C:1	G:0
chr28	10436522	2	36	G:1	A:0
chr28	10437820	2	36	C:1	G:0
chr28	10438297	2	36	G:0.5	T:0.5

chr28	10438805	2	40	GCCAGGAC:1		G:0
chr28	10441394	2	34	G:1	A:0	
chr28	10441797	2	36	G:0.638889		C:0.361111
chr28	10441989	2	36	G:1	T:0	
chr28	10442087	2	38	T:1	A:0	
chr28	10442113	2	38	A:1	AG:0	
chr28	10442807	2	38	A:1	C:0	
chr28	10443124	2	34	C:1	CCT:0	
chr28	10443155	2	36	TA:1	T:0	
chr28	10443174	2	36	A:1	AAAATTT:0	
chr28	10443485	2	36	A:1	T:0	
chr28	10443497	2	36	TA:1	T:0	
chr28	10443670	2	34	T:1	TA:0	
chr28	10443950	2	34	T:0.941176		TAA:0.0588235
chr28	10443955	2	32	A:0.71875		AAT:0.28125
chr28	10444272	2	38	A:1	G:0	
chr28	10444470	2	36	A:1	T:0	
chr28	10444772	2	38	C:1	T:0	
chr28	10445301	2	28	C:1	T:0	
chr28	10445305	2	28	T:1	C:0	
chr28	10445326	2	26	C:1	G:0	
chr28	10445406	2	36	C:1	A:0	
chr28	10445550	2	36	C:1	A:0	
chr28	10445640	2	32	C:1	T:0	
chr28	10446428	2	34	C:1	T:0	
chr28	10446700	2	36	T:1	C:0	
chr28	10446807	2	34	T:1	C:0	
chr28	10446829	2	32	G:1	A:0	
chr28	10446843	2	32	G:1	A:0	
chr28	10447462	2	36	T:0.972222		C:0.0277778
chr28	10447683	2	34	C:1	T:0	
chr28	10447739	2	28	G:0.785714		GT:0.214286
chr28	10447794	2	36	C:0.944444		CAG:0.0555556
chr28	10448124	2	38	A:1	G:0	
chr28	10448364	2	38	A:1	G:0	
chr28	10449013	2	40	G:1	A:0	
chr28	10449165	2	34	T:1	C:0	
chr28	10449174	2	34	G:1	A:0	
chr28	10449975	2	36	C:1	T:0	
chr28	10449977	2	36	A:1	C:0	
chr28	10450144	2	32	A:1	T:0	
chr28	10450153	3	32	TA:0.75	T:0	TAA:0.25
chr28	10450155	2	32	A:1	T:0	
chr28	10451487	2	40	TCA:0.975		T:0.025
chr28	10451760	2	36	C:0.666667		T:0.333333
chr28	10453124	2	38	G:0.552632		T:0.447368
chr28	10453220	2	36	C:0.472222		T:0.527778
chr28	10453221	2	36	T:0.472222		G:0.527778
chr28	10453340	2	36	G:0.555556		A:0.444444
chr28	10453397	2	38	G:0.973684		A:0.0263158
chr28	10453654	2	34	T:0.470588		C:0.529412
chr28	10454031	2	38	C:0.5	T:0.5	
chr28	10454118	2	36	G:1	A:0	
chr28	10455483	2	34	G:1	A:0	

chr28	10455514	2	36	T:1	C:0	
chr28	10456454	2	40	T:0	C:1	
chr28	10456632	2	38	C:1	T:0	
chr28	10457104	2	38	AT:0	A:1	
chr28	10457244	2	38	A:1	T:0	
chr28	10457252	2	38	C:1	T:0	
chr28	10457765	2	36	T:1	G:0	
chr28	10458109	2	32	G:0.96875		A:0.03125
chr28	10458290	2	30	G:0	A:1	
chr28	10458604	2	36	A:0	G:1	
chr28	10458825	2	38	A:0	C:1	
chr28	10459013	2	40	G:1	A:0	
chr28	10460830	2	38	T:1	C:0	
chr28	10460841	2	38	A:1	G:0	
chr28	10461036	2	36	A:1	G:0	
chr28	10461926	2	38	A:1	G:0	
chr28	10462118	2	38	A:0	C:1	
chr28	10462141	2	40	C:1	T:0	
chr28	10462648	2	36	C:1	T:0	
chr28	10462900	2	36	T:1	C:0	
chr28	10463060	2	34	A:1	G:0	
chr28	10463186	2	38	T:0	C:1	
chr28	10463187	2	38	G:0	A:1	
chr28	10463400	2	34	AT:0.676471		A:0.323529
chr28	10463475	2	38	CGGCTTT:0		C:1
chr28	10463618	2	38	C:0	T:1	
chr28	10463649	2	38	A:0	G:1	
chr28	10463654	2	38	A:0	C:1	
chr28	10464467	2	34	CT:0	C:1	
chr28	10465467	2	36	G:0	A:1	
chr28	10466178	2	34	A:0	T:1	
chr28	10466668	2	38	G:0	A:1	
chr28	10467048	2	32	C:0.90625		CT:0.09375
chr28	10468099	2	38	C:0	T:1	
chr28	10468140	2	38	C:0	T:1	
chr28	10468190	2	40	A:0	T:1	
chr28	10468601	2	34	A:0	C:1	
chr28	10468818	2	38	C:0.973684		T:0.0263158
chr28	10469513	2	36	GT:1	G:0	
chr28	10469933	2	38	T:1	C:0	
chr28	10470363	2	38	G:1	A:0	
chr28	10470454	2	38	C:1	T:0	
chr28	10471019	2	36	T:1	C:0	
chr28	10471128	2	38	A:1	G:0	
chr28	10471268	2	38	G:0	T:1	
chr28	10471749	2	36	G:1	C:0	
chr28	10472372	2	34	G:0	C:1	
chr28	10472382	2	34	C:0	T:1	
chr28	10472405	2	32	A:0	G:1	
chr28	10472496	2	32	T:0	C:1	
chr28	10472721	2	34	G:1	A:0	
chr28	10473124	2	38	C:1	T:0	
chr28	10473220	2	38	C:1	A:0	
chr28	10473702	2	38	A:1	G:0	

chr28	10474306	2	36	G:1	T:0
chr28	10474709	2	36	C:0	G:1
chr28	10474800	2	38	A:0	G:1
chr28	10475240	2	38	CAAAT:0	C:1
chr28	10476277	2	32	A:0	AT:1
chr28	10476460	2	36	CAG:0.861111	C:0.138889
chr28	10477100	2	40	G:0	C:1
chr28	10477151	2	38	G:0	A:1
chr28	10477238	2	38	T:1	A:0
chr28	10477792	2	38	TG:0	T:1
chr28	10477931	2	38	G:0	A:1
chr28	10478437	2	36	AT:0	A:1
chr28	10478679	2	30	G:0	A:1
chr28	10478862	2	36	A:0	G:1
chr28	10479039	2	32	C:1	T:0
chr28	10479052	2	32	T:1	C:0
chr28	10479655	2	38	C:1	A:0
chr28	10479729	2	38	TACATACATATACAC:0	T:1
chr28	10480199	2	36	T:1	A:0
chr28	10480614	2	40	C:1	A:0
chr28	10480617	2	40	A:1	T:0
chr28	10480627	2	40	C:1	T:0
chr28	10480657	2	36	A:1	C:0
chr28	10480753	2	38	CAAA:1	C:0
chr28	10480930	2	36	G:0	A:1
chr28	10481065	2	36	T:1	C:0
chr28	10481291	2	36	G:1	A:0
chr28	10481321	2	36	G:1	A:0
chr28	10481705	2	36	G:1	A:0
chr28	10481790	2	36	G:1	A:0
chr28	10482555	2	34	T:1	A:0
chr28	10482662	2	38	A:1	G:0
chr28	10482683	2	40	C:1	CAGTT:0
chr28	10482915	2	40	A:1	C:0
chr28	10483086	2	36	A:1	G:0
chr28	10483165	2	40	G:1	A:0
chr28	10484059	2	36	A:0	T:1
chr28	10484244	2	32	C:1	G:0
chr28	10484531	2	38	AGAGGATGGATAGGTGAAG:0.947368	A:0.0526316
chr28	10484723	2	34	A:1	G:0
chr28	10484729	2	34	C:1	T:0
chr28	10484756	2	32	A:1	AC:0
chr28	10484941	2	36	A:1	G:0
chr28	10485235	2	38	C:1	T:0
chr28	10485354	2	36	G:0.138889	A:0.861111
chr28	10485410	2	36	G:1	A:0
chr28	10485591	2	34	A:1	G:0
chr28	10485726	2	32	T:1	C:0
chr28	10485753	2	34	T:1	C:0
chr28	10486174	2	38	A:0	G:1
chr28	10486240	2	40	C:0	CTAAA:1
chr28	10486311	2	38	T:1	A:0
chr28	10486438	2	30	C:0	A:1

chr28	10486495	2	36	C:0	G:1	
chr28	10487737	2	38	A:0	G:1	
chr28	10487775	2	36	G:0	A:1	
chr28	10488156	2	38	T:1	C:0	
chr28	10488182	2	38	T:1	C:0	
chr28	10488230	2	36	G:0	A:1	
chr28	10488231	2	36	T:0	C:1	
chr28	10488467	2	34	C:1	CT:0	
chr28	10488606	2	40	T:1	C:0	
chr28	10488873	2	36	AC:1	A:0	
chr28	10488986	2	38	G:1	A:0	
chr28	10489224	2	36	A:1	G:0	
chr28	10489255	2	36	A:0	C:1	
chr28	10489259	2	36	A:0	G:1	
chr28	10489306	2	38	C:0	CCA:1	
chr28	10489446	2	34	CA:1	C:0	
chr28	10489453	2	34	G:1	C:0	
chr28	10489887	2	38	G:0	T:1	
chr28	10489978	2	38	T:0	G:1	
chr28	10490118	2	38	T:0	C:1	
chr28	10490313	2	40	A:1	T:0	
chr28	10490316	2	38	G:0	C:1	
chr28	10490690	2	36	G:1	A:0	
chr28	10490875	2	36	G:0	A:1	
chr28	10490928	2	34	GAC:0.823529		G:0.176471
chr28	10490959	2	34	G:0.176471		A:0.823529
chr28	10490970	2	36	T:0.833333		A:0.166667
chr28	10491278	2	38	G:0	A:1	
chr28	10491324	2	38	C:0	T:1	
chr28	10491898	2	36	T:0.888889		C:0.111111
chr28	10492084	2	34	A:0	G:1	
chr28	10492096	2	34	G:1	A:0	
chr28	10492234	2	34	T:1	A:0	
chr28	10492251	2	34	A:1	T:0	
chr28	10492788	2	36	C:1	T:0	
chr28	10493120	2	38	C:0	T:1	
chr28	10493336	2	38	T:1	C:0	
chr28	10493467	2	38	G:1	C:0	
chr28	10493542	2	38	G:1	GC:0	
chr28	10493971	3	28	CTT:0.0357143		C:0.0714286
CT:0.892857						
chr28	10494096	2	38	G:0.842105		C:0.157895
chr28	10494328	2	38	CA:1	C:0	
chr28	10494341	2	34	G:0.970588		A:0.0294118
chr28	10494349	2	34	A:0	G:1	
chr28	10494830	2	36	T:0	C:1	
chr28	10494838	2	36	T:0	C:1	
chr28	10494914	2	36	T:0	C:1	
chr28	10495072	2	32	C:0.8125	T:0.1875	
chr28	10495147	2	30	C:1	T:0	
chr28	10495571	2	36	G:1	T:0	
chr28	10495874	2	38	C:0.815789		T:0.184211
chr28	10495926	2	38	A:0.763158		G:0.236842
chr28	10496120	2	40	TAAGAAGAGGAAAAGAAAAAAAAAAAA:0.85		

T:0.15							
chr28	10496286	2	38	A:0	G:1		
chr28	10496348	2	34	G:0.205882	T:0.794118		
chr28	10496390	2	40	A:0.825	G:0.175		
chr28	10496416	2	40	A:0.8	G:0.2		
chr28	10496501	2	38	T:0.868421	C:0.131579		
chr28	10496502	2	38	G:0.868421	A:0.131579		
chr28	10496557	2	28	G:0.821429	A:0.178571		
chr28	10496630	2	36	C:0.916667	T:0.0833333		
chr28	10496764	2	38	G:0.157895	A:0.842105		
chr28	10496770	2	38	G:1	C:0		
chr28	10496875	2	34	A:0	C:1		
chr28	10497044	2	36	T:0.833333	C:0.166667		
chr28	10497105	2	34	AAGG:0.117647	A:0.882353		
chr28	10497318	2	24	A:0.208333	G:0.791667		
chr28	10497383	2	26	C:0.307692	A:0.692308		
chr28	10497468	2	36	G:0.777778	A:0.222222		
chr28	10497554	2	38	G:0.868421	A:0.131579		
chr28	10497558	2	38	C:0.868421	T:0.131579		
chr28	10497565	2	38	C:0.868421	A:0.131579		
chr28	10497589	2	38	A:0	C:1		
chr28	10497592	2	38	G:0	C:1		
chr28	10497660	2	36	G:0.833333	A:0.166667		
chr28	10497693	2	38	A:0	AC:1		
chr28	10497725	2	38	A:0.815789	C:0.184211		
chr28	10497773	2	36	A:0.833333	G:0.166667		
chr28	10498021	2	36	T:0.111111	C:0.888889		
chr28	10498078	2	38	A:0.131579	G:0.868421		
chr28	10498147	4	32	GCACACA:0.0625	G:0.75	GCA:0.0625	GCACA:0.125
chr28	10498448	2	40	G:0.875	A:0.125		
chr28	10498511	2	40	T:1	G:0		
chr28	10498541	2	40	AGGTCCTGTCTGGTAACAGGACCT:0.85	A:0.15		
chr28	10498710	2	40	C:0.875	T:0.125		
chr28	10498723	2	40	T:0.875	C:0.125		
chr28	10499073	2	30	C:0	CA:1		
chr28	10499137	2	34	C:0.941176	T:0.0588235		
chr28	10499219	2	36	TC:0.916667	T:0.0833333		
chr28	10499282	2	36	TA:0.611111	T:0.388889		
chr28	10499359	2	36	G:0.861111	C:0.138889		
chr28	10499416	2	38	A:0.815789	T:0.184211		
chr28	10499501	2	40	CA:0.9	C:0.1		
chr28	10499571	2	40	G:0.85	A:0.15		
chr28	10499708	2	40	T:0.85	C:0.15		
chr28	10499713	2	40	T:0.85	C:0.15		
chr28	10499878	2	34	A:0	G:1		
chr28	10500007	2	32	T:0.9375	C:0.0625		
chr28	10500100	3	30	T:0.833333	TA:0.0333333	TAA:0.133333	
chr28	10500406	2	34	C:0.852941	T:0.147059		
chr28	10500445	2	36	C:0.888889	T:0.111111		
chr28	10500483	2	34	G:0.852941	A:0.147059		
chr28	10500524	2	36	G:0.861111	A:0.138889		

chr28	10500529	2	36	G:0.861111	A:0.138889	
chr28	10500978	2	30	A:0.866667	G:0.133333	
chr28	10501240	2	34	A:0.735294	G:0.264706	
chr28	10501364	2	36	G:0.888889	C:0.111111	
chr28	10501382	2	38	A:0.868421	G:0.131579	
chr28	10501385	2	38	A:0.868421	G:0.131579	
chr28	10501432	2	40	T:0.875	C:0.125	
chr28	10501443	2	40	A:0.875	G:0.125	
chr28	10501454	2	40	G:1	A:0	
chr28	10501493	2	40	G:0.875	C:0.125	
chr28	10501534	2	36	C:1	T:0	
chr28	10501548	2	36	C:1	T:0	
chr28	10501639	2	38	G:0.894737	A:0.105263	
chr28	10501640	2	38	T:0.894737	G:0.105263	
chr28	10501691	2	40	A:0.825	G:0.175	
chr28	10501816	2	40	T:0.9	C:0.1	
chr28	10501919	2	36	T:0.972222	G:0.0277778	
chr28	10502181	2	34	A:1	C:0	
chr28	10502226	2	30	A:0.9	G:0.1	
chr28	10502518	2	36	A:0.444444	G:0.555556	
chr28	10502522	2	32	G:1	A:0	
chr28	10502601	2	38	T:1	C:0	
chr28	10502730	2	38	C:0	G:1	
chr28	10502893	2	36	T:0.888889	C:0.111111	
chr28	10503172	2	34	G:0.882353	A:0.117647	
chr28	10503199	2	36	C:0.861111	T:0.138889	
chr28	10503801	2	26	CTTTTCTT:0.269231	C:0.730769	
chr28	10503808	2	34	T:0.911765	TTTTTC:0.0882353	
chr28	10503843	2	30	CTTT:0.933333	C:0.0666667	
chr28	10504846	2	36	G:0.944444	T:0.0555556	
chr28	10505578	2	36	TGA:0.888889	T:0.111111	
chr28	10505851	2	34	CT:0.941176	C:0.0588235	
chr28	10506237	2	36	A:0.861111	C:0.138889	
chr28	10506576	2	40	G:0.925	A:0.075	
chr28	10506774	2	40	T:0.075	C:0.925	
chr28	10506846	2	38	T:0.842105	C:0.157895	
chr28	10506862	2	38	C:0.842105	CCTTG:0.157895	
chr28	10506923	2	34	A:0.911765	G:0.0882353	
chr28	10507097	2	34	T:0.852941	C:0.147059	
chr28	10507576	2	34	C:0.852941	T:0.147059	
chr28	10507885	2	36	C:1	G:0	
chr28	10507946	2	30	T:0.866667	C:0.133333	
chr28	10507957	2	18	C:0.5	CT:0.5	
chr28	10508091	2	36	C:1	T:0	
chr28	10508159	3	34	TAA:0.617647	T:0	TA:0.382353
chr28	10508191	2	26	C:1	CT:0	
chr28	10508347	2	38	A:0.921053	G:0.0789474	
chr28	10508357	2	38	T:0.921053	C:0.0789474	
chr28	10508629	2	32	C:1	T:0	
chr28	10509066	2	36	G:0	T:1	
chr28	10511315	2	36	T:0	C:1	
chr28	10512059	2	34	G:1	A:0	

chr28	10512115	2	38	T:0.973684	TTCTCTCTC:0.0263158	
chr28	10512616	2	40	G:0.575	GT:0.425	
chr28	10512620	2	40	T:1	G:0	
chr28	10512669	2	36	T:1	C:0	
chr28	10512749	2	40	T:1	C:0	
chr28	10513085	2	36	A:0	G:1	
chr28	10513322	2	38	C:0.973684	T:0.0263158	
chr28	10513530	2	38	G:1	T:0	
chr28	10514199	2	36	C:0	T:1	
chr28	10514412	2	38	A:0	G:1	
chr28	10514583	2	36	C:0	T:1	
chr28	10515232	2	34	A:0	G:1	
chr28	10515282	2	32	T:0	A:1	
chr28	10515351	3	36	A:1	T:0	AT:0
chr28	10515445	2	32	G:1	A:0	
chr28	10516428	2	36	T:1	G:0	
chr28	10516548	2	38	T:1	C:0	
chr28	10517160	2	36	A:0	G:1	
chr28	10517313	2	36	T:0	C:1	
chr28	10517345	2	36	A:0	G:1	
chr28	10517435	2	20	C:0	T:1	
chr28	10517439	2	20	T:0	TCC:1	
chr28	10517453	2	30	T:1	TCA:0	
chr28	10517690	2	32	G:0.1875	A:0.8125	
chr28	10517787	2	30	C:0	T:1	
chr28	10517820	2	30	C:0.3	T:0.7	
chr28	10518033	2	34	T:1	C:0	
chr28	10518080	2	40	G:0.725	GT:0.275	
chr28	10518081	3	40	G:0.025	T:0.275	GTT:0.7
chr28	10518269	2	28	G:1	A:0	
chr28	10518280	2	28	C:1	G:0	
chr28	10518281	2	30	C:1	T:0	
chr28	10518374	2	30	G:1	C:0	
chr28	10518411	2	32	G:0.65625	C:0.34375	
chr28	10518497	2	30	CG:0.533333	C:0.466667	
chr28	10519342	2	10	AG:0	A:1	
chr28	10519353	2	14	TC:0	T:1	
chr28	10519366	2	16	TC:0	T:1	
chr28	10519388	2	20	CT:0	C:1	
chr28	10519430	2	16	CT:0	C:1	
chr28	10519534	2	20	A:0.15	G:0.85	
chr28	10519546	2	20	A:0.15	ACC:0.85	
chr28	10519548	2	20	A:0.15	G:0.85	
chr28	10519654	2	30	G:1	C:0	
chr28	10519675	2	30	G:0.666667	C:0.333333	
chr28	10519739	2	24	T:0.291667	C:0.708333	
chr28	10519864	2	34	G:0.5	A:0.5	
chr28	10520051	2	10	AG:0.4	A:0.6	
chr28	10520150	2	34	A:1	G:0	
chr28	10520305	2	32	C:1	G:0	
chr28	10520332	2	30	AG:0.666667	A:0.333333	
chr28	10520668	2	34	G:0.735294	A:0.264706	
chr28	10520890	2	40	C:1	CG:0	

chr28	10520935 2	40	C:0.625	T:0.375
chr28	10521102 2	40	A:0.25	G:0.75
chr28	10521620 2	28	C:0.571429	G:0.428571
chr28	10522208 2	36	C:0.25	A:0.75
chr28	10522366 2	40	C:0.65	T:0.35
chr28	10522633 2	40	A:0.35	G:0.65
chr28	10522956 2	30	A:0.233333	AT:0.766667
chr28	10522983 2	34	T:1	G:0
chr28	10523071 2	32	A:0.21875	G:0.78125
chr28	10523174 2	32	G:1	A:0
chr28	10523305 2	34	A:0.588235	G:0.411765
chr28	10523777 2	38	A:0.236842	G:0.763158
chr28	10523822 2	36	G:1	A:0
chr28	10524256 2	34	A:0.617647	G:0.382353
chr28	10524518 2	40	T:0.575	G:0.425
chr28	10524932 2	32	T:0.53125	C:0.46875
chr28	10525567 2	38	G:0.631579	A:0.368421
chr28	10525601 2	38	T:0.684211	A:0.315789
chr28	10525681 2	38	C:0.236842	T:0.763158
chr28	10525713 2	36	A:1	AT:0
chr28	10525755 4	38	CAGAGAGAGAG:0.289474	C:
0.0789474	CAGAGAG:0.5	CAGAGAGAG:0.131579
chr28	10525997 2	36	A:0.277778	C:0.722222
chr28	10526376 5	40	GAATAAATA:0.275	G:0.275	GAATA:
0.175	GAATAAATAAATA:0.225	GAATAAATAAATAAATAAATA:0.05
chr28	10526519 2	34	A:0.205882	G:0.794118
chr28	10527997 2	34	G:0.617647	T:0.382353
chr28	10528186 2	34	GA:0.617647	G:0.382353
chr28	10528530 2	36	A:1	AT:0
chr28	10528700 2	34	G:0.676471	C:0.323529
chr28	10528814 2	34	C:0.617647	T:0.382353
chr28	10528977 2	40	C:1	G:0
chr28	10529218 2	36	T:0.277778	C:0.722222
chr28	10529380 2	36	C:0.194444	G:0.805556
chr28	10529384 2	36	C:0.611111	A:0.388889
chr28	10529489 3	34	TA:0.294118	T:0.676471
TAA:0.0294118
chr28	10529526 2	34	C:0.323529	CTCTG:0.676471
chr28	10530570 2	38	G:1	A:0
chr28	10531210 2	40	A:1	G:0
chr28	10531444 2	38	TG:1	T:0
chr28	10531787 2	34	CA:0.617647	C:0.382353
chr28	10531942 2	36	G:0.555556	A:0.444444
chr28	10532071 2	36	G:0.638889	A:0.361111
chr28	10532897 2	30	C:0.666667	T:0.333333
chr28	10533054 2	34	G:0.323529	C:0.676471
chr28	10533354 2	38	TAGAGG:0.973684	T:0.0263158
chr28	10533459 2	38	G:0.578947	T:0.421053
chr28	10533652 2	36	G:1	A:0
chr28	10533958 2	32	C:0.65625	T:0.34375
chr28	10534843 2	38	G:0.657895	A:0.342105
chr28	10534893 2	36	T:0.694444	C:0.305556
chr28	10535021 3	38	GA:0.631579	G:0.0526316
GAA:0.315789

chr28	10535315	2	38	AC:1	A:0		
chr28	10535331	2	38	G:1	T:0		
chr28	10535811	2	36	C:0.555556	T:0.444444		
chr28	10535898	2	40	T:0.725	G:0.275		
chr28	10535958	2	36	G:0.694444	A:0.305556		
chr28	10536054	2	38	C:0.605263	G:0.394737		
chr28	10536332	2	32	C:0.625	CAG:0.375		
chr28	10536420	2	40	A:0.25	G:0.75		
chr28	10536976	2	40	A:1	G:0		
chr28	10537452	2	38	C:0.236842	CTGAG:0.763158		
chr28	10537507	2	36	T:1	TTG:0		
chr28	10537895	3	36	CTGTGTGTG:0.111111	C:0.805556	CTGTG:0.0833333	
chr28	10538001	3	34	T:0.205882	TA:0.323529	TAA:0.470588	
chr28	10538258	2	30	T:0.266667	C:0.733333		
chr28	10538270	2	30	CA:0.666667	C:0.333333		
chr28	10538288	2	30	C:0.7	T:0.3		
chr28	10538316	2	34	C:0.705882	T:0.294118		
chr28	10538331	2	34	A:0.705882	T:0.294118		
chr28	10538333	2	34	G:0.705882	A:0.294118		
chr28	10538383	3	36	C:0.333333	CCT:0.111111	CCTCTCTCT:0.555556	
chr28	10538390	3	32	ATC:0.21875	A:0.03125	CTC:0.75	
chr28	10538436	2	34	A:0.235294	T:0.764706		
chr28	10538437	2	34	A:0.647059	T:0.352941		
chr28	10538626	2	30	C:0.933333	T:0.0666667		
chr28	10538656	2	38	G:0.894737	A:0.105263		
chr28	10538661	2	38	G:0.631579	A:0.368421		
chr28	10538951	4	40	TTTTA:0.85	T:0.125	TTTTATTTA:0.025	TTTTATTTATTTA:0
chr28	10538997	2	34	CAGAG:0.323529	C:0.676471		
chr28	10539277	2	36	G:0.611111	A:0.388889		
chr28	10539510	2	32	C:0.53125	T:0.46875		
chr28	10539517	2	32	G:0.75	A:0.25		
chr28	10539547	2	30	C:1	T:0		
chr28	10539548	2	30	T:0.666667	A:0.333333		
chr28	10539557	2	32	G:1	T:0		
chr28	10539625	2	36	G:0.611111	C:0.388889		
chr28	10539801	2	32	C:1	T:0		
chr28	10539911	2	36	T:0.305556	C:0.694444		
chr28	10540342	2	38	G:0.578947	A:0.421053		
chr28	10540382	2	38	T:0.763158	C:0.236842		
chr28	10540621	2	32	A:1	G:0		
chr28	10540628	2	34	T:0.294118	A:0.705882		
chr28	10540871	2	30	T:0.666667	A:0.333333		
chr28	10540884	2	32	A:0.625	AT:0.375		
chr28	10540929	2	28	C:0.892857	CAG:0.107143		
chr28	10541015	2	30	CG:0.766667	C:0.233333		
chr28	10541076	2	36	A:0	C:1		
chr28	10541096	2	36	T:0	G:1		
chr28	10541101	2	36	T:0	A:1		
chr28	10541103	2	36	T:0	A:1		

chr28	10541118	2	36	T:0	A:1	
chr28	10541125	2	36	A:0	C:1	
chr28	10541264	2	8	CCT:0.75	C:0.25	
chr28	10541327	2	22	T:0.136364	TA:0.863636	
chr28	10541417	2	34	A:0.617647	G:0.382353	
chr28	10541554	2	16	G:0.5625	A:0.4375	
chr28	10541916	2	38	C:1	T:0	
chr28	10541958	2	34	G:0.617647	A:0.382353	
chr28	10541965	2	34	C:0.617647	T:0.382353	
chr28	10542313	2	38	ACT:0.289474	A:0.710526	
chr28	10542400	2	36	C:0.638889	T:0.361111	
chr28	10542690	2	36	CG:1	C:0	
chr28	10542732	2	36	G:1	A:0	
chr28	10543285	2	38	A:0.578947	G:0.421053	
chr28	10544144	2	40	GATTA:0.675	G:0.325	
chr28	10544407	2	38	GC:0.394737	G:0.605263	
chr28	10544578	2	36	A:0.694444	G:0.305556	
chr28	10544598	2	34	C:0.705882	A:0.294118	
chr28	10544748	2	36	C:1	T:0	
chr28	10544902	2	38	C:0	T:1	
chr28	10544947	2	38	G:0.710526	A:0.289474	
chr28	10545316	2	40	A:0.15	G:0.85	
chr28	10545752	2	34	G:0.647059	A:0.352941	
chr28	10545882	2	40	T:1	C:0	
chr28	10545941	2	38	T:0.605263	C:0.394737	
chr28	10546062	3	32	GC:0.40625	G:0.59375	GCC:0
chr28	10546246	2	30	G:0.633333	A:0.366667	
chr28	10546274	2	30	AT:1	A:0	
chr28	10546470	2	30	T:0	G:1	
chr28	10546482	2	34	ATC:1	A:0	
chr28	10546594	2	30	C:0.6	CCG:0.4	
chr28	10546605	2	30	C:0.6	T:0.4	
chr28	10546670	2	34	A:0.676471	G:0.323529	
chr28	10546749	2	28	G:0.821429	GT:0.178571	
chr28	10546809	3	40	C:0.625	G:0	T:0.375
chr28	10546810	2	40	A:0.625	G:0.375	
chr28	10547311	2	36	C:0.972222	CG:0.0277778	
chr28	10547549	2	36	T:1	C:0	
chr28	10547730	2	34	C:1	T:0	
chr28	10548151	2	36	C:0.444444	G:0.555556	
chr28	10548254	2	34	AAG:0.470588	A:0.529412	
chr28	10548327	2	28	AAAAGAAAG:0.75	A:0.25	
chr28	10548437	2	32	G:0.4375	GAAGAAAGA:0.5625	
chr28	10548616	2	38	T:1	C:0	
chr28	10548914	2	34	G:0.676471	A:0.323529	
chr28	10550186	2	38	T:0.578947	C:0.421053	
chr28	10550235	2	36	C:1	T:0	
chr28	10550733	2	30	TA:0.5	T:0.5	
chr28	10550976	2	36	ATC:0.75	A:0.25	
chr28	10551173	2	36	C:0.694444	G:0.305556	
chr28	10551192	2	36	A:0.972222	AT:0.0277778	
chr28	10551203	2	36	TA:0.861111	T:0.138889	
chr28	10551273	2	38	T:0.894737	TGA:0.105263	

chr28 10551427 2 36 G:0.416667 A:0.583333
chr28 10551575 3 38 CTAAA:0.763158 C:0
CTAAATAAA:0.236842
chr28 10551612 3 38 T:0.578947 A:0.315789
TAAAA:0.105263
chr28 10551999 2 36 G:0.888889 A:0.111111
chr28 10552082 2 36 G:0.527778 C:0.472222
chr28 10552149 2 40 T:0.7 TAATC:0.3
chr28 10552741 2 40 CTT:0.6 C:0.4
chr28 10552743 2 40 T:0.6 TACAC:0.4
chr28 10553306 2 38 G:1 A:0
chr28 10553549 2 40 G:1 C:0
chr28 10554159 2 34 C:1 T:0
chr28 10555699 4 36 CAG:0.833333 C:0.111111
CAGAG:0.0555556 CAGAGAGAG:0
chr28 10556187 2 32 CCAGT:0.875 C:0.125
chr28 10556286 2 30 C:0.766667 CATAT:0.233333
chr28 10556306 2 26 TAC:1 T:0
chr28 10556310 2 26 T:1 TAC:0
chr28 10557662 2 36 AAAC:1 A:0
chr28 10559647 2 40 CGCCACCAT:0.525 C:0.475
chr28 10560511 2 36 T:0.694444 TA:0.305556
chr28 10561385 2 34 G:1 A:0
chr28 10561624 2 34 G:1 A:0
chr28 10561781 2 12 T:0.666667 TA:0.333333
chr28 10561958 2 8 GAGGA:0.875 G:0.125
chr28 10562746 3 38 CCT:0.421053 C:0.0789474
CCTCT:0.5
chr28 10562865 2 36 C:0.888889 G:0.111111
chr28 10563588 2 34 C:1 T:0
chr28 10563736 2 36 G:0.916667 A:0.0833333
chr28 10563944 2 34 CCTCT:0.676471 C:0.323529
chr28 10563995 2 30 TA:0.8 T:0.2
chr28 10563996 2 34 A:1 T:0
chr28 10565183 2 38 C:0.947368 T:0.0526316
chr28 10565409 2 30 TA:0.733333 T:0.266667
chr28 10565818 2 40 T:1 G:0
chr28 10566177 2 40 GT:0.5 G:0.5
chr28 10566240 2 40 GGGAAGAAA:0.85 G:0.15
chr28 10566893 2 38 T:0.473684 C:0.526316
chr28 10567203 2 40 G:0.725 T:0.275
chr28 10567759 2 38 ACAGGCTAACAACTG:0.552632 A:
0.447368
chr28 10568250 2 32 T:1 TA:0
chr28 10568591 2 38 G:1 A:0
chr28 10568623 2 36 A:0.611111 G:0.388889
chr28 10569503 2 36 A:0.555556 G:0.444444
chr28 10569890 2 34 A:0.941176 T:0.0588235
chr28 10569998 2 36 CT:1 C:0
chr28 10571008 2 34 T:0.529412 C:0.470588
chr28 10571845 2 36 T:0.5 C:0.5
chr28 10572371 2 36 A:0.972222 T:0.0277778
chr28 10573035 2 34 C:0.852941 CAAGAA:0.147059
chr28 10573367 2 40 T:0.575 C:0.425

chr28	10573672	2	32	C:0.71875	A:0.28125	
chr28	10574227	2	36	G:1	A:0	
chr28	10574663	2	38	G:0.394737	A:0.605263	
chr28	10574717	2	36	C:0.972222	G:0.0277778	
chr28	10574739	2	38	A:0.578947	AT:0.421053	
chr28	10574749	2	38	A:0.578947	AT:0.421053	
chr28	10574760	2	38	A:0.578947	AG:0.421053	
chr28	10575007	2	38	A:0.473684	G:0.526316	
chr28	10575338	2	36	C:0.444444	CT:0.555556	
chr28	10575340	2	38	T:1	C:0	
chr28	10575737	2	32	G:0.75	A:0.25	
chr28	10576071	3	32	T:0.0625	A:0.34375	TA:0.59375
chr28	10576074	2	32	A:0.65625	AT:0.34375	
chr28	10576100	2	36	A:1	T:0	
chr28	10576262	2	22	C:0.727273	CT:0.272727	
chr28	10576306	2	36	C:1	CAGAG:0	
chr28	10576365	2	34	C:0.764706	T:0.235294	
chr28	10576729	2	32	C:0.9375	T:0.0625	
chr28	10576852	2	36	T:0.583333	C:0.416667	
chr28	10576884	2	32	C:0.84375	CA:0.15625	
chr28	10576892	3	34	A:0.705882	T:0	AT:0.294118
chr28	10576911	2	38	AAAAT:0.710526	A:0.289474	
chr28	10576941	2	36	G:1	A:0	
chr28	10577125	2	32	T:0.9375	TA:0.0625	
chr28	10577126	2	32	T:0.9375	A:0.0625	
chr28	10577379	2	32	G:0.625	A:0.375	
chr28	10577506	2	26	TA:0.730769	T:0.269231	
chr28	10577805	3	36	C:0.694444	CCT:0.305556	CCTCT:0
chr28	10577855	2	38	AAAATAAAT:0.5	A:0.5	
chr28	10578996	2	38	T:0.631579	TAAC:0.368421	
chr28	10579062	2	40	AAG:0.575	A:0.425	
chr28	10580462	2	36	C:0.722222	CT:0.277778	
chr28	10580464	2	34	TA:0.970588	T:0.0294118	
chr28	10581386	2	38	GA:0.526316	G:0.473684	
chr28	10581895	2	38	C:1	T:0	
chr28	10581982	2	38	A:1	T:0	
chr28	10582211	2	38	T:1	C:0	
chr28	10582988	2	36	C:0.694444	T:0.305556	
chr28	10583824	3	40	CA:0.65	C:0	CAAA:0.35
chr28	10584077	2	34	C:0.911765	A:0.0882353	
chr28	10584152	2	34	CCT:0.558824	C:0.441176	
chr28	10584919	2	40	A:0.6	G:0.4	
chr28	10585831	2	40	AT:0.975	A:0.025	
chr28	10586056	2	34	T:0.970588	A:0.0294118	
chr28	10586531	2	28	T:0.535714	C:0.464286	
chr28	10586537	2	28	G:0.607143	A:0.392857	
chr28	10587009	2	40	A:1	AAATG:0	
chr28	10588186	2	36	C:0.527778	CA:0.472222	
chr28	10588731	2	34	C:0.5	T:0.5	
chr28	10589102	2	36	T:0.527778	TA:0.472222	
chr28	10590953	2	32	A:0.65625	G:0.34375	

chr28	10591396	2	34	G:0.941176	GA:0.0588235
chr28	10591455	2	34	T:1	A:0
chr28	10591516	2	28	TG:0.607143	T:0.392857
chr28	10592814	2	28	TA:0.535714	T:0.464286
chr28	10592845	2	34	A:0.470588	G:0.529412
chr28	10593145	2	32	CA:0.53125	C:0.46875
chr28	10594082	2	38	TA:1	T:0
chr28	10594538	2	36	G:0.527778	GA:0.472222
chr28	10594595	2	32	T:0.5625	C:0.4375
chr28	10594959	2	34	T:0.970588	G:0.0294118
chr28	10595189	2	36	T:0.527778	A:0.472222
chr28	10595758	3	36	G:0.777778	GAATA:0.222222
GAATAAATA:0
chr28	10596022	3	26	CTT:0.730769	C:0.115385
CT:0.153846
chr28	10596068	2	32	T:0.6875	TAAAG:0.3125
chr28	10596279	2	38	CCTCTCTCTCTCT:0.421053	C:
0.578947
chr28	10596340	2	34	TA:0.852941	T:0.147059
chr28	10596511	2	40	T:0.6	G:0.4
chr28	10596926	2	32	C:1	T:0
chr28	10596936	2	32	G:1	A:0
chr28	10598903	2	34	A:0.617647	G:0.382353
chr28	10599477	2	30	G:1	A:0
chr28	10600160	2	34	TTTG:0.647059	T:0.352941
chr28	10600172	2	34	G:1	T:0
chr28	10600191	2	32	C:1	CAGAG:0
chr28	10600289	2	38	A:0.631579	G:0.368421
chr28	10600456	2	40	AT:0.575	A:0.425
chr28	10600860	2	38	A:0.552632	C:0.447368
chr28	10601025	2	34	A:0.558824	G:0.441176
chr28	10601393	2	34	C:1	T:0
chr28	10601547	3	34	TA:0.617647	T:0.294118
AA:0.0882353
chr28	10601552	2	36	A:1	T:0
chr28	10601895	2	30	C:0.533333	T:0.466667
chr28	10602230	2	30	A:0.6	G:0.4
chr28	10602417	2	32	A:0.59375	G:0.40625
chr28	10602631	2	40	A:0	T:1
chr28	10602632	2	40	A:0	T:1
chr28	10604178	2	28	A:0.75	T:0.25
chr28	10604330	2	36	TA:0.388889	T:0.611111
chr28	10606620	2	38	T:0.921053	A:0.0789474
chr28	10607017	2	30	C:0.966667	CCT:0.0333333
chr28	10607070	2	28	T:0.464286	TA:0.535714
chr28	10607627	2	34	A:0.617647	AGGCTCCT:0.382353
chr28	10608406	2	40	A:0.625	AGATT:0.375
chr28	10609257	2	38	T:1	TA:0
chr28	10609343	2	38	T:1	C:0
chr28	10609455	2	36	G:0.583333	T:0.416667
chr28	10609610	2	40	C:0.5	T:0.5
chr28	10610000	2	38
CTTAGGATGTTTCAGTTTCTTTTTTTTTTTTTTTTTTTTTTTTTTTTTTTTTTTTTTTTTTTTTTTTT
TT:0	C:1

chr28 10610363 2 36 CT:0.888889 C:0.111111
chr28 10610502 2 34 G:0.911765 A:0.0882353
chr28 10610893 2 34 G:0.705882 C:0.294118
chr28 10611159 2 36 T:1 C:0
chr28 10612139 2 18 C:1 T:0
chr28 10612291 2 18 T:0.277778 TA:0.722222
chr28 10612743 2 40 C:1 T:0
chr28 10612925 2 32 A:0.96875 G:0.03125
chr28 10612928 2 32 A:0.59375 G:0.40625
chr28 10613580 2 36 T:0.555556 C:0.444444
chr28 10613752 2 28 CCT:0.607143 C:0.392857
chr28 10613808 2 34 AT:0.5 A:0.5
chr28 10613812 2 36 T:1 A:0
chr28 10614272 2 28 A:1 C:0
chr28 10614821 2 38 G:0.552632 T:0.447368
chr28 10615196 3 36 ATT:0.555556 A:0 AT:
0.444444
chr28 10615512 2 40 T:0.85 G:0.15
chr28 10617162 4 36 GATTTATTT:0.5 G:0.194444
GATTT:0 GATTTATTTATTT:0.305556
chr28 10617438 2 32 G:1 A:0
chr28 10617682 2 32 C:0.59375 CA:0.40625
chr28 10617699 2 32 C:0 T:1
chr28 10617780 2 36 G:0 A:1
chr28 10617826 2 36 G:0 A:1
chr28 10617832 2 36 CT:0 C:1
chr28 10617839 2 36 T:0 A:1
chr28 10617854 2 34 C:0 A:1
chr28 10617869 2 32 C:0 A:1
chr28 10617903 2 38 T:0 TA:1
chr28 10617918 2 38 A:0 T:1
chr28 10617919 2 38 T:0 A:1
chr28 10617927 2 38 A:0 AT:1
chr28 10617929 2 38 A:0 T:1
chr28 10617963 2 38 TG:0 T:1
chr28 10617977 2 38 GA:0 G:1
chr28 10618000 2 38 G:0 A:1
chr28 10618010 2 38 A:0 AC:1
chr28 10618031 2 38 T:0 C:1
chr28 10618032 2 38 T:0 C:1
chr28 10618246 2 14 CTA:1 C:0
chr28 10618248 2 14 A:0 C:1
chr28 10618872 2 36 T:1 G:0
chr28 10618890 2 36 A:0.944444 G:0.0555556
chr28 10619117 2 36 A:0.583333 T:0.416667
chr28 10619145 3 36 CAGAGAG:0.611111 C:0.0555556
CAG:0.333333
chr28 10619482 2 32 A:0.625 G:0.375
chr28 10619486 2 32 C:0.625 CAG:0.375
chr28 10619721 2 34 GTGGC:0.911765 G:0.0882353
chr28 10619871 2 32 GCT:1 G:0
chr28 10621164 2 34 G:0.117647 T:0.882353
chr28 10621953 2 28 C:0 T:1
chr28 10621964 2 28 A:0 G:1

chr28	10622622	2	38	GC:0.473684	G:0.526316		
chr28	10622643	2	38	G:0.473684	GTGA:0.526316		
chr28	10622647	2	38	TAGG:0.473684	T:0.526316		
chr28	10622771	2	36	T:0.972222	TAAA:0.0277778		
chr28	10622878	2	36	G:0.916667	A:0.0833333		
chr28	10622883	2	36	T:0.916667	C:0.0833333		
chr28	10622935	2	34	T:0.852941	G:0.147059		
chr28	10623376	2	38	C:1	T:0		
chr28	10624357	2	34	G:1	T:0		
chr28	10624425	2	40	C:1	A:0		
chr28	10625989	2	40	T:0.875	A:0.125		
chr28	10626000	2	40	T:0.875	C:0.125		
chr28	10626353	2	40	A:1	T:0		
chr28	10626516	2	38	A:0.947368	G:0.0526316		
chr28	10627065	2	36	T:1	C:0		
chr28	10627402	2	36	A:0.138889	G:0.861111		
chr28	10627470	2	38	A:0.868421	G:0.131579		
chr28	10627479	2	38	A:0.868421	C:0.131579		
chr28	10627673	2	34	A:0.852941	G:0.147059		
chr28	10627837	2	38	G:0.0526316	A:0.947368		
chr28	10628053	4	40	TAA:0.1	T:0.15	TA:0.725	TAAA:0.025
chr28	10628054	2	40	A:1	C:0		
chr28	10628554	2	38	T:0.105263	C:0.894737		
chr28	10628556	2	38	C:0.105263	G:0.894737		
chr28	10628630	2	36	A:1	G:0		
chr28	10628686	2	40	C:0.125	T:0.875		
chr28	10629369	2	36	A:0.888889	G:0.111111		
chr28	10629607	2	34	C:1	T:0		
chr28	10629969	2	40	GGAGAGAGAGAGAGAGA:0.8	G:0.2		
chr28	10630008	2	40	CAGAGA:0.8	C:0.2		
chr28	10630016	2	40	C:0.8	CT:0.2		
chr28	10630026	2	40	GAGAAGCAGGCTCCATGCAGGGA:0.8	G:0.2		
chr28	10630251	2	36	T:0.944444	C:0.0555556		
chr28	10630452	2	38	G:1	T:0		
chr28	10630515	2	34	C:0.0294118	T:0.970588		
chr28	10630780	2	38	T:0.131579	G:0.868421		
chr28	10630830	2	38	A:0.894737	AT:0.105263		
chr28	10630953	2	38	G:0.815789	T:0.184211		
chr28	10630963	2	38	C:1	T:0		
chr28	10631066	2	32	T:0	C:1		
chr28	10631096	2	38	C:0	T:1		
chr28	10631098	2	38	G:0	T:1		
chr28	10631733	2	38	T:0.105263	C:0.894737		
chr28	10631854	2	36	C:0.0833333	A:0.916667		
chr28	10632105	2	34	A:0.0882353	G:0.911765		
chr28	10632233	2	34	T:1	G:0		
chr28	10632242	2	34	A:0.0588235	G:0.941176		
chr28	10632248	2	34	T:1	C:0		
chr28	10632600	2	38	T:1	C:0		
chr28	10632617	2	38	A:1	G:0		
chr28	10632699	2	36	T:0.888889	C:0.111111		
chr28	10632728	2	34	G:1	C:0		

chr28	10632911	2	38	AT:0.947368	A:0.0526316	
chr28	10633163	2	34	G:0	C:1	
chr28	10633165	2	34	C:0	A:1	
chr28	10633202	2	36	ATGTC:1	A:0	
chr28	10633728	2	30	A:1	AT:0	
chr28	10633784	2	34	A:1	G:0	
chr28	10633815	2	38	A:1	T:0	
chr28	10633825	2	38	G:1	C:0	
chr28	10633919	2	38	T:0.105263	A:0.894737	
chr28	10633945	2	36	TA:1	T:0	
chr28	10634021	2	36	C:1	CTCCA:0	
chr28	10634257	2	38	TTAAAAAAAAAAA:1	T:0	
chr28	10634258	3	38	TAAAAAAAA:0.947368	TAAAA:0.0526316	T:0
chr28	10634262	2	38	AAAAAAAAT:1	A:0	
chr28	10634264	2	40	A:0.725	AATATATTC:0.275	
chr28	10634266	3	40	A:0.575	T:0.2	AAAAT:0.225
chr28	10634268	2	40	A:0.8	T:0.2	
chr28	10634269	2	34	AT:1	A:0	
chr28	10634270	3	40	T:0.8	TTC:0.2	TTCAA:0
chr28	10634271	2	38	AAATAAAT:1	A:0	
chr28	10634272	3	40	AAT:0.75	TAT:0.05	A:0.2
chr28	10634274	2	36	TAAA:1	T:0	
chr28	10634279	2	36	A:1	C:0	
chr28	10634281	2	36	ATAAAT:1	A:0	
chr28	10634487	2	34	A:0.117647	G:0.882353	
chr28	10634607	2	32	AAAG:1	A:0	
chr28	10634747	2	34	C:0.0588235	CA:0.941176	
chr28	10634817	2	30	G:0.1	C:0.9	
chr28	10634840	2	28	T:0.178571	C:0.821429	
chr28	10635490	2	28	C:1	A:0	
chr28	10636541	2	18	T:0	G:1	
chr28	10636814	2	34	C:1	CCT:0	
chr28	10637002	2	36	A:0.138889	G:0.861111	
chr28	10637235	2	30	TG:1	T:0	
chr28	10637237	2	30	G:1	T:0	
chr28	10638364	2	34	G:1	GT:0	
chr28	10638365	2	34	G:1	T:0	
chr28	10638401	2	38	G:0.526316	A:0.473684	
chr28	10638429	2	36	C:1	T:0	
chr28	10638694	2	34	G:1	A:0	
chr28	10638697	2	34	T:1	C:0	
chr28	10638806	2	40	T:0.55	C:0.45	
chr28	10638899	2	34	T:0.941176	A:0.0588235	
chr28	10639109	2	36	A:1	G:0	
chr28	10639164	2	38	T:0	C:1	
chr28	10639509	2	32	GT:0.96875	G:0.03125	
chr28	10639510	3	32	T:1	TGG:0	TGGG:0
chr28	10639511	2	30	GT:1	G:0	
chr28	10639512	2	30	T:0.9	G:0.1	
chr28	10639567	3	28	G:0.857143	GA:0.142857	GAA:0
chr28	10639837	2	36	C:0.0555556	T:0.944444	
chr28	10639951	3	38	C:0.657895	CCT:0.289474	

CCTCTCT:0.0526316

chr28	10640000	2	38	AT:1	A:0	
chr28	10640010	2	36	A:0.888889	T:0.111111	
chr28	10640011	2	36	T:0.888889	A:0.111111	
chr28	10640012	2	38	A:1	T:0	
chr28	10640013	2	38	A:1	T:0	
chr28	10640083	2	36	G:0	C:1	
chr28	10640091	2	36	G:0	C:1	
chr28	10640200	2	36	T:0.972222	A:0.0277778	
chr28	10640201	2	32	T:0.9375	A:0.0625	
chr28	10640230	2	34	C:0.970588	CAG:0.0294118	
chr28	10640250	2	34	G:0.147059	GA:0.852941	
chr28	10640251	2	34	G:0.147059	GA:0.852941	
chr28	10640316	2	26	C:0	G:1	
chr28	10640330	2	24	C:1	T:0	
chr28	10640350	2	24	C:0.166667	T:0.833333	
chr28	10640363	2	26	C:0.730769	T:0.269231	
chr28	10640400	2	28	G:0.0357143	GA:0.964286	
chr28	10640523	3	38	CTTT:0	C:1	CTT:0
chr28	10640567	2	36	T:0.138889	A:0.861111	
chr28	10640568	2	36	A:1	T:0	
chr28	10640569	2	36	A:1	T:0	
chr28	10641158	2	40	A:1	C:0	
chr28	10641161	2	40	GA:0	G:1	
chr28	10641648	2	38	T:0.315789	G:0.684211	
chr28	10642984	2	34	G:0.294118	A:0.705882	
chr28	10643363	2	18	C:0	G:1	
chr28	10643973	2	38	T:0.710526	C:0.289474	
chr28	10644364	2	32	C:0.8125	T:0.1875	
chr28	10644502	2	38	T:0.894737	C:0.105263	
chr28	10644704	2	36	G:1	A:0	
chr28	10644856	2	40	C:0.8	CCTCT:0.2	
chr28	10644871	2	36	CTGTG:1	C:0	
chr28	10644873	2	40	G:0.8	C:0.2	
chr28	10644911	2	36	TAAAA:0.194444	T:0.805556	
chr28	10644941	2	38	T:0	C:1	
chr28	10645306	2	32	T:0.78125	G:0.21875	
chr28	10645376	2	34	C:1	T:0	
chr28	10645404	2	36	G:0.861111	C:0.138889	
chr28	10645750	2	34	C:1	T:0	
chr28	10645875	2	36	A:1	AT:0	
chr28	10645877	2	36	A:0.972222	T:0.0277778	
chr28	10645886	2	38	AAAAATAAAAT:0.921053	A:0.0789474	
chr28	10645933	2	38	A:1	T:0	
chr28	10646181	2	38	C:0.868421	T:0.131579	
chr28	10646700	2	36	C:0.805556	A:0.194444	
chr28	10646902	2	34	G:0.794118	A:0.205882	
chr28	10646921	2	34	T:1	C:0	
chr28	10646983	2	38	G:0.763158	C:0.236842	
chr28	10647487	2	36	G:0.305556	GT:0.694444	
chr28	10647679	2	32	C:0.65625	T:0.34375	
chr28	10647999	2	32	G:1	A:0	
chr28	10648322	4	20	TGGCGGCGGCGGCGGC:0.45	T:0.45	

TGGCGGC:0 TGGCGGCGGCGGC:0.1
chr28 10648328 2 20 C:1 T:0
chr28 10648331 2 20 C:0.9 T:0.1
chr28 10648334 2 20 C:1 T:0
chr28 10648337 2 16 C:0.875 T:0.125
chr28 10650449 2 40 GAC:0.875 G:0.125
chr28 10650524 2 36 G:0.861111 A:0.138889
chr28 10651103 2 30 GT:0.7 G:0.3
chr28 10651360 2 40 T:0.925 TTTGATGCGTCTG:0.075
chr28 10653423 3 34 TTC:0.352941 T:0.294118
TTCTC:0.352941
chr28 10654846 2 38 C:1 CCTCT:0
chr28 10654982 2 34 C:0.705882 T:0.294118
chr28 10655254 2 34 C:0.911765 A:0.0882353
chr28 10658845 2 38 G:1 A:0
chr28 10660157 2 34 G:0.911765 GT:0.0882353
chr28 10660205 2 34 A:0.970588 G:0.0294118
chr28 10660330 2 34 C:0.676471 T:0.323529
chr28 10660506 2 32 G:0.8125 GTC:0.1875
chr28 10660516 3 32 C:0.6875 G:0.3125 CTG:0
chr28 10660985 2 36 TA:0.944444 T:0.0555556
chr28 10661219 2 26 C:0.846154 CT:0.153846
chr28 10663314 3 32 CT:0.65625 C:0.21875
CTT:0.125
chr28 10664419 2 26 C:1 CT:0
chr28 10664431 2 32 A:1 AT:0
chr28 10664460 2 32 C:0.78125 CAG:0.21875
chr28 10664886 2 36 C:0.972222 T:0.0277778
chr28 10666011 2 40 A:1 T:0
chr28 10667082 2 38 G:0.868421 A:0.131579
chr28 10668639 3 26 GAAA:0.384615 G:0.461538
GA:0.153846
chr28 10668690 2 24 TC:0.166667 T:0.833333
chr28 10670738 2 40 C:1 T:0
chr28 10673042 2 32 T:1 G:0
chr28 10674395 2 38 C:1 T:0
chr28 10674646 2 34 G:1 A:0
chr28 10675012 2 36 A:1 G:0
chr28 10675344 2 38 G:1 A:0
chr28 10675556 2 36 T:1 C:0
chr28 10676284 2 36 A:0.916667 G:0.0833333
chr28 10676356 2 34 C:1 A:0
chr28 10676947 2 40 TCTGTTTTTGCC:0.95 T:0.05
chr28 10677728 2 34 C:1 T:0
chr28 10677902 2 38 T:0.789474 C:0.210526
chr28 10682717 2 36 A:0.972222 AC:0.0277778
chr28 10685522 2 38 C:0.684211 T:0.315789
chr28 10686538 2 36 G:1 A:0
chr28 10689002 2 40 CTATCATT:0 C:1
chr28 10689011 2 40 CAACACCAGAAATACTTGTACAAGCTT:0
C:1
chr28 10689041 2 40 CAAAAG:0 C:1
chr28 10689052 2 40 TGTAGTCTGTCTAC:0 T:1
chr28 10689067 2 40 AAATTTCTGACATCAG:0 A:1

chr28	10689084	2	40	CCCAAAGGAGAA:0	C:1
chr28	10689096	2	40	T:0	TTG:1
chr28	10690263	2	36	C:1	T:0
chr28	10690732	2	34	T:0.941176	A:0.0588235
chr28	10691590	2	34	C:1	A:0
chr28	10692902	2	40	A:0.6	G:0.4
chr28	10692987	2	36	T:0.75	C:0.25
chr28	10693211	2	26	G:0.769231	GA:0.230769
chr28	10694061	2	38	T:0.684211	C:0.315789
chr28	10694176	2	34	G:0.941176	A:0.0588235
chr28	10694780	2	32	T:0.65625	C:0.34375
chr28	10694786	2	32	C:0.90625	T:0.09375
chr28	10696593	2	34	G:0.705882	A:0.294118
chr28	10696929	2	36	T:0.944444	C:0.0555556
chr28	10696974	2	38	C:0.921053	T:0.0789474
chr28	10697088	2	38	C:1	T:0
chr28	10698154	2	36	G:0.694444	A:0.305556
chr28	10698493	2	36	C:0.361111	CT:0.638889
chr28	10699540	2	36	CCTCT:0.861111	C:0.138889
chr28	10699735	2	36	A:0.666667	G:0.333333
chr28	10699869	2	40	T:0.75	C:0.25
chr28	10700188	2	30	T:0.733333	A:0.266667
chr28	10700658	2	34	G:0.705882	T:0.294118
chr28	10701197	2	32	AG:0.78125	A:0.21875
chr28	10701214	2	36	A:0.75	G:0.25
chr28	10701258	2	38	A:1	G:0
chr28	10701341	2	34	T:0.823529	A:0.176471
chr28	10701625	2	34	A:0.735294	G:0.264706
chr28	10702509	2	40	CTTTCTTTTCT:0.7	C:0.3
chr28	10702592	2	38	G:0.736842	GACAC:0.263158
chr28	10702654	2	34	T:0.647059	C:0.352941
chr28	10703393	2	36	A:0.638889	G:0.361111
chr28	10703679	2	38	C:1	T:0
chr28	10703918	2	40	C:0.9	T:0.1
chr28	10704612	2	38	CT:0.736842	C:0.263158
chr28	10705081	2	36	G:0.777778	A:0.222222
chr28	10705509	2	36	A:0.777778	T:0.222222
chr28	10705881	2	40	T:0.95	TA:0.05
chr28	10706231	2	38	A:0.657895	AT:0.342105
chr28	10706893	2	40	C:0.625	T:0.375
chr28	10707391	2	32	C:0.65625	CAGAG:0.34375
chr28	10707514	2	34	T:0.705882	C:0.294118
chr28	10707996	2	38	G:0.657895	C:0.342105
chr28	10708030	2	34	G:0.705882	GA:0.294118
chr28	10708233	2	36	C:0.666667	T:0.333333
chr28	10708260	2	36	T:0.638889	G:0.361111
chr28	10708964	2	40	A:0.675	G:0.325
chr28	10709326	2	38	A:0.736842	G:0.263158
chr28	10709441	2	36	C:0.666667	T:0.333333
chr28	10710218	2	34	ATT:0.647059	A:0.352941
chr28	10710293	2	32	A:0.5625	T:0.4375
chr28	10710621	2	36	A:0	G:1
chr28	10710791	2	38	GAC:0.789474	G:0.210526
chr28	10711014	2	38	T:0.710526	C:0.289474

chr28 10711234 3 28 AT:0.357143 A:0.321429
ATT:0.321429
chr28 10711887 2 36 C:0.777778 T:0.222222
chr28 10712016 2 38 T:0.736842 C:0.263158
chr28 10712106 2 36 T:1 G:0
chr28 10712617 2 38 AT:0.0263158 A:0.973684
chr28 10712647 2 34 C:0.764706 CAG:0.235294
chr28 10713398 2 32 G:0.6875 A:0.3125
chr28 10713453 2 30 A:1 G:0
chr28 10713526 2 32 G:1 C:0
chr28 10713788 2 40 ATCACC:0.725 A:0.275
chr28 10713889 2 38 T:0.684211 A:0.315789
chr28 10714028 2 38 T:1 C:0
chr28 10714302 2 38 T:0.710526 G:0.289474
chr28 10714438 2 38 C:0.736842 T:0.263158
chr28 10715462 2 40 C:0.625 A:0.375
chr28 10716192 2 22 C:0.909091 CT:0.0909091
chr28 10716452 2 28 T:0.571429 C:0.428571
chr28 10716626 3 28 T:0.642857 TA:0.285714
TAA:0.0714286
chr28 10717050 2 36 G:1 A:0
chr28 10717141 2 34 C:1 A:0
chr28 10717190 3 36 GA:0.638889 G:0.0833333
GAA:0.277778
chr28 10717334 2 36 C:0.666667 G:0.333333
chr28 10717644 2 38 T:0 G:1
chr28 10717691 2 34 A:1 G:0
chr28 10717743 2 36 TA:0.666667 T:0.333333
chr28 10717873 2 30 T:0.7 C:0.3
chr28 10719409 2 36 G:0.75 A:0.25
chr28 10719846 2 36 G:1 T:0
chr28 10719852 2 34 TC:0.794118 T:0.205882
chr28 10719870 2 36 A:0.944444 C:0.0555556
chr28 10721042 2 34 T:0.882353 C:0.117647
chr28 10721098 2 34 T:1 C:0
chr28 10722029 2 40 AAGC:1 A:0
chr28 10722105 2 34 C:0.588235 CTT:0.411765
chr28 10722278 2 40 A:0.825 G:0.175
chr28 10722378 2 34 T:0.852941 G:0.147059
chr28 10722820 2 38 G:0.842105 T:0.157895
chr28 10723336 2 38 A:0.894737 T:0.105263
chr28 10723380 2 34 G:0.852941 A:0.147059
chr28 10723459 2 28 ATT:0.785714 A:0.214286
chr28 10723462 2 30 T:0.933333 TA:0.0666667
chr28 10723470 2 30 T:1 A:0
chr28 10723992 2 34 CCTCTCT:0.852941 C:0.147059
chr28 10724046 2 34 AG:0.911765 A:0.0882353
chr28 10724099 2 34 A:0.852941 G:0.147059
chr28 10724369 2 38 A:0.815789 C:0.184211
chr28 10724901 2 32 A:0.75 G:0.25
chr28 10725057 2 36 CT:0.833333 C:0.166667
chr28 10725067 2 30 TTTTTTTTC:0.833333 T:
0.166667
chr28 10725068 2 30 TTTTTTTC:0.866667 T:

0.133333
chr28 10725642 3 32 CTTT:0.625 C:0.28125
CTTTT:0.09375
chr28 10725688 2 34 CAG:0.823529 C:0.176471
chr28 10725754 2 38 G:0.894737 A:0.105263
chr28 10726592 2 38 T:0.789474 C:0.210526
chr28 10727319 2 28 CA:0.642857 C:0.357143
chr28 10727335 2 26 G:0.769231 GTA:0.230769
chr28 10727645 2 40 C:0.875 A:0.125
chr28 10728488 2 40 G:0.85 A:0.15
chr28 10728500 2 36 GT:0.861111 G:0.138889
chr28 10730049 2 40 C:0.775 T:0.225
chr28 10730584 2 38 A:0.789474 C:0.210526
chr28 10731280 2 40 T:0.85 C:0.15
chr28 10731398 2 38 A:0.815789 G:0.184211
chr28 10732073 2 34 C:0.794118 T:0.205882
chr28 10732241 2 32 T:0.8125 TAAATC:0.1875
chr28 10732717 2 34 G:0.764706 GA:0.235294
chr28 10733060 2 32 A:0.34375 T:0.65625
chr28 10733105 2 36 A:0.777778 G:0.222222
chr28 10733286 2 28 A:0.821429 G:0.178571
chr28 10733508 2 38 C:0.815789 T:0.184211
chr28 10733562 2 30 CTT:0.766667 C:0.233333
chr28 10733613 2 30 C:0.9 CAG:0.1
chr28 10733971 2 36 AT:0.833333 A:0.166667
chr28 10733972 2 36 T:1 A:0
chr28 10733977 2 36 A:0.833333 T:0.166667
chr28 10734120 2 24 A:0.958333 G:0.0416667
chr28 10734164 2 32 A:0.84375 G:0.15625
chr28 10734177 2 36 T:0.805556 TA:0.194444
chr28 10734403 2 36 AAAGGC:0.888889 A:0.111111
chr28 10734434 2 32 G:1 A:0
chr28 10734596 2 34 G:0.823529 A:0.176471
chr28 10735011 2 36 T:0.833333 C:0.166667
chr28 10735131 2 40 G:0.775 A:0.225
chr28 10735165 2 34 CT:0.794118 C:0.205882
chr28 10735204 3 36 C:0.861111 CAG:0 CAGAG:
0.138889
chr28 10735206 3 36 C:0.805556 G:0.194444
CAGAG:0
chr28 10735781 2 36 A:0.944444 T:0.0555556
chr28 10736087 2 36 C:0.944444 T:0.0555556
chr28 10737303 2 38 G:1 C:0
chr28 10737520 2 38 A:0.789474 G:0.210526
chr28 10739199 2 30 G:1 A:0
chr28 10739353 2 34 G:1 A:0
chr28 10739553 3 34 AG:1 A:0 AGG:0
chr28 10739597 2 24 C:0.958333 T:0.0416667
chr28 10740077 2 40 C:1 T:0
chr28 10740145 2 38 C:0.973684 CA:0.0263158
chr28 10740147 2 38 A:0.763158 AT:0.236842
chr28 10740404 3 38 C:0.815789 CT:0.0526316
CTT:0.131579
chr28 10740445 3 40 CAGAGAG:0.625 C:0.125 CAGAG:

0.25
chr28 10740947 2 36 T:0.944444 C:0.0555556
chr28 10741145 2 36 T:1 G:0
chr28 10741571 2 38 T:1 C:0
chr28 10741822 2 38 G:1 A:0
chr28 10742324 2 38 T:1 C:0
chr28 10742707 2 36 C:0.888889 CT:0.111111
chr28 10742710 2 36 T:1 TAA:0
chr28 10743089 2 36 C:1 T:0
chr28 10743465 2 36 C:1 T:0
chr28 10744059 2 36 G:1 C:0
chr28 10744615 2 40 T:0.9 C:0.1
chr28 10744908 2 40 G:1 A:0
chr28 10745602 2 32 T:1 C:0
chr28 10746348 2 36 T:0.972222 C:0.0277778
chr28 10746405 2 38 C:1 T:0
chr28 10746557 2 36 T:0.861111 G:0.138889
chr28 10747138 2 36 C:1 T:0
chr28 10747901 2 30 A:0.9 AAAG:0.1
chr28 10748051 2 32 G:0.9375 A:0.0625
chr28 10748086 2 32 A:0.9375 G:0.0625
chr28 10748125 2 36 G:0.75 T:0.25
chr28 10748217 2 28 TA:0.678571 T:0.321429
chr28 10748235 2 38 A:0.947368 T:0.0526316
chr28 10748238 2 38 A:0.947368 G:0.0526316
chr28 10748313 2 34 TCCTG:1 T:0
chr28 10748702 2 36 C:1 T:0
chr28 10748721 2 34 C:1 T:0
chr28 10750001 2 38 A:0.868421 G:0.131579
chr28 10750349 2 40 T:0.875 C:0.125
chr28 10750796 2 30 AG:1 A:0
chr28 10750958 2 32 C:0.9375 G:0.0625
chr28 10750978 3 34 TAAA:0.705882 T:0.0294118
TAAAA:0.264706
chr28 10751008 2 40 A:1 G:0
chr28 10751976 2 40 A:0.65 G:0.35
chr28 10752176 2 40 A:0.875 G:0.125
chr28 10753060 2 36 CAT:0.888889 C:0.111111
chr28 10753191 4 40 AAAAAC:0.125 A:0.075
AAAAACAAAAC:0.775 AAAAACAAAACAAAAC:0.025
chr28 10753229 3 40 A:1 C:0 AACAAC:0
chr28 10753234 2 40 C:0.9 CAAAA:0.1
chr28 10753435 2 40 C:1 A:0
chr28 10753754 2 38 G:1 A:0
chr28 10755296 2 36 T:1 C:0
chr28 10755601 2 36 A:1 C:0
chr28 10756021 2 38 A:1 G:0
chr28 10756752 2 38 AAAAC:0.815789 A:0.184211
chr28 10757820 2 36 T:0.861111 C:0.138889
chr28 10758996 2 34 G:0.823529 A:0.176471
chr28 10760078 2 38 A:0.921053 AT:0.0789474
chr28 10760230 2 40 T:1 C:0
chr28 10761064 2 34 A:0.823529 ATAGTT:0.176471
chr28 10761196 3 32 CA:0.25 C:0.5 CAA:0.25

chr28	10761567	2	38	A:0.842105
ACAGCTATTTTTGTGTCTG:0.157895
chr28	10761664	2	36	C:0.944444	T:0.0555556
chr28	10761718	2	36	T:1	C:0
chr28	10761997	2	38	AT:1	A:0
chr28	10762095	2	32	C:1	T:0
chr28	10762286	4	34	TAAAA:0.735294	T:0.0294118
TAA:0	TAAA:0.235294
chr28	10762464	2	36	T:1	TATGTC:0
chr28	10762491	2	38	G:1	C:0
chr28	10762522	2	36	T:0.944444	C:0.0555556
chr28	10763694	2	34	G:1	A:0
chr28	10763987	4	40	CAGAG:0.675	C:0.025	CAG:0.3
CAGAGAG:0
chr28	10764143	2	36	T:0.944444	C:0.0555556
chr28	10764367	2	36	C:0.944444	T:0.0555556
chr28	10764591	2	40	T:0.925	C:0.075
chr28	10765710	2	38	A:0.973684	C:0.0263158
chr28	10765969	2	38	T:1	C:0
chr28	10766709	4	36	TAG:0.361111	T:0.0555556
TAGAG:0.555556	TAGAGAG:0.0277778
chr28	10766801	2	32	C:1	T:0
chr28	10767588	2	30	A:0.966667	G:0.0333333
chr28	10767617	2	36	CACA:1	C:0
chr28	10767623	2	36	G:0.944444	T:0.0555556
chr28	10767629	2	36	C:0.944444	A:0.0555556
chr28	10768234	2	38	G:0.973684	GT:0.0263158
chr28	10768246	2	38	T:0.868421	A:0.131579
chr28	10768247	2	38	A:0.921053	T:0.0789474
chr28	10769125	2	38	AATGT:1	A:0
chr28	10769156	2	40	C:0.85	A:0.15
chr28	10769679	2	38	T:1	C:0
chr28	10769736	2	38	A:1	G:0
chr28	10770443	2	38	G:0.921053	C:0.0789474
chr28	10770945	2	36	A:0.888889	G:0.111111
chr28	10771080	2	40	A:0.875	ACT:0.125
chr28	10772205	2	38	TA:0.868421	T:0.131579
chr28	10772387	3	30	ATT:0.9	A:0	AT:0.1
chr28	10772437	2	30	CAG:0.9	C:0.1
chr28	10772439	2	32	G:1	C:0
chr28	10772859	2	34	C:0.911765	T:0.0882353
chr28	10772884	2	38	TA:0.815789	T:0.184211
chr28	10773090	2	38	T:0.421053	C:0.578947
chr28	10773296	2	32	C:1	T:0
chr28	10773321	2	34	C:0.705882	CT:0.294118
chr28	10774112	2	38	CTA:1	C:0
chr28	10774172	3	40	AAAAC:0.95	A:0
AAAACAAAC:0.05
chr28	10774615	2	34	G:1	A:0
chr28	10774791	2	38	G:0.868421	A:0.131579
chr28	10774957	2	36	A:1	C:0
chr28	10775164	2	36	G:0.916667	A:0.0833333
chr28	10776442	2	36	T:0.888889	C:0.111111
chr28	10776469	2	38	AT:1	A:0

chr28	10776941	2	32	A:1	T:0	
chr28	10777379	2	34	C:1	T:0	
chr28	10777681	2	34	A:0.941176	AAAAAG:0.0588235	
chr28	10777772	2	36	C:0.861111	T:0.138889	
chr28	10777871	2	40	T:0.875	A:0.125	
chr28	10778270	2	34	GA:0.970588	G:0.0294118	
chr28	10778309	2	38	C:0.921053	T:0.0789474	
chr28	10778666	2	36	C:1	T:0	
chr28	10779001	2	32	C:1	T:0	
chr28	10779043	2	32	C:1	T:0	
chr28	10779220	2	38	C:1	CT:0	
chr28	10779294	2	36	C:0.916667	T:0.0833333	
chr28	10779582	2	36	G:0.944444	T:0.0555556	
chr28	10779588	2	36	G:1	T:0	
chr28	10780909	2	32	TA:0.9375	T:0.0625	
chr28	10780910	3	32	ATT:0.875	A:0.03125	AT:0.09375
chr28	10781368	2	36	C:1	T:0	
chr28	10781582	2	30	TTC:0.966667	T:0.0333333	
chr28	10781588	2	30	T:0.9	C:0.1	
chr28	10782031	2	34	C:0.911765	T:0.0882353	
chr28	10782115	2	30	T:0.866667	TA:0.133333	
chr28	10782176	2	30	T:0.9	TCTG:0.1	
chr28	10782912	2	36	A:0.916667	G:0.0833333	
chr28	10783073	2	30	T:1	TA:0	
chr28	10783138	2	16	G:1	A:0	
chr28	10783392	2	28	G:1	GC:0	
chr28	10783837	2	30	C:1	T:0	
chr28	10784018	2	24	G:1	T:0	
chr28	10784077	2	16	G:1	C:0	
chr28	10785442	2	28	G:1	A:0	
chr28	10786021	2	34	G:0.941176	A:0.0588235	
chr28	10786049	2	32	T:1	G:0	
chr28	10786062	2	34	G:0.882353	T:0.117647	
chr28	10786076	2	32	G:0	T:1	
chr28	10786136	2	40	C:0	G:1	
chr28	10786220	2	36	T:1	C:0	
chr28	10786342	2	34	G:1	A:0	
chr28	10786343	2	34	G:1	A:0	
chr28	10786477	2	36	G:1	T:0	
chr28	10786561	2	38	C:0.947368	T:0.0526316	
chr28	10786564	2	38	G:1	T:0	
chr28	10786946	2	36	G:1	A:0	
chr28	10787158	2	38	T:1	C:0	
chr28	10787239	2	38	CTTTCT:0.0263158	C:0.973684	
chr28	10787349	2	18	G:0.111111	T:0.888889	
chr28	10787915	2	38	GC:0	G:1	
chr28	10788645	2	34	C:0	G:1	
chr28	10788875	2	38	AGCATCCTGCC:0.947368	A:0.0526316	
chr28	10788888	2	38	A:1	G:0	
chr28	10789147	2	36	C:0.944444	T:0.0555556	
chr28	10789640	2	40	CA:0.975	C:0.025	
chr28	10789643	2	40	TGGGC:0.975	T:0.025	

chr28 10789652 2 40 C:0.975 T:0.025
chr28 10789771 2 36 G:0.777778 A:0.222222
chr28 10789774 2 36 G:1 A:0
chr28 10790103 2 38 T:0.842105 C:0.157895
chr28 10790171 2 36 C:1 T:0
chr28 10790236 3 40 A:0.825 AGATG:0.175
AGATGGATG:0
chr28 10790365 2 38 ATTT:0.947368 A:0.0526316
chr28 10790372 2 40 T:0.9 TA:0.1
chr28 10790396 2 34 G:0.852941 A:0.147059
chr28 10790601 2 32 G:0.96875 C:0.03125
chr28 10790641 2 36 G:1 A:0
chr28 10790672 2 40 G:1 A:0
chr28 10791027 2 32 A:0.84375 G:0.15625
chr28 10791097 2 40 A:1 G:0
chr28 10791288 2 28 G:1 GTGCTAGAGC:0
chr28 10791360 2 20 T:0 C:1
chr28 10791635 2 36 TAAAAAAA:1 T:0
chr28 10791636 2 36 A:0.888889 T:0.111111
chr28 10791651 2 36 A:1 T:0
chr28 10791925 2 38 CT:0.105263 C:0.894737
chr28 10791931 2 38 A:1 T:0
chr28 10792362 2 38 G:1 A:0
chr28 10792475 2 38 C:0 T:1
chr28 10792513 2 36 G:1 A:0
chr28 10792605 2 32 C:0.90625 CATGA:0.09375
chr28 10792682 2 36 G:1 A:0
chr28 10792694 4 38 GCACA:0.157895 G:0.0526316
GCA:0.763158 GCACACACA:0.0263158
chr28 10792696 2 38 A:1 G:0
chr28 10792698 2 38 A:1 G:0
chr28 10792700 2 38 A:1 G:0
chr28 10792702 2 38 A:1 G:0
chr28 10792912 2 34 C:0.0882353 A:0.911765
chr28 10792915 2 36 GT:1 G:0
chr28 10793232 2 34 G:1 A:0
chr28 10793344 2 30 G:0.933333 GA:0.0666667
chr28 10793715 2 38 A:0 C:1
chr28 10793717 2 38 A:1 C:0
chr28 10793778 2 40 A:0.95 G:0.05
chr28 10794281 2 38 C:0.0526316 T:0.947368
chr28 10794711 2 36 ACCC:0.916667 A:0.0833333
chr28 10794779 2 32 C:1 T:0
chr28 10796986 2 36 A:0 G:1
chr28 10797230 2 34 A:0 G:1
chr28 10797858 2 38 AGGGTTGGGGGC:0.0526316 A:
0.947368
chr28 10798030 2 38 T:0 C:1
chr28 10798522 2 38 G:0.105263 A:0.894737
chr28 10798608 2 38 A:0 C:1
chr28 10798699 2 34 C:1 T:0
chr28 10798759 2 38 T:0.894737 C:0.105263
chr28 10798910 2 38 TG:0.0789474 T:0.921053
chr28 10798916 2 38 GCACC:0.921053 G:0.0789474

chr28	10798919	2	38	C:0.0789474	CA:0.921053
chr28	10799285	2	36	C:0.111111	A:0.888889
chr28	10799467	2	22	C:0.0454545	T:0.954545
chr28	10799500	2	26	T:0	C:1
chr28	10799935	2	38	T:0.947368	G:0.0526316
chr28	10800458	2	36	C:1	T:0
chr28	10800816	2	38	T:0.921053	TAA:0.0789474
chr28	10800817	2	38	GCT:0.921053	G:0.0789474
chr28	10800836	2	38	G:0.973684	A:0.0263158
chr28	10801389	2	40	G:1	T:0
chr28	10801683	2	38	G:0.0526316	A:0.947368
chr28	10801900	2	36	G:1	A:0
chr28	10802217	2	38	AAGAG:1	A:0
chr28	10802229	2	38	G:1	A:0
chr28	10803019	2	36	C:0.944444	T:0.0555556
chr28	10803956	2	38	T:0.894737	G:0.105263
chr28	10804116	2	36	G:1	A:0
chr28	10804198	2	34	CTG:0.941176	C:0.0588235
chr28	10804280	2	38	TA:0.973684	T:0.0263158
chr28	10805161	2	40	A:0.95	G:0.05
chr28	10805332	2	40	T:1	TG:0
chr28	10805334	2	40	A:1	AGACAAAAGAAGGAGACAGC:0
chr28	10805337	2	40	GCTCTCTC:1	G:0
chr28	10805458	2	40	G:1	A:0
chr28	10805484	2	38	C:0.921053	T:0.0789474
chr28	10805708	2	36	A:0.0833333	T:0.916667
chr28	10805938	2	32	C:0.90625	T:0.09375
chr28	10806164	2	28	T:1	C:0
chr28	10807229	2	30	A:1	G:0
chr28	10807334	2	36	G:0.916667	A:0.0833333
chr28	10807952	2	38	CT:1	C:0
chr28	10808716	2	38	G:0	T:1
chr28	10808723	2	34	C:0.205882	CTCTT:0.794118
chr28	10808727	2	38	T:0.973684	TTCTTTC:0.0263158
chr28	10808788	2	38	G:1	A:0
chr28	10810294	2	40	G:1	GAAAAAAAAAAAAAAAAAAA:0
chr28	10810296	2	40	A:1	AAAATCTGATGAGGT:0
chr28	10810503	2	36	G:0.916667	A:0.0833333
chr28	10810518	2	34	G:0.911765	T:0.0882353
chr28	10810680	2	40	C:0.075	T:0.925
chr28	10810910	2	38	T:0.105263	C:0.894737
chr28	10811208	2	38	GT:0.0789474	G:0.921053
chr28	10811217	2	38	C:0.0789474	T:0.921053
chr28	10811295	2	38	G:0.0526316	C:0.947368
chr28	10811860	2	36	A:0.944444	G:0.0555556
chr28	10811995	2	34	G:0.941176	T:0.0588235
chr28	10812105	2	36	A:1	G:0
chr28	10812120	2	38	G:0.947368	A:0.0526316
chr28	10812280	2	34	T:0	C:1
chr28	10812446	2	38	C:0.0789474	T:0.921053
chr28	10812841	2	38	G:0.105263	A:0.894737
chr28	10812961	2	36	A:0.888889	G:0.111111
chr28	10813514	2	36	T:0.111111	A:0.888889
chr28	10814023	2	36	A:0.861111	G:0.138889

chr28	10814099	2	38	AT:0.894737	A:0.105263
chr28	10814148	2	38	C:0.789474	CT:0.210526
chr28	10814149	2	38	C:0.210526	T:0.789474
chr28	10814150	3	38	C:0.657895	T:0.210526
CTT:0.131579					
chr28	10814876	2	32	C:0.0625	G:0.9375
chr28	10815237	2	36	G:0.0833333	A:0.916667
chr28	10815616	2	36	G:0.916667	T:0.0833333
chr28	10815779	2	34	CG:1	C:0
chr28	10815825	4	38	CCTCTCTCT:0.184211	C:
0.815789	CCT:0	CTTCTCTCTCT:0			
chr28	10815846	2	32	C:1	G:0
chr28	10815848	2	30	C:0.2	G:0.8
chr28	10815896	2	36	T:0	TA:1
chr28	10816356	2	34	A:0.911765	G:0.0882353
chr28	10816447	2	38	G:1	A:0
chr28	10816779	2	38	G:1	A:0
chr28	10816995	2	36	G:0	A:1
chr28	10817370	2	32	A:0.9375	G:0.0625
chr28	10817535	2	40	G:1	T:0
chr28	10818450	2	38	A:1	G:0
chr28	10819367	2	36	A:0.944444	G:0.0555556
chr28	10819539	2	38	T:0.947368	C:0.0526316
chr28	10819736	2	36	C:1	CG:0
chr28	10819935	3	32	TA:0.53125	T:0.28125
TAA:0.1875					
chr28	10820648	2	32	C:1	T:0
chr28	10821105	2	36	A:0.888889	G:0.111111
chr28	10821281	2	34	G:0.0588235	A:0.941176
chr28	10821414	2	36	G:0.944444	A:0.0555556
chr28	10821971	2	38	C:1	T:0
chr28	10822368	2	38	T:0.0526316	C:0.947368
chr28	10822387	2	38	C:0	T:1
chr28	10823190	2	36	T:0.916667	C:0.0833333
chr28	10823207	2	30	T:0.933333	TA:0.0666667
chr28	10823751	2	38	T:0	A:1
chr28	10823975	2	34	G:1	A:0
chr28	10824004	2	38	C:0	A:1
chr28	10824116	2	34	T:0.941176	C:0.0588235
chr28	10824189	2	40	G:0.975	A:0.025
chr28	10824896	2	34	T:1	C:0
chr28	10825145	2	28	A:0.928571	C:0.0714286
chr28	10825361	2	30	G:1	A:0
chr28	10825928	2	36	A:0.888889	G:0.111111
chr28	10826445	2	36	T:0.0277778	C:0.972222
chr28	10826488	2	36	C:0.972222	T:0.0277778
chr28	10826717	2	36	G:0	A:1
chr28	10827088	2	36	T:0	C:1
chr28	10827157	2	36	G:0.0833333	A:0.916667
chr28	10827382	2	36	T:0.0833333	C:0.916667
chr28	10827768	2	38	CATTT:0.842105	C:0.157895
chr28	10827809	2	36	G:1	A:0
chr28	10828635	2	40	T:0.925	C:0.075
chr28	10829371	2	38	G:1	A:0

chr28	10830034	2	38	T:0.973684	TA:0.0263158	
chr28	10830214	2	34	T:1	TA:0	
chr28	10830246	2	32	G:1	A:0	
chr28	10830435	2	38	A:1	C:0	
chr28	10830652	2	36	T:0.888889	C:0.111111	
chr28	10831011	2	36	G:1	A:0	
chr28	10831614	2	36	T:0.944444	G:0.0555556	
chr28	10831690	2	34	G:0.852941	A:0.147059	
chr28	10832068	2	34	C:1	T:0	
chr28	10832590	2	36	TAAAAA:0.638889	T:0.361111	
chr28	10833107	2	36	C:0.861111	A:0.138889	
chr28	10833406	2	34	G:0.911765	A:0.0882353	
chr28	10833643	2	36	A:1	C:0	
chr28	10833794	2	34	G:0.911765	A:0.0882353	
chr28	10833839	3	40	G:0.4	GTTCTTTTCTTTTCTT:0.6	GTTCTTTTCTTTTCTTTTCTT:0
chr28	10833871	2	40	T:0.925	TC:0.075	
chr28	10833872	2	40	T:0.925	TTTTCTTTTCTTTTCTTTTC:0.075	
chr28	10834198	2	34	A:0.911765	T:0.0882353	
chr28	10834480	2	24	G:0.833333	T:0.166667	
chr28	10835886	2	36	T:1	G:0	
chr28	10836669	2	36	G:0	GC:1	
chr28	10837170	2	34	G:0.911765	A:0.0882353	
chr28	10837946	2	38	G:1	T:0	
chr28	10838357	2	36	G:1	C:0	
chr28	10838463	2	34	T:1	C:0	
chr28	10838727	2	36	C:1	T:0	
chr28	10839018	2	38	AG:0.921053	A:0.0789474	
chr28	10839579	2	34	C:0.0588235	T:0.941176	
chr28	10839669	2	24	A:1	G:0	
chr28	10839670	2	24	T:1	G:0	
chr28	10839780	3	36	TTAAATAAA:0.388889	T:0.527778	TTAAA:0.0833333
chr28	10840014	2	32	G:0	C:1	
chr28	10840673	2	36	G:0.888889	A:0.111111	
chr28	10840824	2	38	A:0	T:1	
chr28	10840924	2	32	C:1	T:0	
chr28	10841198	2	34	G:1	A:0	
chr28	10841364	2	38	T:0	A:1	
chr28	10841596	2	34	T:1	C:0	
chr28	10842018	2	36	A:1	C:0	
chr28	10842498	2	32	T:0	C:1	
chr28	10843285	2	38	G:1	A:0	
chr28	10843350	2	30	A:0.9	G:0.1	
chr28	10843359	2	32	T:1	G:0	
chr28	10843728	2	38	T:0.921053	C:0.0789474	
chr28	10843855	2	36	G:1	C:0	
chr28	10843924	2	36	T:1	C:0	
chr28	10843962	2	38	G:1	A:0	
chr28	10844288	2	36	G:1	C:0	
chr28	10844333	2	40	GACTGAGAGT:0.95	G:0.05	
chr28	10844335	2	40	C:0.15	CT:0.85	
chr28	10844341	2	40	GT:0.15	G:0.85	

chr28	10844570	2	32	A:0	G:1	
chr28	10844616	2	36	A:0	G:1	
chr28	10844668	2	36	CT:0.0555556	C:0.944444	
chr28	10844683	3	36	ATT:0.111111	A:0.888889	AT:0
chr28	10844687	2	36	T:1	A:0	
chr28	10844769	2	34	A:0.0882353	ACT:0.911765	
chr28	10844795	2	32	T:0	C:1	
chr28	10844839	2	34	C:1	T:0	
chr28	10844863	2	32	A:0	G:1	
chr28	10844929	2	40	C:0	T:1	
chr28	10845417	2	34	C:1	T:0	
chr28	10845589	2	38	T:1	C:0	
chr28	10846118	3	36	CTT:0.0833333	C:0.0277778	CT:0.888889
chr28	10846650	2	36	T:1	TAGG:0	
chr28	10846675	2	38	C:1	T:0	
chr28	10846928	2	36	G:0.166667	T:0.833333	
chr28	10847235	2	38	G:1	A:0	
chr28	10847410	2	40	G:1	C:0	
chr28	10847418	2	40	A:0.2	G:0.8	
chr28	10847584	2	34	T:0	A:1	
chr28	10847679	2	0	CAG:-nan	C:-nan	
chr28	10848111	2	32	G:0	A:1	
chr28	10848174	2	30	T:0.233333	C:0.766667	
chr28	10848225	2	34	G:0	A:1	
chr28	10848239	2	32	C:1	A:0	
chr28	10848338	2	30	T:1	TA:0	
chr28	10848394	2	32	T:1	A:0	
chr28	10848395	2	32	T:1	A:0	
chr28	10848499	2	22	A:0.363636	T:0.636364	
chr28	10849025	2	26	GTTTTT:0	G:1	
chr28	10849030	2	26	T:0	TGCCGC:1	
chr28	10849039	2	26	T:1	C:0	
chr28	10849065	2	26	GTTT:1	G:0	
chr28	10849066	2	20	T:0	G:1	
chr28	10849067	2	20	TTTT:0	TCC:1	
chr28	10849069	2	26	T:1	G:0	
chr28	10849070	2	26	T:1	TCC:0	
chr28	10849276	2	36	T:1	C:0	
chr28	10849368	2	38	C:0.789474	G:0.210526	
chr28	10849447	2	38	G:0.236842	A:0.763158	
chr28	10849747	2	36	C:1	T:0	
chr28	10850041	2	40	A:0.275	G:0.725	
chr28	10850060	2	38	T:0	C:1	
chr28	10850147	2	36	CACTG:0.222222	C:0.777778	
chr28	10850252	2	38	G:1	A:0	
chr28	10850338	2	34	G:0.176471	T:0.823529	
chr28	10850349	2	34	G:0.823529	C:0.176471	
chr28	10850419	2	38	T:0	C:1	
chr28	10850681	2	38	T:0	G:1	
chr28	10850687	2	38	T:0	C:1	
chr28	10850927	2	34	G:0.676471	C:0.323529	
chr28	10850955	2	34	A:0.323529	G:0.676471	

chr28 10850988 2 36 C:0.333333 T:0.666667
chr28 10851005 2 36 G:1 A:0
chr28 10851045 2 34 C:0.235294 T:0.764706
chr28 10851749 2 36 TA:0 T:1
chr28 10851825 2 36 T:0.861111 TA:0.138889
chr28 10851934 2 38 A:0.184211 G:0.815789
chr28 10851940 2 38 A:1 G:0
chr28 10852366 2 30 A:0 G:1
chr28 10852426 2 32 T:0 C:1
chr28 10852547 2 38 G:0.157895 A:0.842105
chr28 10852562 2 38 C:0.157895 T:0.842105
chr28 10852702 2 34 C:0.941176 T:0.0588235
chr28 10853084 2 32 C:0.875 T:0.125
chr28 10853383 2 40 G:1 A:0
chr28 10853503 2 40
TGAGCCACCAGAGCCACCCAAGCCATCAGAGCTACCCAAGCCATCCGAGCCACCCAAGCCACCA:
0.15 T:0.85
chr28 10853651 2 36 T:0 A:1
chr28 10853841 2 38 G:0.868421 A:0.131579
chr28 10854052 2 40 TCAAGTGGCCC:0.875 T:0.125
chr28 10854055 2 40 A:0.125 T:0.875
chr28 10854064 2 40 TGG:0.875 T:0.125
chr28 10854067 2 40 C:0.875 A:0.125
chr28 10854492 2 34 G:0.176471 C:0.823529
chr28 10854518 2 34 T:0.941176 TC:0.0588235
chr28 10854706 2 34 C:0.823529 T:0.176471
chr28 10854778 2 36 A:1 G:0
chr28 10854837 2 32 C:0.90625 T:0.09375
chr28 10855201 4 36 TTATCTATCTATC:0.361111 T:
0.333333 TTATCTATC:0.0277778 TTATCTATCTATCTATC:0.277778
chr28 10855616 2 36 A:0.194444 G:0.805556
chr28 10855998 2 40 G:0.8 GGA:0.2
chr28 10856001 2 40 ATCT:0.8 A:0.2
chr28 10856008 2 40 AAAAAAGTTT:0.8 A:0.2
chr28 10856018 2 40 CAA:0.8 C:0.2
chr28 10856024 2 40 GACAAAATTCAAAGTCTGCA:0.8 G:0.2
chr28 10856047 2 40 AGT:0.8 A:0.2
chr28 10856051 2 40 TAG:0.8 T:0.2
chr28 10856054 2 40 T:0.8 TAA:0.2
chr28 10856060 2 40 T:0.8 A:0.2
chr28 10856061 2 40 GTT:0.8 G:0.2
chr28 10856067 2 40 T:0.8 TAA:0.2
chr28 10856069 2 40 T:0.8 A:0.2
chr28 10856605 2 36 GC:0.111111 G:0.888889
chr28 10856834 2 32 G:0.03125 A:0.96875
chr28 10856891 2 40 C:1 T:0
chr28 10857213 4 40 GGAGAGAGAGAGAGA:0.525 G:0.05
GGAGAGAGAGA:0.175 GGAGAGAGAGAGAGAGA:0.25
chr28 10857820 2 32 C:0.90625 CT:0.09375
chr28 10857822 2 28 TC:0.357143 T:0.642857
chr28 10858452 2 36 G:1 A:0
chr28 10858753 2 36 G:1 A:0
chr28 10859031 2 40 C:1 A:0
chr28 10859145 2 38 G:0.921053 A:0.0789474

chr28 10859432 2 38 T:0.131579 C:0.868421
chr28 10859500 3 34 C:0.235294 CCT:0.764706
CCTCT:0
chr28 10859548 3 34 T:0.0882353 TAA:0.794118
TAAA:0.117647
chr28 10859591 2 38 A:0.0789474 G:0.921053
chr28 10859789 3 40 GT:0.625 G:0 GTT:0.375
chr28 10859873 2 40 C:0.075 G:0.925
chr28 10859994 2 38 A:0.973684 G:0.0263158
chr28 10860062 2 38 A:0.947368 G:0.0526316
chr28 10860259 2 34 CAGAAAAA:0.0294118 C:
0.970588
chr28 10860267 2 34 TG:0.0294118 T:0.970588
chr28 10860770 2 38 A:0 G:1
chr28 10861029 2 38 G:1 A:0
chr28 10861190 2 28 G:1 C:0
chr28 10861553 2 34 G:0.0294118 A:0.970588
chr28 10861598 2 36 C:1 T:0
chr28 10861930 2 32 G:0.03125 A:0.96875
chr28 10862118 2 36 C:0.972222 T:0.0277778
chr28 10862629 2 36 A:0 G:1
chr28 10862731 2 32 G:1 T:0
chr28 10862999 2 34 C:0.941176 T:0.0588235
chr28 10863039 2 38 A:1 T:0
chr28 10863951 2 40 C:1 T:0
chr28 10863957 2 40 C:0.075 T:0.925
chr28 10864055 2 38 A:1 G:0
chr28 10864420 2 36 GT:0.0555556 G:0.944444
chr28 10864676 2 36 T:0 A:1
chr28 10864720 2 32 A:0 T:1
chr28 10865160 2 38 TAC:0.0789474 T:0.921053
chr28 10865211 3 36 GT:0.0833333 G:0.916667
GTT:0
chr28 10865255 2 34 C:0 A:1
chr28 10865768 4 36 C:0 CT:0.138889 CTT:
0.805556 CTTT:0.0555556
chr28 10865823 2 38 A:1 AAGAGAGAG:0
chr28 10865863 2 34 C:0.852941 A:0.147059
chr28 10865864 2 34 C:0.852941 G:0.147059
chr28 10866132 2 38 TAGA:0.894737 T:0.105263
chr28 10866499 2 38 G:0.947368 A:0.0526316
chr28 10866730 2 40 A:0.925 G:0.075
chr28 10866868 2 40 C:0.95 A:0.05
chr28 10866882 3 38 T:0.131579 TTTTTTTTC:
0.763158 TTTTTTTTTC:0.105263
chr28 10866963 2 40 T:0.825 C:0.175
chr28 10867622 2 38 T:0.157895 A:0.842105
chr28 10867736 2 32 G:1 C:0
chr28 10868147 2 28 C:0.321429 T:0.678571
chr28 10868162 2 38 C:0.973684 T:0.0263158
chr28 10868166 3 40 CCTCT:0.3 C:0.025
CCTCTCTCTCTCTCTCTCT:0.675
chr28 10868195 2 36 C:0.194444 T:0.805556
chr28 10868318 2 36 T:0.194444 A:0.805556

chr28	10868481	2	32	A:0.09375	T:0.90625
chr28	10868483	2	32	T:0.1875	C:0.8125
chr28	10868521	2	36	CTCTCTCTCTT:0.75	C:0.25
chr28	10868576	2	36	A:0.166667	AAAT:0.833333
chr28	10868578	2	36	A:0.888889	AT:0.111111
chr28	10868758	2	32	C:1	T:0
chr28	10868815	2	36	G:0.166667	C:0.833333
chr28	10868964	2	38	T:0.210526	A:0.789474
chr28	10869130	2	36	C:0	T:1
chr28	10869158	2	34	T:0.852941	G:0.147059
chr28	10869284	2	40	A:0.175	G:0.825
chr28	10869285	2	40	A:0.175	C:0.825
chr28	10869310	2	40	T:0.175	G:0.825
chr28	10869556	2	34	C:0.0882353	CTTT:0.911765
chr28	10869590	2	38	GAGAT:0.947368	G:0.0526316
chr28	10869596	2	38	GAGAC:1	G:0
chr28	10869650	2	40	A:0.1	T:0.9
chr28	10869654	2	40	C:0.275	T:0.725
chr28	10869696	2	36	C:0.25	T:0.75
chr28	10869731	2	38	C:0.131579	A:0.868421
chr28	10869775	2	36	T:0.111111	A:0.888889
chr28	10869897	2	36	C:0.0833333	T:0.916667
chr28	10870079	2	40	C:0.15	T:0.85
chr28	10870392	2	38	A:0.184211	G:0.815789
chr28	10870618	2	36	T:0.194444	G:0.805556
chr28	10870656	2	36	G:0.111111	A:0.888889
chr28	10870776	2	32	G:0.25	GT:0.75
chr28	10870878	2	36	T:0.861111	C:0.138889
chr28	10870990	2	32	A:0	G:1
chr28	10871005	2	34	C:0.882353	A:0.117647
chr28	10871006	2	34	C:0.941176	T:0.0588235
chr28	10871062	2	32	G:0.125	T:0.875
chr28	10871087	2	38	C:0.157895	T:0.842105
chr28	10871128	2	38	T:0.131579	C:0.868421
chr28	10871129	2	38	G:0.131579	A:0.868421
chr28	10871149	2	38	C:0.868421	T:0.131579
chr28	10871212	2	38	G:0.868421	A:0.131579
chr28	10871264	2	32	C:0.15625	A:0.84375
chr28	10871302	2	36	C:0.111111	T:0.888889
chr28	10871347	2	34	T:0.911765	C:0.0882353
chr28	10871425	2	34	C:0.147059	T:0.852941
chr28	10871534	2	36	A:0	G:1
chr28	10871606	2	40	GC:0.9	G:0.1
chr28	10871740	2	38	A:1	G:0
chr28	10871886	2	38	G:0.131579	A:0.868421
chr28	10871887	2	38	T:0.131579	C:0.868421
chr28	10872568	2	38	T:0.0526316	A:0.947368
chr28	10872820	2	38	AG:0.210526	A:0.789474
chr28	10872826	2	38	T:0.868421	TA:0.131579
chr28	10872858	2	40	A:0.175	G:0.825
chr28	10872929	2	40	T:0.875	G:0.125
chr28	10873807	2	38	T:0.184211	C:0.815789
chr28	10874308	2	38	G:0	A:1
chr28	10874488	2	36	C:0.805556	T:0.194444

chr28	10874513	2	38	C:0.842105	T:0.157895	
chr28	10874561	2	34	A:0.941176	C:0.0588235	
chr28	10874875	2	40	T:0.925	C:0.075	
chr28	10874877	2	40	G:1	A:0	
chr28	10875700	2	32	C:0.84375	T:0.15625	
chr28	10876582	2	36	C:0.777778	G:0.222222	
chr28	10876610	2	38	G:0.710526	T:0.289474	
chr28	10876739	2	36	G:0.861111	A:0.138889	
chr28	10877140	2	36	G:0.138889	A:0.861111	
chr28	10877370	2	40	T:0.125	G:0.875	
chr28	10877580	2	32	TCA:0.96875	T:0.03125	
chr28	10877612	3	32	T:0.09375	TA:0.46875	TAAA:0.4375
chr28	10877971	2	38	G:0.947368	T:0.0526316	
chr28	10877998	2	38	C:0.868421	T:0.131579	
chr28	10878014	2	38	C:0.868421	A:0.131579	
chr28	10878049	2	36	A:0.833333	T:0.166667	
chr28	10878119	2	36	A:0	G:1	
chr28	10878826	2	36	C:1	T:0	
chr28	10879239	2	40	T:0.175	A:0.825	
chr28	10879496	2	40	T:0.9	C:0.1	
chr28	10879749	2	38	G:1	C:0	
chr28	10879835	2	40	C:0	G:1	
chr28	10879837	2	38	AT:0.763158	A:0.236842	
chr28	10879933	2	36	T:0.138889	C:0.861111	
chr28	10880214	2	34	C:0.176471	T:0.823529	
chr28	10880333	2	36	G:0.166667	GATTAA:0.833333	
chr28	10880468	2	34	C:0.911765	T:0.0882353	
chr28	10880501	2	32	G:0	A:1	
chr28	10880523	2	34	C:0.823529	T:0.176471	
chr28	10880620	2	34	C:0.323529	G:0.676471	
chr28	10880621	2	38	T:0.105263	TGTGA:0.894737	
chr28	10880633	2	38	T:0.921053	TA:0.0789474	
chr28	10880637	2	36	A:1	AAAAAAAAT:0	
chr28	10880638	3	36	A:0.166667	AAAAAAAT:0.833333	AAAAAAATT:0
chr28	10880657	2	38	A:0.921053	T:0.0789474	
chr28	10880785	2	38	T:0.0789474	A:0.921053	
chr28	10880789	2	38	C:1	A:0	
chr28	10881307	2	36	A:0.166667	C:0.833333	
chr28	10881437	2	38	A:0.894737	G:0.105263	
chr28	10881444	2	36	C:0.111111	T:0.888889	
chr28	10881588	3	32	CTTTT:0.65625	C:0.21875	CTT:0.125
chr28	10881646	2	36	C:1	T:0	
chr28	10881792	2	32	T:0.84375	G:0.15625	
chr28	10881808	2	32	G:0.75	A:0.25	
chr28	10881849	2	32	A:0.78125	G:0.21875	
chr28	10882110	2	30	C:0.766667	CCTCT:0.233333	
chr28	10882163	2	38	C:0.815789	T:0.184211	
chr28	10882434	2	40	G:0.2	C:0.8	
chr28	10882688	2	36	TG:0.194444	T:0.805556	
chr28	10882760	2	36	C:0.861111	A:0.138889	
chr28	10882808	2	36	A:0.888889	G:0.111111	

chr28	10882976	2	38	C:0.210526	G:0.789474		
chr28	10883014	2	34	C:0.823529	T:0.176471		
chr28	10883028	2	36	A:0	G:1		
chr28	10883125	2	32	T:0.875	C:0.125		
chr28	10883240	2	34	TA:0.794118	T:0.205882		
chr28	10883368	2	34	T:0.176471	G:0.823529		
chr28	10883680	2	40	T:0.15	C:0.85		
chr28	10884142	2	36	A:0.0277778	AT:0.972222		
chr28	10884231	2	38	A:0	T:1		
chr28	10884436	2	38	A:1	G:0		
chr28	10884509	2	36	G:1	C:0		
chr28	10884565	2	38	T:0.894737	G:0.105263		
chr28	10884599	4	36	TAAA:0.111111	T:0.277778	TA:0.555556	TAA:0.0555556
chr28	10885261	2	36	T:0	C:1		
chr28	10885283	2	34	G:0	A:1		
chr28	10885292	2	36	G:0.888889	A:0.111111		
chr28	10885993	2	36	T:0.416667	A:0.583333		
chr28	10886462	2	40	TAA:0.125	T:0.875		
chr28	10886477	2	40	C:0.725	A:0.275		
chr28	10886537	2	36	G:1	A:0		
chr28	10887200	2	40	G:0.675	A:0.325		
chr28	10887440	2	38	AT:0.394737	A:0.605263		
chr28	10887451	2	38	TG:1	T:0		
chr28	10887501	2	38	C:1	CT:0		
chr28	10887503	2	38	T:0.394737	TTA:0.605263		
chr28	10887504	2	38	TTTTTTTATTTTTTA:0.947368	T:0.0526316		
chr28	10887511	2	38	A:0.421053	AT:0.578947		
chr28	10887512	2	38	TTTTTTAA:1	T:0		
chr28	10887519	2	38	A:0.421053	T:0.578947		
chr28	10887526	2	38	T:0.947368	TA:0.0526316		
chr28	10887540	2	38	G:0.763158	GT:0.236842		
chr28	10887541	3	38	C:0.131579	A:0.605263	CA:0.263158	
chr28	10887552	2	38	G:0.473684	GAGAGAGGC:0.526316		
chr28	10887723	2	32	C:1	A:0		
chr28	10887724	2	32	C:0.28125	G:0.71875		
chr28	10887906	2	36	T:0.25	A:0.75		
chr28	10887991	2	34	G:0.911765	A:0.0882353		
chr28	10888001	2	34	G:1	A:0		
chr28	10888546	2	36	A:0.305556	G:0.694444		
chr28	10888643	2	38	T:0.473684	C:0.526316		
chr28	10888752	2	40	C:0.3	T:0.7		
chr28	10888856	2	34	A:0.470588	G:0.529412		
chr28	10888873	2	36	A:0.222222	T:0.777778		
chr28	10888979	2	38	A:0.394737	G:0.605263		
chr28	10889159	2	40	A:0.5	G:0.5		
chr28	10889208	2	36	G:0.916667	GTTATT:0.0833333		
chr28	10889388	2	28	G:1	C:0		
chr28	10889581	2	36	TTTTA:0.75	T:0.25		
chr28	10889598	2	38	A:0.763158	G:0.236842		
chr28	10889965	2	32	A:0.28125	AT:0.71875		

chr28 10890035 2 32 C:0.71875 T:0.28125
chr28 10890255 2 38 G:0.578947 A:0.421053
chr28 10890290 2 36 A:0.472222 G:0.527778
chr28 10890352 2 34 G:0.882353 C:0.117647
chr28 10890492 2 36 G:0.583333 A:0.416667
chr28 10890598 3 22 T:0.5 TG:0.5 TGG:0
chr28 10890840 2 6 TGGCGGCGGCGGC:0.5 T:0.5
chr28 10891262 2 30 C:0.0666667 CA:0.933333
chr28 10891267 2 30 G:0.633333 C:0.366667
chr28 10891415 2 34 G:0.647059 A:0.352941
chr28 10891631 2 38 C:0.131579 G:0.868421
chr28 10892104 2 36 G:1 C:0
chr28 10892388 2 40 A:1 C:0
chr28 10892613 3 36 C:0.611111 CT:0.222222
CTTT:0.166667
chr28 10893265 2 36 T:1 A:0
chr28 10893391 2 38 CT:1 C:0
chr28 10893402 2 38 C:0.631579 T:0.368421
chr28 10893436 2 36 G:1 A:0
chr28 10894068 2 38 C:0.578947 T:0.421053
chr28 10894272 2 36 A:0.944444 AT:0.0555556
chr28 10894438 2 38 A:1 T:0
chr28 10894539 2 36 C:0.472222 CAG:0.527778
chr28 10894647 2 36 C:0.416667 A:0.583333
chr28 10894755 2 36 G:0.916667 GT:0.0833333
chr28 10894864 2 34 A:1 T:0
chr28 10894994 2 32 C:0.8125 T:0.1875
chr28 10895137 3 38 TATTG:0.736842 T:0
TATTGATTG:0.263158
chr28 10895171 2 38 GACACAC:0.289474 G:0.710526
chr28 10895298 2 38 G:0.973684 C:0.0263158
chr28 10895383 2 38 GTTTGTT:1 G:0
chr28 10895443 2 38 G:1 A:0
chr28 10895844 2 38 G:1 A:0
chr28 10896146 2 38 T:0.526316 A:0.473684
chr28 10896255 2 38 G:0.947368 C:0.0526316
chr28 10896385 2 32 TA:0.71875 T:0.28125
chr28 10897336 2 38 G:0.0263158 GC:0.973684
chr28 10897637 2 38 A:0 C:1
chr28 10897638 2 38 A:0 T:1
chr28 10897678 2 38 A:0 T:1
chr28 10897680 2 38 G:0 A:1
chr28 10898145 2 40 T:0.05 G:0.95
chr28 10899759 2 40 G:1 A:0
chr28 10900002 2 38 A:0.447368 G:0.552632
chr28 10901102 2 34 A:0.882353 AT:0.117647
chr28 10901787 2 36 AGGATTTTCACTGT:1 A:0
chr28 10902365 4 32 T:0.28125 TTC:0.71875
TTCTC:0 TTCTCTC:0
chr28 10902471 3 36 CT:0.75 C:0.194444 CTT:
0.0555556
chr28 10902624 2 38 G:1 A:0
chr28 10902631 2 38 GGA:0.921053 G:0.0789474
chr28 10903125 2 36 T:0.916667 C:0.0833333

chr28 10905054 2 40 A:1 G:0
chr28 10905636 2 30 C:0.766667 CT:0.233333
chr28 10905757 2 38 C:0.447368 CT:0.552632
chr28 10906371 2 38 C:1 T:0
chr28 10907929 3 34 C:0.617647 CT:0.382353
CTT:0
chr28 10908479 2 34 G:0.411765 GT:0.588235
chr28 10909005 2 30 G:0.5 A:0.5
chr28 10909089 2 38 C:0.947368 T:0.0526316
chr28 10909106 2 40 T:0.425 TAAA:0.575
chr28 10909107 4 40 T:0.225 A:0.575 TAAA:0.05
TTAA:0.15
chr28 10909525 2 38 C:1 CAGAG:0
chr28 10909952 6 38 T:0.131579 TATAG:0.0263158
TATAGATAG:0.394737 TATAGATAGATAG:0.447368
TATAGATAGATAGATAG:0 TATAGATAGATAGATAGATAG:0
chr28 10909963 2 38 A:1 AGATAGATAGATG:0
chr28 10911677 2 38 CT:1 C:0
chr28 10912047 2 36 T:0.888889 A:0.111111
chr28 10912950 2 36 A:0.972222 G:0.0277778
chr28 10913896 2 38 TG:1 T:0
chr28 10914811 2 36 CTA:0.416667 C:0.583333
chr28 10914827 5 38 CAAATAAAT:0.131579 C:
0.315789 CAAAT:0.236842 CAAATAAATAAAT:0.105263
CAAATAAATAAATAAAT:0.210526
chr28 10915294 2 40 C:1 T:0
chr28 10915752 2 38 T:0.657895 A:0.342105
chr28 10915858 2 30 C:1 T:0
chr28 10916032 3 32 C:0.46875 CT:0.46875
CTTT:0.0625
chr28 10916305 2 38 T:0.473684 C:0.526316
chr28 10916365 2 38 C:0.473684 T:0.526316
chr28 10917379 2 28 C:0.178571 CT:0.821429
chr28 10917399 2 30 A:0.633333 C:0.366667
chr28 10918597 2 34 T:1 C:0
chr28 10918758 2 40 CTAAG:1 C:0
chr28 10918832 2 38 C:0.973684 T:0.0263158
chr28 10920076 2 34 C:1 G:0
chr28 10924839 2 36 G:0.611111 A:0.388889
chr28 10924859 2 36 T:1 A:0
chr28 10925321 2 26 GC:0.846154 G:0.153846
chr28 10925332 2 28 CCA:1 C:0
chr28 10925333 2 22 CA:0.5 C:0.5
chr28 10925638 2 38 C:0.736842 T:0.263158
chr28 10925699 2 36 TC:1 T:0
chr28 10926072 2 30 T:0.466667 C:0.533333
chr28 10926654 2 38 A:0.552632 G:0.447368
chr28 10926698 2 32 TA:0.84375 T:0.15625
chr28 10927281 2 34 C:0.382353 T:0.617647
chr28 10927702 2 38 C:1 T:0
chr28 10927966 2 32 T:0.40625 C:0.59375
chr28 10928296 4 38 T:0.210526 TA:0.368421
TAA:0.289474 TAAA:0.131579
chr28 10928307 2 38 A:1 AAC:0

chr28 10929538 2 38 T:0.552632 C:0.447368
chr28 10929724 2 38 G:0.921053 A:0.0789474
chr28 10930563 2 32 G:1 A:0
chr28 10930732 2 16 G:0.3125 C:0.6875
chr28 10931128 2 32 CG:0 C:1
chr28 10931223 2 28 T:0.321429 C:0.678571
chr28 10931289 2 28 A:0.785714 G:0.214286
chr28 10931588 2 36 G:0.333333 C:0.666667
chr28 10931590 2 36 G:0.5 C:0.5
chr28 10931625 2 40 T:0.325 TA:0.675
chr28 10931626 2 40 T:0.325 A:0.675
chr28 10931741 2 40 A:0.65 C:0.35
chr28 10931801 2 32 G:1 C:0
chr28 10931842 2 34 C:0.352941 CAG:0.647059
chr28 10932002 3 40 TAAAAAAAAAAAAACATA:0.325 T:0.1
TAAAAAAAAAAAAAAACATA:0.575
chr28 10932018 2 40 TA:0.525 T:0.475
chr28 10932229 3 30 CTGTG:0.333333 C:0.433333
CTG:0.233333
chr28 10932540 2 36 C:0.416667 A:0.583333
chr28 10932838 2 38 C:1 T:0
chr28 10933289 2 36 A:0.666667 G:0.333333
chr28 10933798 2 38 C:0.973684 CA:0.0263158
chr28 10933842 2 40 T:1 A:0
chr28 10934253 2 38 T:0.447368 C:0.552632
chr28 10934424 2 38 T:0.315789 C:0.684211
chr28 10934487 2 34 A:0.352941 T:0.647059
chr28 10934765 2 38 T:0.421053 C:0.578947
chr28 10934913 2 34 A:1 G:0
chr28 10934991 2 34 TA:0.382353 T:0.617647
chr28 10934992 2 34 A:1 T:0
chr28 10935306 2 38 T:0.631579 A:0.368421
chr28 10935724 2 36 T:0.75 C:0.25
chr28 10935759 3 36 TA:0.555556 T:0.444444
TAA:0
chr28 10935844 2 36 C:0.722222 T:0.277778
chr28 10936650 2 40 C:0.625 T:0.375
chr28 10936790 2 34 A:0.382353 ACT:0.617647
chr28 10936918 2 36 G:1 A:0
chr28 10937108 2 32 C:0.3125 T:0.6875
chr28 10937631 2 40 T:0.45 C:0.55
chr28 10937655 2 40 G:0.7 A:0.3
chr28 10937749 2 34 C:1 T:0
chr28 10937773 2 34 C:1 CATTT:0
chr28 10937780 3 36 T:0.388889 TTTTTTTTAA:
0.555556 TTTTTTTTTAA:0.0555556
chr28 10937947 2 36 G:0.444444 A:0.555556
chr28 10938016 2 38 T:0.763158 C:0.236842
chr28 10938156 2 30 G:1 A:0
chr28 10938218 2 36 CTCTCTTCCCT:0.75 C:0.25
chr28 10938220 2 36 CTCTTCCCT:0.805556 C:
0.194444
chr28 10938222 3 38 CTTCCCT:0.842105 CTCCCT:0.157895
C:0

chr28	10938223	2	40	T:0.8	TC:0.2		
chr28	10938226	2	40	C:0.65	T:0.35		
chr28	10938228	2	40	T:0.65	TC:0.35		
chr28	10938232	2	38	C:0.710526	G:0.289474		
chr28	10938234	2	40	G:1	C:0		
chr28	10938284	2	38	G:0.842105	GAAAGA:0.157895		
chr28	10938529	4	40	TTTTCTTTCTTTCTTTCTTTCTTTCTTTCTTTCTTTCTTTC:0.1	T:0.45	TTTTCTTTC:0.175	TTTTCTTTCTTTCTTTCTTTCTTTCTTTCTTTCTTTCTTTCTTTCTTTC:0.275
chr28	10938824	2	34	A:0.529412	G:0.470588		
chr28	10939148	2	36	C:0.722222	T:0.277778		
chr28	10939244	2	40	T:0.625	G:0.375		
chr28	10939253	2	40	G:0.35	A:0.65		
chr28	10939350	2	38	A:0.368421	G:0.631579		
chr28	10939452	2	38	A:0.763158	G:0.236842		
chr28	10939519	2	34	A:1	G:0		
chr28	10939720	2	36	G:1	A:0		
chr28	10940564	2	36	C:0.916667	T:0.0833333		
chr28	10940679	2	34	C:0.676471	T:0.323529		
chr28	10941347	2	34	C:0.294118	T:0.705882		
chr28	10941594	2	34	C:1	T:0		
chr28	10941647	2	40	G:0.325	GCTCT:0.675		
chr28	10942341	2	40	G:1	C:0		
chr28	10942723	2	38	C:0.736842	T:0.263158		
chr28	10942928	2	38	T:0.684211	G:0.315789		
chr28	10943113	2	36	G:0.694444	A:0.305556		
chr28	10943289	2	36	C:0.583333	G:0.416667		
chr28	10943290	2	36	A:0.583333	G:0.416667		
chr28	10943378	2	38	A:0.578947	G:0.421053		
chr28	10943556	2	34	T:0.647059	C:0.352941		
chr28	10943966	2	38	A:0.657895	G:0.342105		
chr28	10943989	2	36	A:0.638889	G:0.361111		
chr28	10944846	2	34	C:0.529412	T:0.470588		
chr28	10944878	2	36	C:1	A:0		
chr28	10945075	2	34	G:0.941176	A:0.0588235		
chr28	10945214	2	38	T:0.342105	C:0.657895		
chr28	10945434	2	36	G:0.611111	A:0.388889		
chr28	10946291	2	28	C:0.571429	T:0.428571		
chr28	10946391	2	26	G:0.730769	A:0.269231		
chr28	10946568	2	22	A:0	G:1		
chr28	10946632	2	8	A:0.25	G:0.75		
chr28	10948332	2	32	C:0.59375	T:0.40625		
chr28	10948399	2	32	C:0.65625	CT:0.34375		
chr28	10948404	2	32	C:0.65625	CCG:0.34375		
chr28	10948410	2	32	T:0.65625	C:0.34375		
chr28	10949030	2	38	ACAGCTGGCC:0.921053	A:0.0789474		
chr28	10949135	2	40	T:0.425	A:0.575		
chr28	10949245	2	38	T:0.394737	C:0.605263		
chr28	10949618	2	28	A:1	G:0		
chr28	10949636	2	28	T:0.5	TA:0.5		
chr28	10949827	2	34	C:0.676471	T:0.323529		
chr28	10949911	2	34	A:0.323529	C:0.676471		

chr28	10950059	2	34	C:1	T:0	
chr28	10950206	2	34	A:0.911765	C:0.0882353	
chr28	10950222	2	36	G:0.527778	A:0.472222	
chr28	10950323	2	36	C:1	G:0	
chr28	10950328	2	36	C:0.361111	T:0.638889	
chr28	10950411	2	38	C:0.342105	G:0.657895	
chr28	10950548	2	34	C:0.441176	T:0.558824	
chr28	10950605	2	36	C:0.972222	G:0.0277778	
chr28	10951080	2	38	C:0.368421	A:0.631579	
chr28	10951082	2	38	G:0.368421	A:0.631579	
chr28	10951248	2	40	A:1	G:0	
chr28	10951424	2	38	ACCCTGCATGGTTTAC:0.868421	A:0.131579	
chr28	10951510	2	34	C:0.352941	G:0.647059	
chr28	10952305	2	38	A:1	C:0	
chr28	10952502	3	36	A:0.25	C:0.0555556	G:0.694444
chr28	10952514	2	36	A:0.944444	G:0.0555556	
chr28	10952562	2	32	C:0.96875	G:0.03125	
chr28	10952625	2	38	G:0.973684	C:0.0263158	
chr28	10952637	2	38	GTA:0.289474	G:0.710526	
chr28	10952639	2	38	A:0.973684	G:0.0263158	
chr28	10952815	2	34	T:0.352941	C:0.647059	
chr28	10952979	2	36	A:0.472222	G:0.527778	
chr28	10953058	2	34	A:0.941176	T:0.0588235	
chr28	10953067	2	34	T:0.794118	A:0.205882	
chr28	10953068	2	34	T:0.352941	A:0.647059	
chr28	10953078	2	32	AAAAAT:1	A:0	
chr28	10953092	2	34	A:0.352941	G:0.647059	
chr28	10953185	2	20	C:0.45	CA:0.55	
chr28	10953242	2	22	T:0.227273	C:0.772727	
chr28	10953533	2	16	G:1	T:0	
chr28	10953616	2	26	C:0.538462	A:0.461538	
chr28	10953773	2	40	G:0.975	GAGTGTGGA:0.025	
chr28	10953774	2	40	C:0.975	CCACCCATGGCATAGCCAGCAT:0.025	
chr28	10954058	2	32	C:0.25	T:0.75	
chr28	10954059	2	32	A:0.25	G:0.75	
chr28	10954098	2	38	C:0.684211	CAGGGA:0.315789	
chr28	10954143	2	38	T:0.421053	A:0.578947	
chr28	10954226	2	36	T:0.388889	TTTTA:0.611111	
chr28	10954538	2	36	G:0.0833333	C:0.916667	
chr28	10954668	2	34	C:0.735294	T:0.264706	
chr28	10954711	2	34	A:0.911765	AATT:0.0882353	
chr28	10954733	2	36	C:1	T:0	
chr28	10954773	2	38	AGGCG:0.0789474	A:0.921053	
chr28	10955057	2	38	T:0.947368	C:0.0526316	
chr28	10955111	2	34	CACG:0.676471	C:0.323529	
chr28	10955169	2	34	A:0.294118	G:0.705882	
chr28	10955289	2	38	G:0.210526	C:0.789474	
chr28	10955498	2	38	GAC:0.763158	G:0.236842	
chr28	10955581	2	36	G:0.777778	A:0.222222	
chr28	10955860	2	36	G:0.861111	T:0.138889	
chr28	10955862	2	36	A:0.861111	G:0.138889	

chr28	10956211	2	36	A:0.75	C:0.25	
chr28	10956220	2	38	C:0.657895	T:0.342105	
chr28	10956406	2	40	AGTGATTTTTTTTACCTTG:0.625	A:0.375	
chr28	10956444	2	40	T:0.65	A:0.35	
chr28	10956472	3	34	C:0.617647	CAG:0.147059	CAGAGAG:0.235294
chr28	10956634	2	32	AT:0.75	A:0.25	
chr28	10956653	2	32	A:0.625	T:0.375	
chr28	10956654	2	32	A:0.625	T:0.375	
chr28	10956764	2	38	A:0.315789	AT:0.684211	
chr28	10956893	2	34	G:0.970588	A:0.0294118	
chr28	10956988	2	38	A:0.368421	G:0.631579	
chr28	10957438	2	34	G:1	A:0	
chr28	10957724	2	34	T:0.676471	G:0.323529	
chr28	10957781	2	30	C:0.8	CT:0.2	
chr28	10957792	2	30	G:0.5	T:0.5	
chr28	10957793	2	30	GAA:0.8	G:0.2	
chr28	10957794	2	30	A:0.666667	G:0.333333	
chr28	10957804	2	30	A:0.833333	T:0.166667	
chr28	10958111	2	38	G:0.315789	A:0.684211	
chr28	10958233	2	34	AT:0.911765	A:0.0882353	
chr28	10958332	2	36	T:0.638889	G:0.361111	
chr28	10958419	2	38	C:0.657895	A:0.342105	
chr28	10958530	3	36	CT:0.666667	C:0.0277778	CTT:0.305556
chr28	10958549	3	36	CTT:0.444444	C:0.0277778	CT:0.527778
chr28	10958909	2	38	GGAC:0.894737	G:0.105263	
chr28	10958913	2	38	TCA:0.894737	T:0.105263	
chr28	10959445	2	40	C:0.375	T:0.625	
chr28	10959761	2	32	T:1	A:0	
chr28	10959870	2	40	G:1	C:0	
chr28	10959990	2	30	G:0.666667	GAC:0.333333	
chr28	10960491	2	34	TA:0.323529	T:0.676471	
chr28	10960645	2	38	TCTA:1	T:0	
chr28	10960661	2	38	C:0.657895	T:0.342105	
chr28	10960664	2	38	T:0.657895	C:0.342105	
chr28	10960683	2	22	CT:0.545455	C:0.454545	
chr28	10960719	3	34	C:0.588235	CAG:0.323529	CAGAGAG:0.0882353
chr28	10960739	2	28	GGC:1	G:0	
chr28	10961047	2	38	T:0.973684	C:0.0263158	
chr28	10961328	2	40	T:0.75	TTTTTTTTGTTTGTTTTTTTTTTTTTACATCAATAAAATCTTAA:0.25	
chr28	10961337	2	36	ACC:0.472222	A:0.527778	
chr28	10961571	2	36	T:0.388889	G:0.611111	
chr28	10961978	2	36	C:1	T:0	
chr28	10962777	2	28	T:0.321429	C:0.678571	
chr28	10962807	2	40	CGATCCTGGAGACCCGGGATCG:0.975	C:0.025	
chr28	10962928	2	34	TTAAAAAAAA:0.970588	T:0.0294118	
chr28	10962929	2	36	T:0.777778	TA:0.222222	
chr28	10962938	2	34	T:0.382353	TA:0.617647	

chr28	10962939	2	34	A:0.911765	AT:0.0882353		
chr28	10963007	2	36	A:0.972222	G:0.0277778		
chr28	10963122	2	38	A:0.815789	G:0.184211		
chr28	10963320	2	38	CAA:0.631579	C:0.368421		
chr28	10963342	2	38	G:0.552632	C:0.447368		
chr28	10963556	2	40	C:0.65	CT:0.35		
chr28	10963558	2	40	T:0.75	TTCTTTC:0.25		
chr28	10964557	2	34	GC:0.647059	G:0.352941		
chr28	10964800	2	32	C:0.59375	T:0.40625		
chr28	10964924	2	38	T:1	C:0		
chr28	10965013	2	32	C:0.90625	CT:0.09375		
chr28	10965016	2	34	T:0.617647	TTTTC:0.382353		
chr28	10965062	2	38	C:0.263158	G:0.736842		
chr28	10965153	2	34	G:1	A:0		
chr28	10965483	2	32	A:0.5	ACC:0.5		
chr28	10965485	2	36	C:0.861111	CCT:0.138889		
chr28	10965491	2	34	C:0.794118	CCA:0.205882		
chr28	10965492	2	36	A:0.861111	C:0.138889		
chr28	10965784	2	30	AAGG:0.733333	A:0.266667		
chr28	10966005	2	32	A:0.1875	G:0.8125		
chr28	10966282	2	8	G:0.75	C:0.25		
chr28	10966579	2	34	G:0.323529	A:0.676471		
chr28	10966622	2	32	A:0.59375	G:0.40625		
chr28	10966652	2	30	G:0.733333	A:0.266667		
chr28	10966710	2	36	T:0.611111	C:0.388889		
chr28	10966731	2	36	G:0.25	T:0.75		
chr28	10966732	2	36	A:0.25	T:0.75		
chr28	10966981	2	38	G:0.210526	A:0.789474		
chr28	10967204	2	36	G:0.666667	A:0.333333		
chr28	10967625	2	38	C:0.815789	T:0.184211		
chr28	10967688	2	40	A:0.625	G:0.375		
chr28	10967758	2	32	G:0.90625	C:0.09375		
chr28	10967940	2	40	T:0.25	C:0.75		
chr28	10967997	2	40	T:0.675	C:0.325		
chr28	10968267	2	40	A:0.9	T:0.1		
chr28	10968892	2	38	A:0.921053	G:0.0789474		
chr28	10969042	2	40	T:1	C:0		
chr28	10969128	2	38	G:0.842105	A:0.157895		
chr28	10969133	2	40	T:0.675	C:0.325		
chr28	10969630	2	40	A:0.25	G:0.75		
chr28	10969949	2	40	A:0.2	ATTGT:0.8		
chr28	10969976	2	36	G:0.861111	A:0.138889		
chr28	10970109	2	34	G:0.970588	A:0.0294118		
chr28	10970473	2	34	T:0.705882	C:0.294118		
chr28	10970631	2	36	T:0.138889	A:0.861111		
chr28	10970698	2	38	G:0.157895	A:0.842105		
chr28	10971002	2	36	A:1	C:0		
chr28	10971194	2	34	CAG:0.117647	C:0.882353		
chr28	10971312	2	40	C:1	T:0		
chr28	10971398	2	38	G:0.605263	A:0.394737		
chr28	10972104	2	36	G:0.166667	A:0.833333		
chr28	10972185	4	38	ATTT:0.421053	AT:0.105263	ATT:0.421053	A:0.0526316
chr28	10972211	4	40	CTTTATTTATTTATTTATTTATTTATTTATTTA:			

```
0.425	C:0.2	CTTTATTTA:0.05	CTTTATTTATTTA:0.325
chr28	10972309	2	38	T:0.289474	C:0.710526
chr28	10972350	2	38	C:0.763158	T:0.236842
chr28	10972364	2	40	C:0.9	T:0.1
chr28	10972567	2	38	G:0.868421	A:0.131579
chr28	10972570	2	36	G:0.166667	T:0.833333
chr28	10972747	2	38	GCCATCTTTTTCTGAAAAAGC:0.894737
G:0.105263
chr28	10972778	2	32	T:0.75	TC:0.25
chr28	10972787	2	32	G:0.75	GTGGT:0.25
chr28	10973528	2	38	G:0.815789	A:0.184211
chr28	10973608	2	36	A:0.861111	G:0.138889
chr28	10973714	2	36	G:1	T:0
chr28	10973828	2	32	C:1	T:0
chr28	10974020	2	34	C:0.264706	A:0.735294
chr28	10974066	2	34	C:0.176471	T:0.823529
chr28	10974133	2	32	C:0.3125	T:0.6875
chr28	10974294	2	38	C:0.815789	T:0.184211
chr28	10974322	2	34	C:0.911765	T:0.0882353
chr28	10974417	2	38	G:0.605263	A:0.394737
chr28	10974622	2	40	T:0.175	TC:0.825
chr28	10974634	2	40	T:0.175	C:0.825
chr28	10974958	2	40	T:0.175	TTTC:0.825
chr28	10975024	3	36	GTTTT:0.472222	G:0.361111
GT:0.166667
chr28	10975261	2	40	G:1	A:0
chr28	10975398	2	34	T:0.911765	C:0.0882353
chr28	10975516	2	40	A:0.225	G:0.775
chr28	10975656	2	38	G:0.921053	A:0.0789474
chr28	10975693	2	36	AT:1	A:0
chr28	10975698	2	36	T:1	TA:0
chr28	10975702	4	36	TAA:0.166667	T:0	AAA:0
TA:0.833333
chr28	10975704	2	34	A:0.970588	T:0.0294118
chr28	10975763	2	38	G:0.236842	T:0.763158
chr28	10976105	2	8	GA:0	G:1
chr28	10976177	2	22	TTATG:0.318182	T:0.681818
chr28	10976274	2	36	TC:0.194444	T:0.805556
chr28	10976373	2	36	C:0.944444	T:0.0555556
chr28	10976533	2	32	C:0.90625	CAT:0.09375
chr28	10976599	2	34	G:0.117647	C:0.882353
chr28	10976600	2	34	A:0.117647	T:0.882353
chr28	10976984	2	38	T:0.342105	C:0.657895
chr28	10977233	3	36	C:0.444444	CAGAGAG:0.277778
CAGAGAGAGAG:0.277778
chr28	10977288	2	26	T:0.269231	C:0.730769
chr28	10977367	2	30	G:0.433333	A:0.566667
chr28	10977383	2	30	CA:0.9	C:0.1
chr28	10977384	2	32	A:0.34375	G:0.65625
chr28	10977394	2	32	T:0.34375	A:0.65625
chr28	10977396	2	32	T:0.34375	A:0.65625
chr28	10977449	2	24	CGACA:0.916667	C:0.0833333
chr28	10977452	3	34	CAG:0.764706	C:0.0588235
GAG:0.176471
```

chr28	10977531	2	30	CATG:0.266667	C:0.733333		
chr28	10977593	2	26	T:0.0384615	C:0.961538		
chr28	10978165	3	38	T:0.684211	C:0.0789474	G:0.236842	
chr28	10978203	2	38	G:0.868421	T:0.131579		
chr28	10978372	2	36	G:1	A:0		
chr28	10978381	2	36	A:0.611111	C:0.388889		
chr28	10978429	2	36	C:0.166667	CA:0.833333		
chr28	10978430	2	36	C:0.666667	T:0.333333		
chr28	10978442	2	36	G:0.166667	GGTA:0.833333		
chr28	10978513	2	32	A:0.09375	C:0.90625		
chr28	10978703	2	28	A:0.107143	C:0.892857		
chr28	10979470	2	38	C:1	T:0		
chr28	10979777	2	40	G:0.25	GTCTT:0.75		
chr28	10979849	2	40	T:0.625	A:0.375		
chr28	10979907	2	38	G:0.973684	A:0.0263158		
chr28	10979981	2	36	G:1	A:0		
chr28	10980312	2	34	G:0.970588	T:0.0294118		
chr28	10980412	2	36	T:0.888889	TA:0.111111		
chr28	10980524	2	36	A:0.611111	G:0.388889		
chr28	10980567	2	36	T:0.75	A:0.25		
chr28	10980625	2	36	T:0.694444	C:0.305556		
chr28	10981037	2	40	A:0.775	G:0.225		
chr28	10981304	2	38	T:0.763158	C:0.236842		
chr28	10981413	2	36	T:0.722222	C:0.277778		
chr28	10981744	2	36	T:0.75	C:0.25		
chr28	10981920	2	38	C:0.526316	T:0.473684		
chr28	10982003	2	36	A:0.527778	G:0.472222		
chr28	10982720	2	36	A:1	T:0		
chr28	10983100	2	38	G:0.789474	A:0.210526		
chr28	10983107	2	38	G:0.631579	A:0.368421		
chr28	10983144	2	36	C:1	CTTCT:0		
chr28	10983198	3	40	CAGAGAG:0.75	C:0.175	CAGAGAGAG:0.075	
chr28	10983293	2	36	C:0.861111	A:0.138889		
chr28	10983352	2	40	CCTT:1	C:0		
chr28	10983401	2	40	A:0.85	G:0.15		
chr28	10983418	2	38	T:0.842105	G:0.157895		
chr28	10983603	2	34	G:0.176471	A:0.823529		
chr28	10983637	2	38	T:0.210526	C:0.789474		
chr28	10983774	2	36	A:0.333333	AGTCC:0.666667		
chr28	10983818	2	40	G:1	A:0		
chr28	10983977	2	38	G:0.684211	A:0.315789		
chr28	10984009	2	34	G:0.705882	A:0.294118		
chr28	10984411	2	38	C:0.789474	T:0.210526		
chr28	10984568	2	36	C:0.777778	A:0.222222		
chr28	10984869	2	38	G:0.210526	A:0.789474		
chr28	10984997	2	34	T:0.117647	C:0.882353		
chr28	10985087	2	40	C:0.25	T:0.75		
chr28	10985273	4	40	CTG:0.55	C:0.225	CTGTG:0	CTGTGTGTGTGTGTGTGTGTGTGTG:0.225
chr28	10985285	2	40	G:0.725	GTGTGTGTGTGTGTGTGTGTGTGTGTGTGTGTGT:0.275		
chr28	10985313	2	38	A:0.263158	G:0.736842		

chr28	10985337	2	38	A:0.263158	G:0.736842	
chr28	10985486	2	34	A:0.764706	C:0.235294	
chr28	10985523	2	36	T:0.361111	C:0.638889	
chr28	10985564	2	34	C:0.794118	CT:0.205882	
chr28	10985604	2	38	AT:0.789474	A:0.210526	
chr28	10985656	2	38	T:0.710526	C:0.289474	
chr28	10985748	2	34	C:0.735294	CT:0.264706	
chr28	10985921	2	34	A:0.676471	AT:0.323529	
chr28	10986385	2	38	C:0.921053	T:0.0789474	
chr28	10986427	2	36	T:0.805556	C:0.194444	
chr28	10986678	2	38	T:0.842105	G:0.157895	
chr28	10986718	2	40	A:0.8	G:0.2	
chr28	10986842	2	38	G:0.894737	A:0.105263	
chr28	10986885	2	40	G:0.8	T:0.2	
chr28	10987378	2	36	C:0.111111	A:0.888889	
chr28	10987477	2	36	T:0.888889	A:0.111111	
chr28	10987505	2	40	T:0.225	G:0.775	
chr28	10987590	2	40	T:0.225	C:0.775	
chr28	10987599	2	40	C:0.225	G:0.775	
chr28	10987920	2	38	A:0.842105	G:0.157895	
chr28	10987986	2	34	T:0.852941	C:0.147059	
chr28	10988012	2	34	C:1	T:0	
chr28	10988453	2	36	C:0.972222	CG:0.0277778	
chr28	10988522	2	38	C:0.657895	T:0.342105	
chr28	10988580	2	34	A:0.882353	G:0.117647	
chr28	10988882	2	34	C:0.882353	T:0.117647	
chr28	10988978	2	38	CT:0.710526	C:0.289474	
chr28	10989264	2	40	C:0.325	T:0.675	
chr28	10989268	2	40	C:0.325	T:0.675	
chr28	10989464	2	38	A:1	C:0	
chr28	10989866	2	38	C:0.894737	A:0.105263	
chr28	10990280	2	32	A:0.875	T:0.125	
chr28	10990637	2	38	T:0.631579	G:0.368421	
chr28	10990840	2	40	A:0.9	G:0.1	
chr28	10991115	2	34	TTTC:0.705882	T:0.294118	
chr28	10991142	3	26	T:0.423077	TA:0.307692	TAA:0.269231
chr28	10991340	2	36	T:0.527778	C:0.472222	
chr28	10991590	2	36	G:1	A:0	
chr28	10991684	2	34	A:0.941176	T:0.0588235	
chr28	10991696	2	34	T:0.941176	C:0.0588235	
chr28	10991738	2	34	A:0.941176	G:0.0588235	
chr28	10991766	2	34	A:0.882353	G:0.117647	
chr28	10991773	2	34	G:0.882353	A:0.117647	
chr28	10991800	2	36	A:0.472222	G:0.527778	
chr28	10991874	2	36	A:0.861111	C:0.138889	
chr28	10992336	2	32	C:0.9375	T:0.0625	
chr28	10992547	2	36	G:0.833333	A:0.166667	
chr28	10992571	2	36	C:0.888889	A:0.111111	
chr28	10992572	2	36	CG:0.888889	C:0.111111	
chr28	10992573	2	36	G:0.694444	C:0.305556	
chr28	10992700	2	32	T:0.3125	C:0.6875	
chr28	10992714	2	32	C:0.40625	T:0.59375	
chr28	10992715	2	32	G:1	A:0	

chr28 10992822 2 34 G:0.882353 GC:0.117647
chr28 10993329 2 38 GC:0.631579 G:0.368421
chr28 10993330 2 38 CCCCAG:0.473684 C:0.526316
chr28 10993332 2 38 C:0.894737 CTG:0.105263
chr28 10993333 2 38 CAG:0.894737 C:0.105263
chr28 10993335 2 38 G:0.631579 GC:0.368421
chr28 10993525 2 36 A:0.666667 G:0.333333
chr28 10993669 2 32 A:0.65625 G:0.34375
chr28 10993730 2 32 G:1 GGTGT:0
chr28 10993759 2 32 GTA:0.9375 G:0.0625
chr28 10993761 2 28 A:0.821429 G:0.178571
chr28 10993763 2 28 A:0.857143 G:0.142857
chr28 10993946 2 32 GA:0.4375 G:0.5625
chr28 10994209 2 36 TA:0.944444 T:0.0555556
chr28 10994309 2 34 C:0.529412 T:0.470588
chr28 10994311 2 34 A:0.529412 G:0.470588
chr28 10994906 2 36 G:0.333333 A:0.666667
chr28 10995117 2 38 G:1 A:0
chr28 10995247 2 34 T:0.529412 G:0.470588
chr28 10995395 2 34 C:0.323529 G:0.676471
chr28 10995495 2 36 A:0.5 G:0.5
chr28 10995595 2 36 A:0.638889 C:0.361111
chr28 10995777 2 30 CTG:0.433333 C:0.566667
chr28 10996035 2 28 G:0.678571 C:0.321429
chr28 10996211 2 34 A:0.676471 AG:0.323529
chr28 10996475 2 36 G:1 A:0
chr28 10996540 2 32 A:0.375 T:0.625
chr28 10996665 2 38 C:0.868421 T:0.131579
chr28 10996729 2 34 C:0.382353 T:0.617647
chr28 10996829 2 38 A:0.657895 G:0.342105
chr28 10996877 6 38 CGTGTGTGTGTGTGTGT:0.315789
C:0.526316 CGTGTGT:0.0263158 CGTGTGTGT:0.0789474
CGTGTGTGTGTGTGT:0 CGTGTGTGTGTGTGTGTGT:0.0526316
chr28 10996947 2 36 A:0.555556 G:0.444444
chr28 10997231 2 40 A:0.65 G:0.35
chr28 10997649 2 34 G:0.382353 GT:0.617647
chr28 10997759 2 38 A:0.289474 G:0.710526
chr28 10997837 2 36 T:0.555556 C:0.444444
chr28 10997840 2 34 GCC:1 G:0
chr28 10997871 2 36 C:0.916667 G:0.0833333
chr28 10997960 2 38 GGCTCTCTCTCCCCT:0.368421 G:
0.631579
chr28 10998658 2 36 ATG:1 A:0
chr28 10998674 2 36 G:0.555556 GT:0.444444
chr28 10998675 3 38 G:0.578947 GT:0.157895
GTGTGTGT:0.263158
chr28 10998719 2 34 C:0.647059 T:0.352941
chr28 10998878 2 28 A:0.678571 G:0.321429
chr28 10998898 2 28 T:0.678571 C:0.321429
chr28 10998904 2 28 T:0.678571 C:0.321429
chr28 10998976 2 24 G:0.125 C:0.875
chr28 10999062 2 24 C:0.375 CG:0.625
chr28 10999116 2 20 CGT:0.6 C:0.4
chr28 10999539 2 8 G:0 GT:1

chr28	11000448	2	32	G:0.8125	A:0.1875		
chr28	11000873	2	38	G:1	T:0		
chr28	11000897	2	38	G:0.921053	A:0.0789474		
chr28	11001070	2	38	G:1	T:0		
chr28	11001281	2	38	G:0.394737	A:0.605263		
chr28	11001339	2	34	A:0.441176	G:0.558824		
chr28	11001408	2	40	GTTCC:0.475	G:0.525		
chr28	11001623	2	32	T:0.90625	C:0.09375		
chr28	11001813	2	34	T:0.5	C:0.5		
chr28	11001908	2	34	C:0.764706	T:0.235294		
chr28	11001982	2	40	C:0.675	T:0.325		
chr28	11002146	2	34	A:1	AC:0		
chr28	11002454	2	40	AAACAAC:0.9	A:0.1		
chr28	11002457	2	40	C:0.425	T:0.575		
chr28	11002463	2	40	C:0.9	T:0.1		
chr28	11002469	2	40	CAACAACAAT:0.425	C:0.575		
chr28	11003002	4	36	A:0.333333	AT:0.0833333	ATT:0.5	ATTTT:0.0833333
chr28	11003098	2	38	A:0.710526	AACTAAATTTTGCTTATTAATAAAATTTTAAAC:0.289474		
chr28	11003273	2	38	AAAAT:1	A:0		
chr28	11003288	2	40	A:0.95	AT:0.05		
chr28	11003289	2	38	TAAA:1	T:0		
chr28	11003290	2	40	A:0.95	AATT:0.05		
chr28	11003293	2	38	TA:1	T:0		
chr28	11003294	2	38	A:0.947368	T:0.0526316		
chr28	11003334	2	34	A:0.911765	C:0.0882353		
chr28	11003363	2	38	A:0.631579	G:0.368421		
chr28	11003468	2	36	GTTTCTTTTCTTCC:0.972222	G:0.0277778		
chr28	11003555	2	40	T:0.625	TCTA:0.375		
chr28	11003575	2	40	A:0.675	G:0.325		
chr28	11003578	2	40	C:1	T:0		
chr28	11003603	2	34	C:0.970588	CT:0.0294118		
chr28	11003607	2	40	T:0.95	C:0.05		
chr28	11003633	3	38	C:0.421053	CAGAGAG:0.578947	CAGAGAGAGAG:0	
chr28	11003635	2	40	G:1	GAGAGAA:0		
chr28	11003644	2	36	T:0.416667	TGA:0.583333		
chr28	11003846	2	34	C:0.323529	CTT:0.676471		
chr28	11004025	2	28	G:1	A:0		
chr28	11004046	2	28	G:1	A:0		
chr28	11004192	2	36	C:0.611111	T:0.388889		
chr28	11004311	2	36	G:0.361111	A:0.638889		
chr28	11004606	2	32	A:1	G:0		
chr28	11004722	2	30	G:0.633333	A:0.366667		
chr28	11004801	2	40	C:1	A:0		
chr28	11005123	2	38	T:0.421053	C:0.578947		
chr28	11005235	2	32	C:0.4375	T:0.5625		
chr28	11005298	2	40	A:0.625	G:0.375		
chr28	11005327	2	40	G:1	C:0		
chr28	11005435	2	34	T:0.617647	C:0.382353		
chr28	11005509	2	40	G:1	A:0		
chr28	11005663	2	36	G:0.444444	C:0.555556		

chr28	11005676	2	36	G:0.361111	A:0.638889		
chr28	11006028	2	38	T:0.631579	C:0.368421		
chr28	11006142	2	34	C:0.382353	T:0.617647		
chr28	11006203	2	36	T:0.333333	C:0.666667		
chr28	11006223	4	40	C:0.375	CTGAA:0	CTGAATGAACGAA:0.525	CTGAATGAACGAATGAA:0.1
chr28	11006246	2	40	G:0.375	A:0.625		
chr28	11006397	2	34	C:0.529412	CT:0.470588		
chr28	11006726	2	32	T:0.375	C:0.625		
chr28	11006737	2	32	AC:1	A:0		
chr28	11006739	2	30	C:0.4	T:0.6		
chr28	11006741	2	32	C:1	T:0		
chr28	11006750	2	28	G:0.428571	A:0.571429		
chr28	11006751	2	28	C:0.428571	T:0.571429		
chr28	11006837	2	36	A:0.888889	C:0.111111		
chr28	11006936	2	36	C:0.388889	T:0.611111		
chr28	11006979	2	32	A:0.65625	G:0.34375		
chr28	11006991	2	32	A:0.6875	G:0.3125		
chr28	11007002	2	34	G:0.705882	GGA:0.294118		
chr28	11007058	2	32	A:0.71875	T:0.28125		
chr28	11007138	2	36	C:0.583333	T:0.416667		
chr28	11007215	2	36	T:0.361111	C:0.638889		
chr28	11007601	2	36	A:0.638889	C:0.361111		
chr28	11007664	2	38	A:0.631579	T:0.368421		
chr28	11007697	2	36	G:0.583333	T:0.416667		
chr28	11007727	2	34	AG:0.588235	A:0.411765		
chr28	11007782	2	36	A:0.638889	G:0.361111		
chr28	11007923	2	36	C:0.388889	G:0.611111		
chr28	11008346	2	34	T:0.323529	C:0.676471		
chr28	11008356	2	38	G:0.394737	GC:0.605263		
chr28	11008376	2	38	A:0.421053	G:0.578947		
chr28	11008391	2	36	T:0.388889	A:0.611111		
chr28	11008404	2	34	T:1	G:0		
chr28	11008522	2	36	A:0.222222	G:0.777778		
chr28	11008580	2	40	G:0.375	A:0.625		
chr28	11008589	2	40	A:0.375	G:0.625		
chr28	11008669	2	36	AT:0.305556	A:0.694444		
chr28	11008980	2	34	A:0.558824	G:0.441176		
chr28	11008999	2	34	G:0.352941	A:0.647059		
chr28	11009011	2	34	AG:1	A:0		
chr28	11009048	2	36	T:0.694444	C:0.305556		
chr28	11009258	2	34	G:0.558824	A:0.441176		
chr28	11009314	2	34	G:0.735294	A:0.264706		
chr28	11009484	2	36	G:0.361111	C:0.638889		
chr28	11009729	2	34	G:0.852941	A:0.147059		
chr28	11009792	2	34	T:0.264706	C:0.735294		
chr28	11009928	2	36	A:1	C:0		
chr28	11010161	2	38	A:0.368421	G:0.631579		
chr28	11010227	2	36	C:0.583333	A:0.416667		
chr28	11010228	2	36	A:1	C:0		
chr28	11010301	2	30	G:0.4	C:0.6		
chr28	11010623	2	32	C:0.28125	T:0.71875		
chr28	11010763	2	38	A:0.710526	G:0.289474		
chr28	11010769	2	38	A:0.710526	G:0.289474		

chr28 11010775 2 38 AAG:1 A:0
chr28 11010793 2 38 C:1 T:0
chr28 11010810 2 40 C:0.675 CCTTGGG:0.325
chr28 11010926 2 34 T:0.705882 C:0.294118
chr28 11010949 2 34 A:0.705882 T:0.294118
chr28 11011061 2 34 C:1 T:0
chr28 11011219 2 28 T:0.5 C:0.5
chr28 11011252 2 38 G:1 A:0
chr28 11011267 2 38 G:0.421053 A:0.578947
chr28 11011340 3 40 TAA:0.35 T:0.65 TAAAAAA:0
chr28 11011350 2 40 TAAAA:0.35 T:0.65
chr28 11011559 2 34 G:0.411765 A:0.588235
chr28 11011876 2 32 C:1 T:0
chr28 11011927 2 36 C:0.305556 CCT:0.694444
chr28 11011978 3 34 T:0.5 TA:0.0294118 TAA:
0.470588
chr28 11012037 2 38 A:0.394737 ATGTAGGCAGAGG:
0.605263
chr28 11012378 2 36 A:0.666667 G:0.333333
chr28 11012386 2 36 A:0.666667 G:0.333333
chr28 11012542 2 38 G:0.684211 C:0.315789
chr28 11013044 2 38 A:0.315789 C:0.684211
chr28 11013094 2 36 G:0.694444 T:0.305556
chr28 11013431 2 22 G:0.909091 T:0.0909091
chr28 11013511 2 32 C:0.375 T:0.625
chr28 11013592 2 22 G:0.954545 A:0.0454545
chr28 11013647 2 26 TTG:0.192308 T:0.807692
chr28 11013660 2 26 G:0.192308 C:0.807692
chr28 11014025 2 38 C:0.368421 G:0.631579
chr28 11014797 2 30 A:0.766667 G:0.233333
chr28 11015379 2 36 T:0.361111 C:0.638889
chr28 11015536 2 38 C:1 T:0
chr28 11015724 2 32 C:1 T:0
chr28 11015901 2 38 A:1 T:0
chr28 11015942 4 38 C:0.973684 CAG:0 CAGAGAG:
0 CAGAGAGAG:0.0263158
chr28 11015944 4 38 C:0.578947 G:0.0263158
CAGAGAGAG:0.342105 CAGAGAGAGAG:0.0526316
chr28 11015946 2 38 C:0.5 G:0.5
chr28 11016331 2 34 C:0.5 T:0.5
chr28 11016384 2 36 C:0.527778 T:0.472222
chr28 11016567 2 36 C:1 T:0
chr28 11016701 2 20 GGGT:0.75 G:0.25
chr28 11016702 2 20 GGT:0.35 G:0.65
chr28 11017185 2 30 T:1 C:0
chr28 11017239 2 32 G:0.4375 A:0.5625
chr28 11017242 2 34 G:0.529412 A:0.470588
chr28 11017398 2 32 T:1 C:0
chr28 11017488 2 38 C:0.552632 G:0.447368
chr28 11017968 2 38 C:0.394737 T:0.605263
chr28 11018133 2 30 C:0.4 T:0.6
chr28 11018217 2 38 C:1 T:0
chr28 11018324 2 32 GT:1 G:0
chr28 11018561 2 36 C:0.972222 T:0.0277778

chr28	11018591	2	32	C:1	T:0	
chr28	11018792	2	30	A:0.533333	C:0.466667	
chr28	11018807	3	38	TTTTA:0.473684	T:0.394737	TTTTATTTA:0.131579
chr28	11018811	2	34	AT:1	A:0	
chr28	11019252	2	40	G:1	A:0	
chr28	11019519	2	36	A:0.388889	G:0.611111	
chr28	11019735	2	40	C:0.35	T:0.65	
chr28	11020123	2	36	G:0.972222	A:0.0277778	
chr28	11020179	2	36	T:1	C:0	
chr28	11020202	2	36	GT:0.638889	G:0.361111	
chr28	11020349	2	32	G:0.25	GT:0.75	
chr28	11020376	2	32	C:0.96875	T:0.03125	
chr28	11020781	2	30	G:1	A:0	
chr28	11021012	2	38	G:0.605263	T:0.394737	
chr28	11021017	2	38	G:0.605263	A:0.394737	
chr28	11021468	2	36	G:1	A:0	
chr28	11021915	2	40	T:0.35	G:0.65	
chr28	11022233	2	36	C:0.583333	T:0.416667	
chr28	11022346	2	38	A:1	G:0	
chr28	11022767	2	30	A:1	G:0	
chr28	11023439	2	38	G:0.447368	T:0.552632	
chr28	11023556	2	38	C:1	T:0	
chr28	11023666	2	38	T:0.657895	C:0.342105	
chr28	11023689	2	38	T:0.657895	C:0.342105	
chr28	11023703	2	38	G:1	A:0	
chr28	11023783	2	40	C:1	T:0	
chr28	11024066	2	36	G:0.611111	C:0.388889	
chr28	11024127	2	38	T:1	A:0	
chr28	11024250	2	38	T:0.368421	G:0.631579	
chr28	11024596	2	32	C:1	T:0	
chr28	11024783	2	32	A:0.59375	AC:0.40625	
chr28	11025055	2	38	C:1	T:0	
chr28	11025116	2	38	T:0.473684	G:0.526316	
chr28	11025149	2	38	CA:0.894737	C:0.105263	
chr28	11025326	2	40	AG:0.625	A:0.375	
chr28	11025647	2	38	G:1	A:0	
chr28	11025713	2	36	C:0.305556	T:0.694444	
chr28	11026043	2	32	C:0.59375	CTA:0.40625	
chr28	11026217	2	36	G:0.416667	GC:0.583333	
chr28	11026485	2	36	G:1	A:0	
chr28	11026700	2	36	T:0.527778	C:0.472222	
chr28	11026905	2	34	G:1	A:0	
chr28	11027254	2	34	G:0.382353	A:0.617647	
chr28	11027319	2	38	C:1	T:0	
chr28	11027331	2	36	TG:0.361111	T:0.638889	
chr28	11027534	2	32	C:1	T:0	
chr28	11027768	2	36	C:0.638889	G:0.361111	
chr28	11027837	2	36	T:0.583333	C:0.416667	
chr28	11028083	2	36	T:0.888889	G:0.111111	
chr28	11028211	2	40	T:0.95	C:0.05	
chr28	11028231	2	40	A:0.95	G:0.05	
chr28	11028232	2	40	G:0.95	C:0.05	
chr28	11028451	2	36	T:0.416667	C:0.583333	

chr28	11028454	2	36	C:0.416667	T:0.583333		
chr28	11028918	2	40	A:0.975	G:0.025		
chr28	11029112	2	38	C:0.921053	T:0.0789474		
chr28	11029411	2	38	C:1	T:0		
chr28	11029473	2	36	T:0.972222	A:0.0277778		
chr28	11029699	2	34	C:0.911765	T:0.0882353		
chr28	11029723	2	32	T:1	C:0		
chr28	11029790	2	20	G:0.3	C:0.7		
chr28	11029936	2	24	A:0.583333	G:0.416667		
chr28	11030002	2	32	A:0.46875	G:0.53125		
chr28	11030312	2	36	T:0.5	C:0.5		
chr28	11030318	2	34	G:1	T:0		
chr28	11030336	2	30	A:1	G:0		
chr28	11030502	2	38	C:0.421053	CT:0.578947		
chr28	11030527	2	40	C:1	T:0		
chr28	11030746	2	36	T:0.527778	C:0.472222		
chr28	11031145	2	38	G:0.421053	A:0.578947		
chr28	11031243	2	30	A:0.566667	G:0.433333		
chr28	11031297	2	36	A:0.472222	G:0.527778		
chr28	11031331	2	38	C:0.447368	T:0.552632		
chr28	11031449	2	38	G:0.789474	A:0.210526		
chr28	11031494	2	38	C:0.5	G:0.5		
chr28	11031525	2	38	G:0.526316	A:0.473684		
chr28	11031693	2	38	G:1	A:0		
chr28	11031743	2	36	A:0.388889	C:0.611111		
chr28	11031794	2	40	T:1	C:0		
chr28	11031909	2	32	T:0.5	A:0.5		
chr28	11031910	2	32	C:0.5	T:0.5		
chr28	11031948	2	38	T:0.368421	C:0.631579		
chr28	11031967	2	38	C:0.578947	T:0.421053		
chr28	11032012	2	40	C:1	T:0		
chr28	11032121	2	36	GTATAAGA:0.527778	G:0.472222		
chr28	11032172	2	36	T:0.555556	C:0.444444		
chr28	11032245	2	36	T:0.555556	G:0.444444		
chr28	11032317	2	24	T:0.208333	A:0.791667		
chr28	11032686	2	36	C:0.5	CTTT:0.5		
chr28	11032702	4	40	TTTTA:0.65	T:0.075	TTTTATTTA:0.1	TTTTATTTATTTATTTATTTA:0.175
chr28	11032740	2	30	C:0.533333	CAG:0.466667		
chr28	11032795	2	34	G:0.588235	A:0.411765		
chr28	11032824	2	36	C:0.527778	T:0.472222		
chr28	11032875	2	36	T:0.472222	C:0.527778		
chr28	11032919	2	40	TCTTTATCTAGTATGATCTCTAGACACTTTCTTTGC:1	T:0		
chr28	11032957	2	40	T:1	C:0		
chr28	11033126	2	38	C:0.421053	T:0.578947		
chr28	11033139	2	38	G:0.421053	A:0.578947		
chr28	11033239	2	40	A:0.925	C:0.075		
chr28	11033276	2	34	T:0.529412	C:0.470588		
chr28	11033358	2	40	G:1	A:0		
chr28	11033557	2	36	C:1	T:0		
chr28	11033749	2	36	T:0.444444	G:0.555556		
chr28	11033753	2	36	T:0.444444	C:0.555556		

chr28	11034008	2	36	C:0.444444	T:0.555556
chr28	11034015	2	36	G:0.416667	A:0.583333
chr28	11034065	2	38	C:1	T:0
chr28	11034156	2	36	C:0.972222	T:0.0277778
chr28	11034325	2	40	G:0.55	A:0.45
chr28	11034469	2	24	C:0.916667	CTTT:0.0833333
chr28	11034523	2	36	CTT:0.611111	C:0.388889
chr28	11034636	2	34	C:0.588235	T:0.411765
chr28	11034645	2	38	G:0.973684	GT:0.0263158
chr28	11034647	2	38	GCCCCAACC:0.973684	G:
0.0263158
chr28	11034970	2	38	G:0.973684	A:0.0263158
chr28	11035099	2	36	G:0.5	A:0.5
chr28	11035355	2	36	G:1	A:0
chr28	11035439	2	40	C:1	T:0
chr28	11035764	2	34	G:0.558824	A:0.441176
chr28	11035877	2	36	C:0.555556	T:0.444444
chr28	11036037	2	36	C:0.861111	T:0.138889
chr28	11036374	2	36	C:0.444444	T:0.555556
chr28	11036470	2	38	G:0.552632	A:0.447368
chr28	11036491	2	40	C:1	T:0
chr28	11036637	2	38	C:0.526316	A:0.473684
chr28	11036639	2	38	T:0.605263	G:0.394737
chr28	11036705	2	38	G:0.973684	A:0.0263158
chr28	11036874	2	36	C:0.305556	G:0.694444
chr28	11037064	2	38	G:0.447368	A:0.552632
chr28	11037211	2	38	T:0.631579	C:0.368421
chr28	11037431	2	40	G:0.9	A:0.1
chr28	11037491	2	38	CTTTTCT:1	C:0
chr28	11037506	2	38	T:0.684211	C:0.315789
chr28	11037508	2	38	C:0.684211	T:0.315789
chr28	11037518	2	38	C:0.684211	T:0.315789
chr28	11037522	4	40	C:0.575	T:0.3
CTTTCTTTCTTTCTTCTTTTTTTCTTTCTTTCT:0.025
CTTTCTTTCTTTCTTTCTTTCTTTCTTCTTTTTTTCTTTCTTTCTT:0.1
chr28	11037524	2	38	T:0.684211	C:0.315789
chr28	11037538	2	40	A:0.875	T:0.125
chr28	11037815	2	36	G:0.583333	C:0.416667
chr28	11037838	2	38	C:0.973684	T:0.0263158
chr28	11037916	2	32	A:0.96875	T:0.03125
chr28	11037997	2	38	G:1	A:0
chr28	11038002	2	38	T:0.605263	A:0.394737
chr28	11038011	2	38	T:0	C:1
chr28	11038194	2	24	G:1	A:0
chr28	11038344	2	40	ATTCT:1	A:0
chr28	11038412	2	40	C:0.975	T:0.025
chr28	11038442	2	40	G:1	A:0
chr28	11038526	2	36	A:1	G:0
chr28	11038578	2	38	G:0.973684	A:0.0263158
chr28	11038649	2	38	A:1	G:0
chr28	11038656	2	38	A:1	C:0
chr28	11038726	2	40	G:1	A:0
chr28	11038746	2	40	CT:1	C:0
chr28	11038767	2	38	T:0.526316	C:0.473684

chr28	11038955	2	34	C:1	T:0	
chr28	11039000	2	32	A:1	T:0	
chr28	11039001	2	32	G:0.59375		C:0.40625
chr28	11039040	2	34	A:1	G:0	
chr28	11039042	2	34	A:1	G:0	
chr28	11039119	2	38	T:1	G:0	
chr28	11039147	2	40	G:1	A:0	
chr28	11039175	2	36	G:0.972222		A:0.0277778
chr28	11039213	2	38	G:0.947368		A:0.0526316
chr28	11039336	2	36	C:1	T:0	
chr28	11039421	2	38	G:0.973684		A:0.0263158
chr28	11039444	2	38	A:0.973684		G:0.0263158
chr28	11039502	2	36	G:0.972222		GAAGTCCCCT:<br>0.0277778
chr28	11039705	2	34	C:1	T:0	
chr28	11039750	2	36	G:1	T:0	
chr28	11039822	2	38	G:0.947368		A:0.0526316
chr28	11039827	2	38	G:0.421053		C:0.578947
chr28	11039882	2	40	C:1	T:0	
chr28	11039902	2	38	G:0.947368		A:0.0526316
chr28	11039922	2	38	G:1	A:0	
chr28	11039935	2	36	G:0.916667		A:0.0833333
chr28	11039942	2	38	G:0.947368		A:0.0526316
chr28	11040024	2	34	G:1	A:0	
chr28	11040055	2	36	A:0.416667		C:0.583333
chr28	11040084	2	38	C:0.973684		A:0.0263158
chr28	11040134	2	40	T:0.4	C:0.6	
chr28	11040220	2	32	A:1	G:0	
chr28	11040227	2	32	G:1	A:0	
chr28	11040244	2	30	A:1	T:0	
chr28	11040316	2	38	A:0.973684		G:0.0263158
chr28	11040349	2	36	C:0.972222		T:0.0277778
chr28	11040436	2	38	T:0.552632		C:0.447368
chr28	11040688	2	34	G:0.970588		C:0.0294118
chr28	11040769	2	22	T:0.409091		C:0.590909
chr28	11040994	2	34	T:0.529412		C:0.470588
chr28	11041095	2	30	A:0.866667		C:0.133333
chr28	11041311	2	36	G:0.972222		A:0.0277778
chr28	11041433	2	38	C:0.447368		T:0.552632
chr28	11041590	2	40	G:1	A:0	
chr28	11041594	2	40	C:1	T:0	
chr28	11041634	2	34	A:0.970588		G:0.0294118
chr28	11041754	2	36	C:0.888889		T:0.111111
chr28	11041778	2	38	T:0.473684		C:0.526316
chr28	11041959	2	34	T:0.470588		C:0.529412
chr28	11042130	2	34	C:0.882353		A:0.117647
chr28	11042173	2	38	G:0.473684		A:0.526316
chr28	11042503	2	38	C:1	T:0	
chr28	11042585	2	36	T:0.916667		A:0.0833333
chr28	11042646	2	34	T:0.911765		A:0.0882353
chr28	11042665	2	36	A:0.916667		G:0.0833333
chr28	11042676	2	36	A:0.916667		G:0.0833333
chr28	11042708	2	36	T:0.361111		C:0.638889
chr28	11042715	2	36	T:0.777778		C:0.222222

chr28 11042733 2 36 C:0.805556 T:0.194444
chr28 11042792 2 36 A:0.888889 G:0.111111
chr28 11042800 2 38 C:0.342105 CA:0.657895
chr28 11042878 2 32 C:0.875 T:0.125
chr28 11042885 2 32 A:0.875 G:0.125
chr28 11042902 2 32 A:0.875 T:0.125
chr28 11042903 2 32 T:0.875 C:0.125
chr28 11042925 2 30 G:0.866667 GT:0.133333
chr28 11043013 2 30 C:0.933333 T:0.0666667
chr28 11043039 2 38 G:0.947368 C:0.0526316
chr28 11043040 2 38 G:0.947368 T:0.0526316
chr28 11043061 2 38 A:0.947368 G:0.0526316
chr28 11043217 2 36 C:0.888889 T:0.111111
chr28 11043288 2 30 T:0.5 G:0.5
chr28 11043364 2 36 C:0.944444 T:0.0555556
chr28 11043430 2 36 A:0.972222 G:0.0277778
chr28 11043466 2 36 C:0.944444 T:0.0555556
chr28 11043573 2 34 G:0.882353 A:0.117647
chr28 11043743 2 36 T:0.805556 C:0.194444
chr28 11043747 2 36 G:0.805556 A:0.194444
chr28 11043767 2 36 C:0.888889 A:0.111111
chr28 11043779 2 36 A:0.805556 G:0.194444
chr28 11043785 2 38 G:0.947368 A:0.0526316
chr28 11043879 2 40 C:0.85 A:0.15
chr28 11043889 2 40 C:0.85 G:0.15
chr28 11043968 2 38 C:0.973684 A:0.0263158
chr28 11044034 2 36 C:0.805556 T:0.194444
chr28 11044071 2 38 A:0.894737 G:0.105263
chr28 11044072 2 38 T:0.894737 C:0.105263
chr28 11044133 2 38 A:0.5 ACCCAGGGGTAAG:0.5
chr28 11044260 2 34 G:0.441176 C:0.558824
chr28 11044291 2 30 G:0.966667 A:0.0333333
chr28 11044351 2 38 A:0.868421 T:0.131579
chr28 11044353 2 38 C:0.973684 T:0.0263158
chr28 11044494 2 36 G:0.861111 A:0.138889
chr28 11044534 2 36 A:0.972222 G:0.0277778
chr28 11044630 2 36 G:0.638889 C:0.361111
chr28 11044814 2 34 CCT:0.970588 C:0.0294118
chr28 11044910 2 34 G:1 A:0
chr28 11044915 2 34 G:1 T:0
chr28 11044953 2 36 T:0.611111
TGAGACTGAATTTTTTTTTTTTTTTTTTGGAA:0.388889
chr28 11044981 3 38 G:0.947368 GGT:0.0526316
GGTGT:0
chr28 11044996 2 34 GTA:0.823529 G:0.176471
chr28 11044998 2 34 A:0.705882 G:0.294118
chr28 11045201 2 36 C:1 T:0
chr28 11045229 2 36 G:0.555556 T:0.444444
chr28 11045441 2 36 G:0.583333 C:0.416667
chr28 11045483 2 36 G:1 A:0
chr28 11045564 2 38 T:0.842105 C:0.157895
chr28 11045598 2 40 G:0.975 A:0.025
chr28 11045608 2 40 A:0.45 T:0.55
chr28 11045649 2 40 A:0.325 C:0.675

chr28	11045652	2	40	C:0.5	T:0.5	
chr28	11045820	2	34	C:0.529412	T:0.470588	
chr28	11045837	2	36	G:1	A:0	
chr28	11045846	2	34	C:0.941176	T:0.0588235	
chr28	11045875	2	34	T:0.441176	C:0.558824	
chr28	11045956	2	32	G:0.9375	A:0.0625	
chr28	11046093	2	36	A:0.527778	T:0.472222	
chr28	11046283	2	38	C:0.947368	G:0.0526316	
chr28	11046314	3	36	TTTTA:0.75	T:0.111111	TTTTATTTA:0.138889
chr28	11046366	2	38	T:0.736842	TTTTATTTATTTATTTATTTATTTA:0.263158	
chr28	11046734	2	40	C:0.875	T:0.125	
chr28	11046745	2	40	C:0.875	T:0.125	
chr28	11046806	2	38	A:1	G:0	
chr28	11046821	2	34	G:0.705882	A:0.294118	
chr28	11047302	2	36	C:0.5	T:0.5	
chr28	11047309	2	34	C:0.970588	T:0.0294118	
chr28	11047492	2	38	G:0.552632	A:0.447368	
chr28	11047710	2	36	C:1	T:0	
chr28	11047757	2	36	A:0.305556	G:0.694444	
chr28	11047883	2	40	G:0.35	T:0.65	
chr28	11047960	2	36	A:0.916667	T:0.0833333	
chr28	11047997	2	34	T:0.882353	G:0.117647	
chr28	11048566	2	36	A:0.888889	C:0.111111	
chr28	11048570	2	36	A:0.416667	G:0.583333	
chr28	11048798	2	34	A:0.323529	G:0.676471	
chr28	11048852	2	40	C:1	T:0	
chr28	11048868	2	38	C:0.394737	A:0.605263	
chr28	11048974	2	36	C:0.527778	A:0.472222	
chr28	11049105	2	36	C:1	T:0	
chr28	11049165	2	36	GA:0.388889	G:0.611111	
chr28	11049177	2	36	A:0.388889	G:0.611111	
chr28	11049178	2	36	A:0.388889	T:0.611111	
chr28	11049225	2	34	C:1	T:0	
chr28	11049282	2	36	A:0.916667	AG:0.0833333	
chr28	11049309	3	40	TAAAAACAAAAAC:0.525	T:0.1	TAAAAAC:0.375
chr28	11049314	2	40	A:0.925	G:0.075	
chr28	11049392	2	36	C:0.861111	CCTT:0.138889	
chr28	11049477	2	36	C:0.777778	G:0.222222	
chr28	11049681	2	32	T:1	C:0	
chr28	11049791	2	36	T:0.944444	C:0.0555556	
chr28	11049831	2	38	T:0.789474	C:0.210526	
chr28	11049855	2	38	T:0.789474	C:0.210526	
chr28	11050086	2	36	A:0.805556	C:0.194444	
chr28	11050104	2	36	C:0.805556	T:0.194444	
chr28	11050151	2	38	T:0.815789	C:0.184211	
chr28	11050468	2	36	A:0.888889	G:0.111111	
chr28	11050712	2	36	G:1	A:0	
chr28	11050994	2	34	T:0.470588	A:0.529412	
chr28	11051063	2	34	C:0.588235	T:0.411765	
chr28	11051264	2	38	C:0.868421	T:0.131579	
chr28	11051347	2	36	AT:1	A:0	

chr28	11051551	2	34	AC:1	A:0		
chr28	11051910	2	20	C:0.85	CT:0.15		
chr28	11051955	2	32	C:1	T:0		
chr28	11052728	2	36	C:1	T:0		
chr28	11052738	2	36	C:1	T:0		
chr28	11052817	2	38	T:1	C:0		
chr28	11052838	2	40	TTTTCTTTC:1	T:0		
chr28	11052898	2	40	CTTTCTTTCTTTCT:1	C:0		
chr28	11052902	2	40	CTTTCTTTCT:1	C:0		
chr28	11052911	2	40	T:0.975	TTTC:0.025		
chr28	11053169	2	36	C:0.916667	T:0.0833333		
chr28	11053300	2	34	G:1	A:0		
chr28	11054904	2	36	T:0.5	C:0.5		
chr28	11055340	3	34	CT:0.647059	C:0.235294	CTT:0.117647	
chr28	11057389	2	36	A:0.583333	G:0.416667		
chr28	11057902	2	38	T:1	C:0		
chr28	11058160	2	40	C:1	T:0		
chr28	11058630	4	36	ATTT:0.388889	A:0.0833333	AT:0.416667	ATT:0.111111
chr28	11058660	2	36	T:0.888889	C:0.111111		
chr28	11059767	2	30	T:0.433333	TG:0.566667		
chr28	11060285	2	36	C:1	T:0		
chr28	11060514	2	38	C:0.263158	T:0.736842		
chr28	11060525	2	38	C:0.921053	CA:0.0789474		
chr28	11061225	2	36	G:1	A:0		
chr28	11062055	2	38	T:0.894737	A:0.105263		
chr28	11062158	2	38	G:0.578947	A:0.421053		
chr28	11062245	2	40	T:0.5	C:0.5		
chr28	11062255	2	40	A:0.375	C:0.625		
chr28	11062310	2	36	G:0.527778	A:0.472222		
chr28	11062417	2	38	T:0.578947	TA:0.421053		
chr28	11062567	2	38	T:0.578947	C:0.421053		
chr28	11062762	2	38	C:0.605263	T:0.394737		
chr28	11062783	2	38	C:0.605263	G:0.394737		
chr28	11062834	2	34	A:0.441176	T:0.558824		
chr28	11062874	2	36	G:0.916667	C:0.0833333		
chr28	11063078	2	38	A:0	T:1		
chr28	11063083	2	34	T:0.588235	G:0.411765		
chr28	11063122	2	38	T:0.526316	C:0.473684		
chr28	11063134	2	38	T:0.526316	C:0.473684		
chr28	11063207	2	34	C:0.617647	T:0.382353		
chr28	11063288	2	38	T:0.578947	A:0.421053		
chr28	11063324	2	40	G:0.6	A:0.4		
chr28	11063335	2	40	C:0.725	T:0.275		
chr28	11063349	2	40	A:0.6	G:0.4		
chr28	11063403	2	36	A:0.611111	G:0.388889		
chr28	11063568	2	38	G:1	A:0		
chr28	11063647	2	40	A:0.4	G:0.6		
chr28	11063753	2	38	C:0.447368	A:0.552632		
chr28	11063772	2	38	TGTTA:0.447368	T:0.552632		
chr28	11063953	2	36	G:0.416667	GTGTC:0.583333		
chr28	11064493	2	28	T:0.464286	C:0.535714		
chr28	11064863	3	32	TAA:0.375	T:0.5625	TA:	

0.0625
chr28	11064942	2	34	T:1	A:0
chr28	11065209	2	30	A:0.933333	G:0.0666667
chr28	11066114	2	26	CT:0.307692	C:0.692308
chr28	11066583	2	38	A:0.631579	G:0.368421
chr28	11066856	2	40	A:0	T:1
chr28	11066857	2	40	A:0	G:1
chr28	11066898	2	38	A:0	G:1
chr28	11066926	2	36	A:0.555556	AC:0.444444
chr28	11067087	2	36	A:0.861111	G:0.138889
chr28	11067397	2	36	C:1	T:0
chr28	11067465	2	36	T:0	C:1
chr28	11067559	2	36	G:0.972222	T:0.0277778
chr28	11067786	2	36	T:0.416667	C:0.583333
chr28	11068293	2	38	A:0.526316	G:0.473684
chr28	11068294	2	38	G:0.342105	GGA:0.657895
chr28	11068433	2	38	T:0	G:1
chr28	11068476	2	36	CTG:0.638889	C:0.361111
chr28	11068486	2	36	T:0	C:1
chr28	11068487	2	36	T:0	C:1
chr28	11068648	2	22	AT:0.863636	A:0.136364
chr28	11068658	2	22	T:1	A:0
chr28	11069264	2	36	G:0.861111	C:0.138889
chr28	11069542	2	34	G:0.882353	C:0.117647
chr28	11069630	2	38	GCTGGCAAGTGCCTGGGGC:0.684211
G:0.315789
chr28	11069722	2	38	G:1	A:0
chr28	11070083	2	40	AT:1	A:0
chr28	11070732	2	36	C:1	T:0
chr28	11070951	2	32	GT:0.71875	G:0.28125
chr28	11071678	2	36	T:0.444444	C:0.555556
chr28	11071878	2	36	C:1	T:0
chr28	11071976	2	38	C:0.684211	T:0.315789
chr28	11072114	2	36	A:0.833333	T:0.166667
chr28	11072137	2	36	A:0.833333	G:0.166667
chr28	11072430	2	34	T:0.558824	C:0.441176
chr28	11072492	3	28	CTT:0.5	C:0.142857	CT:
0.357143
chr28	11072543	2	38	GAC:1	G:0
chr28	11072547	3	40	CACAG:0.775	C:0
CAGAGAGAGACAG:0.225
chr28	11072549	3	38	CAGAGAG:0.184211	C:0.394737
GAGAGAG:0.421053
chr28	11072840	2	26	G:0.269231	T:0.730769
chr28	11073413	2	40	T:0.625	A:0.375
chr28	11073415	2	40	G:0.625	A:0.375
chr28	11073659	2	34	C:1	T:0
chr28	11073995	2	30	GCTT:0.533333	G:0.466667
chr28	11073998	2	36	T:1	G:0
chr28	11073999	2	36	CTT:1	C:0
chr28	11074002	2	36	T:0.805556	C:0.194444
chr28	11074091	2	34	A:1	G:0
chr28	11074125	2	36	A:0.638889	G:0.361111
chr28	11074183	2	36	C:1	T:0

chr28	11074258	2	40	C:1	T:0	
chr28	11074371	2	38	C:1	CG:0	
chr28	11074479	2	38	A:0.921053	G:0.0789474	
chr28	11074654	2	40	G:1	A:0	
chr28	11074671	2	40	GGC:0.575	G:0.425	
chr28	11074800	2	36	G:0.694444	A:0.305556	
chr28	11074844	2	36	GGT:0.861111	G:0.138889	
chr28	11074860	2	36	G:0.916667	A:0.0833333	
chr28	11074980	2	40	G:1	C:0	
chr28	11075230	2	34	A:1	G:0	
chr28	11075318	2	32	A:1	G:0	
chr28	11075428	2	32	C:1	T:0	
chr28	11075439	2	30	C:1	G:0	
chr28	11075530	2	38	C:0.973684	T:0.0263158	
chr28	11075544	2	38	ATAAGT:0.973684	A:0.0263158	
chr28	11075654	2	36	A:0.888889	AT:0.111111	
chr28	11075655	2	36	A:0.888889	T:0.111111	
chr28	11075670	2	34	C:0.529412	T:0.470588	
chr28	11075741	3	38	TTTATTTA:0.894737	TTTTA:0.0789474	T:0.0263158
chr28	11075748	3	38	ATTTTT:0.473684	A:0.0789474	ATTTTTT:0.447368
chr28	11075791	2	32	A:1	C:0	
chr28	11075888	2	36	G:0.944444	C:0.0555556	
chr28	11075926	2	36	A:0.472222	G:0.527778	
chr28	11075938	2	36	T:1	G:0	
chr28	11075981	2	30	T:0.966667	TC:0.0333333	
chr28	11076189	2	32	AT:1	A:0	
chr28	11076227	2	30	G:1	T:0	
chr28	11076236	2	30	T:1	TG:0	
chr28	11076317	2	34	G:1	C:0	
chr28	11076385	2	36	G:1	A:0	
chr28	11076630	2	34	T:1	C:0	
chr28	11076714	2	40	G:1	A:0	
chr28	11076770	2	38	G:1	A:0	
chr28	11076911	2	38	G:1	A:0	
chr28	11076921	2	38	T:1	A:0	
chr28	11076937	2	36	C:1	T:0	
chr28	11076994	2	36	GCACTGC:0.972222	G:0.0277778	
chr28	11077054	2	38	C:0.894737	A:0.105263	
chr28	11077133	2	36	C:0.944444	T:0.0555556	
chr28	11077297	2	36	C:0.805556	T:0.194444	
chr28	11077318	2	38	A:0.342105	G:0.657895	
chr28	11077319	2	38	T:1	C:0	
chr28	11077447	2	38	T:1	TCCAGGCACAGAGCTACCAA:0	
chr28	11077561	2	32	C:1	T:0	
chr28	11077680	2	34	CTT:1	C:0	
chr28	11077780	2	34	G:1	A:0	
chr28	11077863	2	36	C:0.555556	A:0.444444	
chr28	11077910	2	40	G:1	C:0	
chr28	11078064	2	38	GA:0.657895	G:0.342105	
chr28	11078201	2	36	T:0.527778	C:0.472222	
chr28	11078236	2	38	A:0.631579	G:0.368421	
chr28	11078310	2	34	A:0.470588	G:0.529412	

chr28 11078317 2 34 A:0.647059 C:0.352941
chr28 11078404 2 38 C:0 CG:1
chr28 11078422 2 36 C:0.722222 CGGGCTGCTGTG:
0.277778
chr28 11078501 2 28 A:0.642857 C:0.357143
chr28 11078804 2 36 T:0.527778 C:0.472222
chr28 11078812 2 38 CGGAGGGAGGGTGACTGGCCCG:0.631579
C:0.368421
chr28 11078843 2 38 A:0.0263158 G:0.973684
chr28 11078939 2 34 C:0.941176 T:0.0588235
chr28 11078991 2 34 A:0.970588 G:0.0294118
chr28 11079200 2 38 A:0.921053 G:0.0789474
chr28 11079493 2 40 A:0.925
AATGAGTGCGTGTGCATTCAGTGTAT:0.075
chr28 11079599 2 34 C:0.970588 T:0.0294118
chr28 11079620 2 32 G:0.65625 A:0.34375
chr28 11079735 2 38 C:0.5 T:0.5
chr28 11079947 2 40 T:0.95 C:0.05
chr28 11080056 2 38 G:1 C:0
chr28 11080401 2 30 A:1 G:0
chr28 11080539 2 38 ACAG:1 A:0
chr28 11080543 2 38 G:1 GTGATGA:0
chr28 11080749 2 34 C:0.529412 T:0.470588
chr28 11080756 2 36 T:0.916667 C:0.0833333
chr28 11080853 2 38 TG:1 T:0
chr28 11080976 2 36 T:0.944444 G:0.0555556
chr28 11081239 2 34 A:0.941176 G:0.0588235
chr28 11081330 2 38 CTG:0.894737 C:0.105263
chr28 11081483 2 36 A:0.916667 AC:0.0833333
chr28 11081494 2 36 T:0.444444 G:0.555556
chr28 11081540 2 34 C:0.970588 T:0.0294118
chr28 11081649 2 38 C:1 T:0
chr28 11081757 2 34 C:0.235294 G:0.764706
chr28 11081919 2 34 C:0.941176 T:0.0588235
chr28 11081959 2 40 T:0.325 C:0.675
chr28 11082040 2 40 T:0.925 C:0.075
chr28 11082136 2 38 A:0.973684 C:0.0263158
chr28 11082244 2 38 C:0 T:1
chr28 11082388 2 38 C:0 G:1
chr28 11082442 2 38 C:0 T:1
chr28 11082920 2 36 G:0.972222 A:0.0277778
chr28 11083144 2 38 T:0.921053 C:0.0789474
chr28 11083204 3 40 C:0.875 G:0.05 T:0.075
chr28 11083338 2 36 C:0.638889 CTG:0.361111
chr28 11083603 2 32 A:0.90625 T:0.09375
chr28 11083870 2 36 C:0.472222 T:0.527778
chr28 11083873 2 34 C:1 T:0
chr28 11083874 2 34 G:1 A:0
chr28 11083953 2 36 C:1 T:0
chr28 11084408 2 34 C:0.588235 T:0.411765
chr28 11084893 2 16 T:0.9375 TA:0.0625
chr28 11084944 2 38 GAGAGAGAGAGAGCACGCGCC:0.631579
G:0.368421
chr28 11085077 2 34 G:0.441176 A:0.558824

chr28	11085221	2	36	T:0.444444	C:0.555556	
chr28	11085223	2	36	C:0.916667	A:0.0833333	
chr28	11085484	2	36	G:1	A:0	
chr28	11085598	2	36	GT:1	G:0	
chr28	11085630	2	38	A:1	G:0	
chr28	11085654	2	38	A:0.315789	AGTGAAGTACTGTGG:0.684211	
chr28	11085939	2	34	T:1	C:0	
chr28	11086111	2	38	G:1	A:0	
chr28	11086130	2	36	C:0.611111	CG:0.388889	
chr28	11086334	2	36	C:0.472222	A:0.527778	
chr28	11086385	2	36	G:0.916667	A:0.0833333	
chr28	11086761	2	38	C:0.552632	T:0.447368	
chr28	11086773	2	34	T:0.441176	C:0.558824	
chr28	11086780	2	38	C:1	T:0	
chr28	11087050	2	36	T:0.583333	C:0.416667	
chr28	11087121	2	36	G:1	A:0	
chr28	11087143	2	40	T:1	C:0	
chr28	11087191	2	34	C:1	CT:0	
chr28	11087197	3	34	A:0.176471	G:0.823529	C:0
chr28	11087228	2	32	G:0.90625	T:0.09375	
chr28	11087249	2	30	T:0	C:1	
chr28	11087269	3	34	A:0.147059	G:0.852941	C:0
chr28	11087277	2	34	C:1	T:0	
chr28	11087278	2	34	G:0.911765	A:0.0882353	
chr28	11087407	2	30	C:1	T:0	
chr28	11087609	2	38	C:0.921053	T:0.0789474	
chr28	11087639	2	40	C:0.925	T:0.075	
chr28	11087712	2	38	C:1	A:0	
chr28	11088107	2	36	T:1	A:0	
chr28	11088113	2	36	A:0.888889	G:0.111111	
chr28	11088146	2	38	C:0.921053	T:0.0789474	
chr28	11088216	2	36	T:0.5	A:0.5	
chr28	11088303	2	40	T:1	A:0	
chr28	11088311	2	40	T:1	A:0	
chr28	11088406	2	36	G:0.888889	T:0.111111	
chr28	11088443	2	36	C:1	T:0	
chr28	11088646	2	36	G:1	C:0	
chr28	11088889	2	36	C:0.0833333	G:0.916667	
chr28	11088961	2	32	G:0.09375	T:0.90625	
chr28	11089447	2	32	T:0.875	TG:0.125	
chr28	11089675	2	30	T:1	C:0	
chr28	11089868	2	34	ATGAT:0.911765	A:0.0882353	
chr28	11090312	2	40	C:0.9	T:0.1	
chr28	11090385	2	34	C:0.470588	T:0.529412	
chr28	11090391	2	36	G:1	A:0	
chr28	11090401	2	36	T:1	C:0	
chr28	11090601	2	38	T:0.894737	C:0.105263	
chr28	11090646	2	36	G:0.888889	A:0.111111	
chr28	11090851	2	40	T:1	C:0	
chr28	11091691	2	36	T:1	C:0	
chr28	11091957	2	36	A:0.472222	T:0.527778	

chr28	11091964	2	36	C:1	T:0		
chr28	11091986	2	36	C:0	T:1		
chr28	11092072	2	40	G:1	A:0		
chr28	11092484	2	38	G:0.342105	A:0.657895		
chr28	11092673	2	34	C:1	G:0		
chr28	11092734	2	36	G:0.388889	A:0.611111		
chr28	11092759	2	36	A:0.388889	G:0.611111		
chr28	11092773	4	38	CT:0.657895	C:0.0263158	CTT:0.236842	CTTT:0.0789474
chr28	11092798	2	30	A:0.433333	G:0.566667		
chr28	11093193	2	26	T:1	C:0		
chr28	11093450	2	36	C:1	T:0		
chr28	11093451	2	36	G:1	A:0		
chr28	11093600	2	36	T:0.5	C:0.5		
chr28	11093730	2	38	C:1	T:0		
chr28	11093772	2	36	A:0.388889	C:0.611111		
chr28	11094006	2	38	T:0	C:1		
chr28	11094058	2	34	C:0.470588	T:0.529412		
chr28	11094197	2	38	A:0.368421	C:0.631579		
chr28	11094562	2	36	T:0.361111	C:0.638889		
chr28	11094827	2	32	T:0	C:1		
chr28	11094880	2	36	G:1	C:0		
chr28	11095001	2	16	CTA:0.375	C:0.625		
chr28	11095046	2	34	GAGGT:0.176471	G:0.823529		
chr28	11095051	2	34	CATCACTGTTTCCTCGA:0.176471	C:0.823529		
chr28	11095265	2	30	A:0	G:1		
chr28	11095422	2	30	C:0.6	T:0.4		
chr28	11095450	2	22	T:0.363636	C:0.636364		
chr28	11095473	2	16	G:0.375	GC:0.625		
chr28	11095587	2	34	GC:0.529412	G:0.470588		
chr28	11095611	2	34	T:1	C:0		
chr28	11095636	2	30	T:0	TC:1		
chr28	11095641	2	30	T:0	C:1		
chr28	11095713	2	30	G:0.5	A:0.5		
chr28	11095747	2	32	C:0.5625	T:0.4375		
chr28	11096086	2	38	T:0.368421	TC:0.631579		
chr28	11096095	2	38	T:0.368421	C:0.631579		
chr28	11096168	2	30	T:0.266667	C:0.733333		
chr28	11096230	2	40	C:0.55	G:0.45		
chr28	11096387	2	36	T:0.444444	C:0.555556		
chr28	11096613	2	38	C:0.5	A:0.5		
chr28	11096615	2	38	C:0.5	T:0.5		
chr28	11096744	2	32	C:0.9375	T:0.0625		
chr28	11096805	2	26	G:1	A:0		
chr28	11096887	2	36	C:0.305556	T:0.694444		
chr28	11096899	2	36	TTATAA:0.444444	T:0.555556		
chr28	11097168	2	38	C:0.631579	T:0.368421		
chr28	11097174	2	38	T:0.421053	C:0.578947		
chr28	11097222	2	36	G:0.972222	T:0.0277778		
chr28	11097772	2	36	T:0	C:1		
chr28	11097881	2	34	G:1	T:0		
chr28	11098026	2	32	A:1	C:0		
chr28	11098304	2	32	G:1	T:0		

chr28	11098527	2	38	A:1	G:0	
chr28	11098587	2	32	A:0	G:1	
chr28	11098782	2	34	C:0.941176		CCTT:0.0588235
chr28	11099365	2	36	G:0.555556		C:0.444444
chr28	11099488	2	30	GA:0.0333333		G:0.966667
chr28	11099513	2	34	G:1	A:0	
chr28	11099641	2	32	G:1	A:0	
chr28	11099763	2	34	T:1	C:0	
chr28	11099799	2	32	A:0.375	G:0.625	
chr28	11100187	2	36	T:1	C:0	
chr28	11100307	2	34	T:0.235294		C:0.764706
chr28	11100470	2	32	T:1	C:0	
chr28	11100601	2	34	G:1	A:0	
chr28	11100610	2	36	G:1	C:0	
chr28	11100623	2	38	G:1	A:0	
chr28	11100882	2	40	C:0.425	T:0.575	
chr28	11101093	2	38	T:0.368421		C:0.631579
chr28	11101147	2	36	C:1	T:0	
chr28	11101263	2	34	T:1	TA:0	
chr28	11101367	2	34	G:0.441176		C:0.558824
chr28	11101835	2	38	C:0.5	T:0.5	
chr28	11101862	2	36	T:0	TC:1	
chr28	11101892	2	32	A:0.9375	AC:0.0625	
chr28	11102125	2	36	C:0.472222		T:0.527778
chr28	11102168	2	34	C:0.735294		T:0.264706
chr28	11102205	2	34	C:0.882353		T:0.117647
chr28	11102340	2	32	G:0.4375	A:0.5625	
chr28	11102341	2	32	C:0.625	T:0.375	
chr28	11102815	2	38	C:0.789474		T:0.210526
chr28	11103125	2	36	G:0.416667		A:0.583333
chr28	11103373	2	38	C:1	G:0	
chr28	11103479	2	40	C:0	T:1	
chr28	11103607	2	38	G:0.447368		A:0.552632
chr28	11103837	2	38	A:0.736842		AT:0.263158
chr28	11104180	2	38	G:0	A:1	
chr28	11104411	2	34	G:0	A:1	
chr28	11104762	2	34	A:1	G:0	
chr28	11104855	2	40	TTGTC:0.625		T:0.375
chr28	11105005	2	36	C:0.361111		A:0.638889
chr28	11105189	2	38	T:0	C:1	
chr28	11105214	2	38	G:1	A:0	
chr28	11105290	2	38	C:0.947368		A:0.0526316
chr28	11105354	2	38	T:0.421053		G:0.578947
chr28	11105413	2	38	T:0.473684		A:0.526316
chr28	11105483	2	38	C:1	T:0	
chr28	11105707	2	38	C:1	T:0	
chr28	11105854	2	34	A:0.0294118		AT:0.970588
chr28	11105936	2	36	A:0	C:1	
chr28	11106029	2	28	C:0	T:1	
chr28	11106299	2	40	C:0.05	T:0.95	
chr28	11106656	2	20	G:0.1	C:0.9	
chr28	11106834	2	36	GA:1	G:0	
chr28	11106854	2	32	GGAA:1	G:0	
chr28	11107247	2	34	G:0.970588		T:0.0294118

chr28	11107330	2	38	C:0.131579	G:0.868421
chr28	11107343	2	36	A:1	AG:0
chr28	11107350	2	36	A:0.138889	C:0.861111
chr28	11108437	2	34	A:1	G:0
chr28	11109076	2	38	C:1	T:0
chr28	11109354	2	40	G:0.6	GCCCTCTGT:0.4
chr28	11109367	2	40	C:0.45	CTGTCCCTG:0.55
chr28	11109508	2	38	C:1	G:0
chr28	11109652	2	32	C:0	T:1
chr28	11109667	2	36	G:1	A:0
chr28	11109813	2	30	G:1	T:0
chr28	11110093	2	36	C:0.0277778	A:0.972222
chr28	11110396	2	40	C:0.925	T:0.075
chr28	11110523	2	40	T:0.875	G:0.125
chr28	11110596	2	40	A:1	C:0
chr28	11110844	2	38	G:1	A:0
chr28	11111059	2	32	G:1	A:0
chr28	11111267	2	36	T:1	C:0
chr28	11111378	2	40	T:1	TGCCAGTGGTCCCTGGGCCCCTCTTAGAGAA:0
chr28	11111411	2	40	C:0.925	T:0.075
chr28	11111442	2	40	C:0.925	T:0.075
chr28	11111522	2	34	G:1	C:0
chr28	11111683	2	38	G:1	A:0
chr28	11111688	2	38	A:1	ACT:0
chr28	11111767	2	40	C:1	CTGCCCATTT:0
chr28	11111835	2	36	A:1	G:0
chr28	11111999	2	36	G:1	A:0
chr28	11112156	2	36	G:0.888889	GGT:0.111111
chr28	11112381	2	30	G:1	C:0
chr28	11112479	2	38	T:1	A:0
chr28	11112502	2	38	T:1	C:0
chr28	11112579	2	36	A:1	G:0
chr28	11112915	2	34	T:1	C:0
chr28	11112950	2	32	A:0.96875	C:0.03125
chr28	11113095	2	40	C:1	A:0
chr28	11113144	2	38	C:1	G:0
chr28	11113246	2	38	A:0.368421	G:0.631579
chr28	11113345	2	36	CAGT:1	C:0
chr28	11113470	2	38	C:1	T:0
chr28	11113826	2	40	G:1	C:0
chr28	11113919	2	40	G:0.925	T:0.075
chr28	11113945	2	40	G:0.925	A:0.075
chr28	11114003	2	38	C:0.973684	A:0.0263158
chr28	11114052	2	36	CA:1	C:0
chr28	11114214	2	38	T:0.368421	TG:0.631579
chr28	11114250	2	38	G:0.947368	GGGCATCA:0.0526316
chr28	11114310	2	36	C:1	T:0
chr28	11114488	2	34	G:0	A:1
chr28	11114644	2	36	G:1	C:0
chr28	11114680	2	36	C:0.583333	A:0.416667
chr28	11114745	2	36	C:1	G:0
chr28	11114952	2	38	T:0.684211	G:0.315789

chr28	11115466 2	40	GGTCATCACTCTTTT:1	G:0
chr28	11116483 2	36	G:1	A:0
chr28	11116748 2	36	G:0.944444	A:0.0555556
chr28	11116868 2	40	C:1	T:0
chr28	11117470 2	36	C:1	T:0
chr28	11117661 2	34	C:1	T:0
chr28	11117865 2	36	C:0	G:1
chr28	11117881 2	36	T:1	C:0
chr28	11117882 2	36	G:0.944444	A:0.0555556
chr28	11117970 2	38	G:0.131579	A:0.868421
chr28	11118123 2	38	G:1	T:0
chr28	11118577 2	34	G:1	A:0
chr28	11119076 3	40	G:0.95	GTGTATATATATA:0
GTGTATATATATATATATATATATATATATA:0.05
chr28	11119439 2	36	TA:0.0833333	T:0.916667
chr28	11119460 2	36	C:1	T:0
chr28	11119719 2	38	AGCCGC:0.0526316 A:0.947368
chr28	11119758 2	38	C:1	T:0
chr28	11119763 2	38	G:0.0263158	A:0.973684
chr28	11119851 2	36	A:1	G:0
chr28	11119940 2	32	G:1	A:0
chr28	11119970 2	32	G:0.90625	A:0.09375
chr28	11120461 2	32	C:0.53125	T:0.46875
chr28	11120469 2	34	T:0	G:1
chr28	11120511 2	38	C:0.605263	T:0.394737
chr28	11120515 2	38	A:0	G:1
chr28	11120552 2	38	A:0	G:1
chr28	11120557 2	38	G:1	A:0
chr28	11120715 2	34	GCCTC:0.941176	G:0.0588235
chr28	11120719 2	34	C:0.0882353	G:0.911765
chr28	11121395 2	40	C:1	T:0
chr28	11122447 2	38	G:1	A:0
chr28	11122803 2	40	G:1	A:0
chr28	11122824 2	40	C:0.925	T:0.075
chr28	11122877 2	40	G:0.05	A:0.95
chr28	11122960 2	40	A:1	C:0
chr28	11123223 2	16	AGTGT:0.75	A:0.25
chr28	11123436 2	26	C:0.0769231	T:0.923077
chr28	11123464 3	16	G:0.25	GC:0.25	GCC:0.5
chr28	11123682 2	30	G:1	A:0
chr28	11123715 2	28	T:1	TAG:0
chr28	11123739 2	30	A:0.9	G:0.1
chr28	11123933 2	40	GTCAACCATCCAATGCA:0.925	G:0.075
chr28	11124034 2	40	C:1	T:0
chr28	11124145 2	32	T:0.875	C:0.125
chr28	11124619 2	36	C:1	T:0
chr28	11124663 2	38	AGGTGTCGCTATCTGCT:0.947368
A:0.0526316
chr28	11124729 2	36	T:0.888889	C:0.111111
chr28	11124852 2	40	G:1	C:0
chr28	11124920 2	36	G:0.972222	A:0.0277778
chr28	11125055 2	36	G:1	A:0
chr28	11125076 2	32	T:0	C:1
chr28	11125082 2	32	A:0.59375	G:0.40625

chr28	11125296	2	38	G:0.921053	C:0.0789474
chr28	11125301	2	38	A:1	AG:0
chr28	11126251	2	38	G:0.0263158	A:0.973684
chr28	11126751	2	36	A:0.861111	T:0.138889
chr28	11127161	2	34	T:0.0882353	TG:0.911765
chr28	11127552	2	36	A:0.916667	G:0.0833333
chr28	11127885	2	28	T:0.178571	TG:0.821429
chr28	11127933	2	34	T:0.0882353	C:0.911765
chr28	11128622	2	36	G:0.0277778	A:0.972222
chr28	11128738	2	38	C:1	T:0
chr28	11128850	2	38	T:1	C:0
chr28	11128854	2	34	C:1	T:0
chr28	11128957	2	36	C:1	T:0
chr28	11129294	2	40	C:1	G:0
chr28	11129364	2	34	G:0.0588235	A:0.941176
chr28	11129661	2	38	G:1	A:0
chr28	11129712	2	38	G:0.947368	GAGA:0.0526316
chr28	11129752	2	38	A:0.921053	G:0.0789474
chr28	11129795	2	36	C:0.916667	T:0.0833333
chr28	11129822	2	32	A:0.90625	C:0.09375
chr28	11129834	2	34	C:1	G:0
chr28	11129868	2	32	A:1	G:0
chr28	11130117	2	30	C:1	T:0
chr28	11130274	2	38	A:1	T:0
chr28	11130544	2	36	G:1	A:0
chr28	11130804	2	36	C:1	T:0
chr28	11130815	2	36	C:1	T:0
chr28	11130859	2	34	G:1	A:0
chr28	11131063	2	36	T:1	C:0
chr28	11131224	2	38	A:0.894737	T:0.105263
chr28	11131264	2	40	T:0.125	C:0.875
chr28	11131378	2	38	C:1	T:0
chr28	11131401	2	38	G:1	A:0
chr28	11131888	2	34	C:1	T:0
chr28	11131963	2	38	C:0.0789474	T:0.921053
chr28	11132199	2	38	GAAGC:0.973684	G:0.0263158
chr28	11132234	2	32	C:1	T:0
chr28	11132682	2	36	CTGGGA:1	C:0
chr28	11132688	2	36	T:0.527778	C:0.472222
chr28	11132734	2	38	G:1	T:0
chr28	11133031	2	36	A:1	G:0
chr28	11133123	2	40	A:1	C:0
chr28	11133233	2	38	T:1	C:0
chr28	11133371	2	32	A:1	G:0
chr28	11133787	2	34	T:0.558824	G:0.441176
chr28	11133958	2	36	A:1	T:0
chr28	11134127	2	36	C:0.583333	T:0.416667
chr28	11134128	2	36	A:0.5	G:0.5
chr28	11134207	2	40	G:0.6	GT:0.4
chr28	11134396	2	34	C:1	T:0
chr28	11134854	2	34	C:1	G:0
chr28	11135111	2	38	A:1	T:0
chr28	11135266	2	38	T:1	C:0
chr28	11135305	2	38	A:1	G:0

chr28	11135483	2	38	G:1	T:0		
chr28	11136856	2	34	T:0.382353	G:0.617647		
chr28	11137017	2	34	G:1	A:0		
chr28	11137148	2	38	G:1	T:0		
chr28	11137321	2	34	T:0.5	C:0.5		
chr28	11137812	2	38	C:0.973684	T:0.0263158		
chr28	11138410	2	32	C:0.6875	T:0.3125		
chr28	11138674	2	36	C:0.638889	T:0.361111		
chr28	11139107	2	36	A:0.611111	G:0.388889		
chr28	11139630	2	38	G:0.578947	A:0.421053		
chr28	11139927	2	40	G:0.575	A:0.425		
chr28	11140069	2	34	C:1	T:0		
chr28	11141630	2	30	CT:0.733333	C:0.266667		
chr28	11141693	3	36	C:0.583333	CAGAGAG:0.0833333	CAGAGAGAGAG:0.333333	
chr28	11141931	2	36	A:0.416667	T:0.583333		
chr28	11141962	2	36	C:0.416667	T:0.583333		
chr28	11142267	3	40	T:0.65	TAACAC:0.35	TAACATAACATAACAC:0	
chr28	11142363	2	36	G:0.555556	T:0.444444		
chr28	11142488	2	36	G:0.5	A:0.5		
chr28	11142555	2	38	CT:0.526316	C:0.473684		
chr28	11142679	2	40	A:0.875	G:0.125		
chr28	11142692	2	40	AC:0	A:1		
chr28	11143194	2	34	T:0	C:1		
chr28	11143261	2	34	T:0.529412	C:0.470588		
chr28	11143450	2	38	A:0.368421	G:0.631579		
chr28	11144030	2	40	C:0.575	G:0.425		
chr28	11144307	2	32	T:0.5	C:0.5		
chr28	11144685	2	38	A:0	G:1		
chr28	11144887	2	38	ACTCT:0.631579	A:0.368421		
chr28	11145043	2	38	T:0	C:1		
chr28	11145093	2	34	G:0.911765	T:0.0882353		
chr28	11145162	2	38	A:0.605263	G:0.394737		
chr28	11145354	2	36	A:0.444444	G:0.555556		
chr28	11145417	2	40	T:0.575	C:0.425		
chr28	11145436	2	38	T:0.368421	C:0.631579		
chr28	11145448	2	36	C:0.888889	A:0.111111		
chr28	11145545	2	34	G:0.441176	T:0.558824		
chr28	11145690	2	30	C:0.4	G:0.6		
chr28	11146295	2	38	C:0.473684	T:0.526316		
chr28	11146472	2	38	C:0.605263	A:0.394737		
chr28	11146594	2	36	C:0	A:1		
chr28	11146971	2	36	G:0.166667	GA:0.833333		
chr28	11146974	4	38	G:0.105263	GGGAGA:0.157895	GGGAGAGA:0.315789	GGGAGAGAGA:0.421053
chr28	11147054	2	36	C:0.361111	T:0.638889		
chr28	11147463	2	40	T:1	G:0		
chr28	11147800	2	36	T:0.444444	C:0.555556		
chr28	11147871	2	28	G:0.571429	T:0.428571		
chr28	11148705	2	38	A:0.552632	G:0.447368		
chr28	11148781	2	40	C:0.625	T:0.375		
chr28	11149467	2	34	C:0.382353	G:0.617647		
chr28	11149894	2	36	CT:0.583333	C:0.416667		

chr28	11149941	2	38	G:1	A:0	
chr28	11150015	2	38	T:0.552632	C:0.447368	
chr28	11150447	2	34	T:0.558824	C:0.441176	
chr28	11150546	2	34	G:0.441176	A:0.558824	
chr28	11150858	2	38	G:1	A:0	
chr28	11151445	2	36	G:0.388889	GGA:0.611111	
chr28	11151693	2	34	C:0.705882	G:0.294118	
chr28	11151793	2	38	C:0.605263	T:0.394737	
chr28	11151986	2	36	C:0.555556	A:0.444444	
chr28	11152066	2	40	C:0	T:1	
chr28	11152200	2	34	G:0.617647	A:0.382353	
chr28	11152206	3	36	CTT:0.75	C:0.111111	CTTTT:0.138889
chr28	11152336	2	40	G:0.975	A:0.025	
chr28	11152348	2	40	C:1	T:0	
chr28	11152491	2	38	T:0.473684	A:0.526316	
chr28	11152563	2	38	CAAAT:1	C:0	
chr28	11152752	2	38	T:0.684211	C:0.315789	
chr28	11153222	2	36	A:0.472222	G:0.527778	
chr28	11153233	2	36	G:1	T:0	
chr28	11153341	2	38	G:0.605263	A:0.394737	
chr28	11153357	2	38	A:0.631579	G:0.368421	
chr28	11153836	2	36	T:0.611111	G:0.388889	
chr28	11153872	2	34	T:1	C:0	
chr28	11154148	2	40	C:0.575	T:0.425	
chr28	11154154	2	40	A:0.575	T:0.425	
chr28	11154158	2	40	C:0.125	T:0.875	
chr28	11154931	2	38	G:0.868421	T:0.131579	
chr28	11155137	2	36	TA:0.138889	T:0.861111	
chr28	11155363	2	38	T:0	TC:1	
chr28	11155369	2	38	CA:0	C:1	
chr28	11155378	2	38	G:0	GT:1	
chr28	11155382	2	38	CT:0	C:1	
chr28	11156260	2	32	C:0.84375	A:0.15625	
chr28	11156387	2	36	TA:0.722222	T:0.277778	
chr28	11157509	2	36	G:0.916667	A:0.0833333	
chr28	11157784	2	40	A:0.55	T:0.45	
chr28	11157947	2	36	G:0.0833333	A:0.916667	
chr28	11158269	2	34	C:1	G:0	
chr28	11158274	2	34	C:1	A:0	
chr28	11158471	2	40	TC:1	T:0	
chr28	11159109	2	38	T:0.105263	C:0.894737	
chr28	11159293	2	26	G:1	A:0	
chr28	11159412	2	32	G:0.0625	T:0.9375	
chr28	11159430	2	28	T:0.857143	C:0.142857	
chr28	11159637	2	38	C:0.894737	T:0.105263	
chr28	11159720	2	38	T:0	TA:1	
chr28	11159746	2	36	T:0	A:1	
chr28	11159748	2	36	T:0	G:1	
chr28	11159924	2	36	T:1	C:0	
chr28	11160453	3	36	GTTT:0.0555556	G:0.916667	GTTTT:0.0277778
chr28	11160461	2	36	T:0.0833333	A:0.916667	
chr28	11160509	2	34	G:1	T:0	

chr28	11160610	2	34	C:0.0882353	T:0.911765	
chr28	11160754	2	30	A:0.2	T:0.8	
chr28	11161130	2	36	C:0.888889	T:0.111111	
chr28	11161179	2	40	C:1	T:0	
chr28	11161218	2	40	G:0.125	A:0.875	
chr28	11161643	2	32	T:1	C:0	
chr28	11161658	2	30	T:0.866667	TTTC:0.133333	
chr28	11161676	3	34	C:0.411765	CTTCT:0.147059	CTTCTT:0.441176
chr28	11161715	2	16	G:1	GGA:0	
chr28	11161760	2	16	G:1	T:0	
chr28	11162339	2	36	T:0.916667	C:0.0833333	
chr28	11162664	2	36	G:0.555556	A:0.444444	
chr28	11162702	2	34	A:0.882353	G:0.117647	
chr28	11162782	2	34	A:0.382353	C:0.617647	
chr28	11162799	2	34	T:0.411765	C:0.588235	
chr28	11163218	2	36	G:1	A:0	
chr28	11163562	2	36	T:0.833333	C:0.166667	
chr28	11163603	2	38	G:0.157895	C:0.842105	
chr28	11163783	2	34	TG:1	T:0	
chr28	11164244	2	40	T:0.8	C:0.2	
chr28	11164304	2	40	T:0.125	C:0.875	
chr28	11164358	2	40	C:1	T:0	
chr28	11164857	2	38	A:0.105263	G:0.894737	
chr28	11164976	2	34	T:0.147059	C:0.852941	
chr28	11165039	2	40	T:0.125	C:0.875	
chr28	11165063	2	40	C:1	T:0	
chr28	11165132	2	36	C:1	T:0	
chr28	11165204	2	38	T:0.131579	G:0.868421	
chr28	11165964	2	36	T:1	C:0	
chr28	11166141	2	40	C:1	T:0	
chr28	11166434	2	40	C:0.9	T:0.1	
chr28	11166477	2	40	G:0.925	C:0.075	
chr28	11166790	2	40	C:0.6	T:0.4	
chr28	11166849	2	38	A:0.894737	T:0.105263	
chr28	11167414	2	28	G:0.821429	A:0.178571	
chr28	11168171	2	38	A:0.868421	G:0.131579	
chr28	11168230	2	36	AT:0.444444	A:0.555556	
chr28	11168895	2	38	G:0.921053	A:0.0789474	
chr28	11169451	2	36	T:0.0833333	C:0.916667	
chr28	11169480	2	40	G:0.5	A:0.5	
chr28	11169653	2	36	C:0.166667	T:0.833333	
chr28	11169739	2	36	C:1	T:0	
chr28	11170681	2	36	G:0.583333	A:0.416667	
chr28	11171008	2	40	A:1	G:0	
chr28	11171327	2	36	T:1	C:0	
chr28	11171402	2	38	G:0.131579	T:0.868421	
chr28	11172563	2	38	G:1	A:0	
chr28	11173106	2	38	T:0.421053	C:0.578947	
chr28	11173138	2	38	CCTCCT:0.894737	C:0.105263	
chr28	11173139	2	38	C:0.605263	T:0.394737	
chr28	11173145	2	38	T:0.605263	C:0.394737	
chr28	11173146	2	38	CTTTCTTTCTTTCTTTCTTTCTTTCTTTCTTT:0.894737	C:0.105263	

chr28	11173149 2	38	T:0.605263	C:0.394737
chr28	11173160 2	34
TTCTTTCTTTCTTTCTTTCTTTCTTTCTTTCTTTCTTTCTTTCTTTCTTTCTTTCTTTC:1
T:0
chr28	11173164 2	34
TTCTTTCTTTCTTTCTTTCTTTCTTTCTTTCTTTCTTTCTTTCTTTCTTTCTTTCTTTC:0.647059
T:0.352941
chr28	11173181 2	38	T:0.894737	C:0.105263
chr28	11173185 2	38	T:0.894737	C:0.105263
chr28	11173206 2	38	CT:0.868421	C:0.131579
chr28	11173215 2	38	TTTC:0.868421	T:0.131579
chr28	11173562 2	38	C:0.578947	T:0.421053
chr28	11173968 2	40	CA:0.525 C:0.475
chr28	11174166 2	34	C:0.823529	T:0.176471
chr28	11174171 2	34	T:0.823529	G:0.176471
chr28	11174481 2	38	AT:0.578947	A:0.421053
chr28	11174534 2	36	C:1	T:0
chr28	11174882 2	38	A:0.842105	G:0.157895
chr28	11174963 2	36	G:0.138889	A:0.861111
chr28	11175147 2	38	G:0.5	A:0.5
chr28	11175525 2	40	AACTATGC:0.525	A:0.475
chr28	11176031 2	38	C:0.526316	T:0.473684
chr28	11177011 2	36	C:0.833333	T:0.166667
chr28	11177390 2	34	A:0.147059	G:0.852941
chr28	11178063 2	38	G:0.131579	A:0.868421
chr28	11178400 2	34	C:0.882353	T:0.117647
chr28	11178599 2	38	A:0.868421	G:0.131579
chr28	11178762 2	36	G:0.861111	A:0.138889
chr28	11179142 2	38	A:1	C:0
chr28	11179183 2	34	T:0	C:1
chr28	11179361 2	40	T:0.875	C:0.125
chr28	11180159 2	36	G:1	GT:0
chr28	11180194 3	36	CAA:0	C:0.194444	CA:
0.805556
chr28	11180350 2	34	GGGA:0.882353	G:0.117647
chr28	11181319 2	38	AC:0.894737	A:0.105263
chr28	11181590 2	38	C:0.868421	T:0.131579
chr28	11182207 2	38	T:0.868421	C:0.131579
chr28	11182463 2	34	G:0.852941	A:0.147059
chr28	11183194 2	34	T:0.558824	C:0.441176
chr28	11183281 2	34	C:1	T:0
chr28	11183904 2	36	A:0.277778	G:0.722222
chr28	11183920 2	40	A:1	ATGCTTTGCTT:0
chr28	11183967 2	40	T:1	TCTTTTCTTTTCTTTTCTTTC:0
chr28	11184001 2	40	T:1	TCTTTTCTTTC:0
chr28	11184021 3	40	T:0.6
TTTTCTTTCTTTCTTTCTTTCTTTCTTTC:0.3 TTTTCTTTCTTTCTTTCTTTCTTC:0.1
chr28	11184055 2	40	T:0.975	TTCTTTCTTTCTTTC:0.025
chr28	11184072 2	40	C:0.975	T:0.025
chr28	11184191 2	38	C:0.921053	A:0.0789474
chr28	11184323 2	40	C:0.375	A:0.625
chr28	11184763 2	36	G:0.666667	C:0.333333
chr28	11184784 2	36	G:0.833333	A:0.166667
chr28	11184800 2	36	T:0.833333	C:0.166667

chr28	11184949	2	38	A:0.894737	AT:0.105263	
chr28	11185127	2	36	T:1	C:0	
chr28	11185181	2	36	A:0.916667	T:0.0833333	
chr28	11185384	3	34	A:0.529412	AGT:0.352941	AGTGT:0.117647
chr28	11185526	2	38	A:0.842105	G:0.157895	
chr28	11186189	2	38	AG:1	A:0	
chr28	11186315	2	36	T:0.527778	G:0.472222	
chr28	11186991	2	34	A:0.382353	AT:0.617647	
chr28	11187145	2	34	T:0.852941	A:0.147059	
chr28	11187384	3	30	TAC:0.666667	T:0.166667	TACAC:0.166667
chr28	11187441	2	30	T:0.9	C:0.1	
chr28	11187765	2	32	A:0.75	G:0.25	
chr28	11187923	2	36	A:1	G:0	
chr28	11188230	2	40	A:0.375	T:0.625	
chr28	11188254	2	40	T:0.6	TTG:0.4	
chr28	11188271	2	38	A:0.105263	T:0.894737	
chr28	11188390	2	34	T:1	C:0	
chr28	11188604	2	36	A:1	AT:0	
chr28	11188775	2	26	C:1	T:0	
chr28	11189214	2	36	C:1	T:0	
chr28	11189290	2	38	A:0.552632	ATGC:0.447368	
chr28	11189444	2	40	AC:1	A:0	
chr28	11189943	2	36	A:0.444444	G:0.555556	
chr28	11189985	2	34	G:1	C:0	
chr28	11190134	2	36	G:0.888889	C:0.111111	
chr28	11190159	2	36	T:1	A:0	
chr28	11190487	2	36	A:0.388889	G:0.611111	
chr28	11190612	2	38	C:0.526316	T:0.473684	
chr28	11191046	2	36	C:0.861111	A:0.138889	
chr28	11191548	2	38	G:1	A:0	
chr28	11191866	2	34	A:1	G:0	
chr28	11191907	2	38	A:1	G:0	
chr28	11192501	2	38	T:0.394737	C:0.605263	
chr28	11193109	2	36	G:0.638889	A:0.361111	
chr28	11193126	2	34	C:1	T:0	
chr28	11193387	2	38	A:0	C:1	
chr28	11193427	2	38	C:0.657895	T:0.342105	
chr28	11193547	2	34	A:1	G:0	
chr28	11193767	2	40	C:0.65	G:0.35	
chr28	11194490	2	34	C:0.911765	T:0.0882353	
chr28	11194601	2	38	G:0.894737	A:0.105263	
chr28	11194606	2	38	C:0.368421	CA:0.631579	
chr28	11194629	2	36	C:1	T:0	
chr28	11194866	2	40	G:0.675	A:0.325	
chr28	11195436	2	28	CG:0.821429	C:0.178571	
chr28	11195609	2	30	A:0.666667	T:0.333333	
chr28	11195630	2	32	T:0.65625	A:0.34375	
chr28	11195750	2	38	TTC:0.394737	T:0.605263	
chr28	11195893	2	36	C:0.861111	T:0.138889	
chr28	11196142	2	32	C:0.96875	T:0.03125	
chr28	11196217	2	40	T:0.4	C:0.6	
chr28	11196473	2	38	T:0.526316	C:0.473684	

chr28	11196601	2	38	CTG:0.631579	C:0.368421	
chr28	11197557	2	38	C:1	G:0	
chr28	11197560	2	38	T:0.842105	C:0.157895	
chr28	11197658	2	34	A:1	G:0	
chr28	11198089	2	40	G:1	A:0	
chr28	11198252	2	40	T:0.65	A:0.35	
chr28	11198341	2	40	TTA:0.8	T:0.2	
chr28	11198570	2	38	T:0.921053	C:0.0789474	
chr28	11198628	2	38	A:0.368421	G:0.631579	
chr28	11198887	2	38	T:0.868421	C:0.131579	
chr28	11199236	2	40	AAAG:0.925	A:0.075	
chr28	11199522	2	38	T:0.421053	TAC:0.578947	
chr28	11199864	2	38	A:0.868421	T:0.131579	
chr28	11199915	2	40	T:0.325	C:0.675	
chr28	11200037	2	36	C:0.805556	T:0.194444	
chr28	11200262	2	40	T:0.35	A:0.65	
chr28	11200286	2	38	C:1	T:0	
chr28	11200366	2	32	C:1	T:0	
chr28	11200434	2	40	ATAG:0.925	A:0.075	
chr28	11200515	2	34	A:0.558824	C:0.441176	
chr28	11200585	2	34	A:0.794118	AAG:0.205882	
chr28	11200769	2	34	AT:0.823529	A:0.176471	
chr28	11200827	2	34	C:0.705882	T:0.294118	
chr28	11201104	2	40	G:0.825	A:0.175	
chr28	11201365	2	38	T:1	C:0	
chr28	11201602	2	34	T:1	C:0	
chr28	11201640	2	38	C:0.973684	T:0.0263158	
chr28	11201650	2	38	A:0.947368	G:0.0526316	
chr28	11201836	2	34	G:0.941176	A:0.0588235	
chr28	11202021	2	34	CAAAAA:0.764706	C:0.235294	
chr28	11202039	2	34	CAA:0.764706	C:0.235294	
chr28	11202048	2	38	C:0.763158	A:0.236842	
chr28	11202051	2	34	AAAAAAC:0.764706	A:0.235294	
chr28	11202054	3	38	A:0.763158	AAC:0	AACAAC:0.236842
chr28	11202060	2	38	A:1	C:0	
chr28	11202122	2	34	C:0.970588	T:0.0294118	
chr28	11202791	2	38	T:0.394737	C:0.605263	
chr28	11202793	2	38	G:1	T:0	
chr28	11203019	2	36	G:0.916667	T:0.0833333	
chr28	11203521	2	32	A:0.46875	G:0.53125	
chr28	11203603	2	40	T:0.875	C:0.125	
chr28	11204127	2	36	A:0.638889	G:0.361111	
chr28	11204563	2	36	A:0.916667	T:0.0833333	
chr28	11204776	2	38	G:0.868421	A:0.131579	
chr28	11204816	2	38	T:0.342105	A:0.657895	
chr28	11205012	2	38	ATGTC:0.894737	A:0.105263	
chr28	11205127	2	38	A:0.815789	G:0.184211	
chr28	11205576	2	38	C:0.921053	T:0.0789474	
chr28	11205841	2	38	A:0.684211	C:0.315789	
chr28	11206426	2	36	A:0.138889	G:0.861111	
chr28	11206441	2	36	T:1	G:0	
chr28	11207252	2	38	T:0.578947	G:0.421053	
chr28	11207309	2	36	A:0.916667	T:0.0833333	

chr28 11207665 2 38 A:0 C:1
chr28 11207955 2 38 C:1 T:0
chr28 11208138 2 38 A:0.921053 T:0.0789474
chr28 11208478 3 36 TA:0.111111 T:0.527778
TAA:0.361111
chr28 11209025 2 36 C:1 T:0
chr28 11209347 2 34 G:0 A:1
chr28 11209461 2 40 G:0 A:1
chr28 11209546 2 40 C:1 T:0
chr28 11209700 2 38 G:1 A:0
chr28 11209761 2 38 C:0.921053 A:0.0789474
chr28 11210712 2 36 G:1 T:0
chr28 11211044 2 38 C:0.894737 T:0.105263
chr28 11211268 2 40 G:0 GAACAA:1
chr28 11211479 2 40 G:0.625 A:0.375
chr28 11211761 2 40 G:0.925 A:0.075
chr28 11211804 2 40 CT:0.6 C:0.4
chr28 11211807 3 40 TAA:0.425 T:0.5 TA:0.075
chr28 11211844 2 36 A:0.666667 AGAGAGAGC:
0.333333
chr28 11212061 2 38 G:0.894737 A:0.105263
chr28 11212096 2 38 C:0.973684 A:0.0263158
chr28 11212224 3 28 A:0.785714 AT:0.214286
ATT:0
chr28 11212567 2 38 G:1 A:0
chr28 11212891 2 34 G:1 A:0
chr28 11213287 2 34 C:1 T:0
chr28 11213555 2 40 A:1 G:0
chr28 11213752 2 40 T:0.475 C:0.525
chr28 11214289 2 38 C:0.552632 T:0.447368
chr28 11214381 2 38 AAGG:0.894737 A:0.105263
chr28 11215670 2 36 A:0.444444 G:0.555556
chr28 11215677 2 36 G:0.666667 A:0.333333
chr28 11215769 2 38 G:0.578947 A:0.421053
chr28 11215833 4 36 CTTTT:0.111111 C:0.388889
CT:0.222222 CTT:0.277778
chr28 11216573 2 38 C:1 T:0
chr28 11216594 2 36 T:0 C:1
chr28 11216783 2 40 C:0.55 T:0.45
chr28 11217136 2 30 C:0.6 CT:0.4
chr28 11217137 2 30 T:0.933333 TTC:0.0666667
chr28 11217798 2 32 A:0.90625 T:0.09375
chr28 11217871 2 36 C:0.416667 T:0.583333
chr28 11217999 2 38 A:0.473684 C:0.526316
chr28 11218337 2 38 A:0.605263 G:0.394737
chr28 11218584 2 38 G:0.421053 A:0.578947
chr28 11218635 2 34 G:0.382353 A:0.617647
chr28 11218794 2 36 C:0.416667 A:0.583333
chr28 11218823 2 34 G:0.852941 A:0.147059
chr28 11218863 2 34 C:0 A:1
chr28 11219026 2 40 T:0.475 G:0.525
chr28 11219082 2 34 G:0.5 A:0.5
chr28 11219280 2 38 C:0.578947 G:0.421053
chr28 11219290 2 36 A:0.555556 G:0.444444

chr28 11219442 2 34 CA:0.529412 C:0.470588
chr28 11219505 2 36 C:0.611111 T:0.388889
chr28 11219509 2 36 A:0.611111 G:0.388889
chr28 11219847 3 28 GT:0.714286 G:0.107143
GTT:0.178571
chr28 11220133 2 34 C:0.882353 T:0.117647
chr28 11220284 2 38 C:1 T:0
chr28 11220447 2 34 C:0.647059 T:0.352941
chr28 11220625 2 40 C:0.5 T:0.5
chr28 11220870 2 36 A:0.916667 C:0.0833333
chr28 11220884 2 36 A:0.611111 G:0.388889
chr28 11220953 2 38 G:0.552632 A:0.447368
chr28 11221023 2 40 T:0.575 C:0.425
chr28 11221156 2 38 C:1 G:0
chr28 11221493 3 38 C:0.157895 CT:0.815789
CTT:0.0263158
chr28 11221594 3 40 GTCTCTC:0.625 G:0.225 GTC:0.15
chr28 11221603 2 32 TCTCTCTCACA:1 T:0
chr28 11221609 2 36 T:0.833333 A:0.166667
chr28 11222176 2 34 G:0.529412 T:0.470588
chr28 11222232 2 38 CAG:0.973684 C:0.0263158
chr28 11222269 2 36 C:0.833333 T:0.166667
chr28 11222455 2 32 T:0.40625 C:0.59375
chr28 11222692 2 40 G:0.45 A:0.55
chr28 11222935 2 40 G:1 A:0
chr28 11222971 2 38 T:0.473684 C:0.526316
chr28 11223019 2 40 A:0.6 ACCCCCATTCACCCT:0.4
chr28 11223059 2 40 A:0.45 C:0.55
chr28 11223154 2 34 G:0.117647 A:0.882353
chr28 11223202 2 40 T:0.15 G:0.85
chr28 11223225 2 40 T:1 G:0
chr28 11223363 2 34 C:0.529412 T:0.470588
chr28 11223455 2 36 T:0.555556 C:0.444444
chr28 11223465 2 36 G:0.555556 A:0.444444
chr28 11223475 2 34 G:1 A:0
chr28 11224210 2 38 T:1 C:0
chr28 11224549 2 38 C:1 T:0
chr28 11225235 2 36 A:0.5 G:0.5
chr28 11225598 2 38 C:1 T:0
chr28 11225914 2 34 C:0.882353 CCCA:0.117647
chr28 11226115 2 38 A:0.631579 T:0.368421
chr28 11226301 2 32 C:0.46875 T:0.53125
chr28 11228114 2 30 G:0.566667 GC:0.433333
chr28 11228121 2 32 CG:1 C:0
chr28 11228125 2 32 C:0.59375 G:0.40625
chr28 11228255 2 32 T:0.5625 A:0.4375
chr28 11228427 2 34 G:1 C:0
chr28 11228779 4 40 AAC:0.625 A:0 AACAC:
0.025 AACACACAC:0.35
chr28 11229233 2 34 C:1 T:0
chr28 11229617 2 36 A:0 T:1
chr28 11229875 2 34 C:0.676471 A:0.323529
chr28 11229901 2 28 T:0.571429 C:0.428571
chr28 11229938 2 26 G:0.692308 GC:0.307692

chr28	11230222	2	36	C:0.555556	T:0.444444	
chr28	11230368	2	38	T:1	C:0	
chr28	11230391	2	38	A:1	C:0	
chr28	11230393	2	38	TC:1	T:0	
chr28	11230722	2	34	C:0.5	T:0.5	
chr28	11230730	2	34	C:1	T:0	
chr28	11230739	2	34	C:0	T:1	
chr28	11230799	2	38	GTGTCTCTGTCTC:0.526316	G:0.473684	
chr28	11230977	2	38	C:0.947368	T:0.0526316	
chr28	11231111	2	32	A:0	G:1	
chr28	11231332	2	32	T:0.6875	G:0.3125	
chr28	11231353	2	34	AC:1	A:0	
chr28	11231357	3	34	A:1	C:0	AAC:0
chr28	11231362	3	34	C:0.588235	A:0.411765	CA:0
chr28	11231364	2	34	AAC:1	A:0	
chr28	11231505	2	34	C:0.558824	T:0.441176	
chr28	11231888	2	36	T:0.555556	C:0.444444	
chr28	11231912	2	34	G:0.558824	A:0.441176	
chr28	11232195	2	40	G:0.9	A:0.1	
chr28	11232372	2	40	C:0.5	G:0.5	
chr28	11232389	2	40	C:0.5	T:0.5	
chr28	11232628	2	38	C:0.552632	T:0.447368	
chr28	11232632	2	38	T:0.552632	C:0.447368	
chr28	11232796	2	36	A:0.555556	G:0.444444	
chr28	11232823	2	36	A:0.583333	G:0.416667	
chr28	11232935	2	34	C:0.617647	T:0.382353	
chr28	11233037	2	34	A:0.617647	G:0.382353	
chr28	11233049	2	34	A:0.617647	G:0.382353	
chr28	11233051	2	34	T:0.617647	C:0.382353	
chr28	11233069	2	34	T:0.617647	C:0.382353	
chr28	11233204	2	38	G:1	T:0	
chr28	11233366	2	34	A:0.529412	T:0.470588	
chr28	11233477	2	36	C:0.416667	T:0.583333	
chr28	11234054	3	34	T:0.588235	TA:0.382353	TAA:0.0294118
chr28	11234137	2	36	A:0	C:1	
chr28	11234325	2	38	C:0.526316	G:0.473684	
chr28	11234378	2	36	G:0.527778	T:0.472222	
chr28	11234982	2	34	G:0.529412	A:0.470588	
chr28	11234992	2	34	G:0.529412	A:0.470588	
chr28	11235094	2	32	T:0.40625	C:0.59375	
chr28	11235251	2	34	T:0.588235	C:0.411765	
chr28	11235275	2	36	G:0.944444	A:0.0555556	
chr28	11235614	2	32	A:0.46875	G:0.53125	
chr28	11235736	2	38	C:0.552632	T:0.447368	
chr28	11235988	2	36	T:0.416667	C:0.583333	
chr28	11236283	2	34	G:0.470588	T:0.529412	
chr28	11236285	2	34	G:0.470588	T:0.529412	
chr28	11236615	2	38	G:0.526316	A:0.473684	
chr28	11236617	2	38	A:0.526316	C:0.473684	
chr28	11237023	2	38	C:0.526316	T:0.473684	
chr28	11237126	2	36	A:0.5	AT:0.5	

chr28	11237137	2	36	T:0.5	G:0.5			
chr28	11237162	2	36	C:0.5	G:0.5			
chr28	11237215	2	36	A:0.611111	C:0.388889			
chr28	11237220	2	36	C:0.611111	G:0.388889			
chr28	11237236	2	32	A:0	G:1			
chr28	11237285	2	38	T:0.5	TAA:0.5			
chr28	11237310	2	38	A:0.5	T:0.5			
chr28	11237500	2	38	T:0.921053	C:0.0789474			
chr28	11237567	2	38	A:0.5	G:0.5			
chr28	11237652	2	34	T:0.5	C:0.5			
chr28	11237739	2	40	G:1	A:0			
chr28	11237785	2	40	A:0.95	T:0.05			
chr28	11237827	2	40	A:0.5	G:0.5			
chr28	11237836	2	40	C:0.975	T:0.025			
chr28	11237954	2	38	G:0.473684	C:0.526316			
chr28	11238024	2	38	T:0.473684	G:0.526316			
chr28	11238028	2	38	G:0.473684	A:0.526316			
chr28	11238594	2	38	T:0.526316	C:0.473684			
chr28	11238761	2	32	G:0.59375	C:0.40625			
chr28	11238777	2	34	C:1	T:0			
chr28	11238894	2	38	G:0.578947	A:0.421053			
chr28	11238981	2	40	G:0.5	A:0.5			
chr28	11239148	2	36	G:0.5	A:0.5			
chr28	11239320	2	40	G:0.575	A:0.425			
chr28	11239352	3	32	GT:0.375	G:0.125	GTT:0.5		
chr28	11239856	2	38	TA:1	T:0			
chr28	11240512	2	38	C:0.5	T:0.5			
chr28	11241265	2	38	G:1	T:0			
chr28	11241476	2	28	G:0.5	A:0.5			
chr28	11241518	2	26	C:1	T:0			
chr28	11241600	2	24	G:0.708333	C:0.291667			
chr28	11241628	2	36	T:1	TAAAAAA:0			
chr28	11241629	3	34	T:0.676471	A:0.294118	TAAA:0.0294118		
chr28	11241715	2	38	G:0	A:1			
chr28	11241740	2	34	A:0.5	T:0.5			
chr28	11241788	2	32	T:0.53125	A:0.46875			
chr28	11241891	2	36	C:0.555556	T:0.444444			
chr28	11242060	2	34	G:0.0294118	T:0.970588			
chr28	11242096	2	38	G:1	A:0			
chr28	11242382	2	38	AG:1	A:0			
chr28	11243014	2	34	T:1	C:0			
chr28	11243101	5	38	CT:0.368421	C:0.0789474	CTT:0.131579	CTTT:0.131579	CTTTT:0.289474
chr28	11244344	2	40	C:1	T:0			
chr28	11244417	2	34	A:1	G:0			
chr28	11244560	2	36	ATCTCTCTCTC:0.666667	A:0.333333			
chr28	11244902	2	36	G:0.666667	A:0.333333			
chr28	11245275	2	36	C:0.583333	T:0.416667			
chr28	11245411	2	34	G:0.441176	C:0.558824			
chr28	11245427	2	36	C:1	T:0			
chr28	11245898	2	36	C:0.5	T:0.5			
chr28	11245938	3	36	G:1	A:0	T:0		

chr28	11245946	2	36	C:0.666667	A:0.333333		
chr28	11246330	2	32	G:1	A:0		
chr28	11246627	2	38	A:0.973684	G:0.0263158		
chr28	11246725	2	36	G:0.527778	C:0.472222		
chr28	11246979	2	34	T:1	G:0		
chr28	11247420	2	40	G:1	A:0		
chr28	11247430	2	40	A:0.9	G:0.1		
chr28	11247484	2	38	G:0.868421	A:0.131579		
chr28	11247492	2	38	T:0.947368	C:0.0526316		
chr28	11247544	2	38	T:0.921053	C:0.0789474		
chr28	11248338	2	36	C:1	T:0		
chr28	11248459	2	38	GTCAAA:0.447368	G:0.552632		
chr28	11248466	2	38	G:0.447368	GGCC:0.552632		
chr28	11248468	2	38	C:0.447368	CAG:0.552632		
chr28	11248509	2	38	G:0.921053	A:0.0789474		
chr28	11248833	2	34	C:1	T:0		
chr28	11248948	2	40	G:0.925	C:0.075		
chr28	11249268	2	34	C:1	T:0		
chr28	11249413	2	34	C:1	G:0		
chr28	11249510	2	32	C:1	A:0		
chr28	11249816	2	34	T:0.882353	C:0.117647		
chr28	11249942	2	38	T:1	C:0		
chr28	11249996	2	36	T:0.916667	TTC:0.0833333		
chr28	11250040	2	38	G:0.815789	GTCTC:0.184211		
chr28	11250044	2	36	C:1	CTG:0		
chr28	11250047	2	34	TCTCACACACACA:0.911765	T:0.0882353		
chr28	11250049	2	34	TCACA:1	T:0		
chr28	11250055	2	36	A:1	T:0		
chr28	11250057	2	36	A:1	T:0		
chr28	11250059	2	36	A:1	T:0		
chr28	11250061	2	30	A:1	T:0		
chr28	11250063	2	28	A:1	T:0		
chr28	11250065	2	28	A:1	T:0		
chr28	11250313	2	32	C:0.90625	T:0.09375		
chr28	11250331	2	32	T:0.875	C:0.125		
chr28	11250705	2	36	A:1	G:0		
chr28	11251322	2	34	T:0.911765	C:0.0882353		
chr28	11251447	2	36	T:1	C:0		
chr28	11251462	2	38	G:1	A:0		
chr28	11251590	2	36	GT:1	G:0		
chr28	11251993	2	36	C:1	T:0		
chr28	11252102	2	40	T:1	C:0		
chr28	11252187	2	38	C:0.789474	G:0.210526		
chr28	11252656	2	36	C:1	A:0		
chr28	11252805	4	40	CTTCT:0.675	C:0.225	CTTTCT:0.05	CTTCTTTCT:0.05
chr28	11252860	2	40	C:1	CT:0		
chr28	11253422	2	36	G:1	T:0		
chr28	11253544	2	40	G:0.8	C:0.2		
chr28	11253727	2	34	C:0.794118	G:0.205882		
chr28	11253832	2	40	C:0.85	T:0.15		
chr28	11253984	2	38	G:0.473684	A:0.526316		
chr28	11254421	2	34	C:1	T:0		

chr28	11254589	2	34	G:1	A:0		
chr28	11254881	2	40	A:0.65	G:0.35		
chr28	11255004	2	40	G:1	C:0		
chr28	11255252	2	40	GC:1	G:0		
chr28	11255254	2	40	C:1	A:0		
chr28	11255404	2	34	T:0.882353	C:0.117647		
chr28	11255687	2	38	CT:1	C:0		
chr28	11255734	2	38	T:0.815789	C:0.184211		
chr28	11256193	2	40	T:1	A:0		
chr28	11256194	2	40	A:0.85	G:0.15		
chr28	11256428	2	36	G:0.527778	A:0.472222		
chr28	11256480	2	40	T:0.325	A:0.675		
chr28	11256678	2	38	G:0.815789	A:0.184211		
chr28	11256791	2	30	AG:0.833333	A:0.166667		
chr28	11257115	2	38	G:1	A:0		
chr28	11257131	2	38	A:0.789474	G:0.210526		
chr28	11257256	2	38	A:0.763158	G:0.236842		
chr28	11257622	2	34	T:0.823529	A:0.176471		
chr28	11257676	2	38	T:0.842105	C:0.157895		
chr28	11257751	2	38	G:0.842105	A:0.157895		
chr28	11257777	2	38	T:0.815789	C:0.184211		
chr28	11257868	2	34	T:0.205882	C:0.794118		
chr28	11257917	2	38	A:0.815789	T:0.184211		
chr28	11257919	2	38	A:0.815789	C:0.184211		
chr28	11257986	2	36	C:0.666667	T:0.333333		
chr28	11258344	2	38	G:0.631579	GT:0.368421		
chr28	11258384	2	38	GATGGGTAA:0.947368	G:0.0526316		
chr28	11258487	2	28	C:1	T:0		
chr28	11258540	2	38	A:0	G:1		
chr28	11258557	2	38	A:0.842105	G:0.157895		
chr28	11258598	2	38	A:0	G:1		
chr28	11258631	2	34	A:1	C:0		
chr28	11259057	2	30	C:0.866667	A:0.133333		
chr28	11259219	2	36	G:1	A:0		
chr28	11259309	2	34	C:1	T:0		
chr28	11259354	2	36	T:0.333333	C:0.666667		
chr28	11259463	2	34	G:0.823529	A:0.176471		
chr28	11259464	2	34	T:0.823529	C:0.176471		
chr28	11259560	2	40	T:0.825	TC:0.175		
chr28	11259577	3	40	C:0.425	CT:0.575	CTTTTCTTTTCTTTTCT:0	
chr28	11259591	2	40	C:0.825	CT:0.175		
chr28	11259596	4	40	C:0.825	T:0.175	CTTTTCTTTTCTTTCTTTTCTTTTCTTCTT:0	CTTTCTTTTCTTTTCTTCTT:0
chr28	11259606	2	40	T:0.825	TTTC:0.175		
chr28	11259619	3	40	C:0.8	CTTTCT:0.05	CTTTCTTTTCT:0.15	
chr28	11259668	2	38	TTCTC:0.921053	T:0.0789474		
chr28	11259670	4	36	C:0.527778	CTCCTT:0.0555556	CTCCTTTCCTT:0.416667	CTCCTTTCCTTTCCTT:0
chr28	11259675	2	38	T:0.921053	TTTC:0.0789474		
chr28	11259784	2	38	G:0.526316	T:0.473684		
chr28	11260071	2	36	G:0.861111	T:0.138889		

chr28	11260086	2	38	T:0	TTTTAGATC:1		
chr28	11260125	2	38	C:0.921053	T:0.0789474		
chr28	11260401	2	30	CTA:0.9	C:0.1		
chr28	11260415	2	30	CTTT:1	C:0		
chr28	11260667	2	36	G:0.444444	A:0.555556		
chr28	11260926	2	34	G:0.970588	A:0.0294118		
chr28	11261438	2	36	A:0.916667	AC:0.0833333		
chr28	11261560	2	36	G:0.888889	GTT:0.111111		
chr28	11261562	4	36	A:0.472222	AT:0.416667	ATT:0	T:0.111111
chr28	11262845	3	36	C:0.527778	CT:0.444444	CTT:0.0277778	
chr28	11263466	2	38	G:1	A:0		
chr28	11263663	2	40	A:0.9	C:0.1		
chr28	11263809	2	38	C:1	G:0		
chr28	11264751	2	36	C:0.805556	CTTTTTTTTTTTTTT:0.194444		
chr28	11264822	2	32	G:0.1875	C:0.8125		
chr28	11265008	2	38	C:0.5	T:0.5		
chr28	11265410	2	40	C:0.475	T:0.525		
chr28	11266285	2	38	C:0	CA:1		
chr28	11266576	2	38	TATC:0.868421	T:0.131579		
chr28	11266609	3	40	C:0.85	CATT:0.1	CATCATT:0.05	
chr28	11266641	2	36	A:0.722222	G:0.277778		
chr28	11266863	2	36	G:1	A:0		
chr28	11267548	2	36	T:1	C:0		
chr28	11269073	2	40	TTAATACCTGA:0.825	T:0.175		
chr28	11269991	2	38	C:0.157895	T:0.842105		
chr28	11270027	2	40	C:0.9	T:0.1		
chr28	11270760	2	36	G:0.944444	GAA:0.0555556		
chr28	11270764	3	36	C:0	A:0.0555556	CA:0.944444	
chr28	11271011	2	38	G:1	A:0		
chr28	11271584	2	36	G:0.0833333	A:0.916667		
chr28	11272131	2	30	A:0	G:1		
chr28	11272608	2	36	G:0.944444	A:0.0555556		
chr28	11274137	2	30	GT:0.9	G:0.1		
chr28	11274150	2	34	T:1	G:0		
chr28	11274151	3	34	T:0.176471	G:0.470588	TG:0.352941	
chr28	11274749	2	36	A:1	G:0		
chr28	11274914	2	34	T:1	C:0		
chr28	11274951	2	36	C:0.0555556	T:0.944444		
chr28	11274968	2	36	A:0	ATG:1		
chr28	11275014	2	36	G:0.0833333	A:0.916667		
chr28	11275204	2	38	A:1	C:0		
chr28	11275219	2	38	A:0.526316	G:0.473684		
chr28	11275614	2	40	T:0	C:1		
chr28	11275639	2	38	G:0.842105	A:0.157895		
chr28	11276077	2	38	C:1	T:0		
chr28	11276601	2	34	T:0.911765	G:0.0882353		
chr28	11276831	2	38	GAAGAAC:0.947368	G:0.0526316		
chr28	11276837	2	38	C:0.947368	CGTT:0.0526316		
chr28	11277354	2	40	A:0.925	C:0.075		

chr28	11277635	2	34	A:0.911765	G:0.0882353	
chr28	11277721	2	40	C:0.925	T:0.075	
chr28	11278420	2	36	C:0.638889	T:0.361111	
chr28	11278902	2	34	G:1	A:0	
chr28	11278938	2	34	C:0.941176	T:0.0588235	
chr28	11279515	2	36	A:0.805556	C:0.194444	
chr28	11279669	2	34	T:0.941176	C:0.0588235	
chr28	11279683	2	32	T:0.90625	C:0.09375	
chr28	11279726	2	24	GC:0.333333	G:0.666667	
chr28	11279768	2	36	T:0.944444	C:0.0555556	
chr28	11279772	2	36	AC:0.944444	A:0.0555556	
chr28	11279774	2	38	C:0.210526	A:0.789474	
chr28	11279778	2	36	C:0.944444	A:0.0555556	
chr28	11279779	2	38	C:0.210526	A:0.789474	
chr28	11279780	2	36	AAATT:0.944444	A:0.0555556	
chr28	11279784	2	38	T:0.210526	A:0.789474	
chr28	11279788	2	36	CCAAACCAAACCAAAT:0.944444	C:0.0555556	
chr28	11279789	2	34	C:0.0588235	A:0.941176	
chr28	11279794	2	34	C:0.0588235	A:0.941176	
chr28	11279799	2	34	C:0.176471	A:0.823529	
chr28	11279808	2	36	C:0.888889	T:0.111111	
chr28	11280003	2	36	C:0.944444	T:0.0555556	
chr28	11280036	2	34	C:0.941176	T:0.0588235	
chr28	11280704	2	36	GAGAGGA:0.861111	G:0.138889	
chr28	11280712	2	36	G:0.861111	T:0.138889	
chr28	11282085	2	36	C:0.944444	A:0.0555556	
chr28	11282186	2	40	A:1	T:0	
chr28	11282191	2	40	A:1	T:0	
chr28	11282475	2	36	G:0.916667	C:0.0833333	
chr28	11282869	2	36	G:0.861111	A:0.138889	
chr28	11283417	2	40	CAT:1	C:0	
chr28	11284267	2	34	C:0.764706	T:0.235294	
chr28	11284521	2	36	T:1	C:0	
chr28	11284626	2	36	T:0.944444	C:0.0555556	
chr28	11284660	3	38	TAA:0.421053	T:0.105263	TA:0.473684
chr28	11285707	2	36	C:0.916667	T:0.0833333	
chr28	11286498	2	36	T:1	G:0	
chr28	11287034	2	28	C:0.607143	T:0.392857	
chr28	11287104	2	38	A:1	G:0	
chr28	11287174	2	36	G:1	A:0	
chr28	11287793	2	30	C:0.9	A:0.1	
chr28	11288242	2	32	C:1	A:0	
chr28	11289043	2	38	A:0	G:1	
chr28	11289271	2	40	T:0	C:1	
chr28	11291618	2	36	T:0.25	C:0.75	
chr28	11291962	2	40	A:0	G:1	
chr28	11292808	2	36	G:1	A:0	
chr28	11292889	2	36	C:1	T:0	
chr28	11294552	2	36	CT:0.972222	C:0.0277778	
chr28	11294976	2	26	TA:0.769231	T:0.230769	
chr28	11295338	2	40	C:1	T:0	
chr28	11295532	2	34	C:0.911765	T:0.0882353	

chr28	11295568	2	40	C:0.175	G:0.825		
chr28	11295581	4	40	CTT:0.1	C:0.475	CT:0.2	CTTTT:0.225
chr28	11295610	2	38	A:0.815789	G:0.184211		
chr28	11295881	2	40	G:0.75	A:0.25		
chr28	11296048	2	40	T:0.75	G:0.25		
chr28	11296086	3	38	AT:0.736842	A:0	ATT:0.263158	
chr28	11296136	2	38	T:0.263158	C:0.736842		
chr28	11296485	2	40	T:0.25	G:0.75		
chr28	11296493	2	40	GA:0.25	G:0.75		
chr28	11296594	2	36	G:0.861111	A:0.138889		
chr28	11296597	2	36	C:0.861111	T:0.138889		
chr28	11296669	2	40	T:0.85	C:0.15		
chr28	11296786	2	36	A:0.277778	G:0.722222		
chr28	11296838	2	36	C:0.555556	T:0.444444		
chr28	11296852	2	34	G:0.794118	A:0.205882		
chr28	11296855	2	34	A:0.794118	T:0.205882		
chr28	11297063	2	36	C:0.277778	A:0.722222		
chr28	11297074	2	36	T:0	C:1		
chr28	11297102	2	38	T:0.236842	C:0.763158		
chr28	11297140	2	36	A:0.277778	C:0.722222		
chr28	11297177	2	38	C:0.236842	T:0.763158		
chr28	11297189	2	38	T:0.131579	C:0.868421		
chr28	11297276	2	40	C:0.85	G:0.15		
chr28	11297294	2	40	A:0.85	C:0.15		
chr28	11297441	2	36	C:0	CAAAT:1		
chr28	11297774	2	40	GT:1	G:0		
chr28	11297834	2	40	C:0.775	T:0.225		
chr28	11297841	2	40	T:0	C:1		
chr28	11297904	2	38	A:0	G:1		
chr28	11297938	2	38	C:0.236842	T:0.763158		
chr28	11298093	2	40	C:0.75	T:0.25		
chr28	11298219	2	36	A:0.75	AG:0.25		
chr28	11298538	2	40	C:0.75	T:0.25		
chr28	11299765	2	38	C:1	G:0		
chr28	11299866	2	36	G:0.972222	A:0.0277778		
chr28	11300176	2	38	G:0	A:1		
chr28	11300427	2	40	A:0	G:1		
chr28	11300494	2	40	T:0	C:1		
chr28	11300518	2	40	A:0	G:1		
chr28	11300531	2	40	G:0	A:1		
chr28	11300768	2	32	T:0.03125	A:0.96875		
chr28	11300844	2	36	C:0	T:1		
chr28	11301654	2	38	A:1	G:0		
chr28	11301685	2	36	GT:0.555556	G:0.444444		
chr28	11301776	2	32	C:0.09375	CT:0.90625		
chr28	11301899	2	36	C:0	T:1		
chr28	11302619	2	36	G:0.0277778	C:0.972222		
chr28	11302652	2	34	TGGA:0	T:1		
chr28	11302682	2	34	G:0	A:1		
chr28	11303627	2	38	A:0.868421	G:0.131579		
chr28	11304834	2	34	A:0	G:1		
chr28	11304909	2	38	C:0.868421	T:0.131579		

chr28	11305037	2	40	A:0	C:1	
chr28	11305299	2	38	T:1	G:0	
chr28	11306387	2	36	T:1	C:0	
chr28	11306726	2	30	C:0.566667	CT:0.433333	
chr28	11306894	2	34	G:1	A:0	
chr28	11307128	2	40	GT:0.975	G:0.025	
chr28	11309258	2	38	C:1	T:0	
chr28	11310143	2	30	T:1	A:0	
chr28	11310285	2	36	G:1	A:0	
chr28	11311019	2	36	A:1	G:0	
chr28	11311182	2	40	GTTTTT:0.025	G:0.975	
chr28	11311220	2	34	T:0.0294118	C:0.970588	
chr28	11312459	3	36	TA:0.75	T:0.0833333	TAA: 0.166667
chr28	11312919	2	38	A:0	G:1	
chr28	11313232	2	38	C:0.947368	A:0.0526316	
chr28	11313614	2	38	T:1	C:0	
chr28	11315504	2	38	C:0.131579	G:0.868421	
chr28	11315778	2	36	T:1	C:0	
chr28	11315830	2	38	C:1	A:0	
chr28	11315919	2	36	CG:1	C:0	
chr28	11315973	2	38	CAG:1	C:0	
chr28	11316005	2	34	C:1	T:0	
chr28	11316162	2	36	A:0.888889	C:0.111111	
chr28	11316269	2	38	A:1	G:0	
chr28	11316475	2	34	A:0.117647	G:0.882353	
chr28	11316585	2	22	G:0.136364	C:0.863636	
chr28	11316604	2	20	G:0.9	A:0.1	
chr28	11316863	2	28	G:0.964286	A:0.0357143	
chr28	11317055	2	40	T:0.575	C:0.425	
chr28	11317197	2	36	GGTCC:0.583333	G:0.416667	
chr28	11317201	2	36	CG:0.888889	C:0.111111	
chr28	11317203	2	36	TCCG:0.888889	T:0.111111	
chr28	11317231	3	38	GGTGGGCT:0.763158	G: 0.184211	GGTGGGCTGTGGGCT:0.0526316
chr28	11317313	2	40	A:0.9	G:0.1	
chr28	11317402	2	30	T:0.6	A:0.4	
chr28	11317421	2	34	T:0.794118	A:0.205882	
chr28	11317602	2	38	C:0.868421	T:0.131579	
chr28	11317604	2	38	T:1	TC:0	
chr28	11317638	2	38	TA:0.736842	T:0.263158	
chr28	11317642	2	38	A:0.315789	T:0.684211	
chr28	11317686	2	30	A:0.0666667	AAG:0.933333	
chr28	11318037	2	30	A:0.9	G:0.1	
chr28	11318059	2	30	C:0.9	T:0.1	
chr28	11318401	2	30	C:0.833333	T:0.166667	
chr28	11318755	2	34	C:0.882353	T:0.117647	
chr28	11318974	2	28	C:0.678571	CT:0.321429	
chr28	11319198	2	32	A:0.5	T:0.5	
chr28	11319289	2	38	A:0.921053	T:0.0789474	
chr28	11319292	2	38	T:1	A:0	
chr28	11319395	2	34	A:0.0294118	G:0.970588	
chr28	11319487	2	40	C:1	G:0	
chr28	11319777	2	38	G:1	A:0	

chr28	11320022	3	38	CTATATATATATATATATA:0.131579	C:0.473684	CTATATATATA:0.394737
chr28	11320295	2	34	A:1	AT:0	
chr28	11320463	2	36	G:1	A:0	
chr28	11320692	2	38	A:1	AT:0	
chr28	11321220	3	38	CT:0.657895	C:0.105263	CTT:0.236842
chr28	11321302	2	38	T:0.0263158	G:0.973684	
chr28	11321615	2	36	A:1	AT:0	
chr28	11321688	2	32	G:0.875	C:0.125	
chr28	11321848	2	34	G:1	A:0	
chr28	11321885	2	34	G:1	A:0	
chr28	11321925	2	38	A:0.0263158	G:0.973684	
chr28	11322298	2	38	G:0.421053	A:0.578947	
chr28	11322475	2	36	T:0.944444	A:0.0555556	
chr28	11322736	2	38	T:1	G:0	
chr28	11323142	2	38	G:1	A:0	
chr28	11323350	2	40	G:1	C:0	
chr28	11323370	2	40	TGAG:0.85	T:0.15	
chr28	11324084	2	34	A:0.911765	T:0.0882353	
chr28	11324431	3	36	GT:0.888889	G:0.111111	GTTTT:0
chr28	11324732	2	34	T:1	C:0	
chr28	11325054	2	40	T:1	C:0	
chr28	11325291	2	38	C:0.868421	G:0.131579	
chr28	11325305	2	38	T:0.842105	G:0.157895	
chr28	11325854	2	40	A:0.35	G:0.65	
chr28	11326114	3	40	A:0.475	ACAGACATATCATTTTTTTTTTTTTTTAATTTTTTTTTTT:0.325	ACAGACATATCATTTTTTTTTTTTTTTTAATTTTTTTTTTT:0.2
chr28	11326177	2	36	A:0.916667	T:0.0833333	
chr28	11326221	2	40	T:1	G:0	
chr28	11326512	2	38	A:1	T:0	
chr28	11326707	2	36	C:0.972222	T:0.0277778	
chr28	11326756	2	38	A:1	C:0	
chr28	11326942	2	36	A:1	G:0	
chr28	11327080	2	40	G:1	A:0	
chr28	11327354	2	38	C:0.0263158	G:0.973684	
chr28	11327564	2	34	T:0.764706	G:0.235294	
chr28	11327848	2	32	T:0.90625	C:0.09375	
chr28	11327995	2	34	T:0.382353	A:0.617647	
chr28	11328012	2	34	T:0.882353	A:0.117647	
chr28	11328494	2	28	A:0.357143	T:0.642857	
chr28	11328539	2	26	C:0.384615	T:0.615385	
chr28	11329088	3	38	AT:1	A:0	ATT:0
chr28	11329098	2	38	T:0.921053	TC:0.0789474	
chr28	11329128	2	38	C:1	CACAG:0	
chr28	11329135	2	38	A:0.947368	AG:0.0526316	
chr28	11329136	3	38	A:0.947368	AG:0.0526316	G:0
chr28	11329530	2	38	G:0.0263158	C:0.973684	
chr28	11329742	2	34	T:0.852941	A:0.147059	
chr28	11329922	2	30	G:0.9	GT:0.1	
chr28	11329945	2	18	G:0.388889	A:0.611111	

chr28	11329971	2	26	GTGTATATATATATATATATATATA:1	G:0	
chr28	11329973	2	36	GTATATATATA:0.833333	G:0.166667	
chr28	11330070	2	32	T:1	C:0	
chr28	11330116	3	26	GTT:0.423077	G:0.153846	GT:0.423077
chr28	11330293	2	34	G:0.352941	A:0.647059	
chr28	11331216	2	38	A:1	G:0	
chr28	11331797	2	40	T:0.375	G:0.625	
chr28	11332333	2	38	A:1	G:0	
chr28	11332587	2	36	A:0.0555556	AAAAAC:0.944444	
chr28	11332761	2	36	G:1	A:0	
chr28	11333661	2	40	G:0.025	GT:0.975	
chr28	11333686	2	36	G:1	T:0	
chr28	11333928	2	40	C:0.275	G:0.725	
chr28	11333938	2	40	A:0.025	G:0.975	
chr28	11334020	2	34	G:1	T:0	
chr28	11334067	2	38	C:0.921053	A:0.0789474	
chr28	11334948	2	36	C:0.833333	T:0.166667	
chr28	11335240	2	38	G:1	C:0	
chr28	11335262	2	38	A:0.894737	T:0.105263	
chr28	11335382	2	40	A:1	G:0	
chr28	11336565	2	40	CT:0.875	C:0.125	
chr28	11337174	2	34	C:1	T:0	
chr28	11337189	2	30	G:1	A:0	
chr28	11337192	2	30	T:0	C:1	
chr28	11338940	2	38	G:1	C:0	
chr28	11338972	2	40	T:1	C:0	
chr28	11338995	2	36	G:1	GT:0	
chr28	11339013	2	36	T:1	A:0	
chr28	11339016	2	32	G:0.96875	GATTT:0.03125	
chr28	11339345	2	34	T:0.0588235	C:0.941176	
chr28	11339675	2	34	T:0.823529	C:0.176471	
chr28	11340018	2	38	AT:1	A:0	
chr28	11340028	2	38	T:1	A:0	
chr28	11340268	2	40	C:0.825	T:0.175	
chr28	11340368	2	30	C:0.966667	CT:0.0333333	
chr28	11340584	2	34	A:1	G:0	
chr28	11340714	2	38	C:1	T:0	
chr28	11340837	2	38	G:0	GT:1	
chr28	11340842	2	38	G:0	GT:1	
chr28	11341080	2	38	A:1	C:0	
chr28	11342374	2	38	A:0.526316	G:0.473684	
chr28	11342516	2	40	T:1	C:0	
chr28	11342528	2	40	C:0.025	CCTCT:0.975	
chr28	11342901	2	34	A:0.0588235	G:0.941176	
chr28	11342943	2	32	CTTT:0.4375	C:0.5625	
chr28	11343133	2	40	G:1	T:0	
chr28	11343752	3	40	TTTTG:0.2	T:0.575	TTTTGTTTG:0.225
chr28	11343794	2	40	G:0.9	A:0.1	
chr28	11345173	2	36	C:0.861111	CT:0.138889	
chr28	11345441	2	38	G:1	A:0	
chr28	11345575	2	34	T:1	A:0	

chr28	11345772	2	38	A:1	G:0
chr28	11346071	3	36	AT:0.555556	A:0.111111
ATT:0.333333
chr28	11346075	2	38	T:0.894737	A:0.105263
chr28	11346082	2	36	T:0.972222	A:0.0277778
chr28	11346252	2	36	G:0	A:1
chr28	11346276	2	36	G:0	A:1
chr28	11346283	2	36	T:0	A:1
chr28	11346284	2	36	G:0	A:1
chr28	11346293	2	36	T:0	A:1
chr28	11346369	2	36	CA:0	C:1
chr28	11346741	2	40	G:1	A:0
chr28	11347334	2	36	G:1	GT:0
chr28	11347473	2	38	G:1	C:0
chr28	11347805	2	38	G:1	T:0
chr28	11348225	2	34	GT:1	G:0
chr28	11348241	2	34	G:1	A:0
chr28	11348405	2	36	T:0.0277778	TTC:0.972222
chr28	11348569	2	38	CAG:1	C:0
chr28	11349440	2	38	A:1	G:0
chr28	11349578	2	36	G:1	A:0
chr28	11350096	2	38	T:0.973684	A:0.0263158
chr28	11350268	2	36	T:1	TTGTG:0
chr28	11350327	2	36	G:1	GT:0
chr28	11350916	2	34	A:0.323529	G:0.676471
chr28	11351877	2	40	G:1	C:0
chr28	11352296	2	36	C:1	T:0
chr28	11352433	4	38	CTT:0.684211	C:0.0263158
CTTT:0.236842	CTTTT:0.0526316
chr28	11353222	2	36	T:1	C:0
chr28	11353570	2	34	T:0.0294118	C:0.970588
chr28	11353951	2	34	A:1	AG:0
chr28	11353954	3	34	G:0.0294118	GT:0.941176
GTT:0.0294118
chr28	11354007	3	34	TGTGTGGGGGGGG:0.588235	T:0
TGG:0.411765
chr28	11354009	2	38	TGTGGGGGG:1	T:0
chr28	11354011	2	36	TGGGGGGG:0.638889	T:
0.361111
chr28	11354189	2	34	T:1	C:0
chr28	11355172	2	40	C:1	A:0
chr28	11355407	3	40	CT:0.175	C:0.625	CTT:0.2
chr28	11356045	2	34	G:1	A:0
chr28	11356881	3	38	GAA:0.263158	G:0.0263158
GA:0.710526
chr28	11358289	3	36	TA:0.277778	T:0.722222
TAA:0
chr28	11358300	2	40	A:1	C:0
chr28	11358751	2	38	T:0.763158	A:0.236842
chr28	11358783	2	38	T:0.947368	C:0.0526316
chr28	11358785	2	38	C:1	T:0
chr28	11358995	2	38	A:1	T:0
chr28	11359571	2	40	T:0.9	A:0.1
chr28	11359911	3	32	CT:0.71875	C:0.1875	CTT:

0.09375

chr28	11360031	2	40	A:0.975	G:0.025
chr28	11360684	2	36	C:0	T:1
chr28	11360703	2	36	TTGAC:1	T:0
chr28	11360990	2	34	G:1	C:0
chr28	11361323	2	34	C:1	T:0
chr28	11361862	2	38	A:1	T:0
chr28	11361867	2	38	A:1	G:0
chr28	11362406	2	36	A:0.861111	G:0.138889
chr28	11362523	2	38	A:1	G:0
chr28	11362701	2	40	T:1	C:0
chr28	11362897	2	38	A:0.447368	G:0.552632
chr28	11362904	2	38	CAT:0.973684	C:0.0263158
chr28	11363289	2	40	T:1	C:0
chr28	11363460	2	36	CT:0.805556	C:0.194444
chr28	11363642	2	38	T:0.0263158	C:0.973684
chr28	11363936	2	38	A:0.894737	C:0.105263
chr28	11363960	2	36	G:1	A:0
chr28	11364022	2	38	T:1	C:0
chr28	11364054	2	40	TAAA:1	T:0
chr28	11364938	2	38	C:1	T:0
chr28	11365347	2	38	T:1	C:0
chr28	11366535	2	40	A:0.775	T:0.225
chr28	11367224	2	38	A:1	G:0
chr28	11367266	2	40	G:1	A:0
chr28	11367613	2	38	G:0.0263158	A:0.973684
chr28	11368188	2	38	G:1	C:0
chr28	11368883	2	38	G:1	A:0
chr28	11368930	2	40	G:1	A:0
chr28	11369634	2	36	C:1	T:0
chr28	11369698	2	36	A:0.916667	G:0.0833333
chr28	11369707	2	36	C:1	T:0
chr28	11369865	2	36	C:1	A:0
chr28	11369931	2	38	T:0.0526316	TAC:0.947368
chr28	11370083	2	40	G:0.325	T:0.675
chr28	11370174	2	36	C:0.416667	T:0.583333
chr28	11370406	2	38	G:1	C:0
chr28	11370827	2	38	TTC:0.736842	T:0.263158
chr28	11370879	3	32	CA:0.3125	C:0.28125
CAA:0.40625					
chr28	11371043	2	36	T:0.75	A:0.25
chr28	11372112	2	40	T:1	A:0
chr28	11372115	2	40	A:1	AT:0
chr28	11372357	2	38	G:1	A:0
chr28	11372999	2	36	G:1	A:0
chr28	11373109	2	32	A:1	G:0
chr28	11373317	2	34	A:0.735294	AT:0.264706
chr28	11373539	2	36	G:1	A:0
chr28	11373568	2	36	G:1	A:0
chr28	11374137	2	34	C:0.764706	T:0.235294
chr28	11374318	2	40	G:1	A:0
chr28	11375016	2	40	T:0	A:1
chr28	11375064	2	38	T:1	C:0
chr28	11375204	2	36	C:0.75	CT:0.25

chr28	11375257	2	38	CAG:0.973684	C:0.0263158
chr28	11376138	2	34	G:1	A:0
chr28	11376448	2	34	A:1	T:0
chr28	11376449	2	32	C:0.625	T:0.375
chr28	11376645	2	38	C:1	T:0
chr28	11377176	2	36	C:0.0277778	T:0.972222
chr28	11377785	2	38	GA:1	G:0
chr28	11377847	2	32	T:0.03125	C:0.96875
chr28	11377911	2	36	G:1	A:0
chr28	11377940	2	34	GAT:1	G:0
chr28	11378893	2	24	GT:0.375	G:0.625
chr28	11379024	2	36	C:0.861111	T:0.138889
chr28	11380042	3	40	G:0.875
GCCAAATAGTCTTTTTATTTTTTTTTTTTATTTTTTTATTTTTTTATTTTTTT:0.025
GCCAAATAGTCTTTTTATTTTTTTTTTTTATTTTTTTATTTTTTTTTTTTTTT:0.1
chr28	11380061	2	38	C:1	T:0
chr28	11380180	2	36	C:0.777778	CT:0.222222
chr28	11380262	2	38	A:0.447368	G:0.552632
chr28	11380346	2	40	TAA:0.725	T:0.275
chr28	11380606	2	40	G:1	GTTTAGTAACA:0
chr28	11380786	2	36	TG:0.888889	T:0.111111
chr28	11381145	3	32	A:0.0625	ATT:0.34375	ATTAT:
0.59375
chr28	11381146	2	32	A:1	T:0
chr28	11381457	3	38	CTTAT:0.736842	C:0.236842
CTTATTTAT:0.0263158
chr28	11381771	2	38	C:0.0263158	T:0.973684
chr28	11381789	2	36	G:1	A:0
chr28	11381828	2	36	C:1	A:0
chr28	11382448	2	38	G:1	A:0
chr28	11382674	2	36	C:0	T:1
chr28	11382703	2	36	G:1	A:0
chr28	11382784	2	36	C:0.638889	CT:0.361111
chr28	11382796	2	36	C:0.583333	CT:0.416667
chr28	11382890	2	36	A:1	G:0
chr28	11382915	2	34	CG:0.323529	C:0.676471
chr28	11382929	2	34	C:0.323529	T:0.676471
chr28	11383210	2	32	C:1	T:0
chr28	11384031	2	32	C:1	A:0
chr28	11384336	2	38	T:1	C:0
chr28	11384435	3	36	GTTTTTTTTTTTTT:0.472222	G:
0.277778	GTT:0.25
chr28	11384574	2	32	G:1	A:0
chr28	11384657	2	32	C:0.90625	A:0.09375
chr28	11385087	2	36	C:0.0277778	T:0.972222
chr28	11385266	2	36	C:1	T:0
chr28	11385325	2	32	G:1	A:0
chr28	11385610	2	32	C:0.0625	T:0.9375
chr28	11385832	2	36	CA:0.0277778	C:0.972222
chr28	11385878	2	38	G:1	A:0
chr28	11386115	2	36	C:1	T:0
chr28	11386490	2	36	T:0.388889	A:0.611111
chr28	11386521	2	38	T:1	G:0
chr28	11386861	3	36	TAA:0.5	T:0.0555556	TA:

0.444444
chr28	11387684 2	38	T:1	G:0
chr28	11387819 2	38	ATGATTTTAAT:0.973684	A:
0.0263158
chr28	11388558 2	30	TA:0.733333	T:0.266667
chr28	11388762 2	34	G:0.441176	A:0.558824
chr28	11388982 2	34	A:0.852941	G:0.147059
chr28	11389436 2	40	C:1	CT:0
chr28	11389806 2	40	C:0.9	T:0.1
chr28	11390211 2	38	G:0.921053	A:0.0789474
chr28	11390298 2	38	G:0.0263158	A:0.973684
chr28	11390661 2	40	CTT:0.375	C:0.625
chr28	11390684 2	40	C:1	A:0
chr28	11391337 2	38	G:1	A:0
chr28	11392149 2	36	G:1	A:0
chr28	11392155 2	36	C:1	T:0
chr28	11392243 2	38	GTGAAAT:1	G:0
chr28	11392462 2	40	GAC:1	G:0
chr28	11393180 2	36	G:0.138889	GT:0.861111
chr28	11393771 2	38	G:0.921053
GAGAGAGAGAGAGAGAGAGAGAA:0.0789474
chr28	11393782 2	24	A:0.666667	AG:0.333333
chr28	11393783 3	38	A:0.657895	AG:0.315789
AGAGAGAGAAAGAG:0.0263158
chr28	11393791 2	30	A:0.8	AAGAG:0.2
chr28	11393795 2	32	GAGAA:0.59375	G:0.40625
chr28	11393845 2	38	G:0.921053	GAA:0.0789474
chr28	11393846 2	36	AG:0.222222	A:0.777778
chr28	11393849 2	36	A:1	AAG:0
chr28	11393850 3	38	A:0.263158	G:0.736842
AG:0
chr28	11393870 2	38	GA:1	G:0
chr28	11393942 2	30	T:0.633333	TTA:0.366667
chr28	11393957 2	36	TA:0.916667	T:0.0833333
chr28	11393959 2	38	T:0.868421	TATA:0.131579
chr28	11394662 2	32	G:1	A:0
chr28	11395143 2	38	A:1	T:0
chr28	11395453 2	38	C:1	T:0
chr28	11395604 3	34	CTT:0.705882	C:0.0294118
TTT:0.264706
chr28	11395984 2	36	C:1	T:0
chr28	11396231 2	38	G:0.0263158	T:0.973684
chr28	11396469 2	36	C:0.361111	T:0.638889
chr28	11396726 2	38	T:1	C:0
chr28	11396729 2	38	A:1	G:0
chr28	11396817 2	34	C:1	T:0
chr28	11396858 2	32	C:1	A:0
chr28	11397076 2	40	G:1	A:0
chr28	11397620 2	38	GTT:0.0789474	G:0.921053
chr28	11397684 2	36	AAGAG:0.388889	A:0.611111
chr28	11397748 2	30	A:0.0333333	G:0.966667
chr28	11397771 2	30	C:0.833333	T:0.166667
chr28	11398479 2	38	T:0.263158	C:0.736842
chr28	11398842 2	38	G:1	A:0

chr28	11399261	2	38	T:1	C:0	
chr28	11399472	2	36	C:1	T:0	
chr28	11400792	2	40	A:0.025	T:0.975	
chr28	11401721	2	38	G:0.289474	A:0.710526	
chr28	11401844	2	36	G:1	A:0	
chr28	11401962	2	38	G:0.842105	A:0.157895	
chr28	11402177	2	38	C:0.736842	A:0.263158	
chr28	11402263	2	38	C:1	T:0	
chr28	11402468	2	36	G:0.916667	T:0.0833333	
chr28	11402469	2	36	A:0.916667	T:0.0833333	
chr28	11402596	2	38	T:1	C:0	
chr28	11402600	3	40	CCTCTCTCTCT:0.475	C:0.35	CCTCTCTCTCTCTCT:0.175
chr28	11402645	2	26	TA:0.5	T:0.5	
chr28	11402796	2	36	C:0.75	T:0.25	
chr28	11403468	2	34	C:0.735294	T:0.264706	
chr28	11403533	2	38	A:1	G:0	
chr28	11403536	2	38	T:0.710526	C:0.289474	
chr28	11404631	2	32	T:0.03125	C:0.96875	
chr28	11404670	2	36	A:0.722222	C:0.277778	
chr28	11404882	2	36	G:0	C:1	
chr28	11406064	2	38	C:0.815789	T:0.184211	
chr28	11406676	2	34	T:0.911765	C:0.0882353	
chr28	11406697	2	34	T:0.323529	A:0.676471	
chr28	11406738	2	30	AT:0.0333333	A:0.966667	
chr28	11406755	2	32	G:0.28125	A:0.71875	
chr28	11406933	2	36	G:0.888889	A:0.111111	
chr28	11406993	2	32	T:0.03125	C:0.96875	
chr28	11407521	2	38	G:0.0263158	GT:0.973684	
chr28	11407834	2	30	G:1	A:0	
chr28	11409745	2	32	T:0.25	C:0.75	
chr28	11410506	2	32	T:1	C:0	
chr28	11410817	2	38	G:1	C:0	
chr28	11410823	2	38	AT:0.315789	A:0.684211	
chr28	11411325	2	34	G:0.764706	A:0.235294	
chr28	11411580	2	38	C:0.5	A:0.5	
chr28	11411684	2	32	G:0.375	A:0.625	
chr28	11411741	2	38	T:0.263158	C:0.736842	
chr28	11411795	2	36	A:1	G:0	
chr28	11411861	2	40	T:0.375	C:0.625	
chr28	11411938	2	36	C:0.777778	T:0.222222	
chr28	11411995	2	36	CCCA:0.305556	C:0.694444	
chr28	11412144	2	36	A:0.333333	C:0.666667	
chr28	11412181	2	38	T:0.342105	G:0.657895	
chr28	11412203	2	38	C:0.342105	T:0.657895	
chr28	11412265	2	40	T:0.925	C:0.075	
chr28	11412370	2	40	C:0.575	T:0.425	
chr28	11412429	2	38	G:1	A:0	
chr28	11412463	2	32	A:0.0625	G:0.9375	
chr28	11412904	2	36	C:0.611111	T:0.388889	
chr28	11412963	2	38	A:0.368421	C:0.631579	
chr28	11413178	2	36	A:0.277778	G:0.722222	
chr28	11413588	2	38	T:0.368421	C:0.631579	
chr28	11413779	2	34	G:0.264706	C:0.735294	

chr28	11415240	2	38	C:0.842105	T:0.157895
chr28	11415441	2	38	G:1	GC:0
chr28	11415479	2	40	G:1	A:0
chr28	11415635	2	36	C:1	A:0
chr28	11415710	2	36	C:0.194444	T:0.805556
chr28	11416137	2	38	T:0.210526	G:0.789474
chr28	11416184	2	38	C:1	T:0
chr28	11416385	2	38	C:0.289474	T:0.710526
chr28	11416393	2	38	A:0.289474	G:0.710526
chr28	11416421	2	40	C:0.325	T:0.675
chr28	11416931	2	40	G:0.775	A:0.225
chr28	11417125	2	36	C:0.25	A:0.75
chr28	11417144	2	38	C:0.315789	G:0.684211
chr28	11417358	2	32	G:0.15625	A:0.84375
chr28	11417380	2	34	T:0.294118	C:0.705882
chr28	11417401	2	36	G:0.277778	T:0.722222
chr28	11417573	2	36	C:1	T:0
chr28	11417577	2	36	A:0.25	G:0.75
chr28	11417769	2	38	C:1	T:0
chr28	11418418	2	38	C:0.894737	CTTA:0.105263
chr28	11418658	2	32	G:0.90625	A:0.09375
chr28	11418862	2	30	C:0.233333	T:0.766667
chr28	11419163	2	18	G:0.0555556	A:0.944444
chr28	11419363	2	12	A:0.583333	T:0.416667
chr28	11420115	2	28	C:0.0357143	G:0.964286
chr28	11420140	2	26	G:0.692308	A:0.307692
chr28	11420191	2	26	A:0.346154	T:0.653846
chr28	11420278	2	24	G:0.708333	A:0.291667
chr28	11420342	2	24	G:1	GC:0
chr28	11420347	2	22	G:0	GC:1
chr28	11420354	2	22	C:0	T:1
chr28	11420399	2	24	AC:0	A:1
chr28	11420410	2	24	C:0	T:1
chr28	11420543	2	28	C:0.0714286	G:0.928571
chr28	11420689	2	28	T:0.821429	A:0.178571
chr28	11421086	2	32	C:0.6875	T:0.3125
chr28	11421306	2	36	C:0.0277778	G:0.972222
chr28	11421383	2	36	C:0.388889	T:0.611111
chr28	11421567	2	36	T:0.611111	C:0.388889
chr28	11421735	2	36	C:0.0555556	A:0.944444
chr28	11421942	2	38	T:0.105263	C:0.894737
chr28	11422206	2	34	G:0.0882353	A:0.911765
chr28	11422385	2	34	CT:1	C:0
chr28	11422386	2	34	T:0.0588235	C:0.941176
chr28	11422387	2	34	G:1	C:0
chr28	11422461	2	36	G:0.444444	A:0.555556
chr28	11422483	2	36	G:0.638889	A:0.361111
chr28	11422662	2	34	G:1	C:0
chr28	11422677	2	34	C:0.647059	T:0.352941
chr28	11422834	2	38	C:1	G:0
chr28	11423120	2	34	G:0.147059	A:0.852941
chr28	11423200	2	34	G:0.529412	A:0.470588
chr28	11423349	2	34	G:0.588235	A:0.411765
chr28	11423590	2	34	G:0.0588235	A:0.941176

chr28	11424170	2	40	C:0.1	CT:0.9	
chr28	11424205	2	38	C:0.815789	T:0.184211	
chr28	11424287	2	38	T:1	TG:0	
chr28	11424305	2	36	C:0.5	T:0.5	
chr28	11424429	3	30	ATTTTTTT:0.666667	A:0.2	ATTTT:0.133333
chr28	11424609	2	36	C:0.75	G:0.25	
chr28	11424683	2	30	T:0.7	TTGAAC:0.3	
chr28	11424708	2	32	C:0.84375	T:0.15625	
chr28	11424721	2	32	T:0.59375	C:0.40625	
chr28	11424818	2	34	C:0.823529	T:0.176471	
chr28	11424884	2	30	C:0.733333	T:0.266667	
chr28	11425255	2	38	C:0.868421	T:0.131579	
chr28	11425269	2	38	T:0.342105	C:0.657895	
chr28	11425339	2	40	G:0.85	C:0.15	
chr28	11425346	2	40	G:0.75	A:0.25	
chr28	11425922	2	38	G:0.815789	T:0.184211	
chr28	11425958	2	36	A:0.888889	C:0.111111	
chr28	11426016	2	36	A:0.861111	AT:0.138889	
chr28	11426159	2	40	A:0.875	G:0.125	
chr28	11426266	2	34	G:0.617647	A:0.382353	
chr28	11426282	2	32	T:0.8125	C:0.1875	
chr28	11426438	2	32	T:0.84375	A:0.15625	
chr28	11426621	2	36	A:0.527778	G:0.472222	
chr28	11426737	2	38	A:0.921053	C:0.0789474	
chr28	11426791	2	36	T:0.888889	C:0.111111	
chr28	11427406	2	36	A:0.722222	C:0.277778	
chr28	11427446	2	34	G:0.617647	A:0.382353	
chr28	11427512	2	38	T:0.947368	G:0.0526316	
chr28	11427715	2	34	C:0.882353	T:0.117647	
chr28	11427820	2	40	G:0.7	T:0.3	
chr28	11427838	2	40	T:0.725	C:0.275	
chr28	11427908	2	32	C:1	A:0	
chr28	11428001	2	32	G:0.75	C:0.25	
chr28	11428014	2	38	C:0.789474	T:0.210526	
chr28	11428071	2	36	T:0.583333	C:0.416667	
chr28	11428105	2	34	T:0.794118	G:0.205882	
chr28	11428263	2	36	A:0.805556	G:0.194444	
chr28	11428386	2	38	A:0.868421	G:0.131579	
chr28	11428887	2	40	T:1	C:0	
chr28	11428889	2	40	A:0.775	G:0.225	
chr28	11428965	2	36	A:0.527778	C:0.472222	
chr28	11429020	2	38	A:1	G:0	
chr28	11429207	2	38	GTT:1	G:0	
chr28	11429220	2	40	C:0.775	T:0.225	
chr28	11429733	2	38	A:0.5	T:0.5	
chr28	11429844	2	38	G:0.973684	A:0.0263158	
chr28	11429874	2	36	A:0.472222	G:0.527778	
chr28	11429887	2	38	C:0.763158	CTT:0.236842	
chr28	11430082	2	38	A:0.447368	T:0.552632	
chr28	11430108	2	38	C:0.394737	T:0.605263	
chr28	11430770	2	36	A:1	C:0	
chr28	11431115	2	36	T:0.75	C:0.25	
chr28	11431529	2	38	G:0.631579	C:0.368421	

chr28 11431632 2 36 A:0.805556 G:0.194444
chr28 11431899 2 40 G:1 A:0
chr28 11432698 2 36 T:1 A:0
chr28 11432793 2 38 C:0.605263 T:0.394737
chr28 11432958 2 38 TA:0.815789 T:0.184211
chr28 11433004 2 38 C:0.605263 T:0.394737
chr28 11433034 2 38 C:0.973684 G:0.0263158
chr28 11433414 2 36 T:0.805556 TAAACAA:0.194444
chr28 11434096 2 34 CTTTT:0.735294 C:0.264706
chr28 11434131 2 36 T:0.694444 C:0.305556
chr28 11434149 2 38 G:0.763158 A:0.236842
chr28 11434291 2 40 A:0.8 C:0.2
chr28 11434368 2 40 G:0.45 A:0.55
chr28 11434415 2 36 G:0.833333 A:0.166667
chr28 11434527 2 38 A:0.789474 G:0.210526
chr28 11434650 2 26 T:0.807692 TGTG:0.192308
chr28 11435664 2 34 G:0.676471 A:0.323529
chr28 11435675 2 34 G:0.676471 T:0.323529
chr28 11435691 2 32 C:1 T:0
chr28 11435692 2 32 G:1 T:0
chr28 11435780 2 40 G:0.725 C:0.275
chr28 11436332 2 34 G:0.852941 A:0.147059
chr28 11436376 2 34 A:0.0294118 G:0.970588
chr28 11436526 5 40 GGA:0.325 G:0.025 GGAGA:
0.25 GGAGAGA:0.1 GGAGAGAGA:0.3
chr28 11436632 2 36 C:0.611111 T:0.388889
chr28 11436634 2 36 G:0.805556 A:0.194444
chr28 11436761 2 36 C:1 T:0
chr28 11436807 2 32 T:0.78125 C:0.21875
chr28 11436968 2 40 G:1 A:0
chr28 11437489 2 40 A:0.85 C:0.15
chr28 11437610 2 34 T:0.0294118 C:0.970588
chr28 11437708 2 38 C:0.631579 A:0.368421
chr28 11438036 2 34 C:0.647059 T:0.352941
chr28 11438150 2 38 CT:0.0263158 C:0.973684
chr28 11438221 2 34 T:0.441176 C:0.558824
chr28 11438900 2 34 G:0.529412 A:0.470588
chr28 11439207 2 34 C:0.735294 T:0.264706
chr28 11439218 2 30 T:0.366667 C:0.633333
chr28 11439221 2 32 G:0.625 T:0.375
chr28 11439498 2 36 C:0.916667 T:0.0833333
chr28 11439553 2 36 C:1 T:0
chr28 11439861 2 38 A:0.5 C:0.5
chr28 11439912 2 38 G:0.578947 A:0.421053
chr28 11440127 2 40 C:0.85 T:0.15
chr28 11440362 2 36 C:0.916667 T:0.0833333
chr28 11440596 2 34 A:1 T:0
chr28 11440624 2 34 TG:0.264706 T:0.735294
chr28 11440955 2 36 C:0.805556 A:0.194444
chr28 11440979 2 36 C:0.722222 T:0.277778
chr28 11441034 2 32 C:0.53125 A:0.46875
chr28 11441221 2 38 C:0.842105 T:0.157895
chr28 11441237 2 38 T:0.736842 C:0.263158
chr28 11441467 2 38 C:0.736842 T:0.263158

chr28	11441654	2	38	C:0.368421	T:0.631579	
chr28	11441685	2	38	T:0.842105	G:0.157895	
chr28	11441756	2	40	A:0.325	G:0.675	
chr28	11441841	2	38	T:0.342105	C:0.657895	
chr28	11441881	2	38	G:0.368421	A:0.631579	
chr28	11441889	2	38	T:0.368421	C:0.631579	
chr28	11442035	2	40	A:0.4	C:0.6	
chr28	11442388	2	34	G:0.411765	C:0.588235	
chr28	11442453	2	36	T:1	A:0	
chr28	11443094	2	40	A:0.4	G:0.6	
chr28	11443106	2	40	G:0.825	A:0.175	
chr28	11443191	2	38	G:0.368421	A:0.631579	
chr28	11443231	2	38	A:0.394737	T:0.605263	
chr28	11443345	2	36	T:0.805556	C:0.194444	
chr28	11443407	2	36	C:0.722222	A:0.277778	
chr28	11443603	2	30	CTATCT:0.966667	C:0.0333333	
chr28	11443605	2	36	ATCTAATCT:0.722222	A: 0.277778	
chr28	11443607	2	34	CT:0.764706	C:0.235294	
chr28	11443608	2	40	TA:0.775	T:0.225	
chr28	11443609	2	38	AATCT:0.947368	A:0.0526316	
chr28	11443610	2	32	ATCT:0.71875	A:0.28125	
chr28	11443612	2	40	C:0.775	CA:0.225	
chr28	11443613	2	40	T:0.775	A:0.225	
chr28	11443968	2	40	T:0.8	C:0.2	
chr28	11444045	2	38	G:0.842105	C:0.157895	
chr28	11444217	2	34	G:0.852941	A:0.147059	
chr28	11444259	2	30	C:0.8	T:0.2	
chr28	11444508	3	4	TAA:0.25	T:0.75	TA:0
chr28	11444585	2	38	C:0.842105	G:0.157895	
chr28	11444718	2	36	A:0.722222	G:0.277778	
chr28	11445107	2	38	A:0.789474	AG:0.210526	
chr28	11445115	2	40	G:0.8	C:0.2	
chr28	11446124	2	38	C:0.684211	G:0.315789	
chr28	11446159	2	40	C:0.775	T:0.225	
chr28	11446167	2	40	G:0.775	A:0.225	
chr28	11446192	2	40	C:0.8	T:0.2	
chr28	11446200	2	38	G:0.815789	C:0.184211	
chr28	11446255	2	36	G:1	A:0	
chr28	11446275	2	36	T:0.777778	G:0.222222	
chr28	11446490	2	38	T:0.447368	C:0.552632	
chr28	11446497	2	38	G:0.763158	A:0.236842	
chr28	11446963	2	36	G:1	A:0	
chr28	11447015	2	34	A:0	G:1	
chr28	11447231	2	36	G:0.805556	A:0.194444	
chr28	11447532	2	38	G:0.578947	A:0.421053	
chr28	11447712	2	38	T:0.447368	TATCTC:0.552632	
chr28	11448227	2	40	AGCTGGTCCAT:0.675	A:0.325	
chr28	11448339	2	32	T:0.75	G:0.25	
chr28	11448341	2	32	T:1	C:0	
chr28	11448848	2	40	CAACAGGG:0.8	C:0.2	
chr28	11449428	2	34	C:0.764706	T:0.235294	
chr28	11449436	2	34	AC:0.411765	A:0.588235	
chr28	11449587	2	34	G:0.764706	A:0.235294	

chr28	11449611	2	40	A:0.775	G:0.225
chr28	11449847	2	36	C:0.75	T:0.25
chr28	11449862	2	36	A:0.833333	G:0.166667
chr28	11449948	2	38	A:0.736842	G:0.263158
chr28	11450011	2	40	C:0.8	A:0.2
chr28	11450081	2	34	T:0.794118	G:0.205882
chr28	11450420	2	36	C:1	T:0
chr28	11450463	2	40	C:1	T:0
chr28	11450545	2	40	A:0.8	T:0.2
chr28	11450739	2	38	G:0.684211	A:0.315789
chr28	11450777	2	38	T:0.210526	C:0.789474
chr28	11450789	2	38	A:0.210526	C:0.789474
chr28	11450968	2	38	G:0.315789	A:0.684211
chr28	11451049	2	38	A:0.763158	AGG:0.236842
chr28	11451066	2	38	A:0.236842	G:0.763158
chr28	11451117	2	38	G:0.447368	C:0.552632
chr28	11451126	2	40	C:0.75	CGACTGGTGG:0.25
chr28	11451168	2	38	A:0.736842	C:0.263158
chr28	11451413	2	28	A:0.857143	G:0.142857
chr28	11452138	2	36	T:1	C:0
chr28	11452230	2	26	G:0.730769	A:0.269231
chr28	11452504	2	12	AGG:0.833333	A:0.166667
chr28	11452694	2	30	G:0.766667	T:0.233333
chr28	11452814	2	38	T:0.763158	C:0.236842
chr28	11452912	2	38	C:1	T:0
chr28	11452913	2	38	A:0.763158	G:0.236842
chr28	11452971	2	36	T:1	C:0
chr28	11452972	2	36	TGTG:0.75	T:0.25
chr28	11452982	2	36	C:0.75	G:0.25
chr28	11453002	2	34	T:0.764706	C:0.235294
chr28	11453120	2	34	C:0.735294	A:0.264706
chr28	11453144	2	34	G:0.764706	A:0.235294
chr28	11453154	2	36	T:0.805556	C:0.194444
chr28	11453161	2	36	T:0.805556	G:0.194444
chr28	11453179	2	36	T:0.805556	C:0.194444
chr28	11453221	2	38	G:0.763158	A:0.236842
chr28	11453242	2	38	A:0.763158	C:0.236842
chr28	11453243	2	38	C:0.763158	T:0.236842
chr28	11453355	2	34	C:0.735294	T:0.264706
chr28	11453388	2	32	C:0.71875	CT:0.28125
chr28	11453520	2	36	G:0.777778	A:0.222222
chr28	11453524	2	34	C:0	CT:1
chr28	11453566	2	34	G:0.794118	A:0.205882
chr28	11453615	2	36	C:0.75	T:0.25
chr28	11453665	2	34	AG:1	A:0
chr28	11453669	2	34	G:0.764706	A:0.235294
chr28	11453727	2	36	T:0.722222	A:0.277778
chr28	11453737	2	36	T:0.722222	C:0.277778
chr28	11453738	2	36	A:0.722222	T:0.277778
chr28	11453762	2	36	A:0.694444	G:0.305556
chr28	11453969	2	38	C:0.710526	A:0.289474
chr28	11454093	2	40	GC:0.775	G:0.225
chr28	11454095	2	40	AT:0.775	A:0.225
chr28	11454115	2	40	C:0.775	T:0.225

chr28	11454364	2	38	G:1	A:0	
chr28	11454578	2	32	GA:0.65625	G:0.34375	
chr28	11454670	2	32	GA:1	G:0	
chr28	11454721	2	34	C:0.764706	T:0.235294	
chr28	11454767	2	38	C:1	G:0	
chr28	11454777	2	34	GT:0.911765	G:0.0882353	
chr28	11454794	2	38	G:1	A:0	
chr28	11454897	2	32	C:1	T:0	
chr28	11454945	2	36	G:1	A:0	
chr28	11455006	2	38	G:0.684211	A:0.315789	
chr28	11455089	2	40	G:1	A:0	
chr28	11455146	2	38	TAGGTCAC:0.973684	T:0.0263158	
chr28	11455185	2	36	C:0.833333	A:0.166667	
chr28	11455244	2	40	G:0.725	A:0.275	
chr28	11455431	2	40	C:1	T:0	
chr28	11455510	2	38	A:1	G:0	
chr28	11455519	2	38	T:1	A:0	
chr28	11455548	2	40	T:1	G:0	
chr28	11455648	2	38	A:0.789474	C:0.210526	
chr28	11456272	2	38	T:0.473684	C:0.526316	
chr28	11456386	2	38	A:0.736842	C:0.263158	
chr28	11456427	2	38	C:0.710526	T:0.289474	
chr28	11456677	2	32	T:1	C:0	
chr28	11456918	2	34	A:1	G:0	
chr28	11457146	2	36	A:1	T:0	
chr28	11457445	2	32	A:1	G:0	
chr28	11457551	2	38	C:1	T:0	
chr28	11457766	2	38	G:0.842105	A:0.157895	
chr28	11458514	2	38	C:1	T:0	
chr28	11458601	2	34	G:1	A:0	
chr28	11458672	2	36	A:0.777778	AT:0.222222	
chr28	11458715	2	36	T:1	C:0	
chr28	11458967	2	36	T:1	A:0	
chr28	11459007	2	38	A:1	AG:0	
chr28	11459431	2	36	T:1	A:0	
chr28	11459445	2	36	G:1	A:0	
chr28	11459521	2	28	G:0.857143	A:0.142857	
chr28	11459530	2	32	G:1	A:0	
chr28	11459536	2	36	G:1	A:0	
chr28	11459549	2	36	C:1	T:0	
chr28	11459552	2	38	G:0.815789	A:0.184211	
chr28	11459572	2	38	G:0.815789	A:0.184211	
chr28	11459585	2	40	G:0.875	A:0.125	
chr28	11459667	2	34	A:1	G:0	
chr28	11459673	2	34	AT:0.735294	A:0.264706	
chr28	11459680	2	32	C:0.96875	A:0.03125	
chr28	11459682	2	34	A:0.735294	C:0.264706	
chr28	11459865	3	34	TG:0.558824	T:0.411765	GG:0.0294118
chr28	11459956	2	38	C:1	T:0	
chr28	11460400	2	38	C:0.578947	CT:0.421053	
chr28	11460725	2	40	T:0.775	C:0.225	
chr28	11460854	2	30	C:0.733333	T:0.266667	

chr28 11461325 3 36 TAA:0.611111 T:0.138889
TA:0.25
chr28 11461353 2 32 C:0.8125 T:0.1875
chr28 11461437 2 36 A:0.722222 G:0.277778
chr28 11461946 2 34 G:1 T:0
chr28 11463041 2 36 T:0.638889 C:0.361111
chr28 11463062 2 36 A:0.638889 G:0.361111
chr28 11463108 2 36 T:0.777778 A:0.222222
chr28 11463293 2 32 CG:0.625 C:0.375
chr28 11463420 2 34 T:0.617647 C:0.382353
chr28 11463474 2 38 G:0.894737 T:0.105263
chr28 11464030 2 38 A:0.631579 C:0.368421
chr28 11464210 2 38 T:0.5 C:0.5
chr28 11464364 2 34 A:0.0294118 T:0.970588
chr28 11464436 2 34 C:0.764706 A:0.235294
chr28 11465027 2 36 C:0.472222 G:0.527778
chr28 11465227 2 38 G:0.763158 T:0.236842
chr28 11465587 2 36 T:0.527778 C:0.472222
chr28 11465771 2 40 T:0.775 G:0.225
chr28 11466352 2 38 T:0.5 C:0.5
chr28 11466397 2 36 G:0.777778 A:0.222222
chr28 11466444 2 34 A:0.382353 G:0.617647
chr28 11467469 2 36 C:0.805556 A:0.194444
chr28 11467596 4 40 AGGATGGAT:0.55 A:0.45 AGGAT:0
AGGATGGATGGAT:0
chr28 11469003 2 34 G:1 A:0
chr28 11469347 2 36 T:0.555556 C:0.444444
chr28 11469374 2 38 A:1 T:0
chr28 11469448 2 34 G:1 A:0
chr28 11469466 2 40 C:0.8 T:0.2
chr28 11469490 2 40 G:0.8 A:0.2
chr28 11469498 2 40 A:1 G:0
chr28 11469666 2 36 G:0.777778 C:0.222222
chr28 11469897 2 34 G:0.764706 C:0.235294
chr28 11470230 2 38 T:1 C:0
chr28 11470342 2 36 TGTCA:0.805556 T:0.194444
chr28 11471124 2 38 G:1 C:0
chr28 11471161 2 36 G:1 A:0
chr28 11471168 2 36 C:0.944444 T:0.0555556
chr28 11471323 2 38 GATATAAATCTGCACACA:0.815789
G:0.184211
chr28 11471334 2 38 G:0.710526 A:0.289474
chr28 11471434 2 38 C:1 T:0
chr28 11471804 2 38 T:0.736842 G:0.263158
chr28 11471966 2 36 T:1 C:0
chr28 11472208 2 38 A:0.710526 G:0.289474
chr28 11472488 2 32 T:0.6875 G:0.3125
chr28 11472688 2 34 G:1 A:0
chr28 11472799 2 40 C:1 T:0
chr28 11472986 2 36 T:0.694444 C:0.305556
chr28 11473001 2 36 C:0.638889 T:0.361111
chr28 11473356 2 36 C:0.777778 G:0.222222
chr28 11473384 2 36 G:1 T:0
chr28 11473414 2 36 A:0.75 T:0.25

chr28	11473688	2	38	A:0.789474		G:0.210526
chr28	11474130	2	40	T:0.775	C:0.225	
chr28	11474197	2	36	T:0.694444		C:0.305556
chr28	11474961	2	38	G:1	A:0	
chr28	11475340	2	40	A:1	G:0	
chr28	11475421	2	38	G:1	C:0	
chr28	11475581	2	40	GT:0.8	G:0.2	
chr28	11475811	2	36	G:1	A:0	
chr28	11476024	2	38	G:1	A:0	
chr28	11476538	2	38	A:1	G:0	
chr28	11476652	2	36	G:1	A:0	
chr28	11476824	2	34	G:1	A:0	
chr28	11477140	2	36	G:1	A:0	
chr28	11477164	2	36	G:0.722222		C:0.277778
chr28	11477263	2	38	C:0.868421		CATATCTT:0.131579
chr28	11477418	2	40	C:1	T:0	
chr28	11477480	2	38	T:1	G:0	
chr28	11477587	2	38	T:1	C:0	
chr28	11477592	2	38	C:1	G:0	
chr28	11477687	2	40	G:1	T:0	
chr28	11477721	2	38	C:1	T:0	
chr28	11477917	2	36	G:0.611111		C:0.388889
chr28	11477984	2	40	C:1	T:0	
chr28	11478176	2	40	C:0.95	G:0.05	
chr28	11478211	2	36	T:1	C:0	
chr28	11478307	2	38	T:1	C:0	
chr28	11478461	2	36	G:1	A:0	
chr28	11478477	2	36	T:1	C:0	
chr28	11478481	3	36	C:1	T:0	G:0
chr28	11478581	2	40	G:1	C:0	
chr28	11478646	2	38	T:1	C:0	
chr28	11478861	2	38	C:1	A:0	
chr28	11478872	2	38	A:1	G:0	
chr28	11478879	2	38	T:0.763158		C:0.236842
chr28	11479017	2	34	A:1	G:0	
chr28	11480928	2	36	G:0.888889		T:0.111111
chr28	11481038	2	40	A:1	G:0	
chr28	11481693	2	36	G:1	T:0	
chr28	11482073	2	38	G:1	A:0	
chr28	11482106	2	36	G:1	GA:0	
chr28	11482887	2	38	G:1	T:0	
chr28	11483039	2	38	C:1	G:0	
chr28	11483102	2	34	G:1	GT:0	
chr28	11483166	2	36	C:1	A:0	
chr28	11483215	2	36	A:0.944444		C:0.0555556
chr28	11483322	2	38	T:1	C:0	
chr28	11483461	2	36	G:1	A:0	
chr28	11483593	2	40	T:1	C:0	
chr28	11483649	2	36	T:1	C:0	
chr28	11483657	2	34	C:1	T:0	
chr28	11483764	2	32	T:1	C:0	
chr28	11483777	2	30	A:1	G:0	
chr28	11483812	2	40	A:1	AAAAGAAAAAG:0	
chr28	11483849	2	38	A:1	G:0	

chr28	11484111	2	36	C:1	T:0	
chr28	11484522	2	38	T:1	C:0	
chr28	11484626	2	38	G:1	C:0	
chr28	11484728	2	36	C:1	T:0	
chr28	11484740	2	36	C:1	T:0	
chr28	11484799	2	38	C:1	T:0	
chr28	11484800	2	38	G:1	A:0	
chr28	11484987	2	38	G:1	A:0	
chr28	11485184	2	38	T:1	C:0	
chr28	11485308	2	34	G:1	A:0	
chr28	11485412	2	34	G:1	A:0	
chr28	11485563	2	34	CT:0.941176		C:0.0588235
chr28	11485583	2	36	A:1	G:0	
chr28	11485671	3	38	A:1	C:0	T:0
chr28	11485684	2	38	C:1	T:0	
chr28	11485793	2	38	C:1	T:0	
chr28	11485949	2	38	G:1	T:0	
chr28	11485966	2	40	T:1	TGTTTTG:0	
chr28	11486019	2	36	T:1	C:0	
chr28	11486116	2	36	G:1	A:0	
chr28	11486158	2	32	G:1	A:0	
chr28	11486231	2	38	T:1	C:0	
chr28	11486251	2	40	C:1	G:0	
chr28	11486293	2	36	T:1	TG:0	
chr28	11486369	2	36	A:1	T:0	
chr28	11486603	2	40	T:1	C:0	
chr28	11486821	2	38	C:1	T:0	
chr28	11486830	2	38	A:1	G:0	
chr28	11486883	2	40	A:1	G:0	
chr28	11487163	2	34	G:0.911765		T:0.0882353
chr28	11487466	2	38	T:1	C:0	
chr28	11487880	2	38	T:1	C:0	
chr28	11488166	2	40	C:1	T:0	
chr28	11488286	2	36	TGC:1	T:0	
chr28	11488289	2	40	G:1	GCTCT:0	
chr28	11488291	2	40	G:1	T:0	
chr28	11488341	2	40	A:0.95	G:0.05	
chr28	11488740	2	36	G:1	A:0	
chr28	11488817	2	36	G:1	A:0	
chr28	11488832	2	36	C:1	G:0	
chr28	11489578	2	38	G:0.973684		A:0.0263158
chr28	11489756	2	34	T:0.823529		TA:0.176471
chr28	11489966	2	36	G:1	A:0	
chr28	11490486	2	34	G:1	A:0	
chr28	11490715	2	40	A:1	G:0	
chr28	11490799	2	36	T:1	A:0	
chr28	11490936	2	38	A:1	G:0	
chr28	11490937	2	38	T:1	C:0	
chr28	11490945	2	38	G:1	T:0	
chr28	11491365	2	34	C:1	G:0	
chr28	11491994	2	40	A:0.1	AACT:0.9	
chr28	11492273	2	38	A:1	G:0	
chr28	11492868	2	28	AG:0	A:1	
chr28	11493620	2	36	C:1	A:0	

chr28	11493665	2	28	CG:1	C:0	
chr28	11493738	2	36	G:1	A:0	
chr28	11494754	2	34	G:1	A:0	
chr28	11494775	2	36	AG:1	A:0	
chr28	11495053	2	34	T:1	C:0	
chr28	11495155	2	38	C:1	T:0	
chr28	11495639	2	38	G:1	A:0	
chr28	11495958	2	34	G:1	A:0	
chr28	11496440	2	38	C:1	T:0	
chr28	11496629	2	36	G:1	A:0	
chr28	11496751	2	34	G:1	GT:0	
chr28	11496843	2	38	G:0.473684	A:0.526316	
chr28	11496867	2	38	C:1	T:0	
chr28	11497063	2	36	T:1	C:0	
chr28	11497560	2	36	C:1	T:0	
chr28	11497797	2	38	T:1	A:0	
chr28	11497809	2	38	TA:1	T:0	
chr28	11498015	2	34	A:1	AT:0	
chr28	11499679	2	38	A:1	G:0	
chr28	11501918	2	34	T:1	C:0	
chr28	11502579	2	38	A:1	G:0	
chr28	11502629	2	36	T:1	C:0	
chr28	11502635	2	36	A:1	G:0	
chr28	11502653	2	36	C:1	T:0	
chr28	11502734	2	34	A:1	C:0	
chr28	11503113	2	38	A:1	G:0	
chr28	11503177	2	36	T:1	C:0	
chr28	11503288	2	38	A:1	G:0	
chr28	11503334	2	36	G:1	A:0	
chr28	11503634	2	38	T:1	C:0	
chr28	11504069	2	32	G:1	A:0	
chr28	11504071	2	32	T:1	C:0	
chr28	11504334	2	20	ACC:1	A:0	
chr28	11504721	2	38	CCATTTTT:1	C:0	
chr28	11505153	2	36	G:1	T:0	
chr28	11505207	2	38	G:1	A:0	
chr28	11505367	2	40	A:1	G:0	
chr28	11505524	2	40	C:1	T:0	
chr28	11505676	2	34	C:0.764706	T:0.235294	
chr28	11505773	2	36	G:0.333333	GA:0.666667	
chr28	11505867	2	40	G:1	A:0	
chr28	11506135	3	40	CCT:0.525	C:0	CCTCTCT:0.475
chr28	11506272	2	38	T:1	C:0	
chr28	11506414	2	34	A:0.352941	G:0.647059	
chr28	11506817	2	40	AC:0.325	A:0.675	
chr28	11507238	3	32	TTG:0.34375	T:0	TTGTG:0.65625
chr28	11507382	2	40	A:0.35	G:0.65	
chr28	11507492	2	38	A:0.315789	T:0.684211	
chr28	11507572	2	40	C:0.35	A:0.65	
chr28	11507680	2	36	G:0.361111	A:0.638889	
chr28	11507821	2	38	T:0.421053	C:0.578947	
chr28	11507946	2	38	G:0.342105	T:0.657895	

chr28	11508055	2	38	A:0.342105	G:0.657895		
chr28	11508165	2	40	C:1	T:0		
chr28	11509514	2	36	CT:1	C:0		
chr28	11509614	2	40	G:1	T:0		
chr28	11510272	2	38	T:0.421053	G:0.578947		
chr28	11511212	2	40	C:0.425	CGCCTTGATTATGATACTACACTTGATCTTAGCCAAAGGCCGAGAAGCGAT:0.575		
chr28	11511222	2	40	A:1	G:0		
chr28	11511233	2	40	A:0.95	G:0.05		
chr28	11511316	2	38	C:0.315789	T:0.684211		
chr28	11511841	2	38	T:0.447368	C:0.552632		
chr28	11511913	2	38	C:0.263158	T:0.736842		
chr28	11512011	2	36	TTG:0	T:1		
chr28	11512407	2	36	A:0.666667	T:0.333333		
chr28	11513140	2	30	CT:0.6	C:0.4		
chr28	11513530	2	38	A:0.736842	G:0.263158		
chr28	11513642	2	40	C:1	T:0		
chr28	11514371	2	40	T:0.2	C:0.8		
chr28	11514674	2	40	C:0	CA:1		
chr28	11514693	2	40	C:0	T:1		
chr28	11514765	2	40	A:1	T:0		
chr28	11514797	2	38	G:0.0789474	GATT:0.921053		
chr28	11514978	2	40	C:0.1	G:0.9		
chr28	11515167	2	38	GCTGT:0.105263	G:0.894737		
chr28	11515383	2	34	T:0.0294118	C:0.970588		
chr28	11515793	2	36	C:0.194444	G:0.805556		
chr28	11516163	2	34	CAAGAT:0.852941	C:0.147059		
chr28	11516390	2	36	C:0.777778	CCCA:0.222222		
chr28	11516429	2	38	C:0.789474	T:0.210526		
chr28	11516463	2	36	C:0.777778	T:0.222222		
chr28	11516757	2	36	G:0.222222	A:0.777778		
chr28	11516761	2	36	A:0.0277778	G:0.972222		
chr28	11516798	2	36	C:0.472222	T:0.527778		
chr28	11517070	2	36	G:0.861111	C:0.138889		
chr28	11517763	2	38	A:0.815789	T:0.184211		
chr28	11517929	2	38	C:0.815789	T:0.184211		
chr28	11518074	2	40	C:0.975	T:0.025		
chr28	11518205	2	30	G:0.866667	A:0.133333		
chr28	11518482	2	36	G:0.972222	A:0.0277778		
chr28	11518915	2	36	C:0.861111	T:0.138889		
chr28	11519820	2	36	G:0.194444	A:0.805556		
chr28	11520070	2	36	A:0.0277778	C:0.972222		
chr28	11520739	2	38	A:0.105263	ATTTAT:0.894737		
chr28	11521589	2	36	TCGC:0.194444	T:0.805556		
chr28	11521714	2	36	C:0.277778	A:0.722222		
chr28	11522169	2	38	C:0.210526	T:0.789474		
chr28	11522337	2	32	CT:0.9375	C:0.0625		
chr28	11522354	4	34	T:0.323529	C:0.147059	TC:0.294118	TTC:0.235294
chr28	11522935	2	40	TA:0.275	T:0.725		
chr28	11523560	2	30	G:0.2	GC:0.8		
chr28	11524592	2	32	C:0.96875	T:0.03125		
chr28	11525728	2	34	A:1	G:0		
chr28	11526058	2	34	G:1	A:0		

chr28	11527611	2	38	T:0.263158	C:0.736842	
chr28	11528478	2	38	G:0.815789	A:0.184211	
chr28	11530233	2	38	C:0	CG:1	
chr28	11530244	2	38	A:0	C:1	
chr28	11530269	2	40	C:0	CA:1	
chr28	11530274	2	40	AT:0	A:1	
chr28	11530369	2	36	G:0.972222	A:0.0277778	
chr28	11530818	2	34	C:1	G:0	
chr28	11530874	2	36	C:1	T:0	
chr28	11531543	2	36	G:0.388889	T:0.611111	
chr28	11532105	2	32	T:1	TGAGA:0	
chr28	11532111	2	32	A:0.90625	G:0.09375	
chr28	11532192	2	34	G:1	A:0	
chr28	11532549	2	30	G:1	A:0	
chr28	11532847	2	36	T:1	TACCAC:0	
chr28	11533505	2	40	T:0.25	A:0.75	
chr28	11533827	2	38	A:0.815789	G:0.184211	
chr28	11533856	2	38	T:1	A:0	
chr28	11533911	2	38	T:0.421053	C:0.578947	
chr28	11533989	2	38	C:0.394737	CAG:0.605263	
chr28	11534005	2	38	C:1	T:0	
chr28	11534124	2	38	T:1	C:0	
chr28	11534183	2	38	C:1	CT:0	
chr28	11534650	2	40	G:0.625	A:0.375	
chr28	11534799	2	36	C:1	T:0	
chr28	11535501	2	34	C:0.735294	T:0.264706	
chr28	11535645	2	38	G:0.789474	GA:0.210526	
chr28	11535897	2	38	C:0.815789	T:0.184211	
chr28	11535924	2	36	C:1	T:0	
chr28	11535926	2	36	C:0.75	G:0.25	
chr28	11536344	2	34	C:0.794118	A:0.205882	
chr28	11536414	2	36	G:0.861111	A:0.138889	
chr28	11536599	2	34	CT:0.529412	C:0.470588	
chr28	11536711	2	34	G:0.882353	GA:0.117647	
chr28	11537054	3	36	GTCTC:0.527778	G:0.444444	GTCTCTCTCTCTC:0.0277778
chr28	11537056	2	36	C:0.916667	CTCTCTCTG:0.0833333	
chr28	11537152	2	40	T:0.875	C:0.125	
chr28	11537281	2	34	G:0.852941	A:0.147059	
chr28	11537416	2	40	G:0.975	A:0.025	
chr28	11537696	2	32	C:0.9375	T:0.0625	
chr28	11538009	2	40	A:0.825	C:0.175	
chr28	11538294	2	38	T:1	TC:0	
chr28	11538675	2	36	T:0.805556	C:0.194444	
chr28	11538753	2	36	A:0.805556	G:0.194444	
chr28	11539134	2	36	GC:0.638889	G:0.361111	
chr28	11539147	2	36	T:0.638889	C:0.361111	
chr28	11539150	2	36	T:0.0277778	C:0.972222	
chr28	11539227	2	36	G:0.972222	T:0.0277778	
chr28	11539262	2	40	C:1	T:0	
chr28	11540390	2	34	C:0.970588	T:0.0294118	
chr28	11540567	2	36	C:0.861111	T:0.138889	
chr28	11540668	2	34	C:0.794118	T:0.205882	

chr28	11540718	2	36	G:0.5	C:0.5
chr28	11540928	2	36	G:0.444444	A:0.555556
chr28	11541108	2	34	A:0.5	C:0.5
chr28	11541429	2	36	G:0.972222	A:0.0277778
chr28	11541457	2	38	G:0.789474	T:0.210526
chr28	11541535	2	38	G:0.973684	A:0.0263158
chr28	11541554	2	38	C:1	A:0
chr28	11542693	2	38	C:1	T:0
chr28	11543092	2	36	G:0.25	GCC:0.75
chr28	11543557	2	30	A:0.533333	C:0.466667
chr28	11543704	2	38	A:0.421053	G:0.578947
chr28	11543708	2	38	A:1	C:0
chr28	11543713	2	38	A:0.421053	C:0.578947
chr28	11544014	2	34	G:1	A:0
chr28	11544060	2	38	A:0.973684	G:0.0263158
chr28	11544073	2	36	C:0.972222	T:0.0277778
chr28	11544252	2	36	C:0.888889	T:0.111111
chr28	11544280	2	38	A:0.605263	G:0.394737
chr28	11544391	2	36	C:0.861111	T:0.138889
chr28	11544484	2	34	C:0.5	T:0.5
chr28	11544499	2	34	G:0.970588	GCT:0.0294118
chr28	11544581	2	36	G:0.972222	A:0.0277778
chr28	11544714	2	36	T:0	TG:1
chr28	11544717	2	34	A:0.882353	G:0.117647
chr28	11544799	2	34	G:0.529412	T:0.470588
chr28	11544827	2	36	T:0.805556	C:0.194444
chr28	11544877	2	32	GTGT:0.8125	G:0.1875
chr28	11544883	2	28	GT:0.928571	G:0.0714286
chr28	11544890	2	32	G:0.8125	GC:0.1875
chr28	11544914	2	32	T:0.75	C:0.25
chr28	11544988	2	38	C:0.5	T:0.5
chr28	11545100	2	36	G:0.805556	GC:0.194444
chr28	11545302	2	32	C:0.96875	T:0.03125
chr28	11545375	2	40	A:0.85	G:0.15
chr28	11545391	2	38	A:0.842105	C:0.157895
chr28	11545565	2	34	G:0.529412	T:0.470588
chr28	11545679	2	36	T:1	C:0
chr28	11545691	2	34	T:0.794118	TG:0.205882
chr28	11545745	2	38	C:0.868421	CCTGCTT:0.131579
chr28	11545856	2	34	G:0.382353	C:0.617647
chr28	11545895	2	34	A:0.411765	G:0.588235
chr28	11545936	2	36	G:0.527778	T:0.472222
chr28	11545944	2	36	T:0.527778	C:0.472222
chr28	11546339	2	38	C:0.394737	G:0.605263
chr28	11546711	2	40	T:0	C:1
chr28	11547246	2	34	C:0.529412	T:0.470588
chr28	11547749	2	36	T:0.5	C:0.5
chr28	11547825	2	38	G:0.394737	A:0.605263
chr28	11547909	2	36	A:0.527778	G:0.472222
chr28	11547965	2	34	G:0.558824	A:0.441176
chr28	11548201	2	34	GGC:0.5	G:0.5
chr28	11548684	2	36	A:0.5	C:0.5
chr28	11548728	2	32	G:0.46875	A:0.53125
chr28	11548790	2	38	T:0.973684	C:0.0263158

chr28	11548905	2	40	T:0.45	C:0.55	
chr28	11549158	2	34	C:0.705882	T:0.294118	
chr28	11549225	2	38	G:0.947368	C:0.0526316	
chr28	11549422	2	36	G:0.777778	A:0.222222	
chr28	11549854	2	36	GC:0.5	G:0.5	
chr28	11549886	2	36	A:0.388889	C:0.611111	
chr28	11550451	2	34	G:0.588235	T:0.411765	
chr28	11550453	2	34	C:0.588235	T:0.411765	
chr28	11550474	2	34	C:0.617647	T:0.382353	
chr28	11550527	2	36	T:0.472222	C:0.527778	
chr28	11550532	2	36	A:0.527778	G:0.472222	
chr28	11550589	2	34	G:0.5	GA:0.5	
chr28	11551403	2	40	T:0.725	C:0.275	
chr28	11551428	2	38	G:0.973684	A:0.0263158	
chr28	11551678	2	36	C:0.416667	A:0.583333	
chr28	11551766	2	36	A:0.972222	G:0.0277778	
chr28	11552315	2	38	C:1	T:0	
chr28	11552628	2	38	G:0.973684	C:0.0263158	
chr28	11552797	2	32	A:0.5	G:0.5	
chr28	11552898	2	36	G:0.944444	A:0.0555556	
chr28	11552956	2	36	C:0.833333	T:0.166667	
chr28	11553028	2	38	C:0.973684	T:0.0263158	
chr28	11553058	2	36	C:0.972222	A:0.0277778	
chr28	11553744	2	10	G:0.5	A:0.5	
chr28	11553875	2	30	A:0	C:1	
chr28	11554042	2	32	G:0.0625	A:0.9375	
chr28	11554074	2	34	C:1	T:0	
chr28	11554165	2	30	G:1	T:0	
chr28	11554424	2	36	T:0.944444	C:0.0555556	
chr28	11554540	2	36	G:0.972222	A:0.0277778	
chr28	11554663	3	40	AC:0	A:0.975	ACC:0.025
chr28	11555304	2	38	A:1	C:0	
chr28	11555340	2	36	C:0.972222	A:0.0277778	
chr28	11555434	2	38	A:0.0263158	G:0.973684	
chr28	11555462	2	38	A:0.973684	AC:0.0263158	
chr28	11555463	2	38	G:0.0263158	C:0.973684	
chr28	11555641	2	36	C:0.972222	T:0.0277778	
chr28	11556141	2	30	G:1	A:0	
chr28	11556147	2	32	C:0.46875	T:0.53125	
chr28	11556157	2	30	C:0.966667	T:0.0333333	
chr28	11556412	2	36	C:0.0555556	G:0.944444	
chr28	11556493	2	40	A:0.075	G:0.925	
chr28	11556601	2	32	G:0.53125	A:0.46875	
chr28	11556642	2	34	A:0.0294118	G:0.970588	
chr28	11556693	3	38	TAC:0.473684	T:0	TACACAC: 0.526316
chr28	11556796	2	38	A:0.105263	G:0.894737	
chr28	11557075	2	38	A:0.0526316	G:0.947368	
chr28	11557107	2	34	C:0.0294118	T:0.970588	
chr28	11557257	2	36	TGG:0.0277778	T:0.972222	
chr28	11557409	2	38	TCC:0.5	T:0.5	
chr28	11557492	2	38	C:0.973684	T:0.0263158	
chr28	11558629	2	34	G:1	A:0	
chr28	11558708	3	38	TAA:0.0526316	T:0.0789474	

TA:0.868421					
chr28	11558881	2	32	T:1	C:0
chr28	11558998	2	36	C:0.888889	A:0.111111
chr28	11559044	2	38	T:0.552632	C:0.447368
chr28	11559049	2	38	A:1	G:0
chr28	11559113	2	38	C:0.552632	T:0.447368
chr28	11559336	2	36	G:0.472222	A:0.527778
chr28	11559370	2	38	G:0.973684	A:0.0263158
chr28	11559660	2	36	G:0.5	A:0.5
chr28	11559680	2	36	C:0.972222	A:0.0277778
chr28	11559789	2	38	G:0.552632	A:0.447368
chr28	11560380	2	40	G:1	GAA:0
chr28	11560678	2	30	T:0.8	C:0.2
chr28	11560738	2	36	C:0.777778	T:0.222222
chr28	11560775	2	34	G:0.705882	A:0.294118
chr28	11560960	2	40	ACAGGGTGACTTG:0.4	A:0.6
chr28	11561034	2	40	T:0.45	C:0.55
chr28	11561179	2	36	A:1	G:0
chr28	11561313	2	40	A:1	
ATTTGTGTTGTGTTGTGTTGTATGTCTTTATATGG:0					
chr28	11561358	2	38	T:0.447368	C:0.552632
chr28	11561433	2	40	G:1	A:0
chr28	11561807	2	40	C:0.8	T:0.2
chr28	11562183	2	40	C:1	T:0
chr28	11562308	2	34	A:0.470588	T:0.529412
chr28	11562402	2	34	G:0.823529	A:0.176471
chr28	11562430	2	34	C:1	T:0
chr28	11562453	2	36	G:0.583333	A:0.416667
chr28	11562652	2	38	C:1	T:0
chr28	11562724	2	40	T:0.375	C:0.625
chr28	11562746	2	40	A:0.375	G:0.625
chr28	11562877	2	38	A:0.605263	C:0.394737
chr28	11562957	2	40	G:0.425	A:0.575
chr28	11563024	2	38	G:0.447368	T:0.552632
chr28	11563301	2	36	T:0.444444	C:0.555556
chr28	11563332	2	38	G:0.447368	A:0.552632
chr28	11563436	2	34	T:0.0294118	C:0.970588
chr28	11563631	2	34	C:0.441176	T:0.558824
chr28	11564298	2	32	C:0.5625	T:0.4375
chr28	11564438	2	34	G:0.0294118	GTC:0.970588
chr28	11564743	2	40	T:0.425	C:0.575
chr28	11564765	2	38	C:0.447368	A:0.552632
chr28	11564817	2	34	T:0	C:1
chr28	11564942	2	36	CTCTCT:0.472222	C:0.527778
chr28	11564975	2	38	TA:0.5	T:0.5
chr28	11564976	2	38	A:1	T:0
chr28	11564977	2	36	A:1	T:0
chr28	11564997	2	38	T:0.5	C:0.5
chr28	11565006	2	40	G:0.575	GA:0.425
chr28	11565038	2	40	G:0.475	A:0.525
chr28	11565040	2	40	TGTTA:1	T:0
chr28	11565107	2	38	A:0	C:1
chr28	11565280	2	40	C:0.475	A:0.525
chr28	11565509	2	38	G:0.973684	A:0.0263158

chr28	11565518	2	38	C:0.605263	T:0.394737
chr28	11565525	2	38	C:1	T:0
chr28	11565938	2	32	A:0.03125	T:0.96875
chr28	11566480	2	38	C:0.0263158	A:0.973684
chr28	11566746	2	36	A:1	G:0
chr28	11567133	2	34	A:0.0294118	G:0.970588
chr28	11567398	2	38	C:0.0263158	G:0.973684
chr28	11567410	2	38	T:0.0263158	A:0.973684
chr28	11567464	2	40	G:0.025	C:0.975
chr28	11567663	2	38	A:1	G:0
chr28	11567932	2	40	A:0.025	G:0.975
chr28	11568038	2	40	T:0.025	C:0.975
chr28	11568052	2	38	T:0.0263158	C:0.973684
chr28	11568086	2	38	T:0	C:1
chr28	11568578	2	38	T:0	C:1
chr28	11568998	2	36	C:0.694444	A:0.305556
chr28	11569485	2	34	A:1	AC:0
chr28	11569547	2	36	G:0.0555556	A:0.944444
chr28	11569937	2	36	G:1	A:0
chr28	11570207	2	40	G:0.025	GA:0.975
chr28	11570420	2	38	T:0.0263158	C:0.973684
chr28	11570482	2	36	TC:1	T:0
chr28	11571261	2	32	A:0.59375	T:0.40625
chr28	11571394	2	32	G:0.75	C:0.25
chr28	11571395	2	32	G:0.75	T:0.25
chr28	11571515	2	34	C:0	CT:1
chr28	11571579	2	38	C:0	A:1
chr28	11571582	2	38	T:0	A:1
chr28	11571583	2	38	G:0	A:1
chr28	11571586	2	38	G:0	A:1
chr28	11571687	2	30	C:0.666667	T:0.333333
chr28	11571773	2	26	CCA:0.846154	C:0.153846
chr28	11571812	2	28	T:0.892857	G:0.107143
chr28	11572221	2	6	A:0	G:1
chr28	11572368	2	28	G:0.428571	T:0.571429
chr28	11572559	2	36	T:0.333333	C:0.666667
chr28	11572602	2	26	A:0.576923	T:0.423077
chr28	11572697	2	24	G:0.375	A:0.625
chr28	11572722	2	24	G:0.666667	A:0.333333
chr28	11572829	2	16	A:0.625	T:0.375
chr28	11573250	2	26	T:0.461538	C:0.538462
chr28	11573328	2	32	G:0.78125	A:0.21875
chr28	11573350	2	30	A:0	T:1
chr28	11573351	2	30	A:0	T:1
chr28	11573352	2	30	A:0	T:1
chr28	11573392	2	36	A:0	AT:1
chr28	11573397	2	36	A:0	AT:1
chr28	11573680	2	38	T:0.5	C:0.5
chr28	11573861	2	40	A:0.525	G:0.475
chr28	11573910	2	38	A:0.526316	G:0.473684
chr28	11573959	2	36	A:1	C:0
chr28	11574101	2	40	TTTTCTTTCTTTC:0.525	T:0.475
chr28	11574186	4	40	CTTCCTTTCCTTTCCTTTCCTTTCCTTTCCT:	
0.1	C:0.6	CTTCCTTTCCT:0.2	CTTCCTTTCCTTTCCTTTCCT:0.1		

chr28	11574601	2	38	C:0.0789474	CAAAT:0.921053
chr28	11574693	2	36	T:0.583333	G:0.416667
chr28	11574809	2	38	C:0.578947	CT:0.421053
chr28	11574818	2	38	A:0.605263	T:0.394737
chr28	11575729	2	34	AG:0.588235	A:0.411765
chr28	11576021	2	38	G:1	A:0
chr28	11576230	2	38	A:0.105263	T:0.894737
chr28	11576419	2	38	T:0.105263	TG:0.894737
chr28	11576643	2	40	CAAACAA:0.95	C:0.05
chr28	11576716	2	36	GTA:0.111111	G:0.888889
chr28	11576738	2	38	T:1	A:0
chr28	11576853	2	36	G:0	C:1
chr28	11576869	2	36	C:1	T:0
chr28	11577188	2	28	CCT:0.25	C:0.75
chr28	11577216	2	34	A:0.941176	AT:0.0588235
chr28	11577346	2	38	G:0.131579	A:0.868421
chr28	11577432	2	40	G:1	A:0
chr28	11577541	2	40	C:1	T:0
chr28	11577594	2	38	A:1	G:0
chr28	11577789	2	38	C:0.0263158	A:0.973684
chr28	11577927	2	38	T:0.0263158	TAA:0.973684
chr28	11577973	2	40	T:0.025	C:0.975
chr28	11578112	2	40	C:0.05	T:0.95
chr28	11578215	2	38	A:0.0526316	C:0.947368
chr28	11578269	2	2	C:1	A:0
chr28	11578484	2	32	G:1	A:0
chr28	11578533	2	34	C:0.941176	T:0.0588235
chr28	11578564	2	34	C:0.0294118	A:0.970588
chr28	11578718	2	36	C:0.0555556	T:0.944444
chr28	11578778	2	30	TAC:0.1	T:0.9
chr28	11578794	2	32	G:0.03125	A:0.96875
chr28	11578798	2	28	A:1	ATG:0
chr28	11578865	2	36	A:0.0833333	T:0.916667
chr28	11578932	2	36	A:0.0555556	G:0.944444
chr28	11578956	2	36	C:0.694444	A:0.305556
chr28	11579440	2	40	G:0.175	A:0.825
chr28	11579558	2	40	T:0.025	C:0.975
chr28	11580025	2	34	G:1	A:0
chr28	11580346	2	38	C:0.0526316	T:0.947368
chr28	11580885	2	38	G:0.0263158	T:0.973684
chr28	11581125	2	34	G:1	A:0
chr28	11581162	2	34	G:1	A:0
chr28	11581232	2	40	G:0.975	A:0.025
chr28	11581388	2	34	T:0.0294118	G:0.970588
chr28	11581861	2	40	G:0.025	A:0.975
chr28	11581981	2	40	T:0.975	C:0.025
chr28	11582049	2	38	T:1	A:0
chr28	11582340	2	32	G:1	A:0
chr28	11582811	2	32	C:0.96875	T:0.03125
chr28	11582964	2	36	A:1	C:0
chr28	11583458	2	32	AC:0.9375	A:0.0625
chr28	11583596	2	36	C:0.0555556	T:0.944444
chr28	11583717	2	36	C:0.0277778	A:0.972222
chr28	11583875	2	38	G:0.973684	A:0.0263158

chr28	11583892	2	40	G:0.025	A:0.975	
chr28	11584035	2	36	T:1	C:0	
chr28	11584058	2	36	T:1	G:0	
chr28	11584794	2	40	CT:0.95	C:0.05	
chr28	11585232	2	36	A:0.972222	G:0.0277778	
chr28	11585430	2	30	A:0.0666667	G:0.933333	
chr28	11585454	2	32	G:1	C:0	
chr28	11585532	2	40	A:1	C:0	
chr28	11585712	2	36	C:0.0555556	T:0.944444	
chr28	11585736	2	38	G:1	A:0	
chr28	11587009	2	36	C:0.972222	T:0.0277778	
chr28	11587232	2	32	TGA:0.03125	T:0.96875	
chr28	11587312	2	34	G:0	A:1	
chr28	11587332	2	32	G:0.75	A:0.25	
chr28	11587641	2	34	G:1	A:0	
chr28	11587651	2	34	A:0.0588235	G:0.941176	
chr28	11588148	2	38	A:0.0526316	G:0.947368	
chr28	11588471	3	34	CT:0.588235	C:0.176471	CTT:0.235294
chr28	11588601	2	34	T:0.0294118	C:0.970588	
chr28	11588763	2	36	C:1	T:0	
chr28	11589217	2	40	C:0.05	T:0.95	
chr28	11589258	2	38	T:0.973684	C:0.0263158	
chr28	11589596	2	34	G:1	A:0	
chr28	11589598	2	34	C:0.0294118	T:0.970588	
chr28	11590069	2	32	T:1	C:0	
chr28	11590104	2	32	C:1	T:0	
chr28	11590299	2	32	C:0.0625	A:0.9375	
chr28	11590481	2	38	A:1	G:0	
chr28	11590817	2	36	G:0.0277778	A:0.972222	
chr28	11590925	2	34	T:0.0294118	C:0.970588	
chr28	11591042	2	36	T:1	C:0	
chr28	11591043	2	36	G:1	T:0	
chr28	11591057	2	36	A:1	T:0	
chr28	11591069	2	38	T:1	C:0	
chr28	11591173	2	36	G:1	A:0	
chr28	11591421	2	36	T:1	TG:0	
chr28	11591717	2	38	C:1	T:0	
chr28	11591970	2	34	G:0.0588235	T:0.941176	
chr28	11592040	2	34	T:0.588235	G:0.411765	
chr28	11592531	2	36	A:1	G:0	
chr28	11592748	2	32	T:1	G:0	
chr28	11592995	2	28	G:1	C:0	
chr28	11593095	2	14	A:0.214286	G:0.785714	
chr28	11593209	2	8	G:0.25	A:0.75	
chr28	11593286	2	20	GTGCGGC:0.3	G:0.7	
chr28	11593315	2	24	CGGGCCA:1	C:0	
chr28	11593329	2	26	GGCCAGGGCCAGC:0.5	G:0.5	
chr28	11593513	2	36	C:0.0277778	CG:0.972222	
chr28	11593558	2	38	C:0.631579	T:0.368421	
chr28	11594256	2	34	C:1	CCCCT:0	
chr28	11594264	2	32	T:0.0625	TC:0.9375	
chr28	11594265	3	34	T:0.0588235	TC:0.411765	TCTC:0.529412

chr28 11594286 2 36 AT:0.888889 A:0.111111
chr28 11594291 2 36 T:0.444444 A:0.555556
chr28 11594296 2 36 T:0.888889 A:0.111111
chr28 11594297 4 36 TA:0.194444 T:0.333333
AA:0.277778 TAA:0.194444
chr28 11594331 2 38 C:1 T:0
chr28 11594374 2 38 G:0.736842 A:0.263158
chr28 11594483 2 36 T:0.388889 G:0.611111
chr28 11594670 2 40 G:0.825 T:0.175
chr28 11594671 2 40 T:0.825 C:0.175
chr28 11594796 2 38 A:0.184211 G:0.815789
chr28 11594872 2 40 C:0.725 T:0.275
chr28 11594893 2 40 C:0.125 CAGAA:0.875
chr28 11595014 2 36 A:0.194444 G:0.805556
chr28 11595259 2 32 A:0.21875 T:0.78125
chr28 11595360 2 30 C:0.633333 T:0.366667
chr28 11595626 2 38 C:0.315789 T:0.684211
chr28 11595862 2 38 G:0.605263 T:0.394737
chr28 11595870 2 38 C:0.605263 T:0.394737
chr28 11595883 2 36 T:0.611111 C:0.388889
chr28 11596094 2 38 T:0.578947 C:0.421053
chr28 11596296 2 38 G:1 A:0
chr28 11596429 2 40 T:0.025 C:0.975
chr28 11596526 2 36 A:0.583333 G:0.416667
chr28 11596568 2 38 T:0.552632 A:0.447368
chr28 11596570 2 38 C:1 T:0
chr28 11596721 2 38 G:0.473684 C:0.526316
chr28 11596765 3 38 GTT:0.526316 G:0.131579
GT:0.342105
chr28 11596776 2 38 TTTTTTG:0.868421 T:0.131579
chr28 11596779 2 36 TTTG:1 T:0
chr28 11596782 2 36 G:0.555556 T:0.444444
chr28 11596785 2 36 T:0.555556 G:0.444444
chr28 11596787 2 36 T:0.638889 G:0.361111
chr28 11596788 2 36 TG:0.638889 T:0.361111
chr28 11596789 3 38 GTT:0.447368 G:0.131579
TTT:0.421053
chr28 11596794 2 36 T:1 TG:0
chr28 11596927 2 36 T:0.638889 A:0.361111
chr28 11597021 3 34 C:0.0882353 CT:0.617647
CTT:0.294118
chr28 11597352 2 36 C:0.666667 T:0.333333
chr28 11597367 2 36 G:0.472222 T:0.527778
chr28 11597372 2 36 G:0.666667 A:0.333333
chr28 11597823 2 34 C:0.558824 T:0.441176
chr28 11598185 4 38 CT:0.289474 C:0.105263
CTT:0.157895 CTTT:0.447368
chr28 11598207 2 38 A:0.552632 T:0.447368
chr28 11598277 2 34 G:0.823529 A:0.176471
chr28 11598278 2 34 A:0.823529 T:0.176471
chr28 11598332 2 32 G:0.78125 GGAT:0.21875
chr28 11598335 2 32 C:0.78125 T:0.21875
chr28 11598396 2 32 C:0.6875 CT:0.3125
chr28 11598404 2 34 C:0.0588235 T:0.941176

chr28 11598484 2 36 G:0.833333 T:0.166667
chr28 11598559 2 38 A:1 G:0
chr28 11598737 2 38 C:0.842105 CA:0.157895
chr28 11598800 4 38 CTT:0.105263 C:0.131579
CT:0.394737 CTTT:0.368421
chr28 11598860 2 38 C:0.0526316 T:0.947368
chr28 11599091 2 36 T:0.666667 G:0.333333
chr28 11599188 2 38 C:1 A:0
chr28 11599223 2 38 A:0.657895 C:0.342105
chr28 11599278 2 38 A:0.763158 C:0.236842
chr28 11599456 2 38 T:0.0263158 C:0.973684
chr28 11599457 2 38 G:0.789474 A:0.210526
chr28 11599776 2 40 T:0.9 G:0.1
chr28 11599802 2 38 T:0.578947 A:0.421053
chr28 11599821 2 38 T:0.894737 A:0.105263
chr28 11599924 2 40 G:0.2 A:0.8
chr28 11600197 2 34 GA:0.176471 G:0.823529
chr28 11600932 2 38 T:0.394737 A:0.605263
chr28 11601773 2 38 A:0.973684 C:0.0263158
chr28 11601810 2 40 G:0.45 A:0.55
chr28 11601818 2 40 C:1 T:0
chr28 11601898 2 36 C:0.777778 G:0.222222
chr28 11602097 2 40 C:0.2 A:0.8
chr28 11602103 2 40 T:0.975 G:0.025
chr28 11602416 2 36 T:0.777778 C:0.222222
chr28 11602515 2 38 T:0.710526 C:0.289474
chr28 11602545 2 40 A:0.025 C:0.975
chr28 11602891 2 36 C:0.694444 T:0.305556
chr28 11603063 2 40 T:0.925 G:0.075
chr28 11604252 2 36 T:0.416667 C:0.583333
chr28 11604357 2 36 A:1 T:0
chr28 11604378 4 38 TGAGAGAGAGA:0.421053 T:
0.184211 TGAGA:0.368421 TGAGAGAGA:0.0263158
chr28 11604394 2 34 AG:0.676471 A:0.323529
chr28 11604396 2 34 AGAGAGAG:0.676471 A:
0.323529
chr28 11604458 2 34 C:0.5 G:0.5
chr28 11604478 2 34 TG:0.823529 T:0.176471
chr28 11604541 2 36 A:0.555556 T:0.444444
chr28 11604741 2 38 C:0.973684 A:0.0263158
chr28 11604757 2 40 ATGAAGAAATGGAGACTGTTT:0.7 A:0.3
chr28 11604949 2 40 A:0.425 G:0.575
chr28 11604955 2 40 A:1 G:0
chr28 11605172 2 40 T:0.625 G:0.375
chr28 11605576 2 36 C:0.694444 T:0.305556
chr28 11605909 2 36 C:0.888889 T:0.111111
chr28 11606073 2 36 A:0.722222 G:0.277778
chr28 11606519 2 34 A:0.794118 G:0.205882
chr28 11606879 2 38 CA:0.815789 C:0.184211
chr28 11606945 2 36 T:0.666667 A:0.333333
chr28 11607011 2 32 A:0.75 G:0.25
chr28 11607897 2 38 C:0.236842 T:0.763158
chr28 11608033 2 38 G:0.552632 A:0.447368
chr28 11608180 3 38 TAAACAAAC:0.210526 T:

0.368421	TAAAC:0.421053				
chr28	11608898	2	38	A:0.0789474	G:0.921053
chr28	11608945	2	40	G:0.15	C:0.85
chr28	11609061	2	36	T:0.5	C:0.5
chr28	11609073	2	36	A:0.0833333	T:0.916667
chr28	11609146	2	38	T:0.131579	C:0.868421
chr28	11609265	2	38	T:0.105263	C:0.894737
chr28	11609484	2	32	C:0.8125	T:0.1875
chr28	11609564	2	38	G:0.473684	A:0.526316
chr28	11609578	2	36	C:0.833333	T:0.166667
chr28	11609722	2	36	T:0.638889	G:0.361111
chr28	11609723	2	36	A:0.166667	C:0.833333
chr28	11609803	2	34	A:1	T:0
chr28	11609990	2	36	C:0.277778	A:0.722222
chr28	11610409	2	30	G:0.433333	A:0.566667
chr28	11610793	2	36	TCAG:0.722222	T:0.277778
chr28	11611072	2	38	T:0.447368	C:0.552632
chr28	11611102	2	32	T:0.46875	C:0.53125
chr28	11611344	2	40	T:1	C:0
chr28	11611618	2	34	C:0.470588	G:0.529412
chr28	11611687	2	36	C:0.5	T:0.5
chr28	11611791	2	38	G:1	A:0
chr28	11611845	2	36	A:0.444444	T:0.555556
chr28	11611994	2	38	G:0.947368	A:0.0526316
chr28	11612122	2	38	T:0.5	C:0.5
chr28	11612128	2	38	A:0.973684	C:0.0263158
chr28	11612184	2	36	G:0.527778	A:0.472222
chr28	11612734	2	36	C:1	T:0
chr28	11612749	2	36	G:1	C:0
chr28	11613175	2	36	G:0.972222	A:0.0277778
chr28	11613346	2	34	C:0.911765	T:0.0882353
chr28	11613350	2	34	G:0.911765	T:0.0882353
chr28	11613641	2	40	C:1	A:0
chr28	11613768	2	32	C:1	T:0
chr28	11613900	2	38	G:1	A:0
chr28	11614047	2	38	T:1	C:0
chr28	11614242	2	38	G:0.131579	A:0.868421
chr28	11614331	2	38	C:0.578947	T:0.421053
chr28	11614549	2	38	C:0.973684	T:0.0263158
chr28	11614894	2	38	A:0.552632	G:0.447368
chr28	11615171	2	38	G:0.473684	T:0.526316
chr28	11615352	2	38	T:0.421053	TA:0.578947
chr28	11615568	2	34	C:0.323529	A:0.676471
chr28	11615826	2	38	G:0.684211	A:0.315789
chr28	11615868	2	34	CTA:0.470588	C:0.529412
chr28	11615952	2	40	ATTTTT:0.9	A:0.1
chr28	11615953	2	34		
TTTTTTTTTTTTTTTTTTTTTTTTGGTATTTGAA:0.617647					T:0.382353
chr28	11616070	2	38	C:1	CT:0
chr28	11616117	2	38	CT:0.868421	C:0.131579
chr28	11616268	2	36	C:0.472222	A:0.527778
chr28	11616320	2	34	G:0.5	T:0.5
chr28	11616406	2	38	GTA:1	G:0
chr28	11616536	2	36	CA:0.388889	C:0.611111

chr28	11616567	2	34	TC:0.676471	T:0.323529
chr28	11616568	2	34	C:0.705882	CT:0.294118
chr28	11616613	2	32	A:0.78125	C:0.21875
chr28	11616697	2	32	G:0.03125	A:0.96875
chr28	11616963	2	36	G:0.777778	C:0.222222
chr28	11617017	2	36	T:0.333333	C:0.666667
chr28	11617076	2	36	T:0.444444	G:0.555556
chr28	11617079	2	36	C:0.666667	T:0.333333
chr28	11617455	2	38	G:0.5	A:0.5
chr28	11617492	2	36	T:0.138889	C:0.861111
chr28	11617795	2	38	A:1	G:0
chr28	11618216	2	34	AT:0.970588	A:0.0294118
chr28	11618267	2	40	T:0.25	C:0.75
chr28	11618315	2	38	G:0.5	A:0.5
chr28	11618439	2	34	T:0.794118	C:0.205882
chr28	11618928	2	34	GT:0.764706	G:0.235294
chr28	11618960	2	30	C:1	A:0
chr28	11619029	2	34	T:0.176471	TG:0.823529
chr28	11619034	2	34	C:0.441176	T:0.558824
chr28	11619208	2	30	G:1	C:0
chr28	11619407	2	40	A:0.975	T:0.025
chr28	11619418	2	40	A:0.5	G:0.5
chr28	11619623	2	40	G:0.825	T:0.175
chr28	11619624	2	40	A:0.2	T:0.8
chr28	11619667	2	38	C:1	T:0
chr28	11619802	2	40	T:0.475	A:0.525
chr28	11619807	2	40	G:0.475	T:0.525
chr28	11620066	2	36	G:0.722222	A:0.277778
chr28	11620197	2	38	G:1	A:0
chr28	11620293	2	40	TG:0.2	T:0.8
chr28	11620360	2	40	C:1	T:0
chr28	11620596	2	38	G:0.447368	A:0.552632
chr28	11620674	2	40	G:0.975	A:0.025
chr28	11620751	2	36	A:0.444444	G:0.555556
chr28	11620765	2	36	C:0.694444	T:0.305556
chr28	11620843	2	34	A:0.970588	G:0.0294118
chr28	11620896	2	38	A:0.605263	C:0.394737
chr28	11621240	2	36	A:0.666667	T:0.333333
chr28	11621573	2	40	G:0.475	A:0.525
chr28	11621624	2	34	G:0.794118	A:0.205882
chr28	11621742	2	38	A:0.763158	G:0.236842
chr28	11621773	2	40	T:0.65	C:0.35
chr28	11621848	2	38	G:0.473684	A:0.526316
chr28	11622244	2	34	T:0.705882	A:0.294118
chr28	11623106	2	34	A:0.411765	AATTT:0.588235
chr28	11623164	2	32	C:0.59375	T:0.40625
chr28	11623166	2	32	C:0.59375	T:0.40625
chr28	11623243	2	36	T:0.694444	G:0.305556
chr28	11623314	2	36	G:0.611111	A:0.388889
chr28	11623361	2	36	C:0.583333	T:0.416667
chr28	11623373	2	36	T:0.111111	C:0.888889
chr28	11623385	2	36	G:0.805556	T:0.194444
chr28	11623406	2	34	C:0.647059	T:0.352941
chr28	11623440	2	36	T:1	C:0

chr28	11623894	2	36	C:1	T:0
chr28	11624156	2	36	C:0.805556	T:0.194444
chr28	11624224	2	38	T:0.763158	C:0.236842
chr28	11624878	2	38	A:0.789474	T:0.210526
chr28	11625075	2	40	C:0.275	T:0.725
chr28	11625161	2	32	C:0.21875	T:0.78125
chr28	11625279	2	40	T:0.5	C:0.5
chr28	11625400	2	38	GTCAC:0.973684	G:0.0263158
chr28	11625617	2	36	G:0.138889	A:0.861111
chr28	11625771	2	40	G:0.175	C:0.825
chr28	11625905	3	38	TC:0.236842	T:0.578947
CC:0.184211
chr28	11625915	2	38	T:0.421053	C:0.578947
chr28	11625962	2	38	G:0.157895	GTATT:0.842105
chr28	11626066	3	34	CTGTGTG:0.588235	C:0.352941
CTGTG:0.0588235
chr28	11626244	4	38	CAG:0.526316	C:0.289474
CAGAG:0.0263158	CAGAGAG:0.157895
chr28	11626315	2	38	G:0.973684	A:0.0263158
chr28	11626402	2	32	A:0.3125	G:0.6875
chr28	11626637	2	36	C:0.416667	T:0.583333
chr28	11627103	2	38	C:1	T:0
chr28	11627334	2	36	A:0.805556	G:0.194444
chr28	11627622	2	32	TTC:0.84375	T:0.15625
chr28	11627984	2	40	T:0.725	G:0.275
chr28	11628016	2	36	A:0.75	T:0.25
chr28	11628478	2	38	A:0.131579	G:0.868421
chr28	11628656	2	36	G:1	A:0
chr28	11628658	2	36	G:1	A:0
chr28	11628696	2	40	CTTTATCATATTCTTTATTCTCT:0.75
C:0.25
chr28	11628917	2	34	T:1	A:0
chr28	11629114	2	36	CTTTTTT:0.861111	C:0.138889
chr28	11629359	2	36	A:0.5	G:0.5
chr28	11629750	2	34	T:0.705882	C:0.294118
chr28	11629885	2	34	G:0.764706	A:0.235294
chr28	11629911	2	36	T:0.75	C:0.25
chr28	11629947	2	36	C:1	T:0
chr28	11630066	2	36	T:0.666667	G:0.333333
chr28	11630218	2	38	T:0.736842	C:0.263158
chr28	11630386	2	40	A:0.425	C:0.575
chr28	11630569	2	40	A:0.775	G:0.225
chr28	11630669	2	38	G:0.394737	A:0.605263
chr28	11630972	2	36	C:0.944444	T:0.0555556
chr28	11631002	2	38	C:1	G:0
chr28	11631112	2	40	G:0.65	A:0.35
chr28	11631274	2	40	C:0.5	T:0.5
chr28	11631806	2	36	AT:0.611111	A:0.388889
chr28	11631807	2	36	T:0.666667	A:0.333333
chr28	11631961	2	36	AC:0.527778	A:0.472222
chr28	11631997	2	36	G:0.638889	T:0.361111
chr28	11632048	2	34	G:0.5	A:0.5
chr28	11632389	2	38	A:0.157895	G:0.842105
chr28	11632448	2	36	C:0.638889	G:0.361111

chr28 11633018 2 38 CA:0.552632 C:0.447368
chr28 11633136 2 38 T:0.236842 TC:0.763158
chr28 11633395 2 38 C:1 T:0
chr28 11633438 2 36 T:0.638889 C:0.361111
chr28 11633629 2 36 G:0.833333 A:0.166667
chr28 11633883 2 38 G:0.684211 A:0.315789
chr28 11634053 2 36 T:0.166667 C:0.833333
chr28 11634287 2 36 C:0.972222 T:0.0277778
chr28 11634408 2 38 TGTAA:0.842105 T:0.157895
chr28 11634473 2 40 C:0.225 A:0.775
chr28 11634938 2 40 C:1 CTTA:0
chr28 11634941 2 40 C:1 G:0
chr28 11634943 2 40 A:1 AGAACCTCT:0
chr28 11634945 2 40 A:1 ATTGAAGAACC:0
chr28 11635125 2 34 GC:0.647059 G:0.352941
chr28 11635128 2 34 T:0.647059 G:0.352941
chr28 11635701 2 34 A:0.176471 G:0.823529
chr28 11636000 2 26 G:1 A:0
chr28 11636112 2 34 T:0.470588 C:0.529412
chr28 11636162 3 36 G:0.916667 GAATA:0.0277778
GAATAAATA:0.0555556
chr28 11636195 2 40 A:1 AATACATAAATACATAAATAC:0
chr28 11636202 2 38 A:0.973684 AC:0.0263158
chr28 11636203 6 40 A:0.35 AAAAAAAAT:0.025
AATAAAAAT:0.25 AATAAATAAAAAT:0.35
AATACATAAATAAATAAATAAAAAT:0
ATAAATACATAAATACATAAATAAATAAATAAAAAT:0.025
chr28 11636449 2 40 GCAA:0.75 G:0.25
chr28 11636450 3 40 CA:0.65 C:0.1 CAA:0.25
chr28 11636585 2 32 T:0.6875 C:0.3125
chr28 11636666 2 34 A:0.617647 C:0.382353
chr28 11636699 2 36 C:0.833333 T:0.166667
chr28 11637097 2 30 C:0.766667 T:0.233333
chr28 11637148 2 28 TTC:0.5 T:0.5
chr28 11637876 2 36 A:0.75 C:0.25
chr28 11638360 2 40 T:0.725 C:0.275
chr28 11638539 2 38 T:1 C:0
chr28 11638683 2 40 C:0.225 T:0.775
chr28 11638838 2 40 T:0.75 G:0.25
chr28 11638897 2 40 A:1 AGC:0
chr28 11638902 3 40 A:0.625 ACG:0.375 G:0
chr28 11638910 2 40 G:0.675 GCGCACA:0.325
chr28 11638912 2 36 A:0.888889 G:0.111111
chr28 11639095 2 36 C:1 T:0
chr28 11639874 2 34 C:0.794118 A:0.205882
chr28 11640588 6 40 CATCTATCTATCTATCTATCT:0.25
C:0.35 CATCTATCTATCTATCT:0.125 CATCTATCTATCTATCTATCTATCT:0.275
CATCTATCTATCTATCTATCTATCTATCT:0 CATCTATCTATCTATCTATCTATCTATCTATCT:
0
chr28 11640754 2 38 A:0 C:1
chr28 11640794 2 38 T:0.0526316 TTATC:0.947368
chr28 11640807 2 38 G:1 A:0
chr28 11640936 2 40 A:0.975 T:0.025
chr28 11641278 2 36 A:0.694444 AT:0.305556

chr28	11641403	2	40	A:0.775	C:0.225
chr28	11641509	2	38	G:0.710526	A:0.289474
chr28	11641581	2	38	A:0.789474	G:0.210526
chr28	11641643	2	40	C:0.675	T:0.325
chr28	11641993	2	36	C:0.75	T:0.25
chr28	11642098	2	36	AG:0.666667	A:0.333333
chr28	11642201	2	36	G:0.75	A:0.25
chr28	11642416	2	34	G:0.794118	C:0.205882
chr28	11642575	2	36	G:0.694444	A:0.305556
chr28	11642692	2	34	A:0.617647	G:0.382353
chr28	11642904	2	36	T:0.777778	C:0.222222
chr28	11643368	2	40	CCT:0.975	C:0.025
chr28	11643389	2	40	C:0.65	G:0.35
chr28	11643473	2	36	C:1	T:0
chr28	11643490	2	36	C:0.638889	T:0.361111
chr28	11643556	2	40	A:0.675	G:0.325
chr28	11643956	2	38	G:0.684211	A:0.315789
chr28	11643994	2	40	C:0.7	T:0.3
chr28	11644107	2	38	A:1	G:0
chr28	11644288	2	36	T:0.722222	C:0.277778
chr28	11644717	2	38	A:0.684211	G:0.315789
chr28	11644808	2	40	A:1	G:0
chr28	11645247	2	40	A:0.65	G:0.35
chr28	11646411	2	36	T:0.722222	C:0.277778
chr28	11646414	2	36	C:0.694444	T:0.305556
chr28	11646644	2	38	C:0.842105	T:0.157895
chr28	11647305	2	36	GT:0.611111	G:0.388889
chr28	11648316	2	36	TG:0.777778	T:0.222222
chr28	11648719	2	38	C:0.368421	G:0.631579
chr28	11648877	2	36	G:1	A:0
chr28	11648879	2	36	G:0.472222	GCA:0.527778
chr28	11649448	2	36	C:0.805556	T:0.194444
chr28	11649850	2	36	T:0.972222	C:0.0277778
chr28	11650074	2	38	T:0.473684	C:0.526316
chr28	11650446	2	36	A:1	T:0
chr28	11650763	2	38	T:0.763158	C:0.236842
chr28	11651129	2	30	C:0.666667	CT:0.333333
chr28	11651162	2	34	G:0.705882	A:0.294118
chr28	11651188	2	36	C:0.694444	T:0.305556
chr28	11651220	2	34	G:0.735294	A:0.264706
chr28	11651262	2	36	G:0.722222	C:0.277778
chr28	11651378	2	36	A:0.694444	G:0.305556
chr28	11651404	2	36	C:0.722222	T:0.277778
chr28	11651493	2	34	A:0.617647	T:0.382353
chr28	11651708	2	36	C:0.833333	T:0.166667
chr28	11651773	2	36	G:0.694444	A:0.305556
chr28	11651917	2	38	T:0.657895	A:0.342105
chr28	11651918	2	38	T:0.657895	C:0.342105
chr28	11651981	2	38	C:0.736842	CTT:0.263158
chr28	11652232	2	24	C:0.708333	CCTT:0.291667
chr28	11652278	2	30	C:0.733333	T:0.266667
chr28	11652330	2	22	T:0.681818	C:0.318182
chr28	11652763	2	26	C:0.961538	CTA:0.0384615
chr28	11653163	2	34	GAGA:0.794118	G:0.205882

chr28	11653191	2	34	C:1	T:0	
chr28	11653359	2	36	A:0.75	G:0.25	
chr28	11653409	2	30	C:0.7	A:0.3	
chr28	11653426	2	30	G:0.866667	A:0.133333	
chr28	11653579	2	32	A:0.96875	T:0.03125	
chr28	11653592	2	32	T:0.96875	A:0.03125	
chr28	11654206	2	36	C:0.694444	T:0.305556	
chr28	11654215	2	34	A:0.5	G:0.5	
chr28	11654296	2	36	C:0.5	T:0.5	
chr28	11654401	2	38	T:0.736842	G:0.263158	
chr28	11654527	2	28	C:0.714286	T:0.285714	
chr28	11654533	2	28	T:0.428571	C:0.571429	
chr28	11654573	3	34	CCT:0.5	C:0.294118	CCTCTCT: 0.205882
chr28	11654622	2	32	C:0.75	T:0.25	
chr28	11654717	2	36	G:0.611111	C:0.388889	
chr28	11654744	2	38	T:0.684211	C:0.315789	
chr28	11654788	2	38	C:0.710526	T:0.289474	
chr28	11654806	2	38	G:0.710526	GGA:0.289474	
chr28	11654835	2	38	A:0.657895	G:0.342105	
chr28	11654906	2	40	C:0.75	T:0.25	
chr28	11655174	2	38	C:0.763158	T:0.236842	
chr28	11655291	2	36	C:0.722222	T:0.277778	
chr28	11655316	2	36	G:0.722222	A:0.277778	
chr28	11655319	2	36	G:0.972222	A:0.0277778	
chr28	11655324	2	36	C:0.75	T:0.25	
chr28	11655454	2	38	T:0.710526	TCTTA:0.289474	
chr28	11655790	2	38	T:0.684211	C:0.315789	
chr28	11655791	2	38	G:0.684211	A:0.315789	
chr28	11655925	2	34	A:1	G:0	
chr28	11655948	2	30	G:0.766667	A:0.233333	
chr28	11656006	2	36	C:1	T:0	
chr28	11656314	2	38	A:0.710526	G:0.289474	
chr28	11656333	2	38	A:0.710526	C:0.289474	
chr28	11656345	2	38	A:0.710526	C:0.289474	
chr28	11656370	2	36	A:1	G:0	
chr28	11656439	2	38	C:0.605263	T:0.394737	
chr28	11656683	2	38	C:0.657895	G:0.342105	
chr28	11656689	2	40	C:0.675	G:0.325	
chr28	11656782	2	38	G:0.657895	A:0.342105	
chr28	11656805	2	40	AG:0.7	A:0.3	
chr28	11656806	2	40	G:0.725	A:0.275	
chr28	11656895	2	34	A:0.676471	T:0.323529	
chr28	11657017	2	36	A:1	G:0	
chr28	11657049	2	38	A:0.710526	C:0.289474	
chr28	11657074	2	38	A:1	T:0	
chr28	11657122	2	38	T:1	C:0	
chr28	11657324	2	40	A:0.7	G:0.3	
chr28	11657342	2	40	A:0.7	C:0.3	
chr28	11657425	2	36	A:0.694444	G:0.305556	
chr28	11657472	2	32	CT:0.78125	C:0.21875	
chr28	11657604	2	36	A:0.694444	G:0.305556	
chr28	11657746	2	36	T:0.638889	C:0.361111	
chr28	11657954	5	36	GTTATTATTATTA:0	G:0.555556	

GTTA:0.111111	GTTATTA:0.194444	GTTATTATTA:0.138889				
chr28	11657963	2	36	A:0.944444	G:0.0555556	
chr28	11657969	2	34	A:1	G:0	
chr28	11658263	2	36	A:1	G:0	
chr28	11658441	2	40	C:0.45	A:0.55	
chr28	11658526	2	36	A:1	T:0	
chr28	11658790	2	36	C:1	A:0	
chr28	11658823	2	34	A:0.147059	G:0.852941	
chr28	11659626	2	36	C:0.777778	CT:0.222222	
chr28	11659758	2	36	T:0.777778	C:0.222222	
chr28	11661093	3	40	A:0.175	C:0	T:0.825
chr28	11661214	2	36	T:0.138889	TA:0.861111	
chr28	11661386	2	38	G:1	A:0	
chr28	11661490	2	36	C:1	T:0	
chr28	11661717	2	36	C:0.194444	T:0.805556	
chr28	11661758	2	36	A:0.25	G:0.75	
chr28	11661816	2	34	T:1	A:0	
chr28	11661817	2	34	G:1	T:0	
chr28	11661984	2	38	T:0.184211	C:0.815789	
chr28	11662084	2	34	T:0.794118	C:0.205882	
chr28	11662169	2	34	C:1	T:0	
chr28	11662237	2	36	T:1	TTAGGTAGAATA:0	
chr28	11662282	2	38	G:0.815789	C:0.184211	
chr28	11662462	2	40	AAC:0.65	A:0.35	
chr28	11662480	2	40	ACTGTGAGCTACACTGAC:0.65	A:0.35	
chr28	11662796	2	40	A:0.225	AC:0.775	
chr28	11662804	2	40	C:0.8	T:0.2	
chr28	11662825	2	40	G:0.225	A:0.775	
chr28	11662862	2	40	T:0.175	A:0.825	
chr28	11662884	2	40	A:0.15	T:0.85	
chr28	11663181	2	38	A:1	ACT:0	
chr28	11663292	2	38	A:0.184211	T:0.815789	
chr28	11663403	2	36	T:0.194444	C:0.805556	
chr28	11663419	2	34	T:0.735294	C:0.264706	
chr28	11663575	2	40	C:0.2	T:0.8	
chr28	11663586	2	40	A:0.2	AT:0.8	
chr28	11663630	2	40	C:0.25	T:0.75	
chr28	11663675	2	38	T:0.763158	C:0.236842	
chr28	11663739	2	36	G:0.25	T:0.75	
chr28	11663741	2	36	G:1	C:0	
chr28	11663792	2	40	A:0.225	C:0.775	
chr28	11663894	2	40	GA:0.225	G:0.775	
chr28	11663948	2	40	C:0.25	T:0.75	
chr28	11664110	2	40	A:1	T:0	
chr28	11664191	2	40	T:0.675	C:0.325	
chr28	11664251	2	40	CATA:0.15	C:0.85	
chr28	11664254	2	40	A:1	T:0	
chr28	11664256	2	40	T:1	C:0	
chr28	11664326	2	40	C:0.2	T:0.8	
chr28	11664351	2	40	T:0.2	C:0.8	
chr28	11664406	2	38	G:0.184211	C:0.815789	
chr28	11664486	2	40	A:1	T:0	
chr28	11664713	2	36	G:1	A:0	
chr28	11664724	2	36	G:0.222222	A:0.777778	

chr28	11664901	2	38	C:1	A:0
chr28	11664945	2	38	C:0.447368	A:0.552632
chr28	11664965	2	38	T:1	A:0
chr28	11665294	2	36	A:0.166667	G:0.833333
chr28	11665320	2	34	C:0.705882	T:0.294118
chr28	11665484	2	38	G:0.236842	GA:0.763158
chr28	11665594	2	38	G:0.105263	A:0.894737
chr28	11665678	2	36	T:0.222222	C:0.777778
chr28	11665705	2	36	C:0.722222	T:0.277778
chr28	11665706	2	36	A:0.194444	G:0.805556
chr28	11665989	2	38	C:1	T:0
chr28	11666004	2	38	G:0.789474	C:0.210526
chr28	11666012	2	38	T:1	C:0
chr28	11666106	2	40	G:0.775	A:0.225
chr28	11666130	2	40	T:0.225	C:0.775
chr28	11666159	2	38	G:0.184211	T:0.815789
chr28	11666161	2	38	C:0.184211	T:0.815789
chr28	11666193	2	34	C:0.970588	T:0.0294118
chr28	11666285	2	34	C:0.205882	T:0.794118
chr28	11666395	2	32	G:0.15625	T:0.84375
chr28	11666414	2	30	T:0.166667	C:0.833333
chr28	11666482	2	30	G:0.166667	A:0.833333
chr28	11666629	2	40	G:0.3	T:0.7
chr28	11666647	2	40	C:1	G:0
chr28	11666701	2	38	C:0.210526	T:0.789474
chr28	11666755	2	38	G:0.236842	A:0.763158
chr28	11666762	2	38	C:0.236842	T:0.763158
chr28	11666835	2	38	CATT:0.763158	C:0.236842
chr28	11666869	2	40	A:0.7	T:0.3
chr28	11666939	2	40	A:0.225	T:0.775
chr28	11667041	2	38	C:0.157895	A:0.842105
chr28	11667295	2	40	TAC:0.95	T:0.05
chr28	11667321	2	38	CACACAA:0.684211	C:0.315789
chr28	11667323	2	38	CACAA:0.947368	C:0.0526316
chr28	11667325	2	38	CAA:0.631579	C:0.368421
chr28	11667469	2	40	C:0.7	T:0.3
chr28	11667474	2	40	G:0.275	A:0.725
chr28	11667485	2	40	C:0.5	G:0.5
chr28	11667540	2	40	G:0.225	A:0.775
chr28	11667708	2	34	T:0.823529	TAC:0.176471
chr28	11667740	2	38	A:0.236842	G:0.763158
chr28	11667796	2	36	T:0.222222	C:0.777778
chr28	11667797	2	36	T:0.222222	A:0.777778
chr28	11667809	2	38	G:1	A:0
chr28	11667887	2	36	A:0.75	G:0.25
chr28	11668132	2	38	T:0.526316	C:0.473684
chr28	11668503	2	38	C:1	T:0
chr28	11668526	2	40	GT:1	G:0
chr28	11668732	2	36	GA:1	G:0
chr28	11668754	2	38	C:1	T:0
chr28	11668838	2	36	A:1	G:0
chr28	11668925	2	38	GCCTTTAA:0.947368	G: 0.0526316
chr28	11668967	2	32	T:1	C:0

chr28 11669252 2 36 T:1 C:0
chr28 11669308 2 40 C:1 A:0
chr28 11669781 2 34 G:1 C:0
chr28 11670042 2 40 CAT:1 C:0
chr28 11670043 3 38 A:0.368421 ATG:0.5 ATGTG:
0.131579
chr28 11670061 2 40 G:1 A:0
chr28 11670064 2 40 C:0.925 G:0.075
chr28 11670314 2 34 AC:0.617647 A:0.382353
chr28 11670454 2 36 G:0.972222 A:0.0277778
chr28 11670603 2 36 A:0.222222 T:0.777778
chr28 11671073 2 38 C:0.947368 T:0.0526316
chr28 11671184 2 36 G:0.25 A:0.75
chr28 11671202 2 38 C:0.210526 T:0.789474
chr28 11671346 2 38 T:0.210526 C:0.789474
chr28 11671361 2 38 C:0.210526 T:0.789474
chr28 11671460 2 38 T:0.789474 A:0.210526
chr28 11671813 2 32 C:0.1875 T:0.8125
chr28 11671926 2 40 G:0.85 A:0.15
chr28 11671947 2 40 C:0.225 G:0.775
chr28 11672114 2 40 ACTTTCC:0.8 A:0.2
chr28 11672145 2 40 A:0.8 G:0.2
chr28 11672149 2 40 T:0.225 A:0.775
chr28 11672178 2 40 A:0.75 G:0.25
chr28 11672321 2 38 C:0.789474 G:0.210526
chr28 11672355 2 36 T:0.694444 G:0.305556
chr28 11672417 2 34 TG:0.205882 T:0.794118
chr28 11672566 6 40 TTTTATTTATTTATTTATTTA:0.225
T:0.3 TTTTA:0.2 TTTTATTTA:0.225 TTTTATTTATTTA:0
TTTTATTTATTTATTTA:0.05
chr28 11672615 2 40 TTTA:0.8 T:0.2
chr28 11672855 2 40 GTAAT:0.85 G:0.15
chr28 11672983 2 36 GATTA:0.75 G:0.25
chr28 11674067 2 36 C:0.166667 A:0.833333
chr28 11674195 3 36 TA:0.194444 T:0.583333
TAA:0.222222
chr28 11674415 2 36 T:0.833333 A:0.166667
chr28 11674480 2 40 C:0.825 A:0.175
chr28 11675099 4 32 TAA:0.34375 T:0.21875
TA:0.15625 TAAA:0.28125
chr28 11675118 2 32 GA:0.5625 G:0.4375
chr28 11675396 2 36 C:0.25 CA:0.75
chr28 11675414 2 34 G:0.794118 A:0.205882
chr28 11675566 2 36 G:0.777778 A:0.222222
chr28 11675926 2 36 TA:0.222222 T:0.777778
chr28 11676070 2 36 A:0.972222 AT:0.0277778
chr28 11676090 2 36 A:0.638889 T:0.361111
chr28 11676203 2 36 T:0.222222 TA:0.777778
chr28 11676290 2 38 G:0.736842 A:0.263158
chr28 11676352 2 38 C:0.210526 T:0.789474
chr28 11676692 2 40 TAAA:0.15 T:0.85
chr28 11676696 2 40 A:0.775 T:0.225
chr28 11676739 2 40 T:0.275 C:0.725
chr28 11676836 2 40 C:0.7 G:0.3

chr28	11676889	2	40	C:0.65	T:0.35	
chr28	11677022	2	40	T:0.25	TAGTC:0.75	
chr28	11677158	2	38	A:0.421053	AAG:0.578947	
chr28	11677159	2	38	A:0.736842	AG:0.263158	
chr28	11677164	2	38	T:0.736842	A:0.263158	
chr28	11677170	2	38	A:0.736842	G:0.263158	
chr28	11677360	2	32	C:0.40625	CA:0.59375	
chr28	11677631	3	40	TGAGA:0.5	T:0.175	TGAGAGA:0.325
chr28	11677662	2	38	C:0.736842	T:0.263158	
chr28	11677675	2	38	C:0.210526	A:0.789474	
chr28	11677838	2	38	C:0.184211	G:0.815789	
chr28	11678159	3	38	C:0.526316	CT:0.236842	CCTT:0.236842
chr28	11678183	2	34	T:0.147059	C:0.852941	
chr28	11678370	2	34	A:0.794118	G:0.205882	
chr28	11678849	2	36	T:0.805556	C:0.194444	
chr28	11678978	2	40	A:0.625	AT:0.375	
chr28	11679100	2	40	T:0.975	C:0.025	
chr28	11679252	2	38	C:0.789474	A:0.210526	
chr28	11679465	2	32	C:0.375	T:0.625	
chr28	11679592	2	34	A:0.911765	C:0.0882353	
chr28	11679828	2	26	TC:0.461538	T:0.538462	
chr28	11680019	2	30	C:0.766667	T:0.233333	
chr28	11680206	2	34	C:0.294118	G:0.705882	
chr28	11680249	2	34	C:0.176471	T:0.823529	
chr28	11680371	2	40	G:0.6	A:0.4	
chr28	11680456	2	34	T:0.529412	C:0.470588	
chr28	11680552	2	36	C:0	CA:1	
chr28	11681024	2	40	G:0.525	A:0.475	
chr28	11681265	2	38	T:0.605263	A:0.394737	
chr28	11681314	2	38	C:0.710526	T:0.289474	
chr28	11681488	2	40	CAGAAACTCGGATGG:1	C:0	
chr28	11681515	2	40	G:0.975	A:0.025	
chr28	11681635	2	40	T:0.625	G:0.375	
chr28	11681638	2	40	A:0.8	ACAG:0.2	
chr28	11681822	2	36	T:0.222222	G:0.777778	
chr28	11681960	2	36	C:0.555556	T:0.444444	
chr28	11681964	2	34	C:1	T:0	
chr28	11682021	2	38	G:0.236842	A:0.763158	
chr28	11682113	2	38	T:0.868421	C:0.131579	
chr28	11682143	2	36	A:0.5	G:0.5	
chr28	11682214	2	34	T:0.735294	C:0.264706	
chr28	11682221	2	36	G:0.777778	A:0.222222	
chr28	11682522	2	38	T:0.710526	C:0.289474	
chr28	11682826	2	34	C:0.470588	A:0.529412	
chr28	11682863	2	34	C:1	T:0	
chr28	11682929	2	40	T:0.625	C:0.375	
chr28	11682955	2	40	C:0.675	T:0.325	
chr28	11683463	2	38	A:1	C:0	
chr28	11683516	2	28	TA:0.75	T:0.25	
chr28	11683703	2	36	T:0.944444	TA:0.0555556	
chr28	11683704	3	40	T:0.325	A:0.125	TA:0.55
chr28	11683871	2	36	A:0.527778	T:0.472222	

chr28	11683882	2	36	T:0.527778	C:0.472222		
chr28	11683904	2	36	T:1	C:0		
chr28	11683953	2	40	G:0.525	A:0.475		
chr28	11684268	2	38	T:0.394737	A:0.605263		
chr28	11684270	2	38	C:0.763158	CT:0.236842		
chr28	11684488	2	38	T:0.421053	C:0.578947		
chr28	11684636	2	36	T:0.472222	C:0.527778		
chr28	11684830	2	40	A:0.7	T:0.3		
chr28	11684927	2	34	C:0.529412	G:0.470588		
chr28	11685424	2	38	T:1	C:0		
chr28	11685456	2	36	A:0.583333	G:0.416667		
chr28	11685471	2	38	T:1	C:0		
chr28	11685838	2	40	C:0.775	T:0.225		
chr28	11686012	2	36	C:0.583333	A:0.416667		
chr28	11686046	2	34	C:0.558824	T:0.441176		
chr28	11686094	2	36	G:0.75	T:0.25		
chr28	11686482	2	40	C:0.5	A:0.5		
chr28	11686583	2	40	G:0.475	C:0.525		
chr28	11686733	2	38	A:0.973684	T:0.0263158		
chr28	11687334	2	36	A:0.222222	G:0.777778		
chr28	11687417	2	40	C:0.85	T:0.15		
chr28	11688034	2	40	A:0.775	C:0.225		
chr28	11688227	2	38	T:0.763158	C:0.236842		
chr28	11688665	2	40	A:0.8	T:0.2		
chr28	11688672	2	40	T:0.8	TCTC:0.2		
chr28	11688771	2	36	G:0.555556	A:0.444444		
chr28	11688958	2	34	T:0.852941	TA:0.147059		
chr28	11689308	2	36	T:0.944444	A:0.0555556		
chr28	11689682	2	36	G:0.861111	A:0.138889		
chr28	11690414	2	38	C:0.815789	T:0.184211		
chr28	11690489	2	34	T:1	G:0		
chr28	11690556	2	40	A:0.9	ACAAAGTAGTGTCATC:0.1		
chr28	11690879	2	20	TCC:1	T:0		
chr28	11690883	2	20	CCCCCA:0.45	C:0.55		
chr28	11690885	2	22	CCCA:0.636364	C:0.363636		
chr28	11690888	2	20	ACCC:1	A:0		
chr28	11690889	2	22	C:0.636364	G:0.363636		
chr28	11690977	2	36	T:0.611111	C:0.388889		
chr28	11691100	2	38	T:0.763158	C:0.236842		
chr28	11691179	2	36	T:1	C:0		
chr28	11691215	2	38	C:0.815789	G:0.184211		
chr28	11691342	4	22	TAAA:0.727273	T:0.0909091	TA:0.0909091	TAAAA:0.0909091
chr28	11691381	3	24	C:0.125	CT:0.666667	CTT:0.208333	
chr28	11691535	2	10	AAT:0	A:1		
chr28	11691644	2	20	A:0.1	G:0.9		
chr28	11691670	2	30	T:0.9	C:0.1		
chr28	11691825	2	26	A:0.807692	C:0.192308		
chr28	11691854	2	34	G:0.882353	C:0.117647		
chr28	11691905	2	34	C:0.941176	T:0.0588235		
chr28	11691985	2	38	T:1	TA:0		
chr28	11691988	2	38	T:1	TA:0		
chr28	11691989	2	38	T:0.947368	TA:0.0526316		

chr28	11691991	2	38	T:1	TATTTATTTA:0		
chr28	11691992	2	38	T:0.947368	TATTTATTTA:0.0526316		
chr28	11691996	3	38	T:0.947368	C:0	TC:0.0526316	
chr28	11692094	2	38	T:0.973684	C:0.0263158		
chr28	11692109	2	38	G:0.973684	A:0.0263158		
chr28	11692111	2	38	T:0.973684	G:0.0263158		
chr28	11692146	2	32	T:0.75	C:0.25		
chr28	11692697	4	40	TAAA:0.5	T:0.1	TA:0.225	TAA:0.175
chr28	11692903	2	40	C:0.975	T:0.025		
chr28	11692913	2	36	TGA:0.0277778	T:0.972222		
chr28	11692931	2	40	A:0.925	T:0.075		
chr28	11692946	2	40	AAGAG:0.975	A:0.025		
chr28	11692990	2	36	G:0.972222	T:0.0277778		
chr28	11693149	2	38	C:1	T:0		
chr28	11693163	2	38	G:0.947368	A:0.0526316		
chr28	11693239	2	38	C:0.0263158	T:0.973684		
chr28	11693250	2	36	G:0.805556	A:0.194444		
chr28	11693427	2	36	T:0.944444	TAAATTCTAGCTAGAAAAAAA:0.0555556		
chr28	11693736	2	40	A:0.775	G:0.225		
chr28	11693880	2	38	A:0.947368	G:0.0526316		
chr28	11693898	2	40	C:0.825	T:0.175		
chr28	11694331	2	40	C:0.975	CAGGG:0.025		
chr28	11694333	2	40	T:0.975	TCC:0.025		
chr28	11694335	2	40	T:0.975	TGGGTGGCGCAGCGGTTTGGCGCC:0.025		
chr28	11694776	2	38	A:0.973684	G:0.0263158		
chr28	11694790	2	38	C:0.763158	T:0.236842		
chr28	11695051	2	38	G:0.973684	A:0.0263158		
chr28	11695127	2	36	C:0.861111	T:0.138889		
chr28	11695129	2	36	C:0.861111	T:0.138889		
chr28	11695454	3	36	A:0.75	AT:0.194444	ATT:0.0555556	
chr28	11695464	2	36	A:0.0555556	T:0.944444		
chr28	11695499	2	32	TGAGA:0.28125	T:0.71875		
chr28	11695895	2	38	G:0.736842	T:0.263158		
chr28	11695928	2	36	A:0.805556	G:0.194444		
chr28	11695971	2	36	C:0.805556	CA:0.194444		
chr28	11696043	2	40	T:0.775	C:0.225		
chr28	11696157	2	38	A:0.789474	T:0.210526		
chr28	11696322	2	40	T:0.775	C:0.225		
chr28	11696333	2	40	T:0.75	C:0.25		
chr28	11696491	2	38	T:0.815789	G:0.184211		
chr28	11696563	2	38	T:0.526316	C:0.473684		
chr28	11696569	2	38	A:0.763158	C:0.236842		
chr28	11696667	2	36	C:1	T:0		
chr28	11696696	2	36	G:0.75	A:0.25		
chr28	11696738	2	38	T:0.789474	C:0.210526		
chr28	11696739	2	38	G:1	A:0		
chr28	11696799	2	36	G:1	T:0		
chr28	11696804	2	36	A:0.0277778	G:0.972222		

chr28	11696821	2	38	TC:0.763158	T:0.236842		
chr28	11696879	4	36	TAA:0.5	T:0.0555556	TA:0.25	TAAA:0.194444
chr28	11696890	2	36	A:1	G:0		
chr28	11696987	2	38	C:0.842105	T:0.157895		
chr28	11697054	2	36	T:0.75	C:0.25		
chr28	11697164	2	38	T:0.868421	C:0.131579		
chr28	11697601	2	38	T:0.815789	C:0.184211		
chr28	11697656	2	40	G:0.8	A:0.2		
chr28	11697697	2	36	C:0.888889	T:0.111111		
chr28	11698030	2	40	C:0.825	T:0.175		
chr28	11698157	2	36	A:0.777778	G:0.222222		
chr28	11698169	2	36	C:0.777778	T:0.222222		
chr28	11698275	2	38	T:0.210526	C:0.789474		
chr28	11698653	2	38	TC:0.210526	T:0.789474		
chr28	11698840	2	36	T:0.805556	C:0.194444		
chr28	11698847	2	36	G:0.972222	T:0.0277778		
chr28	11699712	2	36	A:0.75	G:0.25		
chr28	11699795	2	36	C:1	T:0		
chr28	11700007	2	38	TTGAC:0.842105	T:0.157895		
chr28	11700072	2	38	GC:0.210526	G:0.789474		
chr28	11700076	2	38	A:0.210526	ACTGT:0.789474		
chr28	11700159	2	40	T:0.275	C:0.725		
chr28	11700274	2	38	T:0.0263158	C:0.973684		
chr28	11700395	2	36	A:0.75	G:0.25		
chr28	11700691	2	38	A:0.815789	C:0.184211		
chr28	11700988	2	34	A:0.235294	AAAG:0.764706		
chr28	11701131	2	36	C:0.277778	T:0.722222		
chr28	11701418	2	38	A:0.342105	G:0.657895		
chr28	11701430	2	36	CATG:1	C:0		
chr28	11701535	2	34	A:0.205882	C:0.794118		
chr28	11701569	2	40	T:0.175	G:0.825		
chr28	11701671	2	38	A:0.894737	G:0.105263		
chr28	11701845	2	38	C:0.210526	A:0.789474		
chr28	11702069	2	38	A:0.315789	G:0.684211		
chr28	11702330	2	38	T:0.394737	C:0.605263		
chr28	11702460	2	38	G:0.368421	A:0.631579		
chr28	11702480	2	38	A:0.368421	G:0.631579		
chr28	11702530	2	34	T:0.735294	C:0.264706		
chr28	11702555	2	34	AT:0.735294	A:0.264706		
chr28	11702576	4	34	CAG:0.529412	C:0.0882353	CAGAG:0.235294	CAGAGAGAG:0.147059
chr28	11702775	2	38	A:0.973684	G:0.0263158		
chr28	11703062	2	36	C:0.527778	A:0.472222		
chr28	11703929	2	38	G:0.789474	T:0.210526		
chr28	11703942	2	38	A:1	C:0		
chr28	11703970	3	36	A:0.861111	ATG:0.138889	ATGTG:0	
chr28	11703996	4	38	G:0.605263	A:0.131579	GTGTA:0.0526316	GTGTGTGTA:0.210526
chr28	11703997	2	36	T:0.777778	TGTAA:0.222222		
chr28	11703998	2	36	A:0.861111	ATATATG:0.138889		
chr28	11704093	2	40	AACTT:0.925	A:0.075		
chr28	11704168	2	38	T:0.447368	G:0.552632		

chr28	11704368	2	36	C:0.833333	T:0.166667
chr28	11704375	2	36	C:1 G:0	
chr28	11705080	2	40	ACCAAGCAGTG:0.7	A:0.3
chr28	11705272	2	38	G:0.736842	A:0.263158
chr28	11705417	2	30	T:0.0333333	C:0.966667
chr28	11705474	2	36	CCTCT:0.0555556	C:0.944444
chr28	11705581	2	32	G:0.75 GATTT:0.25	
chr28	11706162	2	36	C:0.972222	T:0.0277778
chr28	11706172	2	36	G:1 A:0	
chr28	11706272	2	38	T:0.184211	C:0.815789
chr28	11706273	2	38	G:0.789474	A:0.210526
chr28	11706669	2	38	G:0.973684	A:0.0263158
chr28	11706952	2	34	T:0.441176	G:0.558824
chr28	11707335	2	38	T:0.473684	C:0.526316
chr28	11707436	2	40	A:0.8 ACTTCTT:0.2	
chr28	11707449	2	38	A:0.0526316	C:0.947368
chr28	11707452	2	38	A:0.763158	C:0.236842
chr28	11707559	2	34	G:0.852941	A:0.147059
chr28	11707984	2	36	G:0.972222	A:0.0277778
chr28	11708024	2	34	G:0.0294118	C:0.970588
chr28	11708418	2	30	G:0.866667	A:0.133333
chr28	11708746	2	36	G:1 A:0	
chr28	11708955	2	36	C:1 T:0	
chr28	11709127	4	36	CT:0.277778	C:0.444444
CTT:0.138889		CTTT:0.138889			
chr28	11709326	2	38	C:0.184211	T:0.815789
chr28	11709496	2	36	G:0.944444	A:0.0555556
chr28	11709734	2	38	C:0.473684	T:0.526316
chr28	11710319	2	40	A:0.85 T:0.15	
chr28	11710511	2	40	G:0.85 A:0.15	
chr28	11710516	2	40	A:0.85 G:0.15	
chr28	11710780	2	36	C:0.777778	CTT:0.222222
chr28	11710832	3	38	CAA:0.315789	C:0.263158
CA:0.421053					
chr28	11710891	2	38	GA:1 G:0	
chr28	11711092	2	38	G:0.605263	A:0.394737
chr28	11711396	2	38	G:0.631579	A:0.368421
chr28	11711437	2	36	T:0.861111	C:0.138889
chr28	11711629	2	38	G:1 A:0	
chr28	11711654	2	36	T:0.416667	C:0.583333
chr28	11712607	2	40	C:0.8 T:0.2	
chr28	11712708	2	38	T:0.0263158	C:0.973684
chr28	11712782	2	38	T:0.0263158	G:0.973684
chr28	11712968	2	38	G:0.631579	T:0.368421
chr28	11713049	2	36	C:0.0277778	T:0.972222
chr28	11713254	2	36	T:0.694444	C:0.305556
chr28	11713386	2	36	G:0.0555556	A:0.944444
chr28	11713631	2	34	G:0.647059	A:0.352941
chr28	11713777	2	40	A:0.95 G:0.05	
chr28	11713859	2	36	G:0.388889	A:0.611111
chr28	11713863	2	38	T:0.973684	C:0.0263158
chr28	11713956	2	38	C:0.947368	T:0.0526316
chr28	11714060	2	40	C:0.875 T:0.125	
chr28	11714110	2	38	A:0.0526316	G:0.947368

chr28	11714200 2	36	T:0.666667	A:0.333333
chr28	11714236 2	36	T:0.972222	C:0.0277778
chr28	11714550 2	38	T:0.947368	A:0.0526316
chr28	11714751 2	38	C:0.815789	T:0.184211
chr28	11715190 2	40	C:0.775	T:0.225
chr28	11715237 2	38	G:0.473684	A:0.526316
chr28	11715489 4	40	ATGTG:0.65	A:0.075	ATG:0.15
ATGTGTG:0.125
chr28	11715586 2	36	T:0.833333	G:0.166667
chr28	11715625 2	32	G:0.78125	GA:0.21875
chr28	11715826 2	36	A:1	T:0
chr28	11715885 7	38	AACACACAC:0.289474	A:
0.0789474	AAC:0.263158	AACAC:0.157895	AACACAC:0.157895
AACACACACAC:0	AACACACACACAC:0.0526316
chr28	11716376 2	36	A:0.694444	G:0.305556
chr28	11716569 2	40	C:0.275	T:0.725
chr28	11716825 2	40	T:0.725	C:0.275
chr28	11717109 2	36	C:0.805556	G:0.194444
chr28	11717110 2	40	A:1	ACTGTTT:0
chr28	11717112 2	40	A:1	T:0
chr28	11717115 2	40	A:1	G:0
chr28	11717116 2	40	T:1	TGTGTTCTGTTTTA:0
chr28	11717266 2	36	A:0.388889	G:0.611111
chr28	11717510 2	40	G:0.775	A:0.225
chr28	11717633 2	30	A:0.666667	G:0.333333
chr28	11717756 2	32	G:0.96875	C:0.03125
chr28	11717888 2	34	C:0.735294	T:0.264706
chr28	11718368 2	40	C:0.8	T:0.2
chr28	11718390 2	40	C:0.825	T:0.175
chr28	11718504 2	38	G:0.631579	C:0.368421
chr28	11718548 2	36	C:0.777778	T:0.222222
chr28	11718549 2	36	C:0.666667	T:0.333333
chr28	11718550 2	40	C:0.675	T:0.325
chr28	11718551 2	40	G:0.375	A:0.625
chr28	11718565 2	40	G:0.375	A:0.625
chr28	11718678 2	36	A:0.361111	G:0.638889
chr28	11718698 2	38	C:0.763158	T:0.236842
chr28	11718916 2	38	GA:0.763158	G:0.236842
chr28	11718940 2	40	T:0.525	C:0.475
chr28	11719139 2	40	G:0.625	GTCCA:0.375
chr28	11719152 2	40	C:0.425	G:0.575
chr28	11719208 2	36	G:0.555556	A:0.444444
chr28	11719240 2	36	G:0.75	A:0.25
chr28	11719633 2	36	G:0.722222	A:0.277778
chr28	11720081 2	40	T:0.825	C:0.175
chr28	11720104 2	40	G:1	A:0
chr28	11720159 2	36	G:1	T:0
chr28	11720191 2	34	A:0.558824	G:0.441176
chr28	11720353 2	38	C:0.5	T:0.5
chr28	11720469 2	34	G:0.441176	A:0.558824
chr28	11720544 2	40	G:0.725	A:0.275
chr28	11720960 2	40	A:0.825	T:0.175
chr28	11720994 2	40	T:0.5	C:0.5
chr28	11721002 2	38	A:0.736842	G:0.263158

chr28	11721029	2	38	T:0.973684	A:0.0263158
chr28	11721260	2	40	C:0.025	A:0.975
chr28	11721292	2	38	C:0.789474	G:0.210526
chr28	11721367	2	36	T:0.805556	C:0.194444
chr28	11721379	2	38	C:0.763158	T:0.236842
chr28	11721484	2	36	G:0.0277778	A:0.972222
chr28	11721567	2	38	T:0.0526316	C:0.947368
chr28	11721850	2	38	G:0.605263	A:0.394737
chr28	11722003	2	38	G:0.815789	A:0.184211
chr28	11722131	2	38	G:0.578947	C:0.421053
chr28	11722288	2	34	C:0.0294118	T:0.970588
chr28	11722477	2	36	A:0.861111	G:0.138889
chr28	11722478	2	36	G:0.861111	T:0.138889
chr28	11722493	2	38	C:0.789474	A:0.210526
chr28	11722659	2	40	C:1	T:0
chr28	11722660	2	40	A:0.6	G:0.4
chr28	11722665	2	40	C:0.975	A:0.025
chr28	11722748	2	36	TACTC:1	T:0
chr28	11723119	2	36	G:0.694444	T:0.305556
chr28	11723198	2	38	A:0.684211	G:0.315789
chr28	11723529	2	38	G:0.526316	A:0.473684
chr28	11723617	2	36	T:0.0277778	C:0.972222
chr28	11723841	2	34	C:0.323529	G:0.676471
chr28	11724083	2	38	CA:0.815789	C:0.184211
chr28	11724092	2	38	C:0.815789	T:0.184211
chr28	11724129	2	40	A:0.825	AGTAAT:0.175
chr28	11724174	2	38	T:0.947368	G:0.0526316
chr28	11724300	2	40	G:0.35	A:0.65
chr28	11724624	2	38	A:0.868421	ACACGG:0.131579
chr28	11724765	2	36	C:0.777778	A:0.222222
chr28	11724983	2	40	G:0.4	A:0.6
chr28	11725122	2	36	A:0.833333	C:0.166667
chr28	11725132	2	34	G:0.5	GA:0.5
chr28	11725179	2	38	A:0.289474	G:0.710526
chr28	11725348	2	36	G:0.972222	T:0.0277778
chr28	11725375	2	38	A:0.763158	G:0.236842
chr28	11725670	2	38	C:0.789474	G:0.210526
chr28	11726126	2	38	G:0.263158	A:0.736842
chr28	11726129	2	38	A:0.763158	G:0.236842
chr28	11726369	2	34	A:1	C:0
chr28	11726394	2	36	A:0.583333	C:0.416667
chr28	11726402	2	36	T:1	C:0
chr28	11726408	2	36	G:0.333333	A:0.666667
chr28	11726499	2	38	G:0.973684	C:0.0263158
chr28	11726540	2	34	G:0.558824	A:0.441176
chr28	11726628	2	36	C:0.972222	T:0.0277778
chr28	11726945	2	38	T:0.815789	C:0.184211
chr28	11726980	2	40	G:0.95	A:0.05
chr28	11727124	2	36	A:0.333333	AGTT:0.666667
chr28	11727419	2	38	A:0.0526316	G:0.947368
chr28	11727682	2	40	G:0.975	C:0.025
chr28	11727699	2	40	G:0.975	A:0.025
chr28	11727738	2	36	A:0.333333	T:0.666667
chr28	11727777	2	40	G:0.775	C:0.225

chr28	11727890	2	38	CTTTAT:0.342105	C:0.657895
chr28	11727942	2	38	G:0.526316	C:0.473684
chr28	11728046	2	34	G:1	A:0
chr28	11728195	2	40	G:1	A:0
chr28	11728438	2	32	T:0.75	C:0.25
chr28	11728532	2	36	T:0.75	TA:0.25
chr28	11728568	2	40	T:0.55	C:0.45
chr28	11728934	2	38	T:0	C:1
chr28	11728968	2	36	G:0.694444	A:0.305556
chr28	11729059	2	36	A:0.861111	G:0.138889
chr28	11729062	2	38	AC:0.763158	A:0.236842
chr28	11729189	2	32	A:0.09375	G:0.90625
chr28	11729208	2	30	A:0.566667	AT:0.433333
chr28	11729336	2	34	A:0.764706	T:0.235294
chr28	11729414	2	36	A:0.722222	AT:0.277778
chr28	11729845	2	38	G:0.815789	GT:0.184211
chr28	11729856	2	38	C:0.815789	T:0.184211
chr28	11730457	2	40	T:0.6	A:0.4
chr28	11730557	2	38	C:0.605263	A:0.394737
chr28	11730558	2	38	A:0.605263	T:0.394737
chr28	11730678	2	40	G:1	T:0
chr28	11731028	2	34	C:0.382353	T:0.617647
chr28	11731043	2	34	AT:0.764706	A:0.235294
chr28	11731203	2	34	A:0.735294	G:0.264706
chr28	11731384	2	28	A:0.0357143	C:0.964286
chr28	11731464	2	38	T:0.789474	C:0.210526
chr28	11731567	2	38	A:0.710526	G:0.289474
chr28	11731671	2	38	T:1	C:0
chr28	11731708	2	36	G:0.638889	A:0.361111
chr28	11731739	2	38	T:0.815789	TA:0.184211
chr28	11731740	2	38	G:0.815789	A:0.184211
chr28	11731743	2	40	G:0.75	T:0.25
chr28	11732347	2	38	A:1	C:0
chr28	11732348	2	38	G:1	T:0
chr28	11732452	2	34	T:0.823529	C:0.176471
chr28	11732599	2	38	C:0.842105	T:0.157895
chr28	11732701	2	34	T:0.617647	C:0.382353
chr28	11732767	2	36	G:0.805556	A:0.194444
chr28	11732838	2	36	C:0.694444	T:0.305556
chr28	11732942	2	40	A:0.625	T:0.375
chr28	11733290	2	40	G:0.95	A:0.05
chr28	11733329	2	38	A:0.0263158	G:0.973684
chr28	11733710	2	36	T:0.0833333	C:0.916667
chr28	11733715	2	36	T:0.805556	C:0.194444
chr28	11733775	2	34	T:0.0588235	C:0.941176
chr28	11734031	2	40	G:0.775	A:0.225
chr28	11734088	2	40	T:0.9	G:0.1
chr28	11734722	2	38	G:0.973684	A:0.0263158
chr28	11734753	2	38	T:0.973684	A:0.0263158
chr28	11735153	2	38	C:0.921053	T:0.0789474
chr28	11735175	2	38	C:0.763158	CT:0.236842
chr28	11735301	2	36	T:0.666667	C:0.333333
chr28	11735469	2	40	T:1	G:0
chr28	11735580	2	36	TC:0.805556	T:0.194444

chr28	11735631	2	36	T:0.722222	G:0.277778		
chr28	11735638	2	36	T:0.722222	A:0.277778		
chr28	11735692	2	40	T:0.025	A:0.975		
chr28	11735701	2	40	TAATGTCACTAAGTTTTG:0.75	T:0.25		
chr28	11735782	2	40	C:0.75	T:0.25		
chr28	11735932	2	38	A:0.763158	C:0.236842		
chr28	11736011	2	38	T:0.842105	G:0.157895		
chr28	11736058	3	40	T:0.375	TA:0.45	TAA:0.175	
chr28	11736124	2	38	G:0.789474	A:0.210526		
chr28	11736154	2	40	G:1	A:0		
chr28	11736271	2	38	A:1	G:0		
chr28	11736327	2	36	T:0.805556	C:0.194444		
chr28	11736399	2	34	T:0.294118	C:0.705882		
chr28	11736492	2	34	G:0.735294	A:0.264706		
chr28	11736637	2	36	T:0.777778	A:0.222222		
chr28	11736659	2	34	A:0.764706	T:0.235294		
chr28	11736793	2	38	A:0.763158	G:0.236842		
chr28	11736832	2	36	T:0.833333	G:0.166667		
chr28	11736989	2	36	C:0.944444	A:0.0555556		
chr28	11737047	2	36	G:0.0555556	A:0.944444		
chr28	11737114	2	34	A:0.5	G:0.5		
chr28	11737167	2	36	A:0.0555556	C:0.944444		
chr28	11737431	2	38	T:1	G:0		
chr28	11737474	2	36	C:0.722222	T:0.277778		
chr28	11737555	2	36	C:0.75	T:0.25		
chr28	11737609	2	38	T:0.815789	G:0.184211		
chr28	11737704	2	36	C:1	T:0		
chr28	11737793	2	38	A:1	G:0		
chr28	11737835	2	40	C:0.55	T:0.45		
chr28	11737943	2	38	T:0.684211	C:0.315789		
chr28	11737988	2	32	C:0.75	A:0.25		
chr28	11738674	2	36	A:0.75	G:0.25		
chr28	11738714	2	34	A:0.970588	G:0.0294118		
chr28	11738894	2	36	T:0.944444	C:0.0555556		
chr28	11738992	2	40	G:0.825	C:0.175		
chr28	11739053	2	40	C:0.875	T:0.125		
chr28	11739304	2	36	G:0.694444	A:0.305556		
chr28	11739353	2	36	C:0.861111	CTG:0.138889		
chr28	11739355	4	36	C:0.527778	G:0.138889	CTCTG:0.333333	CTGTG:0
chr28	11739443	2	38	A:0	G:1		
chr28	11739473	2	32	T:0.71875	C:0.28125		
chr28	11739475	2	32	G:0.71875	A:0.28125		
chr28	11739534	3	40	CCT:0.775	C:0.025	CCTCTCT:0.2	
chr28	11739570	2	40	A:0.7	G:0.3		
chr28	11739571	3	40	TAAAAATTAAAAAAAAACTTAAAAAA:0.375	T:0.475	TAAAATTAAAAAAAAACTTAAAAAA:0.15	
chr28	11739579	2	40	AAAAAAAAACTTAAAAAAAAAAATT:0.55	A:0.45		
chr28	11739721	2	32	A:0.75	AAG:0.25		
chr28	11739753	2	34	G:0.941176	A:0.0588235		
chr28	11739762	2	34	A:0.647059	T:0.352941		
chr28	11739763	2	34	G:0.647059	A:0.352941		

chr28	11739831	2	40	T:0.7	C:0.3
chr28	11739926	2	36	G:0.444444	T:0.555556
chr28	11740697	2	38	A:0.368421	C:0.631579
chr28	11740934	2	38	T:0.815789	A:0.184211
chr28	11741024	2	32	C:0.75	T:0.25
chr28	11741241	2	34	C:1	T:0
chr28	11742019	2	38	G:0.631579	A:0.368421
chr28	11742044	2	38	T:0.0526316	G:0.947368
chr28	11742213	2	38	T:0.578947	C:0.421053
chr28	11742283	2	34	C:0.705882	T:0.294118
chr28	11742362	2	40	T:1	C:0
chr28	11742433	2	38	C:0.552632	G:0.447368
chr28	11742883	2	36	G:0.666667	A:0.333333
chr28	11743015	2	34	A:1	C:0
chr28	11743065	2	38	G:0.210526	A:0.789474
chr28	11743074	2	38	C:0.842105	T:0.157895
chr28	11743156	2	32	C:0.96875	T:0.03125
chr28	11743195	2	38	A:0.868421	C:0.131579
chr28	11743291	2	34	G:0.205882	A:0.794118
chr28	11743363	2	34	T:0.852941	C:0.147059
chr28	11743412	2	36	T:0.944444	C:0.0555556
chr28	11743534	2	32	C:0.25	T:0.75
chr28	11743595	2	34	T:0.205882	C:0.794118
chr28	11743633	2	28	C:0.714286	T:0.285714
chr28	11743739	2	38	C:0.263158	T:0.736842
chr28	11743856	2	36	C:0.222222	T:0.777778
chr28	11743871	2	34	C:0	G:1
chr28	11744902	2	34	T:1	C:0
chr28	11745129	2	34	G:0.823529	A:0.176471
chr28	11745150	2	34	C:0.764706	T:0.235294
chr28	11745153	2	34	G:0.294118	A:0.705882
chr28	11745162	2	36	A:1	C:0
chr28	11745296	2	40	G:0.05	A:0.95
chr28	11745342	2	36	C:0.777778	CT:0.222222
chr28	11745693	2	40	A:0.75	T:0.25
chr28	11745711	2	38	G:0.0526316	A:0.947368
chr28	11745848	2	38	A:0.0789474	C:0.921053
chr28	11745895	2	40	C:0.475	G:0.525
chr28	11746260	2	40	C:0.75	A:0.25
chr28	11746288	2	40	A:0.025	ATCT:0.975
chr28	11746398	2	36	C:0.0833333	T:0.916667
chr28	11746450	2	38	G:0.0526316	A:0.947368
chr28	11746455	2	38	C:0.0526316	T:0.947368
chr28	11746530	2	38	A:0.0526316	G:0.947368
chr28	11746608	2	36	C:0.861111	T:0.138889
chr28	11746624	2	40	T:0.05	C:0.95
chr28	11746672	2	38	T:0.815789	C:0.184211
chr28	11746701	2	38	A:0.263158	G:0.736842
chr28	11746769	2	38	T:0.736842	TAGAC:0.263158
chr28	11746774	2	38	A:0.315789	C:0.684211
chr28	11746779	2	38	T:0.0526316	C:0.947368
chr28	11746815	2	40	G:0.8	GC:0.2
chr28	11746863	2	40	T:0.1	G:0.9
chr28	11747207	2	34	T:0.970588	C:0.0294118

chr28	11747361	2	34	T:0.382353	C:0.617647
chr28	11747383	2	32	C:0.40625	T:0.59375
chr28	11747404	2	34	A:0.382353	C:0.617647
chr28	11747492	2	34	T:0.529412	C:0.470588
chr28	11747632	2	38	G:0.763158	T:0.236842
chr28	11747678	2	40	A:0.8	G:0.2
chr28	11747686	2	40	C:1	T:0
chr28	11747705	2	38	G:0.815789	A:0.184211
chr28	11747918	2	38	C:0.815789	T:0.184211
chr28	11748026	2	40	A:0.825	G:0.175
chr28	11748041	2	38	T:0.5	C:0.5
chr28	11748103	2	40	C:0.8	A:0.2
chr28	11748179	2	40	G:0.775	A:0.225
chr28	11748287	2	38	T:0.657895	A:0.342105
chr28	11748536	2	36	A:0.805556	C:0.194444
chr28	11748543	2	40	TA:0.675	T:0.325
chr28	11748559	2	40	G:0.675	T:0.325
chr28	11748669	2	36	A:0.805556	T:0.194444
chr28	11748694	2	36	C:0.805556	T:0.194444
chr28	11748695	2	34	T:0.411765	A:0.588235
chr28	11748760	2	36	G:0.777778	T:0.222222
chr28	11748768	2	36	A:0.777778	T:0.222222
chr28	11748817	2	36	C:0.694444	T:0.305556
chr28	11748826	2	38	T:0.789474	C:0.210526
chr28	11749230	2	36	TC:0.805556	T:0.194444
chr28	11749346	2	38	A:0.0263158	T:0.973684
chr28	11749371	2	38	T:0.526316	TGTC:0.473684
chr28	11749403	2	36	T:0.5	C:0.5
chr28	11749624	2	36	AT:0.833333	A:0.166667
chr28	11749767	2	38	C:0.736842	T:0.263158
chr28	11750075	2	40	G:0.5	T:0.5
chr28	11750108	2	40	C:0.65	T:0.35
chr28	11750118	2	40	G:0.65	A:0.35
chr28	11750298	2	38	A:0.842105	T:0.157895
chr28	11750397	2	40	G:0.5	T:0.5
chr28	11750477	2	40	A:0.6	C:0.4
chr28	11750496	2	36	T:0.583333	G:0.416667
chr28	11750714	2	34	C:0.823529	T:0.176471
chr28	11750793	2	36	T:0.833333	G:0.166667
chr28	11751106	2	40	C:1	T:0
chr28	11751221	2	40	AACTC:0.75	A:0.25
chr28	11751359	2	38	C:0.447368	T:0.552632
chr28	11751412	2	38	A:0.473684	G:0.526316
chr28	11751632	2	34	C:1	G:0
chr28	11751640	2	34	C:1	A:0
chr28	11751707	2	32	G:0.625	C:0.375
chr28	11751732	2	32	A:0.9375	G:0.0625
chr28	11751741	2	34	A:0.735294	G:0.264706
chr28	11751847	2	32	C:0.71875	CT:0.28125
chr28	11751848	2	34	T:1	C:0
chr28	11752068	2	38	G:0.710526	A:0.289474
chr28	11752096	2	40	T:0.7	A:0.3
chr28	11752211	2	40	G:0.975	A:0.025
chr28	11752340	2	36	A:0.666667	G:0.333333

chr28	11752566	2	40	TGTTTTTTG:0.975	T:0.025
chr28	11752614	2	34	ATTTATTT:0.205882	A:0.794118
chr28	11752954	2	38	T:0.710526	C:0.289474
chr28	11752959	2	38	T:0	TA:1
chr28	11752992	2	40	T:0	C:1
chr28	11752993	2	40	G:0	T:1
chr28	11752996	2	40	TA:0	T:1
chr28	11753001	2	40	A:0	AT:1
chr28	11753011	2	40	C:0	T:1
chr28	11753023	2	40	C:0	CT:1
chr28	11753061	2	36	T:0	C:1
chr28	11753063	2	36	G:0	T:1
chr28	11753067	2	36	A:0	AT:1
chr28	11753073	2	36	C:0	CCTAA:1
chr28	11753075	2	36	G:0	A:1
chr28	11753077	2	36	ATAT:0	A:1
chr28	11753083	2	36	G:0	GT:1
chr28	11753088	2	36	ATT:0	A:1
chr28	11753192	2	32	C:0.71875	T:0.28125
chr28	11753196	2	32	T:1	C:0
chr28	11753328	2	38	G:0.0263158	A:0.973684
chr28	11753366	2	38	C:0.789474	G:0.210526
chr28	11753433	2	40	T:0.35	G:0.65
chr28	11753690	2	38	C:0.710526	T:0.289474
chr28	11753833	2	40	A:0.725	G:0.275
chr28	11753862	2	40	TTA:0.7	T:0.3
chr28	11753866	2	40	T:0.7	TCA:0.3
chr28	11753971	2	40	T:0.7	A:0.3
chr28	11753989	2	40	C:0.7	T:0.3
chr28	11754013	2	40	G:0.7	A:0.3
chr28	11754054	2	38	C:1	T:0
chr28	11754316	2	40	C:0.975	T:0.025
chr28	11754371	2	38	T:0.973684	G:0.0263158
chr28	11754373	2	40	C:0.025	T:0.975
chr28	11754464	2	40	A:0.975	G:0.025
chr28	11754469	2	40	G:0.75	A:0.25
chr28	11754654	2	36	G:0.694444	A:0.305556
chr28	11754906	2	28	A:0.75	G:0.25
chr28	11755001	2	26	G:0	A:1
chr28	11755008	2	22	G:0.181818	A:0.818182
chr28	11755023	2	22	AG:0.181818	A:0.818182
chr28	11755177	2	34	A:0.0294118	C:0.970588
chr28	11755253	2	38	C:0.710526	A:0.289474
chr28	11755303	2	36	T:0.722222	C:0.277778
chr28	11755430	2	40	T:0.7	TTTGTA:0.3
chr28	11755523	2	38	AT:0.710526	A:0.289474
chr28	11755599	2	38	A:0.973684	G:0.0263158
chr28	11755688	2	36	G:0.805556	A:0.194444
chr28	11755801	2	38	T:0.973684	C:0.0263158
chr28	11755835	2	40	TCAGTCCC:0.725	T:0.275
chr28	11755867	2	40	G:0.725	C:0.275
chr28	11755935	2	38	C:0.684211	T:0.315789
chr28	11756119	2	36	T:0.777778	C:0.222222

chr28	11756130	2	36	T:0.722222	C:0.277778
chr28	11756251	2	36	C:0.694444	T:0.305556
chr28	11756326	2	36	C:0.694444	G:0.305556
chr28	11756376	2	34	G:0.823529	A:0.176471
chr28	11756482	2	40	C:0.7	CT:0.3
chr28	11756520	2	40	T:0.975	C:0.025
chr28	11756606	2	34	T:0.676471	C:0.323529
chr28	11756788	2	40	C:1	A:0
chr28	11756847	2	38	A:0.973684	G:0.0263158
chr28	11756853	2	38	A:0.710526	G:0.289474
chr28	11756973	2	36	G:0.722222	T:0.277778
chr28	11757038	2	38	A:0.973684	G:0.0263158
chr28	11757176	2	40	T:0.975	C:0.025
chr28	11757276	2	38	A:1	C:0
chr28	11757417	2	34	TG:0.735294	T:0.264706
chr28	11757428	2	34	G:0.735294	A:0.264706
chr28	11757535	2	38	T:0.684211	C:0.315789
chr28	11757544	2	38	G:0.684211	A:0.315789
chr28	11757665	2	38	G:1	T:0
chr28	11757782	2	38	G:0.736842	A:0.263158
chr28	11757820	2	38	C:1	T:0
chr28	11757837	2	38	T:0.736842	A:0.263158
chr28	11757933	2	38	C:0.710526	T:0.289474
chr28	11757943	2	38	A:1	G:0
chr28	11758254	2	32	C:0.71875	T:0.28125
chr28	11758292	2	40	TA:0.75	T:0.25
chr28	11758421	2	34	A:0.705882	G:0.294118
chr28	11758542	7	36	TTATATATA:0.194444	T:
0.361111	TTA:0.0555556	TTATA:0.0277778	TTATATA:0.222222
TTATATATATA:0.0277778	TTATATATATATA:0.111111
chr28	11758583	2	32	G:0.78125	A:0.21875
chr28	11758654	2	36	A:0.722222	G:0.277778
chr28	11758731	2	32	AC:0.96875	A:0.03125
chr28	11758736	2	32	A:0.96875	AG:0.03125
chr28	11758846	2	32	AC:1	A:0
chr28	11759155	2	28	A:0.535714	G:0.464286
chr28	11759257	2	32	AG:0.78125	A:0.21875
chr28	11759580	2	36	G:1	GTA:0
chr28	11759626	2	36	T:0.972222	TGTA:0.0277778
chr28	11759633	3	38	T:0.684211	TAGATTAGA:
0.0263158	TAGATTAGATAGATAGATAGATAGA:0.289474
chr28	11759650	2	38	T:1	TAG:0
chr28	11759652	2	38	T:1	TAGA:0
chr28	11759654	2	38	G:1	A:0
chr28	11759656	2	38	C:1	A:0
chr28	11759659	2	38	T:1	G:0
chr28	11759663	2	38	T:1	G:0
chr28	11759666	2	38	A:1	ATTGG:0
chr28	11759678	2	38	CATTG:1	C:0
chr28	11759687	2	38	TATGTG:1	T:0
chr28	11759696	2	38	G:0.684211	A:0.315789
chr28	11759783	2	38	T:0.763158	C:0.236842
chr28	11759800	2	36	C:0.944444	T:0.0555556
chr28	11759894	2	38	AT:0.657895	A:0.342105

chr28	11759944	2	40	T:0.975	C:0.025		
chr28	11760019	2	34	C:0.970588	CA:0.0294118		
chr28	11760300	2	40	C:0.975	T:0.025		
chr28	11760339	2	38	A:0.763158	G:0.236842		
chr28	11760449	2	40	C:0.75	T:0.25		
chr28	11760463	2	40	C:0.75	T:0.25		
chr28	11760472	2	40	A:0.75	T:0.25		
chr28	11760557	2	40	T:0.7	C:0.3		
chr28	11760585	2	40	A:0.725	G:0.275		
chr28	11760624	2	40	T:0.975	C:0.025		
chr28	11760663	2	38	C:1	CTTTT:0		
chr28	11760665	2	38	T:0.842105	TTTC:0.157895		
chr28	11760699	3	38	C:0.710526	G:0.0526316	CAG:0.236842	
chr28	11760752	2	38	C:0.973684	T:0.0263158		
chr28	11760779	2	38	C:0.736842	A:0.263158		
chr28	11760781	2	38	C:0.973684	T:0.0263158		
chr28	11760813	2	36	C:1	T:0		
chr28	11760906	2	36	A:0.722222	G:0.277778		
chr28	11760940	2	38	C:0.736842	T:0.263158		
chr28	11761008	2	38	C:0.947368	T:0.0526316		
chr28	11761240	2	40	G:0.7	A:0.3		
chr28	11761340	2	36	A:0.638889	G:0.361111		
chr28	11761480	2	38	C:0.973684	T:0.0263158		
chr28	11761669	2	34	C:0.970588	A:0.0294118		
chr28	11761738	2	40	T:0.7	C:0.3		
chr28	11761758	2	40	T:1	TAATAATCATTTTAAAA:0		
chr28	11761798	2	40	CAT:0.975	C:0.025		
chr28	11762033	2	40	C:0.95	T:0.05		
chr28	11762488	2	38	G:0.973684	A:0.0263158		
chr28	11762505	2	38	A:0.973684	G:0.0263158		
chr28	11762679	2	40	CCA:1	C:0		
chr28	11762724	2	38	A:0.973684	G:0.0263158		
chr28	11762764	4	40	TACACACACACAC:0.3	T:0.175	TACACACACACACAC:0.425	TACACACACACACACAC:0.1
chr28	11762802	2	40	C:0.95	T:0.05		
chr28	11762821	2	36	G:0.777778	A:0.222222		
chr28	11762873	2	38	G:0.973684	A:0.0263158		
chr28	11763027	2	36	A:0.666667	ACT:0.333333		
chr28	11763144	2	36	A:1	C:0		
chr28	11763213	2	38	A:0.710526	C:0.289474		
chr28	11763216	2	38	T:1	A:0		
chr28	11763384	2	40	G:1	T:0		
chr28	11763486	2	34	T:1	A:0		
chr28	11763540	2	34	C:0.0294118	A:0.970588		
chr28	11763601	2	40	C:0.7	G:0.3		
chr28	11763663	2	38	TTTC:0.868421	T:0.131579		
chr28	11763713	2	38	G:0.368421	C:0.631579		
chr28	11763777	2	38	G:0.947368	A:0.0526316		
chr28	11764044	2	32	A:0.9375	G:0.0625		
chr28	11764060	2	32	T:0.5625	G:0.4375		
chr28	11764519	2	28	T:1	C:0		
chr28	11764552	2	34	G:1	A:0		
chr28	11764590	2	34	TG:0.323529	T:0.676471		

chr28	11764592	2	34	GTTT:0.823529	G:0.176471		
chr28	11764890	2	40	T:0.975	TAAG:0.025		
chr28	11765223	2	36	AATC:0.944444	A:0.0555556		
chr28	11765369	2	34	C:0.970588	A:0.0294118		
chr28	11765425	2	38	C:0.789474	T:0.210526		
chr28	11765464	2	40	A:0.975	ATTATTTTTTATTTTATTATTT:0.025		
chr28	11765465	2	40	C:0.975	CATTAAATTATTTTAAT:0.025		
chr28	11765466	2	40	C:0.975	T:0.025		
chr28	11765518	2	36	T:0.972222	C:0.0277778		
chr28	11765627	2	40	C:1	T:0		
chr28	11765879	2	38	G:0.710526	T:0.289474		
chr28	11766056	2	34	A:0.970588	C:0.0294118		
chr28	11766099	2	38	A:0.315789	C:0.684211		
chr28	11766106	2	38	T:0.973684	G:0.0263158		
chr28	11766189	2	34	A:0.970588	T:0.0294118		
chr28	11766254	2	32	A:0.34375	G:0.65625		
chr28	11766328	4	38	AAGAGAGAG:0.0789474	A:0.105263	AAGAGAG:0.789474	AAGAGAGAGAGAGAG:0.0263158
chr28	11766490	2	36	CTTTTTTTTTTTT:0.527778	C:0.472222		
chr28	11766584	2	34	C:0.617647	T:0.382353		
chr28	11766717	2	40	A:0.95	G:0.05		
chr28	11767154	2	38	G:0.736842	T:0.263158		
chr28	11767267	2	40	G:0.975	A:0.025		
chr28	11767311	2	36	A:0.222222	G:0.777778		
chr28	11767397	2	38	C:0.973684	T:0.0263158		
chr28	11767479	2	40	A:0.95	G:0.05		
chr28	11767489	2	40	G:0.05	A:0.95		
chr28	11767576	2	34	A:0.323529	T:0.676471		
chr28	11767711	2	38	T:1	C:0		
chr28	11767900	2	38	T:0.710526	C:0.289474		
chr28	11768021	2	38	G:0.0263158	GGAAA:0.973684		
chr28	11768093	2	38	T:0.973684	C:0.0263158		
chr28	11768261	2	36	CA:0	C:1		
chr28	11768466	2	34	C:0.970588	G:0.0294118		
chr28	11768605	2	38	G:1	A:0		
chr28	11768624	2	36	A:0.75	G:0.25		
chr28	11768768	2	34	T:1	C:0		
chr28	11768919	2	36	T:0.0277778	A:0.972222		
chr28	11768996	2	36	A:0.944444	G:0.0555556		
chr28	11769101	2	34	C:0.735294	CTA:0.264706		
chr28	11769122	2	36	A:0.944444	C:0.0555556		
chr28	11769341	2	38	C:1	A:0		
chr28	11769666	2	36	TGGCTTTCCCAAGTAA:1	T:0		
chr28	11769689	2	36	C:0.916667	A:0.0833333		
chr28	11769865	2	38	A:0.342105	G:0.657895		
chr28	11770022	2	38	G:1	C:0		
chr28	11770722	2	38	A:0	G:1		
chr28	11770739	2	38	TG:0.736842	T:0.263158		
chr28	11771238	2	38	C:0.973684	CTTA:0.0263158		
chr28	11771240	2	38	T:0.0263158	TATG:0.973684		
chr28	11771308	2	38	T:1	C:0		
chr28	11771840	2	34	C:0.735294	A:0.264706		

chr28	11772429	2	36	G:0.972222	C:0.0277778	
chr28	11772502	2	38	T:0.736842	G:0.263158	
chr28	11772564	2	34	T:0.0294118	C:0.970588	
chr28	11772595	2	36	AT:0.361111	A:0.638889	
chr28	11772603	2	36	T:1	A:0	
chr28	11772715	2	38	C:0.368421	A:0.631579	
chr28	11773090	2	34	A:0.441176	C:0.558824	
chr28	11773354	2	36	G:0.416667	A:0.583333	
chr28	11773427	2	38	A:0.736842	G:0.263158	
chr28	11774005	2	38	A:0.368421	C:0.631579	
chr28	11774216	2	36	C:1	T:0	
chr28	11774403	2	36	A:0.361111	G:0.638889	
chr28	11774797	2	38	A:0.394737	T:0.605263	
chr28	11774834	2	40	G:0.075	A:0.925	
chr28	11775084	2	38	A:0.342105	G:0.657895	
chr28	11775145	2	38	C:0.947368	T:0.0526316	
chr28	11775177	2	36	GAATT:1	G:0	
chr28	11775270	2	36	A:0.861111	G:0.138889	
chr28	11775358	2	36	C:0.305556	T:0.694444	
chr28	11775428	2	38	A:0.236842	G:0.763158	
chr28	11775447	2	38	G:0.236842	T:0.763158	
chr28	11775501	2	36	A:0.972222	G:0.0277778	
chr28	11775605	2	40	G:0.35	T:0.65	
chr28	11775627	2	40	T:0.35	C:0.65	
chr28	11775871	2	38	A:0.342105	AC:0.657895	
chr28	11775945	2	38	A:0.342105	G:0.657895	
chr28	11775964	2	38	A:0.342105	T:0.657895	
chr28	11776095	2	40	A:1	ATT:0	
chr28	11776097	2	40	T:1	TTTAAAAG:0	
chr28	11776100	2	40	A:1	AGTGATT:0	
chr28	11776103	2	40	A:1	AAAGACAGTG:0	
chr28	11776147	2	38	C:0.421053	T:0.578947	
chr28	11776163	2	38	G:0.394737	A:0.605263	
chr28	11776184	2	36	G:0.972222	A:0.0277778	
chr28	11776207	2	38	C:0.368421	G:0.631579	
chr28	11776297	2	40	T:0.35	C:0.65	
chr28	11776433	2	36	T:0.388889	C:0.611111	
chr28	11776447	2	34	A:0.352941	G:0.647059	
chr28	11777017	2	38	C:0.0789474	T:0.921053	
chr28	11777084	2	38	A:0.947368	G:0.0526316	
chr28	11777250	2	38	G:0.0526316	A:0.947368	
chr28	11777374	2	40	C:0.15	T:0.85	
chr28	11777375	2	40	A:0.15	G:0.85	
chr28	11777393	2	40	GTCGAGGAATGTTCCCTCTATC:0.15	G:0.85	
chr28	11777488	2	38	G:0.105263	A:0.894737	
chr28	11777788	2	38	C:1	G:0	
chr28	11777938	2	36	C:0.166667	T:0.833333	
chr28	11777976	2	32	TA:0.125	T:0.875	
chr28	11778017	2	30	GC:1	G:0	
chr28	11778027	2	32	C:1	CAA:0	
chr28	11778028	3	34	C:0.176471	A:0.823529	CAA:0
chr28	11778091	4	40	CATTTTATTTTATTTTATTTT:0.45		

C:0.05	CATTTTATTTT:0.3	CATTTTATTTTATTTT:0.2
chr28	11778217	2	32	C:1	T:0
chr28	11778246	2	34	C:0.0882353	T:0.911765
chr28	11778341	2	38	A:1	G:0
chr28	11778413	2	40	A:1	G:0
chr28	11778532	2	38	G:1	A:0
chr28	11778575	2	36	A:1	C:0
chr28	11778602	2	30	T:0.1	G:0.9
chr28	11778783	2	36	C:1	T:0
chr28	11778821	2	34	GCC:0.0882353	G:0.911765
chr28	11778825	2	34	C:1	T:0
chr28	11779002	2	28	CATA:1	C:0
chr28	11779171	2	40	G:0.725	A:0.275
chr28	11779249	2	38	G:0.0789474	GAA:0.921053
chr28	11779250	3	38	G:0	A:0.921053	GA:
0.0789474
chr28	11779353	2	40	C:1	T:0
chr28	11779410	2	36	T:1	C:0
chr28	11779573	2	38	C:0.105263	T:0.894737
chr28	11779590	2	38	C:0.342105	T:0.657895
chr28	11780213	2	40	C:0.8	T:0.2
chr28	11780473	2	38	C:0.578947	T:0.421053
chr28	11780645	2	38	A:0.710526	G:0.289474
chr28	11780700	2	36	A:0.944444	T:0.0555556
chr28	11780841	2	38	C:0.789474	T:0.210526
chr28	11780901	2	36	G:0.805556	A:0.194444
chr28	11781118	2	36	C:0.75	A:0.25
chr28	11781195	2	34	A:0.264706	C:0.735294
chr28	11781246	2	36	T:0.777778	G:0.222222
chr28	11781633	2	40	G:0.025	A:0.975
chr28	11781671	2	38	T:1	C:0
chr28	11781690	2	36	A:0.944444	AT:0.0555556
chr28	11782027	2	38	G:1	C:0
chr28	11782187	2	40	A:0.75	T:0.25
chr28	11782207	2	40	A:0.775	G:0.225
chr28	11782276	2	38	G:0.710526	A:0.289474
chr28	11782293	2	38	G:0.657895	A:0.342105
chr28	11782738	2	40	T:0.6	TGGGTCCCTGGGCTC:0.4
chr28	11782739	2	40	AC:0.6	A:0.4
chr28	11782818	2	34	A:1	G:0
chr28	11782888	2	38	T:0.710526	A:0.289474
chr28	11783074	2	34	C:0.823529	CCT:0.176471
chr28	11783132	3	40	A:0.35	AAC:0.65	AACAC:0
chr28	11783381	2	38	A:0.657895	C:0.342105
chr28	11783394	2	38	G:1	A:0
chr28	11783522	2	38	A:0	C:1
chr28	11783544	2	36	A:0.361111	G:0.638889
chr28	11783653	2	36	C:1	T:0
chr28	11783726	2	38	T:0.947368	G:0.0526316
chr28	11783784	2	40	G:1	A:0
chr28	11783812	4	38	T:0.605263	TG:0.0526316
TGGC:0.0526316	TGGCG:0.289474
chr28	11783861	2	38	AT:0.421053	A:0.578947
chr28	11784019	2	38	G:0.315789	A:0.684211

chr28 11784285 2 38 T:0.763158 A:0.236842
chr28 11784421 2 36 A:0.694444 T:0.305556
chr28 11784430 2 36 AT:0.611111 A:0.388889
chr28 11784451 2 38 T:0.0263158 G:0.973684
chr28 11784754 2 38 G:0.657895 A:0.342105
chr28 11784868 2 38 G:0.763158 A:0.236842
chr28 11785165 2 38 C:0.684211 T:0.315789
chr28 11785291 2 34 A:0.705882 C:0.294118
chr28 11785317 2 36 C:0.694444 CT:0.305556
chr28 11785352 2 40 G:0.425 A:0.575
chr28 11785677 2 38 G:0.657895 T:0.342105
chr28 11786110 2 38 C:1 T:0
chr28 11786350 2 38 A:0.473684 G:0.526316
chr28 11786469 2 38 C:0.657895 A:0.342105
chr28 11786795 2 38 T:0.605263 C:0.394737
chr28 11787108 3 36 C:0.611111 CT:0.166667
CTT:0.222222
chr28 11787247 2 38 G:0.947368 A:0.0526316
chr28 11787410 2 40 T:0.625 C:0.375
chr28 11787613 2 36 T:0.694444 G:0.305556
chr28 11787775 2 38 A:0.315789 T:0.684211
chr28 11787811 2 36 AT:0.944444 A:0.0555556
chr28 11787840 2 34 C:0.617647 T:0.382353
chr28 11788062 4 36 CCT:0.611111 C:0.0555556
CCTCT:0.194444 CCTCTCT:0.138889
chr28 11788087 3 36 C:0.75 CTG:0.25 CTCTG:0
chr28 11788253 2 38 G:0.947368 A:0.0526316
chr28 11788301 2 34 A:1 G:0
chr28 11788394 2 36 G:0.972222 A:0.0277778
chr28 11788490 2 36 C:0.805556 G:0.194444
chr28 11788587 2 38 A:0.289474 G:0.710526
chr28 11788962 2 36 CACA:0.944444 C:0.0555556
chr28 11788964 2 38 C:0.842105 T:0.157895
chr28 11788967 7 40 AAAAT:0.25 A:0 TAAAT:
0.275 AAAATAAAT:0.125 AAAATAAATAAAT:0 ATAAAT:0.2
AAAATAAATAAATAAAT:0.15
chr28 11789007 4 40 T:0.475 A:0.175 TAAAA:0.175
TAAATAAAA:0.175
chr28 11789017 2 36 T:0.916667 A:0.0833333
chr28 11789228 2 38 CAG:0.631579 C:0.368421
chr28 11789273 2 40 G:0.95 A:0.05
chr28 11789401 2 38 C:0.868421 CA:0.131579
chr28 11789780 2 38 C:0.736842 T:0.263158
chr28 11789807 2 40 C:0.6 T:0.4
chr28 11789860 2 38 A:0.605263 T:0.394737
chr28 11789949 2 40 G:1 GA:0
chr28 11790376 2 36 G:0.75 T:0.25
chr28 11790436 2 38 A:1 C:0
chr28 11790441 2 38 G:1 A:0
chr28 11790546 2 38 G:0.0526316 A:0.947368
chr28 11790555 2 40 C:0.6 A:0.4
chr28 11791122 2 38 C:1 T:0
chr28 11791647 2 38 T:0.0526316 C:0.947368
chr28 11791873 2 38 T:0 C:1

chr28	11791900	2	34	C:0.588235	T:0.411765		
chr28	11793201	2	36	C:0.694444	T:0.305556		
chr28	11793581	2	34	C:1	T:0		
chr28	11793605	2	36	G:1	A:0		
chr28	11793750	2	38	A:0.605263	AAG:0.394737		
chr28	11793873	2	40	A:0.025	C:0.975		
chr28	11793901	2	38	AT:0.763158	A:0.236842		
chr28	11794282	2	38	T:1	C:0		
chr28	11794300	2	40	G:0.725	T:0.275		
chr28	11794318	2	40	G:0.775	T:0.225		
chr28	11794971	2	36	G:1	A:0		
chr28	11794979	2	34	G:0	A:1		
chr28	11795991	2	38	C:0.736842	T:0.263158		
chr28	11796018	3	36	T:0.194444	TG:0.472222	TTG:0.333333	
chr28	11796027	2	38	G:0.842105	GGA:0.157895		
chr28	11796042	2	38	T:1	C:0		
chr28	11796175	2	40	G:1	A:0		
chr28	11796203	2	38	C:0.815789	T:0.184211		
chr28	11796322	2	40	C:0.75	G:0.25		
chr28	11796550	2	38	T:0.631579	C:0.368421		
chr28	11796653	2	36	G:1	T:0		
chr28	11796680	2	38	A:0.631579	C:0.368421		
chr28	11796783	3	30	TA:0.433333	T:0.233333	TAA:0.333333	
chr28	11796785	2	36	A:1	T:0		
chr28	11796954	2	38	T:1	TATAA:0		
chr28	11796955	4	38	CTAAA:0.315789	C:0.394737	ATAAA:0	CTAAATAAA:0.289474
chr28	11797048	3	32	GAA:0.03125	G:0.6875	GA:0.28125	
chr28	11797348	2	34	C:1	T:0		
chr28	11797612	2	36	C:1	G:0		
chr28	11797870	2	36	TA:0.944444	T:0.0555556		
chr28	11797886	2	34	C:0.735294	CT:0.264706		
chr28	11797924	2	34	C:0.676471	G:0.323529		
chr28	11797926	2	34	C:0.676471	G:0.323529		
chr28	11797930	3	34	AAG:0.470588	A:0.205882	GAG:0.323529	
chr28	11798052	2	34	C:0.617647	T:0.382353		
chr28	11798176	2	36	GA:1	G:0		
chr28	11798392	2	34	T:0.676471	C:0.323529		
chr28	11798395	2	34	C:0.676471	T:0.323529		
chr28	11798517	2	38	AAATG:0.605263	A:0.394737		
chr28	11798537	2	38	GAATGAATA:0.947368	G:0.0526316		
chr28	11798545	2	38	A:1	AATG:0		
chr28	11798566	2	36	A:0.638889	C:0.361111		
chr28	11798574	2	36	A:0.666667	AAG:0.333333		
chr28	11799037	2	38	T:0.657895	C:0.342105		
chr28	11799055	2	36	T:0.388889	G:0.611111		
chr28	11799202	2	28	C:0.857143	G:0.142857		
chr28	11799343	2	40	C:0.75	T:0.25		
chr28	11799356	2	40	C:0.675	T:0.325		

chr28	11799383	2	34	C:0.647059	T:0.352941
chr28	11799462	2	34	A:0.0588235	G:0.941176
chr28	11799673	2	30	C:1	T:0
chr28	11799848	2	38	C:0.394737	T:0.605263
chr28	11799928	2	32	C:0.59375	CCT:0.40625
chr28	11799947	2	32	C:0.78125	G:0.21875
chr28	11800055	2	40	T:0.4	C:0.6
chr28	11800064	2	38	TC:0.421053	T:0.578947
chr28	11800122	2	32	T:0.3125	C:0.6875
chr28	11800129	2	32	T:1	G:0
chr28	11800151	2	36	C:0.75	A:0.25
chr28	11800187	2	36	T:1	C:0
chr28	11800210	2	34	G:1	A:0
chr28	11800343	4	40	CCTCTCTCTCTCT:0.15	C:0.275
CCTCT:0.275	CCTCTCT:0.3
chr28	11800516	2	30	T:0.566667	A:0.433333
chr28	11800593	2	36	A:0.0555556	C:0.944444
chr28	11800604	2	36	AG:0.694444	A:0.305556
chr28	11800720	2	32	A:0.84375	G:0.15625
chr28	11800826	2	36	G:0	A:1
chr28	11800827	2	36	T:0.722222	C:0.277778
chr28	11800915	2	38	T:0.0526316	G:0.947368
chr28	11801101	2	40	T:0.7	A:0.3
chr28	11801371	2	38	G:0.631579	A:0.368421
chr28	11801386	2	38	A:0.605263	G:0.394737
chr28	11801464	2	38	A:1	C:0
chr28	11801555	2	36	G:1	A:0
chr28	11801669	2	38	C:0.473684	T:0.526316
chr28	11801788	2	38	G:1	A:0
chr28	11802005	2	40	G:0.5	C:0.5
chr28	11802011	2	40	G:0.775	A:0.225
chr28	11802016	2	40	T:0.625	C:0.375
chr28	11802098	2	38	C:0.921053	CT:0.0789474
chr28	11802211	2	38	A:0.710526	T:0.289474
chr28	11802315	2	38	GTTTTCTTTCT:0.947368	G:
0.0526316
chr28	11802650	2	38	A:0.447368	C:0.552632
chr28	11802676	2	36	C:0.75	T:0.25
chr28	11802821	2	34	T:0.617647	C:0.382353
chr28	11803773	4	36	CGT:0.138889	C:0.277778
CGTGT:0.472222	CGTGTGTGTGT:0.111111
chr28	11804481	2	38	T:0.105263	C:0.894737
chr28	11804513	2	38	C:1	T:0
chr28	11804562	2	36	C:1	T:0
chr28	11804597	2	34	G:1	A:0
chr28	11804796	2	38	G:1	A:0
chr28	11804985	2	40	G:0.725	A:0.275
chr28	11805043	2	40	C:0.625	G:0.375
chr28	11805140	2	36	AC:0.583333	A:0.416667
chr28	11805141	2	36	C:0.5	A:0.5
chr28	11805286	2	38	A:0.105263	AGAG:0.894737
chr28	11805554	2	40	C:0.45	T:0.55
chr28	11805609	2	38	G:0.605263	T:0.394737
chr28	11805896	2	38	T:0.684211	C:0.315789

chr28	11805899	2	38	A:0.684211	G:0.315789	
chr28	11806045	2	40	G:0.65	T:0.35	
chr28	11806146	2	40	C:0.1	CT:0.9	
chr28	11806176	2	38	CA:0.684211	C:0.315789	
chr28	11806298	2	38	A:0.105263	G:0.894737	
chr28	11806664	2	36	A:0.583333	T:0.416667	
chr28	11807120	2	34	TACC:0.588235	T:0.411765	
chr28	11807136	3	36	CT:0.305556	C:0.416667	CTT:0.277778
chr28	11807147	2	36	A:0.722222	T:0.277778	
chr28	11807307	2	38	CAA:0.5	C:0.5	
chr28	11807387	2	38	G:0.447368	T:0.552632	
chr28	11807555	2	40	A:0.1	T:0.9	
chr28	11807626	2	36	A:0.666667	G:0.333333	
chr28	11807707	2	36	C:0.972222	T:0.0277778	
chr28	11807769	2	36	G:0.833333	A:0.166667	
chr28	11807828	2	38	A:0.631579	G:0.368421	
chr28	11808195	2	38	T:0.710526	C:0.289474	
chr28	11808348	2	36	C:0.611111	T:0.388889	
chr28	11808481	2	34	T:0.676471	C:0.323529	
chr28	11808638	2	34	C:1	T:0	
chr28	11808712	2	34	T:0.764706	C:0.235294	
chr28	11809063	2	40	T:0.125	C:0.875	
chr28	11809072	2	40	T:0.125	C:0.875	
chr28	11809151	2	38	C:0.5	T:0.5	
chr28	11809171	2	38	G:1	A:0	
chr28	11809177	2	38	A:0.105263	G:0.894737	
chr28	11809382	2	38	A:1	G:0	
chr28	11809535	2	40	C:1	T:0	
chr28	11809603	2	40	ATTC:0.825	A:0.175	
chr28	11809604	2	40	TTC:0.775	T:0.225	
chr28	11809605	2	40	TC:0.975	T:0.025	
chr28	11809606	2	40	C:0.8	CT:0.2	
chr28	11809614	2	40	T:1	TA:0	
chr28	11809622	2	40	T:0.5	C:0.5	
chr28	11809676	2	34	G:0.5	A:0.5	
chr28	11809724	2	34	C:0.470588	T:0.529412	
chr28	11809857	2	40	A:0.4	G:0.6	
chr28	11809963	2	38	A:0.526316	G:0.473684	
chr28	11810177	2	38	T:0.657895	A:0.342105	
chr28	11810265	2	40	G:0.65	A:0.35	
chr28	11810439	2	40	G:1	C:0	
chr28	11810719	2	38	T:0.447368	C:0.552632	
chr28	11810788	2	38	T:0.131579	C:0.868421	
chr28	11810820	2	34	C:0	A:1	
chr28	11810822	2	34	T:0	G:1	
chr28	11810869	2	36	C:0.444444	T:0.555556	
chr28	11810984	2	40	C:1	A:0	
chr28	11811150	2	38	G:0.710526	A:0.289474	
chr28	11811157	2	38	G:0.973684	A:0.0263158	
chr28	11811263	2	40	C:0.475	T:0.525	
chr28	11811376	2	38	C:0.736842	T:0.263158	
chr28	11811557	2	40	T:1	G:0	
chr28	11811873	3	38	CTT:0.605263	C:0.184211	

CT:0.210526					
chr28	11811960	2	32	C:0.65625	T:0.34375
chr28	11812022	2	38	G:0.631579	T:0.368421
chr28	11812159	2	36	G:0.944444	A:0.0555556
chr28	11812218	2	38	T:0.105263	A:0.894737
chr28	11812393	2	40	G:0.725	T:0.275
chr28	11812447	2	36	T:1	C:0
chr28	11812641	2	40	G:0.775	C:0.225
chr28	11812831	2	36	G:0.111111	A:0.888889
chr28	11812881	2	38	A:0.368421	AAT:0.631579
chr28	11813079	2	34	G:1	A:0
chr28	11813123	2	34	G:1	A:0
chr28	11813221	2	38	AC:1	A:0
chr28	11813276	2	36	T:0.916667	TA:0.0833333
chr28	11813318	2	38	A:0.105263	G:0.894737
chr28	11813397	2	36	A:1	G:0
chr28	11813433	2	36	C:0.138889	T:0.861111
chr28	11813461	2	32	AAC:1	A:0
chr28	11813649	2	32	C:1	T:0
chr28	11813676	2	36	T:0.111111	TCGAGGTC:0.888889
chr28	11813870	2	38	G:1	GT:0
chr28	11813872	2	38	G:1	GT:0
chr28	11813873	2	38	G:1	GATA:0
chr28	11813877	2	38	G:1	GAAGTGAA:0
chr28	11813879	2	38	ATTC:1	A:0
chr28	11813883	2	38	T:1	G:0
chr28	11813898	2	38	GA:0.0263158	G:0.973684
chr28	11813981	2	34	G:0.117647	A:0.882353
chr28	11813984	2	34	C:1	T:0
chr28	11814013	2	34	T:0	C:1
chr28	11814146	2	38	G:1	GA:0
chr28	11814337	2	32	TCTC:1	T:0
chr28	11814602	2	40	C:1	CA:0
chr28	11814736	2	34	A:1	C:0
chr28	11815053	2	40	A:0.975	G:0.025
chr28	11815772	2	34	T:0.147059	A:0.852941
chr28	11815788	2	36	G:0.0277778	A:0.972222
chr28	11816506	3	38	A:0.631579	AAC:0.0526316
AACACACACAC:0.315789					
chr28	11816632	2	34	T:0.470588	C:0.529412
chr28	11816655	2	34	C:0.470588	T:0.529412
chr28	11816786	2	30	T:0.6	C:0.4
chr28	11816941	2	38	C:1	T:0
chr28	11816942	2	38	A:0.552632	G:0.447368
chr28	11816992	2	38	G:0.552632	C:0.447368
chr28	11816998	2	38	C:0.552632	T:0.447368
chr28	11817018	2	38	A:0.552632	G:0.447368
chr28	11817080	2	34	C:0.588235	T:0.411765
chr28	11817154	2	36	A:0.583333	G:0.416667
chr28	11817388	2	36	A:0.611111	G:0.388889
chr28	11817437	2	36	T:0.527778	A:0.472222
chr28	11817467	2	36	T:0.527778	C:0.472222
chr28	11817485	2	36	C:0.527778	T:0.472222
chr28	11817814	2	34	C:0.5	T:0.5

chr28 11817846 2 38 CTAATT:0.973684 C:0.0263158
chr28 11818123 2 38 C:0.578947 T:0.421053
chr28 11818310 2 32 A:1 G:0
chr28 11818731 2 40 C:0.4 T:0.6
chr28 11819259 2 38 T:0.631579 C:0.368421
chr28 11819408 2 38 A:0.552632 G:0.447368
chr28 11819651 2 36 G:0.444444 A:0.555556
chr28 11819773 2 38 G:0.552632 C:0.447368
chr28 11819835 3 32 CAA:0.0625 C:0.71875
CA:0.21875
chr28 11819852 2 30 A:0.366667 G:0.633333
chr28 11819948 2 38 A:0.552632 G:0.447368
chr28 11819970 2 40
GTTAACTTAAGTTAAGTTAACTTAAGGCTTAAGTTAAGTTA:0 G:1
chr28 11820100 2 36 T:0.888889 C:0.111111
chr28 11820249 2 40 C:0.975 T:0.025
chr28 11820285 3 38 GATAAATAAATAA:0.394737 G:
0.105263 GATAAATAA:0.5
chr28 11820434 2 36 T:0.583333 C:0.416667
chr28 11820503 2 34 C:1 T:0
chr28 11821191 2 38 G:0.921053 GA:0.0789474
chr28 11821468 2 36 T:0.972222 TG:0.0277778
chr28 11822074 2 38 A:0.473684 T:0.526316
chr28 11822106 2 38 C:0.578947 T:0.421053
chr28 11822620 2 38 G:0.815789 T:0.184211
chr28 11823401 2 34 C:0.970588 T:0.0294118
chr28 11823549 2 38 T:0.526316 C:0.473684
chr28 11823682 2 36 G:0.0277778 A:0.972222
chr28 11823745 2 36 A:0.472222 G:0.527778
chr28 11824349 2 38 G:0.921053 C:0.0789474
chr28 11824401 2 40 G:0 A:1
chr28 11824639 2 36 T:0.916667 G:0.0833333
chr28 11824717 2 40 G:0.55 T:0.45
chr28 11825067 2 38 G:0.868421 GC:0.131579
chr28 11825091 2 38 G:0.894737 A:0.105263
chr28 11825265 2 40 G:0.55 A:0.45
chr28 11825271 2 40 T:0.85 C:0.15
chr28 11825284 2 40 C:0.55 A:0.45
chr28 11825581 2 38 A:0.578947 G:0.421053
chr28 11825663 2 34 C:0.382353 A:0.617647
chr28 11825667 2 34 A:0.441176 T:0.558824
chr28 11825919 2 38 T:0.973684 C:0.0263158
chr28 11826023 2 38 G:0.0263158 A:0.973684
chr28 11826264 2 36 C:0.416667 CA:0.583333
chr28 11826297 2 36 C:0.888889 T:0.111111
chr28 11826375 2 40 C:0.325 A:0.675
chr28 11826420 2 36 C:0.0277778 T:0.972222
chr28 11826445 2 36 G:0.472222 C:0.527778
chr28 11826491 2 36 G:0.5 A:0.5
chr28 11826844 2 38 A:0.578947 G:0.421053
chr28 11826905 2 38 C:0.736842 CTAAT:0.263158
chr28 11826973 2 38 T:0.5 C:0.5
chr28 11827081 2 38 CAATAA:0.894737 C:0.105263
chr28 11827150 2 36 A:1 G:0

chr28	11827449	2	40	T:1	C:0
chr28	11827559	2	36	C:0.527778	T:0.472222
chr28	11827649	2	36	G:0.888889	A:0.111111
chr28	11827729	2	40	A:0.9	G:0.1
chr28	11827775	2	40	C:0.5	T:0.5
chr28	11827790	2	40	G:0.85	GAT:0.15
chr28	11827919	2	40	A:0.05	T:0.95
chr28	11827970	2	40	T:0.875	A:0.125
chr28	11827995	2	40	A:0.6	ATTATATG:0.4
chr28	11828110	2	36	C:0.972222	A:0.0277778
chr28	11828111	2	36	G:1	A:0
chr28	11828210	2	38	G:0.789474	A:0.210526
chr28	11828230	2	40	T:0.9	TA:0.1
chr28	11828240	2	40	T:0.925	A:0.075
chr28	11828364	2	38	G:0.631579	A:0.368421
chr28	11828419	2	40	C:0.575	T:0.425
chr28	11828430	2	40	G:0.575	GT:0.425
chr28	11828459	2	40	C:0.525	T:0.475
chr28	11828495	2	38	C:0.0526316	T:0.947368
chr28	11828545	2	40	C:0.65	T:0.35
chr28	11828579	2	36	T:0.611111	C:0.388889
chr28	11828585	2	36	GA:0.638889	G:0.361111
chr28	11828601	2	40	T:0.975	C:0.025
chr28	11828692	2	36	G:0.611111	T:0.388889
chr28	11828724	2	28	A:0.0357143	G:0.964286
chr28	11828765	2	28	G:0.535714	A:0.464286
chr28	11828766	2	28	A:0.178571	G:0.821429
chr28	11830489	2	14	C:0.357143	T:0.642857
chr28	11830516	2	16	ATT:0.3125	A:0.6875
chr28	11830520	2	16	T:0.3125	TAAA:0.6875
chr28	11830549	2	26	TA:0.153846	T:0.846154
chr28	11830623	2	38	G:0.947368	A:0.0526316
chr28	11830972	2	36	TTC:0.5	T:0.5
chr28	11830989	2	38	TC:0.552632	T:0.447368
chr28	11831002	2	36	C:0.5	T:0.5
chr28	11831044	2	38	A:1	G:0
chr28	11831052	2	38	CT:1	C:0
chr28	11831054	2	34	TA:0.529412	T:0.470588
chr28	11831158	2	38	A:0	AG:1
chr28	11831197	2	32	G:0.5625	A:0.4375
chr28	11831230	2	36	ACT:0.5	A:0.5
chr28	11831478	2	38	A:0.526316	G:0.473684
chr28	11831512	2	40	AT:0.025	A:0.975
chr28	11831519	2	40	C:0.575	T:0.425
chr28	11831520	2	40	C:0.55	G:0.45
chr28	11831564	2	40	A:0.625	T:0.375
chr28	11831602	2	36	T:0.972222	A:0.0277778
chr28	11831672	2	38	A:0.552632	G:0.447368
chr28	11831673	2	38	C:0.657895	T:0.342105
chr28	11831689	2	38	ATACC:0.552632	A:0.447368
chr28	11831695	2	38	GGAA:0.552632	G:0.447368
chr28	11831743	2	38	A:0.105263	C:0.894737
chr28	11831811	2	40	T:0.575	C:0.425
chr28	11831902	2	40	C:0.575	T:0.425

chr28 11831994 2 38 G:1 T:0
chr28 11832056 2 38 G:0.973684 A:0.0263158
chr28 11832120 2 38 G:0.552632 A:0.447368
chr28 11832161 2 40 C:0.625 T:0.375
chr28 11832260 2 40 G:0.6 A:0.4
chr28 11832317 2 38 G:0.657895 GA:0.342105
chr28 11832396 2 34 A:1 G:0
chr28 11832428 2 34 A:0.588235 G:0.411765
chr28 11832479 2 36 C:0.555556 A:0.444444
chr28 11832562 2 38 G:0.605263 A:0.394737
chr28 11832586 2 40 T:0.625 C:0.375
chr28 11832644 2 36 T:0.611111 C:0.388889
chr28 11832742 5 38 ATTT:0.0789474 A:0.0526316
AT:0.447368 ATT:0 ATTTT:0.421053
chr28 11832909 2 40 A:0.525 G:0.475
chr28 11832947 2 38 T:1 TA:0
chr28 11832948 2 38 A:1 T:0
chr28 11832949 2 38 G:0.473684 A:0.526316
chr28 11833158 2 40 T:0.975
TTAAAACGTTTAAAAAAAAATAAAAAAAA:0.025
chr28 11833277 2 36 C:0.666667 T:0.333333
chr28 11833284 2 34 T:1 C:0
chr28 11833337 2 34 G:0.617647 A:0.382353
chr28 11833421 2 32 G:0.875 T:0.125
chr28 11833456 2 34 CA:0 C:1
chr28 11833459 2 34 C:0 G:1
chr28 11833491 2 36 G:0.0555556 GAA:0.944444
chr28 11833492 2 36 T:0.0555556 TTAG:0.944444
chr28 11833560 2 40 ATGTGAATCT:0.95 A:0.05
chr28 11833746 2 34 C:0.617647 T:0.382353
chr28 11833798 2 38 T:1 C:0
chr28 11833836 2 36 T:1 C:0
chr28 11834109 2 36 C:0.833333 T:0.166667
chr28 11834218 2 40 C:0.625 CCAAA:0.375
chr28 11834221 2 40 C:0.625 T:0.375
chr28 11834404 2 34 A:0.588235 G:0.411765
chr28 11834520 2 36 G:0.0555556 A:0.944444
chr28 11834541 2 38 G:0.0789474 GT:0.921053
chr28 11834686 2 38 G:0.526316 A:0.473684
chr28 11834708 2 38 G:0.973684 A:0.0263158
chr28 11834721 2 36 C:0.0555556 T:0.944444
chr28 11834734 2 36 C:0.0555556 T:0.944444
chr28 11834783 2 32 G:0.53125 A:0.46875
chr28 11834854 2 38 A:1 G:0
chr28 11835063 2 40 G:0.025 A:0.975
chr28 11835117 2 38 C:0.868421 CA:0.131579
chr28 11835326 2 38 G:0.0263158 A:0.973684
chr28 11835337 3 40 T:0.575 TAAAAATGGGAAGGTAAAAA:
0.275 TAAAAATGGGAAGGTAAAAAA:0.15
chr28 11835457 2 38 G:0.868421 T:0.131579
chr28 11835484 2 36 T:0 A:1
chr28 11835514 2 36 C:0.805556 CA:0.194444
chr28 11835610 2 40 T:0.025 C:0.975
chr28 11835757 2 34 C:1 T:0

chr28	11835894	2	38	C:0.0789474	T:0.921053
chr28	11835919	2	38	A:0.0789474	G:0.921053
chr28	11835996	2	40	T:0.45	A:0.55
chr28	11836065	2	40	T:0.975	C:0.025
chr28	11836313	2	38	A:1	ATGAGAAAATATTTTTTTTTTTT:0
chr28	11836403	2	36	C:0.972222	T:0.0277778
chr28	11836409	2	36	A:0.527778	G:0.472222
chr28	11836574	2	40	T:0.95	G:0.05
chr28	11836609	2	38	C:0.578947	A:0.421053
chr28	11836733	2	38	C:0.0789474	T:0.921053
chr28	11836800	2	40	T:0.125	C:0.875
chr28	11836862	2	40	G:0.125	A:0.875
chr28	11837006	2	40	A:0.1	C:0.9
chr28	11837042	2	38	A:0.605263	T:0.394737
chr28	11837067	2	38	C:0.605263	G:0.394737
chr28	11837127	2	40	A:0.125	C:0.875
chr28	11837136	2	38	C:0.947368	A:0.0526316
chr28	11837245	2	40	T:1	C:0
chr28	11837546	2	38	T:0.578947	C:0.421053
chr28	11837672	2	36	G:0.583333	T:0.416667
chr28	11837742	2	36	A:0.611111	G:0.388889
chr28	11837794	2	34	T:0.647059	A:0.352941
chr28	11837849	2	38	A:0.105263	G:0.894737
chr28	11837878	2	36	T:1	TA:0
chr28	11837879	2	36	T:0.472222	A:0.527778
chr28	11838120	2	36	G:0.583333	A:0.416667
chr28	11838205	2	40	TAATG:0.6	T:0.4
chr28	11838398	2	40	T:0.6	A:0.4
chr28	11838570	2	36	A:0.0555556	G:0.944444
chr28	11838777	2	38	G:0.105263	A:0.894737
chr28	11838809	2	36	ATC:0.972222	A:0.0277778
chr28	11838940	2	36	A:0.111111	G:0.888889
chr28	11839043	2	38	G:0.0789474	T:0.921053
chr28	11839070	2	34	A:0.117647	G:0.882353
chr28	11839158	2	34	C:0.117647	T:0.882353
chr28	11839209	2	40	T:0.1	TAAGA:0.9
chr28	11839356	2	36	G:0.111111	GA:0.888889
chr28	11839467	2	40	G:0.1	C:0.9
chr28	11840040	2	36	T:0.555556	C:0.444444
chr28	11840100	2	38	C:1	A:0
chr28	11840264	2	38	G:0.526316	A:0.473684
chr28	11840358	2	36	T:0.166667	G:0.833333
chr28	11840359	2	36	A:0.166667	G:0.833333
chr28	11840463	2	40	G:0.8	C:0.2
chr28	11841017	2	38	AG:0.605263	A:0.394737
chr28	11841310	2	38	T:0.0263158	C:0.973684
chr28	11841644	2	36	C:0.916667	T:0.0833333
chr28	11841865	2	36	T:0.611111	C:0.388889
chr28	11841892	2	40	A:0.9	G:0.1
chr28	11841941	2	40	G:1	A:0
chr28	11841996	2	40	C:0.025	T:0.975
chr28	11842065	2	36	T:0.0277778	TTATATC:0.972222
chr28	11842081	2	34	A:1	G:0
chr28	11842281	2	38	TA:0.368421	T:0.631579

chr28	11842318	2	40	C:0.025	A:0.975		
chr28	11842384	2	36	T:1	G:0		
chr28	11842436	2	40	A:0	G:1		
chr28	11842615	2	40	T:0.025	C:0.975		
chr28	11842801	2	38	C:0.552632	A:0.447368		
chr28	11842817	2	38	G:1	A:0		
chr28	11842837	2	38	A:1	G:0		
chr28	11842919	2	40	A:0.025	C:0.975		
chr28	11842933	2	40	T:1	A:0		
chr28	11843043	2	38	A:0.473684	G:0.526316		
chr28	11843103	2	38	G:0	GA:1		
chr28	11843108	2	38	AT:0	A:1		
chr28	11843111	2	38	G:0	A:1		
chr28	11843114	2	38	AC:0	A:1		
chr28	11843120	2	38	G:0	A:1		
chr28	11843121	2	38	A:0	G:1		
chr28	11843135	2	38	G:0	T:1		
chr28	11843136	2	38	A:0	G:1		
chr28	11843151	2	38	C:0	A:1		
chr28	11843152	2	38	G:0	C:1		
chr28	11843216	2	36	GC:0	G:1		
chr28	11843431	3	34	G:0.794118	GA:0	GAA:0.205882	
chr28	11843435	2	34	A:0.676471	G:0.323529		
chr28	11843497	2	40	C:0.525	T:0.475		
chr28	11843521	2	40	A:0.75	G:0.25		
chr28	11843596	2	40	C:0.7	T:0.3		
chr28	11843803	2	32	T:0.6875	C:0.3125		
chr28	11843819	4	34	CCTCTCTCTCTCTCTCTCT:0.117647	C:0.0882353	CCTCT:0.382353	CCTCTCTCT:0.411765
chr28	11843936	2	36	G:1	A:0		
chr28	11844118	2	4	C:0	T:1		
chr28	11844333	2	36	C:0.0277778	A:0.972222		
chr28	11844361	2	32	AT:0.71875	A:0.28125		
chr28	11844447	2	36	T:0.75	G:0.25		
chr28	11844576	2	38	C:0.894737	CAAAA:0.105263		
chr28	11845066	2	30	C:0.433333	T:0.566667		
chr28	11845187	2	22	CA:0.454545	C:0.545455		
chr28	11845202	2	22	C:0.454545	A:0.545455		
chr28	11845333	2	36	A:1	G:0		
chr28	11845820	2	8	T:1	A:0		
chr28	11845839	2	8	A:0	G:1		
chr28	11845849	2	14	T:1	G:0		
chr28	11845940	2	38	C:0.447368	T:0.552632		
chr28	11846050	2	14	A:0	G:1		
chr28	11846163	2	34	T:0.794118	G:0.205882		
chr28	11846225	2	36	G:0.583333	A:0.416667		
chr28	11846457	2	36	T:0.0555556	G:0.944444		
chr28	11846915	2	30	GT:1	G:0		
chr28	11846968	2	38	G:0.421053	A:0.578947		
chr28	11847408	2	38	G:0	A:1		
chr28	11847412	2	38	C:0.552632	T:0.447368		
chr28	11847489	2	34	A:0.529412	G:0.470588		
chr28	11847696	2	38	G:0.473684	A:0.526316		

chr28	11847823	2	34	T:0.558824	G:0.441176	
chr28	11848129	2	34	T:0.735294	C:0.264706	
chr28	11848274	2	40	A:1	G:0	
chr28	11848503	2	38	TTTTC:0.578947	T:0.421053	
chr28	11848581	2	34	A:1	C:0	
chr28	11849481	2	38	C:0.789474	T:0.210526	
chr28	11849660	2	38	A:0.921053	T:0.0789474	
chr28	11849668	2	40	A:0.75	G:0.25	
chr28	11849831	2	30	A:0.466667	G:0.533333	
chr28	11850180	2	36	T:0.166667	TA:0.833333	
chr28	11850231	2	40	T:0.025	G:0.975	
chr28	11850436	2	36	T:1	TA:0	
chr28	11850510	2	38	C:0.815789	G:0.184211	
chr28	11850866	2	38	G:0.921053	T:0.0789474	
chr28	11851134	2	38	G:1	A:0	
chr28	11851149	2	34	CT:0.470588	C:0.529412	
chr28	11851158	2	34	T:0.764706	A:0.235294	
chr28	11851160	2	34	A:0.558824	AT:0.441176	
chr28	11851192	2	32	A:0.65625	AAG:0.34375	
chr28	11851261	2	30	C:1	T:0	
chr28	11851340	2	40	C:0.875	T:0.125	
chr28	11851374	2	38	CA:0.973684	C:0.0263158	
chr28	11851486	2	34	G:0.970588	A:0.0294118	
chr28	11851525	2	34	T:0	C:1	
chr28	11851613	2	40	C:1	T:0	
chr28	11851725	2	34	T:0.676471	C:0.323529	
chr28	11851797	2	38	T:0.921053	G:0.0789474	
chr28	11852005	2	36	A:0.805556	T:0.194444	
chr28	11852018	2	36	A:0.333333	G:0.666667	
chr28	11852216	2	36	C:0.888889	T:0.111111	
chr28	11852271	2	34	C:0.558824	A:0.441176	
chr28	11852326	2	36	A:0.0277778	G:0.972222	
chr28	11852709	2	40	T:0.3	C:0.7	
chr28	11852752	2	40	A:0.875	G:0.125	
chr28	11852785	2	40	T:0.275	A:0.725	
chr28	11853025	3	38	CAA:0	C:0.263158	CA: 0.736842
chr28	11853136	2	34	C:0.735294	T:0.264706	
chr28	11853296	2	36	C:0.416667	A:0.583333	
chr28	11853440	2	40	T:0.025	C:0.975	
chr28	11853526	2	40	A:0.975	G:0.025	
chr28	11853567	2	40	G:1	A:0	
chr28	11853933	2	38	A:0.552632	G:0.447368	
chr28	11854146	2	34	T:0.617647	G:0.382353	
chr28	11854209	2	38	G:0.5	A:0.5	
chr28	11854354	2	36	A:0.555556	G:0.444444	
chr28	11854391	2	36	G:0.944444	C:0.0555556	
chr28	11854476	2	32	TA:0.4375	T:0.5625	
chr28	11854506	2	36	C:0.5	T:0.5	
chr28	11854642	2	36	A:0.0555556	T:0.944444	
chr28	11854673	2	32	T:0.5625	A:0.4375	
chr28	11854676	2	30	A:0.5	T:0.5	
chr28	11854708	2	34	C:0.941176	A:0.0588235	
chr28	11855104	2	38	C:0.710526	T:0.289474	

chr28	11855240	2	40	TA:0.525	T:0.475	
chr28	11855243	2	40	CTCTATTTGTATATTTTGACAATCTATTT:0.525	C:0.475	
chr28	11855485	2	38	TAA:0.815789	T:0.184211	
chr28	11855489	2	38	AAAAAATAAAATAAAAT:0.868421	A:0.131579	
chr28	11855491	2	40	A:0.425	AT:0.575	
chr28	11855492	2	40	AAAT:0.975	A:0.025	
chr28	11855495	3	40	TAAAATAAAATAAAA:0.25	T:0.175	TAAATAAAATAAAA:0.575
chr28	11855500	3	40	TA:0.7	TAAA:0.025	T:0.275
chr28	11855501	2	40	A:0.775	AAAT:0.225	
chr28	11855509	2	40	A:0.425	T:0.575	
chr28	11855989	2	34	A:1	G:0	
chr28	11856291	2	36	G:0.611111	A:0.388889	
chr28	11856359	2	36	C:0.444444	A:0.555556	
chr28	11856589	2	36	T:1	C:0	
chr28	11856689	2	38	G:0.473684	A:0.526316	
chr28	11856771	2	36	A:0.555556	C:0.444444	
chr28	11856801	2	36	C:1	T:0	
chr28	11856828	2	36	C:1	T:0	
chr28	11856850	2	36	G:0.972222	T:0.0277778	
chr28	11856984	2	38	C:0.710526	T:0.289474	
chr28	11857174	2	30	GA:0.0333333	G:0.966667	
chr28	11857197	2	32	G:1	A:0	
chr28	11857306	2	38	T:0	A:1	
chr28	11857441	2	40	C:0.05	T:0.95	
chr28	11857656	2	38	G:0.736842	A:0.263158	
chr28	11857712	2	40	G:0.55	A:0.45	
chr28	11857887	2	38	G:0.736842	T:0.263158	
chr28	11857896	2	38	C:0.710526	T:0.289474	
chr28	11858000	2	38	A:0.0526316	C:0.947368	
chr28	11858084	2	38	A:0.710526	G:0.289474	
chr28	11858194	2	38	G:0.710526	T:0.289474	
chr28	11858610	2	40	T:0.3	C:0.7	
chr28	11858912	2	40	C:1	T:0	
chr28	11859084	2	38	C:1	A:0	
chr28	11859194	2	36	C:0.805556	T:0.194444	
chr28	11859376	2	40	C:0.7	T:0.3	
chr28	11859705	2	40	ATTT:0.325	A:0.675	
chr28	11859712	2	40	C:0.95	T:0.05	
chr28	11859833	2	36	T:0.25	TA:0.75	
chr28	11859960	2	32	A:0.6875	T:0.3125	
chr28	11860041	2	38	T:0.447368	C:0.552632	
chr28	11860201	2	36	CAT:0.833333	C:0.166667	
chr28	11860909	2	34	C:0.470588	A:0.529412	
chr28	11861144	2	38	G:0.763158	GTAAAA:0.236842	
chr28	11861147	2	38	G:0.763158	GA:0.236842	
chr28	11861148	2	38	T:0.763158	TAATCTTTTATCTAGG:0.236842	
chr28	11861292	2	34	T:1	C:0	
chr28	11861299	2	34	C:1	CT:0	
chr28	11861520	2	38	C:0.789474	G:0.210526	
chr28	11862021	2	36	C:0.0555556	T:0.944444	

chr28	11862382	2	36	A:0.777778	G:0.222222			
chr28	11862419	2	26	G:0.692308	T:0.307692			
chr28	11862432	2	24	A:0.666667	C:0.333333			
chr28	11862474	2	30	A:0.633333	G:0.366667			
chr28	11862665	2	34	GA:1	G:0			
chr28	11862708	2	32	TG:0.75	T:0.25			
chr28	11862852	2	40	A:0.025	G:0.975			
chr28	11863064	2	38	G:0.763158	C:0.236842			
chr28	11863087	2	40	G:0	A:1			
chr28	11863317	2	36	TCAA:0.75	T:0.25			
chr28	11863409	5	40	AAG:0.2	A:0.25	AAGAGAG:0.15	AAGAGAGAG:0.35	AAGAGAGAGAG:0.05
chr28	11863543	2	38	T:0.0263158	G:0.973684			
chr28	11863601	2	40	C:0.775	A:0.225			
chr28	11863735	2	32	C:1	T:0			
chr28	11863841	2	38	C:0.473684	T:0.526316			
chr28	11863912	2	38	T:0.526316	C:0.473684			
chr28	11864152	2	40	A:0.525	G:0.475			
chr28	11864492	2	40	A:1	G:0			
chr28	11864653	2	38	A:0.815789	T:0.184211			
chr28	11864859	2	38	C:0.0263158	CCT:0.973684			
chr28	11865229	2	38	T:0.526316	C:0.473684			
chr28	11865376	2	38	C:0.526316	G:0.473684			
chr28	11865467	2	26	TAGA:0.807692	T:0.192308			
chr28	11865607	4	40	C:0.225	CTGTGTG:0	CTGTGTGTGTG:0.325	CTGTGTGTGTGTGTG:0.45	
chr28	11865639	2	38	C:0.526316	G:0.473684			
chr28	11865677	2	40	A:0.725	AAATG:0.275			
chr28	11865679	2	34	ATAAATAAG:0.352941	A:0.647059			
chr28	11865687	2	40	G:0.725	A:0.275			
chr28	11865692	3	40	CAAAT:0.725	C:0	TAAAT:0.275		
chr28	11865704	4	40	T:0.725	TAAATA:0	TAAATAAATA:0.275	TAAATAAATAAATA:0	
chr28	11865794	2	34	C:0.970588	T:0.0294118			
chr28	11866188	2	38	T:1	C:0			
chr28	11866467	2	38	G:0.0263158	A:0.973684			
chr28	11866651	2	38	C:0.0263158	G:0.973684			
chr28	11866749	2	32	G:0.125	GA:0.875			
chr28	11866946	2	30	ATTAAT:0.966667	A:0.0333333			
chr28	11866955	2	34	A:0.764706	ATTTAT:0.235294			
chr28	11867008	2	24	C:0.833333	T:0.166667			
chr28	11867265	2	38	C:0.973684	A:0.0263158			
chr28	11867606	2	40	C:0.725	T:0.275			
chr28	11867663	2	38	A:1	C:0			
chr28	11867677	2	38	C:1	T:0			
chr28	11868484	2	38	ATATACT:0.921053	A:0.0789474			
chr28	11868566	2	38	T:0.789474	C:0.210526			
chr28	11868609	2	40	G:1	A:0			
chr28	11868894	2	36	T:0.666667	TG:0.333333			
chr28	11868900	2	36	ATC:0.666667	A:0.333333			
chr28	11868905	2	36	T:0.666667	TCAGACCATGAGGTATATCAAATTAGATGTGGCA:0.333333			

chr28 11869415 2 38 G:1 A:0
chr28 11869471 2 38 C:0.578947 T:0.421053
chr28 11869604 2 38 C:0.789474 G:0.210526
chr28 11869666 2 38 A:0.5 AT:0.5
chr28 11869801 2 38 CT:0.0263158 C:0.973684
chr28 11869826 2 40 C:0.975 T:0.025
chr28 11869857 2 38 A:0.526316 C:0.473684
chr28 11870017 2 40 A:0.55 AT:0.45
chr28 11870084 2 36 T:0.527778 C:0.472222
chr28 11870558 2 34 CA:0.0882353 C:0.911765
chr28 11870749 2 36 T:0.638889 TC:0.361111
chr28 11870751 2 36 C:0.555556 CG:0.444444
chr28 11870758 4 36 C:0.472222 CAA:0.0277778
CCCAAA:0.305556 CCCCAAA:0.194444
chr28 11870817 2 34 G:0.529412 A:0.470588
chr28 11870859 2 34 G:1 A:0
chr28 11871195 2 38 G:0.973684 A:0.0263158
chr28 11871288 2 40 A:0.975 G:0.025
chr28 11871939 2 36 C:0.527778 CAAAA:0.472222
chr28 11871970 2 36 G:0.416667 A:0.583333
chr28 11872004 2 38 G:0.0263158 T:0.973684
chr28 11872102 3 38 GTT:0.289474 G:0.210526
GTTT:0.5
chr28 11872114 2 36 T:0.75 A:0.25
chr28 11872115 3 38 T:0.5 A:0.289474 TAAA:
0.210526
chr28 11872116 2 38 A:0.5 T:0.5
chr28 11872293 4 32 GTT:0.09375 G:0.375 GT:0
GTTT:0.53125
chr28 11872329 2 36 C:0.555556 G:0.444444
chr28 11872333 2 36 C:1 T:0
chr28 11872600 2 34 G:0.558824 GA:0.441176
chr28 11873067 2 36 A:0.444444 AGT:0.555556
chr28 11873070 4 38 A:0.0526316 ATAG:0.236842
ATAGTATAGTATAG:0.342105 ATATAGTATAGTATAGTATAG:0.368421
chr28 11873397 2 40 T:0.025 C:0.975
chr28 11873413 2 40 C:0.8 T:0.2
chr28 11873422 2 40 T:0.025 C:0.975
chr28 11873476 2 38 G:0.973684 A:0.0263158
chr28 11873785 2 38 A:1 G:0
chr28 11873991 2 36 T:0.611111 A:0.388889
chr28 11874303 2 36 A:1 C:0
chr28 11874403 2 34 T:0.0588235 TC:0.941176
chr28 11874613 2 36 C:0.472222 T:0.527778
chr28 11874662 2 38 C:0.0526316 G:0.947368
chr28 11874841 2 38 G:0.526316 A:0.473684
chr28 11874853 2 38 T:1 C:0
chr28 11875008 2 40 CA:1 C:0
chr28 11875013 2 40 A:0.6 AAC:0.4
chr28 11875034 2 40 AC:0.85 A:0.15
chr28 11875039 2 40 AC:0.85 A:0.15
chr28 11875040 3 40 C:1 A:0 CAAA:0
chr28 11875046 2 40 ACC:0.6 A:0.4
chr28 11875047 2 40 C:0.525 A:0.475

chr28	11875048	3	40	C:0.8	CAAA:0.2	CAAAAAAAAA:0
chr28	11875329	2	38	C:0.5	CT:0.5	
chr28	11875353	2	38	G:0.0263158	C:0.973684	
chr28	11875472	2	36	G:0.5	A:0.5	
chr28	11875746	2	38	G:1	A:0	
chr28	11875928	2	38	A:0.473684	G:0.526316	
chr28	11876735	2	40	C:0.475	T:0.525	
chr28	11876758	2	36	A:0.722222	AT:0.277778	
chr28	11876893	2	40	A:0.5	AT:0.5	
chr28	11877045	2	36	T:0.5	C:0.5	
chr28	11877356	2	38	T:0.5	C:0.5	
chr28	11877566	2	36	C:0.972222	T:0.0277778	
chr28	11877714	2	36	A:1	G:0	
chr28	11877788	2	36	AT:0.5	A:0.5	
chr28	11877919	2	34	G:1	A:0	
chr28	11878099	2	40	C:0.525	T:0.475	
chr28	11878140	2	40	T:0.975	C:0.025	
chr28	11878157	2	40	A:0.025	G:0.975	
chr28	11878380	2	34	C:0.558824	T:0.441176	
chr28	11878898	2	36	TC:0.555556	T:0.444444	
chr28	11878899	2	36	C:1	T:0	
chr28	11878981	2	34	A:0.970588	G:0.0294118	
chr28	11879372	2	36	G:0.611111	A:0.388889	
chr28	11879468	2	36	T:0	C:1	
chr28	11879520	2	34	C:0	T:1	
chr28	11879527	2	34	C:0	T:1	
chr28	11879532	2	34	C:0	T:1	
chr28	11879539	2	34	TG:0	T:1	
chr28	11879542	2	34	G:0	GT:1	
chr28	11879590	2	30	G:0	T:1	
chr28	11879696	2	32	A:1	G:0	
chr28	11879839	2	38	TA:0.710526	T:0.289474	
chr28	11879919	2	40	C:1	T:0	
chr28	11880140	2	38	T:0.5	G:0.5	
chr28	11880412	2	38	AG:0	A:1	
chr28	11880549	2	32	AT:0.96875	A:0.03125	
chr28	11880555	2	32	T:0.5625	G:0.4375	
chr28	11881779	2	34	G:0.5	A:0.5	
chr28	11881837	2	36	C:0.444444	T:0.555556	
chr28	11882344	2	38	G:0.526316	T:0.473684	
chr28	11882615	2	36	T:0.416667	C:0.583333	
chr28	11883074	2	38	G:0.973684	C:0.0263158	
chr28	11883409	2	38	G:0.5	A:0.5	
chr28	11883866	2	38	G:1	T:0	
chr28	11883981	2	40	T:0.45	A:0.55	
chr28	11884006	2	38	G:1	T:0	
chr28	11884147	2	38	G:0.447368	A:0.552632	
chr28	11884208	2	36	C:0.972222	T:0.0277778	
chr28	11884563	2	36	A:0.333333	G:0.666667	
chr28	11885008	2	38	G:0.5	A:0.5	
chr28	11885048	2	38	C:1	T:0	
chr28	11885990	2	36	C:0.5	T:0.5	
chr28	11886152	2	26	GAT:0.692308	G:0.307692	
chr28	11886155	2	26	C:0.692308	CA:0.307692	

chr28	11886315	2	34	ATTT:1	A:0	
chr28	11886318	3	34	T:0.705882	A:0.294118	TAAAAAAAAAAAAA:0
chr28	11886414	2	36	G:0.472222	A:0.527778	
chr28	11886615	2	40	G:0.3	T:0.7	
chr28	11887045	2	36	T:0.555556	TA:0.444444	
chr28	11887409	2	36	T:0.638889	C:0.361111	
chr28	11887582	2	38	G:0.605263	A:0.394737	
chr28	11887973	2	40	C:1	T:0	
chr28	11888154	2	36	C:0.472222	T:0.527778	
chr28	11888210	2	36	G:1	A:0	
chr28	11888556	2	36	C:0.666667	G:0.333333	
chr28	11888695	2	38	C:0.973684	A:0.0263158	
chr28	11889398	2	34	C:0.588235	T:0.411765	
chr28	11889609	2	38	G:1	A:0	
chr28	11890634	2	38	G:0.973684	T:0.0263158	
chr28	11890884	2	38	A:1	G:0	
chr28	11891477	2	40	C:0.525	G:0.475	
chr28	11891523	2	40	A:0.975	G:0.025	
chr28	11891537	2	38	C:1	T:0	
chr28	11892124	2	40	G:1	C:0	
chr28	11892190	2	40	C:0.75	T:0.25	
chr28	11892692	2	40	AG:1	A:0	
chr28	11892694	2	40	C:1	T:0	
chr28	11893064	2	38	A:1	G:0	
chr28	11893084	2	38	G:0.973684	C:0.0263158	
chr28	11893095	2	38	C:1	A:0	
chr28	11893182	2	34	GA:0.441176	G:0.558824	
chr28	11893201	2	36	G:0.472222	A:0.527778	
chr28	11893257	2	34	A:0.5	G:0.5	
chr28	11893458	2	36	G:0.5	C:0.5	
chr28	11893603	2	36	C:0.916667	T:0.0833333	
chr28	11894628	2	36	C:0.472222	T:0.527778	
chr28	11895309	2	36	T:0.305556	C:0.694444	
chr28	11896364	2	40	C:1	T:0	
chr28	11897117	2	40	TCCTAAGC:0.675	T:0.325	
chr28	11897146	2	40	A:0.8	G:0.2	
chr28	11897231	2	36	T:0.388889	G:0.611111	
chr28	11897412	2	36	AT:1	A:0	
chr28	11897471	2	38	C:0.736842	T:0.263158	
chr28	11897491	2	38	A:0.736842	C:0.263158	
chr28	11897718	2	32	G:0.78125	A:0.21875	
chr28	11897838	2	34	A:0	T:1	
chr28	11898077	2	30	C:0.666667	T:0.333333	
chr28	11898115	2	32	C:0.5625	T:0.4375	
chr28	11898121	2	36	AATATATATATATATATAT:0.194444	A:0.805556	
chr28	11898593	2	30	ATG:0.733333	A:0.266667	
chr28	11898597	3	32	G:0.5625	GTA:0.15625	GTATA:0.28125
chr28	11898767	2	36	G:1	A:0	
chr28	11898859	2	36	G:0.75	A:0.25	
chr28	11899659	2	36	G:1	A:0	
chr28	11899683	2	34	A:0.941176	G:0.0588235	

chr28	11899909	2	38	A:1	T:0
chr28	11900076	2	38	G:0.710526	A:0.289474
chr28	11900354	2	30	A:1	T:0
chr28	11900702	2	38	C:0.763158	CT:0.236842
chr28	11900739	2	36	CT:1	C:0
chr28	11900952	4	36	GAAA:0.222222	G:0.222222
GAA:0.305556	GAAAAA:0.25
chr28	11900971	2	36	A:0.777778	T:0.222222
chr28	11901038	2	38	T:0.552632	G:0.447368
chr28	11901103	4	34	C:0.705882	CCT:0.0294118
CCTCT:0.205882	CCTCTCT:0.0588235
chr28	11901139	2	34	AAAT:0.705882	A:0.294118
chr28	11901142	2	34	T:0.911765	TA:0.0882353
chr28	11901145	2	34	A:0.970588	ATT:0.0294118
chr28	11901490	2	36	G:1	C:0
chr28	11901529	2	36	G:1	A:0
chr28	11901609	2	38	G:0.552632	A:0.447368
chr28	11901648	2	36	T:0.138889	C:0.861111
chr28	11901796	2	38	C:0.605263	T:0.394737
chr28	11901801	2	38	A:0.236842	T:0.763158
chr28	11902561	2	34	G:0.764706	A:0.235294
chr28	11902834	2	38	A:0.289474	G:0.710526
chr28	11902875	2	40	T:0.975	TTTTCTTTC:0.025
chr28	11903577	2	32	G:0.78125	A:0.21875
chr28	11904397	2	38	A:0.736842	G:0.263158
chr28	11904411	2	40	G:0.975	GA:0.025
chr28	11904423	2	40	T:0.975	TG:0.025
chr28	11904429	2	36	A:0.222222	G:0.777778
chr28	11904966	2	36	T:0.972222	G:0.0277778
chr28	11905046	2	40	A:0.975	G:0.025
chr28	11905088	2	36	A:0.722222	AT:0.277778
chr28	11905201	2	34	C:0.294118	G:0.705882
chr28	11905204	2	34	C:0.794118	T:0.205882
chr28	11905287	2	40	T:0.225	A:0.775
chr28	11905538	2	34	G:0.323529	A:0.676471
chr28	11905562	2	36	C:0.972222	A:0.0277778
chr28	11905567	2	36	G:0.972222	A:0.0277778
chr28	11905695	2	38	T:0.973684	A:0.0263158
chr28	11905737	2	38	GA:1	G:0
chr28	11905896	2	36	AAC:0.944444	A:0.0555556
chr28	11905908	2	38	C:0.315789	A:0.684211
chr28	11906048	2	38	G:0.973684	C:0.0263158
chr28	11906064	2	36	T:0.25	G:0.75
chr28	11906235	2	32	C:0.96875	A:0.03125
chr28	11906252	2	36	A:0.972222	T:0.0277778
chr28	11906403	2	38	A:0.947368	G:0.0526316
chr28	11906470	2	40	A:0.95	G:0.05
chr28	11906582	2	40	G:0.8	C:0.2
chr28	11906587	2	38	A:0.894737	G:0.105263
chr28	11906709	2	40	A:0.95	G:0.05
chr28	11906993	2	38	T:0.947368	C:0.0526316
chr28	11907074	2	36	T:0.944444	TC:0.0555556
chr28	11907188	2	38	C:1	T:0
chr28	11907338	2	34	AC:0.941176	A:0.0588235

chr28	11907340	2	34	A:0.941176	T:0.0588235	
chr28	11907516	2	38	T:0.973684	C:0.0263158	
chr28	11907594	2	34	G:0.323529	A:0.676471	
chr28	11907854	2	36	GAAC:0.25	G:0.75	
chr28	11908150	2	38	T:1	A:0	
chr28	11908333	2	38	A:0.710526	G:0.289474	
chr28	11908483	2	38	A:0.342105	C:0.657895	
chr28	11908607	2	38	A:0.526316	G:0.473684	
chr28	11908661	2	36	CT:1	C:0	
chr28	11908670	2	36	TA:0.777778	T:0.222222	
chr28	11908671	2	36	A:0.972222	T:0.0277778	
chr28	11908792	2	32	C:0.5	T:0.5	
chr28	11908821	2	38	C:0.605263	G:0.394737	
chr28	11908827	2	38	C:0.605263	T:0.394737	
chr28	11909091	2	30	C:0.466667	T:0.533333	
chr28	11909359	2	38	G:0.710526	GTGAA:0.289474	
chr28	11909966	2	40	TGA:0.725	T:0.275	
chr28	11910739	2	40	C:0.75	CAAATATAAAAAAATATAAAAAAA:0.25	
chr28	11910742	2	40	C:0.75	CAAAAAAT:0.25	
chr28	11910910	2	36	G:0.722222	C:0.277778	
chr28	11911220	2	40	CTG:0.975	C:0.025	
chr28	11911433	2	38	G:0.710526	T:0.289474	
chr28	11911657	2	36	C:1	T:0	
chr28	11912001	3	36	CTT:0.805556	C:0.0833333	CT:0.111111
chr28	11912002	2	38	T:0.473684	TC:0.526316	
chr28	11912028	2	38	C:0.684211	T:0.315789	
chr28	11912031	2	38	T:0.684211	C:0.315789	
chr28	11912299	2	40	C:1	T:0	
chr28	11912379	2	40	G:1	GTC:0	
chr28	11912395	2	40	C:1	G:0	
chr28	11912418	2	40	TAAA:1	T:0	
chr28	11912629	2	40	A:0.3	G:0.7	
chr28	11912867	2	40	T:1	C:0	
chr28	11912928	2	36	A:0.305556	C:0.694444	
chr28	11912964	2	36	T:0	TTGA:1	
chr28	11913368	2	32	T:0.6875	A:0.3125	
chr28	11913502	2	40	T:0.725	A:0.275	
chr28	11913526	2	40	C:0.7	T:0.3	
chr28	11913869	2	34	C:0.235294	T:0.764706	
chr28	11914247	2	32	A:0.71875	T:0.28125	
chr28	11914547	2	32	CTTT:0.875	C:0.125	
chr28	11914566	3	32	TAAG:0.625	T:0.25	AAAG:0.125
chr28	11914575	2	28	T:0.642857	C:0.357143	
chr28	11914582	2	28	T:0.642857	TAGAC:0.357143	
chr28	11914621	2	34	C:0.411765	G:0.588235	
chr28	11914627	2	34	T:0.411765	C:0.588235	
chr28	11914642	2	34	C:0.411765	G:0.588235	
chr28	11914711	2	36	C:0.75	T:0.25	
chr28	11915125	2	36	G:0.555556	A:0.444444	
chr28	11915222	2	40	CTCTTACTCTTAGACCT:0.675	C:0.325	
chr28	11915267	2	38	T:0.657895	A:0.342105	

chr28	11915465	2	40	A:0.6	G:0.4	
chr28	11915522	2	34	G:1	T:0	
chr28	11915639	2	38	G:0.526316	A:0.473684	
chr28	11915812	2	40	G:0.675	A:0.325	
chr28	11915856	2	40	T:1	A:0	
chr28	11916054	2	34	A:0.794118	G:0.205882	
chr28	11916275	2	34	G:1	A:0	
chr28	11916371	2	36	T:0.25	G:0.75	
chr28	11916624	2	38	C:1	T:0	
chr28	11916653	2	38	T:0.973684	C:0.0263158	
chr28	11916841	2	36	A:1	G:0	
chr28	11918348	2	26	G:0.576923	A:0.423077	
chr28	11918457	2	28	TA:0.928571	T:0.0714286	
chr28	11918523	2	36	G:0.416667	GTT:0.583333	
chr28	11919089	2	38	C:0.710526	T:0.289474	
chr28	11919381	2	30	A:0.233333	G:0.766667	
chr28	11919506	3	40	GGTGTGTGTGT:0.375	G:0.275	GGTGTGTGTGTGT:0.35
chr28	11920706	2	38	CT:0.526316	C:0.473684	
chr28	11921527	2	34	G:1	A:0	
chr28	11922160	2	38	GA:1	G:0	
chr28	11922375	2	40	A:0.4	T:0.6	
chr28	11922483	2	40	C:1	T:0	
chr28	11922543	2	36	C:1	T:0	
chr28	11922905	2	32	A:1	G:0	
chr28	11923553	2	36	G:0.25	C:0.75	
chr28	11924130	2	38	A:0.421053	G:0.578947	
chr28	11924200	2	38	G:0.631579	GA:0.368421	
chr28	11924543	3	34	T:0.558824	TA:0.294118	TAA:0.147059
chr28	11924749	2	36	C:0.972222	T:0.0277778	
chr28	11924955	2	36	G:0.972222	C:0.0277778	
chr28	11925141	2	36	C:1	T:0	
chr28	11925145	2	36	C:1	T:0	
chr28	11925403	2	40	T:0.55	G:0.45	
chr28	11925465	3	36	TAC:0.666667	T:0.25	TACAC:0.0833333
chr28	11925618	2	36	GTA:0.777778	G:0.222222	
chr28	11926540	2	38	C:1	T:0	
chr28	11926790	2	28	ATAT:1	A:0	
chr28	11927064	2	40	A:0.775	G:0.225	
chr28	11927120	2	36	C:1	A:0	
chr28	11927127	2	36	G:1	A:0	
chr28	11927537	2	38	A:1	T:0	
chr28	11927971	2	40	A:0.975	G:0.025	
chr28	11928193	3	38	GA:0.763158	G:0.184211	GAAAA:0.0526316
chr28	11928202	2	38	A:0.657895	G:0.342105	
chr28	11928669	2	36	C:1	T:0	
chr28	11929228	2	36	A:1	G:0	
chr28	11929501	2	32	A:0.5	T:0.5	
chr28	11929565	2	28	T:0.25	TG:0.75	
chr28	11929912	2	34	A:0.323529	T:0.676471	
chr28	11930389	2	38	G:1	C:0	

chr28	11930492	2	34	A:0.411765	G:0.588235
chr28	11930545	2	34	G:0.147059	GAATA:0.852941
chr28	11930585	2	34	CT:0.970588	C:0.0294118
chr28	11930846	2	36	T:1	C:0
chr28	11931229	2	40	C:1	T:0
chr28	11931810	2	38	A:0.789474	G:0.210526
chr28	11931814	2	38	C:0.973684	CTAAA:0.0263158
chr28	11932298	2	36	G:0.75	T:0.25
chr28	11932598	2	38	GTATATATATATATATATATATATATATATA:
0.473684	G:0.526316
chr28	11932897	2	30	G:0.5	A:0.5
chr28	11933330	2	38	A:0.973684	AAAAT:0.0263158
chr28	11933512	2	36	G:0.555556	GT:0.444444
chr28	11933888	2	40	T:0.025	C:0.975
chr28	11934128	2	36	C:1	T:0
chr28	11934241	2	40	T:0.4	TC:0.6
chr28	11934242	5	40	T:0.3	C:0.1	TCTCTCTC:0.325
TCTCTCTCTCTCTC:0	TCTCTCTCTCTCTCTC:0.275
chr28	11934544	2	34	CAG:0.764706	C:0.235294
chr28	11934741	2	36	G:0.972222	A:0.0277778
chr28	11935044	2	38	T:0.342105	A:0.657895
chr28	11935286	2	38	C:0.973684	CA:0.0263158
chr28	11935773	2	38	CCTCTCTCT:0.657895	C:
0.342105
chr28	11936014	2	38	T:0.947368	TA:0.0526316
chr28	11936934	2	30	T:0.666667	TA:0.333333
chr28	11937093	2	38	G:1	T:0
chr28	11937180	2	38	G:1	A:0
chr28	11937363	2	40	T:1	A:0
chr28	11937914	2	34	T:1	TAA:0
chr28	11937922	2	38	A:0.842105	AT:0.157895
chr28	11937923	2	38	A:0.815789	ATAT:0.184211
chr28	11937925	3	38	A:0.815789	T:0.184211
ATAT:0
chr28	11937926	2	38	A:1	AT:0
chr28	11937927	4	38	A:0.236842	T:0.657895
AT:0.105263	ATAT:0
chr28	11937946	2	32	A:0.25	G:0.75
chr28	11938525	3	40	TA:0.6	T:0.25	TTAAAAAAA:0.15
chr28	11938606	2	32	G:1	A:0
chr28	11938703	2	34	ATTTT:1	A:0
chr28	11938709	2	34	T:1	TAAAAAA:0
chr28	11938710	2	34	A:0.970588	T:0.0294118
chr28	11938757	5	38	AAAATAAATAAATAAATAAAT:0.236842
A:0.342105	AAAAT:0	AAAATAAAT:0.421053	AAAATAAATAAAT:0
chr28	11938798	2	38	AAAT:0.973684	A:0.0263158
chr28	11938805	2	38	TAAATAAATAAAATA:0.973684	T:
0.0263158
chr28	11940234	2	36	C:0.222222	G:0.777778
chr28	11940662	2	38	T:0.815789	A:0.184211
chr28	11941067	2	26	G:1	A:0
chr28	11941333	3	32	GA:0.5625	G:0.375	GAA:
0.0625
chr28	11941949	3	38	CT:0.710526	C:0.236842

CTT:0.0526316
chr28 11942027 2 36 T:0.666667 A:0.333333
chr28 11942083 2 36 C:0.666667 CT:0.333333
chr28 11942208 2 40 A:1 G:0
chr28 11942504 2 40 T:1 C:0
chr28 11943099 2 40 G:0.975 A:0.025
chr28 11943182 2 38 T:0.552632 C:0.447368
chr28 11943470 3 34 C:0.617647 CT:0.294118
CTT:0.0882353
chr28 11943775 2 36 T:1 C:0
chr28 11943783 2 36 G:1 T:0
chr28 11944716 3 40 C:0.825 CTATT:0.175
CTATTTATT:0
chr28 11944733 2 38 TA:0.552632 T:0.447368
chr28 11944735 2 38 TTTATTTA:0.552632 T:
0.447368
chr28 11945088 2 38 A:0.947368 T:0.0526316
chr28 11945937 2 32 G:1 A:0
chr28 11946538 2 36 G:0.611111 A:0.388889
chr28 11946751 2 36 T:1 TG:0
chr28 11946778 2 36 A:0.583333 G:0.416667
chr28 11946871 2 36 C:0.972222 T:0.0277778
chr28 11947351 2 36 C:0.75 T:0.25
chr28 11947671 2 40 C:0.6 T:0.4
chr28 11947690 2 40 T:0.6 TC:0.4
chr28 11948105 2 30 T:0.566667 G:0.433333
chr28 11948372 2 28 G:1 C:0
chr28 11948644 2 34 T:1 C:0
chr28 11949722 2 40 C:1 T:0
chr28 11949766 2 38 G:0.605263 A:0.394737
chr28 11952122 2 38 TA:1 T:0
chr28 11952170 2 40 A:1 T:0
chr28 11953056 2 36 A:0.694444 G:0.305556
chr28 11953367 2 40 A:1 G:0
chr28 11953661 2 38 T:0 A:1
chr28 11953692 2 36 AGATAGAT:1 A:0
chr28 11953710 2 36 AGAT:0.944444 A:0.0555556
chr28 11953926 2 28 TGGGGG:0 T:1
chr28 11953931 2 28 G:0 GTCATT:1
chr28 11953980 2 34 C:0 G:1
chr28 11954267 2 34 GT:0.588235 G:0.411765
chr28 11954676 3 38 C:0.578947 CACAT:0.0526316
CACACAT:0.368421
chr28 11956227 2 38 T:0.947368 TCC:0.0526316
chr28 11957195 2 40 A:1 G:0
chr28 11957224 2 40 G:0.55 A:0.45
chr28 11957389 2 38 GTC:0.657895 G:0.342105
chr28 11957443 2 40 TA:1 T:0
chr28 11957444 2 40 A:1 T:0
chr28 11958561 2 38 C:1 T:0
chr28 11958875 2 38 A:0.394737 G:0.605263
chr28 11959680 2 40 T:0.05 C:0.95
chr28 11959744 2 38 C:0.657895 T:0.342105
chr28 11960152 2 38 C:1 A:0

chr28 11960153 2 38 A:1 G:0
chr28 11960190 2 38 G:0.447368 C:0.552632
chr28 11960659 2 32 T:0.96875 C:0.03125
chr28 11960831 2 38 C:0.5 T:0.5
chr28 11961189 4 38 TAAAA:0.131579 T:0.263158
TA:0.263158 TAA:0.342105
chr28 11961209 2 34 C:0.970588 T:0.0294118
chr28 11961560 2 40 A:1 T:0
chr28 11961614 2 38 T:1 C:0
chr28 11962378 2 36 A:0.972222 G:0.0277778
chr28 11962816 2 28 G:0.428571 T:0.571429
chr28 11963058 2 34 A:1 T:0
chr28 11963604 2 38 G:0.315789 T:0.684211
chr28 11964129 2 38 CCCAACAGT:1 C:0
chr28 11964386 3 38 G:0.921053 GA:0.0789474
GAA:0
chr28 11964876 2 38 A:1 G:0
chr28 11965104 2 36 C:0.638889 CA:0.361111
chr28 11966279 2 36 C:1 T:0
chr28 11966965 2 38 T:1 C:0
chr28 11967211 2 36 G:0.972222 A:0.0277778
chr28 11967480 2 40 C:0.675 G:0.325
chr28 11967512 2 40 T:0.4 C:0.6
chr28 11967802 2 30 T:0.966667 TA:0.0333333
chr28 11967808 2 30 A:0.0333333 AT:0.966667
chr28 11967885 2 30 C:1 T:0
chr28 11968174 2 34 G:1 A:0
chr28 11968322 2 38 C:0.0263158 T:0.973684
chr28 11968856 4 36 TAAA:0.555556 T:0.0555556
TA:0.222222 TAA:0.166667
chr28 11969669 2 34 T:0.676471 TTC:0.323529
chr28 11970830 3 34 G:0.470588 T:0.411765
GT:0.117647
chr28 11971952 2 40 T:0.05 A:0.95
chr28 11972207 2 40 CT:0.675 C:0.325
chr28 11972301 2 38 G:1 A:0
chr28 11973488 2 36 T:0.0833333 A:0.916667
chr28 11973621 2 36 C:0.972222 T:0.0277778
chr28 11974765 6 36 TTTTA:0.444444 T:0
TTTTATTTA:0.111111 TTTTATTTATTTA:0.194444
TTTTATTTATTTATTTA:0.25 TTTTATTTATTTATTTATTTA:0
chr28 11974832 2 32 C:0.75 T:0.25
chr28 11975354 3 40 CCTCTCT:0.675 C:0.325
CCTCTCTCT:0
chr28 11975970 2 40 TAA:0.6 T:0.4
chr28 11976288 2 36 C:0.305556 T:0.694444
chr28 11976388 2 30 C:1 G:0
chr28 11976416 2 40 GA:1 G:0
chr28 11977267 2 38 A:1 T:0
chr28 11977593 2 36 TG:1 T:0
chr28 11977594 2 36 G:0.611111 T:0.388889
chr28 11977941 2 28 G:0.357143 GCT:0.642857
chr28 11978498 2 38 C:0.973684 T:0.0263158
chr28 11978698 2 38 C:1 G:0

chr28 11979036 2 36 C:0 T:1
chr28 11979037 2 36 T:0 A:1
chr28 11979045 2 36 G:0 A:1
chr28 11979095 3 40 C:0 A:0.325 G:0.675
chr28 11979096 2 40 G:0 A:1
chr28 11979117 2 40 C:0 A:1
chr28 11979120 2 40 TG:0 T:1
chr28 11979122 2 40 T:0 A:1
chr28 11979124 2 40 T:0 TA:1
chr28 11979128 2 40 G:0 GA:1
chr28 11979294 2 34 G:0.647059 A:0.352941
chr28 11979403 2 40 A:1 T:0
chr28 11979410 2 38 T:0.631579 TA:0.368421
chr28 11979466 2 38 A:0.973684 G:0.0263158
chr28 11979565 2 36 T:1 G:0
chr28 11979783 2 38 GA:1 G:0
chr28 11979986 2 34 T:0.941176 TA:0.0588235
chr28 11980824 2 38 T:1 A:0
chr28 11981157 2 34 A:1 G:0
chr28 11981193 2 36 T:0.611111 C:0.388889
chr28 11981412 2 32 C:1 A:0
chr28 11981521 2 30 AT:0.5 A:0.5
chr28 11981630 2 18 G:1 A:0
chr28 11981868 2 32 T:0.9375 C:0.0625
chr28 11981921 2 36 G:1 A:0
chr28 11982155 2 38 C:1 T:0
chr28 11982863 2 28 TA:0.821429 T:0.178571
chr28 11982967 2 36 T:0.583333 G:0.416667
chr28 11983015 2 30 C:1 T:0
chr28 11984094 4 30 TA:0.7 T:0 TAA:0.0333333
TAAA:0.266667
chr28 11984180 2 30 A:1 T:0
chr28 11984232 2 34 TA:0.941176 T:0.0588235
chr28 11984843 2 34 T:0.588235 C:0.411765
chr28 11985322 4 40 T:0.625 TACACAC:0.125
TACACACACACAC:0.1 TACACACACACACAC:0.15
chr28 11985341 2 38 A:1 ACACACG:0
chr28 11986832 2 38 C:0.552632 T:0.447368
chr28 11986972 2 40 T:0.45 G:0.55
chr28 11987107 2 38 C:0.631579 T:0.368421
chr28 11987193 2 38 C:1 CT:0
chr28 11987203 3 38 TAA:0.842105 AAA:0.105263
T:0.0526316
chr28 11987204 2 36 A:0.166667 T:0.833333
chr28 11987205 3 38 A:0.605263 T:0.342105
ATTT:0.0526316
chr28 11987394 2 36 G:0.333333 A:0.666667
chr28 11987565 2 36 C:0.611111 G:0.388889
chr28 11987567 2 38 G:0.526316 C:0.473684
chr28 11987569 2 36 T:0.0277778 G:0.972222
chr28 11987614 3 38 A:0.0263158 AC:0.921053
AAC:0.0526316
chr28 11987738 2 34 G:0.588235 A:0.411765
chr28 11987792 2 38 C:1 T:0

chr28	11987831	2	36	CA:0.583333	C:0.416667
chr28	11987838	2	36	AG:0.944444	A:0.0555556
chr28	11987939	2	38	G:0.473684	A:0.526316
chr28	11988358	2	40	T:0.625	A:0.375
chr28	11988666	2	36	C:0.361111	T:0.638889
chr28	11989396	2	40	C:0.05	T:0.95
chr28	11989706	2	40	T:0.85	C:0.15
chr28	11989779	2	40	C:0.15	T:0.85
chr28	11989837	2	40	G:0.8	A:0.2
chr28	11989917	2	40	A:0.175	G:0.825
chr28	11990163	2	34	G:0.852941	A:0.147059
chr28	11990325	2	40	T:0.9	G:0.1
chr28	11990723	2	38	TTGAC:1	T:0
chr28	11990918	2	38	TTGTC:0.973684	T:0.0263158
chr28	11990954	2	36	A:1	T:0
chr28	11991092	2	36	C:0.833333	G:0.166667
chr28	11991267	2	38	C:1	T:0
chr28	11991290	2	36	T:1	C:0
chr28	11991416	2	38	G:0.184211	A:0.815789
chr28	11991771	2	38	C:0.157895	T:0.842105
chr28	11992173	2	36	A:0.0277778	G:0.972222
chr28	11992225	2	32	TA:1	T:0
chr28	11992457	2	36	T:0.916667	C:0.0833333
chr28	11992471	2	38	CT:1	C:0
chr28	11992489	2	38	C:1	A:0
chr28	11992545	2	38	G:1	A:0
chr28	11992674	2	34	GA:0.176471	G:0.823529
chr28	11993152	2	38	A:0.0263158	G:0.973684
chr28	11993700	2	36	A:0	G:1
chr28	11993701	2	38	A:1	G:0
chr28	11994118	2	40	A:1	T:0
chr28	11994154	2	40	G:0.725	A:0.275
chr28	11995746	2	40	T:0.975	C:0.025
chr28	11995875	2	34	T:1	A:0
chr28	11996015	2	38	C:0.921053	T:0.0789474
chr28	11996842	2	38	T:0.131579	C:0.868421
chr28	11996881	2	38	C:1	T:0
chr28	11996935	2	36	G:0.0277778	T:0.972222
chr28	11997298	2	38	G:0.947368	GA:0.0526316
chr28	11997605	2	36	C:1	T:0
chr28	11997790	2	36	G:0.972222	GA:0.0277778
chr28	11997824	2	34	C:1	G:0
chr28	11998542	2	38	C:0.0526316	CT:0.947368
chr28	11998797	2	38	G:0	C:1
chr28	11998803	2	38	G:0.868421	A:0.131579
chr28	11999041	2	38	G:0.973684	A:0.0263158
chr28	11999424	2	40	G:1	T:0
