# Supplementary material for: Genome-Wide Analyses Suggest Mechanisms Involving Early B-Cell Development in Canine IgA Deficiency
Source: PLoS One. 2015 Jul 30;10(7):e0133844. doi: 10.1371/journal.pone.0133844 (PMC4520476; doi:10.1371/journal.pone.0133844)
Supplement: S5 Table — (PDF) [file pone.0133844.s015.pdf]

Table S5: Allele frequencies based on 20 Labradors across chr28: 9000091-11999424 (canfam3)

CHROM	POS	N_ALLELES	N_CHR	{ALLELE:FREQ}				
chr28	9000091	2	38	T:1	C:0			
chr28	9000246	2	38	T:0.894737	TCAA:0.105263			
chr28	9000473	2	40	A:0.825	G:0.175			
chr28	9000522	2	40	T:0.85	C:0.15			
chr28	9000657	2	40	C:0.85	CTTTAT:0.15			
chr28	9000789	2	38	T:0.842105	C:0.157895			
chr28	9000939	2	40	CGG:0.85	C:0.15			
chr28	9001032	2	36	C:0.777778	G:0.222222			
chr28	9001109	2	38	C:0.894737	G:0.105263			
chr28	9001229	5	38	GAGAGAAGAGA:0.210526	G:0.0789474	GAGAGA:0.315789	GAGAGAAGAGAAGAGA:0.0526316	GAGAGAAGAGAAGAGAAGAGAAGAGAAGAGA:0.342105
chr28	9001674	2	38	G:0.842105	A:0.157895			
chr28	9001787	2	40	CA:0.85	C:0.15			
chr28	9001833	2	40	A:0.85	G:0.15			
chr28	9001982	2	40	AT:0.85	A:0.15			
chr28	9002236	2	40	ATCCAGGAC:0.825	A:0.175			
chr28	9002267	2	38	C:0.815789	T:0.184211			
chr28	9002509	2	40	C:0.85	T:0.15			
chr28	9002784	2	40	A:0.825	G:0.175			
chr28	9002831	2	40	C:0.825	T:0.175			
chr28	9003437	2	38	T:0.868421	C:0.131579			
chr28	9003632	2	38	G:0.894737	GCTCT:0.105263			
chr28	9003633	2	38	C:0.894737	CTCTT:0.105263			
chr28	9003904	2	40	C:0.85	T:0.15			
chr28	9004320	2	40	A:0.65	C:0.35			
chr28	9004520	2	40	A:0.65	C:0.35			
chr28	9004564	2	36	A:0.833333	G:0.166667			
chr28	9004919	2	40	T:0.85	C:0.15			
chr28	9005309	2	38	A:0.763158	G:0.236842			
chr28	9005338	2	36	T:0.805556	C:0.194444			
chr28	9005502	2	40	G:0.925	C:0.075			
chr28	9005638	2	38	G:0.973684	A:0.0263158			
chr28	9005688	2	38	A:0.842105	C:0.157895			
chr28	9006019	2	38	A:0.868421	AC:0.131579			
chr28	9006568	2	38	T:0.631579	TA:0.368421			
chr28	9007042	2	36	A:0.888889	C:0.111111			
chr28	9007529	2	38	CA:0.868421	C:0.131579			
chr28	9008103	2	40	T:0.85	C:0.15			
chr28	9008419	2	40	C:0.825	T:0.175			
chr28	9008506	2	38	CAGAG:0.842105	C:0.157895			
chr28	9008594	2	32	C:0.78125	T:0.21875			
chr28	9008801	2	40	C:0.8	CA:0.2			
chr28	9008900	2	36	T:0.75	TA:0.25			
chr28	9009257	2	38	A:0.842105	G:0.157895			
chr28	9009318	2	36	G:0.833333	A:0.166667			
chr28	9009369	2	40	AAC:0.875	A:0.125			
chr28	9009943	2	38	C:0.921053	T:0.0789474			
chr28	9010058	2	38	GTT:0.868421	G:0.131579			
chr28	9010471	2	36	G:0.972222	A:0.0277778			
chr28	9011133	2	34	A:0.823529	C:0.176471			

chr28	9011297	2	38	G:0.868421	A:0.131579			
chr28	9011572	2	38	A:0.868421	T:0.131579			
chr28	9011586	2	38	TGTA:0.868421	T:0.131579			
chr28	9012020	2	40	CCTTTCTTTCT:0.85	C:0.15			
chr28	9012155	2	38	C:0.894737	T:0.105263			
chr28	9012207	2	40	GCTC:0.825	G:0.175			
chr28	9012229	2	40	G:0.825	T:0.175			
chr28	9012232	2	40	A:0.825	C:0.175			
chr28	9012244	2	40	C:0.825	T:0.175			
chr28	9012364	2	38	G:0.894737	A:0.105263			
chr28	9012499	2	36	C:0.833333	T:0.166667			
chr28	9012965	2	38	G:0.868421	A:0.131579			
chr28	9013095	2	40	C:0.85	G:0.15			
chr28	9013098	2	40	A:0.725	G:0.275			
chr28	9013127	2	38	T:0.815789	C:0.184211			
chr28	9013382	2	38	T:0.684211	C:0.315789			
chr28	9013443	2	40	G:0.825	A:0.175			
chr28	9013453	2	40	G:0.825	A:0.175			
chr28	9013626	2	40	C:0.8	T:0.2			
chr28	9013745	2	40	T:0.725	C:0.275			
chr28	9013848	2	40	C:0.875	T:0.125			
chr28	9013894	2	40	C:0.9	T:0.1			
chr28	9013951	2	40	C:0.825	G:0.175			
chr28	9014048	2	40	G:0.875	A:0.125			
chr28	9014089	2	38	A:0.631579	C:0.368421			
chr28	9014093	2	38	C:0.815789	CA:0.184211			
chr28	9014112	2	38	T:0.631579	C:0.368421			
chr28	9014138	2	36	T:0.638889	G:0.361111			
chr28	9014282	2	40	C:0.875	T:0.125			
chr28	9014788	2	40	G:0.875	GTCA:0.125			
chr28	9014789	2	40	G:0.875	GGATCCTGATC:0.125			
chr28	9014793	2	40	C:0.875	T:0.125			
chr28	9014795	2	40	CG:0.875	C:0.125			
chr28	9014797	2	40	G:0.875	T:0.125			
chr28	9014841	2	38	C:0.789474	T:0.210526			
chr28	9014988	2	40	G:0.85	T:0.15			
chr28	9015209	2	40	CAT:0.85	C:0.15			
chr28	9015491	2	40	A:0.925	G:0.075			
chr28	9015823	2	36	T:0.833333	A:0.166667			
chr28	9016088	2	38	C:0.921053	G:0.0789474			
chr28	9016103	2	36	A:0.777778	G:0.222222			
chr28	9016322	2	38	A:1	AAT:0			
chr28	9016326	2	38	CACAT:0.894737	C:0.105263			
chr28	9016330	5	40	T:0.6	C:0.05	TAC:0.175	TACAC:0.025	TACACAC:0.15
chr28	9016360	3	40	C:0.825	G:0.175	CACAG:0		
chr28	9016682	2	38	G:1	GA:0			
chr28	9016800	2	40	T:0.875	A:0.125			
chr28	9016982	2	34	A:0.823529	G:0.176471			
chr28	9017130	3	40	GAA:0.625	G:0.075	GA:0.3		
chr28	9017172	2	36	A:0.75	AAAGC:0.25			
chr28	9017284	2	40	C:0.875	T:0.125			
chr28	9017304	2	40	T:0.875	G:0.125			
chr28	9017446	2	38	C:0.921053	T:0.0789474			

chr28	9017524	2	34	TCTCTCC:0.911765	T:0.0882353			
chr28	9017528	2	36	TCC:0.361111	T:0.638889			
chr28	9017953	2	40	G:0.75	A:0.25			
chr28	9018548	2	38	T:0.105263	G:0.894737			
chr28	9018662	2	36	C:0.805556	G:0.194444			
chr28	9018674	2	36	TA:0.722222	T:0.277778			
chr28	9018856	5	40	CAAATAAATAAAT:0.325	C:0.275	CTAAAT:0.075	CTAAATAAAT:0	CAAATAAATAAATAAAT:0.325
chr28	9018860	2	40	T:1	TCTC:0			
chr28	9018864	2	40	T:0.975	TCTC:0.025			
chr28	9019603	2	38	C:0.894737	T:0.105263			
chr28	9019751	2	38	A:0.894737	AT:0.105263			
chr28	9020694	2	40	C:0.775	CAAAT:0.225			
chr28	9020794	2	40	A:0.875	G:0.125			
chr28	9020798	2	40	A:0.875	G:0.125			
chr28	9021002	2	32	A:1	T:0			
chr28	9022289	2	38	C:0.842105	T:0.157895			
chr28	9022290	2	38	A:0.710526	G:0.289474			
chr28	9022351	2	38	C:0.842105	T:0.157895			
chr28	9022352	2	38	G:0.842105	A:0.157895			
chr28	9022420	2	36	G:0.805556	T:0.194444			
chr28	9022668	2	38	AG:0.894737	A:0.105263			
chr28	9022984	2	30	C:0.733333	CT:0.266667			
chr28	9023016	2	32	G:0.84375	T:0.15625			
chr28	9023157	2	40	C:0.825	CTGTGTG:0.175			
chr28	9023159	3	40	CTG:0.75	C:0.075	GTG:0.175		
chr28	9023202	2	38	T:0.868421	TA:0.131579			
chr28	9023408	2	40	G:0.85	GTAAA:0.15			
chr28	9023450	3	40	TGA:0.45	T:0.275	TGAGA:0.275		
chr28	9023552	2	40	G:0.825	GATC:0.175			
chr28	9024684	2	36	A:0.833333	G:0.166667			
chr28	9024720	4	40	T:0.475	TA:0.1	TAA:0.25	TAAA:0.175	
chr28	9024730	2	40	A:0.7	AAAAAAAAAAAG:0.3			
chr28	9024778	2	36	C:0.888889	T:0.111111			
chr28	9025290	2	40	C:0.9	T:0.1			
chr28	9025339	2	40	T:0.8	TG:0.2			
chr28	9025345	2	40	A:0.8	T:0.2			
chr28	9025361	2	40	T:0.875	A:0.125			
chr28	9025362	2	40	A:0.875	T:0.125			
chr28	9025730	2	40	AAT:0.8	A:0.2			
chr28	9025731	2	40	AT:0.25	A:0.75			
chr28	9025899	2	32	C:0.75	T:0.25			
chr28	9026013	2	40	TG:0.85	T:0.15			
chr28	9026014	2	40	G:0.85	T:0.15			
chr28	9026018	2	40	T:0.85	A:0.15			
chr28	9026023	2	40	T:0.85	A:0.15			
chr28	9026048	2	40	C:0.725	T:0.275			
chr28	9026069	2	40	G:0.85	C:0.15			
chr28	9026275	3	40	TAA:0.7	T:0.125	TA:0.175		
chr28	9026405	2	36	G:0.805556	A:0.194444			
chr28	9026635	2	38	A:0.868421	G:0.131579			
chr28	9026679	2	36	T:0.861111	C:0.138889			
chr28	9026960	2	38	A:0.868421	G:0.131579			

chr28	9027069	2	38	C:0.815789	A:0.184211	
chr28	9028226	2	40	C:0.825	T:0.175	
chr28	9028471	2	40	C:0.85	T:0.15	
chr28	9029031	2	38	C:0.736842	CA:0.263158	
chr28	9029032	2	40	A:0.825	AAAAAAAAAATAAAAAT:0.175	
chr28	9029064	2	38	T:0.631579	C:0.368421	
chr28	9029759	2	34	C:0.705882	CT:0.294118	
chr28	9029808	2	40	CACAGAGAGAGAGAG:0.825	C:0.175	
chr28	9029810	3	40	CAG:0.35	C:0.35	CAGAG:0.3
chr28	9029956	2	36	G:0.805556	GAGCC:0.194444	
chr28	9030144	2	38	A:0.789474	G:0.210526	
chr28	9030185	2	40	T:0.75	A:0.25	
chr28	9030186	2	40	T:0.85	A:0.15	
chr28	9030657	2	36	G:0.888889	A:0.111111	
chr28	9030699	2	38	C:0.684211	T:0.315789	
chr28	9031234	2	38	G:0.789474	A:0.210526	
chr28	9031701	2	38	C:1	T:0	
chr28	9031738	2	40	T:0.875	C:0.125	
chr28	9031741	3	40	GT:0.625	G:0.2	GTT:0.175
chr28	9032465	2	38	A:1	G:0	
chr28	9033585	2	38	T:0.842105	A:0.157895	
chr28	9033586	2	38	T:0.842105	C:0.157895	
chr28	9033837	2	22	TA:0.590909	T:0.409091	
chr28	9034448	2	38	G:0.815789	A:0.184211	
chr28	9035127	2	40	C:0.85	CTTAT:0.15	
chr28	9035331	2	40	T:0.825	TAATA:0.175	
chr28	9036932	2	38	T:0.815789	C:0.184211	
chr28	9037083	2	38	T:0.421053	C:0.578947	
chr28	9037089	2	38	T:0.868421	C:0.131579	
chr28	9037329	2	38	G:1	A:0	
chr28	9037585	2	40	G:0.9	GA:0.1	
chr28	9037594	2	40	C:0.975	A:0.025	
chr28	9037846	2	36	CTG:0.833333	C:0.166667	
chr28	9038349	2	38	A:0.842105	G:0.157895	
chr28	9038962	2	38	G:1	A:0	
chr28	9039044	2	38	T:0.710526	G:0.289474	
chr28	9039059	2	38	C:0.842105	CCTT:0.157895	
chr28	9039125	2	38	C:0.894737	T:0.105263	
chr28	9039672	2	40	G:1	A:0	
chr28	9039766	2	38	G:1	C:0	
chr28	9040250	2	36	T:0.861111	C:0.138889	
chr28	9040258	2	38	GC:1	G:0	
chr28	9040648	2	38	G:1	C:0	
chr28	9040777	2	40	C:1	CACTA:0	
chr28	9040794	2	40	A:1	G:0	
chr28	9040979	2	40	T:1	C:0	
chr28	9040991	2	40	ACT:1	A:0	
chr28	9041256	2	38	G:1	A:0	
chr28	9041508	2	40	A:1	C:0	
chr28	9041559	2	38	G:1	A:0	
chr28	9041584	2	40	CTT:1	C:0	
chr28	9042088	2	36	G:1	A:0	
chr28	9042090	2	36	A:1	T:0	
chr28	9042656	2	40	T:1	G:0	

chr28 9042713 2 40 C:1 T:0
chr28 9043052 2 40 GC:1 G:0
chr28 9046466 2 36 G:1 A:0
chr28 9046907 2 40 A:1 AAC:0
chr28 9047244 2 36 A:0 G:1
chr28 9047405 2 38 G:1 A:0
chr28 9047791 2 34 T:0 TG:1
chr28 9048050 2 34 T:1 C:0
chr28 9048208 2 32 AT:0 A:1
chr28 9048224 2 24 T:1 C:0
chr28 9048896 2 36 GC:1 G:0
chr28 9049363 2 40 AAAG:1 A:0
chr28 9049366 3 40 GAAGAAAGAAAGAAAGA:0.35 G:0.375
GAAGA:0.275
chr28 9049429 2 40 G:1 GA:0
chr28 9050173 2 40 TG:1 T:0
chr28 9050240 2 40 A:1 C:0
chr28 9051784 2 40 G:1 C:0
chr28 9051823 2 36 C:1 T:0
chr28 9051826 2 36 C:1 T:0
chr28 9052674 2 38 A:1 T:0
chr28 9052923 2 32 G:1 A:0
chr28 9052947 4 36 AACACACAC:0.222222 A:
0.222222 AAC:0.333333 AACAC:0.222222
chr28 9053125 2 34 G:1 A:0
chr28 9053140 2 36 A:0.888889 G:0.111111
chr28 9053532 2 36 G:1 C:0
chr28 9055264 2 36 GT:0.666667 G:0.333333
chr28 9056134 2 40 GGCAGCCCCCGTGGT:0.95 G:0.05
chr28 9056143 2 40 C:1 G:0
chr28 9056457 2 36 A:1 G:0
chr28 9057481 2 40 T:1 A:0
chr28 9058292 2 34 T:1 TA:0
chr28 9059203 2 28 ATTTCTT:1 A:0
chr28 9059843 2 36 G:1 C:0
chr28 9060075 4 40 TACAAA:0.8 T:0.2
TACAAAACAAA:0 TACAAAACAAAACAAAACAAA:0
chr28 9060852 2 38 C:0.631579 T:0.368421
chr28 9061609 2 40 A:1 C:0
chr28 9061679 2 40 A:1 G:0
chr28 9061900 2 36 C:1 T:0
chr28 9062033 2 38 TCGCTCACA:1 T:0
chr28 9062147 2 36 G:1 A:0
chr28 9062361 3 22 TGG:0.545455 T:0.0909091
TG:0.363636
chr28 9063282 2 24 AC:0.916667 A:0.0833333
chr28 9063400 2 26 G:0.423077 C:0.576923
chr28 9064386 2 32 C:0.96875 CTT:0.03125
chr28 9064472 2 38 G:1 GC:0
chr28 9064477 2 38 G:1 C:0
chr28 9064668 2 36 G:1 T:0
chr28 9065709 2 38 GA:0.921053 G:0.0789474
chr28 9066664 2 40 G:0.7 A:0.3
chr28 9066770 2 36 A:1 G:0

```
chr28	9067055	2	40	G:1	A:0
chr28	9068116	2	40	A:0.55	AAAAG:0.45
chr28	9068122	2	40	A:1	AAGAAAG:0
chr28	9068140	2	40	G:1	A:0
chr28	9068147	2	40	A:1	AAAG:0
chr28	9068158	3	40	G:0.425	GAAGA:0.325
GGAAGAAAGAAAGAAAGGAAGGAAGAAAGAAAGAAAGA:0.25
chr28	9068456	2	40	C:1	T:0
chr28	9068849	2	40	T:1	TAAAAAA:0
chr28	9068872	2	36	GGAATA:1	G:0
chr28	9069173	2	38	G:1	A:0
chr28	9069266	2	38	A:1	G:0
chr28	9069399	2	30	C:0.633333	CA:0.366667
chr28	9069746	2	40	T:1	C:0
chr28	9070332	2	38	A:1	AGT:0
chr28	9071571	2	38	G:0.842105	T:0.157895
chr28	9071574	2	40	G:1	T:0
chr28	9071694	2	40	A:1	G:0
chr28	9071730	2	40	G:1	T:0
chr28	9072256	2	36	T:1	A:0
chr28	9073177	3	40	TAA:0.6	T:0	TA:0.4
chr28	9073184	2	40	A:0.725	T:0.275
chr28	9073654	2	38	T:1	G:0
chr28	9073869	2	34	T:1	C:0
chr28	9073993	2	40	A:0.65	AT:0.35
chr28	9073995	2	40	A:0.65	T:0.35
chr28	9074033	2	36	CAGATAGAG:0.611111	C:
0.388889
chr28	9074037	3	38	TAGAGAG:0.710526	T:0.0789474
TAGAG:0.210526
chr28	9074750	2	36	TA:0.611111	T:0.388889
chr28	9075130	2	38	C:0.605263	T:0.394737
chr28	9075511	2	40	CT:0.65	C:0.35
chr28	9075676	2	40	A:0.7	G:0.3
chr28	9075684	2	40	T:0.7	A:0.3
chr28	9076002	2	40	C:0.95	T:0.05
chr28	9076092	2	40	G:0.65	C:0.35
chr28	9076158	2	34	T:0.588235	C:0.411765
chr28	9076182	2	34	T:0.588235	C:0.411765
chr28	9076673	2	38	CA:0.657895	C:0.342105
chr28	9076764	2	36	T:0.777778	C:0.222222
chr28	9076812	2	38	G:0.657895	T:0.342105
chr28	9076821	2	38	T:0.657895	G:0.342105
chr28	9076835	2	38	G:0.657895	T:0.342105
chr28	9076846	2	38	C:0.657895	T:0.342105
chr28	9076857	2	38	A:0.657895	T:0.342105
chr28	9076902	2	36	T:0.722222	C:0.277778
chr28	9076908	2	36	T:0.722222	C:0.277778
chr28	9076958	3	36	TA:0.472222	T:0.277778
TAA:0.25
chr28	9077225	2	38	C:0.605263	T:0.394737
chr28	9077262	2	38	T:0.710526	A:0.289474
chr28	9077365	2	38	A:0.763158	G:0.236842
chr28	9077504	2	38	C:0.736842	T:0.263158
```

chr28	9077715	2	38	T:0.868421	C:0.131579	
chr28	9077725	3	38	C:0.868421	CTTT:0.131579	CTTTT:0
chr28	9078016	2	38	GCAGT:0.789474	G:0.210526	
chr28	9078201	2	36	C:0.638889	T:0.361111	
chr28	9078207	2	36	A:0.638889	G:0.361111	
chr28	9078254	2	34	T:0.705882	G:0.294118	
chr28	9078267	2	36	C:0.611111	A:0.388889	
chr28	9078294	2	38	A:0.684211	G:0.315789	
chr28	9078307	2	38	T:0.657895	TG:0.342105	
chr28	9078314	2	38	G:0.657895	A:0.342105	
chr28	9078760	2	40	C:0.55	CT:0.45	
chr28	9079172	2	40	A:0.65	G:0.35	
chr28	9079178	2	40	G:0.65	A:0.35	
chr28	9079196	2	40	A:0.65	G:0.35	
chr28	9079218	2	40	A:0.65	G:0.35	
chr28	9079312	2	38	C:0.684211	T:0.315789	
chr28	9079313	2	38	CT:0.684211	C:0.315789	
chr28	9079508	2	36	C:0.777778	T:0.222222	
chr28	9079509	2	36	A:0.777778	G:0.222222	
chr28	9079784	2	36	C:1	CT:0	
chr28	9080212	2	40	G:1	GCC:0	
chr28	9080216	2	40	C:1	CGA:0	
chr28	9080219	2	40	T:0.725	G:0.275	
chr28	9080220	2	40	C:0.725	A:0.275	
chr28	9080303	2	38	G:0.526316	A:0.473684	
chr28	9080344	2	38	C:1	T:0	
chr28	9080453	2	36	T:0.666667	C:0.333333	
chr28	9080484	2	34	T:0.588235	TA:0.411765	
chr28	9080539	2	38	G:0.605263	A:0.394737	
chr28	9081156	2	34	C:0.676471	CT:0.323529	
chr28	9081540	2	38	A:0.552632	C:0.447368	
chr28	9081732	2	36	C:0.5	A:0.5	
chr28	9082462	2	40	C:0.625	CT:0.375	
chr28	9082581	2	34	G:0.558824	A:0.441176	
chr28	9082712	2	38	CAA:0.526316	C:0.473684	
chr28	9082716	2	38	T:0.526316	C:0.473684	
chr28	9082732	2	38	C:0.578947	CTT:0.421053	
chr28	9082740	2	36	C:0.166667	T:0.833333	
chr28	9083013	2	38	A:0.526316	G:0.473684	
chr28	9083057	2	40	T:0.475	C:0.525	
chr28	9083137	2	40	C:0.525	T:0.475	
chr28	9083675	2	38	CT:0.842105	C:0.157895	
chr28	9084375	2	40	CCT:0.625	C:0.375	
chr28	9084629	2	36	G:0.611111	C:0.388889	
chr28	9084938	2	36	G:0.527778	A:0.472222	
chr28	9085196	2	34	G:0.529412	T:0.470588	
chr28	9085578	2	40	G:0.525	GCTCTCT:0.475	
chr28	9085618	2	38	T:0.605263	A:0.394737	
chr28	9086088	2	38	CTT:0.605263	C:0.394737	
chr28	9086258	2	36	G:0.527778	C:0.472222	
chr28	9086297	2	38	CTTTATA:0.973684	C:0.0263158	
chr28	9086512	2	38	T:1	C:0	
chr28	9086603	2	40	T:0.825	C:0.175	

chr28	9086707	2	40	C:0.65	G:0.35	
chr28	9086913	2	40	T:0.575	C:0.425	
chr28	9087018	2	40	A:0.175	C:0.825	
chr28	9087957	2	38	A:0.5	G:0.5	
chr28	9088172	3	40	TA:0.65	T:0.175	TAA:0.175
chr28	9088444	2	38	TAGCTA:0.710526	T:0.289474	
chr28	9088609	2	26	T:0.423077	TAA:0.576923	
chr28	9088684	2	36	T:0.555556	C:0.444444	
chr28	9088799	2	30	T:0.466667	TAA:0.533333	
chr28	9088927	2	38	C:0.894737	G:0.105263	
chr28	9089022	2	36	C:0.555556	A:0.444444	
chr28	9089105	2	38	C:0.842105	T:0.157895	
chr28	9089203	2	38	T:0.552632	A:0.447368	
chr28	9089232	2	38	C:0.894737	CAGAGAG:0.105263	
chr28	9089415	2	40	C:0.85	T:0.15	
chr28	9090136	2	34	G:0.911765	A:0.0882353	
chr28	9090764	2	38	A:0.842105	C:0.157895	
chr28	9090988	2	40	A:0.525	G:0.475	
chr28	9091191	2	36	C:0.861111	T:0.138889	
chr28	9091307	2	40	C:0.85	T:0.15	
chr28	9091652	2	38	C:0.710526	T:0.289474	
chr28	9091771	2	34	GT:0.764706	G:0.235294	
chr28	9091923	2	38	G:0.605263	A:0.394737	
chr28	9092080	2	38	C:0.842105	G:0.157895	
chr28	9092223	2	40	C:0.65	T:0.35	
chr28	9092909	2	36	CT:0.638889	C:0.361111	
chr28	9093041	2	38	C:0.657895	T:0.342105	
chr28	9093112	2	40	G:0.65	A:0.35	
chr28	9093187	2	36	CCT:0.472222	C:0.527778	
chr28	9093590	2	36	CCT:0.75	C:0.25	
chr28	9093627	2	36	TAAAA:0.805556	T:0.194444	
chr28	9093630	2	36	A:0.944444	AT:0.0555556	
chr28	9093633	2	36	A:0.944444	AT:0.0555556	
chr28	9093635	2	36	A:0.805556	T:0.194444	
chr28	9093641	2	36	A:0.944444	T:0.0555556	
chr28	9094162	2	36	A:0.527778	G:0.472222	
chr28	9094920	2	40	T:0.6	G:0.4	
chr28	9095328	2	38	G:0.868421	A:0.131579	
chr28	9095562	2	34	CTCTG:0.676471	C:0.323529	
chr28	9095605	3	38	AG:0.552632	A:0.315789	AGG:0.131579
chr28	9095719	2	38	G:1	A:0	
chr28	9095822	2	38	G:0.710526	T:0.289474	
chr28	9096051	2	36	A:0.694444	G:0.305556	
chr28	9096278	2	36	T:1	G:0	
chr28	9096366	2	40	TAC:0.475	T:0.525	
chr28	9096449	2	40	C:1	G:0	
chr28	9096503	2	40	C:0.525	CTTTTTTTTTTTT:0.475	
chr28	9096636	2	40	T:0.875	C:0.125	
chr28	9097105	2	36	C:0.722222	T:0.277778	
chr28	9097982	2	38	C:0.684211	T:0.315789	
chr28	9098466	2	40	T:0.825	A:0.175	
chr28	9098761	2	40	AC:0.725	A:0.275	
chr28	9099002	2	40	C:0.875	T:0.125	

chr28	9099249	2	36	T:0.5	C:0.5	
chr28	9099266	2	38	G:0.868421	T:0.131579	
chr28	9099550	2	40	T:0.925	TAA:0.075	
chr28	9099640	2	38	A:0.894737	C:0.105263	
chr28	9100002	2	38	G:0.868421	A:0.131579	
chr28	9100006	2	38	C:1	T:0	
chr28	9100067	2	40	TTCTC:0.875	T:0.125	
chr28	9100103	2	38	GA:0.894737	G:0.105263	
chr28	9100185	2	38	G:0.710526	C:0.289474	
chr28	9100333	2	32	T:0.9375	C:0.0625	
chr28	9100450	3	38	C:0.684211	CTCTG:0.289474	CTCTCTCTCTCTCTG:0.0263158
chr28	9100716	2	34	C:0.882353	A:0.117647	
chr28	9100869	2	40	C:0.85	T:0.15	
chr28	9101114	2	40	T:0.85	C:0.15	
chr28	9101296	2	38	C:0.5	G:0.5	
chr28	9102150	2	40	A:0.825	G:0.175	
chr28	9102269	2	38	G:0.842105	C:0.157895	
chr28	9102282	2	38	A:0.868421	G:0.131579	
chr28	9102285	2	38	G:0.868421	C:0.131579	
chr28	9102675	2	38	CCTGT:0.763158	C:0.236842	
chr28	9103023	2	40	C:0.775	T:0.225	
chr28	9103084	2	36	T:0.694444	A:0.305556	
chr28	9103085	2	38	T:0.473684	A:0.526316	
chr28	9103207	2	36	T:0.861111	C:0.138889	
chr28	9103230	2	36	T:0	C:1	
chr28	9103393	2	40	T:0.9	C:0.1	
chr28	9103559	2	40	G:0.9	A:0.1	
chr28	9104029	2	40	A:0.85	G:0.15	
chr28	9104106	2	38	C:0.868421	G:0.131579	
chr28	9104152	2	40	TAAC:0.75	T:0.25	
chr28	9104461	2	40	T:0.85	TA:0.15	
chr28	9104565	2	38	CAATT:0.921053	C:0.0789474	
chr28	9104842	2	40	C:0.85	CTTTCTTTTCT:0.15	
chr28	9104935	2	38	A:0.921053	G:0.0789474	
chr28	9105010	2	40	CTTTTTTTTGTTTT:0.825	C:0.175	
chr28	9105019	2	40	GT:0.6	G:0.4	
chr28	9105026	2	40	T:0.875	G:0.125	
chr28	9105332	2	38	C:0.921053	G:0.0789474	
chr28	9105405	2	38	G:0.552632	A:0.447368	
chr28	9105780	2	36	G:1	A:0	
chr28	9106125	2	40	TCTTTCCC:0.85	T:0.15	
chr28	9106133	2	40	A:0.85	AGGT:0.15	
chr28	9106477	2	38	A:0.815789	G:0.184211	
chr28	9106924	2	32	G:0.71875	A:0.28125	
chr28	9107300	2	38	G:0.815789	A:0.184211	
chr28	9107320	2	38	C:0.894737	T:0.105263	
chr28	9107462	2	36	G:0.527778	C:0.472222	
chr28	9107744	2	38	C:0.657895	T:0.342105	
chr28	9108048	2	38	A:0.894737	G:0.105263	
chr28	9108085	2	40	G:0.75	A:0.25	
chr28	9108126	2	40	T:0.9	C:0.1	
chr28	9108519	2	36	CTTT:0.861111	C:0.138889	
chr28	9108535	2	36	T:0.861111	A:0.138889	

chr28	9108685	2	40	GAA:0.775	G:0.225	
chr28	9108720	2	38	T:0.842105	C:0.157895	
chr28	9108873	2	40	C:0.85	T:0.15	
chr28	9108950	2	38	C:0.842105	A:0.157895	
chr28	9108980	2	38	AAAAAG:0.868421	A:0.131579	
chr28	9109317	2	40	T:1	A:0	
chr28	9109371	2	38	A:0.842105	G:0.157895	
chr28	9109634	2	34	A:0.823529	G:0.176471	
chr28	9109805	2	40	C:0.85	T:0.15	
chr28	9110262	2	40	A:0.875	G:0.125	
chr28	9110338	2	34	G:0.852941	A:0.147059	
chr28	9110411	2	36	G:1	A:0	
chr28	9110488	2	36	T:0.916667	C:0.0833333	
chr28	9110514	3	38	TA:0.842105	T:0.157895	TAA:0
chr28	9110601	2	38	GA:1	G:0	
chr28	9110604	2	38	GT:1	G:0	
chr28	9110928	2	40	A:0.575	G:0.425	
chr28	9111013	2	38	G:0.815789	A:0.184211	
chr28	9111170	2	40	A:0.875	C:0.125	
chr28	9111254	2	38	G:0.921053	GT:0.0789474	
chr28	9111556	2	40	C:0.55	G:0.45	
chr28	9111695	2	40	A:0.875	G:0.125	
chr28	9112237	2	40	G:0.875	A:0.125	
chr28	9113042	2	40	G:0.875	A:0.125	
chr28	9113206	2	36	C:0.638889	G:0.361111	
chr28	9113447	2	36	TTTA:0.972222	T:0.0277778	
chr28	9113448	2	36	TTA:0.888889	T:0.111111	
chr28	9113458	2	38	TTTTA:0.868421	T:0.131579	
chr28	9113486	2	36	C:0.833333	CAG:0.166667	
chr28	9113601	2	38	C:0.842105	A:0.157895	
chr28	9113608	2	36	T:0.583333	C:0.416667	
chr28	9113777	2	38	TG:0.815789	T:0.184211	
chr28	9113925	2	34	TA:0.911765	T:0.0882353	
chr28	9114091	2	38	G:0.552632	C:0.447368	
chr28	9114192	2	40	C:0.9	A:0.1	
chr28	9114394	2	40	T:0.6	A:0.4	
chr28	9114401	2	40	C:0.875	CAA:0.125	
chr28	9114999	2	36	C:0.861111	T:0.138889	
chr28	9115428	2	38	C:0.894737	T:0.105263	
chr28	9115456	2	40	A:0.925	G:0.075	
chr28	9115654	2	40	A:0.825	G:0.175	
chr28	9115933	2	38	G:0.605263	GTTGT:0.394737	
chr28	9116460	2	40	C:0.9	T:0.1	
chr28	9117028	2	40	T:0.725	A:0.275	
chr28	9117210	2	38	T:0.710526	C:0.289474	
chr28	9117252	2	38	A:0.763158	G:0.236842	
chr28	9117980	2	40	A:0.9	T:0.1	
chr28	9117981	3	40	AAG:0.9	A:0.1	GAG:0
chr28	9118004	2	40	C:0.9	T:0.1	
chr28	9118072	2	36	GAAA:0.888889	G:0.111111	
chr28	9118145	2	36	C:0.861111	T:0.138889	
chr28	9118226	2	40	T:0.85	C:0.15	
chr28	9118408	2	38	T:0.868421	C:0.131579	

chr28	9118672	2	38	T:0.894737	A:0.105263	
chr28	9118674	2	38	C:0.894737	A:0.105263	
chr28	9118781	3	36	CT:0.361111	C:0.361111	CTT:0.277778
chr28	9118803	2	40	TTTTA:0.5	T:0.5	
chr28	9119002	2	38	G:1	T:0	
chr28	9119203	2	36	G:0.833333	A:0.166667	
chr28	9119345	3	38	TA:0.5	T:0.184211	TAAA:0.315789
chr28	9119362	2	38	A:0.894737	AT:0.105263	
chr28	9119470	2	38	T:0.894737	A:0.105263	
chr28	9119870	3	36	AT:0.583333	A:0	ATGTTTT:0.416667
chr28	9120006	2	36	G:0.888889	A:0.111111	
chr28	9120038	2	38	A:0.552632	G:0.447368	
chr28	9120081	2	36	C:0.833333	T:0.166667	
chr28	9120109	2	36	C:0.722222	T:0.277778	
chr28	9120303	2	40	C:0.85	T:0.15	
chr28	9120801	2	40	C:0.5	T:0.5	
chr28	9121069	2	40	G:0.825	A:0.175	
chr28	9121180	2	36	C:0.861111	CT:0.138889	
chr28	9121516	2	34	A:0.617647	G:0.382353	
chr28	9122021	2	40	AGGACATTTGGGCAGTGATGTTTTT:0.725	A:0.275	
chr28	9122067	2	40	T:0.825	TA:0.175	
chr28	9122560	2	36	C:0.75	T:0.25	
chr28	9122818	2	40	G:0.9	A:0.1	
chr28	9122925	2	38	A:0.894737	C:0.105263	
chr28	9123875	2	40	C:0.85	T:0.15	
chr28	9123928	2	40	C:0.85	T:0.15	
chr28	9123957	2	38	T:0.578947	C:0.421053	
chr28	9124006	2	40	CCTCTCTCT:0.875	C:0.125	
chr28	9124042	2	40	A:0.85	T:0.15	
chr28	9124060	2	40	A:0.85	C:0.15	
chr28	9124061	2	40	C:1	A:0	
chr28	9124281	2	40	T:0.8	C:0.2	
chr28	9124572	2	36	T:0.861111	TC:0.138889	
chr28	9124752	2	38	T:0.868421	C:0.131579	
chr28	9124828	3	28	CT:0.357143	C:0.464286	CTT:0.178571
chr28	9124843	2	36	T:0.861111	A:0.138889	
chr28	9124899	2	26	C:0.923077	G:0.0769231	
chr28	9124901	2	26	C:0.923077	G:0.0769231	
chr28	9124972	2	38	G:0.842105	A:0.157895	
chr28	9124982	2	38	G:0.842105	A:0.157895	
chr28	9125096	2	40	A:0.875	G:0.125	
chr28	9126738	2	38	T:0.868421	C:0.131579	
chr28	9127083	2	36	C:0.888889	T:0.111111	
chr28	9127129	2	40	CCTCT:1	C:0	
chr28	9127181	2	40	TTAAAA:0.975	T:0.025	
chr28	9127226	2	40	A:0.85	C:0.15	
chr28	9127250	2	40	G:0.85	GA:0.15	
chr28	9127363	2	40	T:0.85	A:0.15	
chr28	9127803	2	40	T:0.9	TA:0.1	

chr28	9127952	2	38	A:0.921053	G:0.0789474	
chr28	9128086	2	40	T:0.675	C:0.325	
chr28	9128585	2	40	G:0.9	T:0.1	
chr28	9128938	2	38	T:0.815789	C:0.184211	
chr28	9129276	2	40	C:0.825	T:0.175	
chr28	9129297	2	40	C:0.85	A:0.15	
chr28	9129460	2	36	A:0.888889	G:0.111111	
chr28	9129912	2	40	T:0.85	C:0.15	
chr28	9129963	2	38	C:0.526316	T:0.473684	
chr28	9129964	2	38	A:0.526316	G:0.473684	
chr28	9130261	2	36	T:0.916667	C:0.0833333	
chr28	9130795	2	38	C:0.710526	T:0.289474	
chr28	9130813	2	36	T:0.694444	C:0.305556	
chr28	9130936	2	40	T:0.85	C:0.15	
chr28	9130939	2	40	A:0.85	G:0.15	
chr28	9131645	2	20	G:0.85	A:0.15	
chr28	9132326	2	32	T:0.375	C:0.625	
chr28	9132540	2	40	T:0.775	C:0.225	
chr28	9134968	2	38	C:0.894737	T:0.105263	
chr28	9135012	2	28	TA:0.464286	T:0.535714	
chr28	9135153	2	38	C:0.789474	T:0.210526	
chr28	9135492	3	34	TA:0.411765	T:0.411765	TAAA:0.176471
chr28	9135625	2	34	T:0.882353	A:0.117647	
chr28	9136723	2	40	CAT:1	C:0	
chr28	9136768	2	38	T:0.868421	G:0.131579	
chr28	9136846	2	40	A:0.875	T:0.125	
chr28	9136848	2	40	G:0.875	T:0.125	
chr28	9136969	2	40	G:0.875	A:0.125	
chr28	9137075	2	36	CT:0.805556	C:0.194444	
chr28	9137096	2	38	TTTTA:0.657895	T:0.342105	
chr28	9137144	2	38	T:0.894737	C:0.105263	
chr28	9137614	2	38	A:1	C:0	
chr28	9138170	3	40	C:0.65	CCTCT:0.275	CCTCTCTCTCTCTCTCT:0.075
chr28	9138203	2	38	GAATA:0.894737	G:0.105263	
chr28	9138235	2	36	T:0.138889	TA:0.861111	
chr28	9139044	2	38	A:0.947368	G:0.0526316	
chr28	9139062	2	38	C:0.631579	CTTCT:0.368421	
chr28	9139324	2	40	C:0.9	CACAGAGAG:0.1	
chr28	9139613	2	36	G:1	C:0	
chr28	9139644	2	38	G:1	T:0	
chr28	9139804	2	38	G:0.684211	T:0.315789	
chr28	9139909	2	38	C:0.921053	G:0.0789474	
chr28	9140077	2	40	A:1	T:0	
chr28	9140530	2	40	G:0.65	C:0.35	
chr28	9140720	2	40	G:0.85	A:0.15	
chr28	9140804	2	38	C:0.842105	CTG:0.157895	
chr28	9141197	2	40	T:0.925	TA:0.075	
chr28	9141271	2	38	A:0.868421	T:0.131579	
chr28	9141396	2	38	G:0.815789	C:0.184211	
chr28	9142392	2	34	G:0.852941	A:0.147059	
chr28	9142600	2	36	A:0.861111	T:0.138889	
chr28	9142635	2	36	A:0.5	AT:0.5	

chr28	9142668	2	38	TAGAC:0.921053	T:0.0789474
chr28	9142680	3	40	AAGAGAGAG:0.625	A:0.1
AAGAGAGAGAG:0.275
chr28	9142841	3	34	TCC:0.470588	T:0.411765
TC:0.117647
chr28	9142881	2	36	T:0.944444	TA:0.0555556
chr28	9143006	2	40	C:0.575	T:0.425
chr28	9143377	3	40	AT:0.6	A:0.3	ATT:0.1
chr28	9143426	2	40	CAGAGAGAG:0.775	C:0.225
chr28	9143525	2	38	A:0.578947	G:0.421053
chr28	9143900	2	36	TA:0.777778	T:0.222222
chr28	9144440	2	38	G:0.526316	T:0.473684
chr28	9144446	2	38	G:0.868421	A:0.131579
chr28	9144686	2	40	A:0.9	T:0.1
chr28	9145028	2	40	TGAAATATCTGGAAAAAAAAAAAAAAA:0.675
T:0.325
chr28	9145032	2	40	ATATC:0.9	A:0.1
chr28	9145038	2	40	GGAAAAAAAAAAAAAAAGAAATA:0.9
G:0.1
chr28	9145148	2	32	CT:0.59375	C:0.40625
chr28	9145161	2	38	T:0.526316	A:0.473684
chr28	9145164	4	40	GATTTATTT:0.65	G:0.175	GATTT:
0.075	GATTTATTTATTT:0.1
chr28	9145226	2	40	T:0.95	TGAGA:0.05
chr28	9145643	2	40	C:0.825	CAAGAT:0.175
chr28	9145790	2	38	CTCT:0.868421	C:0.131579
chr28	9145810	2	38	T:0.868421	A:0.131579
chr28	9145957	2	40	CACG:0.7	C:0.3
chr28	9146538	2	40	G:0.675	A:0.325
chr28	9146594	2	36	ATC:0.722222	A:0.277778
chr28	9146977	2	34	T:0.823529	A:0.176471
chr28	9147023	2	34	G:1	T:0
chr28	9147135	2	36	TC:0.444444	T:0.555556
chr28	9147162	2	36	T:0.777778	C:0.222222
chr28	9147164	2	36	T:0.777778	C:0.222222
chr28	9147406	2	28	A:0.821429	G:0.178571
chr28	9149465	2	36	TA:0.527778	T:0.472222
chr28	9150836	2	36	GT:0.777778	G:0.222222
chr28	9153248	2	40	C:1	T:0
chr28	9157090	2	40	GT:0.8	G:0.2
chr28	9157133	2	40	CAGAG:1	C:0
chr28	9158152	2	36	AT:1	A:0
chr28	9158201	2	36	GGA:0.833333	G:0.166667
chr28	9158997	2	38	TA:0.921053	T:0.0789474
chr28	9159272	3	40	AAG:0.775	A:0.175	AAGAGAG:
0.05
chr28	9160165	2	40	T:0.85	TA:0.15
chr28	9160327	2	36	CTG:0.166667	C:0.833333
chr28	9160429	2	38	C:0.631579	T:0.368421
chr28	9160757	2	38	AC:1	A:0
chr28	9161374	2	38	T:1	A:0
chr28	9161866	2	40	C:1	T:0
chr28	9162302	2	38	GGA:0.921053	G:0.0789474
chr28	9163197	2	40	GA:0.05	G:0.95

chr28	9163247	2	40	A:0.95	G:0.05
chr28	9164020	2	40	C:1
CTTTGTTCTTCTTTTTTTTTTTTTTT:0
chr28	9164559	2	38	G:1	GT:0
chr28	9164798	2	38	TA:0.578947	T:0.421053
chr28	9165080	3	38	TA:0.763158	T:0.157895
AA:0.0789474
chr28	9165804	2	38	T:0.657895	C:0.342105
chr28	9166894	2	34	C:0.852941	CT:0.147059
chr28	9167364	2	32	C:0.21875	CT:0.78125
chr28	9167454	2	40	C:1	T:0
chr28	9168441	2	36	CAG:0.611111	C:0.388889
chr28	9168549	2	34	A:0.5	G:0.5
chr28	9169426	2	36	A:1	G:0
chr28	9170031	2	36	G:0.833333	A:0.166667
chr28	9170724	2	38	C:0.131579	CAGAG:0.868421
chr28	9171159	2	40	C:0.85	T:0.15
chr28	9175560	2	40	T:0.725	C:0.275
chr28	9175795	2	34	CT:0.823529	C:0.176471
chr28	9178631	2	40	T:1	C:0
chr28	9180681	2	36	A:1	G:0
chr28	9180806	2	38	C:0.631579	T:0.368421
chr28	9180948	2	38	T:1	G:0
chr28	9181107	2	40	C:1	T:0
chr28	9181265	2	40	C:1	T:0
chr28	9184306	3	34	CAG:0.705882	C:0.176471
CAGAG:0.117647
chr28	9187030	2	34	CAAAT:0.441176	C:0.558824
chr28	9187804	2	38	G:0.763158	A:0.236842
chr28	9188964	2	40	T:0.575	C:0.425
chr28	9190252	2	34	CT:0.735294	C:0.264706
chr28	9190810	2	36	G:1	A:0
chr28	9191746	2	40	G:0	T:1
chr28	9194302	2	40	C:1	T:0
chr28	9195381	2	40	T:1	G:0
chr28	9195814	2	40	G:1	A:0
chr28	9197247	2	40	G:1	A:0
chr28	9200444	2	40	G:0.65	A:0.35
chr28	9200919	2	34	CT:0.852941	C:0.147059
chr28	9202338	2	38	C:0.921053	T:0.0789474
chr28	9202393	2	36	AT:0.472222	A:0.527778
chr28	9203008	4	40	CTTCTTTCT:0.425	C:0.075	CTTCT:
0.4	CTTCTTTCTTTCT:0.1
chr28	9203378	2	38	GT:0.763158	G:0.236842
chr28	9204202	2	40	GAC:0.775	G:0.225
chr28	9205805	2	38	CTTCTTCTTCTTCTCT:0.736842	C:
0.263158
chr28	9205814	2	38	CTTCTCT:1	C:0
chr28	9208416	2	40	T:1	A:0
chr28	9208559	2	38	C:1	T:0
chr28	9208833	2	38	C:0.894737	T:0.105263
chr28	9210336	2	36	C:1	T:0
chr28	9211793	2	32	AC:0	A:1
chr28	9214201	2	36	G:1	A:0

chr28 9218725 2 40 C:1 T:0
chr28 9220104 2 38 A:0.973684 AATTC:0.0263158
chr28 9221330 2 36 T:0.166667 C:0.833333
chr28 9222795 2 38 C:1 T:0
chr28 9224068 2 40 G:0.1 A:0.9
chr28 9224369 2 38 A:0.921053 G:0.0789474
chr28 9225820 2 36 C:0.833333 G:0.166667
chr28 9225982 2 38 T:1 G:0
chr28 9226256 2 40 TCAAA:0.85 T:0.15
chr28 9226379 2 40 G:0.875 A:0.125
chr28 9226462 2 36 C:1 T:0
chr28 9229209 2 40 C:1 T:0
chr28 9231342 3 38 C:0.315789 CT:0.210526
CTT:0.473684
chr28 9234080 2 38 C:1 T:0
chr28 9235371 2 36 G:0.888889 A:0.111111
chr28 9235627 2 40 G:1 C:0
chr28 9236536 3 34 T:0.5 TTG:0.264706 TTGTG:
0.235294
chr28 9241046 2 34 T:0.852941 TAC:0.147059
chr28 9241915 2 40 G:0.9 T:0.1
chr28 9242810 2 38 C:1 T:0
chr28 9245246 2 38 G:1 A:0
chr28 9245562 2 40 C:1 CAG:0
chr28 9245594 2 40 GAAACATAGGC:0.9 G:0.1
chr28 9245609 2 40 G:0.9 A:0.1
chr28 9246068 3 30 CT:0.533333 C:0.333333
CTT:0.133333
chr28 9249971 2 32 GA:0.8125 G:0.1875
chr28 9252355 2 40 G:0.65 GC:0.35
chr28 9255857 2 38 A:0.921053 G:0.0789474
chr28 9256832 2 40 T:0.975 C:0.025
chr28 9258279 2 38 T:1 TTTTC:0
chr28 9260451 2 38 TA:0.631579 T:0.368421
chr28 9262984 2 26 C:1 A:0
chr28 9263081 2 34 T:1 A:0
chr28 9263695 2 38 C:0 A:1
chr28 9266238 4 40 TAAATAAAATA:0.375 T:0.275
TAAATA:0 TAAATAAAATAAAATA:0.35
chr28 9266559 2 40 C:1 T:0
chr28 9268562 3 36 T:0.833333 TCA:0.0833333
TCACA:0.0833333
chr28 9270413 2 40 T:0.8 TA:0.2
chr28 9270532 2 40 C:0.925 G:0.075
chr28 9271166 3 40 AAG:0.775 A:0.225 AAGAGAG:
0
chr28 9274164 2 40 A:0.85 T:0.15
chr28 9274623 2 38 G:1 A:0
chr28 9274782 3 38 TTTTA:0.736842 T:0.105263
TTTTATTTA:0.157895
chr28 9276305 2 36 G:1 A:0
chr28 9279792 3 40 TTCTCTCTCTCTC:0.35 T:0.175
TTCTCTC:0.475
chr28 9279868 2 38 A:0.789474 T:0.210526

chr28	9279890	2	38	T:0.131579	TA:0.868421	
chr28	9282265	2	38	C:1	T:0	
chr28	9286422	2	40	A:0.95	G:0.05	
chr28	9289479	2	38	G:0	GT:1	
chr28	9289501	2	38	AG:0	A:1	
chr28	9289508	2	38	C:0	CA:1	
chr28	9289522	2	40	CT:0	C:1	
chr28	9289527	2	40	C:0	CA:1	
chr28	9289532	2	40	AT:0	A:1	
chr28	9289540	2	40	A:0	AC:1	
chr28	9289550	2	40	G:0	C:1	
chr28	9289551	2	40	C:0	T:1	
chr28	9289563	2	38	A:0	AT:1	
chr28	9289582	2	40	T:0	TTG:1	
chr28	9289586	2	40	A:0	AG:1	
chr28	9289594	2	40	A:0	AT:1	
chr28	9289602	2	40	C:0	CT:1	
chr28	9289611	2	40	C:0	CT:1	
chr28	9289613	2	40	A:0	T:1	
chr28	9289628	2	38	T:0	C:1	
chr28	9289678	2	38	G:0	GA:1	
chr28	9289765	3	36	GT:0.638889	G:0.194444	GTT:0.166667
chr28	9291028	2	38	G:0.842105	GA:0.157895	
chr28	9292480	2	36	A:1	G:0	
chr28	9292702	2	36	A:0.638889	AT:0.361111	
chr28	9293543	2	36	A:1	T:0	
chr28	9294590	2	38	CT:0.815789	C:0.184211	
chr28	9295280	2	36	C:0.972222	CTGTG:0.0277778	
chr28	9296459	2	36	C:0.111111	A:0.888889	
chr28	9297124	2	40	TG:0	T:1	
chr28	9297705	2	38	CT:0.736842	C:0.263158	
chr28	9297733	2	38	T:0.763158	A:0.236842	
chr28	9297746	2	34	T:0.647059	G:0.352941	
chr28	9299280	2	34	T:0.823529	TA:0.176471	
chr28	9302028	2	34	G:1	A:0	
chr28	9303306	2	38	C:1	T:0	
chr28	9307062	3	36	TA:0.555556	T:0.25	TAA:0.194444
chr28	9307815	2	36	C:1	T:0	
chr28	9310023	3	38	TA:0.868421	T:0.105263	TAA:0.0263158
chr28	9310572	2	36	G:0.833333	A:0.166667	
chr28	9313118	2	38	C:1	T:0	
chr28	9313791	2	36	TA:0.916667	T:0.0833333	
chr28	9314189	2	40	A:1	T:0	
chr28	9314438	2	36	CATATATATAT:0.416667	C:0.583333	
chr28	9315188	2	38	AC:0.815789	A:0.184211	
chr28	9315208	2	36	A:1	G:0	
chr28	9318528	2	26	TA:0.961538	T:0.0384615	
chr28	9320292	2	40	G:1	A:0	
chr28	9320305	2	38	TG:1	T:0	
chr28	9320833	2	28	G:1	GCA:0	

```
chr28	9322001	2	38	A:1	G:0
chr28	9323951	2	36	G:0	A:1
chr28	9325691	2	40	A:1	G:0
chr28	9326224	2	40	C:0.525	T:0.475
chr28	9330049	2	36	CCT:1	C:0
chr28	9330093	2	38	TA:0.868421	T:0.131579
chr28	9338162	2	38	CT:0.578947	C:0.421053
chr28	9338671	2	40	T:0.6	C:0.4
chr28	9339342	2	40	T:0	C:1
chr28	9340687	2	38	A:0.815789	C:0.184211
chr28	9342346	2	40	CAGAG:1	C:0
chr28	9342381	2	38	G:1	A:0
chr28	9344449	2	40	T:0.7	G:0.3
chr28	9346223	2	40	C:1	CAATA:0
chr28	9346437	2	40	GA:0.7	G:0.3
chr28	9347895	3	36	T:0.138889	TTTTA:0.75
TTTTATTTA:0.111111
chr28	9349792	2	38	T:1	C:0
chr28	9350405	2	36	A:1	G:0
chr28	9351340	4	38	TATAAATAA:0.236842	T:0.5
TATAA:0	TATAAATAAATAA:0.263158
chr28	9352037	2	40	G:0.85	C:0.15
chr28	9355014	2	38	CCT:0.894737	C:0.105263
chr28	9356156	2	34	TA:0.764706	T:0.235294
chr28	9356483	2	36	CT:0.833333	C:0.166667
chr28	9357139	3	38	AT:0.447368	A:0.368421
ATT:0.184211
chr28	9357371	2	40	C:0	A:1
chr28	9357778	2	38	T:1	TA:0
chr28	9359170	2	40	A:1	T:0
chr28	9359227	2	40	GGCAGAGGGAGAA:0.95	G:0.05
chr28	9359764	2	36	TA:0.861111	T:0.138889
chr28	9360813	2	38	C:1	T:0
chr28	9361289	2	36	T:0	C:1
chr28	9361760	2	36	CTG:0.888889	C:0.111111
chr28	9361762	2	36	G:0.861111	C:0.138889
chr28	9362169	2	38	G:1	A:0
chr28	9363389	2	40	T:1	G:0
chr28	9363703	2	40	AAAAGAAAG:0.925	A:0.075
chr28	9363721	3	40	AAGAAAGAAAAAG:0.75	A:0.05
AAAAGAAAAAG:0.2
chr28	9363727	2	40	GAA:1	G:0
chr28	9363729	2	40	A:0.65	AAG:0.35
chr28	9364175	2	40	T:0.55	C:0.45
chr28	9364724	2	38	G:1	A:0
chr28	9365482	2	40	CAA:1	C:0
chr28	9365955	2	40	C:0	T:1
chr28	9366573	2	34	T:1	A:0
chr28	9367828	2	40	A:0	T:1
chr28	9367875	2	36	TA:0.777778	T:0.222222
chr28	9367881	2	36	G:0.777778	C:0.222222
chr28	9368763	2	30	TA:0.9	T:0.1
chr28	9370512	2	38	A:0.842105	G:0.157895
chr28	9371129	2	26	G:0.153846	C:0.846154
```

chr28	9371139	2	26	TCCGCGCCGCCCGCGCCGC:0.192308	T:0.807692
chr28	9371311	2	32	C:0.34375	G:0.65625
chr28	9371383	2	36	G:0.0277778	T:0.972222
chr28	9371461	2	34	C:0.882353	G:0.117647
chr28	9372170	2	40	A:1	G:0
chr28	9372185	2	40	A:1	G:0
chr28	9372781	2	38	CT:0.710526	C:0.289474
chr28	9374018	2	38	G:0.894737	C:0.105263
chr28	9374197	2	34	T:1	C:0
chr28	9374225	2	36	T:0.972222	TTC:0.0277778
chr28	9374408	2	36	C:1	T:0
chr28	9375292	2	36	A:0	C:1
chr28	9375559	2	34	C:1	CCTCT:0
chr28	9376063	2	40	A:0.825	T:0.175
chr28	9376620	2	38	A:0.973684	T:0.0263158
chr28	9376724	2	40	A:0.95	G:0.05
chr28	9377246	2	38	TCA:1	T:0
chr28	9377362	2	40	T:0.95	A:0.05
chr28	9377406	2	40	G:0.975	A:0.025
chr28	9378066	2	40	TA:0.875	T:0.125
chr28	9378352	2	40	T:0.95	C:0.05
chr28	9379155	2	38	CT:0.842105	C:0.157895
chr28	9379714	2	40	AT:0.675	A:0.325
chr28	9379904	2	36	G:0.972222	A:0.0277778
chr28	9379915	2	38	G:0.921053	A:0.0789474
chr28	9381902	2	40	G:0.975	A:0.025
chr28	9382377	2	34	C:0.941176	T:0.0588235
chr28	9382570	2	40	T:0.95	C:0.05
chr28	9382869	2	40	A:0.95	G:0.05
chr28	9383106	2	40	T:0.95	C:0.05
chr28	9383420	2	38	G:0.947368	T:0.0526316
chr28	9383516	2	38	G:0.947368	A:0.0526316
chr28	9383681	2	38	G:0.868421	A:0.131579
chr28	9384144	2	40	G:0.9	T:0.1
chr28	9384153	2	40	T:0.9	C:0.1
chr28	9384469	2	40	C:0.9	CT:0.1
chr28	9384854	2	38	C:0.921053	G:0.0789474
chr28	9384896	2	40	CT:0.975	C:0.025
chr28	9385165	2	36	A:0.972222	G:0.0277778
chr28	9385324	2	38	TG:0.947368	T:0.0526316
chr28	9385492	2	38	G:0.947368	GT:0.0526316
chr28	9385817	2	40	TAA:0.975	T:0.025
chr28	9385948	2	40	A:0.95	G:0.05
chr28	9386281	2	34	AGCT:0.911765	A:0.0882353
chr28	9386287	2	34	T:0.911765	A:0.0882353
chr28	9387287	2	38	C:0.973684	T:0.0263158
chr28	9387331	2	38	C:0.921053	T:0.0789474
chr28	9387708	2	38	A:0.921053	T:0.0789474
chr28	9387720	2	38	A:0.921053	T:0.0789474
chr28	9387722	2	38	T:0.921053	TC:0.0789474
chr28	9388020	2	36	T:0.944444	A:0.0555556
chr28	9388110	2	38	C:0.947368	T:0.0526316
chr28	9388356	2	40	C:0.925	A:0.075

chr28	9388423	2	40	T:0.95	A:0.05	
chr28	9388498	2	38	AT:0.947368	A:0.0526316	
chr28	9388688	2	38	G:0.947368	A:0.0526316	
chr28	9388702	2	38	A:0.947368	G:0.0526316	
chr28	9388808	2	40	CTTCTG:0.975	C:0.025	
chr28	9388923	2	40	G:1	A:0	
chr28	9389027	2	36	C:0.944444	T:0.0555556	
chr28	9389031	2	36	A:0.944444	G:0.0555556	
chr28	9389127	2	40	T:1	G:0	
chr28	9389456	2	38	TA:0.815789	T:0.184211	
chr28	9389930	2	40	G:0.95	A:0.05	
chr28	9390113	2	38	C:0.973684	G:0.0263158	
chr28	9390141	2	40	GTCTCTGCCTCTCTCTCTC:0.425	G:0.575	
chr28	9390148	2	40	C:0.875	CCT:0.125	
chr28	9390206	2	38	AT:0.947368	A:0.0526316	
chr28	9390620	3	40	CTTT:0.125	C:0.1	CTTTT:0.775
chr28	9390863	2	40	G:1	A:0	
chr28	9390877	2	40	A:0.95	G:0.05	
chr28	9391347	2	40	G:0.95	T:0.05	
chr28	9391436	2	38	A:0.947368	G:0.0526316	
chr28	9391460	2	36	C:1	A:0	
chr28	9391949	2	36	TAA:0.777778	T:0.222222	
chr28	9392036	2	38	T:0.947368	C:0.0526316	
chr28	9392240	2	40	T:0.95	C:0.05	
chr28	9392571	2	40	G:0.95	A:0.05	
chr28	9392725	2	40	A:0.95	G:0.05	
chr28	9392832	2	40	G:0.95	T:0.05	
chr28	9393098	2	38	CGTTT:0.947368	C:0.0526316	
chr28	9393371	2	40	G:0.95	A:0.05	
chr28	9393865	2	34	A:0.941176	AAT:0.0588235	
chr28	9393906	2	36	A:0.916667	AC:0.0833333	
chr28	9394201	2	34	C:0.911765	T:0.0882353	
chr28	9394334	2	36	T:0.916667	TA:0.0833333	
chr28	9394397	2	40	C:0.95	CT:0.05	
chr28	9394399	2	40	GCCCAGTTAA:0.95	G:0.05	
chr28	9394573	2	36	G:1	A:0	
chr28	9394893	2	38	C:0.894737	G:0.105263	
chr28	9394988	2	34	C:0.852941	CT:0.147059	
chr28	9395160	2	38	T:0.921053	A:0.0789474	
chr28	9395301	2	40	G:0.95	T:0.05	
chr28	9395358	2	40	G:0.8	T:0.2	
chr28	9397180	2	32	TA:0.9375	T:0.0625	
chr28	9397221	2	38	T:0.947368	TG:0.0526316	
chr28	9397328	2	40	C:0.975	A:0.025	
chr28	9397335	2	40	T:0.975	A:0.025	
chr28	9397356	2	36	CT:0.972222	C:0.0277778	
chr28	9397587	2	38	C:0.947368	T:0.0526316	
chr28	9397666	2	40	A:1	T:0	
chr28	9398030	2	40	A:0.975	G:0.025	
chr28	9398401	2	40	TA:0.95	T:0.05	
chr28	9398419	2	40	T:0.95	TA:0.05	
chr28	9398455	2	40	T:0.95	G:0.05	
chr28	9398642	2	40	A:0.975	T:0.025	

chr28	9398643	2	40	A:0.975	C:0.025	
chr28	9398660	2	40	A:0.975	G:0.025	
chr28	9398733	2	38	C:0.947368	T:0.0526316	
chr28	9399036	2	34	G:0.970588	A:0.0294118	
chr28	9399350	2	40	CCT:0.375	C:0.625	
chr28	9399378	2	40	AAATT:1	A:0	
chr28	9399604	2	38	T:0.947368	C:0.0526316	
chr28	9399605	2	38	G:0.947368	A:0.0526316	
chr28	9399623	2	38	G:0.947368	C:0.0526316	
chr28	9399641	2	38	T:0.947368	C:0.0526316	
chr28	9399673	2	36	G:0.944444	A:0.0555556	
chr28	9399755	3	36	TA:0.75	T:0.111111	TAA:0.138889
chr28	9399791	2	40	A:0.95	C:0.05	
chr28	9399927	2	40	A:0.95	C:0.05	
chr28	9400413	2	38	C:0.947368	T:0.0526316	
chr28	9400472	2	40	T:0.925	TG:0.075	
chr28	9400654	2	38	G:0.973684	A:0.0263158	
chr28	9400813	2	38	GA:0.947368	G:0.0526316	
chr28	9400892	2	36	C:0.944444	T:0.0555556	
chr28	9401088	2	40	C:0.95	A:0.05	
chr28	9401781	2	40	A:0.975	ATGTGTGTGTG:0.025	
chr28	9401846	2	38	G:0.947368	A:0.0526316	
chr28	9401855	2	36	G:0.916667	A:0.0833333	
chr28	9402130	2	36	A:0.888889	AT:0.111111	
chr28	9402175	3	36	CT:0.305556	C:0.583333	CTT:0.111111
chr28	9402275	2	38	T:0.947368	C:0.0526316	
chr28	9402316	2	38	T:0.947368	C:0.0526316	
chr28	9402515	3	36	ATC:0.833333	A:0.111111	ATTCTC:0.0555556
chr28	9402517	2	36	C:1	T:0	
chr28	9403020	2	36	A:0.944444	AT:0.0555556	
chr28	9403055	2	36	AAC:0.944444	A:0.0555556	
chr28	9403414	2	38	T:0.947368	C:0.0526316	
chr28	9403486	2	38	T:0.947368	C:0.0526316	
chr28	9403716	2	38	A:0.947368	G:0.0526316	
chr28	9403888	2	38	C:0.815789	T:0.184211	
chr28	9403982	2	40	G:0.95	C:0.05	
chr28	9404223	2	38	AAT:0.578947	A:0.421053	
chr28	9404561	2	38	G:0.947368	A:0.0526316	
chr28	9404634	2	38	C:0.947368	T:0.0526316	
chr28	9404838	2	38	T:0.947368	C:0.0526316	
chr28	9404885	2	40	T:0.95	G:0.05	
chr28	9405333	2	40	A:0.975	G:0.025	
chr28	9405405	2	36	C:0.944444	A:0.0555556	
chr28	9405444	2	40	G:0.95	T:0.05	
chr28	9405823	2	40	A:0.925	G:0.075	
chr28	9405940	2	40	G:0.975	GA:0.025	
chr28	9406050	2	36	CT:0.916667	C:0.0833333	
chr28	9406264	2	38	G:0.973684	A:0.0263158	
chr28	9406371	2	40	C:0.975	G:0.025	
chr28	9406496	2	40	T:0.925	G:0.075	
chr28	9406604	2	38	A:0.947368	G:0.0526316	

```
chr28	9406877	2	36	C:0.944444	T:0.0555556
chr28	9406943	2	38	G:0.947368	A:0.0526316
chr28	9407001	2	38	GTC:0.921053	G:0.0789474
chr28	9407048	2	38	TA:0.921053	T:0.0789474
chr28	9407144	2	38	G:0.921053	A:0.0789474
chr28	9407251	2	36	G:0.944444	A:0.0555556
chr28	9407317	2	40	CT:0.9	C:0.1
chr28	9407363	2	38	C:0.921053	CAGAG:0.0789474
chr28	9407435	2	38	T:0.921053	C:0.0789474
chr28	9407708	2	40	A:0.975	G:0.025
chr28	9408117	2	38	A:0.947368	G:0.0526316
chr28	9408844	2	32	T:0.9375	C:0.0625
chr28	9409094	5	40	TATAA:0.325	T:0.225
TATAAATAA:0.325	TATAAATAAATAA:0.125	TATAAATAAATAAATAA:0
chr28	9409136	2	40	T:0.925	TAAATAAAA:0.075
chr28	9409832	2	38	A:0.947368	G:0.0526316
chr28	9412296	2	38	G:0.947368	A:0.0526316
chr28	9412365	2	40	C:0.95	T:0.05
chr28	9412764	2	40	T:0.925	A:0.075
chr28	9413209	2	36	C:0.972222	CCT:0.0277778
chr28	9414925	2	40	TA:0.9	T:0.1
chr28	9414938	2	40	TTAA:0.975	T:0.025
chr28	9414939	2	38	TA:0.0526316	T:0.947368
chr28	9414983	2	40	A:0	G:1
chr28	9415014	2	40	A:0	G:1
chr28	9415036	2	40	A:0	G:1
chr28	9415125	2	34	A:0	C:1
chr28	9415544	2	38	T:0.947368	G:0.0526316
chr28	9416238	2	40	C:0.45	CA:0.55
chr28	9416347	2	40	C:0.45	CA:0.55
chr28	9416349	2	40	C:0.45	CA:0.55
chr28	9416351	2	40	G:0.45	GA:0.55
chr28	9416355	2	40	C:0.45	CT:0.55
chr28	9417023	2	40	GTTA:1	G:0
chr28	9417266	2	40	G:0.975	A:0.025
chr28	9417400	2	40	C:0.95	T:0.05
chr28	9417497	2	40	C:0.975	G:0.025
chr28	9418472	2	38	A:0.947368	T:0.0526316
chr28	9418536	2	38	A:0.947368	G:0.0526316
chr28	9419145	2	38	T:0.973684	C:0.0263158
chr28	9419440	3	36	CATATAT:0.527778	C:0.0833333
CATAT:0.388889
chr28	9420740	2	40	T:0.9	A:0.1
chr28	9422117	3	38	TAA:0.0526316	T:0.131579
TA:0.815789
chr28	9422780	2	40	GTTTGTT:0.95	G:0.05
chr28	9423180	2	38	T:0.947368	A:0.0526316
chr28	9423467	2	38	A:0.973684	G:0.0263158
chr28	9423648	2	40	C:0.95	T:0.05
chr28	9423833	2	38	C:1	CT:0
chr28	9423839	2	36	T:1	TG:0
chr28	9423840	2	38	G:0.789474	T:0.210526
chr28	9425194	2	38	T:0.947368	G:0.0526316
chr28	9425684	2	40	C:0.95	T:0.05
```

chr28 9425738 2 40 C:0.95 A:0.05
chr28 9425879 2 40 TA:0.975 T:0.025
chr28 9425882 2 40 T:0.975 C:0.025
chr28 9425956 2 40 G:0.95 T:0.05
chr28 9426387 2 38 A:0.973684 G:0.0263158
chr28 9426951 2 40 T:1 A:0
chr28 9427212 2 40 C:0.95 T:0.05
chr28 9427415 2 36 A:0.944444 C:0.0555556
chr28 9427767 2 40 A:0.95 T:0.05
chr28 9428233 2 40 T:0.975 C:0.025
chr28 9428369 2 40 G:0.95 A:0.05
chr28 9428449 2 40 G:0.95 C:0.05
chr28 9428465 2 40 C:0.975 T:0.025
chr28 9428611 2 36 ATCTCCCTGGTG:0 A:1
chr28 9428625 2 36 TATCTTTC:0 T:1
chr28 9428633 2 36 C:0 CAAAAAAAAAAAAAAAAA:1
chr28 9428636 2 36 C:0 A:1
chr28 9428727 2 36 C:0 A:1
chr28 9428782 2 36 G:0 A:1
chr28 9428833 2 38 C:0.947368 T:0.0526316
chr28 9428852 2 40 C:0 CA:1
chr28 9428890 2 40 G:0.95 C:0.05
chr28 9428914 2 40 T:0.95 C:0.05
chr28 9428931 2 40 T:0 A:1
chr28 9429394 2 40 A:0.95 T:0.05
chr28 9429401 2 40 A:0.95
ATAAATAAATAAATAAATAAATAAATAAATAAAT:0.05
chr28 9429409 2 40 G:0.95 T:0.05
chr28 9429422 2 40 C:0.95 G:0.05
chr28 9429460 2 38 G:0.947368 A:0.0526316
chr28 9429511 2 36 G:0.944444 C:0.0555556
chr28 9429518 2 36 A:1 G:0
chr28 9429977 2 38 C:0.947368 T:0.0526316
chr28 9430651 2 32 TA:0.875 T:0.125
chr28 9431418 2 40 T:0.95 G:0.05
chr28 9431600 2 40 T:0.95 C:0.05
chr28 9431728 2 40 TG:0.95 T:0.05
chr28 9431746 2 40 G:0.95 T:0.05
chr28 9431863 2 36 C:0.944444 T:0.0555556
chr28 9431864 2 36 C:0.944444 G:0.0555556
chr28 9431869 2 36 G:0.944444 T:0.0555556
chr28 9432029 2 40 G:0.975 GAC:0.025
chr28 9432086 2 38 G:0.947368 C:0.0526316
chr28 9432105 2 38 C:0.947368 T:0.0526316
chr28 9432107 2 38 T:0.947368 A:0.0526316
chr28 9432244 2 40 C:1 T:0
chr28 9432247 2 40 C:1
CGGGATCCCTGGGTGGCGCAGCGGTTT:0
chr28 9432303 2 38 T:0.947368 C:0.0526316
chr28 9432351 2 40 A:0.95 G:0.05
chr28 9432764 2 38 A:0.947368 T:0.0526316
chr28 9432963 2 38 G:1 C:0
chr28 9433378 2 40 CAAAT:0.95 C:0.05
chr28 9433708 2 40 T:0.95 C:0.05

chr28	9433904	2	38	C:0.973684	T:0.0263158	
chr28	9434848	2	38	C:0.947368	T:0.0526316	
chr28	9435007	2	36	G:0.944444	A:0.0555556	
chr28	9435282	2	40	A:0.95	T:0.05	
chr28	9435329	2	40	G:0.95	A:0.05	
chr28	9435369	2	40	G:0.9	T:0.1	
chr28	9435417	2	40	A:0.925	C:0.075	
chr28	9435633	2	38	G:0.921053	A:0.0789474	
chr28	9435984	2	36	G:0.75	GTT:0.25	
chr28	9436029	2	40	T:0.95	C:0.05	
chr28	9436182	2	38	C:0.947368	T:0.0526316	
chr28	9436385	2	40	A:0.975	G:0.025	
chr28	9436531	2	40	C:0.925	T:0.075	
chr28	9437044	2	40	GAAAAAAAGA:0.95	G:0.05	
chr28	9437167	3	40	G:0.85	GAAGA:0.1	GAAGAAAGAAAGAAAGA:0.05
chr28	9437220	2	40	AAG:0.95	A:0.05	
chr28	9438308	2	38	T:0.947368	C:0.0526316	
chr28	9438570	2	40	A:0.925	G:0.075	
chr28	9438598	2	40	A:0.95	G:0.05	
chr28	9439112	2	40	G:0.925	A:0.075	
chr28	9439383	2	38	A:0.947368	C:0.0526316	
chr28	9439813	2	40	C:0.95	T:0.05	
chr28	9439816	2	40	C:0.95	T:0.05	
chr28	9439872	2	40	G:0.95	A:0.05	
chr28	9440086	2	40	G:0.975	A:0.025	
chr28	9440269	2	38	T:0.947368	C:0.0526316	
chr28	9440301	2	38	C:0.947368	T:0.0526316	
chr28	9440404	2	34	T:0.852941	TA:0.147059	
chr28	9441330	2	40	T:0.95	C:0.05	
chr28	9441547	3	38	ATT:0.842105	A:0.0789474	AT:0.0789474
chr28	9441578	2	36	A:0.944444	T:0.0555556	
chr28	9441710	2	36	CAG:0.944444	C:0.0555556	
chr28	9441745	2	38	G:0.947368	T:0.0526316	
chr28	9441756	2	38	C:0.947368	T:0.0526316	
chr28	9442441	2	18	G:0	GC:1	
chr28	9442473	2	32	A:0	AG:1	
chr28	9442561	2	40	A:0	C:1	
chr28	9442666	2	40	G:1	A:0	
chr28	9442971	2	40	C:0.95	T:0.05	
chr28	9443556	2	38	CTTTT:0.973684	C:0.0263158	
chr28	9443584	2	38	C:0.973684	T:0.0263158	
chr28	9443764	2	38	A:0.947368	G:0.0526316	
chr28	9443880	2	38	A:0.921053	T:0.0789474	
chr28	9444011	2	40	G:0.95	A:0.05	
chr28	9444061	2	40	ATGT:0.95	A:0.05	
chr28	9444079	2	40	T:0.95	C:0.05	
chr28	9444233	2	38	T:1	A:0	
chr28	9444234	2	38	T:0.921053	A:0.0789474	
chr28	9444364	2	38	A:0.947368	T:0.0526316	
chr28	9444470	2	38	C:0.921053	G:0.0789474	
chr28	9445035	2	38	G:0.921053	A:0.0789474	
chr28	9445149	2	40	T:0.95	G:0.05	

chr28	9445583	2	40	GTTCATTTCTCC:1	G:0	
chr28	9445639	2	38	C:0	CA:1	
chr28	9445772	2	40	T:0.95	G:0.05	
chr28	9445921	2	38	C:0.947368	G:0.0526316	
chr28	9445978	2	40	GACAA:0.975	G:0.025	
chr28	9445984	2	40	C:0.975	T:0.025	
chr28	9446204	2	38	A:0.947368	G:0.0526316	
chr28	9446344	2	40	A:1	T:0	
chr28	9446623	3	40	GACAC:0.05	G:0.8	GAC:0.15
chr28	9447360	2	40	T:0.95	C:0.05	
chr28	9448027	2	36	C:0	CT:1	
chr28	9448308	2	38	T:0.710526	C:0.289474	
chr28	9448777	2	34	A:0.911765	G:0.0882353	
chr28	9448821	2	32	A:0.9375	T:0.0625	
chr28	9448828	2	32	G:0.9375	A:0.0625	
chr28	9449544	2	40	A:0.95	G:0.05	
chr28	9449897	2	40	G:0.9	A:0.1	
chr28	9449905	2	40	A:0.9	G:0.1	
chr28	9450169	2	38	A:0.947368	G:0.0526316	
chr28	9450262	2	40	A:0.95	G:0.05	
chr28	9450367	2	36	C:0.972222	T:0.0277778	
chr28	9450609	2	38	G:0.921053	A:0.0789474	
chr28	9450610	2	38	T:0.921053	A:0.0789474	
chr28	9450900	2	40	G:0.95	A:0.05	
chr28	9451000	2	38	T:0.947368	G:0.0526316	
chr28	9451020	2	38	A:0.947368	G:0.0526316	
chr28	9451201	2	40	T:0.95	C:0.05	
chr28	9451362	2	40	T:0.95	C:0.05	
chr28	9451900	2	40	G:1	T:0	
chr28	9452012	2	36	G:0.944444	A:0.0555556	
chr28	9452028	2	36	T:1	A:0	
chr28	9452129	2	38	T:0.921053	C:0.0789474	
chr28	9452233	2	36	A:0.972222	G:0.0277778	
chr28	9452270	2	32	G:1	A:0	
chr28	9452314	2	30	TG:0.933333	T:0.0666667	
chr28	9452427	2	40	G:0.925	A:0.075	
chr28	9452712	2	38	A:0.947368	G:0.0526316	
chr28	9452734	2	40	T:0.95	G:0.05	
chr28	9453163	3	40	C:0.1	CA:0.75	CAA:0.15
chr28	9453932	2	40	TTATCTCTAAAA:0.9	T:0.1	
chr28	9454106	2	38	A:0.947368	T:0.0526316	
chr28	9454107	2	38	A:0.947368	T:0.0526316	
chr28	9454196	2	38	C:0.947368	T:0.0526316	
chr28	9454395	2	40	T:0.95	TA:0.05	
chr28	9454706	2	38	TC:0.921053	T:0.0789474	
chr28	9454805	2	40	C:0.95	T:0.05	
chr28	9455025	2	40	A:0.95	C:0.05	
chr28	9455167	2	38	T:0.947368	C:0.0526316	
chr28	9455343	2	40	C:0.95	T:0.05	
chr28	9455449	2	38	A:0.947368	G:0.0526316	
chr28	9455723	2	36	A:1	G:0	
chr28	9455757	2	36	T:0.916667	C:0.0833333	
chr28	9455869	2	38	T:1	C:0	
chr28	9455902	2	38	G:1	A:0	

chr28	9456042	2	34	C:0.941176	A:0.0588235	
chr28	9456143	2	38	C:0.973684	A:0.0263158	
chr28	9456168	2	36	C:0.944444	CTGTT:0.0555556	
chr28	9456189	2	36	C:0.944444	T:0.0555556	
chr28	9456651	2	40	C:0.925	A:0.075	
chr28	9456873	2	38	A:0.947368	T:0.0526316	
chr28	9456996	2	40	A:0.925	AGTCAGAATTG:0.075	
chr28	9457229	2	40	C:0.95	T:0.05	
chr28	9457587	2	38	A:0.947368	ATACT:0.0526316	
chr28	9457713	2	40	C:0.95	T:0.05	
chr28	9457811	2	40	T:0.975	TAGAAACTTACTGTTTTAAAAAAAATAAATAAATAAAATAAAATAAAAAAAAAA:0.025	
chr28	9457928	2	40	C:0.95	G:0.05	
chr28	9457936	2	40	A:0.95	G:0.05	
chr28	9458001	2	40	T:0.95	A:0.05	
chr28	9458292	2	38	A:0.973684	G:0.0263158	
chr28	9458778	2	34	G:0.970588	T:0.0294118	
chr28	9458795	2	36	T:0.972222	C:0.0277778	
chr28	9459245	2	38	G:0.947368	T:0.0526316	
chr28	9459498	2	40	CA:0.975	C:0.025	
chr28	9459828	2	40	A:0.95	G:0.05	
chr28	9459891	2	40	A:0.975	C:0.025	
chr28	9460192	2	38	T:0.973684	C:0.0263158	
chr28	9461215	2	40	A:0.95	G:0.05	
chr28	9461439	2	36	A:0.916667	G:0.0833333	
chr28	9462044	2	38	A:1	T:0	
chr28	9462052	3	38	A:0.421053	T:0.184211	AT:0.394737
chr28	9462054	3	38	T:0.447368	A:0.184211	TA:0.368421
chr28	9462408	2	40	G:0.975	A:0.025	
chr28	9462535	2	38	A:0.947368	G:0.0526316	
chr28	9462713	2	38	G:0.947368	T:0.0526316	
chr28	9462752	2	38	G:0.947368	T:0.0526316	
chr28	9462942	2	38	A:0.973684	T:0.0263158	
chr28	9463848	2	36	G:0.916667	C:0.0833333	
chr28	9463906	2	36	CCT:0.888889	C:0.111111	
chr28	9463999	2	40	T:0.925	A:0.075	
chr28	9464086	2	40	CAA:0.95	C:0.05	
chr28	9464290	2	34	C:0.823529	CAAT:0.176471	
chr28	9464350	2	40	G:0.975	A:0.025	
chr28	9464386	3	40	GATAT:0.825	G:0.05	GAT:0.125
chr28	9465510	2	38	C:0.973684	A:0.0263158	
chr28	9465524	3	40	G:0.3	GATTT:0.55	GATTTATTT:0.15
chr28	9465616	2	38	G:1	A:0	
chr28	9465675	2	38	G:1	A:0	
chr28	9466782	2	40	T:0.95	C:0.05	
chr28	9466972	2	38	G:0.947368	A:0.0526316	
chr28	9467047	2	36	G:0.944444	A:0.0555556	
chr28	9467215	2	38	AG:0.921053	A:0.0789474	
chr28	9467298	2	36	C:0.944444	T:0.0555556	
chr28	9467639	2	38	G:0.947368	T:0.0526316	

chr28	9467695	2	38	G:0.973684	A:0.0263158	
chr28	9467980	2	38	C:0.947368	T:0.0526316	
chr28	9468105	2	40	T:1	C:0	
chr28	9468372	2	38	C:0	A:1	
chr28	9468424	2	40	G:0	T:1	
chr28	9468495	2	34	G:0.882353	T:0.117647	
chr28	9468515	2	26	GC:0.923077	G:0.0769231	
chr28	9468894	2	40	A:0.95	G:0.05	
chr28	9468919	2	40	T:0.95	G:0.05	
chr28	9469082	2	36	A:1	C:0	
chr28	9469240	2	36	T:0.916667	C:0.0833333	
chr28	9469513	2	36	C:0.916667	T:0.0833333	
chr28	9469663	2	40	G:0.95	A:0.05	
chr28	9469964	2	38	A:0.947368	C:0.0526316	
chr28	9470952	2	38	A:0.947368	G:0.0526316	
chr28	9471337	2	38	T:1	C:0	
chr28	9471374	2	38	A:0.947368	T:0.0526316	
chr28	9471647	2	38	T:0.947368	C:0.0526316	
chr28	9471683	2	34	C:1	T:0	
chr28	9471820	3	36	TAAA:0.611111	T:0.0277778	TAA:0.361111
chr28	9471824	2	36	A:0.972222	T:0.0277778	
chr28	9471834	2	36	A:0.972222	T:0.0277778	
chr28	9471878	2	36	TA:0.944444	T:0.0555556	
chr28	9472139	2	40	G:0.975	GAATA:0.025	
chr28	9472160	2	40	A:0.975	C:0.025	
chr28	9472164	3	40	TA:0.825	T:0.15	TAA:0.025
chr28	9472507	2	36	A:0.944444	T:0.0555556	
chr28	9472509	2	36	A:0.944444	ATT:0.0555556	
chr28	9472687	2	40	TAA:0.975	T:0.025	
chr28	9472771	2	40	T:0.95	C:0.05	
chr28	9472811	2	40	T:0.925	C:0.075	
chr28	9472976	2	38	C:0.947368	T:0.0526316	
chr28	9473107	2	40	G:0.875	T:0.125	
chr28	9473232	2	36	T:0.527778	C:0.472222	
chr28	9473467	2	38	C:0.973684	G:0.0263158	
chr28	9473630	2	36	G:0.805556	A:0.194444	
chr28	9473921	2	38	C:0.947368	T:0.0526316	
chr28	9474078	2	40	A:0.975	T:0.025	
chr28	9474098	2	40	GT:0.95	G:0.05	
chr28	9474243	2	38	G:0.947368	A:0.0526316	
chr28	9474274	2	36	A:0.944444	G:0.0555556	
chr28	9474534	2	40	C:0.95	G:0.05	
chr28	9475305	2	36	C:0.944444	A:0.0555556	
chr28	9475872	2	40	T:1	TA:0	
chr28	9475873	2	40	A:0.95	T:0.05	
chr28	9477403	2	36	C:1	G:0	
chr28	9477706	2	40	C:0.875	A:0.125	
chr28	9480667	2	38	T:0.947368	C:0.0526316	
chr28	9481732	2	40	G:1	T:0	
chr28	9484016	2	40	T:0.675	C:0.325	
chr28	9484192	2	40	G:0.9	GTA:0.1	
chr28	9484730	2	40	T:0.9	C:0.1	
chr28	9485452	2	38	GTA:0.868421	G:0.131579	

chr28	9485454	2	38	A:0.947368	G:0.0526316
chr28	9485472	2	38	C:0.815789	T:0.184211
chr28	9486159	4	40	ATATTTATT:0.225	A:0.375	ATATT:
0.275	ATATTTATTTATT:0.125
chr28	9486719	3	38	TGA:0.894737	T:0.105263
TGAGA:0
chr28	9487304	2	40	A:1	C:0
chr28	9488801	2	40	AAG:1	A:0
chr28	9489052	2	22	AT:0.727273	A:0.272727
chr28	9489128	2	40	C:1	T:0
chr28	9489474	2	36	T:0	A:1
chr28	9490421	2	38	C:0.789474	T:0.210526
chr28	9492278	2	38	G:0.842105	T:0.157895
chr28	9492364	2	40	GC:1	G:0
chr28	9492496	2	28	TG:0.0714286	T:0.928571
chr28	9492789	2	36	AT:0.111111	A:0.888889
chr28	9492936	2	38	C:1	A:0
chr28	9493004	2	40	G:0.95	T:0.05
chr28	9493238	3	40	TTTTATTTA:0.85	T:0.05	TTTTA:
0.1
chr28	9493368	2	40	G:0.875	A:0.125
chr28	9493572	2	38	C:0.789474	T:0.210526
chr28	9493903	2	38	C:0.789474	T:0.210526
chr28	9494588	2	40	C:1	T:0
chr28	9494885	2	40	CTATGTTTCCTAGT:0.175	C:0.825
chr28	9494918	2	40	C:0.95	T:0.05
chr28	9495016	2	38	C:1	G:0
chr28	9495288	2	32	G:0.1875	T:0.8125
chr28	9495331	2	36	A:0.25	AT:0.75
chr28	9495351	2	38	TTTTAAATTA:0.947368	T:
0.0526316
chr28	9495479	2	32	T:0.75	A:0.25
chr28	9495505	2	34	C:0.235294	A:0.764706
chr28	9495512	2	34	C:0.235294	A:0.764706
chr28	9495626	2	38	C:0.789474	CACAG:0.210526
chr28	9495731	2	38	A:0	G:1
chr28	9495755	2	40	G:0.775	A:0.225
chr28	9495778	2	40	G:0.8	A:0.2
chr28	9496008	2	38	T:0.842105	TATG:0.157895
chr28	9496100	2	36	C:0.805556	T:0.194444
chr28	9496179	2	36	GATT:1	G:0
chr28	9496208	2	38	AG:0.131579	A:0.868421
chr28	9496209	2	38	GGAGA:0.868421	G:0.131579
chr28	9496213	2	38	A:0.131579	AG:0.868421
chr28	9496260	2	34	G:0.147059	T:0.852941
chr28	9496284	3	34	T:0.147059	C:0.852941
G:0
chr28	9496489	2	34	CT:0.794118	C:0.205882
chr28	9496593	2	36	A:0.861111	G:0.138889
chr28	9498183	2	34	A:0.794118	G:0.205882
chr28	9498273	2	40	A:0.825	T:0.175
chr28	9498387	2	34	A:0.911765	G:0.0882353
chr28	9498587	3	38	A:0.184211	AT:0.815789
ATT:0

chr28	9498665	2	38	A:0.789474	G:0.210526
chr28	9498680	2	38	G:0.789474	A:0.210526
chr28	9498726	2	40	T:0.25	G:0.75
chr28	9498764	2	40	G:0.875	A:0.125
chr28	9498915	3	38	GT:0.552632	G:0.236842
GTT:0.210526					
chr28	9499084	2	40	A:0.875	AGTTGTT:0.125
chr28	9499151	2	38	C:0.947368	T:0.0526316
chr28	9499210	2	38	G:0.763158	GAA:0.236842
chr28	9499407	2	36	GA:1	G:0
chr28	9499490	2	38	G:0.815789	T:0.184211
chr28	9499610	2	40	C:0.125	T:0.875
chr28	9499889	2	40	T:0.175	G:0.825
chr28	9499975	2	38	C:0.184211	A:0.815789
chr28	9500610	2	40	A:0.825	G:0.175
chr28	9500809	2	40	A:0.85	G:0.15
chr28	9501246	2	38	C:0.815789	T:0.184211
chr28	9501285	2	36	T:0.166667	C:0.833333
chr28	9501415	3	40	T:0.225	TTA:0.25 TTTA:0.525
chr28	9501431	2	40	AT:0.225	A:0.775
chr28	9501440	2	40	T:0.225	A:0.775
chr28	9501715	2	38	CT:0.236842	C:0.763158
chr28	9501902	2	40	T:0.225	C:0.775
chr28	9502402	2	38	G:0.263158	A:0.736842
chr28	9502449	2	40	C:1	T:0
chr28	9503233	2	40	T:0.2	C:0.8
chr28	9503303	2	36	A:1	G:0
chr28	9503721	2	38	C:1	T:0
chr28	9503885	2	36	T:0.194444	G:0.805556
chr28	9504296	2	38	T:0.868421	C:0.131579
chr28	9504525	2	40	A:0.2	C:0.8
chr28	9505005	2	36	T:0.805556	C:0.194444
chr28	9505226	2	34	A:0.764706	G:0.235294
chr28	9505395	2	40	A:0.825	AAAT:0.175
chr28	9505549	2	40	CTCCTTCCTTTCCTGTATCTTTATGCT:0.175	
C:0.825					
chr28	9505860	2	40	T:0.775	C:0.225
chr28	9506135	2	38	T:0.815789	A:0.184211
chr28	9506136	2	38	C:0.815789	A:0.184211
chr28	9506513	2	38	T:0.184211	A:0.815789
chr28	9506791	2	38	G:0.210526	A:0.789474
chr28	9507084	2	40	A:0.8	G:0.2
chr28	9507340	2	38	T:0.736842	TTCTCTCTCTC:
0.263158					
chr28	9508167	2	38	T:0.842105	C:0.157895
chr28	9508173	2	38	T:1	G:0
chr28	9508449	2	38	A:0.789474	G:0.210526
chr28	9508663	2	40	G:0.35	A:0.65
chr28	9508770	2	36	G:0.805556	A:0.194444
chr28	9509126	2	40	G:0.8	A:0.2
chr28	9509282	2	40	T:0.825	C:0.175
chr28	9509465	2	40	C:0.875	T:0.125
chr28	9509494	2	40	T:0.15	A:0.85
chr28	9510303	2	38	C:0.210526	CT:0.789474

chr28 9510340 2 38 GTTTT:0.263158 G:0.736842
chr28 9510675 3 38 TAA:0.710526 T:0.131579
TA:0.157895
chr28 9511120 2 34 AT:1 A:0
chr28 9511125 3 34 T:0 G:0.823529 TG:
0.176471
chr28 9511342 2 34 CTTT:0.852941 C:0.147059
chr28 9511791 2 40 T:1 C:0
chr28 9512450 3 40 CCTCTCT:0.9 C:0.1 CCTCT:0
chr28 9512527 2 40 A:0.825 C:0.175
chr28 9512624 2 40 A:0.825 C:0.175
chr28 9512645 2 40 TG:0.85 T:0.15
chr28 9513488 5 40 GGT:0.15 G:0.025 GGTGT:0.075
GGTGTGTGT:0.3 GGTGTGTGTGT:0.45
chr28 9513529 2 40 TTGATTTGCATTTCTCTGA:1 T:0
chr28 9513642 2 38 CTT:0.289474 C:0.710526
chr28 9513673 2 40 T:0.2 C:0.8
chr28 9513850 2 40 A:0.15 G:0.85
chr28 9513900 2 30 C:0.3 CTT:0.7
chr28 9514092 2 40 C:0.95 T:0.05
chr28 9514247 2 40 A:1 G:0
chr28 9514276 2 40 C:0.85 T:0.15
chr28 9514391 3 38 CCT:0.763158 C:0.236842
CCTCT:0
chr28 9515554 2 40 C:0.9 T:0.1
chr28 9516333 2 36 A:0.111111 ATAG:0.888889
chr28 9517458 2 38 G:0.894737 T:0.105263
chr28 9517894 2 34 GA:1 G:0
chr28 9517899 2 36 GAAAAGAAAAGAAAAGA:0.861111
G:0.138889
chr28 9517904 2 34 GAAAAGAAAAGA:0.735294 G:
0.264706
chr28 9517909 2 40 GAAAAGA:0.875 G:0.125
chr28 9517914 2 40 GA:0.9 G:0.1
chr28 9517928 3 40 AAAAAGAAAAG:0.325 A:0.425
AAAAAG:0.25
chr28 9518095 2 38 GAGAA:0.921053 G:0.0789474
chr28 9518139 2 40 AAAAGAAAGAAAG:0.85 A:0.15
chr28 9518438 2 36 G:0.722222 T:0.277778
chr28 9518562 2 38 C:0.5 CT:0.5
chr28 9518567 2 38 T:0.5 TA:0.5
chr28 9518776 2 38 G:0.921053 C:0.0789474
chr28 9518823 4 40 TAAAA:0.5 T:0 TAA:0.3
TAAA:0.2
chr28 9518931 2 38 AC:0.184211 A:0.815789
chr28 9518934 2 38 TGGA:0.184211 T:0.815789
chr28 9520117 2 38 C:0.868421 T:0.131579
chr28 9520496 2 40 C:0.425 G:0.575
chr28 9520512 2 40 G:0.425 A:0.575
chr28 9520561 2 38 A:0.131579 T:0.868421
chr28 9520834 2 34 CA:0.205882 C:0.794118
chr28 9520922 2 38 G:0.447368 T:0.552632
chr28 9520997 2 34 G:0.617647 A:0.382353
chr28 9521353 2 38 T:0.736842 A:0.263158

chr28 9521380 2 40 G:0.55 A:0.45
chr28 9521388 2 40 C:0.45 T:0.55
chr28 9522025 2 36 C:0.75 T:0.25
chr28 9522046 3 40 G:0.8 GAA:0.025 GAAA:
0.175
chr28 9522056 2 36 AG:0.416667 A:0.583333
chr28 9522057 2 38 G:0.789474 A:0.210526
chr28 9522239 2 40 A:0.8 T:0.2
chr28 9522566 2 40 G:0.775 T:0.225
chr28 9522631 2 38 C:0.789474 T:0.210526
chr28 9522639 2 38 A:0.552632 G:0.447368
chr28 9522821 2 40 C:0.15 CCTCTGT:0.85
chr28 9523058 2 40 T:0.825 G:0.175
chr28 9523213 2 38 C:0.368421 T:0.631579
chr28 9524088 2 34 G:0.411765 A:0.588235
chr28 9524160 2 36 T:0.833333 TC:0.166667
chr28 9524162 2 36 T:0.833333 C:0.166667
chr28 9524196 2 36 T:0.805556 A:0.194444
chr28 9525081 2 38 G:0.184211 A:0.815789
chr28 9525142 2 40 G:0.825 A:0.175
chr28 9525692 2 38 G:0.236842 C:0.763158
chr28 9525696 2 40 C:0.125 T:0.875
chr28 9526115 2 34 T:0.852941 TA:0.147059
chr28 9526276 2 38 G:0.947368 A:0.0526316
chr28 9526780 2 40 G:0.775 C:0.225
chr28 9527150 2 40 G:0.175 T:0.825
chr28 9527488 2 30 A:0.266667 C:0.733333
chr28 9527492 2 30 C:0.266667 A:0.733333
chr28 9527499 2 38 A:0.105263 AC:0.894737
chr28 9527529 4 40 GAAGAAAGA:0.775 G:0.175 GAAGA:
0.05 GAAGAAAGAAAGA:0
chr28 9527537 2 40 A:1 AAAG:0
chr28 9527764 2 38 A:0.763158 G:0.236842
chr28 9528033 2 34 G:0.705882 T:0.294118
chr28 9528160 2 38 G:0.368421 C:0.631579
chr28 9528182 3 38 T:0.868421 TA:0.131579
TAA:0
chr28 9528207 2 40 A:0.4 AAC:0.6
chr28 9528208 2 40 A:0.825 AC:0.175
chr28 9528209 2 40 A:0.825 AAAC:0.175
chr28 9528216 2 40 A:1 AC:0
chr28 9528253 2 38 T:0.868421 C:0.131579
chr28 9528902 2 38 A:0.184211 G:0.815789
chr28 9529067 2 36 C:0.166667 T:0.833333
chr28 9529360 2 40 C:0.625
CACAGAGAAAGAGAGAGAGAGAGAGAGAGAGGCAGAGAGACA:0.375
chr28 9529491 2 20 G:0.2 GT:0.8
chr28 9529511 2 20 CTTATATTTATTTT:0.6 C:0.4
chr28 9529577 2 10 A:0.8 AAG:0.2
chr28 9529688 2 40 G:0.825 T:0.175
chr28 9529766 2 40 A:0.4 ACC:0.6
chr28 9529773 2 40 T:0.175 C:0.825
chr28 9530088 2 38 A:0.368421 G:0.631579
chr28 9531740 2 38 T:0.842105 C:0.157895

chr28	9532276	2	40	A:0.325	G:0.675	
chr28	9533095	2	40	A:0.175	G:0.825	
chr28	9533300	2	40	T:0.875	G:0.125	
chr28	9533491	2	38	A:0.368421	T:0.631579	
chr28	9533685	2	40	G:0.325	T:0.675	
chr28	9533715	2	40	T:0.95	C:0.05	
chr28	9533792	2	38	G:0.157895	GT:0.842105	
chr28	9533874	2	40	CT:0.85	C:0.15	
chr28	9534245	2	40	T:0.325	TA:0.675	
chr28	9534425	2	40	T:0.9	C:0.1	
chr28	9534450	3	40	CTTTTATTTTA:0.825	C:0.075	
CTTTTA:0.1						
chr28	9534493	2	38	TTTTA:0.315789	T:0.684211	
chr28	9534662	2	38	C:0.263158	T:0.736842	
chr28	9534670	2	36	TTC:0.972222	T:0.0277778	
chr28	9534691	2	36	T:0.25	TTTTA:0.75	
chr28	9534741	2	38	G:0.868421	C:0.131579	
chr28	9534747	2	38	G:0.315789	A:0.684211	
chr28	9535206	2	36	C:0.333333	T:0.666667	
chr28	9535650	2	40	A:0.25	G:0.75	
chr28	9535696	2	40	C:0.15	T:0.85	
chr28	9535967	2	40	T:0.85	C:0.15	
chr28	9536027	2	40	TC:0.975	T:0.025	
chr28	9536028	3	40	C:0.25	CTT:0.625	CTTT:
0.125						
chr28	9536056	2	36	T:0.861111	G:0.138889	
chr28	9536195	2	34	C:0.852941	T:0.147059	
chr28	9536362	2	38	T:0.184211	C:0.815789	
chr28	9536624	3	34	TA:0.794118	T:0.0882353	
TAA:0.117647						
chr28	9537154	2	36	A:0.333333	AT:0.666667	
chr28	9537172	2	38	AAC:0.947368	A:0.0526316	
chr28	9537354	2	36	T:1	A:0	
chr28	9537511	2	38	TA:0.315789	T:0.684211	
chr28	9537555	2	40	A:0.275	T:0.725	
chr28	9537556	2	40	T:0.275	G:0.725	
chr28	9537565	2	40	A:0.35	C:0.65	
chr28	9537697	2	40	G:0.925	C:0.075	
chr28	9537699	2	40	G:0.9	A:0.1	
chr28	9537780	2	40	C:0.925	T:0.075	
chr28	9538134	2	40	T:0.35	TAAAAAAAC:0.65	
chr28	9538161	2	40	TCA:0.35	T:0.65	
chr28	9538230	2	36	C:0.25	G:0.75	
chr28	9538320	2	34	A:0.264706	AG:0.735294	
chr28	9538380	2	30	G:0.233333	C:0.766667	
chr28	9538530	2	30	A:0.2	G:0.8	
chr28	9538894	2	36	CG:0.166667	C:0.833333	
chr28	9539003	2	36	C:0.333333	G:0.666667	
chr28	9539029	2	36	G:0.305556	T:0.694444	
chr28	9539131	2	36	G:0.194444	C:0.805556	
chr28	9539496	2	38	A:0.421053	G:0.578947	
chr28	9539650	2	40	C:0.95	CTCTA:0.05	
chr28	9539693	2	38	A:0.394737	T:0.605263	
chr28	9539856	2	36	A:0.333333	G:0.666667	

chr28	9539946	2	38	C:0.421053	T:0.578947	
chr28	9540151	2	40	G:0.225	A:0.775	
chr28	9540383	2	36	T:0.305556	TA:0.694444	
chr28	9540981	2	40	C:1	T:0	
chr28	9542285	2	36	G:0.361111	C:0.638889	
chr28	9542524	2	40	C:1	G:0	
chr28	9542826	2	34	G:0.382353	C:0.617647	
chr28	9543002	2	40	G:0.175	T:0.825	
chr28	9543081	2	34	G:0.205882	C:0.794118	
chr28	9543089	2	34	T:0.970588	TGTG:0.0294118	
chr28	9543110	2	34	GGT:0.970588	G:0.0294118	
chr28	9543112	2	38	T:0.526316	TGGTG:0.473684	
chr28	9543441	2	36	CCCTCTA:0.861111	C:0.138889	
chr28	9543447	2	36	A:0.333333	C:0.666667	
chr28	9543483	2	38	TCTCA:0.394737	T:0.605263	
chr28	9543487	2	38	A:0.789474	T:0.210526	
chr28	9543735	2	38	C:0.236842	T:0.763158	
chr28	9543860	3	40	AACACACACAC:0.825	A:0.15	AACACACACACAC:0.025
chr28	9544056	2	38	A:0.184211	AT:0.815789	
chr28	9544375	2	38	G:0.263158	C:0.736842	
chr28	9544725	2	38	C:0.368421	CT:0.631579	
chr28	9544848	2	40	G:0.975	GTCA:0.025	
chr28	9545657	2	36	G:0.333333	GC:0.666667	
chr28	9545740	2	40	G:0.325	A:0.675	
chr28	9546080	2	40	C:0.125	CTAAA:0.875	
chr28	9546249	2	38	T:0.315789	A:0.684211	
chr28	9546401	2	38	G:0.131579	T:0.868421	
chr28	9547729	2	38	G:1	A:0	
chr28	9547754	2	38	G:0.236842	GAAACTT:0.763158	
chr28	9547967	2	38	T:0.394737	C:0.605263	
chr28	9548836	2	40	G:1	T:0	
chr28	9548879	2	36	C:1	CCT:0	
chr28	9548927	2	38	T:0.210526	TA:0.789474	
chr28	9549186	2	40	TAA:0.325	T:0.675	
chr28	9549260	2	40	C:1	T:0	
chr28	9549466	2	40	T:0.2	G:0.8	
chr28	9549533	2	38	A:0.447368	G:0.552632	
chr28	9549617	2	40	T:0.275	G:0.725	
chr28	9550879	3	40	GAA:0.15	G:0.75	GA:0.1
chr28	9550955	2	40	T:0.1	G:0.9	
chr28	9551321	2	36	AT:0.833333	A:0.166667	
chr28	9551324	2	36	T:0.166667	A:0.833333	
chr28	9551336	2	36	C:0.833333	T:0.166667	
chr28	9551751	2	38	A:0.815789	G:0.184211	
chr28	9552058	2	38	G:1	A:0	
chr28	9552210	2	38	A:1	G:0	
chr28	9552368	2	38	A:0.894737	G:0.105263	
chr28	9552896	2	38	GA:0.921053	G:0.0789474	
chr28	9552963	2	34	T:0.205882	C:0.794118	
chr28	9553043	2	38	C:0.368421	CCTCT:0.631579	
chr28	9553084	2	38	T:0.894737	TA:0.105263	
chr28	9553085	2	40	A:1	T:0	
chr28	9553087	2	40	A:1	AT:0	

chr28	9553185	2	38	ATTCTT:0.894737	A:0.105263
chr28	9553297	2	40	CAT:0.95	C:0.05
chr28	9553608	2	40	G:0.225	A:0.775
chr28	9553743	2	38	TA:0.842105	T:0.157895
chr28	9554716	2	36	T:0.694444	G:0.305556
chr28	9555719	2	38	A:0.842105	C:0.157895
chr28	9556220	3	40	TA:0.875	T:0.025	TAA:0.1
chr28	9556926	2	38	C:0.921053	G:0.0789474
chr28	9557108	2	40	A:1	T:0
chr28	9557190	2	38	T:0.236842	C:0.763158
chr28	9557298	2	36	A:0.805556	T:0.194444
chr28	9557305	2	36	T:0.805556	A:0.194444
chr28	9557451	2	40	C:1	T:0
chr28	9558305	2	38	AAGAT:0.842105	A:0.157895
chr28	9558311	3	38	G:0.263158	T:0.684211
GATTTATTTATTTATTT:0.0526316
chr28	9558838	2	36	T:0.194444	C:0.805556
chr28	9559014	2	40	G:0.175	GA:0.825
chr28	9559483	2	36	G:0.25	A:0.75
chr28	9559536	6	40
ATTTCTTTTCTTTTCTTTTCTTTTCTTTTCTTTTCT:0.25	A:0.625	ATTTCT:0
ATTTCTTTTCTTTTCT:0.025	ATTTCTTTTCTTTTCTTTTCT:0.1
ATTTCTTTTCTTTTCTTTTCTTTTCT:0
chr28	9559552	2	34	TTTCTTTTCTTTTCTTTTCTTTTCTTTTC:1
T:0
chr28	9560695	2	38	A:0.184211	G:0.815789
chr28	9560734	2	38	C:0.157895	T:0.842105
chr28	9561005	2	30	G:0.233333	GAA:0.766667
chr28	9561271	2	36	T:0.277778	C:0.722222
chr28	9561290	2	36	G:0.25	A:0.75
chr28	9561550	2	36	G:0.361111	A:0.638889
chr28	9561952	2	38	C:0.184211	CCT:0.815789
chr28	9561991	2	38	T:0.894737	A:0.105263
chr28	9561992	2	38	T:0.894737	A:0.105263
chr28	9562048	2	40	T:0.9
TAAGCGTCTGACTCTTAAAAATAAAAAAAAAAATAAATAAATAAAAAAAAAA:0.1
chr28	9562139	3	36	TAAAAA:0.166667	T:0.166667
TAAAA:0.666667
chr28	9562141	2	34	A:0.411765	T:0.588235
chr28	9562226	2	36	C:0.916667	T:0.0833333
chr28	9562307	2	36	A:0.277778	G:0.722222
chr28	9562854	2	12	GTC:0.916667	G:0.0833333
chr28	9562963	2	30	C:0.3	T:0.7
chr28	9563089	2	38	ACTTC:1	A:0
chr28	9563094	2	38	T:1	G:0
chr28	9563687	2	38	T:0.157895	C:0.842105
chr28	9563689	2	38	T:1	C:0
chr28	9563728	2	40	G:1	A:0
chr28	9563806	2	40	C:0.2	T:0.8
chr28	9563872	2	40	A:0.175	G:0.825
chr28	9563966	2	40	T:0.225	C:0.775
chr28	9564087	2	40	T:0.15	A:0.85
chr28	9564157	4	40	CATATATATATATATATAT:0.025	C:0.125
CAT:0.75	CATATATATATATATAT:0.1

chr28	9564232	2	36	A:0.138889	G:0.861111	
chr28	9564455	2	36	C:0.166667	A:0.833333	
chr28	9564517	2	36	C:0.222222	T:0.777778	
chr28	9564662	2	36	ATACC:0.194444	A:0.805556	
chr28	9564839	2	38	A:0.394737	ATCTC:0.605263	
chr28	9564860	2	38	T:0.973684	TC:0.0263158	
chr28	9564863	2	38	T:0.973684	TTC:0.0263158	
chr28	9564880	2	38	C:1	T:0	
chr28	9564988	2	34	C:0.205882	T:0.794118	
chr28	9565137	2	38	G:0.184211	A:0.815789	
chr28	9565255	2	40	T:1	G:0	
chr28	9565492	2	38	T:0.131579	A:0.868421	
chr28	9565976	2	38	CT:1	C:0	
chr28	9566010	2	38	C:0.210526	T:0.789474	
chr28	9566172	2	40	CTTCCTAT:0.175	C:0.825	
chr28	9566326	2	40	T:1	C:0	
chr28	9566408	2	40	T:0.225	C:0.775	
chr28	9566466	2	40	T:1	C:0	
chr28	9566635	2	36	CT:0.194444	C:0.805556	
chr28	9566638	2	36	T:0.222222	C:0.777778	
chr28	9566641	3	38	T:1	TTTTC:0	TTTTTC:0
chr28	9566845	2	36	A:0.194444	T:0.805556	
chr28	9566980	3	36	CAA:0.222222	C:0.777778	CA:0
chr28	9567018	2	36	C:0.166667	T:0.833333	
chr28	9567089	2	36	A:0.166667	G:0.833333	
chr28	9567242	2	36	C:0.166667	T:0.833333	
chr28	9567334	2	38	T:0.184211	G:0.815789	
chr28	9567374	2	36	T:0.222222	TAAAAAA:0.777778	
chr28	9567491	2	40	A:0.175	T:0.825	
chr28	9567567	2	38	A:0.157895	G:0.842105	
chr28	9567838	2	40	T:1	C:0	
chr28	9568410	2	40	C:1	T:0	
chr28	9568453	2	38	A:0.184211	C:0.815789	
chr28	9568758	2	36	T:1	G:0	
chr28	9568823	2	38	T:0.210526	C:0.789474	
chr28	9568867	2	40	T:1	A:0	
chr28	9568868	2	40	A:1	T:0	
chr28	9568876	2	40	A:1	T:0	
chr28	9568938	2	40	TAC:1	T:0	
chr28	9569039	2	38	C:1	CAT:0	
chr28	9569139	2	40	A:0.95	G:0.05	
chr28	9569192	2	40	A:1	AG:0	
chr28	9569449	2	38	T:1	C:0	
chr28	9569456	2	38	T:1	C:0	
chr28	9569549	2	38	A:1	T:0	
chr28	9569567	2	38	A:0.184211	G:0.815789	
chr28	9569730	2	38	A:1	G:0	
chr28	9569792	2	40	C:1	T:0	
chr28	9569865	2	40	T:0.95	TA:0.05	
chr28	9569873	3	40	A:0.15	AAAG:0.025	AAAAAAG:0.825
chr28	9569932	2	36	T:1	G:0	
chr28	9569936	2	36	T:1	C:0	

chr28	9570043	2	40	T:1	A:0		
chr28	9570739	2	38	T:0.236842	TA:0.763158		
chr28	9570895	2	40	C:1	T:0		
chr28	9571027	2	40	T:1	A:0		
chr28	9571090	2	40	A:1	T:0		
chr28	9571210	2	36	C:1	T:0		
chr28	9571231	2	36	TA:0.166667	T:0.833333		
chr28	9571414	2	38	T:0.131579	G:0.868421		
chr28	9571507	2	38	A:0.157895	G:0.842105		
chr28	9571879	2	40	A:0.175	T:0.825		
chr28	9572284	2	32	TCAG:0.40625	T:0.59375		
chr28	9572372	4	28	AG:0.214286	A:0.0357143	AGG:0.321429	AGGG:0.428571
chr28	9574033	2	36	G:1	T:0		
chr28	9574255	2	38	G:0.973684	T:0.0263158		
chr28	9574382	2	38	G:1	C:0		
chr28	9574497	2	38	G:0.868421	GT:0.131579		
chr28	9574508	2	40	T:1	TTTA:0		
chr28	9574595	2	40	C:1	T:0		
chr28	9574685	2	40	C:1	T:0		
chr28	9574911	2	38	G:1	T:0		
chr28	9575111	2	36	T:0.916667	TG:0.0833333		
chr28	9575539	2	40	T:1	TC:0		
chr28	9575563	2	40	G:1	T:0		
chr28	9575754	2	38	A:1	G:0		
chr28	9575783	2	38	A:1	T:0		
chr28	9576019	2	40	A:1	T:0		
chr28	9576029	2	40	A:1	T:0		
chr28	9576357	2	38	T:1	G:0		
chr28	9576358	2	38	T:1	C:0		
chr28	9576589	2	40	G:1	A:0		
chr28	9576677	2	38	C:0.131579	G:0.868421		
chr28	9577179	2	40	ATAAAG:1	A:0		
chr28	9577329	2	32	TG:1	T:0		
chr28	9577593	2	40	A:0.8	G:0.2		
chr28	9577819	2	40	A:1	G:0		
chr28	9577997	2	36	C:1	T:0		
chr28	9578021	2	40	C:0.925	CGGG:0.075		
chr28	9578022	2	40	A:0.925	ATCCCTGGGTGGCGC:0.075		
chr28	9578024	2	40	G:0.925	GCGGTTTGGCGCCTGCCT:0.075		
chr28	9578050	2	40	G:0.875	A:0.125		
chr28	9578247	2	38	A:1	G:0		
chr28	9578336	2	40	A:1	ATTATCTTTATTATTTTCTTTTTTTTTTTTT:0		
chr28	9578932	2	38	T:1	G:0		
chr28	9579011	2	40	A:1	ATATAGAATATATGTCAT:0		
chr28	9579015	2	40	A:1	ATAT:0		
chr28	9579016	2	40	C:1	G:0		
chr28	9579207	2	40	G:1	A:0		
chr28	9579410	2	40	C:1	T:0		
chr28	9579440	2	40	T:1	C:0		
chr28	9579871	2	40	G:1	T:0		
chr28	9580107	2	38	A:0.184211	G:0.815789		
chr28	9580128	2	38	C:0.184211	T:0.815789		

chr28	9580216	2	38	G:0.105263	A:0.894737	
chr28	9580396	2	38	T:1	G:0	
chr28	9580457	2	38	G:1	A:0	
chr28	9580922	2	38	A:1	AT:0	
chr28	9580931	2	38	C:1	CCA:0	
chr28	9581200	2	36	TA:0.916667	T:0.0833333	
chr28	9581214	2	36	AT:0.916667	A:0.0833333	
chr28	9581215	2	36	T:0.222222	A:0.777778	
chr28	9581374	2	36	G:1	A:0	
chr28	9581495	2	36	G:1	A:0	
chr28	9581533	2	38	G:1	A:0	
chr28	9581568	2	40	G:1	A:0	
chr28	9581640	2	40	T:1	C:0	
chr28	9581651	2	40	C:0.875	T:0.125	
chr28	9581821	2	36	G:1	A:0	
chr28	9581918	2	36	A:1	G:0	
chr28	9582135	2	40	T:1	C:0	
chr28	9582608	2	38	A:1	G:0	
chr28	9582966	2	40	A:1	AT:0	
chr28	9582975	2	40	G:1	A:0	
chr28	9582976	2	40	C:0.2	CT:0.8	
chr28	9582986	2	40	T:1	TA:0	
chr28	9583014	2	40	TGAGA:0.95	T:0.05	
chr28	9583915	2	40	A:1	AGT:0	
chr28	9584159	2	38	G:1	GC:0	
chr28	9584161	2	38	G:1	A:0	
chr28	9584202	2	40	A:0.875	G:0.125	
chr28	9584260	2	36	G:1	GTC:0	
chr28	9584262	2	38	C:1	CTA:0	
chr28	9585083	2	38	G:1	A:0	
chr28	9585095	2	38	A:1	G:0	
chr28	9585230	2	38	C:0.868421	T:0.131579	
chr28	9585360	2	36	G:0.888889	GT:0.111111	
chr28	9586291	2	38	A:1	G:0	
chr28	9586670	2	40	G:1	A:0	
chr28	9587213	2	38	C:1	CT:0	
chr28	9587279	2	38	T:1	C:0	
chr28	9587607	2	40	C:1	G:0	
chr28	9587634	2	40	T:1	C:0	
chr28	9587706	2	38	G:1	GT:0	
chr28	9588002	2	36	C:1	T:0	
chr28	9588003	2	36	A:1	G:0	
chr28	9589333	2	36	G:1	A:0	
chr28	9590225	2	36	G:0.138889	A:0.861111	
chr28	9590592	2	40	G:1	T:0	
chr28	9590966	2	38	A:0.157895	G:0.842105	
chr28	9591226	3	40	GTA:0.675	G:0.175	GTATATA:0.15
chr28	9591411	2	36	A:0.138889	T:0.861111	
chr28	9591551	2	40	C:1	T:0	
chr28	9591697	2	40	A:1	C:0	
chr28	9591720	2	40	C:1	T:0	
chr28	9591912	2	38	A:1	AG:0	
chr28	9591913	3	38	AT:0.131579	A:0.868421	

TT:0						
chr28	9592007	2	38	G:1	A:0	
chr28	9592121	2	40	C:1	T:0	
chr28	9592682	2	40	G:0.9	GT:0.1	
chr28	9593230	2	40	T:0.1	C:0.9	
chr28	9593310	2	38	C:1	T:0	
chr28	9593919	2	40	C:1	G:0	
chr28	9593973	2	40	T:1	C:0	
chr28	9594038	2	40	G:1	A:0	
chr28	9595125	2	40	T:1	C:0	
chr28	9595450	2	40	G:0.65	T:0.35	
chr28	9595656	2	40	T:1	C:0	
chr28	9596638	2	38	T:0.842105		C:0.157895
chr28	9596694	2	36	G:1	A:0	
chr28	9597063	2	38	T:1	C:0	
chr28	9599398	2	40	A:1	AT:0	
chr28	9599515	2	38	C:0.868421		T:0.131579
chr28	9599719	2	40	C:1	T:0	
chr28	9599929	2	38	G:1	A:0	
chr28	9599962	3	36	CT:0.555556		C:0.277778
CTT:0.166667						
chr28	9600142	2	40	G:0.9	GAA:0.1	
chr28	9600972	2	38	G:0.0789474		A:0.921053
chr28	9601336	2	36	T:1	C:0	
chr28	9601346	2	32	G:0.625	GTTT:0.375	
chr28	9601688	2	38	A:0.973684		T:0.0263158
chr28	9601819	2	40	C:0.975	CT:0.025	
chr28	9602071	2	38	G:1	T:0	
chr28	9602515	2	40	C:0.875	CAGAG:0.125	
chr28	9602556	2	38	T:0.921053		C:0.0789474
chr28	9602709	2	36	G:1	T:0	
chr28	9602781	2	40	G:0.85	A:0.15	
chr28	9603071	2	38	G:1	A:0	
chr28	9603595	2	40	C:1	T:0	
chr28	9603705	2	40	CAAATGT:1		C:0
chr28	9603737	2	40	G:0.875	A:0.125	
chr28	9603866	2	40	A:0.95	G:0.05	
chr28	9604113	2	40	C:1	CAAT:0	
chr28	9604502	2	40	C:1	T:0	
chr28	9604526	2	40	C:0.125	G:0.875	
chr28	9604595	2	34	A:1	AT:0	
chr28	9604900	2	38	C:0.684211		T:0.315789
chr28	9604964	2	40	A:0.875	AATT:0.125	
chr28	9605189	2	40	T:1	C:0	
chr28	9605274	2	38	C:1	T:0	
chr28	9605900	2	40	A:1	G:0	
chr28	9606015	2	40	A:1	G:0	
chr28	9606378	2	40	A:1	C:0	
chr28	9607071	2	40	A:0.725	G:0.275	
chr28	9607195	2	40	G:1	A:0	
chr28	9607695	2	40	T:0.275	TG:0.725	
chr28	9608901	2	40	C:0.9	T:0.1	
chr28	9609333	2	38	C:0.131579		T:0.868421
chr28	9609384	2	38	A:1	G:0	

chr28	9609453	2	38	AG:1	A:0		
chr28	9609874	2	40	T:1	C:0		
chr28	9609894	2	40	G:1	A:0		
chr28	9609925	2	40	C:0.875	CAAAT:0.125		
chr28	9610424	2	40	C:1	G:0		
chr28	9610717	2	40	T:0.125	A:0.875		
chr28	9610718	3	40	T:0.125	A:0.875	TA:0	
chr28	9610948	2	38	A:0.868421	T:0.131579		
chr28	9611116	2	36	C:0.944444	CTT:0.0555556		
chr28	9612034	2	40	G:1	A:0		
chr28	9612691	2	40	G:1	A:0		
chr28	9612988	2	38	G:1	A:0		
chr28	9613566	2	40	C:0.25	CT:0.75		
chr28	9614922	2	40	ACTTT:0.8	A:0.2		
chr28	9615103	2	40	C:1	G:0		
chr28	9615195	3	40	G:0.175	GA:0.075	GAA:0.75	
chr28	9615206	2	40	C:1	A:0		
chr28	9615427	2	38	T:1	C:0		
chr28	9615564	2	40	T:0.125	G:0.875		
chr28	9616775	2	40	A:0.075	C:0.925		
chr28	9616846	2	40	A:1	C:0		
chr28	9616922	2	40	TA:0.1	T:0.9		
chr28	9616934	2	40	A:0.1	C:0.9		
chr28	9617161	2	38	T:1	C:0		
chr28	9617887	2	36	C:1	T:0		
chr28	9618073	2	38	A:1	T:0		
chr28	9619536	2	38	A:1	G:0		
chr28	9620179	2	40	AG:0.9	A:0.1		
chr28	9620202	2	40	G:1	A:0		
chr28	9620729	2	38	A:0.105263	T:0.894737		
chr28	9620950	2	40	T:0.85	C:0.15		
chr28	9621608	2	38	C:1	T:0		
chr28	9622738	2	38	ATT:0.631579	A:0.368421		
chr28	9623784	2	38	A:0.0789474	G:0.921053		
chr28	9623870	2	40	A:0.95	G:0.05		
chr28	9624327	2	40	C:0.125	T:0.875		
chr28	9624715	2	38	A:0.131579	G:0.868421		
chr28	9625347	2	38	T:0.868421	C:0.131579		
chr28	9625621	2	40	A:1	G:0		
chr28	9626145	2	40	TG:1	T:0		
chr28	9626211	2	40	T:1	C:0		
chr28	9627016	2	40	A:1	C:0		
chr28	9627355	2	38	C:0.894737	A:0.105263		
chr28	9628345	2	40	C:0.1	T:0.9		
chr28	9629034	2	38	A:0.868421	T:0.131579		
chr28	9630247	2	36	A:0.916667	AAG:0.0833333		
chr28	9630576	2	40	C:0.925	CAT:0.075		
chr28	9630904	2	40	A:0.175	G:0.825		
chr28	9630930	2	40	C:1	T:0		
chr28	9630936	2	40	A:0.125	T:0.875		
chr28	9631549	2	36	A:0.861111	T:0.138889		
chr28	9632064	2	40	CT:1	C:0		
chr28	9632137	4	40	G:0.775	GCATT:0.125	GCATTCATT:0	GCATTCATTCATT:0.1

chr28 9632654 2 36 G:0.916667 A:0.0833333
chr28 9632733 2 38 A:0.894737 ATCTG:0.105263
chr28 9632756 2 38 TGGCTACAG:0.947368 T:
0.0526316
chr28 9632783 2 40 G:0.725 A:0.275
chr28 9632961 2 40 T:1 TTTTG:0
chr28 9633726 2 40 C:0.9 T:0.1
chr28 9633821 2 40 GATTT:0.55 G:0.45
chr28 9634589 2 40 C:0.95 CT:0.05
chr28 9634607 2 40 T:0.125 G:0.875
chr28 9634619 2 40 TTTTG:0.15 T:0.85
chr28 9634690 2 38 G:1 A:0
chr28 9634767 2 36 G:0.444444 A:0.555556
chr28 9635248 2 38 G:0.868421 T:0.131579
chr28 9635437 2 38 G:1 A:0
chr28 9635479 2 40 G:0.825 T:0.175
chr28 9635722 2 38 C:0.157895 T:0.842105
chr28 9637485 2 32 G:0.875 A:0.125
chr28 9637689 2 38 G:0.868421 A:0.131579
chr28 9638048 2 40 A:0 AT:1
chr28 9638178 3 38 TAA:0.315789 T:0.0789474
TA:0.605263
chr28 9638628 2 40 C:0.85 T:0.15
chr28 9638765 2 38 G:0.0789474 A:0.921053
chr28 9638891 2 40 AT:0.1 A:0.9
chr28 9638897 2 40 T:0.1 A:0.9
chr28 9638903 2 38 TTTTTA:0.763158 T:0.236842
chr28 9638904 2 38 TTTTA:0.342105 T:0.657895
chr28 9639007 2 38 T:0.157895 C:0.842105
chr28 9639582 2 40 G:1 A:0
chr28 9640520 2 36 T:1 C:0
chr28 9640812 2 38 C:0.842105 T:0.157895
chr28 9640917 2 38 A:1 T:0
chr28 9641210 2 36 C:1 T:0
chr28 9641320 3 30 TAAAAAAA:0.233333 T:
0.333333 TA:0.433333
chr28 9641432 2 34 GAT:0.941176 G:0.0588235
chr28 9641446 4 40 T:0.325
TATATATATATGATGTCATATATATATGATGTCATC:0.225
TATATATATATATGATGTCATATATATATGATGTCATC:0.225
TATATATATATATATGATGTCATATATATATGATGTCATC:0.225
chr28 9641936 2 40 A:1 G:0
chr28 9642104 2 40 C:0.15 G:0.85
chr28 9642174 2 38 G:0.131579 T:0.868421
chr28 9643130 2 36 GA:1 G:0
chr28 9646093 2 40 A:0.9 G:0.1
chr28 9646782 2 36 C:0.0833333 CT:0.916667
chr28 9647574 2 40 T:0.1 TA:0.9
chr28 9647680 3 40 A:0.95 AGTGTGTGT:0
AGTGTGTGTGT:0.05
chr28 9647845 2 36 T:0.888889 C:0.111111
chr28 9648320 2 40 G:1 A:0
chr28 9648412 2 38 C:0.105263 T:0.894737
chr28 9648490 2 40 C:1 T:0

chr28	9648986	2	40	A:0.95	G:0.05	
chr28	9649214	2	38	A:0.105263	G:0.894737	
chr28	9649446	2	38	A:0.894737	C:0.105263	
chr28	9649505	2	38	A:0.868421	G:0.131579	
chr28	9650323	3	36	G:0.111111	GTT:0.388889	GTTT:0.5
chr28	9650634	2	40	C:0.7	A:0.3	
chr28	9650639	2	40	T:1	C:0	
chr28	9651038	2	38	T:0.473684	C:0.526316	
chr28	9651083	2	38	CTT:0.342105	C:0.657895	
chr28	9651369	2	38	A:1	G:0	
chr28	9651668	2	38	T:1	C:0	
chr28	9651680	2	40	G:0.475	A:0.525	
chr28	9652019	2	40	TGTCA:0.4	T:0.6	
chr28	9652056	2	40	G:1	C:0	
chr28	9652957	2	40	T:0.425	C:0.575	
chr28	9653028	2	38	T:0.473684	C:0.526316	
chr28	9653703	2	40	T:1	C:0	
chr28	9653750	2	34	A:1	G:0	
chr28	9653818	2	38	T:0.394737	TTAA:0.605263	
chr28	9654143	2	34	A:0.705882	G:0.294118	
chr28	9654174	2	32	C:0.6875	T:0.3125	
chr28	9654445	2	32	G:0.6875	GC:0.3125	
chr28	9654971	2	38	A:0.578947	G:0.421053	
chr28	9655006	2	38	C:0.447368	T:0.552632	
chr28	9655026	2	38	T:0.394737	C:0.605263	
chr28	9655278	2	40	T:1	TGAGTTACCCCTCACTG:0	
chr28	9655280	2	40	A:0.525	AGTTACCCCTCACTGGG:0.475	
chr28	9655689	2	40	C:1	T:0	
chr28	9655751	2	38	C:1	T:0	
chr28	9655846	2	38	G:0.973684	A:0.0263158	
chr28	9656111	2	36	A:1	G:0	
chr28	9656180	2	36	T:0.416667	G:0.583333	
chr28	9656183	2	36	C:0.416667	CA:0.583333	
chr28	9656400	2	38	G:0.473684	A:0.526316	
chr28	9656541	2	40	A:0.275	C:0.725	
chr28	9656548	2	40	G:0.325	A:0.675	
chr28	9656876	2	32	A:0.40625	T:0.59375	
chr28	9657139	2	30	T:0.633333	C:0.366667	
chr28	9657309	2	36	GT:1	G:0	
chr28	9657397	2	38	G:1	A:0	
chr28	9657624	2	34	T:0.176471	A:0.823529	
chr28	9657795	2	32	C:1	A:0	
chr28	9657904	2	36	G:1	A:0	
chr28	9657940	2	34	A:0.647059	AGCATGCAGT:0.352941	
chr28	9658171	2	40	TC:1	T:0	
chr28	9658397	2	40	T:0.55	C:0.45	
chr28	9658782	2	38	C:1	T:0	
chr28	9658807	2	40	A:0.55	T:0.45	
chr28	9659055	2	36	A:0.611111	AGC:0.388889	
chr28	9659105	2	40	T:0.6	TGCTTCCGCTGTGTCAG:0.4	
chr28	9659176	2	36	G:0.388889	GA:0.611111	
chr28	9659199	2	40	A:0.4	G:0.6	

chr28	9659205	2	40	G:0.4	A:0.6
chr28	9659489	2	40	CAA:1	C:0
chr28	9659589	2	38	C:0.526316	T:0.473684
chr28	9659647	2	34	T:0.352941	C:0.647059
chr28	9659741	2	38	G:1	A:0
chr28	9659752	2	38	T:1	A:0
chr28	9659891	2	36	G:1	GC:0
chr28	9660193	2	34	G:1	T:0
chr28	9660414	2	36	C:1	T:0
chr28	9660529	2	36	C:1	T:0
chr28	9660628	2	40	T:1	G:0
chr28	9660887	2	40	T:0.525	C:0.475
chr28	9661344	2	40	G:1	T:0
chr28	9661812	2	36	C:0.611111	G:0.388889
chr28	9661982	2	40	TAA:0.35	T:0.65
chr28	9661983	2	40	A:1	T:0
chr28	9662011	2	40	C:1	CAG:0
chr28	9662101	2	40	A:0.65	T:0.35
chr28	9662113	2	40	C:1	T:0
chr28	9662146	2	38	T:0.657895	C:0.342105
chr28	9662157	2	38	G:0.684211	A:0.315789
chr28	9662260	2	38	C:1	T:0
chr28	9662584	2	40	A:0.475	T:0.525
chr28	9662786	2	38	T:0.368421	C:0.631579
chr28	9662830	2	38	C:1	T:0
chr28	9663399	2	38	C:1	T:0
chr28	9663510	2	34	A:0.411765	G:0.588235
chr28	9663899	2	38	G:0.605263	A:0.394737
chr28	9664071	2	38	T:0.342105	C:0.657895
chr28	9664231	2	36	T:1	A:0
chr28	9664387	2	40	G:1	A:0
chr28	9664447	2	38	G:0.5	A:0.5
chr28	9664638	2	40	A:0.45	G:0.55
chr28	9665033	2	36	T:0.416667	C:0.583333
chr28	9665243	2	40	G:1	A:0
chr28	9665256	2	40	C:1	G:0
chr28	9665461	2	38	G:0.605263	A:0.394737
chr28	9665513	2	36	C:1	T:0
chr28	9666121	2	38	A:0.447368	T:0.552632
chr28	9666214	2	40	G:1	A:0
chr28	9666242	2	38	A:0.447368	G:0.552632
chr28	9666302	2	38	T:0.368421	G:0.631579
chr28	9666472	2	38	G:0.447368	C:0.552632
chr28	9666500	2	36	G:1	A:0
chr28	9666626	2	34	C:0.705882	CT:0.294118
chr28	9666723	2	38	C:1	T:0
chr28	9666783	2	38	C:1	G:0
chr28	9666948	2	38	G:0.605263	T:0.394737
chr28	9667079	2	40	T:1	C:0
chr28	9667086	2	40	C:1	CCG:0
chr28	9667231	2	36	C:0.666667	A:0.333333
chr28	9667505	2	38	C:0.657895	T:0.342105
chr28	9667701	2	36	C:1	T:0
chr28	9667996	2	40	A:0.55	G:0.45

chr28	9668104	2	38	C:0.684211	T:0.315789	
chr28	9668263	2	40	A:0.425	G:0.575	
chr28	9668410	2	38	C:0	CA:1	
chr28	9668421	2	38	TG:0	T:1	
chr28	9668423	2	38	T:0	A:1	
chr28	9668439	2	38	C:1	T:0	
chr28	9668995	2	38	G:0.526316	A:0.473684	
chr28	9669489	2	40	G:0.375	A:0.625	
chr28	9669533	2	40	T:1	C:0	
chr28	9669608	2	38	T:1	C:0	
chr28	9669803	2	36	T:0.638889	C:0.361111	
chr28	9669900	2	38	T:0.631579	TCC:0.368421	
chr28	9669906	3	38	A:0.578947	AAAAAC:0.0526316	AAAAC:0.368421
chr28	9670366	2	36	A:0	G:1	
chr28	9671318	2	40	G:0.4	A:0.6	
chr28	9671397	2	38	C:0.315789	T:0.684211	
chr28	9671732	2	40	A:0.45	G:0.55	
chr28	9671764	2	40	C:0.95	T:0.05	
chr28	9671862	2	40	T:0.425	C:0.575	
chr28	9671913	2	40	A:1	G:0	
chr28	9671943	2	38	A:0.5	ACTGT:0.5	
chr28	9672157	2	40	C:1	CT:0	
chr28	9672165	2	40	T:0.4	A:0.6	
chr28	9672255	2	38	T:0.5	G:0.5	
chr28	9672418	2	36	A:1	G:0	
chr28	9672819	2	40	C:1	CT:0	
chr28	9672838	2	40	C:0.625	T:0.375	
chr28	9672902	2	36	A:1	G:0	
chr28	9672992	2	28	T:0.642857	C:0.357143	
chr28	9673011	2	28	A:0.642857	C:0.357143	
chr28	9673012	2	26	T:0.730769	C:0.269231	
chr28	9673119	2	22	C:0.363636	T:0.636364	
chr28	9673653	2	32	CA:0.46875	C:0.53125	
chr28	9673654	2	32	A:0.8125	C:0.1875	
chr28	9673757	2	40	C:1	T:0	
chr28	9674395	2	30	C:0.9	T:0.1	
chr28	9674746	2	38	G:1	T:0	
chr28	9674957	2	32	T:0.59375	TA:0.40625	
chr28	9675054	2	38	G:1	GA:0	
chr28	9675246	2	40	C:0.425	CT:0.575	
chr28	9675392	2	40	G:1	A:0	
chr28	9675737	2	40	C:0.625	T:0.375	
chr28	9676395	2	38	C:1	T:0	
chr28	9676547	2	38	C:0.368421	T:0.631579	
chr28	9676732	2	40	G:0.425	A:0.575	
chr28	9676832	2	40	T:0.425	C:0.575	
chr28	9677014	2	40	A:0.475	G:0.525	
chr28	9677144	2	38	A:0.368421	T:0.631579	
chr28	9677639	2	40	A:0.4	ATTG:0.6	
chr28	9677825	2	38	G:0.631579	A:0.368421	
chr28	9677987	2	40	G:1	C:0	
chr28	9678142	2	36	A:0.583333	C:0.416667	
chr28	9678350	2	38	C:0.578947	A:0.421053	

chr28	9678399	2	38	G:0.578947	A:0.421053
chr28	9678745	2	40	G:0.975	T:0.025
chr28	9679435	2	40	A:0.325	G:0.675
chr28	9679624	2	36	A:0.944444	C:0.0555556
chr28	9679710	2	40	A:1	G:0
chr28	9680298	2	38	C:0.394737	T:0.605263
chr28	9680322	2	38	T:0.394737	C:0.605263
chr28	9680453	2	38	C:0.631579	T:0.368421
chr28	9680576	2	38	C:1	T:0
chr28	9680940	2	38	A:0.5	G:0.5
chr28	9681149	2	38	G:0.421053	A:0.578947
chr28	9681230	2	40	T:0.55	C:0.45
chr28	9681350	2	36	C:0.416667	T:0.583333
chr28	9681525	2	40	C:0.925	T:0.075
chr28	9681616	2	36	G:0.638889	A:0.361111
chr28	9682796	2	36	A:1	T:0
chr28	9684073	2	40	A:1	G:0
chr28	9684650	2	38	G:0.578947	GTA:0.421053
chr28	9684944	2	38	T:0.421053	C:0.578947
chr28	9685059	2	40	C:0.425	CCTCTCT:0.575
chr28	9685108	4	40	TAA:0.225	T:0.35	TA:0.075
TAAA:0.35
chr28	9686194	2	40	G:0.325	A:0.675
chr28	9687510	2	38	G:1	A:0
chr28	9687764	2	40	A:0.4	C:0.6
chr28	9688323	2	40	C:0.45	G:0.55
chr28	9688775	2	38	A:1	G:0
chr28	9690095	2	40	A:1	AAAT:0
chr28	9690750	2	40	C:1	CAG:0
chr28	9691046	2	40	T:1	TAA:0
chr28	9691065	2	40	AAAT:0.925	A:0.075
chr28	9691066	2	40	AAT:0.9	A:0.1
chr28	9691068	3	40	TAA:0.95	T:0	TA:0.05
chr28	9691517	2	38	T:1	A:0
chr28	9692141	2	40	A:1	T:0
chr28	9692875	2	36	C:1	T:0
chr28	9693030	2	38	C:1	CG:0
chr28	9693291	2	38	G:0.5	A:0.5
chr28	9693366	2	34	A:0.676471	G:0.323529
chr28	9694301	3	40	ACT:0.85	A:0.15	ACTCT:0
chr28	9694803	2	36	A:1	C:0
chr28	9695387	2	26	TGGGGG:0.192308	T:0.807692
chr28	9696099	2	38	G:0.552632	T:0.447368
chr28	9696868	2	40	T:1	C:0
chr28	9696922	2	40	C:1	T:0
chr28	9697041	3	38	A:0.210526	ATTTCTTTC:
0.657895	ATTTCTTTCTTTC:0.131579
chr28	9697490	2	36	G:0.972222	A:0.0277778
chr28	9697717	2	38	A:1	T:0
chr28	9697967	2	34	A:0.441176	AT:0.558824
chr28	9698894	2	40	CTTTTTTTTTTTT:0.975	C:0.025
chr28	9699500	3	40	G:0.65	GTT:0.275	GTTT:
0.075
chr28	9701740	2	36	G:1	T:0

chr28 9703418 2 38 C:0.578947 T:0.421053
chr28 9703677 2 40 T:1 A:0
chr28 9704056 2 38 T:1 C:0
chr28 9704171 2 40 G:1 A:0
chr28 9704381 2 38 C:1 T:0
chr28 9704426 2 40 T:0.6 C:0.4
chr28 9704625 2 36 C:1 T:0
chr28 9704945 2 36 G:0.555556 A:0.444444
chr28 9706082 2 40 T:0.4 C:0.6
chr28 9706194 2 38 G:0.5 T:0.5
chr28 9706698 2 38 C:0.578947 T:0.421053
chr28 9707018 2 40 C:0.45 CAG:0.55
chr28 9707475 2 36 T:0.527778 C:0.472222
chr28 9708142 2 40 G:0.425 A:0.575
chr28 9708223 4 40 TAATAAAATAAAATAA:0.125 T:0.625
TAATAAAATAA:0 TAATAAAATAAAATAAAATAA:0.25
chr28 9710033 2 36 A:0.0833333 G:0.916667
chr28 9710282 3 40 CATAGAT:0.725 C:0.1
CATAGATATAGAT:0.175
chr28 9710299 2 40 A:0.95 ATC:0.05
chr28 9710301 2 40 A:0.95 AGATCTAGATC:0.05
chr28 9710322 2 40 G:0.825 GATATAGAT:0.175
chr28 9710326 2 40 G:0.95 T:0.05
chr28 9710328 3 40 T:0.75 G:0.075 TATAG:0.175
chr28 9711031 2 40 AGTGT:0.5 A:0.5
chr28 9711258 2 34 G:1 A:0
chr28 9712579 2 38 T:0.894737 TA:0.105263
chr28 9713224 2 38 C:1 G:0
chr28 9713970 2 38 A:0.447368 G:0.552632
chr28 9714649 2 40 CAATTTAATTACTTCAATTAAAAAA:0.05
C:0.95
chr28 9714674 2 40 A:0.05 AGG:0.95
chr28 9715061 2 40 T:0.9 TA:0.1
chr28 9715135 2 40 T:1 A:0
chr28 9715311 2 38 C:1 T:0
chr28 9715329 3 38 TA:0.868421 T:0.105263
TAA:0.0263158
chr28 9715348 2 40 T:0.65 C:0.35
chr28 9715604 2 38 G:0.657895 T:0.342105
chr28 9716009 2 36 G:0.416667 A:0.583333
chr28 9716417 2 38 CT:0.447368 C:0.552632
chr28 9717522 2 36 G:1 A:0
chr28 9717530 5 40 ATGTG:0.05 A:0.55 ATG:0
ATGTGTGTGTG:0.15 ATGTGTGTGTGTG:0.25
chr28 9717732 2 38 C:1 T:0
chr28 9717766 2 38 ACT:0.684211 A:0.315789
chr28 9718146 2 38 T:0.447368 C:0.552632
chr28 9718514 2 40 A:0.525 G:0.475
chr28 9718616 2 40 TA:0.725 T:0.275
chr28 9718710 2 38 A:0.657895 C:0.342105
chr28 9719127 2 40 G:0.4 T:0.6
chr28 9719292 2 38 C:0.578947 T:0.421053
chr28 9719408 2 38 G:1 A:0
chr28 9719755 2 40 A:0.45 C:0.55

chr28	9720134	2	40	C:1	T:0		
chr28	9721639	2	38	T:0.526316	C:0.473684		
chr28	9721686	2	40	C:0.95	T:0.05		
chr28	9721729	2	38	C:0.5	T:0.5		
chr28	9722300	2	38	C:0.605263	T:0.394737		
chr28	9722335	2	36	C:0.444444	T:0.555556		
chr28	9722472	2	40	G:1	A:0		
chr28	9722667	2	38	A:0.421053	C:0.578947		
chr28	9722668	2	38	GT:0.5	G:0.5		
chr28	9722959	2	38	C:1	T:0		
chr28	9723206	2	32	A:1	G:0		
chr28	9723630	2	40	G:0.625	A:0.375		
chr28	9723919	2	40	C:0.475	G:0.525		
chr28	9723997	2	38	G:0.263158	A:0.736842		
chr28	9724006	2	38	A:1	G:0		
chr28	9724574	2	38	G:0.921053	C:0.0789474		
chr28	9724633	2	36	T:1	C:0		
chr28	9724744	2	40	C:1	T:0		
chr28	9724827	2	40	A:1	G:0		
chr28	9724894	2	40	T:1	C:0		
chr28	9724895	2	40	T:1	C:0		
chr28	9725235	2	40	A:1	AC:0		
chr28	9725236	2	40	A:1	T:0		
chr28	9725255	2	40	T:1	C:0		
chr28	9725275	2	40	T:1	C:0		
chr28	9725447	2	38	T:1	G:0		
chr28	9725570	2	36	G:0.222222	A:0.777778		
chr28	9725663	2	38	G:1	A:0		
chr28	9725823	2	38	T:1	C:0		
chr28	9725868	2	40	TG:0.45	T:0.55		
chr28	9725883	2	38	A:1	C:0		
chr28	9725982	2	40	T:1	C:0		
chr28	9726140	2	40	CT:0.95	C:0.05		
chr28	9726488	2	38	T:1	C:0		
chr28	9726505	2	38	TA:0.631579	T:0.368421		
chr28	9727078	2	38	T:0.552632	C:0.447368		
chr28	9727823	2	36	C:1	T:0		
chr28	9727831	2	38	AAAACGT:0.342105	A:0.657895		
chr28	9728288	2	40	T:0.425	C:0.575		
chr28	9728361	2	40	A:0.95	T:0.05		
chr28	9728491	2	38	T:0.5	G:0.5		
chr28	9729023	2	40	G:0.375	A:0.625		
chr28	9729591	4	40	TAATGAATG:0.525	T:0.225	TAATG:0.25	TAATGAATGAATG:0
chr28	9730419	2	38	T:0.5	A:0.5		
chr28	9730820	2	36	T:0.0555556	C:0.944444		
chr28	9730882	2	38	T:0.947368	C:0.0526316		
chr28	9731081	2	38	C:0.421053	T:0.578947		
chr28	9731210	2	40	A:1	AT:0		
chr28	9731212	2	40	A:1	T:0		
chr28	9731223	2	38	GAT:0.526316	G:0.473684		
chr28	9731276	2	40	C:0.45	T:0.55		
chr28	9731289	2	40	T:0.45	G:0.55		
chr28	9731315	2	40	CAAT:0.425	C:0.575		

chr28 9731461 2 38 C:0.368421 T:0.631579
chr28 9731643 2 40 A:0.55 G:0.45
chr28 9731646 2 40 G:1 A:0
chr28 9732250 2 40 C:0.75 G:0.25
chr28 9732289 2 40 TCC:0.5 T:0.5
chr28 9732713 2 36 A:0.416667 C:0.583333
chr28 9732824 2 40 C:0.975 CTATATATATAACTATA:0.025
chr28 9733050 2 40 C:0.95 T:0.05
chr28 9733070 2 40 A:0.475 G:0.525
chr28 9733084 2 40 C:0.95 T:0.05
chr28 9733147 2 38 T:0.947368 A:0.0526316
chr28 9733150 2 38 C:0.947368 T:0.0526316
chr28 9733564 2 36 G:0.472222 A:0.527778
chr28 9733818 2 38 CAGAA:0.447368 C:0.552632
chr28 9733823 2 38 A:0.447368 C:0.552632
chr28 9733861 2 34 A:1 G:0
chr28 9734028 2 38 T:0.473684 C:0.526316
chr28 9734239 2 40 T:0.125 C:0.875
chr28 9734628 3 38 G:0.184211 GTC:0.5 GTCTC:
0.315789
chr28 9734706 2 38 A:0.368421 T:0.631579
chr28 9734721 3 38 CAA:0.289474 C:0.631579
CAAA:0.0789474
chr28 9734868 2 38 G:0.710526 C:0.289474
chr28 9736579 2 38 A:0.684211 G:0.315789
chr28 9736608 2 34 A:1 T:0
chr28 9736672 2 34 T:0.676471 TAAG:0.323529
chr28 9736841 2 38 A:0.315789 G:0.684211
chr28 9737006 4 40 A:0.225 ATG:0.2
ATGTGTGTGTGTGTGTG:0.275 ATGTGTGTGTGTGTGTGTG:0.3
chr28 9737028 2 38 A:0.368421 ATT:0.631579
chr28 9737409 2 40 T:0.325 C:0.675
chr28 9737548 3 38 GT:0.184211 G:0.736842
GTT:0.0789474
chr28 9737645 2 32 A:0.375 C:0.625
chr28 9737667 2 38 C:0.657895 T:0.342105
chr28 9737805 2 40 A:0.3 G:0.7
chr28 9737930 2 40 T:0.775 C:0.225
chr28 9738289 2 38 CA:0.921053 C:0.0789474
chr28 9738986 2 36 T:0.972222 C:0.0277778
chr28 9739027 2 34 G:0.764706 A:0.235294
chr28 9739349 2 38 G:0.710526 A:0.289474
chr28 9739370 2 38 T:0.710526 C:0.289474
chr28 9739420 2 40 C:0.65 T:0.35
chr28 9739441 2 40 T:0.65 C:0.35
chr28 9739464 2 40 T:0.65 C:0.35
chr28 9739475 2 40 G:0.65 A:0.35
chr28 9739585 2 40 C:0.675 T:0.325
chr28 9739593 2 40 G:1 A:0
chr28 9739661 2 40 C:0.6 T:0.4
chr28 9739683 2 38 G:0.736842 T:0.263158
chr28 9739715 3 40 A:0.95 C:0.05 T:0
chr28 9739815 2 40 G:0.75 A:0.25
chr28 9739860 2 38 C:0.763158 T:0.236842

chr28	9739865	2	38	C:0.763158	G:0.236842		
chr28	9739866	2	38	A:0.947368	G:0.0526316		
chr28	9739947	2	40	G:0.675	C:0.325		
chr28	9740055	2	38	G:0.0526316	A:0.947368		
chr28	9740120	2	38	C:0.815789	T:0.184211		
chr28	9740234	2	38	G:0.394737	A:0.605263		
chr28	9740295	2	38	C:1	T:0		
chr28	9740534	2	38	G:1	A:0		
chr28	9740867	2	40	G:0.4	A:0.6		
chr28	9740886	2	40	C:0.65	T:0.35		
chr28	9740999	2	40	CT:1	C:0		
chr28	9741008	4	40	GT:0.05	G:0.65	GTT:0.3	GTTT:0
chr28	9741185	2	38	TCTC:0.736842	T:0.263158		
chr28	9741427	2	40	TTC:0.7	T:0.3		
chr28	9741441	2	40	T:0.7	C:0.3		
chr28	9741450	2	40	T:0.7	C:0.3		
chr28	9741626	2	40	A:0.7	C:0.3		
chr28	9741663	2	40	G:0.475	A:0.525		
chr28	9741924	2	40	G:0.725	A:0.275		
chr28	9741942	2	40	AATTG:1	A:0		
chr28	9742027	2	34	T:0.0588235	C:0.941176		
chr28	9742045	2	34	C:0.764706	T:0.235294		
chr28	9742095	2	38	A:0.736842	AT:0.263158		
chr28	9742175	2	40	G:0.05	A:0.95		
chr28	9742286	2	40	C:0.675	T:0.325		
chr28	9742324	2	40	C:0.725	T:0.275		
chr28	9742332	2	40	A:0.625	G:0.375		
chr28	9742394	2	40	G:0.675	A:0.325		
chr28	9742571	2	38	G:0.947368	A:0.0526316		
chr28	9742760	2	40	T:0.65	G:0.35		
chr28	9742857	2	36	G:0.75	C:0.25		
chr28	9743064	2	40	T:0.7	A:0.3		
chr28	9743116	2	36	T:0.722222	TA:0.277778		
chr28	9743160	2	38	C:0.710526	A:0.289474		
chr28	9743176	2	38	A:0.736842	C:0.263158		
chr28	9743185	2	38	G:0.631579	A:0.368421		
chr28	9743202	2	38	A:0.736842	G:0.263158		
chr28	9743328	2	40	T:0.7	C:0.3		
chr28	9743590	2	40	G:0.875	GGGAT:0.125		
chr28	9743591	4	40	GGATAGATA:0.825	G:0	GGATA: 0.125	GGATAGATAGATA:0.05
chr28	9743595	2	38	A:1	G:0		
chr28	9743634	3	40	T:0.75	TAGATAG:0.05	TGATAGATAGATAG:0.2	
chr28	9743837	2	40	G:1	C:0		
chr28	9743838	2	40	T:0.625	C:0.375		
chr28	9743960	2	36	A:0.277778	T:0.722222		
chr28	9744247	2	40	C:0.6	A:0.4		
chr28	9744622	2	36	A:0.666667	G:0.333333		
chr28	9744849	2	40	G:0.675	A:0.325		
chr28	9745014	2	40	G:0.675	T:0.325		
chr28	9745028	2	40	T:1	TA:0		
chr28	9745205	2	40	C:1	T:0		
chr28	9745251	2	38	T:0.736842	C:0.263158		

chr28	9745261	2	38	G:0.736842	A:0.263158				
chr28	9745314	2	38	G:1	A:0				
chr28	9746163	2	40	T:0.2	G:0.8				
chr28	9746181	2	40	C:0.175	T:0.825				
chr28	9746220	2	40	C:0.2	T:0.8				
chr28	9746611	2	40	TC:1	T:0				
chr28	9746612	2	40	C:0.175	T:0.825				
chr28	9746758	2	40	T:1	C:0				
chr28	9747095	2	36	T:1	TAC:0				
chr28	9747240	2	40	TC:1	T:0				
chr28	9747525	2	40	A:1	T:0				
chr28	9747633	2	34	C:1	T:0				
chr28	9747866	2	38	C:0.131579	T:0.868421				
chr28	9747984	2	38	A:0.0526316	G:0.947368				
chr28	9748265	2	40	G:1	A:0				
chr28	9748530	2	36	G:0.166667	A:0.833333				
chr28	9749203	2	38	A:1	G:0				
chr28	9749315	2	34	C:0.941176	CT:0.0588235				
chr28	9749494	2	38	A:0.210526	G:0.789474				
chr28	9749795	2	38	A:0.131579	G:0.868421				
chr28	9749831	2	36	GC:0.138889	G:0.861111				
chr28	9750270	2	38	G:1	C:0				
chr28	9750347	2	40	A:0.025	C:0.975				
chr28	9750948	2	38	A:0.0526316	G:0.947368				
chr28	9751061	2	34	C:1	G:0				
chr28	9751265	2	38	C:1	T:0				
chr28	9751372	2	36	C:1	T:0				
chr28	9751465	4	38	AT:0.894737	A:0	ATT:0.0526316	ATTTT:0.0526316		
chr28	9752140	3	36	C:0.361111	CTG:0.388889	CTGTG:0.25			
chr28	9752675	2	40	G:1	A:0				
chr28	9752676	2	40	A:1	G:0				
chr28	9753535	2	34	C:0.205882	A:0.794118				
chr28	9753816	2	36	G:0.166667	A:0.833333				
chr28	9754192	2	34	T:0.882353	C:0.117647				
chr28	9754329	2	38	T:0.131579	C:0.868421				
chr28	9754954	2	40	T:1	TTTTTTA:0				
chr28	9754961	2	40	A:1	ATTTATTTTTTTTTTAAATTT:0				
chr28	9755512	2	38	G:0.947368	A:0.0526316				
chr28	9755568	2	40	C:0.15	A:0.85				
chr28	9755764	2	38	G:0.184211	T:0.815789				
chr28	9755816	2	40	A:0.15	G:0.85				
chr28	9755909	2	40	CAAATAATAATAATAATAA:1	C:0				
chr28	9755910	6	40	AAATAATAATAATAAT:0.125	A:0	AAAT:0	AAATAAT:0.35	AAATAATAAT:0.1	AAATAATAATAAT:0.425
chr28	9756064	2	36	A:0.166667	G:0.833333				
chr28	9756106	2	36	C:0.222222	G:0.777778				
chr28	9756139	2	40	T:0.975	TA:0.025				
chr28	9756756	2	38	A:1	G:0				
chr28	9756881	2	36	A:0.166667	G:0.833333				
chr28	9757016	2	40	C:0.175	G:0.825				
chr28	9757207	2	40	T:1	A:0				
chr28	9757230	2	40	C:0.95	T:0.05				

chr28	9757270	2	40	TCCTTCTCTCC:1	T:0
chr28	9757282	2	40	T:1	G:0
chr28	9757346	2	40	TTCTCTCCATAAGTAATGATC:1	T:0
chr28	9757454	2	38	C:0.0526316	T:0.947368
chr28	9757710	2	40	A:1	G:0
chr28	9758156	2	36	C:0.0833333	CTTTTTTTT:0.916667
chr28	9758433	2	40	TA:0.125	T:0.875
chr28	9758746	2	38	T:0.184211	A:0.815789
chr28	9758811	2	36	C:0.972222	T:0.0277778
chr28	9759090	2	38	GA:1	G:0
chr28	9759127	2	40	G:0.95	GAA:0.05
chr28	9759435	2	38	G:0.947368	C:0.0526316
chr28	9759494	2	38	T:0.0526316	TAA:0.947368
chr28	9759580	2	38	C:0.947368	A:0.0526316
chr28	9759649	2	40	T:0.95	C:0.05
chr28	9759874	2	40	T:0.975	C:0.025
chr28	9759938	2	40	A:0.125	G:0.875
chr28	9759994	2	38	G:0.947368	A:0.0526316
chr28	9760373	2	40	T:0.95	C:0.05
chr28	9760542	2	40	G:0.95	A:0.05
chr28	9760660	2	38	C:1	T:0
chr28	9761086	2	40	GA:0.95	G:0.05
chr28	9761098	2	40	A:1	C:0
chr28	9761245	2	38	C:0.973684	CCT:0.0263158
chr28	9761552	2	38	T:0.947368	G:0.0526316
chr28	9761728	2	40	G:0.125	GT:0.875
chr28	9762479	2	38	TG:0.921053	T:0.0789474
chr28	9762767	2	40	AAG:1	A:0
chr28	9763333	2	38	A:1	T:0
chr28	9763478	2	40	C:0.025	T:0.975
chr28	9763486	2	40	T:0.025	C:0.975
chr28	9764176	2	36	C:0.0555556	G:0.944444
chr28	9764669	2	38	G:0.0526316	C:0.947368
chr28	9764759	2	40	G:1	GAAATACAGATGTCAAAAAAAAAAAAA:0
chr28	9765181	2	38	C:0.947368	T:0.0526316
chr28	9765523	2	36	T:0	G:1
chr28	9765634	2	36	C:0.833333	G:0.166667
chr28	9765903	2	38	C:1	T:0
chr28	9766373	2	34	T:0.823529	C:0.176471
chr28	9766593	2	40	GAGAAAGAAAAGAAGAAAGAAAGAAGA:0.15	G:0.85
chr28	9766676	2	36	AAG:0.166667	A:0.833333
chr28	9766683	2	36	G:0.166667	GA:0.833333
chr28	9766993	2	38	G:0.105263	A:0.894737
chr28	9767153	2	40	C:1	T:0
chr28	9767192	2	40	T:0.95	C:0.05
chr28	9767386	2	40	T:1	TTAGA:0
chr28	9767447	2	38	T:0.105263	C:0.894737
chr28	9767480	2	38	G:0.921053	C:0.0789474
chr28	9767673	2	36	G:0.861111	A:0.138889
chr28	9768353	2	38	A:0.131579	G:0.868421
chr28	9768697	2	40	C:0.125	T:0.875

chr28	9769049	2	40	A:0.175	AT:0.825
chr28	9769050	2	40	T:1	TA:0
chr28	9770759	2	38	G:1	C:0
chr28	9771167	2	40	C:0.5	T:0.5
chr28	9771342	2	40	C:0.425	T:0.575
chr28	9771538	2	40	T:0.475	TA:0.525
chr28	9771702	2	40	C:0.425	T:0.575
chr28	9771730	2	40	A:0.375	G:0.625
chr28	9772152	2	38	G:0.447368	T:0.552632
chr28	9772348	2	40	A:0.45	G:0.55
chr28	9772580	2	40	A:0.575	G:0.425
chr28	9772908	2	40	T:0.5	C:0.5
chr28	9772982	2	40	ATG:0.475	A:0.525
chr28	9772985	2	40	CCAGTGTTAA:0.475	C:0.525
chr28	9773464	2	34	C:0.411765	T:0.588235
chr28	9773787	2	38	A:0.5	G:0.5
chr28	9773853	2	36	A:0.611111	G:0.388889
chr28	9773867	2	38	C:0.5	T:0.5
chr28	9774139	2	38	G:0.947368	A:0.0526316
chr28	9774505	2	40	A:0.525	G:0.475
chr28	9774726	2	36	C:0.472222	T:0.527778
chr28	9774815	2	38	C:0.973684	T:0.0263158
chr28	9775141	2	38	G:0.552632	A:0.447368
chr28	9775184	2	38	T:1	TC:0
chr28	9775707	2	40	C:0.5	T:0.5
chr28	9775881	2	40	G:0.525	GAA:0.475
chr28	9776378	2	38	T:0.447368	C:0.552632
chr28	9776533	2	38	C:0.631579	T:0.368421
chr28	9776550	2	40	T:0.925	TC:0.075
chr28	9777051	2	40	G:0.95	A:0.05
chr28	9777185	2	36	G:0.527778	C:0.472222
chr28	9777308	2	36	T:0.444444	C:0.555556
chr28	9777424	2	40	T:0.525	C:0.475
chr28	9777623	2	38	T:0.526316	C:0.473684
chr28	9777957	2	40	C:1	A:0
chr28	9777990	2	40	T:0.475	A:0.525
chr28	9778333	2	38	C:0.947368	T:0.0526316
chr28	9778511	2	38	C:0.447368	T:0.552632
chr28	9778650	2	40	CT:0.5	C:0.5
chr28	9778713	2	40	T:0.475	G:0.525
chr28	9779028	2	38	A:0.552632	G:0.447368
chr28	9779184	2	38	A:0.0789474	G:0.921053
chr28	9779188	2	40	A:1	G:0
chr28	9779472	2	36	C:1	T:0
chr28	9779502	2	38	G:0.5	A:0.5
chr28	9779585	2	40	G:1	A:0
chr28	9779744	2	38	G:1	A:0
chr28	9779765	2	40	C:0.475	T:0.525
chr28	9779849	2	40	G:0.05	C:0.95
chr28	9779986	2	38	C:1	G:0
chr28	9780107	2	38	T:0.552632	C:0.447368
chr28	9780582	2	32	GAAAA:0	G:1
chr28	9780586	2	32	A:0	AGGCGC:1
chr28	9781109	2	28	CT:0.5	C:0.5

chr28	9781370	2	38	G:0.473684	A:0.526316
chr28	9781580	2	38	T:0.263158	C:0.736842
chr28	9781610	2	34	A:0.264706	G:0.735294
chr28	9781893	2	38	C:0.473684	T:0.526316
chr28	9782443	2	40	T:0.475	G:0.525
chr28	9782499	2	34	G:0.705882	A:0.294118
chr28	9782808	2	26	A:0.807692	T:0.192308
chr28	9782809	2	26	C:0.807692	A:0.192308
chr28	9782819	2	20	G:0.65	T:0.35
chr28	9782979	2	32	C:0.71875	T:0.28125
chr28	9783183	2	36	C:0	G:1
chr28	9783199	2	36	T:0.833333	C:0.166667
chr28	9783448	2	36	T:0.527778	C:0.472222
chr28	9783582	2	38	C:0.736842	A:0.263158
chr28	9783784	2	40	A:0.75	G:0.25
chr28	9783843	2	40	G:0.25	T:0.75
chr28	9783881	2	40	T:0.225	G:0.775
chr28	9784009	2	40	G:0.2	C:0.8
chr28	9784056	2	38	G:0.552632	A:0.447368
chr28	9784260	2	36	C:0.527778	T:0.472222
chr28	9784474	2	38	T:0.631579	C:0.368421
chr28	9784506	2	38	C:0.578947	T:0.421053
chr28	9784586	2	36	C:1	G:0
chr28	9784588	2	36	G:0.472222	A:0.527778
chr28	9784622	2	36	T:0.833333	C:0.166667
chr28	9784780	2	40	T:0.65	G:0.35
chr28	9785095	2	40	C:0.7	A:0.3
chr28	9785624	2	36	C:0.75	T:0.25
chr28	9785777	2	40	T:0.75	C:0.25
chr28	9785850	2	38	G:0.736842	A:0.263158
chr28	9785965	2	38	T:0.657895	G:0.342105
chr28	9786023	2	40	G:1	A:0
chr28	9786138	2	38	A:0.736842	G:0.263158
chr28	9786603	2	40	G:0.625	T:0.375
chr28	9786794	2	38	G:0.684211	A:0.315789
chr28	9786881	2	40	C:0.65	T:0.35
chr28	9786990	2	38	C:0.526316	T:0.473684
chr28	9787175	2	38	A:0.736842	G:0.263158
chr28	9787339	2	36	C:0.694444	T:0.305556
chr28	9787422	2	34	T:0.705882	C:0.294118
chr28	9787520	2	36	T:0.777778	G:0.222222
chr28	9787968	2	40	A:0.6	G:0.4
chr28	9788045	2	38	G:0.736842	A:0.263158
chr28	9788135	2	40	T:0.75	A:0.25
chr28	9788454	2	40	G:0.725	A:0.275
chr28	9788807	2	36	G:0.75	A:0.25
chr28	9788899	2	40	T:0.7	A:0.3
chr28	9789035	2	38	CT:0.684211	C:0.315789
chr28	9789068	2	36	C:0.722222	CAAAT:0.277778
chr28	9789180	2	40	C:0.75	T:0.25
chr28	9789612	2	40	T:0.725	C:0.275
chr28	9789645	2	40	G:0.05	A:0.95
chr28	9789859	2	36	C:0.777778	A:0.222222
chr28	9790016	2	40	T:0.775	TAGCC:0.225

chr28	9790026	2	36	TA:0.944444	T:0.0555556			
chr28	9790074	2	38	C:0.763158	T:0.236842			
chr28	9790405	2	36	C:0.972222	T:0.0277778			
chr28	9790419	2	36	G:0.777778	A:0.222222			
chr28	9790455	2	38	T:0	C:1			
chr28	9790526	2	36	A:0.694444	G:0.305556			
chr28	9790662	2	36	C:0.75	T:0.25			
chr28	9791240	2	40	A:0.725	AAG:0.275			
chr28	9791601	2	38	G:0.736842	A:0.263158			
chr28	9791611	2	38	C:0.736842	T:0.263158			
chr28	9792278	2	40	T:0.3	TA:0.7			
chr28	9792359	2	38	G:0.710526	T:0.289474			
chr28	9792736	2	40	C:0.875	T:0.125			
chr28	9792838	2	36	G:0.75	C:0.25			
chr28	9792888	2	40	AC:0.775	A:0.225			
chr28	9793291	2	38	G:0.947368	A:0.0526316			
chr28	9793351	2	40	C:1	T:0			
chr28	9793488	2	38	G:1	A:0			
chr28	9793596	2	40	C:0.725	T:0.275			
chr28	9793667	2	40	G:0.675	A:0.325			
chr28	9793740	2	40	G:0.775	T:0.225			
chr28	9793897	2	38	G:0.815789	A:0.184211			
chr28	9793969	2	40	A:0.7	G:0.3			
chr28	9793999	2	32	G:0.59375	GA:0.40625			
chr28	9794014	2	34	AT:0.735294	A:0.264706			
chr28	9794053	2	40	A:0.7	G:0.3			
chr28	9794505	2	38	A:1	G:0			
chr28	9794625	2	38	A:1	G:0			
chr28	9795001	2	38	C:1	T:0			
chr28	9795058	2	34	C:0.705882	G:0.294118			
chr28	9795444	2	40	A:0	G:1			
chr28	9795515	2	40	T:0	C:1			
chr28	9795678	2	40	A:0.75	G:0.25			
chr28	9795826	2	40	CT:1	C:0			
chr28	9795946	2	40	A:0	G:1			
chr28	9795986	2	40	T:1	C:0			
chr28	9796648	2	36	C:0	T:1			
chr28	9796896	2	38	T:0.657895	C:0.342105			
chr28	9797057	2	36	C:0.527778	A:0.472222			
chr28	9797235	2	40	T:1	C:0			
chr28	9797265	2	38	T:0.631579	C:0.368421			
chr28	9797266	2	40	G:0.65	A:0.35			
chr28	9797282	2	40	C:0.95	T:0.05			
chr28	9797798	2	40	G:1	A:0			
chr28	9798027	2	40	C:0.475	T:0.525			
chr28	9798108	2	38	G:1	A:0			
chr28	9798234	2	40	T:1	C:0			
chr28	9800437	2	38	A:0	G:1			
chr28	9800547	5	40	C:0.075	CA:0.175	CAA:0.1	CAAAAA:0.375	CAAAAAAA:0.275
chr28	9801138	2	38	C:1	T:0			
chr28	9801139	2	38	A:1	G:0			
chr28	9801402	2	36	G:1	T:0			
chr28	9801683	2	40	C:0.95	T:0.05			

chr28	9802136	2	40	C:1	G:0		
chr28	9802306	2	38	C:1	T:0		
chr28	9802363	4	38	C:0	CA:0	CAAAA:0.289474	CAAAAA:0.710526
chr28	9802788	2	40	G:0	A:1		
chr28	9802920	3	40	GAA:0.075	G:0.15	GA:0.775	
chr28	9803120	2	40	C:0.75	T:0.25		
chr28	9803834	2	38	G:0.736842	A:0.263158		
chr28	9803869	2	40	G:0.675	A:0.325		
chr28	9804295	2	38	G:0.684211	A:0.315789		
chr28	9804465	2	38	T:0.710526	C:0.289474		
chr28	9804556	2	40	T:0.675	TA:0.325		
chr28	9804561	2	40	T:0.675	C:0.325		
chr28	9804876	2	40	C:0.675	T:0.325		
chr28	9805551	2	40	T:0.6	C:0.4		
chr28	9805552	2	40	G:0.6	C:0.4		
chr28	9805581	2	40	A:0	ACT:1		
chr28	9806859	2	36	A:1	G:0		
chr28	9807002	4	40	AAC:0.15	A:0.2	AACAC:0.3	AACACACACACAC:0.35
chr28	9807618	2	36	A:0.444444	G:0.555556		
chr28	9808169	2	38	A:0.789474	C:0.210526		
chr28	9808283	2	38	A:0.289474	G:0.710526		
chr28	9808530	2	40	G:1	A:0		
chr28	9809053	2	38	G:0.473684	A:0.526316		
chr28	9809286	2	40	G:0.5	T:0.5		
chr28	9809566	2	34	C:0.5	T:0.5		
chr28	9810156	2	40	C:0.45	G:0.55		
chr28	9810157	2	40	A:0	G:1		
chr28	9810435	2	38	G:0.578947	A:0.421053		
chr28	9810671	2	40	GAGA:0.475	G:0.525		
chr28	9810895	2	38	A:0.605263	G:0.394737		
chr28	9811110	2	34	G:0.617647	A:0.382353		
chr28	9811232	2	36	G:0.611111	C:0.388889		
chr28	9811910	2	38	C:0.526316	T:0.473684		
chr28	9812192	2	40	C:0.5	T:0.5		
chr28	9812358	2	36	G:0.444444	A:0.555556		
chr28	9812908	2	36	T:0.472222	A:0.527778		
chr28	9812918	3	40	AGTGTGTGTGTGTGT:0.1	A:0.475	AGTGTGTGTGT:0.425	
chr28	9813361	2	40	T:0	G:1		
chr28	9813377	2	40	G:0.775	A:0.225		
chr28	9813701	2	38	C:0.526316	A:0.473684		
chr28	9814165	2	36	C:0.388889	T:0.611111		
chr28	9814419	3	40	GAA:0.55	G:0.125	GAAAA:0.325	
chr28	9814618	2	40	G:1	T:0		
chr28	9814674	2	36	A:0	G:1		
chr28	9814903	2	38	C:0.447368	T:0.552632		
chr28	9814907	2	38	T:1	A:0		
chr28	9815148	2	40	A:0	G:1		
chr28	9815254	2	40	T:0.775	TTAATAA:0.225		
chr28	9815653	2	38	G:0	A:1		
chr28	9815684	2	36	G:0.833333	A:0.166667		
chr28	9815850	2	36	G:0.666667	A:0.333333		

chr28	9816208	2	40	T:0	C:1		
chr28	9816217	2	40	C:0	A:1		
chr28	9817044	2	38	A:0.736842	G:0.263158		
chr28	9817417	2	38	G:0.289474	A:0.710526		
chr28	9817556	2	40	T:0.55	C:0.45		
chr28	9818005	3	38	A:0.342105	G:0.157895	T:0.5	
chr28	9818139	2	36	G:0.388889	A:0.611111		
chr28	9818302	2	36	C:0.75	T:0.25		
chr28	9818360	2	36	GGTGA:0.666667	G:0.333333		
chr28	9818551	2	38	T:0.289474	C:0.710526		
chr28	9818586	2	38	G:0.289474	A:0.710526		
chr28	9818719	2	40	C:0.3	T:0.7		
chr28	9818756	2	40	C:0.3	T:0.7		
chr28	9819007	2	40	T:0.375	C:0.625		
chr28	9820122	2	38	T:1	A:0		
chr28	9820376	2	40	G:0	C:1		
chr28	9821600	2	40	T:1	C:0		
chr28	9821617	2	40	A:0.65	AAAAC:0.35		
chr28	9821736	2	40	G:0.675	A:0.325		
chr28	9822122	2	38	GAA:1	G:0		
chr28	9822210	2	40	C:0	T:1		
chr28	9822230	2	38	A:0	C:1		
chr28	9822659	2	38	G:0	A:1		
chr28	9822684	2	36	C:0.305556	T:0.694444		
chr28	9822815	2	38	T:0	C:1		
chr28	9822998	2	40	C:1	CA:0		
chr28	9823379	2	38	G:0.684211	T:0.315789		
chr28	9823507	2	38	T:1	C:0		
chr28	9823706	2	34	G:0.794118	A:0.205882		
chr28	9824113	2	40	A:0.2	G:0.8		
chr28	9824436	2	40	T:0.675	G:0.325		
chr28	9824554	2	38	C:0.552632	T:0.447368		
chr28	9824573	2	40	C:0.8	A:0.2		
chr28	9824820	2	6	A:0.333333	T:0.666667		
chr28	9824902	2	40	G:0	A:1		
chr28	9825043	2	38	A:1	G:0		
chr28	9825151	2	40	CTTTTTTTTTT:0.5	C:0.5		
chr28	9825239	2	36	T:0.638889	C:0.361111		
chr28	9825274	2	36	T:0.25	C:0.75		
chr28	9825381	2	34	T:0.205882	C:0.794118		
chr28	9825714	2	40	C:0.725	T:0.275		
chr28	9825812	2	38	T:0.184211	C:0.815789		
chr28	9826285	2	38	G:1	A:0		
chr28	9826483	2	40	GAAGT:0.5	G:0.5		
chr28	9826672	2	38	C:0.210526	T:0.789474		
chr28	9827433	2	38	TTTTTC:0.710526	T:0.289474		
chr28	9827734	2	34	A:0.205882	C:0.794118		
chr28	9827865	2	38	C:0.263158	T:0.736842		
chr28	9828526	2	38	C:0.210526	A:0.789474		
chr28	9828545	2	40	TACACACACAA:1	T:0		
chr28	9828555	4	40	AACACACACACACAC:0.2	A:0.325	AACAC:0.4	CACACACACACACAC:0.075
chr28	9828679	2	38	C:0.789474	A:0.210526		

chr28	9828690	2	40	TACAA:0.2	T:0.8	
chr28	9828927	2	40	C:0.25	T:0.75	
chr28	9829025	2	40	C:0.7	T:0.3	
chr28	9829252	2	40	T:0.25	C:0.75	
chr28	9829295	2	38	T:0.157895	A:0.842105	
chr28	9829352	2	40	T:0.225	G:0.775	
chr28	9829354	2	40	G:0.225	A:0.775	
chr28	9829438	2	38	G:0.184211	A:0.815789	
chr28	9830182	2	38	G:0.184211	T:0.815789	
chr28	9830309	2	38	T:0.210526	TA:0.789474	
chr28	9830679	2	40	T:0.025	TA:0.975	
chr28	9830811	2	38	C:1	A:0	
chr28	9831040	2	38	G:0.184211	A:0.815789	
chr28	9831068	2	38	C:0.263158	T:0.736842	
chr28	9831687	2	40	T:0.25	C:0.75	
chr28	9831751	2	40	T:0.2	C:0.8	
chr28	9832026	2	40	A:1	G:0	
chr28	9832433	2	38	T:1	C:0	
chr28	9832538	2	40	A:0.2	G:0.8	
chr28	9832873	2	36	CT:0.166667	C:0.833333	
chr28	9833889	2	36	CTT:1	C:0	
chr28	9833997	2	40	T:0.2	C:0.8	
chr28	9834032	2	30	G:0.333333	C:0.666667	
chr28	9834344	2	38	G:0.157895	A:0.842105	
chr28	9834526	2	40	G:0.7	A:0.3	
chr28	9834887	2	34	T:0.205882	G:0.794118	
chr28	9835154	2	40	T:0.75	C:0.25	
chr28	9835283	2	40	T:0.675	G:0.325	
chr28	9835327	2	38	T:0.657895	C:0.342105	
chr28	9836035	2	38	C:0.763158	G:0.236842	
chr28	9836232	2	38	T:0.605263	TGAGAGA:0.394737	
chr28	9836370	2	34	C:0.529412	T:0.470588	
chr28	9836413	2	36	ATGTG:0.472222	A:0.527778	
chr28	9836440	2	36	TGTGTGC:0.666667	T:0.333333	
chr28	9836868	2	40	C:0.675	T:0.325	
chr28	9836958	2	38	C:1	T:0	
chr28	9837299	2	38	C:1	T:0	
chr28	9837424	2	38	C:0.763158	G:0.236842	
chr28	9837820	2	38	C:0.631579	T:0.368421	
chr28	9838398	2	40	A:0.7	G:0.3	
chr28	9838524	2	40	G:0.675	A:0.325	
chr28	9838729	2	40	G:0.6	A:0.4	
chr28	9839753	3	36	G:0.361111	GAT:0.472222	GATAT:0.166667
chr28	9839853	2	30	C:0.233333	CCT:0.766667	
chr28	9840164	2	40	A:1	G:0	
chr28	9840384	2	38	T:0.684211	G:0.315789	
chr28	9840452	2	40	A:0.6	T:0.4	
chr28	9840554	2	40	T:0.625	C:0.375	
chr28	9841083	2	40	C:0.5	G:0.5	
chr28	9841148	2	38	C:1	T:0	
chr28	9841160	2	38	G:0.736842	A:0.263158	
chr28	9842075	2	40	G:0.775	A:0.225	
chr28	9842205	3	40	T:0.7	TAA:0.25	TAAA:0.05

chr28 9842215 3 40 TA:0.225 T:0.475 AA:0.3
chr28 9842217 2 40 A:0.975 T:0.025
chr28 9842220 2 40 A:1 T:0
chr28 9842238 3 40 A:0.2 AT:0.5 AAT:0.3
chr28 9842322 2 40 C:0.675 T:0.325
chr28 9842442 2 38 G:0.684211 C:0.315789
chr28 9842543 2 40 A:0.625 T:0.375
chr28 9842745 2 38 C:0.605263 T:0.394737
chr28 9843409 2 36 T:0.722222 C:0.277778
chr28 9843430 2 36 T:0.722222 C:0.277778
chr28 9843567 2 32 T:0.65625 TAA:0.34375
chr28 9843654 2 32 G:1 C:0
chr28 9843795 2 2 C:1 T:0
chr28 9843984 2 40 T:0.575 C:0.425
chr28 9843998 2 40 G:1 A:0
chr28 9844064 2 40 G:1 GAT:0
chr28 9844773 2 38 C:1 T:0
chr28 9844863 3 34 CA:0.588235 C:0 CAA:
0.411765
chr28 9844902 2 36 C:0.666667 T:0.333333
chr28 9845152 2 38 G:0.684211 A:0.315789
chr28 9845180 2 38 A:0.684211 T:0.315789
chr28 9845491 2 40 A:0.725 G:0.275
chr28 9845837 2 40 TA:0.675 T:0.325
chr28 9845981 2 38 A:0.631579 G:0.368421
chr28 9846137 2 38 A:0.684211 G:0.315789
chr28 9846192 3 40 CAGAG:0.55 C:0.275 CAGAGAG:
0.175
chr28 9846229 2 36 G:0.638889 C:0.361111
chr28 9846345 2 36 TC:0.694444 T:0.305556
chr28 9846363 3 38 T:0.236842 TA:0.631579
TAA:0.131579
chr28 9846436 2 40 G:0.95 A:0.05
chr28 9846461 2 40 C:0.725 T:0.275
chr28 9846494 2 40 T:0.625 C:0.375
chr28 9846687 2 38 C:0.710526 CCT:0.289474
chr28 9846729 2 38 AT:0.5 A:0.5
chr28 9846731 2 38 TTA:1 T:0
chr28 9846732 2 38 TA:0.526316 T:0.473684
chr28 9847396 2 38 C:0.657895 T:0.342105
chr28 9847506 2 40 T:0 C:1
chr28 9847533 2 36 A:0.666667 C:0.333333
chr28 9848139 2 38 T:0.973684 C:0.0263158
chr28 9848246 2 36 G:0.777778 A:0.222222
chr28 9848298 2 40 A:0.1 G:0.9
chr28 9849306 2 38 C:0.131579 T:0.868421
chr28 9849486 2 40 A:0.95 AC:0.05
chr28 9849721 2 40 A:0.15 C:0.85
chr28 9849820 2 38 CACA:0.157895 C:0.842105
chr28 9849928 2 40 A:0.125 T:0.875
chr28 9850403 2 38
CTAGTAGCACCTAGTAGGTGGGAGAGTGAAGCGGGGGCGAAG:0.0789474 C:
0.921053
chr28 9850627 2 40 A:0.975 G:0.025

chr28	9850811	2	36	AT:0.361111	A:0.638889		
chr28	9850888	2	40	C:0.3	CT:0.7		
chr28	9850891	2	40	A:0.3	T:0.7		
chr28	9851276	2	40	A:0.225	C:0.775		
chr28	9851497	2	36	A:0.944444	G:0.0555556		
chr28	9851627	2	40	G:0.325	A:0.675		
chr28	9851629	2	40	TC:1	T:0		
chr28	9851788	2	36	C:1	T:0		
chr28	9851888	2	40	A:0.975	T:0.025		
chr28	9852068	2	40	TGGG:0.275	T:0.725		
chr28	9852170	2	38	ACCT:0.526316	A:0.473684		
chr28	9852576	2	38	A:0.947368	G:0.0526316		
chr28	9852994	2	40	A:0.95	T:0.05		
chr28	9853053	2	40	G:0.5	A:0.5		
chr28	9853304	2	38	C:0.315789	T:0.684211		
chr28	9853494	2	40	A:0.425	G:0.575		
chr28	9853508	2	38	A:0.315789	G:0.684211		
chr28	9853632	2	38	C:0.368421	CGAGG:0.631579		
chr28	9853923	2	34	C:0.823529	A:0.176471		
chr28	9854234	2	36	C:0.416667	T:0.583333		
chr28	9854412	2	36	A:0.5	T:0.5		
chr28	9854497	2	40	AAC:0.95	A:0.05		
chr28	9854514	2	40	C:0.375	G:0.625		
chr28	9854797	2	38	G:0.447368	A:0.552632		
chr28	9855172	2	40	A:0.35	G:0.65		
chr28	9855239	3	38	C:0.157895	CT:0.236842	CTT:0.605263	
chr28	9855333	2	40	A:0.425	C:0.575		
chr28	9855871	2	38	G:0.5	A:0.5		
chr28	9856031	2	38	A:0.394737	AG:0.605263		
chr28	9856260	2	38	G:0.368421	A:0.631579		
chr28	9856378	2	38	G:0.394737	C:0.605263		
chr28	9856454	2	36	CA:0.944444	C:0.0555556		
chr28	9856687	2	40	A:0.475	G:0.525		
chr28	9856755	2	38	C:0.526316	T:0.473684		
chr28	9856858	2	40	C:1	T:0		
chr28	9857134	2	40	T:0.4	C:0.6		
chr28	9858708	2	34	C:1	T:0		
chr28	9859278	2	38	C:0.5	T:0.5		
chr28	9861136	4	40	CCT:0.8	C:0.1	CCTCT:0.05	CCTCTCT:0.05
chr28	9861190	3	36	TA:0.305556	T:0.111111	TAA:0.583333	
chr28	9861661	2	36	C:1	T:0		
chr28	9862142	2	36	C:0.333333	T:0.666667		
chr28	9862228	2	32	GGAGAGA:0.6875	G:0.3125		
chr28	9862257	2	36	GAGAGA:0.75	G:0.25		
chr28	9862263	4	38	GGC:0.657895	GCGC:0.289474	G:0.0526316	GGCGC:0
chr28	9862625	2	40	T:1	C:0		
chr28	9862626	2	40	C:1	T:0		
chr28	9862661	2	38	C:1	T:0		
chr28	9862727	2	40	C:0.975	G:0.025		
chr28	9862798	2	40	G:1	A:0		

chr28	9863474	2	40	C:1	A:0		
chr28	9863582	2	40	A:1	ATG:0		
chr28	9863699	2	40	G:0.5	A:0.5		
chr28	9863896	2	40	C:1	T:0		
chr28	9864348	2	36	G:1	A:0		
chr28	9864350	2	36	T:1	TA:0		
chr28	9864458	2	36	C:0.944444	T:0.0555556		
chr28	9864476	2	36	A:0.416667	G:0.583333		
chr28	9864707	2	38	T:1	A:0		
chr28	9864884	2	40	G:0.375	A:0.625		
chr28	9865059	2	36	A:1	G:0		
chr28	9865602	2	38	C:0.894737	A:0.105263		
chr28	9865684	2	40	A:0.425	G:0.575		
chr28	9865692	2	40	G:1	T:0		
chr28	9865757	2	40	C:1	A:0		
chr28	9865970	2	40	C:1	T:0		
chr28	9866030	2	40	C:1	T:0		
chr28	9866648	4	40	TTTTA:0.175	T:0.05	TTTTATTTA:0.775	TTTTATTTATTTA:0
chr28	9866773	2	36	C:1	T:0		
chr28	9867334	2	40	G:1	A:0		
chr28	9867590	2	40	G:1	A:0		
chr28	9867754	2	38	T:0.421053	C:0.578947		
chr28	9867842	2	38	C:0.973684	G:0.0263158		
chr28	9867917	2	34	C:1	G:0		
chr28	9868206	2	40	A:0.6	T:0.4		
chr28	9868291	2	40	T:0.975	G:0.025		
chr28	9868294	2	40	T:0.425	C:0.575		
chr28	9868531	2	36	A:0.611111	C:0.388889		
chr28	9868569	2	36	T:0.972222	C:0.0277778		
chr28	9868623	2	40	A:0.55	G:0.45		
chr28	9868641	2	40	G:0.575	A:0.425		
chr28	9868719	2	38	G:0.605263	T:0.394737		
chr28	9868812	2	36	C:0.583333	T:0.416667		
chr28	9868859	2	38	A:1	C:0		
chr28	9868965	2	36	TG:0.444444	T:0.555556		
chr28	9869492	2	36	T:0.5	TA:0.5		
chr28	9869832	2	40	C:0.975	T:0.025		
chr28	9869902	2	38	C:1	T:0		
chr28	9870157	2	40	ATTAGT:0.9	A:0.1		
chr28	9871663	2	40	A:0.5	G:0.5		
chr28	9872139	2	40	T:0.475	C:0.525		
chr28	9872724	2	38	T:0.421053	C:0.578947		
chr28	9872888	2	36	T:0.416667	A:0.583333		
chr28	9872930	2	32	G:0.4375	T:0.5625		
chr28	9873655	2	38	G:0.973684	T:0.0263158		
chr28	9874827	2	38	G:0.473684	C:0.526316		
chr28	9875173	2	38	A:1	G:0		
chr28	9875181	2	38	G:0.973684	T:0.0263158		
chr28	9875347	2	40	G:0.525	A:0.475		
chr28	9875558	2	40	T:0.6	C:0.4		
chr28	9875957	2	40	GA:0.5	G:0.5		
chr28	9876029	2	36	C:0.583333	T:0.416667		
chr28	9876312	2	38	A:1	G:0		

chr28	9876381	2	40	T:1	TGGAGGGCCAC:0	
chr28	9876550	2	36	G:0.5	T:0.5	
chr28	9877259	2	40	T:0.6	C:0.4	
chr28	9877592	2	36	T:0.555556	G:0.444444	
chr28	9877635	2	38	G:0.5	GA:0.5	
chr28	9877656	2	40	T:0.375	A:0.625	
chr28	9877730	2	40	A:0.525	G:0.475	
chr28	9878408	2	32	C:0.53125	G:0.46875	
chr28	9878831	2	40	G:0.9	A:0.1	
chr28	9878967	2	40	G:0.45	A:0.55	
chr28	9879096	2	38	T:0.947368	A:0.0526316	
chr28	9879617	2	40	G:1	T:0	
chr28	9880012	2	38	C:0.105263	T:0.894737	
chr28	9880112	2	34	C:0.117647	T:0.882353	
chr28	9880193	2	40	G:0.95	A:0.05	
chr28	9880387	2	40	C:0.825	G:0.175	
chr28	9880464	2	40	CCA:0.975	C:0.025	
chr28	9880508	2	36	G:0.138889	A:0.861111	
chr28	9880510	2	36	A:0.0555556	G:0.944444	
chr28	9880537	2	36	A:0.138889	G:0.861111	
chr28	9880570	2	38	G:0.947368	A:0.0526316	
chr28	9880870	2	38	T:0.157895	TC:0.842105	
chr28	9880874	2	38	T:0.157895	TCTACCA:0.842105	
chr28	9880964	2	38	A:0.131579	G:0.868421	
chr28	9881334	2	34	C:0.882353	G:0.117647	
chr28	9881668	2	38	C:1	T:0	
chr28	9881779	2	40	G:0.95	T:0.05	
chr28	9881780	3	34	AT:0.529412	A:0.352941	ATT:0.117647
chr28	9881785	2	40	T:0.95	A:0.05	
chr28	9882122	2	40	A:0.9	G:0.1	
chr28	9882387	2	38	C:0.894737	A:0.105263	
chr28	9882497	2	34	T:0.970588	C:0.0294118	
chr28	9882690	2	38	T:1	A:0	
chr28	9882745	2	40	G:0.825	A:0.175	
chr28	9882816	2	36	G:0.944444	A:0.0555556	
chr28	9882890	2	38	T:0	C:1	
chr28	9882931	2	40	G:1	A:0	
chr28	9882979	2	38	G:1	A:0	
chr28	9883080	2	38	G:0.868421	C:0.131579	
chr28	9883168	2	40	CAG:0.15	C:0.85	
chr28	9883170	2	40	GA:0.95	G:0.05	
chr28	9883353	2	34	T:0.205882	C:0.794118	
chr28	9883357	2	36	C:1	T:0	
chr28	9883364	2	36	A:0.166667	G:0.833333	
chr28	9883521	2	34	C:0.176471	T:0.823529	
chr28	9883583	2	28	C:0.178571	CCG:0.821429	
chr28	9883586	2	28	A:0.178571	C:0.821429	
chr28	9883756	2	36	AT:0.166667	A:0.833333	
chr28	9883762	2	36	G:0.916667	C:0.0833333	
chr28	9884133	2	38	G:0.894737	A:0.105263	
chr28	9884153	2	40	TA:1	T:0	
chr28	9884223	2	40	G:0.925	A:0.075	
chr28	9884383	2	38	T:0.0526316	C:0.947368	

chr28	9884555	2	36	T:0.888889	G:0.111111		
chr28	9884681	2	36	A:0.888889	G:0.111111		
chr28	9884689	2	38	G:1	A:0		
chr28	9884923	2	40	A:0.8	G:0.2		
chr28	9884992	2	40	A:0.15	T:0.85		
chr28	9885099	4	40	T:0.1	TA:0.1	TAA:0.025	TAAAA:0.775
chr28	9885950	2	40	ATCT:0.95	A:0.05		
chr28	9886081	2	36	T:1	C:0		
chr28	9886147	2	36	T:0.0833333	C:0.916667		
chr28	9886881	2	38	A:1	T:0		
chr28	9887013	2	38	A:0.947368	G:0.0526316		
chr28	9887087	2	40	A:1	C:0		
chr28	9887412	2	38	C:1	T:0		
chr28	9887429	2	40	T:0.95	G:0.05		
chr28	9887864	2	38	A:1	G:0		
chr28	9888421	2	38	G:1	A:0		
chr28	9888500	2	34	C:0.941176	A:0.0588235		
chr28	9889061	2	38	C:1	A:0		
chr28	9889248	2	38	A:0.921053	G:0.0789474		
chr28	9889768	2	40	TA:0.225	T:0.775		
chr28	9889985	2	38	T:1	A:0		
chr28	9890004	2	36	G:0.305556	T:0.694444		
chr28	9890114	2	38	T:0.947368	G:0.0526316		
chr28	9890129	2	38	T:0.947368	A:0.0526316		
chr28	9890444	2	40	C:0.95	T:0.05		
chr28	9890455	2	40	C:0.95	T:0.05		
chr28	9890473	2	40	A:0.95	AC:0.05		
chr28	9890512	2	40	G:0.925	A:0.075		
chr28	9890523	2	40	G:0.925	T:0.075		
chr28	9890737	2	38	A:0.894737	T:0.105263		
chr28	9891212	2	40	G:0.95	A:0.05		
chr28	9891337	2	40	G:1	T:0		
chr28	9891404	2	38	C:0.236842	T:0.763158		
chr28	9891610	2	40	C:0.925	CT:0.075		
chr28	9891719	2	38	C:0.973684	T:0.0263158		
chr28	9891795	2	40	TG:0.925	T:0.075		
chr28	9892170	2	38	C:0.947368	T:0.0526316		
chr28	9892247	2	40	A:0.9	G:0.1		
chr28	9892299	2	40	T:0.95	C:0.05		
chr28	9892543	2	38	A:0.921053	C:0.0789474		
chr28	9892591	2	38	T:0.947368	G:0.0526316		
chr28	9892766	2	36	A:0.944444	G:0.0555556		
chr28	9892877	2	40	C:0.3	A:0.7		
chr28	9892943	2	36	C:0.944444	T:0.0555556		
chr28	9893728	2	40	T:0.325	G:0.675		
chr28	9893742	2	40	G:0.95	C:0.05		
chr28	9893744	2	40	A:0.95	G:0.05		
chr28	9893812	2	38	A:1	G:0		
chr28	9893869	2	36	C:1	G:0		
chr28	9894369	2	38	C:0.947368	CTT:0.0526316		
chr28	9894394	2	36	C:0.305556	T:0.694444		
chr28	9894917	2	40	A:0.95	G:0.05		
chr28	9894930	2	40	C:1	T:0		

chr28	9895380	2	38	T:1	C:0	
chr28	9895399	2	40	G:1	A:0	
chr28	9895686	2	40	C:1	T:0	
chr28	9895992	2	28	CT:0.25	C:0.75	
chr28	9897009	2	30	C:0.233333		T:0.766667
chr28	9897107	2	28	C:0.714286		T:0.285714
chr28	9898734	2	34	G:0.735294		A:0.264706
chr28	9898990	2	38	A:1	T:0	
chr28	9899121	2	40	A:0.95	AT:0.05	
chr28	9900019	2	38	C:1	T:0	
chr28	9900260	2	40	T:0.225	C:0.775	
chr28	9902271	2	40	C:1	A:0	
chr28	9902493	2	40	GTA:0.95	G:0.05	
chr28	9902507	2	40	A:0.475	T:0.525	
chr28	9902509	2	40	A:0.3	T:0.7	
chr28	9902511	2	40	A:0.3	T:0.7	
chr28	9902600	2	40	T:0.55	C:0.45	
chr28	9903002	2	36	C:0.444444		CA:0.555556
chr28	9903712	2	40	C:0.775	T:0.225	
chr28	9904554	2	40	G:0.8	A:0.2	
chr28	9904665	2	36	CT:0.527778		C:0.472222
chr28	9905089	2	40	C:0.5	A:0.5	
chr28	9905601	2	40	A:0.8	T:0.2	
chr28	9905625	2	40	T:0.475	C:0.525	
chr28	9906398	3	40	C:0.4	CA:0.35	CAA:0.25
chr28	9906895	2	40	T:0.95	A:0.05	
chr28	9908236	2	36	C:1	T:0	
chr28	9908829	2	40	TAC:0.925		T:0.075
chr28	9909116	2	36	A:0.472222		T:0.527778
chr28	9909253	3	36	TA:0.527778		T:0.361111
TAA:0.111111						
chr28	9909408	2	38	G:0.473684		A:0.526316
chr28	9910182	2	40	G:0.75	A:0.25	
chr28	9910194	2	40	T:1	C:0	
chr28	9910897	2	40	C:0.525	CAA:0.475	
chr28	9910907	2	40	A:1	C:0	
chr28	9910908	2	40	C:0.35	A:0.65	
chr28	9911166	2	40	TACAGAC:0.45		T:0.55
chr28	9911617	2	40	C:0.55	A:0.45	
chr28	9912096	2	40	TA:0.625	T:0.375	
chr28	9912205	2	36	C:0.805556		T:0.194444
chr28	9912296	2	38	G:1	A:0	
chr28	9912299	2	40	C:1	A:0	
chr28	9912740	2	40	G:1	A:0	
chr28	9913290	2	36	G:1	A:0	
chr28	9914398	2	40	G:0.975	GA:0.025	
chr28	9914533	2	36	C:0.777778		CT:0.222222
chr28	9914702	2	40	C:0.75	T:0.25	
chr28	9915736	3	38	T:0.368421		TA:0.552632
TAA:0.0789474						
chr28	9916602	2	38	C:0.763158		T:0.236842
chr28	9916893	2	38	AGG:0.5	A:0.5	
chr28	9916896	2	38	T:0.5	TAG:0.5	
chr28	9916898	3	38	AAATGAT:0		A:0.5

ATAGTAATGAT:0.5
chr28 9917159 2 40 G:0.45 A:0.55
chr28 9917550 2 38 G:0.710526 A:0.289474
chr28 9917873 2 40 T:0.825 A:0.175
chr28 9917902 2 40 C:0.275 CCA:0.725
chr28 9917957 2 40 C:0.325 G:0.675
chr28 9918034 2 38 C:0.447368 T:0.552632
chr28 9918417 2 40 A:0.525 G:0.475
chr28 9918510 4 40 ATGTGTGTG:0.15 A:0.475 ATGTGTG:
0.3 ATGTGTGTGTGTGTG:0.075
chr28 9918860 2 40 T:0.55 C:0.45
chr28 9918865 2 40 T:0.575 TA:0.425
chr28 9920614 2 40 G:1 C:0
chr28 9920691 2 36 A:0.694444 C:0.305556
chr28 9921321 2 38 A:0.447368 G:0.552632
chr28 9921393 2 38 A:0.815789 T:0.184211
chr28 9921431 2 38 T:1 TA:0
chr28 9921727 2 40 A:0.95 T:0.05
chr28 9921736 2 40 T:0.3 TAAAG:0.7
chr28 9921988 2 38 G:0.526316 A:0.473684
chr28 9922247 3 38 GAGAAAGAA:0.236842 G:
0.684211 GAGAA:0.0789474
chr28 9922309 2 38 GAGA:0.789474 G:0.210526
chr28 9922312 2 34 AAAAG:0.911765 A:0.0882353
chr28 9922350 2 38 AAGAAAGAAAGAAAAAG:0.789474
A:0.210526
chr28 9922389 3 40 AAAAGAAAGAAAG:0.8 A:0.05
AAAAGAAAG:0.15
chr28 9922484 2 38 A:0.605263 AAACAAC:0.394737
chr28 9922491 2 38 A:0.842105 T:0.157895
chr28 9923102 2 40 C:1 T:0
chr28 9923322 2 40 A:0.5 G:0.5
chr28 9923729 2 40 G:0.975 GA:0.025
chr28 9924429 2 40 T:0.5 C:0.5
chr28 9924670 2 40 G:0.475 GA:0.525
chr28 9925035 2 40 T:0.525 C:0.475
chr28 9925087 2 40 C:0.8 T:0.2
chr28 9925180 2 40 A:0.475 T:0.525
chr28 9925490 2 38 G:0.552632 C:0.447368
chr28 9925725 2 38 T:0.236842 C:0.763158
chr28 9927365 2 40 T:0.275 C:0.725
chr28 9927462 2 38 AAT:0.894737 A:0.105263
chr28 9927599 2 38 C:0.605263 T:0.394737
chr28 9928055 2 38 A:0.921053 T:0.0789474
chr28 9928142 2 40 A:1 T:0
chr28 9928654 2 38 C:0.0526316 T:0.947368
chr28 9929062 2 40 CCTCT:0.6 C:0.4
chr28 9929534 2 40 CAAAAA:0.8 C:0.2
chr28 9929677 2 20 T:0.55 TA:0.45
chr28 9930040 2 38 TA:0.5 T:0.5
chr28 9930276 2 38 TA:0.605263 T:0.394737
chr28 9931128 2 24 TA:0.625 T:0.375
chr28 9932271 2 40 C:0.5 T:0.5
chr28 9932368 2 36 T:0.805556 C:0.194444

chr28 9932584 2 32 T:0.4375 C:0.5625
chr28 9932724 2 38 C:0.631579 G:0.368421
chr28 9932870 6 40 AATAGATAG:0.15 A:0.075 AATAG:
0.15 AATAGATAGATAG:0.275 AATAGATAGATAGATAG:0.225
AATAGATAGATAGATAGATAG:0.125
chr28 9934201 2 40 G:1 A:0
chr28 9934316 2 38 C:0.789474 T:0.210526
chr28 9934493 2 40 A:0.225 G:0.775
chr28 9934961 2 38 C:0.526316 CA:0.473684
chr28 9935090 2 40 AT:0.975 A:0.025
chr28 9935179 2 38 G:0.473684 A:0.526316
chr28 9935476 2 40 T:0.95 G:0.05
chr28 9935849 3 36 T:0.75 TA:0.25 TAA:0
chr28 9936028 2 40 GA:0 G:1
chr28 9936036 2 40 G:0 GA:1
chr28 9936130 2 36 G:0.416667 A:0.583333
chr28 9938943 3 38 C:0.736842 CA:0.0526316
CAA:0.210526
chr28 9939204 2 34 AAC:0.676471 A:0.323529
chr28 9939206 2 34 C:0.882353 A:0.117647
chr28 9940272 2 36 A:0.833333 G:0.166667
chr28 9940348 2 38 TTTC:0.842105 T:0.157895
chr28 9942369 2 40 GGAGA:1 G:0
chr28 9944305 2 40 T:0.45 C:0.55
chr28 9945252 2 34 T:0.676471 C:0.323529
chr28 9945832 2 38 TA:0.421053 T:0.578947
chr28 9947583 2 36 T:1 C:0
chr28 9947736 3 38 A:0.710526 AG:0.236842
AGG:0.0526316
chr28 9947747 3 38 A:0.5 AG:0.447368 G:
0.0526316
chr28 9947926 2 40 C:1 A:0
chr28 9947989 2 38 C:0.947368 CA:0.0526316
chr28 9948544 2 38 A:0.973684 C:0.0263158
chr28 9949016 2 38 ACAAT:0.526316 A:0.473684
chr28 9949084 2 40 A:0.5 AT:0.5
chr28 9949465 2 40 T:0.8 A:0.2
chr28 9950701 2 38 T:1 C:0
chr28 9951013 2 36 A:0.416667 G:0.583333
chr28 9951541 2 40 C:0.525 CCTCT:0.475
chr28 9952690 2 38 C:0.842105 T:0.157895
chr28 9953010 2 40 A:1 T:0
chr28 9953318 2 34 CCT:0.323529 C:0.676471
chr28 9953671 2 40 C:0.95 T:0.05
chr28 9953923 3 40 G:0.7 GA:0.05 GAA:0.25
chr28 9954492 2 40 G:0.5 GA:0.5
chr28 9954881 2 38 T:0.578947 G:0.421053
chr28 9954891 2 38 AT:0.736842 A:0.263158
chr28 9954901 2 38 T:0.605263 A:0.394737
chr28 9955119 2 38 T:0.552632 TA:0.447368
chr28 9955544 2 36 G:0.833333 T:0.166667
chr28 9955881 2 38 C:0.526316 G:0.473684
chr28 9956105 2 36 C:0.972222 T:0.0277778
chr28 9956137 3 38 G:0.421053 GTCTC:0.315789

GTCTCTC:0.263158
chr28	9956173	2	34	TA:0.5	T:0.5
chr28	9956424	2	36	A:0.472222	G:0.527778
chr28	9956653	2	40	C:0.5	T:0.5
chr28	9957148	2	40	T:0.45	C:0.55
chr28	9957756	2	38	T:0.421053	A:0.578947
chr28	9957779	2	40	T:0.95	TAAAA:0.05
chr28	9957785	2	40	T:0.9	TAA:0.1
chr28	9957787	2	40	A:0.45	AAT:0.55
chr28	9957794	3	40	TA:0.325	AA:0.125	T:0.55
chr28	9957796	2	38	A:0.921053	AT:0.0789474
chr28	9957802	2	40	A:0.45	T:0.55
chr28	9957821	2	40	T:0.45	C:0.55
chr28	9957952	5	40	CCTCTCTCTCTCTCTCTCT:0.225	C:0.525
CCTCTCTCTCT:0.2	CCTCTCTCTCTCT:0	CCTCTCTCTCTCTCT:0.05
chr28	9958001	2	38	CATAA:0.605263	C:0.394737
chr28	9958025	3	40	A:1	C:0	AATAC:0
chr28	9958242	2	40	CT:1	C:0
chr28	9958526	2	40	A:0.15	T:0.85
chr28	9958909	2	38	C:0.842105	T:0.157895
chr28	9959119	2	38	T:0.263158	C:0.736842
chr28	9959432	2	38	G:0.736842	A:0.263158
chr28	9959710	2	38	C:1	G:0
chr28	9959963	2	40	T:0.775	C:0.225
chr28	9960316	2	36	TA:0.833333	T:0.166667
chr28	9960317	2	38	A:0.789474	T:0.210526
chr28	9960471	2	38	TG:0.921053	T:0.0789474
chr28	9960605	3	38	TA:0.526316	T:0.263158
TAA:0.210526
chr28	9960622	2	38	T:0.578947	TCTG:0.421053
chr28	9960700	2	38	G:0.736842	A:0.263158
chr28	9960975	2	40	A:1	T:0
chr28	9961089	2	40	C:0.8	T:0.2
chr28	9961105	2	40	C:0.975	T:0.025
chr28	9961338	2	40	A:0.525	AG:0.475
chr28	9961482	2	40	C:0.55	CT:0.45
chr28	9961588	2	38	T:1	TC:0
chr28	9961876	2	38	A:0.763158	G:0.236842
chr28	9962049	2	40	CAT:0.75	C:0.25
chr28	9962281	2	38	CCT:0.763158	C:0.236842
chr28	9962470	2	40	T:0.95	C:0.05
chr28	9962583	2	40	A:1
AAGACATCACCCAGTCTACGGCCATACCACCCTGAACGCGCCCGATCTCGTCTGATCTCGGAAGCTAA
GCAGGGTCGGGCCTGGTTAGTACTTGGATGGG:0
chr28	9962672	2	38	T:0.763158	C:0.236842
chr28	9963026	2	40	C:0.825	T:0.175
chr28	9963417	2	40	C:0.75	T:0.25
chr28	9963449	2	40	G:1	A:0
chr28	9963862	2	38	C:0.368421	T:0.631579
chr28	9964118	2	36	C:0.833333	A:0.166667
chr28	9964363	2	40	T:0.75	C:0.25
chr28	9964449	2	40	C:0.7	CCTCT:0.3
chr28	9964485	4	38	T:0.315789	TA:0.447368
TAA:0.236842	TAAA:0

chr28	9964630	2	38	C:0.763158	CCTCT:0.236842		
chr28	9964740	2	40	GA:0.25	G:0.75		
chr28	9964838	2	40	A:0.25	G:0.75		
chr28	9965642	2	38	G:0.631579	A:0.368421		
chr28	9965676	2	40	G:0.75	C:0.25		
chr28	9967175	2	40	A:1	G:0		
chr28	9968483	2	36	A:0.472222	G:0.527778		
chr28	9969013	2	40	T:0.7	C:0.3		
chr28	9969507	2	40	AAAG:1	A:0		
chr28	9969512	2	40	A:1	C:0		
chr28	9969777	3	40	AAC:0.925	A:0.05	AACACAC:0.025	
chr28	9969871	2	38	G:1	A:0		
chr28	9969879	2	38	T:0.342105	C:0.657895		
chr28	9970039	2	38	CGGGAGCCCCGCGAT:0	C:1		
chr28	9971287	2	28	G:0.75	T:0.25		
chr28	9971366	2	30	C:0.9	A:0.1		
chr28	9971397	2	30	G:0.833333	T:0.166667		
chr28	9971574	2	38	C:0.710526	A:0.289474		
chr28	9971681	2	38	G:0.473684	C:0.526316		
chr28	9971687	2	38	A:0.947368	C:0.0526316		
chr28	9972155	2	38	C:1	CTTT:0		
chr28	9972167	2	38	T:0.5	TTA:0.5		
chr28	9972180	2	38	C:0.552632	T:0.447368		
chr28	9972222	2	36	A:1	T:0		
chr28	9972623	2	32	AT:0.9375	A:0.0625		
chr28	9972653	4	36	GT:0.277778	G:0.25	GTTT:0.0555556	GTTTT:0.416667
chr28	9972690	3	36	CAGAG:0.472222	C:0.527778	CAGAGAG:0	
chr28	9973458	2	40	G:0.575	A:0.425		
chr28	9973537	2	40	A:0.425	AAC:0.575		
chr28	9973915	2	40	C:1	CAA:0		
chr28	9974638	2	40	T:0.45	C:0.55		
chr28	9974833	2	40	C:0.475	T:0.525		
chr28	9974848	2	40	C:0.925	T:0.075		
chr28	9974904	2	38	A:0	G:1		
chr28	9974936	2	38	C:0.447368	T:0.552632		
chr28	9975592	3	36	C:0.694444	CACAG:0.305556	CACACAG:0	
chr28	9975627	2	32	A:0.53125	G:0.46875		
chr28	9975839	2	40	T:1	C:0		
chr28	9975887	2	36	T:0.472222	C:0.527778		
chr28	9975979	2	40	A:0.475	C:0.525		
chr28	9976067	2	40	A:0.5	T:0.5		
chr28	9976079	2	40	C:0.5	T:0.5		
chr28	9976406	2	40	T:0.475	A:0.525		
chr28	9976412	2	40	T:0.475	C:0.525		
chr28	9976549	2	36	C:0.444444	T:0.555556		
chr28	9976570	2	36	GTC:0.5	G:0.5		
chr28	9976621	2	38	T:0.526316	G:0.473684		
chr28	9976866	2	38	T:0.368421	TAG:0.631579		
chr28	9977173	2	38	G:1	A:0		
chr28	9977269	2	40	G:0.375	A:0.625		

chr28	9977368	2	40	G:0.475	A:0.525	
chr28	9977531	2	40	C:0.45	CA:0.55	
chr28	9977551	3	40	C:0.975	CA:0.025	CAA:0
chr28	9977615	2	40	T:0.55	C:0.45	
chr28	9977652	2	40	G:1	A:0	
chr28	9977724	2	40	G:0.45	A:0.55	
chr28	9977937	2	40	A:0.5	G:0.5	
chr28	9978248	2	38	A:0.473684	G:0.526316	
chr28	9978328	2	34	A:1	G:0	
chr28	9978417	2	38	T:0.394737	C:0.605263	
chr28	9978479	2	36	A:1	G:0	
chr28	9978950	2	34	T:0.470588	C:0.529412	
chr28	9979983	2	38	C:1	T:0	
chr28	9980549	2	38	C:0.447368	T:0.552632	
chr28	9980829	2	38	T:0.447368	C:0.552632	
chr28	9980925	2	38	C:1	T:0	
chr28	9981357	2	36	G:1	A:0	
chr28	9981483	2	38	G:1	A:0	
chr28	9981866	2	40	A:0.975	ATCCCTGGG:0.025	
chr28	9981870	2	40	C:0.975	CGCAGCGGT:0.025	
chr28	9981975	2	38	T:0.473684	C:0.526316	
chr28	9982005	2	38	A:0.421053	G:0.578947	
chr28	9982050	2	36	C:0.916667	T:0.0833333	
chr28	9982104	2	40	C:0.4	T:0.6	
chr28	9982112	2	40	G:0.4	A:0.6	
chr28	9982162	3	38	CCTCTCT:0.421053	C:0.526316	CCTCT:0.0526316
chr28	9982193	2	34	C:0.470588	G:0.529412	
chr28	9982831	2	40	C:0.425	G:0.575	
chr28	9982863	2	40	C:0.5	T:0.5	
chr28	9983073	2	36	C:0.416667	T:0.583333	
chr28	9983260	2	38	G:0.368421	A:0.631579	
chr28	9983295	2	40	C:0.425	A:0.575	
chr28	9983396	2	36	G:1	A:0	
chr28	9983522	2	40	G:0.5	A:0.5	
chr28	9983533	2	40	T:0.475	C:0.525	
chr28	9983570	2	40	C:0.425	T:0.575	
chr28	9984009	2	38	T:0.421053	C:0.578947	
chr28	9984025	2	40	G:0.45	A:0.55	
chr28	9984223	2	40	C:1	G:0	
chr28	9984291	2	38	C:0.394737	G:0.605263	
chr28	9984452	2	40	T:1	C:0	
chr28	9984534	2	40	C:0.425	G:0.575	
chr28	9984633	2	40	G:1	A:0	
chr28	9984635	2	40	G:0.5	C:0.5	
chr28	9984850	2	40	C:1	T:0	
chr28	9984907	2	40	T:0.45	TA:0.55	
chr28	9985389	2	36	A:1	G:0	
chr28	9985655	2	36	G:1	A:0	
chr28	9985722	2	38	T:0.105263	C:0.894737	
chr28	9985736	2	38	A:0.421053	T:0.578947	
chr28	9985765	2	36	T:1	A:0	
chr28	9985816	2	38	T:1	TA:0	
chr28	9986285	2	40	C:0.475	T:0.525	

chr28	9986551	2	40	T:1	C:0	
chr28	9986841	2	40	G:0.8	T:0.2	
chr28	9986915	2	38	C:1	G:0	
chr28	9987186	2	40	T:0.5	TAG:0.5	
chr28	9987300	2	40	A:1	T:0	
chr28	9987449	2	40	C:1	T:0	
chr28	9987855	2	38	C:1	A:0	
chr28	9987877	2	40	T:0.35	TA:0.65	
chr28	9987927	3	40	AT:0.525	A:0	ATT:0.475
chr28	9988108	2	38	A:1	T:0	
chr28	9988315	2	38	T:1	C:0	
chr28	9988511	2	24	C:0.458333		CA:0.541667
chr28	9989242	2	40	T:1	A:0	
chr28	9990006	2	36	C:1	A:0	
chr28	9990178	2	38	C:0.394737		T:0.605263
chr28	9990183	2	38	C:0.394737		T:0.605263
chr28	9990416	2	38	A:1	G:0	
chr28	9990557	2	38	T:1	A:0	
chr28	9990932	2	40	T:1	C:0	
chr28	9990959	2	38	T:1	C:0	
chr28	9990970	2	38	C:1	T:0	
chr28	9991065	2	40	C:0.5	T:0.5	
chr28	9991102	2	38	T:0.5	G:0.5	
chr28	9991563	2	40	A:1	T:0	
chr28	9991672	2	40	T:0.45	C:0.55	
chr28	9991885	2	34	C:1	T:0	
chr28	9992325	2	38	C:0.368421		T:0.631579
chr28	9992501	2	26	G:0.307692		A:0.692308
chr28	9992546	2	24	AGGAGG:0.291667		A:0.708333
chr28	9992570	2	24	G:0.291667		A:0.708333
chr28	9992722	2	36	G:0.388889		A:0.611111
chr28	9992731	2	36	C:0.388889		A:0.611111
chr28	9992882	2	38	A:1	G:0	
chr28	9992924	2	38	G:0.421053		T:0.578947
chr28	9992928	2	38	A:0.421053		G:0.578947
chr28	9992963	2	36	A:0.416667		G:0.583333
chr28	9993055	2	36	G:0.5	A:0.5	
chr28	9993060	2	36	C:0.5	T:0.5	
chr28	9993145	2	36	C:0.416667		T:0.583333
chr28	9993279	2	36	G:0.444444		A:0.555556
chr28	9993341	2	36	C:0.444444		T:0.555556
chr28	9993410	2	38	T:0.5	C:0.5	
chr28	9993413	2	38	T:0.5	TG:0.5	
chr28	9993962	2	38	C:0.342105		T:0.657895
chr28	9994647	2	36	C:0.416667		A:0.583333
chr28	9994812	2	40	T:0.35	C:0.65	
chr28	9995928	2	40	G:0.525	T:0.475	
chr28	9996072	2	40	G:0.475	A:0.525	
chr28	9996290	2	40	G:0.425	A:0.575	
chr28	9996574	2	40	AAGC:0.45		A:0.55
chr28	9996642	2	38	T:0.421053		C:0.578947
chr28	9997195	2	36	G:0.416667		T:0.583333
chr28	9997477	2	34	C:0.470588		G:0.529412
chr28	9997600	4	38	GATTTATTTATTTATTT:0.447368		

G:0.0263158	GATTTATTTATTTATTTATTT:0.184211
GATTTATTTATTTATTTATTTATTTATTTATTT:0.342105

chr28	9997720	2	36	C:1	T:0	
chr28	9997873	4	38	T:0.368421	TCACA:0.0789474	

TCACACA:0.526316 TCACACACA:0.0263158

chr28	9997890	2	38	C:0.947368	G:0.0526316
chr28	9998077	2	36	C:0.333333	T:0.666667
chr28	9998409	2	36	T:0.361111	C:0.638889
chr28	9998552	2	34	A:0.470588	G:0.529412
chr28	9999121	2	34	C:0.323529	T:0.676471
chr28	10000713	2	38	G:1	A:0
chr28	10001026	2	38	TA:0.421053	T:0.578947
chr28	10002489	2	38	C:0.421053	T:0.578947
chr28	10002497	2	38	GCTCT:0.421053	G:0.578947
chr28	10004928	2	40	GA:0.475	G:0.525
chr28	10004934	2	40	A:0.475	G:0.525
chr28	10004977	2	38	T:0.447368	C:0.552632
chr28	10005188	2	36	C:0.444444	A:0.555556
chr28	10005207	2	38	A:0.5	G:0.5
chr28	10005272	2	36	A:0.444444	G:0.555556
chr28	10005547	2	36	A:0.416667	T:0.583333
chr28	10005764	2	40	A:0.5	G:0.5
chr28	10006211	2	40	C:0.475	T:0.525
chr28	10006365	2	40	T:0.425	C:0.575
chr28	10006642	2	40	C:0.45	T:0.55
chr28	10006683	2	38	G:0.473684	A:0.526316
chr28	10006760	2	38	T:0.447368	TAGCCA:0.552632
chr28	10006950	2	38	G:0.447368	A:0.552632
chr28	10007006	2	40	G:0.475	T:0.525
chr28	10007231	2	38	C:1	T:0
chr28	10007530	2	36	G:0.444444	A:0.555556
chr28	10007900	2	38	G:0.394737	A:0.605263
chr28	10008098	2	38	G:0.947368	A:0.0526316
chr28	10008446	2	38	C:0.421053	G:0.578947
chr28	10009248	2	40	A:0.4	C:0.6
chr28	10009355	2	38	A:0.342105	C:0.657895
chr28	10009415	2	36	C:0.361111	T:0.638889
chr28	10009645	2	40	G:0.4	A:0.6
chr28	10009655	2	40	T:0.4	C:0.6
chr28	10010658	2	38	A:0.5	G:0.5
chr28	10010680	2	38	G:0.5	T:0.5
chr28	10010872	2	38	C:0.473684	T:0.526316
chr28	10011312	2	40	G:0.475	C:0.525
chr28	10011360	2	40	T:1	TCATC:0
chr28	10012507	2	36	T:1	C:0
chr28	10012544	2	40	G:1	A:0
chr28	10013329	2	40	G:0.35	T:0.65
chr28	10013461	2	38	C:0.368421	G:0.631579
chr28	10015175	2	38	AT:0.447368	A:0.552632
chr28	10015379	2	36	CT:0.75	C:0.25
chr28	10015437	2	38	G:1	A:0
chr28	10015601	2	40	C:0.9	CT:0.1
chr28	10015852	2	36	G:1	C:0
chr28	10015955	2	40	G:0.45	A:0.55

chr28	10015988	2	38	G:0.394737	A:0.605263
chr28	10016418	2	38	A:0.447368	G:0.552632
chr28	10016464	2	38	ACT:0.394737	A:0.605263
chr28	10016606	2	40	G:0.9	T:0.1
chr28	10016758	2	40	A:0.5	G:0.5
chr28	10016842	2	38	T:0.447368	C:0.552632
chr28	10017100	2	40	A:1	G:0
chr28	10018096	2	40	A:0.975	G:0.025
chr28	10018353	2	38	G:0.552632	T:0.447368
chr28	10020076	2	38	A:0.5	C:0.5
chr28	10020334	6	40	ATTATTTATTTATTTATTTATT:0.325
A:0	ATTATTTATTTATTTATTTATTTATT:0.05
ATATTTATTTATTTATTTATTTATTTATT:0.45
ATTATTTATTTATTTATTTATTTATTTATT:0.05
ATATTTATTTATTTATTTATTTATTTATTTATT:0.125
chr28	10020378	2	40	T:1	C:0
chr28	10020673	2	40	T:0.45	G:0.55
chr28	10020828	2	38	T:0.394737	C:0.605263
chr28	10021397	2	40	C:0.55	T:0.45
chr28	10022282	2	36	A:0.361111	G:0.638889
chr28	10023320	2	30	G:0.333333	A:0.666667
chr28	10024839	2	40	T:0.475	C:0.525
chr28	10024912	2	40	C:0.425	T:0.575
chr28	10025008	2	40	C:1	T:0
chr28	10025114	2	38	A:0.421053	G:0.578947
chr28	10025601	2	40	T:0.45	C:0.55
chr28	10026279	2	38	G:1	A:0
chr28	10026946	2	40	G:0.475	C:0.525
chr28	10027645	2	38	C:0.868421	T:0.131579
chr28	10027876	2	40	T:0.45	C:0.55
chr28	10028796	2	40	CCTTCCA:0.525	C:0.475
chr28	10028803	2	40	T:0.525	TA:0.475
chr28	10029083	2	38	G:1	A:0
chr28	10029354	2	36	G:0.333333	A:0.666667
chr28	10029920	2	38	G:0.421053	A:0.578947
chr28	10029933	2	36	GGA:0.916667	G:0.0833333
chr28	10030068	2	34	G:0.382353	A:0.617647
chr28	10030773	2	40	T:0.475	C:0.525
chr28	10030790	2	36	C:0.388889	CCT:0.611111
chr28	10030838	2	34	T:0.823529	TAA:0.176471
chr28	10030894	2	40	T:1	A:0
chr28	10031113	2	40	C:1	T:0
chr28	10031128	2	40	G:0.85	A:0.15
chr28	10031274	2	40	G:0.45	A:0.55
chr28	10031469	2	40	CATT:1	C:0
chr28	10031581	2	40	CA:1	C:0
chr28	10032164	2	40	C:0.375	CA:0.625
chr28	10032818	2	38	C:0.5	T:0.5
chr28	10032927	2	34	C:1	A:0
chr28	10033520	2	38	C:0.447368	T:0.552632
chr28	10034022	2	34	A:1	T:0
chr28	10034024	2	34	A:0.5	G:0.5
chr28	10034333	2	38	G:1	A:0
chr28	10034440	2	40	CCT:0.775	C:0.225

chr28	10034758	2	40	A:0.5	T:0.5	
chr28	10035134	2	38	CTCTT:1	C:0	
chr28	10035171	2	34	A:1	G:0	
chr28	10035173	2	32	TA:0.96875	T:0.03125	
chr28	10035182	2	34	A:0.705882	T:0.294118	
chr28	10035532	2	38	G:1	A:0	
chr28	10035735	2	40	G:1	A:0	
chr28	10035823	2	40	AAAAAAT:0.525	A:0.475	
chr28	10036775	2	38	T:1	C:0	
chr28	10037636	2	40	GC:1	G:0	
chr28	10037685	2	40	T:0.5	C:0.5	
chr28	10039396	3	40	TTCTCTC:0.45	T:0	TTCTCTCTC:0.55
chr28	10040013	2	40	C:1	G:0	
chr28	10040175	2	40	A:0.475	T:0.525	
chr28	10040882	2	38	A:0.947368	C:0.0526316	
chr28	10041010	2	38	A:0	G:1	
chr28	10041310	2	40	G:1	A:0	
chr28	10042479	2	40	G:1	A:0	
chr28	10042569	2	40	G:1	A:0	
chr28	10042632	2	38	C:1	T:0	
chr28	10042747	2	40	C:0.35	G:0.65	
chr28	10043976	2	36	A:0.333333	G:0.666667	
chr28	10044855	2	38	A:1	T:0	
chr28	10044930	2	40	A:1	T:0	
chr28	10044931	2	40	A:1	T:0	
chr28	10044951	2	40	C:0.4	T:0.6	
chr28	10045052	2	40	T:1	G:0	
chr28	10045135	2	40	T:1	A:0	
chr28	10045151	2	40	C:1	T:0	
chr28	10045236	2	40	GTAAA:1	G:0	
chr28	10045498	2	40	C:1	T:0	
chr28	10045566	2	36	T:1	A:0	
chr28	10045661	2	38	T:1	C:0	
chr28	10045823	2	40	A:1	G:0	
chr28	10046105	2	40	C:0.85	T:0.15	
chr28	10046511	2	38	C:1	CAA:0	
chr28	10046531	2	36	A:1	G:0	
chr28	10046533	2	36	AC:0.361111	A:0.638889	
chr28	10046770	2	38	C:0.368421	T:0.631579	
chr28	10046871	2	38	T:1	C:0	
chr28	10046949	2	40	C:1	T:0	
chr28	10047007	2	36	C:1	T:0	
chr28	10047016	2	38	GAA:1	G:0	
chr28	10047087	2	40	T:1	TG:0	
chr28	10047088	2	40	G:0.8	GT:0.2	
chr28	10047278	2	38	T:0.342105	C:0.657895	
chr28	10047881	2	36	G:1	A:0	
chr28	10049508	2	40	G:1	T:0	
chr28	10049599	2	36	CTT:0.5	C:0.5	
chr28	10050396	2	38	T:1	C:0	
chr28	10051303	2	40	G:1	A:0	
chr28	10052597	2	36	G:0.166667	T:0.833333	
chr28	10052665	2	40	T:0.175	G:0.825	

chr28	10054158	2	40	T:0.45	TA:0.55	
chr28	10054168	2	40	C:0.45	A:0.55	
chr28	10054326	2	40	A:0.55	T:0.45	
chr28	10054423	2	34	C:0.441176	T:0.558824	
chr28	10054475	2	36	A:0.388889	C:0.611111	
chr28	10055778	2	38	A:0.578947	G:0.421053	
chr28	10057013	2	38	G:1	A:0	
chr28	10057327	2	40	C:0.55	CTG:0.45	
chr28	10057799	2	38	T:1	G:0	
chr28	10058016	2	38	T:0.5	C:0.5	
chr28	10058393	2	36	G:1	A:0	
chr28	10058485	2	40	A:0.4	T:0.6	
chr28	10059372	3	40	C:0.525	CA:0.4	CAA:0.075
chr28	10060023	2	40	G:1	T:0	
chr28	10060123	2	40	T:0.425	C:0.575	
chr28	10060126	2	38	AT:0.894737	A:0.105263	
chr28	10060315	2	40	AG:1	A:0	
chr28	10060710	2	38	A:0.526316	G:0.473684	
chr28	10061091	2	36	G:0.5	A:0.5	
chr28	10061284	3	38	TA:0.763158	T:0.184211	TAAA:0.0526316
chr28	10061832	2	38	T:0.684211	TA:0.315789	
chr28	10061836	2	34	AAATTAATT:1	A:0	
chr28	10061840	2	38	T:0.736842	A:0.263158	
chr28	10061883	2	36	C:1	T:0	
chr28	10062552	2	38	T:0.973684	C:0.0263158	
chr28	10062726	2	38	GT:0.447368	G:0.552632	
chr28	10062863	2	40	T:0	C:1	
chr28	10063276	2	40	A:1	AAT:0	
chr28	10063583	2	40	T:0.45	C:0.55	
chr28	10064160	2	36	GT:0.805556	G:0.194444	
chr28	10064204	2	38	C:0.5	CAGAGAG:0.5	
chr28	10064491	2	40	A:0.5	G:0.5	
chr28	10064587	2	36	T:1	TA:0	
chr28	10064593	2	36	AT:0.638889	A:0.361111	
chr28	10064594	2	36	T:1	A:0	
chr28	10065475	2	40	C:0.375	A:0.625	
chr28	10065578	2	38	A:0.552632	G:0.447368	
chr28	10065761	2	40	G:1	A:0	
chr28	10065988	2	38	C:0.631579	T:0.368421	
chr28	10066201	2	38	TA:0.447368	T:0.552632	
chr28	10066680	2	36	GT:1	G:0	
chr28	10066743	2	40	A:0.475	G:0.525	
chr28	10066979	2	38	C:1	T:0	
chr28	10067114	2	36	C:0.361111	T:0.638889	
chr28	10067307	2	40	G:0.95	A:0.05	
chr28	10067941	2	40	T:1	G:0	
chr28	10067961	2	40	T:1	A:0	
chr28	10068042	2	40	A:0.425	G:0.575	
chr28	10068636	2	36	T:0.666667	C:0.333333	
chr28	10069175	2	36	G:0.666667	GA:0.333333	
chr28	10069892	2	36	T:0.0833333	C:0.916667	
chr28	10070151	2	36	C:1	T:0	
chr28	10070483	3	38	CAAAA:0.842105	C:0	CAAAAA:

0.157895
chr28 10070487 2 38 A:1 C:0
chr28 10070720 2 36 G:1 GT:0
chr28 10070728 2 36 C:1 CT:0
chr28 10070746 2 38 A:1 T:0
chr28 10070777 2 36 C:1 CAG:0
chr28 10070843 2 38 G:0.842105 A:0.157895
chr28 10070878 2 40 GGCTAAAGGCGGC:0.9 G:0.1
chr28 10070947 2 38 G:1 A:0
chr28 10071041 2 40 G:0.5 A:0.5
chr28 10071202 2 40 G:1 A:0
chr28 10071317 2 36 GA:1 G:0
chr28 10071810 2 40 A:0.975 T:0.025
chr28 10072480 2 38 G:1 A:0
chr28 10072523 2 38 AT:1 A:0
chr28 10072534 2 38 AT:1 A:0
chr28 10072535 2 38 T:1 A:0
chr28 10072625 2 38 C:1 G:0
chr28 10072630 2 38 C:1 T:0
chr28 10072761 2 40 A:0.5 C:0.5
chr28 10072909 2 38 A:0.868421 ATGGG:0.131579
chr28 10072913 3 38 GTGGA:0.631579 G:0.342105
GTGGATGGATGGA:0.0263158
chr28 10072942 3 40 T:1 TGGAAGGAA:0
TGGATGGATGGAAGGAA:0
chr28 10072989 2 40 C:1 T:0
chr28 10073256 2 40 C:1 A:0
chr28 10073257 2 40 T:1 TA:0
chr28 10073634 2 30 G:1 A:0
chr28 10073742 2 30 G:1 C:0
chr28 10074158 2 38 C:1 G:0
chr28 10074162 2 40 G:1 A:0
chr28 10074844 2 40 T:1 C:0
chr28 10075230 2 40 C:1 T:0
chr28 10075314 3 40 CTT:0.6 C:0.05 CT:0.35
chr28 10075364 2 30 G:0.866667 GT:0.133333
chr28 10075629 2 40 C:1 A:0
chr28 10075750 2 40 G:1 A:0
chr28 10076043 2 40 C:1 T:0
chr28 10076045 2 40 T:1 A:0
chr28 10076129 2 40 T:1 C:0
chr28 10076485 2 38 T:1 C:0
chr28 10076686 2 34 G:1 A:0
chr28 10076703 2 36 G:0.472222 A:0.527778
chr28 10076850 2 40 C:1 T:0
chr28 10076853 2 40 T:1 C:0
chr28 10076907 2 40 C:1 T:0
chr28 10077169 2 40 C:1 T:0
chr28 10077236 2 40 A:1 AT:0
chr28 10077237 2 40 T:1 TA:0
chr28 10077324 2 40 TG:1 T:0
chr28 10078138 2 40 C:1 T:0
chr28 10078199 2 34 G:1 A:0
chr28 10078207 3 40 TAAAAAATAAATAAATA:1 T:0

TAATAAATA:0
chr28 10078210 5 40 AAAATAAATAAATAAAT:0.425 A:0.05
AAAATAAAT:0.5 AAAATAAATAAAT:0 TAAATAAATAAATAAAT:0.025
chr28 10078261 2 40 AT:1 A:0
chr28 10078263 2 40 AAAT:1 A:0
chr28 10078526 2 38 C:1 T:0
chr28 10079795 2 40 T:1 C:0
chr28 10080015 2 40 CTG:1 C:0
chr28 10080029 2 40 A:0.5 G:0.5
chr28 10080379 2 38 TA:1 T:0
chr28 10080459 2 36 CCGA:1 C:0
chr28 10080475 2 36 C:1 T:0
chr28 10080691 2 38 C:1 T:0
chr28 10080746 2 36 G:1 A:0
chr28 10080917 3 40 GCA:0.75 G:0.05 GCACA:0.2
chr28 10081526 2 40 G:0.475 A:0.525
chr28 10081739 2 38 C:0.552632 CTTCTGAA:0.447368
chr28 10081788 2 38 A:0.552632 G:0.447368
chr28 10082157 2 40 CAGAGAGAGAG:0.6 C:0.4
chr28 10082780 3 40 CAAATAAATAAATAAATAAATAAAT:0.125
C:0.375 CAAATAAAT:0.5
chr28 10082898 2 24 A:0.416667 AGGAG:0.583333
chr28 10084653 2 40 C:1 A:0
chr28 10084683 2 40 C:0.525 G:0.475
chr28 10084892 2 38 G:0.552632 T:0.447368
chr28 10085050 2 40 T:0.6 C:0.4
chr28 10085104 2 40 G:0.625 A:0.375
chr28 10085310 2 30 CT:0.866667 C:0.133333
chr28 10085343 2 32 C:0.25 CT:0.75
chr28 10086152 2 36 T:0.527778 C:0.472222
chr28 10086243 2 40 C:0.625 T:0.375
chr28 10086541 2 38 C:1 A:0
chr28 10086599 2 40 C:0.725 CCT:0.275
chr28 10086657 2 40 T:0.525 TA:0.475
chr28 10086958 2 40 C:0.55 G:0.45
chr28 10087380 2 30 CTT:0.9 C:0.1
chr28 10087430 2 36 C:0.666667 CAG:0.333333
chr28 10089009 2 34 GCT:0.470588 G:0.529412
chr28 10089396 3 38 CCTCTCTCT:0.552632 C:
0.157895 CCT:0.289474
chr28 10089464 2 38 A:0.526316 G:0.473684
chr28 10089623 2 28 TA:0.857143 T:0.142857
chr28 10089847 3 38 GTCTC:0.526316 G:0.0789474
GTC:0.394737
chr28 10089900 2 36 A:0.527778 AAAAAG:0.472222
chr28 10089994 2 36 T:0.527778 C:0.472222
chr28 10090186 2 34 G:0.558824 T:0.441176
chr28 10090333 2 34 G:0.558824 A:0.441176
chr28 10090414 2 32 CCT:0.71875 C:0.28125
chr28 10090458 3 36 A:0.805556 AAAAT:0.0277778
AAAATAAAT:0.166667
chr28 10090502 5 38 T:0.631579 TAAATA:0.157895
TAAATAAATA:0.184211 TAAATAAATAAATA:0.0263158
TAAATAAATAAATAAAATA:0

chr28	10091191 2	34	AAAAAAG:0.970588 A:0.0294118
chr28	10091192 2	34	AAAAAG:0.676471	A:0.323529
chr28	10091349 2	28	C:0.357143	A:0.642857
chr28	10091547 2	34	T:0.558824	TCC:0.441176
chr28	10091937 2	36	TTC:0.5	T:0.5
chr28	10092104 2	40	C:0.525	CA:0.475
chr28	10092126 2	40	T:0.625	TAAAATA:0.375
chr28	10092158 2	32	T:1	TA:0
chr28	10092160 2	40	A:0.975	AAAATAAAAT:0.025
chr28	10092165 2	32	A:1	AAAT:0
chr28	10092348 2	36	G:1	A:0
chr28	10092577 2	40	A:0.55	ATAAAT:0.45
chr28	10092836 2	40	T:0.6	A:0.4
chr28	10092850 2	40	C:0.6	G:0.4
chr28	10093038 2	38	GT:0.526316	G:0.473684
chr28	10093112 2	38	A:0.578947	AT:0.421053
chr28	10093364 2	38	A:0.526316	C:0.473684
chr28	10093366 2	38	C:0.526316	T:0.473684
chr28	10093569 2	38	G:0.631579	C:0.368421
chr28	10093578 2	38	G:0.631579	A:0.368421
chr28	10093621 2	40	G:0.475	A:0.525
chr28	10093670 2	40	T:0.525	C:0.475
chr28	10093679 2	40	G:1	A:0
chr28	10093886 2	40	C:1	CGCATT:0
chr28	10093997 2	40	G:0.55	A:0.45
chr28	10094136 2	40	G:0.475	A:0.525
chr28	10094540 2	40	C:1	G:0
chr28	10094866 2	38	G:1	GA:0
chr28	10095524 2	8	C:0.25	G:0.75
chr28	10095808 2	28	T:1	A:0
chr28	10095961 2	38	C:1	A:0
chr28	10096511 5	40	AAC:0.725	A:0	AACAC:
0.1	AACACAC:0.075	AACACACAC:0.1
chr28	10096535 2	36	C:1	CAT:0
chr28	10096621 2	40	A:1	G:0
chr28	10097262 2	36	CA:1	C:0
chr28	10098620 2	40	G:1	A:0
chr28	10098707 2	36	T:1	C:0
chr28	10099037 2	40	G:1	C:0
chr28	10099202 2	40	C:1	T:0
chr28	10099239 2	40	T:1	TC:0
chr28	10099738 2	36	G:1	T:0
chr28	10100126 2	32	C:1	T:0
chr28	10100782 2	32	T:1	C:0
chr28	10100882 2	38	C:0.5	T:0.5
chr28	10101917 2	38	T:1	TA:0
chr28	10102255 2	38	C:1	T:0
chr28	10102743 2	40	T:1	A:0
chr28	10103171 2	38	G:1	A:0
chr28	10103202 2	38	TA:0.973684	T:0.0263158
chr28	10103505 2	38	T:1	C:0
chr28	10103578 2	38	T:1	A:0
chr28	10103655 2	32	G:1	A:0
chr28	10103847 2	40	T:1	C:0

chr28	10104001	2	38	C:1	A:0	
chr28	10104218	2	40	T:1	C:0	
chr28	10105506	2	32	A:0	T:1	
chr28	10105512	2	32	G:0	T:1	
chr28	10105531	2	38	C:1	T:0	
chr28	10105576	2	40	A:1	AT:0	
chr28	10105579	2	40	A:1	T:0	
chr28	10106044	2	40	T:1	A:0	
chr28	10106203	2	40	ACACCCTGGGC:0.95	A:0.05	
chr28	10106407	2	38	T:1	C:0	
chr28	10106817	2	38	T:1	C:0	
chr28	10107030	2	40	T:1	C:0	
chr28	10107056	2	40	G:1	GT:0	
chr28	10107095	2	38	A:1	G:0	
chr28	10107288	2	38	C:1	A:0	
chr28	10107430	2	38	G:1	A:0	
chr28	10107531	2	38	T:1	A:0	
chr28	10107690	2	40	G:1	A:0	
chr28	10107722	2	40	G:1	A:0	
chr28	10108281	2	36	A:1	C:0	
chr28	10108956	2	40	A:0.65	T:0.35	
chr28	10109651	2	38	T:1	A:0	
chr28	10109700	2	40	C:0.75	G:0.25	
chr28	10110817	2	40	C:0.95	CT:0.05	
chr28	10111283	2	38	G:1	T:0	
chr28	10111747	2	38	C:1	CT:0	
chr28	10112779	2	40	T:1	A:0	
chr28	10112970	2	36	T:1	C:0	
chr28	10113257	2	40	A:0.525	G:0.475	
chr28	10113277	2	38	G:1	A:0	
chr28	10113438	2	40	T:1	G:0	
chr28	10113915	2	40	A:1	G:0	
chr28	10113965	2	40	T:1	A:0	
chr28	10115385	3	40	TAAAA:0.85	T:0.025	TAAA: 0.125
chr28	10115597	2	34	G:1	A:0	
chr28	10115897	2	38	C:0.789474	CGTT:0.210526	
chr28	10116159	2	36	CAG:1	C:0	
chr28	10116680	2	38	G:0.947368	GA:0.0526316	
chr28	10117651	2	40	C:1	T:0	
chr28	10117766	2	36	C:0.944444	CT:0.0555556	
chr28	10119515	2	38	T:1	C:0	
chr28	10119785	2	40	G:1	A:0	
chr28	10120019	2	38	C:1	T:0	
chr28	10120177	2	38	A:1	G:0	
chr28	10120315	2	40	G:1	A:0	
chr28	10120745	2	38	G:1	A:0	
chr28	10121284	2	38	T:1	TTTAC:0	
chr28	10121586	2	40	C:1	T:0	
chr28	10122715	2	32	C:0.53125	CT:0.46875	
chr28	10122759	2	36	G:1	C:0	
chr28	10122974	2	40	ATCATGATCCCAGGG:0.925	A:0.075	
chr28	10123077	2	38	T:1	C:0	
chr28	10124051	2	40	A:1	C:0	

chr28 10124215 2 38 C:1 T:0
chr28 10124244 2 36 A:0.555556 AT:0.444444
chr28 10124862 2 40 ATT:0.5 A:0.5
chr28 10124867 3 40 T:0.5 TAAA:0.5 TA:0
chr28 10124999 4 40 G:0.475 GTCTC:0.2 GTCTCTC:
0.325 GTCTCTCTCTCTCTC:0
chr28 10125084 2 38 A:1 T:0
chr28 10125493 2 38 C:1 T:0
chr28 10125613 2 36 G:0.555556 A:0.444444
chr28 10125652 2 36 A:0.944444 AT:0.0555556
chr28 10125921 2 38 C:1 G:0
chr28 10125998 2 40 C:1 T:0
chr28 10126072 2 38 AC:1 A:0
chr28 10126127 2 36 T:1 A:0
chr28 10126138 2 36 T:0.444444 C:0.555556
chr28 10126617 2 38 T:0.605263 C:0.394737
chr28 10126622 2 38 T:1 C:0
chr28 10126737 2 38 A:0.605263 G:0.394737
chr28 10127431 2 38 T:1 TA:0
chr28 10127853 2 38 G:1 A:0
chr28 10127978 2 40 C:0.525 T:0.475
chr28 10128193 3 38 T:0.578947 TA:0.421053
TAA:0
chr28 10128339 2 40 A:1 G:0
chr28 10128587 2 36 A:0.555556 G:0.444444
chr28 10128663 2 36 G:1 A:0
chr28 10128703 2 34 A:0.676471 G:0.323529
chr28 10128796 2 36 A:1 G:0
chr28 10128797 2 36 A:0.972222 G:0.0277778
chr28 10129048 2 40 C:0.625 T:0.375
chr28 10129091 2 40 T:0.575 A:0.425
chr28 10129195 2 40 ATCT:1 A:0
chr28 10129258 2 40 AG:1 A:0
chr28 10129397 2 38 T:0.605263 G:0.394737
chr28 10129433 2 40 A:0.525 G:0.475
chr28 10129449 2 40 G:1 A:0
chr28 10129496 2 40 T:0 G:1
chr28 10129566 2 38 T:0.552632 A:0.447368
chr28 10129780 4 40 A:0.475 AGTTGTTGTT:0.05
AGTTGTTGTTGTT:0.375 AGTTGTTGTTGTTGTT:0.1
chr28 10130205 2 36 C:1 T:0
chr28 10130209 2 34 T:0.617647 C:0.382353
chr28 10130249 2 34 C:0.735294 CCT:0.264706
chr28 10130303 2 34 TA:0.970588 T:0.0294118
chr28 10130475 2 38 G:1 A:0
chr28 10130573 2 32 G:0.625 A:0.375
chr28 10130657 2 30 C:0.933333 CCT:0.0666667
chr28 10130720 2 32 TAA:0.53125 T:0.46875
chr28 10130743 2 36 C:1 T:0
chr28 10130774 2 40 A:0 G:1
chr28 10130874 2 34 G:0.117647 C:0.882353
chr28 10131021 2 38 T:0 TA:1
chr28 10131487 2 34 G:1 T:0
chr28 10131492 2 34 T:0.735294 G:0.264706

chr28	10131505	2	34	T:0.735294	C:0.264706	
chr28	10131651	2	30	G:0.766667	GC:0.233333	
chr28	10131678	2	36	C:1	T:0	
chr28	10131857	2	40	TG:0.625	T:0.375	
chr28	10132020	2	38	G:0.526316	GT:0.473684	
chr28	10132022	3	38	TC:1	T:0	TCC:0
chr28	10132439	2	40	T:1	C:0	
chr28	10132652	3	38	CT:0.552632	C:0	CTT:0.447368
chr28	10132900	2	34	C:1	T:0	
chr28	10133410	2	36	T:1	TC:0	
chr28	10133788	2	40	G:0.8	GAA:0.2	
chr28	10133978	2	32	T:1	A:0	
chr28	10134071	2	34	C:0.941176	T:0.0588235	
chr28	10134365	2	32	A:1	G:0	
chr28	10134481	2	26	G:1	A:0	
chr28	10134640	2	34	AT:1	A:0	
chr28	10134643	2	34	AAT:1	A:0	
chr28	10134645	2	32	TA:0.25	T:0.75	
chr28	10134653	2	34	A:1	T:0	
chr28	10134828	2	30	C:1	T:0	
chr28	10136462	2	40	C:1	T:0	
chr28	10136854	2	36	T:1	C:0	
chr28	10137070	2	40	GAAAAAGAAAACCAAAAAAAAAAAAAAAAAAA:0.65	G:0.35	
chr28	10137137	2	34	G:1	A:0	
chr28	10137644	2	36	G:1	A:0	
chr28	10137692	2	36	C:0.5	T:0.5	
chr28	10137697	2	38	T:0.552632	A:0.447368	
chr28	10137774	2	36	C:0.638889	CCTCT:0.361111	
chr28	10137795	2	34	C:1	CTG:0	
chr28	10138143	2	38	A:0.631579	G:0.368421	
chr28	10138144	2	38	T:0.631579	A:0.368421	
chr28	10138331	2	38	T:1	C:0	
chr28	10138822	2	38	C:0.526316	G:0.473684	
chr28	10138986	2	40	G:0.475	A:0.525	
chr28	10139094	2	38	T:1	A:0	
chr28	10139240	2	40	T:1	C:0	
chr28	10139597	2	40	A:1	C:0	
chr28	10139797	2	38	G:0.947368	A:0.0526316	
chr28	10140077	2	38	C:0	G:1	
chr28	10140097	2	38	AG:0	A:1	
chr28	10140385	3	38	TA:0.552632	T:0.447368	TAA:0
chr28	10140474	2	36	G:1	A:0	
chr28	10140533	2	32	ATC:0.625	A:0.375	
chr28	10140599	2	38	T:0.157895	A:0.842105	
chr28	10140961	2	38	C:0.5	T:0.5	
chr28	10141158	2	40	G:1	T:0	
chr28	10141522	2	38	G:1	A:0	
chr28	10141979	2	38	G:0.578947	A:0.421053	
chr28	10142270	2	38	G:1	A:0	
chr28	10142335	2	38	T:1	C:0	
chr28	10142667	2	38	G:0.815789	A:0.184211	

chr28	10142776	2	32	CCT:0.6875	C:0.3125		
chr28	10142881	2	40	T:1	G:0		
chr28	10143149	2	40	T:0.6	A:0.4		
chr28	10143281	2	38	G:1	A:0		
chr28	10143371	2	38	C:1	T:0		
chr28	10143432	2	40	A:1	T:0		
chr28	10143856	2	38	A:1	G:0		
chr28	10144051	2	40	G:1	A:0		
chr28	10144301	2	38	T:0.473684	C:0.526316		
chr28	10144486	2	38	A:1	G:0		
chr28	10144544	2	38	CCT:0.473684	C:0.526316		
chr28	10144548	2	40	T:0.95	TCTCTCTCTCTCTCTCTCTGCTTCTCCCTCTGCCTGTGTCTCTGC:0.05		
chr28	10144605	2	38	TA:0.973684	T:0.0263158		
chr28	10144851	2	40	G:1	A:0		
chr28	10145459	2	38	C:0	CTA:1		
chr28	10145579	2	40	T:1	G:0		
chr28	10145854	2	40	T:0	C:1		
chr28	10145985	2	38	G:1	T:0		
chr28	10145992	2	38	T:1	C:0		
chr28	10146039	2	40	GGTGT:0.975	G:0.025		
chr28	10146058	2	40	G:0.55	GTA:0.45		
chr28	10146103	2	38	T:1	C:0		
chr28	10146246	2	40	A:0.7	G:0.3		
chr28	10146279	2	40	T:0.95	C:0.05		
chr28	10147010	2	40	G:0.425	C:0.575		
chr28	10147135	2	38	G:0.0526316	C:0.947368		
chr28	10147191	2	38	T:0.657895	G:0.342105		
chr28	10147472	2	38	A:0.947368	G:0.0526316		
chr28	10147807	2	38	T:0.0526316	TC:0.947368		
chr28	10148232	2	36	GA:0.527778	G:0.472222		
chr28	10148242	2	36	AGAG:0.527778	A:0.472222		
chr28	10148245	3	40	GAAGA:1	G:0	GAAGAAAGA:0	
chr28	10148288	2	38	GAA:0.894737	G:0.105263		
chr28	10148294	2	28	AAG:0.321429	A:0.678571		
chr28	10148300	2	32	GAA:0.3125	G:0.6875		
chr28	10148412	2	40	C:0.95	T:0.05		
chr28	10149146	2	38	T:0.0263158	G:0.973684		
chr28	10149410	2	40	A:0.575	G:0.425		
chr28	10149449	2	38	G:0.763158	T:0.236842		
chr28	10149841	2	38	C:0.552632	CT:0.447368		
chr28	10149893	2	40	A:0.55	T:0.45		
chr28	10149980	2	38	C:0.657895	T:0.342105		
chr28	10149999	2	38	G:1	C:0		
chr28	10150042	2	36	T:1	C:0		
chr28	10150292	2	40	A:0.45	T:0.55		
chr28	10150297	2	40	G:1	T:0		
chr28	10150322	2	40	C:1	T:0		
chr28	10150341	2	38	AT:0.631579	A:0.368421		
chr28	10150377	4	40	CAG:0.65	C:0.35	CAGAGAGAG:0	CAGAGAGAGAG:0
chr28	10150480	2	40	A:1	ACATCCTGAG:0		
chr28	10150723	2	38	CCTTAT:0.631579	C:0.368421		
chr28	10150822	2	40	C:1	A:0		

chr28 10150902 2 40 A:0.05 T:0.95
chr28 10150992 2 40 C:0.05 A:0.95
chr28 10151037 2 38 G:0.0526316 T:0.947368
chr28 10151226 2 36 G:0.388889 A:0.611111
chr28 10151411 2 34 T:0.588235 C:0.411765
chr28 10151543 2 40 AT:0.65 A:0.35
chr28 10152372 2 36 C:0.0555556 T:0.944444
chr28 10152413 4 38 TAA:0.263158 T:0.315789
TA:0.210526 TAAA:0.210526
chr28 10152907 2 38 GA:0.368421 G:0.631579
chr28 10153343 2 38 G:0.473684 A:0.526316
chr28 10153365 2 38 C:0.789474 T:0.210526
chr28 10153418 2 38 C:0.447368 A:0.552632
chr28 10153642 2 36 C:1 T:0
chr28 10153904 2 38 G:0.0526316 A:0.947368
chr28 10153945 2 38 GTC:0.947368 G:0.0526316
chr28 10153996 2 36 T:0.722222 C:0.277778
chr28 10154047 2 34 A:0.676471 G:0.323529
chr28 10154104 2 36 A:0.861111 T:0.138889
chr28 10154196 2 26 C:0.461538 CCT:0.538462
chr28 10154306 2 38 C:0.578947 A:0.421053
chr28 10154338 2 40 C:0.475
CCAAAAAAAAAAACAAAAAAAACAAAAAAAATAAATTAAAAAAAAAAAAAAAAAAAAAAAA:0.525
chr28 10154339 2 40 G:0.475 GAACCTGCCTTCC:0.525
chr28 10154461 3 38 T:0.0526316 TA:0.526316
TAA:0.421053
chr28 10154689 2 40 C:0.5 G:0.5
chr28 10154839 2 38 CCTCT:0.157895 C:0.842105
chr28 10154898 3 40 CA:0.525 C:0.4 CAAA:0.075
chr28 10155088 2 34 TAAAAGTTTTTAAAAA:0.764706 T:
0.235294
chr28 10155093 2 36 GTTTTTAAAAAAAAAGT:0.972222
G:0.0277778
chr28 10155179 2 38 G:0.763158 GA:0.236842
chr28 10155187 2 38 T:0.947368 A:0.0526316
chr28 10155196 2 38 T:0.947368 A:0.0526316
chr28 10155249 2 34 T:0.911765 C:0.0882353
chr28 10155371 2 36 G:0.611111 C:0.388889
chr28 10155420 2 34 A:0.617647 T:0.382353
chr28 10155466 2 34 ATC:0.941176 A:0.0588235
chr28 10155468 2 34 CTCT:0.676471 C:0.323529
chr28 10155470 2 34 C:0.382353 T:0.617647
chr28 10155496 2 34 T:0.676471 TGATA:0.323529
chr28 10155500 2 34 ACT:0.676471 A:0.323529
chr28 10155509 2 34 GAC:0.676471 G:0.323529
chr28 10155611 2 40 GC:0 G:1
chr28 10155632 2 30 T:0.0666667 C:0.933333
chr28 10155666 2 36 T:0.722222 TA:0.277778
chr28 10155667 3 36 T:0 A:0.277778 TA:
0.722222
chr28 10155678 2 36 T:0 TA:1
chr28 10155975 2 36 C:0.611111 CTAA:0.388889
chr28 10156113 2 38 C:0.0526316 A:0.947368
chr28 10156169 2 40 AAATT:0.975 A:0.025

chr28 10156261 2 38 T:0.421053 C:0.578947
chr28 10156606 2 38 G:0.710526 A:0.289474
chr28 10156658 2 26 C:0.0384615 CT:0.961538
chr28 10156835 2 30 G:0.1 A:0.9
chr28 10156897 2 32 T:0 C:1
chr28 10157052 2 28 T:1 TA:0
chr28 10157058 4 28 T:1 TTTTA:0 TTA:0
TTTTATTTATTTA:0
chr28 10157251 2 30 C:0.833333
CTATATGAGTAAAACTTTACAATATTTCTGAAAGATCCAAAAATAGACTGGATCCATCGATAA:
0.166667
chr28 10157831 3 22 TA:0.136364 T:0.863636
TAA:0
chr28 10158165 2 38 GTGTC:0.947368 G:0.0526316
chr28 10158167 3 40 GTCTCTCTC:0.55 G:0.275 GTCTCTC:
0.175
chr28 10158232 3 38 TAAAAAA:0.526316 TA:0.105263
T:0.368421
chr28 10158238 2 36 A:0.611111 ATT:0.388889
chr28 10158290 2 36 A:0.611111 AG:0.388889
chr28 10158392 2 36 T:0.5 C:0.5
chr28 10158447 2 36 CT:0.611111 C:0.388889
chr28 10158486 2 34 T:0.970588 TAC:0.0294118
chr28 10158490 3 40 C:0.8 CAGAGAGAGAGAG:0.15
CAGAGAGAGAGAGAG:0.05
chr28 10158492 2 36 G:1 C:0
chr28 10158537 2 38 G:0.447368 T:0.552632
chr28 10158667 2 36 A:0.611111 G:0.388889
chr28 10159009 2 38 G:0.447368 A:0.552632
chr28 10159257 2 40 G:1 GA:0
chr28 10159626 2 40 A:0.95 T:0.05
chr28 10159654 2 38 T:0.578947 A:0.421053
chr28 10159695 2 40 T:0.95 C:0.05
chr28 10159868 2 32 C:0.84375 CT:0.15625
chr28 10159995 2 28 C:1 T:0
chr28 10160300 2 40 C:0.525 T:0.475
chr28 10160324 2 40 C:0.725 T:0.275
chr28 10160325 2 40 G:0.525 T:0.475
chr28 10160674 2 36 C:0.0555556 A:0.944444
chr28 10161160 2 40 A:1 G:0
chr28 10161412 2 40 TAATTTATA:0.5 T:0.5
chr28 10161423 2 40 GA:1 G:0
chr28 10161772 2 40 TTC:0.65 T:0.35
chr28 10161810 2 40 CTGTGTGTGTGTG:0.35 C:0.65
chr28 10161852 2 38 T:0.842105 A:0.157895
chr28 10161975 2 40 C:0.575 A:0.425
chr28 10162099 2 26 TCA:0.461538 T:0.538462
chr28 10162212 2 38 CACTT:0.552632 C:0.447368
chr28 10162302 4 38 C:0.0526316 CT:0.421053
CTT:0.473684 CTTT:0.0526316
chr28 10162453 2 34 C:0.5 T:0.5
chr28 10162530 2 38 A:0.605263 G:0.394737
chr28 10162553 2 40 C:1 A:0
chr28 10162727 2 40 G:1 A:0

chr28	10162764	2	40	A:0.5	G:0.5		
chr28	10162876	3	40	T:0.4	TAAA:0.225	TAAAA:0.375	
chr28	10162901	2	40	T:0.575	A:0.425		
chr28	10163470	2	26	G:0.961538	T:0.0384615		
chr28	10164070	2	38	GTCTC:0.894737	G:0.105263		
chr28	10164099	2	36	TCTG:0.972222	T:0.0277778		
chr28	10164101	4	40	T:0.75	TC:0.225	TCTCTC:0.025	TCTCTCTCTCTCTC:0
chr28	10164102	2	36	G:0.722222	T:0.277778		
chr28	10164474	2	40	G:0.4	A:0.6		
chr28	10164559	2	40	A:1	AGCGCC:0		
chr28	10164587	2	38	C:0.710526	T:0.289474		
chr28	10164611	2	38	C:0.789474	T:0.210526		
chr28	10164666	2	38	C:0.421053	G:0.578947		
chr28	10164669	2	38	TCTTC:0.684211	T:0.315789		
chr28	10164671	3	40	TTCGTTC:0.35	T:0.55	TGTTC:0.1	
chr28	10164675	2	38	T:0.578947	TC:0.421053		
chr28	10164677	2	38	C:0.578947	CT:0.421053		
chr28	10164702	2	34	A:0	AG:1		
chr28	10164787	2	38	C:0.947368	G:0.0526316		
chr28	10164880	2	40	G:0.3	A:0.7		
chr28	10165039	2	40	TCATG:0.4	T:0.6		
chr28	10165148	3	40	T:0.15	TTCTCTCTCTCTCTCTC:0.3	TTCTCTCTCTCTCTCTCTCTC:0.55	
chr28	10165184	4	36	TAAA:0.194444	T:0.555556	TA:0.222222	TAA:0.0277778
chr28	10165347	2	24	C:0.25	CA:0.75		
chr28	10165388	2	36	C:0.694444	T:0.305556		
chr28	10165697	2	38	G:0.921053	A:0.0789474		
chr28	10165799	2	38	C:0.631579	A:0.368421		
chr28	10165856	2	40	A:0.925	G:0.075		
chr28	10166000	2	38	C:0.921053	T:0.0789474		
chr28	10166114	2	32	T:0.6875	TG:0.3125		
chr28	10166115	3	34	GTTT:0.764706	G:0.0294118	GTT:0.205882	
chr28	10166116	2	38	T:1	TGG:0		
chr28	10166119	2	32	T:1	G:0		
chr28	10166202	2	36	G:0.916667	A:0.0833333		
chr28	10166217	2	36	T:0.916667	C:0.0833333		
chr28	10166314	3	40	TGA:0.85	T:0.125	TGAGAGA:0.025	
chr28	10166335	2	40	GA:0.925	G:0.075		
chr28	10166338	2	40	AGAGGC:0.925	A:0.075		
chr28	10166523	2	40	G:1	GC:0		
chr28	10166524	2	40	T:0.025	C:0.975		
chr28	10166526	2	40	T:1	C:0		
chr28	10166527	2	40	CCA:0.025	C:0.975		
chr28	10166529	2	40	A:1	G:0		
chr28	10166595	2	38	GAGAGAC:1	G:0		
chr28	10166651	2	38	A:0.947368	G:0.0526316		
chr28	10166815	2	40	C:0.625	A:0.375		
chr28	10167035	2	38	T:0.605263	TA:0.394737		
chr28	10167173	2	40	AT:0.65	A:0.35		

chr28	10167306	2	40	T:1	C:0			
chr28	10167353	2	38	C:0.736842	T:0.263158			
chr28	10167432	2	40	C:0.625	T:0.375			
chr28	10167461	2	40	TA:0.7	T:0.3			
chr28	10167532	2	40	G:0.95	A:0.05			
chr28	10167797	2	36	C:0.944444	T:0.0555556			
chr28	10167871	2	36	G:0.0555556	A:0.944444			
chr28	10167923	2	38	A:0.947368	C:0.0526316			
chr28	10167993	2	40	A:0.95	G:0.05			
chr28	10168029	2	38	G:0.0526316	GT:0.947368			
chr28	10168285	2	40	T:0.05	C:0.95			
chr28	10168409	2	34	TTG:0.794118	T:0.205882			
chr28	10168410	2	34	TG:0.5	T:0.5			
chr28	10168483	2	40	A:0.05	G:0.95			
chr28	10168500	2	40	TA:0.05	T:0.95			
chr28	10168720	2	38	A:0.0526316	G:0.947368			
chr28	10169095	2	36	A:0.694444	ATT:0.305556			
chr28	10169138	2	40	C:0.925	A:0.075			
chr28	10169343	2	32	AT:0.65625	A:0.34375			
chr28	10169635	2	40	T:1	A:0			
chr28	10169645	2	40	T:0.65	C:0.35			
chr28	10169770	2	40	A:0.525	G:0.475			
chr28	10169804	3	36	CTT:0.527778	C:0.0833333	CT:0.388889		
chr28	10170040	2	34	T:0.941176	C:0.0588235			
chr28	10170089	2	40	G:1	GT:0			
chr28	10170108	2	38	C:0.947368	T:0.0526316			
chr28	10170182	5	40	T:0.65	TTTTA:0.125	TTATTTA:0.025	TTATTTATTTA:0.05	TTATTTATTTATTTA:0.15
chr28	10170238	2	38	A:0.605263	G:0.394737			
chr28	10170244	2	38	G:0.605263	T:0.394737			
chr28	10170287	2	40	G:0.925	A:0.075			
chr28	10170290	2	40	G:0.925	A:0.075			
chr28	10170527	2	38	T:0.947368	C:0.0526316			
chr28	10170663	2	40	C:0.425	T:0.575			
chr28	10170835	2	38	T:0.0526316	A:0.947368			
chr28	10170916	2	40	TA:0.375	T:0.625			
chr28	10171137	2	36	G:0.0833333	A:0.916667			
chr28	10171237	2	40	C:0.7	T:0.3			
chr28	10171367	2	40	G:0.325	GA:0.675			
chr28	10171752	2	40	T:0.05	C:0.95			
chr28	10171820	2	36	T:0.138889	TAAA:0.861111			
chr28	10171859	2	38	G:0.736842	A:0.263158			
chr28	10171865	2	38	G:0.736842	A:0.263158			
chr28	10171943	2	40	G:0.725	C:0.275			
chr28	10172177	2	38	C:0.631579	A:0.368421			
chr28	10172284	2	38	CT:1	C:0			
chr28	10172425	2	36	A:0.666667	C:0.333333			
chr28	10172799	2	38	C:0.710526	T:0.289474			
chr28	10173033	2	40	A:0.7	G:0.3			
chr28	10173460	2	36	G:0.638889	C:0.361111			
chr28	10174465	2	38	C:0.0526316	T:0.947368			
chr28	10174656	2	40	C:0.675	T:0.325			
chr28	10175018	2	34	TA:0.676471	T:0.323529			

chr28	10175020	2	36	A:1	T:0	
chr28	10175416	2	36	G:0	C:1	
chr28	10175462	2	34	C:0.764706	T:0.235294	
chr28	10175676	2	40	GCAA:0.75	G:0.25	
chr28	10175995	2	40	A:0.625	G:0.375	
chr28	10176036	2	40	G:0.625	A:0.375	
chr28	10176088	2	32	A:0.78125	T:0.21875	
chr28	10176155	2	40	T:0.7	TAAAATATTTTTGTTGAAAAAAAAAAGAAAAAA:0.3	
chr28	10176451	2	38	C:0.657895	T:0.342105	
chr28	10176452	2	38	G:0.947368	A:0.0526316	
chr28	10176636	2	36	G:0.75	A:0.25	
chr28	10177037	2	40	C:0	CA:1	
chr28	10177053	2	40	C:0	CA:1	
chr28	10177172	2	36	G:0.638889	A:0.361111	
chr28	10177588	2	40	T:0.675	A:0.325	
chr28	10177752	2	40	C:0.675	A:0.325	
chr28	10177792	2	38	T:0.631579	C:0.368421	
chr28	10177951	2	40	G:0.625	A:0.375	
chr28	10177995	2	40	C:0.675	T:0.325	
chr28	10178073	2	38	G:0.421053	A:0.578947	
chr28	10178127	2	36	T:0.75	C:0.25	
chr28	10178215	2	34	T:0.764706	TA:0.235294	
chr28	10178216	3	34	T:0.735294	TA:0.0882353	TATA:0.176471
chr28	10178259	2	38	G:0.736842	A:0.263158	
chr28	10178587	2	40	CTGGGGAGATGCTT:0.7	C:0.3	
chr28	10178669	2	36	G:0.75	A:0.25	
chr28	10178780	2	38	CA:0.763158	C:0.236842	
chr28	10178917	2	40	C:0.725	T:0.275	
chr28	10178994	2	40	T:0.75	G:0.25	
chr28	10179163	2	36	C:1	T:0	
chr28	10179338	2	40	T:0.65	C:0.35	
chr28	10179482	2	40	A:0.925	T:0.075	
chr28	10179722	2	32	G:0.6875	GTT:0.3125	
chr28	10179732	2	40	T:0.95	TG:0.05	
chr28	10179736	2	40	T:0.95	TGTTTTTTG:0.05	
chr28	10179777	2	36	G:0.972222	A:0.0277778	
chr28	10179926	2	38	A:0.894737	T:0.105263	
chr28	10180117	2	38	C:0.973684	T:0.0263158	
chr28	10180121	2	38	T:0.973684	C:0.0263158	
chr28	10180448	2	38	A:0.947368	AGAGAGAGAGAGC:0.0526316	
chr28	10180452	2	36	C:0.388889	T:0.611111	
chr28	10180559	2	40	A:0.4	G:0.6	
chr28	10180810	2	36	T:0.944444	C:0.0555556	
chr28	10180918	2	32	TA:0.875	T:0.125	
chr28	10180948	2	38	T:0.973684	A:0.0263158	
chr28	10181070	2	40	A:0.9	AT:0.1	
chr28	10181208	2	40	G:0.375	A:0.625	
chr28	10181272	2	38	A:1	C:0	
chr28	10181355	2	40	C:0.975	T:0.025	
chr28	10181433	2	38	T:1	C:0	
chr28	10181455	2	38	A:1	ATGCTGC:0	

chr28	10181479 2	38	CTTCTGTTT:0.973684	C:
0.0263158
chr28	10181484 2	40	G:0.8	GT:0.2
chr28	10181492 2	36	TTG:1	T:0
chr28	10181494 2	36	G:0.527778	T:0.472222
chr28	10181512 4	36	GTTT:0.5 G:0.0277778	GTTTT:0
TTTT:0.472222
chr28	10182245 2	36	T:0.972222	C:0.0277778
chr28	10182413 2	40	G:0.9	T:0.1
chr28	10182486 2	36	T:0.944444	C:0.0555556
chr28	10182646 2	38	G:0.868421	A:0.131579
chr28	10182848 4	36	CT:0.0833333	C:0.138889
CTT:0.444444	CTTT:0.333333
chr28	10183221 2	40	G:0.85	A:0.15
chr28	10183345 2	36	TAGTC:0.916667	T:0.0833333
chr28	10183420 2	36	T:0.861111	C:0.138889
chr28	10183703 2	40	G:0.9	GTTTTTTTTT:0.1
chr28	10184034 2	32	T:0.78125	TAA:0.21875
chr28	10184066 2	38	C:0.815789	CT:0.184211
chr28	10184150 2	38	A:0.789474	T:0.210526
chr28	10184152 2	36	ATTTATTT:0.5	A:0.5
chr28	10184489 2	38	A:0.289474	G:0.710526
chr28	10184650 2	40
CCAAACTTTCTTTTTCTTTTTTTTTTTTTTTTTTTTTTTTTTTTTTTTTT:0	C:1
chr28	10184933 2	36	T:0.972222	C:0.0277778
chr28	10185046 2	38	TA:0.921053	T:0.0789474
chr28	10185574 2	40	C:0.95	T:0.05
chr28	10185602 2	40	T:0.925	A:0.075
chr28	10185825 2	40	G:0.975	A:0.025
chr28	10186039 2	40	A:0.9	AT:0.1
chr28	10186510 2	38	G:0.552632	A:0.447368
chr28	10187094 2	36	TA:0.333333	T:0.666667
chr28	10187445 2	40	AAAC:0.9 A:0.1
chr28	10187515 2	40	G:0.65	A:0.35
chr28	10187527 2	40	G:0.375	A:0.625
chr28	10187572 2	40	C:0.9	A:0.1
chr28	10187573 2	40	G:0.35	A:0.65
chr28	10187947 2	40	G:0.85
GAAAACATGTCCACACAAAAATGTGTTTAC:0.15
chr28	10188244 2	38	TC:0.947368	T:0.0526316
chr28	10188277 2	30	C:0.933333	G:0.0666667
chr28	10188361 2	38	T:1	TA:0
chr28	10188362 2	38	T:0.526316	A:0.473684
chr28	10188490 2	38	C:0.657895	T:0.342105
chr28	10189517 2	36	TC:0.416667	T:0.583333
chr28	10189654 2	38	G:0.342105	C:0.657895
chr28	10189689 2	38	C:0.342105	T:0.657895
chr28	10189692 2	38	T:0.710526	C:0.289474
chr28	10189913 2	40	A:0.5	G:0.5
chr28	10190601 2	40	G:0.9	A:0.1
chr28	10190834 2	40	T:0.1	A:0.9
chr28	10191435 2	40	C:0.55	G:0.45
chr28	10191496 2	38	A:0.526316	G:0.473684
chr28	10191911 2	36	TA:0.555556	T:0.444444

chr28 10191933 2 36 G:0.555556 A:0.444444
chr28 10192231 2 36 C:1 T:0
chr28 10192255 2 36 C:0.638889 T:0.361111
chr28 10192561 2 32 A:0.9375 T:0.0625
chr28 10192640 2 36 T:0.388889 A:0.611111
chr28 10192664 2 34 C:0.235294 G:0.764706
chr28 10192898 2 20 G:0.35 C:0.65
chr28 10194221 2 32 T:0.46875 C:0.53125
chr28 10194540 3 36 G:1 GTGC:0 GTGCTGC:0
chr28 10194740 2 40 A:0.6 G:0.4
chr28 10195054 2 36 CTT:0.888889 C:0.111111
chr28 10195593 2 36 GTATT:0.583333 G:0.416667
chr28 10195621 3 40 GT:0.4 G:0.1 GTTTTT:0.5
chr28 10196362 2 38 A:0.552632 G:0.447368
chr28 10196855 2 36 CTT:1 C:0
chr28 10196857 4 40 TTTTTC:0.85 T:0.025
TTTCTTTTC:0.125 TTTTTCTTTTCTTTTCTTTTC:0
chr28 10196894 2 40 T:0.925 TTTCTTTTTTCTTTTC:0.075
chr28 10196944 2 40 C:0.975 CCTTT:0.025
chr28 10196965 2 40 CTTTCTTT:1 C:0
chr28 10196972 2 38 T:0.842105 TC:0.157895
chr28 10196985 2 38 C:0.710526 T:0.289474
chr28 10198571 2 40 A:0.3 AAATT:0.7
chr28 10198572 2 40 A:0.925 AATT:0.075
chr28 10198658 4 40 C:0.375 CATAA:0.425
CATAAATAA:0.125 CATAAATAAATAA:0.075
chr28 10199197 2 36 C:0.833333 T:0.166667
chr28 10199651 2 32 C:0.1875 CT:0.8125
chr28 10199887 2 38 TTTTA:0.710526 T:0.289474
chr28 10201240 2 36 CATTT:0.0555556 C:0.944444
chr28 10201918 2 38 C:0.894737 T:0.105263
chr28 10202452 2 38 A:0 G:1
chr28 10204117 2 38 T:0 A:1
chr28 10204662 2 40 T:0.475 A:0.525
chr28 10204694 2 34 C:1 CAG:0
chr28 10204858 2 40 T:0.35 C:0.65
chr28 10204975 2 40 A:0.475 T:0.525
chr28 10206008 2 40 C:1 T:0
chr28 10206285 3 40 TA:0.45 T:0.075 TAA:0.475
chr28 10206466 2 32 C:0 T:1
chr28 10206866 2 30 C:0.566667 CA:0.433333
chr28 10206960 2 38 A:0.447368 AT:0.552632
chr28 10209139 2 30 C:0.4 CT:0.6
chr28 10209599 2 30 C:1 T:0
chr28 10209824 2 40 T:0.75 C:0.25
chr28 10210027 2 40 T:0.525 TA:0.475
chr28 10210545 2 38 G:0.394737 C:0.605263
chr28 10211524 2 38 CAG:1 C:0
chr28 10211592 2 38 C:1 T:0
chr28 10211945 2 40 G:0.5 A:0.5
chr28 10213084 2 40 A:0.5 G:0.5
chr28 10214082 2 38 C:0.973684 T:0.0263158
chr28 10215146 2 40 TAA:1 T:0
chr28 10215148 2 40 A:1 ATTTTTTTTTTTTT:0

chr28	10215235	2	34	A:0.352941		G:0.647059
chr28	10215560	2	28	A:0.464286		C:0.535714
chr28	10215797	2	34	CT:0.647059		C:0.352941
chr28	10216136	2	36	T:0.527778		G:0.472222
chr28	10216903	2	40	TTTTA:0.975		T:0.025
chr28	10217303	2	38	C:0.552632		G:0.447368
chr28	10217443	2	40	T:0.95	G:0.05	
chr28	10217656	2	36	C:0	T:1	
chr28	10217667	2	36	A:0	T:1	
chr28	10217670	2	36	A:0	T:1	
chr28	10217684	2	36	A:0	G:1	
chr28	10217713	2	40	G:0	C:1	
chr28	10217717	2	40	G:0	C:1	
chr28	10217725	2	40	G:0	A:1	
chr28	10217727	2	40	T:0	C:1	
chr28	10217732	2	40	TG:0	T:1	
chr28	10217750	2	40	G:0	T:1	
chr28	10217757	2	40	A:0	G:1	
chr28	10217769	2	40	C:0	CA:1	
chr28	10217772	2	40	G:0	T:1	
chr28	10217773	2	40	AT:0	A:1	
chr28	10217776	2	40	T:0	C:1	
chr28	10217785	2	40	G:0	GC:1	
chr28	10217791	2	40	TCTAG:0	T:1	
chr28	10217796	2	40	A:0	ATC:1	
chr28	10217805	2	40	A:0	T:1	
chr28	10217814	2	40	A:0	C:1	
chr28	10217815	2	40	C:0	A:1	
chr28	10217816	2	40	T:0	G:1	
chr28	10217887	2	38	G:0	A:1	
chr28	10219877	2	40	A:0.575	G:0.425	
chr28	10220208	2	36	G:0	GA:1	
chr28	10220401	2	40	T:0.425	TC:0.575	
chr28	10220414	2	40	A:0.95	T:0.05	
chr28	10220416	2	40	A:0.25	T:0.75	
chr28	10220438	2	38	G:0.552632		GT:0.447368
chr28	10220523	2	36	G:0.472222		A:0.527778
chr28	10220530	2	36	G:1	A:0	
chr28	10220819	2	40	A:0.8	T:0.2	
chr28	10220844	2	40	T:0.5	C:0.5	
chr28	10221024	2	40	A:0	G:1	
chr28	10221026	2	40	A:0	G:1	
chr28	10221031	2	40	AG:0	A:1	
chr28	10221051	2	40	G:0	A:1	
chr28	10221065	2	40	A:0	ATATT:1	
chr28	10221066	2	40	AAGGG:0	A:1	
chr28	10221075	2	40	T:0	A:1	
chr28	10221092	2	40	GAA:0	G:1	
chr28	10221094	2	40	A:0	ATTT:1	
chr28	10221096	2	40	C:0	CT:1	
chr28	10221102	2	40	T:0	TA:1	
chr28	10221130	2	40	G:0	T:1	
chr28	10221169	2	38	A:0	T:1	
chr28	10221188	2	38	G:0	T:1	

chr28 10221251 2 38 C:0.815789 CCT:0.184211
chr28 10221651 2 34 C:0.970588 CT:0.0294118
chr28 10221784 2 38 TTTCTTTC:0.605263 T:0.394737
chr28 10221870 2 38 CTCTTTCTTTCTTTCTTTCTTTCTT:0.736842 C:0.263158
chr28 10222097 2 38 TTCTTTCTTTCTTTCTTCCTTTCTCTCTCTCTCTC:0.342105 T:0.657895
chr28 10225079 2 38 TCCC:0.552632 T:0.447368
chr28 10225082 2 38 C:0.473684 T:0.526316
chr28 10225086 2 36 A:0.833333 ATTC:0.166667
chr28 10225154 2 36 G:1 A:0
chr28 10226261 4 36 CAG:0.166667 C:0.166667 CAGAG:0.416667 CAGAGAG:0.25
chr28 10226865 2 40 A:1 C:0
chr28 10227301 2 40 A:0.525 G:0.475
chr28 10227734 3 38 C:0.263158 CT:0.5 CTT:0.236842
chr28 10227822 2 36 AT:0.75 A:0.25
chr28 10228493 2 38 TAAA:1 T:0
chr28 10228498 2 38 A:1 T:0
chr28 10228609 2 38 C:0.973684 T:0.0263158
chr28 10229110 2 38 GA:0.605263 G:0.394737
chr28 10229543 2 40 G:1 GT:0
chr28 10229764 2 36 AT:0.472222 A:0.527778
chr28 10229828 2 38 C:0.526316 CT:0.473684
chr28 10230082 2 38 C:0.710526 A:0.289474
chr28 10231615 2 38 G:1 C:0
chr28 10231666 5 40 TTATCTATCTATCTATC:0.125 T:0.425 TTATC:0.15 TTATCTATCTATC:0.275 TTATCTATCTATCTATCTATC:0.025
chr28 10232630 2 40 T:0 A:1
chr28 10232644 2 40 ATTT:0 A:1
chr28 10232649 2 40 T:0 TGG:1
chr28 10232651 2 40 A:0 G:1
chr28 10232653 2 40 GC:0 G:1
chr28 10232658 2 40 A:0 G:1
chr28 10232662 2 40 C:0 T:1
chr28 10232681 2 40 C:0 A:1
chr28 10232696 2 40 G:0 T:1
chr28 10232705 2 40 C:0 A:1
chr28 10232731 2 36 A:0 T:1
chr28 10232738 2 36 T:0 A:1
chr28 10232760 2 36 C:0 A:1
chr28 10232886 2 38 GT:0.921053 G:0.0789474
chr28 10233249 2 38 T:0.526316 A:0.473684
chr28 10233584 2 36 CT:0.222222 C:0.777778
chr28 10233628 3 38 A:0.578947 AAG:0.421053 AAGAG:0
chr28 10234058 2 40 T:0.575 A:0.425
chr28 10234981 2 38 TA:0.710526 T:0.289474
chr28 10236023 2 38 CTGATTGAT:0.894737 C:0.105263
chr28 10236871 2 40 C:0.525 A:0.475

chr28	10237348	3	40	T:0.45	TTTTTA:0.4	TTTTTATTTTA:0.15
chr28	10237407	3	40	CAG:0.875	C:0.05	CAGAG:0.075
chr28	10237409	2	40	G:0.525	C:0.475	
chr28	10237514	2	32	G:0.46875	C:0.53125	
chr28	10237537	2	34	G:0.529412	A:0.470588	
chr28	10239051	2	36	AGT:0.972222	A:0.0277778	
chr28	10239081	2	36	T:0.583333	A:0.416667	
chr28	10239174	2	38	T:0.447368	A:0.552632	
chr28	10241743	2	38	C:0.868421	T:0.131579	
chr28	10241847	2	40	A:0.575	T:0.425	
chr28	10241959	2	40	C:0.6	T:0.4	
chr28	10242101	2	40	A:0.5	C:0.5	
chr28	10242211	2	40	AT:0.5	A:0.5	
chr28	10243424	2	38	T:1	C:0	
chr28	10243517	2	40	A:0.5	AAAAAC:0.5	
chr28	10243960	2	40	C:0.525	CCT:0.475	
chr28	10244078	2	40	T:0.475	G:0.525	
chr28	10244190	2	40	C:0.45	A:0.55	
chr28	10244813	2	38	A:0.552632	G:0.447368	
chr28	10245114	3	40	G:0.375	GT:0.525	GTT:0.1
chr28	10245632	2	40	C:1	T:0	
chr28	10246538	2	38	C:0.973684	CT:0.0263158	
chr28	10246807	3	34	CTT:0.676471	C:0.294118	CTTT:0.0294118
chr28	10247331	2	38	TA:0.421053	T:0.578947	
chr28	10247387	2	34	TA:0.882353	T:0.117647	
chr28	10247592	2	28	G:0.607143	GT:0.392857	
chr28	10247617	2	36	C:0.444444	CT:0.555556	
chr28	10247886	2	40	T:0.85	C:0.15	
chr28	10248273	2	38	AC:0.736842	A:0.263158	
chr28	10248337	2	38	T:1	TGAGA:0	
chr28	10250140	2	38	A:0.447368	G:0.552632	
chr28	10250481	2	36	CT:0.75	C:0.25	
chr28	10250920	2	38	T:0.5	C:0.5	
chr28	10251016	2	40	T:1	G:0	
chr28	10252020	2	38	G:0.631579	A:0.368421	
chr28	10254917	2	34	TCA:0.941176	T:0.0588235	
chr28	10254924	2	34	C:0.470588	G:0.529412	
chr28	10255526	2	38	G:0	C:1	
chr28	10255597	2	40	C:0	A:1	
chr28	10255637	2	40	G:0	T:1	
chr28	10255654	2	40	T:0	A:1	
chr28	10255824	2	36	CCT:0.5	C:0.5	
chr28	10256382	2	36	C:0.472222	T:0.527778	
chr28	10256928	2	38	C:1	CA:0	
chr28	10257348	2	38	G:0	A:1	
chr28	10257358	2	38	G:0	A:1	
chr28	10258105	2	38	CT:0.421053	C:0.578947	
chr28	10258193	2	40	A:0.025	G:0.975	
chr28	10259148	2	30	C:1	CAG:0	
chr28	10259732	2	40	T:1	TA:0	
chr28	10259775	3	40	ATTTTAT:0.4	A:0.55	

ATTTTATTTTTAT:0.05
chr28 10259839 2 30 C:0.633333 CAG:0.366667
chr28 10261339 2 40 T:1 A:0
chr28 10261340 2 40 T:1 A:0
chr28 10261358 2 38 T:0.526316 A:0.473684
chr28 10263091 2 36 T:0.0277778 A:0.972222
chr28 10263112 2 36 AGACTGGGAG:0.0277778 A:
0.972222
chr28 10263138 2 12 AAAACCAAAAAAAAC:0 A:1
chr28 10263139 2 12 AAACCAAAAAAAAC:1 A:0
chr28 10263157 2 36 T:0.0277778 A:0.972222
chr28 10263164 2 36 C:0.0277778 A:0.972222
chr28 10263165 2 36 C:0.0277778 T:0.972222
chr28 10263184 2 36 C:0.0277778 T:0.972222
chr28 10263187 2 36 G:0.0277778 T:0.972222
chr28 10263570 2 38
CCTTTTCCTTTTCCTTTTCCTTTTTCTTTTCTTTCTTTT:0.815789 C:0.184211
chr28 10263584 2 40 TTTTCCTTTTTCTTTTCTTTC:1 T:0
chr28 10263600 2 40 CTTT:0.9 C:0.1
chr28 10263781 2 36 GCCCCCCC:0.111111 G:
0.888889
chr28 10263940 2 30 G:0.333333 GTTTT:0.666667
chr28 10264356 2 36 CT:0.277778 C:0.722222
chr28 10264459 2 38 T:0.710526 A:0.289474
chr28 10264992 2 36 C:1 G:0
chr28 10265466 2 38 T:0.868421 A:0.131579
chr28 10265470 2 38 G:0.473684 T:0.526316
chr28 10265967 2 38 AT:0.578947 A:0.421053
chr28 10265975 2 38 T:0.578947 A:0.421053
chr28 10266146 2 36 G:1 GT:0
chr28 10267018 2 38 T:0.473684 C:0.526316
chr28 10267050 2 40 T:0.95 A:0.05
chr28 10267748 2 38 T:0.684211 A:0.315789
chr28 10269982 3 38 A:0.0526316 T:0.5 ATT:
0.447368
chr28 10269988 2 36 T:1 TTA:0
chr28 10271879 2 40 C:0.475 CT:0.525
chr28 10272031 2 36 C:0.888889 CT:0.111111
chr28 10272409 2 36 C:0.583333 CT:0.416667
chr28 10272452 2 34 C:0.882353 CAG:0.117647
chr28 10273279 2 38 C:0.526316 T:0.473684
chr28 10273590 2 40 A:0 T:1
chr28 10273628 2 40 G:0 T:1
chr28 10273855 2 40 C:1 T:0
chr28 10274988 2 36 C:0.527778 CT:0.472222
chr28 10275285 2 38 G:0.473684 A:0.526316
chr28 10275539 2 40 A:0.85 ATATG:0.15
chr28 10275577 2 40 G:0.9 A:0.1
chr28 10275764 2 40 G:0.475 A:0.525
chr28 10275837 2 40 T:0.475 A:0.525
chr28 10277251 2 40 C:0.5 A:0.5
chr28 10277361 2 38 T:0.657895 TTTTTG:0.342105
chr28 10277457 2 40 CTG:0.475 C:0.525
chr28 10277913 2 40 A:0.7 G:0.3

chr28 10278194 2 38 T:0 A:1
chr28 10278201 2 38 C:0 G:1
chr28 10278206 2 38 G:0 T:1
chr28 10278208 2 38 C:0 T:1
chr28 10278214 2 38 TA:0 T:1
chr28 10278235 2 38 C:0 A:1
chr28 10278245 2 38 T:0 A:1
chr28 10278248 2 38 C:0 T:1
chr28 10278252 2 38 T:0 A:1
chr28 10278278 2 38 G:0 T:1
chr28 10278281 2 38 A:0 T:1
chr28 10278287 2 38 G:0 T:1
chr28 10278347 2 38 G:0 T:1
chr28 10278366 2 38 T:0 TG:1
chr28 10278374 2 38 GA:0 G:1
chr28 10278377 2 38 A:0 T:1
chr28 10278380 2 38 C:0 T:1
chr28 10278383 2 38 C:0 T:1
chr28 10278384 2 38 G:0 GT:1
chr28 10278389 2 38 A:0 T:1
chr28 10278392 2 38 GA:0 G:1
chr28 10278414 2 36 G:0 T:1
chr28 10278426 2 38 A:0 AG:1
chr28 10278427 2 38 A:0 AT:1
chr28 10278429 2 38 C:0 T:1
chr28 10278431 2 38 T:0 TA:1
chr28 10278436 2 38 C:0 CA:1
chr28 10278862 5 40 T:0.525 TGAGA:0.325 TGAGAGA:0.075 TGAGAGAGA:0.075 TGAGAGAGAGA:0
chr28 10280142 2 38 C:0.552632 T:0.447368
chr28 10282101 2 40 A:0.5 C:0.5
chr28 10283438 4 40 TATTC:0.225 T:0.275 TATTCATTC:0.075 TATTCATTCATTC:0.425
chr28 10283617 2 38 C:1 T:0
chr28 10283905 4 38 TCACACA:0.421053 T:0.5 TCA:0.0263158 TCACA:0.0526316
chr28 10284389 2 38 CT:1 C:0
chr28 10284402 2 38 T:0.552632 A:0.447368
chr28 10284429 2 34 C:0.617647 CAG:0.382353
chr28 10284649 2 38 T:0.710526 G:0.289474
chr28 10286031 2 38 CT:0.578947 C:0.421053
chr28 10286311 2 36 T:0.972222 TA:0.0277778
chr28 10286602 2 24 TG:0.833333 T:0.166667
chr28 10286866 2 32 A:0.625 AT:0.375
chr28 10288118 2 40 C:0.5 A:0.5
chr28 10289368 2 40 AAATG:0.5 A:0.5
chr28 10290222 2 38 C:0.473684 T:0.526316
chr28 10291300 2 32 T:1 A:0
chr28 10291599 2 38 T:0.5 G:0.5
chr28 10292188 2 36 G:0.805556 GC:0.194444
chr28 10292196 2 36 C:0.861111 A:0.138889
chr28 10293113 2 40 A:0.7 AC:0.3
chr28 10293942 2 40 A:0.9 G:0.1
chr28 10293975 2 38 CTG:0.526316 C:0.473684

chr28	10295027	2	40	G:1	T:0	
chr28	10296691	2	38	C:0.605263	A:0.394737	
chr28	10297215	2	30	A:0.766667	AT:0.233333	
chr28	10297228	2	26	A:0.461538	AT:0.538462	
chr28	10297234	2	32	T:0.78125	TA:0.21875	
chr28	10297242	2	28	A:0.642857	T:0.357143	
chr28	10297584	2	40	T:1	G:0	
chr28	10298321	2	38	T:0.5	G:0.5	
chr28	10299018	2	38	A:0.421053	C:0.578947	
chr28	10299242	2	40	G:0.525	GTGATGA:0.475	
chr28	10299389	2	38	AG:1	A:0	
chr28	10299571	2	40	CT:0.925	C:0.075	
chr28	10299671	2	38	G:0.421053	A:0.578947	
chr28	10300921	2	40	T:0.525	C:0.475	
chr28	10303275	2	38	T:1	TAATAATTAAATA:0	
chr28	10303278	2	34	TA:0.970588	T:0.0294118	
chr28	10303281	2	34	A:0.882353	T:0.117647	
chr28	10303282	2	34	TAAA:0.970588	T:0.0294118	
chr28	10303322	2	38	T:0.842105	TAAATAAA:0.157895	
chr28	10305770	2	38	GT:0.421053	G:0.578947	
chr28	10306110	2	38	G:0.973684	A:0.0263158	
chr28	10306667	3	36	G:0.5	GT:0.0833333	GTT: 0.416667
chr28	10308168	2	40	CTTTG:0.9	C:0.1	
chr28	10308216	2	38	TA:0.631579	T:0.368421	
chr28	10308710	2	40	T:1	C:0	
chr28	10309124	2	40	T:1	TCTTCTCCTTCTC:0	
chr28	10309226	2	38	T:1	A:0	
chr28	10309300	2	36	G:0.416667	A:0.583333	
chr28	10309586	2	38	T:0.473684	TTG:0.526316	
chr28	10309762	2	40	C:1	T:0	
chr28	10314496	2	40	AG:0.425	A:0.575	
chr28	10318186	2	38	T:0.526316	G:0.473684	
chr28	10320740	2	34	C:0.764706	T:0.235294	
chr28	10322045	2	38	T:1	A:0	
chr28	10322857	2	38	G:0.684211	A:0.315789	
chr28	10323774	2	38	A:0.894737	G:0.105263	
chr28	10324368	2	38	TAC:0.710526	T:0.289474	
chr28	10326115	2	40	A:0.5	T:0.5	
chr28	10326851	2	38	T:0.710526	TA:0.289474	
chr28	10327782	2	40	TA:0.55	T:0.45	
chr28	10328178	2	40	AG:1	A:0	
chr28	10328491	2	32	C:0.8125	CCTCT:0.1875	
chr28	10329049	3	36	TA:0.194444	T:0.361111	TAA:0.444444
chr28	10330111	2	38	G:1	A:0	
chr28	10330709	2	28	G:0	T:1	
chr28	10330904	2	38	TA:0.368421	T:0.631579	
chr28	10331042	2	40	A:1	T:0	
chr28	10331876	2	40	G:1	A:0	
chr28	10333265	2	38	C:0.5	T:0.5	
chr28	10333520	2	38	G:0.447368	A:0.552632	
chr28	10335857	2	40	G:1	A:0	
chr28	10336123	2	38	CTTTG:0.447368	C:0.552632	

chr28 10336126 2 38 T:0.552632 G:0.447368
chr28 10336127 2 38 G:0.552632 T:0.447368
chr28 10337878 2 40 TTAGCTGCATG:1 T:0
chr28 10338084 2 38 A:0.526316 AT:0.473684
chr28 10338969 2 40 A:0.5 G:0.5
chr28 10342121 2 40 C:1 G:0
chr28 10345207 2 40 G:0.575 T:0.425
chr28 10346971 2 40 A:0.5 AT:0.5
chr28 10346982 2 40 AT:1 A:0
chr28 10346991 2 40 T:1 A:0
chr28 10347370 2 36 G:1 T:0
chr28 10347881 2 38 T:0.5 C:0.5
chr28 10348188 2 36 G:1 A:0
chr28 10348754 2 40 T:0 C:1
chr28 10350541 2 40 C:0.95 T:0.05
chr28 10352784 2 36 T:0.0277778 G:0.972222
chr28 10353339 2 20 C:0.7 T:0.3
chr28 10353730 2 38 C:0.842105 A:0.157895
chr28 10353892 2 36 C:1 T:0
chr28 10354223 2 36 T:0.666667 C:0.333333
chr28 10354372 2 38 G:0.421053 GC:0.578947
chr28 10354374 2 38 G:0.421053 T:0.578947
chr28 10354908 2 40 C:1 T:0
chr28 10355356 2 36 C:0.777778 CA:0.222222
chr28 10355921 2 40 A:0.425 G:0.575
chr28 10356475 2 34 TG:0.705882 T:0.294118
chr28 10357042 2 40 T:0.55 C:0.45
chr28 10358217 2 38 TA:0.973684 T:0.0263158
chr28 10358632 2 38 C:0.5 A:0.5
chr28 10358988 2 38 G:1 A:0
chr28 10359688 2 38 C:1 T:0
chr28 10360705 2 38 T:0 A:1
chr28 10360707 2 38 A:0 AT:1
chr28 10360711 2 38 G:0 T:1
chr28 10360718 2 38 A:0 AT:1
chr28 10360739 2 40 T:0 G:1
chr28 10360895 2 38 A:0.5 G:0.5
chr28 10361065 2 36 G:0.555556 A:0.444444
chr28 10361098 2 34 T:0.558824 C:0.441176
chr28 10362366 2 38 C:0.5 G:0.5
chr28 10363075 3 36 ATG:0.805556 A:0 ATGTG:
0.194444
chr28 10363085 2 36 G:0.944444 GTA:0.0555556
chr28 10363087 4 38 G:0.815789 A:0.105263
GTA:0 GTATA:0.0789474
chr28 10363501 2 40 C:1 T:0
chr28 10363642 2 38 G:0.394737 A:0.605263
chr28 10363714 2 38 G:0.473684 C:0.526316
chr28 10363864 2 36 T:0.5 C:0.5
chr28 10364030 2 40 G:0.45 C:0.55
chr28 10364043 2 40 G:0.525 A:0.475
chr28 10365147 2 36 G:0.5 A:0.5
chr28 10365157 2 36 A:0.972222 C:0.0277778
chr28 10365235 2 40 G:0.45 GAACGCTGCAGCCC:0.55

chr28	10365596	2	36	G:0.416667	A:0.583333
chr28	10365841	2	36	CGG:0.583333	C:0.416667
chr28	10365890	2	36	T:0.527778	C:0.472222
chr28	10366396	2	36	C:1	T:0
chr28	10366452	2	34	C:1	T:0
chr28	10366773	2	34	G:1	A:0
chr28	10366873	2	38	G:0.5	A:0.5
chr28	10367446	2	38	CCG:0.552632	C:0.447368
chr28	10367453	2	38	C:0.552632	CACACA:0.447368
chr28	10367644	2	38	C:0.394737	T:0.605263
chr28	10367688	2	40	G:0	C:1
chr28	10367732	2	38	G:0.5	T:0.5
chr28	10368120	2	38	T:0.421053	C:0.578947
chr28	10369407	2	34	A:1	AGT:0
chr28	10370816	2	38	G:0	A:1
chr28	10370817	2	38	A:0	T:1
chr28	10370858	2	40	T:0	C:1
chr28	10370865	2	40	C:0	G:1
chr28	10370866	2	40	TCCCC:0	T:1
chr28	10370872	2	40	C:0	CTATG:1
chr28	10370927	2	38	T:0	TA:1
chr28	10374078	2	34	C:0.0882353	T:0.911765
chr28	10377572	2	36	C:1	T:0
chr28	10378750	2	34	A:0.852941	G:0.147059
chr28	10378769	2	34	G:0.823529	A:0.176471
chr28	10379163	2	36	C:0.944444	T:0.0555556
chr28	10379774	2	34	G:0.882353	C:0.117647
chr28	10379827	2	34	T:0.911765	C:0.0882353
chr28	10379987	2	34	A:0.970588	G:0.0294118
chr28	10380811	2	34	C:0.911765	A:0.0882353
chr28	10381643	2	38	A:0.921053	G:0.0789474
chr28	10381703	2	40	C:1	T:0
chr28	10381792	2	38	G:0.921053	A:0.0789474
chr28	10381872	2	40	TCC:0.725	T:0.275
chr28	10381875	2	40	TCTCCTCTCCTCTCCTCCCCTCCC:0.725	T:0.275
chr28	10382353	2	38	C:0.868421	G:0.131579
chr28	10382580	2	36	C:0.416667	T:0.583333
chr28	10383052	2	38	A:0.815789	T:0.184211
chr28	10383887	2	40	A:0.9	G:0.1
chr28	10384028	2	38	A:0.921053	G:0.0789474
chr28	10384349	2	32	C:0.90625	T:0.09375
chr28	10384687	2	38	T:0.921053	C:0.0789474
chr28	10385385	2	38	G:0	C:1
chr28	10385825	2	38	G:0.894737	A:0.105263
chr28	10385939	2	38	A:0.894737	G:0.105263
chr28	10385971	2	36	G:0.861111	GA:0.138889
chr28	10385974	2	36	G:0.861111	A:0.138889
chr28	10386297	2	36	T:0.944444	C:0.0555556
chr28	10386315	2	36	G:0.944444	C:0.0555556
chr28	10386328	2	30	A:1	G:0
chr28	10386501	2	36	G:0.944444	GA:0.0555556
chr28	10386609	2	36	A:0.944444	T:0.0555556
chr28	10386908	2	36	C:0.888889	T:0.111111

chr28	10386918	2	36	T:0.888889	A:0.111111
chr28	10387323	2	36	C:0.861111	T:0.138889
chr28	10387401	2	34	G:1	A:0
chr28	10387594	2	38	T:0.894737	C:0.105263
chr28	10387806	2	38	A:0.894737	G:0.105263
chr28	10388313	2	40	T:0.9	C:0.1
chr28	10388466	2	38	TG:0.921053	T:0.0789474
chr28	10388628	2	40	T:0.9	TA:0.1
chr28	10388841	2	38	A:0.921053	G:0.0789474
chr28	10389161	2	26	C:0.923077	T:0.0769231
chr28	10389928	2	38	A:0.894737	ATTTCATT:0.105263
chr28	10390280	2	36	C:0.916667	T:0.0833333
chr28	10390562	2	32	A:0.96875	C:0.03125
chr28	10390574	2	32	T:0.96875	C:0.03125
chr28	10391260	2	32	CA:0	C:1
chr28	10391476	2	40	G:0.9	C:0.1
chr28	10391557	2	40	C:0.85	T:0.15
chr28	10391721	2	38	G:0.894737	A:0.105263
chr28	10391895	2	36	C:0.888889	T:0.111111
chr28	10392035	2	40	C:0.875	T:0.125
chr28	10392085	2	36	G:0.666667	T:0.333333
chr28	10392319	2	36	CG:0	C:1
chr28	10392365	2	36	G:0.888889	A:0.111111
chr28	10392446	2	34	T:0.823529	C:0.176471
chr28	10392796	2	38	T:0.921053	C:0.0789474
chr28	10392902	2	36	A:0.972222	G:0.0277778
chr28	10393550	2	40	C:0.925	G:0.075
chr28	10393652	2	36	G:1	GC:0
chr28	10393861	2	38	C:0.921053	T:0.0789474
chr28	10394024	2	38	T:0.868421	C:0.131579
chr28	10394271	2	38	AT:0.868421	A:0.131579
chr28	10394288	2	38	C:0.868421	A:0.131579
chr28	10394868	2	38	G:0.842105	A:0.157895
chr28	10394921	2	38	G:0.868421	T:0.131579
chr28	10395152	2	40	G:0.9	A:0.1
chr28	10395369	2	40	G:0.875	C:0.125
chr28	10395401	2	38	T:0.894737	TA:0.105263
chr28	10395424	2	38	C:0.894737	CA:0.105263
chr28	10395599	2	38	G:0.868421	T:0.131579
chr28	10395865	2	38	A:0	T:1
chr28	10395870	2	38	C:0.894737	T:0.105263
chr28	10395873	2	38	C:0	A:1
chr28	10395874	2	38	A:0	T:1
chr28	10395884	2	38	G:0.894737	A:0.105263
chr28	10396284	3	36	A:0.333333	AG:0.111111
AAGAG:0.555556					
chr28	10396416	2	30	G:0.8	C:0.2
chr28	10396706	2	30	C:0.333333	A:0.666667
chr28	10396711	2	32	T:0.71875	A:0.28125
chr28	10396728	2	30	A:0.8	T:0.2
chr28	10397205	2	38	T:0.894737	C:0.105263
chr28	10397433	2	38	T:0.921053	C:0.0789474
chr28	10397476	2	38	T:1	G:0
chr28	10397487	2	40	G:1	A:0

chr28	10397646	2	34	GC:0.941176	G:0.0588235
chr28	10397753	2	40	A:0.9	C:0.1
chr28	10398029	2	38	C:0.921053	T:0.0789474
chr28	10398460	2	40	T:0.85	C:0.15
chr28	10398873	2	40	C:0.9	T:0.1
chr28	10399254	2	40	G:0.9	A:0.1
chr28	10399272	2	40	A:1	AGGAGGAGCAGG:0
chr28	10399274	2	40	CCA:1	C:0
chr28	10399850	2	38	C:1	G:0
chr28	10400340	2	40	C:0.9	T:0.1
chr28	10400437	2	38	G:1	A:0
chr28	10400982	2	36	T:0.916667	C:0.0833333
chr28	10401034	2	38	T:0.868421	C:0.131579
chr28	10401176	2	38	A:0.868421	T:0.131579
chr28	10401361	2	38	A:0.894737	G:0.105263
chr28	10401442	2	38	C:0.921053	T:0.0789474
chr28	10401785	2	38	C:1	T:0
chr28	10401793	2	38	G:1	A:0
chr28	10402031	2	38	G:0.973684	A:0.0263158
chr28	10402078	2	38	T:0.894737	C:0.105263
chr28	10402969	2	40	C:1	T:0
chr28	10403312	2	40	A:0.925	C:0.075
chr28	10403318	2	40	G:0.925	A:0.075
chr28	10403424	2	34	C:0.941176	T:0.0588235
chr28	10403627	2	34	T:0.882353	G:0.117647
chr28	10403866	2	38	T:0	C:1
chr28	10403925	2	38	A:0.947368	G:0.0526316
chr28	10404228	2	38	A:0.921053	G:0.0789474
chr28	10404455	2	28	G:0	GT:1
chr28	10404475	2	28	T:0	TA:1
chr28	10404477	2	28	GA:0	G:1
chr28	10404483	2	28	AC:0	A:1
chr28	10404523	2	38	C:0.315789	CT:0.684211
chr28	10404526	2	38	A:0.315789	G:0.684211
chr28	10404528	2	38	A:0.315789	G:0.684211
chr28	10404530	2	38	CG:0.315789	C:0.684211
chr28	10404532	2	38	CG:0.315789	C:0.684211
chr28	10404536	2	38	C:0.315789	A:0.684211
chr28	10404538	2	38	C:0.315789	A:0.684211
chr28	10404550	2	38	CCCCCCCCCCTCCCG:0.315789	C:0.684211
chr28	10404948	2	38	T:0.921053	C:0.0789474
chr28	10405303	2	40	T:0.9	C:0.1
chr28	10405445	2	34	G:0.617647	A:0.382353
chr28	10405479	2	34	T:0	TG:1
chr28	10405525	2	38	G:0	GT:1
chr28	10405526	2	38	A:0	G:1
chr28	10405552	2	40	A:0	AT:1
chr28	10405745	2	40	T:0.95	C:0.05
chr28	10405770	2	36	G:1	A:0
chr28	10405847	2	34	G:0.882353	A:0.117647
chr28	10406752	2	40	C:1	T:0
chr28	10407069	2	38	G:1	A:0
chr28	10407082	2	36	G:0.916667	A:0.0833333

chr28 10407326 2 38 A:0.921053 AT:0.0789474
chr28 10407690 2 38 T:0.894737 C:0.105263
chr28 10407941 2 40 G:1 A:0
chr28 10407944 2 40 A:1 G:0
chr28 10408200 2 40 T:1 C:0
chr28 10408224 2 40 G:1 A:0
chr28 10408225 2 40 C:0.85 A:0.15
chr28 10408264 2 38 A:0.842105 C:0.157895
chr28 10408297 2 40 G:1 A:0
chr28 10408306 2 40 C:1 T:0
chr28 10409581 2 36 C:1 T:0
chr28 10409893 3 34 CCTCT:0.441176 C:0.235294
CCT:0.323529
chr28 10410057 2 40 G:0.925 A:0.075
chr28 10410061 2 40 T:0.925 C:0.075
chr28 10410483 2 36 T:0.888889 C:0.111111
chr28 10410673 2 36 C:1 A:0
chr28 10410775 2 34 GACT:1 G:0
chr28 10410995 2 36 A:1 T:0
chr28 10411027 2 38 G:0.894737 A:0.105263
chr28 10411592 2 36 C:0.944444 CT:0.0555556
chr28 10411745 2 40 C:1 T:0
chr28 10411760 2 40 A:1 C:0
chr28 10411798 2 40 C:1 T:0
chr28 10411853 2 40 T:1 A:0
chr28 10412125 2 40 T:0.9 C:0.1
chr28 10412330 2 40 A:1 G:0
chr28 10412347 2 40 G:1 A:0
chr28 10412552 2 38 C:1 T:0
chr28 10412857 2 38 T:0.842105 TC:0.157895
chr28 10413499 2 38 C:0.894737 CG:0.105263
chr28 10413599 2 38 C:0.894737 A:0.105263
chr28 10413685 2 36 A:0.916667 G:0.0833333
chr28 10414238 2 34 C:0.882353 CT:0.117647
chr28 10414470 2 38 T:0.921053 C:0.0789474
chr28 10414517 2 38 G:0.947368 A:0.0526316
chr28 10414527 2 38 C:0.947368 T:0.0526316
chr28 10415601 2 40 AAAAAT:1 A:0
chr28 10415621 2 40 TAAAATAAATA:0.925 T:0.075
chr28 10415626 2 40 T:0.975 TAAATA:0.025
chr28 10417377 3 40 CGAGAGAGAGAGA:0.7 C:0.275
CGAGAGA:0.025
chr28 10417383 2 40 A:1 ATC:0
chr28 10417945 2 38 G:0.921053 A:0.0789474
chr28 10418020 2 38 C:0.710526 A:0.289474
chr28 10418409 2 38 C:0.973684 T:0.0263158
chr28 10418813 3 40 CAT:0.775 C:0.1 CATAT:
0.125
chr28 10418846 2 40 T:0.9 A:0.1
chr28 10418874 2 40 G:0.925 GTGTATATA:0.075
chr28 10419697 2 38 A:0.894737 C:0.105263
chr28 10419784 2 38 T:0.815789 TA:0.184211
chr28 10420261 2 40 T:0.85 C:0.15
chr28 10420334 2 38 T:1 C:0

chr28	10420397	2	40	G:0.925	T:0.075
chr28	10420564	2	38	T:0.894737	C:0.105263
chr28	10420766	2	40	A:0.9	G:0.1
chr28	10420781	2	40	A:0.9	G:0.1
chr28	10421296	2	38	T:0.921053	G:0.0789474
chr28	10421541	2	36	T:0.861111	C:0.138889
chr28	10421665	2	30	A:0.833333	G:0.166667
chr28	10422136	2	34	T:0.823529	TA:0.176471
chr28	10423036	2	36	A:0.916667	G:0.0833333
chr28	10423544	2	40	C:0.875	T:0.125
chr28	10423594	2	40	G:0.875	C:0.125
chr28	10423598	2	40	A:0.875	C:0.125
chr28	10423967	2	40	C:1	T:0
chr28	10424083	2	40	A:0.925	G:0.075
chr28	10424214	2	38	C:0.789474	T:0.210526
chr28	10424321	2	38	T:0.921053	TG:0.0789474
chr28	10424506	2	40	C:0.875	T:0.125
chr28	10424645	2	38	A:0.947368	C:0.0526316
chr28	10424808	2	38	G:0.921053	A:0.0789474
chr28	10424814	2	38	A:0.921053	G:0.0789474
chr28	10424852	2	36	C:0.888889	CT:0.111111
chr28	10424875	2	38	A:0.868421	G:0.131579
chr28	10425041	2	38	CGTGTGT:0.947368	C:0.0526316
chr28	10425511	2	40	G:0.925	A:0.075
chr28	10425606	2	40	T:0.925	C:0.075
chr28	10425839	2	38	T:0.868421	A:0.131579
chr28	10425952	2	40	C:0.9	T:0.1
chr28	10427022	2	36	GC:0.888889	G:0.111111
chr28	10427176	2	38	G:0.947368	A:0.0526316
chr28	10427759	2	40	G:0.875	A:0.125
chr28	10427979	2	38	C:1	T:0
chr28	10428209	2	34	C:0.882353	G:0.117647
chr28	10428504	2	38	A:0.921053	G:0.0789474
chr28	10428822	2	28	AT:0.714286	A:0.285714
chr28	10428863	2	36	G:0.888889	A:0.111111
chr28	10429206	2	40	C:0.9	A:0.1
chr28	10429290	2	40	T:1	C:0
chr28	10429814	2	38	TC:0.921053	T:0.0789474
chr28	10430037	2	38	C:0.894737	T:0.105263
chr28	10430172	2	34	C:0.882353	T:0.117647
chr28	10431042	2	40	C:0.975	CT:0.025
chr28	10431233	2	40	G:0.925	A:0.075
chr28	10431502	2	40	T:0.9	C:0.1
chr28	10432230	2	38	G:1	T:0
chr28	10432245	2	40	G:0.95	A:0.05
chr28	10432448	2	38	T:0.842105	A:0.157895
chr28	10432673	2	40	C:0.625	T:0.375
chr28	10433001	2	28	AGAGAGAGGAG:0.928571	A:0.0714286
chr28	10433005	2	26	AGAGGAG:0.769231	A:0.230769
chr28	10433007	2	22	AGGAG:0.409091	A:0.590909
chr28	10434312	2	38	A:0.868421	G:0.131579
chr28	10436203	2	36	C:1	G:0
chr28	10436522	2	38	G:1	A:0

chr28	10437820	2	36	C:0.972222	G:0.0277778	
chr28	10438297	2	40	G:0.6	T:0.4	
chr28	10438805	2	40	GCCAGGAC:0.875	G:0.125	
chr28	10441394	2	40	G:0.975	A:0.025	
chr28	10441797	2	40	G:0.575	C:0.425	
chr28	10441989	2	34	G:0.911765	T:0.0882353	
chr28	10442087	2	36	T:0.861111	A:0.138889	
chr28	10442113	2	36	A:0.888889	AG:0.111111	
chr28	10442807	2	38	A:0.973684	C:0.0263158	
chr28	10443124	2	34	C:0.941176	CCT:0.0588235	
chr28	10443155	2	38	TA:0.947368	T:0.0526316	
chr28	10443174	2	38	A:0.947368	AAAATTT:0.0526316	
chr28	10443485	2	40	A:0.925	T:0.075	
chr28	10443497	2	40	TA:0.925	T:0.075	
chr28	10443670	2	40	T:0.975	TA:0.025	
chr28	10443950	2	38	T:1	TAA:0	
chr28	10443955	2	38	A:0.342105	AAT:0.657895	
chr28	10444272	2	38	A:0.947368	G:0.0526316	
chr28	10444470	2	40	A:0.95	T:0.05	
chr28	10444772	2	40	C:0.975	T:0.025	
chr28	10445301	2	36	C:0.916667	T:0.0833333	
chr28	10445305	2	36	T:1	C:0	
chr28	10445326	2	38	C:0.894737	G:0.105263	
chr28	10445406	2	38	C:0.973684	A:0.0263158	
chr28	10445550	2	40	C:0.925	A:0.075	
chr28	10445640	2	40	C:0.925	T:0.075	
chr28	10446428	2	40	C:1	T:0	
chr28	10446700	2	40	T:0.95	C:0.05	
chr28	10446807	2	36	T:0.944444	C:0.0555556	
chr28	10446829	2	38	G:1	A:0	
chr28	10446843	2	38	G:1	A:0	
chr28	10447462	2	40	T:0.875	C:0.125	
chr28	10447683	2	38	C:0.973684	T:0.0263158	
chr28	10447739	2	36	G:0.833333	GT:0.166667	
chr28	10447794	2	36	C:0.777778	CAG:0.222222	
chr28	10448124	2	34	A:0.911765	G:0.0882353	
chr28	10448364	2	40	A:0.95	G:0.05	
chr28	10449013	2	38	G:0.973684	A:0.0263158	
chr28	10449165	2	36	T:0.972222	C:0.0277778	
chr28	10449174	2	36	G:0.972222	A:0.0277778	
chr28	10449975	2	40	C:0.95	T:0.05	
chr28	10449977	2	40	A:0.95	C:0.05	
chr28	10450144	2	38	A:0.947368	T:0.0526316	
chr28	10450153	3	40	TA:0.9	T:0.05	TAA:0.05
chr28	10450155	2	38	A:0.947368	T:0.0526316	
chr28	10451487	2	40	TCA:0.725	T:0.275	
chr28	10451760	2	40	C:0.725	T:0.275	
chr28	10453124	2	40	G:0.775	T:0.225	
chr28	10453220	2	32	C:0.21875	T:0.78125	
chr28	10453221	2	32	T:0.21875	G:0.78125	
chr28	10453340	2	40	G:0.15	A:0.85	
chr28	10453397	2	36	G:1	A:0	
chr28	10453654	2	40	T:0.2	C:0.8	
chr28	10454031	2	40	C:0.15	T:0.85	

chr28	10454118	2	38	G:0.947368	A:0.0526316
chr28	10455483	2	38	G:0.947368	A:0.0526316
chr28	10455514	2	40	T:0.925	C:0.075
chr28	10456454	2	36	T:0	C:1
chr28	10456632	2	38	C:0.947368	T:0.0526316
chr28	10457104	2	40	AT:0.05	A:0.95
chr28	10457244	2	40	A:0.95	T:0.05
chr28	10457252	2	40	C:0.95	T:0.05
chr28	10457765	2	38	T:0.947368	G:0.0526316
chr28	10458109	2	40	G:0.875	A:0.125
chr28	10458290	2	32	G:0	A:1
chr28	10458604	2	36	A:0	G:1
chr28	10458825	2	40	A:0	C:1
chr28	10459013	2	40	G:0.95	A:0.05
chr28	10460830	2	40	T:0.95	C:0.05
chr28	10460841	2	40	A:0.975	G:0.025
chr28	10461036	2	40	A:1	G:0
chr28	10461926	2	38	A:0.973684	G:0.0263158
chr28	10462118	2	38	A:0.0263158	C:0.973684
chr28	10462141	2	40	C:1	T:0
chr28	10462648	2	34	C:1	T:0
chr28	10462900	2	36	T:0.944444	C:0.0555556
chr28	10463060	2	34	A:0.911765	G:0.0882353
chr28	10463186	2	40	T:0	C:1
chr28	10463187	2	40	G:0	A:1
chr28	10463400	2	40	AT:1	A:0
chr28	10463475	2	38	CGGCTTT:0.0263158	C: 0.973684
chr28	10463618	2	38	C:0	T:1
chr28	10463649	2	38	A:0	G:1
chr28	10463654	2	38	A:0	C:1
chr28	10464467	2	40	CT:0	C:1
chr28	10465467	2	40	G:0.05	A:0.95
chr28	10466178	2	34	A:0	T:1
chr28	10466668	2	38	G:0.0263158	A:0.973684
chr28	10467048	2	34	C:0.852941	CT:0.147059
chr28	10468099	2	38	C:0	T:1
chr28	10468140	2	38	C:0	T:1
chr28	10468190	2	38	A:0	T:1
chr28	10468601	2	36	A:0	C:1
chr28	10468818	2	38	C:1	T:0
chr28	10469513	2	36	GT:1	G:0
chr28	10469933	2	38	T:0.921053	C:0.0789474
chr28	10470363	2	38	G:0.973684	A:0.0263158
chr28	10470454	2	38	C:1	T:0
chr28	10471019	2	40	T:0.95	C:0.05
chr28	10471128	2	38	A:0.921053	G:0.0789474
chr28	10471268	2	38	G:0	T:1
chr28	10471749	2	40	G:1	C:0
chr28	10472372	2	38	G:0	C:1
chr28	10472382	2	38	C:0	T:1
chr28	10472405	2	38	A:0	G:1
chr28	10472496	2	40	T:0	C:1
chr28	10472721	2	40	G:1	A:0

chr28	10473124	2	36	C:1	T:0	
chr28	10473220	2	36	C:1	A:0	
chr28	10473702	2	38	A:0.947368	G:0.0526316	
chr28	10474306	2	40	G:1	T:0	
chr28	10474709	2	40	C:0	G:1	
chr28	10474800	2	40	A:0	G:1	
chr28	10475240	2	40	CAAAT:0.025	C:0.975	
chr28	10476277	2	40	A:0.025	AT:0.975	
chr28	10476460	2	36	CAG:0.888889	C:0.111111	
chr28	10477100	2	40	G:0	C:1	
chr28	10477151	2	38	G:0	A:1	
chr28	10477238	2	40	T:1	A:0	
chr28	10477792	2	40	TG:0	T:1	
chr28	10477931	2	38	G:0	A:1	
chr28	10478437	2	32	AT:0	A:1	
chr28	10478679	2	40	G:0.05	A:0.95	
chr28	10478862	2	38	A:0	G:1	
chr28	10479039	2	40	C:1	T:0	
chr28	10479052	2	40	T:1	C:0	
chr28	10479655	2	38	C:1	A:0	
chr28	10479729	2	40	TACATACATATACAC:0.05		T:0.95
chr28	10480199	2	40	T:0.95	A:0.05	
chr28	10480614	2	38	C:1	A:0	
chr28	10480617	2	40	A:0.95	T:0.05	
chr28	10480627	2	40	C:0.825	T:0.175	
chr28	10480657	2	40	A:0.95	C:0.05	
chr28	10480753	2	34	CAAA:1	C:0	
chr28	10480930	2	40	G:0.075	A:0.925	
chr28	10481065	2	38	T:0.947368	C:0.0526316	
chr28	10481291	2	38	G:1	A:0	
chr28	10481321	2	38	G:0.894737	A:0.105263	
chr28	10481705	2	38	G:0.973684	A:0.0263158	
chr28	10481790	2	36	G:1	A:0	
chr28	10482555	2	38	T:0.947368	A:0.0526316	
chr28	10482662	2	38	A:1	G:0	
chr28	10482683	2	38	C:0.947368	CAGTT:0.0526316	
chr28	10482915	2	38	A:0.947368	C:0.0526316	
chr28	10483086	2	34	A:0.911765	G:0.0882353	
chr28	10483165	2	40	G:1	A:0	
chr28	10484059	2	38	A:0	T:1	
chr28	10484244	2	36	C:1	G:0	
chr28	10484531	2	40	AGAGGATGGATAGGTGAAG:0.9		A:0.1
chr28	10484723	2	40	A:0.95	G:0.05	
chr28	10484729	2	40	C:0.95	T:0.05	
chr28	10484756	2	40	A:0.95	AC:0.05	
chr28	10484941	2	38	A:0.947368	G:0.0526316	
chr28	10485235	2	38	C:0.973684	T:0.0263158	
chr28	10485354	2	40	G:0.05	A:0.95	
chr28	10485410	2	40	G:1	A:0	
chr28	10485591	2	38	A:0.973684	G:0.0263158	
chr28	10485726	2	38	T:1	C:0	
chr28	10485753	2	36	T:0.944444	C:0.0555556	
chr28	10486174	2	40	A:0.075	G:0.925	
chr28	10486240	2	40	C:0.05	CTAAA:0.95	

chr28	10486311	2	38	T:0.973684	A:0.0263158	
chr28	10486438	2	34	C:0.0294118	A:0.970588	
chr28	10486495	2	40	C:0	G:1	
chr28	10487737	2	36	A:0	G:1	
chr28	10487775	2	40	G:0.075	A:0.925	
chr28	10488156	2	40	T:1	C:0	
chr28	10488182	2	40	T:0.95	C:0.05	
chr28	10488230	2	40	G:0	A:1	
chr28	10488231	2	40	T:0	C:1	
chr28	10488467	2	40	C:1	CT:0	
chr28	10488606	2	36	T:0.944444	C:0.0555556	
chr28	10488873	2	40	AC:1	A:0	
chr28	10488986	2	38	G:1	A:0	
chr28	10489224	2	40	A:0.975	G:0.025	
chr28	10489255	2	40	A:0.025	C:0.975	
chr28	10489259	2	40	A:0.025	G:0.975	
chr28	10489306	2	38	C:0.0526316	CCA:0.947368	
chr28	10489446	2	40	CA:0.925	C:0.075	
chr28	10489453	2	40	G:0.925	C:0.075	
chr28	10489887	2	40	G:0	T:1	
chr28	10489978	2	38	T:0	G:1	
chr28	10490118	2	40	T:0	C:1	
chr28	10490313	2	36	A:0.944444	T:0.0555556	
chr28	10490316	2	36	G:0	C:1	
chr28	10490690	2	38	G:1	A:0	
chr28	10490875	2	34	G:0	A:1	
chr28	10490928	2	38	GAC:1	G:0	
chr28	10490959	2	40	G:0.025	A:0.975	
chr28	10490970	2	40	T:1	A:0	
chr28	10491278	2	40	G:0.075	A:0.925	
chr28	10491324	2	40	C:0	T:1	
chr28	10491898	2	36	T:1	C:0	
chr28	10492084	2	40	A:0	G:1	
chr28	10492096	2	40	G:1	A:0	
chr28	10492234	2	38	T:1	A:0	
chr28	10492251	2	38	A:1	T:0	
chr28	10492788	2	40	C:0.95	T:0.05	
chr28	10493120	2	38	C:0	T:1	
chr28	10493336	2	40	T:0.95	C:0.05	
chr28	10493467	2	40	G:1	C:0	
chr28	10493542	2	38	G:1	GC:0	
chr28	10493971	3	38	CTT:0.157895	C:0.131579	CT:0.710526
chr28	10494096	2	34	G:1	C:0	
chr28	10494328	2	40	CA:0.95	C:0.05	
chr28	10494341	2	40	G:0.725	A:0.275	
chr28	10494349	2	40	A:0	G:1	
chr28	10494830	2	40	T:0	C:1	
chr28	10494838	2	40	T:0	C:1	
chr28	10494914	2	34	T:0	C:1	
chr28	10495072	2	40	C:1	T:0	
chr28	10495147	2	38	C:0.973684	T:0.0263158	
chr28	10495571	2	38	G:0.973684	T:0.0263158	
chr28	10495874	2	40	C:1	T:0	

chr28	10495926	2	38	A:0.921053	G:0.0789474		
chr28	10496120	2	40	TAAGAAGAGGAAAAGAAAAAAAAAAAA:0.9	T:0.1		
chr28	10496286	2	38	A:0.0526316	G:0.947368		
chr28	10496348	2	38	G:0.0789474	T:0.921053		
chr28	10496390	2	38	A:0.947368	G:0.0526316		
chr28	10496416	2	38	A:0.947368	G:0.0526316		
chr28	10496501	2	40	T:1	C:0		
chr28	10496502	2	40	G:1	A:0		
chr28	10496557	2	38	G:1	A:0		
chr28	10496630	2	38	C:1	T:0		
chr28	10496764	2	36	G:0.0833333	A:0.916667		
chr28	10496770	2	36	G:1	C:0		
chr28	10496875	2	38	A:0	C:1		
chr28	10497044	2	38	T:1	C:0		
chr28	10497105	2	40	AAGG:0.05	A:0.95		
chr28	10497318	2	24	A:0	G:1		
chr28	10497383	2	28	C:0.0714286	A:0.928571		
chr28	10497468	2	28	G:1	A:0		
chr28	10497554	2	32	G:0.9375	A:0.0625		
chr28	10497558	2	32	C:0.9375	T:0.0625		
chr28	10497565	2	32	C:0.9375	A:0.0625		
chr28	10497589	2	32	A:0	C:1		
chr28	10497592	2	32	G:0	C:1		
chr28	10497660	2	36	G:0.972222	A:0.0277778		
chr28	10497693	2	36	A:0	AC:1		
chr28	10497725	2	38	A:0.973684	C:0.0263158		
chr28	10497773	2	38	A:0.973684	G:0.0263158		
chr28	10498021	2	40	T:0.05	C:0.95		
chr28	10498078	2	40	A:0.025	G:0.975		
chr28	10498147	4	40	GCACACA:0.075	G:0.85	GCA:0.025	GCACA:0.05
chr28	10498448	2	38	G:0.973684	A:0.0263158		
chr28	10498511	2	36	T:1	G:0		
chr28	10498541	2	38	AGGTCCTGTCTGGTAACAGGACCT:0.894737	A:0.105263		
chr28	10498710	2	40	C:0.975	T:0.025		
chr28	10498723	2	40	T:0.975	C:0.025		
chr28	10499073	2	40	C:0	CA:1		
chr28	10499137	2	40	C:0.975	T:0.025		
chr28	10499219	2	40	TC:0.95	T:0.05		
chr28	10499282	2	30	TA:0.633333	T:0.366667		
chr28	10499359	2	38	G:0.921053	C:0.0789474		
chr28	10499416	2	38	A:0.973684	T:0.0263158		
chr28	10499501	2	40	CA:0.95	C:0.05		
chr28	10499571	2	40	G:0.975	A:0.025		
chr28	10499708	2	40	T:0.95	C:0.05		
chr28	10499713	2	40	T:1	C:0		
chr28	10499878	2	36	A:0	G:1		
chr28	10500007	2	40	T:0.975	C:0.025		
chr28	10500100	3	38	T:0.921053	TA:0.0263158	TAA:0.0526316	
chr28	10500406	2	38	C:0.973684	T:0.0263158		
chr28	10500445	2	40	C:0.95	T:0.05		

chr28	10500483	2	40	G:0.975	A:0.025	
chr28	10500524	2	38	G:0.947368	A:0.0526316	
chr28	10500529	2	38	G:0.947368	A:0.0526316	
chr28	10500978	2	32	A:0.9375	G:0.0625	
chr28	10501240	2	40	A:0.975	G:0.025	
chr28	10501364	2	36	G:1	C:0	
chr28	10501382	2	38	A:1	G:0	
chr28	10501385	2	38	A:1	G:0	
chr28	10501432	2	40	T:0.95	C:0.05	
chr28	10501443	2	40	A:0.875	G:0.125	
chr28	10501454	2	40	G:1	A:0	
chr28	10501493	2	40	G:0.95	C:0.05	
chr28	10501534	2	40	C:1	T:0	
chr28	10501548	2	40	C:1	T:0	
chr28	10501639	2	36	G:0.972222	A:0.0277778	
chr28	10501640	2	36	T:0.972222	G:0.0277778	
chr28	10501691	2	38	A:0.947368	G:0.0526316	
chr28	10501816	2	38	T:0.921053	C:0.0789474	
chr28	10501919	2	36	T:0.944444	G:0.0555556	
chr28	10502181	2	38	A:1	C:0	
chr28	10502226	2	36	A:0.916667	G:0.0833333	
chr28	10502518	2	38	A:0.684211	G:0.315789	
chr28	10502522	2	38	G:1	A:0	
chr28	10502601	2	38	T:1	C:0	
chr28	10502730	2	38	C:0	G:1	
chr28	10502893	2	40	T:0.925	C:0.075	
chr28	10503172	2	38	G:1	A:0	
chr28	10503199	2	38	C:1	T:0	
chr28	10503801	2	32	CTTTTCTT:0.1875	C:0.8125	
chr28	10503808	2	40	T:1	TTTTTC:0	
chr28	10503843	2	36	CTTT:0.972222	C:0.0277778	
chr28	10504846	2	34	G:0.941176	T:0.0588235	
chr28	10505578	2	36	TGA:0.972222	T:0.0277778	
chr28	10505851	2	36	CT:1	C:0	
chr28	10506237	2	38	A:0.973684	C:0.0263158	
chr28	10506576	2	38	G:0.947368	A:0.0526316	
chr28	10506774	2	36	T:0.0277778	C:0.972222	
chr28	10506846	2	40	T:0.95	C:0.05	
chr28	10506862	2	40	C:0.95	CCTTG:0.05	
chr28	10506923	2	34	A:0.970588	G:0.0294118	
chr28	10507097	2	38	T:0.947368	C:0.0526316	
chr28	10507576	2	36	C:0.944444	T:0.0555556	
chr28	10507885	2	38	C:1	G:0	
chr28	10507946	2	34	T:0.941176	C:0.0588235	
chr28	10507957	2	18	C:0.277778	CT:0.722222	
chr28	10508091	2	38	C:0.868421	T:0.131579	
chr28	10508159	3	36	TAA:0.833333	T:0.0833333	TA:0.0833333
chr28	10508191	2	28	C:1	CT:0	
chr28	10508347	2	36	A:0.972222	G:0.0277778	
chr28	10508357	2	36	T:0.972222	C:0.0277778	
chr28	10508629	2	40	C:1	T:0	
chr28	10509066	2	38	G:0	T:1	
chr28	10511315	2	40	T:0.075	C:0.925	

chr28	10512059	2	40	G:1	A:0	
chr28	10512115	2	40	T:0.925	TTCTCTCTC:0.075	
chr28	10512616	2	40	G:0.575	GT:0.425	
chr28	10512620	2	40	T:1	G:0	
chr28	10512669	2	40	T:0.975	C:0.025	
chr28	10512749	2	40	T:0.975	C:0.025	
chr28	10513085	2	40	A:0.025	G:0.975	
chr28	10513322	2	40	C:0.8	T:0.2	
chr28	10513530	2	34	G:0.970588	T:0.0294118	
chr28	10514199	2	38	C:0.0263158	T:0.973684	
chr28	10514412	2	38	A:0	G:1	
chr28	10514583	2	38	C:0	T:1	
chr28	10515232	2	38	A:0	G:1	
chr28	10515282	2	38	T:0	A:1	
chr28	10515351	3	38	A:1	T:0	AT:0
chr28	10515445	2	40	G:1	A:0	
chr28	10516428	2	36	T:1	G:0	
chr28	10516548	2	38	T:1	C:0	
chr28	10517160	2	36	A:0	G:1	
chr28	10517313	2	40	T:0.075	C:0.925	
chr28	10517345	2	40	A:0.075	G:0.925	
chr28	10517435	2	18	C:0.222222	T:0.777778	
chr28	10517439	2	18	T:0.222222	TCC:0.777778	
chr28	10517453	2	34	T:0.882353	TCA:0.117647	
chr28	10517690	2	38	G:0.0789474	A:0.921053	
chr28	10517787	2	40	C:0.05	T:0.95	
chr28	10517820	2	40	C:0.075	T:0.925	
chr28	10518033	2	38	T:1	C:0	
chr28	10518080	2	38	G:1	GT:0	
chr28	10518081	3	38	G:0.157895	T:0	GTT:0.842105
chr28	10518269	2	38	G:1	A:0	
chr28	10518280	2	36	C:1	G:0	
chr28	10518281	2	36	C:0.833333	T:0.166667	
chr28	10518374	2	38	G:1	C:0	
chr28	10518411	2	34	G:1	C:0	
chr28	10518497	2	32	CG:1	C:0	
chr28	10519342	2	16	AG:0	A:1	
chr28	10519353	2	14	TC:0	T:1	
chr28	10519366	2	8	TC:0	T:1	
chr28	10519388	2	8	CT:0	C:1	
chr28	10519430	2	10	CT:0	C:1	
chr28	10519534	2	18	A:0.166667	G:0.833333	
chr28	10519546	2	18	A:0.166667	ACC:0.833333	
chr28	10519548	2	18	A:0.166667	G:0.833333	
chr28	10519654	2	34	G:1	C:0	
chr28	10519675	2	36	G:1	C:0	
chr28	10519739	2	32	T:0.4375	C:0.5625	
chr28	10519864	2	30	G:0.366667	A:0.633333	
chr28	10520051	2	16	AG:0.5	A:0.5	
chr28	10520150	2	40	A:1	G:0	
chr28	10520305	2	34	C:1	G:0	
chr28	10520332	2	34	AG:0.382353	A:0.617647	
chr28	10520668	2	40	G:0.375	A:0.625	

chr28	10520890	2	38	C:1	CG:0			
chr28	10520935	2	38	C:0.526316	T:0.473684			
chr28	10521102	2	40	A:0.475	G:0.525			
chr28	10521620	2	32	C:1	G:0			
chr28	10522208	2	38	C:0.526316	A:0.473684			
chr28	10522366	2	36	C:1	T:0			
chr28	10522633	2	38	A:0.447368	G:0.552632			
chr28	10522956	2	34	A:0.352941	AT:0.647059			
chr28	10522983	2	38	T:1	G:0			
chr28	10523071	2	34	A:0.588235	G:0.411765			
chr28	10523174	2	40	G:1	A:0			
chr28	10523305	2	38	A:1	G:0			
chr28	10523777	2	40	A:0.45	G:0.55			
chr28	10523822	2	40	G:1	A:0			
chr28	10524256	2	38	A:0.447368	G:0.552632			
chr28	10524518	2	40	T:0.5	G:0.5			
chr28	10524932	2	40	T:0.4	C:0.6			
chr28	10525567	2	36	G:0.611111	A:0.388889			
chr28	10525601	2	40	T:1	A:0			
chr28	10525681	2	40	C:0.625	T:0.375			
chr28	10525713	2	38	A:1	AT:0			
chr28	10525755	4	40	CAGAGAGAGAG:0	C:0.1	CAGAGAG:0.425	CAGAGAGAG:0.475	
chr28	10525997	2	40	A:0.675	C:0.325			
chr28	10526376	5	40	GAATAAATA:0.675	G:0.25	GAATA:0.05	GAATAAATAAATA:0.025	GAATAAATAAATAAATAAATA:0
chr28	10526519	2	36	A:0.583333	G:0.416667			
chr28	10527997	2	40	G:0.6	T:0.4			
chr28	10528186	2	38	GA:1	G:0			
chr28	10528530	2	36	A:1	AT:0			
chr28	10528700	2	38	G:0.631579	C:0.368421			
chr28	10528814	2	40	C:0.65	T:0.35			
chr28	10528977	2	40	C:1	G:0			
chr28	10529218	2	40	T:0.6	C:0.4			
chr28	10529380	2	36	C:0.611111	G:0.388889			
chr28	10529384	2	36	C:0.638889	A:0.361111			
chr28	10529489	3	38	TA:0.631579	T:0.342105	TAA:0.0263158		
chr28	10529526	2	36	C:0.527778	CTCTG:0.472222			
chr28	10530570	2	40	G:1	A:0			
chr28	10531210	2	40	A:1	G:0			
chr28	10531444	2	36	TG:1	T:0			
chr28	10531787	2	38	CA:1	C:0			
chr28	10531942	2	40	G:1	A:0			
chr28	10532071	2	36	G:0.611111	A:0.388889			
chr28	10532897	2	32	C:0.59375	T:0.40625			
chr28	10533054	2	38	G:0.526316	C:0.473684			
chr28	10533354	2	38	TAGAGG:1	T:0			
chr28	10533459	2	38	G:0.578947	T:0.421053			
chr28	10533652	2	40	G:1	A:0			
chr28	10533958	2	36	C:1	T:0			
chr28	10534843	2	36	G:1	A:0			
chr28	10534893	2	40	T:1	C:0			
chr28	10535021	3	40	GA:0.775	G:0.2	GAA:0.025		

chr28	10535315	2	40	AC:1	A:0		
chr28	10535331	2	40	G:1	T:0		
chr28	10535811	2	36	C:0.611111	T:0.388889		
chr28	10535898	2	38	T:1	G:0		
chr28	10535958	2	36	G:1	A:0		
chr28	10536054	2	38	C:1	G:0		
chr28	10536332	2	38	C:1	CAG:0		
chr28	10536420	2	38	A:0.605263	G:0.394737		
chr28	10536976	2	36	A:1	G:0		
chr28	10537452	2	40	C:0.675	CTGAG:0.325		
chr28	10537507	2	40	T:1	TTG:0		
chr28	10537895	3	36	CTGTGTGTG:0.166667	C:0.666667	CTGTG:0.166667	
chr28	10538001	3	36	T:0.75	TA:0.25	TAA:0	
chr28	10538258	2	40	T:0.675	C:0.325		
chr28	10538270	2	40	CA:0.7	C:0.3		
chr28	10538288	2	40	C:1	T:0		
chr28	10538316	2	38	C:1	T:0		
chr28	10538331	2	38	A:1	T:0		
chr28	10538333	2	38	G:1	A:0		
chr28	10538383	3	40	C:0.55	CCT:0.05	CCTCTCTCT:0.4	
chr28	10538390	3	38	ATC:0.315789	A:0.210526	CTC:0.473684	
chr28	10538436	2	40	A:0.65	T:0.35		
chr28	10538437	2	40	A:1	T:0		
chr28	10538626	2	36	C:0.805556	T:0.194444		
chr28	10538656	2	36	G:0.944444	A:0.0555556		
chr28	10538661	2	38	G:1	A:0		
chr28	10538951	4	40	TTTTA:0.85	T:0.1	TTTTATTTA:0	TTTTATTTATTTA:0.05
chr28	10538997	2	40	CAGAG:0.65	C:0.35		
chr28	10539277	2	40	G:0.975	A:0.025		
chr28	10539510	2	40	C:0.975	T:0.025		
chr28	10539517	2	40	G:0.625	A:0.375		
chr28	10539547	2	40	C:1	T:0		
chr28	10539548	2	40	T:0.975	A:0.025		
chr28	10539557	2	40	G:0.975	T:0.025		
chr28	10539625	2	40	G:0.65	C:0.35		
chr28	10539801	2	38	C:1	T:0		
chr28	10539911	2	40	T:0.6	C:0.4		
chr28	10540342	2	40	G:0.625	A:0.375		
chr28	10540382	2	40	T:1	C:0		
chr28	10540621	2	40	A:1	G:0		
chr28	10540628	2	38	T:0.605263	A:0.394737		
chr28	10540871	2	40	T:1	A:0		
chr28	10540884	2	38	A:0.631579	AT:0.368421		
chr28	10540929	2	32	C:0.90625	CAG:0.09375		
chr28	10541015	2	34	CG:1	C:0		
chr28	10541076	2	40	A:0	C:1		
chr28	10541096	2	40	T:0	G:1		
chr28	10541101	2	40	T:0	A:1		
chr28	10541103	2	40	T:0	A:1		
chr28	10541118	2	40	T:0	A:1		
chr28	10541125	2	40	A:0	C:1		

chr28	10541264	2	26	CCT:0.615385	C:0.384615	
chr28	10541327	2	28	T:0.0357143	TA:0.964286	
chr28	10541417	2	38	A:1	G:0	
chr28	10541554	2	32	G:1	A:0	
chr28	10541916	2	38	C:1	T:0	
chr28	10541958	2	36	G:0.527778	A:0.472222	
chr28	10541965	2	36	C:0.527778	T:0.472222	
chr28	10542313	2	38	ACT:0.657895	A:0.342105	
chr28	10542400	2	38	C:1	T:0	
chr28	10542690	2	36	CG:0.972222	C:0.0277778	
chr28	10542732	2	38	G:1	A:0	
chr28	10543285	2	38	A:1	G:0	
chr28	10544144	2	36	GATTA:1	G:0	
chr28	10544407	2	36	GC:0.555556	G:0.444444	
chr28	10544578	2	38	A:1	G:0	
chr28	10544598	2	36	C:1	A:0	
chr28	10544748	2	36	C:0.944444	T:0.0555556	
chr28	10544902	2	38	C:0.0263158	T:0.973684	
chr28	10544947	2	40	G:1	A:0	
chr28	10545316	2	40	A:0.55	G:0.45	
chr28	10545752	2	38	G:1	A:0	
chr28	10545882	2	40	T:1	C:0	
chr28	10545941	2	38	T:1	C:0	
chr28	10546062	3	34	GC:0.794118	G:0.147059	GCC:0.0588235
chr28	10546246	2	40	G:1	A:0	
chr28	10546274	2	38	AT:1	A:0	
chr28	10546470	2	40	T:0.025	G:0.975	
chr28	10546482	2	38	ATC:1	A:0	
chr28	10546594	2	28	C:1	CCG:0	
chr28	10546605	2	28	C:1	T:0	
chr28	10546670	2	38	A:0.973684	G:0.0263158	
chr28	10546749	2	38	G:0.947368	GT:0.0526316	
chr28	10546809	3	38	C:1	G:0	T:0
chr28	10546810	2	38	A:1	G:0	
chr28	10547311	2	36	C:1	CG:0	
chr28	10547549	2	38	T:1	C:0	
chr28	10547730	2	38	C:1	T:0	
chr28	10548151	2	38	C:0.368421	G:0.631579	
chr28	10548254	2	34	AAG:0.352941	A:0.647059	
chr28	10548327	2	28	AAAAGAAAG:0.75	A:0.25	
chr28	10548437	2	38	G:0.394737	GAAGAAAGA:0.605263	
chr28	10548616	2	38	T:1	C:0	
chr28	10548914	2	38	G:1	A:0	
chr28	10550186	2	38	T:0.868421	C:0.131579	
chr28	10550235	2	38	C:1	T:0	
chr28	10550733	2	30	TA:0.9	T:0.1	
chr28	10550976	2	38	ATC:1	A:0	
chr28	10551173	2	36	C:0.944444	G:0.0555556	
chr28	10551192	2	40	A:1	AT:0	
chr28	10551203	2	34	TA:0.823529	T:0.176471	
chr28	10551273	2	38	T:0.815789	TGA:0.184211	
chr28	10551427	2	36	G:0.805556	A:0.194444	

chr28	10551575	3	38	CTAAA:0.657895	C:0.0263158	CTAAATAAA:0.315789	
chr28	10551612	3	38	T:0.868421	A:0	TAAAA:0.131579	
chr28	10551999	2	34	G:0.882353	A:0.117647		
chr28	10552082	2	40	G:0.925	C:0.075		
chr28	10552149	2	40	T:1	TAATC:0		
chr28	10552741	2	40	CTT:0.9	C:0.1		
chr28	10552743	2	40	T:0.9	TACAC:0.1		
chr28	10553306	2	40	G:1	A:0		
chr28	10553549	2	40	G:1	C:0		
chr28	10554159	2	38	C:1	T:0		
chr28	10555699	4	40	CAG:0.775	C:0.1	CAGAG:0.05	CAGAGAGAG:0.075
chr28	10556187	2	38	CCAGT:0.842105	C:0.157895		
chr28	10556286	2	34	C:0.617647	CATAT:0.382353		
chr28	10556306	2	26	TAC:0.923077	T:0.0769231		
chr28	10556310	2	34	T:0.970588	TAC:0.0294118		
chr28	10557662	2	40	AAAC:1	A:0		
chr28	10559647	2	38	CGCCACCAT:0.868421	C:0.131579		
chr28	10560511	2	36	T:0.944444	TA:0.0555556		
chr28	10561385	2	40	G:1	A:0		
chr28	10561624	2	38	G:1	A:0		
chr28	10561781	2	22	T:0.818182	TA:0.181818		
chr28	10561958	2	16	GAGGA:0.875	G:0.125		
chr28	10562746	3	36	CCT:0.694444	C:0.305556	CCTCT:0	
chr28	10562865	2	40	C:0.95	G:0.05		
chr28	10563588	2	36	C:0.916667	T:0.0833333		
chr28	10563736	2	40	G:0.9	A:0.1		
chr28	10563944	2	38	CCTCT:0.736842	C:0.263158		
chr28	10563995	2	38	TA:0.894737	T:0.105263		
chr28	10563996	2	40	A:0.975	T:0.025		
chr28	10565183	2	40	C:0.95	T:0.05		
chr28	10565409	2	34	TA:0.617647	T:0.382353		
chr28	10565818	2	40	T:0.7	G:0.3		
chr28	10566177	2	38	GT:0.921053	G:0.0789474		
chr28	10566240	2	40	GGGAAGAAA:0.925	G:0.075		
chr28	10566893	2	36	T:0.916667	C:0.0833333		
chr28	10567203	2	38	G:1	T:0		
chr28	10567759	2	40	ACAGGCTAACAACTG:0.8	A:0.2		
chr28	10568250	2	38	T:0.815789	TA:0.184211		
chr28	10568591	2	36	G:1	A:0		
chr28	10568623	2	40	A:0.85	G:0.15		
chr28	10569503	2	38	A:0.894737	G:0.105263		
chr28	10569890	2	38	A:0.842105	T:0.157895		
chr28	10569998	2	36	CT:1	C:0		
chr28	10571008	2	36	T:0.888889	C:0.111111		
chr28	10571845	2	40	T:0.85	C:0.15		
chr28	10572371	2	38	A:0.763158	T:0.236842		
chr28	10573035	2	40	C:0.9	CAAGAA:0.1		
chr28	10573367	2	36	T:0.916667	C:0.0833333		
chr28	10573672	2	38	C:0.894737	A:0.105263		

chr28	10574227	2	36	G:1	A:0	
chr28	10574663	2	40	G:0.9	A:0.1	
chr28	10574717	2	38	C:0.894737	G:0.105263	
chr28	10574739	2	38	A:1	AT:0	
chr28	10574749	2	38	A:1	AT:0	
chr28	10574760	2	38	A:1	AG:0	
chr28	10575007	2	40	A:0.85	G:0.15	
chr28	10575338	2	34	C:0.264706	CT:0.735294	
chr28	10575340	2	36	T:0.972222	C:0.0277778	
chr28	10575737	2	36	G:1	A:0	
chr28	10576071	3	40	T:0.45	A:0.075	TA:0.475
chr28	10576074	2	40	A:0.925	AT:0.075	
chr28	10576100	2	40	A:0.975	T:0.025	
chr28	10576262	2	34	C:0.911765	CT:0.0882353	
chr28	10576306	2	40	C:0.95	CAGAG:0.05	
chr28	10576365	2	40	C:1	T:0	
chr28	10576729	2	38	C:0.894737	T:0.105263	
chr28	10576852	2	38	T:0.894737	C:0.105263	
chr28	10576884	2	38	C:0.815789	CA:0.184211	
chr28	10576892	3	38	A:1	T:0	AT:0
chr28	10576911	2	38	AAAAT:1	A:0	
chr28	10576941	2	38	G:1	A:0	
chr28	10577125	2	36	T:0.916667	TA:0.0833333	
chr28	10577126	2	36	T:0.833333	A:0.166667	
chr28	10577379	2	34	G:1	A:0	
chr28	10577506	2	32	TA:0.78125	T:0.21875	
chr28	10577805	3	38	C:0.815789	CCT:0.157895	CCTCT:0.0263158
chr28	10577855	2	40	AAAATAAAT:0.85	A:0.15	
chr28	10578996	2	40	T:0.9	TAAC:0.1	
chr28	10579062	2	40	AAG:0.85	A:0.15	
chr28	10580462	2	32	C:1	CT:0	
chr28	10580464	2	34	TA:0.882353	T:0.117647	
chr28	10581386	2	38	GA:0.763158	G:0.236842	
chr28	10581895	2	36	C:0.888889	T:0.111111	
chr28	10581982	2	40	A:1	T:0	
chr28	10582211	2	40	T:1	C:0	
chr28	10582988	2	40	C:1	T:0	
chr28	10583824	3	38	CA:0.684211	C:0.131579	CAAA:0.184211
chr28	10584077	2	36	C:0.861111	A:0.138889	
chr28	10584152	2	40	CCT:0.7	C:0.3	
chr28	10584919	2	40	A:0.9	G:0.1	
chr28	10585831	2	40	AT:0.925	A:0.075	
chr28	10586056	2	38	T:0.710526	A:0.289474	
chr28	10586531	2	40	T:0.875	C:0.125	
chr28	10586537	2	40	G:1	A:0	
chr28	10587009	2	38	A:1	AAATG:0	
chr28	10588186	2	36	C:0.527778	CA:0.472222	
chr28	10588731	2	36	C:0.861111	T:0.138889	
chr28	10589102	2	38	T:0.763158	TA:0.236842	
chr28	10590953	2	40	A:0.875	G:0.125	
chr28	10591396	2	40	G:0.9	GA:0.1	
chr28	10591455	2	40	T:0.95	A:0.05	

chr28	10591516	2	36	TG:0.888889	T:0.111111	
chr28	10592814	2	34	TA:0.558824	T:0.441176	
chr28	10592845	2	38	A:0.894737	G:0.105263	
chr28	10593145	2	38	CA:0.894737	C:0.105263	
chr28	10594082	2	40	TA:1	T:0	
chr28	10594538	2	34	G:0.882353	GA:0.117647	
chr28	10594595	2	40	T:0.85	C:0.15	
chr28	10594959	2	38	T:0.605263	G:0.394737	
chr28	10595189	2	40	T:0.925	A:0.075	
chr28	10595758	3	40	G:0.925	GAATA:0.075	GAATAAATA:0
chr28	10596022	3	30	CTT:0.766667	C:0.1	CT:0.133333
chr28	10596068	2	40	T:0.875	TAAAG:0.125	
chr28	10596279	2	40	CCTCTCTCTCTCT:0.75	C:0.25	
chr28	10596340	2	38	TA:1	T:0	
chr28	10596511	2	40	T:1	G:0	
chr28	10596926	2	40	C:1	T:0	
chr28	10596936	2	40	G:1	A:0	
chr28	10598903	2	36	A:0.888889	G:0.111111	
chr28	10599477	2	34	G:0.941176	A:0.0588235	
chr28	10600160	2	38	TTTG:0.921053	T:0.0789474	
chr28	10600172	2	40	G:1	T:0	
chr28	10600191	2	40	C:1	CAGAG:0	
chr28	10600289	2	38	A:0.894737	G:0.105263	
chr28	10600456	2	40	AT:0.9	A:0.1	
chr28	10600860	2	40	A:0.875	C:0.125	
chr28	10601025	2	40	A:0.9	G:0.1	
chr28	10601393	2	38	C:1	T:0	
chr28	10601547	3	38	TA:0.578947	T:0.315789	AA:0.105263
chr28	10601552	2	38	A:0.947368	T:0.0526316	
chr28	10601895	2	36	C:0.833333	T:0.166667	
chr28	10602230	2	38	A:0.894737	G:0.105263	
chr28	10602417	2	36	A:0.861111	G:0.138889	
chr28	10602631	2	34	A:0	T:1	
chr28	10602632	2	34	A:0	T:1	
chr28	10604178	2	36	A:1	T:0	
chr28	10604330	2	38	TA:0.315789	T:0.684211	
chr28	10606620	2	38	T:0.842105	A:0.157895	
chr28	10607017	2	36	C:0.944444	CCT:0.0555556	
chr28	10607070	2	40	T:0.775	TA:0.225	
chr28	10607627	2	40	A:0.925	AGGCTCCT:0.075	
chr28	10608406	2	36	A:0.555556	AGATT:0.444444	
chr28	10609257	2	36	T:1	TA:0	
chr28	10609343	2	38	T:1	C:0	
chr28	10609455	2	34	G:0.882353	T:0.117647	
chr28	10609610	2	38	C:0.842105	T:0.157895	
chr28	10610000	2	40	CTTAGGATGTTTCAGTTTCTTTTTTTTTTTTTTTTTTTTTTTTTTTTTTTTTTTTTTTTTTTTTTTTTTTT:0	C:1	
chr28	10610363	2	34	CT:0.852941	C:0.147059	
chr28	10610502	2	34	G:0.852941	A:0.147059	
chr28	10610893	2	38	G:1	C:0	

chr28	10611159	2	40	T:0.925	C:0.075		
chr28	10612139	2	32	C:1	T:0		
chr28	10612291	2	22	T:0.454545	TA:0.545455		
chr28	10612743	2	36	C:1	T:0		
chr28	10612925	2	38	A:0.973684	G:0.0263158		
chr28	10612928	2	38	A:0.947368	G:0.0526316		
chr28	10613580	2	36	T:0.916667	C:0.0833333		
chr28	10613752	2	36	CCT:0.833333	C:0.166667		
chr28	10613808	2	40	AT:0.9	A:0.1		
chr28	10613812	2	40	T:1	A:0		
chr28	10614272	2	32	A:1	C:0		
chr28	10614821	2	40	G:0.9	T:0.1		
chr28	10615196	3	38	ATT:0.815789	A:0	AT:0.184211	
chr28	10615512	2	36	T:0.916667	G:0.0833333		
chr28	10617162	4	40	GATTTATTT:0.275	G:0.075	GATTT:0.325	GATTTATTTATTT:0.325
chr28	10617438	2	40	G:1	A:0		
chr28	10617682	2	34	C:0.882353	CA:0.117647		
chr28	10617699	2	34	C:0	T:1		
chr28	10617780	2	40	G:0	A:1		
chr28	10617826	2	38	G:0	A:1		
chr28	10617832	2	38	CT:0	C:1		
chr28	10617839	2	38	T:0	A:1		
chr28	10617854	2	38	C:0	A:1		
chr28	10617869	2	36	C:0	A:1		
chr28	10617903	2	40	T:0	TA:1		
chr28	10617918	2	40	A:0	T:1		
chr28	10617919	2	40	T:0	A:1		
chr28	10617927	2	40	A:0	AT:1		
chr28	10617929	2	40	A:0	T:1		
chr28	10617963	2	40	TG:0	T:1		
chr28	10617977	2	40	GA:0	G:1		
chr28	10618000	2	40	G:0	A:1		
chr28	10618010	2	40	A:0	AC:1		
chr28	10618031	2	40	T:0	C:1		
chr28	10618032	2	40	T:0	C:1		
chr28	10618246	2	22	CTA:0.909091	C:0.0909091		
chr28	10618248	2	22	A:0.0909091	C:0.909091		
chr28	10618872	2	40	T:1	G:0		
chr28	10618890	2	40	A:0.9	G:0.1		
chr28	10619117	2	40	A:1	T:0		
chr28	10619145	3	40	CAGAGAG:0.825	C:0.05	CAG:0.125	
chr28	10619482	2	36	A:0.833333	G:0.166667		
chr28	10619486	2	36	C:0.833333	CAG:0.166667		
chr28	10619721	2	40	GTGGC:0.825	G:0.175		
chr28	10619871	2	34	GCT:0.970588	G:0.0294118		
chr28	10621164	2	38	G:0.263158	T:0.736842		
chr28	10621953	2	32	C:0.25	T:0.75		
chr28	10621964	2	34	A:0.323529	G:0.676471		
chr28	10622622	2	38	GC:0.526316	G:0.473684		
chr28	10622643	2	38	G:0.526316	GTGA:0.473684		
chr28	10622647	2	38	TAGG:0.526316	T:0.473684		

chr28	10622771	2	40	T:0.95	TAAA:0.05		
chr28	10622878	2	40	G:0.775	A:0.225		
chr28	10622883	2	40	T:0.775	C:0.225		
chr28	10622935	2	40	T:0.75	G:0.25		
chr28	10623376	2	40	C:1	T:0		
chr28	10624357	2	34	G:0.823529	T:0.176471		
chr28	10624425	2	40	C:0.925	A:0.075		
chr28	10625989	2	38	T:0.684211	A:0.315789		
chr28	10626000	2	36	T:0.722222	C:0.277778		
chr28	10626353	2	40	A:0.95	T:0.05		
chr28	10626516	2	38	A:0.684211	G:0.315789		
chr28	10627065	2	34	T:1	C:0		
chr28	10627402	2	38	A:0.236842	G:0.763158		
chr28	10627470	2	38	A:0.815789	G:0.184211		
chr28	10627479	2	38	A:0.815789	C:0.184211		
chr28	10627673	2	40	A:0.75	G:0.25		
chr28	10627837	2	40	G:0.25	A:0.75		
chr28	10628053	4	38	TAA:0.236842	T:0.157895	TA:0.526316	TAAA:0.0789474
chr28	10628054	2	38	A:1	C:0		
chr28	10628554	2	38	T:0.289474	C:0.710526		
chr28	10628556	2	38	C:0.289474	G:0.710526		
chr28	10628630	2	36	A:1	G:0		
chr28	10628686	2	34	C:0.235294	T:0.764706		
chr28	10629369	2	40	A:0.725	G:0.275		
chr28	10629607	2	40	C:1	T:0		
chr28	10629969	2	40	GGAGAGAGAGAGAGAGA:0.725	G:0.275		
chr28	10630008	2	40	CAGAGA:0.725	C:0.275		
chr28	10630016	2	40	C:0.725	CT:0.275		
chr28	10630026	2	40	GAGAAGCAGGCTCCATGCAGGGA:0.725	G:0.275		
chr28	10630251	2	38	T:0.842105	C:0.157895		
chr28	10630452	2	38	G:0.947368	T:0.0526316		
chr28	10630515	2	36	C:0.25	T:0.75		
chr28	10630780	2	40	T:0.25	G:0.75		
chr28	10630830	2	36	A:0.805556	AT:0.194444		
chr28	10630953	2	36	G:0.833333	T:0.166667		
chr28	10630963	2	36	C:0.972222	T:0.0277778		
chr28	10631066	2	40	T:0	C:1		
chr28	10631096	2	38	C:0	T:1		
chr28	10631098	2	38	G:0	T:1		
chr28	10631733	2	38	T:0	C:1		
chr28	10631854	2	38	C:0.105263	A:0.894737		
chr28	10632105	2	36	A:0.277778	G:0.722222		
chr28	10632233	2	40	T:0.975	G:0.025		
chr28	10632242	2	40	A:0.25	G:0.75		
chr28	10632248	2	40	T:1	C:0		
chr28	10632600	2	40	T:1	C:0		
chr28	10632617	2	38	A:0.789474	G:0.210526		
chr28	10632699	2	40	T:0.775	C:0.225		
chr28	10632728	2	38	G:0.921053	C:0.0789474		
chr28	10632911	2	34	AT:0.617647	A:0.382353		
chr28	10633163	2	36	G:0	C:1		
chr28	10633165	2	36	C:0	A:1		

chr28	10633202	2	38	ATGTC:1	A:0	
chr28	10633728	2	38	A:0.894737	AT:0.105263	
chr28	10633784	2	36	A:0.861111	G:0.138889	
chr28	10633815	2	36	A:0.888889	T:0.111111	
chr28	10633825	2	36	G:0.888889	C:0.111111	
chr28	10633919	2	38	T:0.105263	A:0.894737	
chr28	10633945	2	38	TA:0.894737	T:0.105263	
chr28	10634021	2	36	C:0.888889	CTCCA:0.111111	
chr28	10634257	2	40	TTAAAAAAAAAAA:0.95	T:0.05	
chr28	10634258	3	40	TAAAAAAAA:0.975	TAAAA:0	T:0.025
chr28	10634262	2	40	AAAAAAAAT:1	A:0	
chr28	10634264	2	40	A:0.725	AATATATTC:0.275	
chr28	10634266	3	40	A:0.725	T:0.275	AAAAT:0
chr28	10634268	2	40	A:0.725	T:0.275	
chr28	10634269	2	38	AT:1	A:0	
chr28	10634270	3	40	T:0.725	TTC:0.225	TTCAA: 0.05
chr28	10634271	2	40	AAATAAAT:1	A:0	
chr28	10634272	3	40	AAT:0.775	TAT:0	A:0.225
chr28	10634274	2	38	TAAA:1	T:0	
chr28	10634279	2	38	A:1	C:0	
chr28	10634281	2	38	ATAAAT:1	A:0	
chr28	10634487	2	38	A:0.263158	G:0.736842	
chr28	10634607	2	36	AAAG:1	A:0	
chr28	10634747	2	40	C:0.275	CA:0.725	
chr28	10634817	2	38	G:0.157895	C:0.842105	
chr28	10634840	2	36	T:0.194444	C:0.805556	
chr28	10635490	2	24	C:0.916667	A:0.0833333	
chr28	10636541	2	32	T:0.03125	G:0.96875	
chr28	10636814	2	36	C:0.944444	CCT:0.0555556	
chr28	10637002	2	38	A:0.105263	G:0.894737	
chr28	10637235	2	38	TG:0.894737	T:0.105263	
chr28	10637237	2	38	G:1	T:0	
chr28	10638364	2	40	G:0.95	GT:0.05	
chr28	10638365	2	40	G:0.95	T:0.05	
chr28	10638401	2	40	G:0.85	A:0.15	
chr28	10638429	2	40	C:0.95	T:0.05	
chr28	10638694	2	38	G:0.921053	A:0.0789474	
chr28	10638697	2	38	T:0.973684	C:0.0263158	
chr28	10638806	2	36	T:0.777778	C:0.222222	
chr28	10638899	2	38	T:0.710526	A:0.289474	
chr28	10639109	2	38	A:1	G:0	
chr28	10639164	2	40	T:0.05	C:0.95	
chr28	10639509	2	40	GT:1	G:0	
chr28	10639510	3	40	T:0.95	TGG:0.05	TGGG:0
chr28	10639511	2	40	GT:0.875	G:0.125	
chr28	10639512	2	40	T:0.875	G:0.125	
chr28	10639567	3	38	G:0.736842	GA:0.210526	GAA:0.0526316
chr28	10639837	2	38	C:0.210526	T:0.789474	
chr28	10639951	3	38	C:0.473684	CCT:0.342105	CCTCTCT:0.184211
chr28	10640000	2	36	AT:0.916667	A:0.0833333	
chr28	10640010	2	36	A:0.777778	T:0.222222	

chr28	10640011	2	36	T:0.777778	A:0.222222		
chr28	10640012	2	36	A:0.916667	T:0.0833333		
chr28	10640013	2	36	A:0.916667	T:0.0833333		
chr28	10640083	2	40	G:0	C:1		
chr28	10640091	2	40	G:0	C:1		
chr28	10640200	2	40	T:0.925	A:0.075		
chr28	10640201	2	40	T:0.8	A:0.2		
chr28	10640230	2	38	C:0.868421	CAG:0.131579		
chr28	10640250	2	38	G:0.210526	GA:0.789474		
chr28	10640251	2	38	G:0.210526	GA:0.789474		
chr28	10640316	2	38	C:0.0526316	G:0.947368		
chr28	10640330	2	40	C:1	T:0		
chr28	10640350	2	38	C:0.184211	T:0.815789		
chr28	10640363	2	38	C:1	T:0		
chr28	10640400	2	38	G:0.131579	GA:0.868421		
chr28	10640523	3	40	CTTT:0	C:0.95	CTT:0.05	
chr28	10640567	2	40	T:0.275	A:0.725		
chr28	10640568	2	40	A:0.975	T:0.025		
chr28	10640569	2	40	A:0.975	T:0.025		
chr28	10641158	2	36	A:0.972222	C:0.0277778		
chr28	10641161	2	36	GA:0	G:1		
chr28	10641648	2	38	T:0.263158	G:0.736842		
chr28	10642984	2	38	G:0.289474	A:0.710526		
chr28	10643363	2	30	C:0.0666667	G:0.933333		
chr28	10643973	2	40	T:0.65	C:0.35		
chr28	10644364	2	36	C:0.777778	T:0.222222		
chr28	10644502	2	36	T:0.805556	C:0.194444		
chr28	10644704	2	38	G:0.789474	A:0.210526		
chr28	10644856	2	38	C:0.763158	CCTCT:0.236842		
chr28	10644871	2	38	CTGTG:0.973684	C:0.0263158		
chr28	10644873	2	38	G:0.763158	C:0.236842		
chr28	10644911	2	38	TAAAA:0.289474	T:0.710526		
chr28	10644941	2	40	T:0.05	C:0.95		
chr28	10645306	2	34	T:0.764706	G:0.235294		
chr28	10645376	2	38	C:0.921053	T:0.0789474		
chr28	10645404	2	40	G:0.725	C:0.275		
chr28	10645750	2	30	C:0.9	T:0.1		
chr28	10645875	2	40	A:0.925	AT:0.075		
chr28	10645877	2	38	A:0.710526	T:0.289474		
chr28	10645886	2	40	AAAAATAAAAT:0.95	A:0.05		
chr28	10645933	2	40	A:0.925	T:0.075		
chr28	10646181	2	40	C:0.975	T:0.025		
chr28	10646700	2	40	C:0.7	A:0.3		
chr28	10646902	2	38	G:0.842105	A:0.157895		
chr28	10646921	2	38	T:0.947368	C:0.0526316		
chr28	10646983	2	38	G:0.789474	C:0.210526		
chr28	10647487	2	40	G:0.275	GT:0.725		
chr28	10647679	2	36	C:0.805556	T:0.194444		
chr28	10647999	2	38	G:1	A:0		
chr28	10648322	4	18	TGGCGGCGGCGGCGGC:0.444444	T:0.388889	TGGCGGC:0	TGGCGGCGGCGGC:0.166667
chr28	10648328	2	16	C:1	T:0		
chr28	10648331	2	16	C:0.8125	T:0.1875		
chr28	10648334	2	16	C:1	T:0		

chr28	10648337	2	12	C:0.75	T:0.25	
chr28	10650449	2	38	GAC:0.815789	G:0.184211	
chr28	10650524	2	38	G:1	A:0	
chr28	10651103	2	34	GT:0.617647	G:0.382353	
chr28	10651360	2	40	T:0.95	TTTGATGCGTCTG:0.05	
chr28	10653423	3	34	TTC:0.823529	T:0.147059	TTCTC:0.0294118
chr28	10654846	2	38	C:1	CCTCT:0	
chr28	10654982	2	40	C:0.825	T:0.175	
chr28	10655254	2	40	C:1	A:0	
chr28	10658845	2	38	G:1	A:0	
chr28	10660157	2	32	G:0.5625	GT:0.4375	
chr28	10660205	2	40	A:0.925	G:0.075	
chr28	10660330	2	38	C:0.868421	T:0.131579	
chr28	10660506	2	38	G:0.842105	GTC:0.157895	
chr28	10660516	3	38	C:1	G:0	CTG:0
chr28	10660985	2	38	TA:1	T:0	
chr28	10661219	2	34	C:0.735294	CT:0.264706	
chr28	10663314	3	40	CT:0.7	C:0.125	CTT:0.175
chr28	10664419	2	38	C:0.947368	CT:0.0526316	
chr28	10664431	2	40	A:0.85	AT:0.15	
chr28	10664460	2	38	C:0.763158	CAG:0.236842	
chr28	10664886	2	40	C:0.7	T:0.3	
chr28	10666011	2	40	A:0.975	T:0.025	
chr28	10667082	2	40	G:1	A:0	
chr28	10668639	3	32	GAAA:0.3125	G:0.25	GA:0.4375
chr28	10668690	2	28	TC:0.142857	T:0.857143	
chr28	10670738	2	38	C:0.973684	T:0.0263158	
chr28	10673042	2	40	T:1	G:0	
chr28	10674395	2	40	C:1	T:0	
chr28	10674646	2	38	G:1	A:0	
chr28	10675012	2	34	A:0.970588	G:0.0294118	
chr28	10675344	2	38	G:1	A:0	
chr28	10675556	2	40	T:1	C:0	
chr28	10676284	2	38	A:0.736842	G:0.263158	
chr28	10676356	2	40	C:1	A:0	
chr28	10676947	2	40	TCTGTTTTTGCC:0.975	T:0.025	
chr28	10677728	2	32	C:1	T:0	
chr28	10677902	2	40	T:0.775	C:0.225	
chr28	10682717	2	36	A:1	AC:0	
chr28	10685522	2	38	C:0.815789	T:0.184211	
chr28	10686538	2	36	G:1	A:0	
chr28	10689002	2	38	CTATCATT:0	C:1	
chr28	10689011	2	38	CAACACCAGAAATACTTGTACAAGCTT:0	C:1	
chr28	10689041	2	38	CAAAAG:0	C:1	
chr28	10689052	2	38	TGTAGTCTGTCTAC:0	T:1	
chr28	10689067	2	38	AAATTTCTGACATCAG:0	A:1	
chr28	10689084	2	38	CCCAAAGGAGAA:0	C:1	
chr28	10689096	2	38	T:0	TTG:1	
chr28	10690263	2	36	C:1	T:0	
chr28	10690732	2	38	T:0.921053	A:0.0789474	
chr28	10691590	2	38	C:1	A:0	

chr28	10692902	2	40	A:0.8	G:0.2
chr28	10692987	2	38	T:0.789474	C:0.210526
chr28	10693211	2	34	G:0.558824	GA:0.441176
chr28	10694061	2	40	T:0.825	C:0.175
chr28	10694176	2	40	G:0.9	A:0.1
chr28	10694780	2	36	T:0.777778	C:0.222222
chr28	10694786	2	36	C:1	T:0
chr28	10696593	2	40	G:0.825	A:0.175
chr28	10696929	2	40	T:1	C:0
chr28	10696974	2	40	C:1	T:0
chr28	10697088	2	40	C:1	T:0
chr28	10698154	2	36	G:0.777778	A:0.222222
chr28	10698493	2	34	C:0.323529	CT:0.676471
chr28	10699540	2	40	CCTCT:0.975	C:0.025
chr28	10699735	2	40	A:0.825	G:0.175
chr28	10699869	2	38	T:0.710526	C:0.289474
chr28	10700188	2	36	T:0.805556	A:0.194444
chr28	10700658	2	36	G:0.805556	T:0.194444
chr28	10701197	2	36	AG:0.805556	A:0.194444
chr28	10701214	2	38	A:0.815789	G:0.184211
chr28	10701258	2	38	A:1	G:0
chr28	10701341	2	36	T:0.833333	A:0.166667
chr28	10701625	2	40	A:0.85	G:0.15
chr28	10702509	2	38	CTTTCTTTTCT:0.842105	C:
0.157895					
chr28	10702592	2	38	G:0.868421	GACAC:0.131579
chr28	10702654	2	34	T:0.823529	C:0.176471
chr28	10703393	2	40	A:0.85	G:0.15
chr28	10703679	2	34	C:1	T:0
chr28	10703918	2	38	C:1	T:0
chr28	10704612	2	40	CT:0.775	C:0.225
chr28	10705081	2	40	G:0.75	A:0.25
chr28	10705509	2	38	A:0.763158	T:0.236842
chr28	10705881	2	38	T:0.868421	TA:0.131579
chr28	10706231	2	40	A:0.8	AT:0.2
chr28	10706893	2	40	C:0.725	T:0.275
chr28	10707391	2	40	C:0.725	CAGAG:0.275
chr28	10707514	2	34	T:0.735294	C:0.264706
chr28	10707996	2	38	G:0.815789	C:0.184211
chr28	10708030	2	34	G:0.764706	GA:0.235294
chr28	10708233	2	38	C:0.815789	T:0.184211
chr28	10708260	2	38	T:0.815789	G:0.184211
chr28	10708964	2	40	A:0.775	G:0.225
chr28	10709326	2	38	A:0.842105	G:0.157895
chr28	10709441	2	38	C:0.815789	T:0.184211
chr28	10710218	2	24	ATT:0.791667	A:0.208333
chr28	10710293	2	38	A:0.789474	T:0.210526
chr28	10710621	2	36	A:0	G:1
chr28	10710791	2	34	GAC:0.882353	G:0.117647
chr28	10711014	2	38	T:0.842105	C:0.157895
chr28	10711234	3	36	AT:0.777778	A:0.0555556
ATT:0.166667					
chr28	10711887	2	38	C:0.684211	T:0.315789
chr28	10712016	2	40	T:0.775	C:0.225

chr28 10712106 2 38 T:0.973684 G:0.0263158
chr28 10712617 2 38 AT:0.0263158 A:0.973684
chr28 10712647 2 32 C:0.75 CAG:0.25
chr28 10713398 2 34 G:0.735294 A:0.264706
chr28 10713453 2 32 A:1 G:0
chr28 10713526 2 36 G:1 C:0
chr28 10713788 2 38 ATCACC:0.842105 A:0.157895
chr28 10713889 2 40 T:0.825 A:0.175
chr28 10714028 2 40 T:0.925 C:0.075
chr28 10714302 2 40 T:0.8 G:0.2
chr28 10714438 2 38 C:0.815789 T:0.184211
chr28 10715462 2 38 C:0.842105 A:0.157895
chr28 10716192 2 26 C:0.692308 CT:0.307692
chr28 10716452 2 28 T:0.785714 C:0.214286
chr28 10716626 3 34 T:0.705882 TA:0.264706
TAA:0.0294118
chr28 10717050 2 40 G:0.975 A:0.025
chr28 10717141 2 38 C:1 A:0
chr28 10717190 3 38 GA:0.684211 G:0.131579
GAA:0.184211
chr28 10717334 2 38 C:0.763158 G:0.236842
chr28 10717644 2 40 T:0 G:1
chr28 10717691 2 40 A:0.925 G:0.075
chr28 10717743 2 34 TA:0.823529 T:0.176471
chr28 10717873 2 38 T:0.815789 C:0.184211
chr28 10719409 2 40 G:0.85 A:0.15
chr28 10719846 2 34 G:0.970588 T:0.0294118
chr28 10719852 2 34 TC:0.735294 T:0.264706
chr28 10719870 2 34 A:0.823529 C:0.176471
chr28 10721042 2 38 T:0.842105 C:0.157895
chr28 10721098 2 40 T:1 C:0
chr28 10722029 2 40 AAGC:1 A:0
chr28 10722105 2 38 C:0.815789 CTT:0.184211
chr28 10722278 2 38 A:0.763158 G:0.236842
chr28 10722378 2 40 T:0.825 G:0.175
chr28 10722820 2 40 G:0.775 T:0.225
chr28 10723336 2 38 A:0.789474 T:0.210526
chr28 10723380 2 38 G:0.789474 A:0.210526
chr28 10723459 2 26 ATT:0.923077 A:0.0769231
chr28 10723462 2 26 T:1 TA:0
chr28 10723470 2 26 T:1 A:0
chr28 10723992 2 40 CCTCTCT:0.8 C:0.2
chr28 10724046 2 40 AG:0.875 A:0.125
chr28 10724099 2 40 A:0.7 G:0.3
chr28 10724369 2 40 A:0.8 C:0.2
chr28 10724901 2 40 A:0.775 G:0.225
chr28 10725057 2 38 CT:0.710526 C:0.289474
chr28 10725067 2 34 TTTTTTTTC:0.941176 T:
0.0588235
chr28 10725068 2 34 TTTTTTTC:0.764706 T:
0.235294
chr28 10725642 3 34 CTTT:0.705882 C:0.0882353
CTTTT:0.205882
chr28 10725688 2 36 CAG:0.805556 C:0.194444

chr28	10725754	2	38	G:1	A:0	
chr28	10726592	2	38	T:0.789474	C:0.210526	
chr28	10727319	2	34	CA:0.794118	C:0.205882	
chr28	10727335	2	32	G:0.75	GTA:0.25	
chr28	10727645	2	38	C:0.763158	A:0.236842	
chr28	10728488	2	36	G:0.805556	A:0.194444	
chr28	10728500	2	32	GT:0.8125	G:0.1875	
chr28	10730049	2	36	C:0.861111	T:0.138889	
chr28	10730584	2	38	A:0.815789	C:0.184211	
chr28	10731280	2	40	T:0.8	C:0.2	
chr28	10731398	2	38	A:0.868421	G:0.131579	
chr28	10732073	2	40	C:0.8	T:0.2	
chr28	10732241	2	38	T:0.763158	TAAATC:0.236842	
chr28	10732717	2	38	G:0.736842	GA:0.263158	
chr28	10733060	2	40	A:0.275	T:0.725	
chr28	10733105	2	40	A:0.775	G:0.225	
chr28	10733286	2	32	A:0.90625	G:0.09375	
chr28	10733508	2	38	C:0.842105	T:0.157895	
chr28	10733562	2	32	CTT:0.6875	C:0.3125	
chr28	10733613	2	24	C:0.916667	CAG:0.0833333	
chr28	10733971	2	32	AT:0.875	A:0.125	
chr28	10733972	2	32	T:0.9375	A:0.0625	
chr28	10733977	2	32	A:0.8125	T:0.1875	
chr28	10734120	2	34	A:0.823529	G:0.176471	
chr28	10734164	2	30	A:0.9	G:0.1	
chr28	10734177	2	36	T:0.777778	TA:0.222222	
chr28	10734403	2	36	AAAGGC:1	A:0	
chr28	10734434	2	34	G:1	A:0	
chr28	10734596	2	34	G:0.735294	A:0.264706	
chr28	10735011	2	40	T:0.85	C:0.15	
chr28	10735131	2	40	G:0.75	A:0.25	
chr28	10735165	2	40	CT:0.7	C:0.3	
chr28	10735204	3	40	C:0.9	CAG:0	CAGAG:0.1
chr28	10735206	3	40	C:0.85	G:0.15	CAGAG:0
chr28	10735781	2	40	A:0.975	T:0.025	
chr28	10736087	2	38	C:1	T:0	
chr28	10737303	2	40	G:1	C:0	
chr28	10737520	2	38	A:0.842105	G:0.157895	
chr28	10739199	2	34	G:0.941176	A:0.0588235	
chr28	10739353	2	38	G:0.947368	A:0.0526316	
chr28	10739553	3	38	AG:0.789474	A:0.0526316	AGG:0.157895
chr28	10739597	2	38	C:0.789474	T:0.210526	
chr28	10740077	2	38	C:0.947368	T:0.0526316	
chr28	10740145	2	34	C:0.735294	CA:0.264706	
chr28	10740147	2	38	A:0.657895	AT:0.342105	
chr28	10740404	3	36	C:0.75	CT:0.0833333	CTT:0.166667
chr28	10740445	3	38	CAGAGAG:0.710526	C:0.105263	CAGAG:0.184211
chr28	10740947	2	40	T:0.75	C:0.25	
chr28	10741145	2	38	T:1	G:0	
chr28	10741571	2	32	T:0.9375	C:0.0625	
chr28	10741822	2	38	G:1	A:0	

chr28 10742324 2 40 T:0.95 C:0.05
chr28 10742707 2 38 C:0.763158 CT:0.236842
chr28 10742710 2 38 T:0.921053 TAA:0.0789474
chr28 10743089 2 38 C:1 T:0
chr28 10743465 2 38 C:1 T:0
chr28 10744059 2 36 G:0.944444 C:0.0555556
chr28 10744615 2 38 T:0.763158 C:0.236842
chr28 10744908 2 40 G:1 A:0
chr28 10745602 2 36 T:0.972222 C:0.0277778
chr28 10746348 2 36 T:0.75 C:0.25
chr28 10746405 2 38 C:0.973684 T:0.0263158
chr28 10746557 2 38 T:1 G:0
chr28 10747138 2 40 C:1 T:0
chr28 10747901 2 38 A:0.789474 AAAG:0.210526
chr28 10748051 2 38 G:0.894737 A:0.105263
chr28 10748086 2 38 A:0.763158 G:0.236842
chr28 10748125 2 38 G:0.894737 T:0.105263
chr28 10748217 2 30 TA:0.666667 T:0.333333
chr28 10748235 2 38 A:0.842105 T:0.157895
chr28 10748238 2 38 A:0.842105 G:0.157895
chr28 10748313 2 40 TCCTG:1 T:0
chr28 10748702 2 38 C:1 T:0
chr28 10748721 2 36 C:0.944444 T:0.0555556
chr28 10750001 2 40 A:0.85 G:0.15
chr28 10750349 2 36 T:0.555556 C:0.444444
chr28 10750796 2 36 AG:0.888889 A:0.111111
chr28 10750958 2 32 C:0.875 G:0.125
chr28 10750978 3 36 TAAA:0.833333 T:0.111111
TAAAA:0.0555556
chr28 10751008 2 40 A:1 G:0
chr28 10751976 2 38 A:1 G:0
chr28 10752176 2 40 A:0.7 G:0.3
chr28 10753060 2 34 CAT:0.882353 C:0.117647
chr28 10753191 4 40 AAAAAC:0.3 A:0.05
AAAAACAAAAC:0.625 AAAAACAAAACAAAAC:0.025
chr28 10753229 3 40 A:0.95 C:0 AACAAC:0.05
chr28 10753234 2 40 C:0.775 CAAAA:0.225
chr28 10753435 2 36 C:0.916667 A:0.0833333
chr28 10753754 2 38 G:0.973684 A:0.0263158
chr28 10755296 2 40 T:1 C:0
chr28 10755601 2 40 A:0.95 C:0.05
chr28 10756021 2 38 A:0.973684 G:0.0263158
chr28 10756752 2 38 AAAAC:0.605263 A:0.394737
chr28 10757820 2 38 T:0.868421 C:0.131579
chr28 10758996 2 40 G:0.775 A:0.225
chr28 10760078 2 40 A:0.825 AT:0.175
chr28 10760230 2 40 T:0.95 C:0.05
chr28 10761064 2 40 A:0.75 ATAGTT:0.25
chr28 10761196 3 36 CA:0.444444 C:0.277778
CAA:0.277778
chr28 10761567 2 40 A:0.8 ACAGCTATTTTTGTGTCTG:0.2
chr28 10761664 2 36 C:0.833333 T:0.166667
chr28 10761718 2 38 T:1 C:0
chr28 10761997 2 38 AT:0.973684 A:0.0263158

chr28	10762095 2	34	C:0.970588	T:0.0294118
chr28	10762286 4	40	TAAAA:0.475	T:0.2	TAA:0.15
TAAA:0.175
chr28	10762464 2	36	T:0.944444	TATGTC:0.0555556
chr28	10762491 2	36	G:1	C:0
chr28	10762522 2	36	T:0.833333	C:0.166667
chr28	10763694 2	40	G:1	A:0
chr28	10763987 4	40	CAGAG:0.55	C:0.2	CAG:
0.175	CAGAGAG:0.075
chr28	10764143 2	40	T:0.675	C:0.325
chr28	10764367 2	40	C:0.75	T:0.25
chr28	10764591 2	38	T:0.763158	C:0.236842
chr28	10765710 2	36	A:0.777778	C:0.222222
chr28	10765969 2	38	T:0.947368	C:0.0526316
chr28	10766709 4	38	TAG:0.289474	T:0.342105
TAGAG:0.289474	TAGAGAG:0.0789474
chr28	10766801 2	38	C:0.973684	T:0.0263158
chr28	10767588 2	36	A:0.916667	G:0.0833333
chr28	10767617 2	36	CACA:1	C:0
chr28	10767623 2	38	G:0.789474	T:0.210526
chr28	10767629 2	38	C:0.789474	A:0.210526
chr28	10768234 2	40	G:0.95	GT:0.05
chr28	10768246 2	40	T:0.85	A:0.15
chr28	10768247 2	40	A:0.675	T:0.325
chr28	10769125 2	40	AATGT:1	A:0
chr28	10769156 2	40	C:0.725	A:0.275
chr28	10769679 2	40	T:0.975	C:0.025
chr28	10769736 2	38	A:0.973684	G:0.0263158
chr28	10770443 2	40	G:1	C:0
chr28	10770945 2	40	A:0.8	G:0.2
chr28	10771080 2	36	A:0.722222	ACT:0.277778
chr28	10772205 2	36	TA:0.833333	T:0.166667
chr28	10772387 3	38	ATT:0.5	A:0.0526316	AT:
0.447368
chr28	10772437 2	36	CAG:1	C:0
chr28	10772439 2	36	G:0.888889	C:0.111111
chr28	10772859 2	38	C:0.684211	T:0.315789
chr28	10772884 2	38	TA:0.789474	T:0.210526
chr28	10773090 2	36	T:0.416667	C:0.583333
chr28	10773296 2	36	C:1	T:0
chr28	10773321 2	34	C:0.794118	CT:0.205882
chr28	10774112 2	40	CTA:0.95	C:0.05
chr28	10774172 3	40	AAAAC:0.825	A:0.05
AAAACAAAC:0.125
chr28	10774615 2	40	G:0.975	A:0.025
chr28	10774791 2	40	G:0.8	A:0.2
chr28	10774957 2	40	A:0.975	C:0.025
chr28	10775164 2	38	G:0.736842	A:0.263158
chr28	10776442 2	40	T:0.75	C:0.25
chr28	10776469 2	40	AT:0.925	A:0.075
chr28	10776941 2	38	A:0.763158	T:0.236842
chr28	10777379 2	36	C:0.833333	T:0.166667
chr28	10777681 2	38	A:0.921053	AAAAAG:0.0789474
chr28	10777772 2	40	C:0.825	T:0.175

chr28	10777871	2	38	T:0.736842	A:0.263158	
chr28	10778270	2	40	GA:1	G:0	
chr28	10778309	2	38	C:0.710526	T:0.289474	
chr28	10778666	2	36	C:0.944444	T:0.0555556	
chr28	10779001	2	34	C:0.941176	T:0.0588235	
chr28	10779043	2	38	C:0.973684	T:0.0263158	
chr28	10779220	2	36	C:1	CT:0	
chr28	10779294	2	36	C:0.75	T:0.25	
chr28	10779582	2	40	G:0.825	T:0.175	
chr28	10779588	2	40	G:1	T:0	
chr28	10780909	2	38	TA:0.947368	T:0.0526316	
chr28	10780910	3	36	ATT:0.833333	A:0.0555556	AT:0.111111
chr28	10781368	2	36	C:1	T:0	
chr28	10781582	2	40	TTC:1	T:0	
chr28	10781588	2	40	T:1	C:0	
chr28	10782031	2	36	C:0.833333	T:0.166667	
chr28	10782115	2	36	T:0.861111	TA:0.138889	
chr28	10782176	2	36	T:0.694444	TCTG:0.305556	
chr28	10782912	2	34	A:0.735294	G:0.264706	
chr28	10783073	2	28	T:0.964286	TA:0.0357143	
chr28	10783138	2	16	G:1	A:0	
chr28	10783392	2	28	G:0.607143	GC:0.392857	
chr28	10783837	2	36	C:0.833333	T:0.166667	
chr28	10784018	2	30	G:1	T:0	
chr28	10784077	2	18	G:1	C:0	
chr28	10785442	2	36	G:0.888889	A:0.111111	
chr28	10786021	2	30	G:0.933333	A:0.0666667	
chr28	10786049	2	38	T:0.921053	G:0.0789474	
chr28	10786062	2	38	G:0.763158	T:0.236842	
chr28	10786076	2	38	G:0	T:1	
chr28	10786136	2	40	C:0	G:1	
chr28	10786220	2	38	T:0.973684	C:0.0263158	
chr28	10786342	2	36	G:0.944444	A:0.0555556	
chr28	10786343	2	36	G:0.944444	A:0.0555556	
chr28	10786477	2	40	G:0.9	T:0.1	
chr28	10786561	2	38	C:0.842105	T:0.157895	
chr28	10786564	2	38	G:0.894737	T:0.105263	
chr28	10786946	2	40	G:1	A:0	
chr28	10787158	2	40	T:0.975	C:0.025	
chr28	10787239	2	38	CTTTCT:0.421053	C:0.578947	
chr28	10787349	2	24	G:0.25	T:0.75	
chr28	10787915	2	36	GC:0	G:1	
chr28	10788645	2	36	C:0	G:1	
chr28	10788875	2	40	AGCATCCTGCC:0.875	A:0.125	
chr28	10788888	2	40	A:1	G:0	
chr28	10789147	2	40	C:1	T:0	
chr28	10789640	2	40	CA:0.9	C:0.1	
chr28	10789643	2	40	TGGGC:0.9	T:0.1	
chr28	10789652	2	40	C:0.9	T:0.1	
chr28	10789771	2	36	G:0.805556	A:0.194444	
chr28	10789774	2	36	G:0.944444	A:0.0555556	
chr28	10790103	2	38	T:0.710526	C:0.289474	
chr28	10790171	2	36	C:0.944444	T:0.0555556	

chr28	10790236	3	40	A:0.825	AGATG:0	AGATGGATG:0.175	
chr28	10790365	2	38	ATTT:0.789474	A:0.210526		
chr28	10790372	2	40	T:1	TA:0		
chr28	10790396	2	40	G:1	A:0		
chr28	10790601	2	40	G:0.85	C:0.15		
chr28	10790641	2	40	G:0.95	A:0.05		
chr28	10790672	2	38	G:0.921053	A:0.0789474		
chr28	10791027	2	40	A:0.875	G:0.125		
chr28	10791097	2	36	A:0.888889	G:0.111111		
chr28	10791288	2	38	G:0.868421	GTGCTAGAGC:0.131579		
chr28	10791360	2	28	T:0.25	C:0.75		
chr28	10791635	2	40	TAAAAAAA:0.975	T:0.025		
chr28	10791636	2	38	A:1	T:0		
chr28	10791651	2	40	A:0.975	T:0.025		
chr28	10791925	2	40	CT:0.375	C:0.625		
chr28	10791931	2	40	A:0.925	T:0.075		
chr28	10792362	2	40	G:1	A:0		
chr28	10792475	2	38	C:0.263158	T:0.736842		
chr28	10792513	2	38	G:0.894737	A:0.105263		
chr28	10792605	2	40	C:0.95	CATGA:0.05		
chr28	10792682	2	40	G:0.95	A:0.05		
chr28	10792694	4	40	GCACA:0.3	G:0.025	GCA:0.625	GCACACACA:0.05
chr28	10792696	2	40	A:0.975	G:0.025		
chr28	10792698	2	40	A:0.975	G:0.025		
chr28	10792700	2	40	A:1	G:0		
chr28	10792702	2	40	A:1	G:0		
chr28	10792912	2	36	C:0.305556	A:0.694444		
chr28	10792915	2	36	GT:0.944444	G:0.0555556		
chr28	10793232	2	38	G:1	A:0		
chr28	10793344	2	40	G:0.675	GA:0.325		
chr28	10793715	2	36	A:0.25	C:0.75		
chr28	10793717	2	36	A:0.916667	C:0.0833333		
chr28	10793778	2	36	A:1	G:0		
chr28	10794281	2	40	C:0.325	T:0.675		
chr28	10794711	2	38	ACCC:1	A:0		
chr28	10794779	2	38	C:0.842105	T:0.157895		
chr28	10796986	2	40	A:0.275	G:0.725		
chr28	10797230	2	38	A:0.289474	G:0.710526		
chr28	10797858	2	38	AGGGTTGGGGGC:0.368421	A:0.631579		
chr28	10798030	2	38	T:0.289474	C:0.710526		
chr28	10798522	2	38	G:0.368421	A:0.631579		
chr28	10798608	2	34	A:0.147059	C:0.852941		
chr28	10798699	2	36	C:1	T:0		
chr28	10798759	2	40	T:0.6	C:0.4		
chr28	10798910	2	38	TG:0.315789	T:0.684211		
chr28	10798916	2	40	GCACC:0.725	G:0.275		
chr28	10798919	2	38	C:0.315789	CA:0.684211		
chr28	10799285	2	40	C:0.3	A:0.7		
chr28	10799467	2	38	C:0.368421	T:0.631579		
chr28	10799500	2	38	T:0.368421	C:0.631579		
chr28	10799935	2	36	T:0.555556	G:0.444444		

chr28	10800458	2	40	C:0.9	T:0.1
chr28	10800816	2	40	T:1	TAA:0
chr28	10800817	2	40	GCT:1	G:0
chr28	10800836	2	40	G:0.75	A:0.25
chr28	10801389	2	40	G:0.925	T:0.075
chr28	10801683	2	40	G:0.375	A:0.625
chr28	10801900	2	40	G:0.6	A:0.4
chr28	10802217	2	36	AAGAG:0.75	A:0.25
chr28	10802229	2	36	G:0.972222	A:0.0277778
chr28	10803019	2	40	C:0.975	T:0.025
chr28	10803956	2	38	T:1	G:0
chr28	10804116	2	40	G:0.75	A:0.25
chr28	10804198	2	36	CTG:0.555556	C:0.444444
chr28	10804280	2	38	TA:0.763158	T:0.236842
chr28	10805161	2	38	A:0.736842	G:0.263158
chr28	10805332	2	40	T:0.95	TG:0.05
chr28	10805334	2	40	A:0.95	AGACAAAAGAAGGAGACAGC:0.05
chr28	10805337	2	40	GCTCTCTC:0.95	G:0.05
chr28	10805458	2	40	G:0.775	A:0.225
chr28	10805484	2	40	C:1	T:0
chr28	10805708	2	40	A:0.375	T:0.625
chr28	10805938	2	26	C:1	T:0
chr28	10806164	2	30	T:0.666667	C:0.333333
chr28	10807229	2	40	A:1	G:0
chr28	10807334	2	40	G:0.625	A:0.375
chr28	10807952	2	40	CT:1	C:0
chr28	10808716	2	40	G:0.225	T:0.775
chr28	10808723	2	40	C:0.4	CTCTT:0.6
chr28	10808727	2	40	T:1	TTCTTTC:0
chr28	10808788	2	36	G:1	A:0
chr28	10810294	2	40	G:0.875	GAAAAAAAAAAAAAAAAAAA:0.125
chr28	10810296	2	40	A:0.825	AAAATCTGATGAGGT:0.175
chr28	10810503	2	34	G:0.735294	A:0.264706
chr28	10810518	2	36	G:0.777778	T:0.222222
chr28	10810680	2	38	C:0.289474	T:0.710526
chr28	10810910	2	38	T:0.368421	C:0.631579
chr28	10811208	2	38	GT:0.342105	G:0.657895
chr28	10811217	2	38	C:0.342105	T:0.657895
chr28	10811295	2	40	G:0.275	C:0.725
chr28	10811860	2	36	A:0.666667	G:0.333333
chr28	10811995	2	36	G:0.666667	T:0.333333
chr28	10812105	2	38	A:0.894737	G:0.105263
chr28	10812120	2	38	G:0.657895	A:0.342105
chr28	10812280	2	38	T:0.263158	C:0.736842
chr28	10812446	2	38	C:0.342105	T:0.657895
chr28	10812841	2	34	G:0.235294	A:0.764706
chr28	10812961	2	38	A:1	G:0
chr28	10813514	2	34	T:0.470588	A:0.529412
chr28	10814023	2	38	A:0.815789	G:0.184211
chr28	10814099	2	40	AT:0.7	A:0.3
chr28	10814148	2	38	C:0.921053	CT:0.0789474
chr28	10814149	2	38	C:0.447368	T:0.552632
chr28	10814150	3	38	C:0.921053	T:0.0789474

CTT:0
chr28 10814876 2 30 C:0.2 G:0.8
chr28 10815237 2 36 G:0.333333 A:0.666667
chr28 10815616 2 36 G:0.666667 T:0.333333
chr28 10815779 2 40 CG:0.775 C:0.225
chr28 10815825 4 40 CCTCTCTCT:0.225 C:0.45 CCT:0.15
CTTCTCTCTCT:0.175
chr28 10815846 2 34 C:0.823529 G:0.176471
chr28 10815848 2 30 C:0.533333 G:0.466667
chr28 10815896 2 34 T:0.470588 TA:0.529412
chr28 10816356 2 40 A:0.625 G:0.375
chr28 10816447 2 40 G:0.825 A:0.175
chr28 10816779 2 36 G:0.972222 A:0.0277778
chr28 10816995 2 38 G:0.394737 A:0.605263
chr28 10817370 2 36 A:0.75 G:0.25
chr28 10817535 2 34 G:0.676471 T:0.323529
chr28 10818450 2 38 A:0.894737 G:0.105263
chr28 10819367 2 38 A:0.657895 G:0.342105
chr28 10819539 2 36 T:0.638889 C:0.361111
chr28 10819736 2 38 C:0.947368 CG:0.0526316
chr28 10819935 3 32 TA:0.78125 T:0.1875 TAA:
0.03125
chr28 10820648 2 40 C:0.75 T:0.25
chr28 10821105 2 38 A:1 G:0
chr28 10821281 2 34 G:0.470588 A:0.529412
chr28 10821414 2 40 G:0.825 A:0.175
chr28 10821971 2 38 C:1 T:0
chr28 10822368 2 36 T:0.388889 C:0.611111
chr28 10822387 2 36 C:0.361111 T:0.638889
chr28 10823190 2 40 T:0.65 C:0.35
chr28 10823207 2 40 T:0.675 TA:0.325
chr28 10823751 2 40 T:0.45 A:0.55
chr28 10823975 2 36 G:0.888889 A:0.111111
chr28 10824004 2 38 C:0 A:1
chr28 10824116 2 38 T:0.736842 C:0.263158
chr28 10824189 2 34 G:0.617647 A:0.382353
chr28 10824896 2 32 T:0.78125 C:0.21875
chr28 10825145 2 34 A:0.705882 C:0.294118
chr28 10825361 2 34 G:0.970588 A:0.0294118
chr28 10825928 2 38 A:1 G:0
chr28 10826445 2 36 T:0.277778 C:0.722222
chr28 10826488 2 38 C:0.842105 T:0.157895
chr28 10826717 2 38 G:0.263158 A:0.736842
chr28 10827088 2 40 T:0.25 C:0.75
chr28 10827157 2 36 G:0.305556 A:0.694444
chr28 10827382 2 40 T:0.225 C:0.775
chr28 10827768 2 36 CATTT:0.722222 C:0.277778
chr28 10827809 2 34 G:1 A:0
chr28 10828635 2 40 T:0.6 C:0.4
chr28 10829371 2 40 G:0.85 A:0.15
chr28 10830034 2 36 T:0.694444 TA:0.305556
chr28 10830214 2 40 T:0.825 TA:0.175
chr28 10830246 2 40 G:0.825 A:0.175
chr28 10830435 2 36 A:1 C:0

chr28	10830652	2	40	T:0.75	C:0.25	
chr28	10831011	2	38	G:1	A:0	
chr28	10831614	2	38	T:1	G:0	
chr28	10831690	2	36	G:0.75	A:0.25	
chr28	10832068	2	40	C:0.975	T:0.025	
chr28	10832590	2	34	TAAAAA:0.529412	T:0.470588	
chr28	10833107	2	38	C:0.789474	A:0.210526	
chr28	10833406	2	40	G:0.75	A:0.25	
chr28	10833643	2	38	A:0.736842	C:0.263158	
chr28	10833794	2	40	G:1	A:0	
chr28	10833839	3	40	G:0.625	GTTCTTTTCTTTTCTT:0.225	GTTCTTTTCTTTTCTTTTCTT:0.15
chr28	10833871	2	40	T:0.85	TC:0.15	
chr28	10833872	2	40	T:0.85	TTTTCTTTTCTTTTCTTTTC:0.15	
chr28	10834198	2	36	A:0.777778	T:0.222222	
chr28	10834480	2	36	G:1	T:0	
chr28	10835886	2	38	T:0.815789	G:0.184211	
chr28	10836669	2	32	G:0.28125	GC:0.71875	
chr28	10837170	2	34	G:1	A:0	
chr28	10837946	2	38	G:0.947368	T:0.0526316	
chr28	10838357	2	38	G:0.710526	C:0.289474	
chr28	10838463	2	36	T:0.972222	C:0.0277778	
chr28	10838727	2	36	C:0.75	T:0.25	
chr28	10839018	2	34	AG:1	A:0	
chr28	10839579	2	38	C:0.394737	T:0.605263	
chr28	10839669	2	34	A:0.794118	G:0.205882	
chr28	10839670	2	34	T:0.794118	G:0.205882	
chr28	10839780	3	36	TTAAATAAA:0.333333	T:0.361111	TTAAA:0.305556
chr28	10840014	2	36	G:0.277778	C:0.722222	
chr28	10840673	2	40	G:1	A:0	
chr28	10840824	2	38	A:0.0526316	T:0.947368	
chr28	10840924	2	40	C:1	T:0	
chr28	10841198	2	32	G:0.9375	A:0.0625	
chr28	10841364	2	38	T:0.184211	A:0.815789	
chr28	10841596	2	40	T:0.825	C:0.175	
chr28	10842018	2	36	A:0.722222	C:0.277778	
chr28	10842498	2	40	T:0	C:1	
chr28	10843285	2	34	G:1	A:0	
chr28	10843350	2	34	A:0.764706	G:0.235294	
chr28	10843359	2	34	T:0.970588	G:0.0294118	
chr28	10843728	2	38	T:0.657895	C:0.342105	
chr28	10843855	2	40	G:0.7	C:0.3	
chr28	10843924	2	40	T:0.675	C:0.325	
chr28	10843962	2	36	G:0.611111	A:0.388889	
chr28	10844288	2	40	G:0.75	C:0.25	
chr28	10844333	2	40	GACTGAGAGT:0.675	G:0.325	
chr28	10844335	2	40	C:0.325	CT:0.675	
chr28	10844341	2	40	GT:0.325	G:0.675	
chr28	10844570	2	36	A:0.194444	G:0.805556	
chr28	10844616	2	38	A:0.210526	G:0.789474	
chr28	10844668	2	38	CT:0.315789	C:0.684211	
chr28	10844683	3	38	ATT:0.289474	A:0.657895	AT:0.0526316

chr28	10844687	2	38	T:1	A:0
chr28	10844769	2	38	A:0.315789	ACT:0.684211
chr28	10844795	2	34	T:0.264706	C:0.735294
chr28	10844839	2	36	C:0.944444	T:0.0555556
chr28	10844863	2	40	A:0.225	G:0.775
chr28	10844929	2	38	C:0.184211	T:0.815789
chr28	10845417	2	38	C:0.789474	T:0.210526
chr28	10845589	2	34	T:0.705882	C:0.294118
chr28	10846118	3	38	CTT:0.315789	C:0.0789474
CT:0.605263					
chr28	10846650	2	40	T:0.65	TAGG:0.35
chr28	10846675	2	38	C:0.631579	T:0.368421
chr28	10846928	2	32	G:0.5	T:0.5
chr28	10847235	2	40	G:0.825	A:0.175
chr28	10847410	2	38	G:1	C:0
chr28	10847418	2	38	A:0.0526316	G:0.947368
chr28	10847584	2	40	T:0.275	A:0.725
chr28	10847679	2	10	CAG:0.8	C:0.2
chr28	10848111	2	36	G:0.277778	A:0.722222
chr28	10848174	2	36	T:0.194444	C:0.805556
chr28	10848225	2	36	G:0.0833333	A:0.916667
chr28	10848239	2	38	C:0.921053	A:0.0789474
chr28	10848338	2	28	T:1	TA:0
chr28	10848394	2	32	T:0.9375	A:0.0625
chr28	10848395	2	32	T:0.9375	A:0.0625
chr28	10848499	2	22	A:0.272727	T:0.727273
chr28	10849025	2	24	GTTTTT:0	G:1
chr28	10849030	2	24	T:0	TGCCGC:1
chr28	10849039	2	24	T:0.958333	C:0.0416667
chr28	10849065	2	24	GTTT:1	G:0
chr28	10849066	2	20	T:0.05	G:0.95
chr28	10849067	2	20	TTTT:0.05	TCC:0.95
chr28	10849069	2	24	T:1	G:0
chr28	10849070	2	24	T:1	TCC:0
chr28	10849276	2	34	T:0.764706	C:0.235294
chr28	10849368	2	34	C:0.911765	G:0.0882353
chr28	10849447	2	40	G:0.3	A:0.7
chr28	10849747	2	36	C:0.944444	T:0.0555556
chr28	10850041	2	36	A:0.0277778	G:0.972222
chr28	10850060	2	38	T:0.315789	C:0.684211
chr28	10850147	2	40	CACTG:0.325	C:0.675
chr28	10850252	2	40	G:1	A:0
chr28	10850338	2	38	G:0.394737	T:0.605263
chr28	10850349	2	40	G:0.975	C:0.025
chr28	10850419	2	40	T:0.3	C:0.7
chr28	10850681	2	36	T:0.277778	G:0.722222
chr28	10850687	2	36	T:0.277778	C:0.722222
chr28	10850927	2	38	G:1	C:0
chr28	10850955	2	40	A:0	G:1
chr28	10850988	2	38	C:0.0263158	T:0.973684
chr28	10851005	2	38	G:1	A:0
chr28	10851045	2	38	C:0.289474	T:0.710526
chr28	10851749	2	38	TA:0.210526	T:0.789474
chr28	10851825	2	38	T:0.973684	TA:0.0263158

chr28	10851934	2	40	A:0.275	G:0.725		
chr28	10851940	2	40	A:0.75	G:0.25		
chr28	10852366	2	38	A:0.315789	G:0.684211		
chr28	10852426	2	38	T:0.368421	C:0.631579		
chr28	10852547	2	38	G:0.263158	A:0.736842		
chr28	10852562	2	38	C:0.263158	T:0.736842		
chr28	10852702	2	38	C:1	T:0		
chr28	10853084	2	36	C:0.972222	T:0.0277778		
chr28	10853383	2	38	G:1	A:0		
chr28	10853503	2	40	TGAGCCACCAGAGCCACCCAAGCCATCAGAGCTACCCAAGCCATCCGAGCCACCCAAGCCACCA:0.3	T:0.7		
chr28	10853651	2	38	T:0.236842	A:0.763158		
chr28	10853841	2	38	G:0.973684	A:0.0263158		
chr28	10854052	2	38	TCAAGTGGCCC:0.921053	T: 0.0789474		
chr28	10854055	2	38	A:0.342105	T:0.657895		
chr28	10854064	2	38	TGG:0.921053	T:0.0789474		
chr28	10854067	2	38	C:0.921053	A:0.0789474		
chr28	10854492	2	40	G:0	C:1		
chr28	10854518	2	38	T:1	TC:0		
chr28	10854706	2	36	C:0.972222	T:0.0277778		
chr28	10854778	2	38	A:1	G:0		
chr28	10854837	2	40	C:0.975	T:0.025		
chr28	10855201	4	40	TTATCTATCTATC:0.55	T:0.15	TTATCTATC:0.125	TTATCTATCTATCTATC:0.175
chr28	10855616	2	40	A:0.025	G:0.975		
chr28	10855998	2	40	G:0.975	GGA:0.025		
chr28	10856001	2	40	ATCT:0.975	A:0.025		
chr28	10856008	2	40	AAAAAAGTTT:0.975	A:0.025		
chr28	10856018	2	40	CAA:0.975	C:0.025		
chr28	10856024	2	40	GACAAAATTCAAAGTCTGCA:0.975	G:0.025		
chr28	10856047	2	40	AGT:0.975	A:0.025		
chr28	10856051	2	40	TAG:0.975	T:0.025		
chr28	10856054	2	40	T:0.975	TAA:0.025		
chr28	10856060	2	40	T:0.975	A:0.025		
chr28	10856061	2	40	GTT:0.975	G:0.025		
chr28	10856067	2	40	T:0.975	TAA:0.025		
chr28	10856069	2	40	T:0.975	A:0.025		
chr28	10856605	2	38	GC:0.342105	G:0.657895		
chr28	10856834	2	40	G:0.3	A:0.7		
chr28	10856891	2	38	C:0.710526	T:0.289474		
chr28	10857213	4	40	GGAGAGAGAGAGAGA:0.725	G:0.075	GGAGAGAGAGA:0.05	GGAGAGAGAGAGAGAGA:0.15
chr28	10857820	2	34	C:0.823529	CT:0.176471		
chr28	10857822	2	36	TC:0.694444	T:0.305556		
chr28	10858452	2	36	G:1	A:0		
chr28	10858753	2	40	G:0.95	A:0.05		
chr28	10859031	2	38	C:0.947368	A:0.0526316		
chr28	10859145	2	40	G:0.95	A:0.05		
chr28	10859432	2	36	T:0.305556	C:0.694444		
chr28	10859500	3	38	C:0.236842	CCT:0.578947	CCTCT:0.184211	

chr28 10859548 3 36 T:0.111111 TAA:0.777778
TAAA:0.111111
chr28 10859591 2 38 A:0.0263158 G:0.973684
chr28 10859789 3 38 GT:0.289474 G:0.0263158
GTT:0.684211
chr28 10859873 2 36 C:0.0277778 G:0.972222
chr28 10859994 2 40 A:0.975 G:0.025
chr28 10860062 2 38 A:1 G:0
chr28 10860259 2 40 CAGAAAAA:0.375 C:0.625
chr28 10860267 2 40 TG:0.375 T:0.625
chr28 10860770 2 38 A:0.315789 G:0.684211
chr28 10861029 2 36 G:1 A:0
chr28 10861190 2 38 G:0.552632 C:0.447368
chr28 10861553 2 38 G:0.368421 A:0.631579
chr28 10861598 2 36 C:0.972222 T:0.0277778
chr28 10861930 2 38 G:0.368421 A:0.631579
chr28 10862118 2 38 C:0.973684 T:0.0263158
chr28 10862629 2 38 A:0.394737 G:0.605263
chr28 10862731 2 36 G:1 T:0
chr28 10862999 2 38 C:0.473684 T:0.526316
chr28 10863039 2 38 A:0.947368 T:0.0526316
chr28 10863951 2 40 C:0.7 T:0.3
chr28 10863957 2 40 C:0.375 T:0.625
chr28 10864055 2 38 A:0.789474 G:0.210526
chr28 10864420 2 34 GT:0.441176 G:0.558824
chr28 10864676 2 40 T:0.35 A:0.65
chr28 10864720 2 40 A:0.325 T:0.675
chr28 10865160 2 36 TAC:0.416667 T:0.583333
chr28 10865211 3 34 GT:0.411765 G:0.588235
GTT:0
chr28 10865255 2 38 C:0.342105 A:0.657895
chr28 10865768 4 40 C:0.15 CT:0.25 CTT:0.425
CTTT:0.175
chr28 10865823 2 40 A:0.8 AAGAGAGAG:0.2
chr28 10865863 2 40 C:0.7 A:0.3
chr28 10865864 2 40 C:0.7 G:0.3
chr28 10866132 2 38 TAGA:1 T:0
chr28 10866499 2 40 G:1 A:0
chr28 10866730 2 38 A:0.973684 G:0.0263158
chr28 10866868 2 40 C:0.975 A:0.025
chr28 10866882 3 40 T:0.4 TTTTTTTTC:0.55
TTTTTTTTTC:0.05
chr28 10866963 2 38 T:0.973684 C:0.0263158
chr28 10867622 2 40 T:0.45 A:0.55
chr28 10867736 2 38 G:0.684211 C:0.315789
chr28 10868147 2 32 C:0.65625 T:0.34375
chr28 10868162 2 34 C:0.529412 T:0.470588
chr28 10868166 3 38 CCTCT:0.289474 C:0.421053
CCTCTCTCTCTCTCTCTCT:0.289474
chr28 10868195 2 34 C:0.558824 T:0.441176
chr28 10868318 2 40 T:0.4 A:0.6
chr28 10868481 2 36 A:0.0277778 T:0.972222
chr28 10868483 2 36 T:0.0277778 C:0.972222
chr28 10868521 2 40 CTCTCTCTCTT:0.925 C:0.075

chr28	10868576	2	40	A:0.025	AAAT:0.975
chr28	10868578	2	40	A:1	AT:0
chr28	10868758	2	34	C:0.676471	T:0.323529
chr28	10868815	2	38	G:0.368421	C:0.631579
chr28	10868964	2	40	T:0.375	A:0.625
chr28	10869130	2	36	C:0.277778	T:0.722222
chr28	10869158	2	36	T:0.972222	G:0.0277778
chr28	10869284	2	40	A:0.4	G:0.6
chr28	10869285	2	40	A:0.4	C:0.6
chr28	10869310	2	38	T:0.0263158	G:0.973684
chr28	10869556	2	38	C:0.289474	CTTT:0.710526
chr28	10869590	2	40	GAGAT:1	G:0
chr28	10869596	2	38	GAGAC:0.657895	G:0.342105
chr28	10869650	2	40	A:0.35	T:0.65
chr28	10869654	2	40	C:0.35	T:0.65
chr28	10869696	2	32	C:0.25	T:0.75
chr28	10869731	2	38	C:0.342105	A:0.657895
chr28	10869775	2	40	T:0.4	A:0.6
chr28	10869897	2	38	C:0.315789	T:0.684211
chr28	10870079	2	36	C:0.333333	T:0.666667
chr28	10870392	2	38	A:0.368421	G:0.631579
chr28	10870618	2	40	T:0.4	G:0.6
chr28	10870656	2	40	G:0.45	A:0.55
chr28	10870776	2	32	G:0.4375	GT:0.5625
chr28	10870878	2	38	T:0.973684	C:0.0263158
chr28	10870990	2	34	A:0.294118	G:0.705882
chr28	10871005	2	34	C:1	A:0
chr28	10871006	2	34	C:0.970588	T:0.0294118
chr28	10871062	2	40	G:0.3	T:0.7
chr28	10871087	2	38	C:0.394737	T:0.605263
chr28	10871128	2	36	T:0.388889	C:0.611111
chr28	10871129	2	36	G:0.388889	A:0.611111
chr28	10871149	2	38	C:0.973684	T:0.0263158
chr28	10871212	2	36	G:1	A:0
chr28	10871264	2	38	C:0.263158	A:0.736842
chr28	10871302	2	32	C:0.3125	T:0.6875
chr28	10871347	2	40	T:0.975	C:0.025
chr28	10871425	2	38	C:0.342105	T:0.657895
chr28	10871534	2	38	A:0.263158	G:0.736842
chr28	10871606	2	40	GC:0.95	G:0.05
chr28	10871740	2	38	A:1	G:0
chr28	10871886	2	38	G:0.394737	A:0.605263
chr28	10871887	2	38	T:0.394737	C:0.605263
chr28	10872568	2	38	T:0.0263158	A:0.973684
chr28	10872820	2	34	AG:0.0294118	A:0.970588
chr28	10872826	2	36	T:1	TA:0
chr28	10872858	2	38	A:0.421053	G:0.578947
chr28	10872929	2	38	T:0.973684	G:0.0263158
chr28	10873807	2	40	T:0.45	C:0.55
chr28	10874308	2	38	G:0.184211	A:0.815789
chr28	10874488	2	38	C:1	T:0
chr28	10874513	2	38	C:1	T:0
chr28	10874561	2	40	A:1	C:0
chr28	10874875	2	32	T:1	C:0

chr28	10874877	2	32	G:1	A:0	
chr28	10875700	2	40	C:0.975	T:0.025	
chr28	10876582	2	40	C:1	G:0	
chr28	10876610	2	38	G:0.973684	T:0.0263158	
chr28	10876739	2	40	G:1	A:0	
chr28	10877140	2	36	G:0.416667	A:0.583333	
chr28	10877370	2	40	T:0.025	G:0.975	
chr28	10877580	2	38	TCA:1	T:0	
chr28	10877612	3	40	T:0.25	TA:0.25	TAAA:0.5
chr28	10877971	2	38	G:0.973684	T:0.0263158	
chr28	10877998	2	38	C:0.973684	T:0.0263158	
chr28	10878014	2	38	C:0.973684	A:0.0263158	
chr28	10878049	2	40	A:0.975	T:0.025	
chr28	10878119	2	40	A:0.375	G:0.625	
chr28	10878826	2	40	C:1	T:0	
chr28	10879239	2	38	T:0.447368	A:0.552632	
chr28	10879496	2	40	T:1	C:0	
chr28	10879749	2	38	G:1	C:0	
chr28	10879835	2	40	C:0.3	G:0.7	
chr28	10879837	2	40	AT:0.95	A:0.05	
chr28	10879933	2	40	T:0.025	C:0.975	
chr28	10880214	2	36	C:0.0277778	T:0.972222	
chr28	10880333	2	40	G:0.025	GATTAA:0.975	
chr28	10880468	2	40	C:0.975	T:0.025	
chr28	10880501	2	36	G:0.138889	A:0.861111	
chr28	10880523	2	32	C:0.96875	T:0.03125	
chr28	10880620	2	38	C:0.236842	G:0.763158	
chr28	10880621	2	38	T:0.0789474	TGTGA:0.921053	
chr28	10880633	2	36	T:1	TA:0	
chr28	10880637	2	38	A:0.894737	AAAAAAAAT: 0.105263	
chr28	10880638	3	38	A:0.184211	AAAAAAAT:0.736842	AAAAAAATT:0.0789474
chr28	10880657	2	36	A:1	T:0	
chr28	10880785	2	38	T:0.0263158	A:0.973684	
chr28	10880789	2	38	C:0.631579	A:0.368421	
chr28	10881307	2	38	A:0.0263158	C:0.973684	
chr28	10881437	2	40	A:0.975	G:0.025	
chr28	10881444	2	40	C:0.3	T:0.7	
chr28	10881588	3	38	CTTTT:0.973684	C:0	CTT: 0.0263158
chr28	10881646	2	38	C:0.736842	T:0.263158	
chr28	10881792	2	36	T:1	G:0	
chr28	10881808	2	36	G:0.944444	A:0.0555556	
chr28	10881849	2	38	A:0.973684	G:0.0263158	
chr28	10882110	2	38	C:1	CCTCT:0	
chr28	10882163	2	38	C:0.973684	T:0.0263158	
chr28	10882434	2	40	G:0.025	C:0.975	
chr28	10882688	2	40	TG:0.075	T:0.925	
chr28	10882760	2	38	C:0.921053	A:0.0789474	
chr28	10882808	2	38	A:0.868421	G:0.131579	
chr28	10882976	2	38	C:0.421053	G:0.578947	
chr28	10883014	2	40	C:0.875	T:0.125	
chr28	10883028	2	40	A:0.15	G:0.85	

chr28	10883125	2	40	T:0.875	C:0.125		
chr28	10883240	2	36	TA:0.861111	T:0.138889		
chr28	10883368	2	40	T:0.125	G:0.875		
chr28	10883680	2	40	T:0.125	C:0.875		
chr28	10884142	2	40	A:0.225	AT:0.775		
chr28	10884231	2	40	A:0.3	T:0.7		
chr28	10884436	2	38	A:0.789474	G:0.210526		
chr28	10884509	2	36	G:1	C:0		
chr28	10884565	2	34	T:1	G:0		
chr28	10884599	4	38	TAAA:0.236842	T:0.0789474	TA:0.5	TAA:0.184211
chr28	10885261	2	36	T:0.305556	C:0.694444		
chr28	10885283	2	38	G:0.236842	A:0.763158		
chr28	10885292	2	38	G:0.894737	A:0.105263		
chr28	10885993	2	38	T:0.263158	A:0.736842		
chr28	10886462	2	38	TAA:0.0263158	T:0.973684		
chr28	10886477	2	38	C:0.736842	A:0.263158		
chr28	10886537	2	40	G:1	A:0		
chr28	10887200	2	36	G:0.722222	A:0.277778		
chr28	10887440	2	36	AT:0.305556	A:0.694444		
chr28	10887451	2	34	TG:0.970588	T:0.0294118		
chr28	10887501	2	40	C:0.95	CT:0.05		
chr28	10887503	2	40	T:0.25	TTA:0.75		
chr28	10887504	2	40	TTTTTTTATTTTTTA:0.975	T:0.025		
chr28	10887511	2	40	A:0.3	AT:0.7		
chr28	10887512	2	40	TTTTTTAA:0.95	T:0.05		
chr28	10887519	2	40	A:0.3	T:0.7		
chr28	10887526	2	40	T:0.975	TA:0.025		
chr28	10887540	2	40	G:0.775	GT:0.225		
chr28	10887541	3	40	C:0.025	A:0.75	CA:0.225	
chr28	10887552	2	40	G:0.325	GAGAGAGGC:0.675		
chr28	10887723	2	38	C:1	A:0		
chr28	10887724	2	38	C:0.263158	G:0.736842		
chr28	10887906	2	40	T:0.2	A:0.8		
chr28	10887991	2	40	G:0.975	A:0.025		
chr28	10888001	2	40	G:0.95	A:0.05		
chr28	10888546	2	40	A:0.275	G:0.725		
chr28	10888643	2	40	T:0.275	C:0.725		
chr28	10888752	2	40	C:0.3	T:0.7		
chr28	10888856	2	40	A:0.275	G:0.725		
chr28	10888873	2	40	A:0.225	T:0.775		
chr28	10888979	2	38	A:0.263158	G:0.736842		
chr28	10889159	2	40	A:0.3	G:0.7		
chr28	10889208	2	40	G:0.975	GTTATT:0.025		
chr28	10889388	2	38	G:0.894737	C:0.105263		
chr28	10889581	2	36	TTTTA:0.805556	T:0.194444		
chr28	10889598	2	34	A:0.764706	G:0.235294		
chr28	10889965	2	34	A:0.294118	AT:0.705882		
chr28	10890035	2	36	C:0.861111	T:0.138889		
chr28	10890255	2	36	G:0.305556	A:0.694444		
chr28	10890290	2	36	A:0.694444	G:0.305556		
chr28	10890352	2	38	G:0.868421	C:0.131579		
chr28	10890492	2	36	G:0.638889	A:0.361111		
chr28	10890598	3	34	T:0.529412	TG:0.441176		

TGG:0.0294118
chr28	10890840 2	10	TGGCGGCGGCGGC:0.1	T:0.9
chr28	10891262 2	36	C:0.0277778	CA:0.972222
chr28	10891267 2	36	G:0.972222	C:0.0277778
chr28	10891415 2	36	G:0.388889	A:0.611111
chr28	10891631 2	36	C:0	G:1
chr28	10892104 2	38	G:0.947368	C:0.0526316
chr28	10892388 2	40	A:1	C:0
chr28	10892613 3	40	C:0.5	CT:0.2	CTTT:0.3
chr28	10893265 2	36	T:0.944444	A:0.0555556
chr28	10893391 2	40	CT:0.95	C:0.05
chr28	10893402 2	40	C:0.725	T:0.275
chr28	10893436 2	38	G:1	A:0
chr28	10894068 2	40	C:0.65	T:0.35
chr28	10894272 2	34	A:0.882353	AT:0.117647
chr28	10894438 2	40	A:0.95	T:0.05
chr28	10894539 2	34	C:0.529412	CAG:0.470588
chr28	10894647 2	38	C:0.473684	A:0.526316
chr28	10894755 2	40	G:0.975	GT:0.025
chr28	10894864 2	38	A:1	T:0
chr28	10894994 2	38	C:1	T:0
chr28	10895137 3	40	TATTG:0.4	T:0.05
TATTGATTG:0.55
chr28	10895171 2	40	GACACAC:0.325	G:0.675
chr28	10895298 2	32	G:1	C:0
chr28	10895383 2	40	GTTTGTT:0.95	G:0.05
chr28	10895443 2	40	G:1	A:0
chr28	10895844 2	38	G:1	A:0
chr28	10896146 2	38	T:0.421053	A:0.578947
chr28	10896255 2	32	G:0.96875	C:0.03125
chr28	10896385 2	40	TA:0.6	T:0.4
chr28	10897336 2	38	G:0	GC:1
chr28	10897637 2	38	A:0	C:1
chr28	10897638 2	38	A:0	T:1
chr28	10897678 2	40	A:0	T:1
chr28	10897680 2	40	G:0	A:1
chr28	10898145 2	40	T:0.025	G:0.975
chr28	10899759 2	38	G:0.947368	A:0.0526316
chr28	10900002 2	40	A:0.425	G:0.575
chr28	10901102 2	36	A:1	AT:0
chr28	10901787 2	38	AGGATTTTCACTGT:1 A:0
chr28	10902365 4	40	T:0.225	TTC:0.625	TTCTC:
0.05	TTCTCTC:0.1
chr28	10902471 3	38	CT:0.631579	C:0.236842
CTT:0.131579
chr28	10902624 2	38	G:1	A:0
chr28	10902631 2	38	GGA:1	G:0
chr28	10903125 2	40	T:0.95	C:0.05
chr28	10905054 2	38	A:0.973684	G:0.0263158
chr28	10905636 2	38	C:0.789474	CT:0.210526
chr28	10905757 2	34	C:0.294118	CT:0.705882
chr28	10906371 2	38	C:1	T:0
chr28	10907929 3	38	C:0.447368	CT:0.552632
CTT:0

chr28 10908479 2 36 G:0.222222 GT:0.777778
chr28 10909005 2 34 G:0.470588 A:0.529412
chr28 10909089 2 40 C:1 T:0
chr28 10909106 2 38 T:0.447368 TAAA:0.552632
chr28 10909107 4 38 T:0.315789 A:0.552632
TAAA:0.131579 TTAA:0
chr28 10909525 2 36 C:0.972222 CAGAG:0.0277778
chr28 10909952 6 40 T:0.125 TATAG:0 TATAGATAG:0.05
TATAGATAGATAG:0.65 TATAGATAGATAGATAG:0.125
TATAGATAGATAGATAGATAG:0.05
chr28 10909963 2 40 A:0.975 AGATAGATAGATG:0.025
chr28 10911677 2 40 CT:1 C:0
chr28 10912047 2 36 T:0.944444 A:0.0555556
chr28 10912950 2 40 A:0.925 G:0.075
chr28 10913896 2 38 TG:1 T:0
chr28 10914811 2 32 CTA:0.46875 C:0.53125
chr28 10914827 5 38 CAAATAAAT:0.184211 C:
0.263158 CAAAT:0.0789474 CAAATAAATAAAT:0.394737
CAAATAAATAAATAAAT:0.0789474
chr28 10915294 2 40 C:1 T:0
chr28 10915752 2 36 T:0.666667 A:0.333333
chr28 10915858 2 38 C:0.973684 T:0.0263158
chr28 10916032 3 32 C:0.375 CT:0.375 CTTT:0.25
chr28 10916305 2 38 T:0.421053 C:0.578947
chr28 10916365 2 40 C:0.425 T:0.575
chr28 10917379 2 34 C:0.176471 CT:0.823529
chr28 10917399 2 32 A:0.6875 C:0.3125
chr28 10918597 2 40 T:1 C:0
chr28 10918758 2 40 CTAAG:1 C:0
chr28 10918832 2 40 C:0.65 T:0.35
chr28 10920076 2 38 C:1 G:0
chr28 10924839 2 40 G:0.725 A:0.275
chr28 10924859 2 40 T:0.775 A:0.225
chr28 10925321 2 34 GC:0.882353 G:0.117647
chr28 10925332 2 36 CCA:0.888889 C:0.111111
chr28 10925333 2 34 CA:0.735294 C:0.264706
chr28 10925638 2 40 C:0.925 T:0.075
chr28 10925699 2 36 TC:1 T:0
chr28 10926072 2 38 T:0.447368 C:0.552632
chr28 10926654 2 38 A:0.473684 G:0.526316
chr28 10926698 2 36 TA:0.777778 T:0.222222
chr28 10927281 2 38 C:0.315789 T:0.684211
chr28 10927702 2 40 C:1 T:0
chr28 10927966 2 38 T:0.315789 C:0.684211
chr28 10928296 4 40 T:0.075 TA:0.4 TAA:0.4 TAAA:
0.125
chr28 10928307 2 40 A:0.975 AAC:0.025
chr28 10929538 2 38 T:0.631579 C:0.368421
chr28 10929724 2 40 G:0.975 A:0.025
chr28 10930563 2 30 G:0.933333 A:0.0666667
chr28 10930732 2 18 G:0.388889 C:0.611111
chr28 10931128 2 28 CG:0.392857 C:0.607143
chr28 10931223 2 36 T:0.222222 C:0.777778
chr28 10931289 2 34 A:0.970588 G:0.0294118

chr28 10931588 2 38 G:0.421053 C:0.578947
chr28 10931590 2 36 G:0.5 C:0.5
chr28 10931625 2 38 T:0.447368 TA:0.552632
chr28 10931626 2 38 T:0.447368 A:0.552632
chr28 10931741 2 40 A:0.55 C:0.45
chr28 10931801 2 40 G:1 C:0
chr28 10931842 2 38 C:0.526316 CAG:0.473684
chr28 10932002 3 40 TAAAAAAAAAAAAACATA:0.525 T:0.05
TAAAAAAAAAAAAAAACATA:0.425
chr28 10932018 2 40 TA:0.45 T:0.55
chr28 10932229 3 28 CTGTG:0.535714 C:0.428571
CTG:0.0357143
chr28 10932540 2 38 C:0.421053 A:0.578947
chr28 10932838 2 40 C:1 T:0
chr28 10933289 2 40 A:0.6 G:0.4
chr28 10933798 2 38 C:0.868421 CA:0.131579
chr28 10933842 2 36 T:1 A:0
chr28 10934253 2 40 T:0.5 C:0.5
chr28 10934424 2 40 T:0.325 C:0.675
chr28 10934487 2 38 A:0.342105 T:0.657895
chr28 10934765 2 38 T:0.526316 C:0.473684
chr28 10934913 2 38 A:1 G:0
chr28 10934991 2 32 TA:0.40625 T:0.59375
chr28 10934992 2 32 A:1 T:0
chr28 10935306 2 40 T:0.65 A:0.35
chr28 10935724 2 40 T:0.925 C:0.075
chr28 10935759 3 40 TA:0.65 T:0.325 TAA:0.025
chr28 10935844 2 40 C:0.975 T:0.025
chr28 10936650 2 34 C:0.676471 T:0.323529
chr28 10936790 2 38 A:0.526316 ACT:0.473684
chr28 10936918 2 36 G:0.972222 A:0.0277778
chr28 10937108 2 36 C:0.305556 T:0.694444
chr28 10937631 2 34 T:0.529412 C:0.470588
chr28 10937655 2 36 G:0.638889 A:0.361111
chr28 10937749 2 38 C:0.947368 T:0.0526316
chr28 10937773 2 38 C:0.947368 CATTT:0.0526316
chr28 10937780 3 38 T:0.342105 TTTTTTTTAA:
0.578947 TTTTTTTTTAA:0.0789474
chr28 10937947 2 38 G:0.526316 A:0.473684
chr28 10938016 2 38 T:0.894737 C:0.105263
chr28 10938156 2 34 G:1 A:0
chr28 10938218 2 38 CTCTCTTCCCT:0.789474 C:
0.210526
chr28 10938220 2 38 CTCTTCCCT:0.894737 C:
0.105263
chr28 10938222 3 38 CTTCCCT:0.763158 CTCCCT:0.236842
C:0
chr28 10938223 2 40 T:0.9 TC:0.1
chr28 10938226 2 38 C:0.657895 T:0.342105
chr28 10938228 2 38 T:0.578947 TC:0.421053
chr28 10938232 2 38 C:0.631579 G:0.368421
chr28 10938234 2 38 G:1 C:0
chr28 10938284 2 38 G:0.947368 GAAAGA:0.0526316
chr28 10938529 4 40

TTTTCTTTCTTTCTTTCTTTCTTTCTTTCTTTCTTTCTTTC:0.25	T:0.65
TTTTCTTTC:0.1	TTTTCTTTCTTTCTTTCTTTCTTTCTTTCTTTCTTTCTTTCTTTCTTTC:0

chr28	10938824	2	38	A:0.5	G:0.5
chr28	10939148	2	40	C:0.6	T:0.4
chr28	10939244	2	40	T:0.5	G:0.5
chr28	10939253	2	40	G:0.35	A:0.65
chr28	10939350	2	40	A:0.325	G:0.675
chr28	10939452	2	36	A:0.972222	G:0.0277778
chr28	10939519	2	36	A:0.916667	G:0.0833333
chr28	10939720	2	34	G:1	A:0
chr28	10940564	2	38	C:1	T:0
chr28	10940679	2	38	C:0.736842	T:0.263158
chr28	10941347	2	40	C:0.3	T:0.7
chr28	10941594	2	36	C:1	T:0
chr28	10941647	2	38	G:0.473684	GCTCT:0.526316
chr28	10942341	2	38	G:1	C:0
chr28	10942723	2	40	C:0.95	T:0.05
chr28	10942928	2	40	T:1	G:0
chr28	10943113	2	38	G:0.736842	A:0.263158
chr28	10943289	2	38	C:0.684211	G:0.315789
chr28	10943290	2	38	A:0.684211	G:0.315789
chr28	10943378	2	36	A:0.694444	G:0.305556
chr28	10943556	2	36	T:0.75	C:0.25
chr28	10943966	2	40	A:0.7	G:0.3
chr28	10943989	2	38	A:0.684211	G:0.315789
chr28	10944846	2	40	C:0.4	T:0.6
chr28	10944878	2	40	C:0.95	A:0.05
chr28	10945075	2	40	G:1	A:0
chr28	10945214	2	36	T:0.416667	C:0.583333
chr28	10945434	2	34	G:0.558824	A:0.441176
chr28	10946291	2	34	C:0.352941	T:0.647059
chr28	10946391	2	32	G:0.28125	A:0.71875
chr28	10946568	2	20	A:0.3	G:0.7
chr28	10946632	2	20	A:0.4	G:0.6
chr28	10948332	2	34	C:0.764706	T:0.235294
chr28	10948399	2	24	C:0.916667	CT:0.0833333
chr28	10948404	2	24	C:0.916667	CCG:0.0833333
chr28	10948410	2	24	T:0.916667	C:0.0833333
chr28	10949030	2	38	ACAGCTGGCC:0.921053	A: 0.0789474
chr28	10949135	2	40	T:0.725	A:0.275
chr28	10949245	2	40	T:0.675	C:0.325
chr28	10949618	2	40	A:1	G:0
chr28	10949636	2	36	T:0.833333	TA:0.166667
chr28	10949827	2	38	C:0.973684	T:0.0263158
chr28	10949911	2	40	A:0.375	C:0.625
chr28	10950059	2	38	C:0.789474	T:0.210526
chr28	10950206	2	40	A:0.975	C:0.025
chr28	10950222	2	38	G:0.657895	A:0.342105
chr28	10950323	2	34	C:0.941176	G:0.0588235
chr28	10950328	2	34	C:0.647059	T:0.352941
chr28	10950411	2	34	C:0.676471	G:0.323529
chr28	10950548	2	34	C:0.705882	T:0.294118
chr28	10950605	2	38	C:1	G:0

chr28	10951080	2	36	C:0.75	A:0.25	
chr28	10951082	2	36	G:0.75	A:0.25	
chr28	10951248	2	38	A:1	G:0	
chr28	10951424	2	40	ACCCTGCATGGTTTAC:1	A:0	
chr28	10951510	2	36	C:0.694444	G:0.305556	
chr28	10952305	2	38	A:1	C:0	
chr28	10952502	3	38	A:0.657895	C:0	G:0.342105
chr28	10952514	2	38	A:1	G:0	
chr28	10952562	2	38	C:1	G:0	
chr28	10952625	2	36	G:1	C:0	
chr28	10952637	2	34	GTA:0.676471	G:0.323529	
chr28	10952639	2	36	A:1	G:0	
chr28	10952815	2	40	T:0.625	C:0.375	
chr28	10952979	2	40	A:0.675	G:0.325	
chr28	10953058	2	38	A:0.789474	T:0.210526	
chr28	10953067	2	40	T:0.925	A:0.075	
chr28	10953068	2	40	T:0.775	A:0.225	
chr28	10953078	2	38	AAAAAT:1	A:0	
chr28	10953092	2	40	A:0.775	G:0.225	
chr28	10953185	2	28	C:0.785714	CA:0.214286	
chr28	10953242	2	30	T:0.766667	C:0.233333	
chr28	10953533	2	26	G:0.615385	T:0.384615	
chr28	10953616	2	26	C:0.538462	A:0.461538	
chr28	10953773	2	38	G:0.868421	GAGTGTGGA:0.131579	
chr28	10953774	2	38	C:0.842105	CCACCCATGGCATAGCCAGCAT:0.157895	
chr28	10954058	2	40	C:0.675	T:0.325	
chr28	10954059	2	40	A:0.675	G:0.325	
chr28	10954098	2	40	C:0.25	CAGGGA:0.75	
chr28	10954143	2	38	T:0.736842	A:0.263158	
chr28	10954226	2	36	T:0.777778	TTTTA:0.222222	
chr28	10954538	2	38	G:0.421053	C:0.578947	
chr28	10954668	2	38	C:0.894737	T:0.105263	
chr28	10954711	2	38	A:1	AATT:0	
chr28	10954733	2	40	C:0.975	T:0.025	
chr28	10954773	2	40	AGGCG:0	A:1	
chr28	10955057	2	40	T:0.65	C:0.35	
chr28	10955111	2	38	CACG:0.605263	C:0.394737	
chr28	10955169	2	40	A:0.45	G:0.55	
chr28	10955289	2	36	G:0.0277778	C:0.972222	
chr28	10955498	2	38	GAC:0.894737	G:0.105263	
chr28	10955581	2	40	G:0.9	A:0.1	
chr28	10955860	2	38	G:0.684211	T:0.315789	
chr28	10955862	2	38	A:0.684211	G:0.315789	
chr28	10956211	2	38	A:0.631579	C:0.368421	
chr28	10956220	2	36	C:0.75	T:0.25	
chr28	10956406	2	40	AGTGATTTTTTTTACCTTG:0.675	A:0.325	
chr28	10956444	2	40	T:0.775	A:0.225	
chr28	10956472	3	40	C:0.7	CAG:0	CAGAGAG:0.3
chr28	10956634	2	36	AT:0.638889	A:0.361111	
chr28	10956653	2	36	A:0.722222	T:0.277778	
chr28	10956654	2	36	A:0.722222	T:0.277778	

chr28	10956764	2	38	A:0.421053	AT:0.578947	
chr28	10956893	2	30	G:0.766667	A:0.233333	
chr28	10956988	2	36	A:0.361111	G:0.638889	
chr28	10957438	2	38	G:0.842105	A:0.157895	
chr28	10957724	2	38	T:0.710526	G:0.289474	
chr28	10957781	2	38	C:0.763158	CT:0.236842	
chr28	10957792	2	36	G:0.333333	T:0.666667	
chr28	10957793	2	38	GAA:0.763158	G:0.236842	
chr28	10957794	2	36	A:0.638889	G:0.361111	
chr28	10957804	2	38	A:0.789474	T:0.210526	
chr28	10958111	2	40	G:0.3	A:0.7	
chr28	10958233	2	40	AT:0.6	A:0.4	
chr28	10958332	2	34	T:0.676471	G:0.323529	
chr28	10958419	2	38	C:0.763158	A:0.236842	
chr28	10958530	3	38	CT:0.447368	C:0.263158	CTT:0.289474
chr28	10958549	3	28	CTT:0.321429	C:0.214286	CT:0.464286
chr28	10958909	2	40	GGAC:0.875	G:0.125	
chr28	10958913	2	40	TCA:0.875	T:0.125	
chr28	10959445	2	38	C:0.342105	T:0.657895	
chr28	10959761	2	36	T:1	A:0	
chr28	10959870	2	38	G:1	C:0	
chr28	10959990	2	36	G:0.75	GAC:0.25	
chr28	10960491	2	36	TA:0.361111	T:0.638889	
chr28	10960645	2	38	TCTA:1	T:0	
chr28	10960661	2	38	C:0.710526	T:0.289474	
chr28	10960664	2	38	T:0.710526	C:0.289474	
chr28	10960683	2	32	CT:0.4375	C:0.5625	
chr28	10960719	3	40	C:0.525	CAG:0.225	CAGAGAG:0.25
chr28	10960739	2	40	GGC:0.95	G:0.05	
chr28	10961047	2	40	T:0.725	C:0.275	
chr28	10961328	2	40	T:0.65	TTTTTTTTGTTTGTTTTTTTTTTTTTACATCAATAAAATCTTAA:0.35	
chr28	10961337	2	30	ACC:0.433333	A:0.566667	
chr28	10961571	2	32	T:0.4375	G:0.5625	
chr28	10961978	2	38	C:0.947368	T:0.0526316	
chr28	10962777	2	32	T:0.59375	C:0.40625	
chr28	10962807	2	38	CGATCCTGGAGACCCGGGATCG:0.763158	C:0.236842	
chr28	10962928	2	38	TTAAAAAAAA:0.973684	T:0.0263158	
chr28	10962929	2	38	T:0.894737	TA:0.105263	
chr28	10962938	2	38	T:0.447368	TA:0.552632	
chr28	10962939	2	38	A:1	AT:0	
chr28	10963007	2	38	A:0.868421	G:0.131579	
chr28	10963122	2	36	A:0.972222	G:0.0277778	
chr28	10963320	2	40	CAA:0.75	C:0.25	
chr28	10963342	2	40	G:0.475	C:0.525	
chr28	10963556	2	40	C:0.675	CT:0.325	
chr28	10963558	2	40	T:0.95	TTCTTTC:0.05	
chr28	10964557	2	36	GC:0.638889	G:0.361111	
chr28	10964800	2	36	C:0.666667	T:0.333333	

chr28	10964924	2	40	T:0.975	C:0.025		
chr28	10965013	2	34	C:0.941176	CT:0.0588235		
chr28	10965016	2	36	T:0.444444	TTTTC:0.555556		
chr28	10965062	2	38	C:0.263158	G:0.736842		
chr28	10965153	2	38	G:1	A:0		
chr28	10965483	2	34	A:0.588235	ACC:0.411765		
chr28	10965485	2	38	C:0.763158	CCT:0.236842		
chr28	10965491	2	36	C:0.75	CCA:0.25		
chr28	10965492	2	38	A:0.763158	C:0.236842		
chr28	10965784	2	36	AAGG:0.916667	A:0.0833333		
chr28	10966005	2	36	A:0.916667	G:0.0833333		
chr28	10966282	2	24	G:0.625	C:0.375		
chr28	10966579	2	22	G:0.0909091	A:0.909091		
chr28	10966622	2	32	A:0.875	G:0.125		
chr28	10966652	2	36	G:0.888889	A:0.111111		
chr28	10966710	2	36	T:0.888889	C:0.111111		
chr28	10966731	2	38	G:0.894737	T:0.105263		
chr28	10966732	2	38	A:0.894737	T:0.105263		
chr28	10966981	2	38	G:0.394737	A:0.605263		
chr28	10967204	2	38	G:0.947368	A:0.0526316		
chr28	10967625	2	40	C:0.525	T:0.475		
chr28	10967688	2	40	A:0.125	G:0.875		
chr28	10967758	2	38	G:0.578947	C:0.421053		
chr28	10967940	2	38	T:0.473684	C:0.526316		
chr28	10967997	2	40	T:0.175	C:0.825		
chr28	10968267	2	40	A:0.75	T:0.25		
chr28	10968892	2	40	A:1	G:0		
chr28	10969042	2	36	T:1	C:0		
chr28	10969128	2	40	G:0.475	A:0.525		
chr28	10969133	2	40	T:0.15	C:0.85		
chr28	10969630	2	38	A:0.394737	G:0.605263		
chr28	10969949	2	38	A:0.421053	ATTGT:0.578947		
chr28	10969976	2	38	G:0.631579	A:0.368421		
chr28	10970109	2	36	G:0.805556	A:0.194444		
chr28	10970473	2	40	T:0.2	C:0.8		
chr28	10970631	2	40	T:0.375	A:0.625		
chr28	10970698	2	38	G:0.368421	A:0.631579		
chr28	10971002	2	36	A:0.916667	C:0.0833333		
chr28	10971194	2	36	CAG:0.388889	C:0.611111		
chr28	10971312	2	38	C:0.973684	T:0.0263158		
chr28	10971398	2	38	G:1	A:0		
chr28	10972104	2	36	G:0.388889	A:0.611111		
chr28	10972185	4	38	ATTT:0.0789474	AT:0.473684	ATT:0.263158	A:0.184211
chr28	10972211	4	40	CTTTATTTATTTATTTATTTATTTATTTATTTA:0.25	C:0.225	CTTTATTTA:0.025	CTTTATTTATTTA:0.5
chr28	10972309	2	40	T:0.425	C:0.575		
chr28	10972350	2	38	C:0.552632	T:0.447368		
chr28	10972364	2	36	C:0.555556	T:0.444444		
chr28	10972567	2	38	G:0.552632	A:0.447368		
chr28	10972570	2	38	G:0.421053	T:0.578947		
chr28	10972747	2	38	GCCATCTTTTTCTGAAAAAGC:0.684211	G:0.315789		
chr28	10972778	2	36	T:0.222222	TC:0.777778		

chr28 10972787 2 36 G:0.222222 GTGGT:0.777778
chr28 10973528 2 36 G:0.527778 A:0.472222
chr28 10973608 2 38 A:0.631579 G:0.368421
chr28 10973714 2 34 G:0.764706 T:0.235294
chr28 10973828 2 40 C:0.725 T:0.275
chr28 10974020 2 40 C:0.425 A:0.575
chr28 10974066 2 38 C:0.421053 T:0.578947
chr28 10974133 2 38 C:0.842105 T:0.157895
chr28 10974294 2 38 C:0.631579 T:0.368421
chr28 10974322 2 38 C:0.605263 T:0.394737
chr28 10974417 2 38 G:1 A:0
chr28 10974622 2 40 T:0.4 TC:0.6
chr28 10974634 2 40 T:0.4 C:0.6
chr28 10974958 2 40 T:0.475 TTTC:0.525
chr28 10975024 3 38 GTTTT:0.552632 G:0.236842
GT:0.210526
chr28 10975261 2 38 G:1 A:0
chr28 10975398 2 36 T:0.555556 C:0.444444
chr28 10975516 2 40 A:0.475 G:0.525
chr28 10975656 2 40 G:0.55 A:0.45
chr28 10975693 2 40 AT:0.975 A:0.025
chr28 10975698 2 40 T:0.65 TA:0.35
chr28 10975702 4 40 TAA:0.35 T:0.375 AAA:0.025
TA:0.25
chr28 10975704 2 40 A:0.875 T:0.125
chr28 10975763 2 36 G:0.833333 T:0.166667
chr28 10976105 2 20 GA:0 G:1
chr28 10976177 2 32 TTATG:0.84375 T:0.15625
chr28 10976274 2 38 TC:0.5 T:0.5
chr28 10976373 2 34 C:0.617647 T:0.382353
chr28 10976533 2 22 C:0.863636 CAT:0.136364
chr28 10976599 2 36 G:0.416667 C:0.583333
chr28 10976600 2 36 A:0.416667 T:0.583333
chr28 10976984 2 38 T:0.0789474 C:0.921053
chr28 10977233 3 38 C:0.578947 CAGAGAG:0.315789
CAGAGAGAGAG:0.105263
chr28 10977288 2 38 T:0.473684 C:0.526316
chr28 10977367 2 40 G:0.825 A:0.175
chr28 10977383 2 36 CA:1 C:0
chr28 10977384 2 38 A:0.5 G:0.5
chr28 10977394 2 38 T:0.5 A:0.5
chr28 10977396 2 38 T:0.5 A:0.5
chr28 10977449 2 30 CGACA:0.566667 C:0.433333
chr28 10977452 3 34 CAG:0.823529 C:0.0294118
GAG:0.147059
chr28 10977531 2 30 CATG:0.4 C:0.6
chr28 10977593 2 36 T:0.527778 C:0.472222
chr28 10978165 3 40 T:0.175 C:0.425 G:0.4
chr28 10978203 2 40 G:0.525 T:0.475
chr28 10978372 2 40 G:1 A:0
chr28 10978381 2 40 A:1 C:0
chr28 10978429 2 38 C:0.421053 CA:0.578947
chr28 10978430 2 38 C:0.947368 T:0.0526316
chr28 10978442 2 38 G:0.421053 GGTA:0.578947

chr28	10978513	2	40	A:0.45	C:0.55		
chr28	10978703	2	40	A:0.4	C:0.6		
chr28	10979470	2	38	C:1	T:0		
chr28	10979777	2	38	G:0.368421	GTCTT:0.631579		
chr28	10979849	2	40	T:0.975	A:0.025		
chr28	10979907	2	38	G:0.842105	A:0.157895		
chr28	10979981	2	38	G:0.947368	A:0.0526316		
chr28	10980312	2	38	G:0.736842	T:0.263158		
chr28	10980412	2	38	T:0.552632	TA:0.447368		
chr28	10980524	2	38	A:0.157895	G:0.842105		
chr28	10980567	2	38	T:0.552632	A:0.447368		
chr28	10980625	2	36	T:0.166667	C:0.833333		
chr28	10981037	2	38	A:0.657895	G:0.342105		
chr28	10981304	2	40	T:0.2	C:0.8		
chr28	10981413	2	38	T:0.0789474	C:0.921053		
chr28	10981744	2	38	T:0.184211	C:0.815789		
chr28	10981920	2	38	C:0.973684	T:0.0263158		
chr28	10982003	2	40	A:0.6	G:0.4		
chr28	10982720	2	40	A:1	T:0		
chr28	10983100	2	40	G:0.6	A:0.4		
chr28	10983107	2	40	G:0.225	A:0.775		
chr28	10983144	2	38	C:0.710526	CTTCT:0.289474		
chr28	10983198	3	40	CAGAGAG:0.325	C:0.35	CAGAGAGAG:0.325	
chr28	10983293	2	32	C:0.625	A:0.375		
chr28	10983352	2	36	CCTT:0.972222	C:0.0277778		
chr28	10983401	2	38	A:0.5	G:0.5		
chr28	10983418	2	36	T:0.527778	G:0.472222		
chr28	10983603	2	36	G:0.361111	A:0.638889		
chr28	10983637	2	36	T:0.416667	C:0.583333		
chr28	10983774	2	40	A:0.475	AGTCC:0.525		
chr28	10983818	2	40	G:0.95	A:0.05		
chr28	10983977	2	40	G:0.875	A:0.125		
chr28	10984009	2	38	G:0.657895	A:0.342105		
chr28	10984411	2	40	C:0.475	T:0.525		
chr28	10984568	2	38	C:0.631579	A:0.368421		
chr28	10984869	2	36	G:0.333333	A:0.666667		
chr28	10984997	2	40	T:0.35	C:0.65		
chr28	10985087	2	40	C:0.425	T:0.575		
chr28	10985273	4	38	CTG:0.263158	C:0.315789	CTGTG:0.0526316	CTGTGTGTGTGTGTGTGTGTGTGTG:0.368421
chr28	10985285	2	40	G:0.85	GTGTGTGTGTGTGTGTGTGTGTGTGTGTGTGTGT:0.15		
chr28	10985313	2	40	A:0.375	G:0.625		
chr28	10985337	2	40	A:0.375	G:0.625		
chr28	10985486	2	38	A:0.657895	C:0.342105		
chr28	10985523	2	34	T:0.323529	C:0.676471		
chr28	10985564	2	34	C:0.558824	CT:0.441176		
chr28	10985604	2	38	AT:0.578947	A:0.421053		
chr28	10985656	2	38	T:0.131579	C:0.868421		
chr28	10985748	2	38	C:0.605263	CT:0.394737		
chr28	10985921	2	38	A:0.157895	AT:0.842105		
chr28	10986385	2	38	C:0.578947	T:0.421053		
chr28	10986427	2	36	T:0.638889	C:0.361111		

chr28	10986678	2	36	T:0.527778	G:0.472222	
chr28	10986718	2	36	A:0.555556	G:0.444444	
chr28	10986842	2	36	G:0.611111	A:0.388889	
chr28	10986885	2	36	G:0.444444	T:0.555556	
chr28	10987378	2	40	C:0.375	A:0.625	
chr28	10987477	2	38	T:0.605263	A:0.394737	
chr28	10987505	2	36	T:0.472222	G:0.527778	
chr28	10987590	2	40	T:0.375	C:0.625	
chr28	10987599	2	40	C:0.375	G:0.625	
chr28	10987920	2	38	A:0.605263	G:0.394737	
chr28	10987986	2	34	T:0.558824	C:0.441176	
chr28	10988012	2	38	C:0.947368	T:0.0526316	
chr28	10988453	2	36	C:0.416667	CG:0.583333	
chr28	10988522	2	38	C:0.868421	T:0.131579	
chr28	10988580	2	38	A:0.605263	G:0.394737	
chr28	10988882	2	40	C:0.65	T:0.35	
chr28	10988978	2	38	CT:0.157895	C:0.842105	
chr28	10989264	2	40	C:0.85	T:0.15	
chr28	10989268	2	40	C:0.85	T:0.15	
chr28	10989464	2	38	A:0.921053	C:0.0789474	
chr28	10989866	2	34	C:0.529412	A:0.470588	
chr28	10990280	2	36	A:0.888889	T:0.111111	
chr28	10990637	2	38	T:0.921053	G:0.0789474	
chr28	10990840	2	38	A:0.684211	G:0.315789	
chr28	10991115	2	40	TTTC:0.95	T:0.05	
chr28	10991142	3	40	T:0.575	TA:0.15	TAA:0.275
chr28	10991340	2	38	T:0.473684	C:0.526316	
chr28	10991590	2	40	G:0.95	A:0.05	
chr28	10991684	2	36	A:0.611111	T:0.388889	
chr28	10991696	2	36	T:0.611111	C:0.388889	
chr28	10991738	2	38	A:0.605263	G:0.394737	
chr28	10991766	2	38	A:0.526316	G:0.473684	
chr28	10991773	2	38	G:0.526316	A:0.473684	
chr28	10991800	2	34	A:0.441176	G:0.558824	
chr28	10991874	2	38	A:0.605263	C:0.394737	
chr28	10992336	2	36	C:0.666667	T:0.333333	
chr28	10992547	2	34	G:0.676471	A:0.323529	
chr28	10992571	2	36	C:0.638889	A:0.361111	
chr28	10992572	2	36	CG:0.638889	C:0.361111	
chr28	10992573	2	36	G:0.888889	C:0.111111	
chr28	10992700	2	38	T:0.131579	C:0.868421	
chr28	10992714	2	40	C:0.525	T:0.475	
chr28	10992715	2	38	G:0.578947	A:0.421053	
chr28	10992822	2	38	G:0.631579	GC:0.368421	
chr28	10993329	2	40	GC:0.85	G:0.15	
chr28	10993330	2	40	CCCCAG:0.225	C:0.775	
chr28	10993332	2	40	C:0.925	CTG:0.075	
chr28	10993333	2	40	CAG:0.925	C:0.075	
chr28	10993335	2	40	G:0.85	GC:0.15	
chr28	10993525	2	30	A:1	G:0	
chr28	10993669	2	30	A:0.966667	G:0.0333333	
chr28	10993730	2	36	G:1	GGTGT:0	
chr28	10993759	2	28	GTA:0.821429	G:0.178571	
chr28	10993761	2	28	A:1	G:0	

chr28	10993763	2	32	A:1	G:0				
chr28	10993946	2	36	GA:0.472222	G:0.527778				
chr28	10994209	2	38	TA:0.5	T:0.5				
chr28	10994309	2	34	C:0.617647	T:0.382353				
chr28	10994311	2	34	A:0.617647	G:0.382353				
chr28	10994906	2	36	G:0.111111	A:0.888889				
chr28	10995117	2	38	G:1	A:0				
chr28	10995247	2	38	T:0.526316	G:0.473684				
chr28	10995395	2	38	C:0.0263158	G:0.973684				
chr28	10995495	2	38	A:0.526316	G:0.473684				
chr28	10995595	2	36	A:0.944444	C:0.0555556				
chr28	10995777	2	32	CTG:0.90625	C:0.09375				
chr28	10996035	2	38	G:0.894737	C:0.105263				
chr28	10996211	2	38	A:0.921053	AG:0.0789474				
chr28	10996475	2	38	G:1	A:0				
chr28	10996540	2	36	A:0.0277778	T:0.972222				
chr28	10996665	2	38	C:0.578947	T:0.421053				
chr28	10996729	2	40	C:0.55	T:0.45				
chr28	10996829	2	36	A:0.916667	G:0.0833333				
chr28	10996877	6	40	CGTGTGTGTGTGTGTGT:0.15	C:0.3	CGTGTGT:0.2	CGTGTGTGT:0.15	CGTGTGTGTGTGTGT:0.15	CGTGTGTGTGTGTGTGTGT:0.05
chr28	10996947	2	38	A:0.605263	G:0.394737				
chr28	10997231	2	38	A:0.921053	G:0.0789474				
chr28	10997649	2	40	G:0.1	GT:0.9				
chr28	10997759	2	40	A:0.075	G:0.925				
chr28	10997837	2	38	T:0.526316	C:0.473684				
chr28	10997840	2	38	GCC:0.526316	G:0.473684				
chr28	10997871	2	40	C:0.6	G:0.4				
chr28	10997960	2	40	GGCTCTCTCTCCCCT:0.1	G:0.9				
chr28	10998658	2	38	ATG:0.973684	A:0.0263158				
chr28	10998674	2	38	G:0.447368	GT:0.552632				
chr28	10998675	3	40	G:0.475	GT:0.425	GTGTGTGT:0.1			
chr28	10998719	2	32	C:0.90625	T:0.09375				
chr28	10998878	2	34	A:0.617647	G:0.382353				
chr28	10998898	2	32	T:0.6875	C:0.3125				
chr28	10998904	2	32	T:0.6875	C:0.3125				
chr28	10998976	2	34	G:0.0294118	C:0.970588				
chr28	10999062	2	26	C:0.384615	CG:0.615385				
chr28	10999116	2	20	CGT:0.35	C:0.65				
chr28	10999539	2	16	G:0	GT:1				
chr28	11000448	2	38	G:0.526316	A:0.473684				
chr28	11000873	2	40	G:0.9	T:0.1				
chr28	11000897	2	40	G:0.95	A:0.05				
chr28	11001070	2	38	G:1	T:0				
chr28	11001281	2	38	G:0.131579	A:0.868421				
chr28	11001339	2	38	A:0.131579	G:0.868421				
chr28	11001408	2	40	GTTCC:0.1	G:0.9				
chr28	11001623	2	36	T:1	C:0				
chr28	11001813	2	38	T:0.0526316	C:0.947368				
chr28	11001908	2	40	C:0.9	T:0.1				
chr28	11001982	2	40	C:0.875	T:0.125				
chr28	11002146	2	34	A:1	AC:0				
chr28	11002454	2	38	AAACAAC:0.5	A:0.5				

chr28 11002457 2 38 C:0.631579 T:0.368421
chr28 11002463 2 38 C:0.5 T:0.5
chr28 11002469 2 38 CAACAACAAT:0.631579 C:
0.368421
chr28 11003002 4 38 A:0.0526316 AT:0.394737
ATT:0.552632 ATTTT:0
chr28 11003098 2 40 A:0.975
AACTAAATTTTGCTTATTAATAAAATTTTAAAC:0.025
chr28 11003273 2 38 AAAAT:1 A:0
chr28 11003288 2 38 A:1 AT:0
chr28 11003289 2 38 TAAA:0.894737 T:0.105263
chr28 11003290 2 38 A:1 AATT:0
chr28 11003293 2 38 TA:0.894737 T:0.105263
chr28 11003294 2 38 A:0.973684 T:0.0263158
chr28 11003334 2 38 A:1 C:0
chr28 11003363 2 38 A:0.921053 G:0.0789474
chr28 11003468 2 40 GTTTCTTTTCTTCC:0.975 G:0.025
chr28 11003555 2 40 T:0.425 TCTA:0.575
chr28 11003575 2 40 A:0.9 G:0.1
chr28 11003578 2 40 C:1 T:0
chr28 11003603 2 36 C:0.805556 CT:0.194444
chr28 11003607 2 38 T:1 C:0
chr28 11003633 3 38 C:0.131579 CAGAGAG:0.868421
CAGAGAGAGAG:0
chr28 11003635 2 40 G:1 GAGAGAA:0
chr28 11003644 2 38 T:0.157895 TGA:0.842105
chr28 11003846 2 28 C:0.0714286 CTT:0.928571
chr28 11004025 2 34 G:0.970588 A:0.0294118
chr28 11004046 2 36 G:0.972222 A:0.0277778
chr28 11004192 2 38 C:0.894737 T:0.105263
chr28 11004311 2 38 G:0.105263 A:0.894737
chr28 11004606 2 36 A:0.972222 G:0.0277778
chr28 11004722 2 38 G:0.842105 A:0.157895
chr28 11004801 2 36 C:1 A:0
chr28 11005123 2 38 T:0.0789474 C:0.921053
chr28 11005235 2 36 C:0.0555556 T:0.944444
chr28 11005298 2 38 A:0.921053 G:0.0789474
chr28 11005327 2 40 G:1 C:0
chr28 11005435 2 40 T:0.925 C:0.075
chr28 11005509 2 36 G:1 A:0
chr28 11005663 2 38 G:0.552632 C:0.447368
chr28 11005676 2 38 G:0.0526316 A:0.947368
chr28 11006028 2 38 T:0.894737 C:0.105263
chr28 11006142 2 38 C:0.131579 T:0.868421
chr28 11006203 2 38 T:0.131579 C:0.868421
chr28 11006223 4 40 C:0.1 CTGAA:0.025
CTGAATGAACGAA:0.875 CTGAATGAACGAATGAA:0
chr28 11006246 2 40 G:0.125 A:0.875
chr28 11006397 2 38 C:0.921053 CT:0.0789474
chr28 11006726 2 36 T:0.0555556 C:0.944444
chr28 11006737 2 38 AC:0.684211 A:0.315789
chr28 11006739 2 36 C:0.416667 T:0.583333
chr28 11006741 2 38 C:0.684211 T:0.315789
chr28 11006750 2 40 G:0.125 A:0.875

chr28	11006751	2	40	C:0.125	T:0.875	
chr28	11006837	2	38	A:0.684211	C:0.315789	
chr28	11006936	2	36	C:0.0555556	T:0.944444	
chr28	11006979	2	38	A:0.947368	G:0.0526316	
chr28	11006991	2	38	A:0.947368	G:0.0526316	
chr28	11007002	2	38	G:0.947368	GGA:0.0526316	
chr28	11007058	2	40	A:0.9	T:0.1	
chr28	11007138	2	38	C:0.973684	T:0.0263158	
chr28	11007215	2	38	T:0.0526316	C:0.947368	
chr28	11007601	2	38	A:0.921053	C:0.0789474	
chr28	11007664	2	36	A:0.944444	T:0.0555556	
chr28	11007697	2	38	G:0.921053	T:0.0789474	
chr28	11007727	2	38	AG:0.894737	A:0.105263	
chr28	11007782	2	36	A:0.944444	G:0.0555556	
chr28	11007923	2	38	C:0.0789474	G:0.921053	
chr28	11008346	2	38	T:0.0789474	C:0.921053	
chr28	11008356	2	38	G:0.0789474	GC:0.921053	
chr28	11008376	2	38	A:0.0789474	G:0.921053	
chr28	11008391	2	38	T:0.0789474	A:0.921053	
chr28	11008404	2	38	T:0.763158	G:0.236842	
chr28	11008522	2	36	A:0.0833333	G:0.916667	
chr28	11008580	2	36	G:0.0833333	A:0.916667	
chr28	11008589	2	36	A:0.0833333	G:0.916667	
chr28	11008669	2	38	AT:0.131579	A:0.868421	
chr28	11008980	2	40	A:0.85	G:0.15	
chr28	11008999	2	40	G:0.15	A:0.85	
chr28	11009011	2	40	AG:0.95	A:0.05	
chr28	11009048	2	38	T:0.921053	C:0.0789474	
chr28	11009258	2	40	G:0.9	A:0.1	
chr28	11009314	2	38	G:0.868421	A:0.131579	
chr28	11009484	2	40	G:0.1	C:0.9	
chr28	11009729	2	40	G:1	A:0	
chr28	11009792	2	36	T:0.111111	C:0.888889	
chr28	11009928	2	40	A:1	C:0	
chr28	11010161	2	36	A:0.111111	G:0.888889	
chr28	11010227	2	30	C:0.966667	A:0.0333333	
chr28	11010228	2	32	A:1	C:0	
chr28	11010301	2	32	G:0.03125	C:0.96875	
chr28	11010623	2	36	C:0.111111	T:0.888889	
chr28	11010763	2	38	A:0.894737	G:0.105263	
chr28	11010769	2	38	A:0.894737	G:0.105263	
chr28	11010775	2	38	AAG:1	A:0	
chr28	11010793	2	38	C:1	T:0	
chr28	11010810	2	36	C:0.916667	CCTTGGG:0.0833333	
chr28	11010926	2	40	T:0.875	C:0.125	
chr28	11010949	2	40	A:0.875	T:0.125	
chr28	11011061	2	34	C:1	T:0	
chr28	11011219	2	40	T:0.125	C:0.875	
chr28	11011252	2	38	G:0.789474	A:0.210526	
chr28	11011267	2	38	G:0.157895	A:0.842105	
chr28	11011340	3	38	TAA:0.0526316	T:0.868421	TAAAAAA:0.0789474
chr28	11011350	2	38	TAAAA:0.131579	T:0.868421	
chr28	11011559	2	38	G:0.131579	A:0.868421	

chr28	11011876	2	32	C:0.96875	T:0.03125		
chr28	11011927	2	38	C:0.263158	CCT:0.736842		
chr28	11011978	3	38	T:0.421053	TA:0.210526	TAA:0.368421	
chr28	11012037	2	40	A:0.225	ATGTAGGCAGAGG:0.775		
chr28	11012378	2	40	A:0.875	G:0.125		
chr28	11012386	2	40	A:0.875	G:0.125		
chr28	11012542	2	38	G:0.921053	C:0.0789474		
chr28	11013044	2	36	A:0.138889	C:0.861111		
chr28	11013094	2	34	G:0.794118	T:0.205882		
chr28	11013431	2	32	G:0.75	T:0.25		
chr28	11013511	2	34	C:0.117647	T:0.882353		
chr28	11013592	2	28	G:0.714286	A:0.285714		
chr28	11013647	2	26	TTG:0.230769	T:0.769231		
chr28	11013660	2	26	G:0.230769	C:0.769231		
chr28	11014025	2	34	C:0.0882353	G:0.911765		
chr28	11014797	2	36	A:0.916667	G:0.0833333		
chr28	11015379	2	38	T:0.0789474	C:0.921053		
chr28	11015536	2	38	C:1	T:0		
chr28	11015724	2	36	C:0.916667	T:0.0833333		
chr28	11015901	2	38	A:0.973684	T:0.0263158		
chr28	11015942	4	38	C:0.526316	CAG:0.210526	CAGAGAG:0.184211	CAGAGAGAG:0.0789474
chr28	11015944	4	40	C:0.25	G:0.45	CAGAGAGAG:0.1	CAGAGAGAGAG:0.2
chr28	11015946	2	38	C:0.131579	G:0.868421		
chr28	11016331	2	36	C:0.888889	T:0.111111		
chr28	11016384	2	36	C:0.916667	T:0.0833333		
chr28	11016567	2	36	C:1	T:0		
chr28	11016701	2	30	GGGT:0.6	G:0.4		
chr28	11016702	2	30	GGT:0.466667	G:0.533333		
chr28	11017185	2	34	T:0.970588	C:0.0294118		
chr28	11017239	2	40	G:0.525	A:0.475		
chr28	11017242	2	40	G:0.6	A:0.4		
chr28	11017398	2	38	T:0.973684	C:0.0263158		
chr28	11017488	2	38	C:0.473684	G:0.526316		
chr28	11017968	2	40	C:0.7	T:0.3		
chr28	11018133	2	38	C:0.605263	T:0.394737		
chr28	11018217	2	40	C:0.975	T:0.025		
chr28	11018324	2	38	GT:1	G:0		
chr28	11018561	2	38	C:0.815789	T:0.184211		
chr28	11018591	2	38	C:0.973684	T:0.0263158		
chr28	11018792	2	34	A:0.352941	C:0.647059		
chr28	11018807	3	40	TTTTA:0.75	T:0.1	TTTTATTTA:0.15	
chr28	11018811	2	40	AT:0.975	A:0.025		
chr28	11019252	2	40	G:1	A:0		
chr28	11019519	2	38	A:0.342105	G:0.657895		
chr28	11019735	2	40	C:0.05	T:0.95		
chr28	11020123	2	38	G:0.789474	A:0.210526		
chr28	11020179	2	38	T:0.657895	C:0.342105		
chr28	11020202	2	38	GT:0.605263	G:0.394737		
chr28	11020349	2	34	G:0.382353	GT:0.617647		
chr28	11020376	2	32	C:0.75	T:0.25		

chr28	11020781	2	32	G:0.9375	A:0.0625	
chr28	11021012	2	40	G:0.35	T:0.65	
chr28	11021017	2	40	G:0.35	A:0.65	
chr28	11021468	2	38	G:0.947368		A:0.0526316
chr28	11021915	2	38	T:0.0789474		G:0.921053
chr28	11022233	2	38	C:0.394737		T:0.605263
chr28	11022346	2	40	A:0.675	G:0.325	
chr28	11022767	2	38	A:1	G:0	
chr28	11023439	2	40	G:0.675	T:0.325	
chr28	11023556	2	38	C:0.763158		T:0.236842
chr28	11023666	2	38	T:0.342105		C:0.657895
chr28	11023689	2	38	T:0.342105		C:0.657895
chr28	11023703	2	38	G:1	A:0	
chr28	11023783	2	38	C:1	T:0	
chr28	11024066	2	38	G:0.315789		C:0.684211
chr28	11024127	2	38	T:0.947368		A:0.0526316
chr28	11024250	2	40	T:0.625	G:0.375	
chr28	11024596	2	36	C:1	T:0	
chr28	11024783	2	34	A:0.382353		AC:0.617647
chr28	11025055	2	40	C:1	T:0	
chr28	11025116	2	40	T:0.6	G:0.4	
chr28	11025149	2	40	CA:1	C:0	
chr28	11025326	2	40	AG:0.675	A:0.325	
chr28	11025647	2	38	G:0.789474		A:0.210526
chr28	11025713	2	40	C:0.55	T:0.45	
chr28	11026043	2	40	C:0.3	CTA:0.7	
chr28	11026217	2	36	G:0.666667		GC:0.333333
chr28	11026485	2	36	G:0.972222		A:0.0277778
chr28	11026700	2	38	T:0.394737		C:0.605263
chr28	11026905	2	38	G:0.973684		A:0.0263158
chr28	11027254	2	40	G:0.55	A:0.45	
chr28	11027319	2	38	C:1	T:0	
chr28	11027331	2	40	TG:0.65	T:0.35	
chr28	11027534	2	38	C:1	T:0	
chr28	11027768	2	38	C:0.5	G:0.5	
chr28	11027837	2	36	T:0.472222		C:0.527778
chr28	11028083	2	38	T:0.710526		G:0.289474
chr28	11028211	2	40	T:0.95	C:0.05	
chr28	11028231	2	40	A:0.95	G:0.05	
chr28	11028232	2	40	G:0.95	C:0.05	
chr28	11028451	2	40	T:0.3	C:0.7	
chr28	11028454	2	40	C:0.3	T:0.7	
chr28	11028918	2	40	A:0.825	G:0.175	
chr28	11029112	2	36	C:0.916667		T:0.0833333
chr28	11029411	2	40	C:0.675	T:0.325	
chr28	11029473	2	38	T:0.736842		A:0.263158
chr28	11029699	2	36	C:0.694444		T:0.305556
chr28	11029723	2	36	T:1	C:0	
chr28	11029790	2	20	G:0.55	C:0.45	
chr28	11029936	2	32	A:0.46875		G:0.53125
chr28	11030002	2	34	A:0.470588		G:0.529412
chr28	11030312	2	38	T:0.657895		C:0.342105
chr28	11030318	2	38	G:1	T:0	
chr28	11030336	2	40	A:1	G:0	

chr28 11030502 2 38 C:0.631579 CT:0.368421
chr28 11030527 2 40 C:1 T:0
chr28 11030746 2 40 T:0.325 C:0.675
chr28 11031145 2 40 G:0.425 A:0.575
chr28 11031243 2 36 A:0.638889 G:0.361111
chr28 11031297 2 38 A:0.315789 G:0.684211
chr28 11031331 2 38 C:0.368421 T:0.631579
chr28 11031449 2 40 G:0.65 A:0.35
chr28 11031494 2 40 C:0.45 G:0.55
chr28 11031525 2 38 G:0.394737 A:0.605263
chr28 11031693 2 40 G:1 A:0
chr28 11031743 2 38 A:0.526316 C:0.473684
chr28 11031794 2 40 T:1 C:0
chr28 11031909 2 36 T:0.638889 A:0.361111
chr28 11031910 2 36 C:0.638889 T:0.361111
chr28 11031948 2 38 T:0.552632 C:0.447368
chr28 11031967 2 38 C:0.421053 T:0.578947
chr28 11032012 2 40 C:1 T:0
chr28 11032121 2 40 GTATAAGA:0.575 G:0.425
chr28 11032172 2 38 T:0.421053 C:0.578947
chr28 11032245 2 40 T:0.475 G:0.525
chr28 11032317 2 24 T:0.0833333 A:0.916667
chr28 11032686 2 36 C:0.638889 CTTT:0.361111
chr28 11032702 4 40 TTTTA:0.35 T:0.075
TTTTATTTA:0.325 TTTTATTTATTTATTTATTTA:0.25
chr28 11032740 2 36 C:0.527778 CAG:0.472222
chr28 11032795 2 34 G:0.588235 A:0.411765
chr28 11032824 2 38 C:0.578947 T:0.421053
chr28 11032875 2 34 T:0.617647 C:0.382353
chr28 11032919 2 40
TCTTTATCTAGTATGATCTCTAGACACTTTCTTTGC:1 T:0
chr28 11032957 2 40 T:1 C:0
chr28 11033126 2 38 C:0.526316 T:0.473684
chr28 11033139 2 38 G:0.526316 A:0.473684
chr28 11033239 2 40 A:1 C:0
chr28 11033276 2 40 T:0.7 C:0.3
chr28 11033358 2 38 G:0.973684 A:0.0263158
chr28 11033557 2 40 C:0.825 T:0.175
chr28 11033749 2 40 T:0.575 G:0.425
chr28 11033753 2 40 T:0.575 C:0.425
chr28 11034008 2 38 C:0.5 T:0.5
chr28 11034015 2 38 G:0.157895 A:0.842105
chr28 11034065 2 38 C:1 T:0
chr28 11034156 2 38 C:0.815789 T:0.184211
chr28 11034325 2 38 G:0.447368 A:0.552632
chr28 11034469 2 30 C:0.8 CTTT:0.2
chr28 11034523 2 36 CTT:0.416667 C:0.583333
chr28 11034636 2 36 C:0.416667 T:0.583333
chr28 11034645 2 40 G:0.95 GT:0.05
chr28 11034647 2 40 GCCCCAACC:0.95 G:0.05
chr28 11034970 2 40 G:0.875 A:0.125
chr28 11035099 2 40 G:0.625 A:0.375
chr28 11035355 2 38 G:1 A:0
chr28 11035439 2 40 C:1 T:0

chr28	11035764	2	36	G:0.388889	A:0.611111
chr28	11035877	2	36	C:0.527778	T:0.472222
chr28	11036037	2	34	C:1	T:0
chr28	11036374	2	38	C:0.421053	T:0.578947
chr28	11036470	2	38	G:0.894737	A:0.105263
chr28	11036491	2	40	C:0.725	T:0.275
chr28	11036637	2	40	C:0.425	A:0.575
chr28	11036639	2	40	T:0.85	G:0.15
chr28	11036705	2	40	G:0.775	A:0.225
chr28	11036874	2	38	C:0.157895	G:0.842105
chr28	11037064	2	40	G:0.7	A:0.3
chr28	11037211	2	40	T:0.875	C:0.125
chr28	11037431	2	40	G:0.75	A:0.25
chr28	11037491	2	40	CTTTTCT:0.675	C:0.325
chr28	11037506	2	40	T:0.875	C:0.125
chr28	11037508	2	40	C:0.875	T:0.125
chr28	11037518	2	40	C:0.875	T:0.125
chr28	11037522	4	40	C:0.65	T:0.125
CTTTCTTTCTTTCTTCTTTTTTTCTTTCTTTCT:0.225					
CTTTCTTTCTTTCTTTCTTTCTTTCTTCTTTTTTTCTTTCTTTCTT:0					
chr28	11037524	2	40	T:0.875	C:0.125
chr28	11037538	2	40	A:0.775	T:0.225
chr28	11037815	2	38	G:0.421053	C:0.578947
chr28	11037838	2	40	C:0.875	T:0.125
chr28	11037916	2	38	A:0.736842	T:0.263158
chr28	11037997	2	34	G:0.911765	A:0.0882353
chr28	11038002	2	34	T:0.441176	A:0.558824
chr28	11038011	2	34	T:0	C:1
chr28	11038194	2	16	G:1	A:0
chr28	11038344	2	40	ATTCT:0.9	A:0.1
chr28	11038412	2	40	C:1	T:0
chr28	11038442	2	40	G:1	A:0
chr28	11038526	2	34	A:0.911765	G:0.0882353
chr28	11038578	2	38	G:0.842105	A:0.157895
chr28	11038649	2	36	A:0.805556	G:0.194444
chr28	11038656	2	36	A:0.805556	C:0.194444
chr28	11038726	2	40	G:0.8	A:0.2
chr28	11038746	2	40	CT:0.8	C:0.2
chr28	11038767	2	40	T:0.4	C:0.6
chr28	11038955	2	36	C:0.805556	T:0.194444
chr28	11039000	2	36	A:0.777778	T:0.222222
chr28	11039001	2	36	G:0.833333	C:0.166667
chr28	11039040	2	38	A:0.815789	G:0.184211
chr28	11039042	2	38	A:0.815789	G:0.184211
chr28	11039119	2	36	T:0.861111	G:0.138889
chr28	11039147	2	34	G:0.941176	A:0.0588235
chr28	11039175	2	38	G:0.842105	A:0.157895
chr28	11039213	2	38	G:0.815789	A:0.184211
chr28	11039336	2	38	C:0.842105	T:0.157895
chr28	11039421	2	38	G:0.842105	A:0.157895
chr28	11039444	2	38	A:0.842105	G:0.157895
chr28	11039502	2	40	G:0.85	GAAGTCCCCT:0.15
chr28	11039705	2	40	C:0.75	T:0.25
chr28	11039750	2	40	G:0.7	T:0.3

chr28	11039822	2	38	G:0.868421	A:0.131579
chr28	11039827	2	38	G:0.263158	C:0.736842
chr28	11039882	2	38	C:1	T:0
chr28	11039902	2	38	G:0.789474	A:0.210526
chr28	11039922	2	38	G:0.684211	A:0.315789
chr28	11039935	2	38	G:1	A:0
chr28	11039942	2	38	G:0.789474	A:0.210526
chr28	11040024	2	36	G:0.694444	A:0.305556
chr28	11040055	2	36	A:0.138889	C:0.861111
chr28	11040084	2	36	C:0.75	A:0.25
chr28	11040134	2	34	T:0.117647	C:0.882353
chr28	11040220	2	40	A:0.9	G:0.1
chr28	11040227	2	40	G:0.9	A:0.1
chr28	11040244	2	40	A:0.875	T:0.125
chr28	11040316	2	36	A:0.722222	G:0.277778
chr28	11040349	2	36	C:0.833333	T:0.166667
chr28	11040436	2	38	T:0.394737	C:0.605263
chr28	11040688	2	38	G:0.736842	C:0.263158
chr28	11040769	2	24	T:0.75	C:0.25
chr28	11040994	2	36	T:0.416667	C:0.583333
chr28	11041095	2	40	A:1	C:0
chr28	11041311	2	38	G:0.684211	A:0.315789
chr28	11041433	2	36	C:0.111111	T:0.888889
chr28	11041590	2	36	G:1	A:0
chr28	11041594	2	38	C:0.947368	T:0.0526316
chr28	11041634	2	40	A:0.75	G:0.25
chr28	11041754	2	38	C:1	T:0
chr28	11041778	2	38	T:0.552632	C:0.447368
chr28	11041959	2	34	T:0.382353	C:0.617647
chr28	11042130	2	36	C:0.472222	A:0.527778
chr28	11042173	2	38	G:0.684211	A:0.315789
chr28	11042503	2	36	C:0.75	T:0.25
chr28	11042585	2	40	T:0.75	A:0.25
chr28	11042646	2	38	T:0.552632	A:0.447368
chr28	11042665	2	36	A:0.694444	G:0.305556
chr28	11042676	2	36	A:0.527778	G:0.472222
chr28	11042708	2	32	T:0.0625	C:0.9375
chr28	11042715	2	34	T:0.676471	C:0.323529
chr28	11042733	2	36	C:0.694444	T:0.305556
chr28	11042792	2	38	A:0.710526	G:0.289474
chr28	11042800	2	38	C:0.105263	CA:0.894737
chr28	11042878	2	40	C:0.65	T:0.35
chr28	11042885	2	40	A:0.65	G:0.35
chr28	11042902	2	40	A:0.65	T:0.35
chr28	11042903	2	40	T:0.65	C:0.35
chr28	11042925	2	38	G:0.736842	GT:0.263158
chr28	11043013	2	40	C:0.775	T:0.225
chr28	11043039	2	40	G:0.725	C:0.275
chr28	11043040	2	40	G:0.725	T:0.275
chr28	11043061	2	40	A:0.7	G:0.3
chr28	11043217	2	40	C:0.725	T:0.275
chr28	11043288	2	38	T:0.368421	G:0.631579
chr28	11043364	2	40	C:0.525	T:0.475
chr28	11043430	2	40	A:0.775	G:0.225

chr28	11043466	2	40	C:0.5	T:0.5	
chr28	11043573	2	40	G:0.575	A:0.425	
chr28	11043743	2	40	T:0.5	C:0.5	
chr28	11043747	2	40	G:0.5	A:0.5	
chr28	11043767	2	40	C:1	A:0	
chr28	11043779	2	34	A:0.5	G:0.5	
chr28	11043785	2	34	G:0.852941	A:0.147059	
chr28	11043879	2	40	C:0.6	A:0.4	
chr28	11043889	2	40	C:0.6	G:0.4	
chr28	11043968	2	38	C:0.789474	A:0.210526	
chr28	11044034	2	38	C:0.473684	T:0.526316	
chr28	11044071	2	38	A:0.710526	G:0.289474	
chr28	11044072	2	38	T:0.710526	C:0.289474	
chr28	11044133	2	40	A:0.425	ACCCAGGGGTAAG:0.575	
chr28	11044260	2	38	G:0.342105	C:0.657895	
chr28	11044291	2	38	G:1	A:0	
chr28	11044351	2	36	A:0.388889	T:0.611111	
chr28	11044353	2	36	C:1	T:0	
chr28	11044494	2	40	G:0.525	A:0.475	
chr28	11044534	2	38	A:0.789474	G:0.210526	
chr28	11044630	2	34	G:0.911765	C:0.0882353	
chr28	11044814	2	38	CCT:0.815789	C:0.184211	
chr28	11044910	2	40	G:1	A:0	
chr28	11044915	2	38	G:0.763158	T:0.236842	
chr28	11044953	2	40	T:0.875	TGAGACTGAATTTTTTTTTTTTTTTTTTGGAA:0.125	
chr28	11044981	3	38	G:0.763158	GGT:0.184211	GGTGT:0.0526316
chr28	11044996	2	36	GTA:0.944444	G:0.0555556	
chr28	11044998	2	36	A:0.666667	G:0.333333	
chr28	11045201	2	38	C:1	T:0	
chr28	11045229	2	36	G:0.861111	T:0.138889	
chr28	11045441	2	38	G:0.868421	C:0.131579	
chr28	11045483	2	40	G:1	A:0	
chr28	11045564	2	40	T:0.575	C:0.425	
chr28	11045598	2	40	G:0.825	A:0.175	
chr28	11045608	2	40	A:0.375	T:0.625	
chr28	11045649	2	40	A:0.125	C:0.875	
chr28	11045652	2	40	C:0.525	T:0.475	
chr28	11045820	2	38	C:0.684211	T:0.315789	
chr28	11045837	2	38	G:0.763158	A:0.236842	
chr28	11045846	2	38	C:0.578947	T:0.421053	
chr28	11045875	2	36	T:0.472222	C:0.527778	
chr28	11045956	2	38	G:1	A:0	
chr28	11046093	2	40	A:0.6	T:0.4	
chr28	11046283	2	40	C:0.8	G:0.2	
chr28	11046314	3	40	TTTTA:0.75	T:0.15	TTTTATTTA:0.1
chr28	11046366	2	38	T:0.526316	TTTTATTTATTTATTTATTTATTTA:0.473684	
chr28	11046734	2	40	C:0.525	T:0.475	
chr28	11046745	2	40	C:0.525	T:0.475	
chr28	11046806	2	36	A:1	G:0	
chr28	11046821	2	34	G:0.911765	A:0.0882353	

chr28	11047302	2	38	C:0.526316	T:0.473684	
chr28	11047309	2	38	C:1	T:0	
chr28	11047492	2	40	G:0.55	A:0.45	
chr28	11047710	2	38	C:0.947368	T:0.0526316	
chr28	11047757	2	40	A:0.125	G:0.875	
chr28	11047883	2	36	G:0.111111	T:0.888889	
chr28	11047960	2	38	A:1	T:0	
chr28	11047997	2	38	T:0.526316	G:0.473684	
chr28	11048566	2	38	A:0.947368	C:0.0526316	
chr28	11048570	2	40	A:0.425	G:0.575	
chr28	11048798	2	40	A:0.1	G:0.9	
chr28	11048852	2	40	C:1	T:0	
chr28	11048868	2	38	C:0.105263	A:0.894737	
chr28	11048974	2	40	C:0.625	A:0.375	
chr28	11049105	2	38	C:1	T:0	
chr28	11049165	2	34	GA:0.147059	G:0.852941	
chr28	11049177	2	34	A:0.147059	G:0.852941	
chr28	11049178	2	34	A:0.147059	T:0.852941	
chr28	11049225	2	38	C:1	T:0	
chr28	11049282	2	36	A:0.944444	AG:0.0555556	
chr28	11049309	3	38	TAAAAACAAAAAC:0.605263	T:0	TAAAAAC:0.394737
chr28	11049314	2	38	A:0.526316	G:0.473684	
chr28	11049392	2	36	C:0.5	CCTT:0.5	
chr28	11049477	2	38	C:0.526316	G:0.473684	
chr28	11049681	2	34	T:0.970588	C:0.0294118	
chr28	11049791	2	34	T:1	C:0	
chr28	11049831	2	40	T:0.6	C:0.4	
chr28	11049855	2	40	T:0.6	C:0.4	
chr28	11050086	2	40	A:0.625	C:0.375	
chr28	11050104	2	40	C:0.625	T:0.375	
chr28	11050151	2	40	T:0.525	C:0.475	
chr28	11050468	2	36	A:1	G:0	
chr28	11050712	2	40	G:0.95	A:0.05	
chr28	11050994	2	40	T:0.175	A:0.825	
chr28	11051063	2	38	C:0.763158	T:0.236842	
chr28	11051264	2	38	C:0.5	T:0.5	
chr28	11051347	2	30	AT:1	A:0	
chr28	11051551	2	36	AC:1	A:0	
chr28	11051910	2	24	C:0.625	CT:0.375	
chr28	11051955	2	34	C:1	T:0	
chr28	11052728	2	38	C:1	T:0	
chr28	11052738	2	38	C:1	T:0	
chr28	11052817	2	38	T:1	C:0	
chr28	11052838	2	38	TTTTCTTTC:0.947368	T:0.0526316	
chr28	11052898	2	38	CTTTCTTTCTTTCT:0.973684	C:0.0263158	
chr28	11052902	2	38	CTTTCTTTCT:0.921053	C:0.0789474	
chr28	11052911	2	40	T:0.95	TTTC:0.05	
chr28	11053169	2	36	C:1	T:0	
chr28	11053300	2	38	G:1	A:0	
chr28	11054904	2	38	T:0.815789	C:0.184211	

chr28	11055340	3	38	CT:0.368421	C:0.394737	CTT:0.236842	
chr28	11057389	2	38	A:0.842105	G:0.157895		
chr28	11057902	2	40	T:1	C:0		
chr28	11058160	2	38	C:1	T:0		
chr28	11058630	4	36	ATTT:0.111111	A:0.416667	AT:0.444444	ATT:0.0277778
chr28	11058660	2	34	T:1	C:0		
chr28	11059767	2	36	T:0.5	TG:0.5		
chr28	11060285	2	38	C:0.973684	T:0.0263158		
chr28	11060514	2	38	C:0.105263	T:0.894737		
chr28	11060525	2	36	C:0.972222	CA:0.0277778		
chr28	11061225	2	38	G:0.947368	A:0.0526316		
chr28	11062055	2	40	T:0.6	A:0.4		
chr28	11062158	2	34	G:0.852941	A:0.147059		
chr28	11062245	2	36	T:0.611111	C:0.388889		
chr28	11062255	2	36	A:0.111111	C:0.888889		
chr28	11062310	2	40	G:0.8	A:0.2		
chr28	11062417	2	38	T:0.789474	TA:0.210526		
chr28	11062567	2	40	T:0.775	C:0.225		
chr28	11062762	2	40	C:0.775	T:0.225		
chr28	11062783	2	40	C:0.775	G:0.225		
chr28	11062834	2	40	A:0.225	T:0.775		
chr28	11062874	2	38	G:0.973684	C:0.0263158		
chr28	11063078	2	40	A:0.05	T:0.95		
chr28	11063083	2	40	T:0.825	G:0.175		
chr28	11063122	2	40	T:0.775	C:0.225		
chr28	11063134	2	40	T:0.775	C:0.225		
chr28	11063207	2	34	C:0.852941	T:0.147059		
chr28	11063288	2	40	T:0.8	A:0.2		
chr28	11063324	2	40	G:0.825	A:0.175		
chr28	11063335	2	40	C:0.85	T:0.15		
chr28	11063349	2	40	A:0.825	G:0.175		
chr28	11063403	2	40	A:0.825	G:0.175		
chr28	11063568	2	38	G:1	A:0		
chr28	11063647	2	40	A:0.2	G:0.8		
chr28	11063753	2	38	C:0.131579	A:0.868421		
chr28	11063772	2	38	TGTTA:0.131579	T:0.868421		
chr28	11063953	2	38	G:0.210526	GTGTC:0.789474		
chr28	11064493	2	36	T:0.166667	C:0.833333		
chr28	11064863	3	38	TAA:0.157895	T:0.5	TA:0.342105	
chr28	11064942	2	36	T:0.972222	A:0.0277778		
chr28	11065209	2	30	A:0.533333	G:0.466667		
chr28	11066114	2	34	CT:0.0882353	C:0.911765		
chr28	11066583	2	36	A:0.861111	G:0.138889		
chr28	11066856	2	36	A:0	T:1		
chr28	11066857	2	36	A:0	G:1		
chr28	11066898	2	34	A:0	G:1		
chr28	11066926	2	40	A:0.6	AC:0.4		
chr28	11067087	2	36	A:1	G:0		
chr28	11067397	2	38	C:0.921053	T:0.0789474		
chr28	11067465	2	40	T:0.05	C:0.95		
chr28	11067559	2	38	G:0.868421	T:0.131579		

chr28	11067786	2	40	T:0.675	C:0.325	
chr28	11068293	2	36	A:0.805556	G:0.194444	
chr28	11068294	2	36	G:0.0833333	GGA:0.916667	
chr28	11068433	2	34	T:0	G:1	
chr28	11068476	2	38	CTG:0.894737	C:0.105263	
chr28	11068486	2	38	T:0	C:1	
chr28	11068487	2	38	T:0	C:1	
chr28	11068648	2	34	AT:0.823529	A:0.176471	
chr28	11068658	2	34	T:1	A:0	
chr28	11069264	2	40	G:0.975	C:0.025	
chr28	11069542	2	40	G:1	C:0	
chr28	11069630	2	40	GCTGGCAAGTGCCTGGGGC:0.875	G:0.125	
chr28	11069722	2	40	G:0.95	A:0.05	
chr28	11070083	2	40	AT:0.975	A:0.025	
chr28	11070732	2	34	C:1	T:0	
chr28	11070951	2	40	GT:0.55	G:0.45	
chr28	11071678	2	38	T:0.105263	C:0.894737	
chr28	11071878	2	38	C:0.947368	T:0.0526316	
chr28	11071976	2	36	C:0.888889	T:0.111111	
chr28	11072114	2	36	A:0.638889	T:0.361111	
chr28	11072137	2	36	A:0.583333	G:0.416667	
chr28	11072430	2	36	T:0.527778	C:0.472222	
chr28	11072492	3	30	CTT:0.433333	C:0.266667	CT:0.3
chr28	11072543	2	38	GAC:0.842105	G:0.157895	
chr28	11072547	3	38	CACAG:0.657895	C:0.105263	CAGAGAGAGACAG:0.236842
chr28	11072549	3	38	CAGAGAG:0.552632	C:0.0526316	GAGAGAG:0.394737
chr28	11072840	2	34	G:0.117647	T:0.882353	
chr28	11073413	2	36	T:0.527778	A:0.472222	
chr28	11073415	2	36	G:0.527778	A:0.472222	
chr28	11073659	2	38	C:1	T:0	
chr28	11073995	2	32	GCTT:0.4375	G:0.5625	
chr28	11073998	2	34	T:0.941176	G:0.0588235	
chr28	11073999	2	34	CTT:0.941176	C:0.0588235	
chr28	11074002	2	38	T:0.921053	C:0.0789474	
chr28	11074091	2	36	A:0.944444	G:0.0555556	
chr28	11074125	2	38	A:0.842105	G:0.157895	
chr28	11074183	2	36	C:0.972222	T:0.0277778	
chr28	11074258	2	38	C:1	T:0	
chr28	11074371	2	38	C:0.947368	CG:0.0526316	
chr28	11074479	2	38	A:1	G:0	
chr28	11074654	2	40	G:0.925	A:0.075	
chr28	11074671	2	40	GGC:0.575	G:0.425	
chr28	11074800	2	38	G:0.921053	A:0.0789474	
chr28	11074844	2	38	GGT:0.815789	G:0.184211	
chr28	11074860	2	38	G:0.815789	A:0.184211	
chr28	11074980	2	38	G:1	C:0	
chr28	11075230	2	32	A:0.9375	G:0.0625	
chr28	11075318	2	36	A:0.944444	G:0.0555556	
chr28	11075428	2	36	C:0.916667	T:0.0833333	
chr28	11075439	2	36	C:0.916667	G:0.0833333	
chr28	11075530	2	38	C:0.973684	T:0.0263158	

chr28 11075544 2 38 ATAAGT:0.973684 A:0.0263158
chr28 11075654 2 36 A:0.805556 AT:0.194444
chr28 11075655 2 36 A:0.805556 T:0.194444
chr28 11075670 2 38 C:0.131579 T:0.868421
chr28 11075741 3 38 TTTATTTA:0.578947 TTTTA:
0.289474 T:0.131579
chr28 11075748 3 40 ATTTTT:0.325 A:0.275 ATTTTTT:
0.4
chr28 11075791 2 36 A:0.944444 C:0.0555556
chr28 11075888 2 34 G:0.794118 C:0.205882
chr28 11075926 2 38 A:0.131579 G:0.868421
chr28 11075938 2 38 T:0.763158 G:0.236842
chr28 11075981 2 36 T:1 TC:0
chr28 11076189 2 38 AT:0.973684 A:0.0263158
chr28 11076227 2 40 G:0.925 T:0.075
chr28 11076236 2 40 T:0.925 TG:0.075
chr28 11076317 2 40 G:0.925 C:0.075
chr28 11076385 2 40 G:1 A:0
chr28 11076630 2 36 T:0.916667 C:0.0833333
chr28 11076714 2 38 G:0.947368 A:0.0526316
chr28 11076770 2 38 G:0.921053 A:0.0789474
chr28 11076911 2 40 G:0.95 A:0.05
chr28 11076921 2 40 T:0.925 A:0.075
chr28 11076937 2 40 C:0.95 T:0.05
chr28 11076994 2 40 GCACTGC:0.975 G:0.025
chr28 11077054 2 38 C:1 A:0
chr28 11077133 2 40 C:0.65 T:0.35
chr28 11077297 2 38 C:0.789474 T:0.210526
chr28 11077318 2 38 A:0.131579 G:0.868421
chr28 11077319 2 38 T:0.973684 C:0.0263158
chr28 11077447 2 40 T:0.95 TCCAGGCACAGAGCTACCAA:0.05
chr28 11077561 2 38 C:0.973684 T:0.0263158
chr28 11077680 2 40 CTT:0.95 C:0.05
chr28 11077780 2 40 G:0.975 A:0.025
chr28 11077863 2 40 C:0.6 A:0.4
chr28 11077910 2 40 G:1 C:0
chr28 11078064 2 40 GA:0.925 G:0.075
chr28 11078201 2 38 T:0.578947 C:0.421053
chr28 11078236 2 40 A:0.95 G:0.05
chr28 11078310 2 36 A:0.583333 G:0.416667
chr28 11078317 2 36 A:0.916667 C:0.0833333
chr28 11078404 2 36 C:0.0555556 CG:0.944444
chr28 11078422 2 36 C:0.833333 CGGGCTGCTGTG:
0.166667
chr28 11078501 2 38 A:0.894737 C:0.105263
chr28 11078804 2 40 T:0.2 C:0.8
chr28 11078812 2 40 CGGAGGGAGGGTGACTGGCCCG:0.9
C:0.1
chr28 11078843 2 40 A:0.025 G:0.975
chr28 11078939 2 40 C:0.75 T:0.25
chr28 11078991 2 38 A:0.763158 G:0.236842
chr28 11079200 2 40 A:0.75 G:0.25
chr28 11079493 2 40 A:0.625
AATGAGTGCGTGTGCATTCAGTGTAT:0.375

chr28	11079599	2	40	C:0.725	T:0.275	
chr28	11079620	2	40	G:0.875	A:0.125	
chr28	11079735	2	38	C:0.578947	T:0.421053	
chr28	11079947	2	40	T:0.8	C:0.2	
chr28	11080056	2	40	G:0.95	C:0.05	
chr28	11080401	2	36	A:0.861111	G:0.138889	
chr28	11080539	2	34	ACAG:0.970588	A:0.0294118	
chr28	11080543	2	34	G:0.970588	GTGATGA:0.0294118	
chr28	11080749	2	40	C:0.425	T:0.575	
chr28	11080756	2	40	T:0.825	C:0.175	
chr28	11080853	2	36	TG:0.972222	T:0.0277778	
chr28	11080976	2	40	T:0.875	G:0.125	
chr28	11081239	2	40	A:0.8	G:0.2	
chr28	11081330	2	38	CTG:1	C:0	
chr28	11081483	2	38	A:0.763158	AC:0.236842	
chr28	11081494	2	38	T:0.605263	G:0.394737	
chr28	11081540	2	36	C:0.75	T:0.25	
chr28	11081649	2	34	C:0.970588	T:0.0294118	
chr28	11081757	2	36	C:0.222222	G:0.777778	
chr28	11081919	2	38	C:0.736842	T:0.263158	
chr28	11081959	2	38	T:0.131579	C:0.868421	
chr28	11082040	2	40	T:0.75	C:0.25	
chr28	11082136	2	40	A:0.725	C:0.275	
chr28	11082244	2	40	C:0.025	T:0.975	
chr28	11082388	2	40	C:0.05	G:0.95	
chr28	11082442	2	38	C:0.0263158	T:0.973684	
chr28	11082920	2	40	G:0.825	A:0.175	
chr28	11083144	2	36	T:0.833333	C:0.166667	
chr28	11083204	3	40	C:0.8	G:0	T:0.2
chr28	11083338	2	40	C:0.575	CTG:0.425	
chr28	11083603	2	38	A:0.684211	T:0.315789	
chr28	11083870	2	40	C:0.45	T:0.55	
chr28	11083873	2	38	C:1	T:0	
chr28	11083874	2	38	G:1	A:0	
chr28	11083953	2	40	C:0.925	T:0.075	
chr28	11084408	2	38	C:0.894737	T:0.105263	
chr28	11084893	2	24	T:0.833333	TA:0.166667	
chr28	11084944	2	40	GAGAGAGAGAGAGCACGCGCC:0.6	G:0.4	
chr28	11085077	2	36	G:0.333333	A:0.666667	
chr28	11085221	2	36	T:0.5	C:0.5	
chr28	11085223	2	36	C:0.972222	A:0.0277778	
chr28	11085484	2	40	G:0.95	A:0.05	
chr28	11085598	2	40	GT:1	G:0	
chr28	11085630	2	40	A:1	G:0	
chr28	11085654	2	40	A:0.175	AGTGAAGTACTGTGG:0.825	
chr28	11085939	2	38	T:0.947368	C:0.0526316	
chr28	11086111	2	38	G:1	A:0	
chr28	11086130	2	36	C:0.666667	CG:0.333333	
chr28	11086334	2	38	C:0.473684	A:0.526316	
chr28	11086385	2	40	G:0.7	A:0.3	
chr28	11086761	2	38	C:0.5	T:0.5	
chr28	11086773	2	36	T:0.5	C:0.5	
chr28	11086780	2	38	C:0.973684	T:0.0263158	
chr28	11087050	2	38	T:0.526316	C:0.473684	

chr28	11087121	2	40	G:0.95	A:0.05		
chr28	11087143	2	40	T:0.975	C:0.025		
chr28	11087191	2	38	C:0.973684	CT:0.0263158		
chr28	11087197	3	38	A:0.0789474	G:0.894737	C:0.0263158	
chr28	11087228	2	40	G:0.875	T:0.125		
chr28	11087249	2	40	T:0.125	C:0.875		
chr28	11087269	3	40	A:0.1	G:0.875	C:0.025	
chr28	11087277	2	40	C:0.95	T:0.05		
chr28	11087278	2	40	G:0.925	A:0.075		
chr28	11087407	2	38	C:1	T:0		
chr28	11087609	2	34	C:0.911765	T:0.0882353		
chr28	11087639	2	38	C:0.763158	T:0.236842		
chr28	11087712	2	40	C:1	A:0		
chr28	11088107	2	38	T:0.921053	A:0.0789474		
chr28	11088113	2	38	A:0.684211	G:0.315789		
chr28	11088146	2	38	C:0.868421	T:0.131579		
chr28	11088216	2	38	T:0.552632	A:0.447368		
chr28	11088303	2	38	T:0.921053	A:0.0789474		
chr28	11088311	2	38	T:0.921053	A:0.0789474		
chr28	11088406	2	38	G:0.789474	T:0.210526		
chr28	11088443	2	38	C:0.947368	T:0.0526316		
chr28	11088646	2	38	G:0.973684	C:0.0263158		
chr28	11088889	2	38	C:0.184211	G:0.815789		
chr28	11088961	2	38	G:0.210526	T:0.789474		
chr28	11089447	2	36	T:0.805556	TG:0.194444		
chr28	11089675	2	36	T:0.972222	C:0.0277778		
chr28	11089868	2	38	ATGAT:1	A:0		
chr28	11090312	2	40	C:0.7	T:0.3		
chr28	11090385	2	38	C:0.394737	T:0.605263		
chr28	11090391	2	38	G:0.947368	A:0.0526316		
chr28	11090401	2	38	T:0.947368	C:0.0526316		
chr28	11090601	2	40	T:0.725	C:0.275		
chr28	11090646	2	36	G:0.75	A:0.25		
chr28	11090851	2	36	T:0.972222	C:0.0277778		
chr28	11091691	2	38	T:0.842105	C:0.157895		
chr28	11091957	2	38	A:0.342105	T:0.657895		
chr28	11091964	2	38	C:0.973684	T:0.0263158		
chr28	11091986	2	40	C:0.225	T:0.775		
chr28	11092072	2	38	G:0.763158	A:0.236842		
chr28	11092484	2	34	G:0.352941	A:0.647059		
chr28	11092673	2	38	C:0.973684	G:0.0263158		
chr28	11092734	2	36	G:0.333333	A:0.666667		
chr28	11092759	2	36	A:0.222222	G:0.777778		
chr28	11092773	4	36	CT:0.472222	C:0	CTT:0.361111	CTTT:0.166667
chr28	11092798	2	32	A:0.21875	G:0.78125		
chr28	11093193	2	32	T:0.9375	C:0.0625		
chr28	11093450	2	38	C:1	T:0		
chr28	11093451	2	38	G:1	A:0		
chr28	11093600	2	38	T:0.315789	C:0.684211		
chr28	11093730	2	38	C:0.973684	T:0.0263158		
chr28	11093772	2	40	A:0.15	C:0.85		
chr28	11094006	2	34	T:0.0882353	C:0.911765		

chr28	11094058	2	38	C:0.184211	T:0.815789
chr28	11094197	2	40	A:0.2	C:0.8
chr28	11094562	2	38	T:0.184211	C:0.815789
chr28	11094827	2	32	T:0	C:1
chr28	11094880	2	30	G:1	C:0
chr28	11095001	2	10	CTA:0.2	C:0.8
chr28	11095046	2	34	GAGGT:0.117647	G:0.882353
chr28	11095051	2	34	CATCACTGTTTCCTCGA:0.117647	C:0.882353
chr28	11095265	2	30	A:0.0333333	G:0.966667
chr28	11095422	2	30	C:1	T:0
chr28	11095450	2	30	T:0.1	C:0.9
chr28	11095473	2	22	G:0.363636	GC:0.636364
chr28	11095587	2	32	GC:0.59375	G:0.40625
chr28	11095611	2	32	T:0.96875	C:0.03125
chr28	11095636	2	34	T:0.0294118	TC:0.970588
chr28	11095641	2	34	T:0.0294118	C:0.970588
chr28	11095713	2	34	G:0.235294	A:0.764706
chr28	11095747	2	32	C:0.25	T:0.75
chr28	11096086	2	40	T:0.225	TC:0.775
chr28	11096095	2	40	T:0.225	C:0.775
chr28	11096168	2	38	T:0.105263	C:0.894737
chr28	11096230	2	38	C:0.947368	G:0.0526316
chr28	11096387	2	38	T:0.131579	C:0.868421
chr28	11096613	2	40	C:0.125	A:0.875
chr28	11096615	2	40	C:0.2	T:0.8
chr28	11096744	2	34	C:1	T:0
chr28	11096805	2	36	G:0.972222	A:0.0277778
chr28	11096887	2	40	C:0.125	T:0.875
chr28	11096899	2	40	TTATAA:0.225	T:0.775
chr28	11097168	2	34	C:0.941176	T:0.0588235
chr28	11097174	2	32	T:0.15625	C:0.84375
chr28	11097222	2	36	G:0.888889	T:0.111111
chr28	11097772	2	38	T:0.105263	C:0.894737
chr28	11097881	2	38	G:0.921053	T:0.0789474
chr28	11098026	2	40	A:0.825	C:0.175
chr28	11098304	2	36	G:0.916667	T:0.0833333
chr28	11098527	2	36	A:0.972222	G:0.0277778
chr28	11098587	2	38	A:0.0526316	G:0.947368
chr28	11098782	2	40	C:1	CCTT:0
chr28	11099365	2	34	G:0.823529	C:0.176471
chr28	11099488	2	34	GA:0.0882353	G:0.911765
chr28	11099513	2	36	G:0.833333	A:0.166667
chr28	11099641	2	34	G:0.823529	A:0.176471
chr28	11099763	2	36	T:0.916667	C:0.0833333
chr28	11099799	2	34	A:0.352941	G:0.647059
chr28	11100187	2	36	T:0.972222	C:0.0277778
chr28	11100307	2	40	T:0.15	C:0.85
chr28	11100470	2	40	T:0.925	C:0.075
chr28	11100601	2	38	G:0.789474	A:0.210526
chr28	11100610	2	38	G:0.947368	C:0.0526316
chr28	11100623	2	40	G:0.925	A:0.075
chr28	11100882	2	38	C:0.236842	T:0.763158
chr28	11101093	2	38	T:0.236842	C:0.763158

chr28	11101147	2	38	C:0.973684	T:0.0263158
chr28	11101263	2	40	T:0.925	TA:0.075
chr28	11101367	2	38	G:0.131579	C:0.868421
chr28	11101835	2	34	C:0.205882	T:0.794118
chr28	11101862	2	32	T:0.03125	TC:0.96875
chr28	11101892	2	30	A:0.933333	AC:0.0666667
chr28	11102125	2	36	C:0.194444	T:0.805556
chr28	11102168	2	38	C:1	T:0
chr28	11102205	2	36	C:1	T:0
chr28	11102340	2	40	G:0.25	A:0.75
chr28	11102341	2	40	C:0.9	T:0.1
chr28	11102815	2	34	C:1	T:0
chr28	11103125	2	40	G:0.25	A:0.75
chr28	11103373	2	40	C:0.95	G:0.05
chr28	11103479	2	40	C:0.125	T:0.875
chr28	11103607	2	38	G:0.210526	A:0.789474
chr28	11103837	2	40	A:0.55	AT:0.45
chr28	11104180	2	38	G:0.0526316	A:0.947368
chr28	11104411	2	38	G:0.105263	A:0.894737
chr28	11104762	2	36	A:0.944444	G:0.0555556
chr28	11104855	2	40	TTGTC:0.85	T:0.15
chr28	11105005	2	38	C:0.184211	A:0.815789
chr28	11105189	2	38	T:0.0789474	C:0.921053
chr28	11105214	2	38	G:0.789474	A:0.210526
chr28	11105290	2	40	C:1	A:0
chr28	11105354	2	36	T:0.138889	G:0.861111
chr28	11105413	2	38	T:0.394737	A:0.605263
chr28	11105483	2	40	C:0.9	T:0.1
chr28	11105707	2	40	C:0.85	T:0.15
chr28	11105854	2	34	A:0.323529	AT:0.676471
chr28	11105936	2	40	A:0.075	C:0.925
chr28	11106029	2	38	C:0.131579	T:0.868421
chr28	11106299	2	36	C:0.194444	T:0.805556
chr28	11106656	2	18	G:0.111111	C:0.888889
chr28	11106834	2	32	GA:0.96875	G:0.03125
chr28	11106854	2	32	GGAA:0.84375	G:0.15625
chr28	11107247	2	38	G:0.789474	T:0.210526
chr28	11107330	2	38	C:0.289474	G:0.710526
chr28	11107343	2	38	A:0.973684	AG:0.0263158
chr28	11107350	2	38	A:0.289474	C:0.710526
chr28	11108437	2	40	A:0.975	G:0.025
chr28	11109076	2	40	C:0.95	T:0.05
chr28	11109354	2	40	G:0.65	GCCCTCTGT:0.35
chr28	11109367	2	40	C:0.425	CTGTCCCTG:0.575
chr28	11109508	2	40	C:0.925	G:0.075
chr28	11109652	2	36	C:0.0277778	T:0.972222
chr28	11109667	2	38	G:0.815789	A:0.184211
chr28	11109813	2	36	G:0.75	T:0.25
chr28	11110093	2	38	C:0.315789	A:0.684211
chr28	11110396	2	40	C:0.75	T:0.25
chr28	11110523	2	40	T:0.725	G:0.275
chr28	11110596	2	40	A:0.975	C:0.025
chr28	11110844	2	40	G:0.95	A:0.05
chr28	11111059	2	38	G:0.789474	A:0.210526

chr28	11111267	2	38	T:0.973684	C:0.0263158	
chr28	11111378	2	40	T:0.975	TGCCAGTGGTCCCTGGGCCCCTCTTAGAGAA:0.025	
chr28	11111411	2	40	C:1	T:0	
chr28	11111442	2	40	C:0.8	T:0.2	
chr28	11111522	2	36	G:1	C:0	
chr28	11111683	2	40	G:0.975	A:0.025	
chr28	11111688	2	40	A:0.975	ACT:0.025	
chr28	11111767	2	40	C:0.975	CTGCCCATTT:0.025	
chr28	11111835	2	34	A:1	G:0	
chr28	11111999	2	40	G:1	A:0	
chr28	11112156	2	38	G:0.763158	GGT:0.236842	
chr28	11112381	2	38	G:0.973684	C:0.0263158	
chr28	11112479	2	40	T:0.975	A:0.025	
chr28	11112502	2	40	T:0.95	C:0.05	
chr28	11112579	2	38	A:0.947368	G:0.0526316	
chr28	11112915	2	38	T:1	C:0	
chr28	11112950	2	34	A:0.764706	C:0.235294	
chr28	11113095	2	36	C:0.972222	A:0.0277778	
chr28	11113144	2	40	C:0.975	G:0.025	
chr28	11113246	2	36	A:0.111111	G:0.888889	
chr28	11113345	2	40	CAGT:0.975	C:0.025	
chr28	11113470	2	40	C:0.825	T:0.175	
chr28	11113826	2	38	G:0.973684	C:0.0263158	
chr28	11113919	2	36	G:0.722222	T:0.277778	
chr28	11113945	2	38	G:0.710526	A:0.289474	
chr28	11114003	2	40	C:1	A:0	
chr28	11114052	2	40	CA:0.975	C:0.025	
chr28	11114214	2	40	T:0.1	TG:0.9	
chr28	11114250	2	40	G:0.675	GGGCATCA:0.325	
chr28	11114310	2	40	C:0.95	T:0.05	
chr28	11114488	2	40	G:0.075	A:0.925	
chr28	11114644	2	40	G:0.975	C:0.025	
chr28	11114680	2	38	C:0.894737	A:0.105263	
chr28	11114745	2	36	C:0.944444	G:0.0555556	
chr28	11114952	2	34	T:1	G:0	
chr28	11115466	2	38	GGTCATCACTCTTTT:1	G:0	
chr28	11116483	2	32	G:0.96875	A:0.03125	
chr28	11116748	2	34	G:0.794118	A:0.205882	
chr28	11116868	2	34	C:0.970588	T:0.0294118	
chr28	11117470	2	38	C:1	T:0	
chr28	11117661	2	38	C:0.973684	T:0.0263158	
chr28	11117865	2	38	C:0.0263158	G:0.973684	
chr28	11117881	2	38	T:0.947368	C:0.0526316	
chr28	11117882	2	38	G:0.710526	A:0.289474	
chr28	11117970	2	40	G:0.3	A:0.7	
chr28	11118123	2	40	G:0.75	T:0.25	
chr28	11118577	2	36	G:1	A:0	
chr28	11119076	3	40	G:0.9	GTGTATATATATA:0.1	GTGTATATATATATATATATATATATATATA:0
chr28	11119439	2	34	TA:0.323529	T:0.676471	
chr28	11119460	2	36	C:0.722222	T:0.277778	
chr28	11119719	2	40	AGCCGC:0.275	A:0.725	
chr28	11119758	2	38	C:1	T:0	

chr28	11119763	2	38	G:0.210526	A:0.789474	
chr28	11119851	2	38	A:0.973684	G:0.0263158	
chr28	11119940	2	38	G:0.947368	A:0.0526316	
chr28	11119970	2	40	G:0.75	A:0.25	
chr28	11120461	2	38	C:0.552632	T:0.447368	
chr28	11120469	2	38	T:0.0263158	G:0.973684	
chr28	11120511	2	36	C:0.75	T:0.25	
chr28	11120515	2	36	A:0.0555556	G:0.944444	
chr28	11120552	2	36	A:0.0555556	G:0.944444	
chr28	11120557	2	38	G:0.736842	A:0.263158	
chr28	11120715	2	38	GCCTC:0.763158	G:0.236842	
chr28	11120719	2	38	C:0.315789	G:0.684211	
chr28	11121395	2	34	C:1	T:0	
chr28	11122447	2	36	G:0.972222	A:0.0277778	
chr28	11122803	2	38	G:0.921053	A:0.0789474	
chr28	11122824	2	38	C:0.657895	T:0.342105	
chr28	11122877	2	38	G:0.315789	A:0.684211	
chr28	11122960	2	36	A:0.888889	C:0.111111	
chr28	11123223	2	32	AGTGT:0.4375	A:0.5625	
chr28	11123436	2	34	C:0.352941	T:0.647059	
chr28	11123464	3	20	G:0.15	GC:0.3	GCC:0.55
chr28	11123682	2	36	G:0.833333	A:0.166667	
chr28	11123715	2	34	T:0.941176	TAG:0.0588235	
chr28	11123739	2	32	A:0.71875	G:0.28125	
chr28	11123933	2	40	GTCAACCATCCAATGCA:0.775	G:0.225	
chr28	11124034	2	36	C:0.833333	T:0.166667	
chr28	11124145	2	38	T:0.736842	C:0.263158	
chr28	11124619	2	38	C:0.763158	T:0.236842	
chr28	11124663	2	40	AGGTGTCGCTATCTGCT:0.8	A:0.2	
chr28	11124729	2	38	T:0.736842	C:0.263158	
chr28	11124852	2	38	G:0.763158	C:0.236842	
chr28	11124920	2	36	G:0.916667	A:0.0833333	
chr28	11125055	2	34	G:0.970588	A:0.0294118	
chr28	11125076	2	36	T:0	C:1	
chr28	11125082	2	38	A:0.842105	G:0.157895	
chr28	11125296	2	34	G:1	C:0	
chr28	11125301	2	36	A:0.944444	AG:0.0555556	
chr28	11126251	2	38	G:0.0263158	A:0.973684	
chr28	11126751	2	40	A:0.725	T:0.275	
chr28	11127161	2	38	T:0.315789	TG:0.684211	
chr28	11127552	2	36	A:0.694444	G:0.305556	
chr28	11127885	2	36	T:0.305556	TG:0.694444	
chr28	11127933	2	38	T:0.263158	C:0.736842	
chr28	11128622	2	40	G:0.225	A:0.775	
chr28	11128738	2	36	C:1	T:0	
chr28	11128850	2	36	T:0.777778	C:0.222222	
chr28	11128854	2	38	C:0.973684	T:0.0263158	
chr28	11128957	2	36	C:0.944444	T:0.0555556	
chr28	11129294	2	38	C:0.973684	G:0.0263158	
chr28	11129364	2	38	G:0.263158	A:0.736842	
chr28	11129661	2	38	G:0.973684	A:0.0263158	
chr28	11129712	2	40	G:0.75	GAGA:0.25	
chr28	11129752	2	38	A:0.657895	G:0.342105	
chr28	11129795	2	36	C:0.611111	T:0.388889	

chr28	11129822	2	34	A:1	C:0	
chr28	11129834	2	36	C:0.75	G:0.25	
chr28	11129868	2	38	A:0.973684	G:0.0263158	
chr28	11130117	2	38	C:0.657895	T:0.342105	
chr28	11130274	2	38	A:0.710526	T:0.289474	
chr28	11130544	2	38	G:1	A:0	
chr28	11130804	2	34	C:0.794118	T:0.205882	
chr28	11130815	2	34	C:0.794118	T:0.205882	
chr28	11130859	2	34	G:0.794118	A:0.205882	
chr28	11131063	2	38	T:0.763158	C:0.236842	
chr28	11131224	2	40	A:0.775	T:0.225	
chr28	11131264	2	38	T:0.0526316	C:0.947368	
chr28	11131378	2	38	C:0.789474	T:0.210526	
chr28	11131401	2	38	G:0.789474	A:0.210526	
chr28	11131888	2	32	C:0.96875	T:0.03125	
chr28	11131963	2	36	C:0.194444	T:0.805556	
chr28	11132199	2	40	GAAGC:0.725	G:0.275	
chr28	11132234	2	36	C:0.777778	T:0.222222	
chr28	11132682	2	38	CTGGGA:0.684211	C:0.315789	
chr28	11132688	2	38	T:0.605263	C:0.394737	
chr28	11132734	2	38	G:0.973684	T:0.0263158	
chr28	11133031	2	38	A:0.710526	G:0.289474	
chr28	11133123	2	40	A:0.775	C:0.225	
chr28	11133233	2	36	T:0.75	C:0.25	
chr28	11133371	2	36	A:0.75	G:0.25	
chr28	11133787	2	38	T:0.5	G:0.5	
chr28	11133958	2	40	A:0.8	T:0.2	
chr28	11134127	2	40	C:0.6	T:0.4	
chr28	11134128	2	40	A:0.425	G:0.575	
chr28	11134207	2	40	G:0.725	GT:0.275	
chr28	11134396	2	40	C:0.775	T:0.225	
chr28	11134854	2	36	C:0.777778	G:0.222222	
chr28	11135111	2	40	A:0.725	T:0.275	
chr28	11135266	2	36	T:0.722222	C:0.277778	
chr28	11135305	2	36	A:0.694444	G:0.305556	
chr28	11135483	2	38	G:0.763158	T:0.236842	
chr28	11136856	2	38	T:0.368421	G:0.631579	
chr28	11137017	2	40	G:0.975	A:0.025	
chr28	11137148	2	40	G:1	T:0	
chr28	11137321	2	38	T:0.421053	C:0.578947	
chr28	11137812	2	38	C:0.973684	T:0.0263158	
chr28	11138410	2	38	C:0.736842	T:0.263158	
chr28	11138674	2	36	C:0.694444	T:0.305556	
chr28	11139107	2	38	A:0.842105	G:0.157895	
chr28	11139630	2	38	G:0.736842	A:0.263158	
chr28	11139927	2	38	G:0.684211	A:0.315789	
chr28	11140069	2	38	C:0.789474	T:0.210526	
chr28	11141630	2	36	CT:0.694444	C:0.305556	
chr28	11141693	3	40	C:0.525	CAGAGAG:0.25	CAGAGAGAGAG:0.225
chr28	11141931	2	38	A:0.473684	T:0.526316	
chr28	11141962	2	38	C:0.421053	T:0.578947	
chr28	11142267	3	40	T:0.45	TAACAC:0.3	TAACATAACATAACAC:0.25

chr28	11142363	2	40	G:0.475	T:0.525		
chr28	11142488	2	40	G:0.425	A:0.575		
chr28	11142555	2	38	CT:0.447368	C:0.552632		
chr28	11142679	2	40	A:0.725	G:0.275		
chr28	11142692	2	40	AC:0	A:1		
chr28	11143194	2	38	T:0	C:1		
chr28	11143261	2	38	T:0.447368	C:0.552632		
chr28	11143450	2	40	A:0.475	G:0.525		
chr28	11144030	2	36	C:0.472222	G:0.527778		
chr28	11144307	2	34	T:0.705882	C:0.294118		
chr28	11144685	2	36	A:0	G:1		
chr28	11144887	2	38	ACTCT:0.526316	A:0.473684		
chr28	11145043	2	40	T:0	C:1		
chr28	11145093	2	40	G:0.675	T:0.325		
chr28	11145162	2	38	A:0.657895	G:0.342105		
chr28	11145354	2	38	A:0.394737	G:0.605263		
chr28	11145417	2	40	T:0.85	C:0.15		
chr28	11145436	2	40	T:0.275	C:0.725		
chr28	11145448	2	40	C:0.95	A:0.05		
chr28	11145545	2	38	G:0.289474	T:0.710526		
chr28	11145690	2	34	C:0.5	G:0.5		
chr28	11146295	2	38	C:0.552632	T:0.447368		
chr28	11146472	2	40	C:0.625	A:0.375		
chr28	11146594	2	38	C:0.263158	A:0.736842		
chr28	11146971	2	38	G:0.289474	GA:0.710526		
chr28	11146974	4	40	G:0.25	GGGAGA:0.25	GGGAGAGA:0.15	GGGAGAGAGA:0.35
chr28	11147054	2	36	C:0.583333	T:0.416667		
chr28	11147463	2	36	T:1	G:0		
chr28	11147800	2	36	T:0.888889	C:0.111111		
chr28	11147871	2	38	G:0.842105	T:0.157895		
chr28	11148705	2	38	A:0.789474	G:0.210526		
chr28	11148781	2	36	C:0.777778	T:0.222222		
chr28	11149467	2	38	C:0.184211	G:0.815789		
chr28	11149894	2	40	CT:0.775	C:0.225		
chr28	11149941	2	34	G:0.941176	A:0.0588235		
chr28	11150015	2	40	T:0.85	C:0.15		
chr28	11150447	2	40	T:0.75	C:0.25		
chr28	11150546	2	38	G:0.394737	A:0.605263		
chr28	11150858	2	40	G:1	A:0		
chr28	11151445	2	40	G:0.35	GGA:0.65		
chr28	11151693	2	36	C:0.833333	G:0.166667		
chr28	11151793	2	38	C:0.789474	T:0.210526		
chr28	11151986	2	38	C:0.815789	A:0.184211		
chr28	11152066	2	40	C:0	T:1		
chr28	11152200	2	38	G:0.815789	A:0.184211		
chr28	11152206	3	38	CTT:0.552632	C:0.184211	CTTTT:0.263158	
chr28	11152336	2	38	G:1	A:0		
chr28	11152348	2	40	C:0.85	T:0.15		
chr28	11152491	2	36	T:0.555556	A:0.444444		
chr28	11152563	2	38	CAAAT:1	C:0		
chr28	11152752	2	38	T:0.868421	C:0.131579		
chr28	11153222	2	40	A:0.775	G:0.225		

chr28	11153233	2	40	G:0.75	T:0.25	
chr28	11153341	2	38	G:0.868421	A:0.131579	
chr28	11153357	2	38	A:0.815789	G:0.184211	
chr28	11153836	2	38	T:0.842105	G:0.157895	
chr28	11153872	2	40	T:0.775	C:0.225	
chr28	11154148	2	36	C:0.694444	T:0.305556	
chr28	11154154	2	36	A:0.694444	T:0.305556	
chr28	11154158	2	36	C:0.277778	T:0.722222	
chr28	11154931	2	40	G:0.425	T:0.575	
chr28	11155137	2	40	TA:0.525	T:0.475	
chr28	11155363	2	40	T:0	TC:1	
chr28	11155369	2	40	CA:0	C:1	
chr28	11155378	2	40	G:0	GT:1	
chr28	11155382	2	40	CT:0	C:1	
chr28	11156260	2	38	C:0.736842	A:0.263158	
chr28	11156387	2	36	TA:0.666667	T:0.333333	
chr28	11157509	2	36	G:0.638889	A:0.361111	
chr28	11157784	2	32	A:0.84375	T:0.15625	
chr28	11157947	2	40	G:0.475	A:0.525	
chr28	11158269	2	40	C:0.75	G:0.25	
chr28	11158274	2	40	C:0.75	A:0.25	
chr28	11158471	2	40	TC:0.925	T:0.075	
chr28	11159109	2	38	T:0.5	C:0.5	
chr28	11159293	2	30	G:0.8	A:0.2	
chr28	11159412	2	38	G:0.5	T:0.5	
chr28	11159430	2	36	T:0.472222	C:0.527778	
chr28	11159637	2	38	C:0.815789	T:0.184211	
chr28	11159720	2	40	T:0	TA:1	
chr28	11159746	2	38	T:0	A:1	
chr28	11159748	2	38	T:0	G:1	
chr28	11159924	2	38	T:0.789474	C:0.210526	
chr28	11160453	3	40	GTTT:0.25	G:0.475	GTTTT:0.275
chr28	11160461	2	40	T:0.525	A:0.475	
chr28	11160509	2	38	G:0.815789	T:0.184211	
chr28	11160610	2	36	C:0.527778	T:0.472222	
chr28	11160754	2	40	A:0.475	T:0.525	
chr28	11161130	2	36	C:0.75	T:0.25	
chr28	11161179	2	36	C:0.722222	T:0.277778	
chr28	11161218	2	36	G:0.527778	A:0.472222	
chr28	11161643	2	36	T:0.777778	C:0.222222	
chr28	11161658	2	30	T:0.8	TTTC:0.2	
chr28	11161676	3	34	C:0.470588	CTTCT:0.117647	CTTCTT:0.411765
chr28	11161715	2	26	G:0.423077	GGA:0.576923	
chr28	11161760	2	24	G:0.416667	T:0.583333	
chr28	11162339	2	38	T:0.526316	C:0.473684	
chr28	11162664	2	38	G:0.789474	A:0.210526	
chr28	11162702	2	40	A:0.5	G:0.5	
chr28	11162782	2	38	A:0.394737	C:0.605263	
chr28	11162799	2	40	T:0.375	C:0.625	
chr28	11163218	2	38	G:0.763158	A:0.236842	
chr28	11163562	2	38	T:0.684211	C:0.315789	
chr28	11163603	2	40	G:0.275	C:0.725	

chr28	11163783	2	38	TG:0.973684	T:0.0263158
chr28	11164244	2	38	T:0.552632	C:0.447368
chr28	11164304	2	40	T:0.275	C:0.725
chr28	11164358	2	38	C:0.842105	T:0.157895
chr28	11164857	2	40	A:0.325	G:0.675
chr28	11164976	2	40	T:0.2	C:0.8
chr28	11165039	2	40	T:0.3	C:0.7
chr28	11165063	2	40	C:0.775	T:0.225
chr28	11165132	2	40	C:1	T:0
chr28	11165204	2	38	T:0.315789	G:0.684211
chr28	11165964	2	40	T:0.725	C:0.275
chr28	11166141	2	30	C:1	T:0
chr28	11166434	2	40	C:0.825	T:0.175
chr28	11166477	2	38	G:0.736842	C:0.263158
chr28	11166790	2	40	C:1	T:0
chr28	11166849	2	38	A:0.684211	T:0.315789
chr28	11167414	2	38	G:0.5	A:0.5
chr28	11168171	2	36	A:0.416667	G:0.583333
chr28	11168230	2	40	AT:0.6	A:0.4
chr28	11168895	2	38	G:1	A:0
chr28	11169451	2	38	T:0.236842	C:0.763158
chr28	11169480	2	40	G:0.775	A:0.225
chr28	11169653	2	38	C:0.368421	T:0.631579
chr28	11169739	2	40	C:0.7	T:0.3
chr28	11170681	2	36	G:0.694444	A:0.305556
chr28	11171008	2	36	A:0.75	G:0.25
chr28	11171327	2	40	T:0.875	C:0.125
chr28	11171402	2	40	G:0.25	T:0.75
chr28	11172563	2	38	G:1	A:0
chr28	11173106	2	40	T:0.4	C:0.6
chr28	11173138	2	38	CCTCCT:0.684211	C:0.315789
chr28	11173139	2	38	C:0.763158	T:0.236842
chr28	11173145	2	38	T:0.763158	C:0.236842
chr28	11173146	2	38	CTTTCTTTCTTTCTTTCTTTCTTTCTTTCTTT:0.684211	C:0.315789
chr28	11173149	2	38	T:0.763158	C:0.236842
chr28	11173160	2	36	TTCTTTCTTTCTTTCTTTCTTTCTTTCTTTCTTTCTTTCTTTCTTTCTTTCTTTCTTTCTTTC:0.944444	T:0.0555556
chr28	11173164	2	36	TTCTTTCTTTCTTTCTTTCTTTCTTTCTTTCTTTCTTTCTTTCTTTCTTTCTTTCTTTC:0.833333	T:0.166667
chr28	11173181	2	38	T:0.684211	C:0.315789
chr28	11173185	2	38	T:0.684211	C:0.315789
chr28	11173206	2	38	CT:0.973684	C:0.0263158
chr28	11173215	2	38	TTTC:0.973684	T:0.0263158
chr28	11173562	2	36	C:0.555556	T:0.444444
chr28	11173968	2	38	CA:0.631579	C:0.368421
chr28	11174166	2	40	C:0.675	T:0.325
chr28	11174171	2	40	T:0.675	G:0.325
chr28	11174481	2	40	AT:0.6	A:0.4
chr28	11174534	2	34	C:1	T:0
chr28	11174882	2	38	A:0.736842	G:0.263158
chr28	11174963	2	36	G:0.277778	A:0.722222

chr28 11175147 2 40 G:0.625 A:0.375
chr28 11175525 2 40 AACTATGC:0.575 A:0.425
chr28 11176031 2 36 C:0.666667 T:0.333333
chr28 11177011 2 38 C:0.657895 T:0.342105
chr28 11177390 2 38 A:0.289474 G:0.710526
chr28 11178063 2 38 G:0.263158 A:0.736842
chr28 11178400 2 40 C:0.8 T:0.2
chr28 11178599 2 38 A:0.710526 G:0.289474
chr28 11178762 2 38 G:0.710526 A:0.289474
chr28 11179142 2 40 A:0.8 C:0.2
chr28 11179183 2 40 T:0 C:1
chr28 11179361 2 38 T:0.684211 C:0.315789
chr28 11180159 2 40 G:0.95 GT:0.05
chr28 11180194 3 40 CAA:0.025 C:0.225 CA:0.75
chr28 11180350 2 40 GGGA:0.75 G:0.25
chr28 11181319 2 32 AC:0.6875 A:0.3125
chr28 11181590 2 36 C:0.694444 T:0.305556
chr28 11182207 2 38 T:0.763158 C:0.236842
chr28 11182463 2 38 G:0.736842 A:0.263158
chr28 11183194 2 38 T:0.763158 C:0.236842
chr28 11183281 2 40 C:0.925 T:0.075
chr28 11183904 2 40 A:0.425 G:0.575
chr28 11183920 2 40 A:1 ATGCTTTGCTT:0
chr28 11183967 2 40 T:0.95 TCTTTTCTTTTCTTTTCTTTC:
0.05
chr28 11184001 2 40 T:0.95 TCTTTTCTTTC:0.05
chr28 11184021 3 40 T:0.7
TTTTCTTTCTTTCTTTCTTTCTTTCTTTC:0.225 TTTTCTTTCTTTCTTTCTTTCTTC:
0.075
chr28 11184055 2 40 T:0.925 TTCTTTCTTTCTTTC:0.075
chr28 11184072 2 40 C:0.925 T:0.075
chr28 11184191 2 32 C:0.78125 A:0.21875
chr28 11184323 2 40 C:0.45 A:0.55
chr28 11184763 2 40 G:0.675 C:0.325
chr28 11184784 2 40 G:0.75 A:0.25
chr28 11184800 2 40 T:0.75 C:0.25
chr28 11184949 2 38 A:0.631579 AT:0.368421
chr28 11185127 2 36 T:1 C:0
chr28 11185181 2 40 A:0.65 T:0.35
chr28 11185384 3 34 A:0.411765 AGT:0.352941
AGTGT:0.235294
chr28 11185526 2 36 A:0.666667 G:0.333333
chr28 11186189 2 38 AG:0.947368 A:0.0526316
chr28 11186315 2 40 T:0.6 G:0.4
chr28 11186991 2 38 A:0.368421 AT:0.631579
chr28 11187145 2 38 T:0.684211 A:0.315789
chr28 11187384 3 36 TAC:0.305556 T:0.305556
TACAC:0.388889
chr28 11187441 2 38 T:0.657895 C:0.342105
chr28 11187765 2 36 A:0.861111 G:0.138889
chr28 11187923 2 40 A:0.95 G:0.05
chr28 11188230 2 40 A:0.3 T:0.7
chr28 11188254 2 40 T:0.675 TTG:0.325
chr28 11188271 2 40 A:0.05 T:0.95

chr28	11188390	2	38	T:0.973684	C:0.0263158
chr28	11188604	2	34	A:1	AT:0
chr28	11188775	2	40	C:0.925	T:0.075
chr28	11189214	2	38	C:0.973684	T:0.0263158
chr28	11189290	2	36	A:0.472222	ATGC:0.527778
chr28	11189444	2	40	AC:0.975	A:0.025
chr28	11189943	2	38	A:0.263158	G:0.736842
chr28	11189985	2	40	G:1	C:0
chr28	11190134	2	40	G:0.675	C:0.325
chr28	11190159	2	40	T:0.95	A:0.05
chr28	11190487	2	38	A:0.368421	G:0.631579
chr28	11190612	2	40	C:0.675	T:0.325
chr28	11191046	2	38	C:0.789474	A:0.210526
chr28	11191548	2	38	G:1	A:0
chr28	11191866	2	40	A:0.825	G:0.175
chr28	11191907	2	38	A:0.973684	G:0.0263158
chr28	11192501	2	40	T:0.325	C:0.675
chr28	11193109	2	36	G:0.944444	A:0.0555556
chr28	11193126	2	36	C:1	T:0
chr28	11193387	2	40	A:0	C:1
chr28	11193427	2	40	C:0.975	T:0.025
chr28	11193547	2	38	A:0.921053	G:0.0789474
chr28	11193767	2	38	C:0.973684	G:0.0263158
chr28	11194490	2	40	C:0.6	T:0.4
chr28	11194601	2	38	G:0.789474	A:0.210526
chr28	11194606	2	38	C:0.0263158	CA:0.973684
chr28	11194629	2	38	C:0.973684	T:0.0263158
chr28	11194866	2	40	G:0.975	A:0.025
chr28	11195436	2	26	CG:0.692308	C:0.307692
chr28	11195609	2	38	A:1	T:0
chr28	11195630	2	38	T:1	A:0
chr28	11195750	2	36	TTC:0.611111	T:0.388889
chr28	11195893	2	38	C:0.710526	T:0.289474
chr28	11196142	2	38	C:0.736842	T:0.263158
chr28	11196217	2	40	T:0.25	C:0.75
chr28	11196473	2	40	T:0.55	C:0.45
chr28	11196601	2	38	CTG:0.947368	C:0.0526316
chr28	11197557	2	38	C:0.973684	G:0.0263158
chr28	11197560	2	38	T:0.736842	C:0.263158
chr28	11197658	2	40	A:0.95	G:0.05
chr28	11198089	2	40	G:0.95	A:0.05
chr28	11198252	2	40	T:0.975	A:0.025
chr28	11198341	2	40	TTA:0.775	T:0.225
chr28	11198570	2	38	T:0.789474	C:0.210526
chr28	11198628	2	40	A:0.375	G:0.625
chr28	11198887	2	38	T:0.684211	C:0.315789
chr28	11199236	2	38	AAAG:1	A:0
chr28	11199522	2	38	T:0.315789	TAC:0.684211
chr28	11199864	2	32	A:0.71875	T:0.28125
chr28	11199915	2	34	T:0.264706	C:0.735294
chr28	11200037	2	40	C:0.75	T:0.25
chr28	11200262	2	38	T:0.368421	A:0.631579
chr28	11200286	2	38	C:0.973684	T:0.0263158
chr28	11200366	2	38	C:1	T:0

chr28	11200434	2	36	ATAG:0.694444	A:0.305556	
chr28	11200515	2	40	A:0.95	C:0.05	
chr28	11200585	2	36	A:0.694444	AAG:0.305556	
chr28	11200769	2	34	AT:0.764706	A:0.235294	
chr28	11200827	2	38	C:0.973684	T:0.0263158	
chr28	11201104	2	36	G:0.833333	A:0.166667	
chr28	11201365	2	38	T:1	C:0	
chr28	11201602	2	40	T:0.9	C:0.1	
chr28	11201640	2	38	C:0.973684	T:0.0263158	
chr28	11201650	2	38	A:0.736842	G:0.263158	
chr28	11201836	2	40	G:0.6	A:0.4	
chr28	11202021	2	38	CAAAAA:0.894737	C:0.105263	
chr28	11202039	2	38	CAA:0.894737	C:0.105263	
chr28	11202048	2	40	C:0.725	A:0.275	
chr28	11202051	2	38	AAAAAAC:0.894737	A:0.105263	
chr28	11202054	3	40	A:0.725	AAC:0.25	AACAAC:0.025
chr28	11202060	2	40	A:0.85	C:0.15	
chr28	11202122	2	32	C:0.625	T:0.375	
chr28	11202791	2	40	T:0.325	C:0.675	
chr28	11202793	2	40	G:1	T:0	
chr28	11203019	2	36	G:0.805556	T:0.194444	
chr28	11203521	2	36	A:0.222222	G:0.777778	
chr28	11203603	2	40	T:0.725	C:0.275	
chr28	11204127	2	38	A:0.684211	G:0.315789	
chr28	11204563	2	38	A:1	T:0	
chr28	11204776	2	38	G:0.631579	A:0.368421	
chr28	11204816	2	38	T:0.315789	A:0.684211	
chr28	11205012	2	38	ATGTC:1	A:0	
chr28	11205127	2	38	A:0.736842	G:0.263158	
chr28	11205576	2	40	C:0.6	T:0.4	
chr28	11205841	2	40	A:0.95	C:0.05	
chr28	11206426	2	40	A:0.325	G:0.675	
chr28	11206441	2	40	T:1	G:0	
chr28	11207252	2	38	T:1	G:0	
chr28	11207309	2	38	A:1	T:0	
chr28	11207665	2	40	A:0	C:1	
chr28	11207955	2	40	C:1	T:0	
chr28	11208138	2	40	A:0.7	T:0.3	
chr28	11208478	3	40	TA:0.325	T:0.425	TAA:0.25
chr28	11209025	2	38	C:1	T:0	
chr28	11209347	2	38	G:0	A:1	
chr28	11209461	2	38	G:0	A:1	
chr28	11209546	2	38	C:0.947368	T:0.0526316	
chr28	11209700	2	40	G:0.975	A:0.025	
chr28	11209761	2	40	C:1	A:0	
chr28	11210712	2	40	G:0.925	T:0.075	
chr28	11211044	2	40	C:0.875	T:0.125	
chr28	11211268	2	40	G:0	GAACAA:1	
chr28	11211479	2	40	G:0.7	A:0.3	
chr28	11211761	2	38	G:1	A:0	
chr28	11211804	2	38	CT:0.736842	C:0.263158	
chr28	11211807	3	38	TAA:0.263158	T:0.710526	
TA:0.0263158						
chr28	11211844	2	38	A:0.631579	AGAGAGAGC:	

0.368421
chr28 11212061 2 38 G:0.710526 A:0.289474
chr28 11212096 2 38 C:1 A:0
chr28 11212224 3 36 A:0.694444 AT:0.25 ATT:
0.0555556
chr28 11212567 2 38 G:0.947368 A:0.0526316
chr28 11212891 2 38 G:0.973684 A:0.0263158
chr28 11213287 2 40 C:1 T:0
chr28 11213555 2 38 A:0.973684 G:0.0263158
chr28 11213752 2 34 T:0.352941 C:0.647059
chr28 11214289 2 36 C:0.694444 T:0.305556
chr28 11214381 2 36 AAGG:0.777778 A:0.222222
chr28 11215670 2 40 A:0.225 G:0.775
chr28 11215677 2 40 G:0.575 A:0.425
chr28 11215769 2 38 G:0.763158 A:0.236842
chr28 11215833 4 38 CTTTT:0.131579 C:0.263158
CT:0.421053 CTT:0.184211
chr28 11216573 2 36 C:0.944444 T:0.0555556
chr28 11216594 2 36 T:0 C:1
chr28 11216783 2 36 C:0.611111 T:0.388889
chr28 11217136 2 36 C:0.722222 CT:0.277778
chr28 11217137 2 36 T:0.777778 TTC:0.222222
chr28 11217798 2 40 A:0.675 T:0.325
chr28 11217871 2 36 C:0.388889 T:0.611111
chr28 11217999 2 38 A:0.289474 C:0.710526
chr28 11218337 2 38 A:0.526316 G:0.473684
chr28 11218584 2 38 G:0.315789 A:0.684211
chr28 11218635 2 40 G:0.25 A:0.75
chr28 11218794 2 38 C:0.315789 A:0.684211
chr28 11218823 2 40 G:0.625 A:0.375
chr28 11218863 2 40 C:0 A:1
chr28 11219026 2 38 T:0.473684 G:0.526316
chr28 11219082 2 36 G:0.333333 A:0.666667
chr28 11219280 2 34 C:0.764706 G:0.235294
chr28 11219290 2 36 A:0.583333 G:0.416667
chr28 11219442 2 38 CA:0.657895 C:0.342105
chr28 11219505 2 34 C:0.676471 T:0.323529
chr28 11219509 2 34 A:0.676471 G:0.323529
chr28 11219847 3 36 GT:0.388889 G:0.25 GTT:
0.361111
chr28 11220133 2 40 C:0.725 T:0.275
chr28 11220284 2 40 C:0.975 T:0.025
chr28 11220447 2 40 C:0.675 T:0.325
chr28 11220625 2 40 C:0.7 T:0.3
chr28 11220870 2 40 A:0.675 C:0.325
chr28 11220884 2 38 A:0.710526 G:0.289474
chr28 11220953 2 36 G:0.666667 A:0.333333
chr28 11221023 2 40 T:0.625 C:0.375
chr28 11221156 2 40 C:0.975 G:0.025
chr28 11221493 3 36 C:0.305556 CT:0.611111
CTT:0.0833333
chr28 11221594 3 38 GTCTCTC:0.552632 G:0.368421
GTC:0.0789474
chr28 11221603 2 34 TCTCTCTCACA:0.941176 T:

0.0588235
chr28	11221609	2	38	T:0.710526	A:0.289474
chr28	11222176	2	40	G:0.65	T:0.35
chr28	11222232	2	40	CAG:0.975	C:0.025
chr28	11222269	2	40	C:0.725	T:0.275
chr28	11222455	2	38	T:0.368421	C:0.631579
chr28	11222692	2	38	G:0.315789	A:0.684211
chr28	11222935	2	36	G:1	A:0
chr28	11222971	2	38	T:0.263158	C:0.736842
chr28	11223019	2	40	A:0.725	ACCCCCATTCACCCT:0.275
chr28	11223059	2	40	A:0.375	C:0.625
chr28	11223154	2	38	G:0.368421	A:0.631579
chr28	11223202	2	38	T:0.421053	G:0.578947
chr28	11223225	2	38	T:0.947368	G:0.0526316
chr28	11223363	2	38	C:0.631579	T:0.368421
chr28	11223455	2	40	T:0.625	C:0.375
chr28	11223465	2	40	G:0.625	A:0.375
chr28	11223475	2	40	G:0.975	A:0.025
chr28	11224210	2	36	T:0.972222	C:0.0277778
chr28	11224549	2	38	C:0.973684	T:0.0263158
chr28	11225235	2	38	A:0.605263	G:0.394737
chr28	11225598	2	40	C:0.925	T:0.075
chr28	11225914	2	34	C:0.676471	CCCA:0.323529
chr28	11226115	2	40	A:1	T:0
chr28	11226301	2	34	C:0.647059	T:0.352941
chr28	11228114	2	26	G:0.615385	GC:0.384615
chr28	11228121	2	30	CG:0.933333	C:0.0666667
chr28	11228125	2	30	C:0.566667	G:0.433333
chr28	11228255	2	24	T:0.541667	A:0.458333
chr28	11228427	2	32	G:0.9375	C:0.0625
chr28	11228779	4	40	AAC:0.725	A:0.05	AACAC:
0.025	AACACACAC:0.2
chr28	11229233	2	38	C:1	T:0
chr28	11229617	2	38	A:0	T:1
chr28	11229875	2	36	C:0.638889	A:0.361111
chr28	11229901	2	38	T:0.631579	C:0.368421
chr28	11229938	2	38	G:0.684211	GC:0.315789
chr28	11230222	2	40	C:0.375	T:0.625
chr28	11230368	2	38	T:0.973684	C:0.0263158
chr28	11230391	2	38	A:1	C:0
chr28	11230393	2	38	TC:1	T:0
chr28	11230722	2	36	C:0.388889	T:0.611111
chr28	11230730	2	36	C:0.972222	T:0.0277778
chr28	11230739	2	34	C:0	T:1
chr28	11230799	2	40	GTGTCTCTGTCTC:0.525	G:0.475
chr28	11230977	2	38	C:0.684211	T:0.315789
chr28	11231111	2	40	A:0	G:1
chr28	11231332	2	38	T:0.736842	G:0.263158
chr28	11231353	2	40	AC:1	A:0
chr28	11231357	3	40	A:0.975	C:0	AAC:0.025
chr28	11231362	3	40	C:0.575	A:0.425	CA:0
chr28	11231364	2	40	AAC:0.975	A:0.025
chr28	11231505	2	40	C:0.7	T:0.3
chr28	11231888	2	38	T:0.631579	C:0.368421

chr28	11231912	2	40	G:0.575	A:0.425
chr28	11232195	2	38	G:1	A:0
chr28	11232372	2	36	C:0.694444	G:0.305556
chr28	11232389	2	34	C:0.352941	T:0.647059
chr28	11232628	2	38	C:0.657895	T:0.342105
chr28	11232632	2	38	T:0.657895	C:0.342105
chr28	11232796	2	40	A:0.625	G:0.375
chr28	11232823	2	38	A:0.947368	G:0.0526316
chr28	11232935	2	38	C:0.552632	T:0.447368
chr28	11233037	2	40	A:0.65	G:0.35
chr28	11233049	2	40	A:0.65	G:0.35
chr28	11233051	2	40	T:0.65	C:0.35
chr28	11233069	2	40	T:0.65	C:0.35
chr28	11233204	2	40	G:0.925	T:0.075
chr28	11233366	2	36	A:0.611111	T:0.388889
chr28	11233477	2	38	C:0.394737	T:0.605263
chr28	11234054	3	38	T:0.605263	TA:0.368421
TAA:0.0263158					
chr28	11234137	2	36	A:0.0833333	C:0.916667
chr28	11234325	2	36	C:0.611111	G:0.388889
chr28	11234378	2	40	G:0.625	T:0.375
chr28	11234982	2	36	G:0.444444	A:0.555556
chr28	11234992	2	36	G:0.444444	A:0.555556
chr28	11235094	2	32	T:0.4375	C:0.5625
chr28	11235251	2	36	T:0.555556	C:0.444444
chr28	11235275	2	38	G:0.736842	A:0.263158
chr28	11235614	2	36	A:0.583333	G:0.416667
chr28	11235736	2	36	C:0.583333	T:0.416667
chr28	11235988	2	36	T:0.583333	C:0.416667
chr28	11236283	2	34	G:0.588235	T:0.411765
chr28	11236285	2	34	G:0.588235	T:0.411765
chr28	11236615	2	38	G:0.578947	A:0.421053
chr28	11236617	2	38	A:0.578947	C:0.421053
chr28	11237023	2	38	C:0.657895	T:0.342105
chr28	11237126	2	40	A:0.625	AT:0.375
chr28	11237137	2	40	T:0.625	G:0.375
chr28	11237162	2	40	C:0.625	G:0.375
chr28	11237215	2	40	A:0.625	C:0.375
chr28	11237220	2	40	C:0.625	G:0.375
chr28	11237236	2	38	A:0	G:1
chr28	11237285	2	40	T:0.575	TAA:0.425
chr28	11237310	2	40	A:0.575	T:0.425
chr28	11237500	2	36	T:1	C:0
chr28	11237567	2	36	A:0.527778	G:0.472222
chr28	11237652	2	34	T:0.558824	C:0.441176
chr28	11237739	2	38	G:1	A:0
chr28	11237785	2	38	A:1	T:0
chr28	11237827	2	30	A:0.6	G:0.4
chr28	11237836	2	32	C:0.9375	T:0.0625
chr28	11237954	2	40	G:0.625	C:0.375
chr28	11238024	2	38	T:0.605263	G:0.394737
chr28	11238028	2	38	G:0.605263	A:0.394737
chr28	11238594	2	38	T:0.5	C:0.5
chr28	11238761	2	36	G:0.638889	C:0.361111

chr28 11238777 2 36 C:1 T:0
chr28 11238894 2 34 G:0.588235 A:0.411765
chr28 11238981 2 40 G:0.6 A:0.4
chr28 11239148 2 38 G:0.684211 A:0.315789
chr28 11239320 2 38 G:0.605263 A:0.394737
chr28 11239352 3 38 GT:0.526316 G:0 GTT:0.473684
chr28 11239856 2 40 TA:0.925 T:0.075
chr28 11240512 2 38 C:0.684211 T:0.315789
chr28 11241265 2 38 G:1 T:0
chr28 11241476 2 38 G:0.710526 A:0.289474
chr28 11241518 2 36 C:0.944444 T:0.0555556
chr28 11241600 2 30 G:0.566667 C:0.433333
chr28 11241628 2 38 T:0.947368 TAAAAAA:0.0526316
chr28 11241629 3 38 T:0.657895 A:0.236842 TAAA:0.105263
chr28 11241715 2 40 G:0 A:1
chr28 11241740 2 40 A:0.375 T:0.625
chr28 11241788 2 32 T:0.65625 A:0.34375
chr28 11241891 2 36 C:0.666667 T:0.333333
chr28 11242060 2 40 G:0 T:1
chr28 11242096 2 38 G:0.921053 A:0.0789474
chr28 11242382 2 36 AG:0.972222 A:0.0277778
chr28 11243014 2 38 T:1 C:0
chr28 11243101 5 40 CT:0.25 C:0.1 CTT:0.275 CTTT:0.05 CTTTT:0.325
chr28 11244344 2 38 C:0.894737 T:0.105263
chr28 11244417 2 34 A:1 G:0
chr28 11244560 2 36 ATCTCTCTCTC:0.444444 A:0.555556
chr28 11244902 2 40 G:0.6 A:0.4
chr28 11245275 2 38 C:0.605263 T:0.394737
chr28 11245411 2 40 G:0.65 C:0.35
chr28 11245427 2 40 C:1 T:0
chr28 11245898 2 38 C:0.605263 T:0.394737
chr28 11245938 3 38 G:0.921053 A:0 T:0.0789474
chr28 11245946 2 38 C:0.684211 A:0.315789
chr28 11246330 2 40 G:1 A:0
chr28 11246627 2 38 A:1 G:0
chr28 11246725 2 38 G:0.552632 C:0.447368
chr28 11246979 2 38 T:1 G:0
chr28 11247420 2 34 G:0.941176 A:0.0588235
chr28 11247430 2 36 A:1 G:0
chr28 11247484 2 40 G:0.65 A:0.35
chr28 11247492 2 40 T:1 C:0
chr28 11247544 2 40 T:0.875 C:0.125
chr28 11248338 2 40 C:0.95 T:0.05
chr28 11248459 2 40 GTCAAA:0.3 G:0.7
chr28 11248466 2 40 G:0.3 GGCC:0.7
chr28 11248468 2 40 C:0.3 CAG:0.7
chr28 11248509 2 40 G:0.775 A:0.225
chr28 11248833 2 38 C:0.947368 T:0.0526316
chr28 11248948 2 38 G:0.921053 C:0.0789474

chr28	11249268	2	40	C:0.95	T:0.05		
chr28	11249413	2	38	C:0.921053	G:0.0789474		
chr28	11249510	2	40	C:0.95	A:0.05		
chr28	11249816	2	40	T:0.9	C:0.1		
chr28	11249942	2	40	T:0.875	C:0.125		
chr28	11249996	2	40	T:0.525	TTC:0.475		
chr28	11250040	2	40	G:0.875	GTCTC:0.125		
chr28	11250044	2	36	C:1	CTG:0		
chr28	11250047	2	36	TCTCACACACACA:0.805556	T: 0.194444		
chr28	11250049	2	36	TCACA:1	T:0		
chr28	11250055	2	36	A:1	T:0		
chr28	11250057	2	36	A:1	T:0		
chr28	11250059	2	36	A:1	T:0		
chr28	11250061	2	34	A:1	T:0		
chr28	11250063	2	32	A:1	T:0		
chr28	11250065	2	32	A:1	T:0		
chr28	11250313	2	38	C:0.947368	T:0.0526316		
chr28	11250331	2	38	T:0.736842	C:0.263158		
chr28	11250705	2	40	A:0.95	G:0.05		
chr28	11251322	2	34	T:1	C:0		
chr28	11251447	2	40	T:1	C:0		
chr28	11251462	2	38	G:0.973684	A:0.0263158		
chr28	11251590	2	40	GT:1	G:0		
chr28	11251993	2	38	C:1	T:0		
chr28	11252102	2	40	T:0.95	C:0.05		
chr28	11252187	2	40	C:0.7	G:0.3		
chr28	11252656	2	40	C:0.95	A:0.05		
chr28	11252805	4	40	CTTCT:0.75	C:0.075	CTTTCT: 0.15	CTTCTTTCT:0.025
chr28	11252860	2	40	C:1	CT:0		
chr28	11253422	2	36	G:1	T:0		
chr28	11253544	2	40	G:0.925	C:0.075		
chr28	11253727	2	34	C:0.705882	G:0.294118		
chr28	11253832	2	38	C:0.710526	T:0.289474		
chr28	11253984	2	38	G:0.657895	A:0.342105		
chr28	11254421	2	40	C:0.925	T:0.075		
chr28	11254589	2	38	G:1	A:0		
chr28	11254881	2	38	A:0.763158	G:0.236842		
chr28	11255004	2	40	G:1	C:0		
chr28	11255252	2	38	GC:1	G:0		
chr28	11255254	2	38	C:1	A:0		
chr28	11255404	2	36	T:0.777778	C:0.222222		
chr28	11255687	2	36	CT:0.972222	C:0.0277778		
chr28	11255734	2	36	T:0.75	C:0.25		
chr28	11256193	2	38	T:1	A:0		
chr28	11256194	2	38	A:0.578947	G:0.421053		
chr28	11256428	2	36	G:0.666667	A:0.333333		
chr28	11256480	2	38	T:0.236842	A:0.763158		
chr28	11256678	2	40	G:0.675	A:0.325		
chr28	11256791	2	28	AG:0.892857	A:0.107143		
chr28	11257115	2	38	G:0.947368	A:0.0526316		
chr28	11257131	2	36	A:0.666667	G:0.333333		
chr28	11257256	2	38	A:0.631579	G:0.368421		

chr28 11257622 2 38 T:0.605263 A:0.394737
chr28 11257676 2 38 T:0.710526 C:0.289474
chr28 11257751 2 36 G:0.694444 A:0.305556
chr28 11257777 2 38 T:0.657895 C:0.342105
chr28 11257868 2 40 T:0.325 C:0.675
chr28 11257917 2 38 A:0.710526 T:0.289474
chr28 11257919 2 38 A:0.710526 C:0.289474
chr28 11257986 2 38 C:0.763158 T:0.236842
chr28 11258344 2 38 G:0.684211 GT:0.315789
chr28 11258384 2 38 GATGGGTAA:0.947368 G:
0.0526316
chr28 11258487 2 36 C:0.916667 T:0.0833333
chr28 11258540 2 36 A:0 G:1
chr28 11258557 2 36 A:0.722222 G:0.277778
chr28 11258598 2 36 A:0 G:1
chr28 11258631 2 38 A:0.973684 C:0.0263158
chr28 11259057 2 38 C:0.763158 A:0.236842
chr28 11259219 2 38 G:1 A:0
chr28 11259309 2 36 C:0.972222 T:0.0277778
chr28 11259354 2 34 T:0.323529 C:0.676471
chr28 11259463 2 38 G:0.710526 A:0.289474
chr28 11259464 2 38 T:0.710526 C:0.289474
chr28 11259560 2 40 T:0.775 TC:0.225
chr28 11259577 3 40 C:0.525 CT:0.475
CTTTTCTTTTCTTTTCT:0
chr28 11259591 2 40 C:0.775 CT:0.225
chr28 11259596 4 40 C:0.625 T:0.225
CTTTTCTTTTCTTTCTTTTCTTTTCTTCTT:0.125 CTTTCTTTTCTTTTCTTCTT:
0.025
chr28 11259606 2 40 T:0.775 TTTC:0.225
chr28 11259619 3 40 C:0.75 CTTTCT:0.125
CTTTCTTTTCT:0.125
chr28 11259668 2 40 TTCTC:0.75 T:0.25
chr28 11259670 4 40 C:0.675 CTCCTT:0 CTCCTTTCCTT:0.275
CTCCTTTCCTTTCCTT:0.05
chr28 11259675 2 40 T:0.75 TTTC:0.25
chr28 11259784 2 38 G:0.605263 T:0.394737
chr28 11260071 2 36 G:0.805556 T:0.194444
chr28 11260086 2 40 T:0.075 TTTTAGATC:0.925
chr28 11260125 2 38 C:0.763158 T:0.236842
chr28 11260401 2 38 CTA:0.842105 C:0.157895
chr28 11260415 2 34 CTTT:0.911765 C:0.0882353
chr28 11260667 2 38 G:0.342105 A:0.657895
chr28 11260926 2 40 G:1 A:0
chr28 11261438 2 40 A:0.725 AC:0.275
chr28 11261560 2 38 G:0.763158 GTT:0.236842
chr28 11261562 4 38 A:0.315789 AT:0.368421
ATT:0.0789474 T:0.236842
chr28 11262845 3 40 C:0.25 CT:0.6 CTT:0.15
chr28 11263466 2 40 G:1 A:0
chr28 11263663 2 38 A:1 C:0
chr28 11263809 2 40 C:1 G:0
chr28 11264751 2 40 C:0.775 CTTTTTTTTTTTTTT:0.225
chr28 11264822 2 38 G:0.0263158 C:0.973684

chr28	11265008	2	40	C:0.175	T:0.825	
chr28	11265410	2	34	C:0.205882	T:0.794118	
chr28	11266285	2	40	C:0.025	CA:0.975	
chr28	11266576	2	36	TATC:0.944444	T:0.0555556	
chr28	11266609	3	38	C:1	CATT:0	CATCATT:0
chr28	11266641	2	36	A:0.916667	G:0.0833333	
chr28	11266863	2	38	G:1	A:0	
chr28	11267548	2	38	T:1	C:0	
chr28	11269073	2	40	TTAATACCTGA:0.95	T:0.05	
chr28	11269991	2	38	C:0	T:1	
chr28	11270027	2	38	C:1	T:0	
chr28	11270760	2	40	G:1	GAA:0	
chr28	11270764	3	40	C:0	A:0	CA:1
chr28	11271011	2	36	G:1	A:0	
chr28	11271584	2	38	G:0.0789474	A:0.921053	
chr28	11272131	2	30	A:0	G:1	
chr28	11272608	2	38	G:1	A:0	
chr28	11274137	2	36	GT:0.722222	G:0.277778	
chr28	11274150	2	36	T:1	G:0	
chr28	11274151	3	38	T:0.526316	G:0.447368	TG:0.0263158
chr28	11274749	2	40	A:0.95	G:0.05	
chr28	11274914	2	36	T:0.972222	C:0.0277778	
chr28	11274951	2	40	C:0	T:1	
chr28	11274968	2	40	A:0	ATG:1	
chr28	11275014	2	40	G:0	A:1	
chr28	11275204	2	40	A:1	C:0	
chr28	11275219	2	40	A:0.175	G:0.825	
chr28	11275614	2	40	T:0.025	C:0.975	
chr28	11275639	2	38	G:1	A:0	
chr28	11276077	2	36	C:1	T:0	
chr28	11276601	2	38	T:1	G:0	
chr28	11276831	2	40	GAAGAAC:1	G:0	
chr28	11276837	2	40	C:1	CGTT:0	
chr28	11277354	2	34	A:0.970588	C:0.0294118	
chr28	11277635	2	36	A:1	G:0	
chr28	11277721	2	40	C:1	T:0	
chr28	11278420	2	40	C:0.85	T:0.15	
chr28	11278902	2	40	G:1	A:0	
chr28	11278938	2	40	C:1	T:0	
chr28	11279515	2	38	A:1	C:0	
chr28	11279669	2	34	T:1	C:0	
chr28	11279683	2	34	T:1	C:0	
chr28	11279726	2	26	GC:0.307692	G:0.692308	
chr28	11279768	2	38	T:1	C:0	
chr28	11279772	2	38	AC:1	A:0	
chr28	11279774	2	40	C:0.05	A:0.95	
chr28	11279778	2	38	C:1	A:0	
chr28	11279779	2	40	C:0.05	A:0.95	
chr28	11279780	2	38	AAATT:1	A:0	
chr28	11279784	2	40	T:0.05	A:0.95	
chr28	11279788	2	38	CCAAACCAAACCAAAT:1	C:0	
chr28	11279789	2	38	C:0	A:1	
chr28	11279794	2	38	C:0	A:1	

chr28	11279799	2	38	C:0	A:1		
chr28	11279808	2	38	C:1	T:0		
chr28	11280003	2	38	C:1	T:0		
chr28	11280036	2	38	C:1	T:0		
chr28	11280704	2	36	GAGAGGA:0.972222	G:0.0277778		
chr28	11280712	2	36	G:0.972222	T:0.0277778		
chr28	11282085	2	38	C:1	A:0		
chr28	11282186	2	36	A:0.944444	T:0.0555556		
chr28	11282191	2	36	A:1	T:0		
chr28	11282475	2	40	G:1	C:0		
chr28	11282869	2	40	G:1	A:0		
chr28	11283417	2	38	CAT:0.815789	C:0.184211		
chr28	11284267	2	38	C:0.894737	T:0.105263		
chr28	11284521	2	38	T:1	C:0		
chr28	11284626	2	38	T:1	C:0		
chr28	11284660	3	40	TAA:0.325	T:0.075	TA:0.6	
chr28	11285707	2	38	C:1	T:0		
chr28	11286498	2	36	T:0.944444	G:0.0555556		
chr28	11287034	2	36	C:0.777778	T:0.222222		
chr28	11287104	2	38	A:1	G:0		
chr28	11287174	2	34	G:1	A:0		
chr28	11287793	2	32	C:1	A:0		
chr28	11288242	2	40	C:1	A:0		
chr28	11289043	2	40	A:0	G:1		
chr28	11289271	2	40	T:0	C:1		
chr28	11291618	2	34	T:0.147059	C:0.852941		
chr28	11291962	2	38	A:0	G:1		
chr28	11292808	2	36	G:1	A:0		
chr28	11292889	2	38	C:1	T:0		
chr28	11294552	2	38	CT:0.578947	C:0.421053		
chr28	11294976	2	38	TA:0.552632	T:0.447368		
chr28	11295338	2	36	C:0.888889	T:0.111111		
chr28	11295532	2	36	C:1	T:0		
chr28	11295568	2	36	C:0.361111	G:0.638889		
chr28	11295581	4	38	CTT:0.0526316	C:0.473684	CT:0.0526316	CTTTT:0.421053
chr28	11295610	2	38	A:0.631579	G:0.368421		
chr28	11295881	2	38	G:0.657895	A:0.342105		
chr28	11296048	2	40	T:0.675	G:0.325		
chr28	11296086	3	38	AT:0.657895	A:0.0526316	ATT:0.289474	
chr28	11296136	2	38	T:0.315789	C:0.684211		
chr28	11296485	2	38	T:0.342105	G:0.657895		
chr28	11296493	2	38	GA:0.342105	G:0.657895		
chr28	11296594	2	40	G:0.75	A:0.25		
chr28	11296597	2	40	C:0.75	T:0.25		
chr28	11296669	2	40	T:0.7	C:0.3		
chr28	11296786	2	40	A:0.325	G:0.675		
chr28	11296838	2	40	C:0.5	T:0.5		
chr28	11296852	2	38	G:0.657895	A:0.342105		
chr28	11296855	2	38	A:0.657895	T:0.342105		
chr28	11297063	2	40	C:0.4	A:0.6		
chr28	11297074	2	40	T:0	C:1		
chr28	11297102	2	38	T:0.421053	C:0.578947		

chr28	11297140	2	40	A:0.325	C:0.675	
chr28	11297177	2	40	C:0.35	T:0.65	
chr28	11297189	2	40	T:0.15	C:0.85	
chr28	11297276	2	38	C:0.736842	G:0.263158	
chr28	11297294	2	38	A:0.736842	C:0.263158	
chr28	11297441	2	40	C:0.05	CAAAT:0.95	
chr28	11297774	2	38	GT:1	G:0	
chr28	11297834	2	40	C:0.675	T:0.325	
chr28	11297841	2	40	T:0	C:1	
chr28	11297904	2	40	A:0	G:1	
chr28	11297938	2	40	C:0.425	T:0.575	
chr28	11298093	2	38	C:0.763158	T:0.236842	
chr28	11298219	2	40	A:0.725	AG:0.275	
chr28	11298538	2	40	C:0.7	T:0.3	
chr28	11299765	2	40	C:0.95	G:0.05	
chr28	11299866	2	40	G:0.85	A:0.15	
chr28	11300176	2	38	G:0	A:1	
chr28	11300427	2	40	A:0	G:1	
chr28	11300494	2	40	T:0.025	C:0.975	
chr28	11300518	2	40	A:0.025	G:0.975	
chr28	11300531	2	40	G:0.025	A:0.975	
chr28	11300768	2	36	T:0.138889	A:0.861111	
chr28	11300844	2	38	C:0.0263158	T:0.973684	
chr28	11301654	2	38	A:1	G:0	
chr28	11301685	2	32	GT:0.75	G:0.25	
chr28	11301776	2	38	C:0.342105	CT:0.657895	
chr28	11301899	2	40	C:0.05	T:0.95	
chr28	11302619	2	36	G:0.111111	C:0.888889	
chr28	11302652	2	38	TGGA:0	T:1	
chr28	11302682	2	38	G:0.0526316	A:0.947368	
chr28	11303627	2	38	A:1	G:0	
chr28	11304834	2	40	A:0	G:1	
chr28	11304909	2	36	C:1	T:0	
chr28	11305037	2	36	A:0.0555556	C:0.944444	
chr28	11305299	2	38	T:0.921053	G:0.0789474	
chr28	11306387	2	38	T:0.973684	C:0.0263158	
chr28	11306726	2	40	C:0.6	CT:0.4	
chr28	11306894	2	34	G:1	A:0	
chr28	11307128	2	38	GT:0.947368	G:0.0526316	
chr28	11309258	2	40	C:1	T:0	
chr28	11310143	2	38	T:1	A:0	
chr28	11310285	2	36	G:0.666667	A:0.333333	
chr28	11311019	2	38	A:0.973684	G:0.0263158	
chr28	11311182	2	38	GTTTTT:0.105263	G:0.894737	
chr28	11311220	2	32	T:0.0625	C:0.9375	
chr28	11312459	3	34	TA:0.735294	T:0.117647	TAA:0.147059
chr28	11312919	2	38	A:0.0526316	G:0.947368	
chr28	11313232	2	40	C:0.9	A:0.1	
chr28	11313614	2	40	T:0.925	C:0.075	
chr28	11315504	2	36	C:0.0833333	G:0.916667	
chr28	11315778	2	40	T:1	C:0	
chr28	11315830	2	38	C:0.947368	A:0.0526316	
chr28	11315919	2	40	CG:1	C:0	

chr28	11315973	2	38	CAG:0.973684	C:0.0263158	
chr28	11316005	2	38	C:0.921053	T:0.0789474	
chr28	11316162	2	40	A:0.675	C:0.325	
chr28	11316269	2	40	A:0.95	G:0.05	
chr28	11316475	2	40	A:0.5	G:0.5	
chr28	11316585	2	28	G:0.464286	C:0.535714	
chr28	11316604	2	26	G:0.576923	A:0.423077	
chr28	11316863	2	32	G:1	A:0	
chr28	11317055	2	38	T:0.736842	C:0.263158	
chr28	11317197	2	32	GGTCC:0.625	G:0.375	
chr28	11317201	2	32	CG:1	C:0	
chr28	11317203	2	32	TCCG:1	T:0	
chr28	11317231	3	38	GGTGGGCT:0.894737	G:0.105263	GGTGGGCTGTGGGCT:0
chr28	11317313	2	36	A:0.944444	G:0.0555556	
chr28	11317402	2	38	T:0.763158	A:0.236842	
chr28	11317421	2	38	T:0.973684	A:0.0263158	
chr28	11317602	2	36	C:0.944444	T:0.0555556	
chr28	11317604	2	36	T:0.972222	TC:0.0277778	
chr28	11317638	2	36	TA:0.972222	T:0.0277778	
chr28	11317642	2	36	A:0.111111	T:0.888889	
chr28	11317686	2	32	A:0.09375	AAG:0.90625	
chr28	11318037	2	36	A:0.75	G:0.25	
chr28	11318059	2	36	C:0.75	T:0.25	
chr28	11318401	2	36	C:1	T:0	
chr28	11318755	2	38	C:0.947368	T:0.0526316	
chr28	11318974	2	34	C:0.411765	CT:0.588235	
chr28	11319198	2	36	A:0.444444	T:0.555556	
chr28	11319289	2	40	A:0.925	T:0.075	
chr28	11319292	2	40	T:0.875	A:0.125	
chr28	11319395	2	32	A:0	G:1	
chr28	11319487	2	34	C:0.970588	G:0.0294118	
chr28	11319777	2	40	G:0.9	A:0.1	
chr28	11320022	3	40	CTATATATATATATATATA:0.125	C:0.325	CTATATATATA:0.55
chr28	11320295	2	38	A:0.973684	AT:0.0263158	
chr28	11320463	2	34	G:0.970588	A:0.0294118	
chr28	11320692	2	40	A:0.8	AT:0.2	
chr28	11321220	3	40	CT:0.9	C:0.075	CTT:0.025
chr28	11321302	2	40	T:0.075	G:0.925	
chr28	11321615	2	40	A:0.875	AT:0.125	
chr28	11321688	2	40	G:0.9	C:0.1	
chr28	11321848	2	40	G:0.975	A:0.025	
chr28	11321885	2	40	G:0.825	A:0.175	
chr28	11321925	2	40	A:0.025	G:0.975	
chr28	11322298	2	40	G:0.375	A:0.625	
chr28	11322475	2	40	T:0.9	A:0.1	
chr28	11322736	2	38	T:0.973684	G:0.0263158	
chr28	11323142	2	40	G:0.85	A:0.15	
chr28	11323350	2	40	G:0.975	C:0.025	
chr28	11323370	2	38	TGAG:0.973684	T:0.0263158	
chr28	11324084	2	40	A:0.85	T:0.15	
chr28	11324431	3	40	GT:0.9	G:0.025	GTTTT:0.075
chr28	11324732	2	38	T:0.973684	C:0.0263158	

chr28	11325054	2	40	T:0.975	C:0.025
chr28	11325291	2	38	C:0.947368	G:0.0526316
chr28	11325305	2	36	T:0.916667	G:0.0833333
chr28	11325854	2	40	A:0.35	G:0.65
chr28	11326114	3	40	A:0.425
ACAGACATATCATTTTTTTTTTTTTTTAATTTTTTTTTTT:0.425
ACAGACATATCATTTTTTTTTTTTTTTTAATTTTTTTTTTT:0.15
chr28	11326177	2	36	A:0.972222	T:0.0277778
chr28	11326221	2	38	T:0.973684	G:0.0263158
chr28	11326512	2	36	A:0.972222	T:0.0277778
chr28	11326707	2	36	C:0.916667	T:0.0833333
chr28	11326756	2	36	A:0.972222	C:0.0277778
chr28	11326942	2	40	A:1	G:0
chr28	11327080	2	40	G:0.975	A:0.025
chr28	11327354	2	40	C:0.025	G:0.975
chr28	11327564	2	38	T:1	G:0
chr28	11327848	2	34	T:0.941176	C:0.0588235
chr28	11327995	2	36	T:0.361111	A:0.638889
chr28	11328012	2	40	T:0.85	A:0.15
chr28	11328494	2	28	A:0.321429	T:0.678571
chr28	11328539	2	30	C:0.3	T:0.7
chr28	11329088	3	38	AT:0.868421	A:0.131579
ATT:0
chr28	11329098	2	40	T:0.925	TC:0.075
chr28	11329128	2	40	C:0.975	CACAG:0.025
chr28	11329135	2	40	A:1	AG:0
chr28	11329136	3	40	A:0.975	AG:0	G:0.025
chr28	11329530	2	36	G:0.0833333	C:0.916667
chr28	11329742	2	36	T:0.944444	A:0.0555556
chr28	11329922	2	28	G:0.642857	GT:0.357143
chr28	11329945	2	14	G:0.714286	A:0.285714
chr28	11329971	2	26	GTGTATATATATATATATATATA:1	G:0
chr28	11329973	2	36	GTATATATATA:0.888889	G:
0.111111
chr28	11330070	2	38	T:1	C:0
chr28	11330116	3	30	GTT:0.166667	G:0.233333
GT:0.6
chr28	11330293	2	40	G:0.375	A:0.625
chr28	11331216	2	40	A:0.85	G:0.15
chr28	11331797	2	38	T:0.342105	G:0.657895
chr28	11332333	2	36	A:1	G:0
chr28	11332587	2	40	A:0.1	AAAAAC:0.9
chr28	11332761	2	36	G:1	A:0
chr28	11333661	2	40	G:0.1	GT:0.9
chr28	11333686	2	40	G:1	T:0
chr28	11333928	2	40	C:0.1	G:0.9
chr28	11333938	2	40	A:0.1	G:0.9
chr28	11334020	2	40	G:0.975	T:0.025
chr28	11334067	2	36	C:0.833333	A:0.166667
chr28	11334948	2	40	C:1	T:0
chr28	11335240	2	40	G:0.975	C:0.025
chr28	11335262	2	40	A:0.825	T:0.175
chr28	11335382	2	38	A:1	G:0
chr28	11336565	2	38	CT:0.789474	C:0.210526

chr28	11337174	2	40	C:0.975	T:0.025	
chr28	11337189	2	40	G:0.975	A:0.025	
chr28	11337192	2	40	T:0.075	C:0.925	
chr28	11338940	2	38	G:1	C:0	
chr28	11338972	2	40	T:0.975	C:0.025	
chr28	11338995	2	40	G:0.95	GT:0.05	
chr28	11339013	2	40	T:1	A:0	
chr28	11339016	2	40	G:0.9	GATTT:0.1	
chr28	11339345	2	38	T:0.0526316	C:0.947368	
chr28	11339675	2	38	T:1	C:0	
chr28	11340018	2	38	AT:0.868421	A:0.131579	
chr28	11340028	2	40	T:0.95	A:0.05	
chr28	11340268	2	40	C:1	T:0	
chr28	11340368	2	40	C:0.875	CT:0.125	
chr28	11340584	2	40	A:1	G:0	
chr28	11340714	2	40	C:1	T:0	
chr28	11340837	2	40	G:0	GT:1	
chr28	11340842	2	40	G:0	GT:1	
chr28	11341080	2	40	A:1	C:0	
chr28	11342374	2	36	A:0.5	G:0.5	
chr28	11342516	2	38	T:0.947368	C:0.0526316	
chr28	11342528	2	38	C:0.0526316	CCTCT:0.947368	
chr28	11342901	2	38	A:0.0526316	G:0.947368	
chr28	11342943	2	30	CTTT:0.433333	C:0.566667	
chr28	11343133	2	36	G:0.972222	T:0.0277778	
chr28	11343752	3	40	TTTTG:0.2	T:0.8	TTTTGTTTG:0
chr28	11343794	2	40	G:0.95	A:0.05	
chr28	11345173	2	34	C:0.911765	CT:0.0882353	
chr28	11345441	2	40	G:0.975	A:0.025	
chr28	11345575	2	36	T:0.916667	A:0.0833333	
chr28	11345772	2	40	A:1	G:0	
chr28	11346071	3	36	AT:0.888889	A:0.0833333	ATT:0.0277778
chr28	11346075	2	36	T:1	A:0	
chr28	11346082	2	36	T:1	A:0	
chr28	11346252	2	40	G:0	A:1	
chr28	11346276	2	40	G:0	A:1	
chr28	11346283	2	40	T:0	A:1	
chr28	11346284	2	40	G:0	A:1	
chr28	11346293	2	40	T:0	A:1	
chr28	11346369	2	40	CA:0	C:1	
chr28	11346741	2	40	G:1	A:0	
chr28	11347334	2	38	G:0.921053	GT:0.0789474	
chr28	11347473	2	38	G:1	C:0	
chr28	11347805	2	36	G:0.972222	T:0.0277778	
chr28	11348225	2	40	GT:0.9	G:0.1	
chr28	11348241	2	40	G:0.975	A:0.025	
chr28	11348405	2	38	T:0.131579	TTC:0.868421	
chr28	11348569	2	38	CAG:1	C:0	
chr28	11349440	2	40	A:0.975	G:0.025	
chr28	11349578	2	40	G:0.975	A:0.025	
chr28	11350096	2	40	T:1	A:0	
chr28	11350268	2	38	T:0.973684	TTGTG:0.0263158	

chr28	11350327	2	40	G:1	GT:0		
chr28	11350916	2	40	A:0.225	G:0.775		
chr28	11351877	2	40	G:1	C:0		
chr28	11352296	2	38	C:0.973684	T:0.0263158		
chr28	11352433	4	38	CTT:0.736842	C:0	CTTT:0.210526	CTTTT:0.0526316
chr28	11353222	2	36	T:0.972222	C:0.0277778		
chr28	11353570	2	38	T:0.105263	C:0.894737		
chr28	11353951	2	36	A:1	AG:0		
chr28	11353954	3	34	G:0.0588235	GT:0.941176	GTT:0	
chr28	11354007	3	32	TGTGTGGGGGGGG:0.71875	T:0.09375	TGG:0.1875	
chr28	11354009	2	32	TGTGGGGGGG:0.5625	T:0.4375		
chr28	11354011	2	34	TGGGGGGG:1	T:0		
chr28	11354189	2	38	T:1	C:0		
chr28	11355172	2	40	C:1	A:0		
chr28	11355407	3	40	CT:0.2	C:0.775	CTT:0.025	
chr28	11356045	2	40	G:1	A:0		
chr28	11356881	3	36	GAA:0.222222	G:0.25	GA:0.527778	
chr28	11358289	3	38	TA:0.236842	T:0.684211	TAA:0.0789474	
chr28	11358300	2	40	A:0.95	C:0.05		
chr28	11358751	2	38	T:1	A:0		
chr28	11358783	2	38	T:1	C:0		
chr28	11358785	2	38	C:0.973684	T:0.0263158		
chr28	11358995	2	38	A:1	T:0		
chr28	11359571	2	40	T:0.95	A:0.05		
chr28	11359911	3	38	CT:0.789474	C:0.0526316	CTT:0.157895	
chr28	11360031	2	38	A:1	G:0		
chr28	11360684	2	38	C:0	T:1		
chr28	11360703	2	38	TTGAC:1	T:0		
chr28	11360990	2	40	G:0.975	C:0.025		
chr28	11361323	2	40	C:1	T:0		
chr28	11361862	2	40	A:1	T:0		
chr28	11361867	2	40	A:0.975	G:0.025		
chr28	11362406	2	38	A:1	G:0		
chr28	11362523	2	40	A:1	G:0		
chr28	11362701	2	40	T:1	C:0		
chr28	11362897	2	38	A:0.236842	G:0.763158		
chr28	11362904	2	38	CAT:0.973684	C:0.0263158		
chr28	11363289	2	40	T:0.975	C:0.025		
chr28	11363460	2	34	CT:1	C:0		
chr28	11363642	2	38	T:0.105263	C:0.894737		
chr28	11363936	2	40	A:1	C:0		
chr28	11363960	2	38	G:1	A:0		
chr28	11364022	2	40	T:0.975	C:0.025		
chr28	11364054	2	40	TAAA:0.975	T:0.025		
chr28	11364938	2	38	C:0.973684	T:0.0263158		
chr28	11365347	2	40	T:0.975	C:0.025		
chr28	11366535	2	40	A:1	T:0		
chr28	11367224	2	34	A:0.970588	G:0.0294118		

chr28	11367266	2	40	G:0.975	A:0.025	
chr28	11367613	2	36	G:0.0277778	A:0.972222	
chr28	11368188	2	36	G:1	C:0	
chr28	11368883	2	38	G:1	A:0	
chr28	11368930	2	38	G:1	A:0	
chr28	11369634	2	38	C:1	T:0	
chr28	11369698	2	38	A:1	G:0	
chr28	11369707	2	40	C:1	T:0	
chr28	11369865	2	40	C:0.975	A:0.025	
chr28	11369931	2	38	T:0.105263	TAC:0.894737	
chr28	11370083	2	40	G:0.225	T:0.775	
chr28	11370174	2	38	C:0.157895	T:0.842105	
chr28	11370406	2	34	G:1	C:0	
chr28	11370827	2	40	TTC:0.725	T:0.275	
chr28	11370879	3	34	CA:0.323529	C:0.0294118	CAA:0.647059
chr28	11371043	2	38	T:1	A:0	
chr28	11372112	2	40	T:1	A:0	
chr28	11372115	2	40	A:1	AT:0	
chr28	11372357	2	38	G:1	A:0	
chr28	11372999	2	40	G:1	A:0	
chr28	11373109	2	38	A:0.973684	G:0.0263158	
chr28	11373317	2	36	A:1	AT:0	
chr28	11373539	2	38	G:0.973684	A:0.0263158	
chr28	11373568	2	40	G:0.975	A:0.025	
chr28	11374137	2	40	C:1	T:0	
chr28	11374318	2	40	G:1	A:0	
chr28	11375016	2	40	T:0.05	A:0.95	
chr28	11375064	2	38	T:0.973684	C:0.0263158	
chr28	11375204	2	38	C:0.921053	CT:0.0789474	
chr28	11375257	2	38	CAG:0.973684	C:0.0263158	
chr28	11376138	2	40	G:0.975	A:0.025	
chr28	11376448	2	38	A:1	T:0	
chr28	11376449	2	38	C:1	T:0	
chr28	11376645	2	36	C:0.972222	T:0.0277778	
chr28	11377176	2	34	C:0.0588235	T:0.941176	
chr28	11377785	2	40	GA:1	G:0	
chr28	11377847	2	38	T:0.131579	C:0.868421	
chr28	11377911	2	40	G:1	A:0	
chr28	11377940	2	40	GAT:0.85	G:0.15	
chr28	11378893	2	34	GT:0.294118	G:0.705882	
chr28	11379024	2	38	C:1	T:0	
chr28	11380042	3	40	G:1	GCCAAATAGTCTTTTTATTTTTTTTTTTTATTTTTTTATTTTTTTATTTTTTT:0	GCCAAATAGTCTTTTTATTTTTTTTTTTTATTTTTTTATTTTTTTTTTTTTTT:0
chr28	11380061	2	40	C:1	T:0	
chr28	11380180	2	40	C:1	CT:0	
chr28	11380262	2	38	A:0.473684	G:0.526316	
chr28	11380346	2	36	TAA:1	T:0	
chr28	11380606	2	40	G:0.9	GTTTAGTAACA:0.1	
chr28	11380786	2	40	TG:0.975	T:0.025	
chr28	11381145	3	36	A:0.111111	ATT:0.138889	ATTAT:0.75
chr28	11381146	2	36	A:0.972222	T:0.0277778	

chr28	11381457	3	40	CTTAT:0.975	C:0.025	CTTATTTAT:0
chr28	11381771	2	40	C:0.075	T:0.925	
chr28	11381789	2	40	G:0.975	A:0.025	
chr28	11381828	2	40	C:0.975	A:0.025	
chr28	11382448	2	40	G:1	A:0	
chr28	11382674	2	40	C:0.125	T:0.875	
chr28	11382703	2	40	G:1	A:0	
chr28	11382784	2	34	C:0.970588	CT:0.0294118	
chr28	11382796	2	36	C:0.861111	CT:0.138889	
chr28	11382890	2	38	A:0.973684	G:0.0263158	
chr28	11382915	2	38	CG:0.263158	C:0.736842	
chr28	11382929	2	38	C:0.447368	T:0.552632	
chr28	11383210	2	40	C:1	T:0	
chr28	11384031	2	34	C:1	A:0	
chr28	11384336	2	38	T:1	C:0	
chr28	11384435	3	38	GTTTTTTTTTTTTT:0.736842	G:0.263158	GTT:0
chr28	11384574	2	38	G:1	A:0	
chr28	11384657	2	40	C:1	A:0	
chr28	11385087	2	40	C:0.05	T:0.95	
chr28	11385266	2	40	C:0.975	T:0.025	
chr28	11385325	2	40	G:1	A:0	
chr28	11385610	2	40	C:0.125	T:0.875	
chr28	11385832	2	34	CA:0.117647	C:0.882353	
chr28	11385878	2	38	G:0.973684	A:0.0263158	
chr28	11386115	2	34	C:0.970588	T:0.0294118	
chr28	11386490	2	38	T:0.210526	A:0.789474	
chr28	11386521	2	40	T:0.975	G:0.025	
chr28	11386861	3	36	TAA:0.25	T:0.25	TA:0.5
chr28	11387684	2	40	T:1	G:0	
chr28	11387819	2	40	ATGATTTTAAT:1	A:0	
chr28	11388558	2	38	TA:0.789474	T:0.210526	
chr28	11388762	2	40	G:0.15	A:0.85	
chr28	11388982	2	40	A:0.925	G:0.075	
chr28	11389436	2	36	C:0.972222	CT:0.0277778	
chr28	11389806	2	40	C:0.95	T:0.05	
chr28	11390211	2	38	G:0.947368	A:0.0526316	
chr28	11390298	2	36	G:0.0833333	A:0.916667	
chr28	11390661	2	38	CTT:0.131579	C:0.868421	
chr28	11390684	2	38	C:0.973684	A:0.0263158	
chr28	11391337	2	38	G:1	A:0	
chr28	11392149	2	40	G:0.975	A:0.025	
chr28	11392155	2	40	C:0.975	T:0.025	
chr28	11392243	2	40	GTGAAAT:0.975	G:0.025	
chr28	11392462	2	40	GAC:1	G:0	
chr28	11393180	2	38	G:0.289474	GT:0.710526	
chr28	11393771	2	40	G:0.8	GAGAGAGAGAGAGAGAGAGAGAA:0.2	
chr28	11393782	2	38	A:0.657895	AG:0.342105	
chr28	11393783	3	40	A:0.625	AG:0.35	AGAGAGAGAAAGAG:0.025
chr28	11393791	2	38	A:0.973684	AAGAG:0.0263158	
chr28	11393795	2	38	GAGAA:0.657895	G:0.342105	

chr28	11393845	2	40	G:0.9	GAA:0.1	
chr28	11393846	2	40	AG:0.175	A:0.825	
chr28	11393849	2	40	A:1	AAG:0	
chr28	11393850	3	40	A:0.15	G:0.825	AG:0.025
chr28	11393870	2	40	GA:0.975	G:0.025	
chr28	11393942	2	38	T:0.631579	TTA:0.368421	
chr28	11393957	2	40	TA:1	T:0	
chr28	11393959	2	40	T:0.85	TATA:0.15	
chr28	11394662	2	40	G:1	A:0	
chr28	11395143	2	38	A:0.973684	T:0.0263158	
chr28	11395453	2	40	C:0.975	T:0.025	
chr28	11395604	3	38	CTT:0.868421	C:0.105263	TTT:0.0263158
chr28	11395984	2	34	C:0.970588	T:0.0294118	
chr28	11396231	2	38	G:0.0526316	T:0.947368	
chr28	11396469	2	38	C:0.210526	T:0.789474	
chr28	11396726	2	38	T:0.973684	C:0.0263158	
chr28	11396729	2	38	A:0.973684	G:0.0263158	
chr28	11396817	2	40	C:0.975	T:0.025	
chr28	11396858	2	40	C:0.975	A:0.025	
chr28	11397076	2	40	G:1	A:0	
chr28	11397620	2	36	GTT:0.0833333	G:0.916667	
chr28	11397684	2	40	AAGAG:0.25	A:0.75	
chr28	11397748	2	36	A:0.0555556	G:0.944444	
chr28	11397771	2	36	C:0.916667	T:0.0833333	
chr28	11398479	2	40	T:0.2	C:0.8	
chr28	11398842	2	34	G:1	A:0	
chr28	11399261	2	38	T:0.973684	C:0.0263158	
chr28	11399472	2	36	C:0.972222	T:0.0277778	
chr28	11400792	2	40	A:0.075	T:0.925	
chr28	11401721	2	40	G:0.175	A:0.825	
chr28	11401844	2	40	G:1	A:0	
chr28	11401962	2	38	G:1	A:0	
chr28	11402177	2	38	C:0.710526	A:0.289474	
chr28	11402263	2	38	C:0.973684	T:0.0263158	
chr28	11402468	2	38	G:0.973684	T:0.0263158	
chr28	11402469	2	38	A:0.973684	T:0.0263158	
chr28	11402596	2	36	T:1	C:0	
chr28	11402600	3	40	CCTCTCTCTCT:0.275	C:0.15	CCTCTCTCTCTCTCT:0.575
chr28	11402645	2	22	TA:0.5	T:0.5	
chr28	11402796	2	40	C:1	T:0	
chr28	11403468	2	40	C:0.975	T:0.025	
chr28	11403533	2	38	A:0.973684	G:0.0263158	
chr28	11403536	2	38	T:1	C:0	
chr28	11404631	2	36	T:0.194444	C:0.805556	
chr28	11404670	2	40	A:1	C:0	
chr28	11404882	2	40	G:0.075	C:0.925	
chr28	11406064	2	38	C:1	T:0	
chr28	11406676	2	36	T:0.916667	C:0.0833333	
chr28	11406697	2	38	T:0.263158	A:0.736842	
chr28	11406738	2	38	AT:0.157895	A:0.842105	
chr28	11406755	2	38	G:0.289474	A:0.710526	
chr28	11406933	2	36	G:0.944444	A:0.0555556	

chr28	11406993	2	36	T:0.111111	C:0.888889
chr28	11407521	2	36	G:0.0833333	GT:0.916667
chr28	11407834	2	40	G:0.95	A:0.05
chr28	11409745	2	38	T:0.0526316	C:0.947368
chr28	11410506	2	40	T:1	C:0
chr28	11410817	2	40	G:1	C:0
chr28	11410823	2	38	AT:0.157895	A:0.842105
chr28	11411325	2	34	G:0.764706	A:0.235294
chr28	11411580	2	40	C:0.35	A:0.65
chr28	11411684	2	38	G:0.105263	A:0.894737
chr28	11411741	2	36	T:0	C:1
chr28	11411795	2	34	A:0.970588	G:0.0294118
chr28	11411861	2	36	T:0.222222	C:0.777778
chr28	11411938	2	38	C:0.868421	T:0.131579
chr28	11411995	2	38	CCCA:0.105263	C:0.894737
chr28	11412144	2	38	A:0.184211	C:0.815789
chr28	11412181	2	40	T:0.175	G:0.825
chr28	11412203	2	40	C:0.175	T:0.825
chr28	11412265	2	40	T:0.975	C:0.025
chr28	11412370	2	36	C:0.444444	T:0.555556
chr28	11412429	2	38	G:0.973684	A:0.0263158
chr28	11412463	2	38	A:0.0526316	G:0.947368
chr28	11412904	2	38	C:0.394737	T:0.605263
chr28	11412963	2	38	A:0.131579	C:0.868421
chr28	11413178	2	38	A:0.157895	G:0.842105
chr28	11413588	2	38	T:0.105263	C:0.894737
chr28	11413779	2	36	G:0.0833333	C:0.916667
chr28	11415240	2	36	C:0.777778	T:0.222222
chr28	11415441	2	38	G:1	GC:0
chr28	11415479	2	38	G:0.973684	A:0.0263158
chr28	11415635	2	38	C:1	A:0
chr28	11415710	2	38	C:0.0789474	T:0.921053
chr28	11416137	2	38	T:0.157895	G:0.842105
chr28	11416184	2	40	C:0.875	T:0.125
chr28	11416385	2	40	C:0.1	T:0.9
chr28	11416393	2	40	A:0.1	G:0.9
chr28	11416421	2	38	C:0.0789474	T:0.921053
chr28	11416931	2	40	G:0.8	A:0.2
chr28	11417125	2	38	C:0.0526316	A:0.947368
chr28	11417144	2	40	C:0.05	G:0.95
chr28	11417358	2	34	G:0.0588235	A:0.941176
chr28	11417380	2	38	T:0.0526316	C:0.947368
chr28	11417401	2	38	G:0.0263158	T:0.973684
chr28	11417573	2	36	C:1	T:0
chr28	11417577	2	38	A:0.0526316	G:0.947368
chr28	11417769	2	40	C:1	T:0
chr28	11418418	2	38	C:0.947368	CTTA:0.0526316
chr28	11418658	2	40	G:0.975	A:0.025
chr28	11418862	2	32	C:0.09375	T:0.90625
chr28	11419163	2	32	G:0	A:1
chr28	11419363	2	28	A:0.607143	T:0.392857
chr28	11420115	2	30	C:0.166667	G:0.833333
chr28	11420140	2	34	G:0.941176	A:0.0588235
chr28	11420191	2	28	A:0.25	T:0.75

chr28	11420278	2	28	G:0.75	A:0.25
chr28	11420342	2	30	G:1	GC:0
chr28	11420347	2	30	G:0.1	GC:0.9
chr28	11420354	2	30	C:0.1	T:0.9
chr28	11420399	2	30	AC:0.133333	A:0.866667
chr28	11420410	2	30	C:0.133333	T:0.866667
chr28	11420543	2	24	C:0.166667	G:0.833333
chr28	11420689	2	24	T:0.708333	A:0.291667
chr28	11421086	2	30	C:0.766667	T:0.233333
chr28	11421306	2	36	C:0.111111	G:0.888889
chr28	11421383	2	36	C:0.166667	T:0.833333
chr28	11421567	2	40	T:0.3	C:0.7
chr28	11421735	2	36	C:0.0833333	A:0.916667
chr28	11421942	2	38	T:0.0526316	C:0.947368
chr28	11422206	2	38	G:0.0526316	A:0.947368
chr28	11422385	2	40	CT:0.85	C:0.15
chr28	11422386	2	40	T:0.225	C:0.775
chr28	11422387	2	40	G:0.85	C:0.15
chr28	11422461	2	38	G:0.473684	A:0.526316
chr28	11422483	2	38	G:0.578947	A:0.421053
chr28	11422662	2	38	G:1	C:0
chr28	11422677	2	40	C:0.675	T:0.325
chr28	11422834	2	40	C:0.825	G:0.175
chr28	11423120	2	38	G:0.105263	A:0.894737
chr28	11423200	2	40	G:0.475	A:0.525
chr28	11423349	2	38	G:0.5	A:0.5
chr28	11423590	2	38	G:0.236842	A:0.763158
chr28	11424170	2	38	C:0.0526316	CT:0.947368
chr28	11424205	2	36	C:0.833333	T:0.166667
chr28	11424287	2	36	T:0.861111	TG:0.138889
chr28	11424305	2	36	C:0.388889	T:0.611111
chr28	11424429	3	36	ATTTTTTT:0.694444	A:
0.305556	ATTTT:0				
chr28	11424609	2	38	C:0.842105	G:0.157895
chr28	11424683	2	34	T:0.823529	TTGAAC:0.176471
chr28	11424708	2	32	C:0.96875	T:0.03125
chr28	11424721	2	34	T:0.411765	C:0.588235
chr28	11424818	2	32	C:1	T:0
chr28	11424884	2	28	C:1	T:0
chr28	11425255	2	40	C:0.775	T:0.225
chr28	11425269	2	40	T:0.3	C:0.7
chr28	11425339	2	38	G:0.789474	C:0.210526
chr28	11425346	2	40	G:0.8	A:0.2
chr28	11425922	2	38	G:0.842105	T:0.157895
chr28	11425958	2	40	A:0.85	C:0.15
chr28	11426016	2	36	A:0.805556	AT:0.194444
chr28	11426159	2	40	A:0.8	G:0.2
chr28	11426266	2	34	G:0.617647	A:0.382353
chr28	11426282	2	38	T:0.842105	C:0.157895
chr28	11426438	2	34	T:0.794118	A:0.205882
chr28	11426621	2	40	A:0.5	G:0.5
chr28	11426737	2	40	A:0.8	C:0.2
chr28	11426791	2	40	T:0.85	C:0.15
chr28	11427406	2	38	A:0.736842	C:0.263158

chr28 11427446 2 38 G:0.736842 A:0.263158
chr28 11427512 2 38 T:1 G:0
chr28 11427715 2 38 C:1 T:0
chr28 11427820 2 40 G:0.85 T:0.15
chr28 11427838 2 40 T:0.825 C:0.175
chr28 11427908 2 34 C:1 A:0
chr28 11428001 2 38 G:0.842105 C:0.157895
chr28 11428014 2 38 C:0.842105 T:0.157895
chr28 11428071 2 36 T:0.75 C:0.25
chr28 11428105 2 36 T:0.833333 G:0.166667
chr28 11428263 2 38 A:0.789474 G:0.210526
chr28 11428386 2 38 A:1 G:0
chr28 11428887 2 38 T:0.842105 C:0.157895
chr28 11428889 2 38 A:0.763158 G:0.236842
chr28 11428965 2 40 A:0.625 C:0.375
chr28 11429020 2 40 A:1 G:0
chr28 11429207 2 38 GTT:0.815789 G:0.184211
chr28 11429220 2 38 C:0.789474 T:0.210526
chr28 11429733 2 36 A:0.416667 T:0.583333
chr28 11429844 2 36 G:0.722222 A:0.277778
chr28 11429874 2 38 A:0.605263 G:0.394737
chr28 11429887 2 38 C:0.815789 CTT:0.184211
chr28 11430082 2 36 A:0.555556 T:0.444444
chr28 11430108 2 36 C:0.527778 T:0.472222
chr28 11430770 2 38 A:0.684211 C:0.315789
chr28 11431115 2 40 T:0.775 C:0.225
chr28 11431529 2 40 G:0.85 C:0.15
chr28 11431632 2 40 A:0.875 G:0.125
chr28 11431899 2 40 G:0.8 A:0.2
chr28 11432698 2 36 T:0.805556 A:0.194444
chr28 11432793 2 36 C:0.361111 T:0.638889
chr28 11432958 2 40 TA:1 T:0
chr28 11433004 2 38 C:1 T:0
chr28 11433034 2 40 C:1 G:0
chr28 11433414 2 40 T:0.8 TAAACAA:0.2
chr28 11434096 2 38 CTTTT:0.526316 C:0.473684
chr28 11434131 2 36 T:0.638889 C:0.361111
chr28 11434149 2 36 G:0.638889 A:0.361111
chr28 11434291 2 34 A:0.588235 C:0.411765
chr28 11434368 2 38 G:0.578947 A:0.421053
chr28 11434415 2 40 G:0.8 A:0.2
chr28 11434527 2 34 A:0.617647 G:0.382353
chr28 11434650 2 34 T:0.529412 TGTG:0.470588
chr28 11435664 2 34 G:1 A:0
chr28 11435675 2 36 G:1 T:0
chr28 11435691 2 36 C:0.972222 T:0.0277778
chr28 11435692 2 36 G:0.972222 T:0.0277778
chr28 11435780 2 40 G:0.825 C:0.175
chr28 11436332 2 36 G:0.555556 A:0.444444
chr28 11436376 2 38 A:0.0789474 G:0.921053
chr28 11436526 5 40 GGA:0.325 G:0.275 GGAGA:0.175 GGAGAGA:0.1 GGAGAGAGA:0.125
chr28 11436632 2 36 C:1 T:0
chr28 11436634 2 36 G:0.694444 A:0.305556

chr28	11436761	2	38	C:0.710526	T:0.289474
chr28	11436807	2	36	T:0.583333	C:0.416667
chr28	11436968	2	38	G:0.842105	A:0.157895
chr28	11437489	2	38	A:1	C:0
chr28	11437610	2	38	T:0.0526316	C:0.947368
chr28	11437708	2	38	C:0.421053	A:0.578947
chr28	11438036	2	36	C:0.805556	T:0.194444
chr28	11438150	2	40	CT:0.125	C:0.875
chr28	11438221	2	40	T:0.775	C:0.225
chr28	11438900	2	38	G:0.342105	A:0.657895
chr28	11439207	2	40	C:0.475	T:0.525
chr28	11439218	2	40	T:0.475	C:0.525
chr28	11439221	2	40	G:1	T:0
chr28	11439498	2	38	C:0.263158	T:0.736842
chr28	11439553	2	38	C:0.842105	T:0.157895
chr28	11439861	2	40	A:0.6	C:0.4
chr28	11439912	2	34	G:0.529412	A:0.470588
chr28	11440127	2	36	C:1	T:0
chr28	11440362	2	36	C:1	T:0
chr28	11440596	2	30	A:0.8	T:0.2
chr28	11440624	2	36	TG:0.111111	T:0.888889
chr28	11440955	2	38	C:0.526316	A:0.473684
chr28	11440979	2	38	C:0.5	T:0.5
chr28	11441034	2	34	C:0.764706	A:0.235294
chr28	11441221	2	36	C:0.861111	T:0.138889
chr28	11441237	2	32	T:0.34375	C:0.65625
chr28	11441467	2	38	C:0.868421	T:0.131579
chr28	11441654	2	36	C:0.25	T:0.75
chr28	11441685	2	36	T:0.527778	G:0.472222
chr28	11441756	2	32	A:0.21875	G:0.78125
chr28	11441841	2	36	T:0.277778	C:0.722222
chr28	11441881	2	34	G:0.235294	A:0.764706
chr28	11441889	2	34	T:0.235294	C:0.764706
chr28	11442035	2	40	A:0.275	C:0.725
chr28	11442388	2	40	G:0.275	C:0.725
chr28	11442453	2	40	T:0.975	A:0.025
chr28	11443094	2	40	A:0.05	G:0.95
chr28	11443106	2	40	G:0.425	A:0.575
chr28	11443191	2	38	G:0.105263	A:0.894737
chr28	11443231	2	40	A:0.1	T:0.9
chr28	11443345	2	40	T:0.425	C:0.575
chr28	11443407	2	38	C:0.421053	A:0.578947
chr28	11443603	2	30	CTATCT:0.933333	C:0.0666667
chr28	11443605	2	40	ATCTAATCT:0.9	A:0.1
chr28	11443607	2	40	CT:0.7	C:0.3
chr28	11443608	2	40	TA:0.825	T:0.175
chr28	11443609	2	40	AATCT:0.95	A:0.05
chr28	11443610	2	32	ATCT:0.5	A:0.5
chr28	11443612	2	40	C:0.825	CA:0.175
chr28	11443613	2	40	T:0.825	A:0.175
chr28	11443968	2	40	T:0.45	C:0.55
chr28	11444045	2	40	G:0.55	C:0.45
chr28	11444217	2	38	G:0.526316	A:0.473684
chr28	11444259	2	34	C:0.735294	T:0.264706

chr28	11444508	3	4	TAA:0.25	T:0.75	TA:0
chr28	11444585	2	38	C:0.578947	G:0.421053
chr28	11444718	2	38	A:0.868421	G:0.131579
chr28	11445107	2	38	A:0.473684	AG:0.526316
chr28	11445115	2	36	G:0.5	C:0.5
chr28	11446124	2	40	C:0.875	G:0.125
chr28	11446159	2	38	C:0.473684	T:0.526316
chr28	11446167	2	38	G:0.473684	A:0.526316
chr28	11446192	2	36	C:0.694444	T:0.305556
chr28	11446200	2	36	G:0.444444	C:0.555556
chr28	11446255	2	38	G:1	A:0
chr28	11446275	2	38	T:0.473684	G:0.526316
chr28	11446490	2	32	T:0.1875	C:0.8125
chr28	11446497	2	32	G:0.5625	A:0.4375
chr28	11446963	2	38	G:1	A:0
chr28	11447015	2	36	A:0.111111	G:0.888889
chr28	11447231	2	38	G:0.842105	A:0.157895
chr28	11447532	2	38	G:0.394737	A:0.605263
chr28	11447712	2	38	T:0.631579	TATCTC:0.368421
chr28	11448227	2	40	AGCTGGTCCAT:0.75	A:0.25
chr28	11448339	2	38	T:0.710526	G:0.289474
chr28	11448341	2	38	T:0.868421	C:0.131579
chr28	11448848	2	40	CAACAGGG:0.65	C:0.35
chr28	11449428	2	36	C:0.916667	T:0.0833333
chr28	11449436	2	34	AC:0.735294	A:0.264706
chr28	11449587	2	40	G:0.9	A:0.1
chr28	11449611	2	40	A:0.85	G:0.15
chr28	11449847	2	40	C:0.675	T:0.325
chr28	11449862	2	38	A:0.684211	G:0.315789
chr28	11449948	2	38	A:0.894737	G:0.105263
chr28	11450011	2	40	C:0.925	A:0.075
chr28	11450081	2	40	T:0.475	G:0.525
chr28	11450420	2	40	C:0.825	T:0.175
chr28	11450463	2	40	C:0.825	T:0.175
chr28	11450545	2	40	A:0.925	T:0.075
chr28	11450739	2	40	G:0.85	A:0.15
chr28	11450777	2	38	T:0.289474	C:0.710526
chr28	11450789	2	38	A:0.289474	C:0.710526
chr28	11450968	2	40	G:0.25	A:0.75
chr28	11451049	2	40	A:0.725	AGG:0.275
chr28	11451066	2	40	A:0.2	G:0.8
chr28	11451117	2	40	G:0.5	C:0.5
chr28	11451126	2	40	C:0.75	CGACTGGTGG:0.25
chr28	11451168	2	40	A:0.725	C:0.275
chr28	11451413	2	34	A:0.823529	G:0.176471
chr28	11452138	2	36	T:0.805556	C:0.194444
chr28	11452230	2	30	G:0.766667	A:0.233333
chr28	11452504	2	20	AGG:0.7	A:0.3
chr28	11452694	2	32	G:0.84375	T:0.15625
chr28	11452814	2	40	T:0.775	C:0.225
chr28	11452912	2	38	C:0.842105	T:0.157895
chr28	11452913	2	38	A:0.684211	G:0.315789
chr28	11452971	2	38	T:0.868421	C:0.131579
chr28	11452972	2	38	TGTG:0.815789	T:0.184211

chr28	11452982	2	38	C:0.815789	G:0.184211
chr28	11453002	2	38	T:0.815789	C:0.184211
chr28	11453120	2	40	C:0.8	A:0.2
chr28	11453144	2	38	G:0.947368	A:0.0526316
chr28	11453154	2	38	T:0.789474	C:0.210526
chr28	11453161	2	38	T:0.789474	G:0.210526
chr28	11453179	2	38	T:0.789474	C:0.210526
chr28	11453221	2	38	G:0.815789	A:0.184211
chr28	11453242	2	38	A:0.815789	C:0.184211
chr28	11453243	2	38	C:0.815789	T:0.184211
chr28	11453355	2	38	C:0.894737	T:0.105263
chr28	11453388	2	38	C:0.710526	CT:0.289474
chr28	11453520	2	38	G:0.763158	A:0.236842
chr28	11453524	2	38	C:0	CT:1
chr28	11453566	2	38	G:0.789474	A:0.210526
chr28	11453615	2	38	C:0.842105	T:0.157895
chr28	11453665	2	40	AG:0.875	A:0.125
chr28	11453669	2	40	G:0.825	A:0.175
chr28	11453727	2	40	T:0.825	A:0.175
chr28	11453737	2	40	T:0.825	C:0.175
chr28	11453738	2	40	A:0.825	T:0.175
chr28	11453762	2	40	A:0.725	G:0.275
chr28	11453969	2	38	C:0.736842	A:0.263158
chr28	11454093	2	38	GC:0.710526	G:0.289474
chr28	11454095	2	38	AT:0.710526	A:0.289474
chr28	11454115	2	38	C:0.710526	T:0.289474
chr28	11454364	2	40	G:1	A:0
chr28	11454578	2	40	GA:0.65	G:0.35
chr28	11454670	2	40	GA:0.825	G:0.175
chr28	11454721	2	38	C:0.868421	T:0.131579
chr28	11454767	2	34	C:0.882353	G:0.117647
chr28	11454777	2	32	GT:1	G:0
chr28	11454794	2	38	G:0.842105	A:0.157895
chr28	11454897	2	38	C:0.842105	T:0.157895
chr28	11454945	2	36	G:0.861111	A:0.138889
chr28	11455006	2	36	G:0.833333	A:0.166667
chr28	11455089	2	40	G:0.925	A:0.075
chr28	11455146	2	40	TAGGTCAC:0.875	T:0.125
chr28	11455185	2	38	C:0.947368	A:0.0526316
chr28	11455244	2	36	G:0.888889	A:0.111111
chr28	11455431	2	36	C:0.888889	T:0.111111
chr28	11455510	2	34	A:0.882353	G:0.117647
chr28	11455519	2	34	T:0.882353	A:0.117647
chr28	11455548	2	38	T:0.894737	G:0.105263
chr28	11455648	2	38	A:0.684211	C:0.315789
chr28	11456272	2	40	T:0.7	C:0.3
chr28	11456386	2	40	A:0.825	C:0.175
chr28	11456427	2	38	C:0.736842	T:0.263158
chr28	11456677	2	40	T:0.7	C:0.3
chr28	11456918	2	40	A:0.9	G:0.1
chr28	11457146	2	36	A:0.888889	T:0.111111
chr28	11457445	2	38	A:0.842105	G:0.157895
chr28	11457551	2	40	C:1	T:0
chr28	11457766	2	34	G:0.852941	A:0.147059

chr28 11458514 2 40 C:1 T:0
chr28 11458601 2 38 G:1 A:0
chr28 11458672 2 40 A:0.775 AT:0.225
chr28 11458715 2 38 T:0.868421 C:0.131579
chr28 11458967 2 38 T:1 A:0
chr28 11459007 2 36 A:1 AG:0
chr28 11459431 2 40 T:1 A:0
chr28 11459445 2 40 G:0.9 A:0.1
chr28 11459521 2 36 G:0.833333 A:0.166667
chr28 11459530 2 36 G:0.888889 A:0.111111
chr28 11459536 2 38 G:0.973684 A:0.0263158
chr28 11459549 2 40 C:1 T:0
chr28 11459552 2 40 G:0.85 A:0.15
chr28 11459572 2 40 G:0.85 A:0.15
chr28 11459585 2 40 G:1 A:0
chr28 11459667 2 38 A:0.921053 G:0.0789474
chr28 11459673 2 38 AT:0.657895 A:0.342105
chr28 11459680 2 38 C:0.736842 A:0.263158
chr28 11459682 2 38 A:0.657895 C:0.342105
chr28 11459865 3 36 TG:0.527778 T:0.305556
GG:0.166667
chr28 11459956 2 40 C:0.9 T:0.1
chr28 11460400 2 38 C:0.789474 CT:0.210526
chr28 11460725 2 38 T:0.815789 C:0.184211
chr28 11460854 2 36 C:0.833333 T:0.166667
chr28 11461325 3 36 TAA:0.444444 T:0.111111
TA:0.444444
chr28 11461353 2 36 C:0.777778 T:0.222222
chr28 11461437 2 40 A:0.9 G:0.1
chr28 11461946 2 40 G:1 T:0
chr28 11463041 2 40 T:0.65 C:0.35
chr28 11463062 2 40 A:0.65 G:0.35
chr28 11463108 2 40 T:0.95 A:0.05
chr28 11463293 2 40 CG:0.725 C:0.275
chr28 11463420 2 38 T:0.631579 C:0.368421
chr28 11463474 2 40 G:1 T:0
chr28 11464030 2 38 A:0.736842 C:0.263158
chr28 11464210 2 40 T:0.575 C:0.425
chr28 11464364 2 36 A:0.111111 T:0.888889
chr28 11464436 2 34 C:1 A:0
chr28 11465027 2 38 C:0.631579 G:0.368421
chr28 11465227 2 40 G:0.8 T:0.2
chr28 11465587 2 40 T:0.65 C:0.35
chr28 11465771 2 40 T:0.775 G:0.225
chr28 11466352 2 38 T:0.657895 C:0.342105
chr28 11466397 2 36 G:0.916667 A:0.0833333
chr28 11466444 2 38 A:0.657895 G:0.342105
chr28 11467469 2 38 C:0.973684 A:0.0263158
chr28 11467596 4 40 AGGATGGAT:0.575 A:0.275 AGGAT:0
AGGATGGATGGAT:0.15
chr28 11469003 2 38 G:0.894737 A:0.105263
chr28 11469347 2 40 T:0.65 C:0.35
chr28 11469374 2 40 A:0.875 T:0.125
chr28 11469448 2 38 G:1 A:0

chr28	11469466	2	40	C:0.975	T:0.025
chr28	11469490	2	40	G:0.85	A:0.15
chr28	11469498	2	40	A:0.875	G:0.125
chr28	11469666	2	40	G:0.95	C:0.05
chr28	11469897	2	38	G:1	C:0
chr28	11470230	2	38	T:1	C:0
chr28	11470342	2	38	TGTCA:0.947368	T:0.0526316
chr28	11471124	2	36	G:0.861111	C:0.138889
chr28	11471161	2	38	G:1	A:0
chr28	11471168	2	38	C:1	T:0
chr28	11471323	2	40	GATATAAATCTGCACACA:0.775	G:0.225
chr28	11471334	2	40	G:0.875	A:0.125
chr28	11471434	2	38	C:0.763158	T:0.236842
chr28	11471804	2	38	T:0.763158	G:0.236842
chr28	11471966	2	40	T:0.8	C:0.2
chr28	11472208	2	40	A:0.775	G:0.225
chr28	11472488	2	40	T:0.65	G:0.35
chr28	11472688	2	32	G:0.78125	A:0.21875
chr28	11472799	2	40	C:0.825	T:0.175
chr28	11472986	2	38	T:0.973684	C:0.0263158
chr28	11473001	2	38	C:0.763158	T:0.236842
chr28	11473356	2	40	C:0.75	G:0.25
chr28	11473384	2	38	G:1	T:0
chr28	11473414	2	40	A:0.975	T:0.025
chr28	11473688	2	40	A:1	G:0
chr28	11474130	2	40	T:0.875	C:0.125
chr28	11474197	2	40	T:0.85	C:0.15
chr28	11474961	2	36	G:0.916667	A:0.0833333
chr28	11475340	2	38	A:0.868421	G:0.131579
chr28	11475421	2	40	G:0.875	C:0.125
chr28	11475581	2	38	GT:0.710526	G:0.289474
chr28	11475811	2	38	G:0.894737	A:0.105263
chr28	11476024	2	38	G:0.894737	A:0.105263
chr28	11476538	2	38	A:0.868421	G:0.131579
chr28	11476652	2	36	G:0.916667	A:0.0833333
chr28	11476824	2	38	G:0.868421	A:0.131579
chr28	11477140	2	36	G:0.888889	A:0.111111
chr28	11477164	2	36	G:1	C:0
chr28	11477263	2	40	C:1	CATATCTT:0
chr28	11477418	2	38	C:0.894737	T:0.105263
chr28	11477480	2	40	T:0.9	G:0.1
chr28	11477587	2	38	T:0.868421	C:0.131579
chr28	11477592	2	38	C:0.868421	G:0.131579
chr28	11477687	2	40	G:0.875	T:0.125
chr28	11477721	2	40	C:0.9	T:0.1
chr28	11477917	2	40	G:0.525	C:0.475
chr28	11477984	2	40	C:0.875	T:0.125
chr28	11478176	2	34	C:0.794118	G:0.205882
chr28	11478211	2	36	T:0.861111	C:0.138889
chr28	11478307	2	38	T:0.894737	C:0.105263
chr28	11478461	2	38	G:0.868421	A:0.131579
chr28	11478477	2	38	T:0.894737	C:0.105263
chr28	11478481	3	38	C:0.894737	T:0.105263
G:0					

chr28	11478581	2	40	G:0.9	C:0.1	
chr28	11478646	2	40	T:1	C:0	
chr28	11478861	2	38	C:0.894737	A:0.105263	
chr28	11478872	2	38	A:0.894737	G:0.105263	
chr28	11478879	2	38	T:0.763158	C:0.236842	
chr28	11479017	2	40	A:0.925	G:0.075	
chr28	11480928	2	38	G:0.789474	T:0.210526	
chr28	11481038	2	40	A:0.65	G:0.35	
chr28	11481693	2	36	G:0.805556	T:0.194444	
chr28	11482073	2	40	G:0.8	A:0.2	
chr28	11482106	2	40	G:0.875	GA:0.125	
chr28	11482887	2	40	G:0.85	T:0.15	
chr28	11483039	2	36	C:1	G:0	
chr28	11483102	2	36	G:0.861111	GT:0.138889	
chr28	11483166	2	38	C:1	A:0	
chr28	11483215	2	36	A:0.833333	C:0.166667	
chr28	11483322	2	38	T:1	C:0	
chr28	11483461	2	40	G:1	A:0	
chr28	11483593	2	36	T:0.805556	C:0.194444	
chr28	11483649	2	36	T:1	C:0	
chr28	11483657	2	36	C:0.805556	T:0.194444	
chr28	11483764	2	36	T:0.861111	C:0.138889	
chr28	11483777	2	36	A:0.861111	G:0.138889	
chr28	11483812	2	40	A:1	AAAAGAAAAAG:0	
chr28	11483849	2	38	A:0.631579	G:0.368421	
chr28	11484111	2	40	C:0.7	T:0.3	
chr28	11484522	2	34	T:0.823529	C:0.176471	
chr28	11484626	2	38	G:0.868421	C:0.131579	
chr28	11484728	2	36	C:0.805556	T:0.194444	
chr28	11484740	2	36	C:1	T:0	
chr28	11484799	2	36	C:0.861111	T:0.138889	
chr28	11484800	2	36	G:1	A:0	
chr28	11484987	2	38	G:1	A:0	
chr28	11485184	2	34	T:1	C:0	
chr28	11485308	2	38	G:0.894737	A:0.105263	
chr28	11485412	2	38	G:1	A:0	
chr28	11485563	2	40	CT:0.8	C:0.2	
chr28	11485583	2	40	A:0.85	G:0.15	
chr28	11485671	3	38	A:0.763158	C:0.236842	T:0
chr28	11485684	2	38	C:1	T:0	
chr28	11485793	2	40	C:1	T:0	
chr28	11485949	2	40	G:0.875	T:0.125	
chr28	11485966	2	40	T:0.775	TGTTTTTG:0.225	
chr28	11486019	2	36	T:0.805556	C:0.194444	
chr28	11486116	2	40	G:0.95	A:0.05	
chr28	11486158	2	40	G:0.825	A:0.175	
chr28	11486231	2	38	T:1	C:0	
chr28	11486251	2	36	C:1	G:0	
chr28	11486293	2	36	T:0.638889	TG:0.361111	
chr28	11486369	2	40	A:0.825	T:0.175	
chr28	11486603	2	36	T:0.666667	C:0.333333	
chr28	11486821	2	36	C:0.888889	T:0.111111	
chr28	11486830	2	36	A:0.611111	G:0.388889	

chr28	11486883	2	38	A:1	G:0	
chr28	11487163	2	38	G:1	T:0	
chr28	11487466	2	40	T:0.95	C:0.05	
chr28	11487880	2	34	T:0.705882		C:0.294118
chr28	11488166	2	36	C:0.972222		T:0.0277778
chr28	11488286	2	38	TGC:0.868421		T:0.131579
chr28	11488289	2	40	G:0.825	GCTCT:0.175	
chr28	11488291	2	40	G:0.7	T:0.3	
chr28	11488341	2	40	A:0.575	G:0.425	
chr28	11488740	2	40	G:0.925	A:0.075	
chr28	11488817	2	40	G:0.975	A:0.025	
chr28	11488832	2	38	C:0.552632		G:0.447368
chr28	11489578	2	34	G:0.941176		A:0.0588235
chr28	11489756	2	34	T:0.588235		TA:0.411765
chr28	11489966	2	36	G:1	A:0	
chr28	11490486	2	38	G:1	A:0	
chr28	11490715	2	40	A:0.7	G:0.3	
chr28	11490799	2	38	T:1	A:0	
chr28	11490936	2	40	A:0.875	G:0.125	
chr28	11490937	2	40	T:0.875	C:0.125	
chr28	11490945	2	40	G:0.875	T:0.125	
chr28	11491365	2	38	C:0.868421		G:0.131579
chr28	11491994	2	40	A:0	AACT:1	
chr28	11492273	2	40	A:0.925	G:0.075	
chr28	11492868	2	32	AG:0	A:1	
chr28	11493620	2	38	C:1	A:0	
chr28	11493665	2	34	CG:0.941176		C:0.0588235
chr28	11493738	2	30	G:0.9	A:0.1	
chr28	11494754	2	34	G:0.764706		A:0.235294
chr28	11494775	2	34	AG:0.911765		A:0.0882353
chr28	11495053	2	40	T:0.925	C:0.075	
chr28	11495155	2	40	C:0.925	T:0.075	
chr28	11495639	2	40	G:0.875	A:0.125	
chr28	11495958	2	40	G:0.925	A:0.075	
chr28	11496440	2	40	C:0.85	T:0.15	
chr28	11496629	2	40	G:0.925	A:0.075	
chr28	11496751	2	36	G:0.861111		GT:0.138889
chr28	11496843	2	38	G:0.868421		A:0.131579
chr28	11496867	2	38	C:0.868421		T:0.131579
chr28	11497063	2	40	T:0.85	C:0.15	
chr28	11497560	2	40	C:0.875	T:0.125	
chr28	11497797	2	40	T:0.9	A:0.1	
chr28	11497809	2	40	TA:0.9	T:0.1	
chr28	11498015	2	38	A:0.894737		AT:0.105263
chr28	11499679	2	38	A:0.894737		G:0.105263
chr28	11501918	2	38	T:0.868421		C:0.131579
chr28	11502579	2	40	A:0.9	G:0.1	
chr28	11502629	2	40	T:0.875	C:0.125	
chr28	11502635	2	40	A:0.875	G:0.125	
chr28	11502653	2	40	C:0.875	T:0.125	
chr28	11502734	2	36	A:0.833333		C:0.166667
chr28	11503113	2	38	A:0.868421		G:0.131579
chr28	11503177	2	40	T:0.925	C:0.075	
chr28	11503288	2	40	A:0.8	G:0.2	

chr28	11503334	2	40	G:0.825	A:0.175	
chr28	11503634	2	38	T:0.842105	C:0.157895	
chr28	11504069	2	38	G:0.868421	A:0.131579	
chr28	11504071	2	38	T:0.868421	C:0.131579	
chr28	11504334	2	22	ACC:0.772727	A:0.227273	
chr28	11504721	2	40	CCATTTTT:0.9	C:0.1	
chr28	11505153	2	38	G:0.815789	T:0.184211	
chr28	11505207	2	40	G:0.85	A:0.15	
chr28	11505367	2	40	A:0.875	G:0.125	
chr28	11505524	2	36	C:0.861111	T:0.138889	
chr28	11505676	2	38	C:1	T:0	
chr28	11505773	2	36	G:0.972222	GA:0.0277778	
chr28	11505867	2	40	G:0.925	A:0.075	
chr28	11506135	3	40	CCT:0.85	C:0.15	CCTCTCT:0
chr28	11506272	2	38	T:0.894737	C:0.105263	
chr28	11506414	2	38	A:0.868421	G:0.131579	
chr28	11506817	2	38	AC:0.973684	A:0.0263158	
chr28	11507238	3	40	TTG:0.675	T:0.3	TTGTG: 0.025
chr28	11507382	2	40	A:0.825	G:0.175	
chr28	11507492	2	38	A:0.973684	T:0.0263158	
chr28	11507572	2	38	C:0.973684	A:0.0263158	
chr28	11507680	2	38	G:0.973684	A:0.0263158	
chr28	11507821	2	40	T:0.875	C:0.125	
chr28	11507946	2	38	G:0.868421	T:0.131579	
chr28	11508055	2	36	A:0.972222	G:0.0277778	
chr28	11508165	2	36	C:1	T:0	
chr28	11509514	2	34	CT:0.735294	C:0.264706	
chr28	11509614	2	38	G:0.973684	T:0.0263158	
chr28	11510272	2	38	T:0.973684	G:0.0263158	
chr28	11511212	2	40	C:0.975	CGCCTTGATTATGATACTACACTTGATCTTAGCCAAAGGCCGAGAAGCGAT:0.025	
chr28	11511222	2	40	A:1	G:0	
chr28	11511233	2	40	A:1	G:0	
chr28	11511316	2	40	C:0.975	T:0.025	
chr28	11511841	2	38	T:0.894737	C:0.105263	
chr28	11511913	2	40	C:1	T:0	
chr28	11512011	2	38	TTG:0	T:1	
chr28	11512407	2	38	A:1	T:0	
chr28	11513140	2	36	CT:0.638889	C:0.361111	
chr28	11513530	2	40	A:0.95	G:0.05	
chr28	11513642	2	40	C:0.9	T:0.1	
chr28	11514371	2	38	T:0.947368	C:0.0526316	
chr28	11514674	2	40	C:0	CA:1	
chr28	11514693	2	40	C:0	T:1	
chr28	11514765	2	40	A:1	T:0	
chr28	11514797	2	40	G:0.875	GATT:0.125	
chr28	11514978	2	36	C:0.777778	G:0.222222	
chr28	11515167	2	40	GCTGT:0.85	G:0.15	
chr28	11515383	2	40	T:0.85	C:0.15	
chr28	11515793	2	38	C:0.842105	G:0.157895	
chr28	11516163	2	40	CAAGAT:0.875	C:0.125	
chr28	11516390	2	40	C:0.9	CCCA:0.1	
chr28	11516429	2	38	C:0.894737	T:0.105263	

chr28	11516463	2	40	C:1	T:0		
chr28	11516757	2	36	G:0.833333		A:0.166667	
chr28	11516761	2	36	A:0.666667		G:0.333333	
chr28	11516798	2	40	C:0.85	T:0.15		
chr28	11517070	2	36	G:0.833333		C:0.166667	
chr28	11517763	2	40	A:0.875	T:0.125		
chr28	11517929	2	34	C:0.823529		T:0.176471	
chr28	11518074	2	36	C:1	T:0		
chr28	11518205	2	38	G:0.842105		A:0.157895	
chr28	11518482	2	38	G:0.552632		A:0.447368	
chr28	11518915	2	34	C:0.911765		T:0.0882353	
chr28	11519820	2	40	G:0.8	A:0.2		
chr28	11520070	2	40	A:0.6	C:0.4		
chr28	11520739	2	40	A:0.725	ATTTAT:0.275		
chr28	11521589	2	40	TCGC:0.65		T:0.35	
chr28	11521714	2	40	C:0.65	A:0.35		
chr28	11522169	2	40	C:0.675	T:0.325		
chr28	11522337	2	34	CT:0.941176		C:0.0588235	
chr28	11522354	4	40	T:0.675	C:0.15	TC:0.075	TTC:0.1
chr28	11522935	2	38	TA:0.605263		T:0.394737	
chr28	11523560	2	16	G:0.3125	GC:0.6875		
chr28	11524592	2	40	C:0.55	T:0.45		
chr28	11525728	2	38	A:0.5	G:0.5		
chr28	11526058	2	34	G:1	A:0		
chr28	11527611	2	38	T:0.710526		C:0.289474	
chr28	11528478	2	40	G:0.9	A:0.1		
chr28	11530233	2	36	C:0	CG:1		
chr28	11530244	2	38	A:0	C:1		
chr28	11530269	2	38	C:0	CA:1		
chr28	11530274	2	38	AT:0	A:1		
chr28	11530369	2	36	G:0.5	A:0.5		
chr28	11530818	2	40	C:0.925	G:0.075		
chr28	11530874	2	40	C:0.95	T:0.05		
chr28	11531543	2	38	G:0.736842		T:0.263158	
chr28	11532105	2	40	T:1	TGAGA:0		
chr28	11532111	2	40	A:0.9	G:0.1		
chr28	11532192	2	40	G:1	A:0		
chr28	11532549	2	40	G:0.925	A:0.075		
chr28	11532847	2	40	T:0.9	TACCAC:0.1		
chr28	11533505	2	40	T:0.65	A:0.35		
chr28	11533827	2	40	A:0.9	G:0.1		
chr28	11533856	2	38	T:1	A:0		
chr28	11533911	2	38	T:0.526316		C:0.473684	
chr28	11533989	2	36	C:0.611111		CAG:0.388889	
chr28	11534005	2	36	C:0.888889		T:0.111111	
chr28	11534124	2	40	T:0.925	C:0.075		
chr28	11534183	2	38	C:0.894737		CT:0.105263	
chr28	11534650	2	38	G:0.973684		A:0.0263158	
chr28	11534799	2	40	C:1	T:0		
chr28	11535501	2	38	C:0.815789		T:0.184211	
chr28	11535645	2	34	G:0.970588		GA:0.0294118	
chr28	11535897	2	40	C:0.975	T:0.025		
chr28	11535924	2	40	C:0.95	T:0.05		
chr28	11535926	2	40	C:0.95	G:0.05		

chr28	11536344	2	38	C:1	A:0	
chr28	11536414	2	40	G:1	A:0	
chr28	11536599	2	38	CT:0.526316	C:0.473684	
chr28	11536711	2	38	G:1	GA:0	
chr28	11537054	3	38	GTCTC:0.684211	G:0.263158	GTCTCTCTCTCTC:0.0526316
chr28	11537056	2	38	C:0.973684	CTCTCTCTG: 0.0263158	
chr28	11537152	2	40	T:1	C:0	
chr28	11537281	2	34	G:0.970588	A:0.0294118	
chr28	11537416	2	40	G:0.55	A:0.45	
chr28	11537696	2	36	C:0.944444	T:0.0555556	
chr28	11538009	2	40	A:0.425	C:0.575	
chr28	11538294	2	40	T:1	TC:0	
chr28	11538675	2	34	T:0.941176	C:0.0588235	
chr28	11538753	2	40	A:0.4	G:0.6	
chr28	11539134	2	38	GC:0.605263	G:0.394737	
chr28	11539147	2	40	T:0.625	C:0.375	
chr28	11539150	2	40	T:0.05	C:0.95	
chr28	11539227	2	40	G:0.5	T:0.5	
chr28	11539262	2	40	C:1	T:0	
chr28	11540390	2	38	C:1	T:0	
chr28	11540567	2	40	C:0.95	T:0.05	
chr28	11540668	2	40	C:0.95	T:0.05	
chr28	11540718	2	40	G:0.675	C:0.325	
chr28	11540928	2	38	G:0.684211	A:0.315789	
chr28	11541108	2	38	A:0.105263	C:0.894737	
chr28	11541429	2	38	G:0.342105	A:0.657895	
chr28	11541457	2	38	G:1	T:0	
chr28	11541535	2	38	G:0.447368	A:0.552632	
chr28	11541554	2	38	C:1	A:0	
chr28	11542693	2	40	C:0.4	T:0.6	
chr28	11543092	2	38	G:0.0789474	GCC:0.921053	
chr28	11543557	2	36	A:0.694444	C:0.305556	
chr28	11543704	2	40	A:0.225	G:0.775	
chr28	11543708	2	40	A:0.925	C:0.075	
chr28	11543713	2	40	A:0.225	C:0.775	
chr28	11544014	2	40	G:1	A:0	
chr28	11544060	2	40	A:0.5	G:0.5	
chr28	11544073	2	40	C:0.5	T:0.5	
chr28	11544252	2	34	C:0.970588	T:0.0294118	
chr28	11544280	2	38	A:0.789474	G:0.210526	
chr28	11544391	2	38	C:0.421053	T:0.578947	
chr28	11544484	2	40	C:0.25	T:0.75	
chr28	11544499	2	40	G:0.525	GCT:0.475	
chr28	11544581	2	40	G:0.45	A:0.55	
chr28	11544714	2	36	T:0.0833333	TG:0.916667	
chr28	11544717	2	38	A:0.973684	G:0.0263158	
chr28	11544799	2	36	G:0.194444	T:0.805556	
chr28	11544827	2	40	T:0.5	C:0.5	
chr28	11544877	2	38	GTGT:0.973684	G:0.0263158	
chr28	11544883	2	38	GT:0.447368	G:0.552632	
chr28	11544890	2	38	G:0.973684	GC:0.0263158	
chr28	11544914	2	38	T:0.236842	C:0.763158	

chr28	11544988	2	38	C:0.184211	T:0.815789
chr28	11545100	2	34	G:0.970588	GC:0.0294118
chr28	11545302	2	40	C:0.95	T:0.05
chr28	11545375	2	36	A:0.972222	G:0.0277778
chr28	11545391	2	36	A:0.388889	C:0.611111
chr28	11545565	2	38	G:0.184211	T:0.815789
chr28	11545679	2	36	T:1	C:0
chr28	11545691	2	38	T:0.421053	TG:0.578947
chr28	11545745	2	38	C:0.947368	CCTGCTT:0.0526316
chr28	11545856	2	36	G:0.111111	C:0.888889
chr28	11545895	2	38	A:0.236842	G:0.763158
chr28	11545936	2	40	G:0.225	T:0.775
chr28	11545944	2	40	T:0.225	C:0.775
chr28	11546339	2	32	C:0.65625	G:0.34375
chr28	11546711	2	32	T:0	C:1
chr28	11547246	2	38	C:0.710526	T:0.289474
chr28	11547749	2	38	T:0.710526	C:0.289474
chr28	11547825	2	36	G:0.805556	A:0.194444
chr28	11547909	2	36	A:0.75	G:0.25
chr28	11547965	2	38	G:0.763158	A:0.236842
chr28	11548201	2	38	GGC:0.657895	G:0.342105
chr28	11548684	2	40	A:0.7	C:0.3
chr28	11548728	2	38	G:0.657895	A:0.342105
chr28	11548790	2	38	T:0.421053	C:0.578947
chr28	11548905	2	38	T:0.684211	C:0.315789
chr28	11549158	2	38	C:0.763158	T:0.236842
chr28	11549225	2	38	G:0.578947	C:0.421053
chr28	11549422	2	38	G:1	A:0
chr28	11549854	2	40	GC:0.675	G:0.325
chr28	11549886	2	38	A:0.157895	C:0.842105
chr28	11550451	2	40	G:0.675	T:0.325
chr28	11550453	2	40	C:0.675	T:0.325
chr28	11550474	2	40	C:0.725	T:0.275
chr28	11550527	2	38	T:0.210526	C:0.789474
chr28	11550532	2	38	A:0.736842	G:0.263158
chr28	11550589	2	38	G:0.710526	GA:0.289474
chr28	11551403	2	36	T:1	C:0
chr28	11551428	2	36	G:0.444444	A:0.555556
chr28	11551678	2	40	C:0.725	A:0.275
chr28	11551766	2	38	A:0.526316	G:0.473684
chr28	11552315	2	36	C:0.583333	T:0.416667
chr28	11552628	2	40	G:0.45	C:0.55
chr28	11552797	2	40	A:0.1	G:0.9
chr28	11552898	2	36	G:0.444444	A:0.555556
chr28	11552956	2	34	C:0.941176	T:0.0588235
chr28	11553028	2	36	C:0.5	T:0.5
chr28	11553058	2	38	C:1	A:0
chr28	11553744	2	20	G:0.65	A:0.35
chr28	11553875	2	22	A:0.0909091	C:0.909091
chr28	11554042	2	38	G:0.657895	A:0.342105
chr28	11554074	2	36	C:0.555556	T:0.444444
chr28	11554165	2	36	G:1	T:0
chr28	11554424	2	38	T:0.578947	C:0.421053
chr28	11554540	2	40	G:0.475	A:0.525

chr28	11554663	3	38	AC:0.236842	A:0.394737	ACC:0.368421
chr28	11555304	2	40	A:1	C:0	
chr28	11555340	2	36	C:0.472222	A:0.527778	
chr28	11555434	2	40	A:0.1	G:0.9	
chr28	11555462	2	38	A:0.605263	AC:0.394737	
chr28	11555463	2	38	G:0.131579	C:0.868421	
chr28	11555641	2	40	C:0.475	T:0.525	
chr28	11556141	2	40	G:0.525	A:0.475	
chr28	11556147	2	40	C:0.975	T:0.025	
chr28	11556157	2	38	C:0.947368	T:0.0526316	
chr28	11556412	2	40	C:0.725	G:0.275	
chr28	11556493	2	40	A:0.675	G:0.325	
chr28	11556601	2	40	G:0.625	A:0.375	
chr28	11556642	2	40	A:0.625	G:0.375	
chr28	11556693	3	40	TAC:0.3	T:0.475	TACACAC:0.225
chr28	11556796	2	40	A:0.675	G:0.325	
chr28	11557075	2	30	A:0.0333333	G:0.966667	
chr28	11557107	2	36	C:0.666667	T:0.333333	
chr28	11557257	2	38	TGG:0.631579	T:0.368421	
chr28	11557409	2	40	TCC:0.675	T:0.325	
chr28	11557492	2	40	C:0.475	T:0.525	
chr28	11558629	2	40	G:0.525	A:0.475	
chr28	11558708	3	38	TAA:0.210526	T:0.473684	TA:0.315789
chr28	11558881	2	40	T:0.5	C:0.5	
chr28	11558998	2	40	C:1	A:0	
chr28	11559044	2	38	T:0.973684	C:0.0263158	
chr28	11559049	2	36	A:1	G:0	
chr28	11559113	2	36	C:0.972222	T:0.0277778	
chr28	11559336	2	38	G:0.973684	A:0.0263158	
chr28	11559370	2	38	G:0.526316	A:0.473684	
chr28	11559660	2	36	G:0.583333	A:0.416667	
chr28	11559680	2	36	C:1	A:0	
chr28	11559789	2	40	G:0.975	A:0.025	
chr28	11560380	2	34	G:1	GAA:0	
chr28	11560678	2	40	T:0.975	C:0.025	
chr28	11560738	2	38	C:0.947368	T:0.0526316	
chr28	11560775	2	38	G:0.973684	A:0.0263158	
chr28	11560960	2	38	ACAGGGTGACTTG:0.684211	A:0.315789	
chr28	11561034	2	38	T:0.657895	C:0.342105	
chr28	11561179	2	36	A:1	G:0	
chr28	11561313	2	40	A:0.975	ATTTGTGTTGTGTTGTGTTGTATGTCTTTATATGG:0.025	
chr28	11561358	2	34	T:0.617647	C:0.382353	
chr28	11561433	2	40	G:1	A:0	
chr28	11561807	2	38	C:1	T:0	
chr28	11562183	2	40	C:1	T:0	
chr28	11562308	2	38	A:0.131579	T:0.868421	
chr28	11562402	2	40	G:0.95	A:0.05	
chr28	11562430	2	40	C:1	T:0	
chr28	11562453	2	40	G:0.975	A:0.025	
chr28	11562652	2	40	C:1	T:0	

chr28	11562724	2	40	T:0.175	C:0.825
chr28	11562746	2	40	A:0.175	G:0.825
chr28	11562877	2	40	A:0.975	C:0.025
chr28	11562957	2	38	G:0.0526316	A:0.947368
chr28	11563024	2	36	G:0.166667	T:0.833333
chr28	11563301	2	34	T:0.117647	C:0.882353
chr28	11563332	2	38	G:0.157895	A:0.842105
chr28	11563436	2	40	T:0.1	C:0.9
chr28	11563631	2	40	C:0.15	T:0.85
chr28	11564298	2	40	C:0.125	T:0.875
chr28	11564438	2	40	G:0.125	GTC:0.875
chr28	11564743	2	36	T:0.138889	C:0.861111
chr28	11564765	2	36	C:0.166667	A:0.833333
chr28	11564817	2	32	T:0.03125	C:0.96875
chr28	11564942	2	32	CTCTCT:0.34375	C:0.65625
chr28	11564975	2	34	TA:0.205882	T:0.794118
chr28	11564976	2	30	A:0.966667	T:0.0333333
chr28	11564977	2	34	A:1	T:0
chr28	11564997	2	34	T:0.205882	C:0.794118
chr28	11565006	2	32	G:0.96875	GA:0.03125
chr28	11565038	2	38	G:0.105263	A:0.894737
chr28	11565040	2	38	TGTTA:0.973684	T:0.0263158
chr28	11565107	2	40	A:0.175	C:0.825
chr28	11565280	2	40	C:0.125	A:0.875
chr28	11565509	2	38	G:1	A:0
chr28	11565518	2	38	C:0.684211	T:0.315789
chr28	11565525	2	38	C:1	T:0
chr28	11565938	2	40	A:0.15	T:0.85
chr28	11566480	2	36	C:0.138889	A:0.861111
chr28	11566746	2	40	A:1	G:0
chr28	11567133	2	40	A:0.1	G:0.9
chr28	11567398	2	40	C:0.125	G:0.875
chr28	11567410	2	40	T:0.125	A:0.875
chr28	11567464	2	38	G:0.131579	C:0.868421
chr28	11567663	2	40	A:1	G:0
chr28	11567932	2	40	A:0.125	G:0.875
chr28	11568038	2	40	T:0.125	C:0.875
chr28	11568052	2	40	T:0.15	C:0.85
chr28	11568086	2	38	T:0.105263	C:0.894737
chr28	11568578	2	36	T:0.0833333	C:0.916667
chr28	11568998	2	38	C:0.947368	A:0.0526316
chr28	11569485	2	32	A:1	AC:0
chr28	11569547	2	36	G:0.111111	A:0.888889
chr28	11569937	2	36	G:1	A:0
chr28	11570207	2	40	G:0.125	GA:0.875
chr28	11570420	2	40	T:0.15	C:0.85
chr28	11570482	2	38	TC:0.973684	T:0.0263158
chr28	11571261	2	40	A:0.4	T:0.6
chr28	11571394	2	38	G:0.736842	C:0.263158
chr28	11571395	2	38	G:0.736842	T:0.263158
chr28	11571515	2	36	C:0	CT:1
chr28	11571579	2	38	C:0	A:1
chr28	11571582	2	38	T:0	A:1
chr28	11571583	2	38	G:0	A:1

chr28	11571586	2	38	G:0	A:1		
chr28	11571687	2	36	C:0.972222	T:0.0277778		
chr28	11571773	2	30	CCA:0.8	C:0.2		
chr28	11571812	2	30	T:0.866667	G:0.133333		
chr28	11572221	2	12	A:0.833333	G:0.166667		
chr28	11572368	2	28	G:0.964286	T:0.0357143		
chr28	11572559	2	34	T:0.735294	C:0.264706		
chr28	11572602	2	32	A:0.875	T:0.125		
chr28	11572697	2	30	G:0.833333	A:0.166667		
chr28	11572722	2	34	G:0.970588	A:0.0294118		
chr28	11572829	2	24	A:0.375	T:0.625		
chr28	11573250	2	36	T:0.75	C:0.25		
chr28	11573328	2	34	G:1	A:0		
chr28	11573350	2	40	A:0	T:1		
chr28	11573351	2	40	A:0	T:1		
chr28	11573352	2	40	A:0	T:1		
chr28	11573392	2	40	A:0	AT:1		
chr28	11573397	2	40	A:0	AT:1		
chr28	11573680	2	38	T:0.763158	C:0.236842		
chr28	11573861	2	38	A:0.815789	G:0.184211		
chr28	11573910	2	38	A:0.710526	G:0.289474		
chr28	11573959	2	40	A:1	C:0		
chr28	11574101	2	40	TTTTCTTTCTTTC:0.675	T:0.325		
chr28	11574186	4	40	CTTCCTTTCCTTTCCTTTCCTTTCCTTTCCT:0.175	C:0.475	CTTCCTTTCCT:0.175	CTTCCTTTCCTTTCCTTTCCT:0.175
chr28	11574601	2	40	C:0.2	CAAAT:0.8		
chr28	11574693	2	38	T:0.394737	G:0.605263		
chr28	11574809	2	34	C:0.323529	CT:0.676471		
chr28	11574818	2	36	A:0.527778	T:0.472222		
chr28	11575729	2	38	AG:0.868421	A:0.131579		
chr28	11576021	2	40	G:1	A:0		
chr28	11576230	2	40	A:0.225	T:0.775		
chr28	11576419	2	40	T:0.2	TG:0.8		
chr28	11576643	2	40	CAAACAA:0.8	C:0.2		
chr28	11576716	2	40	GTA:0.175	G:0.825		
chr28	11576738	2	40	T:0.95	A:0.05		
chr28	11576853	2	40	G:0.175	C:0.825		
chr28	11576869	2	40	C:0.95	T:0.05		
chr28	11577188	2	30	CCT:0.3	C:0.7		
chr28	11577216	2	32	A:0.65625	AT:0.34375		
chr28	11577346	2	38	G:0.157895	A:0.842105		
chr28	11577432	2	40	G:0.95	A:0.05		
chr28	11577541	2	40	C:0.925	T:0.075		
chr28	11577594	2	40	A:1	G:0		
chr28	11577789	2	40	C:0.25	A:0.75		
chr28	11577927	2	38	T:0.157895	TAA:0.842105		
chr28	11577973	2	38	T:0.184211	C:0.815789		
chr28	11578112	2	36	C:0.111111	T:0.888889		
chr28	11578215	2	38	A:0.184211	C:0.815789		
chr28	11578269	2	12	C:0.5	A:0.5		
chr28	11578484	2	30	G:0.933333	A:0.0666667		
chr28	11578533	2	36	C:0.833333	T:0.166667		
chr28	11578564	2	38	C:0.157895	A:0.842105		

chr28	11578718	2	38	C:0.289474	T:0.710526	
chr28	11578778	2	40	TAC:0.425	T:0.575	
chr28	11578794	2	40	G:0.2	A:0.8	
chr28	11578798	2	40	A:0.75	ATG:0.25	
chr28	11578865	2	40	A:0.45	T:0.55	
chr28	11578932	2	40	A:0.225	G:0.775	
chr28	11578956	2	38	C:0.736842	A:0.263158	
chr28	11579440	2	38	G:0.157895	A:0.842105	
chr28	11579558	2	40	T:0.15	C:0.85	
chr28	11580025	2	38	G:1	A:0	
chr28	11580346	2	40	C:0.15	T:0.85	
chr28	11580885	2	40	G:0.175	T:0.825	
chr28	11581125	2	36	G:0.722222	A:0.277778	
chr28	11581162	2	36	G:1	A:0	
chr28	11581232	2	34	G:0.735294	A:0.264706	
chr28	11581388	2	40	T:0.2	G:0.8	
chr28	11581861	2	34	G:0.176471	A:0.823529	
chr28	11581981	2	40	T:0.775	C:0.225	
chr28	11582049	2	40	T:0.725	A:0.275	
chr28	11582340	2	38	G:0.736842	A:0.263158	
chr28	11582811	2	32	C:0.8125	T:0.1875	
chr28	11582964	2	40	A:1	C:0	
chr28	11583458	2	40	AC:0.65	A:0.35	
chr28	11583596	2	36	C:0.416667	T:0.583333	
chr28	11583717	2	38	C:0.210526	A:0.789474	
chr28	11583875	2	38	G:0.815789	A:0.184211	
chr28	11583892	2	36	G:0.166667	A:0.833333	
chr28	11584035	2	38	T:1	C:0	
chr28	11584058	2	36	T:1	G:0	
chr28	11584794	2	36	CT:0.833333	C:0.166667	
chr28	11585232	2	40	A:0.8	G:0.2	
chr28	11585430	2	36	A:0.472222	G:0.527778	
chr28	11585454	2	38	G:1	C:0	
chr28	11585532	2	38	A:1	C:0	
chr28	11585712	2	34	C:0.323529	T:0.676471	
chr28	11585736	2	36	G:1	A:0	
chr28	11587009	2	38	C:0.868421	T:0.131579	
chr28	11587232	2	34	TGA:0.147059	T:0.852941	
chr28	11587312	2	36	G:0.361111	A:0.638889	
chr28	11587332	2	36	G:1	A:0	
chr28	11587641	2	38	G:1	A:0	
chr28	11587651	2	38	A:0.473684	G:0.526316	
chr28	11588148	2	38	A:0.368421	G:0.631579	
chr28	11588471	3	38	CT:0.657895	C:0.131579	CTT:0.210526
chr28	11588601	2	36	T:0.166667	C:0.833333	
chr28	11588763	2	40	C:1	T:0	
chr28	11589217	2	38	C:0.473684	T:0.526316	
chr28	11589258	2	40	T:0.75	C:0.25	
chr28	11589596	2	36	G:1	A:0	
chr28	11589598	2	36	C:0.416667	T:0.583333	
chr28	11590069	2	32	T:0.9375	C:0.0625	
chr28	11590104	2	32	C:1	T:0	
chr28	11590299	2	38	C:0.473684	A:0.526316	

chr28	11590481	2	38	A:0.947368	G:0.0526316		
chr28	11590817	2	36	G:0.166667	A:0.833333		
chr28	11590925	2	38	T:0.236842	C:0.763158		
chr28	11591042	2	36	T:0.944444	C:0.0555556		
chr28	11591043	2	36	G:0.944444	T:0.0555556		
chr28	11591057	2	34	A:0.941176	T:0.0588235		
chr28	11591069	2	36	T:0.916667	C:0.0833333		
chr28	11591173	2	40	G:1	A:0		
chr28	11591421	2	38	T:1	TG:0		
chr28	11591717	2	38	C:1	T:0		
chr28	11591970	2	38	G:0.473684	T:0.526316		
chr28	11592040	2	38	T:0.5	G:0.5		
chr28	11592531	2	38	A:1	G:0		
chr28	11592748	2	38	T:1	G:0		
chr28	11592995	2	38	G:1	C:0		
chr28	11593095	2	20	A:0.6	G:0.4		
chr28	11593209	2	8	G:0.875	A:0.125		
chr28	11593286	2	26	GTGCGGC:0.307692	G:0.692308		
chr28	11593315	2	30	CGGGCCA:1	C:0		
chr28	11593329	2	32	GGCCAGGGCCAGC:0.53125	G: 0.46875		
chr28	11593513	2	32	C:0.15625	CG:0.84375		
chr28	11593558	2	32	C:0.4375	T:0.5625		
chr28	11594256	2	40	C:1	CCCCT:0		
chr28	11594264	2	40	T:0.2	TC:0.8		
chr28	11594265	3	40	T:0.2	TC:0.15	TCTC:0.65	
chr28	11594286	2	38	AT:0.921053	A:0.0789474		
chr28	11594291	2	38	T:0.868421	A:0.131579		
chr28	11594296	2	38	T:0.921053	A:0.0789474		
chr28	11594297	4	38	TA:0.315789	T:0.552632	AA:0	TAA:0.131579
chr28	11594331	2	38	C:1	T:0		
chr28	11594374	2	38	G:0.921053	A:0.0789474		
chr28	11594483	2	40	T:0.4	G:0.6		
chr28	11594670	2	40	G:0.55	T:0.45		
chr28	11594671	2	40	T:0.55	C:0.45		
chr28	11594796	2	40	A:0.675	G:0.325		
chr28	11594872	2	40	C:0.925	T:0.075		
chr28	11594893	2	38	C:0.736842	CAGAA:0.263158		
chr28	11595014	2	40	A:0.675	G:0.325		
chr28	11595259	2	40	A:0.725	T:0.275		
chr28	11595360	2	38	C:0.789474	T:0.210526		
chr28	11595626	2	34	C:0.676471	T:0.323529		
chr28	11595862	2	36	G:0.916667	T:0.0833333		
chr28	11595870	2	36	C:0.777778	T:0.222222		
chr28	11595883	2	38	T:0.789474	C:0.210526		
chr28	11596094	2	36	T:0.777778	C:0.222222		
chr28	11596296	2	38	G:1	A:0		
chr28	11596429	2	38	T:0.184211	C:0.815789		
chr28	11596526	2	38	A:0.789474	G:0.210526		
chr28	11596568	2	36	T:0.75	A:0.25		
chr28	11596570	2	36	C:1	T:0		
chr28	11596721	2	36	G:0.388889	C:0.611111		
chr28	11596765	3	38	GTT:0.394737	G:0.473684		

GT:0.131579
chr28 11596776 2 38 TTTTTTG:0.526316 T:0.473684
chr28 11596779 2 38 TTTG:1 T:0
chr28 11596782 2 38 G:0.763158 T:0.236842
chr28 11596785 2 38 T:0.763158 G:0.236842
chr28 11596787 2 38 T:0.868421 G:0.131579
chr28 11596788 2 38 TG:0.868421 T:0.131579
chr28 11596789 3 38 GTT:0.315789 G:0.473684
TTT:0.210526
chr28 11596794 2 38 T:1 TG:0
chr28 11596927 2 38 T:0.921053 A:0.0789474
chr28 11597021 3 36 C:0.277778 CT:0.666667
CTT:0.0555556
chr28 11597352 2 38 C:0.921053 T:0.0789474
chr28 11597367 2 36 G:0.416667 T:0.583333
chr28 11597372 2 38 G:0.921053 A:0.0789474
chr28 11597823 2 36 C:0.75 T:0.25
chr28 11598185 4 40 CT:0.6 C:0.125 CTT:0.2 CTTT:
0.075
chr28 11598207 2 40 A:0.925 T:0.075
chr28 11598277 2 38 G:0.447368 A:0.552632
chr28 11598278 2 38 A:0.447368 T:0.552632
chr28 11598332 2 40 G:0.425 GGAT:0.575
chr28 11598335 2 40 C:0.425 T:0.575
chr28 11598396 2 38 C:0.578947 CT:0.421053
chr28 11598404 2 38 C:0.157895 T:0.842105
chr28 11598484 2 40 G:0.55 T:0.45
chr28 11598559 2 38 A:1 G:0
chr28 11598737 2 40 C:0.95 CA:0.05
chr28 11598800 4 38 CTT:0.210526 C:0.526316
CT:0.184211 CTTT:0.0789474
chr28 11598860 2 38 C:0.184211 T:0.815789
chr28 11599091 2 40 T:0.9 G:0.1
chr28 11599188 2 36 C:1 A:0
chr28 11599223 2 36 A:0.916667 C:0.0833333
chr28 11599278 2 40 A:0.975 C:0.025
chr28 11599456 2 38 T:0.157895 C:0.842105
chr28 11599457 2 38 G:0.5 A:0.5
chr28 11599776 2 40 T:1 G:0
chr28 11599802 2 38 T:0.763158 A:0.236842
chr28 11599821 2 38 T:1 A:0
chr28 11599924 2 38 G:0.815789 A:0.184211
chr28 11600197 2 40 GA:0.775 G:0.225
chr28 11600932 2 40 T:0.75 A:0.25
chr28 11601773 2 40 A:1 C:0
chr28 11601810 2 40 G:0.425 A:0.575
chr28 11601818 2 40 C:1 T:0
chr28 11601898 2 34 C:0.970588 G:0.0294118
chr28 11602097 2 40 C:0.3 A:0.7
chr28 11602103 2 38 T:0.842105 G:0.157895
chr28 11602416 2 40 T:0.925 C:0.075
chr28 11602515 2 40 T:0.925 C:0.075
chr28 11602545 2 40 A:0.2 C:0.8
chr28 11602891 2 40 C:0.9 T:0.1

chr28 11603063 2 36 T:1 G:0
chr28 11604252 2 38 T:0.394737 C:0.605263
chr28 11604357 2 38 A:0.921053 T:0.0789474
chr28 11604378 4 38 TGAGAGAGAGA:0.210526 T:
0.394737 TGAGA:0.236842 TGAGAGAGA:0.157895
chr28 11604394 2 38 AG:0.921053 A:0.0789474
chr28 11604396 2 38 AGAGAGAG:0.921053 A:
0.0789474
chr28 11604458 2 34 C:0.352941 G:0.647059
chr28 11604478 2 36 TG:0.5 T:0.5
chr28 11604541 2 36 A:0.777778 T:0.222222
chr28 11604741 2 38 C:0.921053 A:0.0789474
chr28 11604757 2 40 ATGAAGAAATGGAGACTGTTT:0.575
A:0.425
chr28 11604949 2 36 A:0.388889 G:0.611111
chr28 11604955 2 36 A:1 G:0
chr28 11605172 2 38 T:0.921053 G:0.0789474
chr28 11605576 2 40 C:0.925 T:0.075
chr28 11605909 2 34 C:1 T:0
chr28 11606073 2 34 A:0.823529 G:0.176471
chr28 11606519 2 34 A:0.941176 G:0.0588235
chr28 11606879 2 38 CA:1 C:0
chr28 11606945 2 38 T:0.815789 A:0.184211
chr28 11607011 2 38 A:1 G:0
chr28 11607897 2 40 C:0.6 T:0.4
chr28 11608033 2 36 G:0.75 A:0.25
chr28 11608180 3 40 TAAACAAAC:0.575 T:0.1 TAAAC:
0.325
chr28 11608898 2 40 A:0.625 G:0.375
chr28 11608945 2 40 G:0.675 C:0.325
chr28 11609061 2 36 T:0.694444 C:0.305556
chr28 11609073 2 36 A:0.583333 T:0.416667
chr28 11609146 2 40 T:0.675 C:0.325
chr28 11609265 2 36 T:0.694444 C:0.305556
chr28 11609484 2 32 C:1 T:0
chr28 11609564 2 30 G:0.9 A:0.1
chr28 11609578 2 30 C:1 T:0
chr28 11609722 2 36 T:0.805556 G:0.194444
chr28 11609723 2 36 A:0.694444 C:0.305556
chr28 11609803 2 34 A:0.970588 T:0.0294118
chr28 11609990 2 36 C:0.666667 A:0.333333
chr28 11610409 2 38 G:0.789474 A:0.210526
chr28 11610793 2 36 TCAG:0.916667 T:0.0833333
chr28 11611072 2 40 T:0.75 C:0.25
chr28 11611102 2 36 T:0.75 C:0.25
chr28 11611344 2 38 T:0.973684 C:0.0263158
chr28 11611618 2 38 C:0.684211 G:0.315789
chr28 11611687 2 40 C:0.7 T:0.3
chr28 11611791 2 40 G:1 A:0
chr28 11611845 2 40 A:0.675 T:0.325
chr28 11611994 2 40 G:1 A:0
chr28 11612122 2 38 T:0.631579 C:0.368421
chr28 11612128 2 38 A:0.763158 C:0.236842
chr28 11612184 2 38 G:0.973684 A:0.0263158

chr28	11612734	2	38	C:0.763158	T:0.236842
chr28	11612749	2	40	G:0.825	C:0.175
chr28	11613175	2	40	G:0.75	A:0.25
chr28	11613346	2	40	C:0.975	T:0.025
chr28	11613350	2	40	G:0.975	T:0.025
chr28	11613641	2	40	C:1	A:0
chr28	11613768	2	38	C:1	T:0
chr28	11613900	2	40	G:0.95	A:0.05
chr28	11614047	2	40	T:0.95	C:0.05
chr28	11614242	2	32	G:0.5625	A:0.4375
chr28	11614331	2	36	C:1	T:0
chr28	11614549	2	40	C:0.85	T:0.15
chr28	11614894	2	36	A:0.972222	G:0.0277778
chr28	11615171	2	40	G:0.975	T:0.025
chr28	11615352	2	38	T:0.973684	TA:0.0263158
chr28	11615568	2	40	C:0.95	A:0.05
chr28	11615826	2	40	G:0.975	A:0.025
chr28	11615868	2	40	CTA:1	C:0
chr28	11615952	2	40	ATTTTT:0.8	A:0.2
chr28	11615953	2	38	TTTTTTTTTTTTTTTTTTTTTTTTGGTATTTGAA:0.973684	T:0.0263158
chr28	11616070	2	36	C:0.888889	CT:0.111111
chr28	11616117	2	36	CT:0.916667	C:0.0833333
chr28	11616268	2	40	C:0.9	A:0.1
chr28	11616320	2	38	G:0.894737	T:0.105263
chr28	11616406	2	40	GTA:1	G:0
chr28	11616536	2	38	CA:0.921053	C:0.0789474
chr28	11616567	2	40	TC:0.925	T:0.075
chr28	11616568	2	40	C:0.95	CT:0.05
chr28	11616613	2	38	A:1	C:0
chr28	11616697	2	40	G:0.15	A:0.85
chr28	11616963	2	40	G:0.7	C:0.3
chr28	11617017	2	36	T:0.861111	C:0.138889
chr28	11617076	2	40	T:0.9	G:0.1
chr28	11617079	2	40	C:0.975	T:0.025
chr28	11617455	2	40	G:0.95	A:0.05
chr28	11617492	2	38	T:0.657895	C:0.342105
chr28	11617795	2	38	A:0.947368	G:0.0526316
chr28	11618216	2	40	AT:1	A:0
chr28	11618267	2	38	T:0.736842	C:0.263158
chr28	11618315	2	40	G:0.975	A:0.025
chr28	11618439	2	40	T:0.925	C:0.075
chr28	11618928	2	38	GT:0.973684	G:0.0263158
chr28	11618960	2	40	C:0.9	A:0.1
chr28	11619029	2	38	T:0.605263	TG:0.394737
chr28	11619034	2	38	C:0.894737	T:0.105263
chr28	11619208	2	38	G:0.894737	C:0.105263
chr28	11619407	2	38	A:1	T:0
chr28	11619418	2	38	A:0.868421	G:0.131579
chr28	11619623	2	40	G:0.925	T:0.075
chr28	11619624	2	40	A:0.5	T:0.5
chr28	11619667	2	38	C:0.894737	T:0.105263
chr28	11619802	2	38	T:0.868421	A:0.131579
chr28	11619807	2	38	G:0.868421	T:0.131579

chr28	11620066	2	38	G:0.947368	A:0.0526316		
chr28	11620197	2	40	G:1	A:0		
chr28	11620293	2	40	TG:0.625	T:0.375		
chr28	11620360	2	38	C:0.921053	T:0.0789474		
chr28	11620596	2	38	G:0.894737	A:0.105263		
chr28	11620674	2	38	G:1	A:0		
chr28	11620751	2	40	A:0.95	G:0.05		
chr28	11620765	2	40	C:0.975	T:0.025		
chr28	11620843	2	40	A:1	G:0		
chr28	11620896	2	38	A:0.973684	C:0.0263158		
chr28	11621240	2	40	A:0.75	T:0.25		
chr28	11621573	2	40	G:0.925	A:0.075		
chr28	11621624	2	40	G:0.925	A:0.075		
chr28	11621742	2	40	A:0.675	G:0.325		
chr28	11621773	2	40	T:0.975	C:0.025		
chr28	11621848	2	38	G:0.894737	A:0.105263		
chr28	11622244	2	38	T:0.657895	A:0.342105		
chr28	11623106	2	40	A:0.775	AATTT:0.225		
chr28	11623164	2	40	C:0.725	T:0.275		
chr28	11623166	2	40	C:0.725	T:0.275		
chr28	11623243	2	38	T:0.631579	G:0.368421		
chr28	11623314	2	40	G:0.825	A:0.175		
chr28	11623361	2	38	C:0.763158	T:0.236842		
chr28	11623373	2	38	T:0.684211	C:0.315789		
chr28	11623385	2	40	G:0.925	T:0.075		
chr28	11623406	2	38	C:0.684211	T:0.315789		
chr28	11623440	2	38	T:1	C:0		
chr28	11623894	2	40	C:1	T:0		
chr28	11624156	2	38	C:1	T:0		
chr28	11624224	2	40	T:0.925	C:0.075		
chr28	11624878	2	40	A:0.95	T:0.05		
chr28	11625075	2	40	C:0.65	T:0.35		
chr28	11625161	2	38	C:0.631579	T:0.368421		
chr28	11625279	2	40	T:0.725	C:0.275		
chr28	11625400	2	38	GTCAC:0.921053	G:0.0789474		
chr28	11625617	2	40	G:0.6	A:0.4		
chr28	11625771	2	40	G:0.6	C:0.4		
chr28	11625905	3	38	TC:0.605263	T:0.315789	CC:0.0789474	
chr28	11625915	2	38	T:0.684211	C:0.315789		
chr28	11625962	2	38	G:0.552632	GTATT:0.447368		
chr28	11626066	3	40	CTGTGTG:0.525	C:0.425	CTGTG:0.05	
chr28	11626244	4	40	CAG:0.475	C:0.275	CAGAG:0.2	CAGAGAG:0.05
chr28	11626315	2	40	G:0.825	A:0.175		
chr28	11626402	2	36	A:0.666667	G:0.333333		
chr28	11626637	2	40	C:0.65	T:0.35		
chr28	11627103	2	38	C:0.921053	T:0.0789474		
chr28	11627334	2	40	A:0.925	G:0.075		
chr28	11627622	2	40	TTC:1	T:0		
chr28	11627984	2	40	T:0.975	G:0.025		
chr28	11628016	2	40	A:0.925	T:0.075		
chr28	11628478	2	40	A:0.675	G:0.325		

chr28 11628656 2 36 G:1 A:0
chr28 11628658 2 36 G:0.944444 A:0.0555556
chr28 11628696 2 40 CTTTATCATATTCTTTATTCTCT:0.875
C:0.125
chr28 11628917 2 40 T:0.975 A:0.025
chr28 11629114 2 38 CTTTTTT:0.894737 C:0.105263
chr28 11629359 2 40 A:0.925 G:0.075
chr28 11629750 2 38 T:0.973684 C:0.0263158
chr28 11629885 2 40 G:0.975 A:0.025
chr28 11629911 2 38 T:0.973684 C:0.0263158
chr28 11629947 2 40 C:1 T:0
chr28 11630066 2 38 T:0.973684 G:0.0263158
chr28 11630218 2 40 T:0.975 C:0.025
chr28 11630386 2 40 A:0.6 C:0.4
chr28 11630569 2 38 A:0.842105 G:0.157895
chr28 11630669 2 38 G:0.605263 A:0.394737
chr28 11630972 2 40 C:0.7 T:0.3
chr28 11631002 2 40 C:0.75 G:0.25
chr28 11631112 2 40 G:0.975 A:0.025
chr28 11631274 2 40 C:0.675 T:0.325
chr28 11631806 2 38 AT:0.657895 A:0.342105
chr28 11631807 2 38 T:0.894737 A:0.105263
chr28 11631961 2 36 AC:0.694444 A:0.305556
chr28 11631997 2 36 G:0.916667 T:0.0833333
chr28 11632048 2 40 G:0.65 A:0.35
chr28 11632389 2 40 A:0.525 G:0.475
chr28 11632448 2 40 C:0.925 G:0.075
chr28 11633018 2 40 CA:0.6 C:0.4
chr28 11633136 2 34 T:0.558824 TC:0.441176
chr28 11633395 2 40 C:1 T:0
chr28 11633438 2 40 T:0.925 C:0.075
chr28 11633629 2 38 G:1 A:0
chr28 11633883 2 38 G:0.868421 A:0.131579
chr28 11634053 2 36 T:0.555556 C:0.444444
chr28 11634287 2 36 C:0.722222 T:0.277778
chr28 11634408 2 38 TGTAA:1 T:0
chr28 11634473 2 38 C:0.789474 A:0.210526
chr28 11634938 2 40 C:0.9 CTTA:0.1
chr28 11634941 2 40 C:0.9 G:0.1
chr28 11634943 2 40 A:0.9 AGAACCTCT:0.1
chr28 11634945 2 40 A:0.9 ATTGAAGAACC:0.1
chr28 11635125 2 40 GC:0.925 G:0.075
chr28 11635128 2 40 T:0.925 G:0.075
chr28 11635701 2 38 A:0.631579 G:0.368421
chr28 11636000 2 32 G:0.96875 A:0.03125
chr28 11636112 2 38 T:0.736842 C:0.263158
chr28 11636162 3 38 G:0.684211 GAATA:0
GAATAAATA:0.315789
chr28 11636195 2 40 A:0.975 AATACATAAATACATAAATAC:
0.025
chr28 11636202 2 40 A:0.875 AC:0.125
chr28 11636203 6 40 A:0.575 AAAAAAAAT:0
AATAAAAAT:0.1 AATAAATAAAAAT:0.15
AATACATAAATAAATAAATAAAAAT:0.05

ATAAATACATAAATACATAAATAAATAAATAAAAAT:0.125

chr28	11636449	2	40	GCAA:1	G:0	
chr28	11636450	3	40	CA:0.675	C:0.075	CAA:0.25
chr28	11636585	2	40	T:0.925	C:0.075	
chr28	11636666	2	40	A:0.925	C:0.075	
chr28	11636699	2	40	C:0.925	T:0.075	
chr28	11637097	2	34	C:1	T:0	
chr28	11637148	2	32	TTC:0.875		T:0.125
chr28	11637876	2	38	A:0.921053		C:0.0789474
chr28	11638360	2	38	T:1	C:0	
chr28	11638539	2	40	T:1	C:0	
chr28	11638683	2	40	C:0.6	T:0.4	
chr28	11638838	2	38	T:0.894737		G:0.105263
chr28	11638897	2	38	A:0.947368		AGC:0.0526316
chr28	11638902	3	38	A:0.710526		ACG:0.236842
G:0.0526316						
chr28	11638910	2	38	G:0.894737		GCGCACA:0.105263
chr28	11638912	2	38	A:0.526316		G:0.473684
chr28	11639095	2	36	C:0.944444		T:0.0555556
chr28	11639874	2	36	C:0.972222		A:0.0277778
chr28	11640588	6	40	CATCTATCTATCTATCTATCT:0.2	C:0.1	
CATCTATCTATCTATCT:0.375			CATCTATCTATCTATCTATCTATCT:0.325			
CATCTATCTATCTATCTATCTATCTATCT:0				CATCTATCTATCTATCTATCTATCTATCTATCT:		
0						
chr28	11640754	2	38	A:0.131579		C:0.868421
chr28	11640794	2	40	T:0.15	TTATC:0.85	
chr28	11640807	2	40	G:1	A:0	
chr28	11640936	2	40	A:0.875	T:0.125	
chr28	11641278	2	40	A:0.925	AT:0.075	
chr28	11641403	2	34	A:0.941176		C:0.0588235
chr28	11641509	2	38	G:0.921053		A:0.0789474
chr28	11641581	2	38	A:1	G:0	
chr28	11641643	2	36	C:0.888889		T:0.111111
chr28	11641993	2	36	C:0.805556		T:0.194444
chr28	11642098	2	40	AG:0.75	A:0.25	
chr28	11642201	2	38	G:1	A:0	
chr28	11642416	2	38	G:0.736842		C:0.263158
chr28	11642575	2	38	G:0.842105		A:0.157895
chr28	11642692	2	34	A:0.794118		G:0.205882
chr28	11642904	2	38	T:0.815789		C:0.184211
chr28	11643368	2	38	CCT:1	C:0	
chr28	11643389	2	38	C:0.789474		G:0.210526
chr28	11643473	2	40	C:0.9	T:0.1	
chr28	11643490	2	40	C:0.8	T:0.2	
chr28	11643556	2	40	A:0.8	G:0.2	
chr28	11643956	2	40	G:0.8	A:0.2	
chr28	11643994	2	38	C:0.763158		T:0.236842
chr28	11644107	2	38	A:1	G:0	
chr28	11644288	2	34	T:0.735294		C:0.264706
chr28	11644717	2	38	A:0.789474		G:0.210526
chr28	11644808	2	40	A:0.95	G:0.05	
chr28	11645247	2	40	A:0.8	G:0.2	
chr28	11646411	2	40	T:0.875	C:0.125	
chr28	11646414	2	40	C:0.9	T:0.1	

chr28	11646644	2	38	C:1	T:0	
chr28	11647305	2	34	GT:0.823529		G:0.176471
chr28	11648316	2	34	TG:0.911765		T:0.0882353
chr28	11648719	2	38	C:0.684211		G:0.315789
chr28	11648877	2	40	G:0.975	A:0.025	
chr28	11648879	2	40	G:0.8	GCA:0.2	
chr28	11649448	2	38	C:0.921053		T:0.0789474
chr28	11649850	2	38	T:0.842105		C:0.157895
chr28	11650074	2	36	T:0.694444		C:0.305556
chr28	11650446	2	40	A:0.95	T:0.05	
chr28	11650763	2	38	T:1	C:0	
chr28	11651129	2	38	C:0.789474		CT:0.210526
chr28	11651162	2	38	G:0.789474		A:0.210526
chr28	11651188	2	40	C:0.775	T:0.225	
chr28	11651220	2	38	G:0.789474		A:0.210526
chr28	11651262	2	38	G:0.763158		C:0.236842
chr28	11651378	2	36	A:0.777778		G:0.222222
chr28	11651404	2	36	C:0.805556		T:0.194444
chr28	11651493	2	40	A:0.75	T:0.25	
chr28	11651708	2	40	C:0.9	T:0.1	
chr28	11651773	2	38	G:0.815789		A:0.184211
chr28	11651917	2	40	T:0.775	A:0.225	
chr28	11651918	2	40	T:0.775	C:0.225	
chr28	11651981	2	40	C:0.75	CTT:0.25	
chr28	11652232	2	28	C:0.821429		CCTT:0.178571
chr28	11652278	2	30	C:1	T:0	
chr28	11652330	2	26	T:0.923077		C:0.0769231
chr28	11652763	2	32	C:0.96875		CTA:0.03125
chr28	11653163	2	38	GAGA:0.815789		G:0.184211
chr28	11653191	2	36	C:0.972222		T:0.0277778
chr28	11653359	2	40	A:0.9	G:0.1	
chr28	11653409	2	32	C:0.875	A:0.125	
chr28	11653426	2	32	G:0.875	A:0.125	
chr28	11653579	2	32	A:0.75	T:0.25	
chr28	11653592	2	32	T:0.75	A:0.25	
chr28	11654206	2	24	C:0.875	T:0.125	
chr28	11654215	2	24	A:0.791667		G:0.208333
chr28	11654296	2	36	C:0.722222		T:0.277778
chr28	11654401	2	40	T:0.875	G:0.125	
chr28	11654527	2	38	C:0.868421		T:0.131579
chr28	11654533	2	38	T:0.736842		C:0.263158
chr28	11654573	3	40	CCT:0.45	C:0.25	CCTCTCT:0.3
chr28	11654622	2	40	C:0.8	T:0.2	
chr28	11654717	2	38	G:0.789474		C:0.210526
chr28	11654744	2	40	T:0.8	C:0.2	
chr28	11654788	2	40	C:0.775	T:0.225	
chr28	11654806	2	40	G:0.775	GGA:0.225	
chr28	11654835	2	40	A:0.725	G:0.275	
chr28	11654906	2	40	C:0.875	T:0.125	
chr28	11655174	2	40	C:0.875	T:0.125	
chr28	11655291	2	38	C:0.815789		T:0.184211
chr28	11655316	2	38	G:0.815789		A:0.184211
chr28	11655319	2	38	G:1	A:0	
chr28	11655324	2	38	C:0.894737		T:0.105263

chr28	11655454	2	38	T:0.789474	TCTTA:0.210526			
chr28	11655790	2	40	T:0.825	C:0.175			
chr28	11655791	2	40	G:0.825	A:0.175			
chr28	11655925	2	38	A:0.921053	G:0.0789474			
chr28	11655948	2	40	G:0.8	A:0.2			
chr28	11656006	2	38	C:1	T:0			
chr28	11656314	2	38	A:0.789474	G:0.210526			
chr28	11656333	2	38	A:0.789474	C:0.210526			
chr28	11656345	2	38	A:0.789474	C:0.210526			
chr28	11656370	2	40	A:0.9	G:0.1			
chr28	11656439	2	38	C:0.736842	T:0.263158			
chr28	11656683	2	40	C:0.75	G:0.25			
chr28	11656689	2	40	C:0.95	G:0.05			
chr28	11656782	2	40	G:0.775	A:0.225			
chr28	11656805	2	40	AG:0.8	A:0.2			
chr28	11656806	2	40	G:0.85	A:0.15			
chr28	11656895	2	38	A:0.815789	T:0.184211			
chr28	11657017	2	40	A:0.925	G:0.075			
chr28	11657049	2	38	A:0.789474	C:0.210526			
chr28	11657074	2	38	A:0.921053	T:0.0789474			
chr28	11657122	2	40	T:1	C:0			
chr28	11657324	2	38	A:0.763158	G:0.236842			
chr28	11657342	2	38	A:0.763158	C:0.236842			
chr28	11657425	2	40	A:0.775	G:0.225			
chr28	11657472	2	40	CT:0.925	C:0.075			
chr28	11657604	2	40	A:0.8	G:0.2			
chr28	11657746	2	40	T:0.8	C:0.2			
chr28	11657954	5	40	GTTATTATTATTA:0.1	G:0.3	GTTA:0	GTTATTA:0.225	GTTATTATTA:0.375
chr28	11657963	2	40	A:0.9	G:0.1			
chr28	11657969	2	40	A:1	G:0			
chr28	11658263	2	38	A:0.921053	G:0.0789474			
chr28	11658441	2	38	C:0.631579	A:0.368421			
chr28	11658526	2	38	A:0.921053	T:0.0789474			
chr28	11658790	2	36	C:0.916667	A:0.0833333			
chr28	11658823	2	34	A:0.588235	G:0.411765			
chr28	11659626	2	40	C:0.9	CT:0.1			
chr28	11659758	2	38	T:0.894737	C:0.105263			
chr28	11661093	3	36	A:0.638889	C:0.0833333	T:0.277778		
chr28	11661214	2	40	T:0.6	TA:0.4			
chr28	11661386	2	38	G:0.921053	A:0.0789474			
chr28	11661490	2	36	C:0.888889	T:0.111111			
chr28	11661717	2	38	C:0.631579	T:0.368421			
chr28	11661758	2	40	A:0.7	G:0.3			
chr28	11661816	2	40	T:1	A:0			
chr28	11661817	2	40	G:1	T:0			
chr28	11661984	2	38	T:0.684211	C:0.315789			
chr28	11662084	2	38	T:0.921053	C:0.0789474			
chr28	11662169	2	38	C:0.921053	T:0.0789474			
chr28	11662237	2	38	T:0.947368	TTAGGTAGAATA:0.0526316			
chr28	11662282	2	38	G:0.973684	C:0.0263158			
chr28	11662462	2	40	AAC:0.85	A:0.15			

chr28	11662480	2	40	ACTGTGAGCTACACTGAC:0.85	A:0.15
chr28	11662796	2	38	A:0.657895	AC:0.342105
chr28	11662804	2	38	C:0.921053	T:0.0789474
chr28	11662825	2	40	G:0.7	A:0.3
chr28	11662862	2	40	T:0.7	A:0.3
chr28	11662884	2	40	A:0.75	T:0.25
chr28	11663181	2	36	A:0.972222	ACT:0.0277778
chr28	11663292	2	40	A:0.7	T:0.3
chr28	11663403	2	36	T:0.611111	C:0.388889
chr28	11663419	2	36	T:0.722222	C:0.277778
chr28	11663575	2	38	C:0.578947	T:0.421053
chr28	11663586	2	38	A:0.578947	AT:0.421053
chr28	11663630	2	38	C:0.605263	T:0.394737
chr28	11663675	2	38	T:0.921053	C:0.0789474
chr28	11663739	2	38	G:0.631579	T:0.368421
chr28	11663741	2	38	G:0.921053	C:0.0789474
chr28	11663792	2	38	A:0.578947	C:0.421053
chr28	11663894	2	40	GA:0.525	G:0.475
chr28	11663948	2	38	C:0.526316	T:0.473684
chr28	11664110	2	40	A:0.925	T:0.075
chr28	11664191	2	38	T:0.736842	C:0.263158
chr28	11664251	2	38	CATA:0.605263	C:0.394737
chr28	11664254	2	38	A:0.894737	T:0.105263
chr28	11664256	2	38	T:0.894737	C:0.105263
chr28	11664326	2	40	C:0.625	T:0.375
chr28	11664351	2	40	T:0.625	C:0.375
chr28	11664406	2	40	G:0.675	C:0.325
chr28	11664486	2	40	A:0.9	T:0.1
chr28	11664713	2	40	G:0.95	A:0.05
chr28	11664724	2	40	G:0.625	A:0.375
chr28	11664901	2	40	C:0.925	A:0.075
chr28	11664945	2	40	C:0.875	A:0.125
chr28	11664965	2	40	T:0.95	A:0.05
chr28	11665294	2	38	A:0.552632	G:0.447368
chr28	11665320	2	36	C:0.694444	T:0.305556
chr28	11665484	2	38	G:0.552632	GA:0.447368
chr28	11665594	2	38	G:0.605263	A:0.394737
chr28	11665678	2	38	T:0.5	C:0.5
chr28	11665705	2	36	C:0.694444	T:0.305556
chr28	11665706	2	36	A:0.555556	G:0.444444
chr28	11665989	2	40	C:0.95	T:0.05
chr28	11666004	2	40	G:1	C:0
chr28	11666012	2	40	T:0.95	C:0.05
chr28	11666106	2	40	G:0.9	A:0.1
chr28	11666130	2	38	T:0.578947	C:0.421053
chr28	11666159	2	40	G:0.6	T:0.4
chr28	11666161	2	40	C:0.65	T:0.35
chr28	11666193	2	38	C:1	T:0
chr28	11666285	2	38	C:0.526316	T:0.473684
chr28	11666395	2	40	G:0.55	T:0.45
chr28	11666414	2	40	T:0.575	C:0.425
chr28	11666482	2	40	G:0.55	A:0.45
chr28	11666629	2	38	G:0.605263	T:0.394737
chr28	11666647	2	38	C:0.921053	G:0.0789474

chr28	11666701	2	40	C:0.625	T:0.375	
chr28	11666755	2	40	G:0.725	A:0.275	
chr28	11666762	2	40	C:0.725	T:0.275	
chr28	11666835	2	38	CATT:0.894737	C:0.105263	
chr28	11666869	2	38	A:0.894737	T:0.105263	
chr28	11666939	2	38	A:0.5	T:0.5	
chr28	11667041	2	38	C:0.578947	A:0.421053	
chr28	11667295	2	40	TAC:0.975	T:0.025	
chr28	11667321	2	40	CACACAA:0.975	C:0.025	
chr28	11667323	2	40	CACAA:0.85	C:0.15	
chr28	11667325	2	40	CAA:0.8	C:0.2	
chr28	11667469	2	40	C:0.775	T:0.225	
chr28	11667474	2	40	G:0.575	A:0.425	
chr28	11667485	2	38	C:0.842105	G:0.157895	
chr28	11667540	2	40	G:0.55	A:0.45	
chr28	11667708	2	36	T:0.944444	TAC:0.0555556	
chr28	11667740	2	40	A:0.55	G:0.45	
chr28	11667796	2	40	T:0.575	C:0.425	
chr28	11667797	2	40	T:0.575	A:0.425	
chr28	11667809	2	40	G:0.95	A:0.05	
chr28	11667887	2	38	A:1	G:0	
chr28	11668132	2	38	T:0.605263	C:0.394737	
chr28	11668503	2	40	C:0.95	T:0.05	
chr28	11668526	2	40	GT:0.9	G:0.1	
chr28	11668732	2	38	GA:0.947368	G:0.0526316	
chr28	11668754	2	38	C:0.947368	T:0.0526316	
chr28	11668838	2	36	A:0.972222	G:0.0277778	
chr28	11668925	2	38	GCCTTTAA:0.921053		G: 0.0789474
chr28	11668967	2	34	T:0.941176	C:0.0588235	
chr28	11669252	2	36	T:0.916667	C:0.0833333	
chr28	11669308	2	38	C:0.973684	A:0.0263158	
chr28	11669781	2	40	G:0.95	C:0.05	
chr28	11670042	2	40	CAT:0.95	C:0.05	
chr28	11670043	3	40	A:0.625	ATG:0.375	ATGTG:0
chr28	11670061	2	40	G:0.95	A:0.05	
chr28	11670064	2	40	C:0.95	G:0.05	
chr28	11670314	2	40	AC:0.8	A:0.2	
chr28	11670454	2	38	G:0.921053	A:0.0789474	
chr28	11670603	2	40	A:0.65	T:0.35	
chr28	11671073	2	40	C:0.825	T:0.175	
chr28	11671184	2	40	G:0.675	A:0.325	
chr28	11671202	2	40	C:0.625	T:0.375	
chr28	11671346	2	40	T:0.55	C:0.45	
chr28	11671361	2	40	C:0.55	T:0.45	
chr28	11671460	2	40	T:1	A:0	
chr28	11671813	2	40	C:0.6	T:0.4	
chr28	11671926	2	40	G:1	A:0	
chr28	11671947	2	40	C:0.525	G:0.475	
chr28	11672114	2	40	ACTTTCC:0.975	A:0.025	
chr28	11672145	2	40	A:0.975	G:0.025	
chr28	11672149	2	38	T:0.578947	A:0.421053	
chr28	11672178	2	36	A:0.888889	G:0.111111	
chr28	11672321	2	38	C:1	G:0	

chr28 11672355 2 36 T:0.888889 G:0.111111
chr28 11672417 2 36 TG:0.694444 T:0.305556
chr28 11672566 6 38 TTTTATTTATTTATTTATTTA:0.157895
T:0.578947 TTTTA:0.105263 TTTTATTTA:0.0263158
TTTTATTTATTTA:0.0526316 TTTTATTTATTTATTTA:0.0789474
chr28 11672615 2 38 TTTA:0.868421 T:0.131579
chr28 11672855 2 40 GTAAT:0.825 G:0.175
chr28 11672983 2 36 GATTA:0.833333 G:0.166667
chr28 11674067 2 38 C:0.552632 A:0.447368
chr28 11674195 3 38 TA:0.631579 T:0.342105
TAA:0.0263158
chr28 11674415 2 40 T:1 A:0
chr28 11674480 2 38 C:1 A:0
chr28 11675099 4 38 TAA:0.684211 T:0.0263158
TA:0.0789474 TAAA:0.210526
chr28 11675118 2 38 GA:0.815789 G:0.184211
chr28 11675396 2 38 C:0.605263 CA:0.394737
chr28 11675414 2 38 G:1 A:0
chr28 11675566 2 40 G:0.9 A:0.1
chr28 11675926 2 40 TA:0.625 T:0.375
chr28 11676070 2 38 A:1 AT:0
chr28 11676090 2 40 A:0.8 T:0.2
chr28 11676203 2 40 T:0.7 TA:0.3
chr28 11676290 2 38 G:0.894737 A:0.105263
chr28 11676352 2 40 C:0.675 T:0.325
chr28 11676692 2 38 TAAA:0.605263 T:0.394737
chr28 11676696 2 38 A:0.710526 T:0.289474
chr28 11676739 2 34 T:0.588235 C:0.411765
chr28 11676836 2 36 C:0.777778 G:0.222222
chr28 11676889 2 34 C:0.882353 T:0.117647
chr28 11677022 2 38 T:0.657895 TAGTC:0.342105
chr28 11677158 2 38 A:0.657895 AAG:0.342105
chr28 11677159 2 38 A:1 AG:0
chr28 11677164 2 38 T:1 A:0
chr28 11677170 2 38 A:1 G:0
chr28 11677360 2 40 C:0.675 CA:0.325
chr28 11677631 3 38 TGAGA:0.578947 T:0 TGAGAGA:
0.421053
chr28 11677662 2 38 C:0.921053 T:0.0789474
chr28 11677675 2 38 C:0.684211 A:0.315789
chr28 11677838 2 36 C:0.611111 G:0.388889
chr28 11678159 3 34 C:0.705882 CT:0.117647
CCTT:0.176471
chr28 11678183 2 32 T:0.5 C:0.5
chr28 11678370 2 38 A:1 G:0
chr28 11678849 2 40 T:1 C:0
chr28 11678978 2 38 A:0.789474 AT:0.210526
chr28 11679100 2 34 T:0.911765 C:0.0882353
chr28 11679252 2 38 C:1 A:0
chr28 11679465 2 40 C:0.625 T:0.375
chr28 11679592 2 36 A:1 C:0
chr28 11679828 2 30 TC:0.566667 T:0.433333
chr28 11680019 2 30 C:0.866667 T:0.133333
chr28 11680206 2 36 C:0.666667 G:0.333333

chr28	11680249	2	34	C:0.617647	T:0.382353	
chr28	11680371	2	38	G:0.815789	A:0.184211	
chr28	11680456	2	36	T:0.833333	C:0.166667	
chr28	11680552	2	32	C:0	CA:1	
chr28	11681024	2	36	G:0.861111	A:0.138889	
chr28	11681265	2	40	T:0.925	A:0.075	
chr28	11681314	2	38	C:0.921053	T:0.0789474	
chr28	11681488	2	38	CAGAAACTCGGATGG:0.947368	C:0.0526316	
chr28	11681515	2	38	G:1	A:0	
chr28	11681635	2	38	T:0.815789	G:0.184211	
chr28	11681638	2	38	A:0.894737	ACAG:0.105263	
chr28	11681822	2	40	T:0.575	G:0.425	
chr28	11681960	2	38	C:0.868421	T:0.131579	
chr28	11681964	2	38	C:1	T:0	
chr28	11682021	2	36	G:0.583333	A:0.416667	
chr28	11682113	2	40	T:0.55	C:0.45	
chr28	11682143	2	40	A:0.875	G:0.125	
chr28	11682214	2	38	T:0.921053	C:0.0789474	
chr28	11682221	2	38	G:0.921053	A:0.0789474	
chr28	11682522	2	38	T:0.842105	C:0.157895	
chr28	11682826	2	38	C:0.894737	A:0.105263	
chr28	11682863	2	38	C:0.947368	T:0.0526316	
chr28	11682929	2	36	T:0.888889	C:0.111111	
chr28	11682955	2	36	C:0.888889	T:0.111111	
chr28	11683463	2	38	A:0.973684	C:0.0263158	
chr28	11683516	2	34	TA:1	T:0	
chr28	11683703	2	40	T:1	TA:0	
chr28	11683704	3	40	T:0.8	A:0.025	TA:0.175
chr28	11683871	2	36	A:0.833333	T:0.166667	
chr28	11683882	2	36	T:0.833333	C:0.166667	
chr28	11683904	2	36	T:0.944444	C:0.0555556	
chr28	11683953	2	40	G:0.875	A:0.125	
chr28	11684268	2	38	T:0.868421	A:0.131579	
chr28	11684270	2	38	C:1	CT:0	
chr28	11684488	2	40	T:0.825	C:0.175	
chr28	11684636	2	40	T:0.875	C:0.125	
chr28	11684830	2	38	A:0.789474	T:0.210526	
chr28	11684927	2	40	C:0.875	G:0.125	
chr28	11685424	2	40	T:0.975	C:0.025	
chr28	11685456	2	38	A:0.894737	G:0.105263	
chr28	11685471	2	38	T:0.973684	C:0.0263158	
chr28	11685838	2	40	C:1	T:0	
chr28	11686012	2	36	C:0.888889	A:0.111111	
chr28	11686046	2	38	C:0.842105	T:0.157895	
chr28	11686094	2	38	G:0.921053	T:0.0789474	
chr28	11686482	2	40	C:0.9	A:0.1	
chr28	11686583	2	38	G:0.921053	C:0.0789474	
chr28	11686733	2	40	A:0.925	T:0.075	
chr28	11687334	2	40	A:0.65	G:0.35	
chr28	11687417	2	40	C:1	T:0	
chr28	11688034	2	38	A:1	C:0	
chr28	11688227	2	38	T:0.947368	C:0.0526316	
chr28	11688665	2	40	A:0.725	T:0.275	

chr28	11688672	2	40	T:0.725	TCTC:0.275
chr28	11688771	2	40	G:0.875	A:0.125
chr28	11688958	2	34	T:0.441176	TA:0.558824
chr28	11689308	2	38	T:0.789474	A:0.210526
chr28	11689682	2	38	G:0.710526	A:0.289474
chr28	11690414	2	36	C:0.583333	T:0.416667
chr28	11690489	2	38	T:1	G:0
chr28	11690556	2	40	A:0.65	ACAAAGTAGTGTCATC:0.35
chr28	11690879	2	18	TCC:1	T:0
chr28	11690883	2	16	CCCCCA:0.9375	C:0.0625
chr28	11690885	2	18	CCCA:0.777778	C:0.222222
chr28	11690888	2	18	ACCC:1	A:0
chr28	11690889	2	18	C:0.777778	G:0.222222
chr28	11690977	2	38	T:0.447368	C:0.552632
chr28	11691100	2	38	T:0.736842	C:0.263158
chr28	11691179	2	40	T:1	C:0
chr28	11691215	2	36	C:0.694444	G:0.305556
chr28	11691342	4	32	TAAA:0.5	T:0	TA:0.28125
TAAAA:0.21875
chr28	11691381	3	34	C:0.411765	CT:0.264706
CTT:0.323529
chr28	11691535	2	18	AAT:0.444444	A:0.555556
chr28	11691644	2	20	A:0.45	G:0.55
chr28	11691670	2	22	T:0.727273	C:0.272727
chr28	11691825	2	38	A:0.605263	C:0.394737
chr28	11691854	2	40	G:0.675	C:0.325
chr28	11691905	2	40	C:1	T:0
chr28	11691985	2	40	T:1	TA:0
chr28	11691988	2	40	T:1	TA:0
chr28	11691989	2	40	T:1	TA:0
chr28	11691991	2	40	T:1	TATTTATTTA:0
chr28	11691992	2	40	T:1	TATTTATTTA:0
chr28	11691996	3	40	T:1	C:0	TC:0
chr28	11692094	2	40	T:1	C:0
chr28	11692109	2	40	G:1	A:0
chr28	11692111	2	40	T:1	G:0
chr28	11692146	2	40	T:0.65	C:0.35
chr28	11692697	4	38	TAAA:0.289474	T:0.157895
TA:0.394737	TAA:0.157895
chr28	11692903	2	40	C:1	T:0
chr28	11692913	2	38	TGA:0.210526	T:0.789474
chr28	11692931	2	40	A:0.975	T:0.025
chr28	11692946	2	40	AAGAG:1	A:0
chr28	11692990	2	40	G:1	T:0
chr28	11693149	2	40	C:1	T:0
chr28	11693163	2	40	G:1	A:0
chr28	11693239	2	38	C:0.315789	T:0.684211
chr28	11693250	2	38	G:1	A:0
chr28	11693427	2	40	T:1	TAAATTCTAGCTAGAAAAAAA:0
chr28	11693736	2	40	A:0.6	G:0.4
chr28	11693880	2	38	A:1	G:0
chr28	11693898	2	38	C:0.578947	T:0.421053
chr28	11694331	2	40	C:1	CAGGG:0
chr28	11694333	2	40	T:1	TCC:0

chr28	11694335	2	40	T:1	TGGGTGGCGCAGCGGTTTGGCGCC:0		
chr28	11694776	2	36	A:1	G:0		
chr28	11694790	2	40	C:1	T:0		
chr28	11695051	2	40	G:1	A:0		
chr28	11695127	2	38	C:0.684211	T:0.315789		
chr28	11695129	2	38	C:0.684211	T:0.315789		
chr28	11695454	3	38	A:0.973684	AT:0.0263158	ATT:0	
chr28	11695464	2	38	A:0.315789	T:0.684211		
chr28	11695499	2	36	TGAGA:0.333333	T:0.666667		
chr28	11695895	2	38	G:1	T:0		
chr28	11695928	2	36	A:1	G:0		
chr28	11695971	2	38	C:1	CA:0		
chr28	11696043	2	38	T:1	C:0		
chr28	11696157	2	38	A:1	T:0		
chr28	11696322	2	40	T:1	C:0		
chr28	11696333	2	40	T:0.625	C:0.375		
chr28	11696491	2	40	T:1	G:0		
chr28	11696563	2	38	T:0.710526	C:0.289474		
chr28	11696569	2	38	A:1	C:0		
chr28	11696667	2	38	C:1	T:0		
chr28	11696696	2	38	G:1	A:0		
chr28	11696738	2	38	T:1	C:0		
chr28	11696739	2	38	G:1	A:0		
chr28	11696799	2	40	G:1	T:0		
chr28	11696804	2	40	A:0.35	G:0.65		
chr28	11696821	2	40	TC:1	T:0		
chr28	11696879	4	38	TAA:0.710526	T:0.0263158	TA:0.0789474	TAAA:0.184211
chr28	11696890	2	38	A:1	G:0		
chr28	11696987	2	38	C:1	T:0		
chr28	11697054	2	40	T:0.65	C:0.35		
chr28	11697164	2	38	T:0.684211	C:0.315789		
chr28	11697601	2	38	T:0.657895	C:0.342105		
chr28	11697656	2	40	G:0.7	A:0.3		
chr28	11697697	2	36	C:0.722222	T:0.277778		
chr28	11698030	2	34	C:0.617647	T:0.382353		
chr28	11698157	2	40	A:0.65	G:0.35		
chr28	11698169	2	40	C:0.65	T:0.35		
chr28	11698275	2	40	T:0.55	C:0.45		
chr28	11698653	2	38	TC:0.657895	T:0.342105		
chr28	11698840	2	38	T:1	C:0		
chr28	11698847	2	36	G:1	T:0		
chr28	11699712	2	38	A:0.947368	G:0.0526316		
chr28	11699795	2	38	C:1	T:0		
chr28	11700007	2	40	TTGAC:0.675	T:0.325		
chr28	11700072	2	38	GC:0.473684	G:0.526316		
chr28	11700076	2	38	A:0.473684	ACTGT:0.526316		
chr28	11700159	2	40	T:0.325	C:0.675		
chr28	11700274	2	38	T:0.368421	C:0.631579		
chr28	11700395	2	38	A:0.631579	G:0.368421		
chr28	11700691	2	36	A:0.805556	C:0.194444		
chr28	11700988	2	36	A:0.5	AAAG:0.5		

chr28	11701131	2	38	C:0.342105	T:0.657895		
chr28	11701418	2	38	A:0.315789	G:0.684211		
chr28	11701430	2	38	CATG:1	C:0		
chr28	11701535	2	38	A:0.368421	C:0.631579		
chr28	11701569	2	40	T:0.325	G:0.675		
chr28	11701671	2	38	A:0.736842	G:0.263158		
chr28	11701845	2	40	C:0.375	A:0.625		
chr28	11702069	2	40	A:0.45	G:0.55		
chr28	11702330	2	40	T:0.375	C:0.625		
chr28	11702460	2	38	G:0.473684	A:0.526316		
chr28	11702480	2	38	A:0.473684	G:0.526316		
chr28	11702530	2	40	T:0.7	C:0.3		
chr28	11702555	2	40	AT:0.925	A:0.075		
chr28	11702576	4	40	CAG:0.75	C:0.25	CAGAG:0	CAGAGAGAG:0
chr28	11702775	2	38	A:1	G:0		
chr28	11703062	2	40	C:0.95	A:0.05		
chr28	11703929	2	40	G:1	T:0		
chr28	11703942	2	40	A:0.975	C:0.025		
chr28	11703970	3	40	A:0.725	ATG:0.275	ATGTG:0	
chr28	11703996	4	40	G:0.675	A:0.3	GTGTA:0	GTGTGTGTA:0.025
chr28	11703997	2	40	T:1	TGTAA:0		
chr28	11703998	2	40	A:0.625	ATATATG:0.375		
chr28	11704093	2	40	AACTT:0.7	A:0.3		
chr28	11704168	2	36	T:0.5	G:0.5		
chr28	11704368	2	40	C:0.975	T:0.025		
chr28	11704375	2	40	C:1	G:0		
chr28	11705080	2	40	ACCAAGCAGTG:0.875	A:0.125		
chr28	11705272	2	38	G:1	A:0		
chr28	11705417	2	36	T:0.305556	C:0.694444		
chr28	11705474	2	36	CCTCT:0.361111	C:0.638889		
chr28	11705581	2	40	G:1	GATTT:0		
chr28	11706162	2	38	C:1	T:0		
chr28	11706172	2	38	G:1	A:0		
chr28	11706272	2	40	T:0.725	C:0.275		
chr28	11706273	2	40	G:1	A:0		
chr28	11706669	2	34	G:1	A:0		
chr28	11706952	2	38	T:0.684211	G:0.315789		
chr28	11707335	2	38	T:0.763158	C:0.236842		
chr28	11707436	2	40	A:1	ACTTCTT:0		
chr28	11707449	2	40	A:0.45	C:0.55		
chr28	11707452	2	40	A:0.7	C:0.3		
chr28	11707559	2	36	G:0.638889	A:0.361111		
chr28	11707984	2	36	G:1	A:0		
chr28	11708024	2	36	G:0.25	C:0.75		
chr28	11708418	2	40	G:0.65	A:0.35		
chr28	11708746	2	36	G:0.722222	A:0.277778		
chr28	11708955	2	36	C:1	T:0		
chr28	11709127	4	38	CT:0.236842	C:0.131579	CTT:0.473684	CTTT:0.157895
chr28	11709326	2	38	C:0.894737	T:0.105263		
chr28	11709496	2	36	G:0.805556	A:0.194444		
chr28	11709734	2	40	C:0.9	T:0.1		

chr28 11710319 2 40 A:1 T:0
chr28 11710511 2 36 G:0.555556 A:0.444444
chr28 11710516 2 36 A:0.555556 G:0.444444
chr28 11710780 2 40 C:1 CTT:0
chr28 11710832 3 40 CAA:0.025 C:0.625 CA:0.35
chr28 11710891 2 40 GA:1 G:0
chr28 11711092 2 38 G:0.421053 A:0.578947
chr28 11711396 2 40 G:0.4 A:0.6
chr28 11711437 2 38 T:0.631579 C:0.368421
chr28 11711629 2 38 G:1 A:0
chr28 11711654 2 38 T:0.684211 C:0.315789
chr28 11712607 2 40 C:0.975 T:0.025
chr28 11712708 2 40 T:0.425 C:0.575
chr28 11712782 2 40 T:0.35 G:0.65
chr28 11712968 2 36 G:0.694444 T:0.305556
chr28 11713049 2 38 C:0.394737 T:0.605263
chr28 11713254 2 40 T:0.975 C:0.025
chr28 11713386 2 36 G:0.333333 A:0.666667
chr28 11713631 2 38 G:0.973684 A:0.0263158
chr28 11713777 2 38 A:1 G:0
chr28 11713859 2 40 G:0.625 A:0.375
chr28 11713863 2 40 T:1 C:0
chr28 11713956 2 36 C:1 T:0
chr28 11714060 2 38 C:0.526316 T:0.473684
chr28 11714110 2 38 A:0.315789 G:0.684211
chr28 11714200 2 36 T:0.888889 A:0.111111
chr28 11714236 2 40 T:0.825 C:0.175
chr28 11714550 2 38 T:0.789474 A:0.210526
chr28 11714751 2 38 C:0.684211 T:0.315789
chr28 11715190 2 38 C:0.921053 T:0.0789474
chr28 11715237 2 38 G:0.973684 A:0.0263158
chr28 11715489 4 38 ATGTG:0.473684 A:0.0263158
ATG:0.289474 ATGTGTG:0.210526
chr28 11715586 2 40 T:0.8 G:0.2
chr28 11715625 2 40 G:0.75 GA:0.25
chr28 11715826 2 38 A:1 T:0
chr28 11715885 7 40 AACACACAC:0.025 A:0.075 AAC:
0.375 AACAC:0.2 AACACAC:0.225 AACACACACAC:0
AACACACACACAC:0.1
chr28 11716376 2 38 A:0.789474 G:0.210526
chr28 11716569 2 38 C:0.421053 T:0.578947
chr28 11716825 2 40 T:1 C:0
chr28 11717109 2 40 C:1 G:0
chr28 11717110 2 40 A:1 ACTGTTT:0
chr28 11717112 2 40 A:1 T:0
chr28 11717115 2 40 A:1 G:0
chr28 11717116 2 40 T:1 TGTGTTCTGTTTTA:0
chr28 11717266 2 40 A:0.725 G:0.275
chr28 11717510 2 36 G:1 A:0
chr28 11717633 2 40 A:0.775 G:0.225
chr28 11717756 2 38 G:1 C:0
chr28 11717888 2 40 C:0.95 T:0.05
chr28 11718368 2 40 C:1 T:0
chr28 11718390 2 38 C:1 T:0

chr28	11718504	2	40	G:0.625	C:0.375	
chr28	11718548	2	40	C:1	T:0	
chr28	11718549	2	40	C:0.925	T:0.075	
chr28	11718550	2	38	C:0.631579		T:0.368421
chr28	11718551	2	40	G:0.725	A:0.275	
chr28	11718565	2	40	G:0.725	A:0.275	
chr28	11718678	2	40	A:0.65	G:0.35	
chr28	11718698	2	40	C:1	T:0	
chr28	11718916	2	38	GA:0.921053		G:0.0789474
chr28	11718940	2	40	T:0.65	C:0.35	
chr28	11719139	2	40	G:0.7	GTCCA:0.3	
chr28	11719152	2	40	C:0.7	G:0.3	
chr28	11719208	2	40	G:0.55	A:0.45	
chr28	11719240	2	40	G:1	A:0	
chr28	11719633	2	38	G:1	A:0	
chr28	11720081	2	38	T:1	C:0	
chr28	11720104	2	40	G:0.85	A:0.15	
chr28	11720159	2	38	G:1	T:0	
chr28	11720191	2	38	A:0.684211		G:0.315789
chr28	11720353	2	40	C:0.75	T:0.25	
chr28	11720469	2	38	G:0.710526		A:0.289474
chr28	11720544	2	38	G:1	A:0	
chr28	11720960	2	40	A:1	T:0	
chr28	11720994	2	40	T:0.75	C:0.25	
chr28	11721002	2	40	A:0.95	G:0.05	
chr28	11721029	2	40	T:0.75	A:0.25	
chr28	11721260	2	38	C:0.315789		A:0.684211
chr28	11721292	2	40	C:1	G:0	
chr28	11721367	2	40	T:1	C:0	
chr28	11721379	2	40	C:1	T:0	
chr28	11721484	2	36	G:0.416667		A:0.583333
chr28	11721567	2	38	T:0.342105		C:0.657895
chr28	11721850	2	36	G:0.694444		A:0.305556
chr28	11722003	2	40	G:0.95	A:0.05	
chr28	11722131	2	36	G:0.583333		C:0.416667
chr28	11722288	2	38	C:0.315789		T:0.684211
chr28	11722477	2	40	A:0.55	G:0.45	
chr28	11722478	2	40	G:0.55	T:0.45	
chr28	11722493	2	38	C:1	A:0	
chr28	11722659	2	40	C:1	T:0	
chr28	11722660	2	40	A:0.625	G:0.375	
chr28	11722665	2	40	C:1	A:0	
chr28	11722748	2	40	TACTC:1	T:0	
chr28	11723119	2	36	G:1	T:0	
chr28	11723198	2	40	A:0.975	G:0.025	
chr28	11723529	2	38	G:0.921053		A:0.0789474
chr28	11723617	2	36	T:0.25	C:0.75	
chr28	11723841	2	32	C:0.6875	G:0.3125	
chr28	11724083	2	34	CA:1	C:0	
chr28	11724092	2	34	C:1	T:0	
chr28	11724129	2	40	A:1	AGTAAT:0	
chr28	11724174	2	40	T:0.825	G:0.175	
chr28	11724300	2	40	G:0.45	A:0.55	
chr28	11724624	2	38	A:1	ACACGG:0	

chr28	11724765	2	38	C:0.657895	A:0.342105
chr28	11724983	2	38	G:0.342105	A:0.657895
chr28	11725122	2	40	A:0.6	C:0.4
chr28	11725132	2	40	G:0.35	GA:0.65
chr28	11725179	2	40	A:0.375	G:0.625
chr28	11725348	2	40	G:0.775	T:0.225
chr28	11725375	2	40	A:1	G:0
chr28	11725670	2	40	C:1	G:0
chr28	11726126	2	40	G:0.425	A:0.575
chr28	11726129	2	40	A:1	G:0
chr28	11726369	2	40	A:0.825	C:0.175
chr28	11726394	2	40	A:0.5	C:0.5
chr28	11726402	2	40	T:1	C:0
chr28	11726408	2	40	G:0.45	A:0.55
chr28	11726499	2	40	G:0.825	C:0.175
chr28	11726540	2	40	G:0.6	A:0.4
chr28	11726628	2	40	C:0.775	T:0.225
chr28	11726945	2	40	T:1	C:0
chr28	11726980	2	38	G:0.736842	A:0.263158
chr28	11727124	2	36	A:0.472222	AGTT:0.527778
chr28	11727419	2	38	A:0.394737	G:0.605263
chr28	11727682	2	40	G:0.8	C:0.2
chr28	11727699	2	40	G:0.8	A:0.2
chr28	11727738	2	36	A:0.361111	T:0.638889
chr28	11727777	2	34	G:0.941176	C:0.0588235
chr28	11727890	2	40	CTTTAT:0.375	C:0.625
chr28	11727942	2	40	G:0.375	C:0.625
chr28	11728046	2	40	G:1	A:0
chr28	11728195	2	40	G:1	A:0
chr28	11728438	2	40	T:1	C:0
chr28	11728532	2	38	T:0.973684	TA:0.0263158
chr28	11728568	2	38	T:0.552632	C:0.447368
chr28	11728934	2	36	T:0.444444	C:0.555556
chr28	11728968	2	38	G:1	A:0
chr28	11729059	2	38	A:0.684211	G:0.315789
chr28	11729062	2	38	AC:1	A:0
chr28	11729189	2	38	A:0.394737	G:0.605263
chr28	11729208	2	38	A:0.894737	AT:0.105263
chr28	11729336	2	38	A:0.605263	T:0.394737
chr28	11729414	2	40	A:0.975	AT:0.025
chr28	11729845	2	40	G:0.95	GT:0.05
chr28	11729856	2	40	C:1	T:0
chr28	11730457	2	40	T:0.925	A:0.075
chr28	11730557	2	40	C:0.55	A:0.45
chr28	11730558	2	40	A:0.55	T:0.45
chr28	11730678	2	38	G:1	T:0
chr28	11731028	2	40	C:0.575	T:0.425
chr28	11731043	2	38	AT:0.973684	A:0.0263158
chr28	11731203	2	38	A:1	G:0
chr28	11731384	2	36	A:0.5	C:0.5
chr28	11731464	2	40	T:0.975	C:0.025
chr28	11731567	2	40	A:0.925	G:0.075
chr28	11731671	2	34	T:1	C:0
chr28	11731708	2	38	G:0.921053	A:0.0789474

chr28	11731739	2	38	T:1	TA:0	
chr28	11731740	2	38	G:1	A:0	
chr28	11731743	2	38	G:0.973684	T:0.0263158	
chr28	11732347	2	36	A:1	C:0	
chr28	11732348	2	36	G:1	T:0	
chr28	11732452	2	40	T:1	C:0	
chr28	11732599	2	34	C:1	T:0	
chr28	11732701	2	36	T:0.972222	C:0.0277778	
chr28	11732767	2	38	G:1	A:0	
chr28	11732838	2	40	C:0.9	T:0.1	
chr28	11732942	2	36	A:0.916667	T:0.0833333	
chr28	11733290	2	40	G:1	A:0	
chr28	11733329	2	38	A:0.5	G:0.5	
chr28	11733710	2	36	T:0.555556	C:0.444444	
chr28	11733715	2	36	T:1	C:0	
chr28	11733775	2	38	T:0.631579	C:0.368421	
chr28	11734031	2	34	G:1	A:0	
chr28	11734088	2	40	T:0.675	G:0.325	
chr28	11734722	2	38	G:1	A:0	
chr28	11734753	2	38	T:0.947368	A:0.0526316	
chr28	11735153	2	38	C:1	T:0	
chr28	11735175	2	36	C:0.972222	CT:0.0277778	
chr28	11735301	2	40	T:0.925	C:0.075	
chr28	11735469	2	38	T:1	G:0	
chr28	11735580	2	38	TC:1	T:0	
chr28	11735631	2	38	T:1	G:0	
chr28	11735638	2	38	T:1	A:0	
chr28	11735692	2	40	T:0.575	A:0.425	
chr28	11735701	2	40	TAATGTCACTAAGTTTTG:1	T:0	
chr28	11735782	2	36	C:1	T:0	
chr28	11735932	2	36	A:1	C:0	
chr28	11736011	2	36	T:1	G:0	
chr28	11736058	3	38	T:0.868421	TA:0.131579	TAA:0
chr28	11736124	2	40	G:1	A:0	
chr28	11736154	2	40	G:1	A:0	
chr28	11736271	2	40	A:1	G:0	
chr28	11736327	2	40	T:1	C:0	
chr28	11736399	2	40	T:0.475	C:0.525	
chr28	11736492	2	38	G:1	A:0	
chr28	11736637	2	40	T:1	A:0	
chr28	11736659	2	40	A:1	T:0	
chr28	11736793	2	36	A:0.916667	G:0.0833333	
chr28	11736832	2	36	T:1	G:0	
chr28	11736989	2	40	C:0.975	A:0.025	
chr28	11737047	2	40	G:0.45	A:0.55	
chr28	11737114	2	40	A:0.85	G:0.15	
chr28	11737167	2	40	A:0.575	C:0.425	
chr28	11737431	2	40	T:0.95	G:0.05	
chr28	11737474	2	40	C:0.925	T:0.075	
chr28	11737555	2	38	C:0.973684	T:0.0263158	
chr28	11737609	2	40	T:0.75	G:0.25	
chr28	11737704	2	40	C:1	T:0	
chr28	11737793	2	36	A:1	G:0	

chr28 11737835 2 40 C:0.675 T:0.325
chr28 11737943 2 38 T:0.921053 C:0.0789474
chr28 11737988 2 34 C:0.911765 A:0.0882353
chr28 11738674 2 38 A:0.921053 G:0.0789474
chr28 11738714 2 38 A:0.894737 G:0.105263
chr28 11738894 2 38 T:0.684211 C:0.315789
chr28 11738992 2 36 G:0.972222 C:0.0277778
chr28 11739053 2 40 C:0.925 T:0.075
chr28 11739304 2 38 G:0.921053 A:0.0789474
chr28 11739353 2 40 C:0.925 CTG:0.075
chr28 11739355 4 40 C:0.85 G:0.075 CTCTG:0.075
CTGTG:0
chr28 11739443 2 40 A:0.4 G:0.6
chr28 11739473 2 40 T:0.925 C:0.075
chr28 11739475 2 40 G:0.925 A:0.075
chr28 11739534 3 40 CCT:0.925 C:0.05 CCTCTCT:
0.025
chr28 11739570 2 40 A:0.925 G:0.075
chr28 11739571 3 40 TAAAAATTAAAAAAAAACTTAAAAAA:0.6
T:0.325 TAAAATTAAAAAAAAACTTAAAAAA:0.075
chr28 11739579 2 40 AAAAAAAAACTTAAAAAAAAAAATT:0.875
A:0.125
chr28 11739721 2 36 A:0.972222 AAG:0.0277778
chr28 11739753 2 36 G:0.694444 A:0.305556
chr28 11739762 2 38 A:0.894737 T:0.105263
chr28 11739763 2 38 G:0.894737 A:0.105263
chr28 11739831 2 40 T:0.925 C:0.075
chr28 11739926 2 38 G:0.842105 T:0.157895
chr28 11740697 2 38 A:0.842105 C:0.157895
chr28 11740934 2 40 T:0.85 A:0.15
chr28 11741024 2 38 C:0.921053 T:0.0789474
chr28 11741241 2 40 C:0.975 T:0.025
chr28 11742019 2 36 G:0.916667 A:0.0833333
chr28 11742044 2 38 T:0.447368 G:0.552632
chr28 11742213 2 40 T:0.925 C:0.075
chr28 11742283 2 40 C:0.95 T:0.05
chr28 11742362 2 40 T:1 C:0
chr28 11742433 2 36 C:0.972222 G:0.0277778
chr28 11742883 2 40 G:0.975 A:0.025
chr28 11743015 2 38 A:1 C:0
chr28 11743065 2 40 G:0.75 A:0.25
chr28 11743074 2 40 C:0.95 T:0.05
chr28 11743156 2 38 C:0.973684 T:0.0263158
chr28 11743195 2 38 A:0.947368 C:0.0526316
chr28 11743291 2 40 G:0.6 A:0.4
chr28 11743363 2 40 T:0.9 C:0.1
chr28 11743412 2 38 T:0.894737 C:0.105263
chr28 11743534 2 38 C:0.605263 T:0.394737
chr28 11743595 2 40 T:0.6 C:0.4
chr28 11743633 2 40 C:0.925 T:0.075
chr28 11743739 2 40 C:0.625 T:0.375
chr28 11743856 2 34 C:0.588235 T:0.411765
chr28 11743871 2 34 C:0.352941 G:0.647059
chr28 11744902 2 38 T:1 C:0

chr28	11745129	2	40	G:0.975	A:0.025
chr28	11745150	2	38	C:0.921053	T:0.0789474
chr28	11745153	2	38	G:0.578947	A:0.421053
chr28	11745162	2	38	A:0.921053	C:0.0789474
chr28	11745296	2	38	G:0.605263	A:0.394737
chr28	11745342	2	38	C:0.894737	CT:0.105263
chr28	11745693	2	40	A:0.925	T:0.075
chr28	11745711	2	40	G:0.6	A:0.4
chr28	11745848	2	38	A:0.578947	C:0.421053
chr28	11745895	2	40	C:0.7	G:0.3
chr28	11746260	2	38	C:0.921053	A:0.0789474
chr28	11746288	2	36	A:0.611111	ATCT:0.388889
chr28	11746398	2	40	C:0.65	T:0.35
chr28	11746450	2	40	G:0.65	A:0.35
chr28	11746455	2	40	C:0.65	T:0.35
chr28	11746530	2	40	A:0.6	G:0.4
chr28	11746608	2	40	C:0.95	T:0.05
chr28	11746624	2	40	T:0.55	C:0.45
chr28	11746672	2	40	T:0.9	C:0.1
chr28	11746701	2	40	A:0.65	G:0.35
chr28	11746769	2	40	T:0.925	TAGAC:0.075
chr28	11746774	2	40	A:0.6	C:0.4
chr28	11746779	2	40	T:0.525	C:0.475
chr28	11746815	2	40	G:0.925	GC:0.075
chr28	11746863	2	40	T:0.6	G:0.4
chr28	11747207	2	36	T:1	C:0
chr28	11747361	2	40	T:0.65	C:0.35
chr28	11747383	2	40	C:0.65	T:0.35
chr28	11747404	2	40	A:0.675	C:0.325
chr28	11747492	2	40	T:0.7	C:0.3
chr28	11747632	2	40	G:0.95	T:0.05
chr28	11747678	2	40	A:0.925	G:0.075
chr28	11747686	2	40	C:1	T:0
chr28	11747705	2	40	G:0.9	A:0.1
chr28	11747918	2	40	C:0.925	T:0.075
chr28	11748026	2	40	A:0.9	G:0.1
chr28	11748041	2	40	T:0.9	C:0.1
chr28	11748103	2	38	C:0.894737	A:0.105263
chr28	11748179	2	40	G:0.925	A:0.075
chr28	11748287	2	40	T:1	A:0
chr28	11748536	2	40	A:0.9	C:0.1
chr28	11748543	2	40	TA:1	T:0
chr28	11748559	2	40	G:1	T:0
chr28	11748669	2	38	A:0.921053	T:0.0789474
chr28	11748694	2	38	C:0.921053	T:0.0789474
chr28	11748695	2	38	T:0.894737	A:0.105263
chr28	11748760	2	40	G:0.95	T:0.05
chr28	11748768	2	40	A:0.95	T:0.05
chr28	11748817	2	40	C:0.975	T:0.025
chr28	11748826	2	40	T:0.95	C:0.05
chr28	11749230	2	38	TC:0.894737	T:0.105263
chr28	11749346	2	36	A:0.444444	T:0.555556
chr28	11749371	2	40	T:0.9	TGTC:0.1
chr28	11749403	2	38	T:0.894737	C:0.105263

chr28	11749624	2	40	AT:0.925	A:0.075
chr28	11749767	2	40	C:0.975	T:0.025
chr28	11750075	2	40	G:0.9	T:0.1
chr28	11750108	2	36	C:0.972222	T:0.0277778
chr28	11750118	2	36	G:0.972222	A:0.0277778
chr28	11750298	2	38	A:0.921053	T:0.0789474
chr28	11750397	2	40	G:0.9	T:0.1
chr28	11750477	2	40	A:0.925	C:0.075
chr28	11750496	2	40	T:0.9	G:0.1
chr28	11750714	2	36	C:0.944444	T:0.0555556
chr28	11750793	2	40	T:0.925	G:0.075
chr28	11751106	2	36	C:1	T:0
chr28	11751221	2	40	AACTC:0.975	A:0.025
chr28	11751359	2	38	C:0.894737	T:0.105263
chr28	11751412	2	40	A:0.9	G:0.1
chr28	11751632	2	38	C:0.921053	G:0.0789474
chr28	11751640	2	40	C:1	A:0
chr28	11751707	2	40	G:0.925	C:0.075
chr28	11751732	2	38	A:0.947368	G:0.0526316
chr28	11751741	2	38	A:0.973684	G:0.0263158
chr28	11751847	2	38	C:0.973684	CT:0.0263158
chr28	11751848	2	36	T:0.944444	C:0.0555556
chr28	11752068	2	40	G:0.9	A:0.1
chr28	11752096	2	38	T:0.894737	A:0.105263
chr28	11752211	2	36	G:0.944444	A:0.0555556
chr28	11752340	2	40	A:0.9	G:0.1
chr28	11752566	2	38	TGTTTTTTTG:1	T:0
chr28	11752614	2	38	ATTTATTT:0.605263	A: 0.394737
chr28	11752954	2	36	T:1	C:0
chr28	11752959	2	32	T:0	TA:1
chr28	11752992	2	34	T:0	C:1
chr28	11752993	2	34	G:0	T:1
chr28	11752996	2	34	TA:0	T:1
chr28	11753001	2	34	A:0	AT:1
chr28	11753011	2	34	C:0	T:1
chr28	11753023	2	34	C:0	CT:1
chr28	11753061	2	38	T:0	C:1
chr28	11753063	2	38	G:0	T:1
chr28	11753067	2	38	A:0	AT:1
chr28	11753073	2	38	C:0	CCTAA:1
chr28	11753075	2	38	G:0	A:1
chr28	11753077	2	38	ATAT:0	A:1
chr28	11753083	2	38	G:0	GT:1
chr28	11753088	2	38	ATT:0	A:1
chr28	11753192	2	38	C:0.921053	T:0.0789474
chr28	11753196	2	38	T:0.947368	C:0.0526316
chr28	11753328	2	40	G:0.5	A:0.5
chr28	11753366	2	38	C:0.894737	G:0.105263
chr28	11753433	2	40	T:0.575	G:0.425
chr28	11753690	2	40	C:0.975	T:0.025
chr28	11753833	2	40	A:0.9	G:0.1
chr28	11753862	2	40	TTA:0.9	T:0.1
chr28	11753866	2	40	T:0.9	TCA:0.1

chr28	11753971	2	40	T:0.9	A:0.1	
chr28	11753989	2	40	C:0.9	T:0.1	
chr28	11754013	2	40	G:0.9	A:0.1	
chr28	11754054	2	36	C:0.944444	T:0.0555556	
chr28	11754316	2	40	C:1	T:0	
chr28	11754371	2	34	T:0.911765	G:0.0882353	
chr28	11754373	2	34	C:0.470588	T:0.529412	
chr28	11754464	2	36	A:1	G:0	
chr28	11754469	2	36	G:0.888889	A:0.111111	
chr28	11754654	2	40	G:0.925	A:0.075	
chr28	11754906	2	32	A:0.90625	G:0.09375	
chr28	11755001	2	14	G:0.5	A:0.5	
chr28	11755008	2	14	G:0.714286	A:0.285714	
chr28	11755023	2	14	AG:0.714286	A:0.285714	
chr28	11755177	2	38	A:0.473684	C:0.526316	
chr28	11755253	2	36	C:0.888889	A:0.111111	
chr28	11755303	2	36	T:0.944444	C:0.0555556	
chr28	11755430	2	40	T:0.925	TTTGTA:0.075	
chr28	11755523	2	40	AT:0.9	A:0.1	
chr28	11755599	2	38	A:1	G:0	
chr28	11755688	2	38	G:0.894737	A:0.105263	
chr28	11755801	2	38	T:1	C:0	
chr28	11755835	2	40	TCAGTCCC:0.9	T:0.1	
chr28	11755867	2	40	G:0.9	C:0.1	
chr28	11755935	2	40	C:0.925	T:0.075	
chr28	11756119	2	38	T:0.894737	C:0.105263	
chr28	11756130	2	40	T:0.925	C:0.075	
chr28	11756251	2	38	C:0.921053	T:0.0789474	
chr28	11756326	2	38	C:0.921053	G:0.0789474	
chr28	11756376	2	38	G:0.921053	A:0.0789474	
chr28	11756482	2	38	C:0.921053	CT:0.0789474	
chr28	11756520	2	36	T:1	C:0	
chr28	11756606	2	40	T:0.925	C:0.075	
chr28	11756788	2	38	C:1	A:0	
chr28	11756847	2	38	A:1	G:0	
chr28	11756853	2	36	A:0.888889	G:0.111111	
chr28	11756973	2	38	G:0.868421	T:0.131579	
chr28	11757038	2	38	A:1	G:0	
chr28	11757176	2	40	T:1	C:0	
chr28	11757276	2	40	A:1	C:0	
chr28	11757417	2	40	TG:0.9	T:0.1	
chr28	11757428	2	40	G:0.875	A:0.125	
chr28	11757535	2	38	T:0.894737	C:0.105263	
chr28	11757544	2	38	G:0.894737	A:0.105263	
chr28	11757665	2	40	G:1	T:0	
chr28	11757782	2	38	G:0.973684	A:0.0263158	
chr28	11757820	2	40	C:0.95	T:0.05	
chr28	11757837	2	40	T:0.925	A:0.075	
chr28	11757933	2	40	C:0.85	T:0.15	
chr28	11757943	2	40	A:0.95	G:0.05	
chr28	11758254	2	38	C:0.894737	T:0.105263	
chr28	11758292	2	38	TA:0.894737	T:0.105263	
chr28	11758421	2	38	A:0.921053	G:0.0789474	
chr28	11758542	7	40	TTATATATA:0.3	T:0.025	TTA:

0.025 TTATA:0.05 TTATATA:0.45 TTATATATATA:0.1
TTATATATATATA:0.05

chr28	11758583	2	38	G:0.973684	A:0.0263158	
chr28	11758654	2	34	A:0.941176	G:0.0588235	
chr28	11758731	2	34	AC:0.911765	A:0.0882353	
chr28	11758736	2	34	A:0.911765	AG:0.0882353	
chr28	11758846	2	32	AC:1	A:0	
chr28	11759155	2	36	A:0.888889	G:0.111111	
chr28	11759257	2	32	AG:0.90625	A:0.09375	
chr28	11759580	2	40	G:1	GTA:0	
chr28	11759626	2	40	T:1	TGTA:0	
chr28	11759633	3	40	T:0.9	TAGATTAGA:0.075	TAGATTAGATAGATAGATAGATAGA:0.025
chr28	11759650	2	40	T:1	TAG:0	
chr28	11759652	2	40	T:1	TAGA:0	
chr28	11759654	2	40	G:1	A:0	
chr28	11759656	2	40	C:1	A:0	
chr28	11759659	2	40	T:1	G:0	
chr28	11759663	2	40	T:1	G:0	
chr28	11759666	2	40	A:1	ATTGG:0	
chr28	11759678	2	40	CATTG:1	C:0	
chr28	11759687	2	40	TATGTG:1	T:0	
chr28	11759696	2	40	G:0.95	A:0.05	
chr28	11759783	2	38	T:0.894737	C:0.105263	
chr28	11759800	2	40	C:1	T:0	
chr28	11759894	2	40	AT:0.9	A:0.1	
chr28	11759944	2	40	T:1	C:0	
chr28	11760019	2	40	C:0.975	CA:0.025	
chr28	11760300	2	38	C:1	T:0	
chr28	11760339	2	40	A:0.9	G:0.1	
chr28	11760449	2	40	C:0.975	T:0.025	
chr28	11760463	2	40	C:0.975	T:0.025	
chr28	11760472	2	40	A:0.975	T:0.025	
chr28	11760557	2	38	T:0.894737	C:0.105263	
chr28	11760585	2	40	A:0.975	G:0.025	
chr28	11760624	2	40	T:0.925	C:0.075	
chr28	11760663	2	40	C:1	CTTTT:0	
chr28	11760665	2	40	T:0.95	TTTC:0.05	
chr28	11760699	3	40	C:0.775	G:0.125	CAG:0.1
chr28	11760752	2	38	C:1	T:0	
chr28	11760779	2	36	C:0.916667	A:0.0833333	
chr28	11760781	2	36	C:1	T:0	
chr28	11760813	2	38	C:0.921053	T:0.0789474	
chr28	11760906	2	40	A:0.875	G:0.125	
chr28	11760940	2	40	C:0.875	T:0.125	
chr28	11761008	2	38	C:1	T:0	
chr28	11761240	2	36	G:0.888889	A:0.111111	
chr28	11761340	2	40	A:0.875	G:0.125	
chr28	11761480	2	38	C:1	T:0	
chr28	11761669	2	40	C:1	A:0	
chr28	11761738	2	40	T:0.9	C:0.1	
chr28	11761758	2	40	T:1	TAATAATCATTTTAAAA:0	
chr28	11761798	2	40	CAT:1	C:0	
chr28	11762033	2	38	C:1	T:0	

chr28 11762488 2 40 G:1 A:0
chr28 11762505 2 40 A:1 G:0
chr28 11762679 2 40 CCA:0.925 C:0.075
chr28 11762724 2 38 A:1 G:0
chr28 11762764 4 40 TACACACACACAC:0.75 T:0.125
TACACACACACACAC:0.05 TACACACACACACACAC:0.075
chr28 11762802 2 40 C:1 T:0
chr28 11762821 2 38 G:0.894737 A:0.105263
chr28 11762873 2 40 G:1 A:0
chr28 11763027 2 40 A:0.925 ACT:0.075
chr28 11763144 2 40 A:1 C:0
chr28 11763213 2 40 A:0.9 C:0.1
chr28 11763216 2 40 T:1 A:0
chr28 11763384 2 40 G:1 T:0
chr28 11763486 2 36 T:0.916667 A:0.0833333
chr28 11763540 2 38 C:0.421053 A:0.578947
chr28 11763601 2 38 C:0.894737 G:0.105263
chr28 11763663 2 40 TTTC:0.975 T:0.025
chr28 11763713 2 36 G:0.611111 C:0.388889
chr28 11763777 2 38 G:1 A:0
chr28 11764044 2 38 A:1 G:0
chr28 11764060 2 38 T:0.921053 G:0.0789474
chr28 11764519 2 40 T:1 C:0
chr28 11764552 2 40 G:1 A:0
chr28 11764590 2 34 TG:0.558824 T:0.441176
chr28 11764592 2 38 GTTT:0.973684 G:0.0263158
chr28 11764890 2 36 T:1 TAAG:0
chr28 11765223 2 40 AATC:0.975 A:0.025
chr28 11765369 2 40 C:0.975 A:0.025
chr28 11765425 2 40 C:0.9 T:0.1
chr28 11765464 2 40 A:1 ATTATTTTTTATTTTATTATTT:0
chr28 11765465 2 40 C:1 CATTAAATTATTTTAAT:0
chr28 11765466 2 40 C:1 T:0
chr28 11765518 2 38 T:1 C:0
chr28 11765627 2 36 C:0.916667 T:0.0833333
chr28 11765879 2 36 G:0.916667 T:0.0833333
chr28 11766056 2 36 A:1 C:0
chr28 11766099 2 34 A:0.558824 C:0.441176
chr28 11766106 2 36 T:1 G:0
chr28 11766189 2 36 A:1 T:0
chr28 11766254 2 38 A:0.578947 G:0.421053
chr28 11766328 4 40 AAGAGAGAG:0.425 A:0.075 AAGAGAG:
0.5 AAGAGAGAGAGAGAG:0
chr28 11766490 2 40 CTTTTTTTTTTTT:0.7 C:0.3
chr28 11766584 2 34 C:0.970588 T:0.0294118
chr28 11766717 2 38 A:1 G:0
chr28 11767154 2 38 G:0.973684 T:0.0263158
chr28 11767267 2 38 G:1 A:0
chr28 11767311 2 36 A:0.472222 G:0.527778
chr28 11767397 2 38 C:1 T:0
chr28 11767479 2 38 A:1 G:0
chr28 11767489 2 38 G:0.394737 A:0.605263
chr28 11767576 2 34 A:0.617647 T:0.382353
chr28 11767711 2 38 T:1 C:0

chr28	11767900	2	40	T:0.925	C:0.075
chr28	11768021	2	40	G:0.475	GGAAA:0.525
chr28	11768093	2	40	T:1	C:0
chr28	11768261	2	38	CA:0.5	C:0.5
chr28	11768466	2	38	C:1	G:0
chr28	11768605	2	40	G:1	A:0
chr28	11768624	2	38	A:0.868421	G:0.131579
chr28	11768768	2	40	T:1	C:0
chr28	11768919	2	40	T:0.4	A:0.6
chr28	11768996	2	36	A:1	G:0
chr28	11769101	2	38	C:0.973684	CTA:0.0263158
chr28	11769122	2	38	A:1	C:0
chr28	11769341	2	40	C:1	A:0
chr28	11769666	2	40	TGGCTTTCCCAAGTAA:1	T:0
chr28	11769689	2	40	C:0.925	A:0.075
chr28	11769865	2	38	A:0.526316	G:0.473684
chr28	11770022	2	40	G:0.925	C:0.075
chr28	11770722	2	40	A:0.525	G:0.475
chr28	11770739	2	38	TG:0.894737	T:0.105263
chr28	11771238	2	40	C:1	CTTA:0
chr28	11771240	2	40	T:0.45	TATG:0.55
chr28	11771308	2	36	T:1	C:0
chr28	11771840	2	36	C:0.916667	A:0.0833333
chr28	11772429	2	38	G:0.947368	C:0.0526316
chr28	11772502	2	40	T:0.975	G:0.025
chr28	11772564	2	40	T:0.525	C:0.475
chr28	11772595	2	40	AT:0.7	A:0.3
chr28	11772603	2	40	T:1	A:0
chr28	11772715	2	38	C:0.631579	A:0.368421
chr28	11773090	2	36	A:0.611111	C:0.388889
chr28	11773354	2	38	G:0.631579	A:0.368421
chr28	11773427	2	36	A:0.972222	G:0.0277778
chr28	11774005	2	38	A:0.657895	C:0.342105
chr28	11774216	2	40	C:1	T:0
chr28	11774403	2	38	A:0.710526	G:0.289474
chr28	11774797	2	38	A:0.631579	T:0.368421
chr28	11774834	2	40	G:0.675	A:0.325
chr28	11775084	2	38	A:0.684211	G:0.315789
chr28	11775145	2	38	C:0.973684	T:0.0263158
chr28	11775177	2	40	GAATT:1	G:0
chr28	11775270	2	38	A:1	G:0
chr28	11775358	2	36	C:0.666667	T:0.333333
chr28	11775428	2	38	A:0.631579	G:0.368421
chr28	11775447	2	38	G:0.631579	T:0.368421
chr28	11775501	2	34	A:1	G:0
chr28	11775605	2	36	G:0.666667	T:0.333333
chr28	11775627	2	36	T:0.666667	C:0.333333
chr28	11775871	2	38	A:0.605263	AC:0.394737
chr28	11775945	2	40	A:0.725	G:0.275
chr28	11775964	2	40	A:0.725	T:0.275
chr28	11776095	2	40	A:0.95	ATT:0.05
chr28	11776097	2	40	T:0.95	TTTAAAAG:0.05
chr28	11776100	2	40	A:0.95	AGTGATT:0.05
chr28	11776103	2	40	A:0.95	AAAGACAGTG:0.05

chr28 11776147 2 38 C:0.684211 T:0.315789
chr28 11776163 2 38 G:0.657895 A:0.342105
chr28 11776184 2 38 G:0.973684 A:0.0263158
chr28 11776207 2 40 C:0.675 G:0.325
chr28 11776297 2 36 T:0.666667 C:0.333333
chr28 11776433 2 40 T:0.7 C:0.3
chr28 11776447 2 38 A:0.657895 G:0.342105
chr28 11777017 2 38 C:0.5 T:0.5
chr28 11777084 2 40 A:0.95 G:0.05
chr28 11777250 2 36 G:0.611111 A:0.388889
chr28 11777374 2 38 C:0.631579 T:0.368421
chr28 11777375 2 38 A:0.631579 G:0.368421
chr28 11777393 2 38 GTCGAGGAATGTTCCCTCTATC:0.631579
G:0.368421
chr28 11777488 2 36 G:0.583333 A:0.416667
chr28 11777788 2 40 C:1 G:0
chr28 11777938 2 36 C:0.611111 T:0.388889
chr28 11777976 2 32 TA:0.59375 T:0.40625
chr28 11778017 2 34 GC:0.970588 G:0.0294118
chr28 11778027 2 34 C:1 CAA:0
chr28 11778028 3 36 C:0.5 A:0.416667 CAA:
0.0833333
chr28 11778091 4 38 CATTTTATTTTATTTTATTTT:0.710526
C:0.0526316 CATTTTATTTT:0.0526316 CATTTTATTTTATTTT:0.184211
chr28 11778217 2 36 C:0.972222 T:0.0277778
chr28 11778246 2 40 C:0.65 T:0.35
chr28 11778341 2 36 A:1 G:0
chr28 11778413 2 36 A:1 G:0
chr28 11778532 2 40 G:0.925 A:0.075
chr28 11778575 2 36 A:1 C:0
chr28 11778602 2 38 T:0.684211 G:0.315789
chr28 11778783 2 40 C:1 T:0
chr28 11778821 2 36 GCC:0.555556 G:0.444444
chr28 11778825 2 36 C:1 T:0
chr28 11779002 2 38 CATA:0.973684 C:0.0263158
chr28 11779171 2 38 G:0.973684 A:0.0263158
chr28 11779249 2 36 G:0.694444 GAA:0.305556
chr28 11779250 3 38 G:0.5 A:0.342105 GA:
0.157895
chr28 11779353 2 38 C:0.947368 T:0.0526316
chr28 11779410 2 38 T:0.973684 C:0.0263158
chr28 11779573 2 38 C:0.552632 T:0.447368
chr28 11779590 2 38 C:0.631579 T:0.368421
chr28 11780213 2 40 C:0.925 T:0.075
chr28 11780473 2 40 C:0.75 T:0.25
chr28 11780645 2 38 A:0.921053 G:0.0789474
chr28 11780700 2 40 A:0.825 T:0.175
chr28 11780841 2 38 C:0.921053 T:0.0789474
chr28 11780901 2 40 G:0.925 A:0.075
chr28 11781118 2 38 C:0.921053 A:0.0789474
chr28 11781195 2 38 A:0.5 C:0.5
chr28 11781246 2 38 T:0.921053 G:0.0789474
chr28 11781633 2 40 G:0.5 A:0.5
chr28 11781671 2 40 T:0.975 C:0.025

chr28 11781690 2 38 A:0.789474 AT:0.210526
chr28 11782027 2 38 G:1 C:0
chr28 11782187 2 40 A:0.925 T:0.075
chr28 11782207 2 40 A:0.925 G:0.075
chr28 11782276 2 40 G:0.925 A:0.075
chr28 11782293 2 40 G:0.8 A:0.2
chr28 11782738 2 40 T:0.925 TGGGTCCCTGGGCTC:0.075
chr28 11782739 2 40 AC:0.925 A:0.075
chr28 11782818 2 38 A:0.947368 G:0.0526316
chr28 11782888 2 36 T:0.916667 A:0.0833333
chr28 11783074 2 38 C:0.947368 CCT:0.0526316
chr28 11783132 3 38 A:0.657895 AAC:0.342105
AACAC:0
chr28 11783381 2 40 A:0.75 C:0.25
chr28 11783394 2 40 G:0.95 A:0.05
chr28 11783522 2 38 A:0.552632 C:0.447368
chr28 11783544 2 38 A:0.447368 G:0.552632
chr28 11783653 2 38 C:0.921053 T:0.0789474
chr28 11783726 2 40 T:0.9 G:0.1
chr28 11783784 2 38 G:1 A:0
chr28 11783812 4 40 T:0.75 TG:0.075 TGGC:0.025
TGGCG:0.15
chr28 11783861 2 38 AT:0.631579 A:0.368421
chr28 11784019 2 40 G:0.675 A:0.325
chr28 11784285 2 38 T:0.921053 A:0.0789474
chr28 11784421 2 40 A:0.95 T:0.05
chr28 11784430 2 40 AT:0.775 A:0.225
chr28 11784451 2 38 T:0.421053 G:0.578947
chr28 11784754 2 38 G:0.973684 A:0.0263158
chr28 11784868 2 40 G:0.925 A:0.075
chr28 11785165 2 40 C:0.95 T:0.05
chr28 11785291 2 40 A:0.95 C:0.05
chr28 11785317 2 40 C:0.675 CT:0.325
chr28 11785352 2 40 G:0.675 A:0.325
chr28 11785677 2 38 G:0.815789 T:0.184211
chr28 11786110 2 38 C:1 T:0
chr28 11786350 2 38 A:0.657895 G:0.342105
chr28 11786469 2 36 C:0.944444 A:0.0555556
chr28 11786795 2 38 T:0.684211 C:0.315789
chr28 11787108 3 40 C:0.775 CT:0.2 CTT:0.025
chr28 11787247 2 40 G:0.875 A:0.125
chr28 11787410 2 40 T:0.725 C:0.275
chr28 11787613 2 40 T:0.925 G:0.075
chr28 11787775 2 40 A:0.575 T:0.425
chr28 11787811 2 40 AT:0.9 A:0.1
chr28 11787840 2 38 C:0.710526 T:0.289474
chr28 11788062 4 40 CCT:0.75 C:0.025 CCTCT:0.075
CCTCTCT:0.15
chr28 11788087 3 40 C:0.85 CTG:0.15 CTCTG:0
chr28 11788253 2 36 G:0.861111 A:0.138889
chr28 11788301 2 34 A:1 G:0
chr28 11788394 2 38 G:1 A:0
chr28 11788490 2 38 C:0.921053 G:0.0789474
chr28 11788587 2 40 A:0.55 G:0.45

chr28 11788962 2 36 CACA:1 C:0
chr28 11788964 2 40 C:0.85 T:0.15
chr28 11788967 7 40 AAAAT:0.75 A:0 TAAAT:
0.075 AAAATAAAT:0 AAAATAAATAAAT:0.025 ATAAAT:0.15
AAAATAAATAAATAAAT:0
chr28 11789007 4 40 T:0.6 A:0.15 TAAAA:0.1
TAAATAAAA:0.15
chr28 11789017 2 36 T:0.888889 A:0.111111
chr28 11789228 2 36 CAG:0.833333 C:0.166667
chr28 11789273 2 38 G:0.894737 A:0.105263
chr28 11789401 2 38 C:0.921053 CA:0.0789474
chr28 11789780 2 40 C:1 T:0
chr28 11789807 2 40 C:0.675 T:0.325
chr28 11789860 2 38 A:0.657895 T:0.342105
chr28 11789949 2 40 G:1 GA:0
chr28 11790376 2 40 G:0.925 T:0.075
chr28 11790436 2 40 A:1 C:0
chr28 11790441 2 40 G:1 A:0
chr28 11790546 2 38 G:0.447368 A:0.552632
chr28 11790555 2 38 C:0.578947 A:0.421053
chr28 11791122 2 40 C:0.925 T:0.075
chr28 11791647 2 40 T:0.5 C:0.5
chr28 11791873 2 38 T:0.184211 C:0.815789
chr28 11791900 2 40 C:0.9 T:0.1
chr28 11793201 2 40 C:0.875 T:0.125
chr28 11793581 2 38 C:1 T:0
chr28 11793605 2 40 G:1 A:0
chr28 11793750 2 38 A:0.894737 AAG:0.105263
chr28 11793873 2 40 A:0.5 C:0.5
chr28 11793901 2 40 AT:0.875 A:0.125
chr28 11794282 2 36 T:1 C:0
chr28 11794300 2 36 G:0.944444 T:0.0555556
chr28 11794318 2 38 G:0.921053 T:0.0789474
chr28 11794971 2 38 G:1 A:0
chr28 11794979 2 38 G:0.473684 A:0.526316
chr28 11795991 2 40 C:1 T:0
chr28 11796018 3 40 T:0.725 TG:0.125 TTG:0.15
chr28 11796027 2 40 G:0.725 GGA:0.275
chr28 11796042 2 40 T:1 C:0
chr28 11796175 2 40 G:1 A:0
chr28 11796203 2 36 C:1 T:0
chr28 11796322 2 40 C:0.875 G:0.125
chr28 11796550 2 38 T:0.894737 C:0.105263
chr28 11796653 2 38 G:0.973684 T:0.0263158
chr28 11796680 2 38 A:0.868421 C:0.131579
chr28 11796783 3 36 TA:0.75 T:0.0833333 TAA:
0.166667
chr28 11796785 2 40 A:1 T:0
chr28 11796954 2 40 T:1 TATAA:0
chr28 11796955 4 40 CTAAA:0.55 C:0.4 ATAAA:0
CTAAATAAA:0.05
chr28 11797048 3 38 GAA:0.5 G:0.368421 GA:
0.131579
chr28 11797348 2 38 C:1 T:0

chr28	11797612	2	40	C:0.95	G:0.05		
chr28	11797870	2	40	TA:0.975	T:0.025		
chr28	11797886	2	40	C:0.9	CT:0.1		
chr28	11797924	2	38	C:0.894737	G:0.105263		
chr28	11797926	2	38	C:0.894737	G:0.105263		
chr28	11797930	3	38	AAG:0.710526	A:0.184211	GAG:0.105263	
chr28	11798052	2	38	C:0.894737	T:0.105263		
chr28	11798176	2	36	GA:1	G:0		
chr28	11798392	2	38	T:0.842105	C:0.157895		
chr28	11798395	2	38	C:0.842105	T:0.157895		
chr28	11798517	2	40	AAATG:0.625	A:0.375		
chr28	11798537	2	40	GAATGAATA:1	G:0		
chr28	11798545	2	40	A:0.95	AATG:0.05		
chr28	11798566	2	38	A:0.842105	C:0.157895		
chr28	11798574	2	40	A:1	AAG:0		
chr28	11799037	2	40	T:0.875	C:0.125		
chr28	11799055	2	40	T:0.75	G:0.25		
chr28	11799202	2	36	C:0.972222	G:0.0277778		
chr28	11799343	2	38	C:0.921053	T:0.0789474		
chr28	11799356	2	38	C:0.921053	T:0.0789474		
chr28	11799383	2	28	C:0.857143	T:0.142857		
chr28	11799462	2	40	A:0.525	G:0.475		
chr28	11799673	2	38	C:1	T:0		
chr28	11799848	2	38	C:0.815789	T:0.184211		
chr28	11799928	2	38	C:0.842105	CCT:0.157895		
chr28	11799947	2	40	C:0.875	G:0.125		
chr28	11800055	2	40	T:0.7	C:0.3		
chr28	11800064	2	40	TC:0.7	T:0.3		
chr28	11800122	2	40	T:0.6	C:0.4		
chr28	11800129	2	40	T:1	G:0		
chr28	11800151	2	36	C:1	A:0		
chr28	11800187	2	36	T:1	C:0		
chr28	11800210	2	38	G:0.973684	A:0.0263158		
chr28	11800343	4	36	CCTCTCTCTCTCT:0.722222	C:0.0277778	CCTCT:0.111111	CCTCTCT:0.138889
chr28	11800516	2	36	T:0.722222	A:0.277778		
chr28	11800593	2	34	A:0.705882	C:0.294118		
chr28	11800604	2	34	AG:0.970588	A:0.0294118		
chr28	11800720	2	38	A:0.947368	G:0.0526316		
chr28	11800826	2	38	G:0.526316	A:0.473684		
chr28	11800827	2	38	T:1	C:0		
chr28	11800915	2	40	T:0.475	G:0.525		
chr28	11801101	2	38	T:0.657895	A:0.342105		
chr28	11801371	2	38	G:0.684211	A:0.315789		
chr28	11801386	2	38	A:0.736842	G:0.263158		
chr28	11801464	2	38	A:1	C:0		
chr28	11801555	2	38	G:1	A:0		
chr28	11801669	2	38	C:0.894737	T:0.105263		
chr28	11801788	2	40	G:1	A:0		
chr28	11802005	2	38	G:0.947368	C:0.0526316		
chr28	11802011	2	38	G:0.947368	A:0.0526316		
chr28	11802016	2	38	T:0.710526	C:0.289474		
chr28	11802098	2	40	C:0.55	CT:0.45		

chr28	11802211	2	40	A:1	T:0		
chr28	11802315	2	40	GTTTTCTTTCT:0.95	G:0.05		
chr28	11802650	2	40	A:0.925	C:0.075		
chr28	11802676	2	40	C:0.975	T:0.025		
chr28	11802821	2	38	T:0.789474	C:0.210526		
chr28	11803773	4	40	CGT:0.65	C:0.1	CGTGT:0.25	CGTGTGTGTGT:0
chr28	11804481	2	38	T:0.657895	C:0.342105		
chr28	11804513	2	38	C:1	T:0		
chr28	11804562	2	40	C:1	T:0		
chr28	11804597	2	40	G:1	A:0		
chr28	11804796	2	40	G:1	A:0		
chr28	11804985	2	34	G:1	A:0		
chr28	11805043	2	38	C:0.710526	G:0.289474		
chr28	11805140	2	40	AC:0.7	A:0.3		
chr28	11805141	2	40	C:0.95	A:0.05		
chr28	11805286	2	38	A:0.578947	AGAG:0.421053		
chr28	11805554	2	38	C:0.947368	T:0.0526316		
chr28	11805609	2	40	G:0.7	T:0.3		
chr28	11805896	2	40	T:0.95	C:0.05		
chr28	11805899	2	40	A:1	G:0		
chr28	11806045	2	40	G:0.775	T:0.225		
chr28	11806146	2	40	C:0.65	CT:0.35		
chr28	11806176	2	40	CA:1	C:0		
chr28	11806298	2	40	A:0.65	G:0.35		
chr28	11806664	2	38	A:0.578947	T:0.421053		
chr28	11807120	2	40	TACC:0.675	T:0.325		
chr28	11807136	3	40	CT:0.65	C:0.35	CTT:0	
chr28	11807147	2	40	A:1	T:0		
chr28	11807307	2	40	CAA:0.925	C:0.075		
chr28	11807387	2	38	G:0.947368	T:0.0526316		
chr28	11807555	2	38	A:0.631579	T:0.368421		
chr28	11807626	2	36	A:0.694444	G:0.305556		
chr28	11807707	2	34	C:0.911765	T:0.0882353		
chr28	11807769	2	34	G:0.794118	A:0.205882		
chr28	11807828	2	34	A:0.735294	G:0.264706		
chr28	11808195	2	40	T:0.75	C:0.25		
chr28	11808348	2	40	C:0.725	T:0.275		
chr28	11808481	2	38	T:0.763158	C:0.236842		
chr28	11808638	2	40	C:1	T:0		
chr28	11808712	2	40	T:0.975	C:0.025		
chr28	11809063	2	40	T:0.625	C:0.375		
chr28	11809072	2	40	T:0.625	C:0.375		
chr28	11809151	2	40	C:0.875	T:0.125		
chr28	11809171	2	40	G:1	A:0		
chr28	11809177	2	38	A:0.578947	G:0.421053		
chr28	11809382	2	40	A:0.975	G:0.025		
chr28	11809535	2	40	C:0.95	T:0.05		
chr28	11809603	2	40	ATTC:0.725	A:0.275		
chr28	11809604	2	40	TTC:1	T:0		
chr28	11809605	2	40	TC:1	T:0		
chr28	11809606	2	40	C:1	CT:0		
chr28	11809614	2	40	T:0.95	TA:0.05		
chr28	11809622	2	40	T:0.95	C:0.05		

chr28	11809676	2	36	G:0.972222	A:0.0277778
chr28	11809724	2	36	C:0.861111	T:0.138889
chr28	11809857	2	40	A:0.875	G:0.125
chr28	11809963	2	36	A:0.888889	G:0.111111
chr28	11810177	2	38	T:0.763158	A:0.236842
chr28	11810265	2	38	G:0.736842	A:0.263158
chr28	11810439	2	40	G:1	C:0
chr28	11810719	2	40	T:0.925	C:0.075
chr28	11810788	2	40	T:0.7	C:0.3
chr28	11810820	2	40	C:0	A:1
chr28	11810822	2	40	T:0	G:1
chr28	11810869	2	38	C:0.947368	T:0.0526316
chr28	11810984	2	40	C:0.975	A:0.025
chr28	11811150	2	36	G:1	A:0
chr28	11811157	2	36	G:0.861111	A:0.138889
chr28	11811263	2	40	C:0.95	T:0.05
chr28	11811376	2	38	C:0.973684	T:0.0263158
chr28	11811557	2	40	T:1	G:0
chr28	11811873	3	38	CTT:0.605263	C:0.131579
CT:0.263158					
chr28	11811960	2	38	C:0.789474	T:0.210526
chr28	11812022	2	38	G:0.710526	T:0.289474
chr28	11812159	2	38	G:1	A:0
chr28	11812218	2	36	T:0.805556	A:0.194444
chr28	11812393	2	40	G:1	T:0
chr28	11812447	2	40	T:0.95	C:0.05
chr28	11812641	2	40	G:0.975	C:0.025
chr28	11812831	2	38	G:0.763158	A:0.236842
chr28	11812881	2	40	A:0.725	AAT:0.275
chr28	11813079	2	38	G:1	A:0
chr28	11813123	2	38	G:1	A:0
chr28	11813221	2	36	AC:1	A:0
chr28	11813276	2	36	T:0.777778	TA:0.222222
chr28	11813318	2	40	A:0.675	G:0.325
chr28	11813397	2	36	A:1	G:0
chr28	11813433	2	40	C:0.675	T:0.325
chr28	11813461	2	38	AAC:1	A:0
chr28	11813649	2	36	C:1	T:0
chr28	11813676	2	38	T:0.710526	TCGAGGTC:0.289474
chr28	11813870	2	40	G:1	GT:0
chr28	11813872	2	40	G:1	GT:0
chr28	11813873	2	40	G:1	GATA:0
chr28	11813877	2	40	G:1	GAAGTGAA:0
chr28	11813879	2	40	ATTC:1	A:0
chr28	11813883	2	40	T:1	G:0
chr28	11813898	2	40	GA:0.45	G:0.55
chr28	11813981	2	38	G:0.789474	A:0.210526
chr28	11813984	2	38	C:1	T:0
chr28	11814013	2	38	T:0.473684	C:0.526316
chr28	11814146	2	40	G:0.975	GA:0.025
chr28	11814337	2	38	TCTC:1	T:0
chr28	11814602	2	40	C:1	CA:0
chr28	11814736	2	40	A:1	C:0
chr28	11815053	2	38	A:0.947368	G:0.0526316

chr28	11815772	2	38	T:0.710526	A:0.289474	
chr28	11815788	2	38	G:0.526316	A:0.473684	
chr28	11816506	3	40	A:0.825	AAC:0.175	AACACACACAC:0
chr28	11816632	2	40	T:0.975	C:0.025	
chr28	11816655	2	40	C:0.95	T:0.05	
chr28	11816786	2	38	T:0.973684	C:0.0263158	
chr28	11816941	2	38	C:1	T:0	
chr28	11816942	2	38	A:0.736842	G:0.263158	
chr28	11816992	2	40	G:0.975	C:0.025	
chr28	11816998	2	40	C:0.975	T:0.025	
chr28	11817018	2	40	A:0.975	G:0.025	
chr28	11817080	2	38	C:1	T:0	
chr28	11817154	2	36	A:0.972222	G:0.0277778	
chr28	11817388	2	38	A:0.710526	G:0.289474	
chr28	11817437	2	38	T:0.973684	A:0.0263158	
chr28	11817467	2	38	T:0.973684	C:0.0263158	
chr28	11817485	2	38	C:0.973684	T:0.0263158	
chr28	11817814	2	40	C:0.975	T:0.025	
chr28	11817846	2	40	CTAATT:1	C:0	
chr28	11818123	2	40	C:0.975	T:0.025	
chr28	11818310	2	40	A:1	G:0	
chr28	11818731	2	38	C:0.973684	T:0.0263158	
chr28	11819259	2	40	T:0.975	C:0.025	
chr28	11819408	2	40	A:0.975	G:0.025	
chr28	11819651	2	40	G:0.975	A:0.025	
chr28	11819773	2	38	G:0.973684	C:0.0263158	
chr28	11819835	3	34	CAA:0.382353	C:0.470588	CA:0.147059
chr28	11819852	2	38	A:0.973684	G:0.0263158	
chr28	11819948	2	40	A:0.975	G:0.025	
chr28	11819970	2	40	GTTAACTTAAGTTAAGTTAACTTAAGGCTTAAGTTAAGTTA:0.475	G:0.525	
chr28	11820100	2	40	T:0.75	C:0.25	
chr28	11820249	2	38	C:0.868421	T:0.131579	
chr28	11820285	3	40	GATAAATAAATAA:0.275	G:0.15	GATAAATAA:0.575
chr28	11820434	2	40	T:0.975	C:0.025	
chr28	11820503	2	40	C:0.9	T:0.1	
chr28	11821191	2	38	G:0.868421	GA:0.131579	
chr28	11821468	2	40	T:1	TG:0	
chr28	11822074	2	40	A:0.475	T:0.525	
chr28	11822106	2	40	C:0.975	T:0.025	
chr28	11822620	2	36	G:0.777778	T:0.222222	
chr28	11823401	2	36	C:1	T:0	
chr28	11823549	2	40	T:0.575	C:0.425	
chr28	11823682	2	38	G:0.447368	A:0.552632	
chr28	11823745	2	40	A:0.975	G:0.025	
chr28	11824349	2	40	G:1	C:0	
chr28	11824401	2	40	G:0.4	A:0.6	
chr28	11824639	2	40	T:1	G:0	
chr28	11824717	2	40	G:0.975	T:0.025	
chr28	11825067	2	38	G:1	GC:0	
chr28	11825091	2	38	G:1	A:0	

chr28	11825265	2	38	G:0.473684	A:0.526316
chr28	11825271	2	36	T:0.694444	C:0.305556
chr28	11825284	2	38	C:0.473684	A:0.526316
chr28	11825581	2	38	A:0.973684	G:0.0263158
chr28	11825663	2	40	C:0.425	A:0.575
chr28	11825667	2	40	A:0.45	T:0.55
chr28	11825919	2	40	T:1	C:0
chr28	11826023	2	38	G:0.5	A:0.5
chr28	11826264	2	40	C:0.525	CA:0.475
chr28	11826297	2	40	C:1	T:0
chr28	11826375	2	40	C:0.45	A:0.55
chr28	11826420	2	38	C:0.473684	T:0.526316
chr28	11826445	2	40	G:0.55	C:0.45
chr28	11826491	2	38	G:0.973684	A:0.0263158
chr28	11826844	2	40	A:0.475	G:0.525
chr28	11826905	2	40	C:0.625	CTAAT:0.375
chr28	11826973	2	40	T:0.975	C:0.025
chr28	11827081	2	38	CAATAA:1	C:0
chr28	11827150	2	38	A:1	G:0
chr28	11827449	2	38	T:1	C:0
chr28	11827559	2	40	C:0.975	T:0.025
chr28	11827649	2	38	G:1	A:0
chr28	11827729	2	40	A:1	G:0
chr28	11827775	2	40	C:0.975	T:0.025
chr28	11827790	2	40	G:1	GAT:0
chr28	11827919	2	40	A:0.575	T:0.425
chr28	11827970	2	40	T:1	A:0
chr28	11827995	2	40	A:0.975	ATTATATG:0.025
chr28	11828110	2	40	C:1	A:0
chr28	11828111	2	40	G:0.95	A:0.05
chr28	11828210	2	40	G:0.75	A:0.25
chr28	11828230	2	40	T:1	TA:0
chr28	11828240	2	40	T:1	A:0
chr28	11828364	2	40	G:0.5	A:0.5
chr28	11828419	2	40	C:0.5	T:0.5
chr28	11828430	2	40	G:0.5	GT:0.5
chr28	11828459	2	40	C:0.5	T:0.5
chr28	11828495	2	40	C:0.45	T:0.55
chr28	11828545	2	40	C:0.6	T:0.4
chr28	11828579	2	40	T:0.525	C:0.475
chr28	11828585	2	40	GA:0.525	G:0.475
chr28	11828601	2	40	T:1	C:0
chr28	11828692	2	40	G:0.65	T:0.35
chr28	11828724	2	38	A:0.763158	G:0.236842
chr28	11828765	2	32	G:0.9375	A:0.0625
chr28	11828766	2	32	A:0.9375	G:0.0625
chr28	11830489	2	26	C:1	T:0
chr28	11830516	2	34	ATT:0.882353	A:0.117647
chr28	11830520	2	34	T:0.882353	TAAA:0.117647
chr28	11830549	2	34	TA:0.647059	T:0.352941
chr28	11830623	2	38	G:1	A:0
chr28	11830972	2	36	TTC:0.944444	T:0.0555556
chr28	11830989	2	36	TC:0.555556	T:0.444444
chr28	11831002	2	36	C:0.944444	T:0.0555556

chr28	11831044	2	36	A:1	G:0		
chr28	11831052	2	36	CT:1	C:0		
chr28	11831054	2	36	TA:0.944444	T:0.0555556		
chr28	11831158	2	40	A:0.5	AG:0.5		
chr28	11831197	2	38	G:0.5	A:0.5		
chr28	11831230	2	38	ACT:0.973684	A:0.0263158		
chr28	11831478	2	38	A:0.473684	G:0.526316		
chr28	11831512	2	40	AT:0.475	A:0.525		
chr28	11831519	2	40	C:0.5	T:0.5		
chr28	11831520	2	40	C:0.5	G:0.5		
chr28	11831564	2	40	A:0.525	T:0.475		
chr28	11831602	2	40	T:1	A:0		
chr28	11831672	2	40	A:0.45	G:0.55		
chr28	11831673	2	40	C:0.975	T:0.025		
chr28	11831689	2	40	ATACC:0.45	A:0.55		
chr28	11831695	2	40	GGAA:0.45	G:0.55		
chr28	11831743	2	38	A:0.447368	C:0.552632		
chr28	11831811	2	38	T:0.526316	C:0.473684		
chr28	11831902	2	40	C:0.375	T:0.625		
chr28	11831994	2	38	G:1	T:0		
chr28	11832056	2	40	G:1	A:0		
chr28	11832120	2	36	G:0.416667	A:0.583333		
chr28	11832161	2	40	C:0.5	T:0.5		
chr28	11832260	2	40	G:0.55	A:0.45		
chr28	11832317	2	38	G:0.552632	GA:0.447368		
chr28	11832396	2	38	A:1	G:0		
chr28	11832428	2	38	A:0.552632	G:0.447368		
chr28	11832479	2	40	C:0.45	A:0.55		
chr28	11832562	2	40	G:0.575	A:0.425		
chr28	11832586	2	40	T:0.55	C:0.45		
chr28	11832644	2	38	T:0.473684	C:0.526316		
chr28	11832742	5	40	ATTT:0.575	A:0.025	AT:0.025	ATT:0.1 ATTTT:0.275
chr28	11832909	2	38	A:0.5	G:0.5		
chr28	11832947	2	38	T:1	TA:0		
chr28	11832948	2	38	A:0.815789	T:0.184211		
chr28	11832949	2	38	G:0.526316	A:0.473684		
chr28	11833158	2	40	T:1	TTAAAACGTTTAAAAAAAAATAAAAAAAA:0		
chr28	11833277	2	40	C:0.575	T:0.425		
chr28	11833284	2	40	T:1	C:0		
chr28	11833337	2	38	G:0.605263	A:0.394737		
chr28	11833421	2	40	G:1	T:0		
chr28	11833456	2	40	CA:0	C:1		
chr28	11833459	2	40	C:0	G:1		
chr28	11833491	2	40	G:0	GAA:1		
chr28	11833492	2	40	T:0	TTAG:1		
chr28	11833560	2	40	ATGTGAATCT:1	A:0		
chr28	11833746	2	40	C:0.45	T:0.55		
chr28	11833798	2	38	T:1	C:0		
chr28	11833836	2	36	T:1	C:0		
chr28	11834109	2	38	C:1	T:0		
chr28	11834218	2	40	C:0.975	CCAAA:0.025		
chr28	11834221	2	40	C:0.975	T:0.025		

chr28	11834404	2	38	A:0.552632	G:0.447368	
chr28	11834520	2	40	G:0.425	A:0.575	
chr28	11834541	2	40	G:0.425	GT:0.575	
chr28	11834686	2	40	G:0.45	A:0.55	
chr28	11834708	2	40	G:1	A:0	
chr28	11834721	2	40	C:0.425	T:0.575	
chr28	11834734	2	40	C:0.425	T:0.575	
chr28	11834783	2	34	G:0.970588	A:0.0294118	
chr28	11834854	2	38	A:1	G:0	
chr28	11835063	2	36	G:0.611111	A:0.388889	
chr28	11835117	2	38	C:1	CA:0	
chr28	11835326	2	40	G:0.525	A:0.475	
chr28	11835337	3	40	T:0.975	TAAAAATGGGAAGGTAAAAA:0.025	TAAAAATGGGAAGGTAAAAAA:0
chr28	11835457	2	40	G:1	T:0	
chr28	11835484	2	38	T:0.526316	A:0.473684	
chr28	11835514	2	38	C:0.973684	CA:0.0263158	
chr28	11835610	2	36	T:0.472222	C:0.527778	
chr28	11835757	2	40	C:0.85	T:0.15	
chr28	11835894	2	38	C:0.421053	T:0.578947	
chr28	11835919	2	38	A:0.421053	G:0.578947	
chr28	11835996	2	36	T:0.972222	A:0.0277778	
chr28	11836065	2	40	T:1	C:0	
chr28	11836313	2	40	A:0.825	ATGAGAAAATATTTTTTTTTTTT:0.175	
chr28	11836403	2	40	C:1	T:0	
chr28	11836409	2	40	A:0.5	G:0.5	
chr28	11836574	2	38	T:1	G:0	
chr28	11836609	2	38	C:0.973684	A:0.0263158	
chr28	11836733	2	38	C:0.5	T:0.5	
chr28	11836800	2	38	T:0.5	C:0.5	
chr28	11836862	2	40	G:0.475	A:0.525	
chr28	11837006	2	40	A:0.475	C:0.525	
chr28	11837042	2	40	A:0.975	T:0.025	
chr28	11837067	2	40	C:0.975	G:0.025	
chr28	11837127	2	38	A:0.526316	C:0.473684	
chr28	11837136	2	36	C:1	A:0	
chr28	11837245	2	40	T:0.85	C:0.15	
chr28	11837546	2	40	T:0.975	C:0.025	
chr28	11837672	2	40	G:0.975	T:0.025	
chr28	11837742	2	38	A:0.973684	G:0.0263158	
chr28	11837794	2	40	T:0.575	A:0.425	
chr28	11837849	2	40	A:0.475	G:0.525	
chr28	11837878	2	36	T:0.916667	TA:0.0833333	
chr28	11837879	2	36	T:0.888889	A:0.111111	
chr28	11838120	2	40	G:0.975	A:0.025	
chr28	11838205	2	38	TAATG:0.684211	T:0.315789	
chr28	11838398	2	38	T:0.973684	A:0.0263158	
chr28	11838570	2	40	A:0.55	G:0.45	
chr28	11838777	2	36	G:0.361111	A:0.638889	
chr28	11838809	2	38	ATC:1	A:0	
chr28	11838940	2	38	A:0.421053	G:0.578947	
chr28	11839043	2	38	G:0.473684	T:0.526316	
chr28	11839070	2	38	A:0.473684	G:0.526316	

chr28 11839158 2 38 C:0.5 T:0.5
chr28 11839209 2 38 T:0.526316 TAAGA:0.473684
chr28 11839356 2 38 G:0.447368 GA:0.552632
chr28 11839467 2 38 G:0.526316 C:0.473684
chr28 11840040 2 36 T:0.972222 C:0.0277778
chr28 11840100 2 38 C:0.815789 A:0.184211
chr28 11840264 2 38 G:0.473684 A:0.526316
chr28 11840358 2 40 T:0.475 G:0.525
chr28 11840359 2 40 A:0.475 G:0.525
chr28 11840463 2 40 G:0.7 C:0.3
chr28 11841017 2 40 AG:0.975 A:0.025
chr28 11841310 2 40 T:0.425 C:0.575
chr28 11841644 2 40 C:1 T:0
chr28 11841865 2 34 T:0.5 C:0.5
chr28 11841892 2 36 A:1 G:0
chr28 11841941 2 38 G:1 A:0
chr28 11841996 2 40 C:0.4 T:0.6
chr28 11842065 2 40 T:0.55 TTATATC:0.45
chr28 11842081 2 40 A:1 G:0
chr28 11842281 2 34 TA:0.970588 T:0.0294118
chr28 11842318 2 40 C:0.475 A:0.525
chr28 11842384 2 40 T:1 G:0
chr28 11842436 2 40 A:0.45 G:0.55
chr28 11842615 2 40 T:0.525 C:0.475
chr28 11842801 2 40 C:0.975 A:0.025
chr28 11842817 2 40 G:0.85 A:0.15
chr28 11842837 2 38 A:1 G:0
chr28 11842919 2 38 A:0.473684 C:0.526316
chr28 11842933 2 40 T:0.85 A:0.15
chr28 11843043 2 38 A:0.973684 G:0.0263158
chr28 11843103 2 40 G:0 GA:1
chr28 11843108 2 40 AT:0 A:1
chr28 11843111 2 40 G:0 A:1
chr28 11843114 2 40 AC:0 A:1
chr28 11843120 2 40 G:0 A:1
chr28 11843121 2 40 A:0 G:1
chr28 11843135 2 40 G:0 T:1
chr28 11843136 2 40 A:0 G:1
chr28 11843151 2 40 C:0 A:1
chr28 11843152 2 40 G:0 C:1
chr28 11843216 2 40 GC:0 G:1
chr28 11843431 3 36 G:0.527778 GA:0.222222
GAA:0.25
chr28 11843435 2 38 A:0.947368 G:0.0526316
chr28 11843497 2 40 C:0.5 T:0.5
chr28 11843521 2 40 A:0.925 G:0.075
chr28 11843596 2 40 C:0.7 T:0.3
chr28 11843803 2 34 T:0.911765 C:0.0882353
chr28 11843819 4 40 CCTCTCTCTCTCTCTCTCT:0.55 C:0.275
CCTCT:0.125 CCTCTCTCT:0.05
chr28 11843936 2 40 G:0.825 A:0.175
chr28 11844118 2 12 C:1 T:0
chr28 11844333 2 38 C:0.5 A:0.5
chr28 11844361 2 40 AT:0.725 A:0.275

chr28	11844447	2	40	T:0.7	G:0.3
chr28	11844576	2	40	C:0.75	CAAAA:0.25
chr28	11845066	2	28	C:1	T:0
chr28	11845187	2	32	CA:0.96875	C:0.03125
chr28	11845202	2	32	C:0.96875	A:0.03125
chr28	11845333	2	38	A:0.894737	G:0.105263
chr28	11845820	2	20	T:0.95	A:0.05
chr28	11845839	2	22	A:0.454545	G:0.545455
chr28	11845849	2	22	T:1	G:0
chr28	11845940	2	40	C:0.625	T:0.375
chr28	11846050	2	22	A:0.636364	G:0.363636
chr28	11846163	2	38	T:0.921053	G:0.0789474
chr28	11846225	2	38	G:0.973684	A:0.0263158
chr28	11846457	2	40	T:0.475	G:0.525
chr28	11846915	2	38	GT:0.868421	G:0.131579
chr28	11846968	2	40	G:0.475	A:0.525
chr28	11847408	2	40	G:0.45	A:0.55
chr28	11847412	2	40	C:0.475	T:0.525
chr28	11847489	2	40	A:0.625	G:0.375
chr28	11847696	2	36	G:0.638889	A:0.361111
chr28	11847823	2	40	T:0.975	G:0.025
chr28	11848129	2	38	T:0.973684	C:0.0263158
chr28	11848274	2	40	A:0.825	G:0.175
chr28	11848503	2	40	TTTTC:0.95	T:0.05
chr28	11848581	2	38	A:1	C:0
chr28	11849481	2	36	C:0.694444	T:0.305556
chr28	11849660	2	38	A:1	T:0
chr28	11849668	2	38	A:0.921053	G:0.0789474
chr28	11849831	2	40	A:0.5	G:0.5
chr28	11850180	2	40	T:0.5	TA:0.5
chr28	11850231	2	40	T:0.5	G:0.5
chr28	11850436	2	40	T:0.875	TA:0.125
chr28	11850510	2	38	C:0.921053	G:0.0789474
chr28	11850866	2	40	G:1	T:0
chr28	11851134	2	38	G:0.842105	A:0.157895
chr28	11851149	2	36	CT:0.555556	C:0.444444
chr28	11851158	2	36	T:0.638889	A:0.361111
chr28	11851160	2	38	A:0.973684	AT:0.0263158
chr28	11851192	2	34	A:0.705882	AAG:0.294118
chr28	11851261	2	34	C:0.882353	T:0.117647
chr28	11851340	2	36	C:0.694444	T:0.305556
chr28	11851374	2	38	CA:1	C:0
chr28	11851486	2	40	G:1	A:0
chr28	11851525	2	38	T:0.447368	C:0.552632
chr28	11851613	2	38	C:0.815789	T:0.184211
chr28	11851725	2	40	T:0.875	C:0.125
chr28	11851797	2	38	T:1	G:0
chr28	11852005	2	40	A:0.775	T:0.225
chr28	11852018	2	40	A:0.775	G:0.225
chr28	11852216	2	38	C:0.631579	T:0.368421
chr28	11852271	2	38	C:0.842105	A:0.157895
chr28	11852326	2	36	A:0.472222	G:0.527778
chr28	11852709	2	38	T:0.710526	C:0.289474
chr28	11852752	2	38	A:0.736842	G:0.263158

chr28	11852785	2	34	T:0.735294	A:0.264706	
chr28	11853025	3	40	CAA:0.525	C:0.225	CA:0.25
chr28	11853136	2	40	C:0.7	T:0.3	
chr28	11853296	2	40	C:0.625	A:0.375	
chr28	11853440	2	36	T:0.388889	C:0.611111	
chr28	11853526	2	38	A:1	G:0	
chr28	11853567	2	40	G:1	A:0	
chr28	11853933	2	40	A:0.65	G:0.35	
chr28	11854146	2	40	T:0.85	G:0.15	
chr28	11854209	2	38	G:0.657895	A:0.342105	
chr28	11854354	2	40	A:0.825	G:0.175	
chr28	11854391	2	40	G:1	C:0	
chr28	11854476	2	40	TA:0.675	T:0.325	
chr28	11854506	2	40	C:0.625	T:0.375	
chr28	11854642	2	38	A:0.447368	T:0.552632	
chr28	11854673	2	40	T:0.7	A:0.3	
chr28	11854676	2	40	A:0.7	T:0.3	
chr28	11854708	2	36	C:1	A:0	
chr28	11855104	2	38	C:0.894737	T:0.105263	
chr28	11855240	2	40	TA:0.75	T:0.25	
chr28	11855243	2	40	CTCTATTTGTATATTTTGACAATCTATTT:0.75	C:0.25	
chr28	11855485	2	36	TAA:0.916667	T:0.0833333	
chr28	11855489	2	36	AAAAAATAAAATAAAAT:0.916667	A:0.0833333	
chr28	11855491	2	40	A:0.8	AT:0.2	
chr28	11855492	2	40	AAAT:1	A:0	
chr28	11855495	3	40	TAAAATAAAATAAAA:0.725	T:0.075	TAAATAAAATAAAA:0.2
chr28	11855500	3	40	TA:1	TAAA:0	T:0
chr28	11855501	2	40	A:0.85	AAAT:0.15	
chr28	11855509	2	40	A:0.8	T:0.2	
chr28	11855989	2	36	A:0.888889	G:0.111111	
chr28	11856291	2	40	G:0.675	A:0.325	
chr28	11856359	2	40	C:0.625	A:0.375	
chr28	11856589	2	40	T:1	C:0	
chr28	11856689	2	36	G:0.611111	A:0.388889	
chr28	11856771	2	40	A:0.65	C:0.35	
chr28	11856801	2	38	C:0.894737	T:0.105263	
chr28	11856828	2	38	C:1	T:0	
chr28	11856850	2	40	G:1	T:0	
chr28	11856984	2	36	C:1	T:0	
chr28	11857174	2	38	GA:0.605263	G:0.394737	
chr28	11857197	2	40	G:1	A:0	
chr28	11857306	2	38	T:0.447368	A:0.552632	
chr28	11857441	2	40	C:0.525	T:0.475	
chr28	11857656	2	38	G:0.736842	A:0.263158	
chr28	11857712	2	36	G:0.777778	A:0.222222	
chr28	11857887	2	40	G:0.6	T:0.4	
chr28	11857896	2	40	C:1	T:0	
chr28	11858000	2	40	A:0.475	C:0.525	
chr28	11858084	2	36	A:1	G:0	
chr28	11858194	2	38	G:0.657895	T:0.342105	
chr28	11858610	2	38	T:0.763158	C:0.236842	

chr28	11858912	2	40	C:1	T:0			
chr28	11859084	2	40	C:0.875	A:0.125			
chr28	11859194	2	38	C:1	T:0			
chr28	11859376	2	38	C:0.763158	T:0.236842			
chr28	11859705	2	38	ATTT:0.763158	A:0.236842			
chr28	11859712	2	38	C:1	T:0			
chr28	11859833	2	34	T:0.735294	TA:0.264706			
chr28	11859960	2	38	A:0.973684	T:0.0263158			
chr28	11860041	2	38	T:0.763158	C:0.236842			
chr28	11860201	2	36	CAT:0.916667	C:0.0833333			
chr28	11860909	2	38	C:0.789474	A:0.210526			
chr28	11861144	2	40	G:1	GTAAAA:0			
chr28	11861147	2	40	G:1	GA:0			
chr28	11861148	2	40	T:1	TAATCTTTTATCTAGG:0			
chr28	11861292	2	38	T:1	C:0			
chr28	11861299	2	36	C:1	CT:0			
chr28	11861520	2	40	C:0.875	G:0.125			
chr28	11862021	2	38	C:0.421053	T:0.578947			
chr28	11862382	2	40	A:0.875	G:0.125			
chr28	11862419	2	40	G:0.675	T:0.325			
chr28	11862432	2	40	A:0.975	C:0.025			
chr28	11862474	2	40	A:0.95	G:0.05			
chr28	11862665	2	40	GA:1	G:0			
chr28	11862708	2	38	TG:0.868421	T:0.131579			
chr28	11862852	2	40	A:0.5	G:0.5			
chr28	11863064	2	38	G:1	C:0			
chr28	11863087	2	38	G:0.552632	A:0.447368			
chr28	11863317	2	40	TCAA:0.725	T:0.275			
chr28	11863409	5	38	AAG:0.657895	A:0.236842	AAGAGAG:0.0263158	AAGAGAGAG:0.0263158	AAGAGAGAGAG: 0.0526316
chr28	11863543	2	40	T:0.5	G:0.5			
chr28	11863601	2	40	C:0.7	A:0.3			
chr28	11863735	2	40	C:0.9	T:0.1			
chr28	11863841	2	40	C:0.875	T:0.125			
chr28	11863912	2	40	T:0.9	C:0.1			
chr28	11864152	2	36	A:0.888889	G:0.111111			
chr28	11864492	2	38	A:0.789474	G:0.210526			
chr28	11864653	2	40	A:1	T:0			
chr28	11864859	2	40	C:0.5	CCT:0.5			
chr28	11865229	2	40	T:0.5	C:0.5			
chr28	11865376	2	36	C:0.666667	G:0.333333			
chr28	11865467	2	32	TAGA:0.46875	T:0.53125			
chr28	11865607	4	40	C:0.55	CTGTGTG:0.175	CTGTGTGTGTG:0.15	CTGTGTGTGTGTGTG:0.125	
chr28	11865639	2	40	C:0.875	G:0.125			
chr28	11865677	2	40	A:0.55	AAATG:0.45			
chr28	11865679	2	38	ATAAATAAG:0.894737	A: 0.105263			
chr28	11865687	2	40	G:0.55	A:0.45			
chr28	11865692	3	40	CAAAT:0.55	C:0	TAAAT: 0.45		
chr28	11865704	4	40	T:0.55	TAAATA:0.125	TAAATAAATA:0.075	TAAATAAATAAATA:0.25	

chr28 11865794 2 38 C:1 T:0
chr28 11866188 2 40 T:1 C:0
chr28 11866467 2 40 G:0.45 A:0.55
chr28 11866651 2 40 C:0.4 G:0.6
chr28 11866749 2 38 G:0.657895 GA:0.342105
chr28 11866946 2 38 ATTAAT:1 A:0
chr28 11866955 2 38 A:0.789474 ATTTAT:0.210526
chr28 11867008 2 38 C:0.842105 T:0.157895
chr28 11867265 2 36 C:1 A:0
chr28 11867606 2 40 C:0.85 T:0.15
chr28 11867663 2 38 A:0.868421 C:0.131579
chr28 11867677 2 36 C:0.805556 T:0.194444
chr28 11868484 2 40 ATATACT:1 A:0
chr28 11868566 2 36 T:1 C:0
chr28 11868609 2 36 G:1 A:0
chr28 11868894 2 40 T:0.675 TG:0.325
chr28 11868900 2 40 ATC:0.675 A:0.325
chr28 11868905 2 40 T:0.675
TCAGACCATGAGGTATATCAAATTAGATGTGGCA:0.325
chr28 11869415 2 36 G:1 A:0
chr28 11869471 2 38 C:0.5 T:0.5
chr28 11869604 2 36 C:1 G:0
chr28 11869666 2 34 A:0.911765 AT:0.0882353
chr28 11869801 2 38 CT:0.368421 C:0.631579
chr28 11869826 2 38 C:1 T:0
chr28 11869857 2 38 A:0.894737 C:0.105263
chr28 11870017 2 36 A:0.666667 AT:0.333333
chr28 11870084 2 36 T:0.888889 C:0.111111
chr28 11870558 2 40 CA:0.525 C:0.475
chr28 11870749 2 38 T:0.684211 TC:0.315789
chr28 11870751 2 38 C:0.973684 CG:0.0263158
chr28 11870758 4 38 C:0.894737 CAA:0.0526316
CCCAAA:0.0263158 CCCCAAA:0.0263158
chr28 11870817 2 34 G:0.852941 A:0.147059
chr28 11870859 2 38 G:1 A:0
chr28 11871195 2 40 G:1 A:0
chr28 11871288 2 36 A:0.916667 G:0.0833333
chr28 11871939 2 40 C:0.625 CAAAA:0.375
chr28 11871970 2 40 G:0.9 A:0.1
chr28 11872004 2 40 G:0.45 T:0.55
chr28 11872102 3 36 GTT:0.527778 G:0.0833333
GTTT:0.388889
chr28 11872114 2 36 T:1 A:0
chr28 11872115 3 36 T:0.888889 A:0.0277778
TAAA:0.0833333
chr28 11872116 2 36 A:0.611111 T:0.388889
chr28 11872293 4 40 GTT:0.45 G:0.475 GT:0.025 GTTT:
0.05
chr28 11872329 2 40 C:0.55 G:0.45
chr28 11872333 2 40 C:1 T:0
chr28 11872600 2 40 G:0.55 GA:0.45
chr28 11873067 2 40 A:0.7 AGT:0.3
chr28 11873070 4 40 A:0.45 ATAG:0.025
ATAGTATAGTATAG:0.275 ATATAGTATAGTATAGTATAG:0.25

chr28	11873397	2	38	T:0.447368	C:0.552632	
chr28	11873413	2	36	C:1	T:0	
chr28	11873422	2	38	T:0.447368	C:0.552632	
chr28	11873476	2	38	G:1	A:0	
chr28	11873785	2	40	A:1	G:0	
chr28	11873991	2	40	T:0.725	A:0.275	
chr28	11874303	2	36	A:0.833333	C:0.166667	
chr28	11874403	2	38	T:0.473684	TC:0.526316	
chr28	11874613	2	36	C:0.722222	T:0.277778	
chr28	11874662	2	40	C:0.45	G:0.55	
chr28	11874841	2	38	G:0.868421	A:0.131579	
chr28	11874853	2	40	T:1	C:0	
chr28	11875008	2	36	CA:0.75	C:0.25	
chr28	11875013	2	40	A:0.925	AAC:0.075	
chr28	11875034	2	40	AC:0.975	A:0.025	
chr28	11875039	2	40	AC:0.975	A:0.025	
chr28	11875040	3	40	C:0.975	A:0.025	CAAA:0
chr28	11875046	2	40	ACC:0.75	A:0.25	
chr28	11875047	2	40	C:0.85	A:0.15	
chr28	11875048	3	40	C:0.825	CAAA:0	CAAAAAAAA:0.175
chr28	11875329	2	40	C:0.9	CT:0.1	
chr28	11875353	2	40	G:0.5	C:0.5	
chr28	11875472	2	36	G:0.833333	A:0.166667	
chr28	11875746	2	40	G:1	A:0	
chr28	11875928	2	40	A:0.95	G:0.05	
chr28	11876735	2	40	C:0.725	T:0.275	
chr28	11876758	2	40	A:0.95	AT:0.05	
chr28	11876893	2	36	A:0.805556	AT:0.194444	
chr28	11877045	2	36	T:0.666667	C:0.333333	
chr28	11877356	2	40	T:0.7	C:0.3	
chr28	11877566	2	38	C:1	T:0	
chr28	11877714	2	38	A:1	G:0	
chr28	11877788	2	40	AT:0.75	A:0.25	
chr28	11877919	2	38	G:0.815789	A:0.184211	
chr28	11878099	2	40	C:0.75	T:0.25	
chr28	11878140	2	40	T:1	C:0	
chr28	11878157	2	38	A:0.526316	G:0.473684	
chr28	11878380	2	40	C:0.775	T:0.225	
chr28	11878898	2	40	TC:0.9	T:0.1	
chr28	11878899	2	40	C:0.85	T:0.15	
chr28	11878981	2	36	A:1	G:0	
chr28	11879372	2	38	G:0.763158	A:0.236842	
chr28	11879468	2	38	T:0	C:1	
chr28	11879520	2	36	C:0	T:1	
chr28	11879527	2	36	C:0	T:1	
chr28	11879532	2	36	C:0	T:1	
chr28	11879539	2	36	TG:0	T:1	
chr28	11879542	2	36	G:0	GT:1	
chr28	11879590	2	34	G:0	T:1	
chr28	11879696	2	32	A:1	G:0	
chr28	11879839	2	34	TA:1	T:0	
chr28	11879919	2	40	C:0.85	T:0.15	
chr28	11880140	2	40	T:0.725	G:0.275	
chr28	11880412	2	36	AG:0	A:1	

chr28	11880549	2	40	AT:0.95	A:0.05	
chr28	11880555	2	40	T:0.725	G:0.275	
chr28	11881779	2	36	G:0.722222		A:0.277778
chr28	11881837	2	40	C:0.675	T:0.325	
chr28	11882344	2	38	G:0.736842		T:0.263158
chr28	11882615	2	38	T:0.684211		C:0.315789
chr28	11883074	2	40	G:1	C:0	
chr28	11883409	2	40	G:0.675	A:0.325	
chr28	11883866	2	38	G:1	T:0	
chr28	11883981	2	40	T:0.775	A:0.225	
chr28	11884006	2	40	G:1	T:0	
chr28	11884147	2	40	G:0.65	A:0.35	
chr28	11884208	2	40	C:1	T:0	
chr28	11884563	2	38	A:0.473684		G:0.526316
chr28	11885008	2	38	G:0.763158		A:0.236842
chr28	11885048	2	40	C:1	T:0	
chr28	11885990	2	38	C:0.763158		T:0.236842
chr28	11886152	2	34	GAT:0.970588		G:0.0294118
chr28	11886155	2	34	C:0.970588		CA:0.0294118
chr28	11886315	2	40	ATTT:0.725		A:0.275
chr28	11886318	3	40	T:0.675	A:0.05	TAAAAAAAAAAAA:
0.275						
chr28	11886414	2	40	G:0.675	A:0.325	
chr28	11886615	2	40	G:0.475	T:0.525	
chr28	11887045	2	38	T:0.5	TA:0.5	
chr28	11887409	2	38	T:0.763158		C:0.236842
chr28	11887582	2	38	G:0.684211		A:0.315789
chr28	11887973	2	38	C:1	T:0	
chr28	11888154	2	40	C:0.45	T:0.55	
chr28	11888210	2	38	G:1	A:0	
chr28	11888556	2	38	C:0.736842		G:0.263158
chr28	11888695	2	40	C:1	A:0	
chr28	11889398	2	36	C:0.722222		T:0.277778
chr28	11889609	2	38	G:1	A:0	
chr28	11890634	2	36	G:1	T:0	
chr28	11890884	2	40	A:0.975	G:0.025	
chr28	11891477	2	38	C:0.552632		G:0.447368
chr28	11891523	2	38	A:1	G:0	
chr28	11891537	2	40	C:0.725	T:0.275	
chr28	11892124	2	40	G:0.9	C:0.1	
chr28	11892190	2	40	C:1	T:0	
chr28	11892692	2	38	AG:1	A:0	
chr28	11892694	2	38	C:1	T:0	
chr28	11893064	2	38	A:0.710526		G:0.289474
chr28	11893084	2	38	G:1	C:0	
chr28	11893095	2	38	C:1	A:0	
chr28	11893182	2	38	GA:0.447368		G:0.552632
chr28	11893201	2	38	G:0.473684		A:0.526316
chr28	11893257	2	38	A:0.5	G:0.5	
chr28	11893458	2	36	G:0.666667		C:0.333333
chr28	11893603	2	38	C:1	T:0	
chr28	11894628	2	38	C:0.447368		T:0.552632
chr28	11895309	2	36	T:0.361111		C:0.638889
chr28	11896364	2	40	C:1	T:0	

chr28	11897117	2	40	TCCTAAGC:0.975	T:0.025		
chr28	11897146	2	40	A:1	G:0		
chr28	11897231	2	38	T:0.736842	G:0.263158		
chr28	11897412	2	34	AT:1	A:0		
chr28	11897471	2	38	C:1	T:0		
chr28	11897491	2	38	A:1	C:0		
chr28	11897718	2	36	G:1	A:0		
chr28	11897838	2	40	A:0.5	T:0.5		
chr28	11898077	2	38	C:1	T:0		
chr28	11898115	2	36	C:1	T:0		
chr28	11898121	2	40	AATATATATATATATATAT:0.4	A:0.6		
chr28	11898593	2	32	ATG:1	A:0		
chr28	11898597	3	40	G:0.7	GTA:0.2	GTATA:0.1	
chr28	11898767	2	40	G:0.75	A:0.25		
chr28	11898859	2	40	G:1	A:0		
chr28	11899659	2	38	G:0.736842	A:0.263158		
chr28	11899683	2	38	A:1	G:0		
chr28	11899909	2	40	A:1	T:0		
chr28	11900076	2	40	G:1	A:0		
chr28	11900354	2	40	A:0.925	T:0.075		
chr28	11900702	2	38	C:1	CT:0		
chr28	11900739	2	38	CT:1	C:0		
chr28	11900952	4	36	GAAA:0.527778	G:0	GAA:0.194444	GAAAAA:0.277778
chr28	11900971	2	36	A:1	T:0		
chr28	11901038	2	32	T:0.71875	G:0.28125		
chr28	11901103	4	38	C:0.868421	CCT:0.131579	CCTCT:0	CCTCTCT:0
chr28	11901139	2	38	AAAT:1	A:0		
chr28	11901142	2	38	T:0.868421	TA:0.131579		
chr28	11901145	2	38	A:1	ATT:0		
chr28	11901490	2	38	G:1	C:0		
chr28	11901529	2	40	G:1	A:0		
chr28	11901609	2	34	G:0.264706	A:0.735294		
chr28	11901648	2	36	T:0	C:1		
chr28	11901796	2	38	C:0.263158	T:0.736842		
chr28	11901801	2	38	A:0	T:1		
chr28	11902561	2	40	G:0.775	A:0.225		
chr28	11902834	2	40	A:0	G:1		
chr28	11902875	2	40	T:0.8	TTTTCTTTC:0.2		
chr28	11903577	2	36	G:1	A:0		
chr28	11904397	2	38	A:0.736842	G:0.263158		
chr28	11904411	2	38	G:0.736842	GA:0.263158		
chr28	11904423	2	38	T:0.736842	TG:0.263158		
chr28	11904429	2	38	A:0.473684	G:0.526316		
chr28	11904966	2	36	T:0.694444	G:0.305556		
chr28	11905046	2	40	A:0.725	G:0.275		
chr28	11905088	2	40	A:1	AT:0		
chr28	11905201	2	38	C:0	G:1		
chr28	11905204	2	38	C:0.973684	T:0.0263158		
chr28	11905287	2	40	T:0.2	A:0.8		
chr28	11905538	2	38	G:0.210526	A:0.789474		
chr28	11905562	2	38	C:0.815789	A:0.184211		
chr28	11905567	2	38	G:0.815789	A:0.184211		

chr28	11905695	2	38	T:1	A:0	
chr28	11905737	2	40	GA:1	G:0	
chr28	11905896	2	40	AAC:0.75	A:0.25	
chr28	11905908	2	38	C:0.263158	A:0.736842	
chr28	11906048	2	40	G:0.775	C:0.225	
chr28	11906064	2	40	T:0	G:1	
chr28	11906235	2	36	C:0.777778	A:0.222222	
chr28	11906252	2	36	A:0.777778	T:0.222222	
chr28	11906403	2	38	A:1	G:0	
chr28	11906470	2	40	A:0.8	G:0.2	
chr28	11906582	2	36	G:1	C:0	
chr28	11906587	2	38	A:0.763158	G:0.236842	
chr28	11906709	2	40	A:0.75	G:0.25	
chr28	11906993	2	40	T:0.775	C:0.225	
chr28	11907074	2	38	T:0.736842	TC:0.263158	
chr28	11907188	2	38	C:1	T:0	
chr28	11907338	2	40	AC:0.725	A:0.275	
chr28	11907340	2	40	A:0.725	T:0.275	
chr28	11907516	2	40	T:1	C:0	
chr28	11907594	2	40	G:0.225	A:0.775	
chr28	11907854	2	38	GAAC:0	G:1	
chr28	11908150	2	40	T:0.875	A:0.125	
chr28	11908333	2	38	A:0.315789	G:0.684211	
chr28	11908483	2	38	A:0.736842	C:0.263158	
chr28	11908607	2	38	A:0.657895	G:0.342105	
chr28	11908661	2	38	CT:1	C:0	
chr28	11908670	2	40	TA:0.875	T:0.125	
chr28	11908671	2	40	A:0.875	T:0.125	
chr28	11908792	2	38	C:0.657895	T:0.342105	
chr28	11908821	2	40	C:0.65	G:0.35	
chr28	11908827	2	40	C:0.65	T:0.35	
chr28	11909091	2	38	C:0.736842	T:0.263158	
chr28	11909359	2	36	G:0.972222	GTGAA:0.0277778	
chr28	11909966	2	38	TGA:0.631579	T:0.368421	
chr28	11910739	2	40	C:0.575	CAAATATAAAAAAATATAAAAAAA:0.425	
chr28	11910742	2	40	C:0.575	CAAAAAAT:0.425	
chr28	11910910	2	40	G:0.275	C:0.725	
chr28	11911220	2	40	CTG:1	C:0	
chr28	11911433	2	36	G:0.333333	T:0.666667	
chr28	11911657	2	38	C:1	T:0	
chr28	11912001	3	34	CTT:0.794118	C:0	CT:0.205882
chr28	11912002	2	36	T:0.861111	TC:0.138889	
chr28	11912028	2	32	C:0.375	T:0.625	
chr28	11912031	2	32	T:0.375	C:0.625	
chr28	11912299	2	40	C:1	T:0	
chr28	11912379	2	40	G:0.875	GTC:0.125	
chr28	11912395	2	40	C:1	G:0	
chr28	11912418	2	40	TAAA:0.925	T:0.075	
chr28	11912629	2	40	A:0.725	G:0.275	
chr28	11912867	2	40	T:1	C:0	
chr28	11912928	2	40	A:0.775	C:0.225	
chr28	11912964	2	38	T:0.5	TTGA:0.5	

chr28	11913368	2	38	T:0.263158	A:0.736842
chr28	11913502	2	38	T:0.236842	A:0.763158
chr28	11913526	2	40	C:0.25	T:0.75
chr28	11913869	2	38	C:0.736842	T:0.263158
chr28	11914247	2	40	A:0.325	T:0.675
chr28	11914547	2	40	CTTT:0.85	C:0.15
chr28	11914566	3	40	TAAG:0.775	T:0.075	AAAG:
0.15
chr28	11914575	2	36	T:0.333333	C:0.666667
chr28	11914582	2	36	T:0.333333	TAGAC:0.666667
chr28	11914621	2	36	C:0.722222	G:0.277778
chr28	11914627	2	36	T:0.722222	C:0.277778
chr28	11914642	2	36	C:0.722222	G:0.277778
chr28	11914711	2	38	C:1	T:0
chr28	11915125	2	38	G:0.263158	A:0.736842
chr28	11915222	2	38	CTCTTACTCTTAGACCT:0.342105
C:0.657895
chr28	11915267	2	34	T:0.264706	A:0.735294
chr28	11915465	2	38	A:0.342105	G:0.657895
chr28	11915522	2	38	G:1	T:0
chr28	11915639	2	36	G:0.277778	A:0.722222
chr28	11915812	2	38	G:0.263158	A:0.736842
chr28	11915856	2	40	T:0.8	A:0.2
chr28	11916054	2	34	A:0.205882	G:0.794118
chr28	11916275	2	36	G:1	A:0
chr28	11916371	2	36	T:0.555556	G:0.444444
chr28	11916624	2	38	C:1	T:0
chr28	11916653	2	38	T:1	C:0
chr28	11916841	2	38	A:1	G:0
chr28	11918348	2	14	G:0.857143	A:0.142857
chr28	11918457	2	34	TA:0.735294	T:0.264706
chr28	11918523	2	34	G:0.647059	GTT:0.352941
chr28	11919089	2	38	C:1	T:0
chr28	11919381	2	36	A:0.527778	G:0.472222
chr28	11919506	3	40	GGTGTGTGTGT:0.775	G:0.2
GGTGTGTGTGTGT:0.025
chr28	11920706	2	38	CT:0.789474	C:0.210526
chr28	11921527	2	38	G:0.736842	A:0.263158
chr28	11922160	2	40	GA:0.925	G:0.075
chr28	11922375	2	38	A:0.657895	T:0.342105
chr28	11922483	2	38	C:1	T:0
chr28	11922543	2	38	C:1	T:0
chr28	11922905	2	40	A:1	G:0
chr28	11923553	2	38	G:0.473684	C:0.526316
chr28	11924130	2	40	A:0.725	G:0.275
chr28	11924200	2	40	G:0.675	GA:0.325
chr28	11924543	3	38	T:0.315789	TA:0.394737
TAA:0.289474
chr28	11924749	2	34	C:1	T:0
chr28	11924955	2	38	G:1	C:0
chr28	11925141	2	40	C:0.8	T:0.2
chr28	11925145	2	40	C:0.8	T:0.2
chr28	11925403	2	36	T:0.777778	G:0.222222
chr28	11925465	3	40	TAC:0.325	T:0.4	TACAC:

0.275
chr28 11925618 2 40 GTA:0.8 G:0.2
chr28 11926540 2 38 C:1 T:0
chr28 11926790 2 40 ATAT:0.85 A:0.15
chr28 11927064 2 40 A:1 G:0
chr28 11927120 2 38 C:1 A:0
chr28 11927127 2 38 G:1 A:0
chr28 11927537 2 40 A:0.875 T:0.125
chr28 11927971 2 40 A:0.75 G:0.25
chr28 11928193 3 40 GA:0.75 G:0.05 GAAAA:0.2
chr28 11928202 2 38 A:0.473684 G:0.526316
chr28 11928669 2 38 C:1 T:0
chr28 11929228 2 40 A:0.8 G:0.2
chr28 11929501 2 32 A:0.28125 T:0.71875
chr28 11929565 2 28 T:0.642857 TG:0.357143
chr28 11929912 2 36 A:0.444444 T:0.555556
chr28 11930389 2 40 G:1 C:0
chr28 11930492 2 36 A:0.666667 G:0.333333
chr28 11930545 2 38 G:0.131579 GAATA:0.868421
chr28 11930585 2 36 CT:1 C:0
chr28 11930846 2 40 T:0.925 C:0.075
chr28 11931229 2 38 C:1 T:0
chr28 11931810 2 40 A:1 G:0
chr28 11931814 2 40 C:0.825 CTAAA:0.175
chr28 11932298 2 38 G:0.236842 T:0.763158
chr28 11932598 2 38 GTATATATATATATATATATATATATATATATA:
0.710526 G:0.289474
chr28 11932897 2 24 G:0.375 A:0.625
chr28 11933330 2 40 A:1 AAAAT:0
chr28 11933512 2 40 G:0.275 GT:0.725
chr28 11933888 2 36 T:0.5 C:0.5
chr28 11934128 2 38 C:0.736842 T:0.263158
chr28 11934241 2 40 T:0.75 TC:0.25
chr28 11934242 5 40 T:0.5 C:0.3 TCTCTCTC:0.025
TCTCTCTCTCTCTC:0 TCTCTCTCTCTCTCTC:0.175
chr28 11934544 2 40 CAG:0.975 C:0.025
chr28 11934741 2 40 G:0.725 A:0.275
chr28 11935044 2 36 T:0.472222 A:0.527778
chr28 11935286 2 38 C:0.842105 CA:0.157895
chr28 11935773 2 38 CCTCTCTCT:0.421053 C:
0.578947
chr28 11936014 2 36 T:0.972222 TA:0.0277778
chr28 11936934 2 36 T:0.527778 TA:0.472222
chr28 11937093 2 38 G:0.815789 T:0.184211
chr28 11937180 2 38 G:0.763158 A:0.236842
chr28 11937363 2 40 T:1 A:0
chr28 11937914 2 40 T:1 TAA:0
chr28 11937922 2 40 A:1 AT:0
chr28 11937923 2 40 A:1 ATAT:0
chr28 11937925 3 40 A:0.925 T:0.025 ATAT:0.05
chr28 11937926 2 40 A:0.85 AT:0.15
chr28 11937927 4 40 A:0.15 T:0.25 AT:0.35 ATAT:
0.25
chr28 11937946 2 38 A:0.473684 G:0.526316

chr28	11938525 3	38	TA:0.342105	T:0.473684
TTAAAAAAA:0.184211
chr28	11938606 2	38	G:1	A:0
chr28	11938703 2	32	ATTTT:0.78125	A:0.21875
chr28	11938709 2	32	T:0.78125	TAAAAAA:0.21875
chr28	11938710 2	34	A:1	T:0
chr28	11938757 5	40	AAAATAAATAAATAAATAAAT:0.2 A:0.05
AAAAT:0.125	AAAATAAAT:0.3	AAAATAAATAAAT:0.325
chr28	11938798 2	38	AAAT:1	A:0
chr28	11938805 2	38	TAAATAAATAAAATA:1	T:0
chr28	11940234 2	38	C:0	G:1
chr28	11940662 2	36	T:1	A:0
chr28	11941067 2	30	G:1	A:0
chr28	11941333 3	38	GA:0.578947	G:0.236842
GAA:0.184211
chr28	11941949 3	40	CT:0.5	C:0.425	CTT:0.075
chr28	11942027 2	38	T:0.763158	A:0.236842
chr28	11942083 2	36	C:0.694444	CT:0.305556
chr28	11942208 2	38	A:1	G:0
chr28	11942504 2	40	T:0.925	C:0.075
chr28	11943099 2	40	G:1	A:0
chr28	11943182 2	40	T:0.5	C:0.5
chr28	11943470 3	40	C:0.5	CT:0.3	CTT:0.2
chr28	11943775 2	40	T:1	C:0
chr28	11943783 2	40	G:1	T:0
chr28	11944716 3	38	C:0.815789	CTATT:0.184211
CTATTTATT:0
chr28	11944733 2	38	TA:0.394737	T:0.605263
chr28	11944735 2	38	TTTATTTA:0.394737	T:
0.605263
chr28	11945088 2	40	A:1	T:0
chr28	11945937 2	40	G:1	A:0
chr28	11946538 2	40	G:0.475	A:0.525
chr28	11946751 2	40	T:1	TG:0
chr28	11946778 2	40	A:0.725	G:0.275
chr28	11946871 2	34	C:1	T:0
chr28	11947351 2	40	C:0.75	T:0.25
chr28	11947671 2	40	C:0.675	T:0.325
chr28	11947690 2	40	T:0.675	TC:0.325
chr28	11948105 2	40	T:0.8	G:0.2
chr28	11948372 2	36	G:0.805556	C:0.194444
chr28	11948644 2	40	T:0.875	C:0.125
chr28	11949722 2	40	C:0.775	T:0.225
chr28	11949766 2	38	G:0	A:1
chr28	11952122 2	36	TA:0.944444	T:0.0555556
chr28	11952170 2	40	A:1	T:0
chr28	11953056 2	38	A:0.684211	G:0.315789
chr28	11953367 2	36	A:1	G:0
chr28	11953661 2	38	T:0	A:1
chr28	11953692 2	38	AGATAGAT:0.947368	A:
0.0526316
chr28	11953710 2	38	AGAT:0.894737	A:0.105263
chr28	11953926 2	36	TGGGGG:0 T:1
chr28	11953931 2	36	G:0	GTCATT:1

chr28 11953980 2 34 C:0 G:1
chr28 11954267 2 40 GT:0.45 G:0.55
chr28 11954676 3 40 C:0.475 CACAT:0.25 CACACAT:
0.275
chr28 11956227 2 38 T:1 TCC:0
chr28 11957195 2 38 A:1 G:0
chr28 11957224 2 34 G:0.823529 A:0.176471
chr28 11957389 2 32 GTC:0.6875 G:0.3125
chr28 11957443 2 38 TA:0.763158 T:0.236842
chr28 11957444 2 38 A:1 T:0
chr28 11958561 2 38 C:1 T:0
chr28 11958875 2 38 A:0.236842 G:0.763158
chr28 11959680 2 36 T:0 C:1
chr28 11959744 2 36 C:0.638889 T:0.361111
chr28 11960152 2 38 C:1 A:0
chr28 11960153 2 38 A:1 G:0
chr28 11960190 2 38 G:0.473684 C:0.526316
chr28 11960659 2 40 T:0.9 C:0.1
chr28 11960831 2 40 C:0.275 T:0.725
chr28 11961189 4 40 TAAAA:0 T:0.15 TA:0.65 TAA:0.2
chr28 11961209 2 40 C:0.975 T:0.025
chr28 11961560 2 36 A:1 T:0
chr28 11961614 2 40 T:0.8 C:0.2
chr28 11962378 2 40 A:0.925 G:0.075
chr28 11962816 2 40 G:0.4 T:0.6
chr28 11963058 2 40 A:1 T:0
chr28 11963604 2 40 G:0.25 T:0.75
chr28 11964129 2 40 CCCAACAGT:0.925 C:0.075
chr28 11964386 3 38 G:0.789474 GA:0.131579
GAA:0.0789474
chr28 11964876 2 40 A:0.825 G:0.175
chr28 11965104 2 40 C:0.975 CA:0.025
chr28 11966279 2 40 C:0.85 T:0.15
chr28 11966965 2 40 T:1 C:0
chr28 11967211 2 40 G:1 A:0
chr28 11967480 2 40 C:0.725 G:0.275
chr28 11967512 2 40 T:0.275 C:0.725
chr28 11967802 2 38 T:1 TA:0
chr28 11967808 2 38 A:0 AT:1
chr28 11967885 2 40 C:0.8 T:0.2
chr28 11968174 2 36 G:1 A:0
chr28 11968322 2 36 C:0 T:1
chr28 11968856 4 38 TAAA:0.605263 T:0 TA:
0.184211 TAA:0.210526
chr28 11969669 2 36 T:0.972222 TTC:0.0277778
chr28 11970830 3 38 G:0.5 T:0.289474 GT:
0.210526
chr28 11971952 2 38 T:0 A:1
chr28 11972207 2 40 CT:0.975 C:0.025
chr28 11972301 2 34 G:0.794118 A:0.205882
chr28 11973488 2 38 T:0 A:1
chr28 11973621 2 40 C:1 T:0
chr28 11974765 6 40 TTTTA:0.475 T:0.15
TTTTATTTA:0.075 TTTTATTTATTTA:0.05 TTTTATTTATTTATTTA:0.25

TTTTATTTATTTATTTATTTA:0
chr28 11974832 2 38 C:0.973684 T:0.0263158
chr28 11975354 3 40 CCTCTCT:0.9 C:0.1
CCTCTCTCT:0
chr28 11975970 2 38 TAA:0.868421 T:0.131579
chr28 11976288 2 38 C:0.315789 T:0.684211
chr28 11976388 2 34 C:0.852941 G:0.147059
chr28 11976416 2 34 GA:0.823529 G:0.176471
chr28 11977267 2 38 A:1 T:0
chr28 11977593 2 38 TG:0.973684 T:0.0263158
chr28 11977594 2 38 G:0.684211 T:0.315789
chr28 11977941 2 40 G:0.3 GCT:0.7
chr28 11978498 2 40 C:1 T:0
chr28 11978698 2 40 C:1 G:0
chr28 11979036 2 38 C:0 T:1
chr28 11979037 2 38 T:0 A:1
chr28 11979045 2 38 G:0 A:1
chr28 11979095 3 40 C:0 A:0.075 G:0.925
chr28 11979096 2 40 G:0 A:1
chr28 11979117 2 40 C:0 A:1
chr28 11979120 2 40 TG:0 T:1
chr28 11979122 2 40 T:0 A:1
chr28 11979124 2 40 T:0 TA:1
chr28 11979128 2 40 G:0 GA:1
chr28 11979294 2 34 G:0.911765 A:0.0882353
chr28 11979403 2 40 A:0.9 T:0.1
chr28 11979410 2 40 T:0.675 TA:0.325
chr28 11979466 2 40 A:1 G:0
chr28 11979565 2 36 T:1 G:0
chr28 11979783 2 36 GA:1 G:0
chr28 11979986 2 40 T:1 TA:0
chr28 11980824 2 38 T:0.842105 A:0.157895
chr28 11981157 2 36 A:0.805556 G:0.194444
chr28 11981193 2 40 T:0.7 C:0.3
chr28 11981412 2 38 C:0.868421 A:0.131579
chr28 11981521 2 34 AT:0.294118 A:0.705882
chr28 11981630 2 18 G:0.888889 A:0.111111
chr28 11981868 2 34 T:1 C:0
chr28 11981921 2 34 G:1 A:0
chr28 11982155 2 38 C:1 T:0
chr28 11982863 2 32 TA:0.71875 T:0.28125
chr28 11982967 2 40 T:0.7 G:0.3
chr28 11983015 2 36 C:1 T:0
chr28 11984094 4 40 TA:0.75 T:0 TAA:0.225
TAAA:0.025
chr28 11984180 2 40 A:0.825 T:0.175
chr28 11984232 2 34 TA:0.764706 T:0.235294
chr28 11984843 2 40 T:0.975 C:0.025
chr28 11985322 4 40 T:0.925 TACACAC:0.05
TACACACACACAC:0 TACACACACACACAC:0.025
chr28 11985341 2 40 A:1 ACACACG:0
chr28 11986832 2 38 C:0.894737 T:0.105263
chr28 11986972 2 40 T:0.075 G:0.925
chr28 11987107 2 38 C:0.973684 T:0.0263158

chr28	11987193	2	38	C:1	CT:0	
chr28	11987203	3	38	TAA:0.657895	AAA:0.289474	T:0.0526316
chr28	11987204	2	38	A:0.368421	T:0.631579	
chr28	11987205	3	38	A:0.315789	T:0.631579	ATTT:0.0526316
chr28	11987394	2	36	G:0.0833333	A:0.916667	
chr28	11987565	2	40	C:0.95	G:0.05	
chr28	11987567	2	38	G:0.105263	C:0.894737	
chr28	11987569	2	40	T:0.075	G:0.925	
chr28	11987614	3	38	A:0	AC:0.973684	AAC:0.0263158
chr28	11987738	2	38	G:0.894737	A:0.105263	
chr28	11987792	2	40	C:1	T:0	
chr28	11987831	2	38	CA:0.921053	C:0.0789474	
chr28	11987838	2	38	AG:1	A:0	
chr28	11987939	2	38	G:0.131579	A:0.868421	
chr28	11988358	2	38	T:0.921053	A:0.0789474	
chr28	11988666	2	36	C:0.0833333	T:0.916667	
chr28	11989396	2	36	C:0	T:1	
chr28	11989706	2	38	T:0.947368	C:0.0526316	
chr28	11989779	2	38	C:0.0789474	T:0.921053	
chr28	11989837	2	38	G:0.789474	A:0.210526	
chr28	11989917	2	38	A:0.0789474	G:0.921053	
chr28	11990163	2	38	G:0.710526	A:0.289474	
chr28	11990325	2	40	T:1	G:0	
chr28	11990723	2	36	TTGAC:1	T:0	
chr28	11990918	2	38	TTGTC:1	T:0	
chr28	11990954	2	38	A:0.947368	T:0.0526316	
chr28	11991092	2	40	C:0.95	G:0.05	
chr28	11991267	2	40	C:1	T:0	
chr28	11991290	2	40	T:1	C:0	
chr28	11991416	2	38	G:0.0789474	A:0.921053	
chr28	11991771	2	40	C:0.075	T:0.925	
chr28	11992173	2	40	A:0	G:1	
chr28	11992225	2	38	TA:0.736842	T:0.263158	
chr28	11992457	2	40	T:0.95	C:0.05	
chr28	11992471	2	40	CT:1	C:0	
chr28	11992489	2	38	C:1	A:0	
chr28	11992545	2	40	G:1	A:0	
chr28	11992674	2	38	GA:0.0789474	G:0.921053	
chr28	11993152	2	40	A:0	G:1	
chr28	11993700	2	36	A:0	G:1	
chr28	11993701	2	36	A:1	G:0	
chr28	11994118	2	40	A:1	T:0	
chr28	11994154	2	40	G:0.975	A:0.025	
chr28	11995746	2	40	T:1	C:0	
chr28	11995875	2	38	T:1	A:0	
chr28	11996015	2	40	C:1	T:0	
chr28	11996842	2	40	T:0	C:1	
chr28	11996881	2	40	C:1	T:0	
chr28	11996935	2	40	G:0	T:1	
chr28	11997298	2	38	G:1	GA:0	
chr28	11997605	2	38	C:1	T:0	

chr28	11997790	2	38	G:0.973684	GA:0.0263158
chr28	11997824	2	36	C:1	G:0
chr28	11998542	2	38	C:0	CT:1
chr28	11998797	2	38	G:0	C:1
chr28	11998803	2	38	G:1	A:0
chr28	11999041	2	40	G:1	A:0
chr28	11999424	2	40	G:1	T:0
