# Supplementary material for: Genome-Wide Analyses Suggest Mechanisms Involving Early B-Cell Development in Canine IgA Deficiency
Source: PLoS One. 2015 Jul 30;10(7):e0133844. doi: 10.1371/journal.pone.0133844 (PMC4520476; doi:10.1371/journal.pone.0133844)
Supplement: S8 Table — (PDF) [file pone.0133844.s018.pdf]

Table S8. FST values comparing dog and wolf pools in the CFA28 region

canfam2				canfam3					
middle	chr	start	end	chr	start	end	Fst	SD	SNPs (N)
13075594	chr28	13050594	13100594	chr28	10053171	10103177	0.210821571578906	0.0742238393010564	59
13100594	chr28	13075594	13125594	chr28	10078171	10128177	0.209639672270046	0.0638364531299045	30
13125594	chr28	13100594	13150594	chr28	10103177	10153177	0.155617804504721	-0.410946802904633	56
13150594	chr28	13125594	13175594	chr28	10128177	10178177	0.150125172121944	-0.459220029928627	30
13175594	chr28	13150594	13200594	chr28	10153177	10203177	0.178236373596786	-0.21215845432284	67
13200594	chr28	13175594	13225594	chr28	10178177	10228186	0.192193607738807	-0.0894921833236299	23
13225594	chr28	13200594	13250594	chr28	10203177	10253186	0.173378418802674	-0.254853675540232	15
13275594	chr28	13250594	13300594	chr28	10253186	10303186	0.22788987550815	0.224232442293404	16
13375594	chr28	13350594	13400594	chr28	10353186	10403080	0.358499029487447	1.37212017663807	37
13400594	chr28	13375594	13425594	chr28	10378186	10428080	0.516750733423427	2.76295063800108	19
**13425594**	chr28	13400594	13450594	chr28	10403080	10453080	0.675983075220349	4.16239965432836	29
**13450594**	chr28	13425594	13475594	chr28	10428080	10478044	0.668590437640495	4.09742780707395	18
13475594	chr28	13450594	13500594	chr28	10453080	10503044	0.434254263789246	2.0379119807962	65
13500594	chr28	13475594	13525594	chr28	10478044	10528044	0.30834367911579	0.931318672690581	30
13525594	chr28	13500594	13550594	chr28	10503044	10553044	0.26392609253799	0.540944787947924	45
13550594	chr28	13525594	13575594	chr28	10528044	10578044	0.168082028453502	-0.301402185601631	16
13625594	chr28	13600594	13650594	chr28	10603044	10653044	0.323017269439989	1.06028080006656	42
13650594	chr28	13625594	13675594	chr28	10628044	10678044	0.301129531008174	0.867915520046385	28
13675594	chr28	13650594	13700594	chr28	10653044	10703044	0.418925309543874	1.90319004115801	18
13725594	chr28	13700594	13750594	chr28	10703044	10753044	0.344879129674009	1.25241864376292	34
13750594	chr28	13725594	13775594	chr28	10728044	10778044	0.390265225250085	1.65130448625012	15
13775594	chr28	13750594	13800594	chr28	10753044	10803044	0.415441647225411	1.87257309644833	29
13800594	chr28	13775594	13825594	chr28	10778044	10828044	0.317689736585898	1.01345858714189	19
13825594	chr28	13800594	13850594	chr28	10803044	10853044	0.452044756021461	2.19426769706208	58
13850594	chr28	13825594	13875594	chr28	10828044	10878044	0.460718910912852	2.2705024455638	30
13875594	chr28	13850594	13900594	chr28	10853044	10903044	0.272169507172635	0.613393879454382	86
13900594	chr28	13875594	13925594	chr28	10878044	10928044	0.203600978262248	0.0107640414970156	31
13925594	chr28	13900594	13950594	chr28	10903044	10953044	0.243616238131072	0.362447094258531	42
13950594	chr28	13925594	13975594	chr28	10928044	10978044	0.247335046213539	0.395130670058449	36
