# Supplementary material for: Genome-Wide Analyses Suggest Mechanisms Involving Early B-Cell Development in Canine IgA Deficiency
Source: PLoS One. 2015 Jul 30;10(7):e0133844. doi: 10.1371/journal.pone.0133844 (PMC4520476; doi:10.1371/journal.pone.0133844)
Supplement: S9 Table — (PDF) [file pone.0133844.s019.pdf]

Table S9. Z-transformed average pooled heterozygosity in dog in the CFA28 region

canfam2				canfam3									
middle	chr	start	end	chr	start	end	avg_het	z_score	avg_cov	maj	min	SNPs (N)	position
13075594	chr28	13050594	13100594	chr28	10053171	10103177	0.366469978829782	0.546325700325171	19.8666666666667	904	288	60	13075594
13100594	chr28	13075594	13125594	chr28	10078171	10128177	0.391221016619011	0.896250300740804	18.8666666666667	415	151	30	13100594
13125594	chr28	13100594	13150594	chr28	10103177	10153177	0.314287812004775	-0.191414027219057	18.9827586206897	886	215	58	13125594
13150594	chr28	13125594	13175594	chr28	10128177	10178177	0.33778971732596	0.14085061956277	19.3125	485	133	32	13150594
13175594	chr28	13150594	13200594	chr28	10153177	10203177	0.357606786242604	0.421019878771901	17.0958904109589	957	291	73	13175594
13200594	chr28	13175594	13225594	chr28	10178177	10228186	0.318860762336875	-0.126762684866982	17.5833333333333	338	84	24	13200594
13225594	chr28	13200594	13250594	chr28	10203177	10253186	0.423106497500961	1.3470400515258	20.4	213	93	15	13225594
13275594	chr28	13250594	13300594	chr28	10253186	10303186	0.370437139862054	0.602412529928991	21.5	292	95	18	13275594
13375594	chr28	13350594	13400594	chr28	10353186	10403080	0.261714714617769	-0.934680643044194	19.027027027027	595	109	37	13375594
13400594	chr28	13375594	13425594	chr28	10378186	10428080	0.170047248894986	-2.23065463764241	21.3157894736842	367	38	19	13400594
13425594	chr28	13400594	13450594	chr28	10403080	10453080	0.12030612244898	-2.93388348582496	24.1379310344828	655	45	29	13425594
13450594	chr28	13425594	13475594	chr28	10428080	10478044	0.142011834319527	-2.62701301769682	23.8333333333333	396	33	18	13450594
13475594	chr28	13450594	13500594	chr28	10453080	10503044	0.209069121364256	-1.67897218911004	17.7575757575758	1033	139	66	13475594
13500594	chr28	13475594	13525594	chr28	10478044	10528044	0.276065088757396	-0.731798283953402	16.7741935483871	434	86	31	13500594
13525594	chr28	13500594	13550594	chr28	10503044	10553044	0.311538212737819	-0.230287242677174	15.8723404255319	602	144	47	13525594
13550594	chr28	13525594	13575594	chr28	10528044	10578044	0.321662808641975	-0.0871479849902305	16	230	58	18	13550594
13625594	chr28	13600594	13650594	chr28	10603044	10653044	0.326356372930106	-0.0207914296798969	15.4565217391304	565	146	46	13625594
13650594	chr28	13625594	13675594	chr28	10628044	10678044	0.34652926410358	0.264408364495119	14.5	338	97	30	13650594
13675594	chr28	13650594	13700594	chr28	10653044	10703044	0.215312668298236	-1.59070232807819	21.2777777777778	336	47	18	13675594
13725594	chr28	13700594	13750594	chr28	10703044	10753044	0.271780146482761	-0.792377833224201	21.3142857142857	625	121	35	13725594
13750594	chr28	13725594	13775594	chr28	10728044	10778044	0.221508688577371	-1.50310438819355	22.6	296	43	15	13750594
13775594	chr28	13750594	13800594	chr28	10753044	10803044	0.251621934550575	-1.0773700955904	19.4516129032258	514	89	31	13775594
13800594	chr28	13775594	13825594	chr28	10778044	10828044	0.319305690133316	-0.120472395841157	16.4761904761905	277	69	21	13800594
13825594	chr28	13800594	13850594	chr28	10803044	10853044	0.290733786112601	-0.524415542900363	16.6166666666667	821	176	60	13825594
13850594	chr28	13825594	13875594	chr28	10828044	10878044	0.292053311120367	-0.505760395386433	15.8064516129032	403	87	31	13850594
13875594	chr28	13850594	13900594	chr28	10853044	10903044	0.400072942223596	1.02139683223053	15.9886363636364	1018	389	88	13875594
13900594	chr28	13875594	13925594	chr28	10878044	10928044	0.398627901729511	1.00096717502653	16.7575757575758	401	152	33	13900594
13925594	chr28	13900594	13950594	chr28	10903044	10953044	0.354157421333366	0.372253534191471	17.6976744186047	586	175	43	13925594
13950594	chr28	13925594	13975594	chr28	10928044	10978044	0.348710084449603	0.295240311610175	18.6216216216216	534	155	37	13950594
