# Supplementary material for: Genome-Wide Analyses Suggest Mechanisms Involving Early B-Cell Development in Canine IgA Deficiency
Source: PLoS One. 2015 Jul 30;10(7):e0133844. doi: 10.1371/journal.pone.0133844 (PMC4520476; doi:10.1371/journal.pone.0133844)
Supplement: S10 Table — (PDF) [file pone.0133844.s020.pdf]

Table S10. All GRAIL pathways from GWAS all breeds.

Region_id	Region	uncorr p-value	Genes	'pyruvate'	'lyase'	'oocyte'	'cyclooxygenase'	'prion'	'dermatitis'	'serum'	'insulin'	'atopic'	'matrix'	'ovarian'	'pregnancy'	'carcinoma'	'metalloproteinase'	'gastric'	'complement'	'oocytes'	'igfbp'	'invasion'	'sialic'
**GERMAN SHEPHERD**																							
ggsd6	chr8:63211755-63827575	0.098656727	*GSC*			X	X								X		X			X		X	
		0.13423564	*SERPINA1*					X		X		X	X	X	X	X	X	X	X				X
		0.064372074	*SERPINA3*						X	X			X			X		X	X		X	X	
		0.17150907	*SERPINA4*							X						X						X	
		0.18869457	*SERPINA6*							X	X			X	X			X					
		0.036794515	*SERPINA9*										X										
		0.018702784	*SERPINA12*										X		X								
ggsd7	chr10:69072184-69174348	0.12005742	*FIGLA*							X	X		X						X				
ggsd11	chr23:48439474-48539474	0.0018217369	*GPR149*										X										
ggsd12	chr27:37248047-37454652	0.014281463	*C3AR1*							X	X	X			X		X		X	X			
		0.15884016	*SLC2A3*	X		X					X				X	X				X			
ggsd13	chr5:29188112-29288253	0.17901195	*MMP7*				X		X	X	X			X	X	X							
		0.18210673	*TMEM123*																			X	
**GOLDEN RETRIEVER**																							
ggr1	chr6:18011946-18112312	0.048060378	*ALDOA*	X															X				
		0.12208519	*TBX6*								X												
ggr3	chr26:7114611-7846707	0.14609796	*DIABLO*	X										X		X		X		X			
		0.005586012	*IL31*						X	X		X	X			X							
ggr5	chr29:10808328-11803162	0.16904554	*CLVS1 / RLBP1L1*															X					
**SHAR PEI**																							
gsp1	chr28:10446800-13077479	0.075472269	*MORN4 / C10orf83*																X				
		0.01325097	*HOGA1 /C10orf65/NPL2*		X					X													
		0.16391053	*CHUK*								X				X	X	X					X	
		0.10485278	*CPN1*							X					X				X				
		0.10924305	*HPSE2*										X			X		X		X		X	
		0.10265851	*NKX2-3*							X	X								X				
		0.17215797	*SFRP5*											X	X	X		X				X	
gsp10	chr7:19623829-19723829	0.049370844	*PTGS2*			X		X		X			X	X	X	X	X	X			X	X	
gsp11	chr7:22714328-22814328	0.14052058	*ASTN1*							X													
		0.070564932	*PAPPA2*							X	X		X			X	X		X	X		X	
gsp8	chr7:16146250-16246197	0.16958668	*DHX9*											X					X				
		0.035098271	*NPL*		X																		
			**SUM GENES**	3	1	3	2	2	3	**14**	**9**	3	**10**	7	11	**12**	6	8	**10**	6	2	9	1
			**SUM REGIONS**	3	1	3	2	2	3	**8**	**7**	3	**7**	6	5	**7**	5	5	**7**	5	2	5	1
