# Supplementary material for: Genome-Wide Analyses Suggest Mechanisms Involving Early B-Cell Development in Canine IgA Deficiency
Source: PLoS One. 2015 Jul 30;10(7):e0133844. doi: 10.1371/journal.pone.0133844 (PMC4520476; doi:10.1371/journal.pone.0133844)
Supplement: S11 Table — (PDF) [file pone.0133844.s021.pdf]

Table S11. INRICH results* from IgA associated regions

T_Size	Int_No	Empirical_P	Corrected_P	Target	Description
622	6	3,00E-06	0.00182	GO:0003700	sequence-specific_DNA_binding_transcription_factor_activity
48	3	3,00E-06	0.00182	GO:0032355	response_to_estradiol_stimulus
44	3	9.99999e-06	0.00556999	GO:0001558	regulation_of_cell_growth
160	4	1.1e-05	0.00617299	GO:0000122	negative_regulation_of_transcription_from_RNA_polymerase_II_promoter
557	5	1.6e-05	0.00879199	GO:0006355	regulation_of_transcription,_DNA-dependent
19	2	8.39999e-05	0.034121	GO:0007015	actin_filament_organization
26	2	8.49999e-05	0.034596	GO:0031093	platelet_alpha_granule_lumen
124	3	0.000119	0.045301	GO:0005667	transcription_factor_complex
37	2	0.000151	0.0549709	GO:0034097	response_to_cytokine_stimulus
38	2	0.000314	0.0893319	GO:0016702	oxidoreductase_activity,_acting_on_single_donors_with_incorporation_of _molecular_oxygen,_incorporation_of_two_atoms_of_oxygen
52	2	0.000348	0.0950739	GO:0002576	platelet_degranulation
60	2	0.000428	0.104867	GO:0006006	glucose_metabolic_process
67	2	0.000477	0.10964	GO:0016829	lyase_activity
157	3	0.000547999	0.119056	GO:0030168	platelet_activation
64	2	0.000601999	0.124557	GO:0009653	anatomical_structure_morphogenesis
67	2	0.000601999	0.124557	GO:0032496	response_to_lipopolysaccharide
70	2	0.000727999	0.137657	GO:0014070	response_to_organic_cyclic_substance
39	2	0.000805999	0.146665	GO:0030097	hemopoiesis
321	3	0.001001	0.168941	GO:0043565	sequence-specific_DNA_binding
697	4	0.001055	0.172897	GO:0005730	nucleolus
230	3	0.00111	0.175702	GO:0007596	blood_coagulation
583	4	0.001131	0.177593	GO:0016787	hydrolase_activity
79	2	0.0012	0.185405	GO:0003690	double-stranded_DNA_binding
56	2	0.001235	0.187451	GO:0007389	pattern_specification_process
51	2	0.001313	0.19455	GO:0016477	cell_migration
75	2	0.001655	0.219867	GO:0004222	metalloendopeptidase_activity
364	3	0.002442	0.264807	GO:0006915	apoptosis
498	3	0.002715	0.281437	GO:0005654	nucleoplasm
113	2	0.00338	0.307749	GO:0005813	centrosome
499	3	0.003443	0.308913	GO:0005615	extracellular_space
112	2	0.00452	0.352829	GO:0043234	protein_complex
177	2	0.004555	0.35498	GO:0005975	carbohydrate_metabolic_process
192	2	0.004723	0.359966	GO:0030528	transcription_regulator_activity
80	2	0.00546999	0.382459	GO:0030036	actin_cytoskeleton_organization
134	2	0.00593299	0.398216	GO:0016568	chromatin_modification
145	2	0.00613999	0.407546	GO:0008134	transcription_factor_binding
439	3	0.00865799	0.456225	GO:0005856	cytoskeleton
106	2	0.00927499	0.466609	GO:0051056	regulation_of_small_GTPase_mediated_signal_transduction
263	2	0.011199	0.494678	GO:0016491	oxidoreductase_activity
166	2	0.011648	0.508221	GO:0005578	proteinaceous_extracellular_matrix
613	3	0.012557	0.516034	GO:0007275	multicellular_organismal_development
189	2	0.013636	0.521072	GO:0007264	small_GTPase_mediated_signal_transduction
321	2	0.01498	0.529507	GO:0055114	oxidation-reduction_process
268	2	0.016238	0.541168	GO:0006508	proteolysis
293	2	0.019572	0.577468	GO:0008233	peptidase_activity
198	2	0.020267	0.582399	GO:0003779	actin_binding
301	2	0.023215	0.609116	GO:0005488	binding
319	2	0.030068	0.638468	GO:0030154	cell_differentiation
345	2	0.030622	0.642941	GO:0005575	cellular_component
180	2	0.037092	0.676482	GO:0045202	synapse
909	3	0.047166	0.706319	GO:0005524	ATP_binding

**using 1M permutations and 5-1000 genes per gene sets.*
