# Supplementary material for: Genome-Wide Analyses Suggest Mechanisms Involving Early B-Cell Development in Canine IgA Deficiency
Source: PLoS One. 2015 Jul 30;10(7):e0133844. doi: 10.1371/journal.pone.0133844 (PMC4520476; doi:10.1371/journal.pone.0133844)
Supplement: S12 Table — (PDF) [file pone.0133844.s022.pdf]

```
----------------------------------------------------------------
INRICH v.1.0 : Mon Dec  1 11:23:35 2014
http://atgu.mgh.harvard.edu/inrich
----------------------------------------------------------------
       project-title  (-o)  :  5-1000_go_1000Kperm_canfam3.hg19_gwas
        test-regions  (-a)  :  gwasregions_canfam3new.int
           gene-list  (-g)  :  canfam3.hg19.genes.map
     background-genes (-b)  :  --no background set--
          range-file  (-x)  :  --no ranges--
         target-file  (-t)  :  go.test.set
 target size-filter  (-i,j) :  5..1000
    min-obs threshold (-z)  :  2
           test-type  (-1)  :  INTERVALS
       top-N-regions  (-n)  :   --all--
           kb-window  (-w)  :  0
            map-file  (-m)  :  my_markers_canfam3.snps
       match-density  (-d)  :  0.1
         pre-compute  (-c)  :  YES
         match-genes  (-e)  :  YES
      num-replicates  (-r)  :  1000000
      num-bootstraps  (-q)  :  1000000
         random-seed  (-f)  :  700081734
           display-p  (-p)  :  0.05
----------------------------------------------------------------

1768.89Mb total sequence length on 26 chromosomes
  read 133695 map positions
  read 13878 reference genes
  read 11197 unique genes/targets, in 9086 groups
  118754 total gene/group pairs
  6549 genes/targets not found in main gene-list
  after size filters, 2684 groups remaining
  read 21 intervals
  18 test elements on gene regions
  3 non-genic test elements dropped

After merging, 11883 non-overlapping reference genes
After merging, 10204 non-overlapping genes in target sets
After merging, 18 non-overlapping intervals

133695 SNP counts assigned
Precomputing acceptable positions 18
1000000 first-pass permutations ( completed )
1000000 second-pass permutations ( completed )

Total of 18 intervals tested for 2684 target sets

Proportion unplaced/self-placed interval/permutations = 0.000163056


----------------------------------------------------------------
T_Size  Int_No    Empirical_P Corrected_P     Target
----------------------------------------------------------------
   160       4        1.1e-05  0.00617299     GO:0000122
```

```
negative_regulation_of_transcription_from_RNA_polymerase_II_promoter
    44       3    9.99999e-06  0.00556999     GO:0001558
regulation_of_cell_growth
    52       2       0.000348   0.0950739     GO:0002576
platelet_degranulation
    79       2         0.0012    0.185405     GO:0003690 double-
stranded_DNA_binding
   622       6          3e-06     0.00182     GO:0003700 sequence-
specific_DNA_binding_transcription_factor_activity
   198       2       0.020267    0.582399     GO:0003779
actin_binding
    75       2       0.001655    0.219867     GO:0004222
metalloendopeptidase_activity
   301       2       0.023215    0.609116     GO:0005488 binding
   909       3       0.047166    0.706319     GO:0005524 ATP_binding
   345       2       0.030622    0.642941     GO:0005575
cellular_component
   166       2       0.011648    0.508221     GO:0005578
proteinaceous_extracellular_matrix
   499       3       0.003443    0.308913     GO:0005615
extracellular_space
   498       3       0.002715    0.281437     GO:0005654 nucleoplasm
   124       3       0.000119    0.045301     GO:0005667
transcription_factor_complex
   697       4       0.001055    0.172897     GO:0005730 nucleolus
   113       2        0.00338    0.307749     GO:0005813 centrosome
   439       3     0.00865799    0.456225     GO:0005856
cytoskeleton
   177       2       0.004555     0.35498     GO:0005975
carbohydrate_metabolic_process
    60       2       0.000428    0.104867     GO:0006006
glucose_metabolic_process
   557       5        1.6e-05  0.00879199     GO:0006355
regulation_of_transcription,_DNA-dependent
   268       2       0.016238    0.541168     GO:0006508 proteolysis
   364       3       0.002442    0.264807     GO:0006915 apoptosis
    19       2    8.39999e-05    0.034121     GO:0007015
actin_filament_organization
   189       2       0.013636    0.521072     GO:0007264
small_GTPase_mediated_signal_transduction
   613       3       0.012557    0.516034     GO:0007275
multicellular_organismal_development
    56       2       0.001235    0.187451     GO:0007389
pattern_specification_process
   230       3        0.00111    0.175702     GO:0007596
blood_coagulation
   145       2     0.00613999    0.407546     GO:0008134
transcription_factor_binding
   293       2       0.019572    0.577468     GO:0008233
peptidase_activity
    64       2    0.000601999    0.124557     GO:0009653
anatomical_structure_morphogenesis
    70       2    0.000727999    0.137657     GO:0014070
response_to_organic_cyclic_substance
```

```
    51       2       0.001313     0.19455     GO:0016477
cell_migration
   263       2       0.011199    0.494678     GO:0016491
oxidoreductase_activity
   134       2     0.00593299    0.398216     GO:0016568
chromatin_modification
    38       2       0.000314   0.0893319     GO:0016702
oxidoreductase_activity,_acting_on_single_donors_with_incorporation_
of_molecular_oxygen,_incorporation_of_two_atoms_of_oxygen
   583       4       0.001131    0.177593     GO:0016787
hydrolase_activity
    67       2       0.000477     0.10964     GO:0016829
lyase_activity
    80       2     0.00546999    0.382459     GO:0030036
actin_cytoskeleton_organization
    39       2    0.000805999    0.146665     GO:0030097 hemopoiesis
   319       2       0.030068    0.638468     GO:0030154
cell_differentiation
   157       3    0.000547999    0.119056     GO:0030168
platelet_activation
   192       2       0.004723    0.359966     GO:0030528
transcription_regulator_activity
    26       2    8.49999e-05    0.034596     GO:0031093
platelet_alpha_granule_lumen
    48       3          3e-06     0.00182     GO:0032355
response_to_estradiol_stimulus
    67       2    0.000601999    0.124557     GO:0032496
response_to_lipopolysaccharide
    37       2       0.000151   0.0549709     GO:0034097
response_to_cytokine_stimulus
   112       2        0.00452    0.352829     GO:0043234
protein_complex
   321       3       0.001001    0.168941     GO:0043565 sequence-
specific_DNA_binding
   180       2       0.037092    0.676482     GO:0045202 synapse
   106       2     0.00927499    0.466609     GO:0051056
regulation_of_small_GTPase_mediated_signal_transduction
   321       2        0.01498    0.529507     GO:0055114 oxidation-
reduction_process


-----------------------------------------------------------------
Target_P_Threshold     Uniq_Gene_No_in_Targets      Significance
-----------------------------------------------------------------
             0.001                          20       9.99999e-07
              0.01                          36       9.99999e-07
              0.05                          39       9.99999e-07



Writing output to
5-1000_go_1000Kperm_canfam3.hg19_gwas.out.inrich ...
```

I. Main Analysis Results

_01	T_TARG	N_INT	P	PCORR	LOW_WARN	UNSH	TARGET
_01	5	0	1	1	0	0	GO:0000038 Very_long-chain_fatty_acid_metabolic_process
_01	9	0	1	1	0	0	GO:0000045 Autophagic_vacuole_assembly
_01	13	0	1	1	0	0	GO:0000049 TRNA_binding
_01	9	0	1	1	0	0	GO:0000050 Urea_cycle
_01	14	0	1	1	0	0	GO:0000060 Protein_import_into_nucleus,_translocation
_01	10	0	1	1	0	0	GO:0000062 Acyl-CoA_binding
_01	83	1	1	1	0	0.000423	GO:0000075 Cell_cycle_checkpoint
_01	9	0	1	1	0	0	GO:0000077 DNA_damage_checkpoint
_01	24	0	1	1	0	0	GO:0000079 Regulation_of_cyclin-dependent_protein_kinase_activity
_01	16	0	1	1	0	0	GO:0000080 G1_phase_of_mitotic_cell_cycle
_01	93	0	1	1	0	0	GO:0000082 G1/S_transition_of_mitotic_cell_cycle
_01	16	0	1	1	0	0	GO:0000083 Regulation_of_transcription_involved_in_G1/S_phase_of_mitotic_cell_cycle
_01	74	0	1	1	0	0	GO:0000084 S_phase_of_mitotic_cell_cycle
_01	6	0	1	1	0	0	GO:0000085 G2_phase_of_mitotic_cell_cycle
_01	65	1	1	1	0	0.000423	GO:0000086 G2/M_transition_of_mitotic_cell_cycle
_01	48	0	1	1	0	0	GO:0000087 M_phase_of_mitotic_cell_cycle
_01	16	0	1	1	0	0	GO:0000118 Histone_deacetylase_complex
_01	160	4	1.1e-05	0.00617299	0	0.000205	GO:0000122 Negative_regulation_of_transcription_from_RNA_polymerase_II_promoter
_01	5	0	1	1	0	0	GO:0000123 Histone_acetyltransferase_complex
_01	5	0	1	1	0	0	GO:0000132 Establishment_of_mitotic_spindle_orientation
_01	259	1	1	1	0	0.000423	GO:0000139 Golgi_membrane
_01	7	0	1	1	0	0	GO:0000145 Exocyst
_01	11	0	1	1	0	0	GO:0000146 Microfilament_motor_activity
_01	6	0	1	1	0	0	GO:

0000149 SNARE_binding
_01 38 0 1 1 0 0 GO:
0000151 Ubiquitin_ligase_complex
_01 9 0 1 1 0 0 GO:
0000155 Two-component_sensor_activity
_01 15 0 1 1 0 0 GO:
0000159 Protein_phosphatase_type_2A_complex
_01 32 0 1 1 0 0 GO:
0000165 MAPKKK_cascade
_01 11 0 1 1 0 0 GO:
0000175 3'-5'-exoribonuclease_activity
_01 8 0 1 1 0 0 GO:
0000178 Exosome_(RNase_complex)
_01 5 0 1 1 0 0 GO:
0000183 Chromatin_silencing_at_rDNA
_01 23 0 1 1 0 0 GO:
0000184 Nuclear-transcribed_mRNA_catabolic_process,_nonsense-
mediated_decay
_01 30 1 1 1 0 5e-05 GO:
0000186 Activation_of_MAPKK_activity
_01 59 0 1 1 0 0 GO:
0000187 Activation_of_MAPK_activity
_01 13 0 1 1 0 0 GO:
0000188 Inactivation_of_MAPK_activity
_01 28 0 1 1 0 0 GO:
0000209 Protein_polyubiquitination
_01 54 0 1 1 0 0 GO:
0000216 M/G1_transition_of_mitotic_cell_cycle
_01 22 1 1 1 0 5e-05 GO:
0000226 Microtubule_cytoskeleton_organization
_01 18 0 1 1 0 0 GO:
0000228 Nuclear_chromosome
_01 44 0 1 1 0 0 GO:
0000236 Mitotic_prometaphase
_01 9 0 1 1 0 0 GO:
0000242 Pericentriolar_material
_01 9 0 1 1 0 0 GO:
0000245 Spliceosome_assembly
_01 5 0 1 1 0 0 GO:
0000266 Mitochondrial_fission
_01 193 1 1 1 0 0.000423 GO:
0000278 Mitotic_cell_cycle
_01 106 0 1 1 0 0 GO:
0000287 Magnesium_ion_binding
_01 32 0 1 1 0 0 GO:
0000288 Nuclear-transcribed_mRNA_catabolic_process,_deadenylation-
dependent_decay
_01 14 0 1 1 0 0 GO:
0000289 Nuclear-transcribed_mRNA_poly(A)_tail_shortening
_01 11 0 1 1 0 0 GO:
0000299 Integral_to_membrane_of_membrane_fraction
_01 6 0 1 1 0 0 GO:
0000302 Response_to_reactive_oxygen_species
_01 6 0 1 1 0 0 GO:

0000307 Cyclin-dependent_protein_kinase_holoenzyme_complex
_01 6 0 1 1 0 0 GO:
0000346 Transcription_export_complex
_01 16 0 1 1 0 0 GO:
0000375 RNA_splicing,_via_transesterification_reactions
_01 7 0 1 1 0 0 GO:
0000381
Regulation_of_alternative_nuclear_mRNA_splicing,_via_spliceosome
_01 14 0 1 1 0 0 GO:
0000387 Spliceosomal_snRNP_assembly
_01 89 1 1 1 0 5.2e-05 GO:
0000398 Nuclear_mRNA_splicing,_via_spliceosome
_01 6 0 1 1 0 0 GO:
0000405 Bubble_DNA_binding
_01 9 0 1 1 0 0 GO:
0000421 Autophagic_vacuole_membrane
_01 41 0 1 1 0 0 GO:
0000502 Proteasome_complex
_01 12 0 1 1 0 0 GO:
0000718 Nucleotide-excision_repair,_DNA_damage_removal
_01 14 0 1 1 0 0 GO:
0000722 Telomere_maintenance_via_recombination
_01 28 0 1 1 0 0 GO:
0000723 Telomere_maintenance
_01 16 0 1 1 0 0 GO:
0000724 Double-strand_break_repair_via_homologous_recombination
_01 5 0 1 1 0 0 GO:
0000731 DNA_synthesis_involved_in_DNA_repair
_01 32 0 1 1 0 0 GO:
0000775 Chromosome,_centromeric_region
_01 31 0 1 1 0 0 GO:
0000776 Kinetochore
_01 35 0 1 1 0 0 GO:
0000777 Condensed_chromosome_kinetochore
_01 7 0 1 1 0 0 GO:
0000780 Condensed_nuclear_chromosome,_centromeric_region
_01 18 0 1 1 0 0 GO:
0000781 Chromosome,_telomeric_region
_01 5 0 1 1 0 0 GO:
0000783 Nuclear_telomere_cap_complex
_01 8 0 1 1 0 0 GO:
0000784 Nuclear_chromosome,_telomeric_region
_01 62 1 1 1 0 7.4e-05 GO:
0000785 Chromatin
_01 26 0 1 1 0 0 GO:
0000786 Nucleosome
_01 15 0 1 1 0 0 GO:
0000790 Nuclear_chromatin
_01 15 0 1 1 0 0 GO:
0000793 Condensed_chromosome
_01 11 0 1 1 0 0 GO:
0000794 Condensed_nuclear_chromosome
_01 7 0 1 1 0 0 GO:
0000795 Synaptonemal_complex

_01	21	0	1	1	0	0	GO:
0000902 Cell_morphogenesis
_01	29	0	1	1	0	0	GO:
0000910 Cytokinesis
_01	36	0	1	1	0	0	GO:
0000922 Spindle_pole
_01	19	0	1	1	0	0	GO:
0000932 Cytoplasmic_mRNA_processing_body
_01	6	0	1	1	0	0	GO:
0001302 Replicative_cell_aging
_01	82	0	1	1	0	0	GO:
0001501 Skeletal_system_development
_01	12	0	1	1	0	0	GO:
0001502 Cartilage_condensation
_01	41	0	1	1	0	0	GO:
0001503 Ossification
_01	7	0	1	1	0	0	GO:
0001508 Regulation_of_action_potential
_01	9	1	1	1	0	3.9e-05	GO:
0001516 Prostaglandin_biosynthetic_process
_01	6	0	1	1	0	0	GO:
0001518 Voltage-gated_sodium_channel_complex
_01	7	0	1	1	0	0	GO:
0001522 Pseudouridine_synthesis
_01	86	0	1	1	0	0	GO:
0001525 Angiogenesis
_01	10	0	1	1	0	0	GO:
0001530 Lipopolysaccharide_binding
_01	14	0	1	1	0	0	GO:
0001533 Cornified_envelope
_01	6	0	1	1	0	0	GO:
0001539 Ciliary_or_flagellar_motility
_01	13	0	1	1	0	0	GO:
0001540 Beta-amyloid_binding
_01	26	0	1	1	0	0	GO:
0001541 Ovarian_follicle_development
_01	9	0	1	1	0	0	GO:
0001542 Ovulation_from_ovarian_follicle
_01	6	0	1	1	0	0	GO:
0001553 Luteinization
_01	9	0	1	1	0	0	GO:
0001556 Oocyte_maturation
_01	44	3	9.99999e-06	0.00556999	0
5.53333e-05	GO:0001558 Regulation_of_cell_growth
_01	28	0	1	1	0	0	GO:
0001568 Blood_vessel_development
_01	17	0	1	1	0	0	GO:
0001569 Patterning_of_blood_vessels
_01	33	0	1	1	0	0	GO:
0001570 Vasculogenesis
_01	7	0	1	1	0	0	GO:
0001578 Microtubule_bundle_formation
_01	5	0	1	1	0	0	GO:
0001609 Adenosine_receptor_activity,_G-protein_coupled

_01	7	0	1	1	0	0	GO: 0001614 Purinergic_nucleotide_receptor_activity
_01	11	0	1	1	0	0	GO: 0001619 Lysosphingolipid_and_lysophosphatidic_acid_receptor_activity
_01	25	0	1	1	0	0	GO: 0001649 Osteoblast_differentiation
_01	21	0	1	1	0	0	GO: 0001656 Metanephros_development
_01	29	0	1	1	0	0	GO: 0001657 Ureteric_bud_development
_01	26	0	1	1	0	0	GO: 0001658 Branching_involved_in_ureteric_bud_morphogenesis
_01	14	0	1	1	0	0	GO: 0001659 Temperature_homeostasis
_01	9	0	1	1	0	0	GO: 0001662 Behavioral_fear_response
_01	27	0	1	1	0	0	GO: 0001664 G-protein-coupled_receptor_binding
_01	101	1	1	1	0	0.000339	GO: 0001666 Response_to_hypoxia
_01	20	0	1	1	0	0	GO: 0001669 Acrosomal_vesicle
_01	10	0	1	1	0	0	GO: 0001673 Male_germ_cell_nucleus
_01	6	0	1	1	0	0	GO: 0001676 Long-chain_fatty_acid_metabolic_process
_01	103	0	1	1	0	0	GO: 0001701 In_utero_embryonic_development
_01	15	0	1	1	0	0	GO: 0001702 Gastrulation_with_mouth_forming_second
_01	17	1	1	1	0	5e-05	GO: 0001707 Mesoderm_formation
_01	9	1	1	1	0	5e-05	GO: 0001708 Cell_fate_specification
_01	10	0	1	1	0	0	GO: 0001709 Cell_fate_determination
_01	20	0	1	1	0	0	GO: 0001725 Stress_fiber
_01	40	0	1	1	0	0	GO: 0001726 Ruffle
_01	6	0	1	1	0	0	GO: 0001741 XY_body
_01	12	0	1	1	0	0	GO: 0001750 Photoreceptor_outer_segment
_01	17	0	1	1	0	0	GO: 0001755 Neural_crest_cell_migration
_01	32	0	1	1	0	0	GO: 0001756 Somitogenesis
_01	11	0	1	1	0	0	GO: 0001759 Organ_induction
_01	36	0	1	1	0	0	GO: 0001764 Neuron_migration
_01	11	0	1	1	0	0	GO: 0001772 Immunological_synapse

```
_01     7       0       1       1       0       0       GO:
0001782 B_cell_homeostasis
_01     8       0       1       1       0       0       GO:
0001784 Phosphotyrosine_binding
_01     8       0       1       1       0       0       GO:
0001786 Phosphatidylserine_binding
_01     8       0       1       1       0       0       GO:
0001816 Cytokine_production
_01     5       0       1       1       0       0       GO:
0001817 Regulation_of_cytokine_production
_01     7       0       1       1       0       0       GO:
0001819 Positive_regulation_of_cytokine_production
_01     48      0       1       1       0       0       GO:
0001822 Kidney_development
_01     6       0       1       1       0       0       GO:
0001823 Mesonephros_development
_01     9       0       1       1       0       0       GO:
0001824 Blastocyst_development
_01     7       0       1       1       0       0       GO:
0001829 Trophectodermal_cell_differentiation
_01     17      0       1       1       0       0       GO:
0001836 Release_of_cytochrome_c_from_mitochondria
_01     16      0       1       1       0       0       GO:
0001837 Epithelial_to_mesenchymal_transition
_01     6       0       1       1       0       0       GO:
0001841 Neural_tube_formation
_01     25      0       1       1       0       0       GO:
0001843 Neural_tube_closure
_01     5       0       1       1       0       0       GO:
0001882 Nucleoside_binding
_01     42      0       1       1       0       0       GO:
0001889 Liver_development
_01     13      0       1       1       0       0       GO:
0001890 Placenta_development
_01     11      0       1       1       0       0       GO:
0001892 Embryonic_placenta_development
_01     7       0       1       1       0       0       GO:
0001894 Tissue_homeostasis
_01     7       0       1       1       0       0       GO:
0001917 Photoreceptor_inner_segment
_01     9       0       1       1       0       0       GO:
0001932 Regulation_of_protein_phosphorylation
_01     16      0       1       1       0       0       GO:
0001933 Negative_regulation_of_protein_phosphorylation
_01     30      0       1       1       0       0       GO:
0001934 Positive_regulation_of_protein_phosphorylation
_01     6       0       1       1       0       0       GO:
0001935 Endothelial_cell_proliferation
_01     11      0       1       1       0       0       GO:
0001937 Negative_regulation_of_endothelial_cell_proliferation
_01     28      0       1       1       0       0       GO:
0001938 Positive_regulation_of_endothelial_cell_proliferation
_01     11      0       1       1       0       0       GO:
0001942 Hair_follicle_development
```

_01 5 0 1 1 0 0 GO:
0001944 Vasculature_development
_01 6 0 1 1 0 0 GO:
0001946 Lymphangiogenesis
_01 22 0 1 1 0 0 GO:
0001947 Heart_looping
_01 14 0 1 1 0 0 GO:
0001948 Glycoprotein_binding
_01 6 0 1 1 0 0 GO:
0001952 Regulation_of_cell-matrix_adhesion
_01 5 0 1 1 0 0 GO:
0001953 Negative_regulation_of_cell-matrix_adhesion
_01 9 0 1 1 0 0 GO:
0001954 Positive_regulation_of_cell-matrix_adhesion
_01 5 0 1 1 0 0 GO:
0001955 Blood_vessel_maturation
_01 5 0 1 1 0 0 GO:
0001957 Intramembranous_ossification
_01 8 0 1 1 0 0 GO:
0001958 Endochondral_ossification
_01 5 0 1 1 0 0 GO:
0001960 Negative_regulation_of_cytokine-mediated_signaling_pathway
_01 5 0 1 1 0 0 GO:
0001963 Synaptic_transmission,_dopaminergic
_01 6 0 1 1 0 0 GO:
0001965 G-protein_alpha-subunit_binding
_01 7 0 1 1 0 0 GO:
0001968 Fibronectin_binding
_01 7 0 1 1 0 0 GO:
0001972 Retinoic_acid_binding
_01 16 0 1 1 0 0 GO:
0001974 Blood_vessel_remodeling
_01 14 0 1 1 0 0 GO:
0001975 Response_to_amphetamine
_01 9 0 1 1 0 0 GO:
0002009 Morphogenesis_of_an_epithelium
_01 5 0 1 1 0 0 GO:
0002011 Morphogenesis_of_an_epithelial_sheet
_01 20 1 1 1 0 0.000339 GO:
0002020 Protease_binding
_01 12 0 1 1 0 0 GO:
0002026 Regulation_of_the_force_of_heart_contraction
_01 8 0 1 1 0 0 GO:
0002027 Regulation_of_heart_rate
_01 6 0 1 1 0 0 GO:
0002028 Regulation_of_sodium_ion_transport
_01 17 0 1 1 0 0 GO:
0002039 P53_binding
_01 7 0 1 1 0 0 GO:
0002040 Sprouting_angiogenesis
_01 7 0 1 1 0 0 GO:
0002052 Positive_regulation_of_neuroblast_proliferation
_01 23 0 1 1 0 0 GO:
0002053 Positive_regulation_of_mesenchymal_cell_proliferation

_01 7 0 1 1 0 0 GO:
0002062 Chondrocyte_differentiation
_01 5 0 1 1 0 0 GO:
0002063 Chondrocyte_development
_01 9 0 1 1 0 0 GO:
0002076 Osteoblast_development
_01 5 0 1 1 0 0 GO:
0002087
Regulation_of_respiratory_gaseous_exchange_by_neurological_system_pr
ocess
_01 10 0 1 1 0 0 GO:
0002088 Lens_development_in_camera-type_eye
_01 5 0 1 1 0 0 GO:
0002102 Podosome
_01 7 0 1 1 0 0 GO:
0002244 Hemopoietic_progenitor_cell_differentiation
_01 5 0 1 1 0 0 GO:
0002446 Neutrophil_mediated_immunity
_01 6 0 1 1 0 0 GO:
0002548 Monocyte_chemotaxis
_01 52 2 0.000348 0.0950739 0
0.0001945 GO:0002576 Platelet_degranulation
_01 5 0 1 1 0 0 GO:
0002675 Positive_regulation_of_acute_inflammatory_response
_01 5 0 1 1 0 0 GO:
0002687 Positive_regulation_of_leukocyte_migration
_01 40 0 1 1 0 0 GO:
0002756 MyD88-independent_toll-like_receptor_signaling_pathway
_01 5 0 1 1 0 0 GO:
0002925
Positive_regulation_of_humoral_immune_response_mediated_by_circulati
ng_immunoglobulin
_01 23 0 1 1 0 0 GO:
0003007 Heart_morphogenesis
_01 7 0 1 1 0 0 GO:
0003009 Skeletal_muscle_contraction
_01 5 0 1 1 0 0 GO:
0003016 Respiratory_system_process
_01 9 0 1 1 0 0 GO:
0003151 Outflow_tract_morphogenesis
_01 5 0 1 1 0 0 GO:
0003281 Ventricular_septum_development
_01 5 0 1 1 0 0 GO:
0003337
Mesenchymal_to_epithelial_transition_involved_in_metanephros_morphog
enesis
_01 397 1 1 1 0 0.000423 GO:
0003674 Molecular_function
_01 175 1 1 1 0 5.9e-05 GO:
0003676 Nucleic_acid_binding
_01 9 0 1 1 0 0 GO:
0003678 DNA_helicase_activity
_01 106 0 1 1 0 0 GO:
0003682 Chromatin_binding

```
_01	31	0	1	1	0	0	GO:
0003684 Damaged_DNA_binding
_01	79	2	0.0012	0.185405	0	6.85e-05	GO:
0003690 Double-stranded_DNA_binding
_01	31	0	1	1	0	0	GO:
0003697 Single-stranded_DNA_binding
_01	622	6	3e-06	0.00182	0	0.0002155
GO:0003700 Sequence-
specific_DNA_binding_transcription_factor_activity
_01	92	1	1	1	0	0.000344	GO:
0003702 RNA_polymerase_II_transcription_factor_activity
_01	52	1	1	1	0	6.3e-05	GO:
0003704 Specific_RNA_polymerase_II_transcription_factor_activity
_01	56	1	1	1	0	6.3e-05	GO:
0003705 Sequence-
specific_enhancer_binding_RNA_polymerase_II_transcription_factor_act
ivity
_01	5	0	1	1	0	0	GO:
0003706 Ligand-regulated_transcription_factor_activity
_01	32	0	1	1	0	0	GO:
0003707 Steroid_hormone_receptor_activity
_01	5	0	1	1	0	0	GO:
0003711 Transcription_elongation_regulator_activity
_01	25	0	1	1	0	0	GO:
0003712 Transcription_cofactor_activity
_01	130	0	1	1	0	0	GO:
0003713 Transcription_coactivator_activity
_01	99	0	1	1	0	0	GO:
0003714 Transcription_corepressor_activity
_01	394	1	1	1	0	0.000423	GO:
0003723 RNA_binding
_01	7	1	1	1	0	5.2e-05	GO:
0003724 RNA_helicase_activity
_01	19	1	1	1	0	5.2e-05	GO:
0003725 Double-stranded_RNA_binding
_01	8	0	1	1	0	0	GO:
0003727 Single-stranded_RNA_binding
_01	29	0	1	1	0	0	GO:
0003729 MRNA_binding
_01	14	0	1	1	0	0	GO:
0003730 MRNA_3'-UTR_binding
_01	109	0	1	1	0	0	GO:
0003735 Structural_constituent_of_ribosome
_01	34	0	1	1	0	0	GO:
0003743 Translation_initiation_factor_activity
_01	20	0	1	1	0	0	GO:
0003746 Translation_elongation_factor_activity
_01	25	0	1	1	0	0	GO:
0003755 Peptidyl-prolyl_cis-trans_isomerase_activity
_01	5	0	1	1	0	0	GO:
0003756 Protein_disulfide_isomerase_activity
_01	50	0	1	1	0	0	GO:
0003774 Motor_activity
_01	50	0	1	1	0	0	GO:
```

0003777 Microtubule_motor_activity
_01 198 2 0.020267 0.582399 0 5.95e-05 GO:
0003779 Actin_binding
_01 7 0 1 1 0 0 GO:
0003785 Actin_monomer_binding
_01 5 0 1 1 0 0 GO:
0003796 Lysozyme_activity
_01 28 0 1 1 0 0 GO:
0003823 Antigen_binding
_01 72 0 1 1 0 0 GO:
0003824 Catalytic_activity
_01 3 0 1 1 0 0 GO:
0003840 Gamma-glutamyltransferase_activity
_01 7 0 1 1 0 0 GO:
0003841 1-acylglycerol-3-phosphate_O-acyltransferase_activity
_01 5 0 1 1 0 0 GO:
0003847 1-alkyl-2-acetylglycerophosphocholine_esterase_activity
_01 16 0 1 1 0 0 GO:
0003887 DNA-directed_DNA_polymerase_activity
_01 22 0 1 1 0 0 GO:
0003899 DNA-directed_RNA_polymerase_activity
_01 7 0 1 1 0 0 GO:
0003906 DNA-(apurinic_or_apyrimidinic_site)_lyase_activity
_01 145 1 1 1 0 0.000423 GO:
0003924 GTPase_activity
_01 12 0 1 1 0 0 GO:
0003950 NAD+_ADP-ribosyltransferase_activity
_01 5 0 1 1 0 0 GO:
0003954 NADH_dehydrogenase_activity
_01 7 0 1 1 0 0 GO:
0003993 Acid_phosphatase_activity
_01 7 0 1 1 0 0 GO:
0003995 Acyl-CoA_dehydrogenase_activity
_01 15 1 1 1 0 5.2e-05 GO:
0004003 ATP-dependent_DNA_helicase_activity
_01 11 1 1 1 0 5.2e-05 GO:
0004004 ATP-dependent_RNA_helicase_activity
_01 9 0 1 1 0 0 GO:
0004012 Phospholipid-translocating_ATPase_activity
_01 9 0 1 1 0 0 GO:
0004016 Adenylate_cyclase_activity
_01 6 0 1 1 0 0 GO:
0004017 Adenylate_kinase_activity
_01 7 0 1 1 0 0 GO:
0004029 Aldehyde_dehydrogenase_(NAD)_activity
_01 5 0 1 1 0 0 GO:
0004033 Aldo-keto_reductase_activity
_01 3 0 1 1 0 0 GO:
0004035 Alkaline_phosphatase_activity
_01 6 0 1 1 0 0 GO:
0004065 Arylsulfatase_activity
_01 6 0 1 1 0 0 GO:
0004089 Carbonate_dehydratase_activity
_01 16 0 1 1 0 0 GO:

0004091 Carboxylesterase_activity
_01 5 0 1 1 0 0 GO:
0004114 3',5'-cyclic-nucleotide_phosphodiesterase_activity
_01 9 0 1 1 0 0 GO:
0004115 3',5'-cyclic-AMP_phosphodiesterase_activity
_01 6 0 1 1 0 0 GO:
0004128 Cytochrome-b5_reductase_activity
_01 18 0 1 1 0 0 GO:
0004129 Cytochrome-c_oxidase_activity
_01 11 0 1 1 0 0 GO:
0004143 Diacylglycerol_kinase_activity
_01 10 0 1 1 0 0 GO:
0004175 Endopeptidase_activity
_01 18 0 1 1 0 0 GO:
0004177 Aminopeptidase_activity
_01 14 0 1 1 0 0 GO:
0004180 Carboxypeptidase_activity
_01 17 0 1 1 0 0 GO:
0004181 Metallocarboxypeptidase_activity
_01 5 0 1 1 0 0 GO:
0004185 Serine-type_carboxypeptidase_activity
_01 11 0 1 1 0 0 GO:
0004190 Aspartic-type_endopeptidase_activity
_01 34 0 1 1 0 0 GO:
0004197 Cysteine-type_endopeptidase_activity
_01 10 0 1 1 0 0 GO:
0004198 Calcium-dependent_cysteine-type_endopeptidase_activity
_01 36 0 1 1 0 0 GO:
0004221 Ubiquitin_thiolesterase_activity
_01 75 2 0.001655 0.219867 0 7.35e-05 GO:
0004222 Metalloendopeptidase_activity
_01 94 0 1 1 0 0 GO:
0004252 Serine-type_endopeptidase_activity
_01 14 0 1 1 0 0 GO:
0004298 Threonine-type_endopeptidase_activity
_01 5 0 1 1 0 0 GO:
0004303 Estradiol_17-beta-dehydrogenase_activity
_01 8 0 1 1 0 0 GO:
0004364 Glutathione_transferase_activity
_01 10 0 1 1 0 0 GO:
0004383 Guanylate_cyclase_activity
_01 6 0 1 1 0 0 GO:
0004385 Guanylate_kinase_activity
_01 68 1 1 1 0 5.9e-05 GO:
0004386 Helicase_activity
_01 28 0 1 1 0 0 GO:
0004402 Histone_acetyltransferase_activity
_01 9 0 1 1 0 0 GO:
0004407 Histone_deacetylase_activity
_01 6 0 1 1 0 0 GO:
0004428 Inositol_or_phosphatidylinositol_kinase_activity
_01 19 0 1 1 0 0 GO:
0004435 Phosphoinositide_phospholipase_C_activity
_01 8 0 1 1 0 0 GO:

0004437 Inositol_or_phosphatidylinositol_phosphatase_activity
_01 5 0 1 1 0 0 GO:
0004445 Inositol-polyphosphate_5-phosphatase_activity
_01 5 0 1 1 0 0 GO:
0004467 Long-chain_fatty_acid-CoA_ligase_activity
_01 33 0 1 1 0 0 GO:
0004497 Monooxygenase_activity
_01 5 0 1 1 0 0 GO:
0004500 Dopamine_beta-monooxygenase_activity
_01 10 0 1 1 0 0 GO:
0004518 Nuclease_activity
_01 45 0 1 1 0 0 GO:
0004519 Endonuclease_activity
_01 6 0 1 1 0 0 GO:
0004520 Endodeoxyribonuclease_activity
_01 10 0 1 1 0 0 GO:
0004522 Pancreatic_ribonuclease_activity
_01 5 0 1 1 0 0 GO:
0004523 Ribonuclease_H_activity
_01 5 0 1 1 0 0 GO:
0004526 Ribonuclease_P_activity
_01 26 0 1 1 0 0 GO:
0004527 Exonuclease_activity
_01 5 0 1 1 0 0 GO:
0004536 Deoxyribonuclease_activity
_01 11 0 1 1 0 0 GO:
0004540 Ribonuclease_activity
_01 6 0 1 1 0 0 GO:
0004550 Nucleoside_diphosphate_kinase_activity
_01 8 0 1 1 0 0 GO:
0004553 Hydrolase_activity,_hydrolyzing_O-glycosyl_compounds
_01 6 0 1 1 0 0 GO:
0004571 Mannosyl-oligosaccharide_1,2-alpha-mannosidase_activity
_01 6 0 1 1 0 0 GO:
0004579 Dolichyl-diphosphooligosaccharide-
protein_glycotransferase_activity
_01 10 1 1 1 0 3.9e-05 GO:
0004601 Peroxidase_activity
_01 8 0 1 1 0 0 GO:
0004602 Glutathione_peroxidase_activity
_01 5 0 1 1 0 0 GO:
0004614 Phosphoglucomutase_activity
_01 7 0 1 1 0 0 GO:
0004622 Lysophospholipase_activity
_01 13 0 1 1 0 0 GO:
0004623 Phospholipase_A2_activity
_01 5 0 1 1 0 0 GO:
0004629 Phospholipase_C_activity
_01 5 0 1 1 0 0 GO:
0004652 Polynucleotide_adenylyltransferase_activity
_01 12 0 1 1 0 0 GO:
0004653 Polypeptide_N-acetylgalactosaminyltransferase_activity
_01 112 0 1 1 0 0 GO:
0004672 Protein_kinase_activity

_01 205 1 1 1 0 5e-05 GO:
0004674 Protein_serine/threonine_kinase_activity
_01 5 0 1 1 0 0 GO:
0004675 Transmembrane_receptor_protein_serine/
threonine_kinase_activity
_01 10 0 1 1 0 0 GO:
0004683 Calmodulin-dependent_protein_kinase_activity
_01 4 0 1 1 0 0 GO:
0004691 CAMP-dependent_protein_kinase_activity
_01 15 0 1 1 0 0 GO:
0004693 Cyclin-dependent_protein_kinase_activity
_01 11 0 1 1 0 0 GO:
0004697 Protein_kinase_C_activity
_01 11 0 1 1 0 0 GO:
0004707 MAP_kinase_activity
_01 8 0 1 1 0 0 GO:
0004708 MAP_kinase_kinase_activity
_01 11 0 1 1 0 0 GO:
0004709 MAP_kinase_kinase_kinase_activity
_01 54 0 1 1 0 0 GO:
0004713 Protein_tyrosine_kinase_activity
_01 25 0 1 1 0 0 GO:
0004714 Transmembrane_receptor_protein_tyrosine_kinase_activity
_01 25 0 1 1 0 0 GO:
0004715 Non-membrane_spanning_protein_tyrosine_kinase_activity
_01 7 0 1 1 0 0 GO:
0004716 Receptor_signaling_protein_tyrosine_kinase_activity
_01 25 0 1 1 0 0 GO:
0004721 Phosphoprotein_phosphatase_activity
_01 20 1 1 1 0 5e-05 GO:
0004722 Protein_serine/threonine_phosphatase_activity
_01 57 0 1 1 0 0 GO:
0004725 Protein_tyrosine_phosphatase_activity
_01 7 0 1 1 0 0 GO:
0004767 Sphingomyelin_phosphodiesterase_activity
_01 5 0 1 1 0 0 GO:
0004791 Thioredoxin-disulfide_reductase_activity
_01 8 0 1 1 0 0 GO:
0004806 Triglyceride_lipase_activity
_01 8 0 1 1 0 0 GO:
0004835 Tubulin-tyrosine_ligase_activity
_01 126 1 1 1 0 0.000423 GO:
0004842 Ubiquitin-protein_ligase_activity
_01 18 0 1 1 0 0 GO:
0004843 Ubiquitin-specific_protease_activity
_01 16 0 1 1 0 0 GO:
0004857 Enzyme_inhibitor_activity
_01 7 0 1 1 0 0 GO:
0004859 Phospholipase_inhibitor_activity
_01 14 0 1 1 0 0 GO:
0004860 Protein_kinase_inhibitor_activity
_01 9 0 1 1 0 0 GO:
0004861 Cyclin-dependent_protein_kinase_inhibitor_activity
_01 13 0 1 1 0 0 GO:

0004864 Phosphoprotein_phosphatase_inhibitor_activity
_01 16 0 1 1 0 0 GO:
0004866 Endopeptidase_inhibitor_activity
_01 58 1 1 1 0 0.000339 GO:
0004867 Serine-type_endopeptidase_inhibitor_activity
_01 12 0 1 1 0 0 GO:
0004869 Cysteine-type_endopeptidase_inhibitor_activity
_01 188 0 1 1 0 0 GO:
0004871 Signal_transducer_activity
_01 862 1 1 1 0 5.9e-05 GO:
0004872 Receptor_activity
_01 20 0 1 1 0 0 GO:
0004879 Ligand-dependent_nuclear_receptor_activity
_01 6 0 1 1 0 0 GO:
0004887 Thyroid_hormone_receptor_activity
_01 73 0 1 1 0 0 GO:
0004888 Transmembrane_receptor_activity
_01 11 0 1 1 0 0 GO:
0004889 Nicotinic_acetylcholine-activated_cation-
selective_channel_activity
_01 13 0 1 1 0 0 GO:
0004890 GABA-A_receptor_activity
_01 11 0 1 1 0 0 GO:
0004896 Cytokine_receptor_activity
_01 5 0 1 1 0 0 GO:
0004908 Interleukin-1_receptor_activity
_01 242 1 1 1 0 5.9e-05 GO:
0004930 G-protein_coupled_receptor_activity
_01 7 0 1 1 0 0 GO:
0004931 Extracellular_ATP-gated_cation_channel_activity
_01 6 0 1 1 0 0 GO:
0004935 Adrenergic_receptor_activity
_01 14 0 1 1 0 0 GO:
0004950 Chemokine_receptor_activity
_01 5 0 1 1 0 0 GO:
0004957 Prostaglandin_E_receptor_activity
_01 6 0 1 1 0 0 GO:
0004969 Histamine_receptor_activity
_01 5 0 1 1 0 0 GO:
0004977 Melanocortin_receptor_activity
_01 4 0 1 1 0 0 GO:
0004982 N-formyl_peptide_receptor_activity
_01 6 0 1 1 0 0 GO:
0004983 Neuropeptide_Y_receptor_activity
_01 284 0 1 1 0 0 GO:
0004984 Olfactory_receptor_activity
_01 7 0 1 1 0 0 GO:
0004993 Serotonin_receptor_activity
_01 5 0 1 1 0 0 GO:
0004994 Somatostatin_receptor_activity
_01 13 0 1 1 0 0 GO:
0005001 Transmembrane_receptor_protein_tyrosine_phosphatase_activity
_01 7 0 1 1 0 0 GO:
0005003 Ephrin_receptor_activity

_01 5 0 1 1 0 0 GO:
0005005 Transmembrane-ephrin_receptor_activity
_01 7 0 1 1 0 0 GO:
0005021 Vascular_endothelial_growth_factor_receptor_activity
_01 8 0 1 1 0 0 GO:
0005024 Transforming_growth_factor_beta_receptor_activity
_01 8 0 1 1 0 0 GO:
0005031 Tumor_necrosis_factor_receptor_activity
_01 8 0 1 1 0 0 GO:
0005041 Low-density_lipoprotein_receptor_activity
_01 30 0 1 1 0 0 GO:
0005044 Scavenger_receptor_activity
_01 26 0 1 1 0 0 GO:
0005057 Receptor_signaling_protein_activity
_01 6 0 1 1 0 0 GO:
0005068
Transmembrane_receptor_protein_tyrosine_kinase_adaptor_activity
_01 32 1 1 1 0 4.9e-05 GO:
0005070 SH3/SH2_adaptor_activity
_01 19 0 1 1 0 0 GO:
0005080 Protein_kinase_C_binding
_01 22 0 1 1 0 0 GO:
0005083 Small_GTPase_regulator_activity
_01 84 0 1 1 0 0 GO:
0005085 Guanyl-nucleotide_exchange_factor_activity
_01 11 0 1 1 0 0 GO:
0005086 ARF_guanyl-nucleotide_exchange_factor_activity
_01 5 0 1 1 0 0 GO:
0005088 Ras_guanyl-nucleotide_exchange_factor_activity
_01 45 0 1 1 0 0 GO:
0005089 Rho_guanyl-nucleotide_exchange_factor_activity
_01 10 0 1 1 0 0 GO:
0005095 GTPase_inhibitor_activity
_01 113 1 1 1 0 4.9e-05 GO:
0005096 GTPase_activator_activity
_01 30 0 1 1 0 0 GO:
0005097 Rab_GTPase_activator_activity
_01 12 0 1 1 0 0 GO:
0005099 Ras_GTPase_activator_activity
_01 19 0 1 1 0 0 GO:
0005100 Rho_GTPase_activator_activity
_01 143 0 1 1 0 0 GO:
0005102 Receptor_binding
_01 11 0 1 1 0 0 GO:
0005104 Fibroblast_growth_factor_receptor_binding
_01 9 0 1 1 0 0 GO:
0005109 Frizzled_binding
_01 8 0 1 1 0 0 GO:
0005112 Notch_binding
_01 5 0 1 1 0 0 GO:
0005114 Type_II_transforming_growth_factor_beta_receptor_binding
_01 6 0 1 1 0 0 GO:
0005123 Death_receptor_binding
_01 119 1 1 1 0 0.000423 GO:

```
0005125 Cytokine_activity
_01     9       0       1       1       0       0       GO:
0005126 Cytokine_receptor_binding
_01     8       0       1       1       0       0       GO:
0005132 Interferon-alpha/beta_receptor_binding
_01     5       0       1       1       0       0       GO:
0005138 Interleukin-6_receptor_binding
_01     5       0       1       1       0       0       GO:
0005149 Interleukin-1_receptor_binding
_01     5       0       1       1       0       0       GO:
0005152 Interleukin-1_receptor_antagonist_activity
_01     11      0       1       1       0       0       GO:
0005154 Epidermal_growth_factor_receptor_binding
_01     23      0       1       1       0       0       GO:
0005158 Insulin_receptor_binding
_01     12      0       1       1       0       0       GO:
0005159 Insulin-like_growth_factor_receptor_binding
_01     8       0       1       1       0       0       GO:
0005160 Transforming_growth_factor_beta_receptor_binding
_01     10      0       1       1       0       0       GO:
0005161 Platelet-derived_growth_factor_receptor_binding
_01     13      0       1       1       0       0       GO:
0005164 Tumor_necrosis_factor_receptor_binding
_01     44      0       1       1       0       0       GO:
0005178 Integrin_binding
_01     52      0       1       1       0       0       GO:
0005179 Hormone_activity
_01     17      0       1       1       0       0       GO:
0005184 Neuropeptide_hormone_activity
_01     128     1       1       1       0       6.9e-05 GO:
0005198 Structural_molecule_activity
_01     45      0       1       1       0       0       GO:
0005200 Structural_constituent_of_cytoskeleton
_01     41      0       1       1       0       0       GO:
0005201 Extracellular_matrix_structural_constituent
_01     10      0       1       1       0       0       GO:
0005212 Structural_constituent_of_eye_lens
_01     173     1       1       1       0       5e-05   GO:
0005215 Transporter_activity
_01     99      1       1       1       0       0.000423GO:
0005216 Ion_channel_activity
_01     30      0       1       1       0       0       GO:
0005230 Extracellular_ligand-gated_ion_channel_activity
_01     14      0       1       1       0       0       GO:
0005234 Extracellular-glutamate-gated_ion_channel_activity
_01     13      0       1       1       0       0       GO:
0005242 Inward_rectifier_potassium_channel_activity
_01     8       0       1       1       0       0       GO:
0005243 Gap_junction_channel_activity
_01     105     0       1       1       0       0       GO:
0005244 Voltage-gated_ion_channel_activity
_01     20      0       1       1       0       0       GO:
0005245 Voltage-gated_calcium_channel_activity
_01     11      0       1       1       0       0       GO:
```

0005246 Calcium_channel_regulator_activity
_01 7 0 1 1 0 0 GO:
0005247 Voltage-gated_chloride_channel_activity
_01 8 0 1 1 0 0 GO:
0005248 Voltage-gated_sodium_channel_activity
_01 51 0 1 1 0 0 GO:
0005249 Voltage-gated_potassium_channel_activity
_01 7 0 1 1 0 0 GO:
0005251 Delayed_rectifier_potassium_channel_activity
_01 40 0 1 1 0 0 GO:
0005254 Chloride_channel_activity
_01 9 0 1 1 0 0 GO:
0005261 Cation_channel_activity
_01 35 0 1 1 0 0 GO:
0005262 Calcium_channel_activity
_01 24 0 1 1 0 0 GO:
0005267 Potassium_channel_activity
_01 7 0 1 1 0 0 GO:
0005272 Sodium_channel_activity
_01 8 0 1 1 0 0 GO:
0005313 L-glutamate_transmembrane_transporter_activity
_01 10 0 1 1 0 0 GO:
0005319 Lipid_transporter_activity
_01 9 0 1 1 0 0 GO:
0005328 Neurotransmitter:sodium_symporter_activity
_01 5 0 1 1 0 0 GO:
0005337 Nucleoside_transmembrane_transporter_activity
_01 10 0 1 1 0 0 GO:
0005344 Oxygen_transporter_activity
_01 5 0 1 1 0 0 GO:
0005351 Sugar:hydrogen_symporter_activity
_01 7 0 1 1 0 0 GO:
0005355 Glucose_transmembrane_transporter_activity
_01 9 0 1 1 0 0 GO:
0005385 Zinc_ion_transmembrane_transporter_activity
_01 6 0 1 1 0 0 GO:
0005388 Calcium-transporting_ATPase_activity
_01 7 0 1 1 0 0 GO:
0005391 Sodium:potassium-exchanging_ATPase_activity
_01 13 0 1 1 0 0 GO:
0005452 Inorganic_anion_exchanger_activity
_01 12 0 1 1 0 0 GO:
0005484 SNAP_receptor_activity
_01 5 0 1 1 0 0 GO:
0005487 Nucleocytoplasmic_transporter_activity
_01 301 2 0.023215 0.609116 0 0.0002365
GO:0005488 Binding
_01 15 1 1 1 0 0.000339 GO:
0005496 Steroid_binding
_01 6 0 1 1 0 0 GO:
0005501 Retinoid_binding
_01 10 0 1 1 0 0 GO:
0005504 Fatty_acid_binding
_01 45 0 1 1 0 0 GO:

0005506 Iron_ion_binding
_01 39 0 1 1 0 0 GO:
0005507 Copper_ion_binding
_01 411 1 1 1 0 0.000423 GO:
0005509 Calcium_ion_binding
_01 5 0 1 1 0 0 GO:
0005513 Detection_of_calcium_ion
_01 98 1 1 1 0 6.9e-05 GO:
0005516 Calmodulin_binding
_01 24 0 1 1 0 0 GO:
0005518 Collagen_binding
_01 17 0 1 1 0 0 GO:
0005520 Insulin-like_growth_factor_binding
_01 5 0 1 1 0 0 GO:
0005521 Lamin_binding
_01 5 0 1 1 0 0 GO:
0005522 Profilin_binding
_01 11 0 1 1 0 0 GO:
0005523 Tropomyosin_binding
_01 909 3 0.047166 0.706319 0 5.36667e-05
GO:0005524 ATP_binding
_01 239 1 1 1 0 0.000423 GO:
0005525 GTP_binding
_01 6 0 1 1 0 0 GO:
0005528 FK506_binding
_01 90 0 1 1 0 0 GO:
0005529 Sugar_binding
_01 8 0 1 1 0 0 GO:
0005537 Mannose_binding
_01 13 1 1 1 0 0.000339 GO:
0005539 Glycosaminoglycan_binding
_01 16 0 1 1 0 0 GO:
0005540 Hyaluronic_acid_binding
_01 10 0 1 1 0 0 GO:
0005542 Folic_acid_binding
_01 34 0 1 1 0 0 GO:
0005543 Phospholipid_binding
_01 19 1 1 1 0 5e-05 GO:
0005544 Calcium-dependent_phospholipid_binding
_01 11 0 1 1 0 0 GO:
0005545 Phosphatidylinositol_binding
_01 13 0 1 1 0 0 GO:
0005546 Phosphatidylinositol-4,5-bisphosphate_binding
_01 12 0 1 1 0 0 GO:
0005547 Phosphatidylinositol-3,4,5-trisphosphate_binding
_01 345 2 0.030622 0.642941 0 0.0002435
GO:0005575 Cellular_component
_01 5 0 1 1 0 0 GO:
0005577 Fibrinogen_complex
_01 166 2 0.011648 0.508221 0 0.000211 GO:
0005578 Proteinaceous_extracellular_matrix
_01 7 0 1 1 0 0 GO:
0005579 Membrane_attack_complex
_01 15 0 1 1 0 0 GO:

0005581 Collagen
_01 45 0 1 1 0 0 GO:
0005604 Basement_membrane
_01 10 0 1 1 0 0 GO:
0005605 Basal_lamina
_01 6 0 1 1 0 0 GO:
0005606 Laminin-1_complex
_01 499 3 0.003443 0.308913 0 0.000281667
GO:0005615 Extracellular_space
_01 323 0 1 1 0 0 GO:
0005624 Membrane_fraction
_01 197 0 1 1 0 0 GO:
0005625 Soluble_fraction
_01 22 0 1 1 0 0 GO:
0005626 Insoluble_fraction
_01 64 0 1 1 0 0 GO:
0005635 Nuclear_envelope
_01 22 0 1 1 0 0 GO:
0005637 Nuclear_inner_membrane
_01 15 0 1 1 0 0 GO:
0005640 Nuclear_outer_membrane
_01 38 0 1 1 0 0 GO:
0005643 Nuclear_pore
_01 498 3 0.002715 0.281437 0 0.000183 GO:
0005654 Nucleoplasm
_01 5 0 1 1 0 0 GO:
0005655 Nucleolar_ribonuclease_P_complex
_01 6 0 1 1 0 0 GO:
0005657 Replication_fork
_01 5 0 1 1 0 0 GO:
0005658 Alpha_DNA_polymerase:primase_complex
_01 7 0 1 1 0 0 GO:
0005664 Nuclear_origin_of_replication_recognition_complex
_01 9 0 1 1 0 0 GO:
0005665 DNA-directed_RNA_polymerase_II,_core_complex
_01 5 0 1 1 0 0 GO:
0005666 DNA-directed_RNA_polymerase_III_complex
_01 124 3 0.000119 0.045301 0 0.000157 GO:
0005667 Transcription_factor_complex
_01 10 0 1 1 0 0 GO:
0005669 Transcription_factor_TFIID_complex
_01 11 0 1 1 0 0 GO:
0005671 Ada2/Gcn5/Ada3_transcription_activator_complex
_01 5 0 1 1 0 0 GO:
0005675 Holo_TFIIH_complex
_01 20 1 1 1 0 0.000423 GO:
0005680 Anaphase-promoting_complex
_01 87 0 1 1 0 0 GO:
0005681 Spliceosomal_complex
_01 14 0 1 1 0 0 GO:
0005689 U12-type_spliceosomal_complex
_01 70 1 1 1 0 0.000423 GO:
0005694 Chromosome
_01 9 1 1 1 0 7.4e-05 GO:

0005720 Nuclear_heterochromatin
_01 697 4 0.001055 0.172897 0 0.00021725
GO:0005730 Nucleolus
_01 6 0 1 1 0 0 GO:
0005732 Small_nucleolar_ribonucleoprotein_complex
_01 790 1 1 1 0 0.000423 GO:
0005739 Mitochondrion
_01 11 0 1 1 0 0 GO:
0005740 Mitochondrial_envelope
_01 58 0 1 1 0 0 GO:
0005741 Mitochondrial_outer_membrane
_01 5 0 1 1 0 0 GO:
0005742 Mitochondrial_outer_membrane_translocase_complex
_01 160 0 1 1 0 0 GO:
0005743 Mitochondrial_inner_membrane
_01 5 0 1 1 0 0 GO:
0005744 Mitochondrial_inner_membrane_presequence_translocase_complex
_01 8 0 1 1 0 0 GO:
0005746 Mitochondrial_respiratory_chain
_01 19 0 1 1 0 0 GO:
0005747 Mitochondrial_respiratory_chain_complex_I
_01 10 0 1 1 0 0 GO:
0005753 Mitochondrial_proton-transporting_ATP_synthase_complex
_01 19 1 1 1 0 0.000423 GO:
0005758 Mitochondrial_intermembrane_space
_01 111 0 1 1 0 0 GO:
0005759 Mitochondrial_matrix
_01 13 0 1 1 0 0 GO:
0005761 Mitochondrial_ribosome
_01 13 0 1 1 0 0 GO:
0005762 Mitochondrial_large_ribosomal_subunit
_01 10 0 1 1 0 0 GO:
0005763 Mitochondrial_small_ribosomal_subunit
_01 104 1 1 1 0 5e-05 GO:
0005764 Lysosome
_01 62 0 1 1 0 0 GO:
0005765 Lysosomal_membrane
_01 142 0 1 1 0 0 GO:
0005768 Endosome
_01 54 0 1 1 0 0 GO:
0005769 Early_endosome
_01 26 0 1 1 0 0 GO:
0005770 Late_endosome
_01 8 0 1 1 0 0 GO:
0005771 Multivesicular_body
_01 6 0 1 1 0 0 GO:
0005773 Vacuole
_01 6 0 1 1 0 0 GO:
0005774 Vacuolar_membrane
_01 8 0 1 1 0 0 GO:
0005776 Autophagic_vacuole
_01 57 0 1 1 0 0 GO:
0005777 Peroxisome
_01 20 0 1 1 0 0 GO:

0005778 Peroxisomal_membrane
_01	8	0	1	1	0	0	GO:
0005779 Integral_to_peroxisomal_membrane
_01	18	0	1	1	0	0	GO:
0005782 Peroxisomal_matrix
_01	575	2	0.0593539	0.722286	0	4.45e-05
GO:0005783 Endoplasmic_reticulum
_01	5	0	1	1	0	0	GO:
0005786 Signal_recognition_particle,_endoplasmic_reticulum_targeting
_01	59	1	1	1	0	3.9e-05	GO:
0005788 Endoplasmic_reticulum_lumen
_01	355	1	1	1	0	3.9e-05	GO:
0005789 Endoplasmic_reticulum_membrane
_01	6	0	1	1	0	0	GO:
0005790 Smooth_endoplasmic_reticulum
_01	14	0	1	1	0	0	GO:
0005791 Rough_endoplasmic_reticulum
_01	126	1	1	1	0	3.9e-05	GO:
0005792 Microsome
_01	28	0	1	1	0	0	GO:
0005793 ER-Golgi_intermediate_compartment
_01	574	1	1	1	0	0.000423	GO:
0005794 Golgi_apparatus
_01	17	0	1	1	0	0	GO:
0005795 Golgi_stack
_01	11	0	1	1	0	0	GO:
0005796 Golgi_lumen
_01	8	0	1	1	0	0	GO:
0005798 Golgi-associated_vesicle
_01	13	0	1	1	0	0	GO:
0005801 Cis-Golgi_network
_01	40	0	1	1	0	0	GO:
0005802 Trans-Golgi_network
_01	15	0	1	1	0	0	GO:
0005811 Lipid_particle
_01	113	2	0.00338	0.307749	0	5.1e-05	GO:
0005813 Centrosome
_01	26	0	1	1	0	0	GO:
0005814 Centriole
_01	64	0	1	1	0	0	GO:
0005815 Microtubule_organizing_center
_01	65	0	1	1	0	0	GO:
0005819 Spindle
_01	5	0	1	1	0	0	GO:
0005832 Chaperonin-containing_T-complex
_01	9	0	1	1	0	0	GO:
0005833 Hemoglobin_complex
_01	20	0	1	1	0	0	GO:
0005834 Heterotrimeric_G-protein_complex
_01	5	0	1	1	0	0	GO:
0005838 Proteasome_regulatory_particle
_01	9	0	1	1	0	0	GO:
0005839 Proteasome_core_complex
_01	107	0	1	1	0	0	GO:

0005840 Ribosome
_01	8	0	1	1	0	0	GO:
0005844 Polysome
_01	6	0	1	1	0	0	GO:
0005845 MRNA_cap_binding_complex
_01	7	0	1	1	0	0	GO:
0005852 Eukaryotic_translation_initiation_factor_3_complex
_01	439	3	0.00865799	0.456225	0
0.000180667	GO:0005856 Cytoskeleton
_01	7	0	1	1	0	0	GO:
0005858 Axonemal_dynein_complex
_01	14	0	1	1	0	0	GO:
0005859 Muscle_myosin_complex
_01	7	0	1	1	0	0	GO:
0005861 Troponin_complex
_01	8	0	1	1	0	0	GO:
0005868 Cytoplasmic_dynein_complex
_01	9	0	1	1	0	0	GO:
0005871 Kinesin_complex
_01	145	0	1	1	0	0	GO:
0005874 Microtubule
_01	11	0	1	1	0	0	GO:
0005875 Microtubule_associated_complex
_01	17	0	1	1	0	0	GO:
0005876 Spindle_microtubule
_01	15	0	1	1	0	0	GO:
0005881 Cytoplasmic_microtubule
_01	43	0	1	1	0	0	GO:
0005882 Intermediate_filament
_01	5	0	1	1	0	0	GO:
0005883 Neurofilament
_01	21	0	1	1	0	0	GO:
0005884 Actin_filament
_01	646	2	0.111196	0.827606	0	0.0002375
GO:0005887 Integral_to_plasma_membrane
_01	6	0	1	1	0	0	GO:
0005890 Sodium:potassium-exchanging_ATPase_complex
_01	17	0	1	1	0	0	GO:
0005891 Voltage-gated_calcium_channel_complex
_01	12	0	1	1	0	0	GO:
0005892 Nicotinic_acetylcholine-gated_receptor-channel_complex
_01	35	1	1	1	0	3.9e-05	GO:
0005901 Caveola
_01	17	0	1	1	0	0	GO:
0005902 Microvillus
_01	12	0	1	1	0	0	GO:
0005903 Brush_border
_01	32	0	1	1	0	0	GO:
0005905 Coated_pit
_01	45	0	1	1	0	0	GO:
0005911 Cell-cell_junction
_01	22	0	1	1	0	0	GO:
0005912 Adherens_junction
_01	20	0	1	1	0	0	GO:

0005913 Cell-cell_adherens_junction
_01 5 0 1 1 0 0 GO:
0005915 Zonula_adherens
_01 7 0 1 1 0 0 GO:
0005916 Fascia_adherens
_01 12 0 1 1 0 0 GO:
0005921 Gap_junction
_01 14 0 1 1 0 0 GO:
0005922 Connexon_complex
_01 53 0 1 1 0 0 GO:
0005923 Tight_junction
_01 71 1 1 1 0 0.000344 GO:
0005925 Focal_adhesion
_01 63 0 1 1 0 0 GO:
0005929 Cilium
_01 28 0 1 1 0 0 GO:
0005932 Microtubule_basal_body
_01 56 0 1 1 0 0 GO:
0005938 Cell_cortex
_01 7 0 1 1 0 0 GO:
0005942 Phosphoinositide_3-kinase_complex
_01 5 0 1 1 0 0 GO:
0005952 CAMP-dependent_protein_kinase_complex
_01 177 2 0.004555 0.35498 0 5.1e-05 GO:
0005975 Carbohydrate_metabolic_process
_01 17 0 1 1 0 0 GO:
0005977 Glycogen_metabolic_process
_01 8 0 1 1 0 0 GO:
0005978 Glycogen_biosynthetic_process
_01 13 0 1 1 0 0 GO:
0005980 Glycogen_catabolic_process
_01 5 1 1 1 0 5e-05 GO:
0006000 Fructose_metabolic_process
_01 60 2 0.000428 0.104867 0 0.0001945
GO:0006006 Glucose_metabolic_process
_01 5 0 1 1 0 0 GO:
0006013 Mannose_metabolic_process
_01 9 0 1 1 0 0 GO:
0006024 Glycosaminoglycan_biosynthetic_process
_01 9 0 1 1 0 0 GO:
0006044 N-acetylglucosamine_metabolic_process
_01 9 0 1 1 0 0 GO:
0006071 Glycerol_metabolic_process
_01 7 0 1 1 0 0 GO:
0006081 Cellular_aldehyde_metabolic_process
_01 13 0 1 1 0 0 GO:
0006090 Pyruvate_metabolic_process
_01 43 0 1 1 0 0 GO:
0006091 Generation_of_precursor_metabolites_and_energy
_01 25 1 1 1 0 5e-05 GO:
0006094 Gluconeogenesis
_01 23 1 1 1 0 5e-05 GO:
0006096 Glycolysis
_01 5 0 1 1 0 0 GO:

0006098 Pentose-phosphate_shunt
_01 16 0 1 1 0 0 GO:
0006099 Tricarboxylic_acid_cycle
_01 7 0 1 1 0 0 GO:
0006101 Citrate_metabolic_process
_01 11 0 1 1 0 0 GO:
0006103 2-oxoglutarate_metabolic_process
_01 9 0 1 1 0 0 GO:
0006107 Oxaloacetate_metabolic_process
_01 7 0 1 1 0 0 GO:
0006108 Malate_metabolic_process
_01 71 0 1 1 0 0 GO:
0006112 Energy_reserve_metabolic_process
_01 19 0 1 1 0 0 GO:
0006120 Mitochondrial_electron_transport,_NADH_to_ubiquinone
_01 34 0 1 1 0 0 GO:
0006139
Nucleobase,_nucleoside,_nucleotide_and_nucleic_acid_metabolic_proces
s
_01 27 0 1 1 0 0 GO:
0006144 Purine_base_metabolic_process
_01 12 0 1 1 0 0 GO:
0006164 Purine_nucleotide_biosynthetic_process
_01 8 0 1 1 0 0 GO:
0006182 CGMP_biosynthetic_process
_01 6 0 1 1 0 0 GO:
0006183 GTP_biosynthetic_process
_01 10 0 1 1 0 0 GO:
0006195 Purine_nucleotide_catabolic_process
_01 5 0 1 1 0 0 GO:
0006200 ATP_catabolic_process
_01 15 0 1 1 0 0 GO:
0006206 Pyrimidine_base_metabolic_process
_01 6 0 1 1 0 0 GO:
0006228 UTP_biosynthetic_process
_01 7 0 1 1 0 0 GO:
0006241 CTP_biosynthetic_process
_01 14 0 1 1 0 0 GO:
0006259 DNA_metabolic_process
_01 93 0 1 1 0 0 GO:
0006260 DNA_replication
_01 8 0 1 1 0 0 GO:
0006261 DNA-dependent_DNA_replication
_01 7 0 1 1 0 0 GO:
0006265 DNA_topological_change
_01 7 0 1 1 0 0 GO:
0006268 DNA_unwinding_involved_in_replication
_01 15 0 1 1 0 0 GO:
0006270 DNA-dependent_DNA_replication_initiation
_01 21 0 1 1 0 0 GO:
0006271 DNA_strand_elongation_involved_in_DNA_replication
_01 8 0 1 1 0 0 GO:
0006275 Regulation_of_DNA_replication
_01 159 0 1 1 0 0 GO:

0006281 DNA_repair
_01	23	0	1	1	0	0	GO:
0006283 Transcription-coupled_nucleotide-excision_repair
_01	21	0	1	1	0	0	GO:
0006284 Base-excision_repair
_01	6	0	1	1	0	0	GO:
0006285 Base-excision_repair,_AP_site_formation
_01	37	0	1	1	0	0	GO:
0006289 Nucleotide-excision_repair
_01	10	0	1	1	0	0	GO:
0006297 Nucleotide-excision_repair,_DNA_gap_filling
_01	12	0	1	1	0	0	GO:
0006298 Mismatch_repair
_01	22	0	1	1	0	0	GO:
0006302 Double-strand_break_repair
_01	5	0	1	1	0	0	GO:
0006303 Double-strand_break_repair_via_nonhomologous_end_joining
_01	10	0	1	1	0	0	GO:
0006306 DNA_methylation
_01	17	0	1	1	0	0	GO:
0006309 DNA_fragmentation_involved_in_apoptotic_nuclear_change
_01	39	0	1	1	0	0	GO:
0006310 DNA_recombination
_01	15	1	1	1	0	7.4e-05	GO:
0006325 Chromatin_organization
_01	25	1	1	1	0	5e-05	GO:
0006333 Chromatin_assembly_or_disassembly
_01	36	0	1	1	0	0	GO:
0006334 Nucleosome_assembly
_01	32	1	1	1	0	6.3e-05	GO:
0006338 Chromatin_remodeling
_01	5	0	1	1	0	0	GO:
0006342 Chromatin_silencing
_01	6	0	1	1	0	0	GO:
0006349 Regulation_of_gene_expression_by_genetic_imprinting
_01	122	0	1	1	0	0	GO:
0006350 Transcription
_01	13	0	1	1	0	0	GO:
0006351 Transcription,_DNA-dependent
_01	8	0	1	1	0	0	GO:
0006352 Transcription_initiation,_DNA-dependent
_01	6	0	1	1	0	0	GO:
0006354 Transcription_elongation,_DNA-dependent
_01	557	5	1.6e-05	0.00879199	0	0.000246
GO:0006355 Regulation_of_transcription,_DNA-dependent
_01	152	0	1	1	0	0	GO:
0006357 Regulation_of_transcription_from_RNA_polymerase_II_promoter
_01	16	0	1	1	0	0	GO:
0006360 Transcription_from_RNA_polymerase_I_promoter
_01	16	0	1	1	0	0	GO:
0006361 Transcription_initiation_from_RNA_polymerase_I_promoter
_01	11	0	1	1	0	0	GO:
0006362 Transcription_elongation_from_RNA_polymerase_I_promoter
_01	13	0	1	1	0	0	GO:

0006363 Termination_of_RNA_polymerase_I_transcription
_01 58 0 1 1 0 0 GO:
0006364 RRNA_processing
_01 161 0 1 1 0 0 GO:
0006366 Transcription_from_RNA_polymerase_II_promoter
_01 38 0 1 1 0 0 GO:
0006367 Transcription_initiation_from_RNA_polymerase_II_promoter
_01 31 0 1 1 0 0 GO:
0006368 Transcription_elongation_from_RNA_polymerase_II_promoter
_01 35 0 1 1 0 0 GO:
0006369 Termination_of_RNA_polymerase_II_transcription
_01 17 0 1 1 0 0 GO:
0006370 MRNA_capping
_01 7 0 1 1 0 0 GO:
0006376 MRNA_splice_site_selection
_01 7 0 1 1 0 0 GO:
0006378 MRNA_polyadenylation
_01 8 0 1 1 0 0 GO:
0006379 MRNA_cleavage
_01 19 0 1 1 0 0 GO:
0006383 Transcription_from_RNA_polymerase_III_promoter
_01 9 0 1 1 0 0 GO:
0006385 Transcription_elongation_from_RNA_polymerase_III_promoter
_01 9 0 1 1 0 0 GO:
0006386 Termination_of_RNA_polymerase_III_transcription
_01 57 0 1 1 0 0 GO:
0006396 RNA_processing
_01 166 0 1 1 0 0 GO:
0006397 MRNA_processing
_01 5 0 1 1 0 0 GO:
0006398 Histone_mRNA_3'-end_processing
_01 9 0 1 1 0 0 GO:
0006401 RNA_catabolic_process
_01 7 0 1 1 0 0 GO:
0006402 MRNA_catabolic_process
_01 40 0 1 1 0 0 GO:
0006406 MRNA_export_from_nucleus
_01 171 0 1 1 0 0 GO:
0006412 Translation
_01 15 0 1 1 0 0 GO:
0006413 Translational_initiation
_01 66 0 1 1 0 0 GO:
0006414 Translational_elongation
_01 61 0 1 1 0 0 GO:
0006415 Translational_termination
_01 43 0 1 1 0 0 GO:
0006417 Regulation_of_translation
_01 21 0 1 1 0 0 GO:
0006418 TRNA_aminoacylation_for_protein_translation
_01 16 0 1 1 0 0 GO:
0006446 Regulation_of_translational_initiation
_01 110 0 1 1 0 0 GO:
0006457 Protein_folding
_01 68 0 1 1 0 0 GO:

0006461 Protein_complex_assembly
_01 94 0 1 1 0 0 GO:
0006464 Protein_modification_process
_01 7 0 1 1 0 0 GO:
0006465 Signal_peptide_processing
_01 5 0 1 1 0 0 GO:
0006467 Protein_thiol-disulfide_exchange
_01 300 0 1 1 0 0 GO:
0006468 Protein_phosphorylation
_01 36 0 1 1 0 0 GO:
0006469 Negative_regulation_of_protein_kinase_activity
_01 75 0 1 1 0 0 GO:
0006470 Protein_dephosphorylation
_01 12 0 1 1 0 0 GO:
0006471 Protein_ADP-ribosylation
_01 5 0 1 1 0 0 GO:
0006476 Protein_deacetylation
_01 6 0 1 1 0 0 GO:
0006479 Protein_methylation
_01 41 1 1 1 0 0.000423 GO:
0006486 Protein_glycosylation
_01 12 0 1 1 0 0 GO:
0006487 Protein_N-linked_glycosylation
_01 21 0 1 1 0 0 GO:
0006488 Dolichol-linked_oligosaccharide_biosynthetic_process
_01 8 0 1 1 0 0 GO:
0006491 N-glycan_processing
_01 12 0 1 1 0 0 GO:
0006493 Protein_O-linked_glycosylation
_01 16 0 1 1 0 0 GO:
0006501 C-terminal_protein_lipidation
_01 14 0 1 1 0 0 GO:
0006506 GPI_anchor_biosynthetic_process
_01 268 2 0.016238 0.541168 0 7.35e-05 GO:
0006508 Proteolysis
_01 9 0 1 1 0 0 GO:
0006509 Membrane_protein_ectodomain_proteolysis
_01 79 1 1 1 0 0.000423 GO:
0006511 Ubiquitin-dependent_protein_catabolic_process
_01 30 0 1 1 0 0 GO:
0006520 Cellular_amino_acid_metabolic_process
_01 33 0 1 1 0 0 GO:
0006521 Regulation_of_cellular_amino_acid_metabolic_process
_01 7 0 1 1 0 0 GO:
0006527 Arginine_catabolic_process
_01 5 0 1 1 0 0 GO:
0006536 Glutamate_metabolic_process
_01 10 0 1 1 0 0 GO:
0006541 Glutamine_metabolic_process
_01 5 0 1 1 0 0 GO:
0006546 Glycine_catabolic_process
_01 8 0 1 1 0 0 GO:
0006548 Histidine_catabolic_process
_01 7 0 1 1 0 0 GO:

0006554 Lysine_catabolic_process
_01	6	0	1	1	0	0	GO:
0006559 L-phenylalanine_catabolic_process
_01	5	0	1	1	0	0	GO:
0006569 Tryptophan_catabolic_process
_01	5	0	1	1	0	0	GO:
0006573 Valine_metabolic_process
_01	5	0	1	1	0	0	GO:
0006584 Catecholamine_metabolic_process
_01	6	0	1	1	0	0	GO:
0006590 Thyroid_hormone_generation
_01	5	0	1	1	0	0	GO:
0006595 Polyamine_metabolic_process
_01	5	0	1	1	0	0	GO:
0006600 Creatine_metabolic_process
_01	20	0	1	1	0	0	GO:
0006605 Protein_targeting
_01	16	0	1	1	0	0	GO:
0006606 Protein_import_into_nucleus
_01	8	0	1	1	0	0	GO:
0006607 NLS-bearing_substrate_import_into_nucleus
_01	10	0	1	1	0	0	GO:
0006611 Protein_export_from_nucleus
_01	7	1	1	1	0	5e-05	GO:
0006612 Protein_targeting_to_membrane
_01	5	0	1	1	0	0	GO:
0006613 Cotranslational_protein_targeting_to_membrane
_01	5	0	1	1	0	0	GO:
0006614 SRP-dependent_cotranslational_protein_targeting_to_membrane
_01	6	0	1	1	0	0	GO:
0006621 Protein_retention_in_ER_lumen
_01	5	0	1	1	0	0	GO:
0006622 Protein_targeting_to_lysosome
_01	6	0	1	1	0	0	GO:
0006625 Protein_targeting_to_peroxisome
_01	17	0	1	1	0	0	GO:
0006626 Protein_targeting_to_mitochondrion
_01	133	0	1	1	0	0	GO:
0006629 Lipid_metabolic_process
_01	43	0	1	1	0	0	GO:
0006631 Fatty_acid_metabolic_process
_01	35	1	1	1	0	3.9e-05	GO:
0006633 Fatty_acid_biosynthetic_process
_01	11	0	1	1	0	0	GO:
0006635 Fatty_acid_beta-oxidation
_01	9	0	1	1	0	0	GO:
0006637 Acyl-CoA_metabolic_process
_01	15	0	1	1	0	0	GO:
0006641 Triglyceride_metabolic_process
_01	5	0	1	1	0	0	GO:
0006642 Triglyceride_mobilization
_01	19	0	1	1	0	0	GO:
0006644 Phospholipid_metabolic_process
_01	8	0	1	1	0	0	GO:

0006656 Phosphatidylcholine_biosynthetic_process
_01 25 0 1 1 0 0 GO:
0006665 Sphingolipid_metabolic_process
_01 9 0 1 1 0 0 GO:
0006672 Ceramide_metabolic_process
_01 5 0 1 1 0 0 GO:
0006685 Sphingomyelin_catabolic_process
_01 6 0 1 1 0 0 GO:
0006688 Glycosphingolipid_biosynthetic_process
_01 8 1 1 1 0 3.9e-05 GO:
0006692 Prostanoid_metabolic_process
_01 6 1 1 1 0 3.9e-05 GO:
0006693 Prostaglandin_metabolic_process
_01 15 0 1 1 0 0 GO:
0006694 Steroid_biosynthetic_process
_01 21 0 1 1 0 0 GO:
0006695 Cholesterol_biosynthetic_process
_01 11 0 1 1 0 0 GO:
0006699 Bile_acid_biosynthetic_process
_01 4 0 1 1 0 0 GO:
0006700 C21-steroid_hormone_biosynthetic_process
_01 4 0 1 1 0 0 GO:
0006702 Androgen_biosynthetic_process
_01 4 0 1 1 0 0 GO:
0006704 Glucocorticoid_biosynthetic_process
_01 8 0 1 1 0 0 GO:
0006707 Cholesterol_catabolic_process
_01 6 0 1 1 0 0 GO:
0006729 Tetrahydrobiopterin_biosynthetic_process
_01 13 0 1 1 0 0 GO:
0006730 One-carbon_metabolic_process
_01 5 0 1 1 0 0 GO:
0006734 NADH_metabolic_process
_01 6 0 1 1 0 0 GO:
0006744 Ubiquinone_biosynthetic_process
_01 13 0 1 1 0 0 GO:
0006749 Glutathione_metabolic_process
_01 6 0 1 1 0 0 GO:
0006750 Glutathione_biosynthetic_process
_01 34 1 1 1 0 5e-05 GO:
0006754 ATP_biosynthetic_process
_01 32 0 1 1 0 0 GO:
0006766 Vitamin_metabolic_process
_01 26 0 1 1 0 0 GO:
0006767 Water-soluble_vitamin_metabolic_process
_01 5 0 1 1 0 0 GO:
0006776 Vitamin_A_metabolic_process
_01 7 0 1 1 0 0 GO:
0006778 Porphyrin_metabolic_process
_01 10 0 1 1 0 0 GO:
0006783 Heme_biosynthetic_process
_01 10 0 1 1 0 0 GO:
0006790 Sulfur_compound_metabolic_process
_01 10 0 1 1 0 0 GO:

```
0006796 Phosphate_metabolic_process
_01     18      0       1       1       0       0       GO:
0006800 Oxygen_and_reactive_oxygen_species_metabolic_process
_01     9       0       1       1       0       0       GO:
0006801 Superoxide_metabolic_process
_01     62      1       1       1       0       3.9e-05 GO:
0006805 Xenobiotic_metabolic_process
_01     10      0       1       1       0       0       GO:
0006807 Nitrogen_compound_metabolic_process
_01     7       0       1       1       0       0       GO:
0006809 Nitric_oxide_biosynthetic_process
_01     324     1       1       1       0       0.000339 GO:
0006810 Transport
_01     391     2       0.0635899       0.727532 0      0.000246
GO:0006811 Ion_transport
_01     26      0       1       1       0       0       GO:
0006812 Cation_transport
_01     108     0       1       1       0       0       GO:
0006813 Potassium_ion_transport
_01     77      0       1       1       0       0       GO:
0006814 Sodium_ion_transport
_01     84      0       1       1       0       0       GO:
0006816 Calcium_ion_transport
_01     7       0       1       1       0       0       GO:
0006817 Phosphate_transport
_01     9       0       1       1       0       0       GO:
0006820 Anion_transport
_01     12      0       1       1       0       0       GO:
0006821 Chloride_transport
_01     8       0       1       1       0       0       GO:
0006825 Copper_ion_transport
_01     15      0       1       1       0       0       GO:
0006826 Iron_ion_transport
_01     16      0       1       1       0       0       GO:
0006829 Zinc_ion_transport
_01     23      0       1       1       0       0       GO:
0006833 Water_transport
_01     7       0       1       1       0       0       GO:
0006835 Dicarboxylic_acid_transport
_01     22      0       1       1       0       0       GO:
0006836 Neurotransmitter_transport
_01     7       0       1       1       0       0       GO:
0006839 Mitochondrial_transport
_01     6       0       1       1       0       0       GO:
0006853 Carnitine_shuttle
_01     29      0       1       1       0       0       GO:
0006865 Amino_acid_transport
_01     36      0       1       1       0       0       GO:
0006869 Lipid_transport
_01     5       0       1       1       0       0       GO:
0006873 Cellular_ion_homeostasis
_01     47      0       1       1       0       0       GO:
0006874 Cellular_calcium_ion_homeostasis
_01     7       0       1       1       0       0       GO:
```

0006878 Cellular_copper_ion_homeostasis
_01 14 0 1 1 0 0 GO:
0006879 Cellular_iron_ion_homeostasis
_01 5 0 1 1 0 0 GO:
0006882 Cellular_zinc_ion_homeostasis
_01 8 0 1 1 0 0 GO:
0006884 Cell_volume_homeostasis
_01 12 0 1 1 0 0 GO:
0006885 Regulation_of_pH
_01 122 0 1 1 0 0 GO:
0006886 Intracellular_protein_transport
_01 33 0 1 1 0 0 GO:
0006887 Exocytosis
_01 31 0 1 1 0 0 GO:
0006888 ER_to_Golgi_vesicle-mediated_transport
_01 13 0 1 1 0 0 GO:
0006890 Retrograde_vesicle-mediated_transport,_Golgi_to_ER
_01 11 0 1 1 0 0 GO:
0006891 Intra-Golgi_vesicle-mediated_transport
_01 28 0 1 1 0 0 GO:
0006892 Post-Golgi_vesicle-mediated_transport
_01 5 0 1 1 0 0 GO:
0006895 Golgi_to_endosome_transport
_01 68 0 1 1 0 0 GO:
0006897 Endocytosis
_01 32 0 1 1 0 0 GO:
0006898 Receptor-mediated_endocytosis
_01 14 0 1 1 0 0 GO:
0006904 Vesicle_docking_involved_in_exocytosis
_01 5 0 1 1 0 0 GO:
0006906 Vesicle_fusion
_01 21 0 1 1 0 0 GO:
0006909 Phagocytosis
_01 11 0 1 1 0 0 GO:
0006913 Nucleocytoplasmic_transport
_01 25 0 1 1 0 0 GO:
0006914 Autophagy
_01 364 3 0.002442 0.264807 0 0.000182333
GO:0006915 Apoptosis
_01 128 0 1 1 0 0 GO:
0006916 Anti-apoptosis
_01 115 1 1 1 0 0.000423 GO:
0006917 Induction_of_apoptosis
_01 31 1 1 1 0 0.000423 GO:
0006919 Activation_of_caspase_activity
_01 30 1 1 1 0 7.4e-05 GO:
0006921 Cellular_component_disassembly_involved_in_apoptosis
_01 71 1 1 1 0 3.9e-05 GO:
0006928 Cellular_component_movement
_01 80 0 1 1 0 0 GO:
0006935 Chemotaxis
_01 67 0 1 1 0 0 GO:
0006936 Muscle_contraction
_01 10 0 1 1 0 0 GO:

0006937 Regulation_of_muscle_contraction
_01 10 0 1 1 0 0 GO:
0006939 Smooth_muscle_contraction
_01 8 0 1 1 0 0 GO:
0006940 Regulation_of_smooth_muscle_contraction
_01 7 1 1 1 0 5e-05 GO:
0006941 Striated_muscle_contraction
_01 8 0 1 1 0 0 GO:
0006942 Regulation_of_striated_muscle_contraction
_01 15 0 1 1 0 0 GO:
0006944 Cellular_membrane_fusion
_01 78 1 1 1 0 5e-05 GO:
0006950 Response_to_stress
_01 43 0 1 1 0 0 GO:
0006952 Defense_response
_01 21 1 1 1 0 0.000339 GO:
0006953 Acute-phase_response
_01 151 1 1 1 0 0.000339 GO:
0006954 Inflammatory_response
_01 207 0 1 1 0 0 GO:
0006955 Immune_response
_01 13 0 1 1 0 0 GO:
0006956 Complement_activation
_01 7 0 1 1 0 0 GO:
0006957 Complement_activation,_alternative_pathway
_01 20 0 1 1 0 0 GO:
0006958 Complement_activation,_classical_pathway
_01 22 0 1 1 0 0 GO:
0006959 Humoral_immune_response
_01 37 0 1 1 0 0 GO:
0006968 Cellular_defense_response
_01 9 0 1 1 0 0 GO:
0006970 Response_to_osmotic_stress
_01 58 0 1 1 0 0 GO:
0006974 Response_to_DNA_damage_stimulus
_01 39 0 1 1 0 0 GO:
0006977
DNA_damage_response,_signal_transduction_by_p53_class_mediator_resul
ting_in_cell_cycle_arrest
_01 6 0 1 1 0 0 GO:
0006978
DNA_damage_response,_signal_transduction_by_p53_class_mediator_resul
ting_in_transcription_of_p21_class_mediator
_01 68 1 1 1 0 3.9e-05 GO:
0006979 Response_to_oxidative_stress
_01 5 0 1 1 0 0 GO:
0006983 ER_overload_response
_01 36 0 1 1 0 0 GO:
0006986 Response_to_unfolded_protein
_01 7 0 1 1 0 0 GO:
0007004 Telomere_maintenance_via_telomerase
_01 17 0 1 1 0 0 GO:
0007005 Mitochondrion_organization
_01 31 0 1 1 0 0 GO:

0007010 Cytoskeleton_organization
_01 19 2 8.39999e-05 0.034121 0
0.0002365 GO:0007015 Actin_filament_organization
_01 6 0 1 1 0 0 GO:
0007016 Cytoskeletal_anchoring_at_plasma_membrane
_01 10 0 1 1 0 0 GO:
0007017 Microtubule-based_process
_01 53 0 1 1 0 0 GO:
0007018 Microtubule-based_movement
_01 5 0 1 1 0 0 GO:
0007020 Microtubule_nucleation
_01 7 0 1 1 0 0 GO:
0007026 Negative_regulation_of_microtubule_depolymerization
_01 6 0 1 1 0 0 GO:
0007029 Endoplasmic_reticulum_organization
_01 13 0 1 1 0 0 GO:
0007030 Golgi_organization
_01 11 0 1 1 0 0 GO:
0007031 Peroxisome_organization
_01 12 0 1 1 0 0 GO:
0007032 Endosome_organization
_01 17 0 1 1 0 0 GO:
0007040 Lysosome_organization
_01 8 0 1 1 0 0 GO:
0007043 Cell-cell_junction_assembly
_01 230 0 1 1 0 0 GO:
0007049 Cell_cycle
_01 86 0 1 1 0 0 GO:
0007050 Cell_cycle_arrest
_01 10 0 1 1 0 0 GO:
0007051 Spindle_organization
_01 9 0 1 1 0 0 GO:
0007052 Mitotic_spindle_organization
_01 25 0 1 1 0 0 GO:
0007059 Chromosome_segregation
_01 106 0 1 1 0 0 GO:
0007067 Mitosis
_01 6 0 1 1 0 0 GO:
0007076 Mitotic_chromosome_condensation
_01 7 0 1 1 0 0 GO:
0007080 Mitotic_metaphase_plate_congression
_01 10 0 1 1 0 0 GO:
0007088 Regulation_of_mitosis
_01 5 0 1 1 0 0 GO:
0007089 Traversing_start_control_point_of_mitotic_cell_cycle
_01 6 0 1 1 0 0 GO:
0007090 Regulation_of_S_phase_of_mitotic_cell_cycle
_01 6 0 1 1 0 0 GO:
0007091 Mitotic_metaphase/anaphase_transition
_01 20 1 1 1 0 0.000423 GO:
0007094 Mitotic_cell_cycle_spindle_assembly_checkpoint
_01 5 0 1 1 0 0 GO:
0007099 Centriole_replication
_01 29 0 1 1 0 0 GO:

0007126 Meiosis
_01   5   0   1   1   0   0   GO:
0007130 Synaptonemal_complex_assembly
_01   14   0   1   1   0   0   GO:
0007131 Reciprocal_meiotic_recombination
_01   9   0   1   1   0   0   GO:
0007140 Male_meiosis
_01   5   0   1   1   0   0   GO:
0007141 Male_meiosis_I
_01   63   0   1   1   0   0   GO:
0007154 Cell_communication
_01   342   0   1   1   0   0   GO:
0007155 Cell_adhesion
_01   77   0   1   1   0   0   GO:
0007156 Homophilic_cell_adhesion
_01   12   0   1   1   0   0   GO:
0007157 Heterophilic_cell-cell_adhesion
_01   14   0   1   1   0   0   GO:
0007159 Leukocyte_cell-cell_adhesion
_01   54   0   1   1   0   0   GO:
0007160 Cell-matrix_adhesion
_01   17   0   1   1   0   0   GO:
0007162 Negative_regulation_of_cell_adhesion
_01   15   0   1   1   0   0   GO:
0007163 Establishment_or_maintenance_of_cell_polarity
_01   721   1   1   1   0   4.9e-05 GO:
0007165 Signal_transduction
_01   112   0   1   1   0   0   GO:
0007166 Cell_surface_receptor_linked_signaling_pathway
_01   8   0   1   1   0   0   GO:
0007168 Receptor_guanylyl_cyclase_signaling_pathway
_01   55   0   1   1   0   0   GO:
0007169
Transmembrane_receptor_protein_tyrosine_kinase_signaling_pathway
_01   7   0   1   1   0   0   GO:
0007172 Signal_complex_assembly
_01   49   0   1   1   0   0   GO:
0007173 Epidermal_growth_factor_receptor_signaling_pathway
_01   44   0   1   1   0   0   GO:
0007179 Transforming_growth_factor_beta_receptor_signaling_pathway
_01   5   0   1   1   0   0   GO:
0007183 SMAD_protein_complex_assembly
_01   5   0   1   1   0   0   GO:
0007185
Transmembrane_receptor_protein_tyrosine_phosphatase_signaling_pathwa
y
_01   210   0   1   1   0   0   GO:
0007186 G-protein_coupled_receptor_protein_signaling_pathway
_01   31   0   1   1   0   0   GO:
0007187 G-
protein_signaling,_coupled_to_cyclic_nucleotide_second_messenger
_01   18   0   1   1   0   0   GO:
0007188 G-
protein_signaling,_coupled_to_cAMP_nucleotide_second_messenger

_01 27 0 1 1 0 0 GO:
0007189 Activation_of_adenylate_cyclase_activity_by_G-
protein_signaling_pathway
_01 20 0 1 1 0 0 GO:
0007190 Activation_of_adenylate_cyclase_activity
_01 21 0 1 1 0 0 GO:
0007193 Inhibition_of_adenylate_cyclase_activity_by_G-
protein_signaling_pathway
_01 14 0 1 1 0 0 GO:
0007194 Negative_regulation_of_adenylate_cyclase_activity
_01 24 0 1 1 0 0 GO:
0007200 Activation_of_phospholipase_C_activity_by_G-
protein_coupled_receptor_protein_signaling_pathway_coupled_to_IP3_se
cond_messenger
_01 39 0 1 1 0 0 GO:
0007202 Activation_of_phospholipase_C_activity
_01 70 0 1 1 0 0 GO:
0007204 Elevation_of_cytosolic_calcium_ion_concentration
_01 27 0 1 1 0 0 GO:
0007205 Activation_of_protein_kinase_C_activity_by_G-
protein_coupled_receptor_protein_signaling_pathway
_01 6 0 1 1 0 0 GO:
0007212 Dopamine_receptor_signaling_pathway
_01 6 0 1 1 0 0 GO:
0007213 Muscarinic_acetylcholine_receptor_signaling_pathway
_01 14 0 1 1 0 0 GO:
0007214 Gamma-aminobutyric_acid_signaling_pathway
_01 8 0 1 1 0 0 GO:
0007215 Glutamate_signaling_pathway
_01 6 0 1 1 0 0 GO:
0007217 Tachykinin_receptor_signaling_pathway
_01 62 0 1 1 0 0 GO:
0007218 Neuropeptide_signaling_pathway
_01 35 0 1 1 0 0 GO:
0007219 Notch_signaling_pathway
_01 10 0 1 1 0 0 GO:
0007220 Notch_receptor_processing
_01 13 0 1 1 0 0 GO:
0007223 Wnt_receptor_signaling_pathway,_calcium_modulating_pathway
_01 11 0 1 1 0 0 GO:
0007224 Smoothened_signaling_pathway
_01 34 0 1 1 0 0 GO:
0007229 Integrin-mediated_signaling_pathway
_01 69 1 1 1 0 5.2e-05 GO:
0007243 Intracellular_protein_kinase_cascade
_01 10 0 1 1 0 0 GO:
0007249 I-kappaB_kinase/NF-kappaB_cascade
_01 8 0 1 1 0 0 GO:
0007250 Activation_of_NF-kappaB-inducing_kinase_activity
_01 6 0 1 1 0 0 GO:
0007252 I-kappaB_phosphorylation
_01 29 0 1 1 0 0 GO:
0007254 JNK_cascade
_01 18 0 1 1 0 0 GO:

0007257 Activation_of_JUN_kinase_activity
_01 19 0 1 1 0 0 GO:
0007259 JAK-STAT_cascade
_01 9 0 1 1 0 0 GO:
0007263 Nitric_oxide_mediated_signal_transduction
_01 189 2 0.013636 0.521072 0 0.000236 GO:
0007264 Small_GTPase_mediated_signal_transduction
_01 50 0 1 1 0 0 GO:
0007265 Ras_protein_signal_transduction
_01 27 0 1 1 0 0 GO:
0007266 Rho_protein_signal_transduction
_01 172 0 1 1 0 0 GO:
0007267 Cell-cell_signaling
_01 195 1 1 1 0 5e-05 GO:
0007268 Synaptic_transmission
_01 34 0 1 1 0 0 GO:
0007269 Neurotransmitter_secretion
_01 10 0 1 1 0 0 GO:
0007271 Synaptic_transmission,_cholinergic
_01 8 0 1 1 0 0 GO:
0007274 Neuromuscular_synaptic_transmission
_01 613 3 0.012557 0.516034 0 0.000152667
GO:0007275 Multicellular_organismal_development
_01 7 0 1 1 0 0 GO:
0007276 Gamete_generation
_01 15 0 1 1 0 0 GO:
0007281 Germ_cell_development
_01 179 1 1 1 0 0.000339 GO:
0007283 Spermatogenesis
_01 19 0 1 1 0 0 GO:
0007286 Spermatid_development
_01 10 0 1 1 0 0 GO:
0007292 Female_gamete_generation
_01 17 0 1 1 0 0 GO:
0007338 Single_fertilization
_01 9 0 1 1 0 0 GO:
0007339 Binding_of_sperm_to_zona_pellucida
_01 8 0 1 1 0 0 GO:
0007340 Acrosome_reaction
_01 5 1 1 1 0 0.000339 GO:
0007342 Fusion_of_sperm_to_egg_plasma_membrane
_01 12 0 1 1 0 0 GO:
0007346 Regulation_of_mitotic_cell_cycle
_01 23 0 1 1 0 0 GO:
0007368 Determination_of_left/right_symmetry
_01 14 1 1 1 0 0.000339 GO:
0007369 Gastrulation
_01 6 0 1 1 0 0 GO:
0007379 Segment_specification
_01 56 2 0.001235 0.187451 0 5.75e-05 GO:
0007389 Pattern_specification_process
_01 11 0 1 1 0 0 GO:
0007398 Ectoderm_development
_01 272 1 1 1 0 5e-05 GO:

0007399 Nervous_system_development
_01 8 0 1 1 0 0 GO:
0007405 Neuroblast_proliferation
_01 43 0 1 1 0 0 GO:
0007409 Axonogenesis
_01 200 0 1 1 0 0 GO:
0007411 Axon_guidance
_01 10 0 1 1 0 0 GO:
0007413 Axonal_fasciculation
_01 29 0 1 1 0 0 GO:
0007416 Synapse_assembly
_01 60 0 1 1 0 0 GO:
0007417 Central_nervous_system_development
_01 83 0 1 1 0 0 GO:
0007420 Brain_development
_01 18 0 1 1 0 0 GO:
0007422 Peripheral_nervous_system_development
_01 15 0 1 1 0 0 GO:
0007492 Endoderm_development
_01 21 1 1 1 0 5e-05 GO:
0007498 Mesoderm_development
_01 80 0 1 1 0 0 GO:
0007507 Heart_development
_01 9 0 1 1 0 0 GO:
0007512 Adult_heart_development
_01 62 0 1 1 0 0 GO:
0007517 Muscle_organ_development
_01 30 0 1 1 0 0 GO:
0007519 Skeletal_muscle_tissue_development
_01 7 0 1 1 0 0 GO:
0007520 Myoblast_fusion
_01 11 0 1 1 0 0 GO:
0007528 Neuromuscular_junction_development
_01 5 0 1 1 0 0 GO:
0007530 Sex_determination
_01 12 0 1 1 0 0 GO:
0007548 Sex_differentiation
_01 41 0 1 1 0 0 GO:
0007565 Female_pregnancy
_01 17 1 1 1 0 3.9e-05 GO:
0007566 Embryo_implantation
_01 60 0 1 1 0 0 GO:
0007568 Aging
_01 21 0 1 1 0 0 GO:
0007569 Cell_aging
_01 51 0 1 1 0 0 GO:
0007584 Response_to_nutrient
_01 23 0 1 1 0 0 GO:
0007585 Respiratory_gaseous_exchange
_01 29 0 1 1 0 0 GO:
0007586 Digestion
_01 25 0 1 1 0 0 GO:
0007588 Excretion
_01 8 0 1 1 0 0 GO:

0007589 Body_fluid_secretion
_01 18 0 1 1 0 0 GO:
0007595 Lactation
_01 230 3 0.00111 0.175702 0 0.000270667
GO:0007596 Blood_coagulation
_01 8 0 1 1 0 0 GO:
0007597 Blood_coagulation,_intrinsic_pathway
_01 5 0 1 1 0 0 GO:
0007599 Hemostasis
_01 14 0 1 1 0 0 GO:
0007600 Sensory_perception
_01 126 0 1 1 0 0 GO:
0007601 Visual_perception
_01 19 0 1 1 0 0 GO:
0007602 Phototransduction
_01 68 0 1 1 0 0 GO:
0007605 Sensory_perception_of_sound
_01 291 0 1 1 0 0 GO:
0007608 Sensory_perception_of_smell
_01 21 0 1 1 0 0 GO:
0007610 Behavior
_01 19 0 1 1 0 0 GO:
0007611 Learning_or_memory
_01 20 0 1 1 0 0 GO:
0007612 Learning
_01 22 1 1 1 0 3.9e-05 GO:
0007613 Memory
_01 9 0 1 1 0 0 GO:
0007616 Long-term_memory
_01 5 0 1 1 0 0 GO:
0007617 Mating_behavior
_01 25 0 1 1 0 0 GO:
0007623 Circadian_rhythm
_01 10 0 1 1 0 0 GO:
0007625 Grooming_behavior
_01 37 1 1 1 0 6.4e-05 GO:
0007626 Locomotory_behavior
_01 12 0 1 1 0 0 GO:
0007628 Adult_walking_behavior
_01 18 0 1 1 0 0 GO:
0007631 Feeding_behavior
_01 32 0 1 1 0 0 GO:
0008009 Chemokine_activity
_01 27 0 1 1 0 0 GO:
0008013 Beta-catenin_binding
_01 27 0 1 1 0 0 GO:
0008015 Blood_circulation
_01 25 0 1 1 0 0 GO:
0008016 Regulation_of_heart_contraction
_01 43 0 1 1 0 0 GO:
0008017 Microtubule_binding
_01 5 0 1 1 0 0 GO:
0008020 G-protein_coupled_photoreceptor_activity
_01 36 0 1 1 0 0 GO:

0008021 Synaptic_vesicle
_01 102 0 1 1 0 0 GO:
0008022 Protein_C-terminus_binding
_01 10 0 1 1 0 0 GO:
0008023 Transcription_elongation_factor_complex
_01 41 1 1 1 0 5.9e-05 GO:
0008026 ATP-dependent_helicase_activity
_01 8 0 1 1 0 0 GO:
0008028 Monocarboxylic_acid_transmembrane_transporter_activity
_01 38 0 1 1 0 0 GO:
0008033 TRNA_processing
_01 5 0 1 1 0 0 GO:
0008035 High-density_lipoprotein_binding
_01 7 0 1 1 0 0 GO:
0008037 Cell_recognition
_01 5 0 1 1 0 0 GO:
0008038 Neuron_recognition
_01 10 0 1 1 0 0 GO:
0008045 Motor_axon_guidance
_01 30 0 1 1 0 0 GO:
0008047 Enzyme_activator_activity
_01 21 0 1 1 0 0 GO:
0008060 ARF_GTPase_activator_activity
_01 11 0 1 1 0 0 GO:
0008066 Glutamate_receptor_activity
_01 64 0 1 1 0 0 GO:
0008076 Voltage-gated_potassium_channel_complex
_01 15 0 1 1 0 0 GO:
0008080 N-acetyltransferase_activity
_01 6 0 1 1 0 0 GO:
0008081 Phosphoric_diester_hydrolase_activity
_01 119 1 1 1 0 5.2e-05 GO:
0008083 Growth_factor_activity
_01 6 0 1 1 0 0 GO:
0008091 Spectrin
_01 27 1 1 1 0 5e-05 GO:
0008092 Cytoskeletal_protein_binding
_01 14 0 1 1 0 0 GO:
0008093 Cytoskeletal_adaptor_activity
_01 14 0 1 1 0 0 GO:
0008094 DNA-dependent_ATPase_activity
_01 36 0 1 1 0 0 GO:
0008104 Protein_localization
_01 6 0 1 1 0 0 GO:
0008113 Peptide-methionine-(S)-S-oxide_reductase_activity
_01 145 2 0.00613999 0.407546 0 6.6e-05
GO:0008134 Transcription_factor_binding
_01 13 0 1 1 0 0 GO:
0008135 Translation_factor_activity,_nucleic_acid_binding
_01 18 0 1 1 0 0 GO:
0008137 NADH_dehydrogenase_(ubiquinone)_activity
_01 21 0 1 1 0 0 GO:
0008138 Protein_tyrosine/serine/threonine_phosphatase_activity
_01 7 0 1 1 0 0 GO:

0008139 Nuclear_localization_sequence_binding
_01 8 0 1 1 0 0 GO:
0008143 Poly(A)_RNA_binding
_01 37 0 1 1 0 0 GO:
0008144 Drug_binding
_01 25 0 1 1 0 0 GO:
0008146 Sulfotransferase_activity
_01 361 1 1 1 0 0.000423 GO:
0008150 Biological_process
_01 119 1 1 1 0 8.3e-05 GO:
0008152 Metabolic_process
_01 5 0 1 1 0 0 GO:
0008154 Actin_polymerization_or_depolymerization
_01 11 0 1 1 0 0 GO:
0008156 Negative_regulation_of_DNA_replication
_01 5 0 1 1 0 0 GO:
0008158 Hedgehog_receptor_activity
_01 67 1 1 1 0 0.000423 GO:
0008168 Methyltransferase_activity
_01 5 0 1 1 0 0 GO:
0008180 Signalosome
_01 7 0 1 1 0 0 GO:
0008188 Neuropeptide_receptor_activity
_01 9 0 1 1 0 0 GO:
0008191 Metalloendopeptidase_inhibitor_activity
_01 8 0 1 1 0 0 GO:
0008195 Phosphatidate_phosphatase_activity
_01 10 0 1 1 0 0 GO:
0008198 Ferrous_iron_binding
_01 7 0 1 1 0 0 GO:
0008199 Ferric_iron_binding
_01 5 0 1 1 0 0 GO:
0008200 Ion_channel_inhibitor_activity
_01 80 1 1 1 0 0.000339 GO:
0008201 Heparin_binding
_01 47 0 1 1 0 0 GO:
0008202 Steroid_metabolic_process
_01 37 0 1 1 0 0 GO:
0008203 Cholesterol_metabolic_process
_01 19 0 1 1 0 0 GO:
0008206 Bile_acid_metabolic_process
_01 5 0 1 1 0 0 GO:
0008209 Androgen_metabolic_process
_01 8 0 1 1 0 0 GO:
0008210 Estrogen_metabolic_process
_01 37 1 1 1 0 3.9e-05 GO:
0008217 Regulation_of_blood_pressure
_01 89 0 1 1 0 0 GO:
0008219 Cell_death
_01 293 2 0.019572 0.577468 0 7.35e-05 GO:
0008233 Peptidase_activity
_01 34 0 1 1 0 0 GO:
0008234 Cysteine-type_peptidase_activity
_01 7 0 1 1 0 0 GO:

0008235 Metalloexopeptidase_activity
_01 28 0 1 1 0 0 GO:
0008236 Serine-type_peptidase_activity
_01 58 1 1 1 0 6.4e-05 GO:
0008237 Metallopeptidase_activity
_01 7 0 1 1 0 0 GO:
0008239 Dipeptidyl-peptidase_activity
_01 7 0 1 1 0 0 GO:
0008250 Oligosaccharyltransferase_complex
_01 6 0 1 1 0 0 GO:
0008253 5'-nucleotidase_activity
_01 5 0 1 1 0 0 GO:
0008266 Poly(U)_RNA_binding
_01 5 0 1 1 0 0 GO:
0008271 Secondary_active_sulfate_transmembrane_transporter_activity
_01 10 0 1 1 0 0 GO:
0008272 Sulfate_transport
_01 9 0 1 1 0 0 GO:
0008276 Protein_methyltransferase_activity
_01 18 0 1 1 0 0 GO:
0008277 Regulation_of_G-
protein_coupled_receptor_protein_signaling_pathway
_01 195 1 1 1 0 0.000344 GO:
0008283 Cell_proliferation
_01 220 0 1 1 0 0 GO:
0008284 Positive_regulation_of_cell_proliferation
_01 218 1 1 1 0 3.9e-05 GO:
0008285 Negative_regulation_of_cell_proliferation
_01 97 0 1 1 0 0 GO:
0008286 Insulin_receptor_signaling_pathway
_01 6 1 1 1 0 0.000339 GO:
0008287 Protein_serine/threonine_phosphatase_complex
_01 86 1 1 1 0 3.9e-05 GO:
0008289 Lipid_binding
_01 6 1 1 1 0 6.9e-05 GO:
0008290 F-actin_capping_protein_complex
_01 11 0 1 1 0 0 GO:
0008299 Isoprenoid_biosynthetic_process
_01 33 1 1 1 0 6.3e-05 GO:
0008301 DNA_bending_activity
_01 17 0 1 1 0 0 GO:
0008305 Integrin_complex
_01 6 0 1 1 0 0 GO:
0008306 Associative_learning
_01 33 0 1 1 0 0 GO:
0008307 Structural_constituent_of_muscle
_01 5 0 1 1 0 0 GO:
0008312 7S_RNA_binding
_01 11 0 1 1 0 0 GO:
0008324 Cation_transmembrane_transporter_activity
_01 12 0 1 1 0 0 GO:
0008333 Endosome_to_lysosome_transport
_01 6 0 1 1 0 0 GO:
0008334 Histone_mRNA_metabolic_process

_01 5 0 1 1 0 0 GO:
0008340 Determination_of_adult_lifespan
_01 6 0 1 1 0 0 GO:
0008343 Adult_feeding_behavior
_01 12 0 1 1 0 0 GO:
0008344 Adult_locomotory_behavior
_01 8 0 1 1 0 0 GO:
0008353 RNA_polymerase_II_carboxy-terminal_domain_kinase_activity
_01 43 1 1 1 0 5e-05 GO:
0008360 Regulation_of_cell_shape
_01 12 0 1 1 0 0 GO:
0008373 Sialyltransferase_activity
_01 8 0 1 1 0 0 GO:
0008375 Acetylglucosaminyltransferase_activity
_01 13 1 1 1 0 0.000423 GO:
0008378 Galactosyltransferase_activity
_01 183 1 1 1 0 5.2e-05 GO:
0008380 RNA_splicing
_01 6 0 1 1 0 0 GO:
0008406 Gonad_development
_01 10 0 1 1 0 0 GO:
0008408 3'-5'_exonuclease_activity
_01 6 0 1 1 0 0 GO:
0008409 5'-3'_exonuclease_activity
_01 76 0 1 1 0 0 GO:
0008415 Acyltransferase_activity
_01 5 0 1 1 0 0 GO:
0008417 Fucosyltransferase_activity
_01 12 0 1 1 0 0 GO:
0008430 Selenium_binding
_01 5 0 1 1 0 0 GO:
0008467 [heparan_sulfate]-glucosamine_3-sulfotransferase_1_activity
_01 5 0 1 1 0 0 GO:
0008483 Transaminase_activity
_01 5 0 1 1 0 0 GO:
0008503 Benzodiazepine_receptor_activity
_01 5 0 1 1 0 0 GO:
0008509 Anion_transmembrane_transporter_activity
_01 5 0 1 1 0 0 GO:
0008514 Organic_anion_transmembrane_transporter_activity
_01 4 0 1 1 0 0 GO:
0008519 Ammonium_transmembrane_transporter_activity
_01 5 0 1 1 0 0 GO:
0008527 Taste_receptor_activity
_01 6 0 1 1 0 0 GO:
0008528 Peptide_receptor_activity,_G-protein_coupled
_01 6 0 1 1 0 0 GO:
0008535 Respiratory_chain_complex_IV_assembly
_01 7 0 1 1 0 0 GO:
0008536 Ran_GTPase_binding
_01 14 0 1 1 0 0 GO:
0008542 Visual_learning
_01 38 0 1 1 0 0 GO:
0008543 Fibroblast_growth_factor_receptor_signaling_pathway

_01 54 0 1 1 0 0 GO:
0008544 Epidermis_development
_01 7 0 1 1 0 0 GO:
0008553 Hydrogen-
exporting_ATPase_activity,_phosphorylative_mechanism
_01 47 0 1 1 0 0 GO:
0008565 Protein_transporter_activity
_01 33 0 1 1 0 0 GO:
0008584 Male_gonad_development
_01 7 0 1 1 0 0 GO:
0008585 Female_gonad_development
_01 9 0 1 1 0 0 GO:
0008589 Regulation_of_smoothened_signaling_pathway
_01 6 0 1 1 0 0 GO:
0008595 Anterior/posterior_axis_specification,_embryo
_01 13 0 1 1 0 0 GO:
0008601 Protein_phosphatase_type_2A_regulator_activity
_01 6 0 1 1 0 0 GO:
0008603 CAMP-dependent_protein_kinase_regulator_activity
_01 13 0 1 1 0 0 GO:
0008610 Lipid_biosynthetic_process
_01 52 0 1 1 0 0 GO:
0008624 Induction_of_apoptosis_by_extracellular_signals
_01 6 1 1 1 0 0.000423 GO:
0008625 Induction_of_apoptosis_via_death_domain_receptors
_01 28 1 1 1 0 0.000423 GO:
0008629 Induction_of_apoptosis_by_intracellular_signals
_01 10 0 1 1 0 0 GO:
0008630
DNA_damage_response,_signal_transduction_resulting_in_induction_of_a
poptosis
_01 6 1 1 1 0 0.000423 GO:
0008631 Induction_of_apoptosis_by_oxidative_stress
_01 19 0 1 1 0 0 GO:
0008633 Activation_of_pro-apoptotic_gene_products
_01 5 1 1 1 0 6.3e-05 GO:
0008634 Negative_regulation_of_survival_gene_product_expression
_01 6 1 1 1 0 0.000423 GO:
0008635 Activation_of_caspase_activity_by_cytochrome_c
_01 6 0 1 1 0 0 GO:
0008637 Apoptotic_mitochondrial_changes
_01 22 0 1 1 0 0 GO:
0008643 Carbohydrate_transport
_01 24 0 1 1 0 0 GO:
0008645 Hexose_transport
_01 18 0 1 1 0 0 GO:
0008652 Cellular_amino_acid_biosynthetic_process
_01 29 0 1 1 0 0 GO:
0008654 Phospholipid_biosynthetic_process
_01 7 0 1 1 0 0 GO:
0008656 Caspase_activator_activity
_01 5 1 1 1 0 0.000344 GO:
0008889 Glycerophosphodiester_phosphodiesterase_activity
_01 89 0 1 1 0 0 GO:

0009055 Electron_carrier_activity
_01 27 0 1 1 0 0 GO:
0009058 Biosynthetic_process
_01 7 0 1 1 0 0 GO:
0009060 Aerobic_respiration
_01 6 0 1 1 0 0 GO:
0009062 Fatty_acid_catabolic_process
_01 6 0 1 1 0 0 GO:
0009072 Aromatic_amino_acid_family_metabolic_process
_01 11 0 1 1 0 0 GO:
0009083 Branched_chain_family_amino_acid_catabolic_process
_01 5 0 1 1 0 0 GO:
0009100 Glycoprotein_metabolic_process
_01 6 0 1 1 0 0 GO:
0009101 Glycoprotein_biosynthetic_process
_01 5 0 1 1 0 0 GO:
0009103 Lipopolysaccharide_biosynthetic_process
_01 8 0 1 1 0 0 GO:
0009116 Nucleoside_metabolic_process
_01 18 0 1 1 0 0 GO:
0009117 Nucleotide_metabolic_process
_01 13 0 1 1 0 0 GO:
0009168 Purine_ribonucleoside_monophosphate_biosynthetic_process
_01 12 0 1 1 0 0 GO:
0009267 Cellular_response_to_starvation
_01 6 0 1 1 0 0 GO:
0009268 Response_to_pH
_01 9 0 1 1 0 0 GO:
0009303 RRNA_transcription
_01 12 0 1 1 0 0 GO:
0009306 Protein_secretion
_01 7 0 1 1 0 0 GO:
0009311 Oligosaccharide_metabolic_process
_01 6 0 1 1 0 0 GO:
0009312 Oligosaccharide_biosynthetic_process
_01 16 0 1 1 0 0 GO:
0009314 Response_to_radiation
_01 8 0 1 1 0 0 GO:
0009395 Phospholipid_catabolic_process
_01 6 0 1 1 0 0 GO:
0009396 Folic_acid-containing_compound_biosynthetic_process
_01 35 0 1 1 0 0 GO:
0009408 Response_to_heat
_01 18 0 1 1 0 0 GO:
0009409 Response_to_cold
_01 19 0 1 1 0 0 GO:
0009411 Response_to_UV
_01 14 0 1 1 0 0 GO:
0009416 Response_to_light_stimulus
_01 9 0 1 1 0 0 GO:
0009434 Microtubule-based_flagellum
_01 6 0 1 1 0 0 GO:
0009435 NAD_biosynthetic_process
_01 10 0 1 1 0 0 GO:

0009566 Fertilization
_01 6 0 1 1 0 0 GO:
0009605 Response_to_external_stimulus
_01 11 0 1 1 0 0 GO:
0009607 Response_to_biotic_stimulus
_01 37 0 1 1 0 0 GO:
0009611 Response_to_wounding
_01 33 0 1 1 0 0 GO:
0009612 Response_to_mechanical_stimulus
_01 81 0 1 1 0 0 GO:
0009615 Response_to_virus
_01 8 0 1 1 0 0 GO:
0009617 Response_to_bacterium
_01 6 0 1 1 0 0 GO:
0009629 Response_to_gravity
_01 39 0 1 1 0 0 GO:
0009636 Response_to_toxin
_01 9 0 1 1 0 0 GO:
0009650 UV_protection
_01 5 0 1 1 0 0 GO:
0009651 Response_to_salt_stress
_01 64 2 0.000601999 0.124557 0
0.0001945 GO:0009653 Anatomical_structure_morphogenesis
_01 35 0 1 1 0 0 GO:
0009725 Response_to_hormone_stimulus
_01 7 0 1 1 0 0 GO:
0009743 Response_to_carbohydrate_stimulus
_01 5 0 1 1 0 0 GO:
0009744 Response_to_sucrose_stimulus
_01 38 0 1 1 0 0 GO:
0009749 Response_to_glucose_stimulus
_01 5 1 1 1 0 3.9e-05 GO:
0009750 Response_to_fructose_stimulus
_01 29 0 1 1 0 0 GO:
0009755 Hormone-mediated_signaling_pathway
_01 69 1 1 1 0 6.3e-05 GO:
0009790 Embryo_development
_01 41 0 1 1 0 0 GO:
0009791 Post-embryonic_development
_01 8 0 1 1 0 0 GO:
0009798 Axis_specification
_01 10 0 1 1 0 0 GO:
0009880 Embryonic_pattern_specification
_01 6 0 1 1 0 0 GO:
0009881 Photoreceptor_activity
_01 80 0 1 1 0 0 GO:
0009887 Organ_morphogenesis
_01 22 0 1 1 0 0 GO:
0009888 Tissue_development
_01 80 0 1 1 0 0 GO:
0009897 External_side_of_plasma_membrane
_01 14 0 1 1 0 0 GO:
0009898 Internal_side_of_plasma_membrane
_01 15 0 1 1 0 0 GO:

0009925 Basal_plasma_membrane
_01 9 0 1 1 0 0 GO:
0009948 Anterior/posterior_axis_specification
_01 7 0 1 1 0 0 GO:
0009950 Dorsal/ventral_axis_specification
_01 62 0 1 1 0 0 GO:
0009952 Anterior/posterior_pattern_formation
_01 22 0 1 1 0 0 GO:
0009953 Dorsal/ventral_pattern_formation
_01 10 0 1 1 0 0 GO:
0009954 Proximal/distal_pattern_formation
_01 12 0 1 1 0 0 GO:
0009966 Regulation_of_signal_transduction
_01 7 0 1 1 0 0 GO:
0009967 Positive_regulation_of_signal_transduction
_01 31 0 1 1 0 0 GO:
0009968 Negative_regulation_of_signal_transduction
_01 9 0 1 1 0 0 GO:
0009982 Pseudouridine_synthase_activity
_01 161 0 1 1 0 0 GO:
0009986 Cell_surface
_01 14 0 1 1 0 0 GO:
0009987 Cellular_process
_01 9 0 1 1 0 0 GO:
0010001 Glial_cell_differentiation
_01 63 0 1 1 0 0 GO:
0010008 Endosome_membrane
_01 21 0 1 1 0 0 GO:
0010033 Response_to_organic_substance
_01 12 0 1 1 0 0 GO:
0010035 Response_to_inorganic_substance
_01 5 0 1 1 0 0 GO:
0010038 Response_to_metal_ion
_01 12 0 1 1 0 0 GO:
0010043 Response_to_zinc_ion
_01 8 0 1 1 0 0 GO:
0010165 Response_to_X-ray
_01 9 0 1 1 0 0 GO:
0010181 FMN_binding
_01 18 0 1 1 0 0 GO:
0010212 Response_to_ionizing_radiation
_01 5 0 1 1 0 0 GO:
0010224 Response_to_UV-B
_01 5 0 1 1 0 0 GO:
0010225 Response_to_UV-C
_01 24 1 1 1 0 3.9e-05 GO:
0010243 Response_to_organic_nitrogen
_01 5 0 1 1 0 0 GO:
0010269 Response_to_selenium_ion
_01 9 1 1 1 0 0.000339 GO:
0010288 Response_to_lead_ion
_01 5 0 1 1 0 0 GO:
0010310 Regulation_of_hydrogen_peroxide_metabolic_process
_01 16 0 1 1 0 0 GO:

0010332 Response_to_gamma_radiation
_01	6	0	1	1	0	0	GO:
0010390 Histone_monoubiquitination
_01	263	1	1	1	0	5.2e-05	GO:
0010467 Gene_expression
_01	26	0	1	1	0	0	GO:
0010468 Regulation_of_gene_expression
_01	9	0	1	1	0	0	GO:
0010494 Stress_granule
_01	7	0	1	1	0	0	GO:
0010510 Regulation_of_acetyl-CoA_biosynthetic_process_from_pyruvate
_01	10	0	1	1	0	0	GO:
0010524 Positive_regulation_of_calcium_ion_transport_into_cytosol
_01	5	0	1	1	0	0	GO:
0010544 Negative_regulation_of_platelet_activation
_01	46	1	1	1	0	6.3e-05	GO:
0010551 Regulation_of_gene-
specific_transcription_from_RNA_polymerase_II_promoter
_01	74	1	1	1	0	6.3e-05	GO:
0010552 Positive_regulation_of_gene-
specific_transcription_from_RNA_polymerase_II_promoter
_01	53	0	1	1	0	0	GO:
0010553 Negative_regulation_of_gene-
specific_transcription_from_RNA_polymerase_II_promoter
_01	9	1	1	1	0	3.9e-05	GO:
0010575
Positive_regulation_vascular_endothelial_growth_factor_production
_01	11	0	1	1	0	0	GO:
0010595 Positive_regulation_of_endothelial_cell_migration
_01	6	0	1	1	0	0	GO:
0010596 Negative_regulation_of_endothelial_cell_migration
_01	35	0	1	1	0	0	GO:
0010628 Positive_regulation_of_gene_expression
_01	26	0	1	1	0	0	GO:
0010629 Negative_regulation_of_gene_expression
_01	7	0	1	1	0	0	GO:
0010634 Positive_regulation_of_epithelial_cell_migration
_01	6	0	1	1	0	0	GO:
0010670
Positive_regulation_of_oxygen_and_reactive_oxygen_species_metabolic_
process
_01	11	0	1	1	0	0	GO:
0010718 Positive_regulation_of_epithelial_to_mesenchymal_transition
_01	5	0	1	1	0	0	GO:
0010719 Negative_regulation_of_epithelial_to_mesenchymal_transition
_01	10	0	1	1	0	0	GO:
0010744
Positive_regulation_of_macrophage_derived_foam_cell_differentiation
_01	7	0	1	1	0	0	GO:
0010745
Negative_regulation_of_macrophage_derived_foam_cell_differentiation
_01	6	0	1	1	0	0	GO:
0010765 Positive_regulation_of_sodium_ion_transport
_01	9	0	1	1	0	0	GO:

0010811 Positive_regulation_of_cell-substrate_adhesion
_01 6 0 1 1 0 0 GO:
0010812 Negative_regulation_of_cell-substrate_adhesion
_01 16 0 1 1 0 0 GO:
0010827 Regulation_of_glucose_transport
_01 111 1 1 1 0 6.3e-05 GO:
0010843 Promoter_binding
_01 13 0 1 1 0 0 GO:
0010862 Positive_regulation_of_pathway-
restricted_SMAD_protein_phosphorylation
_01 5 0 1 1 0 0 GO:
0010873 Positive_regulation_of_cholesterol_esterification
_01 7 0 1 1 0 0 GO:
0010875 Positive_regulation_of_cholesterol_efflux
_01 6 0 1 1 0 0 GO:
0010886 Positive_regulation_of_cholesterol_storage
_01 5 0 1 1 0 0 GO:
0010890 Positive_regulation_of_sequestering_of_triglyceride
_01 6 0 1 1 0 0 GO:
0010975 Regulation_of_neuron_projection_development
_01 9 0 1 1 0 0 GO:
0010976 Positive_regulation_of_neuron_projection_development
_01 6 0 1 1 0 0 GO:
0010977 Negative_regulation_of_neuron_projection_development
_01 58 1 1 1 0 0.000423 GO:
0012505 Endomembrane_system
_01 10 0 1 1 0 0 GO:
0012506 Vesicle_membrane
_01 6 0 1 1 0 0 GO:
0012507 ER_to_Golgi_transport_vesicle_membrane
_01 9 0 1 1 0 0 GO:
0014003 Oligodendrocyte_development
_01 5 0 1 1 0 0 GO:
0014032 Neural_crest_cell_development
_01 13 0 1 1 0 0 GO:
0014047 Glutamate_secretion
_01 5 0 1 1 0 0 GO:
0014049 Positive_regulation_of_glutamate_secretion
_01 6 0 1 1 0 0 GO:
0014050 Negative_regulation_of_glutamate_secretion
_01 5 0 1 1 0 0 GO:
0014059 Regulation_of_dopamine_secretion
_01 8 0 1 1 0 0 GO:
0014065 Phosphoinositide_3-kinase_cascade
_01 14 0 1 1 0 0 GO:
0014068 Positive_regulation_of_phosphoinositide_3-kinase_cascade
_01 62 0 1 1 0 0 GO:
0014069 Postsynaptic_density
_01 70 2 0.000727999 0.137657 0 0.000189
GO:0014070 Response_to_organic_cyclic_substance
_01 13 0 1 1 0 0 GO:
0014823 Response_to_activity
_01 5 0 1 1 0 0 GO:
0014902 Myotube_differentiation

_01	10	0	1	1	0	0	GO:
0014911 Positive_regulation_of_smooth_muscle_cell_migration
_01	6	0	1	1	0	0	GO:
0014912 Negative_regulation_of_smooth_muscle_cell_migration
_01	5	0	1	1	0	0	GO:
0015012 Heparan_sulfate_proteoglycan_biosynthetic_process
_01	8	0	1	1	0	0	GO:
0015020 Glucuronosyltransferase_activity
_01	9	0	1	1	0	0	GO:
0015026 Coreceptor_activity
_01	24	0	1	1	0	0	GO:
0015030 Cajal_body
_01	256	0	1	1	0	0	GO:
0015031 Protein_transport
_01	14	0	1	1	0	0	GO:
0015035 Protein_disulfide_oxidoreductase_activity
_01	6	0	1	1	0	0	GO:
0015074 DNA_integration
_01	12	0	1	1	0	0	GO:
0015075 Ion_transmembrane_transporter_activity
_01	14	0	1	1	0	0	GO:
0015078 Hydrogen_ion_transmembrane_transporter_activity
_01	6	0	1	1	0	0	GO:
0015101 Organic_cation_transmembrane_transporter_activity
_01	21	0	1	1	0	0	GO:
0015171 Amino_acid_transmembrane_transporter_activity
_01	5	0	1	1	0	0	GO:
0015175 Neutral_amino_acid_transmembrane_transporter_activity
_01	6	0	1	1	0	0	GO:
0015250 Water_channel_activity
_01	7	0	1	1	0	0	GO:
0015267 Channel_activity
_01	8	0	1	1	0	0	GO:
0015269 Calcium-activated_potassium_channel_activity
_01	6	0	1	1	0	0	GO:
0015277 Kainate_selective_glutamate_receptor_activity
_01	77	0	1	1	0	0	GO:
0015293 Symporter_activity
_01	23	0	1	1	0	0	GO:
0015297 Antiporter_activity
_01	7	0	1	1	0	0	GO:
0015299 Solute:hydrogen_antiporter_activity
_01	5	0	1	1	0	0	GO:
0015301 Anion:anion_antiporter_activity
_01	6	0	1	1	0	0	GO:
0015321 Sodium-
dependent_phosphate_transmembrane_transporter_activity
_01	6	0	1	1	0	0	GO:
0015347 Sodium-
independent_organic_anion_transmembrane_transporter_activity
_01	5	0	1	1	0	0	GO:
0015355
Secondary_active_monocarboxylate_transmembrane_transporter_activity
_01	6	0	1	1	0	0	GO:

0015385 Sodium:hydrogen_antiporter_activity
_01	7	0	1	1	0	0	GO:
0015450 P-P-bond-hydrolysis-driven_protein_transmembrane_transporter_activity
_01	14	0	1	1	0	0	GO:
0015459 Potassium_channel_regulator_activity
_01	8	0	1	1	0	0	GO:
0015464 Acetylcholine_receptor_activity
_01	11	0	1	1	0	0	GO:
0015485 Cholesterol_binding
_01	107	1	1	1	0	5e-05	GO:
0015629 Actin_cytoskeleton
_01	52	0	1	1	0	0	GO:
0015630 Microtubule_cytoskeleton
_01	16	1	1	1	0	5e-05	GO:
0015631 Tubulin_binding
_01	16	0	1	1	0	0	GO:
0015662
ATPase_activity,_coupled_to_transmembrane_movement_of_ions,_phosphorylative_mechanism
_01	5	0	1	1	0	0	GO:
0015671 Oxygen_transport
_01	7	0	1	1	0	0	GO:
0015695 Organic_cation_transport
_01	6	0	1	1	0	0	GO:
0015696 Ammonium_transport
_01	8	0	1	1	0	0	GO:
0015701 Bicarbonate_transport
_01	9	0	1	1	0	0	GO:
0015711 Organic_anion_transport
_01	5	0	1	1	0	0	GO:
0015718 Monocarboxylic_acid_transport
_01	6	0	1	1	0	0	GO:
0015721 Bile_acid_and_bile_salt_transport
_01	36	0	1	1	0	0	GO:
0015758 Glucose_transport
_01	5	0	1	1	0	0	GO:
0015804 Neutral_amino_acid_transport
_01	6	0	1	1	0	0	GO:
0015840 Urea_transport
_01	6	0	1	1	0	0	GO:
0015884 Folic_acid_transport
_01	11	0	1	1	0	0	GO:
0015914 Phospholipid_transport
_01	6	0	1	1	0	0	GO:
0015934 Large_ribosomal_subunit
_01	11	0	1	1	0	0	GO:
0015935 Small_ribosomal_subunit
_01	5	0	1	1	0	0	GO:
0015937 Coenzyme_A_biosynthetic_process
_01	6	0	1	1	0	0	GO:
0015939 Pantothenate_metabolic_process
_01	12	0	1	1	0	0	GO:
0015949 Nucleobase,_nucleoside_and_nucleotide_interconversion

_01 19 0 1 1 0 0 GO:
0015986 ATP_synthesis_coupled_proton_transport
_01 30 0 1 1 0 0 GO:
0015992 Proton_transport
_01 9 0 1 1 0 0 GO:
0016010 Dystrophin-associated_glycoprotein_complex
_01 5 0 1 1 0 0 GO:
0016012 Sarcoglycan_complex
_01 6 0 1 1 0 0 GO:
0016018 Cyclosporin_A_binding
_01 64 1 1 1 0 5e-05 GO:
0016023 Cytoplasmic_membrane-bounded_vesicle
_01 195 0 1 1 0 0 GO:
0016032 Viral_reproduction
_01 56 1 1 1 0 0.000344 GO:
0016042 Lipid_catabolic_process
_01 11 0 1 1 0 0 GO:
0016043 Cellular_component_organization
_01 41 0 1 1 0 0 GO:
0016044 Cellular_membrane_organization
_01 35 0 1 1 0 0 GO:
0016049 Cell_growth
_01 5 0 1 1 0 0 GO:
0016050 Vesicle_organization
_01 13 0 1 1 0 0 GO:
0016051 Carbohydrate_biosynthetic_process
_01 58 0 1 1 0 0 GO:
0016055 Wnt_receptor_signaling_pathway
_01 55 0 1 1 0 0 GO:
0016070 RNA_metabolic_process
_01 33 0 1 1 0 0 GO:
0016071 MRNA_metabolic_process
_01 6 1 1 1 0 5e-05 GO:
0016079 Synaptic_vesicle_exocytosis
_01 6 0 1 1 0 0 GO:
0016126 Sterol_biosynthetic_process
_01 5 0 1 1 0 0 GO:
0016165 Lipoxygenase_activity
_01 8 0 1 1 0 0 GO:
0016180 SnRNA_processing
_01 117 0 1 1 0 0 GO:
0016192 Vesicle-mediated_transport
_01 17 0 1 1 0 0 GO:
0016197 Endosome_transport
_01 7 0 1 1 0 0 GO:
0016208 AMP_binding
_01 9 0 1 1 0 0 GO:
0016209 Antioxidant_activity
_01 6 0 1 1 0 0 GO:
0016226 Iron-sulfur_cluster_assembly
_01 5 0 1 1 0 0 GO:
0016234 Inclusion_body
_01 6 0 1 1 0 0 GO:
0016235 Aggresome

_01 11 0 1 1 0 0 GO:
0016251 General_RNA_polymerase_II_transcription_factor_activity
_01 10 0 1 1 0 0 GO:
0016254 Preassembly_of_GPI_anchor_in_ER_membrane
_01 5 0 1 1 0 0 GO:
0016272 Prefoldin_complex
_01 79 0 1 1 0 0 GO:
0016301 Kinase_activity
_01 7 0 1 1 0 0 GO:
0016303 1-phosphatidylinositol-3-kinase_activity
_01 5 0 1 1 0 0 GO:
0016308 1-phosphatidylinositol-4-phosphate_5-kinase_activity
_01 15 0 1 1 0 0 GO:
0016310 Phosphorylation
_01 9 0 1 1 0 0 GO:
0016311 Dephosphorylation
_01 80 0 1 1 0 0 GO:
0016323 Basolateral_plasma_membrane
_01 117 0 1 1 0 0 GO:
0016324 Apical_plasma_membrane
_01 14 0 1 1 0 0 GO:
0016328 Lateral_plasma_membrane
_01 9 0 1 1 0 0 GO:
0016331 Morphogenesis_of_embryonic_epithelium
_01 54 0 1 1 0 0 GO:
0016337 Cell-cell_adhesion
_01 14 0 1 1 0 0 GO:
0016338 Calcium-independent_cell-cell_adhesion
_01 19 0 1 1 0 0 GO:
0016339 Calcium-dependent_cell-cell_adhesion
_01 7 0 1 1 0 0 GO:
0016342 Catenin_complex
_01 14 0 1 1 0 0 GO:
0016358 Dendrite_development
_01 44 1 1 1 0 7.4e-05 GO:
0016363 Nuclear_matrix
_01 9 0 1 1 0 0 GO:
0016407 Acetyltransferase_activity
_01 5 0 1 1 0 0 GO:
0016409 Palmitoyltransferase_activity
_01 23 0 1 1 0 0 GO:
0016455 RNA_polymerase_II_transcription_mediator_activity
_01 29 0 1 1 0 0 GO:
0016459 Myosin_complex
_01 5 0 1 1 0 0 GO:
0016461 Unconventional_myosin_complex
_01 7 0 1 1 0 0 GO:
0016471 Vacuolar_proton-transporting_V-type_ATPase_complex
_01 51 2 0.001313 0.19455 0 5.7e-05 GO:
0016477 Cell_migration
_01 100 1 1 1 0 0.000423 GO:
0016481 Negative_regulation_of_transcription
_01 14 0 1 1 0 0 GO:
0016485 Protein_processing

_01 12 0 1 1 0 0 GO:
0016486 Peptide_hormone_processing
_01 263 2 0.011199 0.494678 0 0.000231 GO:
0016491 Oxidoreductase_activity
_01 14 0 1 1 0 0 GO:
0016493 C-C_chemokine_receptor_activity
_01 5 0 1 1 0 0 GO:
0016500 Protein-hormone_receptor_activity
_01 4 0 1 1 0 0 GO:
0016503 Pheromone_receptor_activity
_01 10 0 1 1 0 0 GO:
0016514 SWI/SNF_complex
_01 22 0 1 1 0 0 GO:
0016525 Negative_regulation_of_angiogenesis
_01 23 0 1 1 0 0 GO:
0016529 Sarcoplasmic_reticulum
_01 6 0 1 1 0 0 GO:
0016538 Cyclin-dependent_protein_kinase_regulator_activity
_01 5 0 1 1 0 0 GO:
0016558 Protein_import_into_peroxisome_matrix
_01 210 1 1 1 0 6.3e-05 GO:
0016563 Transcription_activator_activity
_01 151 1 1 1 0 7.4e-05 GO:
0016564 Transcription_repressor_activity
_01 6 0 1 1 0 0 GO:
0016565 General_transcriptional_repressor_activity
_01 61 1 1 1 0 6.3e-05 GO:
0016566 Specific_transcriptional_repressor_activity
_01 64 0 1 1 0 0 GO:
0016567 Protein_ubiquitination
_01 134 2 0.00593299 0.398216 0
0.0002485 GO:0016568 Chromatin_modification
_01 8 1 1 1 0 7.4e-05 GO:
0016571 Histone_methylation
_01 8 0 1 1 0 0 GO:
0016573 Histone_acetylation
_01 13 0 1 1 0 0 GO:
0016575 Histone_deacetylation
_01 8 0 1 1 0 0 GO:
0016578 Histone_deubiquitination
_01 17 0 1 1 0 0 GO:
0016579 Protein_deubiquitination
_01 5 0 1 1 0 0 GO:
0016580 Sin3_complex
_01 8 0 1 1 0 0 GO:
0016581 NuRD_complex
_01 5 0 1 1 0 0 GO:
0016585 Chromatin_remodeling_complex
_01 6 0 1 1 0 0 GO:
0016591 DNA-directed_RNA_polymerase_II,_holoenzyme
_01 24 0 1 1 0 0 GO:
0016592 Mediator_complex
_01 7 0 1 1 0 0 GO:
0016594 Glycine_binding

_01 6 0 1 1 0 0 GO:
0016595 Glutamate_binding
_01 10 0 1 1 0 0 GO:
0016597 Amino_acid_binding
_01 6 0 1 1 0 0 GO:
0016601 Rac_protein_signal_transduction
_01 11 0 1 1 0 0 GO:
0016604 Nuclear_body
_01 26 1 1 1 0 7.4e-05 GO:
0016605 PML_body
_01 85 1 1 1 0 0.000423 GO:
0016607 Nuclear_speck
_01 6 0 1 1 0 0 GO:
0016616 Oxidoreductase_activity,_acting_on_the_CH-
OH_group_of_donors,_NAD_or_NADP_as_acceptor
_01 38 2 0.000314 0.0893319 0 0.000231
GO:0016702
Oxidoreductase_activity,_acting_on_single_donors_with_incorporation_
of_molecular_oxygen,_incorporation_of_two_atoms_of_oxygen
_01 9 0 1 1 0 0 GO:
0016705
Oxidoreductase_activity,_acting_on_paired_donors,_with_incorporation
_or_reduction_of_molecular_oxygen
_01 711 2 0.1261 0.836466 0 0.0002365
GO:0016740 Transferase_activity
_01 88 1 1 1 0 0.000423 GO:
0016757 Transferase_activity,_transferring_glycosyl_groups
_01 12 0 1 1 0 0 GO:
0016758 Transferase_activity,_transferring_hexosyl_groups
_01 6 0 1 1 0 0 GO:
0016769 Transferase_activity,_transferring_nitrogenous_groups
_01 15 0 1 1 0 0 GO:
0016773 Phosphotransferase_activity,_alcohol_group_as_acceptor
_01 45 0 1 1 0 0 GO:
0016779 Nucleotidyltransferase_activity
_01 8 0 1 1 0 0 GO:
0016780
Phosphotransferase_activity,_for_other_substituted_phosphate_groups
_01 583 4 0.001131 0.177593 0 0.00012625
GO:0016787 Hydrolase_activity
_01 13 0 1 1 0 0 GO:
0016788 Hydrolase_activity,_acting_on_ester_bonds
_01 5 0 1 1 0 0 GO:
0016790 Thiolester_hydrolase_activity
_01 10 0 1 1 0 0 GO:
0016791 Phosphatase_activity
_01 29 0 1 1 0 0 GO:
0016798 Hydrolase_activity,_acting_on_glycosyl_bonds
_01 9 0 1 1 0 0 GO:
0016805 Dipeptidase_activity
_01 9 0 1 1 0 0 GO:
0016810 Hydrolase_activity,_acting_on_carbon-
nitrogen_(but_not_peptide)_bonds
_01 12 0 1 1 0 0 GO:

0016818
Hydrolase_activity,_acting_on_acid_anhydrides,_in_phosphorus-containing_anhydrides
_01	28	0	1	1	0	0	GO:
0016820
Hydrolase_activity,_acting_on_acid_anhydrides,_catalyzing_transmembrane_movement_of_substances
_01	67	2	0.000477	0.10964	0	5.1e-05	GO:
0016829 Lyase_activity
_01	6	0	1	1	0	0	GO:
0016831 Carboxy-lyase_activity
_01	65	0	1	1	0	0	GO:
0016853 Isomerase_activity
_01	217	1	1	1	0	0.000423	GO:
0016874 Ligase_activity
_01	25	0	1	1	0	0	GO:
0016881 Acid-amino_acid_ligase_activity
_01	81	0	1	1	0	0	GO:
0016887 ATPase_activity
_01	5	0	1	1	0	0	GO:
0016918 Retinal_binding
_01	6	0	1	1	0	0	GO:
0016922 Ligand-dependent_nuclear_receptor_binding
_01	5	0	1	1	0	0	GO:
0016925 Protein_sumoylation
_01	7	0	1	1	0	0	GO:
0016944 RNA_polymerase_II_transcription_elongation_factor_activity
_01	10	0	1	1	0	0	GO:
0016998 Cell_wall_macromolecule_catabolic_process
_01	12	0	1	1	0	0	GO:
0017015
Regulation_of_transforming_growth_factor_beta_receptor_signaling_pathway
_01	6	0	1	1	0	0	GO:
0017016 Ras_GTPase_binding
_01	8	0	1	1	0	0	GO:
0017017 MAP_kinase_tyrosine/serine/threonine_phosphatase_activity
_01	9	0	1	1	0	0	GO:
0017022 Myosin_binding
_01	6	0	1	1	0	0	GO:
0017025 TATA-binding_protein_binding
_01	19	0	1	1	0	0	GO:
0017046 Peptide_hormone_binding
_01	10	0	1	1	0	0	GO:
0017048 Rho_GTPase_binding
_01	7	0	1	1	0	0	GO:
0017049 GTP-Rho_binding
_01	22	0	1	1	0	0	GO:
0017053 Transcriptional_repressor_complex
_01	6	0	1	1	0	0	GO:
0017075 Syntaxin-1_binding
_01	5	0	1	1	0	0	GO:
0017076 Purine_nucleotide_binding
_01	6	0	1	1	0	0	GO:

```
0017091 AU-rich_element_binding
_01      5       0       1       1       0       0       GO:
0017110 Nucleoside-diphosphatase_activity
_01      37      0       1       1       0       0       GO:
0017111 Nucleoside-triphosphatase_activity
_01      7       0       1       1       0       0       GO:
0017119 Golgi_transport_complex
_01      74      0       1       1       0       0       GO:
0017124 SH3_domain_binding
_01      8       0       1       1       0       0       GO:
0017127 Cholesterol_transporter_activity
_01      10      0       1       1       0       0       GO:
0017134 Fibroblast_growth_factor_binding
_01      21      0       1       1       0       0       GO:
0017137 Rab_GTPase_binding
_01      6       0       1       1       0       0       GO:
0017144 Drug_metabolic_process
_01      5       0       1       1       0       0       GO:
0017146 N-methyl-D-aspartate_selective_glutamate_receptor_complex
_01      14      0       1       1       0       0       GO:
0017147 Wnt-protein_binding
_01      13      0       1       1       0       0       GO:
0017148 Negative_regulation_of_translation
_01      7       0       1       1       0       0       GO:
0017153 Sodium:dicarboxylate_symporter_activity
_01      6       0       1       1       0       0       GO:
0017154 Semaphorin_receptor_activity
_01      6       0       1       1       0       0       GO:
0017156 Calcium_ion-dependent_exocytosis
_01      9       0       1       1       0       0       GO:
0017157 Regulation_of_exocytosis
_01      8       1       1       1       0       5e-05   GO:
0017158 Regulation_of_calcium_ion-dependent_exocytosis
_01      8       0       1       1       0       0       GO:
0017166 Vinculin_binding
_01      13      0       1       1       0       0       GO:
0018024 Histone-lysine_N-methyltransferase_activity
_01      21      0       1       1       0       0       GO:
0018105 Peptidyl-serine_phosphorylation
_01      12      0       1       1       0       0       GO:
0018107 Peptidyl-threonine_phosphorylation
_01      21      0       1       1       0       0       GO:
0018108 Peptidyl-tyrosine_phosphorylation
_01      9       0       1       1       0       0       GO:
0018149 Peptide_cross-linking
_01      32      0       1       1       0       0       GO:
0018279 Protein_N-linked_glycosylation_via_asparagine
_01      13      0       1       1       0       0       GO:
0018298 Protein-chromophore_linkage
_01      16      0       1       1       0       0       GO:
0019001 Guanyl_nucleotide_binding
_01      16      0       1       1       0       0       GO:
0019003 GDP_binding
_01      13      0       1       1       0       0       GO:
```

0019005 SCF_ubiquitin_ligase_complex
_01 5 0 1 1 0 0 GO:
0019047 Provirus_integration
_01 6 0 1 1 0 0 GO:
0019048 Virus-host_interaction
_01 62 0 1 1 0 0 GO:
0019058 Viral_infectious_cycle
_01 7 0 1 1 0 0 GO:
0019059 Initiation_of_viral_infection
_01 6 0 1 1 0 0 GO:
0019079 Viral_genome_replication
_01 59 0 1 1 0 0 GO:
0019083 Viral_transcription
_01 5 0 1 1 0 0 GO:
0019104 DNA_N-glycosylase_activity
_01 9 1 1 1 0 0.000339 GO:
0019216 Regulation_of_lipid_metabolic_process
_01 35 0 1 1 0 0 GO:
0019221 Cytokine-mediated_signaling_pathway
_01 8 0 1 1 0 0 GO:
0019228 Regulation_of_action_potential_in_neuron
_01 10 0 1 1 0 0 GO:
0019229 Regulation_of_vasoconstriction
_01 23 0 1 1 0 0 GO:
0019233 Sensory_perception_of_pain
_01 4 0 1 1 0 0 GO:
0019236 Response_to_pheromone
_01 5 0 1 1 0 0 GO:
0019237 Centromeric_DNA_binding
_01 8 0 1 1 0 0 GO:
0019363 Pyridine_nucleotide_biosynthetic_process
_01 17 0 1 1 0 0 GO:
0019370 Leukotriene_biosynthetic_process
_01 7 0 1 1 0 0 GO:
0019430 Removal_of_superoxide_radicals
_01 20 0 1 1 0 0 GO:
0019432 Triglyceride_biosynthetic_process
_01 13 0 1 1 0 0 GO:
0019433 Triglyceride_catabolic_process
_01 5 0 1 1 0 0 GO:
0019439 Aromatic_compound_catabolic_process
_01 6 0 1 1 0 0 GO:
0019538 Protein_metabolic_process
_01 5 0 1 1 0 0 GO:
0019674 NAD_metabolic_process
_01 64 1 1 1 0 5e-05 GO:
0019717 Synaptosome
_01 25 0 1 1 0 0 GO:
0019722 Calcium-mediated_signaling
_01 6 0 1 1 0 0 GO:
0019725 Cellular_homeostasis
_01 6 0 1 1 0 0 GO:
0019752 Carboxylic_acid_metabolic_process
_01 5 0 1 1 0 0 GO:

0019800 Peptide_cross-linking_via_chondroitin_4-
sulfate_glycosaminoglycan
_01 22 0 1 1 0 0 GO:
0019825 Oxygen_binding
_01 10 0 1 1 0 0 GO:
0019827 Stem_cell_maintenance
_01 15 0 1 1 0 0 GO:
0019835 Cytolysis
_01 18 0 1 1 0 0 GO:
0019838 Growth_factor_binding
_01 6 0 1 1 0 0 GO:
0019841 Retinol_binding
_01 16 1 1 1 0 0.000423 GO:
0019843 RRNA_binding
_01 12 0 1 1 0 0 GO:
0019861 Flagellum
_01 8 0 1 1 0 0 GO:
0019882 Antigen_processing_and_presentation
_01 9 0 1 1 0 0 GO:
0019894 Kinesin_binding
_01 6 0 1 1 0 0 GO:
0019897 Extrinsic_to_plasma_membrane
_01 18 0 1 1 0 0 GO:
0019898 Extrinsic_to_membrane
_01 103 1 1 1 0 3.9e-05 GO:
0019899 Enzyme_binding
_01 20 0 1 1 0 0 GO:
0019900 Kinase_binding
_01 86 0 1 1 0 0 GO:
0019901 Protein_kinase_binding
_01 20 0 1 1 0 0 GO:
0019903 Protein_phosphatase_binding
_01 80 1 1 1 0 6.3e-05 GO:
0019904 Protein_domain_specific_binding
_01 9 0 1 1 0 0 GO:
0019905 Syntaxin_binding
_01 10 0 1 1 0 0 GO:
0019915 Lipid_storage
_01 7 0 1 1 0 0 GO:
0019933 CAMP-mediated_signaling
_01 10 0 1 1 0 0 GO:
0019955 Cytokine_binding
_01 9 0 1 1 0 0 GO:
0019992 Diacylglycerol_binding
_01 76 1 1 1 0 3.9e-05 GO:
0020037 Heme_binding
_01 6 0 1 1 0 0 GO:
0021510 Spinal_cord_development
_01 6 0 1 1 0 0 GO:
0021527 Spinal_cord_association_neuron_differentiation
_01 8 0 1 1 0 0 GO:
0021537 Telencephalon_development
_01 7 0 1 1 0 0 GO:
0021549 Cerebellum_development

_01	5	0	1	1	0	0	GO:0021675 Nerve_development
_01	7	0	1	1	0	0	GO:0021756 Striatum_development
_01	14	0	1	1	0	0	GO:0021766 Hippocampus_development
_01	5	0	1	1	0	0	GO:0021772 Olfactory_bulb_development
_01	10	1	1	1	0	0.000339	GO:0021904 Dorsal/ventral_neural_tube_patterning
_01	15	0	1	1	0	0	GO:0021915 Neural_tube_development
_01	5	0	1	1	0	0	GO:0021953 Central_nervous_system_neuron_differentiation
_01	6	0	1	1	0	0	GO:0021954 Central_nervous_system_neuron_development
_01	11	0	1	1	0	0	GO:0021983 Pituitary_gland_development
_01	20	0	1	1	0	0	GO:0021987 Cerebral_cortex_development
_01	15	0	1	1	0	0	GO:0022008 Neurogenesis
_01	24	0	1	1	0	0	GO:0022625 Cytosolic_large_ribosomal_subunit
_01	27	0	1	1	0	0	GO:0022627 Cytosolic_small_ribosomal_subunit
_01	11	0	1	1	0	0	GO:0022857 Transmembrane_transporter_activity
_01	9	0	1	1	0	0	GO:0022891 Substrate-specific_transmembrane_transporter_activity
_01	31	0	1	1	0	0	GO:0022900 Electron_transport_chain
_01	48	0	1	1	0	0	GO:0022904 Respiratory_electron_transport_chain
_01	202	0	1	1	0	0	GO:0023034 Intracellular_signaling_pathway
_01	7	0	1	1	0	0	GO:0030010 Establishment_of_cell_polarity
_01	13	0	1	1	0	0	GO:0030016 Myofibril
_01	22	0	1	1	0	0	GO:0030017 Sarcomere
_01	36	0	1	1	0	0	GO:0030018 Z_disc
_01	50	0	1	1	0	0	GO:0030027 Lamellipodium
_01	25	0	1	1	0	0	GO:0030030 Cell_projection_organization
_01	5	0	1	1	0	0	GO:0030031 Cell_projection_assembly
_01	11	0	1	1	0	0	GO:0030032 Lamellipodium_assembly
_01	80	2	0.00546999	0.382459	0	5.95e-05	GO:0030036 Actin_cytoskeleton_organization

_01 7 0 1 1 0 0 GO:
0030041 Actin_filament_polymerization
_01 8 0 1 1 0 0 GO:
0030048 Actin_filament-based_movement
_01 31 0 1 1 0 0 GO:
0030049 Muscle_filament_sliding
_01 299 2 0.0549819 0.710953 0 4.55e-05
GO:0030054 Cell_junction
_01 17 0 1 1 0 0 GO:
0030057 Desmosome
_01 7 0 1 1 0 0 GO:
0030071 Regulation_of_mitotic_metaphase/anaphase_transition
_01 18 0 1 1 0 0 GO:
0030073 Insulin_secretion
_01 39 2 0.000805999 0.146665 0
0.0005335 GO:0030097 Hemopoiesis
_01 10 0 1 1 0 0 GO:
0030099 Myeloid_cell_differentiation
_01 13 0 1 1 0 0 GO:
0030100 Regulation_of_endocytosis
_01 7 0 1 1 0 0 GO:
0030101 Natural_killer_cell_activation
_01 8 0 1 1 0 0 GO:
0030111 Regulation_of_Wnt_receptor_signaling_pathway
_01 17 0 1 1 0 0 GO:
0030117 Membrane_coat
_01 5 0 1 1 0 0 GO:
0030121 AP-1_adaptor_complex
_01 6 0 1 1 0 0 GO:
0030122 AP-2_adaptor_complex
_01 7 0 1 1 0 0 GO:
0030126 COPI_vesicle_coat
_01 6 0 1 1 0 0 GO:
0030130 Clathrin_coat_of_trans-Golgi_network_vesicle
_01 15 0 1 1 0 0 GO:
0030131 Clathrin_adaptor_complex
_01 27 0 1 1 0 0 GO:
0030133 Transport_vesicle
_01 23 0 1 1 0 0 GO:
0030136 Clathrin-coated_vesicle
_01 19 0 1 1 0 0 GO:
0030139 Endocytic_vesicle
_01 7 0 1 1 0 0 GO:
0030140 Trans-Golgi_network_transport_vesicle
_01 41 0 1 1 0 0 GO:
0030141 Stored_secretory_granule
_01 26 0 1 1 0 0 GO:
0030145 Manganese_ion_binding
_01 6 0 1 1 0 0 GO:
0030146 Diuresis
_01 5 0 1 1 0 0 GO:
0030147 Natriuresis
_01 6 0 1 1 0 0 GO:
0030148 Sphingolipid_biosynthetic_process

_01 319 2 0.030068 0.638468 0 6.65e-05 GO:
0030154 Cell_differentiation
_01 16 0 1 1 0 0 GO:
0030155 Regulation_of_cell_adhesion
_01 9 0 1 1 0 0 GO:
0030159 Receptor_signaling_complex_scaffold_activity
_01 7 0 1 1 0 0 GO:
0030162 Regulation_of_proteolysis
_01 21 0 1 1 0 0 GO:
0030163 Protein_catabolic_process
_01 34 0 1 1 0 0 GO:
0030165 PDZ_domain_binding
_01 8 0 1 1 0 0 GO:
0030166 Proteoglycan_biosynthetic_process
_01 157 3 0.000547999 0.119056 0
0.000270667 GO:0030168 Platelet_activation
_01 10 0 1 1 0 0 GO:
0030169 Low-density_lipoprotein_binding
_01 32 0 1 1 0 0 GO:
0030170 Pyridoxal_phosphate_binding
_01 33 0 1 1 0 0 GO:
0030173 Integral_to_Golgi_membrane
_01 17 0 1 1 0 0 GO:
0030175 Filopodium
_01 37 0 1 1 0 0 GO:
0030176 Integral_to_endoplasmic_reticulum_membrane
_01 7 0 1 1 0 0 GO:
0030177 Positive_regulation_of_Wnt_receptor_signaling_pathway
_01 24 1 1 1 0 0.000339 GO:
0030178 Negative_regulation_of_Wnt_receptor_signaling_pathway
_01 46 0 1 1 0 0 GO:
0030182 Neuron_differentiation
_01 19 0 1 1 0 0 GO:
0030183 B_cell_differentiation
_01 6 0 1 1 0 0 GO:
0030193 Regulation_of_blood_coagulation
_01 7 0 1 1 0 0 GO:
0030195 Negative_regulation_of_blood_coagulation
_01 41 0 1 1 0 0 GO:
0030198 Extracellular_matrix_organization
_01 18 0 1 1 0 0 GO:
0030199 Collagen_fibril_organization
_01 6 0 1 1 0 0 GO:
0030206 Chondroitin_sulfate_biosynthetic_process
_01 24 0 1 1 0 0 GO:
0030216 Keratinocyte_differentiation
_01 11 0 1 1 0 0 GO:
0030217 T_cell_differentiation
_01 13 0 1 1 0 0 GO:
0030218 Erythrocyte_differentiation
_01 7 0 1 1 0 0 GO:
0030225 Macrophage_differentiation
_01 8 0 1 1 0 0 GO:
0030234 Enzyme_regulator_activity

_01 6 0 1 1 0 0 GO:
0030235 Nitric-oxide_synthase_regulator_activity
_01 18 0 1 1 0 0 GO:
0030246 Carbohydrate_binding
_01 8 0 1 1 0 0 GO:
0030247 Polysaccharide_binding
_01 5 0 1 1 0 0 GO:
0030252 Growth_hormone_secretion
_01 8 0 1 1 0 0 GO:
0030261 Chromosome_condensation
_01 5 0 1 1 0 0 GO:
0030274 LIM_domain_binding
_01 7 0 1 1 0 0 GO:
0030276 Clathrin_binding
_01 5 0 1 1 0 0 GO:
0030278 Regulation_of_ossification
_01 10 0 1 1 0 0 GO:
0030279 Negative_regulation_of_ossification
_01 6 0 1 1 0 0 GO:
0030280 Structural_constituent_of_epidermis
_01 15 1 1 1 0 3.9e-05 GO:
0030282 Bone_mineralization
_01 10 0 1 1 0 0 GO:
0030286 Dynein_complex
_01 7 0 1 1 0 0 GO:
0030288 Outer_membrane-bounded_periplasmic_space
_01 5 0 1 1 0 0 GO:
0030295 Protein_kinase_activator_activity
_01 8 0 1 1 0 0 GO:
0030301 Cholesterol_transport
_01 28 0 1 1 0 0 GO:
0030307 Positive_regulation_of_cell_growth
_01 66 0 1 1 0 0 GO:
0030308 Negative_regulation_of_cell_growth
_01 14 0 1 1 0 0 GO:
0030315 T-tubule
_01 8 0 1 1 0 0 GO:
0030316 Osteoclast_differentiation
_01 10 0 1 1 0 0 GO:
0030317 Sperm_motility
_01 7 0 1 1 0 0 GO:
0030318 Melanocyte_differentiation
_01 47 1 1 1 0 6.3e-05 GO:
0030324 Lung_development
_01 13 0 1 1 0 0 GO:
0030325 Adrenal_gland_development
_01 27 0 1 1 0 0 GO:
0030326 Embryonic_limb_morphogenesis
_01 6 0 1 1 0 0 GO:
0030330
DNA_damage_response,_signal_transduction_by_p53_class_mediator
_01 10 0 1 1 0 0 GO:
0030331 Estrogen_receptor_binding
_01 7 0 1 1 0 0 GO:

0030332 Cyclin_binding
_01	22	0	1	1	0	0	GO:
0030334 Regulation_of_cell_migration
_01	52	0	1	1	0	0	GO:
0030335 Positive_regulation_of_cell_migration
_01	33	0	1	1	0	0	GO:
0030336 Negative_regulation_of_cell_migration
_01	20	0	1	1	0	0	GO:
0030374 Ligand-
dependent_nuclear_receptor_transcription_coactivator_activity
_01	8	0	1	1	0	0	GO:
0030384 Phosphoinositide_metabolic_process
_01	56	1	1	1	0	0.000339 GO:
0030414 Peptidase_inhibitor_activity
_01	89	0	1	1	0	0	GO:
0030424 Axon
_01	91	0	1	1	0	0	GO:
0030425 Dendrite
_01	45	0	1	1	0	0	GO:
0030426 Growth_cone
_01	9	0	1	1	0	0	GO:
0030431 Sleep
_01	6	0	1	1	0	0	GO:
0030432 Peristalsis
_01	19	0	1	1	0	0	GO:
0030433 ER-associated_protein_catabolic_process
_01	26	0	1	1	0	0	GO:
0030496 Midbody
_01	5	0	1	1	0	0	GO:
0030500 Regulation_of_bone_mineralization
_01	14	0	1	1	0	0	GO:
0030501 Positive_regulation_of_bone_mineralization
_01	6	0	1	1	0	0	GO:
0030506 Ankyrin_binding
_01	7	1	1	1	0	6.9e-05	GO:
0030507 Spectrin_binding
_01	28	0	1	1	0	0	GO:
0030509 BMP_signaling_pathway
_01	10	0	1	1	0	0	GO:
0030511
Positive_regulation_of_transforming_growth_factor_beta_receptor_sign
aling_pathway
_01	20	0	1	1	0	0	GO:
0030512
Negative_regulation_of_transforming_growth_factor_beta_receptor_sign
aling_pathway
_01	13	0	1	1	0	0	GO:
0030514 Negative_regulation_of_BMP_signaling_pathway
_01	5	0	1	1	0	0	GO:
0030515 SnoRNA_binding
_01	6	0	1	1	0	0	GO:
0030518 Steroid_hormone_receptor_signaling_pathway
_01	12	0	1	1	0	0	GO:
0030520 Estrogen_receptor_signaling_pathway

_01	22	0	1	1	0	0	GO:	0030521	Androgen_receptor_signaling_pathway
_01	192	2	0.004723	0.359966	0	0.000204	GO:	0030528	Transcription_regulator_activity
_01	71	1	1	1	0	5.2e-05	GO:	0030529	Ribonucleoprotein_complex
_01	11	0	1	1	0	0	GO:	0030530	Heterogeneous_nuclear_ribonucleoprotein_complex
_01	11	0	1	1	0	0	GO:	0030532	Small_nuclear_ribonucleoprotein_complex
_01	13	0	1	1	0	0	GO:	0030539	Male_genitalia_development
_01	7	0	1	1	0	0	GO:	0030552	CAMP_binding
_01	19	1	1	1	0	8.3e-05	GO:	0030574	Collagen_catabolic_process
_01	14	0	1	1	0	0	GO:	0030593	Neutrophil_chemotaxis
_01	6	0	1	1	0	0	GO:	0030595	Leukocyte_chemotaxis
_01	7	0	1	1	0	0	GO:	0030658	Transport_vesicle_membrane
_01	47	1	1	1	0	5e-05	GO:	0030659	Cytoplasmic_vesicle_membrane
_01	5	0	1	1	0	0	GO:	0030660	Golgi-associated_vesicle_membrane
_01	9	0	1	1	0	0	GO:	0030665	Clathrin_coated_vesicle_membrane
_01	17	0	1	1	0	0	GO:	0030666	Endocytic_vesicle_membrane
_01	8	0	1	1	0	0	GO:	0030667	Secretory_granule_membrane
_01	32	1	1	1	0	5e-05	GO:	0030672	Synaptic_vesicle_membrane
_01	5	0	1	1	0	0	GO:	0030673	Axolemma
_01	38	0	1	1	0	0	GO:	0030674	Protein_binding,_bridging
_01	6	0	1	1	0	0	GO:	0030675	Rac_GTPase_activator_activity
_01	5	1	1	1	0	3.9e-05	GO:	0030728	Ovulation
_01	9	0	1	1	0	0	GO:	0030742	GTP-dependent_protein_binding
_01	5	0	1	1	0	0	GO:	0030818	Negative_regulation_of_cAMP_biosynthetic_process
_01	21	0	1	1	0	0	GO:	0030819	Positive_regulation_of_cAMP_biosynthetic_process
_01	5	0	1	1	0	0	GO:	0030828	Positive_regulation_of_cGMP_biosynthetic_process
_01	5	0	1	1	0	0	GO:	0030833	Regulation_of_actin_filament_polymerization
_01	8	0	1	1	0	0	GO:	0030838	Positive_regulation_of_actin_filament_polymerization

_01 30 0 1 1 0 0 GO:
0030855 Epithelial_cell_differentiation
_01 5 0 1 1 0 0 GO:
0030856 Regulation_of_epithelial_cell_differentiation
_01 13 0 1 1 0 0 GO:
0030863 Cortical_cytoskeleton
_01 6 0 1 1 0 0 GO:
0030864 Cortical_actin_cytoskeleton
_01 11 0 1 1 0 0 GO:
0030866 Cortical_actin_cytoskeleton_organization
_01 5 0 1 1 0 0 GO:
0030867 Rough_endoplasmic_reticulum_membrane
_01 4 0 1 1 0 0 GO:
0030868 Smooth_endoplasmic_reticulum_membrane
_01 11 0 1 1 0 0 GO:
0030878 Thyroid_gland_development
_01 21 1 1 1 0 5.2e-05 GO:
0030879 Mammary_gland_development
_01 6 0 1 1 0 0 GO:
0030889 Negative_regulation_of_B_cell_proliferation
_01 21 0 1 1 0 0 GO:
0030890 Positive_regulation_of_B_cell_proliferation
_01 8 0 1 1 0 0 GO:
0030897 HOPS_complex
_01 7 0 1 1 0 0 GO:
0030898 Actin-dependent_ATPase_activity
_01 36 1 1 1 0 0.000339 GO:
0030900 Forebrain_development
_01 16 0 1 1 0 0 GO:
0030901 Midbrain_development
_01 12 0 1 1 0 0 GO:
0030902 Hindbrain_development
_01 9 0 1 1 0 0 GO:
0030914 STAGA_complex
_01 10 0 1 1 0 0 GO:
0030949
Positive_regulation_of_vascular_endothelial_growth_factor_receptor_s
ignaling_pathway
_01 16 0 1 1 0 0 GO:
0030968 Endoplasmic_reticulum_unfolded_protein_response
_01 5 0 1 1 0 0 GO:
0030970 Retrograde_protein_transport,_ER_to_cytosol
_01 7 1 1 1 0 5.2e-05 GO:
0030971 Receptor_tyrosine_kinase_binding
_01 5 0 1 1 0 0 GO:
0030983 Mismatched_DNA_binding
_01 6 0 1 1 0 0 GO:
0031000 Response_to_caffeine
_01 83 0 1 1 0 0 GO:
0031012 Extracellular_matrix
_01 5 0 1 1 0 0 GO:
0031013 Troponin_I_binding
_01 14 0 1 1 0 0 GO:
0031016 Pancreas_development

_01 82 0 1 1 0 0 GO:
0031018 Endocrine_pancreas_development
_01 22 0 1 1 0 0 GO:
0031047 Gene_silencing_by_RNA
_01 5 0 1 1 0 0 GO:
0031054 Pre-microRNA_processing
_01 12 0 1 1 0 0 GO:
0031069 Hair_follicle_morphogenesis
_01 39 0 1 1 0 0 GO:
0031072 Heat_shock_protein_binding
_01 6 0 1 1 0 0 GO:
0031080 Nup107-160_complex
_01 6 0 1 1 0 0 GO:
0031091 Platelet_alpha_granule
_01 8 0 1 1 0 0 GO:
0031092 Platelet_alpha_granule_membrane
_01 26 2 8.49999e-05 0.034596 0
0.0001945 GO:0031093 Platelet_alpha_granule_lumen
_01 7 0 1 1 0 0 GO:
0031095 Platelet_dense_tubular_network_membrane
_01 31 0 1 1 0 0 GO:
0031100 Organ_regeneration
_01 6 0 1 1 0 0 GO:
0031103 Axon_regeneration
_01 6 0 1 1 0 0 GO:
0031105 Septin_complex
_01 6 0 1 1 0 0 GO:
0031116 Positive_regulation_of_microtubule_polymerization
_01 29 0 1 1 0 0 GO:
0031124 MRNA_3'-end_processing
_01 8 0 1 1 0 0 GO:
0031143 Pseudopodium
_01 56 1 1 1 0 0.000423 GO:
0031145 Anaphase-promoting_complex-dependent_proteasomal_ubiquitin-
dependent_protein_catabolic_process
_01 9 0 1 1 0 0 GO:
0031146 SCF-dependent_proteasomal_ubiquitin-
dependent_protein_catabolic_process
_01 33 0 1 1 0 0 GO:
0031175 Neuron_projection_development
_01 12 0 1 1 0 0 GO:
0031201 SNARE_complex
_01 12 0 1 1 0 0 GO:
0031214 Biomineral_tissue_development
_01 8 0 1 1 0 0 GO:
0031224 Intrinsic_to_membrane
_01 68 0 1 1 0 0 GO:
0031225 Anchored_to_membrane
_01 10 0 1 1 0 0 GO:
0031227 Intrinsic_to_endoplasmic_reticulum_membrane
_01 5 0 1 1 0 0 GO:
0031234 Extrinsic_to_internal_side_of_plasma_membrane
_01 11 0 1 1 0 0 GO:
0031252 Cell_leading_edge

_01 6 0 1 1 0 0 GO:
0031258 Lamellipodium_membrane
_01 6 0 1 1 0 0 GO:
0031274 Positive_regulation_of_pseudopodium_assembly
_01 9 0 1 1 0 0 GO:
0031290 Retinal_ganglion_cell_axon_guidance
_01 7 0 1 1 0 0 GO:
0031293 Membrane_protein_intracellular_domain_proteolysis
_01 36 0 1 1 0 0 GO:
0031295 T_cell_costimulation
_01 5 0 1 1 0 0 GO:
0031307 Integral_to_mitochondrial_outer_membrane
_01 12 0 1 1 0 0 GO:
0031397 Negative_regulation_of_protein_ubiquitination
_01 17 0 1 1 0 0 GO:
0031398 Positive_regulation_of_protein_ubiquitination
_01 164 1 1 1 0 5e-05 GO:
0031410 Cytoplasmic_vesicle
_01 14 0 1 1 0 0 GO:
0031418 L-ascorbic_acid_binding
_01 7 0 1 1 0 0 GO:
0031419 Cobalamin_binding
_01 26 0 1 1 0 0 GO:
0031424 Keratinization
_01 6 0 1 1 0 0 GO:
0031430 M_band
_01 6 0 1 1 0 0 GO:
0031432 Titin_binding
_01 5 0 1 1 0 0 GO:
0031434 Mitogen-activated_protein_kinase_kinase_binding
_01 5 0 1 1 0 0 GO:
0031435 Mitogen-activated_protein_kinase_kinase_kinase_binding
_01 5 0 1 1 0 0 GO:
0031461 Cullin-RING_ubiquitin_ligase_complex
_01 7 0 1 1 0 0 GO:
0031463 Cul3-RING_ubiquitin_ligase_complex
_01 6 0 1 1 0 0 GO:
0031490 Chromatin_DNA_binding
_01 5 0 1 1 0 0 GO:
0031513 Nonmotile_primary_cilium
_01 5 0 1 1 0 0 GO:
0031514 Motile_cilium
_01 5 0 1 1 0 0 GO:
0031519 PcG_protein_complex
_01 22 0 1 1 0 0 GO:
0031526 Brush_border_membrane
_01 7 0 1 1 0 0 GO:
0031527 Filopodium_membrane
_01 8 0 1 1 0 0 GO:
0031528 Microvillus_membrane
_01 5 0 1 1 0 0 GO:
0031529 Ruffle_organization
_01 15 0 1 1 0 0 GO:
0031532 Actin_cytoskeleton_reorganization

_01 9 0 1 1 0 0 GO:
0031572 G2/M_transition_DNA_damage_checkpoint
_01 8 0 1 1 0 0 GO:
0031581 Hemidesmosome_assembly
_01 6 0 1 1 0 0 GO:
0031593 Polyubiquitin_binding
_01 19 0 1 1 0 0 GO:
0031594 Neuromuscular_junction
_01 8 1 1 1 0 3.9e-05 GO:
0031622 Positive_regulation_of_fever
_01 9 0 1 1 0 0 GO:
0031623 Receptor_internalization
_01 49 0 1 1 0 0 GO:
0031625 Ubiquitin_protein_ligase_binding
_01 10 0 1 1 0 0 GO:
0031647 Regulation_of_protein_stability
_01 10 0 1 1 0 0 GO:
0031648 Protein_destabilization
_01 5 0 1 1 0 0 GO:
0031659 Positive_regulation_of_cyclin-
dependent_protein_kinase_activity_involved_in_G1/S
_01 5 0 1 1 0 0 GO:
0031663 Lipopolysaccharide-mediated_signaling_pathway
_01 10 0 1 1 0 0 GO:
0031667 Response_to_nutrient_levels
_01 13 0 1 1 0 0 GO:
0031668 Cellular_response_to_extracellular_stimulus
_01 5 0 1 1 0 0 GO:
0031672 A_band
_01 7 1 1 1 0 5e-05 GO:
0031674 I_band
_01 24 0 1 1 0 0 GO:
0031901 Early_endosome_membrane
_01 36 0 1 1 0 0 GO:
0031902 Late_endosome_membrane
_01 5 0 1 1 0 0 GO:
0031929 TOR_signaling_cascade
_01 10 0 1 1 0 0 GO:
0031941 Filamentous_actin
_01 5 0 1 1 0 0 GO:
0031953 Negative_regulation_of_protein_autophosphorylation
_01 5 0 1 1 0 0 GO:
0031954 Positive_regulation_of_protein_autophosphorylation
_01 69 0 1 1 0 0 GO:
0031965 Nuclear_membrane
_01 23 0 1 1 0 0 GO:
0031966 Mitochondrial_membrane
_01 13 0 1 1 0 0 GO:
0031982 Vesicle
_01 6 0 1 1 0 0 GO:
0031988 Membrane-bounded_vesicle
_01 5 0 1 1 0 0 GO:
0032007 Negative_regulation_of_TOR_signaling_cascade
_01 9 0 1 1 0 0 GO:

0032008 Positive_regulation_of_TOR_signaling_cascade
_01 11 0 1 1 0 0 GO:
0032012 Regulation_of_ARF_protein_signal_transduction
_01 5 0 1 1 0 0 GO:
0032020 ISG15-protein_conjugation
_01 14 0 1 1 0 0 GO:
0032024 Positive_regulation_of_insulin_secretion
_01 8 0 1 1 0 0 GO:
0032026 Response_to_magnesium_ion
_01 9 0 1 1 0 0 GO:
0032039 Integrator_complex
_01 6 0 1 1 0 0 GO:
0032040 Small-subunit_processome
_01 23 0 1 1 0 0 GO:
0032088 Negative_regulation_of_NF-
kappaB_transcription_factor_activity
_01 7 0 1 1 0 0 GO:
0032091 Negative_regulation_of_protein_binding
_01 9 1 1 1 0 6.9e-05 GO:
0032092 Positive_regulation_of_protein_binding
_01 7 0 1 1 0 0 GO:
0032094 Response_to_food
_01 9 0 1 1 0 0 GO:
0032147 Activation_of_protein_kinase_activity
_01 7 0 1 1 0 0 GO:
0032148 Activation_of_protein_kinase_B_activity
_01 18 0 1 1 0 0 GO:
0032154 Cleavage_furrow
_01 13 0 1 1 0 0 GO:
0032201 Telomere_maintenance_via_semi-conservative_replication
_01 6 0 1 1 0 0 GO:
0032229 Negative_regulation_of_synaptic_transmission,_GABAergic
_01 5 0 1 1 0 0 GO:
0032230 Positive_regulation_of_synaptic_transmission,_GABAergic
_01 5 0 1 1 0 0 GO:
0032259 Methylation
_01 5 0 1 1 0 0 GO:
0032266 Phosphatidylinositol-3-phosphate_binding
_01 8 0 1 1 0 0 GO:
0032270 Positive_regulation_of_cellular_protein_metabolic_process
_01 8 0 1 1 0 0 GO:
0032279 Asymmetric_synapse
_01 7 0 1 1 0 0 GO:
0032308 Positive_regulation_of_prostaglandin_secretion
_01 21 0 1 1 0 0 GO:
0032312 Regulation_of_ARF_GTPase_activity
_01 30 0 1 1 0 0 GO:
0032313 Regulation_of_Rab_GTPase_activity
_01 7 0 1 1 0 0 GO:
0032331 Negative_regulation_of_chondrocyte_differentiation
_01 6 0 1 1 0 0 GO:
0032332 Positive_regulation_of_chondrocyte_differentiation
_01 48 3 3e-06 0.00182 0 0.000147 GO:
0032355 Response_to_estradiol_stimulus

_01 9 0 1 1 0 0 GO:
0032391 Photoreceptor_connecting_cilium
_01 68 0 1 1 0 0 GO:
0032403 Protein_complex_binding
_01 14 0 1 1 0 0 GO:
0032420 Stereocilium
_01 13 0 1 1 0 0 GO:
0032436 Positive_regulation_of_proteasomal_ubiquitin-
dependent_protein_catabolic_process
_01 6 0 1 1 0 0 GO:
0032438 Melanosome_organization
_01 67 2 0.000601999 0.124557 0 0.000189
GO:0032496 Response_to_lipopolysaccharide
_01 6 0 1 1 0 0 GO:
0032508 DNA_duplex_unwinding
_01 30 0 1 1 0 0 GO:
0032526 Response_to_retinoic_acid
_01 17 0 1 1 0 0 GO:
0032570 Response_to_progesterone_stimulus
_01 44 0 1 1 0 0 GO:
0032580 Golgi_cisterna_membrane
_01 27 0 1 1 0 0 GO:
0032582 Negative_regulation_of_gene-specific_transcription
_01 8 0 1 1 0 0 GO:
0032583 Regulation_of_gene-specific_transcription
_01 5 0 1 1 0 0 GO:
0032585 Multivesicular_body_membrane
_01 20 0 1 1 0 0 GO:
0032587 Ruffle_membrane
_01 6 0 1 1 0 0 GO:
0032689 Negative_regulation_of_interferon-gamma_production
_01 8 0 1 1 0 0 GO:
0032715 Negative_regulation_of_interleukin-6_production
_01 12 0 1 1 0 0 GO:
0032720 Negative_regulation_of_tumor_necrosis_factor_production
_01 9 0 1 1 0 0 GO:
0032722 Positive_regulation_of_chemokine_production
_01 9 0 1 1 0 0 GO:
0032728 Positive_regulation_of_interferon-beta_production
_01 6 0 1 1 0 0 GO:
0032729 Positive_regulation_of_interferon-gamma_production
_01 9 0 1 1 0 0 GO:
0032735 Positive_regulation_of_interleukin-12_production
_01 16 0 1 1 0 0 GO:
0032755 Positive_regulation_of_interleukin-6_production
_01 6 0 1 1 0 0 GO:
0032757 Positive_regulation_of_interleukin-8_production
_01 9 0 1 1 0 0 GO:
0032760 Positive_regulation_of_tumor_necrosis_factor_production
_01 7 0 1 1 0 0 GO:
0032781 Positive_regulation_of_ATPase_activity
_01 6 0 1 1 0 0 GO:
0032839 Dendrite_cytoplasm
_01 5 0 1 1 0 0 GO:

0032862 Activation_of_Rho_GTPase_activity
_01 36 0 1 1 0 0 GO:
0032868 Response_to_insulin_stimulus
_01 31 0 1 1 0 0 GO:
0032869 Cellular_response_to_insulin_stimulus
_01 22 0 1 1 0 0 GO:
0032870 Cellular_response_to_hormone_stimulus
_01 8 0 1 1 0 0 GO:
0032874 Positive_regulation_of_stress-activated_MAPK_cascade
_01 14 0 1 1 0 0 GO:
0032880 Regulation_of_protein_localization
_01 5 0 1 1 0 0 GO:
0032925 Regulation_of_activin_receptor_signaling_pathway
_01 6 0 1 1 0 0 GO:
0032947 Protein_complex_scaffold
_01 11 0 1 1 0 0 GO:
0032956 Regulation_of_actin_cytoskeleton_organization
_01 9 0 1 1 0 0 GO:
0032967 Positive_regulation_of_collagen_biosynthetic_process
_01 6 0 1 1 0 0 GO:
0032981 Mitochondrial_respiratory_chain_complex_I_assembly
_01 13 0 1 1 0 0 GO:
0032982 Myosin_filament
_01 12 0 1 1 0 0 GO:
0033017 Sarcoplasmic_reticulum_membrane
_01 14 0 1 1 0 0 GO:
0033077 T_cell_differentiation_in_thymus
_01 11 0 1 1 0 0 GO:
0033116 ER-Golgi_intermediate_compartment_membrane
_01 7 0 1 1 0 0 GO:
0033137 Negative_regulation_of_peptidyl-serine_phosphorylation
_01 14 0 1 1 0 0 GO:
0033138 Positive_regulation_of_peptidyl-serine_phosphorylation
_01 6 0 1 1 0 0 GO:
0033151 V(D)J_recombination
_01 5 0 1 1 0 0 GO:
0033162 Melanosome_membrane
_01 5 0 1 1 0 0 GO:
0033169 Histone_H3-K9_demethylation
_01 6 0 1 1 0 0 GO:
0033180 Proton-transporting_V-type_ATPase,_V1_domain
_01 6 0 1 1 0 0 GO:
0033189 Response_to_vitamin_A
_01 13 0 1 1 0 0 GO:
0033198 Response_to_ATP
_01 8 0 1 1 0 0 GO:
0033209 Tumor_necrosis_factor-mediated_signaling_pathway
_01 5 0 1 1 0 0 GO:
0033235 Positive_regulation_of_protein_sumoylation
_01 9 0 1 1 0 0 GO:
0033276 Transcription_factor_TFTC_complex
_01 13 1 1 1 0 3.9e-05 GO:
0033280 Response_to_vitamin_D
_01 14 0 1 1 0 0 GO:

0033344 Cholesterol_efflux
_01	5	0	1	1	0	0	GO:
0033391 Chromatoid_body
_01	6	0	1	1	0	0	GO:
0033523 Histone_H2B_ubiquitination
_01	7	0	1	1	0	0	GO:
0033540 Fatty_acid_beta-oxidation_using_acyl-CoA_oxidase
_01	6	0	1	1	0	0	GO:
0033558 Protein_deacetylase_activity
_01	8	0	1	1	0	0	GO:
0033574 Response_to_testosterone_stimulus
_01	8	0	1	1	0	0	GO:
0033613 Activating_transcription_factor_binding
_01	6	0	1	1	0	0	GO:
0033700 Phospholipid_efflux
_01	5	0	1	1	0	0	GO:
0033993 Response_to_lipid
_01	6	0	1	1	0	0	GO:
0034045 Pre-autophagosomal_structure_membrane
_01	6	0	1	1	0	0	GO:
0034080 CenH3-containing_nucleosome_assembly_at_centromere
_01	37	2	0.000151 0.0549709	0	0.000189
GO:0034097 Response_to_cytokine_stimulus
_01	41	0	1	1	0	0	GO:
0034138 Toll-like_receptor_3_signaling_pathway
_01	6	0	1	1	0	0	GO:
0034185 Apolipoprotein_binding
_01	9	0	1	1	0	0	GO:
0034199 Activation_of_protein_kinase_A_activity
_01	5	0	1	1	0	0	GO:
0034237 Protein_kinase_A_regulatory_subunit_binding
_01	64	0	1	1	0	0	GO:
0034329 Cell_junction_assembly
_01	24	0	1	1	0	0	GO:
0034332 Adherens_junction_organization
_01	38	0	1	1	0	0	GO:
0034339
Regulation_of_transcription_from_RNA_polymerase_II_promoter_by_nucle
ar_hormone_receptor
_01	7	0	1	1	0	0	GO:
0034341 Response_to_interferon-gamma
_01	13	0	1	1	0	0	GO:
0034361 Very-low-density_lipoprotein_particle
_01	9	0	1	1	0	0	GO:
0034362 Low-density_lipoprotein_particle
_01	12	0	1	1	0	0	GO:
0034364 High-density_lipoprotein_particle
_01	6	0	1	1	0	0	GO:
0034374 Low-density_lipoprotein_particle_remodeling
_01	8	0	1	1	0	0	GO:
0034375 High-density_lipoprotein_particle_remodeling
_01	9	0	1	1	0	0	GO:
0034394 Protein_localization_at_cell_surface
_01	6	0	1	1	0	0	GO:

0034405 Response_to_fluid_shear_stress
_01 8 0 1 1 0 0 GO:
0034446 Substrate_adhesion-dependent_cell_spreading
_01 5 0 1 1 0 0 GO:
0034450 Ubiquitin-ubiquitin_ligase_activity
_01 8 0 1 1 0 0 GO:
0034453 Microtubule_anchoring
_01 5 0 1 1 0 0 GO:
0034464 BBSome
_01 5 0 1 1 0 0 GO:
0034501 Protein_localization_to_kinetochore
_01 8 0 1 1 0 0 GO:
0034504 Protein_localization_to_nucleus
_01 11 1 1 1 0 5.2e-05 GO:
0034605 Cellular_response_to_heat
_01 10 0 1 1 0 0 GO:
0034612 Response_to_tumor_necrosis_factor
_01 12 0 1 1 0 0 GO:
0034613 Cellular_protein_localization
_01 6 0 1 1 0 0 GO:
0034614 Cellular_response_to_reactive_oxygen_species
_01 7 0 1 1 0 0 GO:
0034644 Cellular_response_to_UV
_01 11 0 1 1 0 0 GO:
0034660 NcRNA_metabolic_process
_01 6 0 1 1 0 0 GO:
0034704 Calcium_channel_complex
_01 38 0 1 1 0 0 GO:
0034707 Chloride_channel_complex
_01 6 0 1 1 0 0 GO:
0034713 Type_I_transforming_growth_factor_beta_receptor_binding
_01 6 0 1 1 0 0 GO:
0034976 Response_to_endoplasmic_reticulum_stress
_01 5 0 1 1 0 0 GO:
0034993 SUN-KASH_complex
_01 8 0 1 1 0 0 GO:
0035019 Somatic_stem_cell_maintenance
_01 45 0 1 1 0 0 GO:
0035023 Regulation_of_Rho_protein_signal_transduction
_01 6 0 1 1 0 0 GO:
0035024 Negative_regulation_of_Rho_protein_signal_transduction
_01 5 0 1 1 0 0 GO:
0035025 Positive_regulation_of_Rho_protein_signal_transduction
_01 6 0 1 1 0 0 GO:
0035035 Histone_acetyltransferase_binding
_01 12 0 1 1 0 0 GO:
0035050 Embryonic_heart_tube_development
_01 14 0 1 1 0 0 GO:
0035064 Methylated_histone_residue_binding
_01 13 0 1 1 0 0 GO:
0035085 Cilium_axoneme
_01 37 0 1 1 0 0 GO:
0035091 Phosphoinositide_binding
_01 9 0 1 1 0 0 GO:

0035094 Response_to_nicotine
_01 12 1 1 1 0 0.000423 GO:
0035097 Histone_methyltransferase_complex
_01 10 0 1 1 0 0 GO:
0035115 Embryonic_forelimb_morphogenesis
_01 14 0 1 1 0 0 GO:
0035116 Embryonic_hindlimb_morphogenesis
_01 9 0 1 1 0 0 GO:
0035121 Tail_morphogenesis
_01 5 0 1 1 0 0 GO:
0035136 Forelimb_morphogenesis
_01 7 0 1 1 0 0 GO:
0035162 Embryonic_hemopoiesis
_01 11 0 1 1 0 0 GO:
0035176 Social_behavior
_01 5 0 1 1 0 0 GO:
0035235 Ionotropic_glutamate_receptor_signaling_pathway
_01 8 0 1 1 0 0 GO:
0035249 Synaptic_transmission,_glutamatergic
_01 7 0 1 1 0 0 GO:
0035253 Ciliary_rootlet
_01 15 0 1 1 0 0 GO:
0035264 Multicellular_organism_growth
_01 8 0 1 1 0 0 GO:
0035265 Organ_growth
_01 12 0 1 1 0 0 GO:
0035267 NuA4_histone_acetyltransferase_complex
_01 5 0 1 1 0 0 GO:
0035313 Wound_healing,_spreading_of_epidermal_cells
_01 8 0 1 1 0 0 GO:
0035329 Hippo_signaling_cascade
_01 11 0 1 1 0 0 GO:
0035338 Long-chain_fatty-acyl-CoA_biosynthetic_process
_01 22 0 1 1 0 0 GO:
0040007 Growth
_01 38 0 1 1 0 0 GO:
0040008 Regulation_of_growth
_01 15 0 1 1 0 0 GO:
0040014 Regulation_of_multicellular_organism_growth
_01 15 0 1 1 0 0 GO:
0040018 Positive_regulation_of_multicellular_organism_growth
_01 5 0 1 1 0 0 GO:
0040037
Negative_regulation_of_fibroblast_growth_factor_receptor_signaling_p
athway
_01 12 0 1 1 0 0 GO:
0042056 Chemoattractant_activity
_01 24 0 1 1 0 0 GO:
0042059
Negative_regulation_of_epidermal_growth_factor_receptor_signaling_pa
thway
_01 32 0 1 1 0 0 GO:
0042060 Wound_healing
_01 5 0 1 1 0 0 GO:

0042074 Cell_migration_involved_in_gastrulation
_01	7	0	1	1	0	0	GO:
0042088 T-helper_1_type_immune_response
_01	10	0	1	1	0	0	GO:
0042098 T_cell_proliferation
_01	10	0	1	1	0	0	GO:
0042100 B_cell_proliferation
_01	9	0	1	1	0	0	GO:
0042101 T_cell_receptor_complex
_01	20	0	1	1	0	0	GO:
0042102 Positive_regulation_of_T_cell_proliferation
_01	7	0	1	1	0	0	GO:
0042104 Positive_regulation_of_activated_T_cell_proliferation
_01	15	1	1	1	0	7.4e-05	GO:
0042110 T_cell_activation
_01	12	0	1	1	0	0	GO:
0042113 B_cell_activation
_01	5	0	1	1	0	0	GO:
0042116 Macrophage_activation
_01	53	1	1	1	0	8.3e-05	GO:
0042127 Regulation_of_cell_proliferation
_01	11	0	1	1	0	0	GO:
0042130 Negative_regulation_of_T_cell_proliferation
_01	5	0	1	1	0	0	GO:
0042135 Neurotransmitter_catabolic_process
_01	5	0	1	1	0	0	GO:
0042136 Neurotransmitter_biosynthetic_process
_01	8	0	1	1	0	0	GO:
0042147 Retrograde_transport,_endosome_to_Golgi
_01	5	0	1	1	0	0	GO:
0042153 RPTP-like_protein_binding
_01	19	0	1	1	0	0	GO:
0042157 Lipoprotein_metabolic_process
_01	5	0	1	1	0	0	GO:
0042162 Telomeric_DNA_binding
_01	5	0	1	1	0	0	GO:
0042166 Acetylcholine_binding
_01	16	0	1	1	0	0	GO:
0042169 SH2_domain_binding
_01	8	0	1	1	0	0	GO:
0042176 Regulation_of_protein_catabolic_process
_01	10	0	1	1	0	0	GO:
0042177 Negative_regulation_of_protein_catabolic_process
_01	13	0	1	1	0	0	GO:
0042220 Response_to_cocaine
_01	8	0	1	1	0	0	GO:
0042221 Response_to_chemical_stimulus
_01	14	0	1	1	0	0	GO:
0042246 Tissue_regeneration
_01	16	0	1	1	0	0	GO:
0042254 Ribosome_biogenesis
_01	7	0	1	1	0	0	GO:
0042274 Ribosomal_small_subunit_biogenesis
_01	20	0	1	1	0	0	GO:

0042277 Peptide_binding
_01 5 0 1 1 0 0 GO:
0042288 MHC_class_I_protein_binding
_01 9 0 1 1 0 0 GO:
0042301 Phosphate_binding
_01 6 0 1 1 0 0 GO:
0042310 Vasoconstriction
_01 8 0 1 1 0 0 GO:
0042311 Vasodilation
_01 6 0 1 1 0 0 GO:
0042312 Regulation_of_vasodilation
_01 6 0 1 1 0 0 GO:
0042325 Regulation_of_phosphorylation
_01 9 0 1 1 0 0 GO:
0042326 Negative_regulation_of_phosphorylation
_01 7 0 1 1 0 0 GO:
0042327 Positive_regulation_of_phosphorylation
_01 6 0 1 1 0 0 GO:
0042346 Positive_regulation_of_NF-kappaB_import_into_nucleus
_01 5 0 1 1 0 0 GO:
0042355 L-fucose_catabolic_process
_01 6 0 1 1 0 0 GO:
0042359 Vitamin_D_metabolic_process
_01 5 0 1 1 0 0 GO:
0042382 Paraspeckles
_01 40 0 1 1 0 0 GO:
0042383 Sarcolemma
_01 15 0 1 1 0 0 GO:
0042384 Cilium_assembly
_01 17 0 1 1 0 0 GO:
0042391 Regulation_of_membrane_potential
_01 27 0 1 1 0 0 GO:
0042393 Histone_binding
_01 7 0 1 1 0 0 GO:
0042417 Dopamine_metabolic_process
_01 10 1 1 1 0 6.3e-05 GO:
0042445 Hormone_metabolic_process
_01 37 1 1 1 0 3.9e-05 GO:
0042446 Hormone_biosynthetic_process
_01 10 0 1 1 0 0 GO:
0042462 Eye_photoreceptor_cell_development
_01 63 0 1 1 0 0 GO:
0042470 Melanosome
_01 34 0 1 1 0 0 GO:
0042472 Inner_ear_morphogenesis
_01 12 1 1 1 0 0.000339 GO:
0042474 Middle_ear_morphogenesis
_01 21 0 1 1 0 0 GO:
0042475 Odontogenesis_of_dentine-containing_tooth
_01 19 0 1 1 0 0 GO:
0042476 Odontogenesis
_01 9 0 1 1 0 0 GO:
0042491 Auditory_receptor_cell_differentiation
_01 173 1 1 1 0 3.9e-05 GO:

0042493 Response_to_drug
_01 9 0 1 1 0 0 GO:
0042517
Positive_regulation_of_tyrosine_phosphorylation_of_Stat3_protein
_01 9 0 1 1 0 0 GO:
0042523
Positive_regulation_of_tyrosine_phosphorylation_of_Stat5_protein
_01 27 0 1 1 0 0 GO:
0042542 Response_to_hydrogen_peroxide
_01 17 0 1 1 0 0 GO:
0042552 Myelination
_01 7 0 1 1 0 0 GO:
0042554 Superoxide_anion_generation
_01 11 0 1 1 0 0 GO:
0042562 Hormone_binding
_01 6 0 1 1 0 0 GO:
0042573 Retinoic_acid_metabolic_process
_01 5 0 1 1 0 0 GO:
0042581 Specific_granule
_01 5 0 1 1 0 0 GO:
0042589 Zymogen_granule_membrane
_01 10 0 1 1 0 0 GO:
0042592 Homeostatic_process
_01 34 1 1 1 0 6.3e-05 GO:
0042593 Glucose_homeostasis
_01 7 0 1 1 0 0 GO:
0042594 Response_to_starvation
_01 7 0 1 1 0 0 GO:
0042605 Peptide_antigen_binding
_01 5 0 1 1 0 0 GO:
0042612 MHC_class_I_protein_complex
_01 7 0 1 1 0 0 GO:
0042623 ATPase_activity,_coupled
_01 18 0 1 1 0 0 GO:
0042626
ATPase_activity,_coupled_to_transmembrane_movement_of_substances
_01 9 0 1 1 0 0 GO:
0042627 Chylomicron
_01 28 0 1 1 0 0 GO:
0042632 Cholesterol_homeostasis
_01 6 1 1 1 0 3.9e-05 GO:
0042640 Anagen
_01 24 0 1 1 0 0 GO:
0042645 Mitochondrial_nucleoid
_01 7 0 1 1 0 0 GO:
0042692 Muscle_cell_differentiation
_01 6 0 1 1 0 0 GO:
0042698 Ovulation_cycle
_01 7 0 1 1 0 0 GO:
0042711 Maternal_behavior
_01 5 0 1 1 0 0 GO:
0042713 Sperm_ejaculation
_01 9 0 1 1 0 0 GO:
0042730 Fibrinolysis

_01 24 0 1 1 0 0 GO:
0042733 Embryonic_digit_morphogenesis
_01 30 0 1 1 0 0 GO:
0042734 Presynaptic_membrane
_01 51 0 1 1 0 0 GO:
0042742 Defense_response_to_bacterium
_01 10 0 1 1 0 0 GO:
0042744 Hydrogen_peroxide_catabolic_process
_01 6 0 1 1 0 0 GO:
0042752 Regulation_of_circadian_rhythm
_01 19 0 1 1 0 0 GO:
0042755 Eating_behavior
_01 6 0 1 1 0 0 GO:
0042756 Drinking_behavior
_01 5 0 1 1 0 0 GO:
0042769 DNA_damage_response,_detection_of_DNA_damage
_01 12 0 1 1 0 0 GO:
0042771
DNA_damage_response,_signal_transduction_by_p53_class_mediator_resul
ting_in_induction_of_apoptosis
_01 9 0 1 1 0 0 GO:
0042776 Mitochondrial_ATP_synthesis_coupled_proton_transport
_01 11 0 1 1 0 0 GO:
0042787 Protein_ubiquitination_involved_in_ubiquitin-
dependent_protein_catabolic_process
_01 8 1 1 1 0 0.000423 GO:
0042800 Histone_methyltransferase_activity_(H3-K4_specific)
_01 216 1 1 1 0 5e-05 GO:
0042802 Identical_protein_binding
_01 244 1 1 1 0 6.9e-05 GO:
0042803 Protein_homodimerization_activity
_01 10 0 1 1 0 0 GO:
0042809 Vitamin_D_receptor_binding
_01 10 0 1 1 0 0 GO:
0042813 Wnt_receptor_activity
_01 28 0 1 1 0 0 GO:
0042826 Histone_deacetylase_binding
_01 5 0 1 1 0 0 GO:
0042832 Defense_response_to_protozoan
_01 5 0 1 1 0 0 GO:
0042921 Glucocorticoid_receptor_signaling_pathway
_01 5 0 1 1 0 0 GO:
0042923 Neuropeptide_binding
_01 8 0 1 1 0 0 GO:
0042953 Lipoprotein_transport
_01 116 0 1 1 0 0 GO:
0042981 Regulation_of_apoptosis
_01 5 0 1 1 0 0 GO:
0042994 Cytoplasmic_sequestering_of_transcription_factor
_01 70 0 1 1 0 0 GO:
0042995 Cell_projection
_01 43 0 1 1 0 0 GO:
0043005 Neuron_projection
_01 18 0 1 1 0 0 GO:

```
0043010 Camera-type_eye_development
_01      5       0       1       1       0       0       GO:
0043011 Myeloid_dendritic_cell_differentiation
_01      5       0       1       1       0       0       GO:
0043015 Gamma-tubulin_binding
_01      6       0       1       1       0       0       GO:
0043020 NADPH_oxidase_complex
_01      8       0       1       1       0       0       GO:
0043021 Ribonucleoprotein_binding
_01      12      0       1       1       0       0       GO:
0043022 Ribosome_binding
_01      96      0       1       1       0       0       GO:
0043025 Neuronal_cell_body
_01      6       0       1       1       0       0       GO:
0043027 Caspase_inhibitor_activity
_01      9       0       1       1       0       0       GO:
0043029 T_cell_homeostasis
_01      9       0       1       1       0       0       GO:
0043034 Costamere
_01      5       0       1       1       0       0       GO:
0043043 Peptide_biosynthetic_process
_01      7       0       1       1       0       0       GO:
0043046 DNA_methylation_involved_in_gamete_generation
_01      90      1       1       1       0       3.9e-05 GO:
0043065 Positive_regulation_of_apoptosis
_01      102     0       1       1       0       0       GO:
0043066 Negative_regulation_of_apoptosis
_01      5       0       1       1       0       0       GO:
0043069 Negative_regulation_of_programmed_cell_death
_01      9       0       1       1       0       0       GO:
0043085 Positive_regulation_of_catalytic_activity
_01      6       0       1       1       0       0       GO:
0043087 Regulation_of_GTPase_activity
_01      9       0       1       1       0       0       GO:
0043101 Purine-containing_compound_salvage
_01      6       0       1       1       0       0       GO:
0043113 Receptor_clustering
_01      80      0       1       1       0       0       GO:
0043123 Positive_regulation_of_I-kappaB_kinase/NF-kappaB_cascade
_01      6       0       1       1       0       0       GO:
0043124 Negative_regulation_of_I-kappaB_kinase/NF-kappaB_cascade
_01      5       0       1       1       0       0       GO:
0043129 Surfactant_homeostasis
_01      16      0       1       1       0       0       GO:
0043130 Ubiquitin_binding
_01      5       0       1       1       0       0       GO:
0043149 Stress_fiber_assembly
_01      13      0       1       1       0       0       GO:
0043154 Negative_regulation_of_caspase_activity
_01      30      0       1       1       0       0       GO:
0043161 Proteasomal_ubiquitin-dependent_protein_catabolic_process
_01      16      0       1       1       0       0       GO:
0043169 Cation_binding
_01      5       0       1       1       0       0       GO:
```

0043186 P_granule
_01 5 0 1 1 0 0 GO:
0043190 ATP-binding_cassette_(ABC)_transporter_complex
_01 43 0 1 1 0 0 GO:
0043193 Positive_regulation_of_gene-specific_transcription
_01 21 0 1 1 0 0 GO:
0043195 Terminal_button
_01 29 0 1 1 0 0 GO:
0043197 Dendritic_spine
_01 10 0 1 1 0 0 GO:
0043198 Dendritic_shaft
_01 15 0 1 1 0 0 GO:
0043200 Response_to_amino_acid_stimulus
_01 5 0 1 1 0 0 GO:
0043202 Lysosomal_lumen
_01 22 0 1 1 0 0 GO:
0043204 Perikaryon
_01 5 0 1 1 0 0 GO:
0043206 Fibril_organization
_01 6 0 1 1 0 0 GO:
0043208 Glycosphingolipid_binding
_01 6 0 1 1 0 0 GO:
0043209 Myelin_sheath
_01 130 0 1 1 0 0 GO:
0043231 Intracellular_membrane-bounded_organelle
_01 112 2 0.00452 0.352829 0 0.000189 GO:
0043234 Protein_complex
_01 12 0 1 1 0 0 GO:
0043235 Receptor_complex
_01 8 0 1 1 0 0 GO:
0043236 Laminin_binding
_01 7 0 1 1 0 0 GO:
0043252 Sodium-independent_organic_anion_transport
_01 5 0 1 1 0 0 GO:
0043277 Apoptotic_cell_clearance
_01 13 0 1 1 0 0 GO:
0043278 Response_to_morphine
_01 9 0 1 1 0 0 GO:
0043280 Positive_regulation_of_caspase_activity
_01 6 0 1 1 0 0 GO:
0043292 Contractile_fiber
_01 5 0 1 1 0 0 GO:
0043306 Positive_regulation_of_mast_cell_degranulation
_01 8 0 1 1 0 0 GO:
0043325 Phosphatidylinositol-3,4-bisphosphate_binding
_01 7 0 1 1 0 0 GO:
0043330 Response_to_exogenous_dsRNA
_01 9 0 1 1 0 0 GO:
0043388 Positive_regulation_of_DNA_binding
_01 8 0 1 1 0 0 GO:
0043392 Negative_regulation_of_DNA_binding
_01 6 0 1 1 0 0 GO:
0043393 Regulation_of_protein_binding
_01 7 0 1 1 0 0 GO:

0043395 Heparan_sulfate_proteoglycan_binding
_01	7	0	1	1	0	0	GO:
0043403 Skeletal_muscle_tissue_regeneration
_01	14	0	1	1	0	0	GO:
0043406 Positive_regulation_of_MAP_kinase_activity
_01	17	0	1	1	0	0	GO:
0043407 Negative_regulation_of_MAP_kinase_activity
_01	5	0	1	1	0	0	GO:
0043408 Regulation_of_MAPKKK_cascade
_01	8	0	1	1	0	0	GO:
0043409 Negative_regulation_of_MAPKKK_cascade
_01	31	0	1	1	0	0	GO:
0043410 Positive_regulation_of_MAPKKK_cascade
_01	26	0	1	1	0	0	GO:
0043433 Negative_regulation_of_transcription_factor_activity
_01	40	0	1	1	0	0	GO:
0043434 Response_to_peptide_hormone_stimulus
_01	13	0	1	1	0	0	GO:
0043473 Pigmentation
_01	9	0	1	1	0	0	GO:
0043484 Regulation_of_RNA_splicing
_01	6	0	1	1	0	0	GO:
0043488 Regulation_of_mRNA_stability
_01	8	0	1	1	0	0	GO:
0043491 Protein_kinase_B_signaling_cascade
_01	10	0	1	1	0	0	GO:
0043498 Cell_surface_binding
_01	11	0	1	1	0	0	GO:
0043499 Eukaryotic_cell_surface_binding
_01	13	0	1	1	0	0	GO:
0043507 Positive_regulation_of_JUN_kinase_activity
_01	5	0	1	1	0	0	GO:
0043508 Negative_regulation_of_JUN_kinase_activity
_01	5	0	1	1	0	0	GO:
0043518
Negative_regulation_of_DNA_damage_response,_signal_transduction_by_p
53_class_mediator
_01	38	0	1	1	0	0	GO:
0043524 Negative_regulation_of_neuron_apoptosis
_01	13	0	1	1	0	0	GO:
0043525 Positive_regulation_of_neuron_apoptosis
_01	12	0	1	1	0	0	GO:
0043526 Neuroprotection
_01	11	0	1	1	0	0	GO:
0043531 ADP_binding
_01	7	0	1	1	0	0	GO:
0043534 Blood_vessel_endothelial_cell_migration
_01	7	0	1	1	0	0	GO:
0043536
Positive_regulation_of_blood_vessel_endothelial_cell_migration
_01	5	0	1	1	0	0	GO:
0043537
Negative_regulation_of_blood_vessel_endothelial_cell_migration
_01	8	0	1	1	0	0	GO:

0043542 Endothelial_cell_migration
_01 7 0 1 1 0 0 GO:
0043548 Phosphoinositide_3-kinase_binding
_01 8 0 1 1 0 0 GO:
0043560 Insulin_receptor_substrate_binding
_01 321 3 0.001001 0.168941 0 0.000160667
GO:0043565 Sequence-specific_DNA_binding
_01 5 0 1 1 0 0 GO:
0043568 Positive_regulation_of_insulin-like_growth_factor_receptor_signaling_pathway
_01 5 1 1 1 0 0.000339 GO:
0043583 Ear_development
_01 5 0 1 1 0 0 GO:
0043586 Tongue_development
_01 5 0 1 1 0 0 GO:
0043587 Tongue_morphogenesis
_01 18 0 1 1 0 0 GO:
0043588 Skin_development
_01 10 0 1 1 0 0 GO:
0043616 Keratinocyte_proliferation
_01 8 0 1 1 0 0 GO:
0043621 Protein_self-association
_01 36 0 1 1 0 0 GO:
0043627 Response_to_estrogen_stimulus
_01 9 0 1 1 0 0 GO:
0043679 Axon_terminus
_01 74 0 1 1 0 0 GO:
0043687 Post-translational_protein_modification
_01 11 0 1 1 0 0 GO:
0043691 Reverse_cholesterol_transport
_01 18 0 1 1 0 0 GO:
0043928 Exonucleolytic_nuclear-transcribed_mRNA_catabolic_process_involved_in_deadenylation-dependent_decay
_01 24 0 1 1 0 0 GO:
0043966 Histone_H3_acetylation
_01 13 0 1 1 0 0 GO:
0043967 Histone_H4_acetylation
_01 10 0 1 1 0 0 GO:
0043968 Histone_H2A_acetylation
_01 5 0 1 1 0 0 GO:
0043983 Histone_H4-K12_acetylation
_01 8 0 1 1 0 0 GO:
0043984 Histone_H4-K16_acetylation
_01 9 0 1 1 0 0 GO:
0044130 Negative_regulation_of_growth_of_symbiont_in_host
_01 11 0 1 1 0 0 GO:
0044237 Cellular_metabolic_process
_01 76 0 1 1 0 0 GO:
0044255 Cellular_lipid_metabolic_process
_01 7 0 1 1 0 0 GO:
0044262 Cellular_carbohydrate_metabolic_process
_01 151 0 1 1 0 0 GO:
0044267 Cellular_protein_metabolic_process

_01 180 1 1 1 0 7.4e-05 GO:
0044419 Interspecies_interaction_between_organisms
_01 6 0 1 1 0 0 GO:
0044424 Intracellular_part
_01 6 0 1 1 0 0 GO:
0044428 Nuclear_part
_01 5 0 1 1 0 0 GO:
0044444 Cytoplasmic_part
_01 6 0 1 1 0 0 GO:
0045022 Early_endosome_to_late_endosome_transport
_01 15 0 1 1 0 0 GO:
0045028 Purinergic_nucleotide_receptor_activity,_G-protein_coupled
_01 5 0 1 1 0 0 GO:
0045055 Regulated_secretory_pathway
_01 6 0 1 1 0 0 GO:
0045059 Positive_thymic_T_cell_selection
_01 8 0 1 1 0 0 GO:
0045060 Negative_thymic_T_cell_selection
_01 7 0 1 1 0 0 GO:
0045078 Positive_regulation_of_interferon-gamma_biosynthetic_process
_01 6 0 1 1 0 0 GO:
0045084 Positive_regulation_of_interleukin-12_biosynthetic_process
_01 7 0 1 1 0 0 GO:
0045086 Positive_regulation_of_interleukin-2_biosynthetic_process
_01 125 0 1 1 0 0 GO:
0045087 Innate_immune_response
_01 9 0 1 1 0 0 GO:
0045089 Positive_regulation_of_innate_immune_response
_01 31 0 1 1 0 0 GO:
0045095 Keratin_filament
_01 6 0 1 1 0 0 GO:
0045109 Intermediate_filament_organization
_01 20 0 1 1 0 0 GO:
0045111 Intermediate_filament_cytoskeleton
_01 7 0 1 1 0 0 GO:
0045116 Protein_neddylation
_01 5 0 1 1 0 0 GO:
0045120 Pronucleus
_01 74 0 1 1 0 0 GO:
0045121 Membrane_raft
_01 19 0 1 1 0 0 GO:
0045165 Cell_fate_commitment
_01 5 0 1 1 0 0 GO:
0045176 Apical_protein_localization
_01 25 0 1 1 0 0 GO:
0045177 Apical_part_of_cell
_01 13 0 1 1 0 0 GO:
0045182 Translation_regulator_activity
_01 9 0 1 1 0 0 GO:
0045184 Establishment_of_protein_localization
_01 5 0 1 1 0 0 GO:
0045190 Isotype_switching
_01 180 2 0.037092 0.676482 0 4.55e-05 GO:
0045202 Synapse

_01	110	0	1	1	0	0	GO:
0045211 Postsynaptic_membrane
_01	8	0	1	1	0	0	GO:
0045214 Sarcomere_organization
_01	38	0	1	1	0	0	GO:
0045216 Cell-cell_junction_organization
_01	6	0	1	1	0	0	GO:
0045294 Alpha-catenin_binding
_01	8	0	1	1	0	0	GO:
0045295 Gamma-catenin_binding
_01	19	0	1	1	0	0	GO:
0045296 Cadherin_binding
_01	6	0	1	1	0	0	GO:
0045333 Cellular_respiration
_01	5	0	1	1	0	0	GO:
0045348 Positive_regulation_of_MHC_class_II_biosynthetic_process
_01	5	0	1	1	0	0	GO:
0045410 Positive_regulation_of_interleukin-6_biosynthetic_process
_01	15	1	1	1	0	3.9e-05	GO:
0045429 Positive_regulation_of_nitric_oxide_biosynthetic_process
_01	19	0	1	1	0	0	GO:
0045444 Fat_cell_differentiation
_01	11	0	1	1	0	0	GO:
0045445 Myoblast_differentiation
_01	6	0	1	1	0	0	GO:
0045446 Endothelial_cell_differentiation
_01	585	2	0.0647219	0.738657	0
0.0003835	GO:0045449 Regulation_of_transcription
_01	12	0	1	1	0	0	GO:
0045453 Bone_resorption
_01	47	0	1	1	0	0	GO:
0045454 Cell_redox_homeostasis
_01	42	0	1	1	0	0	GO:
0045471 Response_to_ethanol
_01	15	0	1	1	0	0	GO:
0045494 Photoreceptor_cell_maintenance
_01	5	0	1	1	0	0	GO:
0045499 Chemorepellent_activity
_01	5	0	1	1	0	0	GO:
0045588 Positive_regulation_of_gamma-delta_T_cell_differentiation
_01	10	0	1	1	0	0	GO:
0045595 Regulation_of_cell_differentiation
_01	13	0	1	1	0	0	GO:
0045596 Negative_regulation_of_cell_differentiation
_01	13	0	1	1	0	0	GO:
0045597 Positive_regulation_of_cell_differentiation
_01	10	0	1	1	0	0	GO:
0045599 Negative_regulation_of_fat_cell_differentiation
_01	9	0	1	1	0	0	GO:
0045600 Positive_regulation_of_fat_cell_differentiation
_01	5	0	1	1	0	0	GO:
0045647 Negative_regulation_of_erythrocyte_differentiation
_01	10	0	1	1	0	0	GO:
0045648 Positive_regulation_of_erythrocyte_differentiation

_01	5	0	1	1	0	0	GO:
0045651 Positive_regulation_of_macrophage_differentiation
_01	6	0	1	1	0	0	GO:
0045662 Negative_regulation_of_myoblast_differentiation
_01	5	0	1	1	0	0	GO:
0045663 Positive_regulation_of_myoblast_differentiation
_01	12	0	1	1	0	0	GO:
0045664 Regulation_of_neuron_differentiation
_01	18	0	1	1	0	0	GO:
0045665 Negative_regulation_of_neuron_differentiation
_01	22	1	1	1	0	6.3e-05	GO:
0045666 Positive_regulation_of_neuron_differentiation
_01	6	0	1	1	0	0	GO:
0045667 Regulation_of_osteoblast_differentiation
_01	9	0	1	1	0	0	GO:
0045668 Negative_regulation_of_osteoblast_differentiation
_01	21	0	1	1	0	0	GO:
0045669 Positive_regulation_of_osteoblast_differentiation
_01	10	0	1	1	0	0	GO:
0045671 Negative_regulation_of_osteoclast_differentiation
_01	6	0	1	1	0	0	GO:
0045672 Positive_regulation_of_osteoclast_differentiation
_01	6	0	1	1	0	0	GO:
0045723 Positive_regulation_of_fatty_acid_biosynthetic_process
_01	9	0	1	1	0	0	GO:
0045725 Positive_regulation_of_glycogen_biosynthetic_process
_01	18	0	1	1	0	0	GO:
0045727 Positive_regulation_of_translation
_01	5	0	1	1	0	0	GO:
0045730 Respiratory_burst
_01	11	0	1	1	0	0	GO:
0045732 Positive_regulation_of_protein_catabolic_process
_01	11	0	1	1	0	0	GO:
0045736 Negative_regulation_of_cyclin-
dependent_protein_kinase_activity
_01	10	0	1	1	0	0	GO:
0045739 Positive_regulation_of_DNA_repair
_01	25	0	1	1	0	0	GO:
0045740 Positive_regulation_of_DNA_replication
_01	5	0	1	1	0	0	GO:
0045745 Positive_regulation_of_G-
protein_coupled_receptor_protein_signaling_pathway
_01	5	0	1	1	0	0	GO:
0045746 Negative_regulation_of_Notch_signaling_pathway
_01	5	0	1	1	0	0	GO:
0045747 Positive_regulation_of_Notch_signaling_pathway
_01	7	0	1	1	0	0	GO:
0045749 Negative_regulation_of_S_phase_of_mitotic_cell_cycle
_01	18	0	1	1	0	0	GO:
0045765 Regulation_of_angiogenesis
_01	29	0	1	1	0	0	GO:
0045766 Positive_regulation_of_angiogenesis
_01	27	0	1	1	0	0	GO:
0045768 Positive_regulation_of_anti-apoptosis

_01 9 0 1 1 0 0 GO:
0045773 Positive_regulation_of_axon_extension
_01 14 0 1 1 0 0 GO:
0045776 Negative_regulation_of_blood_pressure
_01 8 0 1 1 0 0 GO:
0045777 Positive_regulation_of_blood_pressure
_01 7 0 1 1 0 0 GO:
0045778 Positive_regulation_of_ossification
_01 5 0 1 1 0 0 GO:
0045779 Negative_regulation_of_bone_resorption
_01 18 0 1 1 0 0 GO:
0045785 Positive_regulation_of_cell_adhesion
_01 28 0 1 1 0 0 GO:
0045786 Negative_regulation_of_cell_cycle
_01 11 0 1 1 0 0 GO:
0045787 Positive_regulation_of_cell_cycle
_01 7 0 1 1 0 0 GO:
0045792 Negative_regulation_of_cell_size
_01 6 0 1 1 0 0 GO:
0045807 Positive_regulation_of_endocytosis
_01 6 0 1 1 0 0 GO:
0045821 Positive_regulation_of_glycolysis
_01 6 0 1 1 0 0 GO:
0045822 Negative_regulation_of_heart_contraction
_01 12 0 1 1 0 0 GO:
0045840 Positive_regulation_of_mitosis
_01 7 0 1 1 0 0 GO:
0045859 Regulation_of_protein_kinase_activity
_01 13 0 1 1 0 0 GO:
0045860 Positive_regulation_of_protein_kinase_activity
_01 6 1 1 1 0 0.000339 GO:
0045861 Negative_regulation_of_proteolysis
_01 6 0 1 1 0 0 GO:
0045862 Positive_regulation_of_proteolysis
_01 5 0 1 1 0 0 GO:
0045879 Negative_regulation_of_smoothened_signaling_pathway
_01 7 0 1 1 0 0 GO:
0045880 Positive_regulation_of_smoothened_signaling_pathway
_01 12 0 1 1 0 0 GO:
0045885 Positive_regulation_of_survival_gene_product_expression
_01 61 0 1 1 0 0 GO:
0045892 Negative_regulation_of_transcription,_DNA-dependent
_01 89 0 1 1 0 0 GO:
0045893 Positive_regulation_of_transcription,_DNA-dependent
_01 11 1 1 1 0 3.9e-05 GO:
0045907 Positive_regulation_of_vasoconstriction
_01 11 0 1 1 0 0 GO:
0045909 Positive_regulation_of_vasodilation
_01 5 0 1 1 0 0 GO:
0045921 Positive_regulation_of_exocytosis
_01 8 0 1 1 0 0 GO:
0045930 Negative_regulation_of_mitotic_cell_cycle
_01 12 1 1 1 0 6.3e-05 GO:
0045931 Positive_regulation_of_mitotic_cell_cycle

_01 106 0 1 1 0 0 GO:
0045941 Positive_regulation_of_transcription
_01 228 1 1 1 0 6.3e-05 GO:
0045944
Positive_regulation_of_transcription_from_RNA_polymerase_II_promoter
_01 5 0 1 1 0 0 GO:
0045947 Negative_regulation_of_translational_initiation
_01 7 0 1 1 0 0 GO:
0045954
Positive_regulation_of_natural_killer_cell_mediated_cytotoxicity
_01 11 1 1 1 0 3.9e-05 GO:
0045987 Positive_regulation_of_smooth_muscle_contraction
_01 5 0 1 1 0 0 GO:
0045995 Regulation_of_embryonic_development
_01 7 0 1 1 0 0 GO:
0046034 ATP_metabolic_process
_01 8 0 1 1 0 0 GO:
0046135 Pyrimidine_nucleoside_catabolic_process
_01 6 0 1 1 0 0 GO:
0046209 Nitric_oxide_metabolic_process
_01 11 0 1 1 0 0 GO:
0046320 Regulation_of_fatty_acid_oxidation
_01 15 0 1 1 0 0 GO:
0046326 Positive_regulation_of_glucose_import
_01 8 0 1 1 0 0 GO:
0046328 Regulation_of_JNK_cascade
_01 9 0 1 1 0 0 GO:
0046329 Negative_regulation_of_JNK_cascade
_01 19 1 1 1 0 5e-05 GO:
0046330 Positive_regulation_of_JNK_cascade
_01 19 0 1 1 0 0 GO:
0046332 SMAD_binding
_01 6 0 1 1 0 0 GO:
0046415 Urate_metabolic_process
_01 7 0 1 1 0 0 GO:
0046427 Positive_regulation_of_JAK-STAT_cascade
_01 5 0 1 1 0 0 GO:
0046470 Phosphatidylcholine_metabolic_process
_01 8 0 1 1 0 0 GO:
0046488 Phosphatidylinositol_metabolic_process
_01 5 0 1 1 0 0 GO:
0046498 S-adenosylhomocysteine_metabolic_process
_01 7 0 1 1 0 0 GO:
0046513 Ceramide_biosynthetic_process
_01 9 0 1 1 0 0 GO:
0046579 Positive_regulation_of_Ras_protein_signal_transduction
_01 6 0 1 1 0 0 GO:
0046580 Negative_regulation_of_Ras_protein_signal_transduction
_01 5 0 1 1 0 0 GO:
0046620 Regulation_of_organ_growth
_01 12 0 1 1 0 0 GO:
0046627 Negative_regulation_of_insulin_receptor_signaling_pathway
_01 6 0 1 1 0 0 GO:
0046638 Positive_regulation_of_alpha-beta_T_cell_differentiation

_01	5	0	1	1	0	0	GO:
0046641 Positive_regulation_of_alpha-beta_T_cell_proliferation
_01	5	0	1	1	0	0	GO:
0046655 Folic_acid_metabolic_process
_01	14	0	1	1	0	0	GO:
0046658 Anchored_to_plasma_membrane
_01	9	0	1	1	0	0	GO:
0046676 Negative_regulation_of_insulin_secretion
_01	20	0	1	1	0	0	GO:
0046677 Response_to_antibiotic
_01	5	0	1	1	0	0	GO:
0046685 Response_to_arsenic
_01	9	0	1	1	0	0	GO:
0046686 Response_to_cadmium_ion
_01	10	0	1	1	0	0	GO:
0046688 Response_to_copper_ion
_01	8	1	1	1	0	3.9e-05	GO:
0046697 Decidualization
_01	8	1	1	1	0	5e-05	GO:
0046716 Muscle_cell_homeostasis
_01	52	0	1	1	0	0	GO:
0046777 Protein_autophosphorylation
_01	6	0	1	1	0	0	GO:
0046784 Intronless_viral_mRNA_export_from_host_nucleus
_01	15	0	1	1	0	0	GO:
0046847 Filopodium_assembly
_01	7	0	1	1	0	0	GO:
0046854 Phosphoinositide_phosphorylation
_01	10	0	1	1	0	0	GO:
0046873 Metal_ion_transmembrane_transporter_activity
_01	5	0	1	1	0	0	GO:
0046875 Ephrin_receptor_binding
_01	8	0	1	1	0	0	GO:
0046887 Positive_regulation_of_hormone_secretion
_01	7	0	1	1	0	0	GO:
0046888 Negative_regulation_of_hormone_secretion
_01	6	0	1	1	0	0	GO:
0046902 Regulation_of_mitochondrial_membrane_permeability
_01	13	0	1	1	0	0	GO:
0046907 Intracellular_transport
_01	5	0	1	1	0	0	GO:
0046914 Transition_metal_ion_binding
_01	10	0	1	1	0	0	GO:
0046928 Regulation_of_neurotransmitter_secretion
_01	6	0	1	1	0	0	GO:
0046930 Pore_complex
_01	10	0	1	1	0	0	GO:
0046933
Hydrogen_ion_transporting_ATP_synthase_activity,_rotational_mechanis
m
_01	13	0	1	1	0	0	GO:
0046961 Proton-transporting_ATPase_activity,_rotational_mechanism
_01	5	0	1	1	0	0	GO:
0046965 Retinoid_X_receptor_binding

_01 16 0 1 1 0 0 GO:
0046966 Thyroid_hormone_receptor_binding
_01 148 1 1 1 0 6.9e-05 GO:
0046982 Protein_heterodimerization_activity
_01 56 0 1 1 0 0 GO:
0046983 Protein_dimerization_activity
_01 5 0 1 1 0 0 GO:
0047372 Acylglycerol_lipase_activity
_01 48 0 1 1 0 0 GO:
0047485 Protein_N-terminus_binding
_01 5 0 1 1 0 0 GO:
0047496 Vesicle_transport_along_microtubule
_01 6 0 1 1 0 0 GO:
0047498 Calcium-dependent_phospholipase_A2_activity
_01 4 0 1 1 0 0 GO:
0047760 Butyrate-CoA_ligase_activity
_01 15 0 1 1 0 0 GO:
0048008 Platelet-derived_growth_factor_receptor_signaling_pathway
_01 8 0 1 1 0 0 GO:
0048009 Insulin-like_growth_factor_receptor_signaling_pathway
_01 12 0 1 1 0 0 GO:
0048010
Vascular_endothelial_growth_factor_receptor_signaling_pathway
_01 146 0 1 1 0 0 GO:
0048011 Nerve_growth_factor_receptor_signaling_pathway
_01 6 0 1 1 0 0 GO:
0048013 Ephrin_receptor_signaling_pathway
_01 45 0 1 1 0 0 GO:
0048015 Phosphoinositide-mediated_signaling
_01 12 0 1 1 0 0 GO:
0048037 Cofactor_binding
_01 6 0 1 1 0 0 GO:
0048038 Quinone_binding
_01 11 1 1 1 0 5e-05 GO:
0048041 Focal_adhesion_assembly
_01 5 0 1 1 0 0 GO:
0048066 Developmental_pigmentation
_01 29 0 1 1 0 0 GO:
0048146 Positive_regulation_of_fibroblast_proliferation
_01 5 0 1 1 0 0 GO:
0048147 Negative_regulation_of_fibroblast_proliferation
_01 5 0 1 1 0 0 GO:
0048148 Behavioral_response_to_cocaine
_01 13 0 1 1 0 0 GO:
0048167 Regulation_of_synaptic_plasticity
_01 12 0 1 1 0 0 GO:
0048168 Regulation_of_neuronal_synaptic_plasticity
_01 10 0 1 1 0 0 GO:
0048169 Regulation_of_long-term_neuronal_synaptic_plasticity
_01 8 0 1 1 0 0 GO:
0048185 Activin_binding
_01 6 1 1 1 0 0.000423 GO:
0048188 Set1C/COMPASS_complex
_01 5 0 1 1 0 0 GO:

0048193 Golgi_vesicle_transport
_01	5	0	1	1	0	0	GO:
0048205 COPI_coating_of_Golgi_vesicle
_01	5	0	1	1	0	0	GO:
0048208 COPII_vesicle_coating
_01	8	0	1	1	0	0	GO:
0048246 Macrophage_chemotaxis
_01	6	0	1	1	0	0	GO:
0048247 Lymphocyte_chemotaxis
_01	10	0	1	1	0	0	GO:
0048255 MRNA_stabilization
_01	7	0	1	1	0	0	GO:
0048260 Positive_regulation_of_receptor-mediated_endocytosis
_01	7	0	1	1	0	0	GO:
0048265 Response_to_pain
_01	5	0	1	1	0	0	GO:
0048268 Clathrin_coat_assembly
_01	19	0	1	1	0	0	GO:
0048286 Lung_alveolus_development
_01	6	0	1	1	0	0	GO:
0048304 Positive_regulation_of_isotype_switching_to_IgG_isotypes
_01	20	0	1	1	0	0	GO:
0048306 Calcium-dependent_protein_binding
_01	5	0	1	1	0	0	GO:
0048339 Paraxial_mesoderm_development
_01	7	0	1	1	0	0	GO:
0048365 Rac_GTPase_binding
_01	6	0	1	1	0	0	GO:
0048384 Retinoic_acid_receptor_signaling_pathway
_01	8	0	1	1	0	0	GO:
0048407 Platelet-derived_growth_factor_binding
_01	12	0	1	1	0	0	GO:
0048468 Cell_development
_01	17	0	1	1	0	0	GO:
0048469 Cell_maturation
_01	236	0	1	1	0	0	GO:
0048471 Perinuclear_region_of_cytoplasm
_01	9	1	1	1	0	6.9e-05	GO:
0048477 Oogenesis
_01	6	0	1	1	0	0	GO:
0048484 Enteric_nervous_system_development
_01	7	0	1	1	0	0	GO:
0048485 Sympathetic_nervous_system_development
_01	10	0	1	1	0	0	GO:
0048487 Beta-tubulin_binding
_01	5	0	1	1	0	0	GO:
0048489 Synaptic_vesicle_transport
_01	14	0	1	1	0	0	GO:
0048511 Rhythmic_process
_01	5	0	1	1	0	0	GO:
0048513 Organ_development
_01	10	0	1	1	0	0	GO:
0048514 Blood_vessel_morphogenesis
_01	12	0	1	1	0	0	GO:

```
0048536 Spleen_development
_01     19      0       1       1       0       0       GO:
0048538 Thymus_development
_01     5       0       1       1       0       0       GO:
0048541 Peyer's_patch_development
_01     14      0       1       1       0       0       GO:
0048545 Response_to_steroid_hormone_stimulus
_01     10      0       1       1       0       0       GO:
0048565 Digestive_tract_development
_01     7       0       1       1       0       0       GO:
0048566 Embryonic_digestive_tract_development
_01     8       0       1       1       0       0       GO:
0048568 Embryonic_organ_development
_01     20      0       1       1       0       0       GO:
0048589 Developmental_growth
_01     12      0       1       1       0       0       GO:
0048593 Camera-type_eye_morphogenesis
_01     7       1       1       1       0       6.9e-05 GO:
0048599 Oocyte_development
_01     6       0       1       1       0       0       GO:
0048617 Embryonic_foregut_morphogenesis
_01     5       1       1       1       0       0.000339GO:
0048644 Muscle_organ_morphogenesis
_01     10      0       1       1       0       0       GO:
0048646 Anatomical_structure_formation_involved_in_morphogenesis
_01     20      1       1       1       0       3.9e-05 GO:
0048661 Positive_regulation_of_smooth_muscle_cell_proliferation
_01     9       0       1       1       0       0       GO:
0048662 Negative_regulation_of_smooth_muscle_cell_proliferation
_01     11      0       1       1       0       0       GO:
0048663 Neuron_fate_commitment
_01     7       1       1       1       0       6.3e-05 GO:
0048665 Neuron_fate_specification
_01     19      0       1       1       0       0       GO:
0048666 Neuron_development
_01     13      0       1       1       0       0       GO:
0048678 Response_to_axon_injury
_01     5       0       1       1       0       0       GO:
0048699 Generation_of_neurons
_01     10      0       1       1       0       0       GO:
0048701 Embryonic_cranial_skeleton_morphogenesis
_01     7       0       1       1       0       0       GO:
0048703 Embryonic_viscerocranium_morphogenesis
_01     31      1       1       1       0       0.000339GO:
0048704 Embryonic_skeletal_system_morphogenesis
_01     17      0       1       1       0       0       GO:
0048705 Skeletal_system_morphogenesis
_01     15      0       1       1       0       0       GO:
0048706 Embryonic_skeletal_system_development
_01     8       0       1       1       0       0       GO:
0048709 Oligodendrocyte_differentiation
_01     8       0       1       1       0       0       GO:
0048738 Cardiac_muscle_tissue_development
_01     6       0       1       1       0       0       GO:
```

0048741 Skeletal_muscle_fiber_development
_01	9	0	1	1	0	0	GO:
0048745 Smooth_muscle_tissue_development
_01	18	1	1	1	0	6.3e-05	GO:
0048754 Branching_morphogenesis_of_a_tube
_01	5	0	1	1	0	0	GO:
0048786 Presynaptic_active_zone
_01	12	0	1	1	0	0	GO:
0048812 Neuron_projection_morphogenesis
_01	7	0	1	1	0	0	GO:
0048813 Dendrite_morphogenesis
_01	16	0	1	1	0	0	GO:
0048839 Inner_ear_development
_01	5	0	1	1	0	0	GO:
0048843
Negative_regulation_of_axon_extension_involved_in_axon_guidance
_01	15	0	1	1	0	0	GO:
0048844 Artery_morphogenesis
_01	5	0	1	1	0	0	GO:
0048853 Forebrain_morphogenesis
_01	9	0	1	1	0	0	GO:
0048873 Homeostasis_of_number_of_cells_within_a_tissue
_01	6	0	1	1	0	0	GO:
0050427 3'-phosphoadenosine_5'-phosphosulfate_metabolic_process
_01	7	0	1	1	0	0	GO:
0050431 Transforming_growth_factor_beta_binding
_01	24	0	1	1	0	0	GO:
0050434 Positive_regulation_of_viral_transcription
_01	9	0	1	1	0	0	GO:
0050482 Arachidonic_acid_secretion
_01	48	0	1	1	0	0	GO:
0050660 Flavin_adenine_dinucleotide_binding
_01	22	0	1	1	0	0	GO:
0050661 NADP_or_NADPH_binding
_01	10	0	1	1	0	0	GO:
0050662 Coenzyme_binding
_01	5	0	1	1	0	0	GO:
0050673 Epithelial_cell_proliferation
_01	28	0	1	1	0	0	GO:
0050679 Positive_regulation_of_epithelial_cell_proliferation
_01	20	0	1	1	0	0	GO:
0050680 Negative_regulation_of_epithelial_cell_proliferation
_01	18	0	1	1	0	0	GO:
0050681 Androgen_receptor_binding
_01	14	0	1	1	0	0	GO:
0050690 Regulation_of_defense_response_to_virus_by_virus
_01	9	0	1	1	0	0	GO:
0050699 WW_domain_binding
_01	5	0	1	1	0	0	GO:
0050700 CARD_domain_binding
_01	11	0	1	1	0	0	GO:
0050714 Positive_regulation_of_protein_secretion
_01	11	0	1	1	0	0	GO:
0050715 Positive_regulation_of_cytokine_secretion

_01 8 0 1 1 0 0 GO:
0050718 Positive_regulation_of_interleukin-1_beta_secretion
_01 8 1 1 1 0 3.9e-05 GO:
0050727 Regulation_of_inflammatory_response
_01 17 0 1 1 0 0 GO:
0050728 Negative_regulation_of_inflammatory_response
_01 16 0 1 1 0 0 GO:
0050729 Positive_regulation_of_inflammatory_response
_01 7 0 1 1 0 0 GO:
0050730 Regulation_of_peptidyl-tyrosine_phosphorylation
_01 34 0 1 1 0 0 GO:
0050731 Positive_regulation_of_peptidyl-tyrosine_phosphorylation
_01 5 0 1 1 0 0 GO:
0050732 Negative_regulation_of_peptidyl-tyrosine_phosphorylation
_01 10 0 1 1 0 0 GO:
0050750 Low-density_lipoprotein_receptor_binding
_01 9 0 1 1 0 0 GO:
0050766 Positive_regulation_of_phagocytosis
_01 9 0 1 1 0 0 GO:
0050768 Negative_regulation_of_neurogenesis
_01 7 0 1 1 0 0 GO:
0050769 Positive_regulation_of_neurogenesis
_01 13 0 1 1 0 0 GO:
0050770 Regulation_of_axonogenesis
_01 12 0 1 1 0 0 GO:
0050771 Negative_regulation_of_axonogenesis
_01 10 0 1 1 0 0 GO:
0050772 Positive_regulation_of_axonogenesis
_01 7 0 1 1 0 0 GO:
0050775 Positive_regulation_of_dendrite_morphogenesis
_01 41 0 1 1 0 0 GO:
0050776 Regulation_of_immune_response
_01 52 0 1 1 0 0 GO:
0050796 Regulation_of_insulin_secretion
_01 5 0 1 1 0 0 GO:
0050804 Regulation_of_synaptic_transmission
_01 5 0 1 1 0 0 GO:
0050806 Positive_regulation_of_synaptic_transmission
_01 5 0 1 1 0 0 GO:
0050808 Synapse_organization
_01 5 0 1 1 0 0 GO:
0050810 Regulation_of_steroid_biosynthetic_process
_01 6 0 1 1 0 0 GO:
0050811 GABA_receptor_binding
_01 26 0 1 1 0 0 GO:
0050821 Protein_stabilization
_01 7 0 1 1 0 0 GO:
0050829 Defense_response_to_Gram-negative_bacterium
_01 15 0 1 1 0 0 GO:
0050830 Defense_response_to_Gram-positive_bacterium
_01 5 0 1 1 0 0 GO:
0050832 Defense_response_to_fungus
_01 7 0 1 1 0 0 GO:
0050839 Cell_adhesion_molecule_binding

_01	9	0	1	1	0	0	GO:
0050840 Extracellular_matrix_binding
_01	12	0	1	1	0	0	GO:
0050850 Positive_regulation_of_calcium-mediated_signaling
_01	49	0	1	1	0	0	GO:
0050852 T_cell_receptor_signaling_pathway
_01	10	0	1	1	0	0	GO:
0050853 B_cell_receptor_signaling_pathway
_01	5	0	1	1	0	0	GO:
0050860 Negative_regulation_of_T_cell_receptor_signaling_pathway
_01	6	0	1	1	0	0	GO:
0050870 Positive_regulation_of_T_cell_activation
_01	5	0	1	1	0	0	GO:
0050872 White_fat_cell_differentiation
_01	18	0	1	1	0	0	GO:
0050873 Brown_fat_cell_differentiation
_01	5	0	1	1	0	0	GO:
0050877 Neurological_system_process
_01	11	0	1	1	0	0	GO:
0050880 Regulation_of_blood_vessel_size
_01	14	0	1	1	0	0	GO:
0050885 Neuromuscular_process_controlling_balance
_01	6	0	1	1	0	0	GO:
0050890 Cognition
_01	5	0	1	1	0	0	GO:
0050892 Intestinal_absorption
_01	406	0	1	1	0	0	GO:
0050896 Response_to_stimulus
_01	61	0	1	1	0	0	GO:
0050900 Leukocyte_migration
_01	5	0	1	1	0	0	GO:
0050901 Leukocyte_tethering_or_rolling
_01	13	0	1	1	0	0	GO:
0050909 Sensory_perception_of_taste
_01	7	0	1	1	0	0	GO:
0050910
Detection_of_mechanical_stimulus_involved_in_sensory_perception_of_s
ound
_01	5	0	1	1	0	0	GO:
0050918 Positive_chemotaxis
_01	5	0	1	1	0	0	GO:
0050919 Negative_chemotaxis
_01	7	0	1	1	0	0	GO:
0050927 Positive_regulation_of_positive_chemotaxis
_01	8	0	1	1	0	0	GO:
0050930 Induction_of_positive_chemotaxis
_01	8	0	1	1	0	0	GO:
0050953 Sensory_perception_of_light_stimulus
_01	6	0	1	1	0	0	GO:
0050957 Equilibrioception
_01	7	0	1	1	0	0	GO:
0050995 Negative_regulation_of_lipid_catabolic_process
_01	8	0	1	1	0	0	GO:
0050999 Regulation_of_nitric-oxide_synthase_activity

_01 8 0 1 1 0 0 GO:
0051000 Positive_regulation_of_nitric-oxide_synthase_activity
_01 7 0 1 1 0 0 GO:
0051006 Positive_regulation_of_lipoprotein_lipase_activity
_01 8 0 1 1 0 0 GO:
0051010 Microtubule_plus-end_binding
_01 33 1 1 1 0 6.9e-05 GO:
0051015 Actin_filament_binding
_01 10 1 1 1 0 6.9e-05 GO:
0051017 Actin_filament_bundle_assembly
_01 5 0 1 1 0 0 GO:
0051018 Protein_kinase_A_binding
_01 5 0 1 1 0 0 GO:
0051019 Mitogen-activated_protein_kinase_binding
_01 11 0 1 1 0 0 GO:
0051020 GTPase_binding
_01 27 0 1 1 0 0 GO:
0051028 MRNA_transport
_01 7 0 1 1 0 0 GO:
0051044
Positive_regulation_of_membrane_protein_ectodomain_proteolysis
_01 106 2 0.00927499 0.466609 0 0.000236
GO:0051056 Regulation_of_small_GTPase_mediated_signal_transduction
_01 10 0 1 1 0 0 GO:
0051059 NF-kappaB_binding
_01 72 0 1 1 0 0 GO:
0051082 Unfolded_protein_binding
_01 19 0 1 1 0 0 GO:
0051084 'de_novo'_posttranslational_protein_folding
_01 8 0 1 1 0 0 GO:
0051085 Chaperone_mediated_protein_folding_requiring_cofactor
_01 18 0 1 1 0 0 GO:
0051087 Chaperone_binding
_01 40 1 1 1 0 6.3e-05 GO:
0051090 Regulation_of_transcription_factor_activity
_01 28 1 1 1 0 6.3e-05 GO:
0051091 Positive_regulation_of_transcription_factor_activity
_01 41 0 1 1 0 0 GO:
0051092 Positive_regulation_of_NF-
kappaB_transcription_factor_activity
_01 12 0 1 1 0 0 GO:
0051117 ATPase_binding
_01 7 0 1 1 0 0 GO:
0051145 Smooth_muscle_cell_differentiation
_01 8 0 1 1 0 0 GO:
0051209 Release_of_sequestered_calcium_ion_into_cytosol
_01 28 0 1 1 0 0 GO:
0051216 Cartilage_development
_01 15 0 1 1 0 0 GO:
0051219 Phosphoprotein_binding
_01 13 0 1 1 0 0 GO:
0051225 Spindle_assembly
_01 27 0 1 1 0 0 GO:
0051246 Regulation_of_protein_metabolic_process

_01 14 0 1 1 0 0 GO:
0051258 Protein_polymerization
_01 20 0 1 1 0 0 GO:
0051259 Protein_oligomerization
_01 47 0 1 1 0 0 GO:
0051260 Protein_homooligomerization
_01 7 0 1 1 0 0 GO:
0051262 Protein_tetramerization
_01 10 0 1 1 0 0 GO:
0051272 Positive_regulation_of_cellular_component_movement
_01 9 0 1 1 0 0 GO:
0051276 Chromosome_organization
_01 8 0 1 1 0 0 GO:
0051281
Positive_regulation_of_release_of_sequestered_calcium_ion_into_cytos
ol
_01 23 0 1 1 0 0 GO:
0051287 NAD_or_NADH_binding
_01 16 0 1 1 0 0 GO:
0051289 Protein_homotetramerization
_01 5 0 1 1 0 0 GO:
0051290 Protein_heterotetramerization
_01 33 0 1 1 0 0 GO:
0051291 Protein_heterooligomerization
_01 10 0 1 1 0 0 GO:
0051297 Centrosome_organization
_01 5 0 1 1 0 0 GO:
0051298 Centrosome_duplication
_01 145 1 1 1 0 0.000423 GO:
0051301 Cell_division
_01 5 0 1 1 0 0 GO:
0051378 Serotonin_binding
_01 47 1 1 1 0 3.9e-05 GO:
0051384 Response_to_glucocorticoid_stimulus
_01 11 0 1 1 0 0 GO:
0051402 Neuron_apoptosis
_01 34 0 1 1 0 0 GO:
0051403 Stress-activated_MAPK_cascade
_01 14 0 1 1 0 0 GO:
0051412 Response_to_corticosterone_stimulus
_01 46 1 1 1 0 0.000423 GO:
0051436 Negative_regulation_of_ubiquitin-
protein_ligase_activity_involved_in_mitotic_cell_cycle
_01 51 1 1 1 0 0.000423 GO:
0051437 Positive_regulation_of_ubiquitin-
protein_ligase_activity_involved_in_mitotic_cell_cycle
_01 52 1 1 1 0 0.000423 GO:
0051439 Regulation_of_ubiquitin-
protein_ligase_activity_involved_in_mitotic_cell_cycle
_01 6 0 1 1 0 0 GO:
0051457 Maintenance_of_protein_location_in_nucleus
_01 7 0 1 1 0 0 GO:
0051480 Cytosolic_calcium_ion_homeostasis
_01 9 0 1 1 0 0 GO:

0051482
Elevation_of_cytosolic_calcium_ion_concentration_involved_in_G-protein_signaling_coupled_to_IP3_second_messenger
_01 8 0 1 1 0 0 GO:
0051491 Positive_regulation_of_filopodium_assembly
_01 11 0 1 1 0 0 GO:
0051496 Positive_regulation_of_stress_fiber_assembly
_01 9 0 1 1 0 0 GO:
0051536 Iron-sulfur_cluster_binding
_01 10 0 1 1 0 0 GO:
0051537 2_iron,_2_sulfur_cluster_binding
_01 15 0 1 1 0 0 GO:
0051539 4_iron,_4_sulfur_cluster_binding
_01 8 1 1 1 0 0.000423 GO:
0051568 Histone_H3-K4_methylation
_01 26 0 1 1 0 0 GO:
0051591 Response_to_cAMP
_01 34 0 1 1 0 0 GO:
0051592 Response_to_calcium_ion
_01 7 0 1 1 0 0 GO:
0051602 Response_to_electrical_stimulus
_01 15 0 1 1 0 0 GO:
0051603 Proteolysis_involved_in_cellular_protein_catabolic_process
_01 11 0 1 1 0 0 GO:
0051605 Protein_maturation_by_peptide_bond_cleavage
_01 16 0 1 1 0 0 GO:
0051607 Defense_response_to_virus
_01 7 0 1 1 0 0 GO:
0051635 Bacterial_cell_surface_binding
_01 5 0 1 1 0 0 GO:
0051642 Centrosome_localization
_01 12 0 1 1 0 0 GO:
0051693 Actin_filament_capping
_01 9 0 1 1 0 0 GO:
0051721 Protein_phosphatase_2A_binding
_01 36 1 1 1 0 3.9e-05 GO:
0051726 Regulation_of_cell_cycle
_01 6 0 1 1 0 0 GO:
0051739 Ammonia_transmembrane_transporter_activity
_01 30 0 1 1 0 0 GO:
0051781 Positive_regulation_of_cell_division
_01 5 0 1 1 0 0 GO:
0051787 Misfolded_protein_binding
_01 30 0 1 1 0 0 GO:
0051789 Response_to_protein_stimulus
_01 5 1 1 1 0 0.000423 GO:
0051864 Histone_demethylase_activity_(H3-K36_specific)
_01 15 0 1 1 0 0 GO:
0051865 Protein_autoubiquitination
_01 7 0 1 1 0 0 GO:
0051879 Hsp90_protein_binding
_01 6 0 1 1 0 0 GO:
0051881 Regulation_of_mitochondrial_membrane_potential
_01 16 0 1 1 0 0 GO:

0051897 Positive_regulation_of_protein_kinase_B_signaling_cascade
_01 5 0 1 1 0 0 GO:
0051898 Negative_regulation_of_protein_kinase_B_signaling_cascade
_01 15 0 1 1 0 0 GO:
0051899 Membrane_depolarization
_01 6 0 1 1 0 0 GO:
0051918 Negative_regulation_of_fibrinolysis
_01 11 1 1 1 0 0.000423 GO:
0051924 Regulation_of_calcium_ion_transport
_01 9 1 1 1 0 0.000423 GO:
0051928 Positive_regulation_of_calcium_ion_transport
_01 13 0 1 1 0 0 GO:
0051930 Regulation_of_sensory_perception_of_pain
_01 6 0 1 1 0 0 GO:
0051965 Positive_regulation_of_synaptogenesis
_01 6 0 1 1 0 0 GO:
0051967 Negative_regulation_of_synaptic_transmission,_glutamatergic
_01 7 1 1 1 0 3.9e-05 GO:
0051968 Positive_regulation_of_synaptic_transmission,_glutamatergic
_01 9 0 1 1 0 0 GO:
0055007 Cardiac_muscle_cell_differentiation
_01 5 0 1 1 0 0 GO:
0055008 Cardiac_muscle_tissue_morphogenesis
_01 5 0 1 1 0 0 GO:
0055009 Atrial_cardiac_muscle_tissue_morphogenesis
_01 19 0 1 1 0 0 GO:
0055010 Ventricular_cardiac_muscle_tissue_morphogenesis
_01 8 0 1 1 0 0 GO:
0055015 Ventricular_cardiac_muscle_cell_development
_01 12 0 1 1 0 0 GO:
0055037 Recycling_endosome
_01 9 0 1 1 0 0 GO:
0055038 Recycling_endosome_membrane
_01 6 0 1 1 0 0 GO:
0055056 D-glucose_transmembrane_transporter_activity
_01 7 0 1 1 0 0 GO:
0055074 Calcium_ion_homeostasis
_01 404 0 1 1 0 0 GO:
0055085 Transmembrane_transport
_01 56 0 1 1 0 0 GO:
0055086 Nucleobase,_nucleoside_and_nucleotide_metabolic_process
_01 10 0 1 1 0 0 GO:
0055088 Lipid_homeostasis
_01 7 0 1 1 0 0 GO:
0055093 Response_to_hyperoxia
_01 321 2 0.01498 0.529507 0 0.000231 GO:
0055114 Oxidation-reduction_process
_01 5 0 1 1 0 0 GO:
0055117 Regulation_of_cardiac_muscle_contraction
_01 5 0 1 1 0 0 GO:
0060017 Parathyroid_gland_development
_01 30 0 1 1 0 0 GO:
0060021 Palate_development
_01 7 0 1 1 0 0 GO:

0060037 Pharyngeal_system_development
_01 5 0 1 1 0 0 GO:
0060039 Pericardium_development
_01 14 0 1 1 0 0 GO:
0060041 Retina_development_in_camera-type_eye
_01 9 0 1 1 0 0 GO:
0060045 Positive_regulation_of_cardiac_muscle_cell_proliferation
_01 13 0 1 1 0 0 GO:
0060048 Cardiac_muscle_contraction
_01 5 0 1 1 0 0 GO:
0060055 Angiogenesis_involved_in_wound_healing
_01 5 0 1 1 0 0 GO:
0060056 Mammary_gland_involution
_01 29 0 1 1 0 0 GO:
0060070 Canonical_Wnt_receptor_signaling_pathway
_01 5 0 1 1 0 0 GO:
0060071 Wnt_receptor_signaling_pathway,_planar_cell_polarity_pathway
_01 11 0 1 1 0 0 GO:
0060079 Regulation_of_excitatory_postsynaptic_membrane_potential
_01 5 0 1 1 0 0 GO:
0060134 Prepulse_inhibition
_01 8 0 1 1 0 0 GO:
0060135 Maternal_process_involved_in_female_pregnancy
_01 5 0 1 1 0 0 GO:
0060155 Platelet_dense_granule_organization
_01 7 0 1 1 0 0 GO:
0060170 Cilium_membrane
_01 8 0 1 1 0 0 GO:
0060173 Limb_development
_01 6 0 1 1 0 0 GO:
0060174 Limb_bud_formation
_01 6 0 1 1 0 0 GO:
0060271 Cilium_morphogenesis
_01 8 0 1 1 0 0 GO:
0060325 Face_morphogenesis
_01 8 0 1 1 0 0 GO:
0060326 Cell_chemotaxis
_01 7 0 1 1 0 0 GO:
0060347 Heart_trabecula_formation
_01 6 0 1 1 0 0 GO:
0060349 Bone_morphogenesis
_01 9 0 1 1 0 0 GO:
0060389 Pathway-restricted_SMAD_protein_phosphorylation
_01 6 0 1 1 0 0 GO:
0060391 Positive_regulation_of_SMAD_protein_import_into_nucleus
_01 13 0 1 1 0 0 GO:
0060395 SMAD_protein_signal_transduction
_01 5 0 1 1 0 0 GO:
0060406 Positive_regulation_of_penile_erection
_01 7 0 1 1 0 0 GO:
0060412 Ventricular_septum_morphogenesis
_01 5 0 1 1 0 0 GO:
0060425 Lung_morphogenesis
_01 6 0 1 1 0 0 GO:

0060426 Lung_vasculature_development
_01 6 1 1 1 0 6.3e-05 GO:
0060441 Epithelial_tube_branching_involved_in_lung_morphogenesis
_01 6 0 1 1 0 0 GO:
0060445 Branching_involved_in_salivary_gland_morphogenesis
_01 7 0 1 1 0 0 GO:
0060484 Lung-associated_mesenchyme_development
_01 5 0 1 1 0 0 GO:
0060527
Prostate_epithelial_cord_arborization_involved_in_prostate_glandular
_acinus_morphogenesis
_01 10 0 1 1 0 0 GO:
0060548 Negative_regulation_of_cell_death
_01 5 0 1 1 0 0 GO:
0060612 Adipose_tissue_development
_01 6 0 1 1 0 0 GO:
0060644 Mammary_gland_epithelial_cell_differentiation
_01 5 0 1 1 0 0 GO:
0060666
Dichotomous_subdivision_of_terminal_units_involved_in_salivary_gland
_branching
_01 5 0 1 1 0 0 GO:
0060670 Branching_involved_in_embryonic_placenta_morphogenesis
_01 5 0 1 1 0 0 GO:
0060687
Regulation_of_branching_involved_in_prostate_gland_morphogenesis
_01 7 0 1 1 0 0 GO:
0060707 Trophoblast_giant_cell_differentiation
_01 11 0 1 1 0 0 GO:
0060716 Labyrinthine_layer_blood_vessel_development
_01 7 1 1 1 0 6.3e-05 GO:
0060740 Prostate_gland_epithelium_morphogenesis
_01 7 0 1 1 0 0 GO:
0060749 Mammary_gland_alveolus_development
_01 5 0 1 1 0 0 GO:
0060828 Regulation_of_canonical_Wnt_receptor_signaling_pathway
_01 5 0 1 1 0 0 GO:
0061036 Positive_regulation_of_cartilage_development
_01 6 0 1 1 0 0 GO:
0061202 Clathrin_sculpted_gamma-
aminobutyric_acid_transport_vesicle_membrane
_01 5 0 1 1 0 0 GO:
0070016 Armadillo_repeat_domain_binding
_01 7 0 1 1 0 0 GO:
0070064 Proline-rich_region_binding
_01 5 0 1 1 0 0 GO:
0070198 Protein_localization_to_chromosome,_telomeric_region
_01 5 0 1 1 0 0 GO:
0070207 Protein_homotrimerization
_01 6 0 1 1 0 0 GO:
0070301 Cellular_response_to_hydrogen_peroxide
_01 9 0 1 1 0 0 GO:
0070328 Triglyceride_homeostasis
_01 10 0 1 1 0 0 GO:

0070330 Aromatase_activity
_01 5 0 1 1 0 0 GO:
0070371 ERK1_and_ERK2_cascade
_01 9 0 1 1 0 0 GO:
0070373 Negative_regulation_of_ERK1_and_ERK2_cascade
_01 29 0 1 1 0 0 GO:
0070374 Positive_regulation_of_ERK1_and_ERK2_cascade
_01 7 0 1 1 0 0 GO:
0070403 NAD_binding
_01 8 0 1 1 0 0 GO:
0070411 I-SMAD_binding
_01 9 0 1 1 0 0 GO:
0070412 R-SMAD_binding
_01 24 0 1 1 0 0 GO:
0070469 Respiratory_chain
_01 10 0 1 1 0 0 GO:
0070491 Repressing_transcription_factor_binding
_01 9 0 1 1 0 0 GO:
0070534 Protein_K63-linked_ubiquitination
_01 6 0 1 1 0 0 GO:
0070536 Protein_K63-linked_deubiquitination
_01 8 0 1 1 0 0 GO:
0070542 Response_to_fatty_acid
_01 16 0 1 1 0 0 GO:
0070555 Response_to_interleukin-1
_01 5 0 1 1 0 0 GO:
0070652 HAUS_complex
_01 6 0 1 1 0 0 GO:
0070688 MLL5-L_complex
_01 5 0 1 1 0 0 GO:
0070695 FHF_complex
_01 7 0 1 1 0 0 GO:
0070742 C2H2_zinc_finger_domain_binding
_01 5 0 1 1 0 0 GO:
0070761 Pre-snoRNP_complex
_01 5 0 1 1 0 0 GO:
0070776 MOZ/MORF_histone_acetyltransferase_complex
_01 16 0 1 1 0 0 GO:
0070830 Tight_junction_assembly
_01 5 0 1 1 0 0 GO:
0070848 Response_to_growth_factor_stimulus
_01 6 0 1 1 0 0 GO:
0070888 E-box_binding
_01 5 1 1 1 0 5.2e-05 GO:
0070934 CRD-mediated_mRNA_stabilization
_01 12 0 1 1 0 0 GO:
0070936 Protein_K48-linked_ubiquitination
_01 5 1 1 1 0 5.2e-05 GO:
0070937 CRD-mediated_mRNA_stability_complex
_01 15 1 1 1 0 0.000423 GO:
0070979 Protein_K11-linked_ubiquitination
_01 12 0 1 1 0 0 GO:
0071044 Histone_mRNA_catabolic_process
_01 6 0 1 1 0 0 GO:

```
0071108 Protein_K48-linked_deubiquitination
_01     6       0       1       1       0       0       GO:
0071157 Negative_regulation_of_cell_cycle_arrest
_01     5       0       1       1       0       0       GO:
0071203 WASH_complex
_01     12      0       1       1       0       0       GO:
0071222 Cellular_response_to_lipopolysaccharide
_01     6       0       1       1       0       0       GO:
0071230 Cellular_response_to_amino_acid_stimulus
_01     13      0       1       1       0       0       GO:
0071300 Cellular_response_to_retinoic_acid
_01     15      0       1       1       0       0       GO:
0071339 MLL1_complex
_01     5       0       1       1       0       0       GO:
0071347 Cellular_response_to_interleukin-1
_01     11      0       1       1       0       0       GO:
0071363 Cellular_response_to_growth_factor_stimulus
_01     7       0       1       1       0       0       GO:
0071407 Cellular_response_to_organic_cyclic_substance
_01     7       0       1       1       0       0       GO:
0071445 Cellular_response_to_protein_stimulus
_01     7       0       1       1       0       0       GO:
0071456 Cellular_response_to_hypoxia
_01     5       0       1       1       0       0       GO:
0071560
Cellular_response_to_transforming_growth_factor_beta_stimulus
_01     8       0       1       1       0       0       GO:
0071564 NpBAF_complex
_01     9       0       1       1       0       0       GO:
0071565 NBAF_complex
_01     5       0       1       1       0       0       GO:
0072357 PTW/PP1_phosphatase_complex
_01     7       0       1       1       0       0       GO:
0080008 CUL4_RING_ubiquitin_ligase_complex
_01     6       0       1       1       0       0       GO:
0080025 Phosphatidylinositol-3,5-bisphosphate_binding
_01     31      0       1       1       0       0       GO:
0090090
Negative_regulation_of_canonical_Wnt_receptor_signaling_pathway
_01     5       0       1       1       0       0       GO:
0090136 Epithelial_cell-cell_adhesion
_01     16      0       1       1       0       0       GO:
0090263
Positive_regulation_of_canonical_Wnt_receptor_signaling_pathway
_01     5       0       1       1       0       0       GO:
0090286 Cytoskeletal_anchoring_at_nuclear_membrane


-------------------------------------------------------------
II. Interval-Gene-Target Summary
-------------------------------------------------------------
_02     INTERVAL GENE_LOC GENE_ID  GENE_DESC       TARGET   P
_02     int20:chrM:7119859..7845353      chrM:7794400..7841032
```

```
51433   ANAPC5 A        GO:0000075 Cell_cycle_checkpoint  1
_O2     int20:chrM:7119859..7845353      chrM:7794400..7841032
51433   ANAPC5 A        GO:0000086 G2/
M_transition_of_mitotic_cell_cycle       1
_O2     int9:chr8:16013494..16106285     chr8:16077537..16083232
3169    FOXA1 F         GO:0000122
Negative_regulation_of_transcription_from_RNA_polymerase_II_promoter
        1.1e-05
_O2     int10:chr8:63223904..63813192    chr8:63705038..63706981
145258  GSC G   GO:0000122
Negative_regulation_of_transcription_from_RNA_polymerase_II_promoter
        1.1e-05
_O2     int13:chr16:45965023..46373321   chr16:46053149..46131685
3660    IRF2 I  GO:0000122
Negative_regulation_of_transcription_from_RNA_polymerase_II_promoter
        1.1e-05
_O2     int16:chrX:24666408..24763994    chrX:24599559..24692559
6304    SATB1 S         GO:0000122
Negative_regulation_of_transcription_from_RNA_polymerase_II_promoter
        1.1e-05
_O2     int20:chrM:7119859..7845353      chrM:7145120..7148771
79369   B3GNT4 U        GO:0000139 Golgi_membrane        1
_O2     int3:chr6:18012525..18106914     chr6:18010887..18029186
9344    TAOK2 T         GO:0000186 Activation_of_MAPKK_activity
1
_O2     int3:chr6:18012525..18106914     chr6:18085139..18092858
5531    PPP4C P         GO:0000226
Microtubule_cytoskeleton_organization     1
_O2     int20:chrM:7119859..7845353      chrM:7794400..7841032
51433   ANAPC5 A        GO:0000278 Mitotic_cell_cycle     1
_O2     int5:chr7:16160915..16235033     chr7:16233741..16292928
1660    DHX9 D  GO:0000398 Nuclear_mRNA_splicing,_via_spliceosome
1
_O2     int16:chrX:24666408..24763994    chrX:24599559..24692559
6304    SATB1 S         GO:0000785 Chromatin     1
_O2     int7:chr7:19633367..19714459     chr7:19668098..19674935
5743    PTGS2 P         GO:0001516
Prostaglandin_biosynthetic_process       1
_O2     int0:chr4:31133158..31204643     chr4:30515861..31557604
10718   NRG3 N  GO:0001558 Regulation_of_cell_growth
9.99999e-06
_O2     int3:chr6:18012525..18106914     chr6:18010887..18029186
9344    TAOK2 T         GO:0001558 Regulation_of_cell_growth
9.99999e-06
_O2     int8:chr7:22714810..22804810     chr7:22772438..23143385
60676   PAPPA2 P        GO:0001558 Regulation_of_cell_growth
9.99999e-06
_O2     int10:chr8:63223904..63813192    chr8:63386661..63400389
5265    SERPINA1 S      GO:0001666 Response_to_hypoxia    1
_O2     int3:chr6:18012525..18106914     chr6:18093188..18098314
6911    TBX6 T  GO:0001707 Mesoderm_formation     1
_O2     int3:chr6:18012525..18106914     chr6:18093188..18098314
6911    TBX6 T  GO:0001708 Cell_fate_specification       1
_O2     int10:chr8:63223904..63813192    chr8:63386661..63400389
```

5265	SERPINA1 S	GO:0002020 Protease_binding	1
_02	int10:chr8:63223904..63813192	chr8:63567114..63577016
5104	SERPINA5 S	GO:0002020 Protease_binding	1
_02	int3:chr6:18012525..18106914	chr6:18069904..18083053
226	ALDOA A	GO:0002576 Platelet_degranulation
0.000348
_02	int10:chr8:63223904..63813192	chr8:63386661..63400389
5265	SERPINA1 S	GO:0002576 Platelet_degranulation
0.000348
_02	int20:chrM:7119859..7845353	chrM:7264614..7295832
605	BCL7A B	GO:0003674 Molecular_function	1
_02	int18:chrX:48440818..48536955	chrX:48407005..48456332
170506	DHX36 D	GO:0003676 Nucleic_acid_binding	1
_02	int9:chr8:16013494..16106285	chr8:16077537..16083232
3169	FOXA1 F	GO:0003690 Double-stranded_DNA_binding
0.0012
_02	int16:chrX:24666408..24763994	chrX:24599559..24692559
6304	SATB1 S	GO:0003690 Double-stranded_DNA_binding
0.0012
_02	int3:chr6:18012525..18106914	chr6:18093188..18098314
6911	TBX6 T	GO:0003700 Sequence-
specific_DNA_binding_transcription_factor_activity	3e-06
_02	int9:chr8:16013494..16106285	chr8:16077537..16083232
3169	FOXA1 F	GO:0003700 Sequence-
specific_DNA_binding_transcription_factor_activity	3e-06
_02	int10:chr8:63223904..63813192	chr8:63705038..63706981
145258	GSC G	GO:0003700 Sequence-
specific_DNA_binding_transcription_factor_activity	3e-06
_02	int13:chr16:45965023..46373321	chr16:46053149..46131685
3660	IRF2 I	GO:0003700 Sequence-
specific_DNA_binding_transcription_factor_activity	3e-06
_02	int16:chrX:24666408..24763994	chrX:24599559..24692559
6304	SATB1 S	GO:0003700 Sequence-
specific_DNA_binding_transcription_factor_activity	3e-06
_02	int20:chrM:7119859..7845353	chrM:7223951..7225043
652963	BTF3P12 B	GO:0003700 Sequence-
specific_DNA_binding_transcription_factor_activity	3e-06
_02	int13:chr16:45965023..46373321	chr16:46053149..46131685
3660	IRF2 I	GO:0003702
RNA_polymerase_II_transcription_factor_activity	1
_02	int9:chr8:16013494..16106285	chr8:16077537..16083232
3169	FOXA1 F	GO:0003704
Specific_RNA_polymerase_II_transcription_factor_activity	1
_02	int9:chr8:16013494..16106285	chr8:16077537..16083232
3169	FOXA1 F	GO:0003705 Sequence-
specific_enhancer_binding_RNA_polymerase_II_transcription_factor_act
ivity	1
_02	int20:chrM:7119859..7845353	chrM:7443827..7467087
23067	SETD1B S	GO:0003723 RNA_binding	1
_02	int5:chr7:16160915..16235033	chr7:16233741..16292928
1660	DHX9 D	GO:0003724 RNA_helicase_activity	1
_02	int5:chr7:16160915..16235033	chr7:16233741..16292928
1660	DHX9 D	GO:0003725 Double-stranded_RNA_binding	1
_02	int3:chr6:18012525..18106914	chr6:18069904..18083053

226	ALDOA A		GO:0003779 Actin_binding	0.020267
_O2	int11:chr10:69076487..69161721	chr10:69056997..69142988
119	ADD2 A	GO:0003779 Actin_binding	0.020267
_O2	int20:chrM:7119859..7845353	chrM:7478035..7489428
54509	RHOF R	GO:0003924 GTPase_activity	1
_O2	int5:chr7:16160915..16235033	chr7:16233741..16292928
1660	DHX9 D	GO:0004003 ATP-dependent_DNA_helicase_activity
1
_O2	int5:chr7:16160915..16235033	chr7:16233741..16292928
1660	DHX9 D	GO:0004004 ATP-dependent_RNA_helicase_activity
1
_O2	int2:chr5:29189236..29284352	chr5:29187858..29196161
4316	MMP7 M	GO:0004222 Metalloendopeptidase_activity	0.001655
_O2	int8:chr7:22714810..22804810	chr7:22772438..23143385
60676	PAPPA2 P		GO:0004222 Metalloendopeptidase_activity
0.001655
_O2	int18:chrX:48440818..48536955	chrX:48407005..48456332
170506	DHX36 D		GO:0004386 Helicase_activity	1
_O2	int7:chr7:19633367..19714459	chr7:19668098..19674935
5743	PTGS2 P		GO:0004601 Peroxidase_activity	1
_O2	int3:chr6:18012525..18106914	chr6:18010887..18029186
9344	TAOK2 T		GO:0004674 Protein_serine/
threonine_kinase_activity	1
_O2	int3:chr6:18012525..18106914	chr6:18085139..18092858
5531	PPP4C P		GO:0004722 Protein_serine/
threonine_phosphatase_activity	1
_O2	int20:chrM:7119859..7845353	chrM:7794400..7841032
51433	ANAPC5 A		GO:0004842 Ubiquitin-
protein_ligase_activity	1
_O2	int10:chr8:63223904..63813192	chr8:63324149..63341782
866	SERPINA6 S		GO:0004867 Serine-
type_endopeptidase_inhibitor_activity	1
_O2	int10:chr8:63223904..63813192	chr8:63379422..63381819
390502	SERPINA2 S		GO:0004867 Serine-
type_endopeptidase_inhibitor_activity	1
_O2	int10:chr8:63223904..63813192	chr8:63386661..63400389
5265	SERPINA1 S		GO:0004867 Serine-
type_endopeptidase_inhibitor_activity	1
_O2	int10:chr8:63223904..63813192	chr8:63436708..63453265
256394	SERPINA11 S		GO:0004867 Serine-
type_endopeptidase_inhibitor_activity	1
_O2	int10:chr8:63223904..63813192	chr8:63473300..63488592
327657	SERPINA9 S		GO:0004867 Serine-
type_endopeptidase_inhibitor_activity	1
_O2	int10:chr8:63223904..63813192	chr8:63497827..63521295
145264	SERPINA12 S		GO:0004867 Serine-
type_endopeptidase_inhibitor_activity	1
_O2	int10:chr8:63223904..63813192	chr8:63552073..63560608
5267	SERPINA4 S		GO:0004867 Serine-
type_endopeptidase_inhibitor_activity	1
_O2	int10:chr8:63223904..63813192	chr8:63567114..63577016
5104	SERPINA5 S		GO:0004867 Serine-
type_endopeptidase_inhibitor_activity	1
_O2	int10:chr8:63223904..63813192	chr8:63588248..63596955

12	SERPINA3 S	GO:0004867 Serine-
type_endopeptidase_inhibitor_activity	1
_02	int18:chrX:48440818..48536955	chrX:48471675..48535300
344758	GPR149 G	GO:0004872 Receptor_activity	1
_02	int18:chrX:48440818..48536955	chrX:48471675..48535300
344758	GPR149 G	GO:0004930 G-
protein_coupled_receptor_activity	1
_02	int12:chr14:42191677..42265654	chr14:42217642..42513368
1124	CHN2 C	GO:0005070 SH3/SH2_adaptor_activity	1
_02	int12:chr14:42191677..42265654	chr14:42217642..42513368
1124	CHN2 C	GO:0005096 GTPase_activator_activity	1
_02	int20:chrM:7119859..7845353	chrM:7173150..7175594
386653	IL31 I	GO:0005125 Cytokine_activity	1
_02	int11:chr10:69076487..69161721	chr10:69056997..69142988
119	ADD2 A	GO:0005198 Structural_molecule_activity	1
_02	int3:chr6:18012525..18106914	chr6:18041311..18046793
8448	DOC2A D	GO:0005215 Transporter_activity	1
_02	int20:chrM:7119859..7845353	chrM:7584448..7599633
84876	ORAI1 O	GO:0005216 Ion_channel_activity	1
_02	int3:chr6:18012525..18106914	chr6:18041311..18046793
8448	DOC2A D	GO:0005488 Binding	0.023215
_02	int20:chrM:7119859..7845353	chrM:7794400..7841032
51433	ANAPC5 A	GO:0005488 Binding	0.023215
_02	int10:chr8:63223904..63813192	chr8:63324149..63341782
866	SERPINA6 S	GO:0005496 Steroid_binding	1
_02	int20:chrM:7119859..7845353	chrM:7308885..7375053
144406	WDR66 W	GO:0005509 Calcium_ion_binding	1
_02	int11:chr10:69076487..69161721	chr10:69056997..69142988
119	ADD2 A	GO:0005516 Calmodulin_binding	1
_02	int3:chr6:18012525..18106914	chr6:18010887..18029186
9344	TAOK2 T	GO:0005524 ATP_binding	0.047166
_02	int5:chr7:16160915..16235033	chr7:16233741..16292928
1660	DHX9 D	GO:0005524 ATP_binding	0.047166
_02	int18:chrX:48440818..48536955	chrX:48407005..48456332
170506	DHX36 D	GO:0005524 ATP_binding	0.047166
_02	int20:chrM:7119859..7845353	chrM:7478035..7489428
54509	RHOF R	GO:0005525 GTP_binding	1
_02	int10:chr8:63223904..63813192	chr8:63567114..63577016
5104	SERPINA5 S	GO:0005539 Glycosaminoglycan_binding
1
_02	int3:chr6:18012525..18106914	chr6:18041311..18046793
8448	DOC2A D	GO:0005544 Calcium-
dependent_phospholipid_binding	1
_02	int8:chr7:22714810..22804810	chr7:22455294..22753932
460	ASTN1 A	GO:0005575 Cellular_component	0.030622
_02	int20:chrM:7119859..7845353	chrM:7264614..7295832
605	BCL7A B	GO:0005575 Cellular_component	0.030622
_02	int2:chr5:29189236..29284352	chr5:29187858..29196161
4316	MMP7 M	GO:0005578 Proteinaceous_extracellular_matrix
0.011648
_02	int10:chr8:63223904..63813192	chr8:63386661..63400389
5265	SERPINA1 S	GO:0005578
Proteinaceous_extracellular_matrix	0.011648
_02	int2:chr5:29189236..29284352	chr5:29187858..29196161

4316 MMP7 M GO:0005615 Extracellular_space 0.003443
_O2 int10:chr8:63223904..63813192 chr8:63324149..63341782
866 SERPINA6 S GO:0005615 Extracellular_space 0.003443
_O2 int10:chr8:63223904..63813192 chr8:63386661..63400389
5265 SERPINA1 S GO:0005615 Extracellular_space 0.003443
_O2 int10:chr8:63223904..63813192 chr8:63552073..63560608
5267 SERPINA4 S GO:0005615 Extracellular_space 0.003443
_O2 int20:chrM:7119859..7845353 chrM:7173150..7175594
386653 IL31 I GO:0005615 Extracellular_space 0.003443
_O2 int5:chr7:16160915..16235033 chr7:16233741..16292928
1660 DHX9 D GO:0005654 Nucleoplasm 0.002715
_O2 int16:chrX:24666408..24763994 chrX:24599559..24692559
6304 SATB1 S GO:0005654 Nucleoplasm 0.002715
_O2 int20:chrM:7119859..7845353 chrM:7794400..7841032
51433 ANAPC5 A GO:0005654 Nucleoplasm 0.002715
_O2 int9:chr8:16013494..16106285 chr8:16077537..16083232
3169 FOXA1 F GO:0005667 Transcription_factor_complex
0.000119
_O2 int10:chr8:63223904..63813192 chr8:63705038..63706981
145258 GSC G GO:0005667 Transcription_factor_complex 0.000119
_O2 int11:chr10:69076487..69161721 chr10:69148923..69162939
344018 FIGLA F GO:0005667 Transcription_factor_complex
0.000119
_O2 int20:chrM:7119859..7845353 chrM:7794400..7841032
51433 ANAPC5 A GO:0005680 Anaphase-promoting_complex
1
_O2 int20:chrM:7119859..7845353 chrM:7443827..7467087
23067 SETD1B S GO:0005694 Chromosome 1
_O2 int16:chrX:24666408..24763994 chrX:24599559..24692559
6304 SATB1 S GO:0005720 Nuclear_heterochromatin
1
_O2 int3:chr6:18012525..18106914 chr6:18010887..18029186
9344 TAOK2 T GO:0005730 Nucleolus 0.001055
_O2 int3:chr6:18012525..18106914 chr6:18098760..18102068
83719 YPEL3 Y GO:0005730 Nucleolus 0.001055
_O2 int5:chr7:16160915..16235033 chr7:16233741..16292928
1660 DHX9 D GO:0005730 Nucleolus 0.001055
_O2 int13:chr16:45965023..46373321 chr16:46053149..46131685
3660 IRF2 I GO:0005730 Nucleolus 0.001055
_O2 int20:chrM:7119859..7845353 chrM:7443827..7467087
23067 SETD1B S GO:0005730 Nucleolus 0.001055
_O2 int20:chrM:7119859..7845353 chrM:7625403..7746298
84678 KDM2B L GO:0005730 Nucleolus 0.001055
_O2 int20:chrM:7119859..7845353 chrM:7123780..7144993
56616 DIABLO D GO:0005739 Mitochondrion 1
_O2 int20:chrM:7119859..7845353 chrM:7123780..7144993
56616 DIABLO D GO:0005758
Mitochondrial_intermembrane_space 1
_O2 int3:chr6:18012525..18106914 chr6:18041311..18046793
8448 DOC2A D GO:0005764 Lysosome 1
_O2 int3:chr6:18012525..18106914 chr6:18053839..18057610
83723 FAM57B F GO:0005783 Endoplasmic_reticulum
0.0593539
_O2 int7:chr7:19633367..19714459 chr7:19668098..19674935

5743	PTGS2 P		GO:0005783 Endoplasmic_reticulum
0.0593539
_O2	int7:chr7:19633367..19714459	chr7:19668098..19674935
5743	PTGS2 P		GO:0005788 Endoplasmic_reticulum_lumen
1
_O2	int7:chr7:19633367..19714459	chr7:19668098..19674935
5743	PTGS2 P		GO:0005789 Endoplasmic_reticulum_membrane
1
_O2	int7:chr7:19633367..19714459	chr7:19668098..19674935
5743	PTGS2 P		GO:0005792 Microsome	1
_O2	int20:chrM:7119859..7845353	chrM:7145120..7148771
79369	B3GNT4 U	GO:0005794 Golgi_apparatus	1
_O2	int3:chr6:18012525..18106914	chr6:18085139..18092858
5531	PPP4C P		GO:0005813 Centrosome	0.00338
_O2	int5:chr7:16160915..16235033	chr7:16233741..16292928
1660	DHX9 D	GO:0005813 Centrosome	0.00338
_O2	int3:chr6:18012525..18106914	chr6:18010887..18029186
9344	TAOK2 T		GO:0005856 Cytoskeleton	0.00865799
_O2	int3:chr6:18012525..18106914	chr6:18085139..18092858
5531	PPP4C P		GO:0005856 Cytoskeleton	0.00865799
_O2	int11:chr10:69076487..69161721	chr10:69056997..69142988
119	ADD2 A	GO:0005856 Cytoskeleton	0.00865799
_O2	int20:chrM:7119859..7845353	chrM:7478035..7489428
54509	RHOF R	GO:0005856 Cytoskeleton	0.00865799
_O2	int0:chr4:31133158..31204643	chr4:30515861..31557604
10718	NRG3 N	GO:0005887 Integral_to_plasma_membrane	0.111196
_O2	int20:chrM:7119859..7845353	chrM:7584448..7599633
84876	ORAI1 O		GO:0005887 Integral_to_plasma_membrane
0.111196
_O2	int7:chr7:19633367..19714459	chr7:19668098..19674935
5743	PTGS2 P		GO:0005901 Caveola	1
_O2	int13:chr16:45965023..46373321	chr16:46053149..46131685
3660	IRF2 I	GO:0005925 Focal_adhesion	1
_O2	int3:chr6:18012525..18106914	chr6:18069904..18083053
226	ALDOA A		GO:0005975 Carbohydrate_metabolic_process
0.004555
_O2	int5:chr7:16160915..16235033	chr7:16190066..16223809
80896	NPL N	GO:0005975 Carbohydrate_metabolic_process 0.004555
_O2	int3:chr6:18012525..18106914	chr6:18069904..18083053
226	ALDOA A		GO:0006000 Fructose_metabolic_process
1
_O2	int3:chr6:18012525..18106914	chr6:18069904..18083053
226	ALDOA A		GO:0006006 Glucose_metabolic_process
0.000428
_O2	int10:chr8:63223904..63813192	chr8:63497827..63521295
145264	SERPINA12 S	GO:0006006 Glucose_metabolic_process
0.000428
_O2	int3:chr6:18012525..18106914	chr6:18069904..18083053
226	ALDOA A		GO:0006094 Gluconeogenesis	1
_O2	int3:chr6:18012525..18106914	chr6:18069904..18083053
226	ALDOA A		GO:0006096 Glycolysis	1
_O2	int16:chrX:24666408..24763994	chrX:24599559..24692559
6304	SATB1 S		GO:0006325 Chromatin_organization
1

_02	int3:chr6:18012525..18106914	chr6:18030062..18033191
8479	HIRIP3 H	GO:0006333
Chromatin_assembly_or_disassembly	1
_02	int9:chr8:16013494..16106285	chr8:16077537..16083232
3169	FOXA1 F	GO:0006338 Chromatin_remodeling	1
_02	int3:chr6:18012525..18106914	chr6:18093188..18098314
6911	TBX6 T	GO:0006355 Regulation_of_transcription,_DNA-
dependent	1.6e-05
_02	int10:chr8:63223904..63813192	chr8:63705038..63706981
145258	GSC G	GO:0006355 Regulation_of_transcription,_DNA-
dependent	1.6e-05
_02	int13:chr16:45965023..46373321	chr16:46053149..46131685
3660	IRF2 I	GO:0006355 Regulation_of_transcription,_DNA-
dependent	1.6e-05
_02	int16:chrX:24666408..24763994	chrX:24599559..24692559
6304	SATB1 S	GO:0006355
Regulation_of_transcription,_DNA-dependent	1.6e-05
_02	int20:chrM:7119859..7845353	chrM:7223951..7225043
652963	BTF3P12 B	GO:0006355
Regulation_of_transcription,_DNA-dependent	1.6e-05
_02	int20:chrM:7119859..7845353	chrM:7145120..7148771
79369	B3GNT4 U	GO:0006486 Protein_glycosylation	1
_02	int2:chr5:29189236..29284352	chr5:29187858..29196161
4316	MMP7 M	GO:0006508 Proteolysis	0.016238
_02	int8:chr7:22714810..22804810	chr7:22772438..23143385
60676	PAPPA2 P	GO:0006508 Proteolysis	0.016238
_02	int20:chrM:7119859..7845353	chrM:7794400..7841032
51433	ANAPC5 A	GO:0006511 Ubiquitin-
dependent_protein_catabolic_process	1
_02	int3:chr6:18012525..18106914	chr6:18010887..18029186
9344	TAOK2 T	GO:0006612 Protein_targeting_to_membrane
1
_02	int7:chr7:19633367..19714459	chr7:19668098..19674935
5743	PTGS2 P	GO:0006633 Fatty_acid_biosynthetic_process
	1
_02	int7:chr7:19633367..19714459	chr7:19668098..19674935
5743	PTGS2 P	GO:0006692 Prostanoid_metabolic_process
1
_02	int7:chr7:19633367..19714459	chr7:19668098..19674935
5743	PTGS2 P	GO:0006693 Prostaglandin_metabolic_process
	1
_02	int3:chr6:18012525..18106914	chr6:18069904..18083053
226	ALDOA A	GO:0006754 ATP_biosynthetic_process
1
_02	int7:chr7:19633367..19714459	chr7:19668098..19674935
5743	PTGS2 P	GO:0006805 Xenobiotic_metabolic_process
1
_02	int10:chr8:63223904..63813192	chr8:63324149..63341782
866	SERPINA6 S	GO:0006810 Transport	1
_02	int11:chr10:69076487..69161721	chr10:69056997..69142988
119	ADD2 A	GO:0006811 Ion_transport	0.0635899
_02	int20:chrM:7119859..7845353	chrM:7584448..7599633
84876	ORAI1 O	GO:0006811 Ion_transport	0.0635899
_02	int3:chr6:18012525..18106914	chr6:18010887..18029186

9344	TAOK2 T		GO:0006915 Apoptosis	0.002442
_02	int16:chrX:24666408..24763994	chrX:24599559..24692559
6304	SATB1 S		GO:0006915 Apoptosis	0.002442
_02	int20:chrM:7119859..7845353	chrM:7123780..7144993
56616	DIABLO D	GO:0006915 Apoptosis	0.002442
_02	int20:chrM:7119859..7845353	chrM:7749375..7767935
80196	RNF34 R		GO:0006915 Apoptosis	0.002442
_02	int20:chrM:7119859..7845353	chrM:7123780..7144993
56616	DIABLO D	GO:0006917 Induction_of_apoptosis
1
_02	int20:chrM:7119859..7845353	chrM:7123780..7144993
56616	DIABLO D	GO:0006919 Activation_of_caspase_activity
1
_02	int16:chrX:24666408..24763994	chrX:24599559..24692559
6304	SATB1 S		GO:0006921
Cellular_component_disassembly_involved_in_apoptosis	1
_02	int7:chr7:19633367..19714459	chr7:19668098..19674935
5743	PTGS2 P		GO:0006928 Cellular_component_movement
1
_02	int3:chr6:18012525..18106914	chr6:18069904..18083053
226	ALDOA A		GO:0006941 Striated_muscle_contraction
1
_02	int3:chr6:18012525..18106914	chr6:18010887..18029186
9344	TAOK2 T		GO:0006950 Response_to_stress	1
_02	int10:chr8:63223904..63813192	chr8:63386661..63400389
5265	SERPINA1 S	GO:0006953 Acute-phase_response	1
_02	int10:chr8:63223904..63813192	chr8:63588248..63596955
12	SERPINA3 S	GO:0006953 Acute-phase_response	1
_02	int10:chr8:63223904..63813192	chr8:63588248..63596955
12	SERPINA3 S	GO:0006954 Inflammatory_response	1
_02	int7:chr7:19633367..19714459	chr7:19668098..19674935
5743	PTGS2 P		GO:0006979 Response_to_oxidative_stress
1
_02	int3:chr6:18012525..18106914	chr6:18069904..18083053
226	ALDOA A		GO:0007015 Actin_filament_organization
8.39999e-05
_02	int20:chrM:7119859..7845353	chrM:7478035..7489428
54509	RHOF R	GO:0007015 Actin_filament_organization
8.39999e-05
_02	int20:chrM:7119859..7845353	chrM:7794400..7841032
51433	ANAPC5 A	GO:0007094
Mitotic_cell_cycle_spindle_assembly_checkpoint	1
_02	int12:chr14:42191677..42265654	chr14:42217642..42513368
1124	CHN2 C	GO:0007165 Signal_transduction	1
_02	int0:chr4:31133158..31204643	chr4:30515861..31557604
10718	NRG3 N	GO:0007243 Intracellular_protein_kinase_cascade
1
_02	int12:chr14:42191677..42265654	chr14:42217642..42513368
1124	CHN2 C	GO:0007264
Small_GTPase_mediated_signal_transduction	0.013636
_02	int20:chrM:7119859..7845353	chrM:7478035..7489428
54509	RHOF R	GO:0007264
Small_GTPase_mediated_signal_transduction	0.013636
_02	int3:chr6:18012525..18106914	chr6:18041311..18046793

8448	DOC2A D		GO:0007268 Synaptic_transmission	1
_O2	int3:chr6:18012525..18106914	chr6:18093188..18098314
6911	TBX6 T	GO:0007275 Multicellular_organismal_development
0.012557
_O2	int10:chr8:63223904..63813192	chr8:63705038..63706981
145258	GSC G	GO:0007275 Multicellular_organismal_development
0.012557
_O2	int11:chr10:69076487..69161721	chr10:69148923..69162939
344018	FIGLA F		GO:0007275
Multicellular_organismal_development	0.012557
_O2	int10:chr8:63223904..63813192	chr8:63567114..63577016
5104	SERPINA5 S	GO:0007283 Spermatogenesis	1
_O2	int10:chr8:63223904..63813192	chr8:63567114..63577016
5104	SERPINA5 S	GO:0007342
Fusion_of_sperm_to_egg_plasma_membrane	1
_O2	int10:chr8:63223904..63813192	chr8:63705038..63706981
145258	GSC G	GO:0007369 Gastrulation	1
_O2	int0:chr4:31133158..31204643	chr4:30515861..31557604
10718	NRG3 N	GO:0007389 Pattern_specification_process	0.001235
_O2	int9:chr8:16013494..16106285	chr8:16077537..16083232
3169	FOXA1 F		GO:0007389 Pattern_specification_process
0.001235
_O2	int3:chr6:18012525..18106914	chr6:18041311..18046793
8448	DOC2A D		GO:0007399 Nervous_system_development
1
_O2	int3:chr6:18012525..18106914	chr6:18093188..18098314
6911	TBX6 T	GO:0007498 Mesoderm_development	1
_O2	int7:chr7:19633367..19714459	chr7:19668098..19674935
5743	PTGS2 P		GO:0007566 Embryo_implantation	1
_O2	int3:chr6:18012525..18106914	chr6:18069904..18083053
226	ALDOA A		GO:0007596 Blood_coagulation	0.00111
_O2	int10:chr8:63223904..63813192	chr8:63386661..63400389
5265	SERPINA1 S	GO:0007596 Blood_coagulation	0.00111
_O2	int20:chrM:7119859..7845353	chrM:7584448..7599633
84876	ORAI1 O		GO:0007596 Blood_coagulation	0.00111
_O2	int7:chr7:19633367..19714459	chr7:19668098..19674935
5743	PTGS2 P		GO:0007613 Memory	1
_O2	int8:chr7:22714810..22804810	chr7:22455294..22753932
460	ASTN1 A		GO:0007626 Locomotory_behavior	1
_O2	int18:chrX:48440818..48536955	chrX:48407005..48456332
170506	DHX36 D		GO:0008026 ATP-dependent_helicase_activity
	1
_O2	int0:chr4:31133158..31204643	chr4:30515861..31557604
10718	NRG3 N	GO:0008083 Growth_factor_activity	1
_O2	int3:chr6:18012525..18106914	chr6:18069904..18083053
226	ALDOA A		GO:0008092 Cytoskeletal_protein_binding
1
_O2	int9:chr8:16013494..16106285	chr8:16077537..16083232
3169	FOXA1 F		GO:0008134 Transcription_factor_binding
0.00613999
_O2	int11:chr10:69076487..69161721	chr10:69148923..69162939
344018	FIGLA F		GO:0008134 Transcription_factor_binding
0.00613999
_O2	int20:chrM:7119859..7845353	chrM:7625403..7746298

84678 KDM2B L GO:0008150 Biological_process 1
_O2 int2:chr5:29189236..29284352 chr5:29187858..29196161
4316 MMP7 M GO:0008152 Metabolic_process 1
_O2 int20:chrM:7119859..7845353 chrM:7443827..7467087
23067 SETD1B S GO:0008168 Methyltransferase_activity
1
_O2 int10:chr8:63223904..63813192 chr8:63567114..63577016
5104 SERPINA5 S GO:0008201 Heparin_binding 1
_O2 int7:chr7:19633367..19714459 chr7:19668098..19674935
5743 PTGS2 P GO:0008217 Regulation_of_blood_pressure
1
_O2 int2:chr5:29189236..29284352 chr5:29187858..29196161
4316 MMP7 M GO:0008233 Peptidase_activity 0.019572
_O2 int8:chr7:22714810..22804810 chr7:22772438..23143385
60676 PAPPA2 P GO:0008233 Peptidase_activity 0.019572
_O2 int8:chr7:22714810..22804810 chr7:22772438..23143385
60676 PAPPA2 P GO:0008237 Metallopeptidase_activity
1
_O2 int13:chr16:45965023..46373321 chr16:46053149..46131685
3660 IRF2 I GO:0008283 Cell_proliferation 1
_O2 int7:chr7:19633367..19714459 chr7:19668098..19674935
5743 PTGS2 P GO:0008285
Negative_regulation_of_cell_proliferation 1
_O2 int10:chr8:63223904..63813192 chr8:63202560..63300353
57718 PPP4R4 P GO:0008287 Protein_serine/
threonine_phosphatase_complex 1
_O2 int7:chr7:19633367..19714459 chr7:19668098..19674935
5743 PTGS2 P GO:0008289 Lipid_binding 1
_O2 int11:chr10:69076487..69161721 chr10:69056997..69142988
119 ADD2 A GO:0008290 F-actin_capping_protein_complex
1
_O2 int9:chr8:16013494..16106285 chr8:16077537..16083232
3169 FOXA1 F GO:0008301 DNA_bending_activity 1
_O2 int3:chr6:18012525..18106914 chr6:18010887..18029186
9344 TAOK2 T GO:0008360 Regulation_of_cell_shape
1
_O2 int3:chr6:18012525..18106914 chr6:18069904..18083053
226 ALDOA A GO:0008360 Regulation_of_cell_shape
1
_O2 int20:chrM:7119859..7845353 chrM:7145120..7148771
79369 B3GNT4 U GO:0008378 Galactosyltransferase_activity
1
_O2 int5:chr7:16160915..16235033 chr7:16233741..16292928
1660 DHX9 D GO:0008380 RNA_splicing 1
_O2 int20:chrM:7119859..7845353 chrM:7123780..7144993
56616 DIABLO D GO:0008625
Induction_of_apoptosis_via_death_domain_receptors 1
_O2 int20:chrM:7119859..7845353 chrM:7123780..7144993
56616 DIABLO D GO:0008629
Induction_of_apoptosis_by_intracellular_signals 1
_O2 int20:chrM:7119859..7845353 chrM:7123780..7144993
56616 DIABLO D GO:0008631
Induction_of_apoptosis_by_oxidative_stress 1
_O2 int9:chr8:16013494..16106285 chr8:16077537..16083232

3169   FOXA1 F   GO:0008634
Negative_regulation_of_survival_gene_product_expression   1
_O2   int20:chrM:7119859..7845353   chrM:7123780..7144993
56616   DIABLO D   GO:0008635
Activation_of_caspase_activity_by_cytochrome_c   1
_O2   int13:chr16:45965023..46373321   chr16:46247401..46358457
133121 ENPP6 E   GO:0008889
Glycerophosphodiester_phosphodiesterase_activity 1
_O2   int3:chr6:18012525..18106914   chr6:18093188..18098314
6911   TBX6 T GO:0009653 Anatomical_structure_morphogenesis
0.000601999
_O2   int10:chr8:63223904..63813192   chr8:63705038..63706981
145258 GSC G   GO:0009653 Anatomical_structure_morphogenesis
0.000601999
_O2   int7:chr7:19633367..19714459   chr7:19668098..19674935
5743   PTGS2 P   GO:0009750 Response_to_fructose_stimulus
1
_O2   int9:chr8:16013494..16106285   chr8:16077537..16083232
3169   FOXA1 F   GO:0009790 Embryo_development   1
_O2   int7:chr7:19633367..19714459   chr7:19668098..19674935
5743   PTGS2 P   GO:0010243 Response_to_organic_nitrogen
1
_O2   int10:chr8:63223904..63813192   chr8:63386661..63400389
5265   SERPINA1 S   GO:0010288 Response_to_lead_ion   1
_O2   int5:chr7:16160915..16235033   chr7:16233741..16292928
1660   DHX9 D GO:0010467 Gene_expression   1
_O2   int9:chr8:16013494..16106285   chr8:16077537..16083232
3169   FOXA1 F   GO:0010551 Regulation_of_gene-
specific_transcription_from_RNA_polymerase_II_promoter   1
_O2   int9:chr8:16013494..16106285   chr8:16077537..16083232
3169   FOXA1 F   GO:0010552 Positive_regulation_of_gene-
specific_transcription_from_RNA_polymerase_II_promoter   1
_O2   int7:chr7:19633367..19714459   chr7:19668098..19674935
5743   PTGS2 P   GO:0010575
Positive_regulation_vascular_endothelial_growth_factor_production
1
_O2   int9:chr8:16013494..16106285   chr8:16077537..16083232
3169   FOXA1 F   GO:0010843 Promoter_binding   1
_O2   int20:chrM:7119859..7845353   chrM:7749375..7767935
80196   RNF34 R   GO:0012505 Endomembrane_system   1
_O2   int7:chr7:19633367..19714459   chr7:19668098..19674935
5743   PTGS2 P   GO:0014070
Response_to_organic_cyclic_substance   0.000727999
_O2   int10:chr8:63223904..63813192   chr8:63386661..63400389
5265   SERPINA1 S   GO:0014070
Response_to_organic_cyclic_substance   0.000727999
_O2   int3:chr6:18012525..18106914   chr6:18069904..18083053
226   ALDOA A   GO:0015629 Actin_cytoskeleton   1
_O2   int3:chr6:18012525..18106914   chr6:18069904..18083053
226   ALDOA A   GO:0015631 Tubulin_binding   1
_O2   int3:chr6:18012525..18106914   chr6:18010887..18029186
9344   TAOK2 T   GO:0016023 Cytoplasmic_membrane-
bounded_vesicle 1
_O2   int13:chr16:45965023..46373321   chr16:46247401..46358457

133121 ENPP6 E GO:0016042 Lipid_catabolic_process
1
_O2 int3:chr6:18012525..18106914 chr6:18041311..18046793
8448 DOC2A D GO:0016079 Synaptic_vesicle_exocytosis
1
_O2 int16:chrX:24666408..24763994 chrX:24599559..24692559
6304 SATB1 S GO:0016363 Nuclear_matrix 1
_O2 int3:chr6:18012525..18106914 chr6:18010887..18029186
9344 TAOK2 T GO:0016477 Cell_migration 0.001313
_O2 int8:chr7:22714810..22804810 chr7:22455294..22753932
460 ASTN1 A GO:0016477 Cell_migration 0.001313
_O2 int20:chrM:7119859..7845353 chrM:7264614..7295832
605 BCL7A B GO:0016481
Negative_regulation_of_transcription 1
_O2 int7:chr7:19633367..19714459 chr7:19668098..19674935
5743 PTGS2 P GO:0016491 Oxidoreductase_activity
0.011199
_O2 int20:chrM:7119859..7845353 chrM:7625403..7746298
84678 KDM2B L GO:0016491 Oxidoreductase_activity
0.011199
_O2 int9:chr8:16013494..16106285 chr8:16077537..16083232
3169 FOXA1 F GO:0016563
Transcription_activator_activity 1
_O2 int16:chrX:24666408..24763994 chrX:24599559..24692559
6304 SATB1 S GO:0016564
Transcription_repressor_activity 1
_O2 int9:chr8:16013494..16106285 chr8:16077537..16083232
3169 FOXA1 F GO:0016566
Specific_transcriptional_repressor_activity 1
_O2 int16:chrX:24666408..24763994 chrX:24599559..24692559
6304 SATB1 S GO:0016568 Chromatin_modification
0.00593299
_O2 int20:chrM:7119859..7845353 chrM:7443827..7467087
23067 SETD1B S GO:0016568 Chromatin_modification
0.00593299
_O2 int20:chrM:7119859..7845353 chrM:7625403..7746298
84678 KDM2B L GO:0016568 Chromatin_modification
0.00593299
_O2 int16:chrX:24666408..24763994 chrX:24599559..24692559
6304 SATB1 S GO:0016571 Histone_methylation 1
_O2 int16:chrX:24666408..24763994 chrX:24599559..24692559
6304 SATB1 S GO:0016605 PML_body 1
_O2 int20:chrM:7119859..7845353 chrM:7443827..7467087
23067 SETD1B S GO:0016607 Nuclear_speck 1
_O2 int20:chrM:7119859..7845353 chrM:7749375..7767935
80196 RNF34 R GO:0016607 Nuclear_speck 1
_O2 int7:chr7:19633367..19714459 chr7:19668098..19674935
5743 PTGS2 P GO:0016702
Oxidoreductase_activity,_acting_on_single_donors_with_incorporation_
of_molecular_oxygen,_incorporation_of_two_atoms_of_oxygen 0.000314
_O2 int20:chrM:7119859..7845353 chrM:7625403..7746298
84678 KDM2B L GO:0016702
Oxidoreductase_activity,_acting_on_single_donors_with_incorporation_
of_molecular_oxygen,_incorporation_of_two_atoms_of_oxygen 0.000314

_02	int3:chr6:18012525..18106914	chr6:18010887..18029186
9344	TAOK2 T		GO:0016740 Transferase_activity	0.1261
_02	int20:chrM:7119859..7845353	chrM:7443827..7467087
23067	SETD1B S	GO:0016740 Transferase_activity	0.1261
_02	int20:chrM:7119859..7845353	chrM:7145120..7148771
79369	B3GNT4 U	GO:0016757
Transferase_activity,_transferring_glycosyl_groups	1
_02	int3:chr6:18012525..18106914	chr6:18085139..18092858
5531	PPP4C P		GO:0016787 Hydrolase_activity	0.001131
_02	int5:chr7:16160915..16235033	chr7:16233741..16292928
1660	DHX9 D	GO:0016787 Hydrolase_activity	0.001131
_02	int13:chr16:45965023..46373321	chr16:46247401..46358457
133121	ENPP6 E		GO:0016787 Hydrolase_activity	0.001131
_02	int18:chrX:48440818..48536955	chrX:48407005..48456332
170506	DHX36 D		GO:0016787 Hydrolase_activity	0.001131
_02	int3:chr6:18012525..18106914	chr6:18069904..18083053
226	ALDOA A		GO:0016829 Lyase_activity	0.000477
_02	int5:chr7:16160915..16235033	chr7:16190066..16223809
80896	NPL N	GO:0016829 Lyase_activity	0.000477
_02	int20:chrM:7119859..7845353	chrM:7749375..7767935
80196	RNF34 R		GO:0016874 Ligase_activity	1
_02	int3:chr6:18012525..18106914	chr6:18041311..18046793
8448	DOC2A D		GO:0017158 Regulation_of_calcium_ion-
dependent_exocytosis	1
_02	int10:chr8:63223904..63813192	chr8:63588248..63596955
12	SERPINA3 S	GO:0019216
Regulation_of_lipid_metabolic_process	1
_02	int3:chr6:18012525..18106914	chr6:18041311..18046793
8448	DOC2A D		GO:0019717 Synaptosome	1
_02	int20:chrM:7119859..7845353	chrM:7625403..7746298
84678	KDM2B L		GO:0019843 RRNA_binding	1
_02	int7:chr7:19633367..19714459	chr7:19668098..19674935
5743	PTGS2 P		GO:0019899 Enzyme_binding	1
_02	int9:chr8:16013494..16106285	chr8:16077537..16083232
3169	FOXA1 F		GO:0019904 Protein_domain_specific_binding
	1
_02	int7:chr7:19633367..19714459	chr7:19668098..19674935
5743	PTGS2 P		GO:0020037 Heme_binding	1
_02	int10:chr8:63223904..63813192	chr8:63705038..63706981
145258	GSC G	GO:0021904 Dorsal/ventral_neural_tube_patterning
1
_02	int3:chr6:18012525..18106914	chr6:18010887..18029186
9344	TAOK2 T		GO:0030036 Actin_cytoskeleton_organization
	0.00546999
_02	int11:chr10:69076487..69161721	chr10:69056997..69142988
119	ADD2 A	GO:0030036 Actin_cytoskeleton_organization
0.00546999
_02	int3:chr6:18012525..18106914	chr6:18041311..18046793
8448	DOC2A D		GO:0030054 Cell_junction 0.0549819
_02	int19:chrY:41275569..41346277	chrY:41314209..41321352
140689	CBLN4 C		GO:0030054 Cell_junction 0.0549819
_02	int1:chr5:6499318..8170565	chr5:7583475..8124265
84623	KIRREL3 K	GO:0030097 Hemopoiesis	0.000805999
_02	int11:chr10:69076487..69161721	chr10:69056997..69142988

119	ADD2 A	GO:0030097 Hemopoiesis	0.000805999
_O2	int8:chr7:22714810..22804810	chr7:22772438..23143385
60676	PAPPA2 P	GO:0030154 Cell_differentiation	0.030068
_O2	int11:chr10:69076487..69161721	chr10:69148923..69162939
344018	FIGLA F	GO:0030154 Cell_differentiation	0.030068
_O2	int3:chr6:18012525..18106914	chr6:18069904..18083053
226	ALDOA A	GO:0030168 Platelet_activation
0.000547999
_O2	int10:chr8:63223904..63813192	chr8:63386661..63400389
5265	SERPINA1 S	GO:0030168 Platelet_activation
0.000547999
_O2	int20:chrM:7119859..7845353	chrM:7584448..7599633
84876	ORAI1 O	GO:0030168 Platelet_activation
0.000547999
_O2	int10:chr8:63223904..63813192	chr8:63705038..63706981
145258	GSC G	GO:0030178
Negative_regulation_of_Wnt_receptor_signaling_pathway	1
_O2	int7:chr7:19633367..19714459	chr7:19668098..19674935
5743	PTGS2 P	GO:0030282 Bone_mineralization	1
_O2	int9:chr8:16013494..16106285	chr8:16077537..16083232
3169	FOXA1 F	GO:0030324 Lung_development	1
_O2	int10:chr8:63223904..63813192	chr8:63379422..63381819
390502	SERPINA2 S	GO:0030414 Peptidase_inhibitor_activity
1
_O2	int10:chr8:63223904..63813192	chr8:63386661..63400389
5265	SERPINA1 S	GO:0030414 Peptidase_inhibitor_activity
1
_O2	int10:chr8:63223904..63813192	chr8:63436708..63453265
256394	SERPINA11 S	GO:0030414 Peptidase_inhibitor_activity
1
_O2	int10:chr8:63223904..63813192	chr8:63473300..63488592
327657	SERPINA9 S	GO:0030414 Peptidase_inhibitor_activity
1
_O2	int10:chr8:63223904..63813192	chr8:63497827..63521295
145264	SERPINA12 S	GO:0030414 Peptidase_inhibitor_activity
1
_O2	int10:chr8:63223904..63813192	chr8:63552073..63560608
5267	SERPINA4 S	GO:0030414 Peptidase_inhibitor_activity
1
_O2	int10:chr8:63223904..63813192	chr8:63567114..63577016
5104	SERPINA5 S	GO:0030414 Peptidase_inhibitor_activity
1
_O2	int10:chr8:63223904..63813192	chr8:63588248..63596955
12	SERPINA3 S	GO:0030414 Peptidase_inhibitor_activity
1
_O2	int11:chr10:69076487..69161721	chr10:69056997..69142988
119	ADD2 A	GO:0030507 Spectrin_binding	1
_O2	int10:chr8:63223904..63813192	chr8:63705038..63706981
145258	GSC G	GO:0030528 Transcription_regulator_activity
0.004723
_O2	int11:chr10:69076487..69161721	chr10:69148923..69162939
344018	FIGLA F	GO:0030528
Transcription_regulator_activity	0.004723
_O2	int5:chr7:16160915..16235033	chr7:16233741..16292928

1660 DHX9 D GO:0030529 Ribonucleoprotein_complex 1
_02 int2:chr5:29189236..29284352 chr5:29187858..29196161
4316 MMP7 M GO:0030574 Collagen_catabolic_process 1
_02 int3:chr6:18012525..18106914 chr6:18010887..18029186
9344 TAOK2 T GO:0030659 Cytoplasmic_vesicle_membrane
1
_02 int3:chr6:18012525..18106914 chr6:18041311..18046793
8448 DOC2A D GO:0030672 Synaptic_vesicle_membrane
1
_02 int7:chr7:19633367..19714459 chr7:19668098..19674935
5743 PTGS2 P GO:0030728 Ovulation 1
_02 int0:chr4:31133158..31204643 chr4:30515861..31557604
10718 NRG3 N GO:0030879 Mammary_gland_development 1
_02 int10:chr8:63223904..63813192 chr8:63705038..63706981
145258 GSC G GO:0030900 Forebrain_development 1
_02 int0:chr4:31133158..31204643 chr4:30515861..31557604
10718 NRG3 N GO:0030971 Receptor_tyrosine_kinase_binding
1
_02 int3:chr6:18012525..18106914 chr6:18069904..18083053
226 ALDOA A GO:0031093 Platelet_alpha_granule_lumen
8.49999e-05
_02 int10:chr8:63223904..63813192 chr8:63386661..63400389
5265 SERPINA1 S GO:0031093 Platelet_alpha_granule_lumen
8.49999e-05
_02 int20:chrM:7119859..7845353 chrM:7794400..7841032
51433 ANAPC5 A GO:0031145 Anaphase-promoting_complex-dependent_proteasomal_ubiquitin-dependent_protein_catabolic_process
1
_02 int3:chr6:18012525..18106914 chr6:18041311..18046793
8448 DOC2A D GO:0031410 Cytoplasmic_vesicle 1
_02 int7:chr7:19633367..19714459 chr7:19668098..19674935
5743 PTGS2 P GO:0031622 Positive_regulation_of_fever
1
_02 int3:chr6:18012525..18106914 chr6:18069904..18083053
226 ALDOA A GO:0031674 I_band 1
_02 int11:chr10:69076487..69161721 chr10:69056997..69142988
119 ADD2 A GO:0032092 Positive_regulation_of_protein_binding
1
_02 int7:chr7:19633367..19714459 chr7:19668098..19674935
5743 PTGS2 P GO:0032355 Response_to_estradiol_stimulus
3e-06
_02 int9:chr8:16013494..16106285 chr8:16077537..16083232
3169 FOXA1 F GO:0032355 Response_to_estradiol_stimulus
3e-06
_02 int10:chr8:63223904..63813192 chr8:63386661..63400389
5265 SERPINA1 S GO:0032355 Response_to_estradiol_stimulus
3e-06
_02 int7:chr7:19633367..19714459 chr7:19668098..19674935
5743 PTGS2 P GO:0032496 Response_to_lipopolysaccharide
0.000601999
_02 int10:chr8:63223904..63813192 chr8:63386661..63400389
5265 SERPINA1 S GO:0032496 Response_to_lipopolysaccharide
0.000601999
_02 int7:chr7:19633367..19714459 chr7:19668098..19674935

5743	PTGS2 P	GO:0033280 Response_to_vitamin_D	1
_O2	int7:chr7:19633367..19714459	chr7:19668098..19674935
5743	PTGS2 P	GO:0034097 Response_to_cytokine_stimulus
0.000151
_O2	int10:chr8:63223904..63813192	chr8:63386661..63400389
5265	SERPINA1 S	GO:0034097 Response_to_cytokine_stimulus
0.000151
_O2	int5:chr7:16160915..16235033	chr7:16233741..16292928
1660	DHX9 D	GO:0034605 Cellular_response_to_heat	1
_O2	int20:chrM:7119859..7845353	chrM:7443827..7467087
23067	SETD1B S	GO:0035097
Histone_methyltransferase_complex	1
_O2	int16:chrX:24666408..24763994	chrX:24599559..24692559
6304	SATB1 S	GO:0042110 T_cell_activation	1
_O2	int2:chr5:29189236..29284352	chr5:29187858..29196161
4316	MMP7 M	GO:0042127 Regulation_of_cell_proliferation
1
_O2	int9:chr8:16013494..16106285	chr8:16077537..16083232
3169	FOXA1 F	GO:0042445 Hormone_metabolic_process
1
_O2	int7:chr7:19633367..19714459	chr7:19668098..19674935
5743	PTGS2 P	GO:0042446 Hormone_biosynthetic_process
1
_O2	int10:chr8:63223904..63813192	chr8:63705038..63706981
145258	GSC G	GO:0042474 Middle_ear_morphogenesis	1
_O2	int7:chr7:19633367..19714459	chr7:19668098..19674935
5743	PTGS2 P	GO:0042493 Response_to_drug	1
_O2	int9:chr8:16013494..16106285	chr8:16077537..16083232
3169	FOXA1 F	GO:0042593 Glucose_homeostasis	1
_O2	int7:chr7:19633367..19714459	chr7:19668098..19674935
5743	PTGS2 P	GO:0042640 Anagen	1
_O2	int20:chrM:7119859..7845353	chrM:7443827..7467087
23067	SETD1B S	GO:0042800
Histone_methyltransferase_activity_(H3-K4_specific)	1
_O2	int3:chr6:18012525..18106914	chr6:18069904..18083053
226	ALDOA A	GO:0042802 Identical_protein_binding
1
_O2	int11:chr10:69076487..69161721	chr10:69056997..69142988
119	ADD2 A	GO:0042803 Protein_homodimerization_activity
1
_O2	int7:chr7:19633367..19714459	chr7:19668098..19674935
5743	PTGS2 P	GO:0043065
Positive_regulation_of_apoptosis	1
_O2	int7:chr7:19633367..19714459	chr7:19668098..19674935
5743	PTGS2 P	GO:0043234 Protein_complex	0.00452
_O2	int10:chr8:63223904..63813192	chr8:63567114..63577016
5104	SERPINA5 S	GO:0043234 Protein_complex	0.00452
_O2	int10:chr8:63223904..63813192	chr8:63705038..63706981
145258	GSC G	GO:0043565 Sequence-specific_DNA_binding	0.001001
_O2	int11:chr10:69076487..69161721	chr10:69148923..69162939
344018	FIGLA F	GO:0043565 Sequence-specific_DNA_binding
0.001001
_O2	int16:chrX:24666408..24763994	chrX:24599559..24692559
6304	SATB1 S	GO:0043565 Sequence-specific_DNA_binding

0.001001
_O2	int10:chr8:63223904..63813192	chr8:63705038..63706981
145258	GSC G	GO:0043583 Ear_development	1
_O2	int16:chrX:24666408..24763994	chrX:24599559..24692559
6304	SATB1 S		GO:0044419
Interspecies_interaction_between_organisms	1
_O2	int3:chr6:18012525..18106914	chr6:18041311..18046793
8448	DOC2A D		GO:0045202 Synapse	0.037092
_O2	int19:chrY:41275569..41346277	chrY:41314209..41321352
140689	CBLN4 C		GO:0045202 Synapse	0.037092
_O2	int7:chr7:19633367..19714459	chr7:19668098..19674935
5743	PTGS2 P		GO:0045429
Positive_regulation_of_nitric_oxide_biosynthetic_process	1
_O2	int13:chr16:45965023..46373321	chr16:46053149..46131685
3660	IRF2 I	GO:0045449 Regulation_of_transcription
0.0647219
_O2	int20:chrM:7119859..7845353	chrM:7443827..7467087
23067	SETD1B S		GO:0045449 Regulation_of_transcription
0.0647219
_O2	int20:chrM:7119859..7845353	chrM:7625403..7746298
84678	KDM2B L		GO:0045449 Regulation_of_transcription
0.0647219
_O2	int9:chr8:16013494..16106285	chr8:16077537..16083232
3169	FOXA1 F		GO:0045666
Positive_regulation_of_neuron_differentiation	1
_O2	int10:chr8:63223904..63813192	chr8:63567114..63577016
5104	SERPINA5 S	GO:0045861
Negative_regulation_of_proteolysis	1
_O2	int7:chr7:19633367..19714459	chr7:19668098..19674935
5743	PTGS2 P		GO:0045907
Positive_regulation_of_vasoconstriction	1
_O2	int9:chr8:16013494..16106285	chr8:16077537..16083232
3169	FOXA1 F		GO:0045931
Positive_regulation_of_mitotic_cell_cycle	1
_O2	int9:chr8:16013494..16106285	chr8:16077537..16083232
3169	FOXA1 F		GO:0045944
Positive_regulation_of_transcription_from_RNA_polymerase_II_promoter
	1
_O2	int7:chr7:19633367..19714459	chr7:19668098..19674935
5743	PTGS2 P		GO:0045987
Positive_regulation_of_smooth_muscle_contraction	1
_O2	int3:chr6:18012525..18106914	chr6:18010887..18029186
9344	TAOK2 T		GO:0046330
Positive_regulation_of_JNK_cascade	1
_O2	int7:chr7:19633367..19714459	chr7:19668098..19674935
5743	PTGS2 P		GO:0046697 Decidualization	1
_O2	int3:chr6:18012525..18106914	chr6:18069904..18083053
226	ALDOA A		GO:0046716 Muscle_cell_homeostasis
1
_O2	int11:chr10:69076487..69161721	chr10:69056997..69142988
119	ADD2 A	GO:0046982 Protein_heterodimerization_activity
1
_O2	int3:chr6:18012525..18106914	chr6:18010887..18029186
9344	TAOK2 T		GO:0048041 Focal_adhesion_assembly

1
_02	int20:chrM:7119859..7845353	chrM:7443827..7467087
23067	SETD1B S	GO:0048188 Set1C/COMPASS_complex	1
_02	int11:chr10:69076487..69161721	chr10:69148923..69162939
344018	FIGLA F	GO:0048477 Oogenesis	1
_02	int11:chr10:69076487..69161721	chr10:69148923..69162939
344018	FIGLA F	GO:0048599 Oocyte_development	1
_02	int10:chr8:63223904..63813192	chr8:63705038..63706981
145258	GSC G	GO:0048644 Muscle_organ_morphogenesis	1
_02	int7:chr7:19633367..19714459	chr7:19668098..19674935
5743	PTGS2 P	GO:0048661
Positive_regulation_of_smooth_muscle_cell_proliferation	1
_02	int9:chr8:16013494..16106285	chr8:16077537..16083232
3169	FOXA1 F	GO:0048665 Neuron_fate_specification
1
_02	int10:chr8:63223904..63813192	chr8:63705038..63706981
145258	GSC G	GO:0048704 Embryonic_skeletal_system_morphogenesis
1
_02	int9:chr8:16013494..16106285	chr8:16077537..16083232
3169	FOXA1 F	GO:0048754
Branching_morphogenesis_of_a_tube	1
_02	int7:chr7:19633367..19714459	chr7:19668098..19674935
5743	PTGS2 P	GO:0050727
Regulation_of_inflammatory_response	1
_02	int11:chr10:69076487..69161721	chr10:69056997..69142988
119	ADD2 A	GO:0051015 Actin_filament_binding	1
_02	int11:chr10:69076487..69161721	chr10:69056997..69142988
119	ADD2 A	GO:0051017 Actin_filament_bundle_assembly	1
_02	int12:chr14:42191677..42265654	chr14:42217642..42513368
1124	CHN2 C	GO:0051056
Regulation_of_small_GTPase_mediated_signal_transduction
0.00927499
_02	int20:chrM:7119859..7845353	chrM:7478035..7489428
54509	RHOF R	GO:0051056
Regulation_of_small_GTPase_mediated_signal_transduction
0.00927499
_02	int9:chr8:16013494..16106285	chr8:16077537..16083232
3169	FOXA1 F	GO:0051090
Regulation_of_transcription_factor_activity	1
_02	int9:chr8:16013494..16106285	chr8:16077537..16083232
3169	FOXA1 F	GO:0051091
Positive_regulation_of_transcription_factor_activity	1
_02	int20:chrM:7119859..7845353	chrM:7794400..7841032
51433	ANAPC5 A	GO:0051301 Cell_division	1
_02	int7:chr7:19633367..19714459	chr7:19668098..19674935
5743	PTGS2 P	GO:0051384
Response_to_glucocorticoid_stimulus	1
_02	int20:chrM:7119859..7845353	chrM:7794400..7841032
51433	ANAPC5 A	GO:0051436
Negative_regulation_of_ubiquitin-
protein_ligase_activity_involved_in_mitotic_cell_cycle	1
_02	int20:chrM:7119859..7845353	chrM:7794400..7841032
51433	ANAPC5 A	GO:0051437
Positive_regulation_of_ubiquitin-

protein_ligase_activity_involved_in_mitotic_cell_cycle 1
_02 int20:chrM:7119859..7845353 chrM:7794400..7841032
51433 ANAPC5 A GO:0051439 Regulation_of_ubiquitin-
protein_ligase_activity_involved_in_mitotic_cell_cycle 1
_02 int20:chrM:7119859..7845353 chrM:7443827..7467087
23067 SETD1B S GO:0051568 Histone_H3-K4_methylation
1
_02 int7:chr7:19633367..19714459 chr7:19668098..19674935
5743 PTGS2 P GO:0051726 Regulation_of_cell_cycle
1
_02 int20:chrM:7119859..7845353 chrM:7625403..7746298
84678 KDM2B L GO:0051864
Histone_demethylase_activity_(H3-K36_specific) 1
_02 int20:chrM:7119859..7845353 chrM:7584448..7599633
84876 ORAI1 O GO:0051924
Regulation_of_calcium_ion_transport 1
_02 int20:chrM:7119859..7845353 chrM:7584448..7599633
84876 ORAI1 O GO:0051928
Positive_regulation_of_calcium_ion_transport 1
_02 int7:chr7:19633367..19714459 chr7:19668098..19674935
5743 PTGS2 P GO:0051968
Positive_regulation_of_synaptic_transmission,_glutamatergic
1
_02 int7:chr7:19633367..19714459 chr7:19668098..19674935
5743 PTGS2 P GO:0055114 Oxidation-reduction_process
0.01498
_02 int20:chrM:7119859..7845353 chrM:7625403..7746298
84678 KDM2B L GO:0055114 Oxidation-reduction_process
0.01498
_02 int9:chr8:16013494..16106285 chr8:16077537..16083232
3169 FOXA1 F GO:0060441
Epithelial_tube_branching_involved_in_lung_morphogenesis 1
_02 int9:chr8:16013494..16106285 chr8:16077537..16083232
3169 FOXA1 F GO:0060740
Prostate_gland_epithelium_morphogenesis 1
_02 int5:chr7:16160915..16235033 chr7:16233741..16292928
1660 DHX9 D GO:0070934 CRD-mediated_mRNA_stabilization
1
_02 int5:chr7:16160915..16235033 chr7:16233741..16292928
1660 DHX9 D GO:0070937 CRD-mediated_mRNA_stability_complex
1
_02 int20:chrM:7119859..7845353 chrM:7794400..7841032
51433 ANAPC5 A GO:0070979 Protein_K11-
linked_ubiquitination 1

III. Interval Summary

_03 INTERVAL N_SNP N_ALTLOC N_GENE GENE_ID GENE_DESC
_03 int0:chr4:31133158..31204643 0 0 1
10718 NRG3 n
_03 int1:chr5:6499318..8170565 0 0 2

84623,100422918 KIRREL3,MIR3167 k ; m ;
_03 int1:chr5:6499318..8170565 0 0 2
399972,28960 FLJ39051,DCPS h ; d ;
_03 int2:chr5:29189236..29284352 0 0 1
4316 MMP7 m
_03 int3:chr6:18012525..18106914 0 0 8
9344 TAOK2 T
_03 int3:chr6:18012525..18106914 0 0 8
8479 HIRIP3 H
_03 int3:chr6:18012525..18106914 0 0 8
283899,8448 INO80E,DOC2A I ; d ;
_03 int3:chr6:18012525..18106914 0 0 8
146378,83723 C16orf92,FAM57B c ; f ;
_03 int3:chr6:18012525..18106914 0 0 8
226 ALDOA a
_03 int3:chr6:18012525..18106914 0 0 8
5531 PPP4C p
_03 int3:chr6:18012525..18106914 0 0 8
6911 TBX6 T
_03 int3:chr6:18012525..18106914 0 0 8
83719 YPEL3 y
_03 int5:chr7:16160915..16235033 0 0 2
80896 NPL N
_03 int5:chr7:16160915..16235033 0 0 2
1660 DHX9 D
_03 int6:chr7:17747493..17832876 0 0 1
81563 C1orf21 c
_03 int7:chr7:19633367..19714459 0 0 1
5743 PTGS2 p
_03 int8:chr7:22714810..22804810 0 0 2
460,574441 ASTN1,MIR488 a ; m ;
_03 int8:chr7:22714810..22804810 0 0 2
60676 PAPPA2 p
_03 int9:chr8:16013494..16106285 0 0 2
145282 MIPOL1 m
_03 int9:chr8:16013494..16106285 0 0 2
3169 FOXA1 f
_03 int10:chr8:63223904..63813192 0 0 11
57718 PPP4R4 p
_03 int10:chr8:63223904..63813192 0 0 11
866 SERPINA6 s
_03 int10:chr8:63223904..63813192 0 0 11
390502 SERPINA2 s
_03 int10:chr8:63223904..63813192 0 0 11
5265 SERPINA1 s
_03 int10:chr8:63223904..63813192 0 0 11
256394 SERPINA11 s
_03 int10:chr8:63223904..63813192 0 0 11
327657 SERPINA9 s
_03 int10:chr8:63223904..63813192 0 0 11
145264 SERPINA12 s
_03 int10:chr8:63223904..63813192 0 0 11
5267 SERPINA4 s
_03 int10:chr8:63223904..63813192 0 0 11

```
5104     SERPINA5 s
_03      int10:chr8:63223904..63813192     0        0        11
12       SERPINA3 s
_03      int10:chr8:63223904..63813192     0        0        11
145258   GSC g
_03      int11:chr10:69076487..69161721    0        0        2
119      ADD2 a
_03      int11:chr10:69076487..69161721    0        0        2
344018   FIGLA f
_03      int12:chr14:42191677..42265654    0        0        1
1124     CHN2 c
_03      int13:chr16:45965023..46373321    0        0        2
3660     IRF2 i
_03      int13:chr16:45965023..46373321    0        0        2
133121   ENPP6 e
_03      int14:chr21:45925954..46020103    0        0        1
338645   LUZP2 l
_03      int16:chrX:24666408..24763994     0        0        1
6304     SATB1 S
_03      int18:chrX:48440818..48536955     0        0        2
170506   DHX36 D
_03      int18:chrX:48440818..48536955     0        0        2
344758   GPR149 G
_03      int19:chrY:41275569..41346277     0        0        1
140689   CBLN4 c
_03      int20:chrM:7119859..7845353       0        0        13
56616    DIABLO d
_03      int20:chrM:7119859..7845353       0        0        13
79369    B3GNT4 U
_03      int20:chrM:7119859..7845353       0        0        13
386653   IL31 i
_03      int20:chrM:7119859..7845353       0        0        13
652963   BTF3P12 b
_03      int20:chrM:7119859..7845353       0        0        13
605      BCL7A B
_03      int20:chrM:7119859..7845353       0        0        13
144406   WDR66 W
_03      int20:chrM:7119859..7845353       0        0        13
23067    SETD1B S
_03      int20:chrM:7119859..7845353       0        0        13
338799   LOC338799 h
_03      int20:chrM:7119859..7845353       0        0        13
54509    RHOF r
_03      int20:chrM:7119859..7845353       0        0        13
84876    ORAI1 O
_03      int20:chrM:7119859..7845353       0        0        13
84678    KDM2B l
_03      int20:chrM:7119859..7845353       0        0        13
80196    RNF34 r
_03      int20:chrM:7119859..7845353       0        0        13
51433    ANAPC5 a
```
