# Supplementary material for: Genome-Wide Analyses Suggest Mechanisms Involving Early B-Cell Development in Canine IgA Deficiency
Source: PLoS One. 2015 Jul 30;10(7):e0133844. doi: 10.1371/journal.pone.0133844 (PMC4520476; doi:10.1371/journal.pone.0133844)
Supplement: S13 Table — (PDF) [file pone.0133844.s023.pdf]

Table S13. Descriptive statistics of IgA levels in four dog breeds

Breed	Number of ind.	IgA min	25% percentile	Median IgA	75% percentile	IgA max	Mean IgA (SD)	Lower 95% CI of the geometric mean
**GSD**	516	0.02	0.13	0.21	0.32	1.35	0.26 (0.19)	0.19
**GR**	162	0.03	0.1	0.18	0.33	1.12	0.26 (0.21)	0.16
**LR**	139	0.03	0.1	0.19	0.32	1.22	0.28 (0.23)	0.16
**SP**	96	0.01	0.04	0.07	0.14	0.56	0.10 (0.10)	0.06
