# Supplementary material for: Genome-Wide Analyses Suggest Mechanisms Involving Early B-Cell Development in Canine IgA Deficiency
Source: PLoS One. 2015 Jul 30;10(7):e0133844. doi: 10.1371/journal.pone.0133844 (PMC4520476; doi:10.1371/journal.pone.0133844)
Supplement: S14 Table — (PDF) [file pone.0133844.s024.pdf]

Table S14. IgA intervals (and number of indidivuals) used in GWAS, specified for each breed.

**Two percentile groups**	**GSD**	**GR**	**LR**	**SP**
group 1 < 25 % perc. (cases)	0.02-0.13 (133)	0.03-0.09 (56)	0.03-0.09 (41)	0.01-0.04 (29)
group 2 > 75 % perc. (controls)	0.33-1.35 (129)	0.34-1.12 (41)	0.33-1.22 (36)	0.14-0.56 (24)
**Three percentile groups**	**GSD**	**GR**	**LR**	**SP**
group 1 < 33,3 % perc.	0.02-0.15 (168)	0.03-0.12 (57)	0.03-0.11 (50)	0.01-0.05 (36)
group 2 33,3-66,7 % perc.	0.16-0.28 (168)	0.13-0.27 (56)	0.12-0.27 (46)	0.06-0.11 (30)
group 3 >66,7 % perc.	0.29-1.35 (160)	0.28-1.12 (56)	0.28-1.22 (45)	0.12-0.56 (28)
**Four percentile groups**	**GSD**	**GR**	**LR**	**SP**
group 1 < 25 % perc.	0.02-0.13 (133)	0.03-0.10 (56)	0.03-0.10 (41)	0.01-0.04 (29)
group 2 25-50 % perc.	0.14-0.21 (118)	0.11-0.18 (33)	0.11-0.19 (34)	0.05-0.07 (19)
group 3 50-75 % perc.	0.22-0.32 (124)	0.19-0.33 (39)	0.20-0.32 (34)	0.08-0.14 (26)
group 4 > 75 % perc.	0.32-1.35 (121)	0.34-1.12 (41)	0.33-1.22 (32)	0.15-0.56 (20)
**Five percentile groups**	**GSD**	**GR**	**LR**	**SP**
group 1 < 20 % perc.	0.02-0.12 (100)	0.03-0.09 (44)	0.03-0.09 (41)	0.01-0.04 (29)
group 2 20-40 % perc.	0.13-0.18 (111)	0.10-0.15 (30)	0.10-0.14 (17)	0.05-0.06 (19)
group 3 40-60 % perc.	0.19-0.25 (90)	0.16-0.23 (30)	0.15-0.23 (33)	0.07-0.08 (10)
group 4 60-80 % perc.	0.26-0.36 (102)	0.24-0.40 (32)	0.24-0.41 (24)	0.09-0.17 (17)
group 5 > 80 % perc.	0.37-1.35 (93)	0.41-1.12 (33)	0.42-1.22 (26)	0.18-0.56 (19)
