# Supplementary material for: Genome-Wide Analyses Suggest Mechanisms Involving Early B-Cell Development in Canine IgA Deficiency
Source: PLoS One. 2015 Jul 30;10(7):e0133844. doi: 10.1371/journal.pone.0133844 (PMC4520476; doi:10.1371/journal.pone.0133844)
Supplement: S15 Table — (PDF) [file pone.0133844.s025.pdf]

Table S15. The complete set of control breeds

IgA control breeds	N	IgA risk breeds	N
Bernese Mountain Dog	12	GSD	370
Border Collie	14	SP	94
Border Terrier	25	GR	185
Boxer	2	LR	302
Brittany Spaniel	12		
Cavalier King Charles Spaniel	2		
Cocker Spaniel	14		
Dachshund	12		
Dalmatian	7		
Doberman Pinscher	25		
English Bulldog	12		
English Setter	12		
Eurasian	12		
Finnish Spitz	12		
Gordon Setter	25		
Greyhound	11		
Greenland Sledge Dog	12		
Irish Wolfhound	11		
Jack Russell Terrier	12		
Newfoundland	25		
Poodle	11		
Rottweiler	12		
Schipperke	25		
Standard Poodle	21		
Terrier Yorkshire	12		
**In total 25 breeds**	350		
