# Supplementary material for: Genome-Wide Analyses Suggest Mechanisms Involving Early B-Cell Development in Canine IgA Deficiency
Source: PLoS One. 2015 Jul 30;10(7):e0133844. doi: 10.1371/journal.pone.0133844 (PMC4520476; doi:10.1371/journal.pone.0133844)
Supplement: S16 Table — (PDF) [file pone.0133844.s026.pdf]

Table S16. Allele frequencies based on eight breeds (160 dogs) across chr28: 9000091-11999424 (canfam3)

CHROM	POS	N_ALLELES	N_CHR	{ALLELE:FREQ}	
chr28	9000091	2	312	T:0.900641	C:0.099359
chr28	9000246	2	304	T:0.947368	TCAA:0.0526316
chr28	9000473	2	302	A:0.933775	G:0.0662252
chr28	9000522	2	312	T:0.935897	C:0.0641026
chr28	9000657	2	308	C:0.935065	CTTTAT:0.0649351
chr28	9000789	2	302	T:0.943709	C:0.0562914
chr28	9000939	2	312	CGG:0.923077	C:0.0769231
chr28	9001032	2	300	C:0.923333	G:0.0766667
chr28	9001109	2	292	C:0.945205	G:0.0547945
chr28	9001229	5	318	GAGAGAAGAGA:0.213836	G:0.132075 GAGAGA:0.273585 GAGAGAAGAGAAGAGA:0.110063 GAGAGAAGAGAAGAGAAGAGAAGAGAAGAGA:0.27044
chr28	9001674	2	310	G:0.93871	A:0.0612903
chr28	9001787	2	298	CA:0.95302	C:0.0469799
chr28	9001833	2	312	A:0.939103	G:0.0608974
chr28	9001982	2	310	AT:0.951613	A:0.0483871
chr28	9002236	2	314	ATCCAGGAC:0.920382	A:0.0796178
chr28	9002267	2	298	C:0.936242	T:0.0637584
chr28	9002509	2	300	C:0.853333	T:0.146667
chr28	9002784	2	304	A:0.9375	G:0.0625
chr28	9002831	2	302	C:0.927152	T:0.0728477
chr28	9003437	2	298	T:0.936242	C:0.0637584
chr28	9003632	2	288	G:0.947917	GCTCT:0.0520833
chr28	9003633	2	288	C:0.940972	CTCTT:0.0590278
chr28	9003904	2	298	C:0.92953	T:0.0704698
chr28	9004320	2	298	A:0.869128	C:0.130872
chr28	9004520	2	310	A:0.86129	C:0.13871
chr28	9004564	2	294	A:0.92517	G:0.0748299
chr28	9004919	2	300	T:0.94	C:0.06
chr28	9005309	2	310	A:0.883871	G:0.116129
chr28	9005338	2	304	T:0.930921	C:0.0690789
chr28	9005502	2	316	G:0.949367	C:0.0506329
chr28	9005638	2	318	G:0.962264	A:0.0377358
chr28	9005688	2	312	A:0.929487	C:0.0705128
chr28	9006019	2	296	A:0.935811	AC:0.0641892
chr28	9006568	2	284	T:0.855634	TA:0.144366
chr28	9007042	2	292	A:0.945205	C:0.0547945
chr28	9007529	2	310	CA:0.945161	C:0.0548387
chr28	9008103	2	300	T:0.966667	C:0.0333333
chr28	9008419	2	304	C:0.967105	T:0.0328947
chr28	9008506	2	290	CAGAG:0.944828	C:0.0551724
chr28	9008594	2	274	C:0.959854	T:0.040146
chr28	9008801	2	308	C:0.951299	CA:0.0487013
chr28	9008900	2	280	T:0.878571	TA:0.121429
chr28	9009257	2	298	A:0.895973	G:0.104027
chr28	9009318	2	286	G:0.968531	A:0.0314685
chr28	9009369	2	300	AAC:0.976667	A:0.0233333
chr28	9009943	2	300	C:0.976667	T:0.0233333
chr28	9010058	2	308	GTT:0.970779	G:0.0292208
chr28	9010471	2	300	G:0.956667	A:0.0433333

chr28	9011133	2	304	A:0.963816	C:0.0361842			
chr28	9011297	2	304	G:0.973684	A:0.0263158			
chr28	9011572	2	302	A:0.97351	T:0.0264901			
chr28	9011586	2	304	TGTA:0.970395	T:0.0296053			
chr28	9012020	2	316	CCTTTCTTTCT:0.914557	C:0.085443			
chr28	9012155	2	302	C:0.97351	T:0.0264901			
chr28	9012207	2	312	GCTC:0.964744	G:0.0352564			
chr28	9012229	2	312	G:0.964744	T:0.0352564			
chr28	9012232	2	312	A:0.964744	C:0.0352564			
chr28	9012244	2	312	C:0.964744	T:0.0352564			
chr28	9012364	2	306	G:0.96732	A:0.0326797			
chr28	9012499	2	282	C:0.960993	T:0.0390071			
chr28	9012965	2	304	G:0.967105	A:0.0328947			
chr28	9013095	2	310	C:0.970968	G:0.0290323			
chr28	9013098	2	308	A:0.805195	G:0.194805			
chr28	9013127	2	302	T:0.966887	C:0.0331126			
chr28	9013382	2	296	T:0.807432	C:0.192568			
chr28	9013443	2	314	G:0.961783	A:0.0382166			
chr28	9013453	2	314	G:0.961783	A:0.0382166			
chr28	9013626	2	316	C:0.85443	T:0.14557			
chr28	9013745	2	300	T:0.8	C:0.2			
chr28	9013848	2	308	C:0.974026	T:0.025974			
chr28	9013894	2	302	C:0.970199	T:0.0298013			
chr28	9013951	2	308	C:0.961039	G:0.038961			
chr28	9014048	2	312	G:0.964744	A:0.0352564			
chr28	9014089	2	306	A:0.781046	C:0.218954			
chr28	9014093	2	300	C:0.936667	CA:0.0633333			
chr28	9014112	2	296	T:0.797297	C:0.202703			
chr28	9014138	2	298	T:0.808725	G:0.191275			
chr28	9014282	2	312	C:0.830128	T:0.169872			
chr28	9014788	2	314	G:0.974522	GTCA:0.0254777			
chr28	9014789	2	314	G:0.974522	GGATCCTGATC:0.0254777			
chr28	9014793	2	314	C:0.974522	T:0.0254777			
chr28	9014795	2	314	CG:0.974522	C:0.0254777			
chr28	9014797	2	314	G:0.974522	T:0.0254777			
chr28	9014841	2	304	C:0.825658	T:0.174342			
chr28	9014988	2	306	G:0.826797	T:0.173203			
chr28	9015209	2	310	CAT:0.835484	C:0.164516			
chr28	9015491	2	308	A:0.980519	G:0.0194805			
chr28	9015823	2	310	T:0.832258	A:0.167742			
chr28	9016088	2	298	C:0.973154	G:0.0268456			
chr28	9016103	2	304	A:0.819079	G:0.180921			
chr28	9016322	2	312	A:0.932692	AAT:0.0673077			
chr28	9016326	2	308	CACAT:0.964286	C:0.0357143			
chr28	9016330	5	314	T:0.461783	C:0.133758	TAC:0.194268	TACAC:0.10828	TACACAC:0.101911
chr28	9016360	3	314	C:0.863057	G:0.0636943	CACAG:0.0732484		
chr28	9016682	2	302	G:0.980132	GA:0.0198675			
chr28	9016800	2	312	T:0.971154	A:0.0288462			
chr28	9016982	2	288	A:0.961806	G:0.0381944			
chr28	9017130	3	306	GAA:0.718954	G:0.0326797			

GA:0.248366
chr28 9017172 2 302 A:0.834437 AAAGC:0.165563
chr28 9017284 2 302 C:0.97351 T:0.0264901
chr28 9017304 2 302 T:0.97351 G:0.0264901
chr28 9017446 2 286 C:0.972028 T:0.027972
chr28 9017524 2 272 TCTCTCC:0.863971 T:0.136029
chr28 9017528 2 276 TCC:0.431159 T:0.568841
chr28 9017953 2 298 G:0.812081 A:0.187919
chr28 9018548 2 310 T:0.13871 G:0.86129
chr28 9018662 2 292 C:0.965753 G:0.0342466
chr28 9018674 2 282 TA:0.794326 T:0.205674
chr28 9018856 5 312 CAAATAAATAAAT:0.532051 C:
0.112179 CTAAAT:0.0512821 CTAAATAAAT:0.0480769
CAAATAAATAAATAAAT:0.25641
chr28 9018860 2 312 T:0.964744 TCTC:0.0352564
chr28 9018864 2 310 T:0.977419 TCTC:0.0225806
chr28 9019603 2 300 C:0.84 T:0.16
chr28 9019751 2 292 A:0.849315 AT:0.150685
chr28 9020694 2 308 C:0.798701 CAAAT:0.201299
chr28 9020794 2 302 A:0.966887 G:0.0331126
chr28 9020798 2 302 A:0.966887 G:0.0331126
chr28 9021002 2 292 A:0.821918 T:0.178082
chr28 9022289 2 308 C:0.834416 T:0.165584
chr28 9022290 2 308 A:0.811688 G:0.188312
chr28 9022351 2 308 C:0.814935 T:0.185065
chr28 9022352 2 308 G:0.814935 A:0.185065
chr28 9022420 2 314 G:0.869427 T:0.130573
chr28 9022668 2 306 AG:0.836601 A:0.163399
chr28 9022984 2 254 C:0.834646 CT:0.165354
chr28 9023016 2 268 G:0.835821 T:0.164179
chr28 9023157 2 310 C:0.967742 CTGTGTG:0.0322581
chr28 9023159 3 312 CTG:0.913462 C:0.0544872
GTG:0.0320513
chr28 9023202 2 288 T:0.951389 TA:0.0486111
chr28 9023408 2 292 G:0.832192 GTAAA:0.167808
chr28 9023450 3 306 TGA:0.647059 T:0.222222
TGAGA:0.130719
chr28 9023552 2 288 G:0.829861 GATC:0.170139
chr28 9024684 2 290 A:0.968966 G:0.0310345
chr28 9024720 4 286 T:0.402098 TA:0.181818
TAA:0.286713 TAAA:0.129371
chr28 9024730 2 308 A:0.801948 AAAAAAAAAAAG:
0.198052
chr28 9024778 2 288 C:0.836806 T:0.163194
chr28 9025290 2 312 C:0.836538 T:0.163462
chr28 9025339 2 300 T:0.956667 TG:0.0433333
chr28 9025345 2 300 A:0.956667 T:0.0433333
chr28 9025361 2 298 T:0.845638 A:0.154362
chr28 9025362 2 298 A:0.845638 T:0.154362
chr28 9025730 2 262 AAT:0.942748 A:0.0572519
chr28 9025731 2 252 AT:0.198413 A:0.801587
chr28 9025899 2 254 C:0.952756 T:0.0472441
chr28 9026013 2 312 TG:0.846154 T:0.153846
chr28 9026014 2 312 G:0.967949 T:0.0320513

chr28	9026018	2	312	T:0.846154	A:0.153846	
chr28	9026023	2	312	T:0.846154	A:0.153846	
chr28	9026048	2	312	C:0.817308	T:0.182692	
chr28	9026069	2	312	G:0.967949	C:0.0320513	
chr28	9026275	3	300	TAA:0.626667	T:0.163333	TA:0.21
chr28	9026405	2	290	G:0.834483	A:0.165517	
chr28	9026635	2	298	A:0.842282	G:0.157718	
chr28	9026679	2	282	T:0.826241	C:0.173759	
chr28	9026960	2	300	A:0.856667	G:0.143333	
chr28	9027069	2	294	C:0.823129	A:0.176871	
chr28	9028226	2	310	C:0.832258	T:0.167742	
chr28	9028471	2	314	C:0.961783	T:0.0382166	
chr28	9029031	2	298	C:0.838926	CA:0.161074	
chr28	9029032	2	318	A:0.940252	AAAAAAAAAATAAAAT:0.0597484	
chr28	9029064	2	310	T:0.812903	C:0.187097	
chr28	9029759	2	278	C:0.766187	CT:0.233813	
chr28	9029808	2	314	CACAGAGAGAGAGAG:0.796178	C:0.203822	
chr28	9029810	3	314	CAG:0.43949	C:0.433121	CAGAG:0.127389
chr28	9029956	2	294	G:0.795918	GAGCC:0.204082	
chr28	9030144	2	304	A:0.871711	G:0.128289	
chr28	9030185	2	302	T:0.791391	A:0.208609	
chr28	9030186	2	302	T:0.966887	A:0.0331126	
chr28	9030657	2	290	G:0.975862	A:0.0241379	
chr28	9030699	2	292	C:0.876712	T:0.123288	
chr28	9031234	2	286	G:0.958042	A:0.041958	
chr28	9031701	2	300	C:0.96	T:0.04	
chr28	9031738	2	298	T:0.828859	C:0.171141	
chr28	9031741	3	286	GT:0.601399	G:0.304196	GTT:0.0944056
chr28	9032465	2	302	A:0.993377	G:0.00662252	
chr28	9033585	2	296	T:0.827703	A:0.172297	
chr28	9033586	2	296	T:0.827703	C:0.172297	
chr28	9033837	2	204	TA:0.637255	T:0.362745	
chr28	9034448	2	306	G:0.964052	A:0.0359477	
chr28	9035127	2	308	C:0.827922	CTTAT:0.172078	
chr28	9035331	2	310	T:0.829032	TAATA:0.170968	
chr28	9036932	2	308	T:0.831169	C:0.168831	
chr28	9037083	2	308	T:0.321429	C:0.678571	
chr28	9037089	2	308	T:0.850649	C:0.149351	
chr28	9037329	2	302	G:0.980132	A:0.0198675	
chr28	9037585	2	302	G:0.831126	GA:0.168874	
chr28	9037594	2	302	C:0.956954	A:0.0430464	
chr28	9037846	2	300	CTG:0.966667	C:0.0333333	
chr28	9038349	2	308	A:0.831169	G:0.168831	
chr28	9038962	2	298	G:0.979866	A:0.0201342	
chr28	9039044	2	294	T:0.880952	G:0.119048	
chr28	9039059	2	302	C:0.84106	CCTT:0.15894	
chr28	9039125	2	304	C:0.986842	T:0.0131579	
chr28	9039672	2	296	G:0.905405	A:0.0945946	
chr28	9039766	2	302	G:0.900662	C:0.0993377	

chr28	9040250	2	294	T:0.87415	C:0.12585		
chr28	9040258	2	298	GC:0.815436	G:0.184564		
chr28	9040648	2	310	G:0.906452	C:0.0935484		
chr28	9040777	2	306	C:0.915033	CACTA:0.0849673		
chr28	9040794	2	298	A:0.912752	G:0.0872483		
chr28	9040979	2	296	T:0.915541	C:0.0844595		
chr28	9040991	2	294	ACT:0.914966	A:0.085034		
chr28	9041256	2	290	G:0.986207	A:0.0137931		
chr28	9041508	2	302	A:0.976821	C:0.0231788		
chr28	9041559	2	280	G:0.917857	A:0.0821429		
chr28	9041584	2	286	CTT:0.912587	C:0.0874126		
chr28	9042088	2	304	G:0.901316	A:0.0986842		
chr28	9042090	2	304	A:0.901316	T:0.0986842		
chr28	9042656	2	302	T:0.900662	G:0.0993377		
chr28	9042713	2	304	C:0.983553	T:0.0164474		
chr28	9043052	2	298	GC:0.899329	G:0.100671		
chr28	9046466	2	294	G:0.918367	A:0.0816327		
chr28	9046907	2	308	A:0.928571	AAC:0.0714286		
chr28	9047244	2	300	A:0	G:1		
chr28	9047405	2	298	G:0.899329	A:0.100671		
chr28	9047791	2	284	T:0.0105634	TG:0.989437		
chr28	9048050	2	272	T:0.930147	C:0.0698529		
chr28	9048208	2	246	AT:0	A:1		
chr28	9048224	2	194	T:0.912371	C:0.0876289		
chr28	9048896	2	296	GC:0.898649	G:0.101351		
chr28	9049363	2	320	AAAG:0.903125	A:0.096875		
chr28	9049366	3	318	GAAGAAAGAAAGAAAGA:0.484277	G:0.339623	GAAGA:0.176101	
chr28	9049429	2	320	G:0.903125	GA:0.096875		
chr28	9050173	2	290	TG:0.913793	T:0.0862069		
chr28	9050240	2	308	A:0.909091	C:0.0909091		
chr28	9051784	2	300	G:0.903333	C:0.0966667		
chr28	9051823	2	302	C:0.903974	T:0.0960265		
chr28	9051826	2	302	C:0.903974	T:0.0960265		
chr28	9052674	2	306	A:0.905229	T:0.0947712		
chr28	9052923	2	278	G:0.910072	A:0.0899281		
chr28	9052947	4	298	AACACACAC:0.244966	A:0.281879	AAC:0.352349	AACAC:0.120805
chr28	9053125	2	296	G:0.908784	A:0.0912162		
chr28	9053140	2	302	A:0.97351	G:0.0264901		
chr28	9053532	2	300	G:0.9	C:0.1		
chr28	9055264	2	300	GT:0.356667	G:0.643333		
chr28	9056134	2	310	GGCAGCCCCCGTGGT:0.916129	G:0.083871		
chr28	9056143	2	310	C:0.941935	G:0.0580645		
chr28	9056457	2	298	A:0.919463	G:0.0805369		
chr28	9057481	2	304	T:0.986842	A:0.0131579		
chr28	9058292	2	294	T:0.891156	TA:0.108844		
chr28	9059203	2	274	ATTTCTT:0.959854	A:0.040146		
chr28	9059843	2	308	G:0.996753	C:0.00324675		
chr28	9060075	4	314	TACAAA:0.840764	T:0.0764331	TACAAAACAAA:0.0414013	TACAAAACAAAACAAAACAAA:0.0414013
chr28	9060852	2	302	C:0.450331	T:0.549669		
chr28	9061609	2	296	A:0.942568	C:0.0574324		

chr28	9061679	2	302	A:0.94702	G:0.0529801	
chr28	9061900	2	300	C:0.936667	T:0.0633333	
chr28	9062033	2	296	TCGCTCACA:0.942568	T:0.0574324	
chr28	9062147	2	298	G:0.936242	A:0.0637584	
chr28	9062361	3	222	TGG:0.716216	T:0.130631	TG:0.153153
chr28	9063282	2	170	AC:0.835294	A:0.164706	
chr28	9063400	2	220	G:0.522727	C:0.477273	
chr28	9064386	2	278	C:0.820144	CTT:0.179856	
chr28	9064472	2	302	G:0.857616	GC:0.142384	
chr28	9064477	2	302	G:0.857616	C:0.142384	
chr28	9064668	2	308	G:0.987013	T:0.012987	
chr28	9065709	2	306	GA:0.954248	G:0.0457516	
chr28	9066664	2	306	G:0.872549	A:0.127451	
chr28	9066770	2	304	A:0.855263	G:0.144737	
chr28	9067055	2	304	G:0.871711	A:0.128289	
chr28	9068116	2	312	A:0.564103	AAAAG:0.435897	
chr28	9068122	2	316	A:0.901899	AAGAAAG:0.0981013	
chr28	9068140	2	308	G:0.886364	A:0.113636	
chr28	9068147	2	316	A:0.914557	AAAG:0.085443	
chr28	9068158	3	316	G:0.462025	GAAGA:0.240506	GGAAGAAAGAAAGAAAGGAAGGAAGAAAGAAAGAAAGA:0.297468
chr28	9068456	2	306	C:0.859477	T:0.140523	
chr28	9068849	2	316	T:0.863924	TAAAAAA:0.136076	
chr28	9068872	2	310	GGAATA:0.893548	G:0.106452	
chr28	9069173	2	304	G:0.845395	A:0.154605	
chr28	9069266	2	298	A:0.875839	G:0.124161	
chr28	9069399	2	244	C:0.668033	CA:0.331967	
chr28	9069746	2	294	T:0.853741	C:0.146259	
chr28	9070332	2	302	A:0.857616	AGT:0.142384	
chr28	9071571	2	306	G:0.937908	T:0.0620915	
chr28	9071574	2	308	G:0.873377	T:0.126623	
chr28	9071694	2	310	A:0.887097	G:0.112903	
chr28	9071730	2	296	G:0.935811	T:0.0641892	
chr28	9072256	2	298	T:0.88255	A:0.11745	
chr28	9073177	3	304	TAA:0.536184	T:0.118421	TA:0.345395
chr28	9073184	2	306	A:0.800654	T:0.199346	
chr28	9073654	2	306	T:0.866013	G:0.133987	
chr28	9073869	2	290	T:0.862069	C:0.137931	
chr28	9073993	2	308	A:0.720779	AT:0.279221	
chr28	9073995	2	308	A:0.720779	T:0.279221	
chr28	9074033	2	302	CAGATAGAG:0.715232	C:0.284768	
chr28	9074037	3	306	TAGAGAG:0.781046	T:0.0294118	TAGAG:0.189542
chr28	9074750	2	304	TA:0.723684	T:0.276316	
chr28	9075130	2	306	C:0.699346	T:0.300654	
chr28	9075511	2	318	CT:0.710692	C:0.289308	
chr28	9075676	2	302	A:0.705298	G:0.294702	
chr28	9075684	2	302	T:0.705298	A:0.294702	
chr28	9076002	2	294	C:0.952381	T:0.047619	
chr28	9076092	2	306	G:0.732026	C:0.267974	

chr28	9076158	2	304	T:0.733553	C:0.266447	
chr28	9076182	2	304	T:0.733553	C:0.266447	
chr28	9076673	2	308	CA:0.733766	C:0.266234	
chr28	9076764	2	298	T:0.724832	C:0.275168	
chr28	9076812	2	308	G:0.737013	T:0.262987	
chr28	9076821	2	308	T:0.737013	G:0.262987	
chr28	9076835	2	308	G:0.737013	T:0.262987	
chr28	9076846	2	308	C:0.737013	T:0.262987	
chr28	9076857	2	308	A:0.737013	T:0.262987	
chr28	9076902	2	288	T:0.732639	C:0.267361	
chr28	9076908	2	288	T:0.732639	C:0.267361	
chr28	9076958	3	284	TA:0.471831	T:0.253521	TAA:0.274648
chr28	9077225	2	308	C:0.733766	T:0.266234	
chr28	9077262	2	304	T:0.746711	A:0.253289	
chr28	9077365	2	308	A:0.847403	G:0.152597	
chr28	9077504	2	300	C:0.753333	T:0.246667	
chr28	9077715	2	306	T:0.784314	C:0.215686	
chr28	9077725	3	308	C:0.785714	CTTT:0.12013	CTTTT:0.0941558
chr28	9078016	2	308	GCAGT:0.850649	G:0.149351	
chr28	9078201	2	308	C:0.733766	T:0.266234	
chr28	9078207	2	308	A:0.733766	G:0.266234	
chr28	9078254	2	302	T:0.731788	G:0.268212	
chr28	9078267	2	300	C:0.73	A:0.27	
chr28	9078294	2	298	A:0.731544	G:0.268456	
chr28	9078307	2	306	T:0.72549	TG:0.27451	
chr28	9078314	2	306	G:0.72549	A:0.27451	
chr28	9078760	2	302	C:0.427152	CT:0.572848	
chr28	9079172	2	314	A:0.735669	G:0.264331	
chr28	9079178	2	314	G:0.735669	A:0.264331	
chr28	9079196	2	314	A:0.735669	G:0.264331	
chr28	9079218	2	314	A:0.735669	G:0.264331	
chr28	9079312	2	308	C:0.717532	T:0.282468	
chr28	9079313	2	308	CT:0.717532	C:0.282468	
chr28	9079508	2	286	C:0.898601	T:0.101399	
chr28	9079509	2	286	A:0.793706	G:0.206294	
chr28	9079784	2	294	C:0.959184	CT:0.0408163	
chr28	9080212	2	294	G:0.952381	GCC:0.047619	
chr28	9080216	2	294	C:0.952381	CGA:0.047619	
chr28	9080219	2	286	T:0.84965	G:0.15035	
chr28	9080220	2	286	C:0.84965	A:0.15035	
chr28	9080303	2	306	G:0.718954	A:0.281046	
chr28	9080344	2	298	C:0.993289	T:0.00671141	
chr28	9080453	2	280	T:0.721429	C:0.278571	
chr28	9080484	2	280	T:0.742857	TA:0.257143	
chr28	9080539	2	298	G:0.724832	A:0.275168	
chr28	9081156	2	224	C:0.839286	CT:0.160714	
chr28	9081540	2	308	A:0.691558	C:0.308442	
chr28	9081732	2	302	C:0.701987	A:0.298013	
chr28	9082462	2	300	C:0.696667	CT:0.303333	
chr28	9082581	2	296	G:0.706081	A:0.293919	
chr28	9082712	2	308	CAA:0.720779	C:0.279221	
chr28	9082716	2	310	T:0.722581	C:0.277419	

chr28	9082732	2	312	C:0.714744	CTT:0.285256
chr28	9082740	2	310	C:0.167742	T:0.832258
chr28	9083013	2	306	A:0.702614	G:0.297386
chr28	9083057	2	308	T:0.698052	C:0.301948
chr28	9083137	2	306	C:0.70915	T:0.29085
chr28	9083675	2	298	CT:0.808725	C:0.191275
chr28	9084375	2	302	CCT:0.715232	C:0.284768
chr28	9084629	2	298	G:0.721477	C:0.278523
chr28	9084938	2	300	G:0.713333	A:0.286667
chr28	9085196	2	294	G:0.727891	T:0.272109
chr28	9085578	2	304	G:0.700658	GCTCTCT:0.299342
chr28	9085618	2	306	T:0.859477	A:0.140523
chr28	9086088	2	300	CTT:0.716667	C:0.283333
chr28	9086258	2	290	G:0.710345	C:0.289655
chr28	9086297	2	308	CTTTATA:0.905844	C:0.0941558
chr28	9086512	2	304	T:0.963816	C:0.0361842
chr28	9086603	2	310	T:0.970968	C:0.0290323
chr28	9086707	2	300	C:0.856667	G:0.143333
chr28	9086913	2	296	T:0.709459	C:0.290541
chr28	9087018	2	298	A:0.194631	C:0.805369
chr28	9087957	2	300	A:0.696667	G:0.303333
chr28	9088172	3	294	TA:0.659864	T:0.129252
TAA:0.210884					
chr28	9088444	2	308	TAGCTA:0.857143	T:0.142857
chr28	9088609	2	240	T:0.666667	TAA:0.333333
chr28	9088684	2	280	T:0.696429	C:0.303571
chr28	9088799	2	226	T:0.646018	TAA:0.353982
chr28	9088927	2	296	C:0.85473	G:0.14527
chr28	9089022	2	272	C:0.735294	A:0.264706
chr28	9089105	2	304	C:0.845395	T:0.154605
chr28	9089203	2	314	T:0.710191	A:0.289809
chr28	9089232	2	310	C:0.854839	CAGAGAG:0.145161
chr28	9089415	2	314	C:0.840764	T:0.159236
chr28	9090136	2	302	G:0.874172	A:0.125828
chr28	9090764	2	302	A:0.850993	C:0.149007
chr28	9090988	2	292	A:0.678082	G:0.321918
chr28	9091191	2	294	C:0.85034	T:0.14966
chr28	9091307	2	312	C:0.842949	T:0.157051
chr28	9091652	2	302	C:0.864238	T:0.135762
chr28	9091771	2	300	GT:0.84 G:0.16	
chr28	9091923	2	310	G:0.825806	A:0.174194
chr28	9092080	2	306	C:0.846405	G:0.153595
chr28	9092223	2	306	C:0.856209	T:0.143791
chr28	9092909	2	294	CT:0.836735	C:0.163265
chr28	9093041	2	300	C:0.863333	T:0.136667
chr28	9093112	2	286	G:0.853147	A:0.146853
chr28	9093187	2	296	CCT:0.689189	C:0.310811
chr28	9093590	2	278	CCT:0.81295	C:0.18705
chr28	9093627	2	292	TAAAA:0.85274	T:0.14726
chr28	9093630	2	304	A:0.980263	AT:0.0197368
chr28	9093633	2	304	A:0.980263	AT:0.0197368
chr28	9093635	2	292	A:0.85274	T:0.14726
chr28	9093641	2	304	A:0.980263	T:0.0197368
chr28	9094162	2	306	A:0.722222	G:0.277778

chr28	9094920	2	310	T:0.851613	G:0.148387	
chr28	9095328	2	292	G:0.869863	A:0.130137	
chr28	9095562	2	298	CTCTG:0.832215	C:0.167785	
chr28	9095605	3	292	AG:0.708904	A:0.164384	AGG:0.126712
chr28	9095719	2	302	G:0.976821	A:0.0231788	
chr28	9095822	2	300	G:0.856667	T:0.143333	
chr28	9096051	2	300	A:0.86	G:0.14	
chr28	9096278	2	292	T:0.996575	G:0.00342466	
chr28	9096366	2	298	TAC:0.724832	T:0.275168	
chr28	9096449	2	288	C:0.989583	G:0.0104167	
chr28	9096503	2	316	C:0.629747	CTTTTTTTTTTTT:0.370253	
chr28	9096636	2	290	T:0.872414	C:0.127586	
chr28	9097105	2	292	C:0.869863	T:0.130137	
chr28	9097982	2	314	C:0.859873	T:0.140127	
chr28	9098466	2	310	T:0.948387	A:0.0516129	
chr28	9098761	2	302	AC:0.864238	A:0.135762	
chr28	9099002	2	308	C:0.944805	T:0.0551948	
chr28	9099249	2	304	T:0.805921	C:0.194079	
chr28	9099266	2	300	G:0.953333	T:0.0466667	
chr28	9099550	2	306	T:0.95098	TAA:0.0490196	
chr28	9099640	2	290	A:0.944828	C:0.0551724	
chr28	9100002	2	288	G:0.951389	A:0.0486111	
chr28	9100006	2	288	C:0.8125	T:0.1875	
chr28	9100067	2	308	TTCTC:0.935065	T:0.0649351	
chr28	9100103	2	306	GA:0.95098	G:0.0490196	
chr28	9100185	2	312	G:0.887821	C:0.112179	
chr28	9100333	2	278	T:0.94964	C:0.0503597	
chr28	9100450	3	316	C:0.841772	CTCTG:0.117089	CTCTCTCTCTCTCTG:0.0411392
chr28	9100716	2	280	C:0.95	A:0.05	
chr28	9100869	2	294	C:0.945578	T:0.0544218	
chr28	9101114	2	300	T:0.943333	C:0.0566667	
chr28	9101296	2	302	C:0.791391	G:0.208609	
chr28	9102150	2	298	A:0.939597	G:0.0604027	
chr28	9102269	2	296	G:0.942568	C:0.0574324	
chr28	9102282	2	300	A:0.943333	G:0.0566667	
chr28	9102285	2	300	G:0.943333	C:0.0566667	
chr28	9102675	2	294	CCTGT:0.911565	C:0.0884354	
chr28	9103023	2	302	C:0.937086	T:0.0629139	
chr28	9103084	2	304	T:0.861842	A:0.138158	
chr28	9103085	2	304	T:0.799342	A:0.200658	
chr28	9103207	2	294	T:0.94898	C:0.0510204	
chr28	9103230	2	288	T:0	C:1	
chr28	9103393	2	308	T:0.954545	C:0.0454545	
chr28	9103559	2	294	G:0.94898	A:0.0510204	
chr28	9104029	2	316	A:0.949367	G:0.0506329	
chr28	9104106	2	302	C:0.950331	G:0.0496689	
chr28	9104152	2	310	TAAC:0.867742	T:0.132258	
chr28	9104461	2	300	T:0.953333	TA:0.0466667	
chr28	9104565	2	306	CAATT:0.944444	C:0.0555556	
chr28	9104842	2	312	C:0.951923	CTTTCTTTTCT:0.0480769	

chr28	9104935	2	302	A:0.953642	G:0.0463576	
chr28	9105010	2	316	CTTTTTTTTGTTTT:0.797468	C:0.202532	
chr28	9105019	2	318	GT:0.827044	G:0.172956	
chr28	9105026	2	318	T:0.959119	G:0.0408805	
chr28	9105332	2	308	C:0.961039	G:0.038961	
chr28	9105405	2	306	G:0.839869	A:0.160131	
chr28	9105780	2	296	G:0.972973	A:0.027027	
chr28	9106125	2	310	TCTTTCCC:0.935484	T:0.0645161	
chr28	9106133	2	310	A:0.935484	AGGT:0.0645161	
chr28	9106477	2	304	A:0.9375	G:0.0625	
chr28	9106924	2	292	G:0.876712	A:0.123288	
chr28	9107300	2	300	G:0.95	A:0.05	
chr28	9107320	2	292	C:0.958904	T:0.0410959	
chr28	9107462	2	310	G:0.825806	C:0.174194	
chr28	9107744	2	310	C:0.86129	T:0.13871	
chr28	9108048	2	306	A:0.954248	G:0.0457516	
chr28	9108085	2	308	G:0.883117	A:0.116883	
chr28	9108126	2	308	T:0.954545	C:0.0454545	
chr28	9108519	2	296	CTTT:0.952703	C:0.0472973	
chr28	9108535	2	296	T:0.925676	A:0.0743243	
chr28	9108685	2	306	GAA:0.931373	G:0.0686275	
chr28	9108720	2	312	T:0.945513	C:0.0544872	
chr28	9108873	2	296	C:0.952703	T:0.0472973	
chr28	9108950	2	278	C:0.94964	A:0.0503597	
chr28	9108980	2	310	AAAAAG:0.929032	A:0.0709677	
chr28	9109317	2	306	T:0.973856	A:0.0261438	
chr28	9109371	2	306	A:0.944444	G:0.0555556	
chr28	9109634	2	292	A:0.941781	G:0.0582192	
chr28	9109805	2	300	C:0.943333	T:0.0566667	
chr28	9110262	2	308	A:0.954545	G:0.0454545	
chr28	9110338	2	296	G:0.949324	A:0.0506757	
chr28	9110411	2	302	G:0.986755	A:0.013245	
chr28	9110488	2	304	T:0.953947	C:0.0460526	
chr28	9110514	3	300	TA:0.903333	T:0.0566667	TAA:0.04
chr28	9110601	2	302	GA:0.993377	G:0.00662252	
chr28	9110604	2	302	GT:0.993377	G:0.00662252	
chr28	9110928	2	302	A:0.817881	G:0.182119	
chr28	9111013	2	306	G:0.944444	A:0.0555556	
chr28	9111170	2	310	A:0.951613	C:0.0483871	
chr28	9111254	2	300	G:0.94	GT:0.06	
chr28	9111556	2	310	C:0.819355	G:0.180645	
chr28	9111695	2	296	A:0.952703	G:0.0472973	
chr28	9112237	2	308	G:0.957792	A:0.0422078	
chr28	9113042	2	302	G:0.950331	A:0.0496689	
chr28	9113206	2	298	C:0.852349	G:0.147651	
chr28	9113447	2	308	TTTA:0.970779	T:0.0292208	
chr28	9113448	2	308	TTA:0.977273	T:0.0227273	
chr28	9113458	2	314	TTTTA:0.936306	T:0.0636943	
chr28	9113486	2	274	C:0.941606	CAG:0.0583942	
chr28	9113601	2	286	C:0.947552	A:0.0524476	
chr28	9113608	2	280	T:0.817857	C:0.182143	

chr28	9113777	2	296	TG:0.945946	T:0.0540541	
chr28	9113925	2	264	TA:0.931818	T:0.0681818	
chr28	9114091	2	288	G:0.8125	C:0.1875	
chr28	9114192	2	312	C:0.955128	A:0.0448718	
chr28	9114394	2	294	T:0.816327	A:0.183673	
chr28	9114401	2	304	C:0.953947	CAA:0.0460526	
chr28	9114999	2	296	C:0.945946	T:0.0540541	
chr28	9115428	2	302	C:0.950331	T:0.0496689	
chr28	9115456	2	312	A:0.961538	G:0.0384615	
chr28	9115654	2	314	A:0.94586	G:0.0541401	
chr28	9115933	2	312	G:0.807692	GTTGT:0.192308	
chr28	9116460	2	312	C:0.961538	T:0.0384615	
chr28	9117028	2	308	T:0.86039	A:0.13961	
chr28	9117210	2	312	T:0.86859	C:0.13141	
chr28	9117252	2	314	A:0.859873	G:0.140127	
chr28	9117980	2	314	A:0.961783	T:0.0382166	
chr28	9117981	3	314	AAG:0.93949	A:0.0382166	GAG:0.022293
chr28	9118004	2	314	C:0.961783	T:0.0382166	
chr28	9118072	2	298	GAAA:0.946309	G:0.0536913	
chr28	9118145	2	300	C:0.946667	T:0.0533333	
chr28	9118226	2	314	T:0.955414	C:0.044586	
chr28	9118408	2	306	T:0.846405	C:0.153595	
chr28	9118672	2	300	T:0.946667	A:0.0533333	
chr28	9118674	2	300	C:0.946667	A:0.0533333	
chr28	9118781	3	292	CT:0.592466	C:0.222603	CTT:0.184932
chr28	9118803	2	298	TTTTA:0.785235	T:0.214765	
chr28	9119002	2	300	G:0.97	T:0.03	
chr28	9119203	2	304	G:0.947368	A:0.0526316	
chr28	9119345	3	280	TA:0.653571	T:0.189286	TAAA:0.157143
chr28	9119362	2	300	A:0.95	AT:0.05	
chr28	9119470	2	308	T:0.951299	A:0.0487013	
chr28	9119870	3	312	AT:0.737179	A:0.108974	ATGTTTT:0.153846
chr28	9120006	2	292	G:0.94863	A:0.0513699	
chr28	9120038	2	294	A:0.812925	G:0.187075	
chr28	9120081	2	302	C:0.950331	T:0.0496689	
chr28	9120109	2	308	C:0.87013	T:0.12987	
chr28	9120303	2	306	C:0.947712	T:0.0522876	
chr28	9120801	2	300	C:0.8	T:0.2	
chr28	9121069	2	298	G:0.946309	A:0.0536913	
chr28	9121180	2	288	C:0.868056	CT:0.131944	
chr28	9121516	2	308	A:0.840909	G:0.159091	
chr28	9122021	2	320	AGGACATTTGGGCAGTGATGTTTTT:0.865625	A:0.134375	
chr28	9122067	2	306	T:0.944444	TA:0.0555556	
chr28	9122560	2	298	C:0.869128	T:0.130872	
chr28	9122818	2	294	G:0.959184	A:0.0408163	
chr28	9122925	2	304	A:0.957237	C:0.0427632	
chr28	9123875	2	300	C:0.946667	T:0.0533333	
chr28	9123928	2	300	C:0.953333	T:0.0466667	
chr28	9123957	2	304	T:0.802632	C:0.197368	

chr28	9124006	2	314	CCTCTCTCT:0.93949	C: 0.0605096	
chr28	9124042	2	312	A:0.951923	T:0.0480769	
chr28	9124060	2	312	A:0.977564	C:0.0224359	
chr28	9124061	2	312	C:0.974359	A:0.025641	
chr28	9124281	2	304	T:0.940789	C:0.0592105	
chr28	9124572	2	294	T:0.94898	TC:0.0510204	
chr28	9124752	2	300	T:0.956667	C:0.0433333	
chr28	9124828	3	274	CT:0.423358	C:0.171533	CTT:0.405109
chr28	9124843	2	300	T:0.956667	A:0.0433333	
chr28	9124899	2	262	C:0.965649	G:0.0343511	
chr28	9124901	2	262	C:0.965649	G:0.0343511	
chr28	9124972	2	294	G:0.955782	A:0.0442177	
chr28	9124982	2	294	G:0.955782	A:0.0442177	
chr28	9125096	2	308	A:0.951299	G:0.0487013	
chr28	9126738	2	302	T:0.953642	C:0.0463576	
chr28	9127083	2	294	C:0.955782	T:0.0442177	
chr28	9127129	2	306	CCTCT:0.970588	C:0.0294118	
chr28	9127181	2	308	TTAAAA:0.967532	T:0.0324675	
chr28	9127226	2	310	A:0.948387	C:0.0516129	
chr28	9127250	2	308	G:0.951299	GA:0.0487013	
chr28	9127363	2	306	T:0.944444	A:0.0555556	
chr28	9127803	2	308	T:0.954545	TA:0.0454545	
chr28	9127952	2	306	A:0.957516	G:0.0424837	
chr28	9128086	2	304	T:0.878289	C:0.121711	
chr28	9128585	2	294	G:0.955782	T:0.0442177	
chr28	9128938	2	292	T:0.945205	C:0.0547945	
chr28	9129276	2	302	C:0.943709	T:0.0562914	
chr28	9129297	2	300	C:0.94	A:0.06	
chr28	9129460	2	298	A:0.95302	G:0.0469799	
chr28	9129912	2	294	T:0.945578	C:0.0544218	
chr28	9129963	2	298	C:0.795302	T:0.204698	
chr28	9129964	2	298	A:0.795302	G:0.204698	
chr28	9130261	2	294	T:0.959184	C:0.0408163	
chr28	9130795	2	302	C:0.857616	T:0.142384	
chr28	9130813	2	302	T:0.86755	C:0.13245	
chr28	9130936	2	312	T:0.945513	C:0.0544872	
chr28	9130939	2	312	A:0.945513	G:0.0544872	
chr28	9131645	2	170	G:0.694118	A:0.305882	
chr28	9132326	2	252	T:0.785714	C:0.214286	
chr28	9132540	2	302	T:0.880795	C:0.119205	
chr28	9134968	2	290	C:0.955172	T:0.0448276	
chr28	9135012	2	218	TA:0.605505	T:0.394495	
chr28	9135153	2	298	C:0.936242	T:0.0637584	
chr28	9135492	3	282	TA:0.535461	T:0.301418	TAAA:0.163121
chr28	9135625	2	308	T:0.961039	A:0.038961	
chr28	9136723	2	304	CAT:0.963816	C:0.0361842	
chr28	9136768	2	310	T:0.945161	G:0.0548387	
chr28	9136846	2	310	A:0.951613	T:0.0483871	
chr28	9136848	2	310	G:0.951613	T:0.0483871	
chr28	9136969	2	310	G:0.948387	A:0.0516129	
chr28	9137075	2	282	CT:0.787234	C:0.212766	

```
chr28	9137096	2	306	TTTTA:0.846405	T:0.153595
chr28	9137144	2	304	T:0.953947	C:0.0460526
chr28	9137614	2	308	A:0.957792	C:0.0422078
chr28	9138170	3	314	C:0.853503	CCTCT:0.101911
CCTCTCTCTCTCTCTCT:0.044586
chr28	9138203	2	298	GAATA:0.949664	G:0.0503356
chr28	9138235	2	292	T:0.267123	TA:0.732877
chr28	9139044	2	304	A:0.960526	G:0.0394737
chr28	9139062	2	306	C:0.813725	CTTCT:0.186275
chr28	9139324	2	300	C:0.956667	CACAGAGAG:
0.0433333
chr28	9139613	2	290	G:0.97931	C:0.0206897
chr28	9139644	2	300	G:0.97	T:0.03
chr28	9139804	2	290	G:0.855172	T:0.144828
chr28	9139909	2	304	C:0.957237	G:0.0427632
chr28	9140077	2	302	A:0.983444	T:0.0165563
chr28	9140530	2	312	G:0.846154	C:0.153846
chr28	9140720	2	308	G:0.948052	A:0.0519481
chr28	9140804	2	306	C:0.944444	CTG:0.0555556
chr28	9141197	2	314	T:0.958599	TA:0.0414013
chr28	9141271	2	310	A:0.948387	T:0.0516129
chr28	9141396	2	296	G:0.942568	C:0.0574324
chr28	9142392	2	304	G:0.950658	A:0.0493421
chr28	9142600	2	302	A:0.94702	T:0.0529801
chr28	9142635	2	296	A:0.787162	AT:0.212838
chr28	9142668	2	300	TAGAC:0.95	T:0.05
chr28	9142680	3	318	AAGAGAGAG:0.672956	A:
0.0754717	AAGAGAGAGAG:0.251572
chr28	9142841	3	248	TCC:0.745968	T:0.197581
TC:0.0564516
chr28	9142881	2	278	T:0.935252	TA:0.0647482
chr28	9143006	2	298	C:0.822148	T:0.177852
chr28	9143377	3	286	AT:0.678322	A:0.206294
ATT:0.115385
chr28	9143426	2	300	CAGAGAGAG:0.843333	C:
0.156667
chr28	9143525	2	298	A:0.822148	G:0.177852
chr28	9143900	2	306	TA:0.895425	T:0.104575
chr28	9144440	2	302	G:0.804636	T:0.195364
chr28	9144446	2	306	G:0.95098	A:0.0490196
chr28	9144686	2	302	A:0.956954	T:0.0430464
chr28	9145028	2	294	TGAAATATCTGGAAAAAAAAAAAAAAA:
0.853741	T:0.146259
chr28	9145032	2	294	ATATC:0.962585	A:0.037415
chr28	9145038	2	294	GGAAAAAAAAAAAAAAAGAAATA:0.962585
G:0.037415
chr28	9145148	2	270	CT:0.618519	C:0.381481
chr28	9145161	2	296	T:0.780405	A:0.219595
chr28	9145164	4	314	GATTTATTT:0.770701	G:
0.0987261	GATTT:0.0382166	GATTTATTTATTT:0.0923567
chr28	9145226	2	304	T:0.950658	TGAGA:0.0493421
chr28	9145643	2	316	C:0.946203	CAAGAT:0.0537975
chr28	9145790	2	312	CTCT:0.945513	C:0.0544872
chr28	9145810	2	312	T:0.945513	A:0.0544872
```

chr28	9145957	2	312	CACG:0.86859	C:0.13141
chr28	9146538	2	306	G:0.856209	A:0.143791
chr28	9146594	2	306	ATC:0.872549	A:0.127451
chr28	9146977	2	282	T:0.812057	A:0.187943
chr28	9147023	2	282	G:0.932624	T:0.0673759
chr28	9147135	2	298	TC:0.66443	T:0.33557
chr28	9147162	2	296	T:0.797297	C:0.202703
chr28	9147164	2	296	T:0.797297	C:0.202703
chr28	9147406	2	216	A:0.939815	G:0.0601852
chr28	9149465	2	250	TA:0.512 T:0.488	
chr28	9150836	2	290	GT:0.775862	G:0.224138
chr28	9153248	2	304	C:0.967105	T:0.0328947
chr28	9157090	2	296	GT:0.733108	G:0.266892
chr28	9157133	2	300	CAGAG:0.923333	C:0.0766667
chr28	9158152	2	280	AT:0.946429	A:0.0535714
chr28	9158201	2	280	GGA:0.839286	G:0.160714
chr28	9158997	2	296	TA:0.959459	T:0.0405405
chr28	9159272	3	302	AAG:0.850993	A:0.115894
AAGAGAG:0.0331126					
chr28	9160165	2	288	T:0.934028	TA:0.0659722
chr28	9160327	2	270	CTG:0.237037	C:0.762963
chr28	9160429	2	306	C:0.781046	T:0.218954
chr28	9160757	2	312	AC:0.990385	A:0.00961538
chr28	9161374	2	306	T:0.973856	A:0.0261438
chr28	9161866	2	312	C:0.958333	T:0.0416667
chr28	9162302	2	302	GGA:0.880795	G:0.119205
chr28	9163197	2	296	GA:0.152027	G:0.847973
chr28	9163247	2	298	A:0.765101	G:0.234899
chr28	9164020	2	318	C:0.974843	
CTTTGTTCTTCTTTTTTTTTTTTTT:0.0251572					
chr28	9164559	2	306	G:0.970588	GT:0.0294118
chr28	9164798	2	300	TA:0.696667	T:0.303333
chr28	9165080	3	306	TA:0.777778	T:0.199346
AA:0.0228758					
chr28	9165804	2	302	T:0.778146	C:0.221854
chr28	9166894	2	276	C:0.887681	CT:0.112319
chr28	9167364	2	284	C:0.323944	CT:0.676056
chr28	9167454	2	292	C:0.982877	T:0.0171233
chr28	9168441	2	256	CAG:0.53125	C:0.46875
chr28	9168549	2	282	A:0.496454	G:0.503546
chr28	9169426	2	312	A:0.958333	G:0.0416667
chr28	9170031	2	302	G:0.801325	A:0.198675
chr28	9170724	2	282	C:0.138298	CAGAG:0.861702
chr28	9171159	2	310	C:0.948387	T:0.0516129
chr28	9175560	2	306	T:0.964052	C:0.0359477
chr28	9175795	2	280	CT:0.828571	C:0.171429
chr28	9178631	2	302	T:0.990066	C:0.00993377
chr28	9180681	2	274	A:0.934307	G:0.0656934
chr28	9180806	2	302	C:0.953642	T:0.0463576
chr28	9180948	2	288	T:0.972222	G:0.0277778
chr28	9181107	2	312	C:0.980769	T:0.0192308
chr28	9181265	2	296	C:0.969595	T:0.0304054
chr28	9184306	3	298	CAG:0.644295	C:0.151007
CAGAG:0.204698					

chr28 9187030 2 290 CAAAT:0.458621 C:0.541379
chr28 9187804 2 290 G:0.824138 A:0.175862
chr28 9188964 2 306 T:0.944444 C:0.0555556
chr28 9190252 2 288 CT:0.927083 C:0.0729167
chr28 9190810 2 298 G:0.97651 A:0.0234899
chr28 9191746 2 288 G:0 T:1
chr28 9194302 2 308 C:0.974026 T:0.025974
chr28 9195381 2 306 T:0.990196 G:0.00980392
chr28 9195814 2 302 G:0.976821 A:0.0231788
chr28 9197247 2 308 G:0.993506 A:0.00649351
chr28 9200444 2 316 G:0.901899 A:0.0981013
chr28 9200919 2 268 CT:0.761194 C:0.238806
chr28 9202338 2 306 C:0.781046 T:0.218954
chr28 9202393 2 242 AT:0.652893 A:0.347107
chr28 9203008 4 310 CTTCTTTCT:0.358065 C:
0.167742 CTTCT:0.167742 CTTCTTTCTTTCT:0.306452
chr28 9203378 2 300 GT:0.71 G:0.29
chr28 9204202 2 292 GAC:0.821918 G:0.178082
chr28 9205805 2 312 CTTCTTCTTCTTCTCT:0.887821 C:
0.112179
chr28 9205814 2 308 CTTCTCT:0.948052 C:0.0519481
chr28 9208416 2 302 T:0.897351 A:0.102649
chr28 9208559 2 278 C:0.971223 T:0.028777
chr28 9208833 2 302 C:0.953642 T:0.0463576
chr28 9210336 2 302 C:0.986755 T:0.013245
chr28 9211793 2 266 AC:0 A:1
chr28 9214201 2 296 G:0.959459 A:0.0405405
chr28 9218725 2 306 C:0.957516 T:0.0424837
chr28 9220104 2 290 A:0.731034 AATTC:0.268966
chr28 9221330 2 262 T:0.351145 C:0.648855
chr28 9222795 2 304 C:0.947368 T:0.0526316
chr28 9224068 2 308 G:0.152597 A:0.847403
chr28 9224369 2 306 A:0.859477 G:0.140523
chr28 9225820 2 288 C:0.878472 G:0.121528
chr28 9225982 2 296 T:0.952703 G:0.0472973
chr28 9226256 2 306 TCAAA:0.764706 T:0.235294
chr28 9226379 2 296 G:0.952703 A:0.0472973
chr28 9226462 2 288 C:0.930556 T:0.0694444
chr28 9229209 2 308 C:0.983766 T:0.0162338
chr28 9231342 3 290 C:0.544828 CT:0.306897
CTT:0.148276
chr28 9234080 2 286 C:0.93007 T:0.0699301
chr28 9235371 2 290 G:0.786207 A:0.213793
chr28 9235627 2 306 G:0.96732 C:0.0326797
chr28 9236536 3 280 T:0.228571 TTG:0.435714
TTGTG:0.335714
chr28 9241046 2 264 T:0.829545 TAC:0.170455
chr28 9241915 2 308 G:0.961039 T:0.038961
chr28 9242810 2 310 C:0.980645 T:0.0193548
chr28 9245246 2 298 G:0.895973 A:0.104027
chr28 9245562 2 314 C:0.805732 CAG:0.194268
chr28 9245594 2 318 GAAACATAGGC:0.915094 G:
0.0849057
chr28 9245609 2 318 G:0.915094 A:0.0849057

chr28 9246068 3 252 CT:0.563492 C:0.218254
CTT:0.218254
chr28 9249971 2 262 GA:0.816794 G:0.183206
chr28 9252355 2 290 G:0.803448 GC:0.196552
chr28 9255857 2 296 A:0.905405 G:0.0945946
chr28 9256832 2 294 T:0.972789 C:0.0272109
chr28 9258279 2 290 T:0.962069 TTTTC:0.037931
chr28 9260451 2 296 TA:0.841216 T:0.158784
chr28 9262984 2 260 C:0.984615 A:0.0153846
chr28 9263081 2 296 T:0.956081 A:0.0439189
chr28 9263695 2 310 C:0.0709677 A:0.929032
chr28 9266238 4 314 TAAATAAAATA:0.401274 T:
0.343949 TAAATA:0.0923567 TAAATAAAATAAAATA:0.16242
chr28 9266559 2 310 C:0.941935 T:0.0580645
chr28 9268562 3 286 T:0.814685 TCA:0.104895
TCACA:0.0804196
chr28 9270413 2 310 T:0.883871 TA:0.116129
chr28 9270532 2 304 C:0.9375 G:0.0625
chr28 9271166 3 314 AAG:0.853503 A:0.130573
AAGAGAG:0.0159236
chr28 9274164 2 308 A:0.938312 T:0.0616883
chr28 9274623 2 290 G:0.982759 A:0.0172414
chr28 9274782 3 302 TTTTA:0.678808 T:0.0761589
TTTTATTTA:0.245033
chr28 9276305 2 298 G:0.983221 A:0.0167785
chr28 9279792 3 306 TTCTCTCTCTCTC:0.575163 T:
0.19281 TTCTCTC:0.232026
chr28 9279868 2 288 A:0.9375 T:0.0625
chr28 9279890 2 284 T:0.292254 TA:0.707746
chr28 9282265 2 300 C:0.993333 T:0.00666667
chr28 9286422 2 300 A:0.913333 G:0.0866667
chr28 9289479 2 298 G:0 GT:1
chr28 9289501 2 298 AG:0 A:1
chr28 9289508 2 298 C:0 CA:1
chr28 9289522 2 292 CT:0 C:1
chr28 9289527 2 292 C:0 CA:1
chr28 9289532 2 292 AT:0 A:1
chr28 9289540 2 294 A:0 AC:1
chr28 9289550 2 294 G:0 C:1
chr28 9289551 2 294 C:0 T:1
chr28 9289563 2 286 A:0.00699301 AT:0.993007
chr28 9289582 2 290 T:0.0137931 TTG:0.986207
chr28 9289586 2 290 A:0.0137931 AG:0.986207
chr28 9289594 2 290 A:0.00689655 AT:0.993103
chr28 9289602 2 284 C:0.00704225 CT:0.992958
chr28 9289611 2 284 C:0 CT:1
chr28 9289613 2 284 A:0 T:1
chr28 9289628 2 282 T:0 C:1
chr28 9289678 2 294 G:0 GA:1
chr28 9289765 3 290 GT:0.686207 G:0.162069
GTT:0.151724
chr28 9291028 2 282 G:0.85461 GA:0.14539
chr28 9292480 2 306 A:0.98366 G:0.0163399
chr28 9292702 2 292 A:0.883562 AT:0.116438

chr28	9293543	2	304	A:0.907895	T:0.0921053		
chr28	9294590	2	284	CT:0.785211	C:0.214789		
chr28	9295280	2	292	C:0.952055	CTGTG:0.0479452		
chr28	9296459	2	308	C:0.305195	A:0.694805		
chr28	9297124	2	308	TG:0	T:1		
chr28	9297705	2	288	CT:0.763889	C:0.236111		
chr28	9297733	2	286	T:0.79021	A:0.20979		
chr28	9297746	2	280	T:0.882143	G:0.117857		
chr28	9299280	2	246	T:0.841463	TA:0.158537		
chr28	9302028	2	294	G:0.969388	A:0.0306122		
chr28	9303306	2	308	C:0.967532	T:0.0324675		
chr28	9307062	3	264	TA:0.522727	T:0.223485	TAA:0.253788	
chr28	9307815	2	298	C:0.922819	T:0.0771812		
chr28	9310023	3	304	TA:0.832237	T:0.121711	TAA:0.0460526	
chr28	9310572	2	312	G:0.948718	A:0.0512821		
chr28	9313118	2	304	C:0.898026	T:0.101974		
chr28	9313791	2	276	TA:0.916667	T:0.0833333		
chr28	9314189	2	282	A:0.960993	T:0.0390071		
chr28	9314438	2	246	CATATATATAT:0.422764	C:0.577236		
chr28	9315188	2	300	AC:0.93	A:0.07		
chr28	9315208	2	302	A:0.996689	G:0.00331126		
chr28	9318528	2	208	TA:0.711538	T:0.288462		
chr28	9320292	2	296	G:0.986486	A:0.0135135		
chr28	9320305	2	288	TG:0.913194	T:0.0868056		
chr28	9320833	2	258	G:0.965116	GCA:0.0348837		
chr28	9322001	2	300	A:0.983333	G:0.0166667		
chr28	9323951	2	298	G:0.11745	A:0.88255		
chr28	9325691	2	312	A:0.996795	G:0.00320513		
chr28	9326224	2	304	C:0.865132	T:0.134868		
chr28	9330049	2	294	CCT:0.962585	C:0.037415		
chr28	9330093	2	298	TA:0.761745	T:0.238255		
chr28	9338162	2	300	CT:0.69	C:0.31		
chr28	9338671	2	304	T:0.875	C:0.125		
chr28	9339342	2	292	T:0.25	C:0.75		
chr28	9340687	2	310	A:0.932258	C:0.0677419		
chr28	9342346	2	306	CAGAG:0.993464	C:0.00653595		
chr28	9342381	2	302	G:0.857616	A:0.142384		
chr28	9344449	2	296	T:0.871622	G:0.128378		
chr28	9346223	2	294	C:0.97619	CAATA:0.0238095		
chr28	9346437	2	302	GA:0.950331	G:0.0496689		
chr28	9347895	3	300	T:0.243333	TTTTA:0.566667	TTTTATTTA:0.19	
chr28	9349792	2	310	T:0.990323	C:0.00967742		
chr28	9350405	2	302	A:0.960265	G:0.0397351		
chr28	9351340	4	308	TATAAATAA:0.282468	T:0.327922	TATAA:0.334416	TATAAATAAATAA:0.0551948
chr28	9352037	2	304	G:0.973684	C:0.0263158		
chr28	9355014	2	284	CCT:0.922535	C:0.0774648		
chr28	9356156	2	266	TA:0.706767	T:0.293233		
chr28	9356483	2	298	CT:0.889262	C:0.110738		
chr28	9357139	3	278	AT:0.798561	A:0.140288		

ATT:0.0611511
chr28	9357371	2	310	C:0	A:1
chr28	9357778	2	300	T:0.993333	TA:0.00666667
chr28	9359170	2	312	A:0.961538	T:0.0384615
chr28	9359227	2	314	GGCAGAGGGAGAA:0.93949	G:
0.0605096
chr28	9359764	2	276	TA:0.876812	T:0.123188
chr28	9360813	2	308	C:0.967532	T:0.0324675
chr28	9361289	2	294	T:0.142857	C:0.857143
chr28	9361760	2	294	CTG:0.931973	C:0.0680272
chr28	9361762	2	296	G:0.847973	C:0.152027
chr28	9362169	2	310	G:0.958065	A:0.0419355
chr28	9363389	2	314	T:0.990446	G:0.00955414
chr28	9363703	2	312	AAAAGAAAG:0.951923	A:
0.0480769
chr28	9363721	3	316	AAGAAAGAAAAAG:0.775316	A:
0.056962	AAAAGAAAAAG:0.167722
chr28	9363727	2	316	GAA:0.927215	G:0.0727848
chr28	9363729	2	320	A:0.765625	AAG:0.234375
chr28	9364175	2	302	T:0.857616	C:0.142384
chr28	9364724	2	300	G:0.98	A:0.02
chr28	9365482	2	312	CAA:0.961538	C:0.0384615
chr28	9365955	2	302	C:0	T:1
chr28	9366573	2	304	T:0.967105	A:0.0328947
chr28	9367828	2	302	A:0	T:1
chr28	9367875	2	294	TA:0.962585	T:0.037415
chr28	9367881	2	294	G:0.962585	C:0.037415
chr28	9368763	2	220	TA:0.727273	T:0.272727
chr28	9370512	2	296	A:0.75	G:0.25
chr28	9371129	2	186	G:0.193548	C:0.806452
chr28	9371139	2	184	TCCGCGCCGCCCGCGCCGC:0.266304
T:0.733696
chr28	9371311	2	226	C:0.362832	G:0.637168
chr28	9371383	2	250	G:0.148	T:0.852
chr28	9371461	2	250	C:0.8	G:0.2
chr28	9372170	2	304	A:0.940789	G:0.0592105
chr28	9372185	2	304	A:0.940789	G:0.0592105
chr28	9372781	2	270	CT:0.62963	C:0.37037
chr28	9374018	2	306	G:0.892157	C:0.107843
chr28	9374197	2	292	T:0.941781	C:0.0582192
chr28	9374225	2	290	T:0.941379	TTC:0.0586207
chr28	9374408	2	296	C:0.922297	T:0.0777027
chr28	9375292	2	304	A:0.0723684	C:0.927632
chr28	9375559	2	302	C:0.913907	CCTCT:0.0860927
chr28	9376063	2	308	A:0.974026	T:0.025974
chr28	9376620	2	300	A:0.93	T:0.07
chr28	9376724	2	304	A:0.9375	G:0.0625
chr28	9377246	2	302	TCA:0.950331	T:0.0496689
chr28	9377362	2	304	T:0.9375	A:0.0625
chr28	9377406	2	304	G:0.9375	A:0.0625
chr28	9378066	2	284	TA:0.855634	T:0.144366
chr28	9378352	2	310	T:0.948387	C:0.0516129
chr28	9379155	2	294	CT:0.853741	C:0.146259
chr28	9379714	2	308	AT:0.607143	A:0.392857

chr28	9379904	2	296	G:0.932432	A:0.0675676	
chr28	9379915	2	306	G:0.98366	A:0.0163399	
chr28	9381902	2	312	G:0.955128	A:0.0448718	
chr28	9382377	2	286	C:0.961538	T:0.0384615	
chr28	9382570	2	292	T:0.955479	C:0.0445205	
chr28	9382869	2	302	A:0.950331	G:0.0496689	
chr28	9383106	2	298	T:0.963087	C:0.0369128	
chr28	9383420	2	308	G:0.954545	T:0.0454545	
chr28	9383516	2	304	G:0.963816	A:0.0361842	
chr28	9383681	2	308	G:0.909091	A:0.0909091	
chr28	9384144	2	304	G:0.934211	T:0.0657895	
chr28	9384153	2	304	T:0.934211	C:0.0657895	
chr28	9384469	2	306	C:0.957516	CT:0.0424837	
chr28	9384854	2	302	C:0.94702	G:0.0529801	
chr28	9384896	2	296	CT:0.949324	C:0.0506757	
chr28	9385165	2	294	A:0.972789	G:0.0272109	
chr28	9385324	2	306	TG:0.95098	T:0.0490196	
chr28	9385492	2	302	G:0.933775	GT:0.0662252	
chr28	9385817	2	312	TAA:0.932692	T:0.0673077	
chr28	9385948	2	294	A:0.945578	G:0.0544218	
chr28	9386281	2	304	AGCT:0.950658	A:0.0493421	
chr28	9386287	2	304	T:0.950658	A:0.0493421	
chr28	9387287	2	296	C:0.952703	T:0.0472973	
chr28	9387331	2	300	C:0.95	T:0.05	
chr28	9387708	2	302	A:0.953642	T:0.0463576	
chr28	9387720	2	302	A:0.953642	T:0.0463576	
chr28	9387722	2	302	T:0.953642	TC:0.0463576	
chr28	9388020	2	306	T:0.941176	A:0.0588235	
chr28	9388110	2	296	C:0.952703	T:0.0472973	
chr28	9388356	2	284	C:0.957746	A:0.0422535	
chr28	9388423	2	302	T:0.970199	A:0.0298013	
chr28	9388498	2	292	AT:0.94863	A:0.0513699	
chr28	9388688	2	298	G:0.966443	A:0.033557	
chr28	9388702	2	298	A:0.966443	G:0.033557	
chr28	9388808	2	314	CTTCTG:0.961783	C:0.0382166	
chr28	9388923	2	302	G:0.996689	A:0.00331126	
chr28	9389027	2	298	C:0.946309	T:0.0536913	
chr28	9389031	2	298	A:0.946309	G:0.0536913	
chr28	9389127	2	316	T:0.990506	G:0.00949367	
chr28	9389456	2	300	TA:0.903333	T:0.0966667	
chr28	9389930	2	310	G:0.958065	A:0.0419355	
chr28	9390113	2	290	C:0.968966	G:0.0310345	
chr28	9390141	2	318	GTCTCTGCCTCTCTCTCTC:0.738994	G:0.261006	
chr28	9390148	2	316	C:0.952532	CCT:0.0474684	
chr28	9390206	2	292	AT:0.952055	A:0.0479452	
chr28	9390620	3	292	CTTT:0.280822	C:0.0616438	CTTTT:0.657534
chr28	9390863	2	312	G:0.891026	A:0.108974	
chr28	9390877	2	310	A:0.96129	G:0.0387097	
chr28	9391347	2	296	G:0.952703	T:0.0472973	
chr28	9391436	2	308	A:0.954545	G:0.0454545	
chr28	9391460	2	306	C:0.928105	A:0.0718954	
chr28	9391949	2	282	TAA:0.822695	T:0.177305	

chr28	9392036	2	302	T:0.966887	C:0.0331126
chr28	9392240	2	302	T:0.950331	C:0.0496689
chr28	9392571	2	298	G:0.949664	A:0.0503356
chr28	9392725	2	308	A:0.951299	G:0.0487013
chr28	9392832	2	314	G:0.961783	T:0.0382166
chr28	9393098	2	310	CGTTT:0.954839	C:0.0451613
chr28	9393371	2	312	G:0.945513	A:0.0544872
chr28	9393865	2	282	A:0.953901	AAT:0.0460993
chr28	9393906	2	292	A:0.958904	AC:0.0410959
chr28	9394201	2	286	C:0.961538	T:0.0384615
chr28	9394334	2	296	T:0.945946	TA:0.0540541
chr28	9394397	2	318	C:0.946541	CT:0.0534591
chr28	9394399	2	318	GCCCAGTTAA:0.946541	G:
0.0534591
chr28	9394573	2	296	G:0.969595	A:0.0304054
chr28	9394893	2	304	C:0.947368	G:0.0526316
chr28	9394988	2	294	C:0.816327	CT:0.183673
chr28	9395160	2	294	T:0.959184	A:0.0408163
chr28	9395301	2	304	G:0.953947	T:0.0460526
chr28	9395358	2	306	G:0.96732	T:0.0326797
chr28	9397180	2	290	TA:0.958621	T:0.0413793
chr28	9397221	2	304	T:0.960526	TG:0.0394737
chr28	9397328	2	304	C:0.950658	A:0.0493421
chr28	9397335	2	304	T:0.950658	A:0.0493421
chr28	9397356	2	298	CT:0.969799	C:0.0302013
chr28	9397587	2	300	C:0.956667	T:0.0433333
chr28	9397666	2	308	A:0.935065	T:0.0649351
chr28	9398030	2	298	A:0.963087	G:0.0369128
chr28	9398401	2	316	TA:0.955696	T:0.0443038
chr28	9398419	2	316	T:0.955696	TA:0.0443038
chr28	9398455	2	316	T:0.955696	G:0.0443038
chr28	9398642	2	280	A:0.95	T:0.05
chr28	9398643	2	280	A:0.95	C:0.05
chr28	9398660	2	280	A:0.95	G:0.05
chr28	9398733	2	284	C:0.957746	T:0.0422535
chr28	9399036	2	276	G:0.971014	A:0.0289855
chr28	9399350	2	312	CCT:0.426282	C:0.573718
chr28	9399378	2	312	AAATT:0.971154	A:0.0288462
chr28	9399604	2	302	T:0.94702	C:0.0529801
chr28	9399605	2	302	G:0.94702	A:0.0529801
chr28	9399623	2	302	G:0.94702	C:0.0529801
chr28	9399641	2	302	T:0.94702	C:0.0529801
chr28	9399673	2	280	G:0.957143	A:0.0428571
chr28	9399755	3	274	TA:0.605839	T:0.19708
TAA:0.19708
chr28	9399791	2	296	A:0.969595	C:0.0304054
chr28	9399927	2	306	A:0.947712	C:0.0522876
chr28	9400413	2	284	C:0.975352	T:0.0246479
chr28	9400472	2	314	T:0.955414	TG:0.044586
chr28	9400654	2	304	G:0.950658	A:0.0493421
chr28	9400813	2	306	GA:0.908497	G:0.0915033
chr28	9400892	2	306	C:0.954248	T:0.0457516
chr28	9401088	2	310	C:0.958065	A:0.0419355
chr28	9401781	2	318	A:0.959119	ATGTGTGTGTG:

0.0408805					
chr28	9401846	2	308	G:0.951299	A:0.0487013
chr28	9401855	2	310	G:0.980645	A:0.0193548
chr28	9402130	2	286	A:0.954545	AT:0.0454545
chr28	9402175	3	258	CT:0.577519	C:0.325581
CTT:0.0968992					
chr28	9402275	2	308	T:0.957792	C:0.0422078
chr28	9402316	2	308	T:0.951299	C:0.0487013
chr28	9402515	3	302	ATC:0.811258	A:0.145695
ATTCTC:0.0430464					
chr28	9402517	2	298	C:0.989933	T:0.0100671
chr28	9403020	2	306	A:0.95098	AT:0.0490196
chr28	9403055	2	300	AAC:0.953333	A:0.0466667
chr28	9403414	2	308	T:0.951299	C:0.0487013
chr28	9403486	2	308	T:0.954545	C:0.0454545
chr28	9403716	2	308	A:0.957792	G:0.0422078
chr28	9403888	2	296	C:0.932432	T:0.0675676
chr28	9403982	2	300	G:0.96 C:0.04	
chr28	9404223	2	292	AAT:0.684932	A:0.315068
chr28	9404561	2	302	G:0.94702	A:0.0529801
chr28	9404634	2	306	C:0.954248	T:0.0457516
chr28	9404838	2	298	T:0.946309	C:0.0536913
chr28	9404885	2	312	T:0.958333	G:0.0416667
chr28	9405333	2	310	A:0.948387	G:0.0516129
chr28	9405405	2	296	C:0.952703	A:0.0472973
chr28	9405444	2	300	G:0.946667	T:0.0533333
chr28	9405823	2	304	A:0.960526	G:0.0394737
chr28	9405940	2	298	G:0.956376	GA:0.0436242
chr28	9406050	2	298	CT:0.956376	C:0.0436242
chr28	9406264	2	314	G:0.958599	A:0.0414013
chr28	9406371	2	294	C:0.962585	G:0.037415
chr28	9406496	2	302	T:0.963576	G:0.0364238
chr28	9406604	2	300	A:0.956667	G:0.0433333
chr28	9406877	2	298	C:0.956376	T:0.0436242
chr28	9406943	2	294	G:0.965986	A:0.0340136
chr28	9407001	2	300	GTC:0.89 G:0.11	
chr28	9407048	2	300	TA:0.823333	T:0.176667
chr28	9407144	2	300	G:0.943333	A:0.0566667
chr28	9407251	2	296	G:0.945946	A:0.0540541
chr28	9407317	2	298	CT:0.902685	C:0.0973154
chr28	9407363	2	296	C:0.959459	CAGAG:0.0405405
chr28	9407435	2	292	T:0.965753	C:0.0342466
chr28	9407708	2	306	A:0.96732	G:0.0326797
chr28	9408117	2	306	A:0.964052	G:0.0359477
chr28	9408844	2	298	T:0.959732	C:0.0402685
chr28	9409094	5	314	TATAA:0.375796	T:0.197452
TATAAATAA:0.296178			TATAAATAAATAA:0.111465		
TATAAATAAATAAATAA:0.0191083					
chr28	9409136	2	318	T:0.918239	TAAATAAAA:
0.081761					
chr28	9409832	2	294	A:0.94898	G:0.0510204
chr28	9412296	2	312	G:0.955128	A:0.0448718
chr28	9412365	2	298	C:0.956376	T:0.0436242
chr28	9412764	2	302	T:0.960265	A:0.0397351

chr28	9413209	2	230	C:0.952174	CCT:0.0478261	
chr28	9414925	2	274	TA:0.894161	T:0.105839	
chr28	9414938	2	282	TTAA:0.964539	T:0.035461	
chr28	9414939	2	272	TA:0.0551471	T:0.944853	
chr28	9414983	2	296	A:0	G:1	
chr28	9415014	2	304	A:0	G:1	
chr28	9415036	2	304	A:0	G:1	
chr28	9415125	2	284	A:0	C:1	
chr28	9415544	2	286	T:0.968531	G:0.0314685	
chr28	9416238	2	318	C:0.446541	CA:0.553459	
chr28	9416347	2	318	C:0.446541	CA:0.553459	
chr28	9416349	2	318	C:0.446541	CA:0.553459	
chr28	9416351	2	318	G:0.446541	GA:0.553459	
chr28	9416355	2	318	C:0.446541	CT:0.553459	
chr28	9417023	2	306	GTTA:0.941176	G:0.0588235	
chr28	9417266	2	302	G:0.953642	A:0.0463576	
chr28	9417400	2	308	C:0.954545	T:0.0454545	
chr28	9417497	2	298	C:0.963087	G:0.0369128	
chr28	9418472	2	304	A:0.957237	T:0.0427632	
chr28	9418536	2	286	A:0.965035	G:0.034965	
chr28	9419145	2	292	T:0.962329	C:0.0376712	
chr28	9419440	3	292	CATATAT:0.260274	C:0.10274	CATAT:0.636986
chr28	9420740	2	306	T:0.924837	A:0.0751634	
chr28	9422117	3	278	TAA:0.165468	T:0.133094	TA:0.701439
chr28	9422780	2	310	GTTTGTT:0.945161	G:0.0548387	
chr28	9423180	2	296	T:0.952703	A:0.0472973	
chr28	9423467	2	304	A:0.960526	G:0.0394737	
chr28	9423648	2	302	C:0.960265	T:0.0397351	
chr28	9423833	2	292	C:0.982877	CT:0.0171233	
chr28	9423839	2	290	T:0.937931	TG:0.062069	
chr28	9423840	2	292	G:0.678082	T:0.321918	
chr28	9425194	2	290	T:0.951724	G:0.0482759	
chr28	9425684	2	302	C:0.950331	T:0.0496689	
chr28	9425738	2	300	C:0.95	A:0.05	
chr28	9425879	2	288	TA:0.958333	T:0.0416667	
chr28	9425882	2	288	T:0.958333	C:0.0416667	
chr28	9425956	2	302	G:0.963576	T:0.0364238	
chr28	9426387	2	304	A:0.950658	G:0.0493421	
chr28	9426951	2	294	T:0.982993	A:0.0170068	
chr28	9427212	2	314	C:0.958599	T:0.0414013	
chr28	9427415	2	300	A:0.956667	C:0.0433333	
chr28	9427767	2	306	A:0.957516	T:0.0424837	
chr28	9428233	2	310	T:0.954839	C:0.0451613	
chr28	9428369	2	302	G:0.960265	A:0.0397351	
chr28	9428449	2	300	G:0.963333	C:0.0366667	
chr28	9428465	2	302	C:0.966887	T:0.0331126	
chr28	9428611	2	292	ATCTCCCTGGTG:0	A:1	
chr28	9428625	2	292	TATCTTTC:0	T:1	
chr28	9428633	2	292	C:0	CAAAAAAAAAAAAAAAAA:1	
chr28	9428636	2	292	C:0	A:1	
chr28	9428727	2	290	C:0	A:1	
chr28	9428782	2	298	G:0	A:1	

chr28	9428833	2	304	C:0.940789	T:0.0592105	
chr28	9428852	2	306	C:0	CA:1	
chr28	9428890	2	310	G:0.954839	C:0.0451613	
chr28	9428914	2	310	T:0.954839	C:0.0451613	
chr28	9428931	2	310	T:0	A:1	
chr28	9429394	2	320	A:0.95625	T:0.04375	
chr28	9429401	2	320	A:0.95625	ATAAATAAATAAATAAATAAATAAATAAATAAAT:0.04375	
chr28	9429409	2	320	G:0.95625	T:0.04375	
chr28	9429422	2	320	C:0.95625	G:0.04375	
chr28	9429460	2	310	G:0.96129	A:0.0387097	
chr28	9429511	2	300	G:0.956667	C:0.0433333	
chr28	9429518	2	300	A:0.976667	G:0.0233333	
chr28	9429977	2	296	C:0.942568	T:0.0574324	
chr28	9430651	2	264	TA:0.94697	T:0.0530303	
chr28	9431418	2	312	T:0.967949	G:0.0320513	
chr28	9431600	2	306	T:0.947712	C:0.0522876	
chr28	9431728	2	298	TG:0.939597	T:0.0604027	
chr28	9431746	2	302	G:0.950331	T:0.0496689	
chr28	9431863	2	298	C:0.956376	T:0.0436242	
chr28	9431864	2	298	C:0.956376	G:0.0436242	
chr28	9431869	2	298	G:0.956376	T:0.0436242	
chr28	9432029	2	300	G:0.956667	GAC:0.0433333	
chr28	9432086	2	298	G:0.966443	C:0.033557	
chr28	9432105	2	298	C:0.966443	T:0.033557	
chr28	9432107	2	298	T:0.966443	A:0.033557	
chr28	9432244	2	314	C:0.977707	T:0.022293	
chr28	9432247	2	314	C:0.977707	CGGGATCCCTGGGTGGCGCAGCGGTTT:0.022293	
chr28	9432303	2	288	T:0.96875	C:0.03125	
chr28	9432351	2	304	A:0.970395	G:0.0296053	
chr28	9432764	2	310	A:0.951613	T:0.0483871	
chr28	9432963	2	304	G:0.980263	C:0.0197368	
chr28	9433378	2	304	CAAAT:0.957237	C:0.0427632	
chr28	9433708	2	300	T:0.946667	C:0.0533333	
chr28	9433904	2	304	C:0.953947	T:0.0460526	
chr28	9434848	2	298	C:0.959732	T:0.0402685	
chr28	9435007	2	300	G:0.95	A:0.05	
chr28	9435282	2	304	A:0.944079	T:0.0559211	
chr28	9435329	2	310	G:0.951613	A:0.0483871	
chr28	9435369	2	304	G:0.944079	T:0.0559211	
chr28	9435417	2	300	A:0.946667	C:0.0533333	
chr28	9435633	2	292	G:0.952055	A:0.0479452	
chr28	9435984	2	286	G:0.755245	GTT:0.244755	
chr28	9436029	2	310	T:0.954839	C:0.0451613	
chr28	9436182	2	300	C:0.963333	T:0.0366667	
chr28	9436385	2	308	A:0.964286	G:0.0357143	
chr28	9436531	2	298	C:0.942953	T:0.057047	
chr28	9437044	2	318	GAAAAAAAGA:0.940252	G: 0.0597484	
chr28	9437167	3	314	G:0.796178	GAAGA:0.0605096	GAAGAAAGAAAGAAAGA:0.143312
chr28	9437220	2	312	AAG:0.974359	A:0.025641	
chr28	9438308	2	306	T:0.95098	C:0.0490196	

chr28	9438570	2	300	A:0.95	G:0.05	
chr28	9438598	2	298	A:0.956376	G:0.0436242	
chr28	9439112	2	308	G:0.954545	A:0.0454545	
chr28	9439383	2	310	A:0.954839	C:0.0451613	
chr28	9439813	2	310	C:0.951613	T:0.0483871	
chr28	9439816	2	310	C:0.951613	T:0.0483871	
chr28	9439872	2	310	G:0.951613	A:0.0483871	
chr28	9440086	2	308	G:0.961039	A:0.038961	
chr28	9440269	2	300	T:0.95	C:0.05	
chr28	9440301	2	308	C:0.954545	T:0.0454545	
chr28	9440404	2	286	T:0.793706	TA:0.206294	
chr28	9441330	2	300	T:0.953333	C:0.0466667	
chr28	9441547	3	304	ATT:0.786184	A:0.0559211	AT:0.157895
chr28	9441578	2	302	A:0.956954	T:0.0430464	
chr28	9441710	2	290	CAG:0.931034	C:0.0689655	
chr28	9441745	2	294	G:0.952381	T:0.047619	
chr28	9441756	2	294	C:0.952381	T:0.047619	
chr28	9442441	2	122	G:0	GC:1	
chr28	9442473	2	260	A:0	AG:1	
chr28	9442561	2	310	A:0.00322581	C:0.996774	
chr28	9442666	2	306	G:0.895425	A:0.104575	
chr28	9442971	2	310	C:0.951613	T:0.0483871	
chr28	9443556	2	310	CTTTT:0.958065	C:0.0419355	
chr28	9443584	2	310	C:0.958065	T:0.0419355	
chr28	9443764	2	294	A:0.94898	G:0.0510204	
chr28	9443880	2	308	A:0.961039	T:0.038961	
chr28	9444011	2	288	G:0.965278	A:0.0347222	
chr28	9444061	2	302	ATGT:0.953642	A:0.0463576	
chr28	9444079	2	302	T:0.953642	C:0.0463576	
chr28	9444233	2	308	T:0.996753	A:0.00324675	
chr28	9444234	2	308	T:0.912338	A:0.0876623	
chr28	9444364	2	294	A:0.952381	T:0.047619	
chr28	9444470	2	310	C:0.958065	G:0.0419355	
chr28	9445035	2	304	G:0.957237	A:0.0427632	
chr28	9445149	2	312	T:0.958333	G:0.0416667	
chr28	9445583	2	318	GTTCATTTCTCC:0.924528	G:0.0754717	
chr28	9445639	2	308	C:0	CA:1	
chr28	9445772	2	300	T:0.946667	G:0.0533333	
chr28	9445921	2	294	C:0.952381	G:0.047619	
chr28	9445978	2	300	GACAA:0.96	G:0.04	
chr28	9445984	2	300	C:0.96	T:0.04	
chr28	9446204	2	308	A:0.967532	G:0.0324675	
chr28	9446344	2	310	A:0.996774	T:0.00322581	
chr28	9446623	3	290	GACAC:0.258621	G:0.582759	GAC:0.158621
chr28	9447360	2	306	T:0.957516	C:0.0424837	
chr28	9448027	2	296	C:0	CT:1	
chr28	9448308	2	308	T:0.886364	C:0.113636	
chr28	9448777	2	294	A:0.945578	G:0.0544218	
chr28	9448821	2	290	A:0.944828	T:0.0551724	
chr28	9448828	2	290	G:0.944828	A:0.0551724	
chr28	9449544	2	310	A:0.948387	G:0.0516129	

chr28	9449897	2	314	G:0.942675	A:0.0573248	
chr28	9449905	2	314	A:0.942675	G:0.0573248	
chr28	9450169	2	296	A:0.962838	G:0.0371622	
chr28	9450262	2	308	A:0.944805	G:0.0551948	
chr28	9450367	2	306	C:0.964052	T:0.0359477	
chr28	9450609	2	302	G:0.953642	A:0.0463576	
chr28	9450610	2	302	T:0.953642	A:0.0463576	
chr28	9450900	2	320	G:0.95	A:0.05	
chr28	9451000	2	304	T:0.960526	G:0.0394737	
chr28	9451020	2	304	A:0.960526	G:0.0394737	
chr28	9451201	2	312	T:0.948718	C:0.0512821	
chr28	9451362	2	300	T:0.95	C:0.05	
chr28	9451900	2	306	G:0.95098	T:0.0490196	
chr28	9452012	2	292	G:0.965753	A:0.0342466	
chr28	9452028	2	288	T:0.934028	A:0.0659722	
chr28	9452129	2	296	T:0.952703	C:0.0472973	
chr28	9452233	2	286	A:0.958042	G:0.041958	
chr28	9452270	2	264	G:0.950758	A:0.0492424	
chr28	9452314	2	234	TG:0.858974	T:0.141026	
chr28	9452427	2	288	G:0.954861	A:0.0451389	
chr28	9452712	2	284	A:0.954225	G:0.0457746	
chr28	9452734	2	286	T:0.947552	G:0.0524476	
chr28	9453163	3	318	C:0.345912	CA:0.559748	CAA:0.0943396
chr28	9453932	2	318	TTATCTCTAAAA:0.927673	T: 0.072327	
chr28	9454106	2	294	A:0.959184	T:0.0408163	
chr28	9454107	2	294	A:0.959184	T:0.0408163	
chr28	9454196	2	300	C:0.963333	T:0.0366667	
chr28	9454395	2	298	T:0.942953	TA:0.057047	
chr28	9454706	2	306	TC:0.95098	T:0.0490196	
chr28	9454805	2	304	C:0.950658	T:0.0493421	
chr28	9455025	2	304	A:0.953947	C:0.0460526	
chr28	9455167	2	292	T:0.958904	C:0.0410959	
chr28	9455343	2	306	C:0.947712	T:0.0522876	
chr28	9455449	2	308	A:0.941558	G:0.0584416	
chr28	9455723	2	300	A:0.96	G:0.04	
chr28	9455757	2	296	T:0.952703	C:0.0472973	
chr28	9455869	2	308	T:0.957792	C:0.0422078	
chr28	9455902	2	298	G:0.956376	A:0.0436242	
chr28	9456042	2	274	C:0.952555	A:0.0474453	
chr28	9456143	2	286	C:0.951049	A:0.048951	
chr28	9456168	2	308	C:0.961039	CTGTT:0.038961	
chr28	9456189	2	308	C:0.961039	T:0.038961	
chr28	9456651	2	312	C:0.948718	A:0.0512821	
chr28	9456873	2	298	A:0.95302	T:0.0469799	
chr28	9456996	2	316	A:0.939873	AGTCAGAATTG: 0.0601266	
chr28	9457229	2	302	C:0.950331	T:0.0496689	
chr28	9457587	2	302	A:0.956954	ATACT:0.0430464	
chr28	9457713	2	306	C:0.954248	T:0.0457516	
chr28	9457811	2	316	T:0.955696	TAGAAACTTACTGTTTTAAAAAAAATAAATAAATAAAATAAAATAAAAAAAAAA:0.0443038	
chr28	9457928	2	308	C:0.954545	G:0.0454545	

chr28	9457936	2	308	A:0.954545	G:0.0454545	
chr28	9458001	2	304	T:0.944079	A:0.0559211	
chr28	9458292	2	304	A:0.957237	G:0.0427632	
chr28	9458778	2	288	G:0.954861	T:0.0451389	
chr28	9458795	2	284	T:0.957746	C:0.0422535	
chr28	9459245	2	310	G:0.941935	T:0.0580645	
chr28	9459498	2	302	CA:0.943709	C:0.0562914	
chr28	9459828	2	310	A:0.948387	G:0.0516129	
chr28	9459891	2	312	A:0.961538	C:0.0384615	
chr28	9460192	2	304	T:0.957237	C:0.0427632	
chr28	9461215	2	306	A:0.947712	G:0.0522876	
chr28	9461439	2	304	A:0.950658	G:0.0493421	
chr28	9462044	2	284	A:0.978873	T:0.0211268	
chr28	9462052	3	290	A:0.827586	T:0.0310345	AT:0.141379
chr28	9462054	3	290	T:0.313793	A:0.0586207	TA:0.627586
chr28	9462408	2	298	G:0.963087	A:0.0369128	
chr28	9462535	2	298	A:0.946309	G:0.0536913	
chr28	9462713	2	308	G:0.961039	T:0.038961	
chr28	9462752	2	312	G:0.948718	T:0.0512821	
chr28	9462942	2	306	A:0.96732	T:0.0326797	
chr28	9463848	2	298	G:0.949664	C:0.0503356	
chr28	9463906	2	292	CCT:0.921233	C:0.0787671	
chr28	9463999	2	310	T:0.958065	A:0.0419355	
chr28	9464086	2	304	CAA:0.963816	C:0.0361842	
chr28	9464290	2	282	C:0.897163	CAAT:0.102837	
chr28	9464350	2	304	G:0.957237	A:0.0427632	
chr28	9464386	3	300	GATAT:0.743333	G:0.0666667	GAT:0.19
chr28	9465510	2	302	C:0.953642	A:0.0463576	
chr28	9465524	3	290	G:0.524138	GATTT:0.258621	GATTTATTT:0.217241
chr28	9465616	2	284	G:0.982394	A:0.0176056	
chr28	9465675	2	274	G:0.959854	A:0.040146	
chr28	9466782	2	308	T:0.957792	C:0.0422078	
chr28	9466972	2	302	G:0.94702	A:0.0529801	
chr28	9467047	2	308	G:0.938312	A:0.0616883	
chr28	9467215	2	302	AG:0.94702	A:0.0529801	
chr28	9467298	2	288	C:0.9375	T:0.0625	
chr28	9467639	2	306	G:0.957516	T:0.0424837	
chr28	9467695	2	308	G:0.954545	A:0.0454545	
chr28	9467980	2	308	C:0.961039	T:0.038961	
chr28	9468105	2	302	T:0.940397	C:0.0596026	
chr28	9468372	2	298	C:0	A:1	
chr28	9468424	2	290	G:0	T:1	
chr28	9468495	2	256	G:0.765625	T:0.234375	
chr28	9468515	2	208	GC:0.8125	G:0.1875	
chr28	9468894	2	304	A:0.953947	G:0.0460526	
chr28	9468919	2	302	T:0.960265	G:0.0397351	
chr28	9469082	2	292	A:0.993151	C:0.00684932	
chr28	9469240	2	290	T:0.951724	C:0.0482759	
chr28	9469513	2	302	C:0.950331	T:0.0496689	
chr28	9469663	2	310	G:0.958065	A:0.0419355	

chr28 9469964 2 308 A:0.948052 C:0.0519481
chr28 9470952 2 302 A:0.940397 G:0.0596026
chr28 9471337 2 298 T:0.963087 C:0.0369128
chr28 9471374 2 290 A:0.965517 T:0.0344828
chr28 9471647 2 304 T:0.953947 C:0.0460526
chr28 9471683 2 290 C:0.965517 T:0.0344828
chr28 9471820 3 302 TAAA:0.513245 T:0.0331126
TAA:0.453642
chr28 9471824 2 300 A:0.97 T:0.03
chr28 9471834 2 300 A:0.97 T:0.03
chr28 9471878 2 270 TA:0.885185 T:0.114815
chr28 9472139 2 314 G:0.94586 GAATA:0.0541401
chr28 9472160 2 314 A:0.94586 C:0.0541401
chr28 9472164 3 314 TA:0.83758 T:0.10828
TAA:0.0541401
chr28 9472507 2 298 A:0.949664 T:0.0503356
chr28 9472509 2 298 A:0.949664 ATT:0.0503356
chr28 9472687 2 294 TAA:0.972789 T:0.0272109
chr28 9472771 2 302 T:0.953642 C:0.0463576
chr28 9472811 2 298 T:0.949664 C:0.0503356
chr28 9472976 2 306 C:0.95098 T:0.0490196
chr28 9473107 2 304 G:0.8125 T:0.1875
chr28 9473232 2 306 T:0.405229 C:0.594771
chr28 9473467 2 298 C:0.956376 G:0.0436242
chr28 9473630 2 294 G:0.969388 A:0.0306122
chr28 9473921 2 312 C:0.945513 T:0.0544872
chr28 9474078 2 304 A:0.963816 T:0.0361842
chr28 9474098 2 300 GT:0.91 G:0.09
chr28 9474243 2 304 G:0.957237 A:0.0427632
chr28 9474274 2 296 A:0.949324 G:0.0506757
chr28 9474534 2 304 C:0.957237 G:0.0427632
chr28 9475305 2 308 C:0.948052 A:0.0519481
chr28 9475872 2 306 T:0.977124 TA:0.0228758
chr28 9475873 2 306 A:0.964052 T:0.0359477
chr28 9477403 2 302 C:0.963576 G:0.0364238
chr28 9477706 2 296 C:0.898649 A:0.101351
chr28 9480667 2 298 T:0.946309 C:0.0536913
chr28 9481732 2 314 G:0.971338 T:0.0286624
chr28 9484016 2 300 T:0.886667 C:0.113333
chr28 9484192 2 308 G:0.905844 GTA:0.0941558
chr28 9484730 2 314 T:0.977707 C:0.022293
chr28 9485452 2 294 GTA:0.952381 G:0.047619
chr28 9485454 2 296 A:0.945946 G:0.0540541
chr28 9485472 2 294 C:0.904762 T:0.0952381
chr28 9486159 4 310 ATATTTATT:0.274194 A:
0.296774 ATATT:0.283871 ATATTTATTTATT:0.145161
chr28 9486719 3 300 TGA:0.82 T:0.123333 TGAGA:
0.0566667
chr28 9487304 2 308 A:0.961039 C:0.038961
chr28 9488801 2 314 AAG:0.936306 A:0.0636943
chr28 9489052 2 204 AT:0.72549 A:0.27451
chr28 9489128 2 298 C:0.966443 T:0.033557
chr28 9489474 2 298 T:0 A:1
chr28 9490421 2 310 C:0.880645 T:0.119355

chr28 9492278 2 306 G:0.888889 T:0.111111
chr28 9492364 2 306 GC:0.862745 G:0.137255
chr28 9492496 2 238 TG:0.537815 T:0.462185
chr28 9492789 2 286 AT:0.283217 A:0.716783
chr28 9492936 2 306 C:0.879085 A:0.120915
chr28 9493004 2 310 G:0.945161 T:0.0548387
chr28 9493238 3 308 TTTTATTTA:0.746753 T:
0.201299 TTTTA:0.0519481
chr28 9493368 2 296 G:0.635135 A:0.364865
chr28 9493572 2 298 C:0.614094 T:0.385906
chr28 9493903 2 310 C:0.587097 T:0.412903
chr28 9494588 2 304 C:0.861842 T:0.138158
chr28 9494885 2 314 CTATGTTTCCTAGT:0.378981 C:
0.621019
chr28 9494918 2 314 C:0.955414 T:0.044586
chr28 9495016 2 294 C:0.853741 G:0.146259
chr28 9495288 2 252 G:0.416667 T:0.583333
chr28 9495331 2 258 A:0.383721 AT:0.616279
chr28 9495351 2 264 TTTTAAATTA:0.893939 T:
0.106061
chr28 9495479 2 240 T:0.554167 A:0.445833
chr28 9495505 2 264 C:0.420455 A:0.579545
chr28 9495512 2 264 C:0.420455 A:0.579545
chr28 9495626 2 284 C:0.454225 CACAG:0.545775
chr28 9495731 2 282 A:0.0531915 G:0.946809
chr28 9495755 2 294 G:0.585034 A:0.414966
chr28 9495778 2 290 G:0.575862 A:0.424138
chr28 9496008 2 292 T:0.60274 TATG:0.39726
chr28 9496100 2 266 C:0.556391 T:0.443609
chr28 9496179 2 292 GATT:0.876712 G:0.123288
chr28 9496208 2 288 AG:0.402778 A:0.597222
chr28 9496209 2 288 GGAGA:0.604167 G:0.395833
chr28 9496213 2 288 A:0.402778 AG:0.597222
chr28 9496260 2 276 G:0.427536 T:0.572464
chr28 9496284 3 276 T:0.427536 C:0.442029
G:0.130435
chr28 9496489 2 290 CT:0.6 C:0.4
chr28 9496593 2 284 A:0.588028 G:0.411972
chr28 9498183 2 304 A:0.615132 G:0.384868
chr28 9498273 2 306 A:0.601307 T:0.398693
chr28 9498387 2 294 A:0.629252 G:0.370748
chr28 9498587 3 294 A:0.394558 AT:0.455782
ATT:0.14966
chr28 9498665 2 302 A:0.486755 G:0.513245
chr28 9498680 2 302 G:0.639073 A:0.360927
chr28 9498726 2 300 T:0.406667 G:0.593333
chr28 9498764 2 294 G:0.840136 A:0.159864
chr28 9498915 3 288 GT:0.444444 G:0.322917
GTT:0.232639
chr28 9499084 2 310 A:0.635484 AGTTGTT:0.364516
chr28 9499151 2 296 C:0.949324 T:0.0506757
chr28 9499210 2 298 G:0.469799 GAA:0.530201
chr28 9499407 2 286 GA:0.986014 G:0.013986
chr28 9499490 2 300 G:0.636667 T:0.363333

chr28	9499610	2	306	C:0.385621	T:0.614379	
chr28	9499889	2	312	T:0.387821	G:0.612179	
chr28	9499975	2	298	C:0.392617	A:0.607383	
chr28	9500610	2	304	A:0.615132	G:0.384868	
chr28	9500809	2	306	A:0.611111	G:0.388889	
chr28	9501246	2	290	C:0.589655	T:0.410345	
chr28	9501285	2	286	T:0.562937	C:0.437063	
chr28	9501415	3	312	T:0.413462	TTA:0.233974	TTTA:0.352564
chr28	9501431	2	312	AT:0.410256	A:0.589744	
chr28	9501440	2	312	T:0.410256	A:0.589744	
chr28	9501715	2	306	CT:0.388889	C:0.611111	
chr28	9501902	2	308	T:0.38961	C:0.61039	
chr28	9502402	2	310	G:0.412903	A:0.587097	
chr28	9502449	2	312	C:0.846154	T:0.153846	
chr28	9503233	2	308	T:0.402597	C:0.597403	
chr28	9503303	2	300	A:0.863333	G:0.136667	
chr28	9503721	2	282	C:0.865248	T:0.134752	
chr28	9503885	2	304	T:0.536184	G:0.463816	
chr28	9504296	2	306	T:0.604575	C:0.395425	
chr28	9504525	2	306	A:0.509804	C:0.490196	
chr28	9505005	2	304	T:0.598684	C:0.401316	
chr28	9505226	2	304	A:0.601974	G:0.398026	
chr28	9505395	2	304	A:0.618421	AAAT:0.381579	
chr28	9505549	2	316	CTCCTTCCTTTCCTGTATCTTTATGCT:0.398734	C:0.601266	
chr28	9505860	2	290	T:0.493103	C:0.506897	
chr28	9506135	2	300	T:0.613333	A:0.386667	
chr28	9506136	2	300	C:0.613333	A:0.386667	
chr28	9506513	2	310	T:0.393548	A:0.606452	
chr28	9506791	2	312	G:0.384615	A:0.615385	
chr28	9507084	2	306	A:0.614379	G:0.385621	
chr28	9507340	2	314	T:0.621019	TTCTCTCTCTC:0.378981	
chr28	9508167	2	290	T:0.655172	C:0.344828	
chr28	9508173	2	298	T:0.993289	G:0.00671141	
chr28	9508449	2	296	A:0.611486	G:0.388514	
chr28	9508663	2	304	G:0.792763	A:0.207237	
chr28	9508770	2	296	G:0.618243	A:0.381757	
chr28	9509126	2	304	G:0.473684	A:0.526316	
chr28	9509282	2	304	T:0.460526	C:0.539474	
chr28	9509465	2	302	C:0.596026	T:0.403974	
chr28	9509494	2	304	T:0.401316	A:0.598684	
chr28	9510303	2	308	C:0.405844	CT:0.594156	
chr28	9510340	2	282	GTTTT:0.421986	G:0.578014	
chr28	9510675	3	300	TAA:0.5	T:0.36	TA:0.14
chr28	9511120	2	240	AT:0.9	A:0.1	
chr28	9511125	3	242	T:0.119835	G:0.466942	TG:0.413223
chr28	9511342	2	268	CTTT:0.58209	C:0.41791	
chr28	9511791	2	314	T:0.863057	C:0.136943	
chr28	9512450	3	292	CCTCTCT:0.688356	C:0.19863	CCTCT:0.113014
chr28	9512527	2	296	A:0.577703	C:0.422297	

chr28	9512624	2	306	A:0.457516	C:0.542484			
chr28	9512645	2	304	TG:0.628289	T:0.371711			
chr28	9513488	5	312	GGT:0.365385	G:0.160256	GGTGT:0.0673077	GGTGTGTGT:0.144231	GGTGTGTGTGT:0.262821
chr28	9513529	2	320	TTGATTTGCATTTCTCTGA:0.840625	T:0.159375			
chr28	9513642	2	280	CTT:0.485714	C:0.514286			
chr28	9513673	2	290	T:0.413793	C:0.586207			
chr28	9513850	2	292	A:0.366438	G:0.633562			
chr28	9513900	2	252	C:0.59127	CTT:0.40873			
chr28	9514092	2	296	C:0.962838	T:0.0371622			
chr28	9514247	2	308	A:0.876623	G:0.123377			
chr28	9514276	2	292	C:0.623288	T:0.376712			
chr28	9514391	3	288	CCT:0.555556	C:0.322917	CCTCT:0.121528		
chr28	9515554	2	310	C:0.816129	T:0.183871			
chr28	9516333	2	302	A:0.264901	ATAG:0.735099			
chr28	9517458	2	306	G:0.784314	T:0.215686			
chr28	9517894	2	264	GA:0.94697	G:0.0530303			
chr28	9517899	2	292	GAAAAGAAAAGAAAAGA:0.780822	G:0.219178			
chr28	9517904	2	288	GAAAAGAAAAGA:0.809028	G:0.190972			
chr28	9517909	2	316	GAAAAGA:0.832278	G:0.167722			
chr28	9517914	2	316	GA:0.939873	G:0.0601266			
chr28	9517928	3	316	AAAAAGAAAAG:0.443038	A:0.338608	AAAAAG:0.218354		
chr28	9518095	2	282	GAGAA:0.897163	G:0.102837			
chr28	9518139	2	310	AAAAGAAAGAAAG:0.845161	A:0.154839			
chr28	9518438	2	278	G:0.766187	T:0.233813			
chr28	9518562	2	304	C:0.493421	CT:0.506579			
chr28	9518567	2	306	T:0.513072	TA:0.486928			
chr28	9518776	2	260	G:0.826923	C:0.173077			
chr28	9518823	4	296	TAAAA:0.462838	T:0.0675676	TAA:0.226351	TAAA:0.243243	
chr28	9518931	2	302	AC:0.298013	A:0.701987			
chr28	9518934	2	302	TGGA:0.298013	T:0.701987			
chr28	9520117	2	310	C:0.903226	T:0.0967742			
chr28	9520496	2	312	C:0.464744	G:0.535256			
chr28	9520512	2	312	G:0.464744	A:0.535256			
chr28	9520561	2	298	A:0.275168	T:0.724832			
chr28	9520834	2	300	CA:0.276667	C:0.723333			
chr28	9520922	2	296	G:0.486486	T:0.513514			
chr28	9520997	2	282	G:0.755319	A:0.244681			
chr28	9521353	2	288	T:0.802083	A:0.197917			
chr28	9521380	2	300	G:0.543333	A:0.456667			
chr28	9521388	2	302	C:0.486755	T:0.513245			
chr28	9522025	2	302	C:0.801325	T:0.198675			
chr28	9522046	3	298	G:0.802013	GAA:0.104027	GAAA:0.0939597		
chr28	9522056	2	286	AG:0.493007	A:0.506993			
chr28	9522057	2	292	G:0.797945	A:0.202055			
chr28	9522239	2	304	A:0.799342	T:0.200658			

chr28 9522566 2 314 G:0.77707 T:0.22293
chr28 9522631 2 310 C:0.787097 T:0.212903
chr28 9522639 2 308 A:0.876623 G:0.123377
chr28 9522821 2 300 C:0.323333 CCTCTGT:0.676667
chr28 9523058 2 300 T:0.613333 G:0.386667
chr28 9523213 2 292 C:0.643836 T:0.356164
chr28 9524088 2 294 G:0.687075 A:0.312925
chr28 9524160 2 292 T:0.59589 TC:0.40411
chr28 9524162 2 292 T:0.59589 C:0.40411
chr28 9524196 2 278 T:0.579137 A:0.420863
chr28 9525081 2 300 G:0.26 A:0.74
chr28 9525142 2 308 G:0.62987 A:0.37013
chr28 9525692 2 306 G:0.647059 C:0.352941
chr28 9525696 2 310 C:0.274194 T:0.725806
chr28 9526115 2 304 T:0.595395 TA:0.404605
chr28 9526276 2 304 G:0.957237 A:0.0427632
chr28 9526780 2 308 G:0.607143 C:0.392857
chr28 9527150 2 310 G:0.26129 T:0.73871
chr28 9527488 2 200 A:0.23 C:0.77
chr28 9527492 2 200 C:0.23 A:0.77
chr28 9527499 2 250 A:0.112 AC:0.888
chr28 9527529 4 316 GAAGAAAGA:0.731013 G:
0.123418 GAAGA:0.0981013 GAAGAAAGAAAGA:0.0474684
chr28 9527537 2 316 A:0.968354 AAAG:0.0316456
chr28 9527764 2 292 A:0.59589 G:0.40411
chr28 9528033 2 282 G:0.631206 T:0.368794
chr28 9528160 2 306 G:0.650327 C:0.349673
chr28 9528182 3 302 T:0.65894 TA:0.175497
TAA:0.165563
chr28 9528207 2 308 A:0.704545 AAC:0.295455
chr28 9528208 2 308 A:0.587662 AC:0.412338
chr28 9528209 2 312 A:0.637821 AAAC:0.362179
chr28 9528216 2 312 A:0.964744 AC:0.0352564
chr28 9528253 2 300 T:0.633333 C:0.366667
chr28 9528902 2 298 A:0.271812 G:0.728188
chr28 9529067 2 298 C:0.248322 T:0.751678
chr28 9529360 2 308 C:0.633117
CACAGAGAAAGAGAGAGAGAGAGAGAGAGAGGCAGAGAGACA:0.366883
chr28 9529491 2 206 G:0.194175 GT:0.805825
chr28 9529511 2 208 CTTATATTTATTTT:0.567308 C:
0.432692
chr28 9529577 2 118 A:0.822034 AAG:0.177966
chr28 9529688 2 306 G:0.591503 T:0.408497
chr28 9529766 2 296 A:0.699324 ACC:0.300676
chr28 9529773 2 298 T:0.258389 C:0.741611
chr28 9530088 2 306 A:0.663399 G:0.336601
chr28 9531740 2 308 T:0.642857 C:0.357143
chr28 9532276 2 312 A:0.660256 G:0.339744
chr28 9533095 2 302 A:0.274834 G:0.725166
chr28 9533300 2 300 T:0.68 G:0.32
chr28 9533491 2 306 A:0.676471 T:0.323529
chr28 9533685 2 304 G:0.634868 T:0.365132
chr28 9533715 2 310 T:0.951613 C:0.0483871
chr28 9533792 2 308 G:0.256494 GT:0.743506

chr28 9533874 2 308 CT:0.662338 C:0.337662
chr28 9534245 2 306 T:0.653595 TA:0.346405
chr28 9534425 2 314 T:0.675159 C:0.324841
chr28 9534450 3 318 CTTTTATTTTA:0.600629 C:
0.358491 CTTTTA:0.0408805
chr28 9534493 2 296 TTTTA:0.621622 T:0.378378
chr28 9534662 2 290 C:0.355172 T:0.644828
chr28 9534670 2 274 TTC:0.894161 T:0.105839
chr28 9534691 2 274 T:0.339416 TTTTA:0.660584
chr28 9534741 2 288 G:0.649306 C:0.350694
chr28 9534747 2 292 G:0.633562 A:0.366438
chr28 9535206 2 290 C:0.675862 T:0.324138
chr28 9535650 2 306 A:0.300654 G:0.699346
chr28 9535696 2 308 C:0.253247 T:0.746753
chr28 9535967 2 300 T:0.63 C:0.37
chr28 9536027 2 308 TC:0.967532 T:0.0324675
chr28 9536028 3 300 C:0.333333 CTT:0.336667
CTTT:0.33
chr28 9536056 2 292 T:0.667808 G:0.332192
chr28 9536195 2 280 C:0.592857 T:0.407143
chr28 9536362 2 300 T:0.3 C:0.7
chr28 9536624 3 286 TA:0.744755 T:0.0979021
TAA:0.157343
chr28 9537154 2 304 A:0.611842 AT:0.388158
chr28 9537172 2 310 AAC:0.954839 A:0.0451613
chr28 9537354 2 300 T:0.99 A:0.01
chr28 9537511 2 302 TA:0.688742 T:0.311258
chr28 9537555 2 306 A:0.627451 T:0.372549
chr28 9537556 2 306 T:0.627451 G:0.372549
chr28 9537565 2 310 A:0.7 C:0.3
chr28 9537697 2 312 G:0.955128 C:0.0448718
chr28 9537699 2 312 G:0.653846 A:0.346154
chr28 9537780 2 306 C:0.964052 T:0.0359477
chr28 9538134 2 316 T:0.632911 TAAAAAAAC:
0.367089
chr28 9538161 2 316 TCA:0.632911 T:0.367089
chr28 9538230 2 286 C:0.608392 G:0.391608
chr28 9538320 2 262 A:0.599237 AG:0.400763
chr28 9538380 2 248 G:0.673387 C:0.326613
chr28 9538530 2 260 A:0.623077 G:0.376923
chr28 9538894 2 246 CG:0.5 C:0.5
chr28 9539003 2 288 C:0.652778 G:0.347222
chr28 9539029 2 296 G:0.655405 T:0.344595
chr28 9539131 2 290 G:0.631034 C:0.368966
chr28 9539496 2 282 A:0.620567 G:0.379433
chr28 9539650 2 310 C:0.951613 CTCTA:0.0483871
chr28 9539693 2 292 A:0.657534 T:0.342466
chr28 9539856 2 298 A:0.667785 G:0.332215
chr28 9539946 2 310 C:0.680645 T:0.319355
chr28 9540151 2 294 G:0.636054 A:0.363946
chr28 9540383 2 278 T:0.661871 TA:0.338129
chr28 9540981 2 310 C:0.970968 T:0.0290323
chr28 9542285 2 306 G:0.683007 C:0.316993
chr28 9542524 2 312 C:0.990385 G:0.00961538

chr28	9542826	2	288	G:0.684028	C:0.315972	
chr28	9543002	2	292	G:0.5	T:0.5	
chr28	9543081	2	282	G:0.585106	C:0.414894	
chr28	9543089	2	292	T:0.976027	TGTG:0.0239726	
chr28	9543110	2	268	GGT:0.955224	G:0.0447761	
chr28	9543112	2	298	T:0.734899	TGGTG:0.265101	
chr28	9543441	2	310	CCCTCTA:0.822581	C:0.177419	
chr28	9543447	2	308	A:0.672078	C:0.327922	
chr28	9543483	2	300	TCTCA:0.686667	T:0.313333	
chr28	9543487	2	300	A:0.846667	T:0.153333	
chr28	9543735	2	298	C:0.526846	T:0.473154	
chr28	9543860	3	316	AACACACACAC:0.537975	A:0.224684	AACACACACACAC:0.237342
chr28	9544056	2	306	A:0.486928	AT:0.513072	
chr28	9544375	2	306	G:0.679739	C:0.320261	
chr28	9544725	2	294	C:0.693878	CT:0.306122	
chr28	9544848	2	308	G:0.957792	GTCA:0.0422078	
chr28	9545657	2	284	G:0.623239	GC:0.376761	
chr28	9545740	2	306	G:0.663399	A:0.336601	
chr28	9546080	2	306	C:0.444444	CTAAA:0.555556	
chr28	9546249	2	306	T:0.614379	A:0.385621	
chr28	9546401	2	302	G:0.423841	T:0.576159	
chr28	9547729	2	300	G:0.986667	A:0.0133333	
chr28	9547754	2	308	G:0.5	GAAACTT:0.5	
chr28	9547967	2	306	T:0.656863	C:0.343137	
chr28	9548836	2	290	G:0.993103	T:0.00689655	
chr28	9548879	2	288	C:0.940972	CCT:0.0590278	
chr28	9548927	2	298	T:0.486577	TA:0.513423	
chr28	9549186	2	310	TAA:0.641935	T:0.358065	
chr28	9549260	2	304	C:0.963816	T:0.0361842	
chr28	9549466	2	306	T:0.503268	G:0.496732	
chr28	9549533	2	306	A:0.699346	G:0.300654	
chr28	9549617	2	308	T:0.659091	G:0.340909	
chr28	9550879	3	292	GAA:0.380137	G:0.383562	GA:0.236301
chr28	9550955	2	306	T:0.5	G:0.5	
chr28	9551321	2	296	AT:0.831081	A:0.168919	
chr28	9551324	2	296	T:0.236486	A:0.763514	
chr28	9551336	2	300	C:0.826667	T:0.173333	
chr28	9551751	2	300	A:0.83	G:0.17	
chr28	9552058	2	312	G:0.99359	A:0.00641026	
chr28	9552210	2	300	A:0.976667	G:0.0233333	
chr28	9552368	2	310	A:0.841935	G:0.158065	
chr28	9552896	2	302	GA:0.847682	G:0.152318	
chr28	9552963	2	296	T:0.5	C:0.5	
chr28	9553043	2	304	C:0.710526	CCTCT:0.289474	
chr28	9553084	2	302	T:0.834437	TA:0.165563	
chr28	9553085	2	308	A:0.993506	T:0.00649351	
chr28	9553087	2	306	A:0.98366	AT:0.0163399	
chr28	9553185	2	310	ATTCTT:0.845161	A:0.154839	
chr28	9553297	2	294	CAT:0.993197	C:0.00680272	
chr28	9553608	2	308	G:0.49026	A:0.50974	
chr28	9553743	2	304	TA:0.832237	T:0.167763	
chr28	9554716	2	300	T:0.893333	G:0.106667	

chr28	9555719	2	306	A:0.823529	C:0.176471
chr28	9556220	3	306	TA:0.823529	T:0.0620915
TAA:0.114379
chr28	9556926	2	306	C:0.872549	G:0.127451
chr28	9557108	2	296	A:0.983108	T:0.0168919
chr28	9557190	2	300	T:0.436667	C:0.563333
chr28	9557298	2	300	A:0.836667	T:0.163333
chr28	9557305	2	298	T:0.825503	A:0.174497
chr28	9557451	2	302	C:0.983444	T:0.0165563
chr28	9558305	2	296	AAGAT:0.959459	A:0.0405405
chr28	9558311	3	308	G:0.243506	T:0.425325
GATTTATTTATTTATTT:0.331169
chr28	9558838	2	280	T:0.592857	C:0.407143
chr28	9559014	2	284	G:0.570423	GA:0.429577
chr28	9559483	2	288	G:0.555556	A:0.444444
chr28	9559536	6	302
ATTTCTTTTCTTTTCTTTTCTTTTCTTTTCTTTTCT:0.321192	A:0.248344
ATTTCT:0.0761589 ATTTCTTTTCTTTTCT:0.0695364
ATTTCTTTTCTTTTCTTTTCT:0.198675	ATTTCTTTTCTTTTCTTTTCTTTTCT:
0.0860927
chr28	9559552	2	290	TTTCTTTTCTTTTCTTTTCTTTTCTTTTC:
0.97931	T:0.0206897
chr28	9560695	2	288	A:0.618056	G:0.381944
chr28	9560734	2	278	C:0.607914	T:0.392086
chr28	9561005	2	270	G:0.544444	GAA:0.455556
chr28	9561271	2	300	T:0.623333	C:0.376667
chr28	9561290	2	296	G:0.658784	A:0.341216
chr28	9561550	2	306	G:0.673203	A:0.326797
chr28	9561952	2	298	C:0.459732	CCT:0.540268
chr28	9561991	2	308	T:0.805195	A:0.194805
chr28	9561992	2	308	T:0.805195	A:0.194805
chr28	9562048	2	320	T:0.828125
TAAGCGTCTGACTCTTAAAAATAAAAAAAAAAATAAATAAATAAAAAAAAAA:0.171875
chr28	9562139	3	302	TAAAAA:0.427152	T:0.228477
TAAAA:0.344371
chr28	9562141	2	280	A:0.685714	T:0.314286
chr28	9562226	2	304	C:0.865132	T:0.134868
chr28	9562307	2	302	A:0.662252	G:0.337748
chr28	9562854	2	114	GTC:0.815789	G:0.184211
chr28	9562963	2	256	C:0.496094	T:0.503906
chr28	9563089	2	300	ACTTC:0.96	A:0.04
chr28	9563094	2	300	T:0.96	G:0.04
chr28	9563687	2	304	T:0.453947	C:0.546053
chr28	9563689	2	304	T:0.828947	C:0.171053
chr28	9563728	2	310	G:0.990323	A:0.00967742
chr28	9563806	2	306	C:0.437908	T:0.562092
chr28	9563872	2	296	A:0.449324	G:0.550676
chr28	9563966	2	304	T:0.417763	C:0.582237
chr28	9564087	2	284	T:0.401408	A:0.598592
chr28	9564157	4	314	CATATATATATATATATAT:0.187898
C:0.343949	CAT:0.404459	CATATATATATATATAT:0.0636943
chr28	9564232	2	286	A:0.370629	G:0.629371
chr28	9564455	2	286	C:0.41958	A:0.58042
chr28	9564517	2	308	C:0.461039	T:0.538961

chr28	9564662	2	298	ATACC:0.459732	A:0.540268	
chr28	9564839	2	302	A:0.622517	ATCTC:0.377483	
chr28	9564860	2	300	T:0.953333	TC:0.0466667	
chr28	9564863	2	300	T:0.953333	TTC:0.0466667	
chr28	9564880	2	294	C:0.959184	T:0.0408163	
chr28	9564988	2	292	C:0.421233	T:0.578767	
chr28	9565137	2	308	G:0.448052	A:0.551948	
chr28	9565255	2	306	T:0.986928	G:0.0130719	
chr28	9565492	2	304	T:0.434211	A:0.565789	
chr28	9565976	2	300	CT:0.933333	C:0.0666667	
chr28	9566010	2	308	C:0.467532	T:0.532468	
chr28	9566172	2	312	CTTCCTAT:0.49359	C:0.50641	
chr28	9566326	2	308	T:0.948052	C:0.0519481	
chr28	9566408	2	308	T:0.448052	C:0.551948	
chr28	9566466	2	308	T:0.983766	C:0.0162338	
chr28	9566635	2	298	CT:0.419463	C:0.580537	
chr28	9566638	2	304	T:0.513158	C:0.486842	
chr28	9566641	3	310	T:0.945161	TTTTC:0.0419355	TTTTTC:0.0129032
chr28	9566845	2	296	A:0.476351	T:0.523649	
chr28	9566980	3	304	CAA:0.460526	C:0.473684	CA:0.0657895
chr28	9567018	2	292	C:0.452055	T:0.547945	
chr28	9567089	2	296	A:0.445946	G:0.554054	
chr28	9567242	2	292	C:0.479452	T:0.520548	
chr28	9567334	2	276	T:0.463768	G:0.536232	
chr28	9567374	2	298	T:0.493289	TAAAAAA:0.506711	
chr28	9567491	2	300	A:0.456667	T:0.543333	
chr28	9567567	2	306	A:0.447712	G:0.552288	
chr28	9567838	2	306	T:0.924837	C:0.0751634	
chr28	9568410	2	312	C:0.935897	T:0.0641026	
chr28	9568453	2	304	A:0.480263	C:0.519737	
chr28	9568758	2	296	T:0.945946	G:0.0540541	
chr28	9568823	2	300	T:0.453333	C:0.546667	
chr28	9568867	2	300	T:0.953333	A:0.0466667	
chr28	9568868	2	304	A:0.944079	T:0.0559211	
chr28	9568876	2	304	A:0.944079	T:0.0559211	
chr28	9568938	2	302	TAC:0.923841	T:0.0761589	
chr28	9569039	2	292	C:0.945205	CAT:0.0547945	
chr28	9569139	2	306	A:0.973856	G:0.0261438	
chr28	9569192	2	306	A:0.964052	AG:0.0359477	
chr28	9569449	2	302	T:0.917219	C:0.0827815	
chr28	9569456	2	302	T:0.917219	C:0.0827815	
chr28	9569549	2	304	A:0.930921	T:0.0690789	
chr28	9569567	2	308	A:0.477273	G:0.522727	
chr28	9569730	2	306	A:0.918301	G:0.0816993	
chr28	9569792	2	298	C:0.95302	T:0.0469799	
chr28	9569865	2	306	T:0.915033	TA:0.0849673	
chr28	9569873	3	316	A:0.490506	AAAG:0.0696203	AAAAAAG:0.439873
chr28	9569932	2	310	T:0.945161	G:0.0548387	
chr28	9569936	2	310	T:0.945161	C:0.0548387	
chr28	9570043	2	310	T:0.948387	A:0.0516129	
chr28	9570739	2	288	T:0.482639	TA:0.517361	

chr28	9570895	2	302	C:0.933775	T:0.0662252
chr28	9571027	2	302	T:0.956954	A:0.0430464
chr28	9571090	2	290	A:0.937931	T:0.062069
chr28	9571210	2	308	C:0.951299	T:0.0487013
chr28	9571231	2	290	TA:0.47931	T:0.52069
chr28	9571414	2	298	T:0.432886	G:0.567114
chr28	9571507	2	306	A:0.46732	G:0.53268
chr28	9571879	2	304	A:0.480263	T:0.519737
chr28	9572284	2	284	TCAG:0.693662	T:0.306338
chr28	9572372	4	236	AG:0.516949	A:0.127119
AGG:0.199153	AGGG:0.15678
chr28	9574033	2	296	G:0.956081	T:0.0439189
chr28	9574255	2	300	G:0.943333	T:0.0566667
chr28	9574382	2	308	G:0.944805	C:0.0551948
chr28	9574497	2	296	G:0.753378	GT:0.246622
chr28	9574508	2	308	T:0.944805	TTTA:0.0551948
chr28	9574595	2	308	C:0.951299	T:0.0487013
chr28	9574685	2	314	C:0.929936	T:0.0700637
chr28	9574911	2	308	G:0.954545	T:0.0454545
chr28	9575111	2	298	T:0.701342	TG:0.298658
chr28	9575539	2	318	T:0.95283	TC:0.0471698
chr28	9575563	2	318	G:0.95283	T:0.0471698
chr28	9575754	2	302	A:0.923841	G:0.0761589
chr28	9575783	2	306	A:0.960784	T:0.0392157
chr28	9576019	2	308	A:0.954545	T:0.0454545
chr28	9576029	2	308	A:0.954545	T:0.0454545
chr28	9576357	2	302	T:0.956954	G:0.0430464
chr28	9576358	2	302	T:0.956954	C:0.0430464
chr28	9576589	2	304	G:0.950658	A:0.0493421
chr28	9576677	2	310	C:0.267742	G:0.732258
chr28	9577179	2	308	ATAAAG:0.935065	A:0.0649351
chr28	9577329	2	184	TG:0.907609	T:0.0923913
chr28	9577593	2	304	A:0.858553	G:0.141447
chr28	9577819	2	304	A:0.980263	G:0.0197368
chr28	9577997	2	308	C:0.941558	T:0.0584416
chr28	9578021	2	318	C:0.943396	CGGG:0.0566038
chr28	9578022	2	318	A:0.943396	ATCCCTGGGTGGCGC:
0.0566038
chr28	9578024	2	318	G:0.943396
GCGGTTTGGCGCCTGCCT:0.0566038
chr28	9578050	2	306	G:0.794118	A:0.205882
chr28	9578247	2	300	A:0.966667	G:0.0333333
chr28	9578336	2	320	A:0.95
ATTATCTTTATTATTTTCTTTTTTTTTTTTT:0.05
chr28	9578932	2	300	T:0.963333	G:0.0366667
chr28	9579011	2	318	A:0.955975
ATATAGAATATATGTCAT:0.0440252
chr28	9579015	2	318	A:0.955975	ATAT:0.0440252
chr28	9579016	2	318	C:0.955975	G:0.0440252
chr28	9579207	2	310	G:0.948387	A:0.0516129
chr28	9579410	2	308	C:0.987013	T:0.012987
chr28	9579440	2	310	T:0.96129	C:0.0387097
chr28	9579871	2	302	G:0.970199	T:0.0298013
chr28	9580107	2	314	A:0.33121	G:0.66879

chr28	9580128	2	316	C:0.265823	T:0.734177	
chr28	9580216	2	300	G:0.216667	A:0.783333	
chr28	9580396	2	304	T:0.960526	G:0.0394737	
chr28	9580457	2	310	G:0.951613	A:0.0483871	
chr28	9580922	2	308	A:0.948052	AT:0.0519481	
chr28	9580931	2	304	C:0.950658	CCA:0.0493421	
chr28	9581200	2	280	TA:0.807143	T:0.192857	
chr28	9581214	2	274	AT:0.79562	A:0.20438	
chr28	9581215	2	278	T:0.546763	A:0.453237	
chr28	9581374	2	290	G:0.941379	A:0.0586207	
chr28	9581495	2	286	G:0.986014	A:0.013986	
chr28	9581533	2	278	G:0.960432	A:0.0395683	
chr28	9581568	2	294	G:0.97619	A:0.0238095	
chr28	9581640	2	310	T:0.945161	C:0.0548387	
chr28	9581651	2	312	C:0.689103	T:0.310897	
chr28	9581821	2	296	G:0.966216	A:0.0337838	
chr28	9581918	2	306	A:0.944444	G:0.0555556	
chr28	9582135	2	296	T:0.956081	C:0.0439189	
chr28	9582608	2	308	A:0.944805	G:0.0551948	
chr28	9582966	2	316	A:0.958861	AT:0.0411392	
chr28	9582975	2	316	G:0.958861	A:0.0411392	
chr28	9582976	2	308	C:0.337662	CT:0.662338	
chr28	9582986	2	316	T:0.958861	TA:0.0411392	
chr28	9583014	2	312	TGAGA:0.935897	T:0.0641026	
chr28	9583915	2	304	A:0.960526	AGT:0.0394737	
chr28	9584159	2	304	G:0.967105	GC:0.0328947	
chr28	9584161	2	304	G:0.967105	A:0.0328947	
chr28	9584202	2	304	A:0.710526	G:0.289474	
chr28	9584260	2	270	G:0.966667	GTC:0.0333333	
chr28	9584262	2	292	C:0.962329	CTA:0.0376712	
chr28	9585083	2	314	G:0.958599	A:0.0414013	
chr28	9585095	2	314	A:0.958599	G:0.0414013	
chr28	9585230	2	306	C:0.69281	T:0.30719	
chr28	9585360	2	292	G:0.736301	GT:0.263699	
chr28	9586291	2	306	A:0.954248	G:0.0457516	
chr28	9586670	2	302	G:0.986755	A:0.013245	
chr28	9587213	2	298	C:0.956376	CT:0.0436242	
chr28	9587279	2	304	T:0.963816	C:0.0361842	
chr28	9587607	2	310	C:0.945161	G:0.0548387	
chr28	9587634	2	316	T:0.949367	C:0.0506329	
chr28	9587706	2	300	G:0.94	GT:0.06	
chr28	9588002	2	298	C:0.95302	T:0.0469799	
chr28	9588003	2	298	A:0.95302	G:0.0469799	
chr28	9589333	2	298	G:0.95302	A:0.0469799	
chr28	9590225	2	310	G:0.270968	A:0.729032	
chr28	9590592	2	310	G:0.958065	T:0.0419355	
chr28	9590966	2	300	A:0.206667	G:0.793333	
chr28	9591226	3	298	GTA:0.644295	G:0.191275	GTATATA:0.16443
chr28	9591411	2	288	A:0.208333	T:0.791667	
chr28	9591551	2	312	C:0.964744	T:0.0352564	
chr28	9591697	2	316	A:0.952532	C:0.0474684	
chr28	9591720	2	314	C:0.961783	T:0.0382166	
chr28	9591912	2	302	A:0.953642	AG:0.0463576	

chr28	9591913	3	302	AT:0.218543	A:0.735099	TT:0.0463576
chr28	9592007	2	312	G:0.964744	A:0.0352564	
chr28	9592121	2	310	C:0.951613	T:0.0483871	
chr28	9592682	2	306	G:0.862745	GT:0.137255	
chr28	9593230	2	304	T:0.217105	C:0.782895	
chr28	9593310	2	294	C:0.955782	T:0.0442177	
chr28	9593919	2	306	C:0.944444	G:0.0555556	
chr28	9593973	2	312	T:0.99359	C:0.00641026	
chr28	9594038	2	302	G:0.953642	A:0.0463576	
chr28	9595125	2	304	T:0.957237	C:0.0427632	
chr28	9595450	2	312	G:0.907051	T:0.0929487	
chr28	9595656	2	306	T:0.947712	C:0.0522876	
chr28	9596638	2	306	T:0.728758	C:0.271242	
chr28	9596694	2	302	G:0.94702	A:0.0529801	
chr28	9597063	2	310	T:0.990323	C:0.00967742	
chr28	9599398	2	292	A:0.921233	AT:0.0787671	
chr28	9599515	2	308	C:0.733766	T:0.266234	
chr28	9599719	2	308	C:0.954545	T:0.0454545	
chr28	9599929	2	296	G:0.966216	A:0.0337838	
chr28	9599962	3	258	CT:0.573643	C:0.162791	CTT:0.263566
chr28	9600142	2	304	G:0.792763	GAA:0.207237	
chr28	9600972	2	314	G:0.27707	A:0.72293	
chr28	9601336	2	296	T:0.969595	C:0.0304054	
chr28	9601346	2	276	G:0.528986	GTTT:0.471014	
chr28	9601688	2	308	A:0.909091	T:0.0909091	
chr28	9601819	2	318	C:0.962264	CT:0.0377358	
chr28	9602071	2	306	G:0.960784	T:0.0392157	
chr28	9602515	2	302	C:0.698675	CAGAG:0.301325	
chr28	9602556	2	296	T:0.692568	C:0.307432	
chr28	9602709	2	298	G:0.959732	T:0.0402685	
chr28	9602781	2	314	G:0.780255	A:0.219745	
chr28	9603071	2	310	G:0.958065	A:0.0419355	
chr28	9603595	2	308	C:0.967532	T:0.0324675	
chr28	9603705	2	312	CAAATGT:0.939103	C:0.0608974	
chr28	9603737	2	308	G:0.688312	A:0.311688	
chr28	9603866	2	294	A:0.942177	G:0.0578231	
chr28	9604113	2	310	C:0.958065	CAAT:0.0419355	
chr28	9604502	2	312	C:0.955128	T:0.0448718	
chr28	9604526	2	308	C:0.253247	G:0.746753	
chr28	9604595	2	298	A:0.95302	AT:0.0469799	
chr28	9604900	2	308	C:0.353896	T:0.646104	
chr28	9604964	2	304	A:0.710526	AATT:0.289474	
chr28	9605189	2	292	T:0.952055	C:0.0479452	
chr28	9605274	2	292	C:0.945205	T:0.0547945	
chr28	9605900	2	316	A:0.977848	G:0.0221519	
chr28	9606015	2	312	A:0.99359	G:0.00641026	
chr28	9606378	2	306	A:0.95098	C:0.0490196	
chr28	9607071	2	304	A:0.375	G:0.625	
chr28	9607195	2	304	G:0.953947	A:0.0460526	
chr28	9607695	2	316	T:0.446203	TG:0.553797	
chr28	9608901	2	312	C:0.772436	T:0.227564	
chr28	9609333	2	306	C:0.313725	T:0.686275	

chr28	9609384	2	312	A:0.987179	G:0.0128205	
chr28	9609453	2	312	AG:0.971154	A:0.0288462	
chr28	9609874	2	304	T:0.950658	C:0.0493421	
chr28	9609894	2	300	G:0.96	A:0.04	
chr28	9609925	2	304	C:0.694079	CAAAT:0.305921	
chr28	9610424	2	294	C:0.952381	G:0.047619	
chr28	9610717	2	304	T:0.279605	A:0.720395	
chr28	9610718	3	304	T:0.236842	A:0.720395	TA:0.0427632
chr28	9610948	2	306	A:0.70915	T:0.29085	
chr28	9611116	2	242	C:0.690083	CTT:0.309917	
chr28	9612034	2	314	G:0.958599	A:0.0414013	
chr28	9612691	2	310	G:0.951613	A:0.0483871	
chr28	9612988	2	296	G:0.956081	A:0.0439189	
chr28	9613566	2	292	C:0.294521	CT:0.705479	
chr28	9614922	2	314	ACTTT:0.729299	A:0.270701	
chr28	9615103	2	310	C:0.941935	G:0.0580645	
chr28	9615195	3	308	G:0.279221	GA:0.0876623	GAA:0.633117
chr28	9615206	2	310	C:0.948387	A:0.0516129	
chr28	9615427	2	308	T:0.961039	C:0.038961	
chr28	9615564	2	304	T:0.322368	G:0.677632	
chr28	9616775	2	310	A:0.258065	C:0.741935	
chr28	9616846	2	300	A:0.853333	C:0.146667	
chr28	9616922	2	292	TA:0.184932	T:0.815068	
chr28	9616934	2	292	A:0.184932	C:0.815068	
chr28	9617161	2	300	T:0.906667	C:0.0933333	
chr28	9617887	2	302	C:0.980132	T:0.0198675	
chr28	9618073	2	300	A:0.903333	T:0.0966667	
chr28	9619536	2	304	A:0.944079	G:0.0559211	
chr28	9620179	2	308	AG:0.659091	A:0.340909	
chr28	9620202	2	312	G:0.855769	A:0.144231	
chr28	9620729	2	312	A:0.227564	T:0.772436	
chr28	9620950	2	304	T:0.671053	C:0.328947	
chr28	9621608	2	310	C:0.993548	T:0.00645161	
chr28	9622738	2	302	ATT:0.880795	A:0.119205	
chr28	9623784	2	314	A:0.219745	G:0.780255	
chr28	9623870	2	314	A:0.898089	G:0.101911	
chr28	9624327	2	310	C:0.232258	T:0.767742	
chr28	9624715	2	312	A:0.320513	G:0.679487	
chr28	9625347	2	304	T:0.680921	C:0.319079	
chr28	9625621	2	300	A:0.926667	G:0.0733333	
chr28	9626145	2	312	TG:0.951923	T:0.0480769	
chr28	9626211	2	306	T:0.993464	C:0.00653595	
chr28	9627016	2	312	A:0.942308	C:0.0576923	
chr28	9627355	2	306	C:0.686275	A:0.313725	
chr28	9628345	2	296	C:0.300676	T:0.699324	
chr28	9629034	2	306	A:0.676471	T:0.323529	
chr28	9630247	2	300	A:0.826667	AAG:0.173333	
chr28	9630576	2	304	C:0.700658	CAT:0.299342	
chr28	9630904	2	306	A:0.22549	G:0.77451	
chr28	9630930	2	316	C:0.96519	T:0.0348101	
chr28	9630936	2	312	A:0.314103	T:0.685897	
chr28	9631549	2	298	A:0.681208	T:0.318792	

chr28 9632064 2 306 CT:0.957516 C:0.0424837
chr28 9632137 4 312 G:0.483974 GCATT:0.214744
GCATTCATT:0.192308 GCATTCATTCATT:0.108974
chr28 9632654 2 302 G:0.788079 A:0.211921
chr28 9632733 2 306 A:0.696078 ATCTG:0.303922
chr28 9632756 2 314 TGGCTACAG:0.958599 T:
0.0414013
chr28 9632783 2 314 G:0.828025 A:0.171975
chr28 9632961 2 304 T:0.986842 TTTTG:0.0131579
chr28 9633726 2 292 C:0.695205 T:0.304795
chr28 9633821 2 256 GATTT:0.578125 G:0.421875
chr28 9634589 2 300 C:0.89 CT:0.11
chr28 9634607 2 314 T:0.305732 G:0.694268
chr28 9634619 2 316 TTTTG:0.31962 T:0.68038
chr28 9634690 2 296 G:0.922297 A:0.0777027
chr28 9634767 2 290 G:0.668966 A:0.331034
chr28 9635248 2 310 G:0.958065 T:0.0419355
chr28 9635437 2 300 G:0.976667 A:0.0233333
chr28 9635479 2 300 G:0.956667 T:0.0433333
chr28 9635722 2 306 C:0.326797 T:0.673203
chr28 9637485 2 302 G:0.672185 A:0.327815
chr28 9637689 2 306 G:0.70915 A:0.29085
chr28 9638048 2 310 A:0 AT:1
chr28 9638178 3 304 TAA:0.263158 T:0.0789474
TA:0.657895
chr28 9638628 2 302 C:0.791391 T:0.208609
chr28 9638765 2 292 G:0.280822 A:0.719178
chr28 9638891 2 312 AT:0.272436 A:0.727564
chr28 9638897 2 312 T:0.272436 A:0.727564
chr28 9638903 2 304 TTTTTA:0.911184 T:0.0888158
chr28 9638904 2 304 TTTTA:0.365132 T:0.634868
chr28 9639007 2 308 T:0.269481 C:0.730519
chr28 9639582 2 298 G:0.973154 A:0.0268456
chr28 9640520 2 298 T:0.963087 C:0.0369128
chr28 9640812 2 300 C:0.713333 T:0.286667
chr28 9640917 2 300 A:0.983333 T:0.0166667
chr28 9641210 2 282 C:0.943262 T:0.0567376
chr28 9641320 3 258 TAAAAAAA:0.321705 T:
0.294574 TA:0.383721
chr28 9641432 2 268 GAT:0.858209 G:0.141791
chr28 9641446 4 316 T:0.455696
TATATATATATGATGTCATATATATATGATGTCATC:0.227848
TATATATATATATGATGTCATATATATATGATGTCATC:0.193038
TATATATATATATATGATGTCATATATATATGATGTCATC:0.123418
chr28 9641936 2 306 A:0.95098 G:0.0490196
chr28 9642104 2 306 C:0.267974 G:0.732026
chr28 9642174 2 300 G:0.306667 T:0.693333
chr28 9643130 2 294 GA:0.921769 G:0.0782313
chr28 9646093 2 310 A:0.732258 G:0.267742
chr28 9646782 2 292 C:0.246575 CT:0.753425
chr28 9647574 2 298 T:0.271812 TA:0.728188
chr28 9647680 3 314 A:0.77707 AGTGTGTGT:0.11465
AGTGTGTGTGT:0.10828
chr28 9647845 2 304 T:0.680921 C:0.319079

chr28	9648320	2	308	G:0.987013	A:0.012987	
chr28	9648412	2	304	C:0.3125	T:0.6875	
chr28	9648490	2	310	C:0.96129	T:0.0387097	
chr28	9648986	2	302	A:0.960265	G:0.0397351	
chr28	9649214	2	312	A:0.24359	G:0.75641	
chr28	9649446	2	306	A:0.777778	C:0.222222	
chr28	9649505	2	312	A:0.766026	G:0.233974	
chr28	9650323	3	266	G:0.236842	GTT:0.353383	GTTT:0.409774
chr28	9650634	2	304	C:0.555921	A:0.444079	
chr28	9650639	2	306	T:0.993464	C:0.00653595	
chr28	9651038	2	302	T:0.443709	C:0.556291	
chr28	9651083	2	304	CTT:0.427632	C:0.572368	
chr28	9651369	2	304	A:0.953947	G:0.0460526	
chr28	9651668	2	312	T:0.945513	C:0.0544872	
chr28	9651680	2	316	G:0.455696	A:0.544304	
chr28	9652019	2	312	TGTCA:0.432692	T:0.567308	
chr28	9652056	2	306	G:0.954248	C:0.0457516	
chr28	9652957	2	302	T:0.44702	C:0.55298	
chr28	9653028	2	306	T:0.444444	C:0.555556	
chr28	9653703	2	312	T:0.948718	C:0.0512821	
chr28	9653750	2	302	A:0.94702	G:0.0529801	
chr28	9653818	2	304	T:0.460526	TTAA:0.539474	
chr28	9654143	2	244	A:0.47541	G:0.52459	
chr28	9654174	2	212	C:0.533019	T:0.466981	
chr28	9654445	2	222	G:0.63964	GC:0.36036	
chr28	9654971	2	302	A:0.602649	G:0.397351	
chr28	9655006	2	308	C:0.464286	T:0.535714	
chr28	9655026	2	306	T:0.385621	C:0.614379	
chr28	9655278	2	316	T:0.96519	TGAGTTACCCCTCACTG:0.0348101	
chr28	9655280	2	316	A:0.623418	AGTTACCCCTCACTGGG:0.376582	
chr28	9655689	2	292	C:0.931507	T:0.0684932	
chr28	9655751	2	286	C:0.986014	T:0.013986	
chr28	9655846	2	304	G:0.940789	A:0.0592105	
chr28	9656111	2	296	A:0.993243	G:0.00675676	
chr28	9656180	2	296	T:0.432432	G:0.567568	
chr28	9656183	2	296	C:0.506757	CA:0.493243	
chr28	9656400	2	304	G:0.509868	A:0.490132	
chr28	9656541	2	306	A:0.405229	C:0.594771	
chr28	9656548	2	306	G:0.493464	A:0.506536	
chr28	9656876	2	260	A:0.469231	T:0.530769	
chr28	9657139	2	206	T:0.558252	C:0.441748	
chr28	9657309	2	284	GT:0.887324	G:0.112676	
chr28	9657397	2	282	G:0.950355	A:0.0496454	
chr28	9657624	2	278	T:0.147482	A:0.852518	
chr28	9657795	2	236	C:0.927966	A:0.0720339	
chr28	9657904	2	276	G:0.963768	A:0.0362319	
chr28	9657940	2	296	A:0.628378	AGCATGCAGT:0.371622	
chr28	9658171	2	302	TC:0.980132	T:0.0198675	
chr28	9658397	2	308	T:0.548701	C:0.451299	
chr28	9658782	2	308	C:0.957792	T:0.0422078	

chr28	9658807	2	310	A:0.445161	T:0.554839
chr28	9659055	2	288	A:0.559028	AGC:0.440972
chr28	9659105	2	316	T:0.566456	TGCTTCCGCTGTGTCAG:0.433544
chr28	9659176	2	310	G:0.393548	GA:0.606452
chr28	9659199	2	314	A:0.452229	G:0.547771
chr28	9659205	2	314	G:0.452229	A:0.547771
chr28	9659489	2	308	CAA:0.957792	C:0.0422078
chr28	9659589	2	300	C:0.566667	T:0.433333
chr28	9659647	2	296	T:0.418919	C:0.581081
chr28	9659741	2	302	G:0.910596	A:0.089404
chr28	9659752	2	302	T:0.956954	A:0.0430464
chr28	9659891	2	290	G:0.910345	GC:0.0896552
chr28	9660193	2	300	G:0.953333	T:0.0466667
chr28	9660414	2	306	C:0.95098	T:0.0490196
chr28	9660529	2	296	C:0.945946	T:0.0540541
chr28	9660628	2	314	T:0.936306	G:0.0636943
chr28	9660887	2	308	T:0.512987	C:0.487013
chr28	9661344	2	298	G:0.963087	T:0.0369128
chr28	9661812	2	282	C:0.570922	G:0.429078
chr28	9661982	2	306	TAA:0.509804	T:0.490196
chr28	9661983	2	306	A:0.977124	T:0.0228758
chr28	9662011	2	300	C:0.95	CAG:0.05
chr28	9662101	2	292	A:0.534247	T:0.465753
chr28	9662113	2	292	C:0.965753	T:0.0342466
chr28	9662146	2	294	T:0.557823	C:0.442177
chr28	9662157	2	294	G:0.602041	A:0.397959
chr28	9662260	2	310	C:0.951613	T:0.0483871
chr28	9662584	2	300	A:0.486667	T:0.513333
chr28	9662786	2	298	T:0.493289	C:0.506711
chr28	9662830	2	306	C:0.954248	T:0.0457516
chr28	9663399	2	300	C:0.953333	T:0.0466667
chr28	9663510	2	300	A:0.433333	G:0.566667
chr28	9663899	2	308	G:0.594156	A:0.405844
chr28	9664071	2	306	T:0.405229	C:0.594771
chr28	9664231	2	304	T:0.947368	A:0.0526316
chr28	9664387	2	310	G:0.9	A:0.1
chr28	9664447	2	306	G:0.578431	A:0.421569
chr28	9664638	2	308	A:0.396104	G:0.603896
chr28	9665033	2	298	T:0.479866	C:0.520134
chr28	9665243	2	302	G:0.950331	A:0.0496689
chr28	9665256	2	300	C:0.91	G:0.09
chr28	9665461	2	300	G:0.586667	A:0.413333
chr28	9665513	2	298	C:0.986577	T:0.0134228
chr28	9666121	2	290	A:0.417241	T:0.582759
chr28	9666214	2	310	G:0.945161	A:0.0548387
chr28	9666242	2	302	A:0.410596	G:0.589404
chr28	9666302	2	302	T:0.44702	G:0.55298
chr28	9666472	2	298	G:0.503356	C:0.496644
chr28	9666500	2	286	G:0.986014	A:0.013986
chr28	9666626	2	256	C:0.640625	CT:0.359375
chr28	9666723	2	310	C:0.954839	T:0.0451613
chr28	9666783	2	304	C:0.950658	G:0.0493421
chr28	9666948	2	298	G:0.533557	T:0.466443

chr28	9667079	2	308	T:0.948052	C:0.0519481	
chr28	9667086	2	308	C:0.964286	CCG:0.0357143	
chr28	9667231	2	306	C:0.535948	A:0.464052	
chr28	9667505	2	298	C:0.604027	T:0.395973	
chr28	9667701	2	296	C:0.956081	T:0.0439189	
chr28	9667996	2	300	A:0.6	G:0.4	
chr28	9668104	2	300	C:0.6	T:0.4	
chr28	9668263	2	314	A:0.496815	G:0.503185	
chr28	9668410	2	308	C:0	CA:1	
chr28	9668421	2	308	TG:0	T:1	
chr28	9668423	2	308	T:0	A:1	
chr28	9668439	2	304	C:0.973684	T:0.0263158	
chr28	9668995	2	308	G:0.584416	A:0.415584	
chr28	9669489	2	302	G:0.496689	A:0.503311	
chr28	9669533	2	302	T:0.94702	C:0.0529801	
chr28	9669608	2	304	T:0.953947	C:0.0460526	
chr28	9669803	2	296	T:0.52027	C:0.47973	
chr28	9669900	2	312	T:0.615385	TCC:0.384615	
chr28	9669906	3	312	A:0.592949	AAAAAC:0.0480769	AAAAC:0.358974
chr28	9670366	2	298	A:0	G:1	
chr28	9671318	2	302	G:0.460265	A:0.539735	
chr28	9671397	2	304	C:0.375	T:0.625	
chr28	9671732	2	304	A:0.503289	G:0.496711	
chr28	9671764	2	306	C:0.954248	T:0.0457516	
chr28	9671862	2	316	T:0.411392	C:0.588608	
chr28	9671913	2	306	A:0.957516	G:0.0424837	
chr28	9671943	2	308	A:0.454545	ACTGT:0.545455	
chr28	9672157	2	312	C:0.951923	CT:0.0480769	
chr28	9672165	2	312	T:0.50641	A:0.49359	
chr28	9672255	2	298	T:0.503356	G:0.496644	
chr28	9672418	2	310	A:0.909677	G:0.0903226	
chr28	9672819	2	298	C:0.966443	CT:0.033557	
chr28	9672838	2	304	C:0.582237	T:0.417763	
chr28	9672902	2	278	A:0.913669	G:0.0863309	
chr28	9672992	2	252	T:0.912698	C:0.0873016	
chr28	9673011	2	252	A:0.912698	C:0.0873016	
chr28	9673012	2	236	T:0.957627	C:0.0423729	
chr28	9673119	2	116	C:0.793103	T:0.206897	
chr28	9673653	2	234	CA:0.534188	C:0.465812	
chr28	9673654	2	234	A:0.752137	C:0.247863	
chr28	9673757	2	280	C:0.964286	T:0.0357143	
chr28	9674395	2	224	C:0.700893	T:0.299107	
chr28	9674746	2	312	G:0.99359	T:0.00641026	
chr28	9674957	2	254	T:0.692913	TA:0.307087	
chr28	9675054	2	306	G:0.892157	GA:0.107843	
chr28	9675246	2	306	C:0.470588	CT:0.529412	
chr28	9675392	2	308	G:0.935065	A:0.0649351	
chr28	9675737	2	306	C:0.503268	T:0.496732	
chr28	9676395	2	298	C:0.949664	T:0.0503356	
chr28	9676547	2	292	C:0.486301	T:0.513699	
chr28	9676732	2	304	G:0.496711	A:0.503289	
chr28	9676832	2	306	T:0.48366	C:0.51634	
chr28	9677014	2	292	A:0.486301	G:0.513699	

chr28	9677144	2	298	A:0.479866	T:0.520134		
chr28	9677639	2	308	A:0.5	ATTG:0.5		
chr28	9677825	2	306	G:0.496732	A:0.503268		
chr28	9677987	2	308	G:0.980519	C:0.0194805		
chr28	9678142	2	308	A:0.50974	C:0.49026		
chr28	9678350	2	312	C:0.50641	A:0.49359		
chr28	9678399	2	304	G:0.509868	A:0.490132		
chr28	9678745	2	310	G:0.970968	T:0.0290323		
chr28	9679435	2	296	A:0.486486	G:0.513514		
chr28	9679624	2	288	A:0.975694	C:0.0243056		
chr28	9679710	2	298	A:0.949664	G:0.0503356		
chr28	9680298	2	306	C:0.486928	T:0.513072		
chr28	9680322	2	306	T:0.486928	C:0.513072		
chr28	9680453	2	306	C:0.571895	T:0.428105		
chr28	9680576	2	308	C:0.954545	T:0.0454545		
chr28	9680940	2	284	A:0.507042	G:0.492958		
chr28	9681149	2	298	G:0.5	A:0.5		
chr28	9681230	2	296	T:0.496622	C:0.503378		
chr28	9681350	2	284	C:0.485915	T:0.514085		
chr28	9681525	2	312	C:0.903846	T:0.0961538		
chr28	9681616	2	286	G:0.493007	A:0.506993		
chr28	9682796	2	304	A:0.963816	T:0.0361842		
chr28	9684073	2	294	A:0.962585	G:0.037415		
chr28	9684650	2	298	G:0.530201	GTA:0.469799		
chr28	9684944	2	292	T:0.458904	C:0.541096		
chr28	9685059	2	298	C:0.560403	CCTCTCT:0.439597		
chr28	9685108	4	292	TAA:0.34589	T:0.44863	TA:0.0650685	TAAA:0.140411
chr28	9686194	2	284	G:0.538732	A:0.461268		
chr28	9687510	2	302	G:0.980132	A:0.0198675		
chr28	9687764	2	302	A:0.437086	C:0.562914		
chr28	9688323	2	292	C:0.469178	G:0.530822		
chr28	9688775	2	304	A:0.953947	G:0.0460526		
chr28	9690095	2	312	A:0.961538	AAAT:0.0384615		
chr28	9690750	2	304	C:0.963816	CAG:0.0361842		
chr28	9691046	2	312	T:0.977564	TAA:0.0224359		
chr28	9691065	2	298	AAAT:0.875839	A:0.124161		
chr28	9691066	2	302	AAT:0.834437	A:0.165563		
chr28	9691068	3	312	TAA:0.942308	T:0.00641026	TA:0.0512821	
chr28	9691517	2	304	T:0.960526	A:0.0394737		
chr28	9692141	2	304	A:0.960526	T:0.0394737		
chr28	9692875	2	290	C:0.951724	T:0.0482759		
chr28	9693030	2	294	C:0.94898	CG:0.0510204		
chr28	9693291	2	284	G:0.440141	A:0.559859		
chr28	9693366	2	294	A:0.435374	G:0.564626		
chr28	9694301	3	300	ACT:0.85	A:0.0866667	ACTCT: 0.0633333	
chr28	9694803	2	304	A:0.967105	C:0.0328947		
chr28	9695387	2	174	TGGGGG:0.408046	T:0.591954		
chr28	9696099	2	308	G:0.444805	T:0.555195		
chr28	9696868	2	308	T:0.954545	C:0.0454545		
chr28	9696922	2	306	C:0.924837	T:0.0751634		
chr28	9697041	3	302	A:0.304636	ATTTCTTTC:		

0.483444 ATTTCTTTCTTTC:0.211921
chr28 9697490 2 304 G:0.990132 A:0.00986842
chr28 9697717 2 302 A:0.97351 T:0.0264901
chr28 9697967 2 288 A:0.46875 AT:0.53125
chr28 9698894 2 312 CTTTTTTTTTTTT:0.913462 C:
0.0865385
chr28 9699500 3 298 G:0.516779 GTT:0.352349
GTTT:0.130872
chr28 9701740 2 298 G:0.946309 T:0.0536913
chr28 9703418 2 310 C:0.512903 T:0.487097
chr28 9703677 2 302 T:0.986755 A:0.013245
chr28 9704056 2 306 T:0.934641 C:0.0653595
chr28 9704171 2 298 G:0.95302 A:0.0469799
chr28 9704381 2 302 C:0.960265 T:0.0397351
chr28 9704426 2 302 T:0.417219 C:0.582781
chr28 9704625 2 298 C:0.949664 T:0.0503356
chr28 9704945 2 294 G:0.442177 A:0.557823
chr28 9706082 2 308 T:0.5 C:0.5
chr28 9706194 2 298 G:0.432886 T:0.567114
chr28 9706698 2 312 C:0.426282 T:0.573718
chr28 9707018 2 310 C:0.554839 CAG:0.445161
chr28 9707475 2 296 T:0.662162 C:0.337838
chr28 9708142 2 308 G:0.63961 A:0.36039
chr28 9708223 4 318 TAATAAAATAAAATAA:0.147799 T:
0.525157 TAATAAAATAA:0.106918 TAATAAAATAAAATAAAATAA:0.220126
chr28 9710033 2 294 A:0.0952381 G:0.904762
chr28 9710282 3 316 CATAGAT:0.696203 C:0.208861
CATAGATATAGAT:0.0949367
chr28 9710299 2 316 A:0.911392 ATC:0.0886076
chr28 9710301 2 316 A:0.911392 AGATCTAGATC:
0.0886076
chr28 9710322 2 316 G:0.908228 GATATAGAT:
0.0917722
chr28 9710326 2 314 G:0.904459 T:0.0955414
chr28 9710328 3 316 T:0.806962 G:0.0981013
TATAG:0.0949367
chr28 9711031 2 310 AGTGT:0.667742 A:0.332258
chr28 9711258 2 298 G:0.959732 A:0.0402685
chr28 9712579 2 300 T:0.933333 TA:0.0666667
chr28 9713224 2 302 C:0.983444 G:0.0165563
chr28 9713970 2 292 A:0.561644 G:0.438356
chr28 9714649 2 318 CAATTTAATTACTTCAATTAAAAAA:0.132075
C:0.867925
chr28 9714674 2 318 A:0.132075 AGG:0.867925
chr28 9715061 2 298 T:0.879195 TA:0.120805
chr28 9715135 2 306 T:0.944444 A:0.0555556
chr28 9715311 2 304 C:0.986842 T:0.0131579
chr28 9715329 3 308 TA:0.675325 T:0.217532
TAA:0.107143
chr28 9715348 2 312 T:0.426282 C:0.573718
chr28 9715604 2 298 G:0.45302 T:0.54698
chr28 9716009 2 302 G:0.543046 A:0.456954
chr28 9716417 2 292 CT:0.59589 C:0.40411
chr28 9717522 2 302 G:0.950331 A:0.0496689

chr28 9717530 5 314 ATGTG:0.146497 A:0.286624
ATG:0.0605096 ATGTGTGTGTG:0.127389 ATGTGTGTGTGTG:0.378981
chr28 9717732 2 308 C:0.987013 T:0.012987
chr28 9717766 2 302 ACT:0.456954 A:0.543046
chr28 9718146 2 302 T:0.546358 C:0.453642
chr28 9718514 2 306 A:0.447712 G:0.552288
chr28 9718616 2 258 TA:0.689922 T:0.310078
chr28 9718710 2 294 A:0.438776 C:0.561224
chr28 9719127 2 302 G:0.57947 T:0.42053
chr28 9719292 2 300 C:0.436667 T:0.563333
chr28 9719408 2 306 G:0.960784 A:0.0392157
chr28 9719755 2 306 A:0.69281 C:0.30719
chr28 9720134 2 304 C:0.960526 T:0.0394737
chr28 9721639 2 290 T:0.5 C:0.5
chr28 9721686 2 300 C:0.993333 T:0.00666667
chr28 9721729 2 298 C:0.52349 T:0.47651
chr28 9722300 2 306 C:0.46732 T:0.53268
chr28 9722335 2 302 C:0.533113 T:0.466887
chr28 9722472 2 306 G:0.95098 A:0.0490196
chr28 9722667 2 294 A:0.517007 C:0.482993
chr28 9722668 2 292 GT:0.643836 G:0.356164
chr28 9722959 2 300 C:0.976667 T:0.0233333
chr28 9723206 2 272 A:0.985294 G:0.0147059
chr28 9723630 2 294 G:0.503401 A:0.496599
chr28 9723919 2 302 C:0.509934 G:0.490066
chr28 9723997 2 302 G:0.476821 A:0.523179
chr28 9724006 2 310 A:0.990323 G:0.00967742
chr28 9724574 2 306 G:0.977124 C:0.0228758
chr28 9724633 2 302 T:0.980132 C:0.0198675
chr28 9724744 2 308 C:0.983766 T:0.0162338
chr28 9724827 2 300 A:0.986667 G:0.0133333
chr28 9724894 2 296 T:0.983108 C:0.0168919
chr28 9724895 2 296 T:0.983108 C:0.0168919
chr28 9725235 2 318 A:0.990566 AC:0.00943396
chr28 9725236 2 318 A:0.990566 T:0.00943396
chr28 9725255 2 318 T:0.990566 C:0.00943396
chr28 9725275 2 318 T:0.990566 C:0.00943396
chr28 9725447 2 302 T:0.986755 G:0.013245
chr28 9725570 2 302 G:0.460265 A:0.539735
chr28 9725663 2 300 G:0.986667 A:0.0133333
chr28 9725823 2 304 T:0.986842 C:0.0131579
chr28 9725868 2 308 TG:0.626623 T:0.373377
chr28 9725883 2 300 A:0.986667 C:0.0133333
chr28 9725982 2 304 T:0.990132 C:0.00986842
chr28 9726140 2 300 CT:0.91 C:0.09
chr28 9726488 2 302 T:0.94702 C:0.0529801
chr28 9726505 2 300 TA:0.466667 T:0.533333
chr28 9727078 2 302 T:0.466887 C:0.533113
chr28 9727823 2 302 C:0.960265 T:0.0397351
chr28 9727831 2 310 AAAACGT:0.470968 A:0.529032
chr28 9728288 2 310 T:0.519355 C:0.480645
chr28 9728361 2 308 A:0.896104 T:0.103896
chr28 9728491 2 304 T:0.467105 G:0.532895
chr28 9729023 2 300 G:0.613333 A:0.386667

chr28 9729591 4 318 TAATGAATG:0.191824 T:
0.235849 TAATG:0.534591 TAATGAATGAATG:0.0377358
chr28 9730419 2 304 T:0.638158 A:0.361842
chr28 9730820 2 306 T:0.0882353 C:0.911765
chr28 9730882 2 308 T:0.883117 C:0.116883
chr28 9731081 2 296 C:0.530405 T:0.469595
chr28 9731210 2 312 A:0.974359 AT:0.025641
chr28 9731212 2 312 A:0.974359 T:0.025641
chr28 9731223 2 306 GAT:0.464052 G:0.535948
chr28 9731276 2 304 C:0.532895 T:0.467105
chr28 9731289 2 304 T:0.532895 G:0.467105
chr28 9731315 2 306 CAAT:0.513072 C:0.486928
chr28 9731461 2 308 C:0.538961 T:0.461039
chr28 9731643 2 306 A:0.620915 G:0.379085
chr28 9731646 2 306 G:0.957516 A:0.0424837
chr28 9732250 2 302 C:0.834437 G:0.165563
chr28 9732289 2 310 TCC:0.629032 T:0.370968
chr28 9732713 2 300 A:0.63 C:0.37
chr28 9732824 2 310 C:0.919355
CTATATATATAACTATA:0.0806452
chr28 9733050 2 308 C:0.886364 T:0.113636
chr28 9733070 2 308 A:0.636364 G:0.363636
chr28 9733084 2 306 C:0.885621 T:0.114379
chr28 9733147 2 286 T:0.888112 A:0.111888
chr28 9733150 2 286 C:0.888112 T:0.111888
chr28 9733564 2 306 G:0.643791 A:0.356209
chr28 9733818 2 304 CAGAA:0.641447 C:0.358553
chr28 9733823 2 304 A:0.641447 C:0.358553
chr28 9733861 2 296 A:0.986486 G:0.0135135
chr28 9734028 2 296 T:0.469595 C:0.530405
chr28 9734239 2 312 T:0.323718 C:0.676282
chr28 9734628 3 306 G:0.20915 GTC:0.379085
GTCTC:0.411765
chr28 9734706 2 298 A:0.627517 T:0.372483
chr28 9734721 3 292 CAA:0.482877 C:0.373288
CAAA:0.143836
chr28 9734868 2 292 G:0.787671 C:0.212329
chr28 9736579 2 302 A:0.662252 G:0.337748
chr28 9736608 2 288 A:0.996528 T:0.00347222
chr28 9736672 2 268 T:0.656716 TAAG:0.343284
chr28 9736841 2 292 A:0.356164 G:0.643836
chr28 9737006 4 314 A:0.292994 ATG:0.226115
ATGTGTGTGTGTGTGTG:0.286624 ATGTGTGTGTGTGTGTGTG:0.194268
chr28 9737028 2 290 A:0.365517 ATT:0.634483
chr28 9737409 2 300 T:0.35 C:0.65
chr28 9737548 3 296 GT:0.307432 G:0.621622
GTT:0.0709459
chr28 9737645 2 300 A:0.343333 C:0.656667
chr28 9737667 2 298 C:0.661074 T:0.338926
chr28 9737805 2 308 A:0.350649 G:0.649351
chr28 9737930 2 300 T:0.793333 C:0.206667
chr28 9738289 2 302 CA:0.837748 C:0.162252
chr28 9738986 2 292 T:0.84589 C:0.15411
chr28 9739027 2 282 G:0.815603 A:0.184397

chr28	9739349	2	310	G:0.796774	A:0.203226		
chr28	9739370	2	310	T:0.796774	C:0.203226		
chr28	9739420	2	312	C:0.782051	T:0.217949		
chr28	9739441	2	312	T:0.782051	C:0.217949		
chr28	9739464	2	312	T:0.782051	C:0.217949		
chr28	9739475	2	312	G:0.782051	A:0.217949		
chr28	9739585	2	304	C:0.796053	T:0.203947		
chr28	9739593	2	308	G:0.948052	A:0.0519481		
chr28	9739661	2	304	C:0.654605	T:0.345395		
chr28	9739683	2	296	G:0.814189	T:0.185811		
chr28	9739715	3	304	A:0.861842	C:0.105263	T:0.0328947	
chr28	9739815	2	314	G:0.821656	A:0.178344		
chr28	9739860	2	304	C:0.825658	T:0.174342		
chr28	9739865	2	304	C:0.825658	G:0.174342		
chr28	9739866	2	302	A:0.887417	G:0.112583		
chr28	9739947	2	304	G:0.828947	C:0.171053		
chr28	9740055	2	304	G:0.164474	A:0.835526		
chr28	9740120	2	308	C:0.827922	T:0.172078		
chr28	9740234	2	290	G:0.351724	A:0.648276		
chr28	9740295	2	292	C:0.945205	T:0.0547945		
chr28	9740534	2	308	G:0.941558	A:0.0584416		
chr28	9740867	2	298	G:0.35906	A:0.64094		
chr28	9740886	2	308	C:0.788961	T:0.211039		
chr28	9740999	2	306	CT:0.954248	C:0.0457516		
chr28	9741008	4	300	GT:0.176667	G:0.636667	GTT:0.136667	GTTT:0.05
chr28	9741185	2	308	TCTC:0.795455	T:0.204545		
chr28	9741427	2	306	TTC:0.79085	T:0.20915		
chr28	9741441	2	306	T:0.79085	C:0.20915		
chr28	9741450	2	306	T:0.79085	C:0.20915		
chr28	9741626	2	298	A:0.795302	C:0.204698		
chr28	9741663	2	296	G:0.601351	A:0.398649		
chr28	9741924	2	308	G:0.801948	A:0.198052		
chr28	9741942	2	310	AATTG:0.974194	A:0.0258065		
chr28	9742027	2	292	T:0.109589	C:0.890411		
chr28	9742045	2	298	C:0.818792	T:0.181208		
chr28	9742095	2	304	A:0.809211	AT:0.190789		
chr28	9742175	2	306	G:0.0980392	A:0.901961		
chr28	9742286	2	302	C:0.798013	T:0.201987		
chr28	9742324	2	302	C:0.804636	T:0.195364		
chr28	9742332	2	304	A:0.657895	G:0.342105		
chr28	9742394	2	306	G:0.79085	A:0.20915		
chr28	9742571	2	304	G:0.878289	A:0.121711		
chr28	9742760	2	302	T:0.678808	G:0.321192		
chr28	9742857	2	306	G:0.80719	C:0.19281		
chr28	9743064	2	310	T:0.809677	A:0.190323		
chr28	9743116	2	306	T:0.816993	TA:0.183007		
chr28	9743160	2	300	C:0.806667	A:0.193333		
chr28	9743176	2	302	A:0.807947	C:0.192053		
chr28	9743185	2	302	G:0.63245	A:0.36755		
chr28	9743202	2	306	A:0.803922	G:0.196078		
chr28	9743328	2	316	T:0.696203	C:0.303797		
chr28	9743590	2	320	G:0.94375	GGGAT:0.05625		

chr28	9743591	4	320	GGATAGATA:0.625	G:0.065625	GGATA:0.21875	GGATAGATAGATA:0.090625
chr28	9743595	2	316	A:0.981013	G:0.0189873		
chr28	9743634	3	320	T:0.83125	TAGATAG:0.059375	TGATAGATAGATAG:0.109375	
chr28	9743837	2	298	G:0.95302	C:0.0469799		
chr28	9743838	2	298	T:0.64094	C:0.35906		
chr28	9743960	2	298	A:0.338926	T:0.661074		
chr28	9744247	2	308	C:0.75	A:0.25		
chr28	9744622	2	300	A:0.776667	G:0.223333		
chr28	9744849	2	310	G:0.76129	A:0.23871		
chr28	9745014	2	306	G:0.764706	T:0.235294		
chr28	9745028	2	306	T:0.95098	TA:0.0490196		
chr28	9745205	2	302	C:0.983444	T:0.0165563		
chr28	9745251	2	312	T:0.740385	C:0.259615		
chr28	9745261	2	312	G:0.740385	A:0.259615		
chr28	9745314	2	306	G:0.960784	A:0.0392157		
chr28	9746163	2	302	T:0.483444	G:0.516556		
chr28	9746181	2	310	C:0.490323	T:0.509677		
chr28	9746220	2	312	C:0.49359	T:0.50641		
chr28	9746611	2	294	TC:0.952381	T:0.047619		
chr28	9746612	2	294	C:0.506803	T:0.493197		
chr28	9746758	2	304	T:0.953947	C:0.0460526		
chr28	9747095	2	290	T:0.941379	TAC:0.0586207		
chr28	9747240	2	316	TC:0.898734	T:0.101266		
chr28	9747525	2	300	A:0.92	T:0.08		
chr28	9747633	2	284	C:0.96831	T:0.0316901		
chr28	9747866	2	300	C:0.333333	T:0.666667		
chr28	9747984	2	300	A:0.12	G:0.88		
chr28	9748265	2	308	G:0.938312	A:0.0616883		
chr28	9748530	2	302	G:0.503311	A:0.496689		
chr28	9749203	2	306	A:0.901961	G:0.0980392		
chr28	9749315	2	286	C:0.933566	CT:0.0664336		
chr28	9749494	2	308	A:0.373377	G:0.626623		
chr28	9749795	2	290	A:0.22069	G:0.77931		
chr28	9749831	2	294	GC:0.261905	G:0.738095		
chr28	9750270	2	304	G:0.953947	C:0.0460526		
chr28	9750347	2	302	A:0.0960265	C:0.903974		
chr28	9750948	2	300	A:0.123333	G:0.876667		
chr28	9751061	2	290	C:0.910345	G:0.0896552		
chr28	9751265	2	300	C:0.95	T:0.05		
chr28	9751372	2	292	C:0.958904	T:0.0410959		
chr28	9751465	4	298	AT:0.677852	A:0.11745	ATT:0.0738255	ATTTT:0.130872
chr28	9752140	3	290	C:0.303448	CTG:0.558621	CTGTG:0.137931	
chr28	9752675	2	312	G:0.945513	A:0.0544872		
chr28	9752676	2	312	A:0.945513	G:0.0544872		
chr28	9753535	2	290	C:0.382759	A:0.617241		
chr28	9753816	2	294	G:0.336735	A:0.663265		
chr28	9754192	2	294	T:0.673469	C:0.326531		
chr28	9754329	2	302	T:0.31457	C:0.68543		
chr28	9754954	2	316	T:0.968354	TTTTTTA:0.0316456		
chr28	9754961	2	316	A:0.968354			

ATTTATTTTTTTTTAAATTT:0.0316456
chr28 9755512 2 298 G:0.889262 A:0.110738
chr28 9755568 2 308 C:0.444805 A:0.555195
chr28 9755764 2 280 G:0.4 T:0.6
chr28 9755816 2 296 A:0.439189 G:0.560811
chr28 9755909 2 298 CAAATAATAATAATAATAA:0.946309
C:0.0536913
chr28 9755910 6 312 AAATAATAATAATAAT:0.272436 A:
0.0608974 AAAT:0.0416667 AAATAAT:0.221154 AAATAATAAT:
0.198718 AAATAATAATAAT:0.205128
chr28 9756064 2 274 A:0.412409 G:0.587591
chr28 9756106 2 278 C:0.442446 G:0.557554
chr28 9756139 2 310 T:0.922581 TA:0.0774194
chr28 9756756 2 300 A:0.94 G:0.06
chr28 9756881 2 302 A:0.453642 G:0.546358
chr28 9757016 2 306 C:0.398693 G:0.601307
chr28 9757207 2 314 T:0.929936 A:0.0700637
chr28 9757230 2 310 C:0.877419 T:0.122581
chr28 9757270 2 320 TCCTTCTCTCC:0.9375 T:0.0625
chr28 9757282 2 320 T:0.9375 G:0.0625
chr28 9757346 2 320 TTCTCTCCATAAGTAATGATC:0.9375
T:0.0625
chr28 9757454 2 298 C:0.416107 T:0.583893
chr28 9757710 2 296 A:0.956081 G:0.0439189
chr28 9758156 2 296 C:0.344595 CTTTTTTTT:
0.655405
chr28 9758433 2 304 TA:0.256579 T:0.743421
chr28 9758746 2 304 T:0.463816 A:0.536184
chr28 9758811 2 300 C:0.893333 T:0.106667
chr28 9759090 2 306 GA:0.931373 G:0.0686275
chr28 9759127 2 308 G:0.821429 GAA:0.178571
chr28 9759435 2 296 G:0.847973 C:0.152027
chr28 9759494 2 246 T:0.0528455 TAA:0.947154
chr28 9759580 2 300 C:0.816667 A:0.183333
chr28 9759649 2 296 T:0.841216 C:0.158784
chr28 9759874 2 296 T:0.847973 C:0.152027
chr28 9759938 2 304 A:0.243421 G:0.756579
chr28 9759994 2 294 G:0.840136 A:0.159864
chr28 9760373 2 304 T:0.838816 C:0.161184
chr28 9760542 2 294 G:0.836735 A:0.163265
chr28 9760660 2 298 C:0.92953 T:0.0704698
chr28 9761086 2 296 GA:0.891892 G:0.108108
chr28 9761098 2 298 A:0.949664 C:0.0503356
chr28 9761245 2 284 C:0.795775 CCT:0.204225
chr28 9761552 2 314 T:0.83121 G:0.16879
chr28 9761728 2 304 G:0.486842 GT:0.513158
chr28 9762479 2 302 TG:0.834437 T:0.165563
chr28 9762767 2 308 AAG:0.970779 A:0.0292208
chr28 9763333 2 306 A:0.95098 T:0.0490196
chr28 9763478 2 312 C:0.163462 T:0.836538
chr28 9763486 2 312 T:0.11859 C:0.88141
chr28 9764176 2 296 C:0.14527 G:0.85473
chr28 9764669 2 298 G:0.16443 C:0.83557
chr28 9764759 2 318 G:0.95283

GAAATACAGATGTCAAAAAAAAAAAAA:0.0471698
chr28 9765181 2 300 C:0.91 T:0.09
chr28 9765523 2 298 T:0 G:1
chr28 9765634 2 300 C:0.706667 G:0.293333
chr28 9765903 2 298 C:0.939597 T:0.0604027
chr28 9766373 2 230 T:0.730435 C:0.269565
chr28 9766593 2 302 GAGAAAGAAAAGAAGAAAGAAAGAAGA:
0.274834 G:0.725166
chr28 9766676 2 296 AAG:0.304054 A:0.695946
chr28 9766683 2 296 G:0.304054 GA:0.695946
chr28 9766993 2 308 G:0.25974 A:0.74026
chr28 9767153 2 304 C:0.950658 T:0.0493421
chr28 9767192 2 294 T:0.894558 C:0.105442
chr28 9767386 2 306 T:0.95098 TTAGA:0.0490196
chr28 9767447 2 308 T:0.253247 C:0.746753
chr28 9767480 2 302 G:0.735099 C:0.264901
chr28 9767673 2 314 G:0.738854 A:0.261146
chr28 9768353 2 302 A:0.258278 G:0.741722
chr28 9768697 2 306 C:0.434641 T:0.565359
chr28 9769049 2 302 A:0.490066 AT:0.509934
chr28 9769050 2 300 T:0.953333 TA:0.0466667
chr28 9770759 2 304 G:0.953947 C:0.0460526
chr28 9771167 2 308 C:0.542208 T:0.457792
chr28 9771342 2 302 C:0.456954 T:0.543046
chr28 9771538 2 298 T:0.627517 TA:0.372483
chr28 9771702 2 306 C:0.69281 T:0.30719
chr28 9771730 2 308 A:0.396104 G:0.603896
chr28 9772152 2 300 G:0.623333 T:0.376667
chr28 9772348 2 302 A:0.599338 G:0.400662
chr28 9772580 2 306 A:0.598039 G:0.401961
chr28 9772908 2 306 T:0.611111 C:0.388889
chr28 9772982 2 314 ATG:0.627389 A:0.372611
chr28 9772985 2 314 CCAGTGTTAA:0.627389 C:
0.372611
chr28 9773464 2 300 C:0.65 T:0.35
chr28 9773787 2 292 A:0.599315 G:0.400685
chr28 9773853 2 288 A:0.604167 G:0.395833
chr28 9773867 2 294 C:0.608844 T:0.391156
chr28 9774139 2 300 G:0.8 A:0.2
chr28 9774505 2 310 A:0.664516 G:0.335484
chr28 9774726 2 306 C:0.457516 T:0.542484
chr28 9774815 2 302 C:0.847682 T:0.152318
chr28 9775141 2 306 G:0.519608 A:0.480392
chr28 9775184 2 302 T:0.953642 TC:0.0463576
chr28 9775707 2 302 C:0.612583 T:0.387417
chr28 9775881 2 304 G:0.618421 GAA:0.381579
chr28 9776378 2 294 T:0.469388 C:0.530612
chr28 9776533 2 304 C:0.513158 T:0.486842
chr28 9776550 2 300 T:0.836667 TC:0.163333
chr28 9777051 2 304 G:0.842105 A:0.157895
chr28 9777185 2 308 G:0.636364 C:0.363636
chr28 9777308 2 302 T:0.625828 C:0.374172
chr28 9777424 2 298 T:0.64094 C:0.35906
chr28 9777623 2 308 T:0.623377 C:0.376623

chr28	9777957	2	306	C:0.970588	A:0.0294118
chr28	9777990	2	302	T:0.615894	A:0.384106
chr28	9778333	2	302	C:0.874172	T:0.125828
chr28	9778511	2	302	C:0.619205	T:0.380795
chr28	9778650	2	310	CT:0.629032	C:0.370968
chr28	9778713	2	306	T:0.513072	G:0.486928
chr28	9779028	2	302	A:0.400662	G:0.599338
chr28	9779184	2	302	A:0.125828	G:0.874172
chr28	9779188	2	306	A:0.905229	G:0.0947712
chr28	9779472	2	300	C:0.976667	T:0.0233333
chr28	9779502	2	304	G:0.473684	A:0.526316
chr28	9779585	2	304	G:0.898026	A:0.101974
chr28	9779744	2	300	G:0.943333	A:0.0566667
chr28	9779765	2	306	C:0.529412	T:0.470588
chr28	9779849	2	298	G:0.114094	C:0.885906
chr28	9779986	2	300	C:0.9	G:0.1
chr28	9780107	2	294	T:0.721088	C:0.278912
chr28	9780582	2	234	GAAAA:0	G:1
chr28	9780586	2	234	A:0	AGGCGC:1
chr28	9781109	2	236	CT:0.567797	C:0.432203
chr28	9781370	2	284	G:0.556338	A:0.443662
chr28	9781580	2	302	T:0.34106	C:0.65894
chr28	9781610	2	292	A:0.311644	G:0.688356
chr28	9781893	2	300	C:0.576667	T:0.423333
chr28	9782443	2	294	T:0.591837	G:0.408163
chr28	9782499	2	294	G:0.761905	A:0.238095
chr28	9782808	2	194	A:0.778351	T:0.221649
chr28	9782809	2	194	C:0.778351	A:0.221649
chr28	9782819	2	178	G:0.578652	T:0.421348
chr28	9782979	2	260	C:0.792308	T:0.207692
chr28	9783183	2	300	C:0	G:1
chr28	9783199	2	300	T:0.776667	C:0.223333
chr28	9783448	2	288	T:0.565972	C:0.434028
chr28	9783582	2	302	C:0.764901	A:0.235099
chr28	9783784	2	302	A:0.774834	G:0.225166
chr28	9783843	2	302	G:0.34106	T:0.65894
chr28	9783881	2	306	T:0.336601	G:0.663399
chr28	9784009	2	312	G:0.310897	C:0.689103
chr28	9784056	2	308	G:0.584416	A:0.415584
chr28	9784260	2	294	C:0.581633	T:0.418367
chr28	9784474	2	292	T:0.756849	C:0.243151
chr28	9784506	2	292	C:0.585616	T:0.414384
chr28	9784586	2	302	C:0.983444	G:0.0165563
chr28	9784588	2	300	G:0.576667	A:0.423333
chr28	9784622	2	292	T:0.777397	C:0.222603
chr28	9784780	2	306	T:0.764706	G:0.235294
chr28	9785095	2	292	C:0.773973	A:0.226027
chr28	9785624	2	300	C:0.77	T:0.23
chr28	9785777	2	300	T:0.77	C:0.23
chr28	9785850	2	304	G:0.779605	A:0.220395
chr28	9785965	2	306	T:0.650327	G:0.349673
chr28	9786023	2	308	G:0.993506	A:0.00649351
chr28	9786138	2	286	A:0.636364	G:0.363636
chr28	9786603	2	306	G:0.764706	T:0.235294

chr28	9786794	2	296	G:0.77027	A:0.22973
chr28	9786881	2	304	C:0.746711	T:0.253289
chr28	9786990	2	294	C:0.697279	T:0.302721
chr28	9787175	2	296	A:0.743243	G:0.256757
chr28	9787339	2	300	C:0.763333	T:0.236667
chr28	9787422	2	296	T:0.763514	C:0.236486
chr28	9787520	2	290	T:0.772414	G:0.227586
chr28	9787968	2	310	A:0.758065	G:0.241935
chr28	9788045	2	300	G:0.766667	A:0.233333
chr28	9788135	2	302	T:0.761589	A:0.238411
chr28	9788454	2	304	G:0.759868	A:0.240132
chr28	9788807	2	302	G:0.768212	A:0.231788
chr28	9788899	2	312	T:0.740385	A:0.259615
chr28	9789035	2	282	CT:0.751773	C:0.248227
chr28	9789068	2	298	C:0.795302	CAAAT:0.204698
chr28	9789180	2	302	C:0.745033	T:0.254967
chr28	9789612	2	292	T:0.763699	C:0.236301
chr28	9789645	2	292	G:0.113014	A:0.886986
chr28	9789859	2	284	C:0.792254	A:0.207746
chr28	9790016	2	306	T:0.787582	TAGCC:0.212418
chr28	9790026	2	286	TA:0.867133	T:0.132867
chr28	9790074	2	284	C:0.739437	T:0.260563
chr28	9790405	2	296	C:0.915541	T:0.0844595
chr28	9790419	2	296	G:0.763514	A:0.236486
chr28	9790455	2	288	T:0.118056	C:0.881944
chr28	9790526	2	292	A:0.736301	G:0.263699
chr28	9790662	2	296	C:0.773649	T:0.226351
chr28	9791240	2	310	A:0.767742	AAG:0.232258
chr28	9791601	2	306	G:0.764706	A:0.235294
chr28	9791611	2	306	C:0.764706	T:0.235294
chr28	9792278	2	280	T:0.417857	TA:0.582143
chr28	9792359	2	306	G:0.767974	T:0.232026
chr28	9792736	2	300	C:0.973333	T:0.0266667
chr28	9792838	2	298	G:0.808725	C:0.191275
chr28	9792888	2	304	AC:0.796053	A:0.203947
chr28	9793291	2	308	G:0.993506	A:0.00649351
chr28	9793351	2	312	C:0.967949	T:0.0320513
chr28	9793488	2	290	G:0.965517	A:0.0344828
chr28	9793596	2	312	C:0.798077	T:0.201923
chr28	9793667	2	310	G:0.812903	A:0.187097
chr28	9793740	2	306	G:0.800654	T:0.199346
chr28	9793897	2	294	G:0.812925	A:0.187075
chr28	9793969	2	300	A:0.786667	G:0.213333
chr28	9793999	2	284	G:0.630282	GA:0.369718
chr28	9794014	2	294	AT:0.785714	A:0.214286
chr28	9794053	2	288	A:0.777778	G:0.222222
chr28	9794505	2	300	A:0.99	G:0.01
chr28	9794625	2	304	A:0.970395	G:0.0296053
chr28	9795001	2	292	C:0.982877	T:0.0171233
chr28	9795058	2	290	C:0.72069	G:0.27931
chr28	9795444	2	304	A:0.0361842	G:0.963816
chr28	9795515	2	298	T:0.033557	C:0.966443
chr28	9795678	2	298	A:0.781879	G:0.218121
chr28	9795826	2	308	CT:0.967532	C:0.0324675

chr28	9795946	2	302	A:0.0397351	G:0.960265
chr28	9795986	2	302	T:0.983444	C:0.0165563
chr28	9796648	2	302	C:0.0596026	T:0.940397
chr28	9796896	2	292	T:0.643836	C:0.356164
chr28	9797057	2	304	C:0.6875	A:0.3125
chr28	9797235	2	294	T:0.959184	C:0.0408163
chr28	9797265	2	298	T:0.647651	C:0.352349
chr28	9797266	2	302	G:0.701987	A:0.298013
chr28	9797282	2	300	C:0.946667	T:0.0533333
chr28	9797798	2	302	G:0.950331	A:0.0496689
chr28	9798027	2	308	C:0.548701	T:0.451299
chr28	9798108	2	306	G:0.947712	A:0.0522876
chr28	9798234	2	314	T:0.942675	C:0.0573248
chr28	9800437	2	306	A:0.0392157	G:0.960784
chr28	9800547	5	308	C:0.172078	CA:0.126623
CAA:0.136364	CAAAAA:0.386364	CAAAAAAA:0.178571
chr28	9801138	2	300	C:0.96	T:0.04
chr28	9801139	2	300	A:0.96	G:0.04
chr28	9801402	2	290	G:0.968966	T:0.0310345
chr28	9801683	2	288	C:0.920139	T:0.0798611
chr28	9802136	2	298	C:0.932886	G:0.0671141
chr28	9802306	2	290	C:0.972414	T:0.0275862
chr28	9802363	4	300	C:0.08	CA:0.0466667	CAAAA:
0.313333	CAAAAA:0.56
chr28	9802788	2	310	G:0.103226	A:0.896774
chr28	9802920	3	294	GAA:0.166667	G:0.129252
GA:0.704082
chr28	9803120	2	310	C:0.687097	T:0.312903
chr28	9803834	2	302	G:0.715232	A:0.284768
chr28	9803869	2	290	G:0.696552	A:0.303448
chr28	9804295	2	308	G:0.694805	A:0.305195
chr28	9804465	2	304	T:0.690789	C:0.309211
chr28	9804556	2	314	T:0.687898	TA:0.312102
chr28	9804561	2	314	T:0.687898	C:0.312102
chr28	9804876	2	306	C:0.705882	T:0.294118
chr28	9805551	2	304	T:0.671053	C:0.328947
chr28	9805552	2	304	G:0.671053	C:0.328947
chr28	9805581	2	302	A:0.115894	ACT:0.884106
chr28	9806859	2	298	A:0.996644	G:0.0033557
chr28	9807002	4	310	AAC:0.267742	A:0.151613
AACAC:0.222581	AACACACACACAC:0.358065
chr28	9807618	2	304	A:0.578947	G:0.421053
chr28	9808169	2	308	A:0.928571	C:0.0714286
chr28	9808283	2	296	A:0.415541	G:0.584459
chr28	9808530	2	300	G:0.96	A:0.04
chr28	9809053	2	298	G:0.604027	A:0.395973
chr28	9809286	2	298	G:0.540268	T:0.459732
chr28	9809566	2	300	C:0.61	T:0.39
chr28	9810156	2	302	C:0.586093	G:0.413907
chr28	9810157	2	302	A:0.125828	G:0.874172
chr28	9810435	2	298	G:0.607383	A:0.392617
chr28	9810671	2	308	GAGA:0.561688	G:0.438312
chr28	9810895	2	302	A:0.615894	G:0.384106
chr28	9811110	2	302	G:0.609272	A:0.390728

chr28	9811232	2	294	G:0.585034	C:0.414966	
chr28	9811910	2	298	C:0.583893	T:0.416107	
chr28	9812192	2	310	C:0.580645	T:0.419355	
chr28	9812358	2	300	G:0.58	A:0.42	
chr28	9812908	2	302	T:0.576159	A:0.423841	
chr28	9812918	3	318	AGTGTGTGTGTGTGT:0.147799	A:0.443396	AGTGTGTGTGT:0.408805
chr28	9813361	2	294	T:0.129252	G:0.870748	
chr28	9813377	2	296	G:0.847973	A:0.152027	
chr28	9813701	2	298	C:0.580537	A:0.419463	
chr28	9814165	2	290	C:0.558621	T:0.441379	
chr28	9814419	3	302	GAA:0.549669	G:0.122517	GAAAA:0.327815
chr28	9814618	2	302	G:0.950331	T:0.0496689	
chr28	9814674	2	300	A:0.14	G:0.86	
chr28	9814903	2	306	C:0.555556	T:0.444444	
chr28	9814907	2	306	T:0.980392	A:0.0196078	
chr28	9815148	2	312	A:0.121795	G:0.878205	
chr28	9815254	2	312	T:0.862179	TTAATAA:0.137821	
chr28	9815653	2	296	G:0.138514	A:0.861486	
chr28	9815684	2	306	G:0.924837	A:0.0751634	
chr28	9815850	2	306	G:0.643791	A:0.356209	
chr28	9816208	2	312	T:0.13141	C:0.86859	
chr28	9816217	2	312	C:0.13141	A:0.86859	
chr28	9817044	2	302	A:0.84106	G:0.15894	
chr28	9817417	2	304	G:0.407895	A:0.592105	
chr28	9817556	2	308	T:0.574675	C:0.425325	
chr28	9818005	3	294	A:0.469388	G:0.142857	T:0.387755
chr28	9818139	2	290	G:0.448276	A:0.551724	
chr28	9818302	2	300	C:0.956667	T:0.0433333	
chr28	9818360	2	304	GGTGA:0.710526	G:0.289474	
chr28	9818551	2	298	T:0.446309	C:0.553691	
chr28	9818586	2	294	G:0.42517	A:0.57483	
chr28	9818719	2	306	C:0.431373	T:0.568627	
chr28	9818756	2	300	C:0.426667	T:0.573333	
chr28	9819007	2	302	T:0.443709	C:0.556291	
chr28	9820122	2	302	T:0.983444	A:0.0165563	
chr28	9820376	2	304	G:0.125	C:0.875	
chr28	9821600	2	300	T:0.95	C:0.05	
chr28	9821617	2	302	A:0.675497	AAAAC:0.324503	
chr28	9821736	2	308	G:0.688312	A:0.311688	
chr28	9822122	2	304	GAA:0.983553	G:0.0164474	
chr28	9822210	2	308	C:0.126623	T:0.873377	
chr28	9822230	2	304	A:0.115132	C:0.884868	
chr28	9822659	2	306	G:0.133987	A:0.866013	
chr28	9822684	2	304	C:0.421053	T:0.578947	
chr28	9822815	2	302	T:0.122517	C:0.877483	
chr28	9822998	2	300	C:0.996667	CA:0.00333333	
chr28	9823379	2	300	G:0.683333	T:0.316667	
chr28	9823507	2	304	T:0.986842	C:0.0131579	
chr28	9823706	2	296	G:0.841216	A:0.158784	
chr28	9824113	2	294	A:0.258503	G:0.741497	
chr28	9824436	2	294	T:0.70068	G:0.29932	

chr28	9824554	2	300	C:0.57	T:0.43		
chr28	9824573	2	300	C:0.846667	A:0.153333		
chr28	9824820	2	108	A:0.685185	T:0.314815		
chr28	9824902	2	302	G:0.13245	A:0.86755		
chr28	9825043	2	310	A:0.987097	G:0.0129032		
chr28	9825151	2	310	CTTTTTTTTTT:0.587097	C:0.412903		
chr28	9825239	2	302	T:0.688742	C:0.311258		
chr28	9825274	2	304	T:0.292763	C:0.707237		
chr28	9825381	2	278	T:0.28777	C:0.71223		
chr28	9825714	2	310	C:0.945161	T:0.0548387		
chr28	9825812	2	310	T:0.280645	C:0.719355		
chr28	9826285	2	296	G:0.959459	A:0.0405405		
chr28	9826483	2	310	GAAGT:0.6	G:0.4		
chr28	9826672	2	300	C:0.293333	T:0.706667		
chr28	9827433	2	296	TTTTTC:0.662162	T:0.337838		
chr28	9827734	2	300	A:0.28	C:0.72		
chr28	9827865	2	312	C:0.291667	T:0.708333		
chr28	9828526	2	302	C:0.264901	A:0.735099		
chr28	9828545	2	306	TACACACACAA:0.96732	T:0.0326797		
chr28	9828555	4	318	AACACACACACACAC:0.283019	A:0.31761	AACAC:0.345912	CACACACACACACAC:0.0534591
chr28	9828679	2	304	C:0.851974	A:0.148026		
chr28	9828690	2	308	TACAA:0.298701	T:0.701299		
chr28	9828927	2	308	C:0.305195	T:0.694805		
chr28	9829025	2	308	C:0.691558	T:0.308442		
chr28	9829252	2	310	T:0.277419	C:0.722581		
chr28	9829295	2	310	T:0.277419	A:0.722581		
chr28	9829352	2	316	T:0.287975	G:0.712025		
chr28	9829354	2	316	G:0.287975	A:0.712025		
chr28	9829438	2	304	G:0.289474	A:0.710526		
chr28	9830182	2	308	G:0.272727	T:0.727273		
chr28	9830309	2	296	T:0.297297	TA:0.702703		
chr28	9830679	2	312	T:0.0833333	TA:0.916667		
chr28	9830811	2	298	C:0.983221	A:0.0167785		
chr28	9831040	2	308	G:0.266234	A:0.733766		
chr28	9831068	2	306	C:0.277778	T:0.722222		
chr28	9831687	2	306	T:0.29085	C:0.70915		
chr28	9831751	2	312	T:0.266026	C:0.733974		
chr28	9832026	2	302	A:0.976821	G:0.0231788		
chr28	9832433	2	302	T:0.897351	C:0.102649		
chr28	9832538	2	304	A:0.253289	G:0.746711		
chr28	9832873	2	294	CT:0.278912	C:0.721088		
chr28	9833889	2	304	CTT:0.950658	C:0.0493421		
chr28	9833997	2	292	T:0.328767	C:0.671233		
chr28	9834032	2	260	G:0.403846	C:0.596154		
chr28	9834344	2	296	G:0.260135	A:0.739865		
chr28	9834526	2	310	G:0.680645	A:0.319355		
chr28	9834887	2	294	T:0.268707	G:0.731293		
chr28	9835154	2	316	T:0.727848	C:0.272152		
chr28	9835283	2	304	T:0.654605	G:0.345395		
chr28	9835327	2	308	T:0.681818	C:0.318182		
chr28	9836035	2	306	C:0.70915	G:0.29085		

```
chr28	9836232	2	310	T:0.696774	TGAGAGA:0.303226
chr28	9836370	2	282	C:0.677305	T:0.322695
chr28	9836413	2	280	ATGTG:0.571429	A:0.428571
chr28	9836440	2	280	TGTGTGC:0.742857	T:0.257143
chr28	9836868	2	308	C:0.704545	T:0.295455
chr28	9836958	2	306	C:0.964052	T:0.0359477
chr28	9837299	2	308	C:0.954545	T:0.0454545
chr28	9837424	2	304	C:0.690789	G:0.309211
chr28	9837820	2	312	C:0.673077	T:0.326923
chr28	9838398	2	310	A:0.706452	G:0.293548
chr28	9838524	2	296	G:0.699324	A:0.300676
chr28	9838729	2	308	G:0.659091	A:0.340909
chr28	9839753	3	298	G:0.362416	GAT:0.392617
GATAT:0.244966
chr28	9839853	2	256	C:0.214844	CCT:0.785156
chr28	9840164	2	306	A:0.898693	G:0.101307
chr28	9840384	2	304	T:0.680921	G:0.319079
chr28	9840452	2	306	A:0.653595	T:0.346405
chr28	9840554	2	314	T:0.681529	C:0.318471
chr28	9841083	2	310	C:0.6	G:0.4
chr28	9841148	2	296	C:0.989865	T:0.0101351
chr28	9841160	2	292	G:0.695205	A:0.304795
chr28	9842075	2	278	G:0.690647	A:0.309353
chr28	9842205	3	314	T:0.694268	TAA:0.22293
TAAA:0.0828025
chr28	9842215	3	314	TA:0.292994	T:0.401274
AA:0.305732
chr28	9842217	2	310	A:0.987097	T:0.0129032
chr28	9842220	2	308	A:0.899351	T:0.100649
chr28	9842238	3	314	A:0.178344	AT:0.515924
AAT:0.305732
chr28	9842322	2	304	C:0.664474	T:0.335526
chr28	9842442	2	302	G:0.695364	C:0.304636
chr28	9842543	2	308	A:0.694805	T:0.305195
chr28	9842745	2	298	C:0.667785	T:0.332215
chr28	9843409	2	286	T:0.695804	C:0.304196
chr28	9843430	2	286	T:0.695804	C:0.304196
chr28	9843567	2	286	T:0.695804	TAA:0.304196
chr28	9843654	2	254	G:0.88189	C:0.11811
chr28	9843795	2	12	C:0.666667	T:0.333333
chr28	9843984	2	298	T:0.657718	C:0.342282
chr28	9843998	2	302	G:0.884106	A:0.115894
chr28	9844064	2	304	G:0.983553	GAT:0.0164474
chr28	9844773	2	298	C:0.986577	T:0.0134228
chr28	9844863	3	288	CA:0.527778	C:0.142361
CAA:0.329861
chr28	9844902	2	298	C:0.694631	T:0.305369
chr28	9845152	2	310	G:0.683871	A:0.316129
chr28	9845180	2	304	A:0.707237	T:0.292763
chr28	9845491	2	296	A:0.695946	G:0.304054
chr28	9845837	2	316	TA:0.689873	T:0.310127
chr28	9845981	2	286	A:0.688811	G:0.311189
chr28	9846137	2	306	A:0.689542	G:0.310458
chr28	9846192	3	300	CAGAG:0.566667	C:0.29	CAGAGAG:
```

0.143333
chr28	9846229	2	294	G:0.697279	C:0.302721
chr28	9846345	2	274	TC:0.70073	T:0.29927
chr28	9846363	3	288	T:0.368056	TA:0.520833
TAA:0.111111
chr28	9846436	2	312	G:0.929487	A:0.0705128
chr28	9846461	2	310	C:0.703226	T:0.296774
chr28	9846494	2	302	T:0.688742	C:0.311258
chr28	9846687	2	292	C:0.688356	CCT:0.311644
chr28	9846729	2	292	AT:0.606164	A:0.393836
chr28	9846731	2	292	TTA:0.986301	T:0.0136986
chr28	9846732	2	288	TA:0.538194	T:0.461806
chr28	9847396	2	296	C:0.662162	T:0.337838
chr28	9847506	2	302	T:0.13245	C:0.86755
chr28	9847533	2	292	A:0.64726	C:0.35274
chr28	9848139	2	292	T:0.835616	C:0.164384
chr28	9848246	2	298	G:0.828859	A:0.171141
chr28	9848298	2	290	A:0.144828	G:0.855172
chr28	9849306	2	302	C:0.294702	T:0.705298
chr28	9849486	2	298	A:0.822148	AC:0.177852
chr28	9849721	2	304	A:0.322368	C:0.677632
chr28	9849820	2	292	CACA:0.328767	C:0.671233
chr28	9849928	2	300	A:0.333333	T:0.666667
chr28	9850403	2	316
CTAGTAGCACCTAGTAGGTGGGAGAGTGAAGCGGGGGCGAAG:0.14557	C:0.85443
chr28	9850627	2	312	A:0.836538	G:0.163462
chr28	9850811	2	298	AT:0.446309	A:0.553691
chr28	9850888	2	300	C:0.35	CT:0.65
chr28	9850891	2	300	A:0.35	T:0.65
chr28	9851276	2	302	A:0.162252	C:0.837748
chr28	9851497	2	298	A:0.822148	G:0.177852
chr28	9851627	2	308	G:0.457792	A:0.542208
chr28	9851629	2	306	TC:0.957516	T:0.0424837
chr28	9851788	2	292	C:0.910959	T:0.0890411
chr28	9851888	2	294	A:0.812925	T:0.187075
chr28	9852068	2	252	TGGG:0.238095	T:0.761905
chr28	9852170	2	300	ACCT:0.546667	A:0.453333
chr28	9852576	2	310	A:0.809677	G:0.190323
chr28	9852994	2	312	A:0.820513	T:0.179487
chr28	9853053	2	298	G:0.567114	A:0.432886
chr28	9853304	2	296	C:0.195946	T:0.804054
chr28	9853494	2	304	A:0.361842	G:0.638158
chr28	9853508	2	304	A:0.184211	G:0.815789
chr28	9853632	2	302	C:0.377483	CGAGG:0.622517
chr28	9853923	2	294	C:0.952381	A:0.047619
chr28	9854234	2	296	C:0.378378	T:0.621622
chr28	9854412	2	300	A:0.383333	T:0.616667
chr28	9854497	2	306	AAC:0.993464	A:0.00653595
chr28	9854514	2	308	C:0.204545	G:0.795455
chr28	9854797	2	306	G:0.333333	A:0.666667
chr28	9855172	2	302	A:0.370861	G:0.629139
chr28	9855239	3	296	C:0.135135	CT:0.239865
CTT:0.625
chr28	9855333	2	300	A:0.38	C:0.62

chr28 9855871 2 304 G:0.368421 A:0.631579
chr28 9856031 2 308 A:0.357143 AG:0.642857
chr28 9856260 2 302 G:0.34106 A:0.65894
chr28 9856378 2 296 G:0.378378 C:0.621622
chr28 9856454 2 292 CA:0.883562 C:0.116438
chr28 9856687 2 304 A:0.358553 G:0.641447
chr28 9856755 2 298 C:0.372483 T:0.627517
chr28 9856858 2 304 C:0.986842 T:0.0131579
chr28 9857134 2 306 T:0.388889 C:0.611111
chr28 9858708 2 282 C:0.914894 T:0.0851064
chr28 9859278 2 294 C:0.578231 T:0.421769
chr28 9861136 4 302 CCT:0.615894 C:0.198675
CCTCT:0.0993377 CCTCTCT:0.0860927
chr28 9861190 3 280 TA:0.421429 T:0.153571
TAA:0.425
chr28 9861661 2 300 C:0.906667 T:0.0933333
chr28 9862142 2 302 C:0.450331 T:0.549669
chr28 9862228 2 246 GGAGAGA:0.845528 G:0.154472
chr28 9862257 2 264 GAGAGA:0.753788 G:0.246212
chr28 9862263 4 300 GGC:0.596667 GCGC:0.27
G:0.0866667 GGCGC:0.0466667
chr28 9862625 2 304 T:0.976974 C:0.0230263
chr28 9862626 2 304 C:0.976974 T:0.0230263
chr28 9862661 2 302 C:0.990066 T:0.00993377
chr28 9862727 2 306 C:0.937908 G:0.0620915
chr28 9862798 2 302 G:0.917219 A:0.0827815
chr28 9863474 2 312 C:0.983974 A:0.0160256
chr28 9863582 2 290 A:0.986207 ATG:0.0137931
chr28 9863699 2 298 G:0.510067 A:0.489933
chr28 9863896 2 304 C:0.980263 T:0.0197368
chr28 9864348 2 296 G:0.986486 A:0.0135135
chr28 9864350 2 296 T:0.962838 TA:0.0371622
chr28 9864458 2 298 C:0.916107 T:0.0838926
chr28 9864476 2 304 A:0.203947 G:0.796053
chr28 9864707 2 300 T:0.976667 A:0.0233333
chr28 9864884 2 298 G:0.489933 A:0.510067
chr28 9865059 2 304 A:0.980263 G:0.0197368
chr28 9865602 2 302 C:0.976821 A:0.0231788
chr28 9865684 2 306 A:0.375817 G:0.624183
chr28 9865692 2 310 G:0.906452 T:0.0935484
chr28 9865757 2 302 C:0.970199 A:0.0298013
chr28 9865970 2 304 C:0.983553 T:0.0164474
chr28 9866030 2 314 C:0.980892 T:0.0191083
chr28 9866648 4 312 TTTTA:0.317308 T:0.0705128
TTTTATTTA:0.538462 TTTTATTTATTTA:0.0737179
chr28 9866773 2 296 C:0.989865 T:0.0101351
chr28 9867334 2 306 G:0.970588 A:0.0294118
chr28 9867590 2 300 G:0.98 A:0.02
chr28 9867754 2 292 T:0.304795 C:0.695205
chr28 9867842 2 298 C:0.892617 G:0.107383
chr28 9867917 2 294 C:0.979592 G:0.0204082
chr28 9868206 2 312 A:0.807692 T:0.192308
chr28 9868291 2 302 T:0.996689 G:0.00331126
chr28 9868294 2 302 T:0.307947 C:0.692053

chr28	9868531	2	296	A:0.550676	C:0.449324
chr28	9868569	2	300	T:0.883333	C:0.116667
chr28	9868623	2	306	A:0.555556	G:0.444444
chr28	9868641	2	306	G:0.803922	A:0.196078
chr28	9868719	2	288	G:0.597222	T:0.402778
chr28	9868812	2	296	C:0.817568	T:0.182432
chr28	9868859	2	300	A:0.973333	C:0.0266667
chr28	9868965	2	282	TG:0.425532	T:0.574468
chr28	9869492	2	262	T:0.698473	TA:0.301527
chr28	9869832	2	310	C:0.858065	T:0.141935
chr28	9869902	2	296	C:0.945946	T:0.0540541
chr28	9870157	2	318	ATTAGT:0.91195	A:0.0880503
chr28	9871663	2	304	A:0.523026	G:0.476974
chr28	9872139	2	312	T:0.439103	C:0.560897
chr28	9872724	2	308	T:0.535714	C:0.464286
chr28	9872888	2	296	T:0.439189	A:0.560811
chr28	9872930	2	290	G:0.444828	T:0.555172
chr28	9873655	2	308	G:0.918831	T:0.0811688
chr28	9874827	2	312	G:0.516026	C:0.483974
chr28	9875173	2	304	A:0.907895	G:0.0921053
chr28	9875181	2	306	G:0.888889	T:0.111111
chr28	9875347	2	294	G:0.561224	A:0.438776
chr28	9875558	2	304	T:0.578947	C:0.421053
chr28	9875957	2	304	GA:0.539474	G:0.460526
chr28	9876029	2	304	C:0.664474	T:0.335526
chr28	9876312	2	292	A:0.989726	G:0.010274
chr28	9876381	2	316	T:0.977848	TGGAGGGCCAC:0.0221519
chr28	9876550	2	290	G:0.406897	T:0.593103
chr28	9877259	2	304	T:0.565789	C:0.434211
chr28	9877592	2	312	T:0.676282	G:0.323718
chr28	9877635	2	312	G:0.503205	GA:0.496795
chr28	9877656	2	314	T:0.299363	A:0.700637
chr28	9877730	2	310	A:0.56129	G:0.43871
chr28	9878408	2	298	C:0.302013	G:0.697987
chr28	9878831	2	300	G:0.93	A:0.07
chr28	9878967	2	306	G:0.69281	A:0.30719
chr28	9879096	2	310	T:0.86129	A:0.13871
chr28	9879617	2	290	G:0.972414	T:0.0275862
chr28	9880012	2	300	C:0.456667	T:0.543333
chr28	9880112	2	288	C:0.444444	T:0.555556
chr28	9880193	2	304	G:0.842105	A:0.157895
chr28	9880387	2	284	C:0.552817	G:0.447183
chr28	9880464	2	296	CCA:0.780405	C:0.219595
chr28	9880508	2	290	G:0.451724	A:0.548276
chr28	9880510	2	290	A:0.237931	G:0.762069
chr28	9880537	2	278	A:0.478417	G:0.521583
chr28	9880570	2	284	G:0.855634	A:0.144366
chr28	9880870	2	310	T:0.248387	TC:0.751613
chr28	9880874	2	310	T:0.248387	TCTACCA:0.751613
chr28	9880964	2	302	A:0.407285	G:0.592715
chr28	9881334	2	258	C:0.581395	G:0.418605
chr28	9881668	2	298	C:0.916107	T:0.0838926
chr28	9881779	2	306	G:0.849673	T:0.150327

chr28	9881780	3	296	AT:0.530405	A:0.222973	ATT:0.246622	
chr28	9881785	2	306	T:0.849673	A:0.150327		
chr28	9882122	2	300	A:0.766667	G:0.233333		
chr28	9882387	2	306	C:0.663399	A:0.336601		
chr28	9882497	2	294	T:0.891156	C:0.108844		
chr28	9882690	2	310	T:0.919355	A:0.0806452		
chr28	9882745	2	306	G:0.75817	A:0.24183		
chr28	9882816	2	302	G:0.854305	A:0.145695		
chr28	9882890	2	302	T:0.0794702	C:0.92053		
chr28	9882931	2	302	G:0.92053	A:0.0794702		
chr28	9882979	2	304	G:0.914474	A:0.0855263		
chr28	9883080	2	294	G:0.72449	C:0.27551		
chr28	9883168	2	312	CAG:0.471154	C:0.528846		
chr28	9883170	2	312	GA:0.791667	G:0.208333		
chr28	9883353	2	286	T:0.370629	C:0.629371		
chr28	9883357	2	292	C:0.914384	T:0.0856164		
chr28	9883364	2	284	A:0.253521	G:0.746479		
chr28	9883521	2	274	C:0.39781	T:0.60219		
chr28	9883583	2	234	C:0.41453	CCG:0.58547		
chr28	9883586	2	234	A:0.41453	C:0.58547		
chr28	9883756	2	288	AT:0.274306	A:0.725694		
chr28	9883762	2	288	G:0.927083	C:0.0729167		
chr28	9884133	2	306	G:0.732026	A:0.267974		
chr28	9884153	2	306	TA:0.924837	T:0.0751634		
chr28	9884223	2	306	G:0.748366	A:0.251634		
chr28	9884383	2	308	T:0.331169	C:0.668831		
chr28	9884555	2	304	T:0.618421	G:0.381579		
chr28	9884681	2	304	A:0.638158	G:0.361842		
chr28	9884689	2	306	G:0.918301	A:0.0816993		
chr28	9884923	2	304	A:0.611842	G:0.388158		
chr28	9884992	2	300	A:0.39	T:0.61		
chr28	9885099	4	312	T:0.0480769	TA:0.166667	TAA:0.237179	TAAAA:0.548077
chr28	9885950	2	302	ATCT:0.877483	A:0.122517		
chr28	9886081	2	304	T:0.950658	C:0.0493421		
chr28	9886147	2	298	T:0.181208	C:0.818792		
chr28	9886881	2	306	A:0.98366	T:0.0163399		
chr28	9887013	2	310	A:0.864516	G:0.135484		
chr28	9887087	2	304	A:0.976974	C:0.0230263		
chr28	9887412	2	300	C:0.973333	T:0.0266667		
chr28	9887429	2	298	T:0.862416	G:0.137584		
chr28	9887864	2	302	A:0.976821	G:0.0231788		
chr28	9888421	2	286	G:0.975524	A:0.0244755		
chr28	9888500	2	300	C:0.866667	A:0.133333		
chr28	9889061	2	304	C:0.983553	A:0.0164474		
chr28	9889248	2	302	A:0.86755	G:0.13245		
chr28	9889768	2	300	TA:0.5	T:0.5		
chr28	9889985	2	296	T:0.986486	A:0.0135135		
chr28	9890004	2	304	G:0.503289	T:0.496711		
chr28	9890114	2	310	T:0.893548	G:0.106452		
chr28	9890129	2	304	T:0.891447	A:0.108553		
chr28	9890444	2	310	C:0.883871	T:0.116129		
chr28	9890455	2	310	C:0.883871	T:0.116129		

chr28	9890473	2	310	A:0.883871	AC:0.116129	
chr28	9890512	2	300	G:0.863333	A:0.136667	
chr28	9890523	2	300	G:0.863333	T:0.136667	
chr28	9890737	2	310	A:0.880645	T:0.119355	
chr28	9891212	2	290	G:0.889655	A:0.110345	
chr28	9891337	2	294	G:0.979592	T:0.0204082	
chr28	9891404	2	302	C:0.357616	T:0.642384	
chr28	9891610	2	314	C:0.875796	CT:0.124204	
chr28	9891719	2	306	C:0.875817	T:0.124183	
chr28	9891795	2	304	TG:0.858553	T:0.141447	
chr28	9892170	2	312	C:0.875	T:0.125	
chr28	9892247	2	300	A:0.89	G:0.11	
chr28	9892299	2	318	T:0.858491	C:0.141509	
chr28	9892543	2	306	A:0.859477	C:0.140523	
chr28	9892591	2	312	T:0.839744	G:0.160256	
chr28	9892766	2	310	A:0.874194	G:0.125806	
chr28	9892877	2	306	C:0.522876	A:0.477124	
chr28	9892943	2	294	C:0.87415	T:0.12585	
chr28	9893728	2	314	T:0.519108	G:0.480892	
chr28	9893742	2	314	G:0.850318	C:0.149682	
chr28	9893744	2	314	A:0.866242	G:0.133758	
chr28	9893812	2	304	A:0.983553	G:0.0164474	
chr28	9893869	2	300	C:0.96	G:0.04	
chr28	9894369	2	302	C:0.884106	CTT:0.115894	
chr28	9894394	2	300	C:0.496667	T:0.503333	
chr28	9894917	2	304	A:0.881579	G:0.118421	
chr28	9894930	2	298	C:0.979866	T:0.0201342	
chr28	9895380	2	302	T:0.940397	C:0.0596026	
chr28	9895399	2	308	G:0.980519	A:0.0194805	
chr28	9895686	2	308	C:0.980519	T:0.0194805	
chr28	9895992	2	244	CT:0.504098	C:0.495902	
chr28	9897009	2	276	C:0.315217	T:0.684783	
chr28	9897107	2	238	C:0.789916	T:0.210084	
chr28	9898734	2	242	G:0.818182	A:0.181818	
chr28	9898990	2	312	A:0.983974	T:0.0160256	
chr28	9899121	2	298	A:0.895973	AT:0.104027	
chr28	9900019	2	304	C:0.986842	T:0.0131579	
chr28	9900260	2	312	T:0.317308	C:0.682692	
chr28	9902271	2	312	C:0.961538	A:0.0384615	
chr28	9902493	2	298	GTA:0.902685	G:0.0973154	
chr28	9902507	2	298	A:0.610738	T:0.389262	
chr28	9902509	2	298	A:0.469799	T:0.530201	
chr28	9902511	2	298	A:0.469799	T:0.530201	
chr28	9902600	2	312	T:0.788462	C:0.211538	
chr28	9903002	2	296	C:0.256757	CA:0.743243	
chr28	9903712	2	314	C:0.675159	T:0.324841	
chr28	9904554	2	306	G:0.797386	A:0.202614	
chr28	9904665	2	288	CT:0.663194	C:0.336806	
chr28	9905089	2	304	C:0.677632	A:0.322368	
chr28	9905601	2	310	A:0.796774	T:0.203226	
chr28	9905625	2	312	T:0.320513	C:0.679487	
chr28	9906398	3	272	C:0.305147	CA:0.371324	CAA:0.323529
chr28	9906895	2	306	T:0.852941	A:0.147059	

chr28 9908236 2 302 C:0.996689 T:0.00331126
chr28 9908829 2 306 TAC:0.862745 T:0.137255
chr28 9909116 2 306 A:0.316993 T:0.683007
chr28 9909253 3 294 TA:0.520408 T:0.35034
TAA:0.129252
chr28 9909408 2 308 G:0.340909 A:0.659091
chr28 9910182 2 306 G:0.781046 A:0.218954
chr28 9910194 2 308 T:0.957792 C:0.0422078
chr28 9910897 2 310 C:0.7 CAA:0.3
chr28 9910907 2 310 A:0.983871 C:0.016129
chr28 9910908 2 308 C:0.50974 A:0.49026
chr28 9911166 2 310 TACAGAC:0.325806 T:0.674194
chr28 9911617 2 302 C:0.649007 A:0.350993
chr28 9912096 2 250 TA:0.608 T:0.392
chr28 9912205 2 300 C:0.8 T:0.2
chr28 9912296 2 304 G:0.973684 A:0.0263158
chr28 9912299 2 306 C:0.993464 A:0.00653595
chr28 9912740 2 308 G:0.970779 A:0.0292208
chr28 9913290 2 302 G:0.960265 A:0.0397351
chr28 9914398 2 298 G:0.946309 GA:0.0536913
chr28 9914533 2 298 C:0.765101 CT:0.234899
chr28 9914702 2 302 C:0.688742 T:0.311258
chr28 9915736 3 306 T:0.405229 TA:0.447712
TAA:0.147059
chr28 9916602 2 302 C:0.788079 T:0.211921
chr28 9916893 2 312 AGG:0.673077 A:0.326923
chr28 9916896 2 312 T:0.673077 TAG:0.326923
chr28 9916898 3 312 AAATGAT:0.0416667 A:
0.307692 ATAGTAATGAT:0.650641
chr28 9917159 2 300 G:0.31 A:0.69
chr28 9917550 2 302 G:0.794702 A:0.205298
chr28 9917873 2 312 T:0.798077 A:0.201923
chr28 9917902 2 312 C:0.464744 CCA:0.535256
chr28 9917957 2 306 C:0.477124 G:0.522876
chr28 9918034 2 308 C:0.655844 T:0.344156
chr28 9918417 2 302 A:0.642384 G:0.357616
chr28 9918510 4 320 ATGTGTGTG:0.3375 A:0.334375
ATGTGTG:0.190625 ATGTGTGTGTGTGTG:0.1375
chr28 9918860 2 310 T:0.664516 C:0.335484
chr28 9918865 2 312 T:0.679487 TA:0.320513
chr28 9920614 2 298 G:0.939597 C:0.0604027
chr28 9920691 2 296 A:0.665541 C:0.334459
chr28 9921321 2 304 A:0.664474 G:0.335526
chr28 9921393 2 312 A:0.846154 T:0.153846
chr28 9921431 2 310 T:0.990323 TA:0.00967742
chr28 9921727 2 316 A:0.85443 T:0.14557
chr28 9921736 2 316 T:0.471519 TAAAG:0.528481
chr28 9921988 2 308 G:0.665584 A:0.334416
chr28 9922247 3 316 GAGAAAGAA:0.322785 G:
0.525316 GAGAA:0.151899
chr28 9922309 2 316 GAGA:0.829114 G:0.170886
chr28 9922312 2 292 AAAAG:0.873288 A:0.126712
chr28 9922350 2 316 AAGAAAGAAAGAAAAAG:0.829114
A:0.170886

chr28 9922389 3 318 AAAAGAAAGAAAG:0.754717 A:
0.157233 AAAAGAAAG:0.0880503
chr28 9922484 2 308 A:0.711039 AAACAAC:0.288961
chr28 9922491 2 308 A:0.808442 T:0.191558
chr28 9923102 2 302 C:0.980132 T:0.0198675
chr28 9923322 2 302 A:0.672185 G:0.327815
chr28 9923729 2 298 G:0.973154 GA:0.0268456
chr28 9924429 2 310 T:0.33871 C:0.66129
chr28 9924670 2 292 G:0.671233 GA:0.328767
chr28 9925035 2 312 T:0.669872 C:0.330128
chr28 9925087 2 316 C:0.816456 T:0.183544
chr28 9925180 2 318 A:0.679245 T:0.320755
chr28 9925490 2 308 G:0.672078 C:0.327922
chr28 9925725 2 304 T:0.457237 C:0.542763
chr28 9927365 2 316 T:0.477848 C:0.522152
chr28 9927462 2 312 AAT:0.967949 A:0.0320513
chr28 9927599 2 304 C:0.8125 T:0.1875
chr28 9928055 2 308 A:0.818182 T:0.181818
chr28 9928142 2 318 A:0.996855 T:0.00314465
chr28 9928654 2 302 C:0.0761589 T:0.923841
chr28 9929062 2 296 CCTCT:0.358108 C:0.641892
chr28 9929534 2 292 CAAAAA:0.715753 C:0.284247
chr28 9929677 2 160 T:0.7125 TA:0.2875
chr28 9930040 2 298 TA:0.308725 T:0.691275
chr28 9930276 2 304 TA:0.483553 T:0.516447
chr28 9931128 2 206 TA:0.626214 T:0.373786
chr28 9932271 2 308 C:0.331169 T:0.668831
chr28 9932368 2 296 T:0.827703 C:0.172297
chr28 9932584 2 292 T:0.688356 C:0.311644
chr28 9932724 2 286 C:0.688811 G:0.311189
chr28 9932870 6 316 AATAGATAG:0.148734 A:
0.0822785 AATAG:0.0727848 AATAGATAGATAG:0.262658
AATAGATAGATAGATAG:0.25 AATAGATAGATAGATAGATAG:0.183544
chr28 9934201 2 310 G:0.977419 A:0.0225806
chr28 9934316 2 304 C:0.799342 T:0.200658
chr28 9934493 2 310 A:0.309677 G:0.690323
chr28 9934961 2 286 C:0.332168 CA:0.667832
chr28 9935090 2 308 AT:0.866883 A:0.133117
chr28 9935179 2 294 G:0.319728 A:0.680272
chr28 9935476 2 306 T:0.862745 G:0.137255
chr28 9935849 3 292 T:0.684932 TA:0.287671
TAA:0.0273973
chr28 9936028 2 314 GA:0 G:1
chr28 9936036 2 314 G:0 GA:1
chr28 9936130 2 304 G:0.651316 A:0.348684
chr28 9938943 3 304 C:0.733553 CA:0.0394737
CAA:0.226974
chr28 9939204 2 286 AAC:0.727273 A:0.272727
chr28 9939206 2 284 C:0.901408 A:0.0985915
chr28 9940272 2 298 A:0.808725 G:0.191275
chr28 9940348 2 304 TTTC:0.802632 T:0.197368
chr28 9942369 2 314 GGAGA:0.980892 G:0.0191083
chr28 9944305 2 306 T:0.676471 C:0.323529
chr28 9945252 2 292 T:0.506849 C:0.493151

chr28 9945832 2 308 TA:0.311688 T:0.688312
chr28 9947583 2 312 T:0.983974 C:0.0160256
chr28 9947736 3 310 A:0.525806 AG:0.332258
AGG:0.141935
chr28 9947747 3 314 A:0.321656 AG:0.538217
G:0.140127
chr28 9947926 2 300 C:0.986667 A:0.0133333
chr28 9947989 2 300 C:0.823333 CA:0.176667
chr28 9948544 2 300 A:0.996667 C:0.00333333
chr28 9949016 2 304 ACAAT:0.335526 A:0.664474
chr28 9949084 2 310 A:0.33871 AT:0.66129
chr28 9949465 2 306 T:0.813725 A:0.186275
chr28 9950701 2 298 T:0.97651 C:0.0234899
chr28 9951013 2 290 A:0.686207 G:0.313793
chr28 9951541 2 290 C:0.410345 CCTCT:0.589655
chr28 9952690 2 308 C:0.795455 T:0.204545
chr28 9953010 2 308 A:0.983766 T:0.0162338
chr28 9953318 2 262 CCT:0.263359 C:0.736641
chr28 9953671 2 314 C:0.869427 T:0.130573
chr28 9953923 3 306 G:0.660131 GA:0.24183
GAA:0.0980392
chr28 9954492 2 300 G:0.663333 GA:0.336667
chr28 9954881 2 304 T:0.6875 G:0.3125
chr28 9954891 2 298 AT:0.828859 A:0.171141
chr28 9954901 2 300 T:0.696667 A:0.303333
chr28 9955119 2 296 T:0.672297 TA:0.327703
chr28 9955544 2 306 G:0.816993 T:0.183007
chr28 9955881 2 308 C:0.688312 G:0.311688
chr28 9956105 2 292 C:0.962329 T:0.0376712
chr28 9956137 3 302 G:0.384106 GTCTC:0.211921
GTCTCTC:0.403974
chr28 9956173 2 294 TA:0.346939 T:0.653061
chr28 9956424 2 298 A:0.325503 G:0.674497
chr28 9956653 2 288 C:0.3125 T:0.6875
chr28 9957148 2 310 T:0.332258 C:0.667742
chr28 9957756 2 284 T:0.672535 A:0.327465
chr28 9957779 2 300 T:0.933333 TAAAA:0.0666667
chr28 9957785 2 304 T:0.865132 TAA:0.134868
chr28 9957787 2 302 A:0.665563 AAT:0.334437
chr28 9957794 3 302 TA:0.433775 AA:0.231788
T:0.334437
chr28 9957796 2 284 A:0.841549 AT:0.158451
chr28 9957802 2 302 A:0.665563 T:0.334437
chr28 9957821 2 302 T:0.665563 C:0.334437
chr28 9957952 5 308 CCTCTCTCTCTCTCTCTCT:0.25 C:
0.418831 CCTCTCTCTCT:0.207792 CCTCTCTCTCTCT:0.0746753
CCTCTCTCTCTCTCT:0.0487013
chr28 9958001 2 292 CATAA:0.623288 C:0.376712
chr28 9958025 3 308 A:0.886364 C:0.0811688
AATAC:0.0324675
chr28 9958242 2 296 CT:0.956081 C:0.0439189
chr28 9958526 2 308 A:0.321429 T:0.678571
chr28 9958909 2 310 C:0.806452 T:0.193548
chr28 9959119 2 304 T:0.332237 C:0.667763

chr28 9959432 2 298 G:0.667785 A:0.332215
chr28 9959710 2 310 C:0.983871 G:0.016129
chr28 9959963 2 308 T:0.685065 C:0.314935
chr28 9960316 2 296 TA:0.638514 T:0.361486
chr28 9960317 2 302 A:0.698675 T:0.301325
chr28 9960471 2 288 TG:0.989583 T:0.0104167
chr28 9960605 3 288 TA:0.534722 T:0.149306
TAA:0.315972
chr28 9960622 2 304 T:0.358553 TCTG:0.641447
chr28 9960700 2 304 G:0.802632 A:0.197368
chr28 9960975 2 298 A:0.926174 T:0.0738255
chr28 9961089 2 308 C:0.701299 T:0.298701
chr28 9961105 2 312 C:0.839744 T:0.160256
chr28 9961338 2 316 A:0.341772 AG:0.658228
chr28 9961482 2 314 C:0.684713 CT:0.315287
chr28 9961588 2 312 T:0.958333 TC:0.0416667
chr28 9961876 2 304 A:0.680921 G:0.319079
chr28 9962049 2 302 CAT:0.788079 C:0.211921
chr28 9962281 2 280 CCT:0.825 C:0.175
chr28 9962470 2 298 T:0.862416 C:0.137584
chr28 9962583 2 320 A:0.98125
AAGACATCACCCAGTCTACGGCCATACCACCCTGAACGCGCCCGATCTCGTCTGATCTCGGAAGCTAA
GCAGGGTCGGGCCTGGTTAGTACTTGGATGGG:0.01875
chr28 9962672 2 310 T:0.680645 C:0.319355
chr28 9963026 2 304 C:0.703947 T:0.296053
chr28 9963417 2 302 C:0.639073 T:0.360927
chr28 9963449 2 304 G:0.976974 A:0.0230263
chr28 9963862 2 314 C:0.464968 T:0.535032
chr28 9964118 2 308 C:0.672078 A:0.327922
chr28 9964363 2 302 T:0.68543 C:0.31457
chr28 9964449 2 314 C:0.678344 CCTCT:0.321656
chr28 9964485 4 296 T:0.368243 TA:0.361486
TAA:0.233108 TAAA:0.0371622
chr28 9964630 2 302 C:0.824503 CCTCT:0.175497
chr28 9964740 2 316 GA:0.243671 G:0.756329
chr28 9964838 2 306 A:0.313725 G:0.686275
chr28 9965642 2 302 G:0.642384 A:0.357616
chr28 9965676 2 300 G:0.663333 C:0.336667
chr28 9967175 2 308 A:0.967532 G:0.0324675
chr28 9968483 2 312 A:0.663462 G:0.336538
chr28 9969013 2 314 T:0.496815 C:0.503185
chr28 9969507 2 294 AAAG:0.982993 A:0.0170068
chr28 9969512 2 294 A:0.979592 C:0.0204082
chr28 9969777 3 312 AAC:0.689103 A:0.102564
AACACAC:0.208333
chr28 9969871 2 306 G:0.924837 A:0.0751634
chr28 9969879 2 302 T:0.208609 C:0.791391
chr28 9970039 2 300 CGGGAGCCCCGCGAT:0.03 C:0.97
chr28 9971287 2 210 G:0.614286 T:0.385714
chr28 9971366 2 246 C:0.703252 A:0.296748
chr28 9971397 2 228 G:0.714912 T:0.285088
chr28 9971574 2 276 C:0.612319 A:0.387681
chr28 9971681 2 276 G:0.757246 C:0.242754
chr28 9971687 2 274 A:0.755474 C:0.244526

chr28	9972155	2	294	C:0.965986	CTTT:0.0340136		
chr28	9972167	2	292	T:0.791096	TTA:0.208904		
chr28	9972180	2	290	C:0.631034	T:0.368966		
chr28	9972222	2	296	A:0.929054	T:0.0709459		
chr28	9972623	2	298	AT:0.942953	A:0.057047		
chr28	9972653	4	302	GT:0.192053	G:0.466887	GTTT:0.13245	GTTTT:0.208609
chr28	9972690	3	292	CAGAG:0.630137	C:0.287671	CAGAGAG:0.0821918	
chr28	9973458	2	312	G:0.788462	A:0.211538		
chr28	9973537	2	308	A:0.642857	AAC:0.357143		
chr28	9973915	2	308	C:0.876623	CAA:0.123377		
chr28	9974638	2	306	T:0.598039	C:0.401961		
chr28	9974833	2	304	C:0.621711	T:0.378289		
chr28	9974848	2	304	C:0.973684	T:0.0263158		
chr28	9974904	2	300	A:0.04	G:0.96		
chr28	9974936	2	300	C:0.606667	T:0.393333		
chr28	9975592	3	300	C:0.773333	CACAG:0.14	CACACAG:0.0866667	
chr28	9975627	2	262	A:0.698473	G:0.301527		
chr28	9975839	2	300	T:0.896667	C:0.103333		
chr28	9975887	2	298	T:0.610738	C:0.389262		
chr28	9975979	2	304	A:0.595395	C:0.404605		
chr28	9976067	2	312	A:0.608974	T:0.391026		
chr28	9976079	2	312	C:0.608974	T:0.391026		
chr28	9976406	2	308	T:0.613636	A:0.386364		
chr28	9976412	2	308	T:0.613636	C:0.386364		
chr28	9976549	2	290	C:0.6	T:0.4		
chr28	9976570	2	284	GTC:0.640845	G:0.359155		
chr28	9976621	2	306	T:0.686275	G:0.313725		
chr28	9976866	2	300	T:0.586667	TAG:0.413333		
chr28	9977173	2	302	G:0.956954	A:0.0430464		
chr28	9977269	2	306	G:0.578431	A:0.421569		
chr28	9977368	2	302	G:0.599338	A:0.400662		
chr28	9977531	2	308	C:0.61039	CA:0.38961		
chr28	9977551	3	310	C:0.874194	CA:0.0774194	CAA:0.0483871	
chr28	9977615	2	310	T:0.63871	C:0.36129		
chr28	9977652	2	310	G:0.980645	A:0.0193548		
chr28	9977724	2	292	G:0.589041	A:0.410959		
chr28	9977937	2	308	A:0.607143	G:0.392857		
chr28	9978248	2	306	A:0.627451	G:0.372549		
chr28	9978328	2	306	A:0.901961	G:0.0980392		
chr28	9978417	2	308	T:0.594156	C:0.405844		
chr28	9978479	2	304	A:0.980263	G:0.0197368		
chr28	9978950	2	306	T:0.611111	C:0.388889		
chr28	9979983	2	292	C:0.979452	T:0.0205479		
chr28	9980549	2	298	C:0.714765	T:0.285235		
chr28	9980829	2	288	T:0.583333	C:0.416667		
chr28	9980925	2	300	C:0.993333	T:0.00666667		
chr28	9981357	2	298	G:0.986577	A:0.0134228		
chr28	9981483	2	288	G:0.878472	A:0.121528		
chr28	9981866	2	308	A:0.980519	ATCCCTGGG:0.0194805		

chr28	9981870	2	308	C:0.980519	CGCAGCGGT:0.0194805	
chr28	9981975	2	302	T:0.596026	C:0.403974	
chr28	9982005	2	298	A:0.620805	G:0.379195	
chr28	9982050	2	302	C:0.910596	T:0.089404	
chr28	9982104	2	306	C:0.584967	T:0.415033	
chr28	9982112	2	306	G:0.584967	A:0.415033	
chr28	9982162	3	302	CCTCTCT:0.417219	C:0.433775	CCTCT:0.149007
chr28	9982193	2	290	C:0.572414	G:0.427586	
chr28	9982831	2	304	C:0.575658	G:0.424342	
chr28	9982863	2	310	C:0.606452	T:0.393548	
chr28	9983073	2	290	C:0.6	T:0.4	
chr28	9983260	2	302	G:0.549669	A:0.450331	
chr28	9983295	2	284	C:0.721831	A:0.278169	
chr28	9983396	2	304	G:0.980263	A:0.0197368	
chr28	9983522	2	308	G:0.594156	A:0.405844	
chr28	9983533	2	302	T:0.57947	C:0.42053	
chr28	9983570	2	308	C:0.574675	T:0.425325	
chr28	9984009	2	306	T:0.598039	C:0.401961	
chr28	9984025	2	314	G:0.601911	A:0.398089	
chr28	9984223	2	308	C:0.987013	G:0.012987	
chr28	9984291	2	300	C:0.573333	G:0.426667	
chr28	9984452	2	306	T:0.98366	C:0.0163399	
chr28	9984534	2	310	C:0.603226	G:0.396774	
chr28	9984633	2	302	G:0.97351	A:0.0264901	
chr28	9984635	2	302	G:0.609272	C:0.390728	
chr28	9984850	2	312	C:0.987179	T:0.0128205	
chr28	9984907	2	290	T:0.734483	TA:0.265517	
chr28	9985389	2	290	A:0.97931	G:0.0206897	
chr28	9985655	2	294	G:0.982993	A:0.0170068	
chr28	9985722	2	292	T:0.140411	C:0.859589	
chr28	9985736	2	290	A:0.6	T:0.4	
chr28	9985765	2	286	T:0.975524	A:0.0244755	
chr28	9985816	2	300	T:0.98	TA:0.02	
chr28	9986285	2	312	C:0.714744	T:0.285256	
chr28	9986551	2	304	T:0.990132	C:0.00986842	
chr28	9986841	2	298	G:0.869128	T:0.130872	
chr28	9986915	2	298	C:0.97651	G:0.0234899	
chr28	9987186	2	300	T:0.62	TAG:0.38	
chr28	9987300	2	306	A:0.986928	T:0.0130719	
chr28	9987449	2	302	C:0.97351	T:0.0264901	
chr28	9987855	2	312	C:0.971154	A:0.0288462	
chr28	9987877	2	310	T:0.590323	TA:0.409677	
chr28	9987927	3	304	AT:0.611842	A:0.128289	ATT:0.259868
chr28	9988108	2	314	A:0.984076	T:0.0159236	
chr28	9988315	2	308	T:0.993506	C:0.00649351	
chr28	9988511	2	186	C:0.446237	CA:0.553763	
chr28	9989242	2	286	T:0.986014	A:0.013986	
chr28	9990006	2	306	C:0.98366	A:0.0163399	
chr28	9990178	2	310	C:0.709677	T:0.290323	
chr28	9990183	2	308	C:0.571429	T:0.428571	
chr28	9990416	2	296	A:0.983108	G:0.0168919	

chr28	9990557	2	302	T:0.983444	A:0.0165563		
chr28	9990932	2	300	T:0.983333	C:0.0166667		
chr28	9990959	2	296	T:0.97973	C:0.0202703		
chr28	9990970	2	296	C:0.97973	T:0.0202703		
chr28	9991065	2	302	C:0.718543	T:0.281457		
chr28	9991102	2	300	T:0.706667	G:0.293333		
chr28	9991563	2	304	A:0.983553	T:0.0164474		
chr28	9991672	2	302	T:0.708609	C:0.291391		
chr28	9991885	2	288	C:0.975694	T:0.0243056		
chr28	9992325	2	258	C:0.581395	T:0.418605		
chr28	9992501	2	162	G:0.691358	A:0.308642		
chr28	9992546	2	158	AGGAGG:0.455696	A:0.544304		
chr28	9992570	2	158	G:0.455696	A:0.544304		
chr28	9992722	2	254	G:0.555118	A:0.444882		
chr28	9992731	2	254	C:0.555118	A:0.444882		
chr28	9992882	2	300	A:0.986667	G:0.0133333		
chr28	9992924	2	304	G:0.601974	T:0.398026		
chr28	9992928	2	304	A:0.601974	G:0.398026		
chr28	9992963	2	298	A:0.573826	G:0.426174		
chr28	9993055	2	286	G:0.611888	A:0.388112		
chr28	9993060	2	286	C:0.611888	T:0.388112		
chr28	9993145	2	298	C:0.577181	T:0.422819		
chr28	9993279	2	298	G:0.590604	A:0.409396		
chr28	9993341	2	292	C:0.592466	T:0.407534		
chr28	9993410	2	300	T:0.573333	C:0.426667		
chr28	9993413	2	300	T:0.573333	TG:0.426667		
chr28	9993962	2	302	C:0.576159	T:0.423841		
chr28	9994647	2	304	C:0.740132	A:0.259868		
chr28	9994812	2	298	T:0.587248	C:0.412752		
chr28	9995928	2	306	G:0.604575	T:0.395425		
chr28	9996072	2	304	G:0.753289	A:0.246711		
chr28	9996290	2	306	G:0.715686	A:0.284314		
chr28	9996574	2	310	AAGC:0.73871	A:0.26129		
chr28	9996642	2	300	T:0.596667	C:0.403333		
chr28	9997195	2	288	G:0.607639	T:0.392361		
chr28	9997477	2	272	C:0.764706	G:0.235294		
chr28	9997600	4	314	GATTTATTTATTTATTT:0.442675	G:0.226115	GATTTATTTATTTATTTATTT:0.210191	GATTTATTTATTTATTTATTTATTTATTTATTT:0.121019
chr28	9997720	2	258	C:0.883721	T:0.116279		
chr28	9997873	4	308	T:0.616883	TCACA:0.146104	TCACACA:0.201299	TCACACACA:0.0357143
chr28	9997890	2	308	C:0.902597	G:0.0974026		
chr28	9998077	2	302	C:0.768212	T:0.231788		
chr28	9998409	2	308	T:0.727273	C:0.272727		
chr28	9998552	2	296	A:0.591216	G:0.408784		
chr28	9999121	2	292	C:0.592466	T:0.407534		
chr28	10000713	2	300	G:0.986667	A:0.0133333		
chr28	10001026	2	260	TA:0.588462	T:0.411538		
chr28	10002489	2	298	C:0.604027	T:0.395973		
chr28	10002497	2	296	GCTCT:0.675676	G:0.324324		
chr28	10004928	2	306	GA:0.620915	G:0.379085		
chr28	10004934	2	306	A:0.620915	G:0.379085		
chr28	10004977	2	300	T:0.756667	C:0.243333		

chr28 10005188 2 304 C:0.615132 A:0.384868
chr28 10005207 2 304 A:0.631579 G:0.368421
chr28 10005272 2 296 A:0.621622 G:0.378378
chr28 10005547 2 302 A:0.612583 T:0.387417
chr28 10005764 2 312 A:0.733974 G:0.266026
chr28 10006211 2 310 C:0.619355 T:0.380645
chr28 10006365 2 310 T:0.609677 C:0.390323
chr28 10006642 2 304 C:0.618421 T:0.381579
chr28 10006683 2 302 G:0.721854 A:0.278146
chr28 10006760 2 306 T:0.624183 TAGCCA:0.375817
chr28 10006950 2 306 G:0.614379 A:0.385621
chr28 10007006 2 298 G:0.604027 T:0.395973
chr28 10007231 2 300 C:0.98 T:0.02
chr28 10007530 2 276 G:0.601449 A:0.398551
chr28 10007900 2 298 G:0.607383 A:0.392617
chr28 10008098 2 296 G:0.820946 A:0.179054
chr28 10008446 2 304 C:0.730263 G:0.269737
chr28 10009248 2 308 A:0.597403 C:0.402597
chr28 10009355 2 304 A:0.595395 C:0.404605
chr28 10009415 2 296 C:0.591216 T:0.408784
chr28 10009645 2 304 G:0.618421 A:0.381579
chr28 10009655 2 304 T:0.618421 C:0.381579
chr28 10010658 2 306 A:0.611111 G:0.388889
chr28 10010680 2 308 G:0.623377 T:0.376623
chr28 10010872 2 292 C:0.613014 T:0.386986
chr28 10011312 2 302 G:0.60596 C:0.39404
chr28 10011360 2 308 T:0.983766 TCATC:0.0162338
chr28 10012507 2 300 T:0.94 C:0.06
chr28 10012544 2 302 G:0.874172 A:0.125828
chr28 10013329 2 314 G:0.694268 T:0.305732
chr28 10013461 2 298 C:0.573826 G:0.426174
chr28 10015175 2 300 AT:0.576667 A:0.423333
chr28 10015379 2 290 CT:0.793103 C:0.206897
chr28 10015437 2 298 G:0.885906 A:0.114094
chr28 10015601 2 304 C:0.917763 CT:0.0822368
chr28 10015852 2 284 G:0.866197 C:0.133803
chr28 10015955 2 304 G:0.713816 A:0.286184
chr28 10015988 2 294 G:0.636054 A:0.363946
chr28 10016418 2 308 A:0.594156 G:0.405844
chr28 10016464 2 292 ACT:0.613014 A:0.386986
chr28 10016606 2 302 G:0.850993 T:0.149007
chr28 10016758 2 314 A:0.729299 G:0.270701
chr28 10016842 2 298 T:0.755034 C:0.244966
chr28 10017100 2 302 A:0.903974 G:0.0960265
chr28 10018096 2 306 A:0.980392 G:0.0196078
chr28 10018353 2 306 G:0.764706 T:0.235294
chr28 10020076 2 300 A:0.616667 C:0.383333
chr28 10020334 6 316 ATTATTTATTTATTTATTTATT:0.357595
A:0.158228 ATTATTTATTTATTTATTTATTTATT:0.107595
ATATTTATTTATTTATTTATTTATTTATT:0.199367
ATTATTTATTTATTTATTTATTTATTTATT:0.123418
ATATTTATTTATTTATTTATTTATTTATTTATT:0.0537975
chr28 10020378 2 312 T:0.849359 C:0.150641
chr28 10020673 2 308 T:0.600649 G:0.399351

chr28	10020828	2	294	T:0.605442	C:0.394558	
chr28	10021397	2	306	C:0.728758	T:0.271242	
chr28	10022282	2	302	A:0.629139	G:0.370861	
chr28	10023320	2	298	G:0.610738	A:0.389262	
chr28	10024839	2	308	T:0.600649	C:0.399351	
chr28	10024912	2	314	C:0.605096	T:0.394904	
chr28	10025008	2	306	C:0.931373	T:0.0686275	
chr28	10025114	2	304	A:0.578947	G:0.421053	
chr28	10025601	2	302	T:0.589404	C:0.410596	
chr28	10026279	2	304	G:0.960526	A:0.0394737	
chr28	10026946	2	308	G:0.603896	C:0.396104	
chr28	10027645	2	310	C:0.906452	T:0.0935484	
chr28	10027876	2	300	T:0.636667	C:0.363333	
chr28	10028796	2	310	CCTTCCA:0.635484	C:0.364516	
chr28	10028803	2	310	T:0.635484	TA:0.364516	
chr28	10029083	2	298	G:0.848993	A:0.151007	
chr28	10029354	2	304	G:0.763158	A:0.236842	
chr28	10029920	2	314	G:0.767516	A:0.232484	
chr28	10029933	2	306	GGA:0.944444	G:0.0555556	
chr28	10030068	2	296	G:0.628378	A:0.371622	
chr28	10030773	2	278	T:0.618705	C:0.381295	
chr28	10030790	2	260	C:0.796154	CCT:0.203846	
chr28	10030838	2	274	T:0.718978	TAA:0.281022	
chr28	10030894	2	292	T:0.866438	A:0.133562	
chr28	10031113	2	306	C:0.846405	T:0.153595	
chr28	10031128	2	300	G:0.943333	A:0.0566667	
chr28	10031274	2	300	G:0.616667	A:0.383333	
chr28	10031469	2	306	CATT:0.954248	C:0.0457516	
chr28	10031581	2	302	CA:0.854305	C:0.145695	
chr28	10032164	2	304	C:0.789474	CA:0.210526	
chr28	10032818	2	300	C:0.62	T:0.38	
chr28	10032927	2	288	C:0.979167	A:0.0208333	
chr28	10033520	2	306	C:0.75817	T:0.24183	
chr28	10034022	2	288	A:0.833333	T:0.166667	
chr28	10034024	2	290	A:0.57931	G:0.42069	
chr28	10034333	2	302	G:0.980132	A:0.0198675	
chr28	10034440	2	292	CCT:0.84589	C:0.15411	
chr28	10034758	2	296	A:0.597973	T:0.402027	
chr28	10035134	2	302	CTCTT:0.97351	C:0.0264901	
chr28	10035171	2	300	A:0.983333	G:0.0166667	
chr28	10035173	2	298	TA:0.956376	T:0.0436242	
chr28	10035182	2	302	A:0.480132	T:0.519868	
chr28	10035532	2	304	G:0.855263	A:0.144737	
chr28	10035735	2	286	G:0.86014	A:0.13986	
chr28	10035823	2	308	AAAAAAT:0.616883	A:0.383117	
chr28	10036775	2	308	T:0.970779	C:0.0292208	
chr28	10037636	2	298	GC:0.986577	G:0.0134228	
chr28	10037685	2	308	T:0.63961	C:0.36039	
chr28	10039396	3	306	TTCTCTC:0.712418	T:0.0588235	TTCTCTCTC:0.228758
chr28	10040013	2	300	C:0.986667	G:0.0133333	
chr28	10040175	2	308	A:0.577922	T:0.422078	
chr28	10040882	2	312	A:0.971154	C:0.0288462	
chr28	10041010	2	310	A:0.00645161	G:0.993548	

chr28	10041310	2	318	G:0.981132	A:0.0188679	
chr28	10042479	2	304	G:0.986842	A:0.0131579	
chr28	10042569	2	308	G:0.983766	A:0.0162338	
chr28	10042632	2	306	C:0.98366	T:0.0163399	
chr28	10042747	2	308	C:0.477273	G:0.522727	
chr28	10043976	2	294	A:0.455782	G:0.544218	
chr28	10044855	2	294	A:0.833333	T:0.166667	
chr28	10044930	2	304	A:0.855263	T:0.144737	
chr28	10044931	2	304	A:0.855263	T:0.144737	
chr28	10044951	2	306	C:0.673203	T:0.326797	
chr28	10045052	2	292	T:0.832192	G:0.167808	
chr28	10045135	2	304	T:0.838816	A:0.161184	
chr28	10045151	2	304	C:0.838816	T:0.161184	
chr28	10045236	2	304	GTAAA:0.842105	G:0.157895	
chr28	10045498	2	302	C:0.824503	T:0.175497	
chr28	10045566	2	306	T:0.820261	A:0.179739	
chr28	10045661	2	308	T:0.837662	C:0.162338	
chr28	10045823	2	314	A:0.840764	G:0.159236	
chr28	10046105	2	300	C:0.91	T:0.09	
chr28	10046511	2	292	C:0.811644	CAA:0.188356	
chr28	10046531	2	286	A:0.807692	G:0.192308	
chr28	10046533	2	286	AC:0.503497	A:0.496503	
chr28	10046770	2	306	C:0.686275	T:0.313725	
chr28	10046871	2	298	T:0.828859	C:0.171141	
chr28	10046949	2	302	C:0.817881	T:0.182119	
chr28	10047007	2	306	C:0.830065	T:0.169935	
chr28	10047016	2	304	GAA:0.832237	G:0.167763	
chr28	10047087	2	304	T:0.855263	TG:0.144737	
chr28	10047088	2	292	G:0.900685	GT:0.0993151	
chr28	10047278	2	310	T:0.483871	C:0.516129	
chr28	10047881	2	300	G:0.98	A:0.02	
chr28	10049508	2	304	G:0.845395	T:0.154605	
chr28	10049599	2	258	CTT:0.802326	C:0.197674	
chr28	10050396	2	308	T:0.850649	C:0.149351	
chr28	10051303	2	302	G:0.956954	A:0.0430464	
chr28	10052597	2	282	G:0.585106	T:0.414894	
chr28	10052665	2	290	T:0.613793	G:0.386207	
chr28	10054158	2	290	T:0.755172	TA:0.244828	
chr28	10054168	2	290	C:0.755172	A:0.244828	
chr28	10054326	2	298	A:0.436242	T:0.563758	
chr28	10054423	2	302	C:0.748344	T:0.251656	
chr28	10054475	2	302	A:0.721854	C:0.278146	
chr28	10055778	2	306	A:0.415033	G:0.584967	
chr28	10057013	2	312	G:0.974359	A:0.025641	
chr28	10057327	2	302	C:0.298013	CTG:0.701987	
chr28	10057799	2	312	T:0.817308	G:0.182692	
chr28	10058016	2	294	T:0.595238	C:0.404762	
chr28	10058393	2	286	G:0.835664	A:0.164336	
chr28	10058485	2	300	A:0.59	T:0.41	
chr28	10059372	3	290	C:0.248276	CA:0.527586	CAA:0.224138
chr28	10060023	2	304	G:0.838816	T:0.161184	
chr28	10060123	2	302	T:0.589404	C:0.410596	
chr28	10060126	2	298	AT:0.825503	A:0.174497	

chr28	10060315	2	296	AG:0.834459	A:0.165541	
chr28	10060710	2	302	A:0.231788	G:0.768212	
chr28	10061091	2	294	G:0.595238	A:0.404762	
chr28	10061284	3	294	TA:0.578231	T:0.391156	TAAA:0.0306122
chr28	10061832	2	318	T:0.575472	TA:0.424528	
chr28	10061836	2	298	AAATTAATT:0.842282	A:0.157718	
chr28	10061840	2	314	T:0.83121	A:0.16879	
chr28	10061883	2	306	C:0.931373	T:0.0686275	
chr28	10062552	2	298	T:0.818792	C:0.181208	
chr28	10062726	2	300	GT:0.573333	G:0.426667	
chr28	10062863	2	314	T:0.00636943	C:0.993631	
chr28	10063276	2	318	A:0.836478	AAT:0.163522	
chr28	10063583	2	304	T:0.740132	C:0.259868	
chr28	10064160	2	300	GT:0.776667	G:0.223333	
chr28	10064204	2	306	C:0.493464	CAGAGAG:0.506536	
chr28	10064491	2	310	A:0.409677	G:0.590323	
chr28	10064587	2	302	T:0.804636	TA:0.195364	
chr28	10064593	2	300	AT:0.46	A:0.54	
chr28	10064594	2	302	T:0.804636	A:0.195364	
chr28	10065475	2	284	C:0.556338	A:0.443662	
chr28	10065578	2	296	A:0.442568	G:0.557432	
chr28	10065761	2	288	G:0.972222	A:0.0277778	
chr28	10065988	2	294	C:0.42517	T:0.57483	
chr28	10066201	2	276	TA:0.565217	T:0.434783	
chr28	10066680	2	300	GT:0.84	G:0.16	
chr28	10066743	2	306	A:0.598039	G:0.401961	
chr28	10066979	2	298	C:0.818792	T:0.181208	
chr28	10067114	2	292	C:0.565068	T:0.434932	
chr28	10067307	2	304	G:0.993421	A:0.00657895	
chr28	10067941	2	298	T:0.818792	G:0.181208	
chr28	10067961	2	298	T:0.818792	A:0.181208	
chr28	10068042	2	306	A:0.575163	G:0.424837	
chr28	10068636	2	290	T:0.241379	C:0.758621	
chr28	10069175	2	270	G:0.72963	GA:0.27037	
chr28	10069892	2	282	T:0.489362	C:0.510638	
chr28	10070151	2	296	C:0.962838	T:0.0371622	
chr28	10070483	3	302	CAAAA:0.86755	C:0.0529801	CAAAAA:0.0794702
chr28	10070487	2	300	A:0.833333	C:0.166667	
chr28	10070720	2	300	G:0.796667	GT:0.203333	
chr28	10070728	2	300	C:0.796667	CT:0.203333	
chr28	10070746	2	308	A:0.970779	T:0.0292208	
chr28	10070777	2	302	C:0.854305	CAG:0.145695	
chr28	10070843	2	306	G:0.53268	A:0.46732	
chr28	10070878	2	314	GGCTAAAGGCGGC:0.789809	G:0.210191	
chr28	10070947	2	292	G:0.794521	A:0.205479	
chr28	10071041	2	304	G:0.230263	A:0.769737	
chr28	10071202	2	306	G:0.803922	A:0.196078	
chr28	10071317	2	306	GA:0.781046	G:0.218954	
chr28	10071810	2	310	A:0.796774	T:0.203226	
chr28	10072480	2	308	G:0.785714	A:0.214286	

chr28 10072523 2 306 AT:0.80719 A:0.19281
chr28 10072534 2 292 AT:0.972603 A:0.0273973
chr28 10072535 2 306 T:0.80719 A:0.19281
chr28 10072625 2 288 C:0.791667 G:0.208333
chr28 10072630 2 288 C:0.791667 T:0.208333
chr28 10072761 2 306 A:0.46732 C:0.53268
chr28 10072909 2 312 A:0.894231 ATGGG:0.105769
chr28 10072913 3 314 GTGGA:0.535032 G:0.43949
GTGGATGGATGGA:0.0254777
chr28 10072942 3 318 T:0.77673 TGGAAGGAA:
0.0691824 TGGATGGATGGAAGGAA:0.154088
chr28 10072989 2 312 C:0.807692 T:0.192308
chr28 10073256 2 302 C:0.768212 A:0.231788
chr28 10073257 2 302 T:0.768212 TA:0.231788
chr28 10073634 2 220 G:0.781818 A:0.218182
chr28 10073742 2 258 G:0.771318 C:0.228682
chr28 10074158 2 316 C:0.806962 G:0.193038
chr28 10074162 2 318 G:0.981132 A:0.0188679
chr28 10074844 2 300 T:0.826667 C:0.173333
chr28 10075230 2 292 C:0.825342 T:0.174658
chr28 10075314 3 278 CTT:0.327338 C:0.118705
CT:0.553957
chr28 10075364 2 208 G:0.6875 GT:0.3125
chr28 10075629 2 306 C:0.826797 A:0.173203
chr28 10075750 2 304 G:0.822368 A:0.177632
chr28 10076043 2 314 C:0.802548 T:0.197452
chr28 10076045 2 314 T:0.802548 A:0.197452
chr28 10076129 2 314 T:0.980892 C:0.0191083
chr28 10076485 2 312 T:0.971154 C:0.0288462
chr28 10076686 2 306 G:0.813725 A:0.186275
chr28 10076703 2 308 G:0.448052 A:0.551948
chr28 10076850 2 296 C:0.800676 T:0.199324
chr28 10076853 2 296 T:0.800676 C:0.199324
chr28 10076907 2 306 C:0.79085 T:0.20915
chr28 10077169 2 314 C:0.802548 T:0.197452
chr28 10077236 2 306 A:0.970588 AT:0.0294118
chr28 10077237 2 306 T:0.826797 TA:0.173203
chr28 10077324 2 296 TG:0.814189 T:0.185811
chr28 10078138 2 300 C:0.813333 T:0.186667
chr28 10078199 2 290 G:0.824138 A:0.175862
chr28 10078207 3 308 TAAAAAATAAATAAATA:0.909091
T:0.0551948 TAATAAATA:0.0357143
chr28 10078210 5 310 AAAATAAATAAATAAAT:0.477419
A:0.0580645 AAAATAAAT:0.290323 AAAATAAATAAAT:0.125806
TAAATAAATAAATAAAT:0.0483871
chr28 10078261 2 304 AT:0.986842 A:0.0131579
chr28 10078263 2 304 AAAT:0.986842 A:0.0131579
chr28 10078526 2 296 C:0.983108 T:0.0168919
chr28 10079795 2 312 T:0.785256 C:0.214744
chr28 10080015 2 302 CTG:0.976821 C:0.0231788
chr28 10080029 2 302 A:0.496689 G:0.503311
chr28 10080379 2 306 TA:0.803922 T:0.196078
chr28 10080459 2 296 CCGA:0.804054 C:0.195946
chr28 10080475 2 294 C:0.846939 T:0.153061

chr28	10080691	2	310	C:0.987097	T:0.0129032			
chr28	10080746	2	292	G:0.811644	A:0.188356			
chr28	10080917	3	300	GCA:0.66	G:0.0633333	GCACA:0.276667		
chr28	10081526	2	312	G:0.24359	A:0.75641			
chr28	10081739	2	310	C:0.274194	CTTCTGAA:0.725806			
chr28	10081788	2	306	A:0.277778	G:0.722222			
chr28	10082157	2	316	CAGAGAGAGAG:0.303797	C:0.696203			
chr28	10082780	3	302	CAAATAAATAAATAAATAAATAAAT:0.112583	C:0.486755	CAAATAAAT:0.400662		
chr28	10082898	2	178	A:0.370787	AGGAG:0.629213			
chr28	10084653	2	302	C:0.970199	A:0.0298013			
chr28	10084683	2	306	C:0.25817	G:0.74183			
chr28	10084892	2	310	G:0.251613	T:0.748387			
chr28	10085050	2	316	T:0.275316	C:0.724684			
chr28	10085104	2	306	G:0.27451	A:0.72549			
chr28	10085310	2	236	CT:0.788136	C:0.211864			
chr28	10085343	2	276	C:0.485507	CT:0.514493			
chr28	10086152	2	296	T:0.304054	C:0.695946			
chr28	10086243	2	300	C:0.286667	T:0.713333			
chr28	10086541	2	294	C:0.979592	A:0.0204082			
chr28	10086599	2	294	C:0.401361	CCT:0.598639			
chr28	10086657	2	300	T:0.306667	TA:0.693333			
chr28	10086958	2	318	C:0.279874	G:0.720126			
chr28	10087380	2	250	CTT:0.872	C:0.128			
chr28	10087430	2	276	C:0.844203	CAG:0.155797			
chr28	10089009	2	308	GCT:0.266234	G:0.733766			
chr28	10089396	3	304	CCTCTCTCT:0.286184	C:0.328947	CCT:0.384868		
chr28	10089464	2	292	A:0.284247	G:0.715753			
chr28	10089623	2	192	TA:0.828125	T:0.171875			
chr28	10089847	3	288	GTCTC:0.309028	G:0.118056	GTC:0.572917		
chr28	10089900	2	304	A:0.309211	AAAAAG:0.690789			
chr28	10089994	2	296	T:0.263514	C:0.736486			
chr28	10090186	2	286	G:0.248252	T:0.751748			
chr28	10090333	2	258	G:0.468992	A:0.531008			
chr28	10090414	2	244	CCT:0.348361	C:0.651639			
chr28	10090458	3	300	A:0.896667	AAAAT:0.05	AAAATAAAT:0.0533333		
chr28	10090502	5	312	T:0.403846	TAAATA:0.221154	TAAATAAATA:0.13141	TAAATAAATAAATA:0.0737179	TAAATAAATAAATAAAATA:0.169872
chr28	10091191	2	264	AAAAAAG:0.784091	A:0.215909			
chr28	10091192	2	260	AAAAAG:0.523077	A:0.476923			
chr28	10091349	2	268	C:0.261194	A:0.738806			
chr28	10091547	2	268	T:0.320896	TCC:0.679104			
chr28	10091937	2	270	TTC:0.22963	T:0.77037			
chr28	10092104	2	290	C:0.244828	CA:0.755172			
chr28	10092126	2	304	T:0.289474	TAAAATA:0.710526			
chr28	10092158	2	250	T:0.952	TA:0.048			
chr28	10092160	2	294	A:0.877551	AAAATAAAAT:0.122449			

chr28	10092165	2	250	A:0.952	AAAT:0.048
chr28	10092348	2	258	G:0.790698	A:0.209302
chr28	10092577	2	304	A:0.279605	ATAAAT:0.720395
chr28	10092836	2	298	T:0.285235	A:0.714765
chr28	10092850	2	298	C:0.285235	G:0.714765
chr28	10093038	2	296	GT:0.266892	G:0.733108
chr28	10093112	2	302	A:0.278146	AT:0.721854
chr28	10093364	2	306	A:0.254902	C:0.745098
chr28	10093366	2	306	C:0.254902	T:0.745098
chr28	10093569	2	310	G:0.287097	C:0.712903
chr28	10093578	2	310	G:0.287097	A:0.712903
chr28	10093621	2	312	G:0.266026	A:0.733974
chr28	10093670	2	316	T:0.25	C:0.75
chr28	10093679	2	316	G:0.974684	A:0.0253165
chr28	10093886	2	304	C:0.766447	CGCATT:0.233553
chr28	10093997	2	304	G:0.467105	A:0.532895
chr28	10094136	2	308	G:0.438312	A:0.561688
chr28	10094540	2	304	C:0.871711	G:0.128289
chr28	10094866	2	292	G:0.825342	GA:0.174658
chr28	10095524	2	102	C:0.509804	G:0.490196
chr28	10095808	2	260	T:0.8	A:0.2
chr28	10095961	2	302	C:0.794702	A:0.205298
chr28	10096511	5	316	AAC:0.553797	A:0.0348101
AACAC:0.129747		AACACAC:0.189873		AACACACAC:0.0917722	
chr28	10096535	2	304	C:0.792763	CAT:0.207237
chr28	10096621	2	300	A:0.79	G:0.21
chr28	10097262	2	300	CA:0.926667	C:0.0733333
chr28	10098620	2	296	G:0.810811	A:0.189189
chr28	10098707	2	298	T:0.815436	C:0.184564
chr28	10099037	2	302	G:0.81457	C:0.18543
chr28	10099202	2	306	C:0.862745	T:0.137255
chr28	10099239	2	308	T:0.801948	TC:0.198052
chr28	10099738	2	304	G:0.8125	T:0.1875
chr28	10100126	2	294	C:0.982993	T:0.0170068
chr28	10100782	2	300	T:0.963333	C:0.0366667
chr28	10100882	2	306	C:0.395425	T:0.604575
chr28	10101917	2	312	T:0.814103	TA:0.185897
chr28	10102255	2	300	C:0.806667	T:0.193333
chr28	10102743	2	312	T:0.798077	A:0.201923
chr28	10103171	2	292	G:0.982877	A:0.0171233
chr28	10103202	2	288	TA:0.784722	T:0.215278
chr28	10103505	2	306	T:0.803922	C:0.196078
chr28	10103578	2	300	T:0.82	A:0.18
chr28	10103655	2	296	G:0.804054	A:0.195946
chr28	10103847	2	302	T:0.937086	C:0.0629139
chr28	10104001	2	294	C:0.816327	A:0.183673
chr28	10104218	2	308	T:0.811688	C:0.188312
chr28	10105506	2	290	A:0	T:1
chr28	10105512	2	290	G:0	T:1
chr28	10105531	2	304	C:0.976974	T:0.0230263
chr28	10105576	2	304	A:0.838816	AT:0.161184
chr28	10105579	2	304	A:0.838816	T:0.161184
chr28	10106044	2	304	T:0.815789	A:0.184211
chr28	10106203	2	314	ACACCCTGGGC:0.754777	A:

0.245223
chr28	10106407	2	308	T:0.801948	C:0.198052
chr28	10106817	2	300	T:0.803333	C:0.196667
chr28	10107030	2	298	T:0.805369	C:0.194631
chr28	10107056	2	296	G:0.807432	GT:0.192568
chr28	10107095	2	302	A:0.81457	G:0.18543
chr28	10107288	2	302	C:0.804636	A:0.195364
chr28	10107430	2	310	G:0.803226	A:0.196774
chr28	10107531	2	310	T:0.8	A:0.2
chr28	10107690	2	312	G:0.948718	A:0.0512821
chr28	10107722	2	312	G:0.820513	A:0.179487
chr28	10108281	2	284	A:0.78169	C:0.21831
chr28	10108956	2	304	A:0.835526	T:0.164474
chr28	10109651	2	312	T:0.820513	A:0.179487
chr28	10109700	2	314	C:0.968153	G:0.0318471
chr28	10110817	2	282	C:0.70922	CT:0.29078
chr28	10111283	2	278	G:0.852518	T:0.147482
chr28	10111747	2	306	C:0.826797	CT:0.173203
chr28	10112779	2	308	T:0.792208	A:0.207792
chr28	10112970	2	294	T:0.778912	C:0.221088
chr28	10113257	2	300	A:0.486667	G:0.513333
chr28	10113277	2	306	G:0.810458	A:0.189542
chr28	10113438	2	310	T:0.806452	G:0.193548
chr28	10113915	2	300	A:0.806667	G:0.193333
chr28	10113965	2	302	T:0.791391	A:0.208609
chr28	10115385	3	300	TAAAA:0.693333	T:0.07	TAAA:
0.236667
chr28	10115597	2	282	G:0.93617	A:0.0638298
chr28	10115897	2	284	C:0.954225	CGTT:0.0457746
chr28	10116159	2	284	CAG:0.859155	C:0.140845
chr28	10116680	2	246	G:0.906504	GA:0.0934959
chr28	10117651	2	304	C:0.822368	T:0.177632
chr28	10117766	2	292	C:0.818493	CT:0.181507
chr28	10119515	2	302	T:0.993377	C:0.00662252
chr28	10119785	2	308	G:0.821429	A:0.178571
chr28	10120019	2	292	C:0.976027	T:0.0239726
chr28	10120177	2	300	A:0.83	G:0.17
chr28	10120315	2	302	G:0.817881	A:0.182119
chr28	10120745	2	298	G:0.959732	A:0.0402685
chr28	10121284	2	312	T:0.826923	TTTAC:0.173077
chr28	10121586	2	308	C:0.811688	T:0.188312
chr28	10122715	2	256	C:0.488281	CT:0.511719
chr28	10122759	2	286	G:0.77972	C:0.22028
chr28	10122974	2	316	ATCATGATCCCAGGG:0.778481	A:
0.221519
chr28	10123077	2	304	T:0.792763	C:0.207237
chr28	10124051	2	306	A:0.810458	C:0.189542
chr28	10124215	2	306	C:0.800654	T:0.199346
chr28	10124244	2	294	A:0.513605	AT:0.486395
chr28	10124862	2	302	ATT:0.513245	A:0.486755
chr28	10124867	3	302	T:0.304636	TAAA:0.486755
TA:0.208609
chr28	10124999	4	320	G:0.46875	GTCTC:0.115625
GTCTCTC:0.253125 GTCTCTCTCTCTCTC:0.1625

chr28	10125084	2	300	A:0.816667	T:0.183333
chr28	10125493	2	296	C:0.827703	T:0.172297
chr28	10125613	2	294	G:0.513605	A:0.486395
chr28	10125652	2	266	A:0.879699	AT:0.120301
chr28	10125921	2	294	C:0.969388	G:0.0306122
chr28	10125998	2	312	C:0.983974	T:0.0160256
chr28	10126072	2	308	AC:0.814935	A:0.185065
chr28	10126127	2	304	T:0.809211	A:0.190789
chr28	10126138	2	300	T:0.523333	C:0.476667
chr28	10126617	2	300	T:0.536667	C:0.463333
chr28	10126622	2	298	T:0.963087	C:0.0369128
chr28	10126737	2	294	A:0.326531	G:0.673469
chr28	10127431	2	296	T:0.847973	TA:0.152027
chr28	10127853	2	302	G:0.970199	A:0.0298013
chr28	10127978	2	302	C:0.516556	T:0.483444
chr28	10128193	3	296	T:0.310811	TA:0.655405
TAA:0.0337838					
chr28	10128339	2	298	A:0.808725	G:0.191275
chr28	10128587	2	300	A:0.27 G:0.73	
chr28	10128663	2	290	G:0.796552	A:0.203448
chr28	10128703	2	274	A:0.354015	G:0.645985
chr28	10128796	2	306	A:0.964052	G:0.0359477
chr28	10128797	2	306	A:0.787582	G:0.212418
chr28	10129048	2	306	C:0.539216	T:0.460784
chr28	10129091	2	306	T:0.330065	A:0.669935
chr28	10129195	2	306	ATCT:0.977124	A:0.0228758
chr28	10129258	2	306	AG:0.977124	A:0.0228758
chr28	10129397	2	306	T:0.519608	G:0.480392
chr28	10129433	2	298	A:0.305369	G:0.694631
chr28	10129449	2	302	G:0.791391	A:0.208609
chr28	10129496	2	308	T:0.00649351	G:0.993506
chr28	10129566	2	300	T:0.516667	A:0.483333
chr28	10129780	4	316	A:0.316456	AGTTGTTGTT:
0.224684	AGTTGTTGTTGTT:0.246835			AGTTGTTGTTGTTGTT:0.212025	
chr28	10130205	2	288	C:0.982639	T:0.0173611
chr28	10130209	2	284	T:0.341549	C:0.658451
chr28	10130249	2	254	C:0.712598	CCT:0.287402
chr28	10130303	2	280	TA:0.789286	T:0.210714
chr28	10130475	2	292	G:0.972603	A:0.0273973
chr28	10130573	2	280	G:0.553571	A:0.446429
chr28	10130657	2	216	C:0.805556	CCT:0.194444
chr28	10130720	2	268	TAA:0.5 T:0.5	
chr28	10130743	2	290	C:0.796552	T:0.203448
chr28	10130774	2	302	A:0.00662252	G:0.993377
chr28	10130874	2	242	G:0.132231	C:0.867769
chr28	10131021	2	248	T:0 TA:1	
chr28	10131487	2	266	G:0.973684	T:0.0263158
chr28	10131492	2	276	T:0.32971	G:0.67029
chr28	10131505	2	276	T:0.32971	C:0.67029
chr28	10131651	2	258	G:0.55814	GC:0.44186
chr28	10131678	2	280	C:0.985714	T:0.0142857
chr28	10131857	2	282	TG:0.546099	T:0.453901
chr28	10132020	2	298	G:0.510067	GT:0.489933
chr28	10132022	3	298	TC:0.791946	T:0.0167785

TCC:0.191275
chr28 10132439 2 300 T:0.83 C:0.17
chr28 10132652 3 292 CT:0.332192 C:0.202055
CTT:0.465753
chr28 10132900 2 292 C:0.979452 T:0.0205479
chr28 10133410 2 296 T:0.787162 TC:0.212838
chr28 10133788 2 284 G:0.841549 GAA:0.158451
chr28 10133978 2 198 T:0.919192 A:0.0808081
chr28 10134071 2 192 C:0.651042 T:0.348958
chr28 10134365 2 198 A:0.646465 G:0.353535
chr28 10134481 2 196 G:0.617347 A:0.382653
chr28 10134640 2 204 AT:0.647059 A:0.352941
chr28 10134643 2 204 AAT:0.647059 A:0.352941
chr28 10134645 2 196 TA:0.561224 T:0.438776
chr28 10134653 2 204 A:0.647059 T:0.352941
chr28 10134828 2 182 C:0.648352 T:0.351648
chr28 10136462 2 302 C:0.97351 T:0.0264901
chr28 10136854 2 300 T:0.983333 C:0.0166667
chr28 10137070 2 318 GAAAAAGAAAACCAAAAAAAAAAAAAAAAAA:
0.361635 G:0.638365
chr28 10137137 2 268 G:0.820896 A:0.179104
chr28 10137644 2 294 G:0.826531 A:0.173469
chr28 10137692 2 290 C:0.486207 T:0.513793
chr28 10137697 2 290 T:0.52069 A:0.47931
chr28 10137774 2 290 C:0.62069 CCTCT:0.37931
chr28 10137795 2 286 C:0.821678 CTG:0.178322
chr28 10138143 2 302 A:0.331126 G:0.668874
chr28 10138144 2 302 T:0.331126 A:0.668874
chr28 10138331 2 290 T:0.837931 C:0.162069
chr28 10138822 2 278 C:0.535971 G:0.464029
chr28 10138986 2 296 G:0.290541 A:0.709459
chr28 10139094 2 296 T:0.817568 A:0.182432
chr28 10139240 2 306 T:0.986928 C:0.0130719
chr28 10139597 2 296 A:0.800676 C:0.199324
chr28 10139797 2 310 G:0.958065 A:0.0419355
chr28 10140077 2 316 C:0 G:1
chr28 10140097 2 316 AG:0 A:1
chr28 10140385 3 296 TA:0.287162 T:0.523649
TAA:0.189189
chr28 10140474 2 288 G:0.96875 A:0.03125
chr28 10140533 2 274 ATC:0.321168 A:0.678832
chr28 10140599 2 310 T:0.132258 A:0.867742
chr28 10140961 2 304 C:0.490132 T:0.509868
chr28 10141158 2 300 G:0.986667 T:0.0133333
chr28 10141522 2 312 G:0.977564 A:0.0224359
chr28 10141979 2 298 G:0.298658 A:0.701342
chr28 10142270 2 302 G:0.966887 A:0.0331126
chr28 10142335 2 298 T:0.97651 C:0.0234899
chr28 10142667 2 296 G:0.908784 A:0.0912162
chr28 10142776 2 284 CCT:0.785211 C:0.214789
chr28 10142881 2 304 T:0.970395 G:0.0296053
chr28 10143149 2 306 T:0.529412 A:0.470588
chr28 10143281 2 290 G:0.968966 A:0.0310345
chr28 10143371 2 294 C:0.97619 T:0.0238095

chr28 10143432 2 310 A:0.964516 T:0.0354839
chr28 10143856 2 306 A:0.761438 G:0.238562
chr28 10144051 2 306 G:0.977124 A:0.0228758
chr28 10144301 2 306 T:0.513072 C:0.486928
chr28 10144486 2 288 A:0.975694 G:0.0243056
chr28 10144544 2 270 CCT:0.511111 C:0.488889
chr28 10144548 2 320 T:0.81875
TCTCTCTCTCTCTCTCTCTGCTTCTCCCTCTGCCTGTGTCTCTGC:0.18125
chr28 10144605 2 300 TA:0.95 T:0.05
chr28 10144851 2 308 G:0.977273 A:0.0227273
chr28 10145459 2 304 C:0.253289 CTA:0.746711
chr28 10145579 2 306 T:0.754902 G:0.245098
chr28 10145854 2 304 T:0.220395 C:0.779605
chr28 10145985 2 304 G:0.980263 T:0.0197368
chr28 10145992 2 304 T:0.980263 C:0.0197368
chr28 10146039 2 306 GGTGT:0.80719 G:0.19281
chr28 10146058 2 314 G:0.576433 GTA:0.423567
chr28 10146103 2 306 T:0.784314 C:0.215686
chr28 10146246 2 310 A:0.641935 G:0.358065
chr28 10146279 2 312 T:0.660256 C:0.339744
chr28 10147010 2 312 G:0.666667 C:0.333333
chr28 10147135 2 298 G:0.288591 C:0.711409
chr28 10147191 2 304 T:0.667763 G:0.332237
chr28 10147472 2 302 A:0.695364 G:0.304636
chr28 10147807 2 304 T:0.269737 TC:0.730263
chr28 10148232 2 298 GA:0.751678 G:0.248322
chr28 10148242 2 298 AGAG:0.751678 A:0.248322
chr28 10148245 3 316 GAAGA:0.841772 G:0.0917722
GAAGAAAGA:0.0664557
chr28 10148288 2 310 GAA:0.919355 G:0.0806452
chr28 10148294 2 242 AAG:0.677686 A:0.322314
chr28 10148300 2 256 GAA:0.601562 G:0.398438
chr28 10148412 2 312 C:0.939103 T:0.0608974
chr28 10149146 2 304 T:0.299342 G:0.700658
chr28 10149410 2 300 A:0.653333 G:0.346667
chr28 10149449 2 302 G:0.870861 T:0.129139
chr28 10149841 2 300 C:0.363333 CT:0.636667
chr28 10149893 2 288 A:0.597222 T:0.402778
chr28 10149980 2 300 C:0.653333 T:0.346667
chr28 10149999 2 296 G:0.97973 C:0.0202703
chr28 10150042 2 278 T:0.97482 C:0.0251799
chr28 10150292 2 308 A:0.649351 T:0.350649
chr28 10150297 2 310 G:0.980645 T:0.0193548
chr28 10150322 2 310 C:0.980645 T:0.0193548
chr28 10150341 2 288 AT:0.635417 A:0.364583
chr28 10150377 4 312 CAG:0.477564 C:0.330128
CAGAGAGAG:0.150641 CAGAGAGAGAG:0.0416667
chr28 10150480 2 310 A:0.980645 ACATCCTGAG:
0.0193548
chr28 10150723 2 314 CCTTAT:0.649682 C:0.350318
chr28 10150822 2 314 C:0.977707 A:0.022293
chr28 10150902 2 312 A:0.259615 T:0.740385
chr28 10150992 2 312 C:0.282051 A:0.717949
chr28 10151037 2 298 G:0.275168 T:0.724832

chr28	10151226	2	304	G:0.667763	A:0.332237		
chr28	10151411	2	288	T:0.642361	C:0.357639		
chr28	10151543	2	306	AT:0.372549	A:0.627451		
chr28	10152372	2	302	C:0.311258	T:0.688742		
chr28	10152413	4	300	TAA:0.39	T:0.263333	TA:0.186667	TAAA:0.16
chr28	10152907	2	298	GA:0.634228	G:0.365772		
chr28	10153343	2	294	G:0.639456	A:0.360544		
chr28	10153365	2	302	C:0.874172	T:0.125828		
chr28	10153418	2	304	C:0.641447	A:0.358553		
chr28	10153642	2	280	C:0.953571	T:0.0464286		
chr28	10153904	2	302	G:0.288079	A:0.711921		
chr28	10153945	2	298	GTC:0.728188	G:0.271812		
chr28	10153996	2	302	T:0.668874	C:0.331126		
chr28	10154047	2	288	A:0.40625	G:0.59375		
chr28	10154104	2	266	A:0.928571	T:0.0714286		
chr28	10154196	2	210	C:0.528571	CCT:0.471429		
chr28	10154306	2	294	C:0.632653	A:0.367347		
chr28	10154338	2	316	C:0.651899	CCAAAAAAAAAAACAAAAAAAACAAAAAAAAATAAATTAAAAAAAAAAAAAAAAAAAAAAAA:0.348101		
chr28	10154339	2	316	G:0.651899	GAACCTGCCTTCC:0.348101		
chr28	10154461	3	264	T:0.318182	TA:0.371212	TAA:0.310606	
chr28	10154689	2	314	C:0.66879	G:0.33121		
chr28	10154839	2	222	CCTCT:0.387387	C:0.612613		
chr28	10154898	3	278	CA:0.392086	C:0.341727	CAAA:0.266187	
chr28	10155088	2	256	TAAAAGTTTTTAAAAA:0.667969	T:0.332031		
chr28	10155093	2	266	GTTTTTAAAAAAAAAGT:0.74812	G:0.25188		
chr28	10155179	2	282	G:0.712766	GA:0.287234		
chr28	10155187	2	282	T:0.606383	A:0.393617		
chr28	10155196	2	282	T:0.70922	A:0.29078		
chr28	10155249	2	234	T:0.67094	C:0.32906		
chr28	10155371	2	276	G:0.40942	C:0.59058		
chr28	10155420	2	286	A:0.402098	T:0.597902		
chr28	10155466	2	240	ATC:0.741667	A:0.258333		
chr28	10155468	2	244	CTCT:0.651639	C:0.348361		
chr28	10155470	2	240	C:0.625	T:0.375		
chr28	10155496	2	244	T:0.651639	TGATA:0.348361		
chr28	10155500	2	244	ACT:0.651639	A:0.348361		
chr28	10155509	2	244	GAC:0.651639	G:0.348361		
chr28	10155611	2	308	GC:0	G:1		
chr28	10155632	2	270	T:0.277778	C:0.722222		
chr28	10155666	2	282	T:0.698582	TA:0.301418		
chr28	10155667	3	292	T:0.0273973	A:0.325342	TA:0.64726	
chr28	10155678	2	284	T:0	TA:1		
chr28	10155975	2	306	C:0.666667	CTAA:0.333333		
chr28	10156113	2	298	C:0.278523	A:0.721477		
chr28	10156169	2	304	AAATT:0.990132	A:0.00986842		

chr28 10156261 2 310 T:0.635484 C:0.364516
chr28 10156606 2 294 G:0.721088 A:0.278912
chr28 10156658 2 226 C:0.433628 CT:0.566372
chr28 10156835 2 228 G:0.5 A:0.5
chr28 10156897 2 258 T:0.344961 C:0.655039
chr28 10157052 2 256 T:0.832031 TA:0.167969
chr28 10157058 4 256 T:0.636719 TTTTA:0.0390625
TTA:0.167969 TTTTATTTATTTA:0.15625
chr28 10157251 2 264 C:0.609848
CTATATGAGTAAAACTTTACAATATTTCTGAAAGATCCAAAAATAGACTGGATCCATCGATAA:
0.390152
chr28 10157831 3 150 TA:0.3 T:0.646667 TAA:
0.0533333
chr28 10158165 2 294 GTGTC:0.819728 G:0.180272
chr28 10158167 3 306 GTCTCTCTC:0.676471 G:
0.222222 GTCTCTC:0.101307
chr28 10158232 3 294 TAAAAAA:0.384354 TA:0.292517
T:0.323129
chr28 10158238 2 272 A:0.650735 ATT:0.349265
chr28 10158290 2 288 A:0.642361 AG:0.357639
chr28 10158392 2 300 T:0.67 C:0.33
chr28 10158447 2 284 CT:0.65493 C:0.34507
chr28 10158486 2 294 T:0.727891 TAC:0.272109
chr28 10158490 3 316 C:0.737342 CAGAGAGAGAGAG:
0.189873 CAGAGAGAGAGAGAG:0.0727848
chr28 10158492 2 298 G:0.959732 C:0.0402685
chr28 10158537 2 298 G:0.620805 T:0.379195
chr28 10158667 2 294 A:0.656463 G:0.343537
chr28 10159009 2 298 G:0.654362 A:0.345638
chr28 10159257 2 302 G:0.986755 GA:0.013245
chr28 10159626 2 312 A:0.733974 T:0.266026
chr28 10159654 2 304 T:0.371711 A:0.628289
chr28 10159695 2 300 T:0.666667 C:0.333333
chr28 10159868 2 130 C:0.738462 CT:0.261538
chr28 10159995 2 128 C:0.90625 T:0.09375
chr28 10160300 2 290 C:0.62069 T:0.37931
chr28 10160324 2 300 C:0.833333 T:0.166667
chr28 10160325 2 300 G:0.32 T:0.68
chr28 10160674 2 304 C:0.259868 A:0.740132
chr28 10161160 2 310 A:0.967742 G:0.0322581
chr28 10161412 2 312 TAATTTATA:0.397436 T:
0.602564
chr28 10161423 2 312 GA:0.971154 G:0.0288462
chr28 10161772 2 294 TTC:0.642857 T:0.357143
chr28 10161810 2 314 CTGTGTGTGTGTG:0.630573 C:
0.369427
chr28 10161852 2 294 T:0.945578 A:0.0544218
chr28 10161975 2 306 C:0.369281 A:0.630719
chr28 10162099 2 272 TCA:0.683824 T:0.316176
chr28 10162212 2 310 CACTT:0.658065 C:0.341935
chr28 10162302 4 300 C:0.323333 CT:0.343333
CTT:0.253333 CTTT:0.08
chr28 10162453 2 284 C:0.690141 T:0.309859
chr28 10162530 2 296 A:0.695946 G:0.304054

chr28 10162553 2 308 C:0.977273 A:0.0227273
chr28 10162727 2 290 G:0.982759 A:0.0172414
chr28 10162764 2 296 A:0.337838 G:0.662162
chr28 10162876 3 292 T:0.671233 TAAA:0.140411
TAAAA:0.188356
chr28 10162901 2 290 T:0.641379 A:0.358621
chr28 10163470 2 166 G:0.837349 T:0.162651
chr28 10164070 2 294 GTCTC:0.942177 G:0.0578231
chr28 10164099 2 290 TCTG:0.931034 T:0.0689655
chr28 10164101 4 316 T:0.594937 TC:0.110759
TCTCTC:0.0411392 TCTCTCTCTCTC:0.253165
chr28 10164102 2 306 G:0.571895 T:0.428105
chr28 10164474 2 306 G:0.588235 A:0.411765
chr28 10164559 2 310 A:0.987097 AGCGCC:0.0129032
chr28 10164587 2 304 C:0.697368 T:0.302632
chr28 10164611 2 300 C:0.876667 T:0.123333
chr28 10164666 2 298 C:0.57047 G:0.42953
chr28 10164669 2 296 TCTTC:0.706081 T:0.293919
chr28 10164671 3 312 TTCGTTC:0.400641 T:0.410256
TGTTC:0.189103
chr28 10164675 2 294 T:0.482993 TC:0.517007
chr28 10164677 2 294 C:0.482993 CT:0.517007
chr28 10164702 2 290 A:0.0206897 AG:0.97931
chr28 10164787 2 300 C:0.713333 G:0.286667
chr28 10164880 2 314 G:0.579618 A:0.420382
chr28 10165039 2 300 TCATG:0.596667 T:0.403333
chr28 10165148 3 314 T:0.375796
TTCTCTCTCTCTCTCTC:0.292994 TTCTCTCTCTCTCTCTCTC:0.33121
chr28 10165184 4 282 TAAA:0.368794 T:0.347518
TA:0.187943 TAA:0.0957447
chr28 10165347 2 218 C:0.311927 CA:0.688073
chr28 10165388 2 284 C:0.707746 T:0.292254
chr28 10165697 2 300 G:0.703333 A:0.296667
chr28 10165799 2 304 C:0.427632 A:0.572368
chr28 10165856 2 312 A:0.730769 G:0.269231
chr28 10166000 2 302 C:0.721854 T:0.278146
chr28 10166114 2 288 T:0.746528 TG:0.253472
chr28 10166115 3 286 GTTT:0.867133 G:0.0314685
GTT:0.101399
chr28 10166116 2 298 T:0.714765 TGG:0.285235
chr28 10166119 2 284 T:0.975352 G:0.0246479
chr28 10166202 2 314 G:0.72293 A:0.27707
chr28 10166217 2 314 T:0.72293 C:0.27707
chr28 10166314 3 304 TGA:0.766447 T:0.111842
TGAGAGA:0.121711
chr28 10166335 2 298 GA:0.744966 G:0.255034
chr28 10166338 2 298 AGAGGC:0.744966 A:0.255034
chr28 10166523 2 302 G:0.986755 GC:0.013245
chr28 10166524 2 300 T:0.29 C:0.71
chr28 10166526 2 302 T:0.986755 C:0.013245
chr28 10166527 2 300 CCA:0.29 C:0.71
chr28 10166529 2 302 A:0.986755 G:0.013245
chr28 10166595 2 302 GAGAGAC:0.758278 G:0.241722
chr28 10166651 2 292 A:0.702055 G:0.297945

chr28 10166815 2 278 C:0.471223 A:0.528777
chr28 10167035 2 216 T:0.615741 TA:0.384259
chr28 10167173 2 300 AT:0.666667 A:0.333333
chr28 10167306 2 256 T:0.972656 C:0.0273438
chr28 10167353 2 254 C:0.614173 T:0.385827
chr28 10167432 2 256 C:0.625 T:0.375
chr28 10167461 2 260 TA:0.615385 T:0.384615
chr28 10167532 2 298 G:0.758389 A:0.241611
chr28 10167797 2 298 C:0.728188 T:0.271812
chr28 10167871 2 308 G:0.324675 A:0.675325
chr28 10167923 2 310 A:0.709677 C:0.290323
chr28 10167993 2 300 A:0.706667 G:0.293333
chr28 10168029 2 284 G:0.292254 GT:0.707746
chr28 10168285 2 302 T:0.291391 C:0.708609
chr28 10168409 2 288 TTG:0.913194 T:0.0868056
chr28 10168410 2 274 TG:0.587591 T:0.412409
chr28 10168483 2 306 A:0.300654 G:0.699346
chr28 10168500 2 306 TA:0.300654 T:0.699346
chr28 10168720 2 306 A:0.300654 G:0.699346
chr28 10169095 2 292 A:0.69863 ATT:0.30137
chr28 10169138 2 306 C:0.934641 A:0.0653595
chr28 10169343 2 166 AT:0.855422 A:0.144578
chr28 10169635 2 306 T:0.990196 A:0.00980392
chr28 10169645 2 304 T:0.427632 C:0.572368
chr28 10169770 2 314 A:0.691083 G:0.308917
chr28 10169804 3 304 CTT:0.394737 C:0.299342
CT:0.305921
chr28 10170040 2 290 T:0.993103 C:0.00689655
chr28 10170089 2 316 G:0.981013 GT:0.0189873
chr28 10170108 2 298 C:0.687919 T:0.312081
chr28 10170182 5 314 T:0.735669 TTTTA:0.0350318
TTATTTA:0.089172 TTATTTATTTA:0.0191083 TTATTTATTTATTTA:0.121019
chr28 10170238 2 298 A:0.724832 G:0.275168
chr28 10170244 2 298 G:0.724832 T:0.275168
chr28 10170287 2 306 G:0.954248 A:0.0457516
chr28 10170290 2 296 G:0.722973 A:0.277027
chr28 10170527 2 308 T:0.720779 C:0.279221
chr28 10170663 2 300 C:0.613333 T:0.386667
chr28 10170835 2 304 T:0.289474 A:0.710526
chr28 10170916 2 312 TA:0.605769 T:0.394231
chr28 10171137 2 306 G:0.297386 A:0.702614
chr28 10171237 2 304 C:0.713816 T:0.286184
chr28 10171367 2 306 G:0.565359 GA:0.434641
chr28 10171752 2 288 T:0.236111 C:0.763889
chr28 10171820 2 258 T:0.22093 TAAA:0.77907
chr28 10171859 2 296 G:0.719595 A:0.280405
chr28 10171865 2 296 G:0.719595 A:0.280405
chr28 10171943 2 302 G:0.731788 C:0.268212
chr28 10172177 2 308 C:0.704545 A:0.295455
chr28 10172284 2 304 CT:0.973684 C:0.0263158
chr28 10172425 2 304 A:0.713816 C:0.286184
chr28 10172799 2 302 C:0.711921 T:0.288079
chr28 10173033 2 310 A:0.729032 G:0.270968
chr28 10173460 2 294 G:0.721088 C:0.278912

chr28 10174465 2 296 C:0.283784 T:0.716216
chr28 10174656 2 304 C:0.707237 T:0.292763
chr28 10175018 2 268 TA:0.731343 T:0.268657
chr28 10175020 2 272 A:0.933824 T:0.0661765
chr28 10175416 2 296 G:0 C:1
chr28 10175462 2 288 C:0.739583 T:0.260417
chr28 10175676 2 302 GCAA:0.721854 G:0.278146
chr28 10175995 2 298 A:0.711409 G:0.288591
chr28 10176036 2 296 G:0.706081 A:0.293919
chr28 10176088 2 288 A:0.732639 T:0.267361
chr28 10176155 2 314 T:0.726115
TAAAATATTTTTGTTGAAAAAAAAAAGAAAAAA:0.273885
chr28 10176451 2 300 C:0.7 T:0.3
chr28 10176452 2 300 G:0.926667 A:0.0733333
chr28 10176636 2 298 G:0.718121 A:0.281879
chr28 10177037 2 302 C:0 CA:1
chr28 10177053 2 302 C:0 CA:1
chr28 10177172 2 290 G:0.696552 A:0.303448
chr28 10177588 2 314 T:0.719745 A:0.280255
chr28 10177752 2 296 C:0.709459 A:0.290541
chr28 10177792 2 288 T:0.732639 C:0.267361
chr28 10177951 2 310 G:0.732258 A:0.267742
chr28 10177995 2 298 C:0.751678 T:0.248322
chr28 10178073 2 286 G:0.573427 A:0.426573
chr28 10178127 2 300 T:0.75 C:0.25
chr28 10178215 2 298 T:0.781879 TA:0.218121
chr28 10178216 3 300 T:0.78 TA:0.0566667 TATA:
0.163333
chr28 10178259 2 298 G:0.748322 A:0.251678
chr28 10178587 2 314 CTGGGGAGATGCTT:0.726115 C:
0.273885
chr28 10178669 2 292 G:0.746575 A:0.253425
chr28 10178780 2 304 CA:0.736842 C:0.263158
chr28 10178917 2 292 C:0.722603 T:0.277397
chr28 10178994 2 308 T:0.746753 G:0.253247
chr28 10179163 2 298 C:0.959732 T:0.0402685
chr28 10179338 2 312 T:0.798077 C:0.201923
chr28 10179482 2 312 A:0.865385 T:0.134615
chr28 10179722 2 270 G:0.52963 GTT:0.47037
chr28 10179732 2 316 T:0.873418 TG:0.126582
chr28 10179736 2 316 T:0.870253 TGTTTTTTTG:
0.129747
chr28 10179777 2 288 G:0.861111 A:0.138889
chr28 10179926 2 292 A:0.873288 T:0.126712
chr28 10180117 2 308 C:0.876623 T:0.123377
chr28 10180121 2 308 T:0.876623 C:0.123377
chr28 10180448 2 312 A:0.871795 AGAGAGAGAGAGC:
0.128205
chr28 10180452 2 294 C:0.55102 T:0.44898
chr28 10180559 2 294 A:0.418367 G:0.581633
chr28 10180810 2 272 T:0.871324 C:0.128676
chr28 10180918 2 288 TA:0.916667 T:0.0833333
chr28 10180948 2 300 T:0.853333 A:0.146667
chr28 10181070 2 306 A:0.856209 AT:0.143791

chr28 10181208 2 314 G:0.585987 A:0.414013
chr28 10181272 2 316 A:0.96519 C:0.0348101
chr28 10181355 2 306 C:0.895425 T:0.104575
chr28 10181433 2 312 T:0.961538 C:0.0384615
chr28 10181455 2 314 A:0.980892 ATGCTGC:0.0191083
chr28 10181479 2 308 CTTCTGTTT:0.899351 C:
0.100649
chr28 10181484 2 316 G:0.756329 GT:0.243671
chr28 10181492 2 298 TTG:0.946309 T:0.0536913
chr28 10181494 2 306 G:0.663399 T:0.336601
chr28 10181512 4 302 GTTT:0.470199 G:0.102649
GTTTT:0.0562914 TTTT:0.370861
chr28 10182245 2 304 T:0.828947 C:0.171053
chr28 10182413 2 300 G:0.8 T:0.2
chr28 10182486 2 302 T:0.807947 C:0.192053
chr28 10182646 2 306 G:0.80719 A:0.19281
chr28 10182848 4 294 CT:0.102041 C:0.197279
CTT:0.346939 CTTT:0.353741
chr28 10183221 2 310 G:0.793548 A:0.206452
chr28 10183345 2 306 TAGTC:0.800654 T:0.199346
chr28 10183420 2 300 T:0.793333 C:0.206667
chr28 10183703 2 320 G:0.90625 GTTTTTTTTTT:
0.09375
chr28 10184034 2 260 T:0.784615 TAA:0.215385
chr28 10184066 2 278 C:0.81295 CT:0.18705
chr28 10184150 2 306 A:0.660131 T:0.339869
chr28 10184152 2 308 ATTTATTT:0.652597 A:
0.347403
chr28 10184489 2 304 A:0.421053 G:0.578947
chr28 10184650 2 320
CCAAACTTTCTTTTTCTTTTTTTTTTTTTTTTTTTTTTTTTTTTTTTTTT:0.028125 C:
0.971875
chr28 10184933 2 304 T:0.930921 C:0.0690789
chr28 10185046 2 308 TA:0.779221 T:0.220779
chr28 10185574 2 294 C:0.959184 T:0.0408163
chr28 10185602 2 304 T:0.930921 A:0.0690789
chr28 10185825 2 300 G:0.993333 A:0.00666667
chr28 10186039 2 304 A:0.766447 AT:0.233553
chr28 10186510 2 312 G:0.333333 A:0.666667
chr28 10187094 2 310 TA:0.435484 T:0.564516
chr28 10187445 2 308 AAAC:0.772727 A:0.227273
chr28 10187515 2 306 G:0.565359 A:0.434641
chr28 10187527 2 306 G:0.444444 A:0.555556
chr28 10187572 2 296 C:0.763514 A:0.236486
chr28 10187573 2 296 G:0.432432 A:0.567568
chr28 10187947 2 310 G:0.606452
GAAAACATGTCCACACAAAAATGTGTTTAC:0.393548
chr28 10188244 2 302 TC:0.890728 T:0.109272
chr28 10188277 2 288 C:0.6875 G:0.3125
chr28 10188361 2 298 T:0.949664 TA:0.0503356
chr28 10188362 2 302 T:0.331126 A:0.668874
chr28 10188490 2 306 C:0.627451 T:0.372549
chr28 10189517 2 280 TC:0.739286 T:0.260714
chr28 10189654 2 272 G:0.654412 C:0.345588

chr28 10189689 2 284 C:0.623239 T:0.376761
chr28 10189692 2 282 T:0.666667 C:0.333333
chr28 10189913 2 294 A:0.663265 G:0.336735
chr28 10190601 2 308 G:0.974026 A:0.025974
chr28 10190834 2 302 T:0.321192 A:0.678808
chr28 10191435 2 310 C:0.341935 G:0.658065
chr28 10191496 2 298 A:0.338926 G:0.661074
chr28 10191911 2 242 TA:0.669421 T:0.330579
chr28 10191933 2 242 G:0.669421 A:0.330579
chr28 10192231 2 286 C:0.961538 T:0.0384615
chr28 10192255 2 286 C:0.667832 T:0.332168
chr28 10192561 2 274 A:0.854015 T:0.145985
chr28 10192640 2 270 T:0.544444 A:0.455556
chr28 10192664 2 258 C:0.244186 G:0.755814
chr28 10192898 2 154 G:0.623377 C:0.376623
chr28 10194221 2 252 T:0.698413 C:0.301587
chr28 10194540 3 282 G:0.865248 GTGC:0.0673759
GTGCTGC:0.0673759
chr28 10194740 2 300 A:0.656667 G:0.343333
chr28 10195054 2 298 CTT:0.862416 C:0.137584
chr28 10195593 2 306 GTATT:0.77451 G:0.22549
chr28 10195621 3 316 GT:0.64557 G:0.0696203
GTTTTT:0.28481
chr28 10196362 2 308 A:0.36039 G:0.63961
chr28 10196855 2 286 CTT:0.968531 C:0.0314685
chr28 10196857 4 316 TTTTTC:0.740506 T:0.056962
TTTCTTTTC:0.155063 TTTTTCTTTTCTTTTCTTTTC:0.0474684
chr28 10196894 2 316 T:0.908228 TTTCTTTTTTCTTTTC:
0.0917722
chr28 10196944 2 316 C:0.971519 CCTTT:0.028481
chr28 10196965 2 312 CTTTCTTT:0.971154 C:
0.0288462
chr28 10196972 2 310 T:0.851613 TC:0.148387
chr28 10196985 2 308 C:0.775974 T:0.224026
chr28 10198571 2 296 A:0.280405 AAATT:0.719595
chr28 10198572 2 298 A:0.879195 AATT:0.120805
chr28 10198658 4 318 C:0.386792 CATAA:0.361635
CATAAATAA:0.144654 CATAAATAAATAA:0.106918
chr28 10199197 2 294 C:0.884354 T:0.115646
chr28 10199651 2 294 C:0.142857 CT:0.857143
chr28 10199887 2 294 TTTTA:0.863946 T:0.136054
chr28 10201240 2 292 CATTT:0.0239726 C:0.976027
chr28 10201918 2 296 C:0.837838 T:0.162162
chr28 10202452 2 304 A:0.0263158 G:0.973684
chr28 10204117 2 310 T:0.0225806 A:0.977419
chr28 10204662 2 298 T:0.35906 A:0.64094
chr28 10204694 2 270 C:0.992593 CAG:0.00740741
chr28 10204858 2 304 T:0.328947 C:0.671053
chr28 10204975 2 304 A:0.332237 T:0.667763
chr28 10206008 2 306 C:0.957516 T:0.0424837
chr28 10206285 3 294 TA:0.479592 T:0.0646259
TAA:0.455782
chr28 10206466 2 296 C:0 T:1
chr28 10206866 2 262 C:0.717557 CA:0.282443

chr28	10206960	2	296	A:0.486486	AT:0.513514
chr28	10209139	2	250	C:0.5	CT:0.5
chr28	10209599	2	266	C:0.954887	T:0.0451128
chr28	10209824	2	302	T:0.86755	C:0.13245
chr28	10210027	2	308	T:0.363636	TA:0.636364
chr28	10210545	2	310	G:0.651613	C:0.348387
chr28	10211524	2	300	CAG:0.95	C:0.05
chr28	10211592	2	284	C:0.901408	T:0.0985915
chr28	10211945	2	294	G:0.360544	A:0.639456
chr28	10213084	2	300	A:0.343333	G:0.656667
chr28	10214082	2	298	C:0.959732	T:0.0402685
chr28	10215146	2	316	TAA:0.933544	T:0.0664557
chr28	10215148	2	316	A:0.933544	ATTTTTTTTTTTTT:0.0664557
chr28	10215235	2	274	A:0.635036	G:0.364964
chr28	10215560	2	194	A:0.309278	C:0.690722
chr28	10215797	2	270	CT:0.614815	C:0.385185
chr28	10216136	2	302	T:0.665563	G:0.334437
chr28	10216903	2	300	TTTTA:0.92	T:0.08
chr28	10217303	2	304	C:0.355263	G:0.644737
chr28	10217443	2	306	T:0.918301	G:0.0816993
chr28	10217656	2	298	C:0.0033557	T:0.996644
chr28	10217667	2	298	A:0	T:1
chr28	10217670	2	298	A:0	T:1
chr28	10217684	2	290	A:0	G:1
chr28	10217713	2	300	G:0	C:1
chr28	10217717	2	300	G:0	C:1
chr28	10217725	2	300	G:0	A:1
chr28	10217727	2	300	T:0	C:1
chr28	10217732	2	300	TG:0	T:1
chr28	10217750	2	304	G:0	T:1
chr28	10217757	2	304	A:0	G:1
chr28	10217769	2	312	C:0	CA:1
chr28	10217772	2	312	G:0	T:1
chr28	10217773	2	312	AT:0	A:1
chr28	10217776	2	312	T:0	C:1
chr28	10217785	2	316	G:0	GC:1
chr28	10217791	2	316	TCTAG:0	T:1
chr28	10217796	2	316	A:0	ATC:1
chr28	10217805	2	316	A:0	T:1
chr28	10217814	2	316	A:0	C:1
chr28	10217815	2	316	C:0	A:1
chr28	10217816	2	316	T:0	G:1
chr28	10217887	2	302	G:0	A:1
chr28	10219877	2	296	A:0.648649	G:0.351351
chr28	10220208	2	288	G:0.0208333	GA:0.979167
chr28	10220401	2	306	T:0.630719	TC:0.369281
chr28	10220414	2	304	A:0.855263	T:0.144737
chr28	10220416	2	304	A:0.302632	T:0.697368
chr28	10220438	2	288	G:0.420139	GT:0.579861
chr28	10220523	2	300	G:0.35	A:0.65
chr28	10220530	2	300	G:0.983333	A:0.0166667
chr28	10220819	2	304	A:0.927632	T:0.0723684
chr28	10220844	2	276	T:0.684783	C:0.315217

chr28 10221024 2 310 A:0 G:1
chr28 10221026 2 310 A:0 G:1
chr28 10221031 2 310 AG:0 A:1
chr28 10221051 2 310 G:0 A:1
chr28 10221065 2 310 A:0 ATATT:1
chr28 10221066 2 310 AAGGG:0 A:1
chr28 10221075 2 310 T:0 A:1
chr28 10221092 2 310 GAA:0 G:1
chr28 10221094 2 310 A:0 ATTT:1
chr28 10221096 2 310 C:0 CT:1
chr28 10221102 2 310 T:0 TA:1
chr28 10221130 2 310 G:0 T:1
chr28 10221169 2 292 A:0 T:1
chr28 10221188 2 292 G:0 T:1
chr28 10221251 2 284 C:0.96831 CCT:0.0316901
chr28 10221651 2 282 C:0.960993 CT:0.0390071
chr28 10221784 2 302 TTTCTTTC:0.556291 T:0.443709
chr28 10221870 2 280 CTCTTTCTTTCTTTCTTTCTTTCTT:0.771429 C:0.228571
chr28 10222097 2 316 TTCTTTCTTTCTTTCTTCCTTTCTCTCTCTCTCTC:0.443038 T:0.556962
chr28 10225079 2 294 TCCC:0.37415 T:0.62585
chr28 10225082 2 294 C:0.642857 T:0.357143
chr28 10225086 2 258 A:0.852713 ATTC:0.147287
chr28 10225154 2 278 G:0.971223 A:0.028777
chr28 10226261 4 304 CAG:0.450658 C:0.0921053 CAGAG:0.256579 CAGAGAG:0.200658
chr28 10226865 2 308 A:0.961039 C:0.038961
chr28 10227301 2 308 A:0.37013 G:0.62987
chr28 10227734 3 244 C:0.196721 CT:0.553279 CTT:0.25
chr28 10227822 2 292 AT:0.667808 A:0.332192
chr28 10228493 2 314 TAAA:0.987261 T:0.0127389
chr28 10228498 2 314 A:0.987261 T:0.0127389
chr28 10228609 2 308 C:0.863636 T:0.136364
chr28 10229110 2 308 GA:0.720779 G:0.279221
chr28 10229543 2 302 G:0.963576 GT:0.0364238
chr28 10229764 2 302 AT:0.642384 A:0.357616
chr28 10229828 2 274 C:0.627737 CT:0.372263
chr28 10230082 2 300 C:0.86 A:0.14
chr28 10231615 2 302 G:0.956954 C:0.0430464
chr28 10231666 5 318 TTATCTATCTATCTATC:0.191824 T:0.188679 TTATC:0.110063 TTATCTATCTATC:0.380503 TTATCTATCTATCTATCTATC:0.128931
chr28 10232630 2 306 T:0.00326797 A:0.996732
chr28 10232644 2 306 ATTT:0.00326797 A:0.996732
chr28 10232649 2 306 T:0.00326797 TGG:0.996732
chr28 10232651 2 306 A:0.00326797 G:0.996732
chr28 10232653 2 306 GC:0.00326797 G:0.996732
chr28 10232658 2 306 A:0.00326797 G:0.996732
chr28 10232662 2 306 C:0.00326797 T:0.996732
chr28 10232681 2 306 C:0.00326797 A:0.996732
chr28 10232696 2 306 G:0.00326797 T:0.996732

chr28	10232705	2	306	C:0.00326797	A:0.996732	
chr28	10232731	2	300	A:0	T:1	
chr28	10232738	2	300	T:0	A:1	
chr28	10232760	2	300	C:0	A:1	
chr28	10232886	2	284	GT:0.806338	G:0.193662	
chr28	10233249	2	302	T:0.34106	A:0.65894	
chr28	10233584	2	284	CT:0.362676	C:0.637324	
chr28	10233628	3	290	A:0.672414	AAG:0.296552	AAGAG:0.0310345
chr28	10234058	2	304	T:0.355263	A:0.644737	
chr28	10234981	2	308	TA:0.863636	T:0.136364	
chr28	10236023	2	314	CTGATTGAT:0.872611	C:0.127389	
chr28	10236871	2	294	C:0.340136	A:0.659864	
chr28	10237348	3	318	T:0.704403	TTTTTA:0.257862	TTTTTATTTTA:0.0377358
chr28	10237407	3	306	CAG:0.820261	C:0.0718954	CAGAG:0.107843
chr28	10237409	2	298	G:0.691275	C:0.308725	
chr28	10237514	2	266	G:0.654135	C:0.345865	
chr28	10237537	2	282	G:0.663121	A:0.336879	
chr28	10239051	2	294	AGT:0.952381	A:0.047619	
chr28	10239081	2	290	T:0.668966	A:0.331034	
chr28	10239174	2	304	T:0.654605	A:0.345395	
chr28	10241743	2	292	C:0.952055	T:0.0479452	
chr28	10241847	2	304	A:0.375	T:0.625	
chr28	10241959	2	298	C:0.788591	T:0.211409	
chr28	10242101	2	306	A:0.647059	C:0.352941	
chr28	10242211	2	304	AT:0.572368	A:0.427632	
chr28	10243424	2	314	T:0.990446	C:0.00955414	
chr28	10243517	2	320	A:0.421875	AAAAAC:0.578125	
chr28	10243960	2	302	C:0.36755	CCT:0.63245	
chr28	10244078	2	306	T:0.333333	G:0.666667	
chr28	10244190	2	296	C:0.324324	A:0.675676	
chr28	10244813	2	308	A:0.655844	G:0.344156	
chr28	10245114	3	290	G:0.375862	GT:0.47931	GTT:0.144828
chr28	10245632	2	308	C:0.954545	T:0.0454545	
chr28	10246538	2	308	C:0.941558	CT:0.0584416	
chr28	10246807	3	268	CTT:0.436567	C:0.466418	CTTT:0.0970149
chr28	10247331	2	286	TA:0.632867	T:0.367133	
chr28	10247387	2	284	TA:0.841549	T:0.158451	
chr28	10247592	2	228	G:0.684211	GT:0.315789	
chr28	10247617	2	270	C:0.651852	CT:0.348148	
chr28	10247886	2	308	T:0.62987	C:0.37013	
chr28	10248273	2	302	AC:0.837748	A:0.162252	
chr28	10248337	2	306	T:0.954248	TGAGA:0.0457516	
chr28	10250140	2	298	A:0.681208	G:0.318792	
chr28	10250481	2	308	CT:0.850649	C:0.149351	
chr28	10250920	2	306	T:0.650327	C:0.349673	
chr28	10251016	2	312	T:0.987179	G:0.0128205	
chr28	10252020	2	306	G:0.74183	A:0.25817	
chr28	10254917	2	268	TCA:0.929104	T:0.0708955	

chr28	10254924	2	270	C:0.392593	G:0.607407	
chr28	10255526	2	284	G:0.00704225	C:0.992958	
chr28	10255597	2	306	C:0	A:1	
chr28	10255637	2	308	G:0	T:1	
chr28	10255654	2	308	T:0	A:1	
chr28	10255824	2	260	CCT:0.676923	C:0.323077	
chr28	10256382	2	300	C:0.65	T:0.35	
chr28	10256928	2	302	C:0.94702	CA:0.0529801	
chr28	10257348	2	286	G:0	A:1	
chr28	10257358	2	286	G:0	A:1	
chr28	10258105	2	298	CT:0.399329	C:0.600671	
chr28	10258193	2	292	A:0.00342466	G:0.996575	
chr28	10259148	2	276	C:0.934783	CAG:0.0652174	
chr28	10259732	2	302	T:0.970199	TA:0.0298013	
chr28	10259775	3	308	ATTTTAT:0.62987	A:0.301948	ATTTTATTTTTAT:0.0681818
chr28	10259839	2	266	C:0.5	CAG:0.5	
chr28	10261339	2	306	T:0.96732	A:0.0326797	
chr28	10261340	2	306	T:0.895425	A:0.104575	
chr28	10261358	2	296	T:0.344595	A:0.655405	
chr28	10263091	2	294	T:0.0204082	A:0.979592	
chr28	10263112	2	294	AGACTGGGAG:0.0204082	A:0.979592	
chr28	10263138	2	84	AAAACCAAAAAAAAC:0.119048	A:0.880952	
chr28	10263139	2	84	AAACCAAAAAAAAC:0.928571	A:0.0714286	
chr28	10263157	2	294	T:0.0204082	A:0.979592	
chr28	10263164	2	294	C:0.0204082	A:0.979592	
chr28	10263165	2	294	C:0.0204082	T:0.979592	
chr28	10263184	2	294	C:0.0204082	T:0.979592	
chr28	10263187	2	294	G:0.0204082	T:0.979592	
chr28	10263570	2	312	CCTTTTCCTTTTCCTTTTCCTTTTTCTTTTCTTTCTTTT:0.926282	C:0.0737179	
chr28	10263584	2	314	TTTTCCTTTTTCTTTTCTTTC:0.980892	T:0.0191083	
chr28	10263600	2	316	CTTT:0.946203	C:0.0537975	
chr28	10263781	2	192	GCCCCCCC:0.192708	G:0.807292	
chr28	10263940	2	214	G:0.443925	GTTTT:0.556075	
chr28	10264356	2	288	CT:0.333333	C:0.666667	
chr28	10264459	2	292	T:0.832192	A:0.167808	
chr28	10264992	2	296	C:0.993243	G:0.00675676	
chr28	10265466	2	296	T:0.952703	A:0.0472973	
chr28	10265470	2	296	G:0.64527	T:0.35473	
chr28	10265967	2	308	AT:0.659091	A:0.340909	
chr28	10265975	2	308	T:0.659091	A:0.340909	
chr28	10266146	2	294	G:0.959184	GT:0.0408163	
chr28	10267018	2	308	T:0.659091	C:0.340909	
chr28	10267050	2	310	T:0.932258	A:0.0677419	
chr28	10267748	2	300	T:0.78	A:0.22	
chr28	10269982	3	308	A:0.0616883	T:0.324675	ATT:0.613636
chr28	10269988	2	300	T:0.973333	TTA:0.0266667	

chr28	10271879	2	304	C:0.641447	CT:0.358553			
chr28	10272031	2	284	C:0.841549	CT:0.158451			
chr28	10272409	2	282	C:0.659574	CT:0.340426			
chr28	10272452	2	260	C:0.876923	CAG:0.123077			
chr28	10273279	2	308	C:0.340909	T:0.659091			
chr28	10273590	2	316	A:0	T:1			
chr28	10273628	2	308	G:0	T:1			
chr28	10273855	2	308	C:0.964286	T:0.0357143			
chr28	10274988	2	310	C:0.645161	CT:0.354839			
chr28	10275285	2	312	G:0.641026	A:0.358974			
chr28	10275539	2	292	A:0.65411	ATATG:0.34589			
chr28	10275577	2	304	G:0.871711	A:0.128289			
chr28	10275764	2	304	G:0.345395	A:0.654605			
chr28	10275837	2	308	T:0.318182	A:0.681818			
chr28	10277251	2	306	C:0.653595	A:0.346405			
chr28	10277361	2	304	T:0.674342	TTTTTG:0.325658			
chr28	10277457	2	300	CTG:0.66	C:0.34			
chr28	10277913	2	304	A:0.845395	G:0.154605			
chr28	10278194	2	292	T:0	A:1			
chr28	10278201	2	292	C:0	G:1			
chr28	10278206	2	292	G:0	T:1			
chr28	10278208	2	292	C:0	T:1			
chr28	10278214	2	292	TA:0	T:1			
chr28	10278235	2	294	C:0	A:1			
chr28	10278245	2	294	T:0	A:1			
chr28	10278248	2	294	C:0	T:1			
chr28	10278252	2	294	T:0	A:1			
chr28	10278278	2	306	G:0	T:1			
chr28	10278281	2	306	A:0	T:1			
chr28	10278287	2	306	G:0	T:1			
chr28	10278347	2	310	G:0	T:1			
chr28	10278366	2	304	T:0	TG:1			
chr28	10278374	2	304	GA:0	G:1			
chr28	10278377	2	304	A:0	T:1			
chr28	10278380	2	304	C:0	T:1			
chr28	10278383	2	304	C:0	T:1			
chr28	10278384	2	304	G:0	GT:1			
chr28	10278389	2	304	A:0	T:1			
chr28	10278392	2	304	GA:0	G:1			
chr28	10278414	2	284	G:0	T:1			
chr28	10278426	2	306	A:0.0588235	AG:0.941176			
chr28	10278427	2	306	A:0.0588235	AT:0.941176			
chr28	10278429	2	306	C:0.0588235	T:0.941176			
chr28	10278431	2	306	T:0.0588235	TA:0.941176			
chr28	10278436	2	306	C:0.0588235	CA:0.941176			
chr28	10278862	5	308	T:0.418831	TGAGA:0.275974	TGAGAGA:0.11039	TGAGAGAGA:0.149351	TGAGAGAGAGA:0.0454545
chr28	10280142	2	306	C:0.640523	T:0.359477			
chr28	10282101	2	310	A:0.332258	C:0.667742			
chr28	10283438	4	316	TATTC:0.262658	T:0.496835	TATTCATTC:0.0506329	TATTCATTCATTC:0.189873	
chr28	10283617	2	302	C:0.950331	T:0.0496689			
chr28	10283905	4	304	TCACACA:0.384868	T:0.529605	TCA:0.0328947	TCACA:0.0526316	

chr28	10284389	2	300	CT:0.916667	C:0.0833333	
chr28	10284402	2	300	T:0.35	A:0.65	
chr28	10284429	2	282	C:0.43617	CAG:0.56383	
chr28	10284649	2	304	T:0.855263	G:0.144737	
chr28	10286031	2	290	CT:0.334483	C:0.665517	
chr28	10286311	2	280	T:0.925	TA:0.075	
chr28	10286602	2	184	TG:0.679348	T:0.320652	
chr28	10286866	2	286	A:0.423077	AT:0.576923	
chr28	10288118	2	304	C:0.351974	A:0.648026	
chr28	10289368	2	314	AAATG:0.659236	A:0.340764	
chr28	10290222	2	306	C:0.362745	T:0.637255	
chr28	10291300	2	304	T:0.983553	A:0.0164474	
chr28	10291599	2	302	T:0.327815	G:0.672185	
chr28	10292188	2	302	G:0.956954	GC:0.0430464	
chr28	10292196	2	304	C:0.868421	A:0.131579	
chr28	10293113	2	296	A:0.763514	AC:0.236486	
chr28	10293942	2	314	A:0.872611	G:0.127389	
chr28	10293975	2	312	CTG:0.355769	C:0.644231	
chr28	10295027	2	314	G:0.993631	T:0.00636943	
chr28	10296691	2	304	C:0.345395	A:0.654605	
chr28	10297215	2	170	A:0.864706	AT:0.135294	
chr28	10297228	2	146	A:0.342466	AT:0.657534	
chr28	10297234	2	172	T:0.877907	TA:0.122093	
chr28	10297242	2	152	A:0.796053	T:0.203947	
chr28	10297584	2	308	T:0.951299	G:0.0487013	
chr28	10298321	2	272	T:0.341912	G:0.658088	
chr28	10299018	2	310	A:0.329032	C:0.670968	
chr28	10299242	2	304	G:0.351974	GTGATGA:0.648026	
chr28	10299389	2	280	AG:0.942857	A:0.0571429	
chr28	10299571	2	296	CT:0.915541	C:0.0844595	
chr28	10299671	2	292	G:0.633562	A:0.366438	
chr28	10300921	2	296	T:0.320946	C:0.679054	
chr28	10303275	2	310	T:0.893548	TAATAATTAAATA:0.106452	
chr28	10303278	2	272	TA:0.838235	T:0.161765	
chr28	10303281	2	260	A:0.811538	T:0.188462	
chr28	10303282	2	272	TAAA:0.838235	T:0.161765	
chr28	10303322	2	308	T:0.892857	TAAATAAA:0.107143	
chr28	10305770	2	304	GT:0.351974	G:0.648026	
chr28	10306110	2	310	G:0.977419	A:0.0225806	
chr28	10306667	3	284	G:0.362676	GT:0.137324	GTT:0.5
chr28	10308168	2	300	CTTTG:0.95	C:0.05	
chr28	10308216	2	274	TA:0.715328	T:0.284672	
chr28	10308710	2	306	T:0.986928	C:0.0130719	
chr28	10309124	2	316	T:0.905063	TCTTCTCCTTCTC:0.0949367	
chr28	10309226	2	308	T:0.99026	A:0.00974026	
chr28	10309300	2	298	G:0.630872	A:0.369128	
chr28	10309586	2	300	T:0.33	TTG:0.67	
chr28	10309762	2	314	C:0.977707	T:0.022293	
chr28	10314496	2	308	AG:0.318182	A:0.681818	
chr28	10318186	2	296	T:0.351351	G:0.648649	
chr28	10320740	2	304	C:0.868421	T:0.131579	

chr28	10322045	2	310	T:0.96129	A:0.0387097	
chr28	10322857	2	304	G:0.861842	A:0.138158	
chr28	10323774	2	308	A:0.857143	G:0.142857	
chr28	10324368	2	286	TAC:0.853147	T:0.146853	
chr28	10326115	2	306	A:0.69281	T:0.30719	
chr28	10326851	2	290	T:0.834483	TA:0.165517	
chr28	10327782	2	306	TA:0.660131	T:0.339869	
chr28	10328178	2	308	AG:0.974026	A:0.025974	
chr28	10328491	2	276	C:0.532609	CCTCT:0.467391	
chr28	10329049	3	270	TA:0.277778	T:0.444444	TAA:0.277778
chr28	10330111	2	298	G:0.989933	A:0.0100671	
chr28	10330709	2	258	G:0	T:1	
chr28	10330904	2	294	TA:0.547619	T:0.452381	
chr28	10331042	2	300	A:0.983333	T:0.0166667	
chr28	10331876	2	306	G:0.980392	A:0.0196078	
chr28	10333265	2	302	C:0.688742	T:0.311258	
chr28	10333520	2	296	G:0.668919	A:0.331081	
chr28	10335857	2	310	G:0.987097	A:0.0129032	
chr28	10336123	2	312	CTTTG:0.679487	C:0.320513	
chr28	10336126	2	312	T:0.326923	G:0.673077	
chr28	10336127	2	312	G:0.326923	T:0.673077	
chr28	10337878	2	308	TTAGCTGCATG:0.922078	T:0.0779221	
chr28	10338084	2	300	A:0.68	AT:0.32	
chr28	10338969	2	312	A:0.666667	G:0.333333	
chr28	10342121	2	306	C:0.964052	G:0.0359477	
chr28	10345207	2	296	G:0.341216	T:0.658784	
chr28	10346971	2	294	A:0.687075	AT:0.312925	
chr28	10346982	2	304	AT:0.921053	A:0.0789474	
chr28	10346991	2	306	T:0.970588	A:0.0294118	
chr28	10347370	2	292	G:0.972603	T:0.0273973	
chr28	10347881	2	294	T:0.687075	C:0.312925	
chr28	10348188	2	290	G:0.986207	A:0.0137931	
chr28	10348754	2	300	T:0	C:1	
chr28	10350541	2	308	C:0.974026	T:0.025974	
chr28	10352784	2	288	T:0.291667	G:0.708333	
chr28	10353339	2	190	C:0.731579	T:0.268421	
chr28	10353730	2	288	C:0.822917	A:0.177083	
chr28	10353892	2	290	C:0.97931	T:0.0206897	
chr28	10354223	2	296	T:0.736486	C:0.263514	
chr28	10354372	2	296	G:0.672297	GC:0.327703	
chr28	10354374	2	296	G:0.672297	T:0.327703	
chr28	10354908	2	300	C:0.986667	T:0.0133333	
chr28	10355356	2	274	C:0.715328	CA:0.284672	
chr28	10355921	2	306	A:0.323529	G:0.676471	
chr28	10356475	2	244	TG:0.82377	T:0.17623	
chr28	10357042	2	288	T:0.697917	C:0.302083	
chr28	10358217	2	270	TA:0.87037	T:0.12963	
chr28	10358632	2	294	C:0.333333	A:0.666667	
chr28	10358988	2	296	G:0.952703	A:0.0472973	
chr28	10359688	2	306	C:0.980392	T:0.0196078	
chr28	10360705	2	300	T:0	A:1	
chr28	10360707	2	300	A:0	AT:1	

chr28	10360711	2	300	G:0	T:1		
chr28	10360718	2	300	A:0	AT:1		
chr28	10360739	2	304	T:0	G:1		
chr28	10360895	2	306	A:0.316993	G:0.683007		
chr28	10361065	2	302	G:0.307947	A:0.692053		
chr28	10361098	2	298	T:0.318792	C:0.681208		
chr28	10362366	2	300	C:0.303333	G:0.696667		
chr28	10363075	3	282	ATG:0.882979	A:0.0780142	ATGTG:0.0390071	
chr28	10363085	2	274	G:0.883212	GTA:0.116788		
chr28	10363087	4	288	G:0.541667	A:0.204861	GTA:0.0625	GTATA:0.190972
chr28	10363501	2	310	C:0.96129	T:0.0387097		
chr28	10363642	2	304	G:0.322368	A:0.677632		
chr28	10363714	2	304	G:0.654605	C:0.345395		
chr28	10363864	2	282	T:0.319149	C:0.680851		
chr28	10364030	2	302	G:0.655629	C:0.344371		
chr28	10364043	2	306	G:0.336601	A:0.663399		
chr28	10365147	2	270	G:0.677778	A:0.322222		
chr28	10365157	2	270	A:0.974074	C:0.0259259		
chr28	10365235	2	308	G:0.672078	GAACGCTGCAGCCC:0.327922		
chr28	10365596	2	288	G:0.694444	A:0.305556		
chr28	10365841	2	300	CGG:0.33	C:0.67		
chr28	10365890	2	302	T:0.304636	C:0.695364		
chr28	10366396	2	298	C:0.95302	T:0.0469799		
chr28	10366452	2	292	C:0.952055	T:0.0479452		
chr28	10366773	2	280	G:0.935714	A:0.0642857		
chr28	10366873	2	282	G:0.301418	A:0.698582		
chr28	10367446	2	304	CCG:0.756579	C:0.243421		
chr28	10367453	2	304	C:0.756579	CACACA:0.243421		
chr28	10367644	2	292	C:0.688356	T:0.311644		
chr28	10367688	2	282	G:0	C:1		
chr28	10367732	2	286	G:0.304196	T:0.695804		
chr28	10368120	2	304	T:0.305921	C:0.694079		
chr28	10369407	2	278	A:0.917266	AGT:0.0827338		
chr28	10370816	2	296	G:0	A:1		
chr28	10370817	2	296	A:0	T:1		
chr28	10370858	2	302	T:0	C:1		
chr28	10370865	2	302	C:0	G:1		
chr28	10370866	2	302	TCCCC:0	T:1		
chr28	10370872	2	302	C:0	CTATG:1		
chr28	10370927	2	268	T:0	TA:1		
chr28	10374078	2	300	C:0.45	T:0.55		
chr28	10377572	2	298	C:0.996644	T:0.0033557		
chr28	10378750	2	276	A:0.847826	G:0.152174		
chr28	10378769	2	276	G:0.851449	A:0.148551		
chr28	10379163	2	250	C:0.872	T:0.128		
chr28	10379774	2	276	G:0.836957	C:0.163043		
chr28	10379827	2	288	T:0.854167	C:0.145833		
chr28	10379987	2	286	A:0.867133	G:0.132867		
chr28	10380811	2	304	C:0.842105	A:0.157895		
chr28	10381643	2	284	A:0.866197	G:0.133803		
chr28	10381703	2	288	C:0.996528	T:0.00347222		

chr28	10381792	2	260	G:0.938462	A:0.0615385
chr28	10381872	2	310	TCC:0.683871	T:0.316129
chr28	10381875	2	310	TCTCCTCTCCTCTCCTCCCCTCCC:0.683871	T:0.316129
chr28	10382353	2	292	C:0.849315	G:0.150685
chr28	10382580	2	290	C:0.565517	T:0.434483
chr28	10383052	2	294	A:0.85034	T:0.14966
chr28	10383887	2	302	A:0.86755	G:0.13245
chr28	10384028	2	298	A:0.85906	G:0.14094
chr28	10384349	2	284	C:0.869718	T:0.130282
chr28	10384687	2	302	T:0.880795	C:0.119205
chr28	10385385	2	294	G:0.0102041	C:0.989796
chr28	10385825	2	300	G:0.863333	A:0.136667
chr28	10385939	2	310	A:0.870968	G:0.129032
chr28	10385971	2	298	G:0.872483	GA:0.127517
chr28	10385974	2	298	G:0.872483	A:0.127517
chr28	10386297	2	286	T:0.867133	C:0.132867
chr28	10386315	2	286	G:0.867133	C:0.132867
chr28	10386328	2	264	A:0.977273	G:0.0227273
chr28	10386501	2	272	G:0.893382	GA:0.106618
chr28	10386609	2	280	A:0.875	T:0.125
chr28	10386908	2	306	C:0.862745	T:0.137255
chr28	10386918	2	306	T:0.862745	A:0.137255
chr28	10387323	2	298	C:0.862416	T:0.137584
chr28	10387401	2	288	G:0.961806	A:0.0381944
chr28	10387594	2	296	T:0.861486	C:0.138514
chr28	10387806	2	258	A:0.856589	G:0.143411
chr28	10388313	2	298	T:0.872483	C:0.127517
chr28	10388466	2	294	TG:0.867347	T:0.132653
chr28	10388628	2	302	T:0.86755	TA:0.13245
chr28	10388841	2	280	A:0.842857	G:0.157143
chr28	10389161	2	198	C:0.868687	T:0.131313
chr28	10389928	2	302	A:0.847682	ATTTCATT:0.152318
chr28	10390280	2	298	C:0.869128	T:0.130872
chr28	10390562	2	274	A:0.90146	C:0.0985401
chr28	10390574	2	280	T:0.914286	C:0.0857143
chr28	10391260	2	258	CA:0	C:1
chr28	10391476	2	292	G:0.866438	C:0.133562
chr28	10391557	2	292	C:0.849315	T:0.150685
chr28	10391721	2	292	G:0.84589	A:0.15411
chr28	10391895	2	298	C:0.865772	T:0.134228
chr28	10392035	2	296	C:0.871622	T:0.128378
chr28	10392085	2	292	G:0.797945	T:0.202055
chr28	10392319	2	290	CG:0	C:1
chr28	10392365	2	292	G:0.859589	A:0.140411
chr28	10392446	2	298	T:0.855705	C:0.144295
chr28	10392796	2	290	T:0.858621	C:0.141379
chr28	10392902	2	282	A:0.893617	G:0.106383
chr28	10393550	2	294	C:0.846939	G:0.153061
chr28	10393652	2	292	G:0.876712	GC:0.123288
chr28	10393861	2	296	C:0.871622	T:0.128378
chr28	10394024	2	292	T:0.84589	C:0.15411
chr28	10394271	2	284	AT:0.862676	A:0.137324
chr28	10394288	2	290	C:0.848276	A:0.151724

chr28	10394868	2	308	G:0.853896	A:0.146104	
chr28	10394921	2	302	G:0.854305	T:0.145695	
chr28	10395152	2	312	G:0.871795	A:0.128205	
chr28	10395369	2	296	G:0.85473	C:0.14527	
chr28	10395401	2	304	T:0.855263	TA:0.144737	
chr28	10395424	2	304	C:0.855263	CA:0.144737	
chr28	10395599	2	294	G:0.853741	T:0.146259	
chr28	10395865	2	290	A:0	T:1	
chr28	10395870	2	298	C:0.848993	T:0.151007	
chr28	10395873	2	290	C:0	A:1	
chr28	10395874	2	290	A:0	T:1	
chr28	10395884	2	298	G:0.848993	A:0.151007	
chr28	10396284	3	280	A:0.282143	AG:0.121429	AAGAG:0.596429
chr28	10396416	2	266	G:0.842105	C:0.157895	
chr28	10396706	2	226	C:0.207965	A:0.792035	
chr28	10396711	2	242	T:0.892562	A:0.107438	
chr28	10396728	2	238	A:0.962185	T:0.0378151	
chr28	10397205	2	294	T:0.880952	C:0.119048	
chr28	10397433	2	302	T:0.86755	C:0.13245	
chr28	10397476	2	296	T:0.986486	G:0.0135135	
chr28	10397487	2	296	G:0.989865	A:0.0101351	
chr28	10397646	2	284	GC:0.876761	G:0.123239	
chr28	10397753	2	304	A:0.845395	C:0.154605	
chr28	10398029	2	308	C:0.863636	T:0.136364	
chr28	10398460	2	304	T:0.851974	C:0.148026	
chr28	10398873	2	298	C:0.875839	T:0.124161	
chr28	10399254	2	300	G:0.87	A:0.13	
chr28	10399272	2	314	A:0.993631	AGGAGGAGCAGG:0.00636943	
chr28	10399274	2	314	CCA:0.993631	C:0.00636943	
chr28	10399850	2	304	C:0.993421	G:0.00657895	
chr28	10400340	2	306	C:0.862745	T:0.137255	
chr28	10400437	2	296	G:0.986486	A:0.0135135	
chr28	10400982	2	296	T:0.871622	C:0.128378	
chr28	10401034	2	288	T:0.878472	C:0.121528	
chr28	10401176	2	304	A:0.858553	T:0.141447	
chr28	10401361	2	298	A:0.865772	G:0.134228	
chr28	10401442	2	296	C:0.868243	T:0.131757	
chr28	10401785	2	296	C:0.983108	T:0.0168919	
chr28	10401793	2	296	G:0.983108	A:0.0168919	
chr28	10402031	2	296	G:0.864865	A:0.135135	
chr28	10402078	2	286	T:0.853147	C:0.146853	
chr28	10402969	2	302	C:0.986755	T:0.013245	
chr28	10403312	2	308	A:0.883117	C:0.116883	
chr28	10403318	2	308	G:0.883117	A:0.116883	
chr28	10403424	2	300	C:0.87	T:0.13	
chr28	10403627	2	288	T:0.864583	G:0.135417	
chr28	10403866	2	284	T:0.0176056	C:0.982394	
chr28	10403925	2	282	A:0.897163	G:0.102837	
chr28	10404228	2	276	A:0.884058	G:0.115942	
chr28	10404455	2	242	G:0	GT:1	
chr28	10404475	2	242	T:0	TA:1	
chr28	10404477	2	242	GA:0	G:1	

chr28	10404483	2	242	AC:0	A:1	
chr28	10404523	2	282	C:0.29078	CT:0.70922	
chr28	10404526	2	282	A:0.29078	G:0.70922	
chr28	10404528	2	282	A:0.29078	G:0.70922	
chr28	10404530	2	282	CG:0.29078	C:0.70922	
chr28	10404532	2	282	CG:0.29078	C:0.70922	
chr28	10404536	2	282	C:0.29078	A:0.70922	
chr28	10404538	2	282	C:0.29078	A:0.70922	
chr28	10404550	2	282	CCCCCCCCCCCTCCCG:0.29078	C:0.70922	
chr28	10404948	2	296	T:0.885135	C:0.114865	
chr28	10405303	2	302	T:0.877483	C:0.122517	
chr28	10405445	2	284	G:0.81338	A:0.18662	
chr28	10405479	2	270	T:0	TG:1	
chr28	10405525	2	280	G:0	GT:1	
chr28	10405526	2	280	A:0	G:1	
chr28	10405552	2	302	A:0	AT:1	
chr28	10405745	2	296	T:0.885135	C:0.114865	
chr28	10405770	2	288	G:0.993056	A:0.00694444	
chr28	10405847	2	286	G:0.867133	A:0.132867	
chr28	10406752	2	304	C:0.914474	T:0.0855263	
chr28	10407069	2	298	G:0.989933	A:0.0100671	
chr28	10407082	2	294	G:0.880952	A:0.119048	
chr28	10407326	2	302	A:0.877483	AT:0.122517	
chr28	10407690	2	296	T:0.871622	C:0.128378	
chr28	10407941	2	302	G:0.990066	A:0.00993377	
chr28	10407944	2	302	A:0.990066	G:0.00993377	
chr28	10408200	2	310	T:0.990323	C:0.00967742	
chr28	10408224	2	310	G:0.990323	A:0.00967742	
chr28	10408225	2	304	C:0.963816	A:0.0361842	
chr28	10408264	2	306	A:0.954248	C:0.0457516	
chr28	10408297	2	308	G:0.996753	A:0.00324675	
chr28	10408306	2	308	C:0.996753	T:0.00324675	
chr28	10409581	2	298	C:0.993289	T:0.00671141	
chr28	10409893	3	288	CCTCT:0.444444	C:0.295139	CCT:0.260417
chr28	10410057	2	300	G:0.88	A:0.12	
chr28	10410061	2	300	T:0.88	C:0.12	
chr28	10410483	2	294	T:0.840136	C:0.159864	
chr28	10410673	2	298	C:0.989933	A:0.0100671	
chr28	10410775	2	296	GACT:0.983108	G:0.0168919	
chr28	10410995	2	308	A:0.99026	T:0.00974026	
chr28	10411027	2	302	G:0.874172	A:0.125828	
chr28	10411592	2	300	C:0.916667	CT:0.0833333	
chr28	10411745	2	306	C:0.986928	T:0.0130719	
chr28	10411760	2	306	A:0.986928	C:0.0130719	
chr28	10411798	2	300	C:0.993333	T:0.00666667	
chr28	10411853	2	302	T:0.990066	A:0.00993377	
chr28	10412125	2	304	T:0.858553	C:0.141447	
chr28	10412330	2	302	A:0.990066	G:0.00993377	
chr28	10412347	2	302	G:0.990066	A:0.00993377	
chr28	10412552	2	300	C:0.996667	T:0.00333333	
chr28	10412857	2	274	T:0.846715	TC:0.153285	
chr28	10413499	2	308	C:0.866883	CG:0.133117	

chr28	10413599	2	302	C:0.903974	A:0.0960265	
chr28	10413685	2	308	A:0.876623	G:0.123377	
chr28	10414238	2	290	C:0.986207	CT:0.0137931	
chr28	10414470	2	288	T:0.871528	C:0.128472	
chr28	10414517	2	284	G:0.869718	A:0.130282	
chr28	10414527	2	284	C:0.869718	T:0.130282	
chr28	10415601	2	294	AAAAAT:0.97619	A:0.0238095	
chr28	10415621	2	298	TAAAATAAATA:0.932886	T:0.0671141	
chr28	10415626	2	314	T:0.863057	TAAATA:0.136943	
chr28	10417377	3	310	CGAGAGAGAGAGA:0.674194	C:0.222581	CGAGAGA:0.103226
chr28	10417383	2	316	A:0.993671	ATC:0.00632911	
chr28	10417945	2	304	G:0.881579	A:0.118421	
chr28	10418020	2	298	C:0.761745	A:0.238255	
chr28	10418409	2	286	C:0.986014	T:0.013986	
chr28	10418813	3	296	CAT:0.547297	C:0.138514	CATAT:0.314189
chr28	10418846	2	298	T:0.875839	A:0.124161	
chr28	10418874	2	308	G:0.886364	GTGTATATA:0.113636	
chr28	10419697	2	300	A:0.856667	C:0.143333	
chr28	10419784	2	290	T:0.775862	TA:0.224138	
chr28	10420261	2	304	T:0.865132	C:0.134868	
chr28	10420334	2	314	T:0.984076	C:0.0159236	
chr28	10420397	2	318	G:0.874214	T:0.125786	
chr28	10420564	2	300	T:0.86	C:0.14	
chr28	10420766	2	312	A:0.858974	G:0.141026	
chr28	10420781	2	312	A:0.858974	G:0.141026	
chr28	10421296	2	306	T:0.872549	G:0.127451	
chr28	10421541	2	296	T:0.864865	C:0.135135	
chr28	10421665	2	284	A:0.862676	G:0.137324	
chr28	10422136	2	296	T:0.837838	TA:0.162162	
chr28	10423036	2	302	A:0.890728	G:0.109272	
chr28	10423544	2	310	C:0.858065	T:0.141935	
chr28	10423594	2	306	G:0.862745	C:0.137255	
chr28	10423598	2	306	A:0.862745	C:0.137255	
chr28	10423967	2	306	C:0.970588	T:0.0294118	
chr28	10424083	2	296	A:0.868243	G:0.131757	
chr28	10424214	2	308	C:0.834416	T:0.165584	
chr28	10424321	2	306	T:0.875817	TG:0.124183	
chr28	10424506	2	310	C:0.870968	T:0.129032	
chr28	10424645	2	298	A:0.872483	C:0.127517	
chr28	10424808	2	290	G:0.872414	A:0.127586	
chr28	10424814	2	290	A:0.872414	G:0.127586	
chr28	10424852	2	286	C:0.846154	CT:0.153846	
chr28	10424875	2	300	A:0.846667	G:0.153333	
chr28	10425041	2	288	CGTGTGT:0.645833	C:0.354167	
chr28	10425511	2	306	G:0.846405	A:0.153595	
chr28	10425606	2	298	T:0.869128	C:0.130872	
chr28	10425839	2	298	T:0.838926	A:0.161074	
chr28	10425952	2	302	C:0.874172	T:0.125828	
chr28	10427022	2	304	GC:0.871711	G:0.128289	
chr28	10427176	2	294	G:0.877551	A:0.122449	

chr28	10427759	2	308	G:0.857143	A:0.142857
chr28	10427979	2	312	C:0.990385	T:0.00961538
chr28	10428209	2	294	C:0.853741	G:0.146259
chr28	10428504	2	300	A:0.866667	G:0.133333
chr28	10428822	2	250	AT:0.7	A:0.3
chr28	10428863	2	284	G:0.859155	A:0.140845
chr28	10429206	2	312	C:0.875	A:0.125
chr28	10429290	2	308	T:0.957792	C:0.0422078
chr28	10429814	2	308	TC:0.876623	T:0.123377
chr28	10430037	2	296	C:0.837838	T:0.162162
chr28	10430172	2	298	C:0.865772	T:0.134228
chr28	10431042	2	286	C:0.937063	CT:0.0629371
chr28	10431233	2	282	G:0.858156	A:0.141844
chr28	10431502	2	296	T:0.861486	C:0.138514
chr28	10432230	2	288	G:0.989583	T:0.0104167
chr28	10432245	2	288	G:0.878472	A:0.121528
chr28	10432448	2	302	T:0.864238	A:0.135762
chr28	10432673	2	310	C:0.729032	T:0.270968
chr28	10433001	2	210	AGAGAGAGGAG:0.942857	A: 0.0571429
chr28	10433005	2	192	AGAGGAG:0.786458	A:0.213542
chr28	10433007	2	198	AGGAG:0.363636	A:0.636364
chr28	10434312	2	306	A:0.879085	G:0.120915
chr28	10436203	2	302	C:0.986755	G:0.013245
chr28	10436522	2	294	G:0.982993	A:0.0170068
chr28	10437820	2	298	C:0.996644	G:0.0033557
chr28	10438297	2	286	G:0.63986	T:0.36014
chr28	10438805	2	314	GCCAGGAC:0.980892	G: 0.0191083
chr28	10441394	2	300	G:0.996667	A:0.00333333
chr28	10441797	2	312	G:0.653846	C:0.346154
chr28	10441989	2	292	G:0.869863	T:0.130137
chr28	10442087	2	298	T:0.85906	A:0.14094
chr28	10442113	2	296	A:0.834459	AG:0.165541
chr28	10442807	2	296	A:0.97973	C:0.0202703
chr28	10443124	2	276	C:0.985507	CCT:0.0144928
chr28	10443155	2	304	TA:0.986842	T:0.0131579
chr28	10443174	2	304	A:0.986842	AAAATTT:0.0131579
chr28	10443485	2	298	A:0.986577	T:0.0134228
chr28	10443497	2	298	TA:0.986577	T:0.0134228
chr28	10443670	2	302	T:0.980132	TA:0.0198675
chr28	10443950	2	276	T:0.945652	TAA:0.0543478
chr28	10443955	2	266	A:0.43985	AAT:0.56015
chr28	10444272	2	306	A:0.98366	G:0.0163399
chr28	10444470	2	308	A:0.977273	T:0.0227273
chr28	10444772	2	304	C:0.996711	T:0.00328947
chr28	10445301	2	288	C:0.989583	T:0.0104167
chr28	10445305	2	290	T:0.996552	C:0.00344828
chr28	10445326	2	298	C:0.986577	G:0.0134228
chr28	10445406	2	294	C:0.996599	A:0.00340136
chr28	10445550	2	308	C:0.977273	A:0.0227273
chr28	10445640	2	298	C:0.989933	T:0.0100671
chr28	10446428	2	298	C:0.989933	T:0.0100671
chr28	10446700	2	298	T:0.969799	C:0.0302013

chr28	10446807	2	302	T:0.980132	C:0.0198675	
chr28	10446829	2	294	G:0.993197	A:0.00680272	
chr28	10446843	2	296	G:0.986486	A:0.0135135	
chr28	10447462	2	308	T:0.967532	C:0.0324675	
chr28	10447683	2	292	C:0.982877	T:0.0171233	
chr28	10447739	2	278	G:0.780576	GT:0.219424	
chr28	10447794	2	266	C:0.815789	CAG:0.184211	
chr28	10448124	2	292	A:0.976027	G:0.0239726	
chr28	10448364	2	302	A:0.983444	G:0.0165563	
chr28	10449013	2	312	G:0.987179	A:0.0128205	
chr28	10449165	2	304	T:0.986842	C:0.0131579	
chr28	10449174	2	304	G:0.986842	A:0.0131579	
chr28	10449975	2	300	C:0.983333	T:0.0166667	
chr28	10449977	2	300	A:0.983333	C:0.0166667	
chr28	10450144	2	298	A:0.986577	T:0.0134228	
chr28	10450153	3	300	TA:0.88	T:0.0133333	TAA:0.106667
chr28	10450155	2	298	A:0.986577	T:0.0134228	
chr28	10451487	2	316	TCA:0.800633	T:0.199367	
chr28	10451760	2	300	C:0.756667	T:0.243333	
chr28	10453124	2	304	G:0.720395	T:0.279605	
chr28	10453220	2	296	C:0.273649	T:0.726351	
chr28	10453221	2	296	T:0.273649	G:0.726351	
chr28	10453340	2	300	G:0.25	A:0.75	
chr28	10453397	2	296	G:0.996622	A:0.00337838	
chr28	10453654	2	296	T:0.253378	C:0.746622	
chr28	10454031	2	314	C:0.235669	T:0.764331	
chr28	10454118	2	298	G:0.993289	A:0.00671141	
chr28	10455483	2	300	G:0.99	A:0.01	
chr28	10455514	2	290	T:0.989655	C:0.0103448	
chr28	10456454	2	302	T:0.00331126	C:0.996689	
chr28	10456632	2	298	C:0.989933	T:0.0100671	
chr28	10457104	2	304	AT:0.0394737	A:0.960526	
chr28	10457244	2	308	A:0.993506	T:0.00649351	
chr28	10457252	2	308	C:0.993506	T:0.00649351	
chr28	10457765	2	300	T:0.873333	G:0.126667	
chr28	10458109	2	296	G:0.962838	A:0.0371622	
chr28	10458290	2	284	G:0.00352113	A:0.996479	
chr28	10458604	2	288	A:0.0104167	G:0.989583	
chr28	10458825	2	302	A:0.00993377	C:0.990066	
chr28	10459013	2	308	G:0.993506	A:0.00649351	
chr28	10460830	2	304	T:0.986842	C:0.0131579	
chr28	10460841	2	308	A:0.99026	G:0.00974026	
chr28	10461036	2	298	A:0.996644	G:0.0033557	
chr28	10461926	2	312	A:0.990385	G:0.00961538	
chr28	10462118	2	302	A:0.00993377	C:0.990066	
chr28	10462141	2	310	C:0.993548	T:0.00645161	
chr28	10462648	2	294	C:0.993197	T:0.00680272	
chr28	10462900	2	300	T:0.986667	C:0.0133333	
chr28	10463060	2	288	A:0.982639	G:0.0173611	
chr28	10463186	2	290	T:0.00689655	C:0.993103	
chr28	10463187	2	290	G:0.00689655	A:0.993103	
chr28	10463400	2	296	AT:0.962838	A:0.0371622	
chr28	10463475	2	308	CGGCTTT:0.012987	C:0.987013	

chr28	10463618	2	290	C:0.00689655	T:0.993103
chr28	10463649	2	300	A:0.00666667	G:0.993333
chr28	10463654	2	300	A:0.00666667	C:0.993333
chr28	10464467	2	286	CT:0.00699301	C:0.993007
chr28	10465467	2	296	G:0.0439189	A:0.956081
chr28	10466178	2	286	A:0	T:1
chr28	10466668	2	302	G:0.00993377	A:0.990066
chr28	10467048	2	264	C:0.848485	CT:0.151515
chr28	10468099	2	300	C:0.01	T:0.99
chr28	10468140	2	298	C:0.0100671	T:0.989933
chr28	10468190	2	292	A:0.010274	T:0.989726
chr28	10468601	2	286	A:0.0104895	C:0.98951
chr28	10468818	2	298	C:0.989933	T:0.0100671
chr28	10469513	2	300	GT:0.993333	G:0.00666667
chr28	10469933	2	308	T:0.99026	C:0.00974026
chr28	10470363	2	302	G:0.960265	A:0.0397351
chr28	10470454	2	300	C:0.993333	T:0.00666667
chr28	10471019	2	306	T:0.986928	C:0.0130719
chr28	10471128	2	304	A:0.983553	G:0.0164474
chr28	10471268	2	304	G:0.00657895	T:0.993421
chr28	10471749	2	308	G:0.993506	C:0.00649351
chr28	10472372	2	288	G:0.0173611	C:0.982639
chr28	10472382	2	288	C:0.0173611	T:0.982639
chr28	10472405	2	284	A:0.0105634	G:0.989437
chr28	10472496	2	302	T:0.00662252	C:0.993377
chr28	10472721	2	306	G:0.954248	A:0.0457516
chr28	10473124	2	302	C:0.966887	T:0.0331126
chr28	10473220	2	300	C:0.99	A:0.01
chr28	10473702	2	304	A:0.940789	G:0.0592105
chr28	10474306	2	304	G:0.957237	T:0.0427632
chr28	10474709	2	308	C:0.00974026	G:0.99026
chr28	10474800	2	306	A:0.00653595	G:0.993464
chr28	10475240	2	306	CAAAT:0.0653595	C:0.934641
chr28	10476277	2	292	A:0.0205479	AT:0.979452
chr28	10476460	2	298	CAG:0.885906	C:0.114094
chr28	10477100	2	302	G:0.00662252	C:0.993377
chr28	10477151	2	306	G:0.00653595	A:0.993464
chr28	10477238	2	304	T:0.953947	A:0.0460526
chr28	10477792	2	306	TG:0.00653595	T:0.993464
chr28	10477931	2	296	G:0.00675676	A:0.993243
chr28	10478437	2	286	AT:0.0034965	A:0.996503
chr28	10478679	2	274	G:0.0693431	A:0.930657
chr28	10478862	2	294	A:0.00680272	G:0.993197
chr28	10479039	2	306	C:0.993464	T:0.00653595
chr28	10479052	2	306	T:0.993464	C:0.00653595
chr28	10479655	2	308	C:0.993506	A:0.00649351
chr28	10479729	2	314	TACATACATATACAC:0.0573248	T: 0.942675
chr28	10480199	2	296	T:0.878378	A:0.121622
chr28	10480614	2	294	C:0.945578	A:0.0544218
chr28	10480617	2	296	A:0.959459	T:0.0405405
chr28	10480627	2	300	C:0.93	T:0.07
chr28	10480657	2	298	A:0.916107	C:0.0838926
chr28	10480753	2	302	CAAA:0.950331	C:0.0496689

chr28	10480930	2	304	G:0.0526316	A:0.947368
chr28	10481065	2	300	T:0.906667	C:0.0933333
chr28	10481291	2	296	G:0.983108	A:0.0168919
chr28	10481321	2	300	G:0.986667	A:0.0133333
chr28	10481705	2	302	G:0.937086	A:0.0629139
chr28	10481790	2	300	G:0.946667	A:0.0533333
chr28	10482555	2	284	T:0.957746	A:0.0422535
chr28	10482662	2	306	A:0.96732	G:0.0326797
chr28	10482683	2	308	C:0.961039	CAGTT:0.038961
chr28	10482915	2	306	A:0.954248	C:0.0457516
chr28	10483086	2	284	A:0.954225	G:0.0457746
chr28	10483165	2	310	G:0.967742	A:0.0322581
chr28	10484059	2	292	A:0.0410959	T:0.958904
chr28	10484244	2	266	C:0.913534	G:0.0864662
chr28	10484531	2	314	AGAGGATGGATAGGTGAAG:0.920382	A:0.0796178
chr28	10484723	2	298	A:0.949664	G:0.0503356
chr28	10484729	2	298	C:0.949664	T:0.0503356
chr28	10484756	2	296	A:0.945946	AC:0.0540541
chr28	10484941	2	298	A:0.956376	G:0.0436242
chr28	10485235	2	290	C:0.996552	T:0.00344828
chr28	10485354	2	302	G:0.0596026	A:0.940397
chr28	10485410	2	298	G:0.989933	A:0.0100671
chr28	10485591	2	294	A:0.945578	G:0.0544218
chr28	10485726	2	290	T:0.962069	C:0.037931
chr28	10485753	2	286	T:0.909091	C:0.0909091
chr28	10486174	2	306	A:0.0980392	G:0.901961
chr28	10486240	2	314	C:0.0700637	CTAAA:0.929936
chr28	10486311	2	304	T:0.914474	A:0.0855263
chr28	10486438	2	296	C:0.114865	A:0.885135
chr28	10486495	2	310	C:0.0612903	G:0.93871
chr28	10487737	2	294	A:0.047619	G:0.952381
chr28	10487775	2	300	G:0.103333	A:0.896667
chr28	10488156	2	308	T:0.957792	C:0.0422078
chr28	10488182	2	300	T:0.993333	C:0.00666667
chr28	10488230	2	302	G:0.0529801	A:0.94702
chr28	10488231	2	302	T:0.0529801	C:0.94702
chr28	10488467	2	300	C:0.966667	CT:0.0333333
chr28	10488606	2	308	T:0.935065	C:0.0649351
chr28	10488873	2	302	AC:0.960265	A:0.0397351
chr28	10488986	2	306	G:0.964052	A:0.0359477
chr28	10489224	2	288	A:0.913194	G:0.0868056
chr28	10489255	2	292	A:0.0993151	C:0.900685
chr28	10489259	2	292	A:0.0993151	G:0.900685
chr28	10489306	2	300	C:0.103333	CCA:0.896667
chr28	10489446	2	304	CA:0.894737	C:0.105263
chr28	10489453	2	304	G:0.894737	C:0.105263
chr28	10489887	2	310	G:0.0516129	T:0.948387
chr28	10489978	2	302	T:0.0529801	G:0.94702
chr28	10490118	2	308	T:0.0584416	C:0.941558
chr28	10490313	2	310	A:0.993548	T:0.00645161
chr28	10490316	2	306	G:0.0457516	C:0.954248
chr28	10490690	2	292	G:0.955479	A:0.0445205
chr28	10490875	2	302	G:0.0430464	A:0.956954

chr28	10490928	2	300	GAC:0.923333	G:0.0766667	
chr28	10490959	2	304	G:0.125	A:0.875	
chr28	10490970	2	310	T:0.929032	A:0.0709677	
chr28	10491278	2	312	G:0.0512821	A:0.948718	
chr28	10491324	2	310	C:0.0419355	T:0.958065	
chr28	10491898	2	304	T:0.930921	C:0.0690789	
chr28	10492084	2	278	A:0.0467626	G:0.953237	
chr28	10492096	2	284	G:0.954225	A:0.0457746	
chr28	10492234	2	296	T:0.945946	A:0.0540541	
chr28	10492251	2	296	A:0.945946	T:0.0540541	
chr28	10492788	2	306	C:0.993464	T:0.00653595	
chr28	10493120	2	300	C:0.0533333	T:0.946667	
chr28	10493336	2	306	T:0.993464	C:0.00653595	
chr28	10493467	2	310	G:0.958065	C:0.0419355	
chr28	10493542	2	300	G:0.953333	GC:0.0466667	
chr28	10493971	3	294	CTT:0.159864	C:0.115646	CT:0.72449
chr28	10494096	2	290	G:0.927586	C:0.0724138	
chr28	10494328	2	304	CA:0.993421	C:0.00657895	
chr28	10494341	2	302	G:0.874172	A:0.125828	
chr28	10494349	2	302	A:0.0463576	G:0.953642	
chr28	10494830	2	296	T:0.0506757	C:0.949324	
chr28	10494838	2	296	T:0.0506757	C:0.949324	
chr28	10494914	2	276	T:0.0652174	C:0.934783	
chr28	10495072	2	300	C:0.92	T:0.08	
chr28	10495147	2	282	C:0.996454	T:0.0035461	
chr28	10495571	2	302	G:0.996689	T:0.00331126	
chr28	10495874	2	304	C:0.907895	T:0.0921053	
chr28	10495926	2	300	A:0.906667	G:0.0933333	
chr28	10496120	2	316	TAAGAAGAGGAAAAGAAAAAAAAAAAA:0.901899	T:0.0981013	
chr28	10496286	2	296	A:0.0608108	G:0.939189	
chr28	10496348	2	300	G:0.14	T:0.86	
chr28	10496390	2	304	A:0.914474	G:0.0855263	
chr28	10496416	2	308	A:0.905844	G:0.0941558	
chr28	10496501	2	302	T:0.927152	C:0.0728477	
chr28	10496502	2	302	G:0.927152	A:0.0728477	
chr28	10496557	2	282	G:0.925532	A:0.0744681	
chr28	10496630	2	292	C:0.934932	T:0.0650685	
chr28	10496764	2	302	G:0.238411	A:0.761589	
chr28	10496770	2	302	G:0.910596	C:0.089404	
chr28	10496875	2	296	A:0.0405405	C:0.959459	
chr28	10497044	2	296	T:0.918919	C:0.0810811	
chr28	10497105	2	300	AAGG:0.13	A:0.87	
chr28	10497318	2	220	A:0.181818	G:0.818182	
chr28	10497383	2	232	C:0.189655	A:0.810345	
chr28	10497468	2	278	G:0.881295	A:0.118705	
chr28	10497554	2	278	G:0.899281	A:0.100719	
chr28	10497558	2	278	C:0.899281	T:0.100719	
chr28	10497565	2	278	C:0.899281	A:0.100719	
chr28	10497589	2	292	A:0	C:1	
chr28	10497592	2	292	G:0	C:1	
chr28	10497660	2	304	G:0.914474	A:0.0855263	
chr28	10497693	2	300	A:0	AC:1	

chr28 10497725 2 306 A:0.859477 C:0.140523
chr28 10497773 2 296 A:0.875 G:0.125
chr28 10498021 2 294 T:0.112245 C:0.887755
chr28 10498078 2 298 A:0.130872 G:0.869128
chr28 10498147 4 302 GCACACA:0.0860927 G:
0.788079 GCA:0.0496689 GCACA:0.0761589
chr28 10498448 2 302 G:0.92053 A:0.0794702
chr28 10498511 2 302 T:0.943709 G:0.0562914
chr28 10498541 2 316 AGGTCCTGTCTGGTAACAGGACCT:0.882911
A:0.117089
chr28 10498710 2 316 C:0.920886 T:0.0791139
chr28 10498723 2 316 T:0.920886 C:0.0791139
chr28 10499073 2 302 C:0.0463576 CA:0.953642
chr28 10499137 2 292 C:0.952055 T:0.0479452
chr28 10499219 2 286 TC:0.944056 T:0.0559441
chr28 10499282 2 286 TA:0.678322 T:0.321678
chr28 10499359 2 304 G:0.901316 C:0.0986842
chr28 10499416 2 296 A:0.932432 T:0.0675676
chr28 10499501 2 312 CA:0.932692 C:0.0673077
chr28 10499571 2 306 G:0.928105 A:0.0718954
chr28 10499708 2 300 T:0.923333 C:0.0766667
chr28 10499713 2 302 T:0.930464 C:0.0695364
chr28 10499878 2 294 A:0.037415 G:0.962585
chr28 10500007 2 294 T:0.921769 C:0.0782313
chr28 10500100 3 302 T:0.903974 TA:0.0331126
TAA:0.0629139
chr28 10500406 2 302 C:0.927152 T:0.0728477
chr28 10500445 2 302 C:0.92053 T:0.0794702
chr28 10500483 2 296 G:0.929054 A:0.0709459
chr28 10500524 2 296 G:0.925676 A:0.0743243
chr28 10500529 2 296 G:0.925676 A:0.0743243
chr28 10500978 2 260 A:0.880769 G:0.119231
chr28 10501240 2 296 A:0.918919 G:0.0810811
chr28 10501364 2 290 G:0.913793 C:0.0862069
chr28 10501382 2 294 A:0.914966 G:0.085034
chr28 10501385 2 294 A:0.914966 G:0.085034
chr28 10501432 2 298 T:0.909396 C:0.090604
chr28 10501443 2 304 A:0.805921 G:0.194079
chr28 10501454 2 306 G:0.934641 A:0.0653595
chr28 10501493 2 306 G:0.977124 C:0.0228758
chr28 10501534 2 302 C:0.927152 T:0.0728477
chr28 10501548 2 300 C:0.953333 T:0.0466667
chr28 10501639 2 306 G:0.901961 A:0.0980392
chr28 10501640 2 306 T:0.901961 G:0.0980392
chr28 10501691 2 314 A:0.898089 G:0.101911
chr28 10501816 2 306 T:0.888889 C:0.111111
chr28 10501919 2 296 T:0.881757 G:0.118243
chr28 10502181 2 302 A:0.940397 C:0.0596026
chr28 10502226 2 278 A:0.910072 G:0.0899281
chr28 10502518 2 290 A:0.786207 G:0.213793
chr28 10502522 2 288 G:0.940972 A:0.0590278
chr28 10502601 2 306 T:0.95098 C:0.0490196
chr28 10502730 2 302 C:0.0430464 G:0.956954
chr28 10502893 2 294 T:0.918367 C:0.0816327

chr28	10503172	2	294	G:0.986395	A:0.0136054	
chr28	10503199	2	302	C:0.983444	T:0.0165563	
chr28	10503801	2	262	CTTTTCTT:0.255725	C:0.744275	
chr28	10503808	2	302	T:0.990066	TTTTTC:0.00993377	
chr28	10503843	2	276	CTTT:0.98913	C:0.0108696	
chr28	10504846	2	288	G:0.986111	T:0.0138889	
chr28	10505578	2	292	TGA:0.979452	T:0.0205479	
chr28	10505851	2	282	CT:0.858156	C:0.141844	
chr28	10506237	2	292	A:0.979452	C:0.0205479	
chr28	10506576	2	314	G:0.984076	A:0.0159236	
chr28	10506774	2	304	T:0.118421	C:0.881579	
chr28	10506846	2	308	T:0.974026	C:0.025974	
chr28	10506862	2	308	C:0.974026	CCTTG:0.025974	
chr28	10506923	2	292	A:0.986301	G:0.0136986	
chr28	10507097	2	282	T:0.975177	C:0.0248227	
chr28	10507576	2	282	C:0.975177	T:0.0248227	
chr28	10507885	2	300	C:0.99	G:0.01	
chr28	10507946	2	256	T:0.976562	C:0.0234375	
chr28	10507957	2	172	C:0.203488	CT:0.796512	
chr28	10508091	2	290	C:0.975862	T:0.0241379	
chr28	10508159	3	278	TAA:0.705036	T:0.0971223	TA:0.197842
chr28	10508191	2	244	C:0.877049	CT:0.122951	
chr28	10508347	2	300	A:0.886667	G:0.113333	
chr28	10508357	2	300	T:0.886667	C:0.113333	
chr28	10508629	2	296	C:0.925676	T:0.0743243	
chr28	10509066	2	304	G:0.105263	T:0.894737	
chr28	10511315	2	312	T:0.108974	C:0.891026	
chr28	10512059	2	296	G:0.885135	A:0.114865	
chr28	10512115	2	314	T:0.904459	TTCTCTCTC:0.0955414	
chr28	10512616	2	308	G:0.737013	GT:0.262987	
chr28	10512620	2	310	T:0.964516	G:0.0354839	
chr28	10512669	2	302	T:0.92053	C:0.0794702	
chr28	10512749	2	302	T:0.900662	C:0.0993377	
chr28	10513085	2	298	A:0.124161	G:0.875839	
chr28	10513322	2	304	C:0.878289	T:0.121711	
chr28	10513530	2	304	G:0.917763	T:0.0822368	
chr28	10514199	2	298	C:0.0838926	T:0.916107	
chr28	10514412	2	296	A:0.0777027	G:0.922297	
chr28	10514583	2	306	C:0.0980392	T:0.901961	
chr28	10515232	2	298	A:0.114094	G:0.885906	
chr28	10515282	2	298	T:0.104027	A:0.895973	
chr28	10515351	3	284	A:0.90493	T:0.0387324	AT:0.056338
chr28	10515445	2	298	G:0.979866	A:0.0201342	
chr28	10516428	2	302	T:0.923841	G:0.0761589	
chr28	10516548	2	292	T:0.917808	C:0.0821918	
chr28	10517160	2	296	A:0.0506757	G:0.949324	
chr28	10517313	2	306	T:0.147059	C:0.852941	
chr28	10517345	2	306	A:0.147059	G:0.852941	
chr28	10517435	2	180	C:0.3	T:0.7	
chr28	10517439	2	180	T:0.3	TCC:0.7	

chr28	10517453	2	264	T:0.92803	TCA:0.0719697		
chr28	10517690	2	300	G:0.216667	A:0.783333		
chr28	10517787	2	300	C:0.183333	T:0.816667		
chr28	10517820	2	290	C:0.224138	T:0.775862		
chr28	10518033	2	298	T:0.949664	C:0.0503356		
chr28	10518080	2	294	G:0.962585	GT:0.037415		
chr28	10518081	3	298	G:0.174497	T:0.0369128	GTT:0.788591	
chr28	10518269	2	282	G:0.968085	A:0.0319149		
chr28	10518280	2	276	C:0.974638	G:0.0253623		
chr28	10518281	2	280	C:0.910714	T:0.0892857		
chr28	10518374	2	296	G:0.952703	C:0.0472973		
chr28	10518411	2	272	G:0.959559	C:0.0404412		
chr28	10518497	2	232	CG:0.866379	C:0.133621		
chr28	10519342	2	154	AG:0	A:1		
chr28	10519353	2	146	TC:0	T:1		
chr28	10519366	2	132	TC:0	T:1		
chr28	10519388	2	132	CT:0	C:1		
chr28	10519430	2	134	CT:0	C:1		
chr28	10519534	2	182	A:0.28022	G:0.71978		
chr28	10519546	2	182	A:0.28022	ACC:0.71978		
chr28	10519548	2	182	A:0.28022	G:0.71978		
chr28	10519654	2	252	G:0.920635	C:0.0793651		
chr28	10519675	2	254	G:0.956693	C:0.0433071		
chr28	10519739	2	232	T:0.599138	C:0.400862		
chr28	10519864	2	228	G:0.627193	A:0.372807		
chr28	10520051	2	130	AG:0.646154	A:0.353846		
chr28	10520150	2	286	A:0.975524	G:0.0244755		
chr28	10520305	2	268	C:0.973881	G:0.0261194		
chr28	10520332	2	288	AG:0.6875	A:0.3125		
chr28	10520668	2	302	G:0.698675	A:0.301325		
chr28	10520890	2	292	C:0.979452	CG:0.0205479		
chr28	10520935	2	294	C:0.72449	T:0.27551		
chr28	10521102	2	308	A:0.633117	G:0.366883		
chr28	10521620	2	254	C:0.948819	G:0.0511811		
chr28	10522208	2	290	C:0.658621	A:0.341379		
chr28	10522366	2	308	C:0.948052	T:0.0519481		
chr28	10522633	2	312	A:0.657051	G:0.342949		
chr28	10522956	2	284	A:0.661972	AT:0.338028		
chr28	10522983	2	290	T:0.986207	G:0.0137931		
chr28	10523071	2	286	A:0.65035	G:0.34965		
chr28	10523174	2	292	G:0.965753	A:0.0342466		
chr28	10523305	2	292	A:0.945205	G:0.0547945		
chr28	10523777	2	302	A:0.665563	G:0.334437		
chr28	10523822	2	300	G:0.976667	A:0.0233333		
chr28	10524256	2	310	A:0.7	G:0.3		
chr28	10524518	2	308	T:0.720779	G:0.279221		
chr28	10524932	2	300	T:0.696667	C:0.303333		
chr28	10525567	2	304	G:0.756579	A:0.243421		
chr28	10525601	2	304	T:0.934211	A:0.0657895		
chr28	10525681	2	306	C:0.673203	T:0.326797		
chr28	10525713	2	290	A:0.975862	AT:0.0241379		
chr28	10525755	4	316	CAGAGAGAGAG:0.120253	C:0.0886076	CAGAGAG:0.329114	CAGAGAGAG:0.462025

chr28 10525997 2 302 A:0.682119 C:0.317881
chr28 10526376 5 318 GAATAAATA:0.566038 G:
0.179245 GAATA:0.0566038 GAATAAATAAATA:0.154088
GAATAAATAAATAAATAAATA:0.0440252
chr28 10526519 2 290 A:0.682759 G:0.317241
chr28 10527997 2 312 G:0.733974 T:0.266026
chr28 10528186 2 298 GA:0.949664 G:0.0503356
chr28 10528530 2 302 A:0.983444 AT:0.0165563
chr28 10528700 2 282 G:0.737589 C:0.262411
chr28 10528814 2 308 C:0.746753 T:0.253247
chr28 10528977 2 316 C:0.974684 G:0.0253165
chr28 10529218 2 314 T:0.671975 C:0.328025
chr28 10529380 2 300 C:0.673333 G:0.326667
chr28 10529384 2 302 C:0.754967 A:0.245033
chr28 10529489 3 288 TA:0.576389 T:0.302083
TAA:0.121528
chr28 10529526 2 304 C:0.694079 CTCTG:0.305921
chr28 10530570 2 302 G:0.983444 A:0.0165563
chr28 10531210 2 308 A:0.980519 G:0.0194805
chr28 10531444 2 300 TG:0.98 T:0.02
chr28 10531787 2 304 CA:0.950658 C:0.0493421
chr28 10531942 2 304 G:0.947368 A:0.0526316
chr28 10532071 2 302 G:0.748344 A:0.251656
chr28 10532897 2 220 C:0.7 T:0.3
chr28 10533054 2 270 G:0.7 C:0.3
chr28 10533354 2 308 TAGAGG:0.977273 T:0.0227273
chr28 10533459 2 302 G:0.718543 T:0.281457
chr28 10533652 2 288 G:0.982639 A:0.0173611
chr28 10533958 2 290 C:0.962069 T:0.037931
chr28 10534843 2 302 G:0.953642 A:0.0463576
chr28 10534893 2 306 T:0.960784 C:0.0392157
chr28 10535021 3 306 GA:0.843137 G:0.0849673
GAA:0.0718954
chr28 10535315 2 312 AC:0.983974 A:0.0160256
chr28 10535331 2 308 G:0.983766 T:0.0162338
chr28 10535811 2 298 C:0.728188 T:0.271812
chr28 10535898 2 306 T:0.928105 G:0.0718954
chr28 10535958 2 304 G:0.934211 A:0.0657895
chr28 10536054 2 300 C:0.93 G:0.07
chr28 10536332 2 298 C:0.936242 CAG:0.0637584
chr28 10536420 2 306 A:0.650327 G:0.349673
chr28 10536976 2 308 A:0.977273 G:0.0227273
chr28 10537452 2 312 C:0.676282 CTGAG:0.323718
chr28 10537507 2 296 T:0.939189 TTG:0.0608108
chr28 10537895 3 304 CTGTGTGTG:0.286184 C:
0.453947 CTGTG:0.259868
chr28 10538001 3 296 T:0.682432 TA:0.246622
TAA:0.0709459
chr28 10538258 2 292 T:0.674658 C:0.325342
chr28 10538270 2 294 CA:0.778912 C:0.221088
chr28 10538288 2 292 C:0.934932 T:0.0650685
chr28 10538316 2 296 C:0.939189 T:0.0608108
chr28 10538331 2 296 A:0.939189 T:0.0608108
chr28 10538333 2 296 G:0.939189 A:0.0608108

chr28	10538383	3	312	C:0.692308	CCT:0.0608974	CCTCTCTCT:0.246795	
chr28	10538390	3	298	ATC:0.52349	A:0.154362	CTC:0.322148	
chr28	10538436	2	296	A:0.719595	T:0.280405		
chr28	10538437	2	298	A:0.959732	T:0.0402685		
chr28	10538626	2	290	C:0.827586	T:0.172414		
chr28	10538656	2	306	G:0.862745	A:0.137255		
chr28	10538661	2	314	G:0.920382	A:0.0796178		
chr28	10538951	4	318	TTTTA:0.606918	T:0.194969	TTTTATTTA:0.169811	TTTTATTTATTTA:0.0283019
chr28	10538997	2	292	CAGAG:0.671233	C:0.328767		
chr28	10539277	2	306	G:0.915033	A:0.0849673		
chr28	10539510	2	298	C:0.912752	T:0.0872483		
chr28	10539517	2	298	G:0.761745	A:0.238255		
chr28	10539547	2	294	C:0.972789	T:0.0272109		
chr28	10539548	2	292	T:0.958904	A:0.0410959		
chr28	10539557	2	298	G:0.936242	T:0.0637584		
chr28	10539625	2	306	G:0.735294	C:0.264706		
chr28	10539801	2	302	C:0.976821	T:0.0231788		
chr28	10539911	2	304	T:0.6875	C:0.3125		
chr28	10540342	2	306	G:0.732026	A:0.267974		
chr28	10540382	2	300	T:0.943333	C:0.0566667		
chr28	10540621	2	308	A:0.974026	G:0.025974		
chr28	10540628	2	306	T:0.696078	A:0.303922		
chr28	10540871	2	302	T:0.943709	A:0.0562914		
chr28	10540884	2	290	A:0.755172	AT:0.244828		
chr28	10540929	2	246	C:0.869919	CAG:0.130081		
chr28	10541015	2	270	CG:0.974074	C:0.0259259		
chr28	10541076	2	302	A:0	C:1		
chr28	10541096	2	302	T:0	G:1		
chr28	10541101	2	302	T:0	A:1		
chr28	10541103	2	302	T:0	A:1		
chr28	10541118	2	302	T:0	A:1		
chr28	10541125	2	302	A:0	C:1		
chr28	10541264	2	146	CCT:0.376712	C:0.623288		
chr28	10541327	2	246	T:0.101626	TA:0.898374		
chr28	10541417	2	282	A:0.929078	G:0.070922		
chr28	10541554	2	246	G:0.939024	A:0.0609756		
chr28	10541916	2	302	C:0.966887	T:0.0331126		
chr28	10541958	2	306	G:0.728758	A:0.271242		
chr28	10541965	2	306	C:0.728758	T:0.271242		
chr28	10542313	2	296	ACT:0.665541	A:0.334459		
chr28	10542400	2	306	C:0.928105	T:0.0718954		
chr28	10542690	2	304	CG:0.950658	C:0.0493421		
chr28	10542732	2	298	G:0.973154	A:0.0268456		
chr28	10543285	2	304	A:0.947368	G:0.0526316		
chr28	10544144	2	310	GATTA:0.954839	G:0.0451613		
chr28	10544407	2	282	GC:0.489362	G:0.510638		
chr28	10544578	2	296	A:0.962838	G:0.0371622		
chr28	10544598	2	292	C:0.965753	A:0.0342466		
chr28	10544748	2	298	C:0.97651	T:0.0234899		
chr28	10544902	2	304	C:0.0657895	T:0.934211		
chr28	10544947	2	312	G:0.958333	A:0.0416667		

chr28 10545316 2 302 A:0.470199 G:0.529801
chr28 10545752 2 292 G:0.958904 A:0.0410959
chr28 10545882 2 304 T:0.980263 C:0.0197368
chr28 10545941 2 296 T:0.949324 C:0.0506757
chr28 10546062 3 244 GC:0.704918 G:0.221311
GCC:0.0737705
chr28 10546246 2 286 G:0.958042 A:0.041958
chr28 10546274 2 288 AT:0.96875 A:0.03125
chr28 10546470 2 288 T:0.0451389 G:0.954861
chr28 10546482 2 290 ATC:0.982759 A:0.0172414
chr28 10546594 2 236 C:0.949153 CCG:0.0508475
chr28 10546605 2 236 C:0.949153 T:0.0508475
chr28 10546670 2 288 A:0.927083 G:0.0729167
chr28 10546749 2 276 G:0.913043 GT:0.0869565
chr28 10546809 3 292 C:0.934932 G:0.0136986
T:0.0513699
chr28 10546810 2 292 A:0.934932 G:0.0650685
chr28 10547311 2 286 C:0.947552 CG:0.0524476
chr28 10547549 2 308 T:0.977273 C:0.0227273
chr28 10547730 2 292 C:0.979452 T:0.0205479
chr28 10548151 2 296 C:0.337838 G:0.662162
chr28 10548254 2 246 AAG:0.386179 A:0.613821
chr28 10548327 2 212 AAAAGAAAG:0.726415 A:
0.273585
chr28 10548437 2 280 G:0.403571 GAAGAAAGA:
0.596429
chr28 10548616 2 300 T:0.956667 C:0.0433333
chr28 10548914 2 298 G:0.95302 A:0.0469799
chr28 10550186 2 310 T:0.654839 C:0.345161
chr28 10550235 2 300 C:0.956667 T:0.0433333
chr28 10550733 2 234 TA:0.606838 T:0.393162
chr28 10550976 2 304 ATC:0.970395 A:0.0296053
chr28 10551173 2 278 C:0.830935 G:0.169065
chr28 10551192 2 298 A:0.946309 AT:0.0536913
chr28 10551203 2 264 TA:0.814394 T:0.185606
chr28 10551273 2 302 T:0.715232 TGA:0.284768
chr28 10551427 2 274 G:0.656934 A:0.343066
chr28 10551575 3 304 CTAAA:0.671053 C:0.0394737
CTAAATAAA:0.289474
chr28 10551612 3 304 T:0.680921 A:0.0690789
TAAAA:0.25
chr28 10551999 2 292 G:0.688356 A:0.311644
chr28 10552082 2 300 G:0.65 C:0.35
chr28 10552149 2 314 T:0.955414 TAATC:0.044586
chr28 10552741 2 310 CTT:0.66129 C:0.33871
chr28 10552743 2 310 T:0.66129 TACAC:0.33871
chr28 10553306 2 308 G:0.977273 A:0.0227273
chr28 10553549 2 314 G:0.984076 C:0.0159236
chr28 10554159 2 302 C:0.990066 T:0.00993377
chr28 10555699 4 306 CAG:0.683007 C:0.0882353
CAGAG:0.120915 CAGAGAGAG:0.107843
chr28 10556187 2 284 CCAGT:0.697183 C:0.302817
chr28 10556286 2 258 C:0.813953 CATAT:0.186047
chr28 10556306 2 190 TAC:0.936842 T:0.0631579

chr28	10556310	2	240	T:0.958333	TAC:0.0416667	
chr28	10557662	2	310	AAAC:0.983871	A:0.016129	
chr28	10559647	2	310	CGCCACCAT:0.63871	C:0.36129	
chr28	10560511	2	286	T:0.660839	TA:0.339161	
chr28	10561385	2	300	G:0.99	A:0.01	
chr28	10561624	2	272	G:0.944853	A:0.0551471	
chr28	10561781	2	138	T:0.485507	TA:0.514493	
chr28	10561958	2	86	GAGGA:0.732558	G:0.267442	
chr28	10562746	3	298	CCT:0.483221	C:0.318792	CCTCT:0.197987
chr28	10562865	2	300	C:0.81	G:0.19	
chr28	10563588	2	288	C:0.934028	T:0.0659722	
chr28	10563736	2	314	G:0.697452	A:0.302548	
chr28	10563944	2	290	CCTCT:0.613793	C:0.386207	
chr28	10563995	2	288	TA:0.704861	T:0.295139	
chr28	10563996	2	304	A:0.957237	T:0.0427632	
chr28	10565183	2	298	C:0.852349	T:0.147651	
chr28	10565409	2	282	TA:0.535461	T:0.464539	
chr28	10565818	2	312	T:0.875	G:0.125	
chr28	10566177	2	302	GT:0.615894	G:0.384106	
chr28	10566240	2	308	GGGAAGAAA:0.711039	G:0.288961	
chr28	10566893	2	296	T:0.655405	C:0.344595	
chr28	10567203	2	312	G:0.961538	T:0.0384615	
chr28	10567759	2	310	ACAGGCTAACAACTG:0.612903	A:0.387097	
chr28	10568250	2	290	T:0.955172	TA:0.0448276	
chr28	10568591	2	304	G:0.990132	A:0.00986842	
chr28	10568623	2	304	A:0.628289	G:0.371711	
chr28	10569503	2	294	A:0.666667	G:0.333333	
chr28	10569890	2	294	A:0.70068	T:0.29932	
chr28	10569998	2	300	CT:0.966667	C:0.0333333	
chr28	10571008	2	302	T:0.639073	C:0.360927	
chr28	10571845	2	310	T:0.667742	C:0.332258	
chr28	10572371	2	304	A:0.894737	T:0.105263	
chr28	10573035	2	308	C:0.688312	CAAGAA:0.311688	
chr28	10573367	2	314	T:0.643312	C:0.356688	
chr28	10573672	2	304	C:0.671053	A:0.328947	
chr28	10574227	2	302	G:0.970199	A:0.0298013	
chr28	10574663	2	294	G:0.615646	A:0.384354	
chr28	10574717	2	282	C:0.712766	G:0.287234	
chr28	10574739	2	310	A:0.945161	AT:0.0548387	
chr28	10574749	2	310	A:0.945161	AT:0.0548387	
chr28	10574760	2	310	A:0.945161	AG:0.0548387	
chr28	10575007	2	302	A:0.629139	G:0.370861	
chr28	10575338	2	286	C:0.451049	CT:0.548951	
chr28	10575340	2	298	T:0.889262	C:0.110738	
chr28	10575737	2	280	G:0.967857	A:0.0321429	
chr28	10576071	3	284	T:0.334507	A:0.31338	TA:0.352113
chr28	10576074	2	284	A:0.68662	AT:0.31338	
chr28	10576100	2	302	A:0.996689	T:0.00331126	
chr28	10576262	2	238	C:0.680672	CT:0.319328	

chr28	10576306	2	294	C:0.955782	CAGAG:0.0442177	
chr28	10576365	2	296	C:0.969595	T:0.0304054	
chr28	10576729	2	282	C:0.836879	T:0.163121	
chr28	10576852	2	308	T:0.646104	C:0.353896	
chr28	10576884	2	288	C:0.704861	CA:0.295139	
chr28	10576892	3	298	A:0.936242	T:0.0234899	AT:0.0402685
chr28	10576911	2	300	AAAAT:0.95	A:0.05	
chr28	10576941	2	294	G:0.969388	A:0.0306122	
chr28	10577125	2	272	T:0.856618	TA:0.143382	
chr28	10577126	2	278	T:0.705036	A:0.294964	
chr28	10577379	2	290	G:0.958621	A:0.0413793	
chr28	10577506	2	256	TA:0.585938	T:0.414062	
chr28	10577805	3	298	C:0.708054	CCT:0.265101	CCTCT:0.0268456
chr28	10577855	2	310	AAAATAAAT:0.63871	A:0.36129	
chr28	10578996	2	310	T:0.645161	TAAC:0.354839	
chr28	10579062	2	310	AAG:0.658065	A:0.341935	
chr28	10580462	2	286	C:0.947552	CT:0.0524476	
chr28	10580464	2	280	TA:0.925	T:0.075	
chr28	10581386	2	294	GA:0.639456	G:0.360544	
chr28	10581895	2	302	C:0.917219	T:0.0827815	
chr28	10581982	2	308	A:0.996753	T:0.00324675	
chr28	10582211	2	306	T:0.95098	C:0.0490196	
chr28	10582988	2	304	C:0.960526	T:0.0394737	
chr28	10583824	3	302	CA:0.695364	C:0.109272	CAAA:0.195364
chr28	10584077	2	290	C:0.713793	A:0.286207	
chr28	10584152	2	296	CCT:0.601351	C:0.398649	
chr28	10584919	2	304	A:0.628289	G:0.371711	
chr28	10585831	2	320	AT:0.8	A:0.2	
chr28	10586056	2	304	T:0.858553	A:0.141447	
chr28	10586531	2	286	T:0.646853	C:0.353147	
chr28	10586537	2	286	G:0.954545	A:0.0454545	
chr28	10587009	2	312	A:0.913462	AAATG:0.0865385	
chr28	10588186	2	298	C:0.473154	CA:0.526846	
chr28	10588731	2	304	C:0.615132	T:0.384868	
chr28	10589102	2	298	T:0.818792	TA:0.181208	
chr28	10590953	2	290	A:0.665517	G:0.334483	
chr28	10591396	2	298	G:0.869128	GA:0.130872	
chr28	10591455	2	306	T:0.964052	A:0.0359477	
chr28	10591516	2	258	TG:0.713178	T:0.286822	
chr28	10592814	2	288	TA:0.625	T:0.375	
chr28	10592845	2	302	A:0.649007	G:0.350993	
chr28	10593145	2	290	CA:0.855172	C:0.144828	
chr28	10594082	2	304	TA:0.976974	T:0.0230263	
chr28	10594538	2	294	G:0.608844	GA:0.391156	
chr28	10594595	2	304	T:0.631579	C:0.368421	
chr28	10594959	2	306	T:0.823529	G:0.176471	
chr28	10595189	2	306	T:0.633987	A:0.366013	
chr28	10595758	3	310	G:0.793548	GAATA:0.164516	GAATAAATA:0.0419355
chr28	10596022	3	250	CTT:0.62	C:0.196	CT:0.184

chr28	10596068 2	292	T:0.643836	TAAAG:0.356164
chr28	10596279 2	316	CCTCTCTCTCTCT:0.503165	C:
0.496835
chr28	10596340 2	290	TA:0.931034	T:0.0689655
chr28	10596511 2	308	T:0.944805	G:0.0551948
chr28	10596926 2	292	C:0.976027	T:0.0239726
chr28	10596936 2	290	G:0.951724	A:0.0482759
chr28	10598903 2	290	A:0.655172	G:0.344828
chr28	10599477 2	286	G:0.884615	A:0.115385
chr28	10600160 2	300	TTTG:0.673333	T:0.326667
chr28	10600172 2	304	G:0.967105	T:0.0328947
chr28	10600191 2	300	C:0.973333	CAGAG:0.0266667
chr28	10600289 2	292	A:0.657534	G:0.342466
chr28	10600456 2	308	AT:0.62987	A:0.37013
chr28	10600860 2	308	A:0.623377	C:0.376623
chr28	10601025 2	290	A:0.644828	G:0.355172
chr28	10601393 2	302	C:0.960265	T:0.0397351
chr28	10601547 3	298	TA:0.45302	T:0.238255
AA:0.308725
chr28	10601552 2	300	A:0.873333	T:0.126667
chr28	10601895 2	294	C:0.615646	T:0.384354
chr28	10602230 2	280	A:0.628571	G:0.371429
chr28	10602417 2	286	A:0.601399	G:0.398601
chr28	10602631 2	300	A:0	T:1
chr28	10602632 2	300	A:0	T:1
chr28	10604178 2	270	A:0.974074	T:0.0259259
chr28	10604330 2	300	TA:0.27	T:0.73
chr28	10606620 2	304	T:0.713816	A:0.286184
chr28	10607017 2	258	C:0.790698	CCT:0.209302
chr28	10607070 2	296	T:0.635135	TA:0.364865
chr28	10607627 2	304	A:0.664474	AGGCTCCT:0.335526
chr28	10608406 2	308	A:0.516234	AGATT:0.483766
chr28	10609257 2	308	T:0.970779	TA:0.0292208
chr28	10609343 2	308	T:0.99026	C:0.00974026
chr28	10609455 2	296	G:0.64527	T:0.35473
chr28	10609610 2	312	C:0.621795	T:0.378205
chr28	10610000 2	316
CTTAGGATGTTTCAGTTTCTTTTTTTTTTTTTTTTTTTTTTTTTTTTTTTTTTTTTTTTTTTTTTTTTT
TT:0	C:1
chr28	10610363 2	290	CT:0.906897	C:0.0931034
chr28	10610502 2	290	G:0.682759	A:0.317241
chr28	10610893 2	300	G:0.966667	C:0.0333333
chr28	10611159 2	298	T:0.922819	C:0.0771812
chr28	10612139 2	192	C:0.947917	T:0.0520833
chr28	10612291 2	132	T:0.560606	TA:0.439394
chr28	10612743 2	304	C:0.953947	T:0.0460526
chr28	10612925 2	298	A:0.865772	G:0.134228
chr28	10612928 2	298	A:0.644295	G:0.355705
chr28	10613580 2	284	T:0.647887	C:0.352113
chr28	10613752 2	280	CCT:0.603571	C:0.396429
chr28	10613808 2	300	AT:0.66	A:0.34
chr28	10613812 2	302	T:0.980132	A:0.0198675
chr28	10614272 2	240	A:0.979167	C:0.0208333
chr28	10614821 2	294	G:0.622449	T:0.377551

chr28	10615196	3	294	ATT:0.639456	A:0.0510204
AT:0.309524
chr28	10615512	2	304	T:0.694079	G:0.305921
chr28	10617162	4	316	GATTTATTT:0.398734	G:
0.189873	GATTT:0.193038	GATTTATTTATTT:0.218354
chr28	10617438	2	290	G:0.937931	A:0.062069
chr28	10617682	2	288	C:0.635417	CA:0.364583
chr28	10617699	2	288	C:0	T:1
chr28	10617780	2	300	G:0	A:1
chr28	10617826	2	300	G:0	A:1
chr28	10617832	2	300	CT:0	C:1
chr28	10617839	2	300	T:0	A:1
chr28	10617854	2	298	C:0	A:1
chr28	10617869	2	294	C:0	A:1
chr28	10617903	2	312	T:0	TA:1
chr28	10617918	2	312	A:0	T:1
chr28	10617919	2	312	T:0	A:1
chr28	10617927	2	312	A:0	AT:1
chr28	10617929	2	312	A:0	T:1
chr28	10617963	2	312	TG:0	T:1
chr28	10617977	2	312	GA:0	G:1
chr28	10618000	2	312	G:0	A:1
chr28	10618010	2	312	A:0	AC:1
chr28	10618031	2	312	T:0	C:1
chr28	10618032	2	312	T:0	C:1
chr28	10618246	2	128	CTA:0.851562	C:0.148438
chr28	10618248	2	128	A:0.15625	C:0.84375
chr28	10618872	2	300	T:0.98	G:0.02
chr28	10618890	2	300	A:0.87	G:0.13
chr28	10619117	2	300	A:0.946667	T:0.0533333
chr28	10619145	3	304	CAGAGAG:0.578947	C:0.0953947
CAG:0.325658
chr28	10619482	2	286	A:0.646853	G:0.353147
chr28	10619486	2	286	C:0.646853	CAG:0.353147
chr28	10619721	2	296	GTGGC:0.716216	G:0.283784
chr28	10619871	2	260	GCT:0.980769	G:0.0192308
chr28	10621164	2	288	G:0.444444	T:0.555556
chr28	10621953	2	226	C:0.402655	T:0.597345
chr28	10621964	2	244	A:0.389344	G:0.610656
chr28	10622622	2	316	GC:0.617089	G:0.382911
chr28	10622643	2	316	G:0.617089	GTGA:0.382911
chr28	10622647	2	316	TAGG:0.617089	T:0.382911
chr28	10622771	2	316	T:0.920886	TAAA:0.0791139
chr28	10622878	2	302	G:0.705298	A:0.294702
chr28	10622883	2	302	T:0.705298	C:0.294702
chr28	10622935	2	286	T:0.671329	G:0.328671
chr28	10623376	2	312	C:0.958333	T:0.0416667
chr28	10624357	2	282	G:0.620567	T:0.379433
chr28	10624425	2	294	C:0.853741	A:0.146259
chr28	10625989	2	310	T:0.532258	A:0.467742
chr28	10626000	2	308	T:0.525974	C:0.474026
chr28	10626353	2	300	A:0.88	T:0.12
chr28	10626516	2	304	A:0.565789	G:0.434211
chr28	10627065	2	292	T:0.996575	C:0.00342466

chr28	10627402	2	304	A:0.401316	G:0.598684		
chr28	10627470	2	308	A:0.594156	G:0.405844		
chr28	10627479	2	308	A:0.594156	C:0.405844		
chr28	10627673	2	298	A:0.630872	G:0.369128		
chr28	10627837	2	300	G:0.353333	A:0.646667		
chr28	10628053	4	294	TAA:0.272109	T:0.105442	TA:0.482993	TAAA:0.139456
chr28	10628054	2	300	A:0.983333	C:0.0166667		
chr28	10628554	2	302	T:0.397351	C:0.602649		
chr28	10628556	2	302	C:0.397351	G:0.602649		
chr28	10628630	2	292	A:0.965753	G:0.0342466		
chr28	10628686	2	300	C:0.343333	T:0.656667		
chr28	10629369	2	300	A:0.626667	G:0.373333		
chr28	10629607	2	296	C:0.989865	T:0.0101351		
chr28	10629969	2	318	GGAGAGAGAGAGAGAGA:0.584906	G:0.415094		
chr28	10630008	2	318	CAGAGA:0.584906	C:0.415094		
chr28	10630016	2	318	C:0.584906	CT:0.415094		
chr28	10630026	2	318	GAGAAGCAGGCTCCATGCAGGGA:0.584906	G:0.415094		
chr28	10630251	2	308	T:0.795455	C:0.204545		
chr28	10630452	2	292	G:0.880137	T:0.119863		
chr28	10630515	2	300	C:0.38	T:0.62		
chr28	10630780	2	302	T:0.380795	G:0.619205		
chr28	10630830	2	286	A:0.688811	AT:0.311189		
chr28	10630953	2	286	G:0.646853	T:0.353147		
chr28	10630963	2	284	C:0.908451	T:0.0915493		
chr28	10631066	2	298	T:0	C:1		
chr28	10631096	2	308	C:0	T:1		
chr28	10631098	2	308	G:0	T:1		
chr28	10631733	2	310	T:0.0806452	C:0.919355		
chr28	10631854	2	308	C:0.188312	A:0.811688		
chr28	10632105	2	290	A:0.389655	G:0.610345		
chr28	10632233	2	304	T:0.940789	G:0.0592105		
chr28	10632242	2	304	A:0.355263	G:0.644737		
chr28	10632248	2	306	T:0.973856	C:0.0261438		
chr28	10632600	2	306	T:0.973856	C:0.0261438		
chr28	10632617	2	298	A:0.734899	G:0.265101		
chr28	10632699	2	284	T:0.640845	C:0.359155		
chr28	10632728	2	280	G:0.867857	C:0.132143		
chr28	10632911	2	242	AT:0.789256	A:0.210744		
chr28	10633163	2	278	G:0	C:1		
chr28	10633165	2	278	C:0	A:1		
chr28	10633202	2	302	ATGTC:0.970199	A:0.0298013		
chr28	10633728	2	286	A:0.884615	AT:0.115385		
chr28	10633784	2	292	A:0.859589	G:0.140411		
chr28	10633815	2	300	A:0.86	T:0.14		
chr28	10633825	2	300	G:0.86	C:0.14		
chr28	10633919	2	298	T:0.275168	A:0.724832		
chr28	10633945	2	310	TA:0.867742	T:0.132258		
chr28	10634021	2	294	C:0.887755	CTCCA:0.112245		
chr28	10634257	2	312	TTAAAAAAAAAAA:0.907051	T:0.0929487		
chr28	10634258	3	312	TAAAAAAAA:0.913462	TAAAA:		

0.0416667 T:0.0448718
chr28 10634262 2 312 AAAAAAAAT:0.964744 A:
0.0352564
chr28 10634264 2 318 A:0.805031 AATATATTC:
0.194969
chr28 10634266 3 316 A:0.731013 T:0.237342
AAAAT:0.0316456
chr28 10634268 2 314 A:0.773885 T:0.226115
chr28 10634269 2 302 AT:0.970199 A:0.0298013
chr28 10634270 3 318 T:0.779874 TTC:0.160377
TTCAA:0.0597484
chr28 10634271 2 308 AAATAAAT:0.970779 A:
0.0292208
chr28 10634272 3 318 AAT:0.808176 TAT:0.0314465
A:0.160377
chr28 10634274 2 308 TAAA:0.977273 T:0.0227273
chr28 10634279 2 308 A:0.977273 C:0.0227273
chr28 10634281 2 308 ATAAAT:0.977273 A:0.0227273
chr28 10634487 2 300 A:0.41 G:0.59
chr28 10634607 2 296 AAAG:0.956081 A:0.0439189
chr28 10634747 2 280 C:0.375 CA:0.625
chr28 10634817 2 284 G:0.383803 C:0.616197
chr28 10634840 2 282 T:0.365248 C:0.634752
chr28 10635490 2 196 C:0.923469 A:0.0765306
chr28 10636541 2 208 T:0.0913462 G:0.908654
chr28 10636814 2 284 C:0.848592 CCT:0.151408
chr28 10637002 2 302 A:0.109272 G:0.890728
chr28 10637235 2 274 TG:0.90146 T:0.0985401
chr28 10637237 2 274 G:0.959854 T:0.040146
chr28 10638364 2 294 G:0.969388 GT:0.0306122
chr28 10638365 2 294 G:0.969388 T:0.0306122
chr28 10638401 2 306 G:0.722222 A:0.277778
chr28 10638429 2 308 C:0.970779 T:0.0292208
chr28 10638694 2 306 G:0.823529 A:0.176471
chr28 10638697 2 306 T:0.849673 C:0.150327
chr28 10638806 2 314 T:0.547771 C:0.452229
chr28 10638899 2 298 T:0.580537 A:0.419463
chr28 10639109 2 296 A:0.912162 G:0.0878378
chr28 10639164 2 306 T:0.120915 C:0.879085
chr28 10639509 2 298 GT:0.966443 G:0.033557
chr28 10639510 3 302 T:0.877483 TGG:0.0728477
TGGG:0.0496689
chr28 10639511 2 294 GT:0.748299 G:0.251701
chr28 10639512 2 294 T:0.806122 G:0.193878
chr28 10639567 3 278 G:0.741007 GA:0.226619
GAA:0.0323741
chr28 10639837 2 292 C:0.414384 T:0.585616
chr28 10639951 3 304 C:0.506579 CCT:0.319079
CCTCTCT:0.174342
chr28 10640000 2 290 AT:0.972414 A:0.0275862
chr28 10640010 2 274 A:0.70438 T:0.29562
chr28 10640011 2 274 T:0.70438 A:0.29562
chr28 10640012 2 290 A:0.972414 T:0.0275862
chr28 10640013 2 290 A:0.972414 T:0.0275862

chr28	10640083	2	296	G:0	C:1		
chr28	10640091	2	296	G:0	C:1		
chr28	10640200	2	304	T:0.973684	A:0.0263158		
chr28	10640201	2	296	T:0.763514	A:0.236486		
chr28	10640230	2	284	C:0.78169	CAG:0.21831		
chr28	10640250	2	282	G:0.407801	GA:0.592199		
chr28	10640251	2	282	G:0.407801	GA:0.592199		
chr28	10640316	2	280	C:0.139286	G:0.860714		
chr28	10640330	2	292	C:0.969178	T:0.0308219		
chr28	10640350	2	282	C:0.326241	T:0.673759		
chr28	10640363	2	288	C:0.972222	T:0.0277778		
chr28	10640400	2	292	G:0.181507	GA:0.818493		
chr28	10640523	3	306	CTTT:0.00326797	C:0.869281	CTT:0.127451	
chr28	10640567	2	298	T:0.409396	A:0.590604		
chr28	10640568	2	298	A:0.97651	T:0.0234899		
chr28	10640569	2	298	A:0.97651	T:0.0234899		
chr28	10641158	2	294	A:0.870748	C:0.129252		
chr28	10641161	2	292	GA:0.0273973	G:0.972603		
chr28	10641648	2	300	T:0.48	G:0.52		
chr28	10642984	2	298	G:0.473154	A:0.526846		
chr28	10643363	2	228	C:0.201754	G:0.798246		
chr28	10643973	2	312	T:0.830128	C:0.169872		
chr28	10644364	2	292	C:0.64726	T:0.35274		
chr28	10644502	2	284	T:0.556338	C:0.443662		
chr28	10644704	2	282	G:0.907801	A:0.0921986		
chr28	10644856	2	306	C:0.69281	CCTCT:0.30719		
chr28	10644871	2	292	CTGTG:0.85274	C:0.14726		
chr28	10644873	2	306	G:0.69281	C:0.30719		
chr28	10644911	2	302	TAAAA:0.539735	T:0.460265		
chr28	10644941	2	302	T:0.13245	C:0.86755		
chr28	10645306	2	292	T:0.633562	G:0.366438		
chr28	10645376	2	302	C:0.870861	T:0.129139		
chr28	10645404	2	304	G:0.661184	C:0.338816		
chr28	10645750	2	284	C:0.84507	T:0.15493		
chr28	10645875	2	310	A:0.851613	AT:0.148387		
chr28	10645877	2	308	A:0.743506	T:0.256494		
chr28	10645886	2	312	AAAAATAAAAT:0.836538	A:0.163462		
chr28	10645933	2	308	A:0.847403	T:0.152597		
chr28	10646181	2	304	C:0.858553	T:0.141447		
chr28	10646700	2	296	C:0.560811	A:0.439189		
chr28	10646902	2	294	G:0.598639	A:0.401361		
chr28	10646921	2	296	T:0.881757	C:0.118243		
chr28	10646983	2	306	G:0.565359	C:0.434641		
chr28	10647487	2	304	G:0.625	GT:0.375		
chr28	10647679	2	296	C:0.567568	T:0.432432		
chr28	10647999	2	298	G:0.989933	A:0.0100671		
chr28	10648322	4	180	TGGCGGCGGCGGCGGC:0.438889	T:0.366667	TGGCGGC:0.0888889	TGGCGGCGGCGGC:0.105556
chr28	10648328	2	164	C:0.97561	T:0.0243902		
chr28	10648331	2	162	C:0.882716	T:0.117284		
chr28	10648334	2	160	C:0.975	T:0.025		
chr28	10648337	2	128	C:0.734375	T:0.265625		

chr28	10650449	2	302	GAC:0.798013	G:0.201987	
chr28	10650524	2	292	G:0.982877	A:0.0171233	
chr28	10651103	2	260	GT:0.653846	G:0.346154	
chr28	10651360	2	316	T:0.860759	TTTGATGCGTCTG:0.139241	
chr28	10653423	3	276	TTC:0.565217	T:0.358696	TTCTC:0.076087
chr28	10654846	2	312	C:0.977564	CCTCT:0.0224359	
chr28	10654982	2	306	C:0.660131	T:0.339869	
chr28	10655254	2	310	C:0.990323	A:0.00967742	
chr28	10658845	2	300	G:0.976667	A:0.0233333	
chr28	10660157	2	266	G:0.796992	GT:0.203008	
chr28	10660205	2	290	A:0.865517	G:0.134483	
chr28	10660330	2	296	C:0.638514	T:0.361486	
chr28	10660506	2	294	G:0.727891	GTC:0.272109	
chr28	10660516	3	296	C:0.888514	G:0.0608108	CTG:0.0506757
chr28	10660985	2	298	TA:0.92953	T:0.0704698	
chr28	10661219	2	236	C:0.817797	CT:0.182203	
chr28	10663314	3	274	CT:0.591241	C:0.120438	CTT:0.288321
chr28	10664419	2	286	C:0.870629	CT:0.129371	
chr28	10664431	2	290	A:0.924138	AT:0.0758621	
chr28	10664460	2	292	C:0.640411	CAG:0.359589	
chr28	10664886	2	306	C:0.866013	T:0.133987	
chr28	10666011	2	306	A:0.960784	T:0.0392157	
chr28	10667082	2	312	G:0.983974	A:0.0160256	
chr28	10668639	3	242	GAAA:0.53719	G:0.161157	GA:0.301653
chr28	10668690	2	218	TC:0.5	T:0.5	
chr28	10670738	2	302	C:0.887417	T:0.112583	
chr28	10673042	2	296	T:0.945946	G:0.0540541	
chr28	10674395	2	302	C:0.950331	T:0.0496689	
chr28	10674646	2	290	G:0.989655	A:0.0103448	
chr28	10675012	2	292	A:0.952055	G:0.0479452	
chr28	10675344	2	304	G:0.970395	A:0.0296053	
chr28	10675556	2	310	T:0.987097	C:0.0129032	
chr28	10676284	2	304	A:0.914474	G:0.0855263	
chr28	10676356	2	294	C:0.969388	A:0.0306122	
chr28	10676947	2	316	TCTGTTTTTGCC:0.924051	T:0.0759494	
chr28	10677728	2	278	C:0.964029	T:0.0359712	
chr28	10677902	2	306	T:0.647059	C:0.352941	
chr28	10682717	2	296	A:0.969595	AC:0.0304054	
chr28	10685522	2	292	C:0.671233	T:0.328767	
chr28	10686538	2	300	G:0.996667	A:0.00333333	
chr28	10689002	2	316	CTATCATT:0	C:1	
chr28	10689011	2	316	CAACACCAGAAATACTTGTACAAGCTT:0	C:1	
chr28	10689041	2	316	CAAAAG:0	C:1	
chr28	10689052	2	316	TGTAGTCTGTCTAC:0	T:1	
chr28	10689067	2	316	AAATTTCTGACATCAG:0	A:1	
chr28	10689084	2	316	CCCAAAGGAGAA:0	C:1	
chr28	10689096	2	316	T:0	TTG:1	

chr28	10690263	2	300	C:0.996667	T:0.00333333
chr28	10690732	2	296	T:0.918919	A:0.0810811
chr28	10691590	2	286	C:0.968531	A:0.0314685
chr28	10692902	2	312	A:0.628205	G:0.371795
chr28	10692987	2	298	T:0.654362	C:0.345638
chr28	10693211	2	242	G:0.632231	GA:0.367769
chr28	10694061	2	306	T:0.647059	C:0.352941
chr28	10694176	2	304	G:0.838816	A:0.161184
chr28	10694780	2	280	T:0.610714	C:0.389286
chr28	10694786	2	284	C:0.961268	T:0.0387324
chr28	10696593	2	304	G:0.638158	A:0.361842
chr28	10696929	2	308	T:0.983766	C:0.0162338
chr28	10696974	2	300	C:0.99	T:0.01
chr28	10697088	2	304	C:0.967105	T:0.0328947
chr28	10698154	2	276	G:0.626812	A:0.373188
chr28	10698493	2	284	C:0.489437	CT:0.510563
chr28	10699540	2	298	CCTCT:0.92953	C:0.0704698
chr28	10699735	2	294	A:0.629252	G:0.370748
chr28	10699869	2	312	T:0.628205	C:0.371795
chr28	10700188	2	302	T:0.645695	A:0.354305
chr28	10700658	2	294	G:0.622449	T:0.377551
chr28	10701197	2	280	AG:0.685714	A:0.314286
chr28	10701214	2	290	A:0.689655	G:0.310345
chr28	10701258	2	300	A:0.99	G:0.01
chr28	10701341	2	290	T:0.672414	A:0.327586
chr28	10701625	2	296	A:0.655405	G:0.344595
chr28	10702509	2	312	CTTTCTTTTCT:0.660256	C: 0.339744
chr28	10702592	2	304	G:0.651316	GACAC:0.348684
chr28	10702654	2	288	T:0.621528	C:0.378472
chr28	10703393	2	306	A:0.627451	G:0.372549
chr28	10703679	2	302	C:0.956954	T:0.0430464
chr28	10703918	2	308	C:0.987013	T:0.012987
chr28	10704612	2	302	CT:0.612583	C:0.387417
chr28	10705081	2	292	G:0.660959	A:0.339041
chr28	10705509	2	306	A:0.640523	T:0.359477
chr28	10705881	2	310	T:0.880645	TA:0.119355
chr28	10706231	2	302	A:0.65894	AT:0.34106
chr28	10706893	2	310	C:0.664516	T:0.335484
chr28	10707391	2	302	C:0.649007	CAGAG:0.350993
chr28	10707514	2	286	T:0.632867	C:0.367133
chr28	10707996	2	302	G:0.678808	C:0.321192
chr28	10708030	2	302	G:0.668874	GA:0.331126
chr28	10708233	2	298	C:0.667785	T:0.332215
chr28	10708260	2	310	T:0.658065	G:0.341935
chr28	10708964	2	308	A:0.655844	G:0.344156
chr28	10709326	2	300	A:0.673333	G:0.326667
chr28	10709441	2	300	C:0.676667	T:0.323333
chr28	10710218	2	238	ATT:0.621849	A:0.378151
chr28	10710293	2	292	A:0.650685	T:0.349315
chr28	10710621	2	282	A:0	G:1
chr28	10710791	2	298	GAC:0.701342	G:0.298658
chr28	10711014	2	310	T:0.677419	C:0.322581
chr28	10711234	3	272	AT:0.591912	A:0.0919118

ATT:0.316176

chr28	10711887	2	300	C:0.636667	T:0.363333	
chr28	10712016	2	314	T:0.678344	C:0.321656	
chr28	10712106	2	306	T:0.977124	G:0.0228758	
chr28	10712617	2	288	AT:0.232639	A:0.767361	
chr28	10712647	2	272	C:0.6875	CAG:0.3125	
chr28	10713398	2	300	G:0.656667	A:0.343333	
chr28	10713453	2	290	A:0.982759	G:0.0172414	
chr28	10713526	2	276	G:0.981884	C:0.0181159	
chr28	10713788	2	304	ATCACC:0.674342	A:0.325658	
chr28	10713889	2	304	T:0.664474	A:0.335526	
chr28	10714028	2	308	T:0.941558	C:0.0584416	
chr28	10714302	2	300	T:0.643333	G:0.356667	
chr28	10714438	2	314	C:0.656051	T:0.343949	
chr28	10715462	2	290	C:0.668966	A:0.331034	
chr28	10716192	2	200	C:0.685	CT:0.315	
chr28	10716452	2	252	T:0.630952	C:0.369048	
chr28	10716626	3	260	T:0.626923	TA:0.180769	TAA:0.192308
chr28	10717050	2	302	G:0.953642	A:0.0463576	
chr28	10717141	2	288	C:0.979167	A:0.0208333	
chr28	10717190	3	292	GA:0.613014	G:0.0856164	GAA:0.30137
chr28	10717334	2	288	C:0.659722	G:0.340278	
chr28	10717644	2	296	T:0.155405	G:0.844595	
chr28	10717691	2	294	A:0.989796	G:0.0102041	
chr28	10717743	2	270	TA:0.677778	T:0.322222	
chr28	10717873	2	288	T:0.677083	C:0.322917	
chr28	10719409	2	288	G:0.722222	A:0.277778	
chr28	10719846	2	280	G:0.996429	T:0.00357143	
chr28	10719852	2	264	TC:0.674242	T:0.325758	
chr28	10719870	2	276	A:0.869565	C:0.130435	
chr28	10721042	2	286	T:0.727273	C:0.272727	
chr28	10721098	2	294	T:0.945578	C:0.0544218	
chr28	10722029	2	312	AAGC:0.958333	A:0.0416667	
chr28	10722105	2	278	C:0.636691	CTT:0.363309	
chr28	10722278	2	306	A:0.689542	G:0.310458	
chr28	10722378	2	308	T:0.720779	G:0.279221	
chr28	10722820	2	304	G:0.680921	T:0.319079	
chr28	10723336	2	308	A:0.665584	T:0.334416	
chr28	10723380	2	302	G:0.65894	A:0.34106	
chr28	10723459	2	172	ATT:0.901163	A:0.0988372	
chr28	10723462	2	180	T:0.955556	TA:0.0444444	
chr28	10723470	2	186	T:0.983871	A:0.016129	
chr28	10723992	2	310	CCTCTCT:0.693548	C:0.306452	
chr28	10724046	2	308	AG:0.75974	A:0.24026	
chr28	10724099	2	310	A:0.664516	G:0.335484	
chr28	10724369	2	312	A:0.679487	C:0.320513	
chr28	10724901	2	306	A:0.683007	G:0.316993	
chr28	10725057	2	302	CT:0.738411	C:0.261589	
chr28	10725067	2	284	TTTTTTTTC:0.774648	T: 0.225352	
chr28	10725068	2	284	TTTTTTTC:0.901408	T: 0.0985915	

chr28 10725642 3 278 CTTT:0.557554 C:0.338129
CTTTT:0.104317
chr28 10725688 2 270 CAG:0.655556 C:0.344444
chr28 10725754 2 292 G:0.986301 A:0.0136986
chr28 10726592 2 312 T:0.679487 C:0.320513
chr28 10727319 2 264 CA:0.69697 C:0.30303
chr28 10727335 2 264 G:0.655303 GTA:0.344697
chr28 10727645 2 312 C:0.689103 A:0.310897
chr28 10728488 2 308 G:0.672078 A:0.327922
chr28 10728500 2 292 GT:0.715753 G:0.284247
chr28 10730049 2 308 C:0.681818 T:0.318182
chr28 10730584 2 302 A:0.68543 C:0.31457
chr28 10731280 2 316 T:0.689873 C:0.310127
chr28 10731398 2 304 A:0.684211 G:0.315789
chr28 10732073 2 300 C:0.673333 T:0.326667
chr28 10732241 2 304 T:0.684211 TAAATC:0.315789
chr28 10732717 2 292 G:0.678082 GA:0.321918
chr28 10733060 2 300 A:0.596667 T:0.403333
chr28 10733105 2 308 A:0.675325 G:0.324675
chr28 10733286 2 268 A:0.80597 G:0.19403
chr28 10733508 2 302 C:0.678808 T:0.321192
chr28 10733562 2 230 CTT:0.7 C:0.3
chr28 10733613 2 208 C:0.706731 CAG:0.293269
chr28 10733971 2 290 AT:0.775862 A:0.224138
chr28 10733972 2 288 T:0.913194 A:0.0868056
chr28 10733977 2 292 A:0.688356 T:0.311644
chr28 10734120 2 244 A:0.704918 G:0.295082
chr28 10734164 2 266 A:0.725564 G:0.274436
chr28 10734177 2 278 T:0.78777 TA:0.21223
chr28 10734403 2 294 AAAGGC:0.938776 A:0.0612245
chr28 10734434 2 286 G:0.972028 A:0.027972
chr28 10734596 2 294 G:0.663265 A:0.336735
chr28 10735011 2 300 T:0.686667 C:0.313333
chr28 10735131 2 310 G:0.674194 A:0.325806
chr28 10735165 2 300 CT:0.693333 C:0.306667
chr28 10735204 3 306 C:0.764706 CAG:0.0392157
CAGAG:0.196078
chr28 10735206 3 306 C:0.640523 G:0.29085
CAGAG:0.0686275
chr28 10735781 2 294 A:0.92517 T:0.0748299
chr28 10736087 2 306 C:0.96732 T:0.0326797
chr28 10737303 2 312 G:0.987179 C:0.0128205
chr28 10737520 2 314 A:0.652866 G:0.347134
chr28 10739199 2 276 G:0.880435 A:0.119565
chr28 10739353 2 286 G:0.870629 A:0.129371
chr28 10739553 3 282 AG:0.797872 A:0.134752
AGG:0.0673759
chr28 10739597 2 268 C:0.776119 T:0.223881
chr28 10740077 2 306 C:0.872549 T:0.127451
chr28 10740145 2 296 C:0.915541 CA:0.0844595
chr28 10740147 2 280 A:0.539286 AT:0.460714
chr28 10740404 3 298 C:0.734899 CT:0.147651
CTT:0.11745
chr28 10740445 3 306 CAGAGAG:0.683007 C:0.160131

CAGAG:0.156863					
chr28	10740947	2	306	T:0.875817	C:0.124183
chr28	10741145	2	310	T:0.964516	G:0.0354839
chr28	10741571	2	294	T:0.884354	C:0.115646
chr28	10741822	2	312	G:0.980769	A:0.0192308
chr28	10742324	2	308	T:0.876623	C:0.123377
chr28	10742707	2	300	C:0.89 CT:0.11	
chr28	10742710	2	298	T:0.895973	TAA:0.104027
chr28	10743089	2	298	C:0.942953	T:0.057047
chr28	10743465	2	298	C:0.969799	T:0.0302013
chr28	10744059	2	302	G:0.884106	C:0.115894
chr28	10744615	2	304	T:0.773026	C:0.226974
chr28	10744908	2	308	G:0.987013	A:0.012987
chr28	10745602	2	290	T:0.996552	C:0.00344828
chr28	10746348	2	298	T:0.879195	C:0.120805
chr28	10746405	2	294	C:0.897959	T:0.102041
chr28	10746557	2	308	T:0.983766	G:0.0162338
chr28	10747138	2	310	C:0.967742	T:0.0322581
chr28	10747901	2	294	A:0.673469	AAAG:0.326531
chr28	10748051	2	294	G:0.901361	A:0.0986395
chr28	10748086	2	292	A:0.777397	G:0.222603
chr28	10748125	2	298	G:0.909396	T:0.090604
chr28	10748217	2	238	TA:0.57563	T:0.42437
chr28	10748235	2	300	A:0.886667	T:0.113333
chr28	10748238	2	300	A:0.886667	G:0.113333
chr28	10748313	2	306	TCCTG:0.941176	T:0.0588235
chr28	10748702	2	296	C:0.983108	T:0.0168919
chr28	10748721	2	292	C:0.873288	T:0.126712
chr28	10750001	2	314	A:0.799363	G:0.200637
chr28	10750349	2	302	T:0.549669	C:0.450331
chr28	10750796	2	284	AG:0.862676	A:0.137324
chr28	10750958	2	272	C:0.893382	G:0.106618
chr28	10750978	3	286	TAAA:0.716783	T:0.0804196
TAAAA:0.202797					
chr28	10751008	2	288	A:0.982639	G:0.0173611
chr28	10751976	2	310	A:0.948387	G:0.0516129
chr28	10752176	2	310	A:0.751613	G:0.248387
chr28	10753060	2	290	CAT:0.906897	C:0.0931034
chr28	10753191	4	318	AAAAAC:0.443396	A:0.0566038
AAAAACAAAAC:0.446541			AAAAACAAAACAAAAC:0.0534591		
chr28	10753229	3	318	A:0.902516	C:0.0880503
AACAAC:0.00943396					
chr28	10753234	2	314	C:0.773885	CAAAA:0.226115
chr28	10753435	2	300	C:0.873333	A:0.126667
chr28	10753754	2	300	G:0.893333	A:0.106667
chr28	10755296	2	308	T:0.951299	C:0.0487013
chr28	10755601	2	304	A:0.868421	C:0.131579
chr28	10756021	2	298	A:0.88255	G:0.11745
chr28	10756752	2	304	AAAAC:0.776316	A:0.223684
chr28	10757820	2	302	T:0.903974	C:0.0960265
chr28	10758996	2	300	G:0.846667	A:0.153333
chr28	10760078	2	308	A:0.785714	AT:0.214286
chr28	10760230	2	310	T:0.874194	C:0.125806
chr28	10761064	2	308	A:0.772727	ATAGTT:0.227273

chr28 10761196 3 288 CA:0.4375 C:0.409722
CAA:0.152778
chr28 10761567 2 316 A:0.743671
ACAGCTATTTTTGTGTCTG:0.256329
chr28 10761664 2 294 C:0.928571 T:0.0714286
chr28 10761718 2 312 T:0.990385 C:0.00961538
chr28 10761997 2 294 AT:0.887755 A:0.112245
chr28 10762095 2 276 C:0.898551 T:0.101449
chr28 10762286 4 286 TAAAA:0.465035 T:0.118881
TAA:0.157343 TAAA:0.258741
chr28 10762464 2 296 T:0.885135 TATGTC:0.114865
chr28 10762491 2 296 G:0.976351 C:0.0236486
chr28 10762522 2 304 T:0.888158 C:0.111842
chr28 10763694 2 296 G:0.966216 A:0.0337838
chr28 10763987 4 316 CAGAG:0.53481 C:0.117089
CAG:0.234177 CAGAGAG:0.113924
chr28 10764143 2 300 T:0.76 C:0.24
chr28 10764367 2 300 C:0.886667 T:0.113333
chr28 10764591 2 314 T:0.754777 C:0.245223
chr28 10765710 2 298 A:0.781879 C:0.218121
chr28 10765969 2 306 T:0.970588 C:0.0294118
chr28 10766709 4 298 TAG:0.261745 T:0.251678
TAGAG:0.342282 TAGAGAG:0.144295
chr28 10766801 2 274 C:0.857664 T:0.142336
chr28 10767588 2 300 A:0.956667 G:0.0433333
chr28 10767617 2 302 CACA:0.980132 C:0.0198675
chr28 10767623 2 304 G:0.875 T:0.125
chr28 10767629 2 304 C:0.875 A:0.125
chr28 10768234 2 298 G:0.889262 GT:0.110738
chr28 10768246 2 300 T:0.786667 A:0.213333
chr28 10768247 2 300 A:0.75 T:0.25
chr28 10769125 2 314 AATGT:0.936306 A:0.0636943
chr28 10769156 2 304 C:0.756579 A:0.243421
chr28 10769679 2 302 T:0.877483 C:0.122517
chr28 10769736 2 308 A:0.883117 G:0.116883
chr28 10770443 2 296 G:0.989865 C:0.0101351
chr28 10770945 2 302 A:0.761589 G:0.238411
chr28 10771080 2 306 A:0.751634 ACT:0.248366
chr28 10772205 2 302 TA:0.781457 T:0.218543
chr28 10772387 3 270 ATT:0.703704 A:0.148148
AT:0.148148
chr28 10772437 2 252 CAG:0.940476 C:0.0595238
chr28 10772439 2 264 G:0.962121 C:0.0378788
chr28 10772859 2 302 C:0.748344 T:0.251656
chr28 10772884 2 306 TA:0.852941 T:0.147059
chr28 10773090 2 304 T:0.707237 C:0.292763
chr28 10773296 2 280 C:0.939286 T:0.0607143
chr28 10773321 2 268 C:0.794776 CT:0.205224
chr28 10774112 2 306 CTA:0.879085 C:0.120915
chr28 10774172 3 308 AAAAC:0.75 A:0.123377
AAAACAAAC:0.126623
chr28 10774615 2 302 G:0.890728 A:0.109272
chr28 10774791 2 314 G:0.869427 A:0.130573
chr28 10774957 2 300 A:0.873333 C:0.126667

chr28	10775164	2	306	G:0.761438	A:0.238562	
chr28	10776442	2	304	T:0.782895	C:0.217105	
chr28	10776469	2	306	AT:0.986928	A:0.0130719	
chr28	10776941	2	294	A:0.921769	T:0.0782313	
chr28	10777379	2	298	C:0.95302	T:0.0469799	
chr28	10777681	2	294	A:0.92517	AAAAAG:0.0748299	
chr28	10777772	2	306	C:0.856209	T:0.143791	
chr28	10777871	2	304	T:0.763158	A:0.236842	
chr28	10778270	2	292	GA:0.938356	G:0.0616438	
chr28	10778309	2	304	C:0.766447	T:0.233553	
chr28	10778666	2	302	C:0.890728	T:0.109272	
chr28	10779001	2	296	C:0.871622	T:0.128378	
chr28	10779043	2	304	C:0.878289	T:0.121711	
chr28	10779220	2	292	C:0.962329	CT:0.0376712	
chr28	10779294	2	292	C:0.876712	T:0.123288	
chr28	10779582	2	306	G:0.885621	T:0.114379	
chr28	10779588	2	308	G:0.983766	T:0.0162338	
chr28	10780909	2	300	TA:0.95	T:0.05	
chr28	10780910	3	300	ATT:0.786667	A:0.116667	AT:0.0966667
chr28	10781368	2	306	C:0.98366	T:0.0163399	
chr28	10781582	2	288	TTC:0.947917	T:0.0520833	
chr28	10781588	2	300	T:0.99	C:0.01	
chr28	10782031	2	284	C:0.75	T:0.25	
chr28	10782115	2	258	T:0.883721	TA:0.116279	
chr28	10782176	2	276	T:0.731884	TCTG:0.268116	
chr28	10782912	2	284	A:0.746479	G:0.253521	
chr28	10783073	2	252	T:0.876984	TA:0.123016	
chr28	10783138	2	172	G:0.860465	A:0.139535	
chr28	10783392	2	214	G:0.873832	GC:0.126168	
chr28	10783837	2	264	C:0.833333	T:0.166667	
chr28	10784018	2	208	G:0.9375	T:0.0625	
chr28	10784077	2	170	G:0.888235	C:0.111765	
chr28	10785442	2	266	G:0.954887	A:0.0451128	
chr28	10786021	2	282	G:0.843972	A:0.156028	
chr28	10786049	2	304	T:0.967105	G:0.0328947	
chr28	10786062	2	304	G:0.753289	T:0.246711	
chr28	10786076	2	302	G:0	T:1	
chr28	10786136	2	304	C:0	G:1	
chr28	10786220	2	296	T:0.956081	C:0.0439189	
chr28	10786342	2	304	G:0.891447	A:0.108553	
chr28	10786343	2	304	G:0.960526	A:0.0394737	
chr28	10786477	2	306	G:0.954248	T:0.0457516	
chr28	10786561	2	302	C:0.834437	T:0.165563	
chr28	10786564	2	302	G:0.960265	T:0.0397351	
chr28	10786946	2	310	G:0.977419	A:0.0225806	
chr28	10787158	2	306	T:0.96732	C:0.0326797	
chr28	10787239	2	296	CTTTCT:0.297297	C:0.702703	
chr28	10787349	2	196	G:0.331633	T:0.668367	
chr28	10787915	2	306	GC:0	G:1	
chr28	10788645	2	298	C:0	G:1	
chr28	10788875	2	316	AGCATCCTGCC:0.936709	A:0.0632911	
chr28	10788888	2	316	A:0.89557	G:0.10443	

chr28	10789147	2	312	C:0.990385	T:0.00961538		
chr28	10789640	2	310	CA:0.951613	C:0.0483871		
chr28	10789643	2	310	TGGGC:0.951613	T:0.0483871		
chr28	10789652	2	310	C:0.951613	T:0.0483871		
chr28	10789771	2	302	G:0.725166	A:0.274834		
chr28	10789774	2	302	G:0.97351	A:0.0264901		
chr28	10790103	2	310	T:0.696774	C:0.303226		
chr28	10790171	2	290	C:0.962069	T:0.037931		
chr28	10790236	3	314	A:0.694268	AGATG:0.11465	AGATGGATG:0.191083	
chr28	10790365	2	296	ATTT:0.847973	A:0.152027		
chr28	10790372	2	314	T:0.984076	TA:0.0159236		
chr28	10790396	2	290	G:0.97931	A:0.0206897		
chr28	10790601	2	302	G:0.831126	C:0.168874		
chr28	10790641	2	302	G:0.937086	A:0.0629139		
chr28	10790672	2	304	G:0.970395	A:0.0296053		
chr28	10791027	2	306	A:0.826797	G:0.173203		
chr28	10791097	2	300	A:0.96	G:0.04		
chr28	10791288	2	278	G:0.938849	GTGCTAGAGC:0.0611511		
chr28	10791360	2	146	T:0.246575	C:0.753425		
chr28	10791635	2	312	TAAAAAAA:0.964744	T:0.0352564		
chr28	10791636	2	308	A:0.987013	T:0.012987		
chr28	10791651	2	312	A:0.964744	T:0.0352564		
chr28	10791925	2	316	CT:0.306962	C:0.693038		
chr28	10791931	2	316	A:0.882911	T:0.117089		
chr28	10792362	2	302	G:0.960265	A:0.0397351		
chr28	10792475	2	290	C:0.127586	T:0.872414		
chr28	10792513	2	288	G:0.986111	A:0.0138889		
chr28	10792605	2	298	C:0.95302	CATGA:0.0469799		
chr28	10792682	2	306	G:0.957516	A:0.0424837		
chr28	10792694	4	318	GCACA:0.18239	G:0.0943396	GCA:0.606918	GCACACACA:0.116352
chr28	10792696	2	316	A:0.981013	G:0.0189873		
chr28	10792698	2	312	A:0.964744	G:0.0352564		
chr28	10792700	2	308	A:0.983766	G:0.0162338		
chr28	10792702	2	308	A:0.983766	G:0.0162338		
chr28	10792912	2	306	C:0.300654	A:0.699346		
chr28	10792915	2	308	GT:0.967532	G:0.0324675		
chr28	10793232	2	304	G:0.986842	A:0.0131579		
chr28	10793344	2	294	G:0.717687	GA:0.282313		
chr28	10793715	2	284	A:0.235915	C:0.764085		
chr28	10793717	2	282	A:0.929078	C:0.070922		
chr28	10793778	2	296	A:0.989865	G:0.0101351		
chr28	10794281	2	304	C:0.292763	T:0.707237		
chr28	10794711	2	304	ACCC:0.983553	A:0.0164474		
chr28	10794779	2	300	C:0.973333	T:0.0266667		
chr28	10796986	2	292	A:0.263699	G:0.736301		
chr28	10797230	2	300	A:0.303333	G:0.696667		
chr28	10797858	2	312	AGGGTTGGGGGC:0.301282	A:0.698718		
chr28	10798030	2	298	T:0.221477	C:0.778523		
chr28	10798522	2	306	G:0.316993	A:0.683007		

chr28	10798608	2	304	A:0.105263	C:0.894737
chr28	10798699	2	280	C:0.971429	T:0.0285714
chr28	10798759	2	296	T:0.692568	C:0.307432
chr28	10798910	2	290	TG:0.331034	T:0.668966
chr28	10798916	2	290	GCACC:0.693103	G:0.306897
chr28	10798919	2	290	C:0.331034	CA:0.668966
chr28	10799285	2	284	C:0.278169	A:0.721831
chr28	10799467	2	284	C:0.299296	T:0.700704
chr28	10799500	2	282	T:0.322695	C:0.677305
chr28	10799935	2	296	T:0.692568	G:0.307432
chr28	10800458	2	306	C:0.960784	T:0.0392157
chr28	10800816	2	302	T:0.986755	TAA:0.013245
chr28	10800817	2	302	GCT:0.986755	G:0.013245
chr28	10800836	2	296	G:0.753378	A:0.246622
chr28	10801389	2	312	G:0.958333	T:0.0416667
chr28	10801683	2	306	G:0.284314	A:0.715686
chr28	10801900	2	304	G:0.703947	A:0.296053
chr28	10802217	2	302	AAGAG:0.745033	A:0.254967
chr28	10802229	2	304	G:0.9375	A:0.0625
chr28	10803019	2	304	C:0.986842	T:0.0131579
chr28	10803956	2	306	T:0.986928	G:0.0130719
chr28	10804116	2	300	G:0.716667	A:0.283333
chr28	10804198	2	286	CTG:0.699301	C:0.300699
chr28	10804280	2	302	TA:0.748344	T:0.251656
chr28	10805161	2	300	A:0.71	G:0.29
chr28	10805332	2	316	T:0.962025	TG:0.0379747
chr28	10805334	2	316	A:0.962025	AGACAAAAGAAGGAGACAGC:0.0379747
chr28	10805337	2	316	GCTCTCTC:0.962025	G:0.0379747
chr28	10805458	2	308	G:0.756494	A:0.243506
chr28	10805484	2	306	C:0.986928	T:0.0130719
chr28	10805708	2	308	A:0.311688	T:0.688312
chr28	10805938	2	262	C:0.984733	T:0.0152672
chr28	10806164	2	258	T:0.697674	C:0.302326
chr28	10807229	2	302	A:0.986755	G:0.013245
chr28	10807334	2	302	G:0.708609	A:0.291391
chr28	10807952	2	308	CT:0.967532	C:0.0324675
chr28	10808716	2	304	G:0.203947	T:0.796053
chr28	10808723	2	294	C:0.418367	CTCTT:0.581633
chr28	10808727	2	310	T:0.951613	TTCTTTC:0.0483871
chr28	10808788	2	300	G:0.956667	A:0.0433333
chr28	10810294	2	320	G:0.825	GAAAAAAAAAAAAAAAAAAA:0.175
chr28	10810296	2	320	A:0.790625	AAAATCTGATGAGGT:0.209375
chr28	10810503	2	296	G:0.709459	A:0.290541
chr28	10810518	2	290	G:0.72069	T:0.27931
chr28	10810680	2	304	C:0.289474	T:0.710526
chr28	10810910	2	294	T:0.292517	C:0.707483
chr28	10811208	2	308	GT:0.314935	G:0.685065
chr28	10811217	2	308	C:0.314935	T:0.685065
chr28	10811295	2	304	G:0.299342	C:0.700658
chr28	10811860	2	292	A:0.732877	G:0.267123

chr28 10811995 2 278 G:0.708633 T:0.291367
chr28 10812105 2 286 A:0.944056 G:0.0559441
chr28 10812120 2 288 G:0.725694 A:0.274306
chr28 10812280 2 294 T:0.255102 C:0.744898
chr28 10812446 2 294 C:0.309524 T:0.690476
chr28 10812841 2 298 G:0.288591 A:0.711409
chr28 10812961 2 312 A:0.983974 G:0.0160256
chr28 10813514 2 288 T:0.329861 A:0.670139
chr28 10814023 2 290 A:0.931034 G:0.0689655
chr28 10814099 2 302 AT:0.701987 A:0.298013
chr28 10814148 2 306 C:0.764706 CT:0.235294
chr28 10814149 2 306 C:0.53268 T:0.46732
chr28 10814150 3 306 C:0.738562 T:0.238562
CTT:0.0228758
chr28 10814876 2 250 C:0.192 G:0.808
chr28 10815237 2 292 G:0.291096 A:0.708904
chr28 10815616 2 300 G:0.71 T:0.29
chr28 10815779 2 288 CG:0.708333 C:0.291667
chr28 10815825 4 314 CCTCTCTCT:0.187898 C:
0.646497 CCT:0.0191083 CTTCTCTCTCT:0.146497
chr28 10815846 2 282 C:0.978723 G:0.0212766
chr28 10815848 2 266 C:0.255639 G:0.744361
chr28 10815896 2 294 T:0.319728 TA:0.680272
chr28 10816356 2 300 A:0.69 G:0.31
chr28 10816447 2 304 G:0.947368 A:0.0526316
chr28 10816779 2 284 G:0.971831 A:0.028169
chr28 10816995 2 296 G:0.287162 A:0.712838
chr28 10817370 2 296 A:0.722973 G:0.277027
chr28 10817535 2 290 G:0.744828 T:0.255172
chr28 10818450 2 298 A:0.963087 G:0.0369128
chr28 10819367 2 300 A:0.72 G:0.28
chr28 10819539 2 302 T:0.705298 C:0.294702
chr28 10819736 2 294 C:0.931973 CG:0.0680272
chr28 10819935 3 258 TA:0.810078 T:0.108527
TAA:0.0813953
chr28 10820648 2 296 C:0.695946 T:0.304054
chr28 10821105 2 306 A:0.986928 G:0.0130719
chr28 10821281 2 294 G:0.29932 A:0.70068
chr28 10821414 2 304 G:0.904605 A:0.0953947
chr28 10821971 2 302 C:0.943709 T:0.0562914
chr28 10822368 2 300 T:0.31 C:0.69
chr28 10822387 2 308 C:0.262987 T:0.737013
chr28 10823190 2 304 T:0.707237 C:0.292763
chr28 10823207 2 294 T:0.693878 TA:0.306122
chr28 10823751 2 314 T:0.308917 A:0.691083
chr28 10823975 2 286 G:0.954545 A:0.0454545
chr28 10824004 2 302 C:0 A:1
chr28 10824116 2 298 T:0.738255 C:0.261745
chr28 10824189 2 300 G:0.73 A:0.27
chr28 10824896 2 266 T:0.778195 C:0.221805
chr28 10825145 2 246 A:0.703252 C:0.296748
chr28 10825361 2 280 G:0.878571 A:0.121429
chr28 10825928 2 302 A:0.986755 G:0.013245
chr28 10826445 2 300 T:0.28 C:0.72

chr28	10826488	2	298	C:0.765101	T:0.234899	
chr28	10826717	2	296	G:0.233108	A:0.766892	
chr28	10827088	2	304	T:0.233553	C:0.766447	
chr28	10827157	2	302	G:0.261589	A:0.738411	
chr28	10827382	2	294	T:0.244898	C:0.755102	
chr28	10827768	2	294	CATTT:0.744898	C:0.255102	
chr28	10827809	2	276	G:0.981884	A:0.0181159	
chr28	10828635	2	310	T:0.712903	C:0.287097	
chr28	10829371	2	310	G:0.951613	A:0.0483871	
chr28	10830034	2	284	T:0.753521	TA:0.246479	
chr28	10830214	2	294	T:0.77551	TA:0.22449	
chr28	10830246	2	284	G:0.771127	A:0.228873	
chr28	10830435	2	288	A:0.958333	C:0.0416667	
chr28	10830652	2	302	T:0.731788	C:0.268212	
chr28	10831011	2	288	G:0.996528	A:0.00347222	
chr28	10831614	2	298	T:0.993289	G:0.00671141	
chr28	10831690	2	300	G:0.736667	A:0.263333	
chr28	10832068	2	302	C:0.980132	T:0.0198675	
chr28	10832590	2	298	TAAAAA:0.573826	T:0.426174	
chr28	10833107	2	296	C:0.75	A:0.25	
chr28	10833406	2	298	G:0.714765	A:0.285235	
chr28	10833643	2	294	A:0.755102	C:0.244898	
chr28	10833794	2	306	G:0.990196	A:0.00980392	
chr28	10833839	3	314	G:0.589172	GTTCTTTTCTTTTCTT:0.248408	GTTCTTTTCTTTTCTTTTCTT:0.16242
chr28	10833871	2	314	T:0.920382	TC:0.0796178	
chr28	10833872	2	314	T:0.920382	TTTTCTTTTCTTTTCTTTTC:0.0796178	
chr28	10834198	2	292	A:0.743151	T:0.256849	
chr28	10834480	2	198	G:0.979798	T:0.020202	
chr28	10835886	2	286	T:0.79021	G:0.20979	
chr28	10836669	2	282	G:0.223404	GC:0.776596	
chr28	10837170	2	288	G:0.9375	A:0.0625	
chr28	10837946	2	304	G:0.868421	T:0.131579	
chr28	10838357	2	296	G:0.699324	C:0.300676	
chr28	10838463	2	294	T:0.887755	C:0.112245	
chr28	10838727	2	290	C:0.703448	T:0.296552	
chr28	10839018	2	284	AG:0.989437	A:0.0105634	
chr28	10839579	2	288	C:0.423611	T:0.576389	
chr28	10839669	2	262	A:0.732824	G:0.267176	
chr28	10839670	2	262	T:0.732824	G:0.267176	
chr28	10839780	3	294	TTAAATAAA:0.394558	T:0.377551	TTAAA:0.227891
chr28	10840014	2	278	G:0.28777	C:0.71223	
chr28	10840673	2	306	G:0.986928	A:0.0130719	
chr28	10840824	2	298	A:0.0771812	T:0.922819	
chr28	10840924	2	286	C:0.954545	T:0.0454545	
chr28	10841198	2	284	G:0.869718	A:0.130282	
chr28	10841364	2	298	T:0.181208	A:0.818792	
chr28	10841596	2	298	T:0.718121	C:0.281879	
chr28	10842018	2	300	A:0.7	C:0.3	
chr28	10842498	2	286	T:0	C:1	
chr28	10843285	2	284	G:0.943662	A:0.056338	
chr28	10843350	2	282	A:0.705674	G:0.294326	

chr28	10843359	2	284	T:0.901408	G:0.0985915	
chr28	10843728	2	302	T:0.642384	C:0.357616	
chr28	10843855	2	304	G:0.680921	C:0.319079	
chr28	10843924	2	302	T:0.665563	C:0.334437	
chr28	10843962	2	306	G:0.663399	A:0.336601	
chr28	10844288	2	306	G:0.660131	C:0.339869	
chr28	10844333	2	312	GACTGAGAGT:0.650641	G: 0.349359	
chr28	10844335	2	312	C:0.375	CT:0.625	
chr28	10844341	2	312	GT:0.375	G:0.625	
chr28	10844570	2	262	A:0.270992	G:0.729008	
chr28	10844616	2	298	A:0.285235	G:0.714765	
chr28	10844668	2	294	CT:0.248299	C:0.751701	
chr28	10844683	3	294	ATT:0.22449	A:0.676871	AT:0.0986395
chr28	10844687	2	294	T:0.938776	A:0.0612245	
chr28	10844769	2	288	A:0.340278	ACT:0.659722	
chr28	10844795	2	290	T:0.313793	C:0.686207	
chr28	10844839	2	278	C:0.877698	T:0.122302	
chr28	10844863	2	290	A:0.293103	G:0.706897	
chr28	10844929	2	306	C:0.124183	T:0.875817	
chr28	10845417	2	298	C:0.674497	T:0.325503	
chr28	10845589	2	294	T:0.693878	C:0.306122	
chr28	10846118	3	304	CTT:0.319079	C:0.0559211	CT:0.625
chr28	10846650	2	304	T:0.690789	TAGG:0.309211	
chr28	10846675	2	304	C:0.700658	T:0.299342	
chr28	10846928	2	294	G:0.442177	T:0.557823	
chr28	10847235	2	302	G:0.943709	A:0.0562914	
chr28	10847410	2	314	G:0.968153	C:0.0318471	
chr28	10847418	2	314	A:0.0955414	G:0.904459	
chr28	10847584	2	296	T:0.280405	A:0.719595	
chr28	10847679	2	72	CAG:0.5	C:0.5	
chr28	10848111	2	296	G:0.307432	A:0.692568	
chr28	10848174	2	284	T:0.112676	C:0.887324	
chr28	10848225	2	282	G:0.258865	A:0.741135	
chr28	10848239	2	282	C:0.85461	A:0.14539	
chr28	10848338	2	266	T:0.966165	TA:0.0338346	
chr28	10848394	2	262	T:0.828244	A:0.171756	
chr28	10848395	2	262	T:0.870229	A:0.129771	
chr28	10848499	2	224	A:0.40625	T:0.59375	
chr28	10849025	2	196	GTTTTT:0	G:1	
chr28	10849030	2	196	T:0	TGCCGC:1	
chr28	10849039	2	200	T:0.86	C:0.14	
chr28	10849065	2	192	GTTT:0.880208	G:0.119792	
chr28	10849066	2	170	T:0.170588	G:0.829412	
chr28	10849067	2	170	TTTT:0.170588	TCC:0.829412	
chr28	10849069	2	192	T:0.880208	G:0.119792	
chr28	10849070	2	192	T:0.880208	TCC:0.119792	
chr28	10849276	2	292	T:0.763699	C:0.236301	
chr28	10849368	2	298	C:0.771812	G:0.228188	
chr28	10849447	2	308	G:0.415584	A:0.584416	
chr28	10849747	2	304	C:0.875	T:0.125	
chr28	10850041	2	308	A:0.0941558	G:0.905844	

chr28 10850060 2 304 T:0.240132 C:0.759868
chr28 10850147 2 310 CACTG:0.377419 C:0.622581
chr28 10850252 2 302 G:0.943709 A:0.0562914
chr28 10850338 2 300 G:0.396667 T:0.603333
chr28 10850349 2 300 G:0.926667 C:0.0733333
chr28 10850419 2 302 T:0.248344 C:0.751656
chr28 10850681 2 298 T:0.234899 G:0.765101
chr28 10850687 2 298 T:0.234899 C:0.765101
chr28 10850927 2 300 G:0.85 C:0.15
chr28 10850955 2 296 A:0.135135 G:0.864865
chr28 10850988 2 302 C:0.221854 T:0.778146
chr28 10851005 2 298 G:0.939597 A:0.0604027
chr28 10851045 2 296 C:0.381757 T:0.618243
chr28 10851749 2 308 TA:0.214286 T:0.785714
chr28 10851825 2 300 T:0.906667 TA:0.0933333
chr28 10851934 2 300 A:0.386667 G:0.613333
chr28 10851940 2 302 A:0.768212 G:0.231788
chr28 10852366 2 290 A:0.241379 G:0.758621
chr28 10852426 2 290 T:0.258621 C:0.741379
chr28 10852547 2 298 G:0.385906 A:0.614094
chr28 10852562 2 298 C:0.385906 T:0.614094
chr28 10852702 2 298 C:0.989933 T:0.0100671
chr28 10853084 2 280 C:0.928571 T:0.0714286
chr28 10853383 2 304 G:0.9375 A:0.0625
chr28 10853503 2 320
TGAGCCACCAGAGCCACCCAAGCCATCAGAGCTACCCAAGCCATCCGAGCCACCCAAGCCACCA:
0.353125 T:0.646875
chr28 10853651 2 302 T:0.221854 A:0.778146
chr28 10853841 2 288 G:0.923611 A:0.0763889
chr28 10854052 2 314 TCAAGTGGCCC:0.907643 T:
0.0923567
chr28 10854055 2 314 A:0.378981 T:0.621019
chr28 10854064 2 314 TGG:0.907643 T:0.0923567
chr28 10854067 2 314 C:0.907643 A:0.0923567
chr28 10854492 2 304 G:0.148026 C:0.851974
chr28 10854518 2 290 T:0.989655 TC:0.0103448
chr28 10854706 2 298 C:0.828859 T:0.171141
chr28 10854778 2 292 A:0.958904 G:0.0410959
chr28 10854837 2 300 C:0.856667 T:0.143333
chr28 10855201 4 312 TTATCTATCTATC:0.471154 T:
0.0705128 TTATCTATC:0.163462 TTATCTATCTATCTATC:
0.294872
chr28 10855616 2 300 A:0.156667 G:0.843333
chr28 10855998 2 320 G:0.8375 GGA:0.1625
chr28 10856001 2 320 ATCT:0.8375 A:0.1625
chr28 10856008 2 320 AAAAAAGTTT:0.8375 A:0.1625
chr28 10856018 2 320 CAA:0.8375 C:0.1625
chr28 10856024 2 320 GACAAAATTCAAAGTCTGCA:0.8375
G:0.1625
chr28 10856047 2 320 AGT:0.8375 A:0.1625
chr28 10856051 2 320 TAG:0.8375 T:0.1625
chr28 10856054 2 320 T:0.8375 TAA:0.1625
chr28 10856060 2 320 T:0.8375 A:0.1625
chr28 10856061 2 320 GTT:0.8375 G:0.1625

chr28 10856067 2 320 T:0.8375 TAA:0.1625
chr28 10856069 2 320 T:0.8375 A:0.1625
chr28 10856605 2 302 GC:0.387417 G:0.612583
chr28 10856834 2 288 G:0.361111 A:0.638889
chr28 10856891 2 302 C:0.764901 T:0.235099
chr28 10857213 4 316 GGAGAGAGAGAGAGA:0.553797 G:
0.126582 GGAGAGAGAGA:0.126582 GGAGAGAGAGAGAGAGA:0.193038
chr28 10857820 2 278 C:0.809353 CT:0.190647
chr28 10857822 2 272 TC:0.683824 T:0.316176
chr28 10858452 2 286 G:0.982517 A:0.0174825
chr28 10858753 2 302 G:0.860927 A:0.139073
chr28 10859031 2 308 C:0.980519 A:0.0194805
chr28 10859145 2 304 G:0.845395 A:0.154605
chr28 10859432 2 292 T:0.366438 C:0.633562
chr28 10859500 3 292 C:0.174658 CCT:0.660959
CCTCT:0.164384
chr28 10859548 3 288 T:0.121528 TAA:0.711806
TAAA:0.166667
chr28 10859591 2 308 A:0.0779221 G:0.922078
chr28 10859789 3 308 GT:0.454545 G:0.0422078
GTT:0.503247
chr28 10859873 2 308 C:0.155844 G:0.844156
chr28 10859994 2 312 A:0.875 G:0.125
chr28 10860062 2 304 A:0.875 G:0.125
chr28 10860259 2 308 CAGAAAAA:0.288961 C:
0.711039
chr28 10860267 2 308 TG:0.288961 T:0.711039
chr28 10860770 2 306 A:0.238562 G:0.761438
chr28 10861029 2 294 G:0.945578 A:0.0544218
chr28 10861190 2 256 G:0.691406 C:0.308594
chr28 10861553 2 298 G:0.395973 A:0.604027
chr28 10861598 2 302 C:0.877483 T:0.122517
chr28 10861930 2 292 G:0.39726 A:0.60274
chr28 10862118 2 306 C:0.869281 T:0.130719
chr28 10862629 2 294 A:0.285714 G:0.714286
chr28 10862731 2 274 G:0.945255 T:0.0547445
chr28 10862999 2 296 C:0.692568 T:0.307432
chr28 10863039 2 298 A:0.885906 T:0.114094
chr28 10863951 2 312 C:0.737179 T:0.262821
chr28 10863957 2 312 C:0.426282 T:0.573718
chr28 10864055 2 302 A:0.741722 G:0.258278
chr28 10864420 2 296 GT:0.402027 G:0.597973
chr28 10864676 2 308 T:0.256494 A:0.743506
chr28 10864720 2 300 A:0.27 T:0.73
chr28 10865160 2 314 TAC:0.414013 T:0.585987
chr28 10865211 3 298 GT:0.379195 G:0.583893
GTT:0.0369128
chr28 10865255 2 296 C:0.371622 A:0.628378
chr28 10865768 4 292 C:0.263699 CT:0.136986
CTT:0.458904 CTTT:0.140411
chr28 10865823 2 312 A:0.788462 AAGAGAGAG:
0.211538
chr28 10865863 2 296 C:0.695946 A:0.304054
chr28 10865864 2 296 C:0.695946 G:0.304054

chr28 10866132 2 304 TAGA:0.947368 T:0.0526316
chr28 10866499 2 310 G:0.945161 A:0.0548387
chr28 10866730 2 302 A:0.903974 G:0.0960265
chr28 10866868 2 310 C:0.929032 A:0.0709677
chr28 10866882 3 314 T:0.410828 TTTTTTTTC:
0.487261 TTTTTTTTTC:0.101911
chr28 10866963 2 302 T:0.837748 C:0.162252
chr28 10867622 2 294 T:0.42517 A:0.57483
chr28 10867736 2 284 G:0.764085 C:0.235915
chr28 10868147 2 262 C:0.515267 T:0.484733
chr28 10868162 2 300 C:0.773333 T:0.226667
chr28 10868166 3 316 CCTCT:0.313291 C:0.21519
CCTCTCTCTCTCTCTCTCT:0.471519
chr28 10868195 2 290 C:0.465517 T:0.534483
chr28 10868318 2 310 T:0.412903 A:0.587097
chr28 10868481 2 280 A:0.0857143 T:0.914286
chr28 10868483 2 280 T:0.167857 C:0.832143
chr28 10868521 2 308 CTCTCTCTCTT:0.818182 C:
0.181818
chr28 10868576 2 298 A:0.151007 AAAT:0.848993
chr28 10868578 2 298 A:0.926174 AT:0.0738255
chr28 10868758 2 286 C:0.741259 T:0.258741
chr28 10868815 2 288 G:0.381944 C:0.618056
chr28 10868964 2 306 T:0.418301 A:0.581699
chr28 10869130 2 292 C:0.260274 T:0.739726
chr28 10869158 2 288 T:0.871528 G:0.128472
chr28 10869284 2 310 A:0.432258 G:0.567742
chr28 10869285 2 310 A:0.432258 C:0.567742
chr28 10869310 2 310 T:0.16129 G:0.83871
chr28 10869556 2 276 C:0.376812 CTTT:0.623188
chr28 10869590 2 304 GAGAT:0.967105 G:0.0328947
chr28 10869596 2 292 GAGAC:0.767123 G:0.232877
chr28 10869650 2 298 A:0.322148 T:0.677852
chr28 10869654 2 296 C:0.398649 T:0.601351
chr28 10869696 2 282 C:0.397163 T:0.602837
chr28 10869731 2 298 C:0.372483 A:0.627517
chr28 10869775 2 290 T:0.403448 A:0.596552
chr28 10869897 2 290 C:0.4 T:0.6
chr28 10870079 2 296 C:0.385135 T:0.614865
chr28 10870392 2 306 A:0.441176 G:0.558824
chr28 10870618 2 296 T:0.439189 G:0.560811
chr28 10870656 2 294 G:0.421769 A:0.578231
chr28 10870776 2 264 G:0.439394 GT:0.560606
chr28 10870878 2 282 T:0.868794 C:0.131206
chr28 10870990 2 278 A:0.266187 G:0.733813
chr28 10871005 2 286 C:0.940559 A:0.0594406
chr28 10871006 2 286 C:0.888112 T:0.111888
chr28 10871062 2 292 G:0.414384 T:0.585616
chr28 10871087 2 298 C:0.432886 T:0.567114
chr28 10871128 2 292 T:0.39726 C:0.60274
chr28 10871129 2 292 G:0.39726 A:0.60274
chr28 10871149 2 300 C:0.85 T:0.15
chr28 10871212 2 296 G:0.885135 A:0.114865
chr28 10871264 2 298 C:0.419463 A:0.580537

chr28	10871302	2	286	C:0.381119	T:0.618881	
chr28	10871347	2	302	T:0.887417	C:0.112583	
chr28	10871425	2	298	C:0.389262	T:0.610738	
chr28	10871534	2	298	A:0.268456	G:0.731544	
chr28	10871606	2	310	GC:0.919355	G:0.0806452	
chr28	10871740	2	302	A:0.950331	G:0.0496689	
chr28	10871886	2	304	G:0.434211	A:0.565789	
chr28	10871887	2	304	T:0.434211	C:0.565789	
chr28	10872568	2	300	T:0.1	A:0.9	
chr28	10872820	2	300	AG:0.176667	A:0.823333	
chr28	10872826	2	304	T:0.983553	TA:0.0164474	
chr28	10872858	2	306	A:0.434641	G:0.565359	
chr28	10872929	2	308	T:0.863636	G:0.136364	
chr28	10873807	2	310	T:0.432258	C:0.567742	
chr28	10874308	2	292	G:0.0993151	A:0.900685	
chr28	10874488	2	302	C:0.847682	T:0.152318	
chr28	10874513	2	298	C:0.919463	T:0.0805369	
chr28	10874561	2	302	A:0.933775	C:0.0662252	
chr28	10874875	2	288	T:0.989583	C:0.0104167	
chr28	10874877	2	286	G:0.979021	A:0.020979	
chr28	10875700	2	306	C:0.846405	T:0.153595	
chr28	10876582	2	296	C:0.925676	G:0.0743243	
chr28	10876610	2	300	G:0.83	T:0.17	
chr28	10876739	2	300	G:0.84	A:0.16	
chr28	10877140	2	294	G:0.408163	A:0.591837	
chr28	10877370	2	302	T:0.145695	G:0.854305	
chr28	10877580	2	278	TCA:0.942446	T:0.057554	
chr28	10877612	3	276	T:0.282609	TA:0.34058	TAAA:0.376812
chr28	10877971	2	302	G:0.927152	T:0.0728477	
chr28	10877998	2	304	C:0.845395	T:0.154605	
chr28	10878014	2	304	C:0.845395	A:0.154605	
chr28	10878049	2	300	A:0.846667	T:0.153333	
chr28	10878119	2	300	A:0.25	G:0.75	
chr28	10878826	2	300	C:0.97	T:0.03	
chr28	10879239	2	312	T:0.413462	A:0.586538	
chr28	10879496	2	310	T:0.980645	C:0.0193548	
chr28	10879749	2	302	G:0.966887	C:0.0331126	
chr28	10879835	2	312	C:0.272436	G:0.727564	
chr28	10879837	2	308	AT:0.831169	A:0.168831	
chr28	10879933	2	302	T:0.135762	C:0.864238	
chr28	10880214	2	294	C:0.14966	T:0.85034	
chr28	10880333	2	310	G:0.141935	GATTAA:0.858065	
chr28	10880468	2	286	C:0.839161	T:0.160839	
chr28	10880501	2	282	G:0.0957447	A:0.904255	
chr28	10880523	2	284	C:0.838028	T:0.161972	
chr28	10880620	2	306	C:0.254902	G:0.745098	
chr28	10880621	2	314	T:0.127389	TGTGA:0.872611	
chr28	10880633	2	310	T:0.954839	TA:0.0451613	
chr28	10880637	2	306	A:0.859477	AAAAAAAAT: 0.140523	
chr28	10880638	3	306	A:0.356209	AAAAAAAT:0.633987	AAAAAAATT:0.00980392
chr28	10880657	2	310	A:0.954839	T:0.0451613	

chr28 10880785 2 296 T:0.0810811 A:0.918919
chr28 10880789 2 296 C:0.898649 A:0.101351
chr28 10881307 2 304 A:0.167763 C:0.832237
chr28 10881437 2 308 A:0.863636 G:0.136364
chr28 10881444 2 308 C:0.383117 T:0.616883
chr28 10881588 3 292 CTTTT:0.818493 C:0.0993151
CTT:0.0821918
chr28 10881646 2 298 C:0.899329 T:0.100671
chr28 10881792 2 286 T:0.912587 G:0.0874126
chr28 10881808 2 292 G:0.921233 A:0.0787671
chr28 10881849 2 296 A:0.847973 G:0.152027
chr28 10882110 2 292 C:0.859589 CCTCT:0.140411
chr28 10882163 2 288 C:0.847222 T:0.152778
chr28 10882434 2 306 G:0.169935 C:0.830065
chr28 10882688 2 298 TG:0.171141 T:0.828859
chr28 10882760 2 296 C:0.817568 A:0.182432
chr28 10882808 2 306 A:0.800654 G:0.199346
chr28 10882976 2 308 C:0.418831 G:0.581169
chr28 10883014 2 304 C:0.8125 T:0.1875
chr28 10883028 2 300 A:0.09 G:0.91
chr28 10883125 2 290 T:0.817241 C:0.182759
chr28 10883240 2 290 TA:0.786207 T:0.213793
chr28 10883368 2 306 T:0.179739 G:0.820261
chr28 10883680 2 306 T:0.189542 C:0.810458
chr28 10884142 2 304 A:0.240132 AT:0.759868
chr28 10884231 2 308 A:0.237013 T:0.762987
chr28 10884436 2 294 A:0.789116 G:0.210884
chr28 10884509 2 300 G:0.983333 C:0.0166667
chr28 10884565 2 296 T:0.97973 G:0.0202703
chr28 10884599 4 298 TAAA:0.221477 T:0.157718
TA:0.463087 TAA:0.157718
chr28 10885261 2 270 T:0.144444 C:0.855556
chr28 10885283 2 278 G:0.147482 A:0.852518
chr28 10885292 2 286 G:0.786713 A:0.213287
chr28 10885993 2 304 T:0.421053 A:0.578947
chr28 10886462 2 308 TAA:0.142857 T:0.857143
chr28 10886477 2 312 C:0.730769 A:0.269231
chr28 10886537 2 306 G:0.973856 A:0.0261438
chr28 10887200 2 308 G:0.717532 A:0.282468
chr28 10887440 2 306 AT:0.401961 A:0.598039
chr28 10887451 2 308 TG:0.996753 T:0.00324675
chr28 10887501 2 318 C:0.886792 CT:0.113208
chr28 10887503 2 318 T:0.41195 TTA:0.58805
chr28 10887504 2 318 TTTTTTTATTTTTTA:0.871069 T:
0.128931
chr28 10887511 2 318 A:0.446541 AT:0.553459
chr28 10887512 2 316 TTTTTTAA:0.993671 T:
0.00632911
chr28 10887519 2 318 A:0.446541 T:0.553459
chr28 10887526 2 318 T:0.871069 TA:0.128931
chr28 10887540 2 318 G:0.767296 GT:0.232704
chr28 10887541 3 318 C:0.166667 A:0.591195
CA:0.242138
chr28 10887552 2 318 G:0.474843 GAGAGAGGC:

0.525157
chr28 10887723 2 294 C:0.969388 A:0.0306122
chr28 10887724 2 294 C:0.278912 G:0.721088
chr28 10887906 2 306 T:0.251634 A:0.748366
chr28 10887991 2 300 G:0.84 A:0.16
chr28 10888001 2 308 G:0.87987 A:0.12013
chr28 10888546 2 304 A:0.276316 G:0.723684
chr28 10888643 2 304 T:0.407895 C:0.592105
chr28 10888752 2 316 C:0.294304 T:0.705696
chr28 10888856 2 304 A:0.421053 G:0.578947
chr28 10888873 2 302 A:0.245033 T:0.754967
chr28 10888979 2 308 A:0.292208 G:0.707792
chr28 10889159 2 316 A:0.439873 G:0.560127
chr28 10889208 2 308 G:0.86039 GTTATT:0.13961
chr28 10889388 2 288 G:0.868056 C:0.131944
chr28 10889581 2 300 TTTTA:0.87 T:0.13
chr28 10889598 2 298 A:0.869128 G:0.130872
chr28 10889965 2 270 A:0.214815 AT:0.785185
chr28 10890035 2 280 C:0.871429 T:0.128571
chr28 10890255 2 300 G:0.313333 A:0.686667
chr28 10890290 2 294 A:0.758503 G:0.241497
chr28 10890352 2 278 G:0.751799 C:0.248201
chr28 10890492 2 282 G:0.677305 A:0.322695
chr28 10890598 3 242 T:0.396694 TG:0.516529
TGG:0.0867769
chr28 10890840 2 112 TGGCGGCGGCGGC:0.294643 T:
0.705357
chr28 10891262 2 258 C:0.0968992 CA:0.903101
chr28 10891267 2 262 G:0.80916 C:0.19084
chr28 10891415 2 292 G:0.472603 A:0.527397
chr28 10891631 2 294 C:0.132653 G:0.867347
chr28 10892104 2 300 G:0.873333 C:0.126667
chr28 10892388 2 304 A:0.980263 C:0.0197368
chr28 10892613 3 290 C:0.582759 CT:0.231034
CTTT:0.186207
chr28 10893265 2 296 T:0.881757 A:0.118243
chr28 10893391 2 310 CT:0.877419 C:0.122581
chr28 10893402 2 308 C:0.824675 T:0.175325
chr28 10893436 2 308 G:0.987013 A:0.012987
chr28 10894068 2 304 C:0.809211 T:0.190789
chr28 10894272 2 268 A:0.86194 AT:0.13806
chr28 10894438 2 316 A:0.993671 T:0.00632911
chr28 10894539 2 278 C:0.482014 CAG:0.517986
chr28 10894647 2 300 C:0.44 A:0.56
chr28 10894755 2 304 G:0.940789 GT:0.0592105
chr28 10894864 2 298 A:0.97651 T:0.0234899
chr28 10894994 2 290 C:0.975862 T:0.0241379
chr28 10895137 3 314 TATTG:0.347134 T:0.0541401
TATTGATTG:0.598726
chr28 10895171 2 312 GACACAC:0.179487 G:0.820513
chr28 10895298 2 296 G:0.925676 C:0.0743243
chr28 10895383 2 308 GTTTGTT:0.87987 G:0.12013
chr28 10895443 2 310 G:0.980645 A:0.0193548
chr28 10895844 2 302 G:0.983444 A:0.0165563

chr28 10896146 2 308 T:0.418831 A:0.581169
chr28 10896255 2 298 G:0.989933 C:0.0100671
chr28 10896385 2 288 TA:0.763889 T:0.236111
chr28 10897336 2 306 G:0.0816993 GC:0.918301
chr28 10897637 2 314 A:0 C:1
chr28 10897638 2 314 A:0 T:1
chr28 10897678 2 310 A:0 T:1
chr28 10897680 2 310 G:0 A:1
chr28 10898145 2 302 T:0.0993377 G:0.900662
chr28 10899759 2 304 G:0.858553 A:0.141447
chr28 10900002 2 306 A:0.415033 G:0.584967
chr28 10901102 2 290 A:0.975862 AT:0.0241379
chr28 10901787 2 310 AGGATTTTCACTGT:0.983871 A:
0.016129
chr28 10902365 4 304 T:0.256579 TTC:0.536184
TTCTC:0.128289 TTCTCTC:0.0789474
chr28 10902471 3 302 CT:0.652318 C:0.168874
CTT:0.178808
chr28 10902624 2 308 G:0.967532 A:0.0324675
chr28 10902631 2 298 GGA:0.969799 G:0.0302013
chr28 10903125 2 304 T:0.980263 C:0.0197368
chr28 10905054 2 312 A:0.961538 G:0.0384615
chr28 10905636 2 268 C:0.697761 CT:0.302239
chr28 10905757 2 290 C:0.358621 CT:0.641379
chr28 10906371 2 300 C:0.986667 T:0.0133333
chr28 10907929 3 272 C:0.345588 CT:0.595588
CTT:0.0588235
chr28 10908479 2 286 G:0.195804 GT:0.804196
chr28 10909005 2 258 G:0.375969 A:0.624031
chr28 10909089 2 298 C:0.922819 T:0.0771812
chr28 10909106 2 286 T:0.454545 TAAA:0.545455
chr28 10909107 4 290 T:0.2 A:0.555172 TAAA:
0.213793 TTAA:0.0310345
chr28 10909525 2 298 C:0.902685 CAGAG:0.0973154
chr28 10909952 6 318 T:0.185535 TATAG:0.122642
TATAGATAG:0.216981 TATAGATAGATAG:0.380503
TATAGATAGATAGATAG:0.0754717 TATAGATAGATAGATAGATAG:0.0188679
chr28 10909963 2 318 A:0.965409 AGATAGATAGATG:
0.0345912
chr28 10911677 2 300 CT:0.963333 C:0.0366667
chr28 10912047 2 294 T:0.969388 A:0.0306122
chr28 10912950 2 306 A:0.862745 G:0.137255
chr28 10913896 2 306 TG:0.98366 T:0.0163399
chr28 10914811 2 292 CTA:0.339041 C:0.660959
chr28 10914827 5 312 CAAATAAAT:0.221154 C:
0.269231 CAAAT:0.160256 CAAATAAATAAAT:0.185897
CAAATAAATAAATAAAT:0.163462
chr28 10915294 2 310 C:0.974194 T:0.0258065
chr28 10915752 2 302 T:0.81457 A:0.18543
chr28 10915858 2 290 C:0.996552 T:0.00344828
chr28 10916032 3 266 C:0.289474 CT:0.357143
CTTT:0.353383
chr28 10916305 2 290 T:0.431034 C:0.568966
chr28 10916365 2 310 C:0.445161 T:0.554839

chr28	10917379	2	238	C:0.390756	CT:0.609244
chr28	10917399	2	268	A:0.850746	C:0.149254
chr28	10918597	2	302	T:0.97351	C:0.0264901
chr28	10918758	2	314	CTAAG:0.996815	C:0.00318471
chr28	10918832	2	302	C:0.857616	T:0.142384
chr28	10920076	2	294	C:0.972789	G:0.0272109
chr28	10924839	2	296	G:0.810811	A:0.189189
chr28	10924859	2	300	T:0.916667	A:0.0833333
chr28	10925321	2	242	GC:0.92562	G:0.0743802
chr28	10925332	2	250	CCA:0.924	C:0.076
chr28	10925333	2	218	CA:0.678899	C:0.321101
chr28	10925638	2	308	C:0.912338	T:0.0876623
chr28	10925699	2	294	TC:0.982993	T:0.0170068
chr28	10926072	2	290	T:0.424138	C:0.575862
chr28	10926654	2	298	A:0.469799	G:0.530201
chr28	10926698	2	270	TA:0.740741	T:0.259259
chr28	10927281	2	298	C:0.177852	T:0.822148
chr28	10927702	2	300	C:0.973333	T:0.0266667
chr28	10927966	2	296	T:0.175676	C:0.824324
chr28	10928296	4	300	T:0.15	TA:0.413333	TAA:
0.353333	TAAA:0.0833333
chr28	10928307	2	308	A:0.99026	AAC:0.00974026
chr28	10929538	2	304	T:0.657895	C:0.342105
chr28	10929724	2	306	G:0.934641	A:0.0653595
chr28	10930563	2	220	G:0.940909	A:0.0590909
chr28	10930732	2	126	G:0.230159	C:0.769841
chr28	10931128	2	204	CG:0.127451	C:0.872549
chr28	10931223	2	268	T:0.242537	C:0.757463
chr28	10931289	2	282	A:0.900709	G:0.0992908
chr28	10931588	2	306	G:0.379085	C:0.620915
chr28	10931590	2	304	G:0.460526	C:0.539474
chr28	10931625	2	312	T:0.275641	TA:0.724359
chr28	10931626	2	312	T:0.275641	A:0.724359
chr28	10931741	2	308	A:0.412338	C:0.587662
chr28	10931801	2	276	G:0.978261	C:0.0217391
chr28	10931842	2	280	C:0.296429	CAG:0.703571
chr28	10932002	3	318	TAAAAAAAAAAAAACATA:0.311321
T:0.144654	TAAAAAAAAAAAAAAACATA:0.544025
chr28	10932018	2	316	TA:0.471519	T:0.528481
chr28	10932229	3	222	CTGTG:0.445946	C:0.292793
CTG:0.261261
chr28	10932540	2	306	C:0.633987	A:0.366013
chr28	10932838	2	312	C:0.974359	T:0.025641
chr28	10933289	2	308	A:0.782468	G:0.217532
chr28	10933798	2	288	C:0.847222	CA:0.152778
chr28	10933842	2	306	T:0.980392	A:0.0196078
chr28	10934253	2	298	T:0.657718	C:0.342282
chr28	10934424	2	298	T:0.201342	C:0.798658
chr28	10934487	2	302	A:0.225166	T:0.774834
chr28	10934765	2	302	T:0.612583	C:0.387417
chr28	10934913	2	302	A:0.943709	G:0.0562914
chr28	10934991	2	276	TA:0.333333	T:0.666667
chr28	10934992	2	278	A:0.931655	T:0.0683453
chr28	10935306	2	310	T:0.76129	A:0.23871

chr28 10935724 2 296 T:0.898649 C:0.101351
chr28 10935759 3 304 TA:0.684211 T:0.263158
TAA:0.0526316
chr28 10935844 2 304 C:0.904605 T:0.0953947
chr28 10936650 2 306 C:0.761438 T:0.238562
chr28 10936790 2 296 A:0.621622 ACT:0.378378
chr28 10936918 2 294 G:0.959184 A:0.0408163
chr28 10937108 2 290 C:0.251724 T:0.748276
chr28 10937631 2 298 T:0.61745 C:0.38255
chr28 10937655 2 300 G:0.76 A:0.24
chr28 10937749 2 296 C:0.871622 T:0.128378
chr28 10937773 2 296 C:0.942568 CATTT:0.0574324
chr28 10937780 3 302 T:0.251656 TTTTTTTTAA:
0.655629 TTTTTTTTTAA:0.0927152
chr28 10937947 2 290 G:0.62069 A:0.37931
chr28 10938016 2 306 T:0.872549 C:0.127451
chr28 10938156 2 266 G:0.951128 A:0.0488722
chr28 10938218 2 292 CTCTCTTCCCT:0.804795 C:
0.195205
chr28 10938220 2 294 CTCTTCCCT:0.908163 C:
0.0918367
chr28 10938222 3 300 CTTCCCT:0.64 CTCCCT:0.34
C:0.02
chr28 10938223 2 314 T:0.888535 TC:0.111465
chr28 10938226 2 308 C:0.545455 T:0.454545
chr28 10938228 2 310 T:0.493548 TC:0.506452
chr28 10938232 2 296 C:0.733108 G:0.266892
chr28 10938234 2 310 G:0.958065 C:0.0419355
chr28 10938284 2 304 G:0.927632 GAAAGA:0.0723684
chr28 10938529 4 320
TTTTCTTTCTTTCTTTCTTTCTTTCTTTCTTTCTTTCTTTC:0.18125 T:0.68125
TTTTCTTTC:0.08125
TTTTCTTTCTTTCTTTCTTTCTTTCTTTCTTTCTTTCTTTCTTTCTTTC:0.05625
chr28 10938824 2 304 A:0.621711 G:0.378289
chr28 10939148 2 308 C:0.753247 T:0.246753
chr28 10939244 2 316 T:0.411392 G:0.588608
chr28 10939253 2 316 G:0.262658 A:0.737342
chr28 10939350 2 308 A:0.24026 G:0.75974
chr28 10939452 2 300 A:0.913333 G:0.0866667
chr28 10939519 2 300 A:0.966667 G:0.0333333
chr28 10939720 2 298 G:0.996644 A:0.0033557
chr28 10940564 2 300 C:0.99 T:0.01
chr28 10940679 2 296 C:0.760135 T:0.239865
chr28 10941347 2 302 C:0.251656 T:0.748344
chr28 10941594 2 294 C:0.982993 T:0.0170068
chr28 10941647 2 302 G:0.271523 GCTCT:0.728477
chr28 10942341 2 306 G:0.957516 C:0.0424837
chr28 10942723 2 304 C:0.904605 T:0.0953947
chr28 10942928 2 314 T:0.958599 G:0.0414013
chr28 10943113 2 300 G:0.763333 A:0.236667
chr28 10943289 2 310 C:0.73871 G:0.26129
chr28 10943290 2 310 A:0.73871 G:0.26129
chr28 10943378 2 304 A:0.726974 G:0.273026
chr28 10943556 2 282 T:0.758865 C:0.241135

chr28	10943966	2	312	A:0.746795	G:0.253205	
chr28	10943989	2	306	A:0.748366	G:0.251634	
chr28	10944846	2	306	C:0.339869	T:0.660131	
chr28	10944878	2	314	C:0.869427	A:0.130573	
chr28	10945075	2	296	G:0.986486	A:0.0135135	
chr28	10945214	2	296	T:0.280405	C:0.719595	
chr28	10945434	2	284	G:0.369718	A:0.630282	
chr28	10946291	2	290	C:0.386207	T:0.613793	
chr28	10946391	2	274	G:0.335766	A:0.664234	
chr28	10946568	2	190	A:0.2	G:0.8	
chr28	10946632	2	162	A:0.117284	G:0.882716	
chr28	10948332	2	272	C:0.856618	T:0.143382	
chr28	10948399	2	230	C:0.8	CT:0.2	
chr28	10948404	2	230	C:0.8	CCG:0.2	
chr28	10948410	2	230	T:0.8	C:0.2	
chr28	10949030	2	312	ACAGCTGGCC:0.942308	A:0.0576923	
chr28	10949135	2	308	T:0.762987	A:0.237013	
chr28	10949245	2	304	T:0.746711	C:0.253289	
chr28	10949618	2	296	A:0.972973	G:0.027027	
chr28	10949636	2	278	T:0.816547	TA:0.183453	
chr28	10949827	2	306	C:0.901961	T:0.0980392	
chr28	10949911	2	300	A:0.563333	C:0.436667	
chr28	10950059	2	294	C:0.942177	T:0.0578231	
chr28	10950206	2	304	A:0.986842	C:0.0131579	
chr28	10950222	2	304	G:0.763158	A:0.236842	
chr28	10950323	2	292	C:0.917808	G:0.0821918	
chr28	10950328	2	296	C:0.753378	T:0.246622	
chr28	10950411	2	292	C:0.732877	G:0.267123	
chr28	10950548	2	294	C:0.744898	T:0.255102	
chr28	10950605	2	298	C:0.996644	G:0.0033557	
chr28	10951080	2	294	C:0.751701	A:0.248299	
chr28	10951082	2	294	G:0.751701	A:0.248299	
chr28	10951248	2	304	A:0.983553	G:0.0164474	
chr28	10951424	2	316	ACCCTGCATGGTTTAC:0.943038	A:0.056962	
chr28	10951510	2	298	C:0.744966	G:0.255034	
chr28	10952305	2	298	A:0.966443	C:0.033557	
chr28	10952502	3	306	A:0.761438	C:0.00653595	G:0.232026
chr28	10952514	2	306	A:0.993464	G:0.00653595	
chr28	10952562	2	294	C:0.996599	G:0.00340136	
chr28	10952625	2	310	G:0.990323	C:0.00967742	
chr28	10952637	2	302	GTA:0.735099	G:0.264901	
chr28	10952639	2	310	A:0.990323	G:0.00967742	
chr28	10952815	2	302	T:0.738411	C:0.261589	
chr28	10952979	2	298	A:0.761745	G:0.238255	
chr28	10953058	2	298	A:0.919463	T:0.0805369	
chr28	10953067	2	306	T:0.915033	A:0.0849673	
chr28	10953068	2	306	T:0.748366	A:0.251634	
chr28	10953078	2	294	AAAAAT:0.962585	A:0.037415	
chr28	10953092	2	306	A:0.748366	G:0.251634	
chr28	10953185	2	250	C:0.816	CA:0.184	
chr28	10953242	2	224	T:0.78125	C:0.21875	

chr28 10953533 2 204 G:0.838235 T:0.161765
chr28 10953616 2 228 C:0.820175 A:0.179825
chr28 10953773 2 318 G:0.908805 GAGTGTGGA:
0.091195
chr28 10953774 2 318 C:0.889937
CCACCCATGGCATAGCCAGCAT:0.110063
chr28 10954058 2 292 C:0.787671 T:0.212329
chr28 10954059 2 292 A:0.787671 G:0.212329
chr28 10954098 2 308 C:0.233766 CAGGGA:0.766234
chr28 10954143 2 308 T:0.795455 A:0.204545
chr28 10954226 2 292 T:0.804795 TTTTA:0.195205
chr28 10954538 2 288 G:0.600694 C:0.399306
chr28 10954668 2 294 C:0.880952 T:0.119048
chr28 10954711 2 300 A:0.99 AATT:0.01
chr28 10954733 2 294 C:0.979592 T:0.0204082
chr28 10954773 2 300 AGGCG:0.246667 A:0.753333
chr28 10955057 2 302 T:0.731788 C:0.268212
chr28 10955111 2 294 CACG:0.632653 C:0.367347
chr28 10955169 2 298 A:0.355705 G:0.644295
chr28 10955289 2 300 G:0.0833333 C:0.916667
chr28 10955498 2 304 GAC:0.907895 G:0.0921053
chr28 10955581 2 306 G:0.911765 A:0.0882353
chr28 10955860 2 292 G:0.705479 T:0.294521
chr28 10955862 2 292 A:0.705479 G:0.294521
chr28 10956211 2 298 A:0.577181 C:0.422819
chr28 10956220 2 296 C:0.831081 T:0.168919
chr28 10956406 2 312 AGTGATTTTTTTACCTTG:0.557692
A:0.442308
chr28 10956444 2 310 T:0.822581 A:0.177419
chr28 10956472 3 296 C:0.635135 CAG:0.0912162
CAGAGAG:0.273649
chr28 10956634 2 274 AT:0.821168 A:0.178832
chr28 10956653 2 286 A:0.573427 T:0.426573
chr28 10956654 2 286 A:0.573427 T:0.426573
chr28 10956764 2 306 A:0.385621 AT:0.614379
chr28 10956893 2 264 G:0.784091 A:0.215909
chr28 10956988 2 304 A:0.368421 G:0.631579
chr28 10957438 2 292 G:0.917808 A:0.0821918
chr28 10957724 2 298 T:0.627517 G:0.372483
chr28 10957781 2 288 C:0.680556 CT:0.319444
chr28 10957792 2 284 G:0.443662 T:0.556338
chr28 10957793 2 288 GAA:0.680556 G:0.319444
chr28 10957794 2 284 A:0.802817 G:0.197183
chr28 10957804 2 294 A:0.673469 T:0.326531
chr28 10958111 2 312 G:0.419872 A:0.580128
chr28 10958233 2 298 AT:0.681208 A:0.318792
chr28 10958332 2 288 T:0.815972 G:0.184028
chr28 10958419 2 300 C:0.83 A:0.17
chr28 10958530 3 294 CT:0.520408 C:0.29932
CTT:0.180272
chr28 10958549 3 264 CTT:0.443182 C:0.155303
CT:0.401515
chr28 10958909 2 304 GGAC:0.930921 G:0.0690789
chr28 10958913 2 304 TCA:0.930921 T:0.0690789

chr28	10959445	2	306	C:0.405229	T:0.594771	
chr28	10959761	2	298	T:0.979866	A:0.0201342	
chr28	10959870	2	310	G:0.967742	C:0.0322581	
chr28	10959990	2	278	G:0.838129	GAC:0.161871	
chr28	10960491	2	284	TA:0.383803	T:0.616197	
chr28	10960645	2	300	TCTA:0.97	T:0.03	
chr28	10960661	2	296	C:0.547297	T:0.452703	
chr28	10960664	2	296	T:0.547297	C:0.452703	
chr28	10960683	2	220	CT:0.336364	C:0.663636	
chr28	10960719	3	300	C:0.55	CAG:0.213333	CAGAGAG:0.236667
chr28	10960739	2	278	GGC:0.967626	G:0.0323741	
chr28	10961047	2	312	T:0.705128	C:0.294872	
chr28	10961328	2	318	T:0.72327	TTTTTTTTGTTTGTTTTTTTTTTTTTACATCAATAAAATCTTAA:0.27673	
chr28	10961337	2	270	ACC:0.559259	A:0.440741	
chr28	10961571	2	266	T:0.447368	G:0.552632	
chr28	10961978	2	292	C:0.945205	T:0.0547945	
chr28	10962777	2	258	T:0.468992	C:0.531008	
chr28	10962807	2	316	CGATCCTGGAGACCCGGGATCG:0.753165	C:0.246835	
chr28	10962928	2	308	TTAAAAAAAA:0.951299	T:0.0487013	
chr28	10962929	2	310	T:0.909677	TA:0.0903226	
chr28	10962938	2	308	T:0.522727	TA:0.477273	
chr28	10962939	2	308	A:0.99026	AT:0.00974026	
chr28	10963007	2	298	A:0.949664	G:0.0503356	
chr28	10963122	2	292	A:0.914384	G:0.0856164	
chr28	10963320	2	298	CAA:0.818792	C:0.181208	
chr28	10963342	2	300	G:0.546667	C:0.453333	
chr28	10963556	2	306	C:0.79085	CT:0.20915	
chr28	10963558	2	318	T:0.902516	TTCTTTC:0.0974843	
chr28	10964557	2	294	GC:0.816327	G:0.183673	
chr28	10964800	2	292	C:0.787671	T:0.212329	
chr28	10964924	2	308	T:0.974026	C:0.025974	
chr28	10965013	2	290	C:0.910345	CT:0.0896552	
chr28	10965016	2	302	T:0.589404	TTTTC:0.410596	
chr28	10965062	2	306	C:0.401961	G:0.598039	
chr28	10965153	2	288	G:0.975694	A:0.0243056	
chr28	10965483	2	276	A:0.637681	ACC:0.362319	
chr28	10965485	2	290	C:0.786207	CCT:0.213793	
chr28	10965491	2	282	C:0.638298	CCA:0.361702	
chr28	10965492	2	290	A:0.786207	C:0.213793	
chr28	10965784	2	276	AAGG:0.884058	A:0.115942	
chr28	10966005	2	260	A:0.807692	G:0.192308	
chr28	10966282	2	186	G:0.774194	C:0.225806	
chr28	10966579	2	234	G:0.106838	A:0.893162	
chr28	10966622	2	280	A:0.889286	G:0.110714	
chr28	10966652	2	272	G:0.926471	A:0.0735294	
chr28	10966710	2	262	T:0.89313	C:0.10687	
chr28	10966731	2	284	G:0.806338	T:0.193662	
chr28	10966732	2	284	A:0.806338	T:0.193662	
chr28	10966981	2	304	G:0.457237	A:0.542763	
chr28	10967204	2	300	G:0.896667	A:0.103333	

chr28 10967625 2 316 C:0.490506 T:0.509494
chr28 10967688 2 314 A:0.16879 G:0.83121
chr28 10967758 2 280 G:0.817857 C:0.182143
chr28 10967940 2 304 T:0.523026 C:0.476974
chr28 10967997 2 306 T:0.375817 C:0.624183
chr28 10968267 2 312 A:0.852564 T:0.147436
chr28 10968892 2 314 A:0.990446 G:0.00955414
chr28 10969042 2 312 T:0.964744 C:0.0352564
chr28 10969128 2 314 G:0.490446 A:0.509554
chr28 10969133 2 316 T:0.325949 C:0.674051
chr28 10969630 2 298 A:0.513423 G:0.486577
chr28 10969949 2 306 A:0.503268 ATTGT:0.496732
chr28 10969976 2 304 G:0.822368 A:0.177632
chr28 10970109 2 292 G:0.945205 A:0.0547945
chr28 10970473 2 298 T:0.325503 C:0.674497
chr28 10970631 2 304 T:0.473684 A:0.526316
chr28 10970698 2 304 G:0.476974 A:0.523026
chr28 10971002 2 292 A:0.989726 C:0.010274
chr28 10971194 2 294 CAG:0.496599 C:0.503401
chr28 10971312 2 292 C:0.866438 T:0.133562
chr28 10971398 2 304 G:0.947368 A:0.0526316
chr28 10972104 2 296 G:0.466216 A:0.533784
chr28 10972185 4 302 ATTT:0.195364 AT:0.201987
ATT:0.543046 A:0.0596026
chr28 10972211 4 318 CTTTATTTATTTATTTATTTATTTATTTATTTA:
0.292453 C:0.248428 CTTTATTTA:0.119497 CTTTATTTATTTA:
0.339623
chr28 10972309 2 300 T:0.47 C:0.53
chr28 10972350 2 290 C:0.510345 T:0.489655
chr28 10972364 2 292 C:0.815068 T:0.184932
chr28 10972567 2 308 G:0.814935 A:0.185065
chr28 10972570 2 306 G:0.493464 T:0.506536
chr28 10972747 2 314 GCCATCTTTTTCTGAAAAAGC:0.850318
G:0.149682
chr28 10972778 2 300 T:0.33 TC:0.67
chr28 10972787 2 300 G:0.33 GTGGT:0.67
chr28 10973528 2 300 G:0.526667 A:0.473333
chr28 10973608 2 302 A:0.539735 G:0.460265
chr28 10973714 2 294 G:0.972789 T:0.0272109
chr28 10973828 2 286 C:0.888112 T:0.111888
chr28 10974020 2 288 C:0.496528 A:0.503472
chr28 10974066 2 280 C:0.435714 T:0.564286
chr28 10974133 2 284 C:0.827465 T:0.172535
chr28 10974294 2 306 C:0.51634 T:0.48366
chr28 10974322 2 294 C:0.527211 T:0.472789
chr28 10974417 2 302 G:0.94702 A:0.0529801
chr28 10974622 2 312 T:0.467949 TC:0.532051
chr28 10974634 2 312 T:0.467949 C:0.532051
chr28 10974958 2 320 T:0.49375 TTTC:0.50625
chr28 10975024 3 294 GTTTT:0.636054 G:0.217687
GT:0.146259
chr28 10975261 2 306 G:0.96732 A:0.0326797
chr28 10975398 2 304 T:0.506579 C:0.493421
chr28 10975516 2 304 A:0.503289 G:0.496711

chr28	10975656	2	312	G:0.820513	A:0.179487		
chr28	10975693	2	304	AT:0.851974	A:0.148026		
chr28	10975698	2	306	T:0.885621	TA:0.114379		
chr28	10975702	4	306	TAA:0.496732	T:0.117647	AAA:0.147059	TA:0.238562
chr28	10975704	2	302	A:0.94702	T:0.0529801		
chr28	10975763	2	284	G:0.665493	T:0.334507		
chr28	10976105	2	136	GA:0	G:1		
chr28	10976177	2	224	TTATG:0.754464	T:0.245536		
chr28	10976274	2	296	TC:0.510135	T:0.489865		
chr28	10976373	2	286	C:0.842657	T:0.157343		
chr28	10976533	2	226	C:0.920354	CAT:0.079646		
chr28	10976599	2	280	G:0.460714	C:0.539286		
chr28	10976600	2	280	A:0.460714	T:0.539286		
chr28	10976984	2	288	T:0.121528	C:0.878472		
chr28	10977233	3	302	C:0.645695	CAGAGAG:0.208609	CAGAGAGAGAG:0.145695	
chr28	10977288	2	250	T:0.504	C:0.496		
chr28	10977367	2	286	G:0.695804	A:0.304196		
chr28	10977383	2	284	CA:0.989437	C:0.0105634		
chr28	10977384	2	290	A:0.517241	G:0.482759		
chr28	10977394	2	290	T:0.517241	A:0.482759		
chr28	10977396	2	290	T:0.517241	A:0.482759		
chr28	10977449	2	244	CGACA:0.827869	C:0.172131		
chr28	10977452	3	280	CAG:0.771429	C:0.132143	GAG:0.0964286	
chr28	10977531	2	250	CATG:0.516	C:0.484		
chr28	10977593	2	274	T:0.49635	C:0.50365		
chr28	10978165	3	302	T:0.347682	C:0.155629	G:0.496689	
chr28	10978203	2	296	G:0.810811	T:0.189189		
chr28	10978372	2	304	G:0.996711	A:0.00328947		
chr28	10978381	2	312	A:0.945513	C:0.0544872		
chr28	10978429	2	304	C:0.467105	CA:0.532895		
chr28	10978430	2	304	C:0.901316	T:0.0986842		
chr28	10978442	2	304	G:0.467105	GGTA:0.532895		
chr28	10978513	2	286	A:0.486014	C:0.513986		
chr28	10978703	2	278	A:0.492806	C:0.507194		
chr28	10979470	2	304	C:0.976974	T:0.0230263		
chr28	10979777	2	314	G:0.496815	GTCTT:0.503185		
chr28	10979849	2	312	T:0.916667	A:0.0833333		
chr28	10979907	2	302	G:0.937086	A:0.0629139		
chr28	10979981	2	304	G:0.861842	A:0.138158		
chr28	10980312	2	300	G:0.876667	T:0.123333		
chr28	10980412	2	306	T:0.820261	TA:0.179739		
chr28	10980524	2	310	A:0.312903	G:0.687097		
chr28	10980567	2	300	T:0.483333	A:0.516667		
chr28	10980625	2	294	T:0.326531	C:0.673469		
chr28	10981037	2	298	A:0.530201	G:0.469799		
chr28	10981304	2	306	T:0.346405	C:0.653595		
chr28	10981413	2	296	T:0.189189	C:0.810811		
chr28	10981744	2	302	T:0.34106	C:0.65894		
chr28	10981920	2	312	C:0.839744	T:0.160256		
chr28	10982003	2	302	A:0.758278	G:0.241722		

chr28	10982720	2	298	A:0.973154	T:0.0268456		
chr28	10983100	2	294	G:0.554422	A:0.445578		
chr28	10983107	2	296	G:0.351351	A:0.648649		
chr28	10983144	2	298	C:0.889262	CTTCT:0.110738		
chr28	10983198	3	308	CAGAGAG:0.467532	C:0.383117	CAGAGAGAG:0.149351	
chr28	10983293	2	276	C:0.82971	A:0.17029		
chr28	10983352	2	296	CCTT:0.844595	C:0.155405		
chr28	10983401	2	296	A:0.510135	G:0.489865		
chr28	10983418	2	302	T:0.503311	G:0.496689		
chr28	10983603	2	298	G:0.503356	A:0.496644		
chr28	10983637	2	304	T:0.503289	C:0.496711		
chr28	10983774	2	306	A:0.496732	AGTCC:0.503268		
chr28	10983818	2	314	G:0.850318	A:0.149682		
chr28	10983977	2	290	G:0.893103	A:0.106897		
chr28	10984009	2	298	G:0.503356	A:0.496644		
chr28	10984411	2	300	C:0.466667	T:0.533333		
chr28	10984568	2	304	C:0.532895	A:0.467105		
chr28	10984869	2	300	G:0.453333	A:0.546667		
chr28	10984997	2	298	T:0.459732	C:0.540268		
chr28	10985087	2	298	C:0.513423	T:0.486577		
chr28	10985273	4	314	CTG:0.484076	C:0.264331	CTGTG:0.0477707	CTGTGTGTGTGTGTGTGTGTGTGTG:0.203822
chr28	10985285	2	316	G:0.886076	GTGTGTGTGTGTGTGTGTGTGTGTGTGTGTGTGT:0.113924		
chr28	10985313	2	296	A:0.537162	G:0.462838		
chr28	10985337	2	296	A:0.537162	G:0.462838		
chr28	10985486	2	298	A:0.47651	C:0.52349		
chr28	10985523	2	290	T:0.517241	C:0.482759		
chr28	10985564	2	290	C:0.47931	CT:0.52069		
chr28	10985604	2	310	AT:0.509677	A:0.490323		
chr28	10985656	2	314	T:0.33758	C:0.66242		
chr28	10985748	2	296	C:0.516892	CT:0.483108		
chr28	10985921	2	304	A:0.3125	AT:0.6875		
chr28	10986385	2	300	C:0.826667	T:0.173333		
chr28	10986427	2	296	T:0.506757	C:0.493243		
chr28	10986678	2	292	T:0.479452	G:0.520548		
chr28	10986718	2	298	A:0.493289	G:0.506711		
chr28	10986842	2	306	G:0.826797	A:0.173203		
chr28	10986885	2	306	G:0.490196	T:0.509804		
chr28	10987378	2	306	C:0.493464	A:0.506536		
chr28	10987477	2	296	T:0.827703	A:0.172297		
chr28	10987505	2	302	T:0.543046	G:0.456954		
chr28	10987590	2	308	T:0.512987	C:0.487013		
chr28	10987599	2	308	C:0.512987	G:0.487013		
chr28	10987920	2	286	A:0.493007	G:0.506993		
chr28	10987986	2	276	T:0.521739	C:0.478261		
chr28	10988012	2	288	C:0.857639	T:0.142361		
chr28	10988453	2	288	C:0.815972	CG:0.184028		
chr28	10988522	2	298	C:0.88255	T:0.11745		
chr28	10988580	2	298	A:0.848993	G:0.151007		
chr28	10988882	2	308	C:0.831169	T:0.168831		
chr28	10988978	2	306	CT:0.320261	C:0.679739		
chr28	10989264	2	302	C:0.688742	T:0.311258		

chr28	10989268	2	302	C:0.688742	T:0.311258
chr28	10989464	2	304	A:0.845395	C:0.154605
chr28	10989866	2	300	C:0.833333	A:0.166667
chr28	10990280	2	290	A:0.934483	T:0.0655172
chr28	10990637	2	302	T:0.877483	G:0.122517
chr28	10990840	2	308	A:0.834416	G:0.165584
chr28	10991115	2	304	TTTC:0.901316	T:0.0986842
chr28	10991142	3	290	T:0.703448	TA:0.158621
TAA:0.137931					
chr28	10991340	2	294	T:0.704082	C:0.295918
chr28	10991590	2	308	G:0.993506	A:0.00649351
chr28	10991684	2	304	A:0.855263	T:0.144737
chr28	10991696	2	304	T:0.855263	C:0.144737
chr28	10991738	2	298	A:0.842282	G:0.157718
chr28	10991766	2	302	A:0.821192	G:0.178808
chr28	10991773	2	302	G:0.821192	A:0.178808
chr28	10991800	2	296	A:0.550676	G:0.449324
chr28	10991874	2	302	A:0.81457	C:0.18543
chr28	10992336	2	286	C:0.839161	T:0.160839
chr28	10992547	2	262	G:0.851145	A:0.148855
chr28	10992571	2	274	C:0.850365	A:0.149635
chr28	10992572	2	274	CG:0.850365	C:0.149635
chr28	10992573	2	272	G:0.893382	C:0.106618
chr28	10992700	2	288	T:0.118056	C:0.881944
chr28	10992714	2	290	C:0.286207	T:0.713793
chr28	10992715	2	288	G:0.836806	A:0.163194
chr28	10992822	2	282	G:0.847518	GC:0.152482
chr28	10993329	2	304	GC:0.871711	G:0.128289
chr28	10993330	2	302	CCCCAG:0.261589	C:0.738411
chr28	10993332	2	302	C:0.877483	CTG:0.122517
chr28	10993333	2	302	CAG:0.877483	C:0.122517
chr28	10993335	2	304	G:0.871711	GC:0.128289
chr28	10993525	2	268	A:0.955224	G:0.0447761
chr28	10993669	2	240	A:0.920833	G:0.0791667
chr28	10993730	2	274	G:0.967153	GGTGT:0.0328467
chr28	10993759	2	222	GTA:0.882883	G:0.117117
chr28	10993761	2	220	A:0.918182	G:0.0818182
chr28	10993763	2	228	A:0.95614	G:0.0438596
chr28	10993946	2	292	GA:0.280822	G:0.719178
chr28	10994209	2	294	TA:0.806122	T:0.193878
chr28	10994309	2	294	C:0.721088	T:0.278912
chr28	10994311	2	294	A:0.721088	G:0.278912
chr28	10994906	2	296	G:0.108108	A:0.891892
chr28	10995117	2	292	G:0.996575	A:0.00342466
chr28	10995247	2	290	T:0.317241	G:0.682759
chr28	10995395	2	292	C:0.119863	G:0.880137
chr28	10995495	2	296	A:0.692568	G:0.307432
chr28	10995595	2	274	A:0.894161	C:0.105839
chr28	10995777	2	212	CTG:0.839623	C:0.160377
chr28	10996035	2	268	G:0.873134	C:0.126866
chr28	10996211	2	294	A:0.894558	AG:0.105442
chr28	10996475	2	300	G:0.97 A:0.03	
chr28	10996540	2	292	A:0.0993151	T:0.900685
chr28	10996665	2	298	C:0.812081	T:0.187919

chr28 10996729 2 298 C:0.332215 T:0.667785
chr28 10996829 2 292 A:0.876712 G:0.123288
chr28 10996877 6 316 CGTGTGTGTGTGTGTGT:0.243671
C:0.367089 CGTGTGT:0.107595 CGTGTGTGT:0.0791139
CGTGTGTGTGTGTGT:0.110759 CGTGTGTGTGTGTGTGTGT:0.0917722
chr28 10996947 2 288 A:0.71875 G:0.28125
chr28 10997231 2 296 A:0.85473 G:0.14527
chr28 10997649 2 300 G:0.163333 GT:0.836667
chr28 10997759 2 300 A:0.1 G:0.9
chr28 10997837 2 298 T:0.691275 C:0.308725
chr28 10997840 2 300 GCC:0.83 G:0.17
chr28 10997871 2 300 C:0.81 G:0.19
chr28 10997960 2 310 GGCTCTCTCTCCCCT:0.132258 G:
0.867742
chr28 10998658 2 294 ATG:0.982993 A:0.0170068
chr28 10998674 2 298 G:0.701342 GT:0.298658
chr28 10998675 3 312 G:0.714744 GT:0.173077
GTGTGTGT:0.112179
chr28 10998719 2 288 C:0.892361 T:0.107639
chr28 10998878 2 264 A:0.784091 G:0.215909
chr28 10998898 2 270 T:0.751852 C:0.248148
chr28 10998904 2 270 T:0.751852 C:0.248148
chr28 10998976 2 260 G:0.111538 C:0.888462
chr28 10999062 2 208 C:0.230769 CG:0.769231
chr28 10999116 2 182 CGT:0.521978 C:0.478022
chr28 10999539 2 70 G:0 GT:1
chr28 11000448 2 272 G:0.595588 A:0.404412
chr28 11000873 2 306 G:0.875817 T:0.124183
chr28 11000897 2 308 G:0.938312 A:0.0616883
chr28 11001070 2 290 G:0.986207 T:0.0137931
chr28 11001281 2 300 G:0.143333 A:0.856667
chr28 11001339 2 304 A:0.131579 G:0.868421
chr28 11001408 2 312 GTTCC:0.153846 G:0.846154
chr28 11001623 2 304 T:0.983553 C:0.0164474
chr28 11001813 2 292 T:0.119863 C:0.880137
chr28 11001908 2 296 C:0.922297 T:0.0777027
chr28 11001982 2 298 C:0.869128 T:0.130872
chr28 11002146 2 294 A:0.986395 AC:0.0136054
chr28 11002454 2 310 AAACAAC:0.645161 A:0.354839
chr28 11002457 2 314 C:0.5 T:0.5
chr28 11002463 2 310 C:0.645161 T:0.354839
chr28 11002469 2 314 CAACAACAAT:0.5 C:0.5
chr28 11003002 4 306 A:0.147059 AT:0.297386
ATT:0.513072 ATTTT:0.0424837
chr28 11003098 2 308 A:0.912338
AACTAAATTTTGCTTATTAATAAAATTTTAAAC:0.0876623
chr28 11003273 2 304 AAAAT:0.986842 A:0.0131579
chr28 11003288 2 316 A:0.96519 AT:0.0348101
chr28 11003289 2 306 TAAA:0.98366 T:0.0163399
chr28 11003290 2 316 A:0.96519 AATT:0.0348101
chr28 11003293 2 306 TA:0.98366 T:0.0163399
chr28 11003294 2 306 A:0.830065 T:0.169935
chr28 11003334 2 294 A:0.986395 C:0.0136054
chr28 11003363 2 302 A:0.874172 G:0.125828

chr28 11003468 2 304 GTTTCTTTTCTTCC:0.9375 G:0.0625
chr28 11003555 2 314 T:0.528662 TCTA:0.471338
chr28 11003575 2 314 A:0.859873 G:0.140127
chr28 11003578 2 314 C:0.993631 T:0.00636943
chr28 11003603 2 282 C:0.804965 CT:0.195035
chr28 11003607 2 312 T:0.990385 C:0.00961538
chr28 11003633 3 304 C:0.161184 CAGAGAG:0.819079
CAGAGAGAGAG:0.0197368
chr28 11003635 2 316 G:0.974684 GAGAGAA:0.0253165
chr28 11003644 2 294 T:0.142857 TGA:0.857143
chr28 11003846 2 284 C:0.179577 CTT:0.820423
chr28 11004025 2 282 G:0.975177 A:0.0248227
chr28 11004046 2 288 G:0.982639 A:0.0173611
chr28 11004192 2 296 C:0.898649 T:0.101351
chr28 11004311 2 284 G:0.116197 A:0.883803
chr28 11004606 2 296 A:0.972973 G:0.027027
chr28 11004722 2 298 G:0.88255 A:0.11745
chr28 11004801 2 304 C:0.967105 A:0.0328947
chr28 11005123 2 302 T:0.115894 C:0.884106
chr28 11005235 2 290 C:0.103448 T:0.896552
chr28 11005298 2 300 A:0.89 G:0.11
chr28 11005327 2 310 G:0.977419 C:0.0225806
chr28 11005435 2 298 T:0.909396 C:0.090604
chr28 11005509 2 304 G:0.980263 A:0.0197368
chr28 11005663 2 302 G:0.460265 C:0.539735
chr28 11005676 2 306 G:0.0849673 A:0.915033
chr28 11006028 2 304 T:0.865132 C:0.134868
chr28 11006142 2 300 C:0.136667 T:0.863333
chr28 11006203 2 296 T:0.121622 C:0.878378
chr28 11006223 4 318 C:0.116352 CTGAA:0.0125786
CTGAATGAACGAA:0.820755 CTGAATGAACGAATGAA:0.0503145
chr28 11006246 2 318 G:0.128931 A:0.871069
chr28 11006397 2 302 C:0.864238 CT:0.135762
chr28 11006726 2 288 T:0.121528 C:0.878472
chr28 11006737 2 286 AC:0.884615 A:0.115385
chr28 11006739 2 278 C:0.23741 T:0.76259
chr28 11006741 2 286 C:0.884615 T:0.115385
chr28 11006750 2 284 G:0.112676 A:0.887324
chr28 11006751 2 284 C:0.112676 T:0.887324
chr28 11006837 2 296 A:0.763514 C:0.236486
chr28 11006936 2 304 C:0.118421 T:0.881579
chr28 11006979 2 300 A:0.906667 G:0.0933333
chr28 11006991 2 302 A:0.907285 G:0.0927152
chr28 11007002 2 298 G:0.916107 GGA:0.0838926
chr28 11007058 2 294 A:0.884354 T:0.115646
chr28 11007138 2 306 C:0.888889 T:0.111111
chr28 11007215 2 304 T:0.0921053 C:0.907895
chr28 11007601 2 286 A:0.881119 C:0.118881
chr28 11007664 2 284 A:0.897887 T:0.102113
chr28 11007697 2 298 G:0.872483 T:0.127517
chr28 11007727 2 290 AG:0.868966 A:0.131034
chr28 11007782 2 300 A:0.89 G:0.11
chr28 11007923 2 292 C:0.136986 G:0.863014
chr28 11008346 2 290 T:0.12069 C:0.87931

chr28	11008356	2	304	G:0.134868	GC:0.865132
chr28	11008376	2	294	A:0.139456	G:0.860544
chr28	11008391	2	294	T:0.108844	A:0.891156
chr28	11008404	2	292	T:0.89726	G:0.10274
chr28	11008522	2	294	A:0.0714286	G:0.928571
chr28	11008580	2	308	G:0.12013	A:0.87987
chr28	11008589	2	308	A:0.12013	G:0.87987
chr28	11008669	2	296	AT:0.108108	A:0.891892
chr28	11008980	2	298	A:0.852349	G:0.147651
chr28	11008999	2	300	G:0.13 A:0.87	
chr28	11009011	2	294	AG:0.97619	A:0.0238095
chr28	11009048	2	284	T:0.897887	C:0.102113
chr28	11009258	2	284	G:0.830986	A:0.169014
chr28	11009314	2	284	G:0.883803	A:0.116197
chr28	11009484	2	308	G:0.113636	C:0.886364
chr28	11009729	2	302	G:0.983444	A:0.0165563
chr28	11009792	2	292	T:0.106164	C:0.893836
chr28	11009928	2	298	A:0.993289	C:0.00671141
chr28	11010161	2	284	A:0.116197	G:0.883803
chr28	11010227	2	288	C:0.902778	A:0.0972222
chr28	11010228	2	290	A:0.996552	C:0.00344828
chr28	11010301	2	272	G:0.110294	C:0.889706
chr28	11010623	2	290	C:0.103448	T:0.896552
chr28	11010763	2	296	A:0.891892	G:0.108108
chr28	11010769	2	296	A:0.891892	G:0.108108
chr28	11010775	2	300	AAG:0.966667	A:0.0333333
chr28	11010793	2	300	C:0.966667	T:0.0333333
chr28	11010810	2	316	C:0.892405	CCTTGGG:0.107595
chr28	11010926	2	310	T:0.893548	C:0.106452
chr28	11010949	2	310	A:0.893548	T:0.106452
chr28	11011061	2	286	C:0.982517	T:0.0174825
chr28	11011219	2	288	T:0.111111	C:0.888889
chr28	11011252	2	296	G:0.891892	A:0.108108
chr28	11011267	2	298	G:0.147651	A:0.852349
chr28	11011340	3	314	TAA:0.124204	T:0.850318
TAAAAAA:0.0254777					
chr28	11011350	2	312	TAAAA:0.144231	T:0.855769
chr28	11011559	2	278	G:0.104317	A:0.895683
chr28	11011876	2	274	C:0.967153	T:0.0328467
chr28	11011927	2	282	C:0.237589	CCT:0.762411
chr28	11011978	3	282	T:0.379433	TA:0.336879
TAA:0.283688					
chr28	11012037	2	310	A:0.170968	ATGTAGGCAGAGG:
0.829032					
chr28	11012378	2	304	A:0.871711	G:0.128289
chr28	11012386	2	304	A:0.871711	G:0.128289
chr28	11012542	2	308	G:0.905844	C:0.0941558
chr28	11013044	2	296	A:0.118243	C:0.881757
chr28	11013094	2	280	G:0.878571	T:0.121429
chr28	11013431	2	212	G:0.924528	T:0.0754717
chr28	11013511	2	272	C:0.0919118	T:0.908088
chr28	11013592	2	206	G:0.81068	A:0.18932
chr28	11013647	2	252	TTG:0.130952	T:0.869048
chr28	11013660	2	252	G:0.130952	C:0.869048

chr28 11014025 2 300 C:0.126667 G:0.873333
chr28 11014797 2 266 A:0.913534 G:0.0864662
chr28 11015379 2 298 T:0.107383 C:0.892617
chr28 11015536 2 298 C:0.989933 T:0.0100671
chr28 11015724 2 290 C:0.972414 T:0.0275862
chr28 11015901 2 292 A:0.979452 T:0.0205479
chr28 11015942 4 304 C:0.746711 CAG:0.0888158
CAGAGAG:0.111842 CAGAGAGAG:0.0526316
chr28 11015944 4 314 C:0.394904 G:0.254777
CAGAGAGAG:0.226115 CAGAGAGAGAG:0.124204
chr28 11015946 2 294 C:0.258503 G:0.741497
chr28 11016331 2 286 C:0.846154 T:0.153846
chr28 11016384 2 290 C:0.889655 T:0.110345
chr28 11016567 2 296 C:0.996622 T:0.00337838
chr28 11016701 2 226 GGGT:0.743363 G:0.256637
chr28 11016702 2 226 GGT:0.283186 G:0.716814
chr28 11017185 2 290 T:0.97931 C:0.0206897
chr28 11017239 2 280 G:0.446429 A:0.553571
chr28 11017242 2 284 G:0.514085 A:0.485915
chr28 11017398 2 284 T:0.975352 C:0.0246479
chr28 11017488 2 298 C:0.513423 G:0.486577
chr28 11017968 2 308 C:0.49026 T:0.50974
chr28 11018133 2 292 C:0.513699 T:0.486301
chr28 11018217 2 304 C:0.947368 T:0.0526316
chr28 11018324 2 296 GT:0.993243 G:0.00675676
chr28 11018561 2 300 C:0.773333 T:0.226667
chr28 11018591 2 292 C:0.972603 T:0.0273973
chr28 11018792 2 294 A:0.503401 C:0.496599
chr28 11018807 3 314 TTTTA:0.665605 T:0.216561
TTTTATTTA:0.117834
chr28 11018811 2 308 AT:0.974026 A:0.025974
chr28 11019252 2 304 G:0.990132 A:0.00986842
chr28 11019519 2 302 A:0.364238 G:0.635762
chr28 11019735 2 296 C:0.0878378 T:0.912162
chr28 11020123 2 302 G:0.771523 A:0.228477
chr28 11020179 2 304 T:0.871711 C:0.128289
chr28 11020202 2 308 GT:0.772727 G:0.227273
chr28 11020349 2 256 G:0.207031 GT:0.792969
chr28 11020376 2 254 C:0.748031 T:0.251969
chr28 11020781 2 268 G:0.966418 A:0.0335821
chr28 11021012 2 296 G:0.483108 T:0.516892
chr28 11021017 2 296 G:0.483108 A:0.516892
chr28 11021468 2 290 G:0.968966 A:0.0310345
chr28 11021915 2 302 T:0.0927152 G:0.907285
chr28 11022233 2 302 C:0.519868 T:0.480132
chr28 11022346 2 294 A:0.734694 G:0.265306
chr28 11022767 2 270 A:0.97037 G:0.0296296
chr28 11023439 2 306 G:0.496732 T:0.503268
chr28 11023556 2 308 C:0.876623 T:0.123377
chr28 11023666 2 298 T:0.510067 C:0.489933
chr28 11023689 2 296 T:0.516892 C:0.483108
chr28 11023703 2 294 G:0.911565 A:0.0884354
chr28 11023783 2 306 C:0.996732 T:0.00326797
chr28 11024066 2 300 G:0.48 C:0.52

chr28	11024127	2	306	T:0.973856	A:0.0261438
chr28	11024250	2	308	T:0.461039	G:0.538961
chr28	11024596	2	292	C:0.989726	T:0.010274
chr28	11024783	2	292	A:0.496575	AC:0.503425
chr28	11025055	2	302	C:0.976821	T:0.0231788
chr28	11025116	2	294	T:0.493197	G:0.506803
chr28	11025149	2	300	CA:0.986667	C:0.0133333
chr28	11025326	2	308	AG:0.779221	A:0.220779
chr28	11025647	2	300	G:0.876667	A:0.123333
chr28	11025713	2	312	C:0.503205	T:0.496795
chr28	11026043	2	298	C:0.446309	CTA:0.553691
chr28	11026217	2	294	G:0.520408	GC:0.479592
chr28	11026485	2	298	G:0.936242	A:0.0637584
chr28	11026700	2	298	T:0.536913	C:0.463087
chr28	11026905	2	294	G:0.959184	A:0.0408163
chr28	11027254	2	308	G:0.438312	A:0.561688
chr28	11027319	2	312	C:0.974359	T:0.025641
chr28	11027331	2	312	TG:0.496795	T:0.503205
chr28	11027534	2	304	C:0.980263	T:0.0197368
chr28	11027768	2	298	C:0.577181	G:0.422819
chr28	11027837	2	296	T:0.530405	C:0.469595
chr28	11028083	2	296	T:0.679054	G:0.320946
chr28	11028211	2	308	T:0.935065	C:0.0649351
chr28	11028231	2	308	A:0.935065	G:0.0649351
chr28	11028232	2	308	G:0.935065	C:0.0649351
chr28	11028451	2	300	T:0.2 C:0.8	
chr28	11028454	2	300	C:0.2 T:0.8	
chr28	11028918	2	314	A:0.764331	G:0.235669
chr28	11029112	2	298	C:0.95302	T:0.0469799
chr28	11029411	2	316	C:0.762658	T:0.237342
chr28	11029473	2	298	T:0.761745	A:0.238255
chr28	11029699	2	260	C:0.753846	T:0.246154
chr28	11029723	2	252	T:0.988095	C:0.0119048
chr28	11029790	2	146	G:0.479452	C:0.520548
chr28	11029936	2	244	A:0.532787	G:0.467213
chr28	11030002	2	254	A:0.496063	G:0.503937
chr28	11030312	2	286	T:0.479021	C:0.520979
chr28	11030318	2	288	G:0.965278	T:0.0347222
chr28	11030336	2	294	A:0.959184	G:0.0408163
chr28	11030502	2	304	C:0.447368	CT:0.552632
chr28	11030527	2	308	C:0.954545	T:0.0454545
chr28	11030746	2	300	T:0.516667	C:0.483333
chr28	11031145	2	300	G:0.473333	A:0.526667
chr28	11031243	2	296	A:0.496622	G:0.503378
chr28	11031297	2	302	A:0.440397	G:0.559603
chr28	11031331	2	306	C:0.454248	T:0.545752
chr28	11031449	2	304	G:0.697368	A:0.302632
chr28	11031494	2	300	C:0.493333	G:0.506667
chr28	11031525	2	308	G:0.457792	A:0.542208
chr28	11031693	2	302	G:0.993377	A:0.00662252
chr28	11031743	2	298	A:0.536913	C:0.463087
chr28	11031794	2	306	T:0.990196	C:0.00980392
chr28	11031909	2	294	T:0.530612	A:0.469388
chr28	11031910	2	294	C:0.530612	T:0.469388

chr28 11031948 2 304 T:0.503289 C:0.496711
chr28 11031967 2 300 C:0.47 T:0.53
chr28 11032012 2 310 C:0.970968 T:0.0290323
chr28 11032121 2 306 GTATAAGA:0.535948 G:
0.464052
chr28 11032172 2 296 T:0.456081 C:0.543919
chr28 11032245 2 296 T:0.52027 G:0.47973
chr28 11032317 2 192 T:0.307292 A:0.692708
chr28 11032686 2 290 C:0.503448 CTTT:0.496552
chr28 11032702 4 320 TTTTA:0.315625 T:0.175
TTTTATTTA:0.315625 TTTTATTTATTTATTTATTTA:0.19375
chr28 11032740 2 286 C:0.503497 CAG:0.496503
chr28 11032795 2 284 G:0.510563 A:0.489437
chr28 11032824 2 288 C:0.506944 T:0.493056
chr28 11032875 2 284 T:0.482394 C:0.517606
chr28 11032919 2 320
TCTTTATCTAGTATGATCTCTAGACACTTTCTTTGC:0.978125 T:0.021875
chr28 11032957 2 320 T:0.978125 C:0.021875
chr28 11033126 2 304 C:0.483553 T:0.516447
chr28 11033139 2 304 G:0.483553 A:0.516447
chr28 11033239 2 312 A:0.990385 C:0.00961538
chr28 11033276 2 306 T:0.506536 C:0.493464
chr28 11033358 2 306 G:0.970588 A:0.0294118
chr28 11033557 2 302 C:0.748344 T:0.251656
chr28 11033749 2 312 T:0.477564 G:0.522436
chr28 11033753 2 312 T:0.477564 C:0.522436
chr28 11034008 2 306 C:0.506536 T:0.493464
chr28 11034015 2 308 G:0.146104 A:0.853896
chr28 11034065 2 308 C:0.99026 T:0.00974026
chr28 11034156 2 306 C:0.764706 T:0.235294
chr28 11034325 2 302 G:0.463576 A:0.536424
chr28 11034469 2 250 C:0.696 CTTT:0.304
chr28 11034523 2 288 CTT:0.496528 C:0.503472
chr28 11034636 2 290 C:0.503448 T:0.496552
chr28 11034645 2 308 G:0.954545 GT:0.0454545
chr28 11034647 2 308 GCCCCAACC:0.954545 G:
0.0454545
chr28 11034970 2 300 G:0.763333 A:0.236667
chr28 11035099 2 314 G:0.522293 A:0.477707
chr28 11035355 2 306 G:0.973856 A:0.0261438
chr28 11035439 2 308 C:0.977273 T:0.0227273
chr28 11035764 2 298 G:0.493289 A:0.506711
chr28 11035877 2 306 C:0.509804 T:0.490196
chr28 11036037 2 302 C:0.983444 T:0.0165563
chr28 11036374 2 300 C:0.253333 T:0.746667
chr28 11036470 2 308 G:0.866883 A:0.133117
chr28 11036491 2 304 C:0.884868 T:0.115132
chr28 11036637 2 310 C:0.487097 A:0.512903
chr28 11036639 2 310 T:0.903226 G:0.0967742
chr28 11036705 2 308 G:0.762987 A:0.237013
chr28 11036874 2 290 C:0.0827586 G:0.917241
chr28 11037064 2 310 G:0.512903 A:0.487097
chr28 11037211 2 308 T:0.902597 C:0.0974026
chr28 11037431 2 308 G:0.75 A:0.25

chr28	11037491	2	302	CTTTTCT:0.847682	C:0.152318
chr28	11037506	2	304	T:0.911184	C:0.0888158
chr28	11037508	2	304	C:0.911184	T:0.0888158
chr28	11037518	2	304	C:0.911184	T:0.0888158
chr28	11037522	4	320	C:0.659375	T:0.084375
CTTTCTTTCTTTCTTCTTTTTTTCTTTCTTTCT:0.23125
CTTTCTTTCTTTCTTTCTTTCTTTCTTCTTTTTTTCTTTCTTTCTT:0.025
chr28	11037524	2	304	T:0.911184	C:0.0888158
chr28	11037538	2	320	A:0.74375	T:0.25625
chr28	11037815	2	302	G:0.5	C:0.5
chr28	11037838	2	310	C:0.932258	T:0.0677419
chr28	11037916	2	298	A:0.768456	T:0.231544
chr28	11037997	2	286	G:0.765734	A:0.234266
chr28	11038002	2	286	T:0.503497	A:0.496503
chr28	11038011	2	284	T:0.028169	C:0.971831
chr28	11038194	2	180	G:0.905556	A:0.0944444
chr28	11038344	2	318	ATTCT:0.861635	A:0.138365
chr28	11038412	2	314	C:0.996815	T:0.00318471
chr28	11038442	2	310	G:0.983871	A:0.016129
chr28	11038526	2	298	A:0.771812	G:0.228188
chr28	11038578	2	310	G:0.758065	A:0.241935
chr28	11038649	2	310	A:0.748387	G:0.251613
chr28	11038656	2	310	A:0.748387	C:0.251613
chr28	11038726	2	314	G:0.757962	A:0.242038
chr28	11038746	2	314	CT:0.757962	C:0.242038
chr28	11038767	2	302	T:0.496689	C:0.503311
chr28	11038955	2	300	C:0.746667	T:0.253333
chr28	11039000	2	300	A:0.743333	T:0.256667
chr28	11039001	2	300	G:0.906667	C:0.0933333
chr28	11039040	2	300	A:0.753333	G:0.246667
chr28	11039042	2	300	A:0.753333	G:0.246667
chr28	11039119	2	300	T:0.84	G:0.16
chr28	11039147	2	296	G:0.871622	A:0.128378
chr28	11039175	2	294	G:0.816327	A:0.183673
chr28	11039213	2	302	G:0.817881	A:0.182119
chr28	11039336	2	300	C:0.753333	T:0.246667
chr28	11039421	2	302	G:0.758278	A:0.241722
chr28	11039444	2	302	A:0.758278	G:0.241722
chr28	11039502	2	314	G:0.761146	GAAGTCCCCT:
0.238854
chr28	11039705	2	306	C:0.771242	T:0.228758
chr28	11039750	2	304	G:0.815789	T:0.184211
chr28	11039822	2	300	G:0.766667	A:0.233333
chr28	11039827	2	300	G:0.326667	C:0.673333
chr28	11039882	2	314	C:0.996815	T:0.00318471
chr28	11039902	2	310	G:0.770968	A:0.229032
chr28	11039922	2	294	G:0.860544	A:0.139456
chr28	11039935	2	298	G:0.986577	A:0.0134228
chr28	11039942	2	310	G:0.770968	A:0.229032
chr28	11040024	2	298	G:0.828859	A:0.171141
chr28	11040055	2	298	A:0.100671	C:0.899329
chr28	11040084	2	294	C:0.738095	A:0.261905
chr28	11040134	2	304	T:0.0986842	C:0.901316
chr28	11040220	2	296	A:0.756757	G:0.243243

chr28	11040227	2	296	G:0.756757	A:0.243243
chr28	11040244	2	292	A:0.753425	T:0.246575
chr28	11040316	2	286	A:0.730769	G:0.269231
chr28	11040349	2	296	C:0.739865	T:0.260135
chr28	11040436	2	300	T:0.506667	C:0.493333
chr28	11040688	2	278	G:0.823741	C:0.176259
chr28	11040769	2	202	T:0.683168	C:0.316832
chr28	11040994	2	274	T:0.485401	C:0.514599
chr28	11041095	2	296	A:0.986486	C:0.0135135
chr28	11041311	2	298	G:0.812081	A:0.187919
chr28	11041433	2	292	C:0.113014	T:0.886986
chr28	11041590	2	310	G:0.990323	A:0.00967742
chr28	11041594	2	312	C:0.971154	T:0.0288462
chr28	11041634	2	308	A:0.74026	G:0.25974
chr28	11041754	2	298	C:0.986577	T:0.0134228
chr28	11041778	2	300	T:0.706667	C:0.293333
chr28	11041959	2	284	T:0.482394	C:0.517606
chr28	11042130	2	274	C:0.594891	A:0.405109
chr28	11042173	2	284	G:0.482394	A:0.517606
chr28	11042503	2	298	C:0.902685	T:0.0973154
chr28	11042585	2	302	T:0.857616	A:0.142384
chr28	11042646	2	290	T:0.617241	A:0.382759
chr28	11042665	2	290	A:0.834483	G:0.165517
chr28	11042676	2	290	A:0.582759	G:0.417241
chr28	11042708	2	294	T:0.085034	C:0.914966
chr28	11042715	2	300	T:0.786667	C:0.213333
chr28	11042733	2	294	C:0.806122	T:0.193878
chr28	11042792	2	294	A:0.79932	G:0.20068
chr28	11042800	2	296	C:0.0777027	CA:0.922297
chr28	11042878	2	310	C:0.812903	T:0.187097
chr28	11042885	2	310	A:0.812903	G:0.187097
chr28	11042902	2	310	A:0.812903	T:0.187097
chr28	11042903	2	310	T:0.812903	C:0.187097
chr28	11042925	2	286	G:0.821678	GT:0.178322
chr28	11043013	2	298	C:0.744966	T:0.255034
chr28	11043039	2	308	G:0.86039	C:0.13961
chr28	11043040	2	308	G:0.86039	T:0.13961
chr28	11043061	2	308	A:0.811688	G:0.188312
chr28	11043217	2	306	C:0.803922	T:0.196078
chr28	11043288	2	298	T:0.469799	G:0.530201
chr28	11043364	2	310	C:0.587097	T:0.412903
chr28	11043430	2	310	A:0.76129	G:0.23871
chr28	11043466	2	304	C:0.569079	T:0.430921
chr28	11043573	2	298	G:0.587248	A:0.412752
chr28	11043743	2	308	T:0.551948	C:0.448052
chr28	11043747	2	308	G:0.551948	A:0.448052
chr28	11043767	2	308	C:0.977273	A:0.0227273
chr28	11043779	2	298	A:0.563758	G:0.436242
chr28	11043785	2	300	G:0.77 A:0.23	
chr28	11043879	2	306	C:0.584967	A:0.415033
chr28	11043889	2	306	C:0.584967	G:0.415033
chr28	11043968	2	302	C:0.738411	A:0.261589
chr28	11044034	2	294	C:0.537415	T:0.462585
chr28	11044071	2	304	A:0.809211	G:0.190789

chr28 11044072 2 304 T:0.809211 C:0.190789
chr28 11044133 2 314 A:0.541401 ACCCAGGGGTAAG:
0.458599
chr28 11044260 2 304 G:0.476974 C:0.523026
chr28 11044291 2 290 G:0.975862 A:0.0241379
chr28 11044351 2 300 A:0.55 T:0.45
chr28 11044353 2 300 C:0.99 T:0.01
chr28 11044494 2 292 G:0.568493 A:0.431507
chr28 11044534 2 302 A:0.741722 G:0.258278
chr28 11044630 2 294 G:0.918367 C:0.0816327
chr28 11044814 2 302 CCT:0.784768 C:0.215232
chr28 11044910 2 302 G:0.963576 A:0.0364238
chr28 11044915 2 298 G:0.751678 T:0.248322
chr28 11044953 2 316 T:0.911392
TGAGACTGAATTTTTTTTTTTTTTTTTTGGAA:0.0886076
chr28 11044981 3 298 G:0.755034 GGT:0.100671
GGTGT:0.144295
chr28 11044996 2 282 GTA:0.968085 G:0.0319149
chr28 11044998 2 282 A:0.680851 G:0.319149
chr28 11045201 2 298 C:0.956376 T:0.0436242
chr28 11045229 2 290 G:0.889655 T:0.110345
chr28 11045441 2 304 G:0.898026 C:0.101974
chr28 11045483 2 314 G:0.958599 A:0.0414013
chr28 11045564 2 306 T:0.565359 C:0.434641
chr28 11045598 2 316 G:0.765823 A:0.234177
chr28 11045608 2 316 A:0.484177 T:0.515823
chr28 11045649 2 320 A:0.090625 C:0.909375
chr28 11045652 2 320 C:0.49375 T:0.50625
chr28 11045820 2 296 C:0.743243 T:0.256757
chr28 11045837 2 302 G:0.877483 A:0.122517
chr28 11045846 2 300 C:0.586667 T:0.413333
chr28 11045875 2 298 T:0.516779 C:0.483221
chr28 11045956 2 300 G:0.993333 A:0.00666667
chr28 11046093 2 300 A:0.486667 T:0.513333
chr28 11046283 2 296 C:0.925676 G:0.0743243
chr28 11046314 3 300 TTTTA:0.743333 T:0.106667
TTTTATTTA:0.15
chr28 11046366 2 310 T:0.603226
TTTTATTTATTTATTTATTTATTTA:0.396774
chr28 11046734 2 306 C:0.630719 T:0.369281
chr28 11046745 2 306 C:0.630719 T:0.369281
chr28 11046806 2 304 A:0.832237 G:0.167763
chr28 11046821 2 296 G:0.925676 A:0.0743243
chr28 11047302 2 290 C:0.489655 T:0.510345
chr28 11047309 2 294 C:0.996599 T:0.00340136
chr28 11047492 2 292 G:0.503425 A:0.496575
chr28 11047710 2 298 C:0.966443 T:0.033557
chr28 11047757 2 300 A:0.07 G:0.93
chr28 11047883 2 296 G:0.0844595 T:0.915541
chr28 11047960 2 298 A:0.983221 T:0.0167785
chr28 11047997 2 292 T:0.60274 G:0.39726
chr28 11048566 2 298 A:0.936242 C:0.0637584
chr28 11048570 2 302 A:0.506623 G:0.493377
chr28 11048798 2 294 A:0.0578231 G:0.942177

chr28 11048852 2 300 C:0.976667 T:0.0233333
chr28 11048868 2 296 C:0.0945946 A:0.905405
chr28 11048974 2 296 C:0.496622 A:0.503378
chr28 11049105 2 282 C:0.971631 T:0.0283688
chr28 11049165 2 278 GA:0.122302 G:0.877698
chr28 11049177 2 278 A:0.122302 G:0.877698
chr28 11049178 2 278 A:0.122302 T:0.877698
chr28 11049225 2 270 C:0.977778 T:0.0222222
chr28 11049282 2 286 A:0.940559 AG:0.0594406
chr28 11049309 3 310 TAAAAACAAAAAC:0.445161 T:
0.170968 TAAAAAC:0.383871
chr28 11049314 2 310 A:0.687097 G:0.312903
chr28 11049392 2 296 C:0.631757 CCTT:0.368243
chr28 11049477 2 298 C:0.620805 G:0.379195
chr28 11049681 2 294 T:0.972789 C:0.0272109
chr28 11049791 2 294 T:0.982993 C:0.0170068
chr28 11049831 2 306 T:0.617647 C:0.382353
chr28 11049855 2 306 T:0.617647 C:0.382353
chr28 11050086 2 314 A:0.617834 C:0.382166
chr28 11050104 2 314 C:0.617834 T:0.382166
chr28 11050151 2 312 T:0.608974 C:0.391026
chr28 11050468 2 292 A:0.962329 G:0.0376712
chr28 11050712 2 298 G:0.963087 A:0.0369128
chr28 11050994 2 298 T:0.147651 A:0.852349
chr28 11051063 2 306 C:0.820261 T:0.179739
chr28 11051264 2 292 C:0.636986 T:0.363014
chr28 11051347 2 284 AT:0.989437 A:0.0105634
chr28 11051551 2 294 AC:0.986395 A:0.0136054
chr28 11051910 2 246 C:0.630081 CT:0.369919
chr28 11051955 2 286 C:0.972028 T:0.027972
chr28 11052728 2 294 C:0.986395 T:0.0136054
chr28 11052738 2 294 C:0.986395 T:0.0136054
chr28 11052817 2 298 T:0.989933 C:0.0100671
chr28 11052838 2 310 TTTTCTTTC:0.970968 T:
0.0290323
chr28 11052898 2 310 CTTTCTTTCTTTCT:0.983871 C:
0.016129
chr28 11052902 2 316 CTTTCTTTCT:0.962025 C:
0.0379747
chr28 11052911 2 320 T:0.971875 TTTC:0.028125
chr28 11053169 2 296 C:0.989865 T:0.0101351
chr28 11053300 2 292 G:0.993151 A:0.00684932
chr28 11054904 2 310 T:0.858065 C:0.141935
chr28 11055340 3 298 CT:0.419463 C:0.375839
CTT:0.204698
chr28 11057389 2 300 A:0.883333 G:0.116667
chr28 11057902 2 300 T:0.973333 C:0.0266667
chr28 11058160 2 302 C:0.963576 T:0.0364238
chr28 11058630 4 300 ATTT:0.103333 A:0.353333
AT:0.446667 ATT:0.0966667
chr28 11058660 2 288 T:0.986111 C:0.0138889
chr28 11059767 2 286 T:0.5 TG:0.5
chr28 11060285 2 300 C:0.96 T:0.04
chr28 11060514 2 304 C:0.0789474 T:0.921053

chr28	11060525	2	302	C:0.950331	CA:0.0496689	
chr28	11061225	2	296	G:0.966216	A:0.0337838	
chr28	11062055	2	304	T:0.654605	A:0.345395	
chr28	11062158	2	302	G:0.84106	A:0.15894	
chr28	11062245	2	302	T:0.503311	C:0.496689	
chr28	11062255	2	302	A:0.142384	C:0.857616	
chr28	11062310	2	298	G:0.832215	A:0.167785	
chr28	11062417	2	296	T:0.824324	TA:0.175676	
chr28	11062567	2	312	T:0.823718	C:0.176282	
chr28	11062762	2	310	C:0.835484	T:0.164516	
chr28	11062783	2	310	C:0.835484	G:0.164516	
chr28	11062834	2	310	A:0.174194	T:0.825806	
chr28	11062874	2	304	G:0.957237	C:0.0427632	
chr28	11063078	2	308	A:0.214286	T:0.785714	
chr28	11063083	2	306	T:0.846405	G:0.153595	
chr28	11063122	2	302	T:0.824503	C:0.175497	
chr28	11063134	2	302	T:0.824503	C:0.175497	
chr28	11063207	2	290	C:0.906897	T:0.0931034	
chr28	11063288	2	298	T:0.842282	A:0.157718	
chr28	11063324	2	314	G:0.850318	A:0.149682	
chr28	11063335	2	314	C:0.901274	T:0.0987261	
chr28	11063349	2	314	A:0.850318	G:0.149682	
chr28	11063403	2	308	A:0.863636	G:0.136364	
chr28	11063568	2	302	G:0.97351	A:0.0264901	
chr28	11063647	2	306	A:0.147059	G:0.852941	
chr28	11063753	2	304	C:0.154605	A:0.845395	
chr28	11063772	2	310	TGTTA:0.151613	T:0.848387	
chr28	11063953	2	306	G:0.169935	GTGTC:0.830065	
chr28	11064493	2	278	T:0.158273	C:0.841727	
chr28	11064863	3	280	TAA:0.303571	T:0.475	TA: 0.221429
chr28	11064942	2	288	T:0.965278	A:0.0347222	
chr28	11065209	2	244	A:0.729508	G:0.270492	
chr28	11066114	2	278	CT:0.0935252	C:0.906475	
chr28	11066583	2	308	A:0.892857	G:0.107143	
chr28	11066856	2	306	A:0	T:1	
chr28	11066857	2	306	A:0	G:1	
chr28	11066898	2	300	A:0	G:1	
chr28	11066926	2	302	A:0.748344	AC:0.251656	
chr28	11067087	2	304	A:0.983553	G:0.0164474	
chr28	11067397	2	300	C:0.77	T:0.23	
chr28	11067465	2	304	T:0.210526	C:0.789474	
chr28	11067559	2	302	G:0.930464	T:0.0695364	
chr28	11067786	2	298	T:0.748322	C:0.251678	
chr28	11068293	2	300	A:0.77	G:0.23	
chr28	11068294	2	304	G:0.111842	GGA:0.888158	
chr28	11068433	2	304	T:0	G:1	
chr28	11068476	2	286	CTG:0.870629	C:0.129371	
chr28	11068486	2	288	T:0	C:1	
chr28	11068487	2	288	T:0	C:1	
chr28	11068648	2	274	AT:0.729927	A:0.270073	
chr28	11068658	2	274	T:0.99635	A:0.00364964	
chr28	11069264	2	300	G:0.953333	C:0.0466667	
chr28	11069542	2	298	G:0.986577	C:0.0134228	

chr28	11069630	2	318	GCTGGCAAGTGCCTGGGGC:0.858491	G:0.141509	
chr28	11069722	2	308	G:0.853896	A:0.146104	
chr28	11070083	2	312	AT:0.788462	A:0.211538	
chr28	11070732	2	296	C:0.972973	T:0.027027	
chr28	11070951	2	288	GT:0.423611	G:0.576389	
chr28	11071678	2	290	T:0.124138	C:0.875862	
chr28	11071878	2	304	C:0.855263	T:0.144737	
chr28	11071976	2	296	C:0.888514	T:0.111486	
chr28	11072114	2	302	A:0.635762	T:0.364238	
chr28	11072137	2	296	A:0.608108	G:0.391892	
chr28	11072430	2	294	T:0.510204	C:0.489796	
chr28	11072492	3	250	CTT:0.304	C:0.476	CT:0.22
chr28	11072543	2	300	GAC:0.84	G:0.16	
chr28	11072547	3	312	CACAG:0.679487	C:0.163462	CAGAGAGAGACAG:0.157051
chr28	11072549	3	308	CAGAGAG:0.512987	C:0.103896	GAGAGAG:0.383117
chr28	11072840	2	262	G:0.110687	T:0.889313	
chr28	11073413	2	298	T:0.52349	A:0.47651	
chr28	11073415	2	298	G:0.52349	A:0.47651	
chr28	11073659	2	292	C:0.989726	T:0.010274	
chr28	11073995	2	264	GCTT:0.693182	G:0.306818	
chr28	11073998	2	284	T:0.964789	G:0.0352113	
chr28	11073999	2	284	CTT:0.964789	C:0.0352113	
chr28	11074002	2	292	T:0.952055	C:0.0479452	
chr28	11074091	2	294	A:0.965986	G:0.0340136	
chr28	11074125	2	298	A:0.879195	G:0.120805	
chr28	11074183	2	294	C:0.969388	T:0.0306122	
chr28	11074258	2	304	C:0.835526	T:0.164474	
chr28	11074371	2	308	C:0.961039	CG:0.038961	
chr28	11074479	2	294	A:0.989796	G:0.0102041	
chr28	11074654	2	316	G:0.962025	A:0.0379747	
chr28	11074671	2	318	GGC:0.550314	G:0.449686	
chr28	11074800	2	294	G:0.901361	A:0.0986395	
chr28	11074844	2	298	GGT:0.728188	G:0.271812	
chr28	11074860	2	298	G:0.718121	A:0.281879	
chr28	11074980	2	300	G:0.97	C:0.03	
chr28	11075230	2	280	A:0.957143	G:0.0428571	
chr28	11075318	2	288	A:0.965278	G:0.0347222	
chr28	11075428	2	290	C:0.962069	T:0.037931	
chr28	11075439	2	290	C:0.962069	G:0.037931	
chr28	11075530	2	298	C:0.956376	T:0.0436242	
chr28	11075544	2	298	ATAAGT:0.956376	A:0.0436242	
chr28	11075654	2	294	A:0.714286	AT:0.285714	
chr28	11075655	2	294	A:0.714286	T:0.285714	
chr28	11075670	2	296	C:0.131757	T:0.868243	
chr28	11075741	3	294	TTTATTTA:0.64966	TTTTA:0.268707	T:0.0816327
chr28	11075748	3	308	ATTTTT:0.282468	A:0.256494	ATTTTTT:0.461039
chr28	11075791	2	276	A:0.807971	C:0.192029	
chr28	11075888	2	282	G:0.897163	C:0.102837	
chr28	11075926	2	294	A:0.153061	G:0.846939	

chr28	11075938	2	296	T:0.733108	G:0.266892
chr28	11075981	2	286	T:0.968531	TC:0.0314685
chr28	11076189	2	292	AT:0.945205	A:0.0547945
chr28	11076227	2	300	G:0.963333	T:0.0366667
chr28	11076236	2	300	T:0.963333	TG:0.0366667
chr28	11076317	2	292	G:0.962329	C:0.0376712
chr28	11076385	2	300	G:0.98	A:0.02
chr28	11076630	2	290	T:0.955172	C:0.0448276
chr28	11076714	2	302	G:0.966887	A:0.0331126
chr28	11076770	2	310	G:0.964516	A:0.0354839
chr28	11076911	2	300	G:0.96	A:0.04
chr28	11076921	2	296	T:0.817568	A:0.182432
chr28	11076937	2	294	C:0.965986	T:0.0340136
chr28	11076994	2	312	GCACTGC:0.942308	G:0.0576923
chr28	11077054	2	296	C:0.986486	A:0.0135135
chr28	11077133	2	296	C:0.851351	T:0.148649
chr28	11077297	2	300	C:0.696667	T:0.303333
chr28	11077318	2	300	A:0.1	G:0.9
chr28	11077319	2	302	T:0.966887	C:0.0331126
chr28	11077447	2	314	T:0.977707	TCCAGGCACAGAGCTACCAA:0.022293
chr28	11077561	2	296	C:0.966216	T:0.0337838
chr28	11077680	2	302	CTT:0.963576	C:0.0364238
chr28	11077780	2	302	G:0.966887	A:0.0331126
chr28	11077863	2	302	C:0.596026	A:0.403974
chr28	11077910	2	304	G:0.993421	C:0.00657895
chr28	11078064	2	306	GA:0.928105	G:0.0718954
chr28	11078201	2	292	T:0.623288	C:0.376712
chr28	11078236	2	302	A:0.917219	G:0.0827815
chr28	11078310	2	292	A:0.565068	G:0.434932
chr28	11078317	2	292	A:0.921233	C:0.0787671
chr28	11078404	2	290	C:0.237931	CG:0.762069
chr28	11078422	2	302	C:0.890728	CGGGCTGCTGTG:0.109272
chr28	11078501	2	274	A:0.919708	C:0.080292
chr28	11078804	2	310	T:0.406452	C:0.593548
chr28	11078812	2	314	CGGAGGGAGGGTGACTGGCCCG:0.869427	C:0.130573
chr28	11078843	2	312	A:0.189103	G:0.810897
chr28	11078939	2	288	C:0.725694	T:0.274306
chr28	11078991	2	294	A:0.721088	G:0.278912
chr28	11079200	2	304	A:0.697368	G:0.302632
chr28	11079493	2	316	A:0.693038	AATGAGTGCGTGTGCATTCAGTGTAT:0.306962
chr28	11079599	2	306	C:0.732026	T:0.267974
chr28	11079620	2	302	G:0.907285	A:0.0927152
chr28	11079735	2	308	C:0.678571	T:0.321429
chr28	11079947	2	302	T:0.741722	C:0.258278
chr28	11080056	2	302	G:0.956954	C:0.0430464
chr28	11080401	2	276	A:0.916667	G:0.0833333
chr28	11080539	2	304	ACAG:0.973684	A:0.0263158
chr28	11080543	2	304	G:0.973684	GTGATGA:0.0263158
chr28	11080749	2	286	C:0.597902	T:0.402098
chr28	11080756	2	290	T:0.737931	C:0.262069

chr28	11080853	2	304	TG:0.970395	T:0.0296053	
chr28	11080976	2	308	T:0.753247	G:0.246753	
chr28	11081239	2	294	A:0.765306	G:0.234694	
chr28	11081330	2	308	CTG:0.987013	C:0.012987	
chr28	11081483	2	302	A:0.735099	AC:0.264901	
chr28	11081494	2	304	T:0.674342	G:0.325658	
chr28	11081540	2	302	C:0.725166	T:0.274834	
chr28	11081649	2	300	C:0.976667	T:0.0233333	
chr28	11081757	2	290	C:0.272414	G:0.727586	
chr28	11081919	2	296	C:0.733108	T:0.266892	
chr28	11081959	2	306	T:0.0816993	C:0.918301	
chr28	11082040	2	304	T:0.726974	C:0.273026	
chr28	11082136	2	308	A:0.727273	C:0.272727	
chr28	11082244	2	310	C:0.183871	T:0.816129	
chr28	11082388	2	306	C:0.218954	G:0.781046	
chr28	11082442	2	306	C:0.189542	T:0.810458	
chr28	11082920	2	306	G:0.738562	A:0.261438	
chr28	11083144	2	302	T:0.715232	C:0.284768	
chr28	11083204	3	314	C:0.694268	G:0.00636943	T:0.299363
chr28	11083338	2	306	C:0.627451	CTG:0.372549	
chr28	11083603	2	288	A:0.701389	T:0.298611	
chr28	11083870	2	296	C:0.418919	T:0.581081	
chr28	11083873	2	292	C:0.989726	T:0.010274	
chr28	11083874	2	292	G:0.989726	A:0.010274	
chr28	11083953	2	304	C:0.967105	T:0.0328947	
chr28	11084408	2	302	C:0.913907	T:0.0860927	
chr28	11084893	2	130	T:0.584615	TA:0.415385	
chr28	11084944	2	306	GAGAGAGAGAGAGCACGCGCC:0.650327	G:0.349673	
chr28	11085077	2	284	G:0.380282	A:0.619718	
chr28	11085221	2	292	T:0.428082	C:0.571918	
chr28	11085223	2	290	C:0.955172	A:0.0448276	
chr28	11085484	2	310	G:0.777419	A:0.222581	
chr28	11085598	2	308	GT:0.99026	G:0.00974026	
chr28	11085630	2	304	A:0.947368	G:0.0526316	
chr28	11085654	2	318	A:0.113208	AGTGAAGTACTGTGG:0.886792	
chr28	11085939	2	298	T:0.788591	C:0.211409	
chr28	11086111	2	304	G:0.990132	A:0.00986842	
chr28	11086130	2	294	C:0.629252	CG:0.370748	
chr28	11086334	2	298	C:0.61745	A:0.38255	
chr28	11086385	2	300	G:0.706667	A:0.293333	
chr28	11086761	2	298	C:0.620805	T:0.379195	
chr28	11086773	2	290	T:0.6	C:0.4	
chr28	11086780	2	296	C:0.85473	T:0.14527	
chr28	11087050	2	296	T:0.60473	C:0.39527	
chr28	11087121	2	302	G:0.970199	A:0.0298013	
chr28	11087143	2	312	T:0.980769	C:0.0192308	
chr28	11087191	2	298	C:0.979866	CT:0.0201342	
chr28	11087197	3	298	A:0.228188	G:0.751678	C:0.0201342
chr28	11087228	2	290	G:0.758621	T:0.241379	
chr28	11087249	2	294	T:0.251701	C:0.748299	

chr28	11087269	3	304	A:0.246711	G:0.730263	C:0.0230263	
chr28	11087277	2	304	C:0.973684	T:0.0263158		
chr28	11087278	2	304	G:0.792763	A:0.207237		
chr28	11087407	2	300	C:0.99	T:0.01		
chr28	11087609	2	300	C:0.923333	T:0.0766667		
chr28	11087639	2	304	C:0.898026	T:0.101974		
chr28	11087712	2	312	C:0.99359	A:0.00641026		
chr28	11088107	2	306	T:0.960784	A:0.0392157		
chr28	11088113	2	304	A:0.674342	G:0.325658		
chr28	11088146	2	300	C:0.763333	T:0.236667		
chr28	11088216	2	304	T:0.671053	A:0.328947		
chr28	11088303	2	308	T:0.961039	A:0.038961		
chr28	11088311	2	308	T:0.961039	A:0.038961		
chr28	11088406	2	298	G:0.899329	T:0.100671		
chr28	11088443	2	286	C:0.804196	T:0.195804		
chr28	11088646	2	302	G:0.788079	C:0.211921		
chr28	11088889	2	294	C:0.47619	G:0.52381		
chr28	11088961	2	278	G:0.478417	T:0.521583		
chr28	11089447	2	290	T:0.737931	TG:0.262069		
chr28	11089675	2	286	T:0.804196	C:0.195804		
chr28	11089868	2	294	ATGAT:0.816327	A:0.183673		
chr28	11090312	2	304	C:0.509868	T:0.490132		
chr28	11090385	2	298	C:0.57047	T:0.42953		
chr28	11090391	2	306	G:0.794118	A:0.205882		
chr28	11090401	2	306	T:0.794118	C:0.205882		
chr28	11090601	2	304	T:0.549342	C:0.450658		
chr28	11090646	2	302	G:0.678808	A:0.321192		
chr28	11090851	2	310	T:0.8	C:0.2		
chr28	11091691	2	304	T:0.904605	C:0.0953947		
chr28	11091957	2	306	A:0.529412	T:0.470588		
chr28	11091964	2	308	C:0.818182	T:0.181818		
chr28	11091986	2	312	C:0.298077	T:0.701923		
chr28	11092072	2	300	G:0.89	A:0.11		
chr28	11092484	2	308	G:0.50974	A:0.49026		
chr28	11092673	2	290	C:0.806897	G:0.193103		
chr28	11092734	2	288	G:0.541667	A:0.458333		
chr28	11092759	2	294	A:0.306122	G:0.693878		
chr28	11092773	4	300	CT:0.536667	C:0.113333	CTT:0.256667	CTTT:0.0933333
chr28	11092798	2	272	A:0.518382	G:0.481618		
chr28	11093193	2	262	T:0.767176	C:0.232824		
chr28	11093450	2	306	C:0.957516	T:0.0424837		
chr28	11093451	2	306	G:0.954248	A:0.0457516		
chr28	11093600	2	298	T:0.536913	C:0.463087		
chr28	11093730	2	308	C:0.967532	T:0.0324675		
chr28	11093772	2	308	A:0.0909091	C:0.909091		
chr28	11094006	2	300	T:0.216667	C:0.783333		
chr28	11094058	2	304	C:0.296053	T:0.703947		
chr28	11094197	2	286	A:0.293706	C:0.706294		
chr28	11094562	2	292	T:0.321918	C:0.678082		
chr28	11094827	2	262	T:0.221374	C:0.778626		
chr28	11094880	2	264	G:0.768939	C:0.231061		
chr28	11095001	2	148	CTA:0.506757	C:0.493243		

chr28	11095046	2	282	GAGGT:0.262411	G:0.737589
chr28	11095051	2	282	CATCACTGTTTCCTCGA:0.262411	C:0.737589
chr28	11095265	2	256	A:0.242188	G:0.757812
chr28	11095422	2	260	C:0.953846	T:0.0461538
chr28	11095450	2	226	T:0.247788	C:0.752212
chr28	11095473	2	182	G:0.510989	GC:0.489011
chr28	11095587	2	262	GC:0.729008	G:0.270992
chr28	11095611	2	268	T:0.80597	C:0.19403
chr28	11095636	2	280	T:0.214286	TC:0.785714
chr28	11095641	2	280	T:0.214286	C:0.785714
chr28	11095713	2	276	G:0.293478	A:0.706522
chr28	11095747	2	276	C:0.32971	T:0.67029
chr28	11096086	2	294	T:0.292517	TC:0.707483
chr28	11096095	2	294	T:0.292517	C:0.707483
chr28	11096168	2	288	T:0.0659722	C:0.934028
chr28	11096230	2	296	C:0.912162	G:0.0878378
chr28	11096387	2	294	T:0.115646	C:0.884354
chr28	11096613	2	312	C:0.099359	A:0.900641
chr28	11096615	2	312	C:0.342949	T:0.657051
chr28	11096744	2	254	C:0.992126	T:0.00787402
chr28	11096805	2	268	G:0.955224	A:0.0447761
chr28	11096887	2	314	C:0.0700637	T:0.929936
chr28	11096899	2	312	TTATAA:0.323718	T:0.676282
chr28	11097168	2	282	C:0.904255	T:0.0957447
chr28	11097174	2	280	T:0.332143	C:0.667857
chr28	11097222	2	276	G:0.753623	T:0.246377
chr28	11097772	2	298	T:0.241611	C:0.758389
chr28	11097881	2	284	G:0.753521	T:0.246479
chr28	11098026	2	280	A:0.742857	C:0.257143
chr28	11098304	2	274	G:0.762774	T:0.237226
chr28	11098527	2	296	A:0.996622	G:0.00337838
chr28	11098587	2	294	A:0.204082	G:0.795918
chr28	11098782	2	296	C:0.993243	CCTT:0.00675676
chr28	11099365	2	272	G:0.889706	C:0.110294
chr28	11099488	2	278	GA:0.197842	G:0.802158
chr28	11099513	2	290	G:0.782759	A:0.217241
chr28	11099641	2	286	G:0.737762	A:0.262238
chr28	11099763	2	284	T:0.950704	C:0.0492958
chr28	11099799	2	284	A:0.56338	G:0.43662
chr28	11100187	2	282	T:0.975177	C:0.0248227
chr28	11100307	2	286	T:0.0769231	C:0.923077
chr28	11100470	2	280	T:0.960714	C:0.0392857
chr28	11100601	2	294	G:0.785714	A:0.214286
chr28	11100610	2	296	G:0.959459	C:0.0405405
chr28	11100623	2	296	G:0.959459	A:0.0405405
chr28	11100882	2	304	C:0.309211	T:0.690789
chr28	11101093	2	290	T:0.317241	C:0.682759
chr28	11101147	2	298	C:0.963087	T:0.0369128
chr28	11101263	2	292	T:0.938356	TA:0.0616438
chr28	11101367	2	300	G:0.25	C:0.75
chr28	11101835	2	302	C:0.298013	T:0.701987
chr28	11101862	2	284	T:0.190141	TC:0.809859
chr28	11101892	2	264	A:0.825758	AC:0.174242

chr28	11102125	2	292	C:0.291096	T:0.708904
chr28	11102168	2	288	C:0.965278	T:0.0347222
chr28	11102205	2	288	C:0.986111	T:0.0138889
chr28	11102340	2	294	G:0.292517	A:0.707483
chr28	11102341	2	294	C:0.904762	T:0.0952381
chr28	11102815	2	294	C:0.972789	T:0.0272109
chr28	11103125	2	304	G:0.296053	A:0.703947
chr28	11103373	2	298	C:0.966443	G:0.033557
chr28	11103479	2	306	C:0.179739	T:0.820261
chr28	11103607	2	296	G:0.293919	A:0.706081
chr28	11103837	2	316	A:0.566456	AT:0.433544
chr28	11104180	2	310	G:0.206452	A:0.793548
chr28	11104411	2	292	G:0.202055	A:0.797945
chr28	11104762	2	298	A:0.825503	G:0.174497
chr28	11104855	2	314	TTGTC:0.910828	T:0.089172
chr28	11105005	2	306	C:0.284314	A:0.715686
chr28	11105189	2	304	T:0.200658	C:0.799342
chr28	11105214	2	296	G:0.787162	A:0.212838
chr28	11105290	2	302	C:0.990066	A:0.00993377
chr28	11105354	2	302	T:0.0960265	G:0.903974
chr28	11105413	2	310	T:0.522581	A:0.477419
chr28	11105483	2	300	C:0.806667	T:0.193333
chr28	11105707	2	302	C:0.788079	T:0.211921
chr28	11105854	2	290	A:0.334483	AT:0.665517
chr28	11105936	2	304	A:0.197368	C:0.802632
chr28	11106029	2	288	C:0.201389	T:0.798611
chr28	11106299	2	306	C:0.418301	T:0.581699
chr28	11106656	2	152	G:0.394737	C:0.605263
chr28	11106834	2	272	GA:0.863971	G:0.136029
chr28	11106854	2	276	GGAA:0.818841	G:0.181159
chr28	11107247	2	296	G:0.766892	T:0.233108
chr28	11107330	2	304	C:0.447368	G:0.552632
chr28	11107343	2	306	A:0.960784	AG:0.0392157
chr28	11107350	2	308	A:0.448052	C:0.551948
chr28	11108437	2	288	A:0.798611	G:0.201389
chr28	11109076	2	306	C:0.830065	T:0.169935
chr28	11109354	2	310	G:0.567742	GCCCTCTGT: 0.432258
chr28	11109367	2	310	C:0.519355	CTGTCCCTG: 0.480645
chr28	11109508	2	300	C:0.833333	G:0.166667
chr28	11109652	2	290	C:0.0344828	T:0.965517
chr28	11109667	2	300	G:0.863333	A:0.136667
chr28	11109813	2	294	G:0.761905	T:0.238095
chr28	11110093	2	302	C:0.433775	A:0.566225
chr28	11110396	2	300	C:0.733333	T:0.266667
chr28	11110523	2	300	T:0.583333	G:0.416667
chr28	11110596	2	302	A:0.854305	C:0.145695
chr28	11110844	2	304	G:0.973684	A:0.0263158
chr28	11111059	2	298	G:0.751678	A:0.248322
chr28	11111267	2	304	T:0.828947	C:0.171053
chr28	11111378	2	316	T:0.838608	TGCCAGTGGTCCCTGGGCCCCTCTTAGAGAA:0.161392
chr28	11111411	2	302	C:0.990066	T:0.00993377

chr28	11111442	2	302	C:0.761589	T:0.238411	
chr28	11111522	2	302	G:0.831126	C:0.168874	
chr28	11111683	2	294	G:0.85034	A:0.14966	
chr28	11111688	2	294	A:0.85034	ACT:0.14966	
chr28	11111767	2	310	C:0.832258	CTGCCCATTT:0.167742	
chr28	11111835	2	284	A:0.84507	G:0.15493	
chr28	11111999	2	288	G:0.986111	A:0.0138889	
chr28	11112156	2	294	G:0.765306	GGT:0.234694	
chr28	11112381	2	296	G:0.814189	C:0.185811	
chr28	11112479	2	306	T:0.843137	A:0.156863	
chr28	11112502	2	308	T:0.844156	C:0.155844	
chr28	11112579	2	308	A:0.834416	G:0.165584	
chr28	11112915	2	294	T:0.826531	C:0.173469	
chr28	11112950	2	282	A:0.776596	C:0.223404	
chr28	11113095	2	308	C:0.86039	A:0.13961	
chr28	11113144	2	308	C:0.834416	G:0.165584	
chr28	11113246	2	286	A:0.0979021	G:0.902098	
chr28	11113345	2	302	CAGT:0.834437	C:0.165563	
chr28	11113470	2	308	C:0.899351	T:0.100649	
chr28	11113826	2	294	G:0.860544	C:0.139456	
chr28	11113919	2	298	G:0.741611	T:0.258389	
chr28	11113945	2	308	G:0.74026	A:0.25974	
chr28	11114003	2	308	C:0.996753	A:0.00324675	
chr28	11114052	2	300	CA:0.83	C:0.17	
chr28	11114214	2	296	T:0.0844595	TG:0.915541	
chr28	11114250	2	306	G:0.74183	GGGCATCA:0.25817	
chr28	11114310	2	302	C:0.960265	T:0.0397351	
chr28	11114488	2	288	G:0.0416667	A:0.958333	
chr28	11114644	2	308	G:0.840909	C:0.159091	
chr28	11114680	2	298	C:0.899329	A:0.100671	
chr28	11114745	2	296	C:0.962838	G:0.0371622	
chr28	11114952	2	286	T:0.958042	G:0.041958	
chr28	11115466	2	312	GGTCATCACTCTTTT:0.826923	G:0.173077	
chr28	11116483	2	300	G:0.85	A:0.15	
chr28	11116748	2	304	G:0.766447	A:0.233553	
chr28	11116868	2	300	C:0.966667	T:0.0333333	
chr28	11117470	2	296	C:0.969595	T:0.0304054	
chr28	11117661	2	302	C:0.837748	T:0.162252	
chr28	11117865	2	298	C:0.0268456	G:0.973154	
chr28	11117881	2	302	T:0.953642	C:0.0463576	
chr28	11117882	2	302	G:0.586093	A:0.413907	
chr28	11117970	2	306	G:0.424837	A:0.575163	
chr28	11118123	2	298	G:0.775168	T:0.224832	
chr28	11118577	2	288	G:0.829861	A:0.170139	
chr28	11119076	3	320	G:0.725	GTGTATATATATA:0.190625	GTGTATATATATATATATATATATATATATA:0.084375
chr28	11119439	2	294	TA:0.265306	T:0.734694	
chr28	11119460	2	296	C:0.584459	T:0.415541	
chr28	11119719	2	306	AGCCGC:0.470588	A:0.529412	
chr28	11119758	2	302	C:0.993377	T:0.00662252	
chr28	11119763	2	302	G:0.453642	A:0.546358	
chr28	11119851	2	298	A:0.83557	G:0.16443	

chr28	11119940	2	290	G:0.962069	A:0.037931	
chr28	11119970	2	280	G:0.757143	A:0.242857	
chr28	11120461	2	286	C:0.625874	T:0.374126	
chr28	11120469	2	288	T:0.0381944	G:0.961806	
chr28	11120511	2	290	C:0.817241	T:0.182759	
chr28	11120515	2	288	A:0.0381944	G:0.961806	
chr28	11120552	2	292	A:0.0342466	G:0.965753	
chr28	11120557	2	296	G:0.591216	A:0.408784	
chr28	11120715	2	294	GCCTC:0.840136	G:0.159864	
chr28	11120719	2	294	C:0.221088	G:0.778912	
chr28	11121395	2	294	C:0.962585	T:0.037415	
chr28	11122447	2	298	G:0.966443	A:0.033557	
chr28	11122803	2	312	G:0.964744	A:0.0352564	
chr28	11122824	2	312	C:0.576923	T:0.423077	
chr28	11122877	2	308	G:0.448052	A:0.551948	
chr28	11122960	2	296	A:0.929054	C:0.0709459	
chr28	11123223	2	202	AGTGT:0.554455	A:0.445545	
chr28	11123436	2	178	C:0.398876	T:0.601124	
chr28	11123464	3	106	G:0.0849057	GC:0.537736	GCC:0.377358
chr28	11123682	2	274	G:0.912409	A:0.0875912	
chr28	11123715	2	268	T:0.955224	TAG:0.0447761	
chr28	11123739	2	268	A:0.58209	G:0.41791	
chr28	11123933	2	314	GTCAACCATCCAATGCA:0.598726	G:0.401274	
chr28	11124034	2	298	C:0.624161	T:0.375839	
chr28	11124145	2	276	T:0.561594	C:0.438406	
chr28	11124619	2	296	C:0.753378	T:0.246622	
chr28	11124663	2	318	AGGTGTCGCTATCTGCT:0.666667	A:0.333333	
chr28	11124729	2	288	T:0.607639	C:0.392361	
chr28	11124852	2	296	G:0.766892	C:0.233108	
chr28	11124920	2	288	G:0.965278	A:0.0347222	
chr28	11125055	2	282	G:0.840426	A:0.159574	
chr28	11125076	2	280	T:0.0107143	C:0.989286	
chr28	11125082	2	286	A:0.902098	G:0.0979021	
chr28	11125296	2	282	G:0.989362	C:0.0106383	
chr28	11125301	2	284	A:0.838028	AG:0.161972	
chr28	11126251	2	304	G:0.0427632	A:0.957237	
chr28	11126751	2	302	A:0.701987	T:0.298013	
chr28	11127161	2	296	T:0.506757	TG:0.493243	
chr28	11127552	2	288	A:0.520833	G:0.479167	
chr28	11127885	2	292	T:0.520548	TG:0.479452	
chr28	11127933	2	292	T:0.489726	C:0.510274	
chr28	11128622	2	300	G:0.456667	A:0.543333	
chr28	11128738	2	302	C:0.97351	T:0.0264901	
chr28	11128850	2	312	T:0.772436	C:0.227564	
chr28	11128854	2	310	C:0.790323	T:0.209677	
chr28	11128957	2	300	C:0.963333	T:0.0366667	
chr28	11129294	2	304	C:0.809211	G:0.190789	
chr28	11129364	2	288	G:0.451389	A:0.548611	
chr28	11129661	2	308	G:0.814935	A:0.185065	
chr28	11129712	2	308	G:0.555195	GAGA:0.444805	
chr28	11129752	2	300	A:0.533333	G:0.466667	

chr28	11129795	2	286	C:0.534965	T:0.465035	
chr28	11129822	2	298	A:0.989933	C:0.0100671	
chr28	11129834	2	298	C:0.768456	G:0.231544	
chr28	11129868	2	292	A:0.804795	G:0.195205	
chr28	11130117	2	290	C:0.706897	T:0.293103	
chr28	11130274	2	308	A:0.762987	T:0.237013	
chr28	11130544	2	312	G:0.804487	A:0.195513	
chr28	11130804	2	300	C:0.77	T:0.23	
chr28	11130815	2	300	C:0.77	T:0.23	
chr28	11130859	2	280	G:0.767857	A:0.232143	
chr28	11131063	2	300	T:0.793333	C:0.206667	
chr28	11131224	2	302	A:0.854305	T:0.145695	
chr28	11131264	2	296	T:0.0574324	C:0.942568	
chr28	11131378	2	308	C:0.782468	T:0.217532	
chr28	11131401	2	308	G:0.782468	A:0.217532	
chr28	11131888	2	264	C:0.818182	T:0.181818	
chr28	11131963	2	298	C:0.248322	T:0.751678	
chr28	11132199	2	292	GAAGC:0.763699	G:0.236301	
chr28	11132234	2	268	C:0.75	T:0.25	
chr28	11132682	2	302	CTGGGA:0.748344	C:0.251656	
chr28	11132688	2	300	T:0.593333	C:0.406667	
chr28	11132734	2	298	G:0.825503	T:0.174497	
chr28	11133031	2	302	A:0.764901	G:0.235099	
chr28	11133123	2	306	A:0.761438	C:0.238562	
chr28	11133233	2	300	T:0.746667	C:0.253333	
chr28	11133371	2	278	A:0.730216	G:0.269784	
chr28	11133787	2	298	T:0.657718	G:0.342282	
chr28	11133958	2	294	A:0.765306	T:0.234694	
chr28	11134127	2	296	C:0.760135	T:0.239865	
chr28	11134128	2	294	A:0.35034	G:0.64966	
chr28	11134207	2	310	G:0.774194	GT:0.225806	
chr28	11134396	2	306	C:0.787582	T:0.212418	
chr28	11134854	2	284	C:0.767606	G:0.232394	
chr28	11135111	2	300	A:0.77	T:0.23	
chr28	11135266	2	298	T:0.755034	C:0.244966	
chr28	11135305	2	300	A:0.776667	G:0.223333	
chr28	11135483	2	290	G:0.775862	T:0.224138	
chr28	11136856	2	292	T:0.208904	G:0.791096	
chr28	11137017	2	290	G:0.813793	A:0.186207	
chr28	11137148	2	306	G:0.816993	T:0.183007	
chr28	11137321	2	292	T:0.236301	C:0.763699	
chr28	11137812	2	300	C:0.853333	T:0.146667	
chr28	11138410	2	288	C:0.815972	T:0.184028	
chr28	11138674	2	300	C:0.796667	T:0.203333	
chr28	11139107	2	300	A:0.783333	G:0.216667	
chr28	11139630	2	310	G:0.803226	A:0.196774	
chr28	11139927	2	302	G:0.764901	A:0.235099	
chr28	11140069	2	298	C:0.768456	T:0.231544	
chr28	11141630	2	246	CT:0.723577	C:0.276423	
chr28	11141693	3	310	C:0.487097	CAGAGAG:0.322581	CAGAGAGAGAG:0.190323
chr28	11141931	2	302	A:0.430464	T:0.569536	
chr28	11141962	2	308	C:0.399351	T:0.600649	
chr28	11142267	3	318	T:0.603774	TAACAC:0.138365	

TAACATAACATAACAC:0.257862					
chr28	11142363	2	304	G:0.529605	T:0.470395
chr28	11142488	2	300	G:0.51 A:0.49	
chr28	11142555	2	306	CT:0.542484	C:0.457516
chr28	11142679	2	304	A:0.805921	G:0.194079
chr28	11142692	2	302	AC:0.00331126	A:0.996689
chr28	11143194	2	302	T:0.00331126	C:0.996689
chr28	11143261	2	302	T:0.456954	C:0.543046
chr28	11143450	2	308	A:0.441558	G:0.558442
chr28	11144030	2	296	C:0.557432	G:0.442568
chr28	11144307	2	276	T:0.48913	C:0.51087
chr28	11144685	2	298	A:0.0033557	G:0.996644
chr28	11144887	2	308	ACTCT:0.584416	A:0.415584
chr28	11145043	2	298	T:0.0033557	C:0.996644
chr28	11145093	2	296	G:0.820946	T:0.179054
chr28	11145162	2	302	A:0.791391	G:0.208609
chr28	11145354	2	304	A:0.200658	G:0.799342
chr28	11145417	2	308	T:0.844156	C:0.155844
chr28	11145436	2	302	T:0.198675	C:0.801325
chr28	11145448	2	302	C:0.933775	A:0.0662252
chr28	11145545	2	300	G:0.19 T:0.81	
chr28	11145690	2	274	C:0.60219	G:0.39781
chr28	11146295	2	300	C:0.456667	T:0.543333
chr28	11146472	2	306	C:0.784314	A:0.215686
chr28	11146594	2	308	C:0.24026	A:0.75974
chr28	11146971	2	298	G:0.177852	GA:0.822148
chr28	11146974	4	310	G:0.135484	GGGAGA:0.283871
GGGAGAGA:0.164516			GGGAGAGAGA:0.416129		
chr28	11147054	2	278	C:0.42446	T:0.57554
chr28	11147463	2	290	T:0.844828	G:0.155172
chr28	11147800	2	278	T:0.773381	C:0.226619
chr28	11147871	2	282	G:0.648936	T:0.351064
chr28	11148705	2	308	A:0.62013	G:0.37987
chr28	11148781	2	300	C:0.646667	T:0.353333
chr28	11149467	2	292	C:0.10274	G:0.89726
chr28	11149894	2	292	CT:0.640411	C:0.359589
chr28	11149941	2	296	G:0.837838	A:0.162162
chr28	11150015	2	308	T:0.665584	C:0.334416
chr28	11150447	2	300	T:0.63 C:0.37	
chr28	11150546	2	306	G:0.24183	A:0.75817
chr28	11150858	2	300	G:0.96 A:0.04	
chr28	11151445	2	298	G:0.234899	GGA:0.765101
chr28	11151693	2	288	C:0.659722	G:0.340278
chr28	11151793	2	304	C:0.641447	T:0.358553
chr28	11151986	2	304	C:0.648026	A:0.351974
chr28	11152066	2	306	C:0.00653595	T:0.993464
chr28	11152200	2	296	G:0.658784	A:0.341216
chr28	11152206	3	304	CTT:0.694079	C:0.121711
CTTTT:0.184211					
chr28	11152336	2	312	G:0.958333	A:0.0416667
chr28	11152348	2	314	C:0.929936	T:0.0700637
chr28	11152491	2	310	T:0.435484	A:0.564516
chr28	11152563	2	306	CAAAT:0.993464	C:0.00653595
chr28	11152752	2	312	T:0.663462	C:0.336538

chr28	11153222	2	302	A:0.63245	G:0.36755	
chr28	11153233	2	296	G:0.773649	T:0.226351	
chr28	11153341	2	298	G:0.714765	A:0.285235	
chr28	11153357	2	298	A:0.697987	G:0.302013	
chr28	11153836	2	298	T:0.677852	G:0.322148	
chr28	11153872	2	300	T:0.79	C:0.21	
chr28	11154148	2	302	C:0.788079	T:0.211921	
chr28	11154154	2	302	A:0.788079	T:0.211921	
chr28	11154158	2	300	C:0.236667	T:0.763333	
chr28	11154931	2	302	G:0.536424	T:0.463576	
chr28	11155137	2	290	TA:0.451724	T:0.548276	
chr28	11155363	2	310	T:0	TC:1	
chr28	11155369	2	310	CA:0	C:1	
chr28	11155378	2	310	G:0	GT:1	
chr28	11155382	2	310	CT:0	C:1	
chr28	11156260	2	298	C:0.765101	A:0.234899	
chr28	11156387	2	280	TA:0.682143	T:0.317857	
chr28	11157509	2	298	G:0.755034	A:0.244966	
chr28	11157784	2	288	A:0.802083	T:0.197917	
chr28	11157947	2	292	G:0.458904	A:0.541096	
chr28	11158269	2	306	C:0.761438	G:0.238562	
chr28	11158274	2	306	C:0.761438	A:0.238562	
chr28	11158471	2	314	TC:0.990446	T:0.00955414	
chr28	11159109	2	294	T:0.435374	C:0.564626	
chr28	11159293	2	268	G:0.936567	A:0.0634328	
chr28	11159412	2	248	G:0.455645	T:0.544355	
chr28	11159430	2	252	T:0.535714	C:0.464286	
chr28	11159637	2	302	C:0.798013	T:0.201987	
chr28	11159720	2	302	T:0	TA:1	
chr28	11159746	2	296	T:0	A:1	
chr28	11159748	2	296	T:0	G:1	
chr28	11159924	2	302	T:0.774834	C:0.225166	
chr28	11160453	3	304	GTTT:0.371711	G:0.536184	GTTTT:0.0921053
chr28	11160461	2	300	T:0.456667	A:0.543333	
chr28	11160509	2	300	G:0.776667	T:0.223333	
chr28	11160610	2	284	C:0.46831	T:0.53169	
chr28	11160754	2	302	A:0.456954	T:0.543046	
chr28	11161130	2	300	C:0.783333	T:0.216667	
chr28	11161179	2	296	C:0.780405	T:0.219595	
chr28	11161218	2	290	G:0.437931	A:0.562069	
chr28	11161643	2	276	T:0.771739	C:0.228261	
chr28	11161658	2	238	T:0.907563	TTTC:0.092437	
chr28	11161676	3	270	C:0.411111	CTTCT:0.22963	CTTCTT:0.359259
chr28	11161715	2	186	G:0.634409	GGA:0.365591	
chr28	11161760	2	166	G:0.590361	T:0.409639	
chr28	11162339	2	296	T:0.547297	C:0.452703	
chr28	11162664	2	296	G:0.810811	A:0.189189	
chr28	11162702	2	306	A:0.545752	G:0.454248	
chr28	11162782	2	294	A:0.360544	C:0.639456	
chr28	11162799	2	296	T:0.344595	C:0.655405	
chr28	11163218	2	302	G:0.774834	A:0.225166	
chr28	11163562	2	310	T:0.777419	C:0.222581	

chr28	11163603	2	316	G:0.231013	C:0.768987
chr28	11163783	2	304	TG:0.871711	T:0.128289
chr28	11164244	2	300	T:0.536667	C:0.463333
chr28	11164304	2	306	T:0.218954	C:0.781046
chr28	11164358	2	304	C:0.786184	T:0.213816
chr28	11164857	2	298	A:0.218121	G:0.781879
chr28	11164976	2	304	T:0.233553	C:0.766447
chr28	11165039	2	316	T:0.21519	C:0.78481
chr28	11165063	2	310	C:0.783871	T:0.216129
chr28	11165132	2	302	C:0.956954	T:0.0430464
chr28	11165204	2	306	T:0.24183	G:0.75817
chr28	11165964	2	308	T:0.782468	C:0.217532
chr28	11166141	2	290	C:0.965517	T:0.0344828
chr28	11166434	2	312	C:0.794872	T:0.205128
chr28	11166477	2	306	G:0.781046	C:0.218954
chr28	11166790	2	308	C:0.944805	T:0.0551948
chr28	11166849	2	298	A:0.775168	T:0.224832
chr28	11167414	2	268	G:0.481343	A:0.518657
chr28	11168171	2	304	A:0.476974	G:0.523026
chr28	11168230	2	298	AT:0.536913	A:0.463087
chr28	11168895	2	302	G:0.990066	A:0.00993377
chr28	11169451	2	296	T:0.266892	C:0.733108
chr28	11169480	2	310	G:0.777419	A:0.222581
chr28	11169653	2	302	C:0.271523	T:0.728477
chr28	11169739	2	300	C:0.766667	T:0.233333
chr28	11170681	2	300	G:0.57	A:0.43
chr28	11171008	2	302	A:0.771523	G:0.228477
chr28	11171327	2	306	T:0.921569	C:0.0784314
chr28	11171402	2	298	G:0.278523	T:0.721477
chr28	11172563	2	312	G:0.983974	A:0.0160256
chr28	11173106	2	318	T:0.408805	C:0.591195
chr28	11173138	2	312	CCTCCT:0.852564	C:0.147436
chr28	11173139	2	314	C:0.617834	T:0.382166
chr28	11173145	2	314	T:0.617834	C:0.382166
chr28	11173146	2	312	CTTTCTTTCTTTCTTTCTTTCTTTCTTTCTTT:0.852564	C:0.147436
chr28	11173149	2	314	T:0.617834	C:0.382166
chr28	11173160	2	274	TTCTTTCTTTCTTTCTTTCTTTCTTTCTTTCTTTCTTTCTTTCTTTCTTTCTTTCTTTC:0.883212	T:0.116788
chr28	11173164	2	274	TTCTTTCTTTCTTTCTTTCTTTCTTTCTTTCTTTCTTTCTTTCTTTCTTTCTTTC:0.824818	T:0.175182
chr28	11173181	2	312	T:0.852564	C:0.147436
chr28	11173185	2	312	T:0.852564	C:0.147436
chr28	11173206	2	316	CT:0.958861	C:0.0411392
chr28	11173215	2	316	TTTC:0.958861	T:0.0411392
chr28	11173562	2	296	C:0.655405	T:0.344595
chr28	11173968	2	312	CA:0.586538	C:0.413462
chr28	11174166	2	308	C:0.688312	T:0.311688
chr28	11174171	2	308	T:0.688312	G:0.311688
chr28	11174481	2	316	AT:0.585443	A:0.414557
chr28	11174534	2	302	C:0.976821	T:0.0231788
chr28	11174882	2	312	A:0.730769	G:0.269231

chr28 11174963 2 298 G:0.275168 A:0.724832
chr28 11175147 2 310 G:0.564516 A:0.435484
chr28 11175525 2 316 AACTATGC:0.582278 A:
0.417722
chr28 11176031 2 306 C:0.584967 T:0.415033
chr28 11177011 2 284 C:0.693662 T:0.306338
chr28 11177390 2 294 A:0.261905 G:0.738095
chr28 11178063 2 302 G:0.288079 A:0.711921
chr28 11178400 2 294 C:0.738095 T:0.261905
chr28 11178599 2 304 A:0.717105 G:0.282895
chr28 11178762 2 304 G:0.717105 A:0.282895
chr28 11179142 2 306 A:0.754902 C:0.245098
chr28 11179183 2 304 T:0 C:1
chr28 11179361 2 308 T:0.74026 C:0.25974
chr28 11180159 2 300 G:0.96 GT:0.04
chr28 11180194 3 298 CAA:0.033557 C:0.308725
CA:0.657718
chr28 11180350 2 296 GGGA:0.739865 G:0.260135
chr28 11181319 2 276 AC:0.724638 A:0.275362
chr28 11181590 2 286 C:0.70979 T:0.29021
chr28 11182207 2 300 T:0.713333 C:0.286667
chr28 11182463 2 300 G:0.753333 A:0.246667
chr28 11183194 2 294 T:0.789116 C:0.210884
chr28 11183281 2 306 C:0.960784 T:0.0392157
chr28 11183904 2 294 A:0.244898 G:0.755102
chr28 11183920 2 306 A:0.993464 ATGCTTTGCTT:
0.00653595
chr28 11183967 2 308 T:0.844156
TCTTTTCTTTTCTTTTCTTTC:0.155844
chr28 11184001 2 312 T:0.961538 TCTTTTCTTTC:
0.0384615
chr28 11184021 3 312 T:0.599359
TTTTCTTTCTTTCTTTCTTTCTTTCTTTC:0.346154 TTTTCTTTCTTTCTTTCTTTCTTC:
0.0544872
chr28 11184055 2 302 T:0.953642 TTCTTTCTTTCTTTC:
0.0463576
chr28 11184072 2 302 C:0.953642 T:0.0463576
chr28 11184191 2 280 C:0.767857 A:0.232143
chr28 11184323 2 306 C:0.254902 A:0.745098
chr28 11184763 2 302 G:0.824503 C:0.175497
chr28 11184784 2 308 G:0.74026 A:0.25974
chr28 11184800 2 308 T:0.74026 C:0.25974
chr28 11184949 2 314 A:0.726115 AT:0.273885
chr28 11185127 2 302 T:0.996689 C:0.00331126
chr28 11185181 2 310 A:0.741935 T:0.258065
chr28 11185384 3 284 A:0.429577 AGT:0.34507
AGTGT:0.225352
chr28 11185526 2 290 A:0.7 G:0.3
chr28 11186189 2 304 AG:0.960526 A:0.0394737
chr28 11186315 2 304 T:0.5 G:0.5
chr28 11186991 2 296 A:0.209459 AT:0.790541
chr28 11187145 2 296 T:0.668919 A:0.331081
chr28 11187384 3 282 TAC:0.322695 T:0.397163
TACAC:0.280142

chr28	11187441	2	284	T:0.683099	C:0.316901
chr28	11187765	2	306	A:0.934641	G:0.0653595
chr28	11187923	2	308	A:0.970779	G:0.0292208
chr28	11188230	2	308	A:0.188312	T:0.811688
chr28	11188254	2	306	T:0.767974	TTG:0.232026
chr28	11188271	2	296	A:0.0472973	T:0.952703
chr28	11188390	2	290	T:0.965517	C:0.0344828
chr28	11188604	2	298	A:0.95302	AT:0.0469799
chr28	11188775	2	300	C:0.96	T:0.04
chr28	11189214	2	292	C:0.962329	T:0.0376712
chr28	11189290	2	304	A:0.546053	ATGC:0.453947
chr28	11189444	2	300	AC:0.96	A:0.04
chr28	11189943	2	308	A:0.194805	G:0.805195
chr28	11189985	2	300	G:0.996667	C:0.00333333
chr28	11190134	2	306	G:0.74183	C:0.25817
chr28	11190159	2	302	T:0.963576	A:0.0364238
chr28	11190487	2	292	A:0.19863	G:0.80137
chr28	11190612	2	306	C:0.513072	T:0.486928
chr28	11191046	2	308	C:0.753247	A:0.246753
chr28	11191548	2	298	G:0.97651	A:0.0234899
chr28	11191866	2	296	A:0.925676	G:0.0743243
chr28	11191907	2	298	A:0.969799	G:0.0302013
chr28	11192501	2	300	T:0.186667	C:0.813333
chr28	11193109	2	298	G:0.862416	A:0.137584
chr28	11193126	2	292	C:0.969178	T:0.0308219
chr28	11193387	2	298	A:0.0033557	C:0.996644
chr28	11193427	2	306	C:0.859477	T:0.140523
chr28	11193547	2	310	A:0.96129	G:0.0387097
chr28	11193767	2	304	C:0.851974	G:0.148026
chr28	11194490	2	304	C:0.621711	T:0.378289
chr28	11194601	2	312	G:0.740385	A:0.259615
chr28	11194606	2	308	C:0.165584	CA:0.834416
chr28	11194629	2	298	C:0.969799	T:0.0302013
chr28	11194866	2	308	G:0.866883	A:0.133117
chr28	11195436	2	216	CG:0.680556	C:0.319444
chr28	11195609	2	280	A:0.853571	T:0.146429
chr28	11195630	2	288	T:0.850694	A:0.149306
chr28	11195750	2	302	TTC:0.460265	T:0.539735
chr28	11195893	2	310	C:0.619355	T:0.380645
chr28	11196142	2	304	C:0.667763	T:0.332237
chr28	11196217	2	314	T:0.191083	C:0.808917
chr28	11196473	2	306	T:0.457516	C:0.542484
chr28	11196601	2	306	CTG:0.836601	C:0.163399
chr28	11197557	2	312	C:0.961538	G:0.0384615
chr28	11197560	2	312	T:0.721154	C:0.278846
chr28	11197658	2	312	A:0.974359	G:0.025641
chr28	11198089	2	308	G:0.977273	A:0.0227273
chr28	11198252	2	312	T:0.852564	A:0.147436
chr28	11198341	2	314	TTA:0.719745	T:0.280255
chr28	11198570	2	304	T:0.733553	C:0.266447
chr28	11198628	2	304	A:0.1875	G:0.8125
chr28	11198887	2	304	T:0.651316	C:0.348684
chr28	11199236	2	308	AAAG:0.970779	A:0.0292208
chr28	11199522	2	298	T:0.194631	TAC:0.805369

chr28	11199864	2	292	A:0.715753	T:0.284247	
chr28	11199915	2	300	T:0.183333	C:0.816667	
chr28	11200037	2	310	C:0.703226	T:0.296774	
chr28	11200262	2	306	T:0.196078	A:0.803922	
chr28	11200286	2	306	C:0.964052	T:0.0359477	
chr28	11200366	2	296	C:0.989865	T:0.0101351	
chr28	11200434	2	302	ATAG:0.665563	A:0.334437	
chr28	11200515	2	306	A:0.849673	C:0.150327	
chr28	11200585	2	298	A:0.708054	AAG:0.291946	
chr28	11200769	2	280	AT:0.664286	A:0.335714	
chr28	11200827	2	292	C:0.876712	T:0.123288	
chr28	11201104	2	302	G:0.725166	A:0.274834	
chr28	11201365	2	312	T:0.983974	C:0.0160256	
chr28	11201602	2	308	T:0.967532	C:0.0324675	
chr28	11201640	2	310	C:0.977419	T:0.0225806	
chr28	11201650	2	308	A:0.821429	G:0.178571	
chr28	11201836	2	292	G:0.630137	A:0.369863	
chr28	11202021	2	272	CAAAAA:0.75	C:0.25	
chr28	11202039	2	272	CAA:0.75	C:0.25	
chr28	11202048	2	282	C:0.663121	A:0.336879	
chr28	11202051	2	272	AAAAAAC:0.75	A:0.25	
chr28	11202054	3	284	A:0.665493	AAC:0.221831	AACAAC:0.112676
chr28	11202060	2	284	A:0.81338	C:0.18662	
chr28	11202122	2	240	C:0.625	T:0.375	
chr28	11202791	2	300	T:0.223333	C:0.776667	
chr28	11202793	2	300	G:0.97	T:0.03	
chr28	11203019	2	296	G:0.679054	T:0.320946	
chr28	11203521	2	294	A:0.187075	G:0.812925	
chr28	11203603	2	318	T:0.72327	C:0.27673	
chr28	11204127	2	290	A:0.82069	G:0.17931	
chr28	11204563	2	306	A:0.980392	T:0.0196078	
chr28	11204776	2	302	G:0.619205	A:0.380795	
chr28	11204816	2	304	T:0.184211	A:0.815789	
chr28	11205012	2	312	ATGTC:0.974359	A:0.025641	
chr28	11205127	2	306	A:0.702614	G:0.297386	
chr28	11205576	2	300	C:0.616667	T:0.383333	
chr28	11205841	2	298	A:0.875839	C:0.124161	
chr28	11206426	2	300	A:0.363333	G:0.636667	
chr28	11206441	2	304	T:0.996711	G:0.00328947	
chr28	11207252	2	282	T:0.843972	G:0.156028	
chr28	11207309	2	300	A:0.97	T:0.03	
chr28	11207665	2	296	A:0.00337838	C:0.996622	
chr28	11207955	2	310	C:0.977419	T:0.0225806	
chr28	11208138	2	316	A:0.664557	T:0.335443	
chr28	11208478	3	304	TA:0.286184	T:0.473684	TAA:0.240132
chr28	11209025	2	306	C:0.996732	T:0.00326797	
chr28	11209347	2	300	G:0.00333333	A:0.996667	
chr28	11209461	2	302	G:0.00331126	A:0.996689	
chr28	11209546	2	312	C:0.974359	T:0.025641	
chr28	11209700	2	304	G:0.970395	A:0.0296053	
chr28	11209761	2	300	C:0.99	A:0.01	
chr28	11210712	2	300	G:0.963333	T:0.0366667	

chr28	11211044	2	306	C:0.892157	T:0.107843		
chr28	11211268	2	310	G:0.016129	GAACAA:0.983871		
chr28	11211479	2	314	G:0.812102	A:0.187898		
chr28	11211761	2	308	G:0.977273	A:0.0227273		
chr28	11211804	2	296	CT:0.648649	C:0.351351		
chr28	11211807	3	300	TAA:0.406667	T:0.54	TA: 0.0533333	
chr28	11211844	2	298	A:0.600671	AGAGAGAGC: 0.399329		
chr28	11212061	2	302	G:0.645695	A:0.354305		
chr28	11212096	2	300	C:0.986667	A:0.0133333		
chr28	11212224	3	282	A:0.560284	AT:0.361702	ATT:0.0780142	
chr28	11212567	2	308	G:0.899351	A:0.100649		
chr28	11212891	2	296	G:0.952703	A:0.0472973		
chr28	11213287	2	310	C:0.996774	T:0.00322581		
chr28	11213555	2	310	A:0.977419	G:0.0225806		
chr28	11213752	2	314	T:0.401274	C:0.598726		
chr28	11214289	2	306	C:0.594771	T:0.405229		
chr28	11214381	2	306	AAGG:0.764706	A:0.235294		
chr28	11215670	2	298	A:0.214765	G:0.785235		
chr28	11215677	2	298	G:0.61745	A:0.38255		
chr28	11215769	2	300	G:0.806667	A:0.193333		
chr28	11215833	4	300	CTTTT:0.1	C:0.366667	CT:0.356667	CTT:0.176667
chr28	11216573	2	306	C:0.980392	T:0.0196078		
chr28	11216594	2	296	T:0.00337838	C:0.996622		
chr28	11216783	2	290	C:0.575862	T:0.424138		
chr28	11217136	2	248	C:0.596774	CT:0.403226		
chr28	11217137	2	258	T:0.717054	TTC:0.282946		
chr28	11217798	2	298	A:0.66443	T:0.33557		
chr28	11217871	2	302	C:0.427152	T:0.572848		
chr28	11217999	2	302	A:0.430464	C:0.569536		
chr28	11218337	2	308	A:0.538961	G:0.461039		
chr28	11218584	2	300	G:0.43	A:0.57		
chr28	11218635	2	298	G:0.42953	A:0.57047		
chr28	11218794	2	306	C:0.434641	A:0.565359		
chr28	11218823	2	304	G:0.621711	A:0.378289		
chr28	11218863	2	298	C:0.0033557	A:0.996644		
chr28	11219026	2	298	T:0.473154	G:0.526846		
chr28	11219082	2	290	G:0.468966	A:0.531034		
chr28	11219280	2	294	C:0.812925	G:0.187075		
chr28	11219290	2	296	A:0.554054	G:0.445946		
chr28	11219442	2	292	CA:0.534247	C:0.465753		
chr28	11219505	2	300	C:0.576667	T:0.423333		
chr28	11219509	2	300	A:0.576667	G:0.423333		
chr28	11219847	3	282	GT:0.43617	G:0.180851	GTT:0.382979	
chr28	11220133	2	294	C:0.744898	T:0.255102		
chr28	11220284	2	302	C:0.94702	T:0.0529801		
chr28	11220447	2	302	C:0.801325	T:0.198675		
chr28	11220625	2	300	C:0.57	T:0.43		
chr28	11220870	2	294	A:0.707483	C:0.292517		
chr28	11220884	2	296	A:0.584459	G:0.415541		

chr28 11220953 2 300 G:0.563333 A:0.436667
chr28 11221023 2 294 T:0.55102 C:0.44898
chr28 11221156 2 308 C:0.961039 G:0.038961
chr28 11221493 3 298 C:0.348993 CT:0.560403
CTT:0.090604
chr28 11221594 3 302 GTCTCTC:0.460265 G:0.291391
GTC:0.248344
chr28 11221603 2 264 TCTCTCTCACA:0.939394 T:
0.0606061
chr28 11221609 2 290 T:0.672414 A:0.327586
chr28 11222176 2 302 G:0.751656 T:0.248344
chr28 11222232 2 302 CAG:0.940397 C:0.0596026
chr28 11222269 2 302 C:0.698675 T:0.301325
chr28 11222455 2 300 T:0.26 C:0.74
chr28 11222692 2 306 G:0.19281 A:0.80719
chr28 11222935 2 302 G:0.953642 A:0.0463576
chr28 11222971 2 310 T:0.203226 C:0.796774
chr28 11223019 2 318 A:0.578616 ACCCCCATTCACCCT:
0.421384
chr28 11223059 2 304 A:0.259868 C:0.740132
chr28 11223154 2 292 G:0.315068 A:0.684932
chr28 11223202 2 308 T:0.331169 G:0.668831
chr28 11223225 2 308 T:0.974026 G:0.025974
chr28 11223363 2 298 C:0.741611 T:0.258389
chr28 11223455 2 300 T:0.56 C:0.44
chr28 11223465 2 300 G:0.56 A:0.44
chr28 11223475 2 300 G:0.943333 A:0.0566667
chr28 11224210 2 312 T:0.942308 C:0.0576923
chr28 11224549 2 304 C:0.953947 T:0.0460526
chr28 11225235 2 296 A:0.472973 G:0.527027
chr28 11225598 2 304 C:0.960526 T:0.0394737
chr28 11225914 2 274 C:0.737226 CCCA:0.262774
chr28 11226115 2 292 A:0.945205 T:0.0547945
chr28 11226301 2 258 C:0.806202 T:0.193798
chr28 11228114 2 226 G:0.628319 GC:0.371681
chr28 11228121 2 238 CG:0.97479 C:0.0252101
chr28 11228125 2 234 C:0.645299 G:0.354701
chr28 11228255 2 242 T:0.797521 A:0.202479
chr28 11228427 2 286 G:0.968531 C:0.0314685
chr28 11228779 4 310 AAC:0.609677 A:0.0290323
AACAC:0.0387097 AACACACAC:0.322581
chr28 11229233 2 300 C:0.926667 T:0.0733333
chr28 11229617 2 288 A:0.0104167 T:0.989583
chr28 11229875 2 288 C:0.465278 A:0.534722
chr28 11229901 2 288 T:0.611111 C:0.388889
chr28 11229938 2 292 G:0.64726 GC:0.35274
chr28 11230222 2 308 C:0.412338 T:0.587662
chr28 11230368 2 302 T:0.976821 C:0.0231788
chr28 11230391 2 304 A:0.970395 C:0.0296053
chr28 11230393 2 304 TC:0.970395 T:0.0296053
chr28 11230722 2 288 C:0.392361 T:0.607639
chr28 11230730 2 290 C:0.975862 T:0.0241379
chr28 11230739 2 284 C:0.0105634 T:0.989437
chr28 11230799 2 310 GTGTCTCTGTCTC:0.603226 G:

0.396774
chr28 11230977 2 292 C:0.770548 T:0.229452
chr28 11231111 2 294 A:0 G:1
chr28 11231332 2 260 T:0.646154 G:0.353846
chr28 11231353 2 290 AC:0.958621 A:0.0413793
chr28 11231357 3 290 A:0.937931 C:0.0413793
AAC:0.0206897
chr28 11231362 3 290 C:0.434483 A:0.52069
CA:0.0448276
chr28 11231364 2 290 AAC:0.97931 A:0.0206897
chr28 11231505 2 298 C:0.651007 T:0.348993
chr28 11231888 2 308 T:0.487013 C:0.512987
chr28 11231912 2 312 G:0.483974 A:0.516026
chr28 11232195 2 314 G:0.987261 A:0.0127389
chr28 11232372 2 302 C:0.523179 G:0.476821
chr28 11232389 2 302 C:0.536424 T:0.463576
chr28 11232628 2 298 C:0.526846 T:0.473154
chr28 11232632 2 298 T:0.526846 C:0.473154
chr28 11232796 2 298 A:0.533557 G:0.466443
chr28 11232823 2 300 A:0.866667 G:0.133333
chr28 11232935 2 302 C:0.516556 T:0.483444
chr28 11233037 2 304 A:0.539474 G:0.460526
chr28 11233049 2 304 A:0.539474 G:0.460526
chr28 11233051 2 304 T:0.539474 C:0.460526
chr28 11233069 2 304 T:0.539474 C:0.460526
chr28 11233204 2 308 G:0.99026 T:0.00974026
chr28 11233366 2 296 A:0.496622 T:0.503378
chr28 11233477 2 300 C:0.523333 T:0.476667
chr28 11234054 3 284 T:0.485915 TA:0.454225
TAA:0.0598592
chr28 11234137 2 298 A:0.0436242 C:0.956376
chr28 11234325 2 308 C:0.519481 G:0.480519
chr28 11234378 2 302 G:0.503311 T:0.496689
chr28 11234982 2 284 G:0.517606 A:0.482394
chr28 11234992 2 284 G:0.517606 A:0.482394
chr28 11235094 2 256 T:0.503906 C:0.496094
chr28 11235251 2 300 T:0.486667 C:0.513333
chr28 11235275 2 302 G:0.798013 A:0.201987
chr28 11235614 2 294 A:0.496599 G:0.503401
chr28 11235736 2 294 C:0.452381 T:0.547619
chr28 11235988 2 304 T:0.470395 C:0.529605
chr28 11236283 2 290 G:0.534483 T:0.465517
chr28 11236285 2 290 G:0.534483 T:0.465517
chr28 11236615 2 304 G:0.5 A:0.5
chr28 11236617 2 304 A:0.5 C:0.5
chr28 11237023 2 308 C:0.535714 T:0.464286
chr28 11237126 2 304 A:0.532895 AT:0.467105
chr28 11237137 2 304 T:0.532895 G:0.467105
chr28 11237162 2 304 C:0.532895 G:0.467105
chr28 11237215 2 308 A:0.538961 C:0.461039
chr28 11237220 2 308 C:0.538961 G:0.461039
chr28 11237236 2 302 A:0.0165563 G:0.983444
chr28 11237285 2 310 T:0.519355 TAA:0.480645
chr28 11237310 2 310 A:0.519355 T:0.480645

chr28 11237500 2 296 T:0.989865 C:0.0101351
chr28 11237567 2 310 A:0.516129 G:0.483871
chr28 11237652 2 298 T:0.526846 C:0.473154
chr28 11237739 2 304 G:0.967105 A:0.0328947
chr28 11237785 2 302 A:0.993377 T:0.00662252
chr28 11237827 2 290 A:0.493103 G:0.506897
chr28 11237836 2 290 C:0.882759 T:0.117241
chr28 11237954 2 300 G:0.513333 C:0.486667
chr28 11238024 2 306 T:0.51634 G:0.48366
chr28 11238028 2 306 G:0.51634 A:0.48366
chr28 11238594 2 304 T:0.539474 C:0.460526
chr28 11238761 2 284 G:0.545775 C:0.454225
chr28 11238777 2 300 C:0.973333 T:0.0266667
chr28 11238894 2 300 G:0.533333 A:0.466667
chr28 11238981 2 298 G:0.520134 A:0.479866
chr28 11239148 2 304 G:0.536184 A:0.463816
chr28 11239320 2 300 G:0.523333 A:0.476667
chr28 11239352 3 276 GT:0.456522 G:0.0905797
GTT:0.452899
chr28 11239856 2 300 TA:0.983333 T:0.0166667
chr28 11240512 2 270 C:0.533333 T:0.466667
chr28 11241265 2 296 G:0.952703 T:0.0472973
chr28 11241476 2 286 G:0.548951 A:0.451049
chr28 11241518 2 264 C:0.969697 T:0.030303
chr28 11241600 2 234 G:0.534188 C:0.465812
chr28 11241628 2 304 T:0.901316 TAAAAAA:0.0986842
chr28 11241629 3 306 T:0.617647 A:0.281046
TAAA:0.101307
chr28 11241715 2 306 G:0.0196078 A:0.980392
chr28 11241740 2 304 A:0.5 T:0.5
chr28 11241788 2 270 T:0.559259 A:0.440741
chr28 11241891 2 298 C:0.563758 T:0.436242
chr28 11242060 2 294 G:0.0340136 T:0.965986
chr28 11242096 2 300 G:0.966667 A:0.0333333
chr28 11242382 2 300 AG:0.976667 A:0.0233333
chr28 11243014 2 290 T:0.955172 C:0.0448276
chr28 11243101 5 314 CT:0.248408 C:0.0477707
CTT:0.318471 CTTT:0.10828 CTTTT:0.27707
chr28 11244344 2 292 C:0.969178 T:0.0308219
chr28 11244417 2 290 A:0.975862 G:0.0241379
chr28 11244560 2 292 ATCTCTCTCTC:0.363014 A:
0.636986
chr28 11244902 2 304 G:0.549342 A:0.450658
chr28 11245275 2 306 C:0.519608 T:0.480392
chr28 11245411 2 296 G:0.506757 C:0.493243
chr28 11245427 2 298 C:0.986577 T:0.0134228
chr28 11245898 2 304 C:0.486842 T:0.513158
chr28 11245938 3 304 G:0.950658 A:0.0394737
T:0.00986842
chr28 11245946 2 302 C:0.817881 A:0.182119
chr28 11246330 2 296 G:0.993243 A:0.00675676
chr28 11246627 2 294 A:0.97619 G:0.0238095
chr28 11246725 2 294 G:0.503401 C:0.496599
chr28 11246979 2 286 T:0.979021 G:0.020979

chr28	11247420	2	298	G:0.973154	A:0.0268456		
chr28	11247430	2	308	A:0.987013	G:0.012987		
chr28	11247484	2	316	G:0.737342	A:0.262658		
chr28	11247492	2	314	T:0.993631	C:0.00636943		
chr28	11247544	2	302	T:0.956954	C:0.0430464		
chr28	11248338	2	304	C:0.993421	T:0.00657895		
chr28	11248459	2	304	GTCAAA:0.486842	G:0.513158		
chr28	11248466	2	304	G:0.486842	GGCC:0.513158		
chr28	11248468	2	304	C:0.486842	CAG:0.513158		
chr28	11248509	2	306	G:0.761438	A:0.238562		
chr28	11248833	2	300	C:0.97	T:0.03		
chr28	11248948	2	312	G:0.964744	C:0.0352564		
chr28	11249268	2	300	C:0.993333	T:0.00666667		
chr28	11249413	2	304	C:0.973684	G:0.0263158		
chr28	11249510	2	298	C:0.993289	A:0.00671141		
chr28	11249816	2	306	T:0.905229	C:0.0947712		
chr28	11249942	2	300	T:0.963333	C:0.0366667		
chr28	11249996	2	298	T:0.661074	TTC:0.338926		
chr28	11250040	2	316	G:0.727848	GTCTC:0.272152		
chr28	11250044	2	310	C:0.993548	CTG:0.00645161		
chr28	11250047	2	280	TCTCACACACACA:0.821429	T:0.178571		
chr28	11250049	2	306	TCACA:0.986928	T:0.0130719		
chr28	11250055	2	308	A:0.99026	T:0.00974026		
chr28	11250057	2	308	A:0.99026	T:0.00974026		
chr28	11250059	2	308	A:0.99026	T:0.00974026		
chr28	11250061	2	284	A:0.992958	T:0.00704225		
chr28	11250063	2	274	A:0.989051	T:0.0109489		
chr28	11250065	2	274	A:0.989051	T:0.0109489		
chr28	11250313	2	298	C:0.959732	T:0.0402685		
chr28	11250331	2	296	T:0.75	C:0.25		
chr28	11250705	2	300	A:0.95	G:0.05		
chr28	11251322	2	300	T:0.99	C:0.01		
chr28	11251447	2	298	T:0.989933	C:0.0100671		
chr28	11251462	2	296	G:0.935811	A:0.0641892		
chr28	11251590	2	298	GT:0.95302	G:0.0469799		
chr28	11251993	2	310	C:0.990323	T:0.00967742		
chr28	11252102	2	302	T:0.983444	C:0.0165563		
chr28	11252187	2	310	C:0.751613	G:0.248387		
chr28	11252656	2	314	C:0.974522	A:0.0254777		
chr28	11252805	4	316	CTTCT:0.75	C:0.0474684	CTTTCT:0.136076	CTTCTTTCT:0.0664557
chr28	11252860	2	316	C:0.987342	CT:0.0126582		
chr28	11253422	2	300	G:0.946667	T:0.0533333		
chr28	11253544	2	306	G:0.941176	C:0.0588235		
chr28	11253727	2	294	C:0.755102	G:0.244898		
chr28	11253832	2	302	C:0.741722	T:0.258278		
chr28	11253984	2	296	G:0.523649	A:0.476351		
chr28	11254421	2	304	C:0.990132	T:0.00986842		
chr28	11254589	2	298	G:0.959732	A:0.0402685		
chr28	11254881	2	302	A:0.824503	G:0.175497		
chr28	11255004	2	306	G:0.95098	C:0.0490196		
chr28	11255252	2	302	GC:0.983444	G:0.0165563		
chr28	11255254	2	302	C:0.983444	A:0.0165563		

chr28 11255404 2 304 T:0.743421 C:0.256579
chr28 11255687 2 294 CT:0.986395 C:0.0136054
chr28 11255734 2 302 T:0.745033 C:0.254967
chr28 11256193 2 306 T:0.960784 A:0.0392157
chr28 11256194 2 308 A:0.746753 G:0.253247
chr28 11256428 2 306 G:0.718954 A:0.281046
chr28 11256480 2 310 T:0.190323 A:0.809677
chr28 11256678 2 302 G:0.711921 A:0.288079
chr28 11256791 2 220 AG:0.818182 A:0.181818
chr28 11257115 2 312 G:0.99359 A:0.00641026
chr28 11257131 2 308 A:0.714286 G:0.285714
chr28 11257256 2 300 A:0.73 G:0.27
chr28 11257622 2 302 T:0.738411 A:0.261589
chr28 11257676 2 298 T:0.761745 C:0.238255
chr28 11257751 2 306 G:0.75817 A:0.24183
chr28 11257777 2 310 T:0.735484 C:0.264516
chr28 11257868 2 308 T:0.363636 C:0.636364
chr28 11257917 2 306 A:0.745098 T:0.254902
chr28 11257919 2 306 A:0.745098 C:0.254902
chr28 11257986 2 298 C:0.842282 T:0.157718
chr28 11258344 2 284 G:0.767606 GT:0.232394
chr28 11258384 2 304 GATGGGTAA:0.888158 G:
0.111842
chr28 11258487 2 282 C:0.989362 T:0.0106383
chr28 11258540 2 276 A:0 G:1
chr28 11258557 2 274 A:0.711679 G:0.288321
chr28 11258598 2 296 A:0 G:1
chr28 11258631 2 288 A:0.975694 C:0.0243056
chr28 11259057 2 268 C:0.768657 A:0.231343
chr28 11259219 2 278 G:0.931655 A:0.0683453
chr28 11259309 2 292 C:0.996575 T:0.00342466
chr28 11259354 2 286 T:0.22028 C:0.77972
chr28 11259463 2 288 G:0.711806 A:0.288194
chr28 11259464 2 288 T:0.711806 C:0.288194
chr28 11259560 2 318 T:0.801887 TC:0.198113
chr28 11259577 3 320 C:0.3125 CT:0.634375
CTTTTCTTTTCTTTTCT:0.053125
chr28 11259591 2 318 C:0.801887 CT:0.198113
chr28 11259596 4 320 C:0.540625 T:0.196875
CTTTTCTTTTCTTTCTTTTCTTTTCTTCTT:0.2 CTTTCTTTTCTTTTCTTCTT:
0.0625
chr28 11259606 2 318 T:0.801887 TTTC:0.198113
chr28 11259619 3 316 C:0.718354 CTTTCT:0.193038
CTTTCTTTTCT:0.0886076
chr28 11259668 2 304 TTCTC:0.802632 T:0.197368
chr28 11259670 4 308 C:0.538961 CTCCTT:0.191558
CTCCTTTCCTT:0.246753 CTCCTTTCCTTTCCTT:0.0227273
chr28 11259675 2 304 T:0.802632 TTTC:0.197368
chr28 11259784 2 288 G:0.5 T:0.5
chr28 11260071 2 284 G:0.757042 T:0.242958
chr28 11260086 2 312 T:0.102564 TTTTAGATC:
0.897436
chr28 11260125 2 294 C:0.755102 T:0.244898
chr28 11260401 2 288 CTA:0.881944 C:0.118056

chr28	11260415	2	278	CTTT:0.960432	C:0.0395683		
chr28	11260667	2	300	G:0.193333	A:0.806667		
chr28	11260926	2	300	G:0.996667	A:0.00333333		
chr28	11261438	2	300	A:0.77	AC:0.23		
chr28	11261560	2	308	G:0.746753	GTT:0.253247		
chr28	11261562	4	308	A:0.220779	AT:0.441558	ATT:0.0811688	T:0.256494
chr28	11262845	3	314	C:0.328025	CT:0.582803	CTT:0.089172	
chr28	11263466	2	308	G:0.951299	A:0.0487013		
chr28	11263663	2	300	A:0.986667	C:0.0133333		
chr28	11263809	2	306	C:0.98366	G:0.0163399		
chr28	11264751	2	302	C:0.711921	CTTTTTTTTTTTTTT:0.288079		
chr28	11264822	2	290	G:0.158621	C:0.841379		
chr28	11265008	2	304	C:0.269737	T:0.730263		
chr28	11265410	2	300	C:0.26	T:0.74		
chr28	11266285	2	308	C:0.0616883	CA:0.938312		
chr28	11266576	2	304	TATC:0.907895	T:0.0921053		
chr28	11266609	3	318	C:0.855346	CATT:0.0943396	CATCATT:0.0503145	
chr28	11266641	2	302	A:0.880795	G:0.119205		
chr28	11266863	2	308	G:0.905844	A:0.0941558		
chr28	11267548	2	306	T:0.95098	C:0.0490196		
chr28	11269073	2	318	TTAATACCTGA:0.968553	T:0.0314465		
chr28	11269991	2	304	C:0.174342	T:0.825658		
chr28	11270027	2	304	C:0.986842	T:0.0131579		
chr28	11270760	2	304	G:0.868421	GAA:0.131579		
chr28	11270764	3	302	C:0.0165563	A:0.165563	CA:0.817881	
chr28	11271011	2	302	G:0.897351	A:0.102649		
chr28	11271584	2	304	G:0.174342	A:0.825658		
chr28	11272131	2	276	A:0.057971	G:0.942029		
chr28	11272608	2	310	G:0.890323	A:0.109677		
chr28	11274137	2	268	GT:0.664179	G:0.335821		
chr28	11274150	2	290	T:0.913793	G:0.0862069		
chr28	11274151	3	296	T:0.516892	G:0.415541	TG:0.0675676	
chr28	11274749	2	304	A:0.993421	G:0.00657895		
chr28	11274914	2	302	T:0.996689	C:0.00331126		
chr28	11274951	2	306	C:0.111111	T:0.888889		
chr28	11274968	2	312	A:0.0512821	ATG:0.948718		
chr28	11275014	2	300	G:0.133333	A:0.866667		
chr28	11275204	2	304	A:0.9375	C:0.0625		
chr28	11275219	2	298	A:0.251678	G:0.748322		
chr28	11275614	2	302	T:0.0596026	C:0.940397		
chr28	11275639	2	298	G:0.922819	A:0.0771812		
chr28	11276077	2	294	C:0.952381	T:0.047619		
chr28	11276601	2	300	T:0.92	G:0.08		
chr28	11276831	2	306	GAAGAAC:0.924837	G:0.0751634		
chr28	11276837	2	306	C:0.924837	CGTT:0.0751634		
chr28	11277354	2	298	A:0.862416	C:0.137584		
chr28	11277635	2	290	A:0.989655	G:0.0103448		

chr28	11277721	2	308	C:0.915584	T:0.0844156	
chr28	11278420	2	306	C:0.843137	T:0.156863	
chr28	11278902	2	296	G:0.935811	A:0.0641892	
chr28	11278938	2	298	C:0.92953	T:0.0704698	
chr28	11279515	2	292	A:0.976027	C:0.0239726	
chr28	11279669	2	286	T:0.923077	C:0.0769231	
chr28	11279683	2	292	T:0.931507	C:0.0684932	
chr28	11279726	2	212	GC:0.226415	G:0.773585	
chr28	11279768	2	306	T:0.947712	C:0.0522876	
chr28	11279772	2	306	AC:0.947712	A:0.0522876	
chr28	11279774	2	312	C:0.163462	A:0.836538	
chr28	11279778	2	306	C:0.947712	A:0.0522876	
chr28	11279779	2	312	C:0.163462	A:0.836538	
chr28	11279780	2	306	AAATT:0.947712	A:0.0522876	
chr28	11279784	2	312	T:0.163462	A:0.836538	
chr28	11279788	2	306	CCAAACCAAACCAAAT:0.947712	C:0.0522876	
chr28	11279789	2	298	C:0.0637584	A:0.936242	
chr28	11279794	2	298	C:0.0637584	A:0.936242	
chr28	11279799	2	292	C:0.150685	A:0.849315	
chr28	11279808	2	302	C:0.897351	T:0.102649	
chr28	11280003	2	290	C:0.944828	T:0.0551724	
chr28	11280036	2	292	C:0.941781	T:0.0582192	
chr28	11280704	2	302	GAGAGGA:0.910596	G:0.089404	
chr28	11280712	2	302	G:0.910596	T:0.089404	
chr28	11282085	2	298	C:0.92953	A:0.0704698	
chr28	11282186	2	308	A:0.941558	T:0.0584416	
chr28	11282191	2	308	A:0.948052	T:0.0519481	
chr28	11282475	2	306	G:0.908497	C:0.0915033	
chr28	11282869	2	302	G:0.92053	A:0.0794702	
chr28	11283417	2	306	CAT:0.915033	C:0.0849673	
chr28	11284267	2	308	C:0.935065	T:0.0649351	
chr28	11284521	2	310	T:0.896774	C:0.103226	
chr28	11284626	2	308	T:0.902597	C:0.0974026	
chr28	11284660	3	294	TAA:0.251701	T:0.173469	TA:0.57483
chr28	11285707	2	290	C:0.937931	T:0.062069	
chr28	11286498	2	294	T:0.993197	G:0.00680272	
chr28	11287034	2	290	C:0.851724	T:0.148276	
chr28	11287104	2	296	A:0.939189	G:0.0608108	
chr28	11287174	2	290	G:0.937931	A:0.062069	
chr28	11287793	2	254	C:0.893701	A:0.106299	
chr28	11288242	2	286	C:0.968531	A:0.0314685	
chr28	11289043	2	302	A:0	G:1	
chr28	11289271	2	304	T:0	C:1	
chr28	11291618	2	292	T:0.0821918	C:0.917808	
chr28	11291962	2	310	A:0	G:1	
chr28	11292808	2	296	G:0.986486	A:0.0135135	
chr28	11292889	2	306	C:0.977124	T:0.0228758	
chr28	11294552	2	298	CT:0.802013	C:0.197987	
chr28	11294976	2	268	TA:0.63806	T:0.36194	
chr28	11295338	2	308	C:0.987013	T:0.012987	
chr28	11295532	2	300	C:0.93	T:0.07	
chr28	11295568	2	308	C:0.198052	G:0.801948	

chr28	11295581	4	312	CTT:0.115385	C:0.570513
CT:0.11859	CTTTT:0.195513
chr28	11295610	2	308	A:0.801948	G:0.198052
chr28	11295881	2	304	G:0.786184	A:0.213816
chr28	11296048	2	316	T:0.775316	G:0.224684
chr28	11296086	3	308	AT:0.74026	A:0.0584416
ATT:0.201299
chr28	11296136	2	304	T:0.210526	C:0.789474
chr28	11296485	2	312	T:0.224359	G:0.775641
chr28	11296493	2	312	GA:0.224359	G:0.775641
chr28	11296594	2	310	G:0.816129	A:0.183871
chr28	11296597	2	310	C:0.816129	T:0.183871
chr28	11296669	2	316	T:0.806962	C:0.193038
chr28	11296786	2	302	A:0.215232	G:0.784768
chr28	11296838	2	302	C:0.443709	T:0.556291
chr28	11296852	2	302	G:0.771523	A:0.228477
chr28	11296855	2	302	A:0.771523	T:0.228477
chr28	11297063	2	306	C:0.24183	A:0.75817
chr28	11297074	2	308	T:0	C:1
chr28	11297102	2	304	T:0.230263	C:0.769737
chr28	11297140	2	296	A:0.222973	C:0.777027
chr28	11297177	2	306	C:0.215686	T:0.784314
chr28	11297189	2	308	T:0.178571	C:0.821429
chr28	11297276	2	310	C:0.816129	G:0.183871
chr28	11297294	2	310	A:0.816129	C:0.183871
chr28	11297441	2	302	C:0.0198675	CAAAT:0.980132
chr28	11297774	2	304	GT:0.996711	G:0.00328947
chr28	11297834	2	310	C:0.790323	T:0.209677
chr28	11297841	2	310	T:0	C:1
chr28	11297904	2	298	A:0	G:1
chr28	11297938	2	298	C:0.218121	T:0.781879
chr28	11298093	2	308	C:0.795455	T:0.204545
chr28	11298219	2	308	A:0.792208	AG:0.207792
chr28	11298538	2	300	C:0.783333	T:0.216667
chr28	11299765	2	314	C:0.993631	G:0.00636943
chr28	11299866	2	300	G:0.913333	A:0.0866667
chr28	11300176	2	306	G:0	A:1
chr28	11300427	2	310	A:0	G:1
chr28	11300494	2	318	T:0.00314465	C:0.996855
chr28	11300518	2	318	A:0.00314465	G:0.996855
chr28	11300531	2	318	G:0.00314465	A:0.996855
chr28	11300768	2	300	T:0.0566667	A:0.943333
chr28	11300844	2	300	C:0.00333333	T:0.996667
chr28	11301654	2	294	A:0.972789	G:0.0272109
chr28	11301685	2	268	GT:0.716418	G:0.283582
chr28	11301776	2	278	C:0.151079	CT:0.848921
chr28	11301899	2	284	C:0.0105634	T:0.989437
chr28	11302619	2	298	G:0.0469799	C:0.95302
chr28	11302652	2	292	TGGA:0	T:1
chr28	11302682	2	292	G:0.00684932	A:0.993151
chr28	11303627	2	298	A:0.728188	G:0.271812
chr28	11304834	2	302	A:0	G:1
chr28	11304909	2	300	C:0.983333	T:0.0166667
chr28	11305037	2	308	A:0.00649351	C:0.993506

chr28	11305299	2	302	T:0.970199	G:0.0298013	
chr28	11306387	2	302	T:0.970199	C:0.0298013	
chr28	11306726	2	296	C:0.682432	CT:0.317568	
chr28	11306894	2	290	G:0.982759	A:0.0172414	
chr28	11307128	2	300	GT:0.936667	G:0.0633333	
chr28	11309258	2	302	C:0.976821	T:0.0231788	
chr28	11310143	2	286	T:0.996503	A:0.0034965	
chr28	11310285	2	292	G:0.90411	A:0.0958904	
chr28	11311019	2	304	A:0.970395	G:0.0296053	
chr28	11311182	2	310	GTTTTT:0.0354839	G:0.964516	
chr28	11311220	2	278	T:0.0755396	C:0.92446	
chr28	11312459	3	288	TA:0.5625	T:0.0833333	TAA:0.354167
chr28	11312919	2	302	A:0.0298013	G:0.970199	
chr28	11313232	2	302	C:0.950331	A:0.0496689	
chr28	11313614	2	312	T:0.961538	C:0.0384615	
chr28	11315504	2	312	C:0.192308	G:0.807692	
chr28	11315778	2	310	T:0.932258	C:0.0677419	
chr28	11315830	2	304	C:0.976974	A:0.0230263	
chr28	11315919	2	296	CG:0.969595	C:0.0304054	
chr28	11315973	2	294	CAG:0.979592	C:0.0204082	
chr28	11316005	2	292	C:0.972603	T:0.0273973	
chr28	11316162	2	302	A:0.794702	C:0.205298	
chr28	11316269	2	284	A:0.971831	G:0.028169	
chr28	11316475	2	266	A:0.424812	G:0.575188	
chr28	11316585	2	206	G:0.42233	C:0.57767	
chr28	11316604	2	192	G:0.578125	A:0.421875	
chr28	11316863	2	242	G:0.954545	A:0.0454545	
chr28	11317055	2	290	T:0.762069	C:0.237931	
chr28	11317197	2	262	GGTCC:0.736641	G:0.263359	
chr28	11317201	2	266	CG:0.796992	C:0.203008	
chr28	11317203	2	266	TCCG:0.796992	T:0.203008	
chr28	11317231	3	302	GGTGGGCT:0.854305	G:0.112583	GGTGGGCTGTGGGCT:0.0331126
chr28	11317313	2	306	A:0.803922	G:0.196078	
chr28	11317402	2	296	T:0.618243	A:0.381757	
chr28	11317421	2	300	T:0.8	A:0.2	
chr28	11317602	2	306	C:0.977124	T:0.0228758	
chr28	11317604	2	306	T:0.996732	TC:0.00326797	
chr28	11317638	2	306	TA:0.830065	T:0.169935	
chr28	11317642	2	306	A:0.316993	T:0.683007	
chr28	11317686	2	278	A:0.147482	AAG:0.852518	
chr28	11318037	2	272	A:0.904412	G:0.0955882	
chr28	11318059	2	272	C:0.904412	T:0.0955882	
chr28	11318401	2	282	C:0.815603	T:0.184397	
chr28	11318755	2	294	C:0.945578	T:0.0544218	
chr28	11318974	2	204	C:0.553922	CT:0.446078	
chr28	11319198	2	290	A:0.57931	T:0.42069	
chr28	11319289	2	304	A:0.953947	T:0.0460526	
chr28	11319292	2	306	T:0.960784	A:0.0392157	
chr28	11319395	2	274	A:0.116788	G:0.883212	
chr28	11319487	2	306	C:0.993464	G:0.00653595	
chr28	11319777	2	306	G:0.986928	A:0.0130719	
chr28	11320022	3	310	CTATATATATATATATATA:0.203226		

C:0.280645	CTATATATATA:0.516129
chr28	11320295	2	296	A:0.996622	AT:0.00337838
chr28	11320463	2	304	G:0.996711	A:0.00328947
chr28	11320692	2	312	A:0.919872	AT:0.0801282
chr28	11321220	3	304	CT:0.733553	C:0.0789474
CTT:0.1875
chr28	11321302	2	306	T:0.120915	G:0.879085
chr28	11321615	2	300	A:0.956667	AT:0.0433333
chr28	11321688	2	298	G:0.932886	C:0.0671141
chr28	11321848	2	308	G:0.993506	A:0.00649351
chr28	11321885	2	308	G:0.925325	A:0.0746753
chr28	11321925	2	308	A:0.116883	G:0.883117
chr28	11322298	2	306	G:0.424837	A:0.575163
chr28	11322475	2	308	T:0.938312	A:0.0616883
chr28	11322736	2	304	T:0.993421	G:0.00657895
chr28	11323142	2	306	G:0.954248	A:0.0457516
chr28	11323350	2	310	G:0.990323	C:0.00967742
chr28	11323370	2	300	TGAG:0.933333	T:0.0666667
chr28	11324084	2	310	A:0.932258	T:0.0677419
chr28	11324431	3	306	GT:0.908497	G:0.0686275
GTTTT:0.0228758
chr28	11324732	2	304	T:0.993421	C:0.00657895
chr28	11325054	2	306	T:0.993464	C:0.00653595
chr28	11325291	2	306	C:0.937908	G:0.0620915
chr28	11325305	2	308	T:0.931818	G:0.0681818
chr28	11325854	2	312	A:0.413462	G:0.586538
chr28	11326114	3	320	A:0.48125
ACAGACATATCATTTTTTTTTTTTTTTAATTTTTTTTTTT:0.271875
ACAGACATATCATTTTTTTTTTTTTTTTAATTTTTTTTTTT:0.246875
chr28	11326177	2	300	A:0.95	T:0.05
chr28	11326221	2	298	T:0.993289	G:0.00671141
chr28	11326512	2	302	A:0.993377	T:0.00662252
chr28	11326707	2	298	C:0.939597	T:0.0604027
chr28	11326756	2	300	A:0.993333	C:0.00666667
chr28	11326942	2	308	A:0.967532	G:0.0324675
chr28	11327080	2	304	G:0.993421	A:0.00657895
chr28	11327354	2	302	C:0.119205	G:0.880795
chr28	11327564	2	298	T:0.83557	G:0.16443
chr28	11327848	2	288	T:0.940972	C:0.0590278
chr28	11327995	2	296	T:0.442568	A:0.557432
chr28	11328012	2	306	T:0.918301	A:0.0816993
chr28	11328494	2	236	A:0.40678	T:0.59322
chr28	11328539	2	218	C:0.394495	T:0.605505
chr28	11329088	3	298	AT:0.845638	A:0.127517
ATT:0.0268456
chr28	11329098	2	302	T:0.950331	TC:0.0496689
chr28	11329128	2	306	C:0.973856	CACAG:0.0261438
chr28	11329135	2	300	A:0.986667	AG:0.0133333
chr28	11329136	3	306	A:0.957516	AG:0.0163399
G:0.0261438
chr28	11329530	2	294	G:0.139456	C:0.860544
chr28	11329742	2	300	T:0.94	A:0.06
chr28	11329922	2	246	G:0.821138	GT:0.178862
chr28	11329945	2	164	G:0.567073	A:0.432927

chr28 11329971 2 234 GTGTATATATATATATATATATA:0.961538
G:0.0384615
chr28 11329973 2 286 GTATATATATA:0.912587 G:
0.0874126
chr28 11330070 2 276 T:0.931159 C:0.0688406
chr28 11330116 3 234 GTT:0.405983 G:0.132479
GT:0.461538
chr28 11330293 2 294 G:0.401361 A:0.598639
chr28 11331216 2 312 A:0.977564 G:0.0224359
chr28 11331797 2 304 T:0.421053 G:0.578947
chr28 11332333 2 312 A:0.974359 G:0.025641
chr28 11332587 2 312 A:0.141026 AAAAAC:0.858974
chr28 11332761 2 300 G:0.976667 A:0.0233333
chr28 11333661 2 312 G:0.134615 GT:0.865385
chr28 11333686 2 310 G:0.970968 T:0.0290323
chr28 11333928 2 312 C:0.371795 G:0.628205
chr28 11333938 2 314 A:0.136943 G:0.863057
chr28 11334020 2 300 G:0.99 T:0.01
chr28 11334067 2 304 C:0.924342 A:0.0756579
chr28 11334948 2 312 C:0.980769 T:0.0192308
chr28 11335240 2 308 G:0.99026 C:0.00974026
chr28 11335262 2 308 A:0.928571 T:0.0714286
chr28 11335382 2 308 A:0.948052 G:0.0519481
chr28 11336565 2 318 CT:0.86478 C:0.13522
chr28 11337174 2 310 C:0.990323 T:0.00967742
chr28 11337189 2 304 G:0.990132 A:0.00986842
chr28 11337192 2 306 T:0.111111 C:0.888889
chr28 11338940 2 312 G:0.996795 C:0.00320513
chr28 11338972 2 318 T:0.993711 C:0.00628931
chr28 11338995 2 310 G:0.970968 GT:0.0290323
chr28 11339013 2 314 T:0.977707 A:0.022293
chr28 11339016 2 302 G:0.970199 GATTT:0.0298013
chr28 11339345 2 296 T:0.118243 C:0.881757
chr28 11339675 2 284 T:0.841549 C:0.158451
chr28 11340018 2 304 AT:0.921053 A:0.0789474
chr28 11340028 2 308 T:0.970779 A:0.0292208
chr28 11340268 2 306 C:0.836601 T:0.163399
chr28 11340368 2 288 C:0.909722 CT:0.0902778
chr28 11340584 2 308 A:0.931818 G:0.0681818
chr28 11340714 2 304 C:0.927632 T:0.0723684
chr28 11340837 2 308 G:0 GT:1
chr28 11340842 2 308 G:0 GT:1
chr28 11341080 2 306 A:0.990196 C:0.00980392
chr28 11342374 2 302 A:0.60596 G:0.39404
chr28 11342516 2 308 T:0.977273 C:0.0227273
chr28 11342528 2 310 C:0.129032 CCTCT:0.870968
chr28 11342901 2 298 A:0.120805 G:0.879195
chr28 11342943 2 264 CTTT:0.534091 C:0.465909
chr28 11343133 2 302 G:0.990066 T:0.00993377
chr28 11343752 3 314 TTTTG:0.242038 T:0.627389
TTTTGTTTG:0.130573
chr28 11343794 2 304 G:0.957237 A:0.0427632
chr28 11345173 2 284 C:0.876761 CT:0.123239
chr28 11345441 2 310 G:0.980645 A:0.0193548

chr28 11345575 2 296 T:0.972973 A:0.027027
chr28 11345772 2 312 A:0.987179 G:0.0128205
chr28 11346071 3 294 AT:0.751701 A:0.0816327
ATT:0.166667
chr28 11346075 2 302 T:0.953642 A:0.0463576
chr28 11346082 2 298 T:0.97651 A:0.0234899
chr28 11346252 2 306 G:0 A:1
chr28 11346276 2 306 G:0 A:1
chr28 11346283 2 306 T:0 A:1
chr28 11346284 2 306 G:0 A:1
chr28 11346293 2 306 T:0 A:1
chr28 11346369 2 308 CA:0 C:1
chr28 11346741 2 316 G:0.981013 A:0.0189873
chr28 11347334 2 302 G:0.97351 GT:0.0264901
chr28 11347473 2 308 G:0.983766 C:0.0162338
chr28 11347805 2 306 G:0.990196 T:0.00980392
chr28 11348225 2 302 GT:0.89404 G:0.10596
chr28 11348241 2 306 G:0.993464 A:0.00653595
chr28 11348405 2 302 T:0.139073 TTC:0.860927
chr28 11348569 2 304 CAG:0.963816 C:0.0361842
chr28 11349440 2 312 A:0.99359 G:0.00641026
chr28 11349578 2 308 G:0.980519 A:0.0194805
chr28 11350096 2 306 T:0.924837 A:0.0751634
chr28 11350268 2 308 T:0.993506 TTGTG:0.00649351
chr28 11350327 2 300 G:0.976667 GT:0.0233333
chr28 11350916 2 304 A:0.414474 G:0.585526
chr28 11351877 2 312 G:0.983974 C:0.0160256
chr28 11352296 2 296 C:0.993243 T:0.00675676
chr28 11352433 4 306 CTT:0.686275 C:0.0228758
CTTT:0.179739 CTTTT:0.111111
chr28 11353222 2 300 T:0.993333 C:0.00666667
chr28 11353570 2 306 T:0.124183 C:0.875817
chr28 11353951 2 292 A:0.941781 AG:0.0582192
chr28 11353954 3 292 G:0.215753 GT:0.760274
GTT:0.0239726
chr28 11354007 3 240 TGTGTGGGGGGGG:0.7375 T:
0.0333333 TGG:0.229167
chr28 11354009 2 252 TGTGGGGGGG:0.809524 T:
0.190476
chr28 11354011 2 268 TGGGGGGG:0.779851 T:
0.220149
chr28 11354189 2 296 T:0.969595 C:0.0304054
chr28 11355172 2 310 C:0.948387 A:0.0516129
chr28 11355407 3 310 CT:0.254839 C:0.590323
CTT:0.154839
chr28 11356045 2 308 G:0.983766 A:0.0162338
chr28 11356881 3 304 GAA:0.233553 G:0.128289
GA:0.638158
chr28 11358289 3 292 TA:0.308219 T:0.613014
TAA:0.0787671
chr28 11358300 2 308 A:0.993506 C:0.00649351
chr28 11358751 2 306 T:0.816993 A:0.183007
chr28 11358783 2 312 T:0.88141 C:0.11859
chr28 11358785 2 312 C:0.99359 T:0.00641026

chr28	11358995	2	314	A:0.996815	T:0.00318471	
chr28	11359571	2	316	T:0.924051	A:0.0759494	
chr28	11359911	3	292	CT:0.640411	C:0.273973	CTT:0.0856164
chr28	11360031	2	300	A:0.966667	G:0.0333333	
chr28	11360684	2	302	C:0.00331126	T:0.996689	
chr28	11360703	2	306	TTGAC:0.977124	T:0.0228758	
chr28	11360990	2	308	G:0.99026	C:0.00974026	
chr28	11361323	2	306	C:0.98366	T:0.0163399	
chr28	11361862	2	306	A:0.986928	T:0.0130719	
chr28	11361867	2	310	A:0.996774	G:0.00322581	
chr28	11362406	2	302	A:0.983444	G:0.0165563	
chr28	11362523	2	304	A:0.983553	G:0.0164474	
chr28	11362701	2	312	T:0.977564	C:0.0224359	
chr28	11362897	2	310	A:0.416129	G:0.583871	
chr28	11362904	2	310	CAT:0.980645	C:0.0193548	
chr28	11363289	2	308	T:0.974026	C:0.025974	
chr28	11363460	2	300	CT:0.826667	C:0.173333	
chr28	11363642	2	308	T:0.146104	C:0.853896	
chr28	11363936	2	306	A:0.95098	C:0.0490196	
chr28	11363960	2	302	G:0.983444	A:0.0165563	
chr28	11364022	2	310	T:0.993548	C:0.00645161	
chr28	11364054	2	312	TAAA:0.99359	T:0.00641026	
chr28	11364938	2	302	C:0.996689	T:0.00331126	
chr28	11365347	2	314	T:0.977707	C:0.022293	
chr28	11366535	2	316	A:0.825949	T:0.174051	
chr28	11367224	2	298	A:0.993289	G:0.00671141	
chr28	11367266	2	312	G:0.99359	A:0.00641026	
chr28	11367613	2	306	G:0.140523	A:0.859477	
chr28	11368188	2	306	G:0.98366	C:0.0163399	
chr28	11368883	2	308	G:0.983766	A:0.0162338	
chr28	11368930	2	312	G:0.977564	A:0.0224359	
chr28	11369634	2	308	C:0.983766	T:0.0162338	
chr28	11369698	2	312	A:0.942308	G:0.0576923	
chr28	11369707	2	314	C:0.990446	T:0.00955414	
chr28	11369865	2	308	C:0.993506	A:0.00649351	
chr28	11369931	2	300	T:0.133333	TAC:0.866667	
chr28	11370083	2	314	G:0.426752	T:0.573248	
chr28	11370174	2	290	C:0.417241	T:0.582759	
chr28	11370406	2	288	G:0.986111	C:0.0138889	
chr28	11370827	2	298	TTC:0.778523	T:0.221477	
chr28	11370879	3	280	CA:0.310714	C:0.203571	CAA:0.485714
chr28	11371043	2	304	T:0.825658	A:0.174342	
chr28	11372112	2	310	T:0.996774	A:0.00322581	
chr28	11372115	2	310	A:0.983871	AT:0.016129	
chr28	11372357	2	310	G:0.983871	A:0.016129	
chr28	11372999	2	304	G:0.976974	A:0.0230263	
chr28	11373109	2	296	A:0.993243	G:0.00675676	
chr28	11373317	2	290	A:0.82069	AT:0.17931	
chr28	11373539	2	300	G:0.99	A:0.01	
chr28	11373568	2	308	G:0.993506	A:0.00649351	
chr28	11374137	2	302	C:0.817881	T:0.182119	
chr28	11374318	2	304	G:0.983553	A:0.0164474	

chr28 11375016 2 314 T:0.111465 A:0.888535
chr28 11375064 2 306 T:0.993464 C:0.00653595
chr28 11375204 2 306 C:0.781046 CT:0.218954
chr28 11375257 2 310 CAG:0.945161 C:0.0548387
chr28 11376138 2 300 G:0.993333 A:0.00666667
chr28 11376448 2 298 A:0.979866 T:0.0201342
chr28 11376449 2 294 C:0.785714 T:0.214286
chr28 11376645 2 310 C:0.990323 T:0.00967742
chr28 11377176 2 304 C:0.138158 T:0.861842
chr28 11377785 2 302 GA:0.986755 G:0.013245
chr28 11377847 2 300 T:0.146667 C:0.853333
chr28 11377911 2 310 G:0.980645 A:0.0193548
chr28 11377940 2 310 GAT:0.980645 G:0.0193548
chr28 11378893 2 248 GT:0.354839 G:0.645161
chr28 11379024 2 304 C:0.861842 T:0.138158
chr28 11380042 3 320 G:0.85
GCCAAATAGTCTTTTTATTTTTTTTTTTTATTTTTTTATTTTTTTATTTTTTT:0.109375
GCCAAATAGTCTTTTTATTTTTTTTTTTTATTTTTTTATTTTTTTTTTTTTTT:0.040625
chr28 11380061 2 310 C:0.996774 T:0.00322581
chr28 11380180 2 306 C:0.816993 CT:0.183007
chr28 11380262 2 304 A:0.578947 G:0.421053
chr28 11380346 2 304 TAA:0.819079 T:0.180921
chr28 11380606 2 316 G:0.987342 GTTTAGTAACA:
0.0126582
chr28 11380786 2 290 TG:0.962069 T:0.037931
chr28 11381145 3 290 A:0.189655 ATT:0.293103
ATTAT:0.517241
chr28 11381146 2 290 A:0.965517 T:0.0344828
chr28 11381457 3 310 CTTAT:0.758065 C:0.177419
CTTATTTAT:0.0645161
chr28 11381771 2 312 C:0.115385 T:0.884615
chr28 11381789 2 296 G:0.993243 A:0.00675676
chr28 11381828 2 304 C:0.980263 A:0.0197368
chr28 11382448 2 308 G:0.980519 A:0.0194805
chr28 11382674 2 304 C:0.148026 T:0.851974
chr28 11382703 2 304 G:0.980263 A:0.0197368
chr28 11382784 2 288 C:0.788194 CT:0.211806
chr28 11382796 2 292 C:0.756849 CT:0.243151
chr28 11382890 2 292 A:0.993151 G:0.00684932
chr28 11382915 2 292 CG:0.424658 C:0.575342
chr28 11382929 2 294 C:0.578231 T:0.421769
chr28 11383210 2 294 C:0.979592 T:0.0204082
chr28 11384031 2 292 C:0.979452 A:0.0205479
chr28 11384336 2 294 T:0.982993 C:0.0170068
chr28 11384435 3 312 GTTTTTTTTTTTTT:0.612179 G:
0.176282 GTT:0.211538
chr28 11384574 2 298 G:0.979866 A:0.0201342
chr28 11384657 2 290 C:0.989655 A:0.0103448
chr28 11385087 2 306 C:0.137255 T:0.862745
chr28 11385266 2 312 C:0.99359 T:0.00641026
chr28 11385325 2 310 G:0.980645 A:0.0193548
chr28 11385610 2 302 C:0.145695 T:0.854305
chr28 11385832 2 296 CA:0.135135 C:0.864865
chr28 11385878 2 308 G:0.996753 A:0.00324675

chr28	11386115	2	296	C:0.989865	T:0.0101351	
chr28	11386490	2	298	T:0.422819	A:0.577181	
chr28	11386521	2	308	T:0.993506	G:0.00649351	
chr28	11386861	3	292	TAA:0.400685	T:0.14726	TA:0.452055
chr28	11387684	2	306	T:0.970588	G:0.0294118	
chr28	11387819	2	312	ATGATTTTAAT:0.948718	A: 0.0512821	
chr28	11388558	2	276	TA:0.710145	T:0.289855	
chr28	11388762	2	300	G:0.403333	A:0.596667	
chr28	11388982	2	302	A:0.94702	G:0.0529801	
chr28	11389436	2	300	C:0.993333	CT:0.00666667	
chr28	11389806	2	310	C:0.954839	T:0.0451613	
chr28	11390211	2	304	G:0.963816	A:0.0361842	
chr28	11390298	2	304	G:0.128289	A:0.871711	
chr28	11390661	2	312	CTT:0.400641	C:0.599359	
chr28	11390684	2	312	C:0.996795	A:0.00320513	
chr28	11391337	2	302	G:0.993377	A:0.00662252	
chr28	11392149	2	308	G:0.996753	A:0.00324675	
chr28	11392155	2	308	C:0.996753	T:0.00324675	
chr28	11392243	2	314	GTGAAAT:0.980892	G:0.0191083	
chr28	11392462	2	312	GAC:0.977564	G:0.0224359	
chr28	11393180	2	296	G:0.25	GT:0.75	
chr28	11393771	2	308	G:0.87987	GAGAGAGAGAGAGAGAGAGAGAA:0.12013	
chr28	11393782	2	246	A:0.609756	AG:0.390244	
chr28	11393783	3	308	A:0.548701	AG:0.318182	AGAGAGAGAAAGAG:0.133117
chr28	11393791	2	276	A:0.876812	AAGAG:0.123188	
chr28	11393795	2	288	GAGAA:0.725694	G:0.274306	
chr28	11393845	2	310	G:0.790323	GAA:0.209677	
chr28	11393846	2	298	AG:0.305369	A:0.694631	
chr28	11393849	2	306	A:0.95098	AAG:0.0490196	
chr28	11393850	3	312	A:0.266026	G:0.673077	AG:0.0608974
chr28	11393870	2	312	GA:0.939103	G:0.0608974	
chr28	11393942	2	270	T:0.766667	TTA:0.233333	
chr28	11393957	2	300	TA:0.936667	T:0.0633333	
chr28	11393959	2	312	T:0.913462	TATA:0.0865385	
chr28	11394662	2	298	G:0.986577	A:0.0134228	
chr28	11395143	2	304	A:0.993421	T:0.00657895	
chr28	11395453	2	308	C:0.993506	T:0.00649351	
chr28	11395604	3	290	CTT:0.765517	C:0.0586207	TTT:0.175862
chr28	11395984	2	290	C:0.996552	T:0.00344828	
chr28	11396231	2	308	G:0.116883	T:0.883117	
chr28	11396469	2	300	C:0.403333	T:0.596667	
chr28	11396726	2	312	T:0.99359	C:0.00641026	
chr28	11396729	2	312	A:0.99359	G:0.00641026	
chr28	11396817	2	298	C:0.993289	T:0.00671141	
chr28	11396858	2	304	C:0.993421	A:0.00657895	
chr28	11397076	2	308	G:0.987013	A:0.012987	
chr28	11397620	2	298	GTT:0.147651	G:0.852349	
chr28	11397684	2	300	AAGAG:0.4	A:0.6	

chr28	11397748	2	284	A:0.126761	G:0.873239
chr28	11397771	2	288	C:0.930556	T:0.0694444
chr28	11398479	2	302	T:0.384106	C:0.615894
chr28	11398842	2	294	G:0.97619	A:0.0238095
chr28	11399261	2	298	T:0.989933	C:0.0100671
chr28	11399472	2	292	C:0.989726	T:0.010274
chr28	11400792	2	296	A:0.141892	T:0.858108
chr28	11401721	2	314	G:0.410828	A:0.589172
chr28	11401844	2	306	G:0.973856	A:0.0261438
chr28	11401962	2	308	G:0.980519	A:0.0194805
chr28	11402177	2	306	C:0.784314	A:0.215686
chr28	11402263	2	300	C:0.993333	T:0.00666667
chr28	11402468	2	298	G:0.966443	T:0.033557
chr28	11402469	2	298	A:0.966443	T:0.033557
chr28	11402596	2	286	T:0.972028	C:0.027972
chr28	11402600	3	316	CCTCTCTCTCT:0.322785	C:
0.303797	CCTCTCTCTCTCTCT:0.373418				
chr28	11402645	2	242	TA:0.475207	T:0.524793
chr28	11402796	2	300	C:0.843333	T:0.156667
chr28	11403468	2	306	C:0.820261	T:0.179739
chr28	11403533	2	312	A:0.99359	G:0.00641026
chr28	11403536	2	310	T:0.819355	C:0.180645
chr28	11404631	2	288	T:0.142361	C:0.857639
chr28	11404670	2	308	A:0.831169	C:0.168831
chr28	11404882	2	298	G:0.114094	C:0.885906
chr28	11406064	2	298	C:0.83557	T:0.16443
chr28	11406676	2	294	T:0.959184	C:0.0408163
chr28	11406697	2	300	T:0.396667	A:0.603333
chr28	11406738	2	292	AT:0.157534	A:0.842466
chr28	11406755	2	300	G:0.41 A:0.59	
chr28	11406933	2	280	G:0.964286	A:0.0357143
chr28	11406993	2	296	T:0.152027	C:0.847973
chr28	11407521	2	294	G:0.146259	GT:0.853741
chr28	11407834	2	290	G:0.993103	A:0.00689655
chr28	11409745	2	294	T:0.302721	C:0.697279
chr28	11410506	2	284	T:0.954225	C:0.0457746
chr28	11410817	2	290	G:0.955172	C:0.0448276
chr28	11410823	2	292	AT:0.277397	A:0.722603
chr28	11411325	2	280	G:0.864286	A:0.135714
chr28	11411580	2	300	C:0.486667	A:0.513333
chr28	11411684	2	290	G:0.351724	A:0.648276
chr28	11411741	2	298	T:0.298658	C:0.701342
chr28	11411795	2	292	A:0.989726	G:0.010274
chr28	11411861	2	294	T:0.357143	C:0.642857
chr28	11411938	2	306	C:0.866013	T:0.133987
chr28	11411995	2	304	CCCA:0.332237	C:0.667763
chr28	11412144	2	296	A:0.344595	C:0.655405
chr28	11412181	2	308	T:0.344156	G:0.655844
chr28	11412203	2	308	C:0.344156	T:0.655844
chr28	11412265	2	312	T:0.987179	C:0.0128205
chr28	11412370	2	304	C:0.519737	T:0.480263
chr28	11412429	2	294	G:0.97619	A:0.0238095
chr28	11412463	2	296	A:0.131757	G:0.868243
chr28	11412904	2	298	C:0.52349	T:0.47651

chr28	11412963	2	292	A:0.315068	C:0.684932
chr28	11413178	2	296	A:0.331081	G:0.668919
chr28	11413588	2	294	T:0.343537	C:0.656463
chr28	11413779	2	296	G:0.327703	C:0.672297
chr28	11415240	2	298	C:0.872483	T:0.127517
chr28	11415441	2	296	G:0.949324	GC:0.0506757
chr28	11415479	2	302	G:0.990066	A:0.00993377
chr28	11415635	2	294	C:0.989796	A:0.0102041
chr28	11415710	2	298	C:0.295302	T:0.704698
chr28	11416137	2	306	T:0.30719	G:0.69281
chr28	11416184	2	302	C:0.927152	T:0.0728477
chr28	11416385	2	300	C:0.306667	T:0.693333
chr28	11416393	2	300	A:0.306667	G:0.693333
chr28	11416421	2	300	C:0.306667	T:0.693333
chr28	11416931	2	298	G:0.862416	A:0.137584
chr28	11417125	2	294	C:0.309524	A:0.690476
chr28	11417144	2	296	C:0.317568	G:0.682432
chr28	11417358	2	294	G:0.319728	A:0.680272
chr28	11417380	2	298	T:0.305369	C:0.694631
chr28	11417401	2	294	G:0.319728	T:0.680272
chr28	11417573	2	292	C:0.934932	T:0.0650685
chr28	11417577	2	292	A:0.308219	G:0.691781
chr28	11417769	2	302	C:0.976821	T:0.0231788
chr28	11418418	2	302	C:0.937086	CTTA:0.0629139
chr28	11418658	2	304	G:0.973684	A:0.0263158
chr28	11418862	2	262	C:0.305344	T:0.694656
chr28	11419163	2	218	G:0.261468	A:0.738532
chr28	11419363	2	178	A:0.578652	T:0.421348
chr28	11420115	2	218	C:0.155963	G:0.844037
chr28	11420140	2	222	G:0.761261	A:0.238739
chr28	11420191	2	190	A:0.431579	T:0.568421
chr28	11420278	2	198	G:0.843434	A:0.156566
chr28	11420342	2	200	G:0.905 GC:0.095	
chr28	11420347	2	200	G:0.25 GC:0.75	
chr28	11420354	2	200	C:0.25 T:0.75	
chr28	11420399	2	208	AC:0.153846	A:0.846154
chr28	11420410	2	208	C:0.153846	T:0.846154
chr28	11420543	2	220	C:0.154545	G:0.845455
chr28	11420689	2	244	T:0.840164	A:0.159836
chr28	11421086	2	252	C:0.845238	T:0.154762
chr28	11421306	2	262	C:0.133588	G:0.866412
chr28	11421383	2	290	C:0.365517	T:0.634483
chr28	11421567	2	296	T:0.476351	C:0.523649
chr28	11421735	2	296	C:0.138514	A:0.861486
chr28	11421942	2	306	T:0.143791	C:0.856209
chr28	11422206	2	296	G:0.111486	A:0.888514
chr28	11422385	2	298	CT:0.97651	C:0.0234899
chr28	11422386	2	298	T:0.187919	C:0.812081
chr28	11422387	2	298	G:0.97651	C:0.0234899
chr28	11422461	2	290	G:0.486207	A:0.513793
chr28	11422483	2	292	G:0.688356	A:0.311644
chr28	11422662	2	302	G:0.966887	C:0.0331126
chr28	11422677	2	300	C:0.79 T:0.21	
chr28	11422834	2	304	C:0.970395	G:0.0296053

chr28	11423120	2	288	G:0.145833	A:0.854167	
chr28	11423200	2	286	G:0.496503	A:0.503497	
chr28	11423349	2	288	G:0.489583	A:0.510417	
chr28	11423590	2	298	G:0.147651	A:0.852349	
chr28	11424170	2	300	C:0.136667	CT:0.863333	
chr28	11424205	2	294	C:0.863946	T:0.136054	
chr28	11424287	2	284	T:0.978873	TG:0.0211268	
chr28	11424305	2	280	C:0.489286	T:0.510714	
chr28	11424429	3	282	ATTTTTTT:0.684397	A:0.156028	ATTTT:0.159574
chr28	11424609	2	274	C:0.879562	G:0.120438	
chr28	11424683	2	262	T:0.866412	TTGAAC:0.133588	
chr28	11424708	2	266	C:0.894737	T:0.105263	
chr28	11424721	2	274	T:0.489051	C:0.510949	
chr28	11424818	2	258	C:0.786822	T:0.213178	
chr28	11424884	2	228	C:0.868421	T:0.131579	
chr28	11425255	2	298	C:0.879195	T:0.120805	
chr28	11425269	2	296	T:0.297297	C:0.702703	
chr28	11425339	2	310	G:0.880645	C:0.119355	
chr28	11425346	2	308	G:0.866883	A:0.133117	
chr28	11425922	2	298	G:0.879195	T:0.120805	
chr28	11425958	2	296	A:0.888514	C:0.111486	
chr28	11426016	2	292	A:0.863014	AT:0.136986	
chr28	11426159	2	310	A:0.880645	G:0.119355	
chr28	11426266	2	300	G:0.763333	A:0.236667	
chr28	11426282	2	304	T:0.898026	C:0.101974	
chr28	11426438	2	288	T:0.881944	A:0.118056	
chr28	11426621	2	302	A:0.728477	G:0.271523	
chr28	11426737	2	302	A:0.89404	C:0.10596	
chr28	11426791	2	296	T:0.888514	C:0.111486	
chr28	11427406	2	310	A:0.816129	C:0.183871	
chr28	11427446	2	304	G:0.805921	A:0.194079	
chr28	11427512	2	304	T:0.993421	G:0.00657895	
chr28	11427715	2	298	C:0.986577	T:0.0134228	
chr28	11427820	2	310	G:0.864516	T:0.135484	
chr28	11427838	2	306	T:0.869281	C:0.130719	
chr28	11427908	2	294	C:0.935374	A:0.0646259	
chr28	11428001	2	292	G:0.890411	C:0.109589	
chr28	11428014	2	298	C:0.889262	T:0.110738	
chr28	11428071	2	300	T:0.81	C:0.19	
chr28	11428105	2	296	T:0.881757	G:0.118243	
chr28	11428263	2	294	A:0.880952	G:0.119048	
chr28	11428386	2	308	A:0.925325	G:0.0746753	
chr28	11428887	2	310	T:0.974194	C:0.0258065	
chr28	11428889	2	310	A:0.887097	G:0.112903	
chr28	11428965	2	310	A:0.770968	C:0.229032	
chr28	11429020	2	308	A:0.987013	G:0.012987	
chr28	11429207	2	310	GTT:0.974194	G:0.0258065	
chr28	11429220	2	312	C:0.894231	T:0.105769	
chr28	11429733	2	298	A:0.379195	T:0.620805	
chr28	11429844	2	300	G:0.896667	A:0.103333	
chr28	11429874	2	300	A:0.766667	G:0.233333	
chr28	11429887	2	300	C:0.886667	CTT:0.113333	
chr28	11430082	2	298	A:0.744966	T:0.255034	

chr28	11430108	2	302	C:0.738411	T:0.261589
chr28	11430770	2	304	A:0.960526	C:0.0394737
chr28	11431115	2	312	T:0.891026	C:0.108974
chr28	11431529	2	304	G:0.858553	C:0.141447
chr28	11431632	2	304	A:0.901316	G:0.0986842
chr28	11431899	2	310	G:0.919355	A:0.0806452
chr28	11432698	2	300	T:0.973333	A:0.0266667
chr28	11432793	2	302	C:0.370861	T:0.629139
chr28	11432958	2	298	TA:0.932886	T:0.0671141
chr28	11433004	2	306	C:0.872549	T:0.127451
chr28	11433034	2	304	C:0.996711	G:0.00328947
chr28	11433414	2	312	T:0.894231	TAAACAA:0.105769
chr28	11434096	2	294	CTTTT:0.85034	C:0.14966
chr28	11434131	2	294	T:0.853741	C:0.146259
chr28	11434149	2	292	G:0.863014	A:0.136986
chr28	11434291	2	300	A:0.87	C:0.13
chr28	11434368	2	306	G:0.398693	A:0.601307
chr28	11434415	2	304	G:0.884868	A:0.115132
chr28	11434527	2	292	A:0.873288	G:0.126712
chr28	11434650	2	272	T:0.856618	TGTG:0.143382
chr28	11435664	2	298	G:0.899329	A:0.100671
chr28	11435675	2	296	G:0.902027	T:0.097973
chr28	11435691	2	296	C:0.996622	T:0.00337838
chr28	11435692	2	296	G:0.996622	T:0.00337838
chr28	11435780	2	296	G:0.878378	C:0.121622
chr28	11436332	2	300	G:0.876667	A:0.123333
chr28	11436376	2	292	A:0.123288	G:0.876712
chr28	11436526	5	302	GGA:0.274834	G:0.125828
GGAGA:0.175497		GGAGAGA:0.172185		GGAGAGAGA:0.251656	
chr28	11436632	2	294	C:0.863946	T:0.136054
chr28	11436634	2	294	G:0.87415	A:0.12585
chr28	11436761	2	296	C:0.861486	T:0.138514
chr28	11436807	2	286	T:0.863636	C:0.136364
chr28	11436968	2	304	G:0.980263	A:0.0197368
chr28	11437489	2	298	A:0.956376	C:0.0436242
chr28	11437610	2	296	T:0.125	C:0.875
chr28	11437708	2	306	C:0.392157	A:0.607843
chr28	11438036	2	298	C:0.865772	T:0.134228
chr28	11438150	2	296	CT:0.128378	C:0.871622
chr28	11438221	2	298	T:0.761745	C:0.238255
chr28	11438900	2	300	G:0.446667	A:0.553333
chr28	11439207	2	296	C:0.47973	T:0.52027
chr28	11439218	2	294	T:0.370748	C:0.629252
chr28	11439221	2	296	G:0.891892	T:0.108108
chr28	11439498	2	296	C:0.462838	T:0.537162
chr28	11439553	2	306	C:0.977124	T:0.0228758
chr28	11439861	2	302	A:0.731788	C:0.268212
chr28	11439912	2	298	G:0.741611	A:0.258389
chr28	11440127	2	298	C:0.979866	T:0.0201342
chr28	11440362	2	294	C:0.894558	T:0.105442
chr28	11440596	2	290	A:0.975862	T:0.0241379
chr28	11440624	2	296	TG:0.243243	T:0.756757
chr28	11440955	2	290	C:0.517241	A:0.482759
chr28	11440979	2	296	C:0.516892	T:0.483108

chr28	11441034	2	280	C:0.782143	A:0.217857	
chr28	11441221	2	296	C:0.807432	T:0.192568	
chr28	11441237	2	290	T:0.493103	C:0.506897	
chr28	11441467	2	304	C:0.815789	T:0.184211	
chr28	11441654	2	296	C:0.260135	T:0.739865	
chr28	11441685	2	294	T:0.510204	G:0.489796	
chr28	11441756	2	290	A:0.268966	G:0.731034	
chr28	11441841	2	296	T:0.263514	C:0.736486	
chr28	11441881	2	298	G:0.255034	A:0.744966	
chr28	11441889	2	298	T:0.255034	C:0.744966	
chr28	11442035	2	312	A:0.285256	C:0.714744	
chr28	11442388	2	298	G:0.291946	C:0.708054	
chr28	11442453	2	304	T:0.996711	A:0.00328947	
chr28	11443094	2	302	A:0.235099	G:0.764901	
chr28	11443106	2	300	G:0.496667	A:0.503333	
chr28	11443191	2	314	G:0.248408	A:0.751592	
chr28	11443231	2	310	A:0.23871	T:0.76129	
chr28	11443345	2	304	T:0.483553	C:0.516447	
chr28	11443407	2	298	C:0.463087	A:0.536913	
chr28	11443603	2	280	CTATCT:0.928571	C:0.0714286	
chr28	11443605	2	304	ATCTAATCT:0.884868	A:0.115132	
chr28	11443607	2	298	CT:0.64094	C:0.35906	
chr28	11443608	2	310	TA:0.73871	T:0.26129	
chr28	11443609	2	308	AATCT:0.935065	A:0.0649351	
chr28	11443610	2	288	ATCT:0.545139	A:0.454861	
chr28	11443612	2	310	C:0.73871	CA:0.26129	
chr28	11443613	2	310	T:0.73871	A:0.26129	
chr28	11443968	2	306	T:0.490196	C:0.509804	
chr28	11444045	2	312	G:0.516026	C:0.483974	
chr28	11444217	2	298	G:0.510067	A:0.489933	
chr28	11444259	2	280	C:0.817857	T:0.182143	
chr28	11444508	3	84	TAA:0.464286	T:0.416667	TA:0.119048
chr28	11444585	2	292	C:0.541096	G:0.458904	
chr28	11444718	2	302	A:0.930464	G:0.0695364	
chr28	11445107	2	296	A:0.506757	AG:0.493243	
chr28	11445115	2	298	G:0.597315	C:0.402685	
chr28	11446124	2	302	C:0.801325	G:0.198675	
chr28	11446159	2	316	C:0.509494	T:0.490506	
chr28	11446167	2	316	G:0.509494	A:0.490506	
chr28	11446192	2	310	C:0.8	T:0.2	
chr28	11446200	2	304	G:0.506579	C:0.493421	
chr28	11446255	2	302	G:0.970199	A:0.0298013	
chr28	11446275	2	296	T:0.5	G:0.5	
chr28	11446490	2	304	T:0.292763	C:0.707237	
chr28	11446497	2	304	G:0.526316	A:0.473684	
chr28	11446963	2	300	G:0.933333	A:0.0666667	
chr28	11447015	2	288	A:0.152778	G:0.847222	
chr28	11447231	2	306	G:0.823529	A:0.176471	
chr28	11447532	2	298	G:0.47651	A:0.52349	
chr28	11447712	2	308	T:0.691558	TATCTC:0.308442	
chr28	11448227	2	314	AGCTGGTCCAT:0.770701	A:0.229299	

chr28	11448339	2	292	T:0.777397	G:0.222603
chr28	11448341	2	294	T:0.982993	C:0.0170068
chr28	11448848	2	318	CAACAGGG:0.764151	C: 0.235849
chr28	11449428	2	292	C:0.952055	T:0.0479452
chr28	11449436	2	290	AC:0.693103	A:0.306897
chr28	11449587	2	300	G:0.94	A:0.06
chr28	11449611	2	306	A:0.931373	G:0.0686275
chr28	11449847	2	294	C:0.792517	T:0.207483
chr28	11449862	2	290	A:0.806897	G:0.193103
chr28	11449948	2	308	A:0.941558	G:0.0584416
chr28	11450011	2	314	C:0.942675	A:0.0573248
chr28	11450081	2	294	T:0.520408	G:0.479592
chr28	11450420	2	306	C:0.973856	T:0.0261438
chr28	11450463	2	312	C:0.974359	T:0.025641
chr28	11450545	2	302	A:0.943709	T:0.0562914
chr28	11450739	2	310	G:0.877419	A:0.122581
chr28	11450777	2	310	T:0.358065	C:0.641935
chr28	11450789	2	310	A:0.358065	C:0.641935
chr28	11450968	2	312	G:0.371795	A:0.628205
chr28	11451049	2	296	A:0.918919	AGG:0.0810811
chr28	11451066	2	296	A:0.381757	G:0.618243
chr28	11451117	2	296	G:0.452703	C:0.547297
chr28	11451126	2	312	C:0.923077	CGACTGGTGG: 0.0769231
chr28	11451168	2	302	A:0.913907	C:0.0860927
chr28	11451413	2	240	A:0.779167	G:0.220833
chr28	11452138	2	264	T:0.969697	C:0.030303
chr28	11452230	2	252	G:0.678571	A:0.321429
chr28	11452504	2	128	AGG:0.757812	A:0.242188
chr28	11452694	2	250	G:0.736	T:0.264
chr28	11452814	2	290	T:0.734483	C:0.265517
chr28	11452912	2	294	C:0.979592	T:0.0204082
chr28	11452913	2	294	A:0.721088	G:0.278912
chr28	11452971	2	282	T:0.978723	C:0.0212766
chr28	11452972	2	286	TGTG:0.762238	T:0.237762
chr28	11452982	2	286	C:0.762238	G:0.237762
chr28	11453002	2	288	T:0.777778	C:0.222222
chr28	11453120	2	304	C:0.746711	A:0.253289
chr28	11453144	2	304	G:0.950658	A:0.0493421
chr28	11453154	2	304	T:0.809211	C:0.190789
chr28	11453161	2	300	T:0.756667	G:0.243333
chr28	11453179	2	300	T:0.756667	C:0.243333
chr28	11453221	2	296	G:0.753378	A:0.246622
chr28	11453242	2	296	A:0.753378	C:0.246622
chr28	11453243	2	296	C:0.753378	T:0.246622
chr28	11453355	2	296	C:0.773649	T:0.226351
chr28	11453388	2	292	C:0.746575	CT:0.253425
chr28	11453520	2	294	G:0.758503	A:0.241497
chr28	11453524	2	294	C:0	CT:1
chr28	11453566	2	292	G:0.777397	A:0.222603
chr28	11453615	2	302	C:0.807947	T:0.192053
chr28	11453665	2	294	AG:0.945578	A:0.0544218
chr28	11453669	2	298	G:0.822148	A:0.177852

chr28	11453727	2	298	T:0.758389	A:0.241611
chr28	11453737	2	298	T:0.758389	C:0.241611
chr28	11453738	2	298	A:0.758389	T:0.241611
chr28	11453762	2	290	A:0.706897	G:0.293103
chr28	11453969	2	302	C:0.754967	A:0.245033
chr28	11454093	2	308	GC:0.753247	G:0.246753
chr28	11454095	2	308	AT:0.753247	A:0.246753
chr28	11454115	2	308	C:0.753247	T:0.246753
chr28	11454364	2	302	G:0.960265	A:0.0397351
chr28	11454578	2	290	GA:0.693103	G:0.306897
chr28	11454670	2	290	GA:0.968966	G:0.0310345
chr28	11454721	2	286	C:0.769231	T:0.230769
chr28	11454767	2	290	C:0.97931	G:0.0206897
chr28	11454777	2	284	GT:0.947183	G:0.0528169
chr28	11454794	2	302	G:0.97351	A:0.0264901
chr28	11454897	2	304	C:0.976974	T:0.0230263
chr28	11454945	2	298	G:0.97651	A:0.0234899
chr28	11455006	2	304	G:0.789474	A:0.210526
chr28	11455089	2	298	G:0.986577	A:0.0134228
chr28	11455146	2	312	TAGGTCAC:0.967949	T: 0.0320513
chr28	11455185	2	302	C:0.950331	A:0.0496689
chr28	11455244	2	302	G:0.778146	A:0.221854
chr28	11455431	2	306	C:0.98366	T:0.0163399
chr28	11455510	2	308	A:0.983766	G:0.0162338
chr28	11455519	2	308	T:0.983766	A:0.0162338
chr28	11455548	2	306	T:0.98366	G:0.0163399
chr28	11455648	2	306	A:0.748366	C:0.251634
chr28	11456272	2	306	T:0.702614	C:0.297386
chr28	11456386	2	312	A:0.798077	C:0.201923
chr28	11456427	2	310	C:0.787097	T:0.212903
chr28	11456677	2	306	T:0.960784	C:0.0392157
chr28	11456918	2	306	A:0.98366	G:0.0163399
chr28	11457146	2	296	A:0.983108	T:0.0168919
chr28	11457445	2	298	A:0.97651	G:0.0234899
chr28	11457551	2	300	C:0.95 T:0.05	
chr28	11457766	2	296	G:0.820946	A:0.179054
chr28	11458514	2	306	C:0.964052	T:0.0359477
chr28	11458601	2	298	G:0.963087	A:0.0369128
chr28	11458672	2	300	A:0.75 AT:0.25	
chr28	11458715	2	304	T:0.976974	C:0.0230263
chr28	11458967	2	302	T:0.956954	A:0.0430464
chr28	11459007	2	300	A:0.956667	AG:0.0433333
chr28	11459431	2	304	T:0.957237	A:0.0427632
chr28	11459445	2	300	G:0.98 A:0.02	
chr28	11459521	2	282	G:0.829787	A:0.170213
chr28	11459530	2	284	G:0.940141	A:0.0598592
chr28	11459536	2	294	G:0.979592	A:0.0204082
chr28	11459549	2	298	C:0.956376	T:0.0436242
chr28	11459552	2	296	G:0.827703	A:0.172297
chr28	11459572	2	296	G:0.827703	A:0.172297
chr28	11459585	2	294	G:0.908163	A:0.0918367
chr28	11459667	2	304	A:0.986842	G:0.0131579
chr28	11459673	2	304	AT:0.75 A:0.25	

chr28 11459680 2 302 C:0.807947 A:0.192053
chr28 11459682 2 304 A:0.75 C:0.25
chr28 11459865 3 272 TG:0.558824 T:0.3125 GG:
0.128676
chr28 11459956 2 302 C:0.983444 T:0.0165563
chr28 11460400 2 304 C:0.730263 CT:0.269737
chr28 11460725 2 302 T:0.811258 C:0.188742
chr28 11460854 2 280 C:0.807143 T:0.192857
chr28 11461325 3 278 TAA:0.586331 T:0.125899
TA:0.28777
chr28 11461353 2 294 C:0.792517 T:0.207483
chr28 11461437 2 300 A:0.953333 G:0.0466667
chr28 11461946 2 298 G:0.956376 T:0.0436242
chr28 11463041 2 306 T:0.748366 C:0.251634
chr28 11463062 2 306 A:0.748366 G:0.251634
chr28 11463108 2 298 T:0.966443 A:0.033557
chr28 11463293 2 298 CG:0.765101 C:0.234899
chr28 11463420 2 298 T:0.751678 C:0.248322
chr28 11463474 2 308 G:0.918831 T:0.0811688
chr28 11464030 2 308 A:0.769481 C:0.230519
chr28 11464210 2 308 T:0.727273 C:0.272727
chr28 11464364 2 300 A:0.14 T:0.86
chr28 11464436 2 304 C:0.973684 A:0.0263158
chr28 11465027 2 296 C:0.753378 G:0.246622
chr28 11465227 2 304 G:0.819079 T:0.180921
chr28 11465587 2 304 T:0.720395 C:0.279605
chr28 11465771 2 308 T:0.743506 G:0.256494
chr28 11466352 2 310 T:0.741935 C:0.258065
chr28 11466397 2 304 G:0.963816 A:0.0361842
chr28 11466444 2 294 A:0.676871 G:0.323129
chr28 11467469 2 294 C:0.972789 A:0.0272109
chr28 11467596 4 318 AGGATGGAT:0.556604 A:
0.261006 AGGAT:0.0314465 AGGATGGATGGAT:0.150943
chr28 11469003 2 296 G:0.986486 A:0.0135135
chr28 11469347 2 308 T:0.655844 C:0.344156
chr28 11469374 2 310 A:0.977419 T:0.0225806
chr28 11469448 2 300 G:0.966667 A:0.0333333
chr28 11469466 2 310 C:0.970968 T:0.0290323
chr28 11469490 2 300 G:0.95 A:0.05
chr28 11469498 2 308 A:0.983766 G:0.0162338
chr28 11469666 2 308 G:0.925325 C:0.0746753
chr28 11469897 2 308 G:0.974026 C:0.025974
chr28 11470230 2 304 T:0.957237 C:0.0427632
chr28 11470342 2 300 TGTCA:0.81 T:0.19
chr28 11471124 2 308 G:0.980519 C:0.0194805
chr28 11471161 2 300 G:0.953333 A:0.0466667
chr28 11471168 2 300 C:0.97 T:0.03
chr28 11471323 2 314 GATATAAATCTGCACACA:0.815287
G:0.184713
chr28 11471334 2 314 G:0.847134 A:0.152866
chr28 11471434 2 308 C:0.863636 T:0.136364
chr28 11471804 2 290 T:0.782759 G:0.217241
chr28 11471966 2 296 T:0.905405 C:0.0945946
chr28 11472208 2 304 A:0.759868 G:0.240132

chr28	11472488	2	298	T:0.748322	G:0.251678	
chr28	11472688	2	292	G:0.907534	A:0.0924658	
chr28	11472799	2	316	C:0.924051	T:0.0759494	
chr28	11472986	2	304	T:0.842105	C:0.157895	
chr28	11473001	2	298	C:0.744966	T:0.255034	
chr28	11473356	2	306	C:0.771242	G:0.228758	
chr28	11473384	2	304	G:0.990132	T:0.00986842	
chr28	11473414	2	302	A:0.847682	T:0.152318	
chr28	11473688	2	316	A:0.974684	G:0.0253165	
chr28	11474130	2	310	T:0.832258	C:0.167742	
chr28	11474197	2	304	T:0.822368	C:0.177632	
chr28	11474961	2	294	G:0.989796	A:0.0102041	
chr28	11475340	2	310	A:0.980645	G:0.0193548	
chr28	11475421	2	312	G:0.983974	C:0.0160256	
chr28	11475581	2	304	GT:0.769737	G:0.230263	
chr28	11475811	2	294	G:0.986395	A:0.0136054	
chr28	11476024	2	304	G:0.983553	A:0.0164474	
chr28	11476538	2	304	A:0.983553	G:0.0164474	
chr28	11476652	2	292	G:0.989726	A:0.010274	
chr28	11476824	2	296	G:0.983108	A:0.0168919	
chr28	11477140	2	304	G:0.986842	A:0.0131579	
chr28	11477164	2	306	G:0.96732	C:0.0326797	
chr28	11477263	2	318	C:0.981132	CATATCTT: 0.0188679	
chr28	11477418	2	306	C:0.986928	T:0.0130719	
chr28	11477480	2	316	T:0.987342	G:0.0126582	
chr28	11477587	2	306	T:0.98366	C:0.0163399	
chr28	11477592	2	306	C:0.98366	G:0.0163399	
chr28	11477687	2	312	G:0.983974	T:0.0160256	
chr28	11477721	2	304	C:0.986842	T:0.0131579	
chr28	11477917	2	300	G:0.33	C:0.67	
chr28	11477984	2	316	C:0.984177	T:0.0158228	
chr28	11478176	2	302	C:0.761589	G:0.238411	
chr28	11478211	2	298	T:0.983221	C:0.0167785	
chr28	11478307	2	298	T:0.986577	C:0.0134228	
chr28	11478461	2	292	G:0.982877	A:0.0171233	
chr28	11478477	2	296	T:0.986486	C:0.0135135	
chr28	11478481	3	296	C:0.918919	T:0.0135135	G:0.0675676
chr28	11478581	2	314	G:0.987261	C:0.0127389	
chr28	11478646	2	312	T:0.974359	C:0.025641	
chr28	11478861	2	312	C:0.987179	A:0.0128205	
chr28	11478872	2	312	A:0.987179	G:0.0128205	
chr28	11478879	2	310	T:0.774194	C:0.225806	
chr28	11479017	2	308	A:0.99026	G:0.00974026	
chr28	11480928	2	294	G:0.860544	T:0.139456	
chr28	11481038	2	302	A:0.880795	G:0.119205	
chr28	11481693	2	296	G:0.915541	T:0.0844595	
chr28	11482073	2	312	G:0.910256	A:0.0897436	
chr28	11482106	2	306	G:0.98366	GA:0.0163399	
chr28	11482887	2	302	G:0.92053	T:0.0794702	
chr28	11483039	2	308	C:0.961039	G:0.038961	
chr28	11483102	2	302	G:0.983444	GT:0.0165563	
chr28	11483166	2	308	C:0.964286	A:0.0357143	

chr28	11483215	2	296	A:0.929054	C:0.0709459	
chr28	11483322	2	306	T:0.964052	C:0.0359477	
chr28	11483461	2	304	G:0.963816	A:0.0361842	
chr28	11483593	2	306	T:0.872549	C:0.127451	
chr28	11483649	2	300	T:0.956667	C:0.0433333	
chr28	11483657	2	296	C:0.976351	T:0.0236486	
chr28	11483764	2	292	T:0.941781	C:0.0582192	
chr28	11483777	2	284	A:0.922535	G:0.0774648	
chr28	11483812	2	316	A:0.962025	AAAAGAAAAAG:0.0379747	
chr28	11483849	2	306	A:0.846405	G:0.153595	
chr28	11484111	2	298	C:0.842282	T:0.157718	
chr28	11484522	2	294	T:0.979592	C:0.0204082	
chr28	11484626	2	288	G:0.979167	C:0.0208333	
chr28	11484728	2	296	C:0.898649	T:0.101351	
chr28	11484740	2	296	C:0.959459	T:0.0405405	
chr28	11484799	2	294	C:0.982993	T:0.0170068	
chr28	11484800	2	296	G:0.966216	A:0.0337838	
chr28	11484987	2	306	G:0.957516	A:0.0424837	
chr28	11485184	2	296	T:0.962838	C:0.0371622	
chr28	11485308	2	302	G:0.986755	A:0.013245	
chr28	11485412	2	294	G:0.952381	A:0.047619	
chr28	11485563	2	300	CT:0.803333	C:0.196667	
chr28	11485583	2	308	A:0.876623	G:0.123377	
chr28	11485671	3	304	A:0.878289	C:0.0822368	T:0.0394737
chr28	11485684	2	304	C:0.960526	T:0.0394737	
chr28	11485793	2	304	C:0.953947	T:0.0460526	
chr28	11485949	2	304	G:0.980263	T:0.0197368	
chr28	11485966	2	318	T:0.886792	TGTTTTTG:0.113208	
chr28	11486019	2	294	T:0.914966	C:0.085034	
chr28	11486116	2	294	G:0.965986	A:0.0340136	
chr28	11486158	2	292	G:0.917808	A:0.0821918	
chr28	11486231	2	290	T:0.965517	C:0.0344828	
chr28	11486251	2	298	C:0.97651	G:0.0234899	
chr28	11486293	2	302	T:0.824503	TG:0.175497	
chr28	11486369	2	304	A:0.907895	T:0.0921053	
chr28	11486603	2	300	T:0.83	C:0.17	
chr28	11486821	2	304	C:0.986842	T:0.0131579	
chr28	11486830	2	300	A:0.83	G:0.17	
chr28	11486883	2	304	A:0.957237	G:0.0427632	
chr28	11487163	2	304	G:0.990132	T:0.00986842	
chr28	11487466	2	304	T:0.940789	C:0.0592105	
chr28	11487880	2	302	T:0.834437	C:0.165563	
chr28	11488166	2	304	C:0.930921	T:0.0690789	
chr28	11488286	2	304	TGC:0.934211	T:0.0657895	
chr28	11488289	2	318	G:0.921384	GCTCT:0.0786164	
chr28	11488291	2	318	G:0.902516	T:0.0974843	
chr28	11488341	2	318	A:0.77673	G:0.22327	
chr28	11488740	2	300	G:0.933333	A:0.0666667	
chr28	11488817	2	308	G:0.931818	A:0.0681818	
chr28	11488832	2	308	C:0.798701	G:0.201299	
chr28	11489578	2	286	G:0.98951	A:0.0104895	
chr28	11489756	2	258	T:0.728682	TA:0.271318	

chr28	11489966	2	304	G:0.960526	A:0.0394737
chr28	11490486	2	298	G:0.986577	A:0.0134228
chr28	11490715	2	318	A:0.90566	G:0.0943396
chr28	11490799	2	302	T:0.913907	A:0.0860927
chr28	11490936	2	312	A:0.961538	G:0.0384615
chr28	11490937	2	312	T:0.961538	C:0.0384615
chr28	11490945	2	312	G:0.961538	T:0.0384615
chr28	11491365	2	306	C:0.960784	G:0.0392157
chr28	11491994	2	300	A:0.0333333	AACT:0.966667
chr28	11492273	2	302	A:0.980132	G:0.0198675
chr28	11492868	2	250	AG:0	A:1
chr28	11493620	2	300	C:0.983333	A:0.0166667
chr28	11493665	2	282	CG:0.975177	C:0.0248227
chr28	11493738	2	284	G:0.975352	A:0.0246479
chr28	11494754	2	290	G:0.934483	A:0.0655172
chr28	11494775	2	296	AG:0.97973	A:0.0202703
chr28	11495053	2	306	T:0.980392	C:0.0196078
chr28	11495155	2	304	C:0.990132	T:0.00986842
chr28	11495639	2	302	G:0.970199	A:0.0298013
chr28	11495958	2	292	G:0.965753	A:0.0342466
chr28	11496440	2	310	C:0.964516	T:0.0354839
chr28	11496629	2	300	G:0.973333	A:0.0266667
chr28	11496751	2	294	G:0.955782	GT:0.0442177
chr28	11496843	2	302	G:0.678808	A:0.321192
chr28	11496867	2	298	C:0.963087	T:0.0369128
chr28	11497063	2	304	T:0.957237	C:0.0427632
chr28	11497560	2	298	C:0.969799	T:0.0302013
chr28	11497797	2	304	T:0.973684	A:0.0263158
chr28	11497809	2	304	TA:0.973684	T:0.0263158
chr28	11498015	2	294	A:0.969388	AT:0.0306122
chr28	11499679	2	308	A:0.970779	G:0.0292208
chr28	11501918	2	288	T:0.96875	C:0.03125
chr28	11502579	2	300	A:0.973333	G:0.0266667
chr28	11502629	2	310	T:0.964516	C:0.0354839
chr28	11502635	2	310	A:0.964516	G:0.0354839
chr28	11502653	2	308	C:0.964286	T:0.0357143
chr28	11502734	2	304	A:0.963816	C:0.0361842
chr28	11503113	2	302	A:0.97351	G:0.0264901
chr28	11503177	2	300	T:0.973333	C:0.0266667
chr28	11503288	2	306	A:0.957516	G:0.0424837
chr28	11503334	2	304	G:0.957237	A:0.0427632
chr28	11503634	2	304	T:0.963816	C:0.0361842
chr28	11504069	2	278	G:0.953237	A:0.0467626
chr28	11504071	2	278	T:0.953237	C:0.0467626
chr28	11504334	2	142	ACC:0.93662	A:0.0633803
chr28	11504721	2	310	CCATTTTT:0.948387	C: 0.0516129
chr28	11505153	2	290	G:0.958621	T:0.0413793
chr28	11505207	2	308	G:0.961039	A:0.038961
chr28	11505367	2	302	A:0.970199	G:0.0298013
chr28	11505524	2	308	C:0.967532	T:0.0324675
chr28	11505676	2	306	C:0.921569	T:0.0784314
chr28	11505773	2	298	G:0.681208	GA:0.318792
chr28	11505867	2	318	G:0.974843	A:0.0251572

chr28 11506135 3 316 CCT:0.64557 C:0.0632911
CCTCTCT:0.291139
chr28 11506272 2 312 T:0.971154 C:0.0288462
chr28 11506414 2 298 A:0.620805 G:0.379195
chr28 11506817 2 298 AC:0.687919 A:0.312081
chr28 11507238 3 296 TTG:0.577703 T:0.138514
TTGTG:0.283784
chr28 11507382 2 306 A:0.637255 G:0.362745
chr28 11507492 2 300 A:0.663333 T:0.336667
chr28 11507572 2 306 C:0.633987 A:0.366013
chr28 11507680 2 308 G:0.649351 A:0.350649
chr28 11507821 2 308 T:0.649351 C:0.350649
chr28 11507946 2 310 G:0.63871 T:0.36129
chr28 11508055 2 302 A:0.668874 G:0.331126
chr28 11508165 2 310 C:0.990323 T:0.00967742
chr28 11509514 2 266 CT:0.860902 C:0.139098
chr28 11509614 2 302 G:0.927152 T:0.0728477
chr28 11510272 2 296 T:0.679054 G:0.320946
chr28 11511212 2 316 C:0.683544
CGCCTTGATTATGATACTACACTTGATCTTAGCCAAAAGGCCGAGAAGCGAT:0.316456
chr28 11511222 2 314 A:0.914013 G:0.0859873
chr28 11511233 2 316 A:0.96519 G:0.0348101
chr28 11511316 2 306 C:0.676471 T:0.323529
chr28 11511841 2 302 T:0.672185 C:0.327815
chr28 11511913 2 310 C:0.664516 T:0.335484
chr28 11512011 2 276 TTG:0.0108696 T:0.98913
chr28 11512407 2 300 A:0.91 T:0.09
chr28 11513140 2 238 CT:0.634454 C:0.365546
chr28 11513530 2 302 A:0.798013 G:0.201987
chr28 11513642 2 310 C:0.967742 T:0.0322581
chr28 11514371 2 310 T:0.629032 C:0.370968
chr28 11514674 2 316 C:0 CA:1
chr28 11514693 2 316 C:0 T:1
chr28 11514765 2 312 A:0.980769 T:0.0192308
chr28 11514797 2 314 G:0.535032 GATT:0.464968
chr28 11514978 2 304 C:0.526316 G:0.473684
chr28 11515167 2 306 GCTGT:0.545752 G:0.454248
chr28 11515383 2 304 T:0.526316 C:0.473684
chr28 11515793 2 302 C:0.549669 G:0.450331
chr28 11516163 2 304 CAAGAT:0.907895 C:0.0921053
chr28 11516390 2 300 C:0.916667 CCCA:0.0833333
chr28 11516429 2 302 C:0.907285 T:0.0927152
chr28 11516463 2 300 C:0.973333 T:0.0266667
chr28 11516757 2 304 G:0.555921 A:0.444079
chr28 11516761 2 306 A:0.444444 G:0.555556
chr28 11516798 2 306 C:0.771242 T:0.228758
chr28 11517070 2 296 G:0.898649 C:0.101351
chr28 11517763 2 304 A:0.924342 T:0.0756579
chr28 11517929 2 292 C:0.90411 T:0.0958904
chr28 11518074 2 298 C:0.963087 T:0.0369128
chr28 11518205 2 292 G:0.924658 A:0.0753425
chr28 11518482 2 296 G:0.800676 A:0.199324
chr28 11518915 2 286 C:0.944056 T:0.0559441
chr28 11519820 2 288 G:0.541667 A:0.458333

chr28	11520070	2	294	A:0.435374	C:0.564626		
chr28	11520739	2	310	A:0.480645	ATTTAT:0.519355		
chr28	11521589	2	294	TCGC:0.462585	T:0.537415		
chr28	11521714	2	292	C:0.482877	A:0.517123		
chr28	11522169	2	284	C:0.492958	T:0.507042		
chr28	11522337	2	270	CT:0.914815	C:0.0851852		
chr28	11522354	4	294	T:0.527211	C:0.12585	TC:0.163265	TTC:0.183673
chr28	11522935	2	310	TA:0.490323	T:0.509677		
chr28	11523560	2	196	G:0.357143	GC:0.642857		
chr28	11524592	2	302	C:0.764901	T:0.235099		
chr28	11525728	2	290	A:0.813793	G:0.186207		
chr28	11526058	2	272	G:0.966912	A:0.0330882		
chr28	11527611	2	300	T:0.523333	C:0.476667		
chr28	11528478	2	312	G:0.919872	A:0.0801282		
chr28	11530233	2	282	C:0	CG:1		
chr28	11530244	2	292	A:0	C:1		
chr28	11530269	2	298	C:0	CA:1		
chr28	11530274	2	298	AT:0	A:1		
chr28	11530369	2	306	G:0.80719	A:0.19281		
chr28	11530818	2	290	C:0.968966	G:0.0310345		
chr28	11530874	2	294	C:0.972789	T:0.0272109		
chr28	11531543	2	306	G:0.54902	T:0.45098		
chr28	11532105	2	294	T:0.952381	TGAGA:0.047619		
chr28	11532111	2	292	A:0.928082	G:0.0719178		
chr28	11532192	2	296	G:0.962838	A:0.0371622		
chr28	11532549	2	288	G:0.965278	A:0.0347222		
chr28	11532847	2	308	T:0.964286	TACCAC:0.0357143		
chr28	11533505	2	308	T:0.496753	A:0.503247		
chr28	11533827	2	302	A:0.887417	G:0.112583		
chr28	11533856	2	308	T:0.977273	A:0.0227273		
chr28	11533911	2	308	T:0.487013	C:0.512987		
chr28	11533989	2	288	C:0.5	CAG:0.5		
chr28	11534005	2	292	C:0.962329	T:0.0376712		
chr28	11534124	2	308	T:0.970779	C:0.0292208		
chr28	11534183	2	308	C:0.967532	CT:0.0324675		
chr28	11534650	2	312	G:0.903846	A:0.0961538		
chr28	11534799	2	304	C:0.957237	T:0.0427632		
chr28	11535501	2	302	C:0.688742	T:0.311258		
chr28	11535645	2	292	G:0.924658	GA:0.0753425		
chr28	11535897	2	304	C:0.921053	T:0.0789474		
chr28	11535924	2	298	C:0.973154	T:0.0268456		
chr28	11535926	2	298	C:0.919463	G:0.0805369		
chr28	11536344	2	302	C:0.92053	A:0.0794702		
chr28	11536414	2	298	G:0.949664	A:0.0503356		
chr28	11536599	2	256	CT:0.523438	C:0.476562		
chr28	11536711	2	292	G:0.934932	GA:0.0650685		
chr28	11537054	3	312	GTCTC:0.532051	G:0.426282	GTCTCTCTCTCTC:0.0416667	
chr28	11537056	2	312	C:0.967949	CTCTCTCTG:0.0320513		
chr28	11537152	2	308	T:0.928571	C:0.0714286		
chr28	11537281	2	280	G:0.932143	A:0.0678571		
chr28	11537416	2	306	G:0.751634	A:0.248366		

chr28	11537696	2	290	C:0.948276	T:0.0517241
chr28	11538009	2	314	A:0.703822	C:0.296178
chr28	11538294	2	304	T:0.976974	TC:0.0230263
chr28	11538675	2	292	T:0.931507	C:0.0684932
chr28	11538753	2	302	A:0.688742	G:0.311258
chr28	11539134	2	302	GC:0.622517	G:0.377483
chr28	11539147	2	306	T:0.633987	C:0.366013
chr28	11539150	2	300	T:0.0266667	C:0.973333
chr28	11539227	2	308	G:0.792208	T:0.207792
chr28	11539262	2	312	C:0.964744	T:0.0352564
chr28	11540390	2	296	C:0.972973	T:0.027027
chr28	11540567	2	302	C:0.933775	T:0.0662252
chr28	11540668	2	292	C:0.921233	T:0.0787671
chr28	11540718	2	300	G:0.556667	C:0.443333
chr28	11540928	2	292	G:0.561644	A:0.438356
chr28	11541108	2	302	A:0.357616	C:0.642384
chr28	11541429	2	298	G:0.788591	A:0.211409
chr28	11541457	2	296	G:0.969595	T:0.0304054
chr28	11541535	2	300	G:0.783333	A:0.216667
chr28	11541554	2	304	C:0.996711	A:0.00328947
chr28	11542693	2	304	C:0.782895	T:0.217105
chr28	11543092	2	300	G:0.28 GCC:0.72	
chr28	11543557	2	292	A:0.589041	C:0.410959
chr28	11543704	2	304	A:0.381579	G:0.618421
chr28	11543708	2	302	A:0.990066	C:0.00993377
chr28	11543713	2	304	A:0.381579	C:0.618421
chr28	11544014	2	300	G:0.986667	A:0.0133333
chr28	11544060	2	310	A:0.787097	G:0.212903
chr28	11544073	2	306	C:0.787582	T:0.212418
chr28	11544252	2	290	C:0.924138	T:0.0758621
chr28	11544280	2	294	A:0.656463	G:0.343537
chr28	11544391	2	294	C:0.717687	T:0.282313
chr28	11544484	2	300	C:0.416667	T:0.583333
chr28	11544499	2	302	G:0.807947	GCT:0.192053
chr28	11544581	2	294	G:0.77551	A:0.22449
chr28	11544714	2	296	T:0.175676	TG:0.824324
chr28	11544717	2	298	A:0.949664	G:0.0503356
chr28	11544799	2	296	G:0.402027	T:0.597973
chr28	11544827	2	294	T:0.714286	C:0.285714
chr28	11544877	2	296	GTGT:0.949324	G:0.0506757
chr28	11544883	2	276	GT:0.771739	G:0.228261
chr28	11544890	2	296	G:0.949324	GC:0.0506757
chr28	11544914	2	292	T:0.496575	C:0.503425
chr28	11544988	2	302	C:0.36755	T:0.63245
chr28	11545100	2	290	G:0.937931	GC:0.062069
chr28	11545302	2	298	C:0.983221	T:0.0167785
chr28	11545375	2	288	A:0.940972	G:0.0590278
chr28	11545391	2	282	A:0.72695	C:0.27305
chr28	11545565	2	274	G:0.405109	T:0.594891
chr28	11545679	2	268	T:0.988806	C:0.011194
chr28	11545691	2	260	T:0.742308	TG:0.257692
chr28	11545745	2	290	C:0.924138	CCTGCTT:0.0758621
chr28	11545856	2	276	G:0.387681	C:0.612319
chr28	11545895	2	274	A:0.386861	G:0.613139

chr28	11545936	2	290	G:0.365517	T:0.634483	
chr28	11545944	2	290	T:0.365517	C:0.634483	
chr28	11546339	2	292	C:0.506849	G:0.493151	
chr28	11546711	2	294	T:0	C:1	
chr28	11547246	2	300	C:0.586667	T:0.413333	
chr28	11547749	2	296	T:0.564189	C:0.435811	
chr28	11547825	2	300	G:0.58	A:0.42	
chr28	11547909	2	292	A:0.568493	G:0.431507	
chr28	11547965	2	294	G:0.57483	A:0.42517	
chr28	11548201	2	278	GGC:0.611511	G:0.388489	
chr28	11548684	2	300	A:0.6	C:0.4	
chr28	11548728	2	296	G:0.560811	A:0.439189	
chr28	11548790	2	302	T:0.798013	C:0.201987	
chr28	11548905	2	302	T:0.582781	C:0.417219	
chr28	11549158	2	290	C:0.67931	T:0.32069	
chr28	11549225	2	298	G:0.815436	C:0.184564	
chr28	11549422	2	296	G:0.972973	A:0.027027	
chr28	11549854	2	298	GC:0.536913	G:0.463087	
chr28	11549886	2	292	A:0.315068	C:0.684932	
chr28	11550451	2	300	G:0.506667	T:0.493333	
chr28	11550453	2	300	C:0.506667	T:0.493333	
chr28	11550474	2	300	C:0.6	T:0.4	
chr28	11550527	2	302	T:0.327815	C:0.672185	
chr28	11550532	2	300	A:0.536667	G:0.463333	
chr28	11550589	2	280	G:0.517857	GA:0.482143	
chr28	11551403	2	312	T:0.913462	C:0.0865385	
chr28	11551428	2	304	G:0.782895	A:0.217105	
chr28	11551678	2	314	C:0.503185	A:0.496815	
chr28	11551766	2	300	A:0.796667	G:0.203333	
chr28	11552315	2	302	C:0.811258	T:0.188742	
chr28	11552628	2	308	G:0.769481	C:0.230519	
chr28	11552797	2	294	A:0.309524	G:0.690476	
chr28	11552898	2	306	G:0.764706	A:0.235294	
chr28	11552956	2	304	C:0.973684	T:0.0263158	
chr28	11553028	2	292	C:0.80137	T:0.19863	
chr28	11553058	2	298	C:0.996644	A:0.0033557	
chr28	11553744	2	134	G:0.432836	A:0.567164	
chr28	11553875	2	216	A:0.166667	C:0.833333	
chr28	11554042	2	264	G:0.378788	A:0.621212	
chr28	11554074	2	278	C:0.805755	T:0.194245	
chr28	11554165	2	292	G:0.972603	T:0.0273973	
chr28	11554424	2	296	T:0.793919	C:0.206081	
chr28	11554540	2	304	G:0.786184	A:0.213816	
chr28	11554663	3	292	AC:0.195205	A:0.640411	ACC:0.164384
chr28	11555304	2	302	A:0.990066	C:0.00993377	
chr28	11555340	2	302	C:0.798013	A:0.201987	
chr28	11555434	2	296	A:0.182432	G:0.817568	
chr28	11555462	2	286	A:0.839161	AC:0.160839	
chr28	11555463	2	286	G:0.167832	C:0.832168	
chr28	11555641	2	294	C:0.785714	T:0.214286	
chr28	11556141	2	280	G:0.803571	A:0.196429	
chr28	11556147	2	278	C:0.866906	T:0.133094	
chr28	11556157	2	272	C:0.952206	T:0.0477941	

chr28 11556412 2 304 C:0.388158 G:0.611842
chr28 11556493 2 308 A:0.37987 G:0.62013
chr28 11556601 2 302 G:0.523179 A:0.476821
chr28 11556642 2 294 A:0.367347 G:0.632653
chr28 11556693 3 304 TAC:0.361842 T:0.213816
TACACAC:0.424342
chr28 11556796 2 302 A:0.360927 G:0.639073
chr28 11557075 2 286 A:0.153846 G:0.846154
chr28 11557107 2 294 C:0.370748 T:0.629252
chr28 11557257 2 276 TGG:0.282609 T:0.717391
chr28 11557409 2 304 TCC:0.496711 T:0.503289
chr28 11557492 2 302 C:0.791391 T:0.208609
chr28 11558629 2 302 G:0.791391 A:0.208609
chr28 11558708 3 302 TAA:0.178808 T:0.218543
TA:0.602649
chr28 11558881 2 296 T:0.777027 C:0.222973
chr28 11558998 2 316 C:0.987342 A:0.0126582
chr28 11559044 2 308 T:0.873377 C:0.126623
chr28 11559049 2 306 A:0.98366 G:0.0163399
chr28 11559113 2 298 C:0.869128 T:0.130872
chr28 11559336 2 302 G:0.857616 A:0.142384
chr28 11559370 2 302 G:0.784768 A:0.215232
chr28 11559660 2 298 G:0.506711 A:0.493289
chr28 11559680 2 306 C:0.996732 A:0.00326797
chr28 11559789 2 310 G:0.854839 A:0.145161
chr28 11560380 2 280 G:0.975 GAA:0.025
chr28 11560678 2 280 T:0.917857 C:0.0821429
chr28 11560738 2 294 C:0.911565 T:0.0884354
chr28 11560775 2 292 G:0.90411 A:0.0958904
chr28 11560960 2 316 ACAGGGTGACTTG:0.481013 A:
0.518987
chr28 11561034 2 298 T:0.513423 C:0.486577
chr28 11561179 2 298 A:0.983221 G:0.0167785
chr28 11561313 2 318 A:0.996855
ATTTGTGTTGTGTTGTGTTGTATGTCTTTATATGG:0.00314465
chr28 11561358 2 296 T:0.47973 C:0.52027
chr28 11561433 2 316 G:0.984177 A:0.0158228
chr28 11561807 2 306 C:0.937908 T:0.0620915
chr28 11562183 2 302 C:0.993377 T:0.00662252
chr28 11562308 2 296 A:0.283784 T:0.716216
chr28 11562402 2 300 G:0.82 A:0.18
chr28 11562430 2 302 C:0.956954 T:0.0430464
chr28 11562453 2 306 G:0.869281 A:0.130719
chr28 11562652 2 300 C:0.98 T:0.02
chr28 11562724 2 314 T:0.270701 C:0.729299
chr28 11562746 2 314 A:0.270701 G:0.729299
chr28 11562877 2 314 A:0.863057 C:0.136943
chr28 11562957 2 304 G:0.279605 A:0.720395
chr28 11563024 2 296 G:0.277027 T:0.722973
chr28 11563301 2 288 T:0.28125 C:0.71875
chr28 11563332 2 298 G:0.288591 A:0.711409
chr28 11563436 2 290 T:0.172414 C:0.827586
chr28 11563631 2 296 C:0.293919 T:0.706081
chr28 11564298 2 294 C:0.319728 T:0.680272

chr28	11564438	2	292	G:0.174658	GTC:0.825342
chr28	11564743	2	302	T:0.304636	C:0.695364
chr28	11564765	2	290	C:0.289655	A:0.710345
chr28	11564817	2	280	T:0.164286	C:0.835714
chr28	11564942	2	286	CTCTCT:0.391608	C:0.608392
chr28	11564975	2	306	TA:0.339869	T:0.660131
chr28	11564976	2	304	A:0.973684	T:0.0263158
chr28	11564977	2	302	A:0.943709	T:0.0562914
chr28	11564997	2	306	T:0.339869	C:0.660131
chr28	11565006	2	302	G:0.857616	GA:0.142384
chr28	11565038	2	314	G:0.308917	A:0.691083
chr28	11565040	2	312	TGTTA:0.955128	T:0.0448718
chr28	11565107	2	296	A:0.179054	C:0.820946
chr28	11565280	2	308	C:0.305195	A:0.694805
chr28	11565509	2	300	G:0.976667	A:0.0233333
chr28	11565518	2	300	C:0.566667	T:0.433333
chr28	11565525	2	304	C:0.983553	T:0.0164474
chr28	11565938	2	300	A:0.163333	T:0.836667
chr28	11566480	2	308	C:0.172078	A:0.827922
chr28	11566746	2	308	A:0.980519	G:0.0194805
chr28	11567133	2	296	A:0.168919	G:0.831081
chr28	11567398	2	312	C:0.192308	G:0.807692
chr28	11567410	2	312	T:0.192308	A:0.807692
chr28	11567464	2	310	G:0.187097	C:0.812903
chr28	11567663	2	310	A:0.996774	G:0.00322581
chr28	11567932	2	310	A:0.158065	G:0.841935
chr28	11568038	2	310	T:0.190323	C:0.809677
chr28	11568052	2	302	T:0.195364	C:0.804636
chr28	11568086	2	294	T:0.163265	C:0.836735
chr28	11568578	2	296	T:0.185811	C:0.814189
chr28	11568998	2	290	C:0.741379	A:0.258621
chr28	11569485	2	272	A:0.974265	AC:0.0257353
chr28	11569547	2	298	G:0.16443	A:0.83557
chr28	11569937	2	302	G:0.983444	A:0.0165563
chr28	11570207	2	308	G:0.175325	GA:0.824675
chr28	11570420	2	296	T:0.182432	C:0.817568
chr28	11570482	2	308	TC:0.977273	T:0.0227273
chr28	11571261	2	278	A:0.507194	T:0.492806
chr28	11571394	2	292	G:0.804795	C:0.195205
chr28	11571395	2	292	G:0.804795	T:0.195205
chr28	11571515	2	298	C:0	CT:1
chr28	11571579	2	304	C:0	A:1
chr28	11571582	2	304	T:0	A:1
chr28	11571583	2	304	G:0	A:1
chr28	11571586	2	304	G:0	A:1
chr28	11571687	2	246	C:0.918699	T:0.0813008
chr28	11571773	2	240	CCA:0.679167	C:0.320833
chr28	11571812	2	226	T:0.831858	G:0.168142
chr28	11572221	2	90	A:0.411111	G:0.588889
chr28	11572368	2	210	G:0.67619	T:0.32381
chr28	11572559	2	280	T:0.475	C:0.525
chr28	11572602	2	232	A:0.599138	T:0.400862
chr28	11572697	2	192	G:0.578125	A:0.421875
chr28	11572722	2	214	G:0.906542	A:0.0934579

chr28	11572829	2	160	A:0.475	T:0.525		
chr28	11573250	2	282	T:0.588652	C:0.411348		
chr28	11573328	2	282	G:0.975177	A:0.0248227		
chr28	11573350	2	296	A:0	T:1		
chr28	11573351	2	296	A:0	T:1		
chr28	11573352	2	296	A:0	T:1		
chr28	11573392	2	304	A:0.00657895	AT:0.993421		
chr28	11573397	2	304	A:0.00657895	AT:0.993421		
chr28	11573680	2	306	T:0.571895	C:0.428105		
chr28	11573861	2	304	A:0.605263	G:0.394737		
chr28	11573910	2	306	A:0.604575	G:0.395425		
chr28	11573959	2	306	A:0.98366	C:0.0163399		
chr28	11574101	2	318	TTTTCTTTCTTTC:0.597484	T:0.402516		
chr28	11574186	4	320	CTTCCTTTCCTTTCCTTTCCTTTCCTTTCCT:0.215625	C:0.3375	CTTCCTTTCCT:0.2	CTTCCTTTCCTTTCCTTTCCT:0.246875
chr28	11574601	2	312	C:0.214744	CAAAT:0.785256		
chr28	11574693	2	302	T:0.612583	G:0.387417		
chr28	11574809	2	300	C:0.516667	CT:0.483333		
chr28	11574818	2	302	A:0.615894	T:0.384106		
chr28	11575729	2	292	AG:0.708904	A:0.291096		
chr28	11576021	2	306	G:0.996732	A:0.00326797		
chr28	11576230	2	312	A:0.198718	T:0.801282		
chr28	11576419	2	308	T:0.224026	TG:0.775974		
chr28	11576643	2	310	CAAACAA:0.887097	C:0.112903		
chr28	11576716	2	300	GTA:0.18	G:0.82		
chr28	11576738	2	304	T:0.815789	A:0.184211		
chr28	11576853	2	294	G:0.391156	C:0.608844		
chr28	11576869	2	296	C:0.847973	T:0.152027		
chr28	11577188	2	218	CCT:0.334862	C:0.665138		
chr28	11577216	2	232	A:0.87931	AT:0.12069		
chr28	11577346	2	304	G:0.203947	A:0.796053		
chr28	11577432	2	312	G:0.810897	A:0.189103		
chr28	11577541	2	314	C:0.808917	T:0.191083		
chr28	11577594	2	308	A:0.980519	G:0.0194805		
chr28	11577789	2	300	C:0.35	A:0.65		
chr28	11577927	2	302	T:0.172185	TAA:0.827815		
chr28	11577973	2	304	T:0.161184	C:0.838816		
chr28	11578112	2	292	C:0.10274	T:0.89726		
chr28	11578215	2	286	A:0.216783	C:0.783217		
chr28	11578269	2	78	C:0.923077	A:0.0769231		
chr28	11578484	2	278	G:0.791367	A:0.208633		
chr28	11578533	2	296	C:0.905405	T:0.0945946		
chr28	11578564	2	286	C:0.216783	A:0.783217		
chr28	11578718	2	296	C:0.402027	T:0.597973		
chr28	11578778	2	290	TAC:0.503448	T:0.496552		
chr28	11578794	2	290	G:0.17931	A:0.82069		
chr28	11578798	2	282	A:0.890071	ATG:0.109929		
chr28	11578865	2	298	A:0.47651	T:0.52349		
chr28	11578932	2	290	A:0.2	G:0.8		
chr28	11578956	2	286	C:0.531469	A:0.468531		
chr28	11579440	2	310	G:0.193548	A:0.806452		
chr28	11579558	2	308	T:0.350649	C:0.649351		
chr28	11580025	2	308	G:0.977273	A:0.0227273		

chr28	11580346	2	310	C:0.312903	T:0.687097
chr28	11580885	2	302	G:0.34106	T:0.65894
chr28	11581125	2	298	G:0.895973	A:0.104027
chr28	11581162	2	288	G:0.965278	A:0.0347222
chr28	11581232	2	294	G:0.904762	A:0.0952381
chr28	11581388	2	304	T:0.190789	G:0.809211
chr28	11581861	2	296	G:0.192568	A:0.807432
chr28	11581981	2	314	T:0.872611	C:0.127389
chr28	11582049	2	304	T:0.901316	A:0.0986842
chr28	11582340	2	274	G:0.879562	A:0.120438
chr28	11582811	2	272	C:0.900735	T:0.0992647
chr28	11582964	2	296	A:0.976351	C:0.0236486
chr28	11583458	2	270	AC:0.718519	A:0.281481
chr28	11583596	2	296	C:0.496622	T:0.503378
chr28	11583717	2	302	C:0.195364	A:0.804636
chr28	11583875	2	304	G:0.904605	A:0.0953947
chr28	11583892	2	308	G:0.188312	A:0.811688
chr28	11584035	2	306	T:0.986928	C:0.0130719
chr28	11584058	2	294	T:0.982993	G:0.0170068
chr28	11584794	2	294	CT:0.772109	C:0.227891
chr28	11585232	2	300	A:0.896667	G:0.103333
chr28	11585430	2	296	A:0.459459	G:0.540541
chr28	11585454	2	304	G:0.980263	C:0.0197368
chr28	11585532	2	308	A:0.967532	C:0.0324675
chr28	11585712	2	294	C:0.462585	T:0.537415
chr28	11585736	2	298	G:0.97651	A:0.0234899
chr28	11587009	2	306	C:0.918301	T:0.0816993
chr28	11587232	2	282	TGA:0.187943	T:0.812057
chr28	11587312	2	288	G:0.440972	A:0.559028
chr28	11587332	2	282	G:0.971631	A:0.0283688
chr28	11587641	2	294	G:0.986395	A:0.0136054
chr28	11587651	2	292	A:0.438356	G:0.561644
chr28	11588148	2	308	A:0.412338	G:0.587662
chr28	11588471	3	296	CT:0.638514	C:0.185811
CTT:0.175676					
chr28	11588601	2	294	T:0.176871	C:0.823129
chr28	11588763	2	310	C:0.974194	T:0.0258065
chr28	11589217	2	306	C:0.496732	T:0.503268
chr28	11589258	2	310	T:0.703226	C:0.296774
chr28	11589596	2	302	G:0.993377	A:0.00662252
chr28	11589598	2	302	C:0.493377	T:0.506623
chr28	11590069	2	290	T:0.848276	C:0.151724
chr28	11590104	2	278	C:0.985612	T:0.0143885
chr28	11590299	2	298	C:0.503356	A:0.496644
chr28	11590481	2	302	A:0.860927	G:0.139073
chr28	11590817	2	294	G:0.312925	A:0.687075
chr28	11590925	2	302	T:0.168874	C:0.831126
chr28	11591042	2	304	T:0.861842	C:0.138158
chr28	11591043	2	304	G:0.861842	T:0.138158
chr28	11591057	2	304	A:0.871711	T:0.128289
chr28	11591069	2	306	T:0.849673	C:0.150327
chr28	11591173	2	308	G:0.983766	A:0.0162338
chr28	11591421	2	298	T:0.986577	TG:0.0134228
chr28	11591717	2	302	C:0.97351	T:0.0264901

chr28	11591970	2	294	G:0.435374	T:0.564626		
chr28	11592040	2	290	T:0.813793	G:0.186207		
chr28	11592531	2	304	A:0.980263	G:0.0197368		
chr28	11592748	2	296	T:0.976351	G:0.0236486		
chr28	11592995	2	264	G:0.92803	C:0.0719697		
chr28	11593095	2	202	A:0.445545	G:0.554455		
chr28	11593209	2	156	G:0.782051	A:0.217949		
chr28	11593286	2	178	GTGCGGC:0.522472	G:0.477528		
chr28	11593315	2	214	CGGGCCA:0.995327	C:0.0046729		
chr28	11593329	2	236	GGCCAGGGCCAGC:0.741525	G:0.258475		
chr28	11593513	2	278	C:0.241007	CG:0.758993		
chr28	11593558	2	284	C:0.725352	T:0.274648		
chr28	11594256	2	304	C:0.983553	CCCCT:0.0164474		
chr28	11594264	2	296	T:0.310811	TC:0.689189		
chr28	11594265	3	302	T:0.294702	TC:0.317881	TCTC:0.387417	
chr28	11594286	2	296	AT:0.881757	A:0.118243		
chr28	11594291	2	296	T:0.658784	A:0.341216		
chr28	11594296	2	296	T:0.881757	A:0.118243		
chr28	11594297	4	306	TA:0.418301	T:0.281046	AA:0.189542	TAA:0.111111
chr28	11594331	2	310	C:0.925806	T:0.0741935		
chr28	11594374	2	310	G:0.754839	A:0.245161		
chr28	11594483	2	302	T:0.470199	G:0.529801		
chr28	11594670	2	310	G:0.780645	T:0.219355		
chr28	11594671	2	310	T:0.780645	C:0.219355		
chr28	11594796	2	310	A:0.519355	G:0.480645		
chr28	11594872	2	314	C:0.703822	T:0.296178		
chr28	11594893	2	312	C:0.50641	CAGAA:0.49359		
chr28	11595014	2	302	A:0.509934	G:0.490066		
chr28	11595259	2	302	A:0.509934	T:0.490066		
chr28	11595360	2	290	C:0.831034	T:0.168966		
chr28	11595626	2	304	C:0.509868	T:0.490132		
chr28	11595862	2	302	G:0.662252	T:0.337748		
chr28	11595870	2	304	C:0.825658	T:0.174342		
chr28	11595883	2	300	T:0.826667	C:0.173333		
chr28	11596094	2	306	T:0.777778	C:0.222222		
chr28	11596296	2	308	G:0.977273	A:0.0227273		
chr28	11596429	2	314	T:0.254777	C:0.745223		
chr28	11596526	2	302	A:0.788079	G:0.211921		
chr28	11596568	2	290	T:0.786207	A:0.213793		
chr28	11596570	2	292	C:0.989726	T:0.010274		
chr28	11596721	2	298	G:0.483221	C:0.516779		
chr28	11596765	3	306	GTT:0.48366	G:0.24183	GT:0.27451	
chr28	11596776	2	306	TTTTTTG:0.75817	T:0.24183		
chr28	11596779	2	304	TTTG:0.976974	T:0.0230263		
chr28	11596782	2	308	G:0.811688	T:0.188312		
chr28	11596785	2	308	T:0.811688	G:0.188312		
chr28	11596787	2	304	T:0.723684	G:0.276316		
chr28	11596788	2	304	TG:0.723684	T:0.276316		
chr28	11596789	3	310	GTT:0.577419	G:0.23871	TTT:0.183871	

chr28	11596794	2	304	T:0.976974	TG:0.0230263
chr28	11596927	2	302	T:0.721854	A:0.278146
chr28	11597021	3	290	C:0.296552	CT:0.441379
CTT:0.262069
chr28	11597352	2	308	C:0.720779	T:0.279221
chr28	11597367	2	304	G:0.473684	T:0.526316
chr28	11597372	2	308	G:0.720779	A:0.279221
chr28	11597823	2	298	C:0.771812	T:0.228188
chr28	11598185	4	304	CT:0.411184	C:0.148026
CTT:0.138158	CTTT:0.302632
chr28	11598207	2	304	A:0.700658	T:0.299342
chr28	11598277	2	296	G:0.760135	A:0.239865
chr28	11598278	2	296	A:0.760135	T:0.239865
chr28	11598332	2	294	G:0.751701	GGAT:0.248299
chr28	11598335	2	294	C:0.751701	T:0.248299
chr28	11598396	2	268	C:0.75	CT:0.25
chr28	11598404	2	272	C:0.253676	T:0.746324
chr28	11598484	2	306	G:0.771242	T:0.228758
chr28	11598559	2	310	A:0.974194	G:0.0258065
chr28	11598737	2	300	C:0.883333	CA:0.116667
chr28	11598800	4	306	CTT:0.287582	C:0.222222
CT:0.245098	CTTT:0.245098
chr28	11598860	2	306	C:0.294118	T:0.705882
chr28	11599091	2	300	T:0.72	G:0.28
chr28	11599188	2	304	C:0.986842	A:0.0131579
chr28	11599223	2	310	A:0.73871	C:0.26129
chr28	11599278	2	308	A:0.834416	C:0.165584
chr28	11599456	2	308	T:0.272727	C:0.727273
chr28	11599457	2	312	G:0.766026	A:0.233974
chr28	11599776	2	308	T:0.967532	G:0.0324675
chr28	11599802	2	302	T:0.794702	A:0.205298
chr28	11599821	2	304	T:0.917763	A:0.0822368
chr28	11599924	2	304	G:0.519737	A:0.480263
chr28	11600197	2	300	GA:0.54	G:0.46
chr28	11600932	2	306	T:0.673203	A:0.326797
chr28	11601773	2	308	A:0.935065	C:0.0649351
chr28	11601810	2	314	G:0.550955	A:0.449045
chr28	11601818	2	314	C:0.977707	T:0.022293
chr28	11601898	2	296	C:0.814189	G:0.185811
chr28	11602097	2	304	C:0.447368	A:0.552632
chr28	11602103	2	302	T:0.897351	G:0.102649
chr28	11602416	2	304	T:0.825658	C:0.174342
chr28	11602515	2	310	T:0.825806	C:0.174194
chr28	11602545	2	310	A:0.283871	C:0.716129
chr28	11602891	2	312	C:0.826923	T:0.173077
chr28	11603063	2	314	T:0.964968	G:0.0350318
chr28	11604252	2	304	T:0.506579	C:0.493421
chr28	11604357	2	304	A:0.990132	T:0.00986842
chr28	11604378	4	312	TGAGAGAGAGA:0.391026	T:
0.294872	TGAGA:0.205128	TGAGAGAGA:0.108974
chr28	11604394	2	304	AG:0.825658	A:0.174342
chr28	11604396	2	304	AGAGAGAG:0.825658	A:
0.174342
chr28	11604458	2	292	C:0.493151	G:0.506849

chr28	11604478	2	296	TG:0.712838	T:0.287162	
chr28	11604541	2	302	A:0.781457	T:0.218543	
chr28	11604741	2	306	C:0.986928	A:0.0130719	
chr28	11604757	2	318	ATGAAGAAATGGAGACTGTTT:0.679245	A:0.320755	
chr28	11604949	2	310	A:0.477419	G:0.522581	
chr28	11604955	2	310	A:0.974194	G:0.0258065	
chr28	11605172	2	298	T:0.738255	G:0.261745	
chr28	11605576	2	304	C:0.786184	T:0.213816	
chr28	11605909	2	302	C:0.890728	T:0.109272	
chr28	11606073	2	292	A:0.636986	G:0.363014	
chr28	11606519	2	284	A:0.788732	G:0.211268	
chr28	11606879	2	306	CA:0.977124	C:0.0228758	
chr28	11606945	2	308	T:0.603896	A:0.396104	
chr28	11607011	2	300	A:0.94	G:0.06	
chr28	11607897	2	312	C:0.429487	T:0.570513	
chr28	11608033	2	292	G:0.804795	A:0.195205	
chr28	11608180	3	310	TAAACAAAC:0.435484	T:0.319355	TAAAC:0.245161
chr28	11608898	2	312	A:0.394231	G:0.605769	
chr28	11608945	2	320	G:0.4125	C:0.5875	
chr28	11609061	2	304	T:0.786184	C:0.213816	
chr28	11609073	2	306	A:0.395425	T:0.604575	
chr28	11609146	2	304	T:0.398026	C:0.601974	
chr28	11609265	2	302	T:0.410596	C:0.589404	
chr28	11609484	2	276	C:0.938406	T:0.0615942	
chr28	11609564	2	274	G:0.510949	A:0.489051	
chr28	11609578	2	276	C:0.815217	T:0.184783	
chr28	11609722	2	282	T:0.822695	G:0.177305	
chr28	11609723	2	284	A:0.419014	C:0.580986	
chr28	11609803	2	284	A:0.975352	T:0.0246479	
chr28	11609990	2	302	C:0.42053	A:0.57947	
chr28	11610409	2	294	G:0.806122	A:0.193878	
chr28	11610793	2	302	TCAG:0.791391	T:0.208609	
chr28	11611072	2	306	T:0.611111	C:0.388889	
chr28	11611102	2	292	T:0.633562	C:0.366438	
chr28	11611344	2	304	T:0.983553	C:0.0164474	
chr28	11611618	2	300	C:0.63	G:0.37	
chr28	11611687	2	306	C:0.611111	T:0.388889	
chr28	11611791	2	306	G:0.973856	A:0.0261438	
chr28	11611845	2	302	A:0.619205	T:0.380795	
chr28	11611994	2	310	G:0.848387	A:0.151613	
chr28	11612122	2	308	T:0.642857	C:0.357143	
chr28	11612128	2	308	A:0.892857	C:0.107143	
chr28	11612184	2	298	G:0.90604	A:0.0939597	
chr28	11612734	2	300	C:0.89	T:0.11	
chr28	11612749	2	306	G:0.911765	C:0.0882353	
chr28	11613175	2	314	G:0.875796	A:0.124204	
chr28	11613346	2	300	C:0.963333	T:0.0366667	
chr28	11613350	2	300	G:0.963333	T:0.0366667	
chr28	11613641	2	310	C:0.993548	A:0.00645161	
chr28	11613768	2	286	C:0.986014	T:0.013986	
chr28	11613900	2	314	G:0.987261	A:0.0127389	
chr28	11614047	2	304	T:0.993421	C:0.00657895	

chr28	11614242	2	304	G:0.394737	A:0.605263
chr28	11614331	2	300	C:0.833333	T:0.166667
chr28	11614549	2	316	C:0.825949	T:0.174051
chr28	11614894	2	306	A:0.826797	G:0.173203
chr28	11615171	2	314	G:0.821656	T:0.178344
chr28	11615352	2	306	T:0.810458	TA:0.189542
chr28	11615568	2	298	C:0.815436	A:0.184564
chr28	11615826	2	300	G:0.93 A:0.07	
chr28	11615868	2	302	CTA:0.817881	C:0.182119
chr28	11615952	2	318	ATTTTT:0.77673	A:0.22327
chr28	11615953	2	308	TTTTTTTTTTTTTTTTTTTTTTTTGGTATTTGAA:0.935065	T:0.0649351
chr28	11616070	2	300	C:0.976667	CT:0.0233333
chr28	11616117	2	302	CT:0.798013	C:0.201987
chr28	11616268	2	298	C:0.798658	A:0.201342
chr28	11616320	2	302	G:0.811258	T:0.188742
chr28	11616406	2	302	GTA:0.956954	G:0.0430464
chr28	11616536	2	298	CA:0.798658	C:0.201342
chr28	11616567	2	290	TC:0.882759	T:0.117241
chr28	11616568	2	292	C:0.917808	CT:0.0821918
chr28	11616613	2	292	A:0.934932	C:0.0650685
chr28	11616697	2	290	G:0.186207	A:0.813793
chr28	11616963	2	308	G:0.594156	C:0.405844
chr28	11617017	2	298	T:0.802013	C:0.197987
chr28	11617076	2	302	T:0.811258	G:0.188742
chr28	11617079	2	302	C:0.933775	T:0.0662252
chr28	11617455	2	312	G:0.820513	A:0.179487
chr28	11617492	2	306	T:0.398693	C:0.601307
chr28	11617795	2	306	A:0.993464	G:0.00653595
chr28	11618216	2	306	AT:0.957516	A:0.0424837
chr28	11618267	2	304	T:0.430921	C:0.569079
chr28	11618315	2	304	G:0.832237	A:0.167763
chr28	11618439	2	272	T:0.797794	C:0.202206
chr28	11618928	2	284	GT:0.947183	G:0.0528169
chr28	11618960	2	288	C:0.986111	A:0.0138889
chr28	11619029	2	310	T:0.419355	TG:0.580645
chr28	11619034	2	310	C:0.809677	T:0.190323
chr28	11619208	2	296	G:0.986486	C:0.0135135
chr28	11619407	2	308	A:0.954545	T:0.0454545
chr28	11619418	2	308	A:0.811688	G:0.188312
chr28	11619623	2	306	G:0.941176	T:0.0588235
chr28	11619624	2	306	A:0.385621	T:0.614379
chr28	11619667	2	306	C:0.986928	T:0.0130719
chr28	11619802	2	308	T:0.808442	A:0.191558
chr28	11619807	2	308	G:0.808442	T:0.191558
chr28	11620066	2	306	G:0.941176	A:0.0588235
chr28	11620197	2	310	G:0.958065	A:0.0419355
chr28	11620293	2	316	TG:0.411392	T:0.588608
chr28	11620360	2	304	C:0.990132	T:0.00986842
chr28	11620596	2	306	G:0.852941	A:0.147059
chr28	11620674	2	318	G:0.933962	A:0.0660377
chr28	11620751	2	306	A:0.810458	G:0.189542
chr28	11620765	2	310	C:0.935484	T:0.0645161
chr28	11620843	2	300	A:0.953333	G:0.0466667

chr28	11620896	2	308	A:0.928571	C:0.0714286		
chr28	11621240	2	302	A:0.549669	T:0.450331		
chr28	11621573	2	310	G:0.867742	A:0.132258		
chr28	11621624	2	306	G:0.934641	A:0.0653595		
chr28	11621742	2	302	A:0.559603	G:0.440397		
chr28	11621773	2	316	T:0.933544	C:0.0664557		
chr28	11621848	2	306	G:0.862745	A:0.137255		
chr28	11622244	2	306	T:0.513072	A:0.486928		
chr28	11623106	2	296	A:0.597973	AATTT:0.402027		
chr28	11623164	2	298	C:0.674497	T:0.325503		
chr28	11623166	2	298	C:0.674497	T:0.325503		
chr28	11623243	2	298	T:0.667785	G:0.332215		
chr28	11623314	2	300	G:0.686667	A:0.313333		
chr28	11623361	2	300	C:0.676667	T:0.323333		
chr28	11623373	2	298	T:0.369128	C:0.630872		
chr28	11623385	2	308	G:0.795455	T:0.204545		
chr28	11623406	2	304	C:0.648026	T:0.351974		
chr28	11623440	2	306	T:0.993464	C:0.00653595		
chr28	11623894	2	306	C:0.986928	T:0.0130719		
chr28	11624156	2	304	C:0.940789	T:0.0592105		
chr28	11624224	2	302	T:0.768212	C:0.231788		
chr28	11624878	2	308	A:0.795455	T:0.204545		
chr28	11625075	2	314	C:0.382166	T:0.617834		
chr28	11625161	2	302	C:0.370861	T:0.629139		
chr28	11625279	2	318	T:0.603774	C:0.396226		
chr28	11625400	2	306	GTCAC:0.98366	G:0.0163399		
chr28	11625617	2	312	G:0.349359	A:0.650641		
chr28	11625771	2	318	G:0.361635	C:0.638365		
chr28	11625905	3	304	TC:0.381579	T:0.421053	CC:0.197368	
chr28	11625915	2	304	T:0.578947	C:0.421053		
chr28	11625962	2	308	G:0.37013	GTATT:0.62987		
chr28	11626066	3	298	CTGTGTG:0.486577	C:0.432886	CTGTG:0.0805369	
chr28	11626244	4	306	CAG:0.473856	C:0.205882	CAGAG:0.143791	CAGAGAG:0.176471
chr28	11626315	2	292	G:0.80137	A:0.19863		
chr28	11626402	2	298	A:0.395973	G:0.604027		
chr28	11626637	2	310	C:0.587097	T:0.412903		
chr28	11627103	2	306	C:0.990196	T:0.00980392		
chr28	11627334	2	308	A:0.805195	G:0.194805		
chr28	11627622	2	294	TTC:0.982993	T:0.0170068		
chr28	11627984	2	310	T:0.941935	G:0.0580645		
chr28	11628016	2	304	A:0.776316	T:0.223684		
chr28	11628478	2	308	A:0.392857	G:0.607143		
chr28	11628656	2	304	G:0.944079	A:0.0559211		
chr28	11628658	2	302	G:0.980132	A:0.0198675		
chr28	11628696	2	320	CTTTATCATATTCTTTATTCTCT:0.78125	C:0.21875		
chr28	11628917	2	282	T:0.932624	A:0.0673759		
chr28	11629114	2	300	CTTTTTT:0.756667	C:0.243333		
chr28	11629359	2	308	A:0.717532	G:0.282468		
chr28	11629750	2	306	T:0.937908	C:0.0620915		
chr28	11629885	2	308	G:0.951299	A:0.0487013		

chr28 11629911 2 298 T:0.942953 C:0.057047
chr28 11629947 2 308 C:0.922078 T:0.0779221
chr28 11630066 2 304 T:0.934211 G:0.0657895
chr28 11630218 2 308 T:0.941558 C:0.0584416
chr28 11630386 2 310 A:0.429032 C:0.570968
chr28 11630569 2 302 A:0.718543 G:0.281457
chr28 11630669 2 306 G:0.434641 A:0.565359
chr28 11630972 2 312 C:0.772436 T:0.227564
chr28 11631002 2 312 C:0.807692 G:0.192308
chr28 11631112 2 310 G:0.929032 A:0.0709677
chr28 11631274 2 310 C:0.512903 T:0.487097
chr28 11631806 2 290 AT:0.589655 A:0.410345
chr28 11631807 2 296 T:0.834459 A:0.165541
chr28 11631961 2 302 AC:0.562914 A:0.437086
chr28 11631997 2 294 G:0.877551 T:0.122449
chr28 11632048 2 298 G:0.52349 A:0.47651
chr28 11632389 2 310 A:0.377419 G:0.622581
chr28 11632448 2 308 C:0.844156 G:0.155844
chr28 11633018 2 306 CA:0.529412 C:0.470588
chr28 11633136 2 300 T:0.36 TC:0.64
chr28 11633395 2 310 C:0.990323 T:0.00967742
chr28 11633438 2 296 T:0.844595 C:0.155405
chr28 11633629 2 298 G:0.942953 A:0.057047
chr28 11633883 2 298 G:0.845638 A:0.154362
chr28 11634053 2 302 T:0.36755 C:0.63245
chr28 11634287 2 296 C:0.793919 T:0.206081
chr28 11634408 2 304 TGTAA:0.930921 T:0.0690789
chr28 11634473 2 306 C:0.562092 A:0.437908
chr28 11634938 2 320 C:0.9875 CTTA:0.0125
chr28 11634941 2 320 C:0.9875 G:0.0125
chr28 11634943 2 320 A:0.9875 AGAACCTCT:0.0125
chr28 11634945 2 320 A:0.9875 ATTGAAGAACC:0.0125
chr28 11635125 2 306 GC:0.718954 G:0.281046
chr28 11635128 2 306 T:0.718954 G:0.281046
chr28 11635701 2 302 A:0.370861 G:0.629139
chr28 11636000 2 262 G:0.973282 A:0.0267176
chr28 11636112 2 288 T:0.652778 C:0.347222
chr28 11636162 3 296 G:0.773649 GAATA:0.0810811
GAATAAATA:0.14527
chr28 11636195 2 316 A:0.943038
AATACATAAATACATAAATAC:0.056962
chr28 11636202 2 312 A:0.932692 AC:0.0673077
chr28 11636203 6 318 A:0.484277 AAAAAAAAT:
0.0220126 AATAAAAAT:0.147799 AATAAATAAAAAT:0.242138
AATACATAAATAAATAAATAAAAAT:0.0377358
ATAAATACATAAATACATAAATAAATAAATAAAAAT:0.0660377
chr28 11636449 2 304 GCAA:0.944079 G:0.0559211
chr28 11636450 3 300 CA:0.64 C:0.106667 CAA:
0.253333
chr28 11636585 2 298 T:0.775168 C:0.224832
chr28 11636666 2 306 A:0.901961 C:0.0980392
chr28 11636699 2 300 C:0.79 T:0.21
chr28 11637097 2 262 C:0.923664 T:0.0763359
chr28 11637148 2 242 TTC:0.85124 T:0.14876

chr28	11637876	2	296	A:0.912162	C:0.0878378				
chr28	11638360	2	300	T:0.88	C:0.12				
chr28	11638539	2	306	T:0.996732	C:0.00326797				
chr28	11638683	2	314	C:0.382166	T:0.617834				
chr28	11638838	2	300	T:0.916667	G:0.0833333				
chr28	11638897	2	306	A:0.970588	AGC:0.0294118				
chr28	11638902	3	306	A:0.555556	ACG:0.415033	G:0.0294118			
chr28	11638910	2	306	G:0.921569	GCGCACA:0.0784314				
chr28	11638912	2	284	A:0.820423	G:0.179577				
chr28	11639095	2	296	C:0.993243	T:0.00675676				
chr28	11639874	2	280	C:0.971429	A:0.0285714				
chr28	11640588	6	320	CATCTATCTATCTATCTATCT:0.2875	C:0.125	CATCTATCTATCTATCT:0.16875	CATCTATCTATCTATCTATCTATCT:0.315625	CATCTATCTATCTATCTATCTATCTATCT:0.0875	CATCTATCTATCTATCTATCTATCTATCTATCT:0.015625
chr28	11640754	2	304	A:0.246711	C:0.753289				
chr28	11640794	2	312	T:0.208333	TTATC:0.791667				
chr28	11640807	2	310	G:0.987097	A:0.0129032				
chr28	11640936	2	308	A:0.831169	T:0.168831				
chr28	11641278	2	308	A:0.912338	AT:0.0876623				
chr28	11641403	2	300	A:0.916667	C:0.0833333				
chr28	11641509	2	314	G:0.914013	A:0.0859873				
chr28	11641581	2	304	A:0.973684	G:0.0263158				
chr28	11641643	2	302	C:0.910596	T:0.089404				
chr28	11641993	2	300	C:0.74	T:0.26				
chr28	11642098	2	304	AG:0.720395	A:0.279605				
chr28	11642201	2	294	G:0.887755	A:0.112245				
chr28	11642416	2	292	G:0.760274	C:0.239726				
chr28	11642575	2	302	G:0.738411	A:0.261589				
chr28	11642692	2	296	A:0.739865	G:0.260135				
chr28	11642904	2	288	T:0.75	C:0.25				
chr28	11643368	2	296	CCT:0.942568	C:0.0574324				
chr28	11643389	2	302	C:0.728477	G:0.271523				
chr28	11643473	2	300	C:0.986667	T:0.0133333				
chr28	11643490	2	304	C:0.743421	T:0.256579				
chr28	11643556	2	306	A:0.75817	G:0.24183				
chr28	11643956	2	304	G:0.736842	A:0.263158				
chr28	11643994	2	306	C:0.72549	T:0.27451				
chr28	11644107	2	298	A:0.969799	G:0.0302013				
chr28	11644288	2	300	T:0.74	C:0.26				
chr28	11644717	2	302	A:0.751656	G:0.248344				
chr28	11644808	2	312	A:0.99359	G:0.00641026				
chr28	11645247	2	308	A:0.746753	G:0.253247				
chr28	11646411	2	308	T:0.75974	C:0.24026				
chr28	11646414	2	308	C:0.772727	T:0.227273				
chr28	11646644	2	308	C:0.948052	T:0.0519481				
chr28	11647305	2	288	GT:0.704861	G:0.295139				
chr28	11648316	2	298	TG:0.768456	T:0.231544				
chr28	11648719	2	308	C:0.525974	G:0.474026				
chr28	11648877	2	300	G:0.973333	A:0.0266667				
chr28	11648879	2	300	G:0.573333	GCA:0.426667				
chr28	11649448	2	292	C:0.770548	T:0.229452				
chr28	11649850	2	306	T:0.918301	C:0.0816993				

chr28	11650074	2	306	T:0.53268	C:0.46732	
chr28	11650446	2	304	A:0.993421	T:0.00657895	
chr28	11650763	2	302	T:0.864238	C:0.135762	
chr28	11651129	2	302	C:0.738411	CT:0.261589	
chr28	11651162	2	298	G:0.758389	A:0.241611	
chr28	11651188	2	300	C:0.75	T:0.25	
chr28	11651220	2	298	G:0.751678	A:0.248322	
chr28	11651262	2	302	G:0.735099	C:0.264901	
chr28	11651378	2	304	A:0.743421	G:0.256579	
chr28	11651404	2	306	C:0.754902	T:0.245098	
chr28	11651493	2	304	A:0.753289	T:0.246711	
chr28	11651708	2	300	C:0.786667	T:0.213333	
chr28	11651773	2	304	G:0.756579	A:0.243421	
chr28	11651917	2	308	T:0.74026	A:0.25974	
chr28	11651918	2	308	T:0.74026	C:0.25974	
chr28	11651981	2	304	C:0.743421	CTT:0.256579	
chr28	11652232	2	210	C:0.757143	CCTT:0.242857	
chr28	11652278	2	220	C:0.809091	T:0.190909	
chr28	11652330	2	184	T:0.673913	C:0.326087	
chr28	11652763	2	262	C:0.843511	CTA:0.156489	
chr28	11653163	2	302	GAGA:0.791391	G:0.208609	
chr28	11653191	2	296	C:0.97973	T:0.0202703	
chr28	11653359	2	308	A:0.792208	G:0.207792	
chr28	11653409	2	288	C:0.746528	A:0.253472	
chr28	11653426	2	292	G:0.828767	A:0.171233	
chr28	11653579	2	226	A:0.818584	T:0.181416	
chr28	11653592	2	226	T:0.818584	A:0.181416	
chr28	11654206	2	224	C:0.808036	T:0.191964	
chr28	11654215	2	212	A:0.561321	G:0.438679	
chr28	11654296	2	286	C:0.576923	T:0.423077	
chr28	11654401	2	308	T:0.798701	G:0.201299	
chr28	11654527	2	282	C:0.762411	T:0.237589	
chr28	11654533	2	278	T:0.55036	C:0.44964	
chr28	11654573	3	304	CCT:0.552632	C:0.236842	CCTCTCT:0.210526
chr28	11654622	2	294	C:0.778912	T:0.221088	
chr28	11654717	2	306	G:0.72549	C:0.27451	
chr28	11654744	2	302	T:0.738411	C:0.261589	
chr28	11654788	2	308	C:0.753247	T:0.246753	
chr28	11654806	2	308	G:0.753247	GGA:0.246753	
chr28	11654835	2	302	A:0.754967	G:0.245033	
chr28	11654906	2	312	C:0.769231	T:0.230769	
chr28	11655174	2	312	C:0.775641	T:0.224359	
chr28	11655291	2	310	C:0.754839	T:0.245161	
chr28	11655316	2	310	G:0.754839	A:0.245161	
chr28	11655319	2	306	G:0.996732	A:0.00326797	
chr28	11655324	2	306	C:0.777778	T:0.222222	
chr28	11655454	2	314	T:0.748408	TCTTA:0.251592	
chr28	11655790	2	306	T:0.75817	C:0.24183	
chr28	11655791	2	306	G:0.75817	A:0.24183	
chr28	11655925	2	308	A:0.99026	G:0.00974026	
chr28	11655948	2	306	G:0.745098	A:0.254902	
chr28	11656006	2	304	C:0.970395	T:0.0296053	
chr28	11656314	2	302	A:0.745033	G:0.254967	

chr28 11656333 2 302 A:0.745033 C:0.254967
chr28 11656345 2 302 A:0.745033 C:0.254967
chr28 11656370 2 306 A:0.986928 G:0.0130719
chr28 11656439 2 312 C:0.714744 T:0.285256
chr28 11656683 2 310 C:0.729032 G:0.270968
chr28 11656689 2 314 C:0.847134 G:0.152866
chr28 11656782 2 308 G:0.75974 A:0.24026
chr28 11656805 2 304 AG:0.766447 A:0.233553
chr28 11656806 2 304 G:0.773026 A:0.226974
chr28 11656895 2 298 A:0.734899 T:0.265101
chr28 11657017 2 310 A:0.990323 G:0.00967742
chr28 11657049 2 314 A:0.761146 C:0.238854
chr28 11657074 2 308 A:0.99026 T:0.00974026
chr28 11657122 2 314 T:0.929936 C:0.0700637
chr28 11657324 2 312 A:0.753205 G:0.246795
chr28 11657342 2 312 A:0.753205 C:0.246795
chr28 11657425 2 304 A:0.753289 G:0.246711
chr28 11657472 2 298 CT:0.788591 C:0.211409
chr28 11657604 2 302 A:0.738411 G:0.261589
chr28 11657746 2 302 T:0.741722 C:0.258278
chr28 11657954 5 312 GTTATTATTATTA:0.173077 G:
0.384615 GTTA:0.0480769 GTTATTA:0.25 GTTATTATTA:0.144231
chr28 11657963 2 310 A:0.874194 G:0.125806
chr28 11657969 2 302 A:0.963576 G:0.0364238
chr28 11658263 2 304 A:0.976974 G:0.0230263
chr28 11658441 2 306 C:0.522876 A:0.477124
chr28 11658526 2 300 A:0.99 T:0.01
chr28 11658790 2 310 C:0.990323 A:0.00967742
chr28 11658823 2 300 A:0.39 G:0.61
chr28 11659626 2 310 C:0.806452 CT:0.193548
chr28 11659758 2 306 T:0.800654 C:0.199346
chr28 11661093 3 306 A:0.398693 C:0.00980392
T:0.591503
chr28 11661214 2 300 T:0.376667 TA:0.623333
chr28 11661386 2 290 G:0.989655 A:0.0103448
chr28 11661490 2 302 C:0.986755 T:0.013245
chr28 11661717 2 296 C:0.39527 T:0.60473
chr28 11661758 2 304 A:0.417763 G:0.582237
chr28 11661816 2 304 T:0.921053 A:0.0789474
chr28 11661817 2 304 G:0.921053 T:0.0789474
chr28 11661984 2 302 T:0.403974 C:0.596026
chr28 11662084 2 302 T:0.804636 C:0.195364
chr28 11662169 2 302 C:0.990066 T:0.00993377
chr28 11662237 2 312 T:0.99359 TTAGGTAGAATA:
0.00641026
chr28 11662282 2 306 G:0.973856 C:0.0261438
chr28 11662462 2 318 AAC:0.732704 A:0.267296
chr28 11662480 2 318 ACTGTGAGCTACACTGAC:0.732704
A:0.267296
chr28 11662796 2 308 A:0.396104 AC:0.603896
chr28 11662804 2 308 C:0.779221 T:0.220779
chr28 11662825 2 306 G:0.405229 A:0.594771
chr28 11662862 2 310 T:0.403226 A:0.596774
chr28 11662884 2 308 A:0.418831 T:0.581169

chr28	11663181	2	300	A:0.996667	ACT:0.00333333
chr28	11663292	2	302	A:0.423841	T:0.576159
chr28	11663403	2	300	T:0.36 C:0.64	
chr28	11663419	2	304	T:0.713816	C:0.286184
chr28	11663575	2	314	C:0.382166	T:0.617834
chr28	11663586	2	314	A:0.382166	AT:0.617834
chr28	11663630	2	318	C:0.386792	T:0.613208
chr28	11663675	2	312	T:0.785256	C:0.214744
chr28	11663739	2	302	G:0.380795	T:0.619205
chr28	11663741	2	304	G:0.990132	C:0.00986842
chr28	11663792	2	310	A:0.390323	C:0.609677
chr28	11663894	2	306	GA:0.392157	G:0.607843
chr28	11663948	2	302	C:0.370861	T:0.629139
chr28	11664110	2	310	A:0.990323	T:0.00967742
chr28	11664191	2	310	T:0.729032	C:0.270968
chr28	11664251	2	312	CATA:0.378205	C:0.621795
chr28	11664254	2	312	A:0.987179	T:0.0128205
chr28	11664256	2	312	T:0.987179	C:0.0128205
chr28	11664326	2	312	C:0.375 T:0.625	
chr28	11664351	2	312	T:0.375 C:0.625	
chr28	11664406	2	290	G:0.406897	C:0.593103
chr28	11664486	2	306	A:0.986928	T:0.0130719
chr28	11664713	2	294	G:0.993197	A:0.00680272
chr28	11664724	2	296	G:0.347973	A:0.652027
chr28	11664901	2	306	C:0.990196	A:0.00980392
chr28	11664945	2	298	C:0.651007	A:0.348993
chr28	11664965	2	304	T:0.993421	A:0.00657895
chr28	11665294	2	306	A:0.375817	G:0.624183
chr28	11665320	2	300	C:0.726667	T:0.273333
chr28	11665484	2	298	G:0.365772	GA:0.634228
chr28	11665594	2	306	G:0.388889	A:0.611111
chr28	11665678	2	300	T:0.373333	C:0.626667
chr28	11665705	2	296	C:0.719595	T:0.280405
chr28	11665706	2	296	A:0.361486	G:0.638514
chr28	11665989	2	312	C:0.99359	T:0.00641026
chr28	11666004	2	308	G:0.87987	C:0.12013
chr28	11666012	2	312	T:0.99359	C:0.00641026
chr28	11666106	2	310	G:0.796774	A:0.203226
chr28	11666130	2	310	T:0.367742	C:0.632258
chr28	11666159	2	316	G:0.360759	T:0.639241
chr28	11666161	2	316	C:0.370253	T:0.629747
chr28	11666193	2	310	C:0.970968	T:0.0290323
chr28	11666285	2	296	C:0.368243	T:0.631757
chr28	11666395	2	296	G:0.347973	T:0.652027
chr28	11666414	2	298	T:0.375839	C:0.624161
chr28	11666482	2	306	G:0.388889	A:0.611111
chr28	11666629	2	314	G:0.414013	T:0.585987
chr28	11666647	2	316	C:0.990506	G:0.00949367
chr28	11666701	2	304	C:0.365132	T:0.634868
chr28	11666755	2	318	G:0.399371	A:0.600629
chr28	11666762	2	318	C:0.399371	T:0.600629
chr28	11666835	2	300	CATT:0.78	C:0.22
chr28	11666869	2	304	A:0.786184	T:0.213816
chr28	11666939	2	302	A:0.357616	T:0.642384

chr28 11667041 2 300 C:0.346667 A:0.653333
chr28 11667295 2 318 TAC:0.930818 T:0.0691824
chr28 11667321 2 312 CACACAA:0.862179 C:0.137821
chr28 11667323 2 312 CACAA:0.887821 C:0.112179
chr28 11667325 2 312 CAA:0.737179 C:0.262821
chr28 11667469 2 298 C:0.724832 T:0.275168
chr28 11667474 2 300 G:0.36 A:0.64
chr28 11667485 2 298 C:0.637584 G:0.362416
chr28 11667540 2 304 G:0.365132 A:0.634868
chr28 11667708 2 290 T:0.875862 TAC:0.124138
chr28 11667740 2 290 A:0.386207 G:0.613793
chr28 11667796 2 296 T:0.364865 C:0.635135
chr28 11667797 2 296 T:0.364865 A:0.635135
chr28 11667809 2 296 G:0.976351 A:0.0236486
chr28 11667887 2 304 A:0.884868 G:0.115132
chr28 11668132 2 308 T:0.50974 C:0.49026
chr28 11668503 2 302 C:0.980132 T:0.0198675
chr28 11668526 2 310 GT:0.974194 G:0.0258065
chr28 11668732 2 296 GA:0.983108 G:0.0168919
chr28 11668754 2 298 C:0.97651 T:0.0234899
chr28 11668838 2 296 A:0.986486 G:0.0135135
chr28 11668925 2 306 GCCTTTAA:0.941176 G:
0.0588235
chr28 11668967 2 282 T:0.978723 C:0.0212766
chr28 11669252 2 304 T:0.980263 C:0.0197368
chr28 11669308 2 302 C:0.983444 A:0.0165563
chr28 11669781 2 298 G:0.97651 C:0.0234899
chr28 11670042 2 320 CAT:0.978125 C:0.021875
chr28 11670043 3 310 A:0.480645 ATG:0.470968
ATGTG:0.0483871
chr28 11670061 2 320 G:0.978125 A:0.021875
chr28 11670064 2 320 C:0.959375 G:0.040625
chr28 11670314 2 304 AC:0.763158 A:0.236842
chr28 11670454 2 294 G:0.986395 A:0.0136054
chr28 11670603 2 302 A:0.387417 T:0.612583
chr28 11671073 2 310 C:0.896774 T:0.103226
chr28 11671184 2 310 G:0.467742 A:0.532258
chr28 11671202 2 306 C:0.437908 T:0.562092
chr28 11671346 2 296 T:0.439189 C:0.560811
chr28 11671361 2 302 C:0.423841 T:0.576159
chr28 11671460 2 310 T:0.945161 A:0.0548387
chr28 11671813 2 298 C:0.436242 T:0.563758
chr28 11671926 2 306 G:0.941176 A:0.0588235
chr28 11671947 2 308 C:0.418831 G:0.581169
chr28 11672114 2 318 ACTTTCC:0.940252 A:0.0597484
chr28 11672145 2 318 A:0.940252 G:0.0597484
chr28 11672149 2 308 T:0.431818 A:0.568182
chr28 11672178 2 304 A:0.766447 G:0.233553
chr28 11672321 2 302 C:0.943709 G:0.0562914
chr28 11672355 2 304 T:0.782895 G:0.217105
chr28 11672417 2 294 TG:0.442177 T:0.557823
chr28 11672566 6 312 TTTTATTTATTTATTTATTTA:0.310897
T:0.317308 TTTTA:0.105769 TTTTATTTA:0.144231
TTTTATTTATTTA:0.0769231 TTTTATTTATTTATTTA:0.0448718

chr28 11672615 2 312 TTTA:0.820513 T:0.179487
chr28 11672855 2 316 GTAAT:0.759494 G:0.240506
chr28 11672983 2 310 GATTA:0.73871 G:0.26129
chr28 11674067 2 298 C:0.419463 A:0.580537
chr28 11674195 3 296 TA:0.412162 T:0.503378
TAA:0.0844595
chr28 11674415 2 310 T:0.948387 A:0.0516129
chr28 11674480 2 310 C:0.941935 A:0.0580645
chr28 11675099 4 278 TAA:0.431655 T:0.158273
TA:0.183453 TAAA:0.226619
chr28 11675118 2 284 GA:0.661972 G:0.338028
chr28 11675396 2 298 C:0.446309 CA:0.553691
chr28 11675414 2 296 G:0.945946 A:0.0540541
chr28 11675566 2 306 G:0.784314 A:0.215686
chr28 11675926 2 302 TA:0.450331 T:0.549669
chr28 11676070 2 304 A:0.9375 AT:0.0625
chr28 11676090 2 306 A:0.738562 T:0.261438
chr28 11676203 2 304 T:0.463816 TA:0.536184
chr28 11676290 2 302 G:0.761589 A:0.238411
chr28 11676352 2 298 C:0.439597 T:0.560403
chr28 11676692 2 306 TAAA:0.411765 T:0.588235
chr28 11676696 2 302 A:0.754967 T:0.245033
chr28 11676739 2 306 T:0.421569 C:0.578431
chr28 11676836 2 304 C:0.730263 G:0.269737
chr28 11676889 2 304 C:0.746711 T:0.253289
chr28 11677022 2 310 T:0.448387 TAGTC:0.551613
chr28 11677158 2 306 A:0.509804 AAG:0.490196
chr28 11677159 2 306 A:0.928105 AG:0.0718954
chr28 11677164 2 306 T:0.928105 A:0.0718954
chr28 11677170 2 306 A:0.928105 G:0.0718954
chr28 11677360 2 298 C:0.687919 CA:0.312081
chr28 11677631 3 304 TGAGA:0.575658 T:0.0822368
TGAGAGA:0.342105
chr28 11677662 2 296 C:0.804054 T:0.195946
chr28 11677675 2 292 C:0.472603 A:0.527397
chr28 11677838 2 304 C:0.414474 G:0.585526
chr28 11678159 3 298 C:0.496644 CT:0.261745
CCTT:0.241611
chr28 11678183 2 274 T:0.332117 C:0.667883
chr28 11678370 2 310 A:0.941935 G:0.0580645
chr28 11678849 2 298 T:0.936242 C:0.0637584
chr28 11678978 2 312 A:0.650641 AT:0.349359
chr28 11679100 2 310 T:0.932258 C:0.0677419
chr28 11679252 2 306 C:0.937908 A:0.0620915
chr28 11679465 2 292 C:0.5 T:0.5
chr28 11679592 2 286 A:0.965035 C:0.034965
chr28 11679828 2 240 TC:0.458333 T:0.541667
chr28 11680019 2 288 C:0.774306 T:0.225694
chr28 11680206 2 282 C:0.48227 G:0.51773
chr28 11680249 2 286 C:0.454545 T:0.545455
chr28 11680371 2 298 G:0.630872 A:0.369128
chr28 11680456 2 286 T:0.667832 C:0.332168
chr28 11680552 2 296 C:0 CA:1
chr28 11681024 2 296 G:0.655405 A:0.344595

chr28 11681265 2 306 T:0.696078 A:0.303922
chr28 11681314 2 302 C:0.801325 T:0.198675
chr28 11681488 2 314 CAGAAACTCGGATGG:0.958599 C:
0.0414013
chr28 11681515 2 300 G:0.976667 A:0.0233333
chr28 11681635 2 308 T:0.672078 G:0.327922
chr28 11681638 2 306 A:0.761438 ACAG:0.238562
chr28 11681822 2 308 T:0.38961 G:0.61039
chr28 11681960 2 306 C:0.676471 T:0.323529
chr28 11681964 2 304 C:0.953947 T:0.0460526
chr28 11682021 2 306 G:0.375817 A:0.624183
chr28 11682113 2 306 T:0.852941 C:0.147059
chr28 11682143 2 302 A:0.675497 G:0.324503
chr28 11682214 2 300 T:0.76 C:0.24
chr28 11682221 2 302 G:0.791391 A:0.208609
chr28 11682522 2 304 T:0.769737 C:0.230263
chr28 11682826 2 294 C:0.653061 A:0.346939
chr28 11682863 2 292 C:0.979452 T:0.0205479
chr28 11682929 2 314 T:0.684713 C:0.315287
chr28 11682955 2 312 C:0.692308 T:0.307692
chr28 11683463 2 302 A:0.986755 C:0.013245
chr28 11683516 2 286 TA:0.951049 T:0.048951
chr28 11683703 2 300 T:0.95 TA:0.05
chr28 11683704 3 304 T:0.641447 A:0.0756579
TA:0.282895
chr28 11683871 2 300 A:0.66 T:0.34
chr28 11683882 2 300 T:0.66 C:0.34
chr28 11683904 2 300 T:0.98 C:0.02
chr28 11683953 2 310 G:0.670968 A:0.329032
chr28 11684268 2 308 T:0.675325 A:0.324675
chr28 11684270 2 308 C:0.935065 CT:0.0649351
chr28 11684488 2 304 T:0.671053 C:0.328947
chr28 11684636 2 306 T:0.656863 C:0.343137
chr28 11684830 2 306 A:0.754902 T:0.245098
chr28 11684927 2 310 C:0.674194 G:0.325806
chr28 11685424 2 300 T:0.98 C:0.02
chr28 11685456 2 302 A:0.662252 G:0.337748
chr28 11685471 2 306 T:0.980392 C:0.0196078
chr28 11685838 2 308 C:0.931818 T:0.0681818
chr28 11686012 2 302 C:0.678808 A:0.321192
chr28 11686046 2 296 C:0.682432 T:0.317568
chr28 11686094 2 304 G:0.763158 T:0.236842
chr28 11686482 2 304 C:0.694079 A:0.305921
chr28 11686583 2 312 G:0.698718 C:0.301282
chr28 11686733 2 304 A:0.927632 T:0.0723684
chr28 11687334 2 298 A:0.42953 G:0.57047
chr28 11687417 2 310 C:0.945161 T:0.0548387
chr28 11688034 2 312 A:0.907051 C:0.0929487
chr28 11688227 2 312 T:0.810897 C:0.189103
chr28 11688665 2 312 A:0.86859 T:0.13141
chr28 11688672 2 312 T:0.86859 TCTC:0.13141
chr28 11688771 2 300 G:0.7 A:0.3
chr28 11688958 2 278 T:0.669065 TA:0.330935
chr28 11689308 2 310 T:0.790323 A:0.209677

chr28	11689682	2	308	G:0.899351	A:0.100649		
chr28	11690414	2	302	C:0.857616	T:0.142384		
chr28	11690489	2	296	T:0.972973	G:0.027027		
chr28	11690556	2	318	A:0.889937	ACAAAGTAGTGTCATC:0.110063		
chr28	11690879	2	168	TCC:0.809524	T:0.190476		
chr28	11690883	2	156	CCCCCA:0.711538	C:0.288462		
chr28	11690885	2	164	CCCA:0.79878	C:0.20122		
chr28	11690888	2	168	ACCC:0.809524	A:0.190476		
chr28	11690889	2	164	C:0.79878	G:0.20122		
chr28	11690977	2	270	T:0.67037	C:0.32963		
chr28	11691100	2	284	T:0.859155	C:0.140845		
chr28	11691179	2	288	T:0.972222	C:0.0277778		
chr28	11691215	2	276	C:0.786232	G:0.213768		
chr28	11691342	4	248	TAAA:0.584677	T:0.149194	TA:0.0967742	TAAAA:0.169355
chr28	11691381	3	232	C:0.224138	CT:0.577586	CTT:0.198276	
chr28	11691535	2	146	AAT:0.287671	A:0.712329		
chr28	11691644	2	174	A:0.172414	G:0.827586		
chr28	11691670	2	212	T:0.825472	C:0.174528		
chr28	11691825	2	274	A:0.781022	C:0.218978		
chr28	11691854	2	290	G:0.862069	C:0.137931		
chr28	11691905	2	296	C:0.918919	T:0.0810811		
chr28	11691985	2	314	T:0.984076	TA:0.0159236		
chr28	11691988	2	314	T:0.984076	TA:0.0159236		
chr28	11691989	2	314	T:0.942675	TA:0.0573248		
chr28	11691991	2	314	T:0.984076	TATTTATTTA:0.0159236		
chr28	11691992	2	314	T:0.942675	TATTTATTTA:0.0573248		
chr28	11691996	3	314	T:0.923567	C:0.0191083	TC:0.0573248	
chr28	11692094	2	312	T:0.926282	C:0.0737179		
chr28	11692109	2	312	G:0.926282	A:0.0737179		
chr28	11692111	2	312	T:0.926282	G:0.0737179		
chr28	11692146	2	300	T:0.8	C:0.2		
chr28	11692697	4	298	TAAA:0.40604	T:0.191275	TA:0.194631	TAA:0.208054
chr28	11692903	2	316	C:0.920886	T:0.0791139		
chr28	11692913	2	294	TGA:0.159864	T:0.840136		
chr28	11692931	2	316	A:0.962025	T:0.0379747		
chr28	11692946	2	312	AAGAG:0.935897	A:0.0641026		
chr28	11692990	2	302	G:0.907285	T:0.0927152		
chr28	11693149	2	304	C:0.980263	T:0.0197368		
chr28	11693163	2	300	G:0.92	A:0.08		
chr28	11693239	2	308	C:0.172078	T:0.827922		
chr28	11693250	2	306	G:0.905229	A:0.0947712		
chr28	11693427	2	308	T:0.915584	TAAATTCTAGCTAGAAAAAAA:0.0844156		
chr28	11693736	2	308	A:0.769481	G:0.230519		
chr28	11693880	2	310	A:0.922581	G:0.0774194		
chr28	11693898	2	314	C:0.863057	T:0.136943		
chr28	11694331	2	320	C:0.975	CAGGG:0.025		

chr28 11694333 2 320 T:0.975 TCC:0.025
chr28 11694335 2 320 T:0.975 TGGGTGGCGCAGCGGTTTGGCGCC:
0.025
chr28 11694776 2 292 A:0.928082 G:0.0719178
chr28 11694790 2 300 C:0.906667 T:0.0933333
chr28 11695051 2 308 G:0.925325 A:0.0746753
chr28 11695127 2 306 C:0.879085 T:0.120915
chr28 11695129 2 306 C:0.879085 T:0.120915
chr28 11695454 3 298 A:0.815436 AT:0.120805
ATT:0.0637584
chr28 11695464 2 298 A:0.201342 T:0.798658
chr28 11695499 2 278 TGAGA:0.302158 T:0.697842
chr28 11695895 2 300 G:0.82 T:0.18
chr28 11695928 2 294 A:0.846939 G:0.153061
chr28 11695971 2 290 C:0.851724 CA:0.148276
chr28 11696043 2 302 T:0.821192 C:0.178808
chr28 11696157 2 310 A:0.835484 T:0.164516
chr28 11696322 2 308 T:0.837662 C:0.162338
chr28 11696333 2 304 T:0.851974 C:0.148026
chr28 11696491 2 308 T:0.847403 G:0.152597
chr28 11696563 2 306 T:0.503268 C:0.496732
chr28 11696569 2 304 A:0.835526 C:0.164474
chr28 11696667 2 298 C:0.979866 T:0.0201342
chr28 11696696 2 298 G:0.912752 A:0.0872483
chr28 11696738 2 302 T:0.84106 C:0.15894
chr28 11696739 2 302 G:0.937086 A:0.0629139
chr28 11696799 2 306 G:0.98366 T:0.0163399
chr28 11696804 2 306 A:0.189542 G:0.810458
chr28 11696821 2 310 TC:0.9 T:0.1
chr28 11696879 4 298 TAA:0.503356 T:0.100671
TA:0.0704698 TAAA:0.325503
chr28 11696890 2 304 A:0.963816 G:0.0361842
chr28 11696987 2 296 C:0.868243 T:0.131757
chr28 11697054 2 308 T:0.873377 C:0.126623
chr28 11697164 2 308 T:0.873377 C:0.126623
chr28 11697601 2 306 T:0.862745 C:0.137255
chr28 11697656 2 310 G:0.864516 A:0.135484
chr28 11697697 2 302 C:0.877483 T:0.122517
chr28 11698030 2 312 C:0.855769 T:0.144231
chr28 11698157 2 308 A:0.857143 G:0.142857
chr28 11698169 2 306 C:0.859477 T:0.140523
chr28 11698275 2 304 T:0.292763 C:0.707237
chr28 11698653 2 306 TC:0.415033 T:0.584967
chr28 11698840 2 310 T:0.896774 C:0.103226
chr28 11698847 2 308 G:0.948052 T:0.0519481
chr28 11699712 2 308 A:0.876623 G:0.123377
chr28 11699795 2 304 C:0.973684 T:0.0263158
chr28 11700007 2 316 TTGAC:0.873418 T:0.126582
chr28 11700072 2 312 GC:0.384615 G:0.615385
chr28 11700076 2 312 A:0.384615 ACTGT:0.615385
chr28 11700159 2 314 T:0.359873 C:0.640127
chr28 11700274 2 304 T:0.194079 C:0.805921
chr28 11700395 2 306 A:0.852941 G:0.147059
chr28 11700691 2 300 A:0.726667 C:0.273333

chr28	11700988	2	282	A:0.351064	AAAG:0.648936		
chr28	11701131	2	310	C:0.370968	T:0.629032		
chr28	11701418	2	304	A:0.368421	G:0.631579		
chr28	11701430	2	298	CATG:0.996644	C:0.0033557		
chr28	11701535	2	300	A:0.373333	C:0.626667		
chr28	11701569	2	310	T:0.367742	G:0.632258		
chr28	11701671	2	304	A:0.766447	G:0.233553		
chr28	11701845	2	310	C:0.390323	A:0.609677		
chr28	11702069	2	306	A:0.395425	G:0.604575		
chr28	11702330	2	292	T:0.383562	C:0.616438		
chr28	11702460	2	310	G:0.4	A:0.6		
chr28	11702480	2	310	A:0.4	G:0.6		
chr28	11702530	2	298	T:0.872483	C:0.127517		
chr28	11702555	2	292	AT:0.808219	A:0.191781		
chr28	11702576	4	306	CAG:0.594771	C:0.212418	CAGAG:0.140523	CAGAGAGAG:0.0522876
chr28	11702775	2	306	A:0.921569	G:0.0784314		
chr28	11703062	2	306	C:0.754902	A:0.245098		
chr28	11703929	2	308	G:0.918831	T:0.0811688		
chr28	11703942	2	302	A:0.953642	C:0.0463576		
chr28	11703970	3	306	A:0.72549	ATG:0.235294	ATGTG:0.0392157	
chr28	11703996	4	318	G:0.77044	A:0.106918	GTGTA:0.072327	GTGTGTGTA:0.0503145
chr28	11703997	2	312	T:0.916667	TGTAA:0.0833333		
chr28	11703998	2	312	A:0.86859	ATATATG:0.13141		
chr28	11704093	2	310	AACTT:0.883871	A:0.116129		
chr28	11704168	2	308	T:0.425325	G:0.574675		
chr28	11704368	2	306	C:0.872549	T:0.127451		
chr28	11704375	2	306	C:0.918301	G:0.0816993		
chr28	11705080	2	318	ACCAAGCAGTG:0.792453	A:0.207547		
chr28	11705272	2	298	G:0.899329	A:0.100671		
chr28	11705417	2	276	T:0.15942	C:0.84058		
chr28	11705474	2	284	CCTCT:0.221831	C:0.778169		
chr28	11705581	2	300	G:0.91	GATTT:0.09		
chr28	11706162	2	308	C:0.944805	T:0.0551948		
chr28	11706172	2	304	G:0.976974	A:0.0230263		
chr28	11706272	2	304	T:0.322368	C:0.677632		
chr28	11706273	2	304	G:0.904605	A:0.0953947		
chr28	11706669	2	300	G:0.996667	A:0.00333333		
chr28	11706952	2	306	T:0.529412	G:0.470588		
chr28	11707335	2	296	T:0.537162	C:0.462838		
chr28	11707436	2	316	A:0.952532	ACTTCTT:0.0474684		
chr28	11707449	2	310	A:0.23871	C:0.76129		
chr28	11707452	2	312	A:0.814103	C:0.185897		
chr28	11707559	2	294	G:0.877551	A:0.122449		
chr28	11707984	2	300	G:0.92	A:0.08		
chr28	11708024	2	290	G:0.182759	C:0.817241		
chr28	11708418	2	294	G:0.870748	A:0.129252		
chr28	11708746	2	294	G:0.891156	A:0.108844		
chr28	11708955	2	306	C:0.960784	T:0.0392157		
chr28	11709127	4	304	CT:0.263158	C:0.299342	CTT:0.309211	CTTT:0.128289

chr28 11709326 2 310 C:0.603226 T:0.396774
chr28 11709496 2 304 G:0.789474 A:0.210526
chr28 11709734 2 304 C:0.654605 T:0.345395
chr28 11710319 2 316 A:0.943038 T:0.056962
chr28 11710511 2 304 G:0.684211 A:0.315789
chr28 11710516 2 304 A:0.684211 G:0.315789
chr28 11710780 2 300 C:0.95 CTT:0.05
chr28 11710832 3 314 CAA:0.194268 C:0.375796
CA:0.429936
chr28 11710891 2 310 GA:0.974194 G:0.0258065
chr28 11711092 2 294 G:0.602041 A:0.397959
chr28 11711396 2 314 G:0.585987 A:0.414013
chr28 11711437 2 304 T:0.868421 C:0.131579
chr28 11711629 2 310 G:0.996774 A:0.00322581
chr28 11711654 2 308 T:0.461039 C:0.538961
chr28 11712607 2 310 C:0.941935 T:0.0580645
chr28 11712708 2 300 T:0.196667 C:0.803333
chr28 11712782 2 306 T:0.19281 G:0.80719
chr28 11712968 2 302 G:0.794702 T:0.205298
chr28 11713049 2 300 C:0.186667 T:0.813333
chr28 11713254 2 300 T:0.846667 C:0.153333
chr28 11713386 2 300 G:0.176667 A:0.823333
chr28 11713631 2 306 G:0.803922 A:0.196078
chr28 11713777 2 308 A:0.993506 G:0.00649351
chr28 11713859 2 308 G:0.74026 A:0.25974
chr28 11713863 2 312 T:0.926282 C:0.0737179
chr28 11713956 2 306 C:0.921569 T:0.0784314
chr28 11714060 2 308 C:0.866883 T:0.133117
chr28 11714110 2 306 A:0.189542 G:0.810458
chr28 11714200 2 298 T:0.802013 A:0.197987
chr28 11714236 2 308 T:0.737013 C:0.262987
chr28 11714550 2 304 T:0.733553 A:0.266447
chr28 11714751 2 302 C:0.781457 T:0.218543
chr28 11715190 2 306 C:0.810458 T:0.189542
chr28 11715237 2 302 G:0.847682 A:0.152318
chr28 11715489 4 308 ATGTG:0.590909 A:0.136364
ATG:0.168831 ATGTGTG:0.103896
chr28 11715586 2 296 T:0.695946 G:0.304054
chr28 11715625 2 288 G:0.684028 GA:0.315972
chr28 11715826 2 302 A:0.980132 T:0.0198675
chr28 11715885 7 312 AACACACAC:0.11859 A:
0.144231 AAC:0.147436 AACAC:0.198718 AACACAC:0.185897
AACACACACAC:0.121795 AACACACACACAC:0.0833333
chr28 11716376 2 302 A:0.678808 G:0.321192
chr28 11716569 2 304 C:0.25 T:0.75
chr28 11716825 2 308 T:0.944805 C:0.0551948
chr28 11717109 2 312 C:0.951923 G:0.0480769
chr28 11717110 2 318 A:0.968553 ACTGTTT:0.0314465
chr28 11717112 2 318 A:0.968553 T:0.0314465
chr28 11717115 2 318 A:0.968553 G:0.0314465
chr28 11717116 2 318 T:0.968553 TGTGTTCTGTTTTA:
0.0314465
chr28 11717266 2 300 A:0.483333 G:0.516667
chr28 11717510 2 308 G:0.948052 A:0.0519481

chr28	11717633	2	304	A:0.671053	G:0.328947
chr28	11717756	2	306	G:0.993464	C:0.00653595
chr28	11717888	2	302	C:0.81457	T:0.18543
chr28	11718368	2	318	C:0.95283	T:0.0471698
chr28	11718390	2	318	C:0.955975	T:0.0440252
chr28	11718504	2	308	G:0.698052	C:0.301948
chr28	11718548	2	314	C:0.942675	T:0.0573248
chr28	11718549	2	314	C:0.805732	T:0.194268
chr28	11718550	2	318	C:0.704403	T:0.295597
chr28	11718551	2	320	G:0.5	A:0.5
chr28	11718565	2	320	G:0.5	A:0.5
chr28	11718678	2	308	A:0.454545	G:0.545455
chr28	11718698	2	306	C:0.944444	T:0.0555556
chr28	11718916	2	312	GA:0.810897	G:0.189103
chr28	11718940	2	316	T:0.702532	C:0.297468
chr28	11719139	2	316	G:0.547468	GTCCA:0.452532
chr28	11719152	2	316	C:0.481013	G:0.518987
chr28	11719208	2	312	G:0.698718	A:0.301282
chr28	11719240	2	312	G:0.942308	A:0.0576923
chr28	11719633	2	308	G:0.925325	A:0.0746753
chr28	11720081	2	310	T:0.945161	C:0.0548387
chr28	11720104	2	308	G:0.74026	A:0.25974
chr28	11720159	2	306	G:0.970588	T:0.0294118
chr28	11720191	2	298	A:0.486577	G:0.513423
chr28	11720353	2	316	C:0.474684	T:0.525316
chr28	11720469	2	298	G:0.463087	A:0.536913
chr28	11720544	2	314	G:0.942675	A:0.0573248
chr28	11720960	2	314	A:0.977707	T:0.022293
chr28	11720994	2	312	T:0.503205	C:0.496795
chr28	11721002	2	312	A:0.817308	G:0.182692
chr28	11721029	2	306	T:0.745098	A:0.254902
chr28	11721260	2	310	C:0.167742	A:0.832258
chr28	11721292	2	314	C:0.93949	G:0.0605096
chr28	11721367	2	312	T:0.948718	C:0.0512821
chr28	11721379	2	310	C:0.948387	T:0.0516129
chr28	11721484	2	306	G:0.19281	A:0.80719
chr28	11721567	2	304	T:0.1875	C:0.8125
chr28	11721850	2	308	G:0.694805	A:0.305195
chr28	11722003	2	300	G:0.843333	A:0.156667
chr28	11722131	2	296	G:0.712838	C:0.287162
chr28	11722288	2	298	C:0.194631	T:0.805369
chr28	11722477	2	304	A:0.855263	G:0.144737
chr28	11722478	2	304	G:0.855263	T:0.144737
chr28	11722493	2	308	C:0.944805	A:0.0551948
chr28	11722659	2	314	C:0.977707	T:0.022293
chr28	11722660	2	314	A:0.707006	G:0.292994
chr28	11722665	2	316	C:0.927215	A:0.0727848
chr28	11722748	2	314	TACTC:0.914013	T:0.0859873
chr28	11723119	2	296	G:0.939189	T:0.0608108
chr28	11723198	2	304	A:0.825658	G:0.174342
chr28	11723529	2	302	G:0.768212	A:0.231788
chr28	11723617	2	292	T:0.167808	C:0.832192
chr28	11723841	2	258	C:0.624031	G:0.375969
chr28	11724083	2	294	CA:0.938776	C:0.0612245

chr28	11724092	2	294	C:0.938776	T:0.0612245
chr28	11724129	2	310	A:0.945161	AGTAAT:0.0548387
chr28	11724174	2	308	T:0.733766	G:0.266234
chr28	11724300	2	314	G:0.38535	A:0.61465
chr28	11724624	2	308	A:0.957792	ACACGG:0.0422078
chr28	11724765	2	300	C:0.87 A:0.13	
chr28	11724983	2	310	G:0.370968	A:0.629032
chr28	11725122	2	300	A:0.86 C:0.14	
chr28	11725132	2	294	G:0.401361	GA:0.598639
chr28	11725179	2	314	A:0.363057	G:0.636943
chr28	11725348	2	308	G:0.733766	T:0.266234
chr28	11725375	2	310	A:0.941935	G:0.0580645
chr28	11725670	2	314	C:0.942675	G:0.0573248
chr28	11726126	2	306	G:0.392157	A:0.607843
chr28	11726129	2	308	A:0.941558	G:0.0584416
chr28	11726369	2	302	A:0.745033	C:0.254967
chr28	11726394	2	310	A:0.464516	C:0.535484
chr28	11726402	2	308	T:0.938312	C:0.0616883
chr28	11726408	2	308	G:0.396104	A:0.603896
chr28	11726499	2	314	G:0.719745	C:0.280255
chr28	11726540	2	304	G:0.700658	A:0.299342
chr28	11726628	2	310	C:0.725806	T:0.274194
chr28	11726945	2	312	T:0.945513	C:0.0544872
chr28	11726980	2	310	G:0.709677	A:0.290323
chr28	11727124	2	302	A:0.39404	AGTT:0.60596
chr28	11727419	2	308	A:0.198052	G:0.801948
chr28	11727682	2	306	G:0.74183	C:0.25817
chr28	11727699	2	306	G:0.74183	A:0.25817
chr28	11727738	2	290	A:0.393103	T:0.606897
chr28	11727777	2	296	G:0.864865	C:0.135135
chr28	11727890	2	310	CTTTAT:0.393548	C:0.606452
chr28	11727942	2	306	G:0.45098	C:0.54902
chr28	11728046	2	294	G:0.972789	A:0.0272109
chr28	11728195	2	316	G:0.987342	A:0.0126582
chr28	11728438	2	298	T:0.946309	C:0.0536913
chr28	11728532	2	298	T:0.869128	TA:0.130872
chr28	11728568	2	304	T:0.532895	C:0.467105
chr28	11728934	2	302	T:0.188742	C:0.811258
chr28	11728968	2	300	G:0.946667	A:0.0533333
chr28	11729059	2	302	A:0.884106	G:0.115894
chr28	11729062	2	300	AC:0.946667	A:0.0533333
chr28	11729189	2	302	A:0.195364	G:0.804636
chr28	11729208	2	284	A:0.778169	AT:0.221831
chr28	11729336	2	308	A:0.844156	T:0.155844
chr28	11729414	2	300	A:0.933333	AT:0.0666667
chr28	11729845	2	302	G:0.94702	GT:0.0529801
chr28	11729856	2	308	C:0.957792	T:0.0422078
chr28	11730457	2	310	T:0.787097	A:0.212903
chr28	11730557	2	300	C:0.593333	A:0.406667
chr28	11730558	2	300	A:0.593333	T:0.406667
chr28	11730678	2	314	G:0.904459	T:0.0955414
chr28	11731028	2	298	C:0.409396	T:0.590604
chr28	11731043	2	294	AT:0.72449	A:0.27551
chr28	11731203	2	296	A:0.939189	G:0.0608108

chr28	11731384	2	292	A:0.229452	C:0.770548	
chr28	11731464	2	308	T:0.88961	C:0.11039	
chr28	11731567	2	314	A:0.812102	G:0.187898	
chr28	11731671	2	300	T:0.996667	C:0.00333333	
chr28	11731708	2	304	G:0.773026	A:0.226974	
chr28	11731739	2	304	T:0.950658	TA:0.0493421	
chr28	11731740	2	304	G:0.950658	A:0.0493421	
chr28	11731743	2	306	G:0.849673	T:0.150327	
chr28	11732347	2	302	A:0.990066	C:0.00993377	
chr28	11732348	2	302	G:0.990066	T:0.00993377	
chr28	11732452	2	306	T:0.941176	C:0.0588235	
chr28	11732599	2	308	C:0.948052	T:0.0519481	
chr28	11732701	2	288	T:0.677083	C:0.322917	
chr28	11732767	2	304	G:0.953947	A:0.0460526	
chr28	11732838	2	306	C:0.676471	T:0.323529	
chr28	11732942	2	296	A:0.658784	T:0.341216	
chr28	11733290	2	312	G:0.961538	A:0.0384615	
chr28	11733329	2	312	A:0.147436	G:0.852564	
chr28	11733710	2	310	T:0.380645	C:0.619355	
chr28	11733715	2	310	T:0.945161	C:0.0548387	
chr28	11733775	2	304	T:0.401316	C:0.598684	
chr28	11734031	2	298	G:0.939597	A:0.0604027	
chr28	11734088	2	310	T:0.874194	G:0.125806	
chr28	11734722	2	302	G:0.996689	A:0.00331126	
chr28	11734753	2	302	T:0.791391	A:0.208609	
chr28	11735153	2	304	C:0.911184	T:0.0888158	
chr28	11735175	2	292	C:0.921233	CT:0.0787671	
chr28	11735301	2	300	T:0.7	C:0.3	
chr28	11735469	2	302	T:0.976821	G:0.0231788	
chr28	11735580	2	304	TC:0.9375	T:0.0625	
chr28	11735631	2	300	T:0.943333	G:0.0566667	
chr28	11735638	2	300	T:0.943333	A:0.0566667	
chr28	11735692	2	316	T:0.164557	A:0.835443	
chr28	11735701	2	316	TAATGTCACTAAGTTTTG:0.93038	T:0.0696203	
chr28	11735782	2	310	C:0.941935	T:0.0580645	
chr28	11735932	2	288	A:0.944444	C:0.0555556	
chr28	11736011	2	292	T:0.955479	G:0.0445205	
chr28	11736058	3	306	T:0.607843	TA:0.333333	TAA:0.0588235
chr28	11736124	2	312	G:0.939103	A:0.0608974	
chr28	11736154	2	308	G:0.970779	A:0.0292208	
chr28	11736271	2	306	A:0.973856	G:0.0261438	
chr28	11736327	2	306	T:0.954248	C:0.0457516	
chr28	11736399	2	304	T:0.4375	C:0.5625	
chr28	11736492	2	300	G:0.94	A:0.06	
chr28	11736637	2	308	T:0.944805	A:0.0551948	
chr28	11736659	2	304	A:0.940789	T:0.0592105	
chr28	11736793	2	300	A:0.713333	G:0.286667	
chr28	11736832	2	296	T:0.952703	G:0.0472973	
chr28	11736989	2	286	C:0.863636	A:0.136364	
chr28	11737047	2	298	G:0.171141	A:0.828859	
chr28	11737114	2	306	A:0.650327	G:0.349673	
chr28	11737167	2	306	A:0.405229	C:0.594771	

chr28	11737431	2	308	T:0.993506	G:0.00649351
chr28	11737474	2	308	C:0.711039	T:0.288961
chr28	11737555	2	296	C:0.945946	T:0.0540541
chr28	11737609	2	306	T:0.885621	G:0.114379
chr28	11737704	2	306	C:0.970588	T:0.0294118
chr28	11737793	2	304	A:0.960526	G:0.0394737
chr28	11737835	2	306	C:0.754902	T:0.245098
chr28	11737943	2	310	T:0.677419	C:0.322581
chr28	11737988	2	300	C:0.696667	A:0.303333
chr28	11738674	2	302	A:0.682119	G:0.317881
chr28	11738714	2	300	A:0.766667	G:0.233333
chr28	11738894	2	304	T:0.881579	C:0.118421
chr28	11738992	2	306	G:0.947712	C:0.0522876
chr28	11739053	2	312	C:0.951923	T:0.0480769
chr28	11739304	2	300	G:0.716667	A:0.283333
chr28	11739353	2	304	C:0.947368	CTG:0.0526316
chr28	11739355	4	306	C:0.650327	G:0.0588235
CTCTG:0.267974		CTGTG:0.0228758			
chr28	11739443	2	302	A:0.139073	G:0.860927
chr28	11739473	2	292	T:0.941781	C:0.0582192
chr28	11739475	2	292	G:0.941781	A:0.0582192
chr28	11739534	3	312	CCT:0.884615	C:0.0608974
CCTCTCT:0.0544872					
chr28	11739570	2	308	A:0.698052	G:0.301948
chr28	11739571	3	308	TAAAAATTAAAAAAAAACTTAAAAAA:0.50974	
	T:0.431818		TAAAATTAAAAAAAAACTTAAAAAA:0.0584416		
chr28	11739579	2	308	AAAAAAAAACTTAAAAAAAAAAATT:0.642857	
	A:0.357143				
chr28	11739721	2	288	A:0.9375	AAG:0.0625
chr28	11739753	2	288	G:0.885417	A:0.114583
chr28	11739762	2	288	A:0.690972	T:0.309028
chr28	11739763	2	288	G:0.690972	A:0.309028
chr28	11739831	2	310	T:0.683871	C:0.316129
chr28	11739926	2	304	G:0.611842	T:0.388158
chr28	11740697	2	306	A:0.598039	C:0.401961
chr28	11740934	2	310	T:0.887097	A:0.112903
chr28	11741024	2	298	C:0.932886	T:0.0671141
chr28	11741241	2	304	C:0.996711	T:0.00328947
chr28	11742019	2	302	G:0.718543	A:0.281457
chr28	11742044	2	298	T:0.389262	G:0.610738
chr28	11742213	2	308	T:0.704545	C:0.295455
chr28	11742283	2	298	C:0.939597	T:0.0604027
chr28	11742362	2	308	T:0.993506	C:0.00649351
chr28	11742433	2	302	C:0.678808	G:0.321192
chr28	11742883	2	308	G:0.931818	A:0.0681818
chr28	11743015	2	302	A:0.970199	C:0.0298013
chr28	11743065	2	310	G:0.474194	A:0.525806
chr28	11743074	2	312	C:0.945513	T:0.0544872
chr28	11743156	2	300	C:0.8	T:0.2
chr28	11743195	2	300	A:0.95	C:0.05
chr28	11743291	2	308	G:0.438312	A:0.561688
chr28	11743363	2	302	T:0.943709	C:0.0562914
chr28	11743412	2	302	T:0.754967	C:0.245033
chr28	11743534	2	304	C:0.476974	T:0.523026

chr28	11743595	2	294	T:0.469388	C:0.530612
chr28	11743633	2	294	C:0.717687	T:0.282313
chr28	11743739	2	312	C:0.467949	T:0.532051
chr28	11743856	2	298	C:0.473154	T:0.526846
chr28	11743871	2	296	C:0.135135	G:0.864865
chr28	11744902	2	294	T:0.982993	C:0.0170068
chr28	11745129	2	296	G:0.945946	A:0.0540541
chr28	11745150	2	296	C:0.706081	T:0.293919
chr28	11745153	2	296	G:0.486486	A:0.513514
chr28	11745162	2	296	A:0.989865	C:0.0101351
chr28	11745296	2	312	G:0.423077	A:0.576923
chr28	11745342	2	302	C:0.927152	CT:0.0728477
chr28	11745693	2	310	A:0.725806	T:0.274194
chr28	11745711	2	306	G:0.424837	A:0.575163
chr28	11745848	2	288	A:0.4375	C:0.5625
chr28	11745895	2	304	C:0.75	G:0.25
chr28	11746260	2	302	C:0.721854	A:0.278146
chr28	11746288	2	304	A:0.450658	ATCT:0.549342
chr28	11746398	2	296	C:0.472973	T:0.527027
chr28	11746450	2	302	G:0.463576	A:0.536424
chr28	11746455	2	302	C:0.463576	T:0.536424
chr28	11746530	2	296	A:0.405405	G:0.594595
chr28	11746608	2	298	C:0.949664	T:0.0503356
chr28	11746624	2	304	T:0.434211	C:0.565789
chr28	11746672	2	302	T:0.943709	C:0.0562914
chr28	11746701	2	304	A:0.486842	G:0.513158
chr28	11746769	2	306	T:0.931373	TAGAC:0.0686275
chr28	11746774	2	306	A:0.509804	C:0.490196
chr28	11746779	2	308	T:0.438312	C:0.561688
chr28	11746815	2	302	G:0.937086	GC:0.0629139
chr28	11746863	2	302	T:0.347682	G:0.652318
chr28	11747207	2	296	T:0.972973	C:0.027027
chr28	11747361	2	310	T:0.503226	C:0.496774
chr28	11747383	2	302	C:0.5	T:0.5
chr28	11747404	2	306	A:0.493464	C:0.506536
chr28	11747492	2	296	T:0.787162	C:0.212838
chr28	11747632	2	310	G:0.935484	T:0.0645161
chr28	11747678	2	314	A:0.936306	G:0.0636943
chr28	11747686	2	314	C:0.974522	T:0.0254777
chr28	11747705	2	312	G:0.939103	A:0.0608974
chr28	11747918	2	308	C:0.743506	T:0.256494
chr28	11748026	2	300	A:0.936667	G:0.0633333
chr28	11748041	2	302	T:0.84106	C:0.15894
chr28	11748103	2	308	C:0.931818	A:0.0681818
chr28	11748179	2	320	G:0.93125	A:0.06875
chr28	11748287	2	300	T:0.896667	A:0.103333
chr28	11748536	2	304	A:0.9375	C:0.0625
chr28	11748543	2	308	TA:0.896104	T:0.103896
chr28	11748559	2	308	G:0.896104	T:0.103896
chr28	11748669	2	308	A:0.941558	T:0.0584416
chr28	11748694	2	308	C:0.941558	T:0.0584416
chr28	11748695	2	302	T:0.837748	A:0.162252
chr28	11748760	2	314	G:0.94586	T:0.0541401
chr28	11748768	2	314	A:0.94586	T:0.0541401

chr28	11748817	2	304	C:0.904605	T:0.0953947
chr28	11748826	2	306	T:0.931373	C:0.0686275
chr28	11749230	2	304	TC:0.940789	T:0.0592105
chr28	11749346	2	302	A:0.162252	T:0.837748
chr28	11749371	2	306	T:0.859477	TGTC:0.140523
chr28	11749403	2	306	T:0.846405	C:0.153595
chr28	11749624	2	304	AT:0.9375	A:0.0625
chr28	11749767	2	310	C:0.896774	T:0.103226
chr28	11750075	2	300	G:0.846667	T:0.153333
chr28	11750108	2	304	C:0.884868	T:0.115132
chr28	11750118	2	304	G:0.884868	A:0.115132
chr28	11750298	2	308	A:0.944805	T:0.0551948
chr28	11750397	2	304	G:0.835526	T:0.164474
chr28	11750477	2	308	A:0.87013	C:0.12987
chr28	11750496	2	298	T:0.862416	G:0.137584
chr28	11750714	2	300	C:0.943333	T:0.0566667
chr28	11750793	2	300	T:0.946667	G:0.0533333
chr28	11751106	2	308	C:0.961039	T:0.038961
chr28	11751221	2	310	AACTC:0.887097	A:0.112903
chr28	11751359	2	304	C:0.822368	T:0.177632
chr28	11751412	2	312	A:0.833333	G:0.166667
chr28	11751632	2	302	C:0.738411	G:0.261589
chr28	11751640	2	304	C:0.993421	A:0.00657895
chr28	11751707	2	298	G:0.852349	C:0.147651
chr28	11751732	2	302	A:0.910596	G:0.089404
chr28	11751741	2	302	A:0.943709	G:0.0562914
chr28	11751847	2	284	C:0.926056	CT:0.0739437
chr28	11751848	2	286	T:0.912587	C:0.0874126
chr28	11752068	2	296	G:0.847973	A:0.152027
chr28	11752096	2	302	T:0.837748	A:0.162252
chr28	11752211	2	296	G:0.952703	A:0.0472973
chr28	11752340	2	310	A:0.845161	G:0.154839
chr28	11752566	2	308	TGTTTTTTG:0.964286	T:0.0357143
chr28	11752614	2	298	ATTTATTT:0.47651	A:0.52349
chr28	11752954	2	292	T:0.938356	C:0.0616438
chr28	11752959	2	282	T:0	TA:1
chr28	11752992	2	302	T:0	C:1
chr28	11752993	2	302	G:0	T:1
chr28	11752996	2	302	TA:0	T:1
chr28	11753001	2	302	A:0	AT:1
chr28	11753011	2	302	C:0	T:1
chr28	11753023	2	302	C:0	CT:1
chr28	11753061	2	306	T:0	C:1
chr28	11753063	2	306	G:0	T:1
chr28	11753067	2	306	A:0	AT:1
chr28	11753073	2	306	C:0	CCTAA:1
chr28	11753075	2	306	G:0	A:1
chr28	11753077	2	306	ATAT:0	A:1
chr28	11753083	2	306	G:0	GT:1
chr28	11753088	2	306	ATT:0	A:1
chr28	11753192	2	298	C:0.875839	T:0.124161
chr28	11753196	2	298	T:0.966443	C:0.033557
chr28	11753328	2	308	G:0.162338	A:0.837662

chr28	11753366	2	306	C:0.892157	G:0.107843
chr28	11753433	2	312	T:0.298077	G:0.701923
chr28	11753690	2	310	C:0.935484	T:0.0645161
chr28	11753833	2	310	A:0.851613	G:0.148387
chr28	11753862	2	314	TTA:0.850318	T:0.149682
chr28	11753866	2	314	T:0.850318	TCA:0.149682
chr28	11753971	2	320	T:0.865625	A:0.134375
chr28	11753989	2	320	C:0.865625	T:0.134375
chr28	11754013	2	320	G:0.865625	A:0.134375
chr28	11754054	2	306	C:0.96732	T:0.0326797
chr28	11754316	2	312	C:0.961538	T:0.0384615
chr28	11754371	2	310	T:0.912903	G:0.0870968
chr28	11754373	2	312	C:0.147436	T:0.852564
chr28	11754464	2	304	A:0.996711	G:0.00328947
chr28	11754469	2	304	G:0.898026	A:0.101974
chr28	11754654	2	308	G:0.886364	A:0.113636
chr28	11754906	2	280	A:0.896429	G:0.103571
chr28	11755001	2	154	G:0.116883	A:0.883117
chr28	11755008	2	148	G:0.655405	A:0.344595
chr28	11755023	2	148	AG:0.655405	A:0.344595
chr28	11755177	2	274	A:0.182482	C:0.817518
chr28	11755253	2	276	C:0.873188	A:0.126812
chr28	11755303	2	298	T:0.875839	C:0.124161
chr28	11755430	2	312	T:0.891026	TTTGTA:0.108974
chr28	11755523	2	304	AT:0.904605	A:0.0953947
chr28	11755599	2	312	A:0.961538	G:0.0384615
chr28	11755688	2	308	G:0.902597	A:0.0974026
chr28	11755801	2	312	T:0.958333	C:0.0416667
chr28	11755835	2	316	TCAGTCCC:0.901899	T:0.0981013
chr28	11755867	2	316	G:0.901899	C:0.0981013
chr28	11755935	2	312	C:0.903846	T:0.0961538
chr28	11756119	2	300	T:0.893333	C:0.106667
chr28	11756130	2	298	T:0.85906	C:0.14094
chr28	11756251	2	302	C:0.897351	T:0.102649
chr28	11756326	2	304	C:0.878289	G:0.121711
chr28	11756376	2	306	G:0.915033	A:0.0849673
chr28	11756482	2	306	C:0.898693	CT:0.101307
chr28	11756520	2	304	T:0.967105	C:0.0328947
chr28	11756606	2	304	T:0.858553	C:0.141447
chr28	11756788	2	306	C:0.957516	A:0.0424837
chr28	11756847	2	304	A:0.970395	G:0.0296053
chr28	11756853	2	302	A:0.86755	G:0.13245
chr28	11756973	2	310	G:0.893548	T:0.106452
chr28	11757038	2	310	A:0.958065	G:0.0419355
chr28	11757176	2	316	T:0.968354	C:0.0316456
chr28	11757276	2	310	A:0.964516	C:0.0354839
chr28	11757417	2	298	TG:0.899329	T:0.100671
chr28	11757428	2	294	G:0.867347	A:0.132653
chr28	11757535	2	312	T:0.852564	C:0.147436
chr28	11757544	2	312	G:0.852564	A:0.147436
chr28	11757665	2	306	G:0.977124	T:0.0228758
chr28	11757782	2	306	G:0.937908	A:0.0620915
chr28	11757820	2	308	C:0.993506	T:0.00649351

chr28 11757837 2 310 T:0.864516 A:0.135484
chr28 11757933 2 314 C:0.894904 T:0.105096
chr28 11757943 2 316 A:0.993671 G:0.00632911
chr28 11758254 2 300 C:0.87 T:0.13
chr28 11758292 2 306 TA:0.895425 T:0.104575
chr28 11758421 2 276 A:0.873188 G:0.126812
chr28 11758542 7 312 TTATATATA:0.246795 T:
0.137821 TTA:0.0576923 TTATA:0.0480769 TTATATA:0.282051
TTATATATATA:0.0865385 TTATATATATATA:0.141026
chr28 11758583 2 288 G:0.913194 A:0.0868056
chr28 11758654 2 272 A:0.922794 G:0.0772059
chr28 11758731 2 286 AC:0.951049 A:0.048951
chr28 11758736 2 286 A:0.951049 AG:0.048951
chr28 11758846 2 274 AC:0.985401 A:0.0145985
chr28 11759155 2 264 A:0.681818 G:0.318182
chr28 11759257 2 256 AG:0.820312 A:0.179688
chr28 11759580 2 300 G:0.976667 GTA:0.0233333
chr28 11759626 2 312 T:0.964744 TGTA:0.0352564
chr28 11759633 3 318 T:0.899371 TAGATTAGA:
0.0377358 TAGATTAGATAGATAGATAGATAGA:0.0628931
chr28 11759650 2 318 T:0.990566 TAG:0.00943396
chr28 11759652 2 318 T:0.990566 TAGA:0.00943396
chr28 11759654 2 318 G:0.990566 A:0.00943396
chr28 11759656 2 318 C:0.990566 A:0.00943396
chr28 11759659 2 318 T:0.990566 G:0.00943396
chr28 11759663 2 318 T:0.990566 G:0.00943396
chr28 11759666 2 318 A:0.990566 ATTGG:0.00943396
chr28 11759678 2 318 CATTG:0.990566 C:0.00943396
chr28 11759687 2 318 TATGTG:0.990566 T:0.00943396
chr28 11759696 2 318 G:0.883648 A:0.116352
chr28 11759783 2 302 T:0.89404 C:0.10596
chr28 11759800 2 306 C:0.964052 T:0.0359477
chr28 11759894 2 308 AT:0.857143 A:0.142857
chr28 11759944 2 312 T:0.961538 C:0.0384615
chr28 11760019 2 310 C:0.951613 CA:0.0483871
chr28 11760300 2 314 C:0.971338 T:0.0286624
chr28 11760339 2 306 A:0.908497 G:0.0915033
chr28 11760449 2 312 C:0.939103 T:0.0608974
chr28 11760463 2 312 C:0.939103 T:0.0608974
chr28 11760472 2 314 A:0.735669 T:0.264331
chr28 11760557 2 316 T:0.863924 C:0.136076
chr28 11760585 2 316 A:0.936709 G:0.0632911
chr28 11760624 2 318 T:0.971698 C:0.0283019
chr28 11760663 2 316 C:0.984177 CTTTT:0.0158228
chr28 11760665 2 316 T:0.946203 TTTC:0.0537975
chr28 11760699 3 304 C:0.865132 G:0.0493421
CAG:0.0855263
chr28 11760752 2 302 C:0.996689 T:0.00331126
chr28 11760779 2 300 C:0.91 A:0.09
chr28 11760781 2 300 C:0.996667 T:0.00333333
chr28 11760813 2 296 C:0.966216 T:0.0337838
chr28 11760906 2 306 A:0.872549 G:0.127451
chr28 11760940 2 312 C:0.897436 T:0.102564
chr28 11761008 2 302 C:0.963576 T:0.0364238

chr28 11761240 2 304 G:0.891447 A:0.108553
chr28 11761340 2 304 A:0.832237 G:0.167763
chr28 11761480 2 308 C:0.974026 T:0.025974
chr28 11761669 2 306 C:0.96732 A:0.0326797
chr28 11761738 2 316 T:0.851266 C:0.148734
chr28 11761758 2 320 T:0.99375
TAATAATCATTTTAAAA:0.00625
chr28 11761798 2 316 CAT:0.96519 C:0.0348101
chr28 11762033 2 312 C:0.958333 T:0.0416667
chr28 11762488 2 306 G:0.973856 A:0.0261438
chr28 11762505 2 302 A:0.966887 G:0.0331126
chr28 11762679 2 310 CCA:0.958065 C:0.0419355
chr28 11762724 2 300 A:0.96 G:0.04
chr28 11762764 4 318 TACACACACACAC:0.493711 T:
0.292453 TACACACACACACAC:0.113208 TACACACACACACACAC:0.100629
chr28 11762802 2 318 C:0.965409 T:0.0345912
chr28 11762821 2 298 G:0.90604 A:0.0939597
chr28 11762873 2 306 G:0.977124 A:0.0228758
chr28 11763027 2 304 A:0.855263 ACT:0.144737
chr28 11763144 2 298 A:0.969799 C:0.0302013
chr28 11763213 2 314 A:0.894904 C:0.105096
chr28 11763216 2 312 T:0.974359 A:0.025641
chr28 11763384 2 296 G:0.969595 T:0.0304054
chr28 11763486 2 300 T:0.96 A:0.04
chr28 11763540 2 304 C:0.161184 A:0.838816
chr28 11763601 2 302 C:0.86755 G:0.13245
chr28 11763663 2 308 TTTC:0.75974 T:0.24026
chr28 11763713 2 298 G:0.302013 C:0.697987
chr28 11763777 2 294 G:0.962585 A:0.037415
chr28 11764044 2 300 A:0.956667 G:0.0433333
chr28 11764060 2 298 T:0.855705 G:0.144295
chr28 11764519 2 286 T:0.982517 C:0.0174825
chr28 11764552 2 298 G:0.983221 A:0.0167785
chr28 11764590 2 264 TG:0.30303 T:0.69697
chr28 11764592 2 282 GTTT:0.964539 G:0.035461
chr28 11764890 2 306 T:0.964052 TAAG:0.0359477
chr28 11765223 2 310 AATC:0.767742 A:0.232258
chr28 11765369 2 306 C:0.964052 A:0.0359477
chr28 11765425 2 310 C:0.870968 T:0.129032
chr28 11765464 2 320 A:0.971875
ATTATTTTTTATTTTATTATTT:0.028125
chr28 11765465 2 320 C:0.971875
CATTAAATTATTTTAAT:0.028125
chr28 11765466 2 320 C:0.971875 T:0.028125
chr28 11765518 2 306 T:0.970588 C:0.0294118
chr28 11765627 2 304 C:0.970395 T:0.0296053
chr28 11765879 2 294 G:0.714286 T:0.285714
chr28 11766056 2 296 A:0.966216 C:0.0337838
chr28 11766099 2 304 A:0.282895 C:0.717105
chr28 11766106 2 304 T:0.963816 G:0.0361842
chr28 11766189 2 300 A:0.963333 T:0.0366667
chr28 11766254 2 292 A:0.284247 G:0.715753
chr28 11766328 4 316 AAGAGAGAG:0.177215 A:
0.281646 AAGAGAG:0.503165 AAGAGAGAGAGAGAG:0.0379747

chr28	11766490	2	314	CTTTTTTTTTTTT:0.372611	C:0.627389
chr28	11766584	2	294	C:0.918367	T:0.0816327
chr28	11766717	2	300	A:0.966667	G:0.0333333
chr28	11767154	2	308	G:0.931818	T:0.0681818
chr28	11767267	2	296	G:0.972973	A:0.027027
chr28	11767311	2	290	A:0.289655	G:0.710345
chr28	11767397	2	304	C:0.957237	T:0.0427632
chr28	11767479	2	308	A:0.967532	G:0.0324675
chr28	11767489	2	304	G:0.138158	A:0.861842
chr28	11767576	2	280	A:0.339286	T:0.660714
chr28	11767711	2	308	T:0.928571	C:0.0714286
chr28	11767900	2	312	T:0.900641	C:0.099359
chr28	11768021	2	310	G:0.180645	GGAAA:0.819355
chr28	11768093	2	302	T:0.960265	C:0.0397351
chr28	11768261	2	298	CA:0.16443	C:0.83557
chr28	11768466	2	298	C:0.969799	G:0.0302013
chr28	11768605	2	308	G:0.970779	A:0.0292208
chr28	11768624	2	302	A:0.847682	G:0.152318
chr28	11768768	2	294	T:0.979592	C:0.0204082
chr28	11768919	2	310	T:0.164516	A:0.835484
chr28	11768996	2	302	A:0.937086	G:0.0629139
chr28	11769101	2	306	C:0.928105	CTA:0.0718954
chr28	11769122	2	306	A:0.96732	C:0.0326797
chr28	11769341	2	306	C:0.980392	A:0.0196078
chr28	11769666	2	310	TGGCTTTCCCAAGTAA:0.980645	T:0.0193548
chr28	11769689	2	310	C:0.883871	A:0.116129
chr28	11769865	2	310	A:0.322581	G:0.677419
chr28	11770022	2	298	G:0.959732	C:0.0402685
chr28	11770722	2	302	A:0.162252	G:0.837748
chr28	11770739	2	304	TG:0.898026	T:0.101974
chr28	11771238	2	314	C:0.968153	CTTA:0.0318471
chr28	11771240	2	314	T:0.181529	TATG:0.818471
chr28	11771308	2	310	T:0.970968	C:0.0290323
chr28	11771840	2	304	C:0.904605	A:0.0953947
chr28	11772429	2	300	G:0.746667	C:0.253333
chr28	11772502	2	302	T:0.933775	G:0.0662252
chr28	11772564	2	302	T:0.15894	C:0.84106
chr28	11772595	2	302	AT:0.55298	A:0.44702
chr28	11772603	2	304	T:0.990132	A:0.00986842
chr28	11772715	2	302	C:0.589404	A:0.410596
chr28	11773090	2	296	A:0.594595	C:0.405405
chr28	11773354	2	310	G:0.593548	A:0.406452
chr28	11773427	2	304	A:0.9375	G:0.0625
chr28	11774005	2	306	A:0.562092	C:0.437908
chr28	11774216	2	308	C:0.987013	T:0.012987
chr28	11774403	2	296	A:0.584459	G:0.415541
chr28	11774797	2	308	A:0.581169	T:0.418831
chr28	11774834	2	310	G:0.5	A:0.5
chr28	11775084	2	302	A:0.572848	G:0.427152
chr28	11775145	2	300	C:0.753333	T:0.246667
chr28	11775177	2	294	GAATT:0.979592	G:0.0204082
chr28	11775270	2	286	A:0.982517	G:0.0174825

chr28	11775358	2	294	C:0.571429	T:0.428571		
chr28	11775428	2	302	A:0.556291	G:0.443709		
chr28	11775447	2	302	G:0.556291	T:0.443709		
chr28	11775501	2	296	A:0.959459	G:0.0405405		
chr28	11775605	2	310	G:0.577419	T:0.422581		
chr28	11775627	2	310	T:0.577419	C:0.422581		
chr28	11775871	2	306	A:0.584967	AC:0.415033		
chr28	11775945	2	314	A:0.576433	G:0.423567		
chr28	11775964	2	314	A:0.576433	T:0.423567		
chr28	11776095	2	318	A:0.962264	ATT:0.0377358		
chr28	11776097	2	318	T:0.962264	TTTAAAAG:0.0377358		
chr28	11776100	2	318	A:0.962264	AGTGATT:0.0377358		
chr28	11776103	2	318	A:0.962264	AAAGACAGTG:0.0377358		
chr28	11776147	2	300	C:0.583333	T:0.416667		
chr28	11776163	2	306	G:0.591503	A:0.408497		
chr28	11776184	2	304	G:0.766447	A:0.233553		
chr28	11776207	2	310	C:0.583871	G:0.416129		
chr28	11776297	2	306	T:0.584967	C:0.415033		
chr28	11776433	2	308	T:0.568182	C:0.431818		
chr28	11776447	2	302	A:0.559603	G:0.440397		
chr28	11777017	2	302	C:0.44702	T:0.55298		
chr28	11777084	2	306	A:0.767974	G:0.232026		
chr28	11777250	2	290	G:0.458621	A:0.541379		
chr28	11777374	2	312	C:0.464744	T:0.535256		
chr28	11777375	2	312	A:0.464744	G:0.535256		
chr28	11777393	2	312	GTCGAGGAATGTTCCCTCTATC:0.464744	G:0.535256		
chr28	11777488	2	294	G:0.465986	A:0.534014		
chr28	11777788	2	304	C:0.980263	G:0.0197368		
chr28	11777938	2	290	C:0.482759	T:0.517241		
chr28	11777976	2	278	TA:0.298561	T:0.701439		
chr28	11778017	2	290	GC:0.962069	G:0.037931		
chr28	11778027	2	296	C:0.976351	CAA:0.0236486		
chr28	11778028	3	306	C:0.251634	A:0.666667	CAA:0.0816993	
chr28	11778091	4	316	CATTTTATTTTATTTTATTTT:0.607595	C:0.0474684	CATTTTATTTT:0.14557	CATTTTATTTTATTTT:0.199367
chr28	11778217	2	284	C:0.771127	T:0.228873		
chr28	11778246	2	278	C:0.471223	T:0.528777		
chr28	11778341	2	298	A:0.983221	G:0.0167785		
chr28	11778413	2	308	A:0.974026	G:0.025974		
chr28	11778532	2	300	G:0.986667	A:0.0133333		
chr28	11778575	2	294	A:0.969388	C:0.0306122		
chr28	11778602	2	290	T:0.503448	G:0.496552		
chr28	11778783	2	300	C:0.98	T:0.02		
chr28	11778821	2	282	GCC:0.198582	G:0.801418		
chr28	11778825	2	286	C:0.975524	T:0.0244755		
chr28	11779002	2	292	CATA:0.986301	C:0.0136986		
chr28	11779171	2	300	G:0.913333	A:0.0866667		
chr28	11779249	2	292	G:0.489726	GAA:0.510274		
chr28	11779250	3	294	G:0.159864	A:0.55102	GA:0.289116	

chr28	11779353	2	312	C:0.961538	T:0.0384615		
chr28	11779410	2	312	T:0.785256	C:0.214744		
chr28	11779573	2	304	C:0.4375	T:0.5625		
chr28	11779590	2	302	C:0.602649	T:0.397351		
chr28	11780213	2	314	C:0.738854	T:0.261146		
chr28	11780473	2	312	C:0.801282	T:0.198718		
chr28	11780645	2	304	A:0.743421	G:0.256579		
chr28	11780700	2	308	A:0.756494	T:0.243506		
chr28	11780841	2	306	C:0.732026	T:0.267974		
chr28	11780901	2	310	G:0.729032	A:0.270968		
chr28	11781118	2	298	C:0.711409	A:0.288591		
chr28	11781195	2	300	A:0.276667	C:0.723333		
chr28	11781246	2	300	T:0.73	G:0.27		
chr28	11781633	2	310	G:0.158065	A:0.841935		
chr28	11781671	2	308	T:0.941558	C:0.0584416		
chr28	11781690	2	308	A:0.730519	AT:0.269481		
chr28	11782027	2	306	G:0.977124	C:0.0228758		
chr28	11782187	2	298	A:0.714765	T:0.285235		
chr28	11782207	2	298	A:0.728188	G:0.271812		
chr28	11782276	2	300	G:0.703333	A:0.296667		
chr28	11782293	2	300	G:0.85	A:0.15		
chr28	11782738	2	316	T:0.696203	TGGGTCCCTGGGCTC:0.303797		
chr28	11782739	2	316	AC:0.696203	A:0.303797		
chr28	11782818	2	298	A:0.993289	G:0.00671141		
chr28	11782888	2	306	T:0.718954	A:0.281046		
chr28	11783074	2	288	C:0.791667	CCT:0.208333		
chr28	11783132	3	310	A:0.532258	AAC:0.441935	AACAC:0.0258065	
chr28	11783381	2	298	A:0.832215	C:0.167785		
chr28	11783394	2	306	G:0.95098	A:0.0490196		
chr28	11783522	2	308	A:0.133117	C:0.866883		
chr28	11783544	2	304	A:0.210526	G:0.789474		
chr28	11783653	2	306	C:0.944444	T:0.0555556		
chr28	11783726	2	302	T:0.738411	G:0.261589		
chr28	11783784	2	308	G:0.980519	A:0.0194805		
chr28	11783812	4	308	T:0.594156	TG:0.201299	TGGC:0.0487013	TGGCG:0.155844
chr28	11783861	2	302	AT:0.546358	A:0.453642		
chr28	11784019	2	312	G:0.50641	A:0.49359		
chr28	11784285	2	302	T:0.725166	A:0.274834		
chr28	11784421	2	312	A:0.897436	T:0.102564		
chr28	11784430	2	306	AT:0.836601	A:0.163399		
chr28	11784451	2	310	T:0.170968	G:0.829032		
chr28	11784754	2	304	G:0.907895	A:0.0921053		
chr28	11784868	2	296	G:0.908784	A:0.0912162		
chr28	11785165	2	304	C:0.898026	T:0.101974		
chr28	11785291	2	310	A:0.903226	C:0.0967742		
chr28	11785317	2	304	C:0.825658	CT:0.174342		
chr28	11785352	2	312	G:0.737179	A:0.262821		
chr28	11785677	2	312	G:0.823718	T:0.176282		
chr28	11786110	2	304	C:0.970395	T:0.0296053		
chr28	11786350	2	306	A:0.748366	G:0.251634		
chr28	11786469	2	302	C:0.89404	A:0.10596		

chr28 11786795 2 308 T:0.788961 C:0.211039
chr28 11787108 3 304 C:0.605263 CT:0.332237
CTT:0.0625
chr28 11787247 2 310 G:0.764516 A:0.235484
chr28 11787410 2 312 T:0.826923 C:0.173077
chr28 11787613 2 302 T:0.887417 G:0.112583
chr28 11787775 2 306 A:0.490196 T:0.509804
chr28 11787811 2 312 AT:0.753205 A:0.246795
chr28 11787840 2 306 C:0.787582 T:0.212418
chr28 11788062 4 304 CCT:0.523026 C:0.0526316
CCTCT:0.355263 CCTCTCT:0.0690789
chr28 11788087 3 306 C:0.843137 CTG:0.133987
CTCTG:0.0228758
chr28 11788253 2 290 G:0.758621 A:0.241379
chr28 11788301 2 282 A:0.971631 G:0.0283688
chr28 11788394 2 310 G:0.96129 A:0.0387097
chr28 11788490 2 296 C:0.753378 G:0.246622
chr28 11788587 2 306 A:0.464052 G:0.535948
chr28 11788962 2 304 CACA:0.963816 C:0.0361842
chr28 11788964 2 316 C:0.920886 T:0.0791139
chr28 11788967 7 318 AAAAT:0.437107 A:0.0408805
TAAAT:0.138365 AAAATAAAT:0.166667 AAAATAAATAAAT:0.0849057
ATAAAT:0.081761 AAAATAAATAAATAAAT:0.0503145
chr28 11789007 4 318 T:0.704403 A:0.106918
TAAAA:0.106918 TAAATAAAA:0.081761
chr28 11789017 2 302 T:0.761589 A:0.238411
chr28 11789228 2 304 CAG:0.805921 C:0.194079
chr28 11789273 2 310 G:0.751613 A:0.248387
chr28 11789401 2 312 C:0.923077 CA:0.0769231
chr28 11789780 2 304 C:0.957237 T:0.0427632
chr28 11789807 2 302 C:0.572848 T:0.427152
chr28 11789860 2 296 A:0.564189 T:0.435811
chr28 11789949 2 312 G:0.977564 GA:0.0224359
chr28 11790376 2 308 G:0.720779 T:0.279221
chr28 11790436 2 316 A:0.971519 C:0.028481
chr28 11790441 2 312 G:0.996795 A:0.00320513
chr28 11790546 2 300 G:0.15 A:0.85
chr28 11790555 2 298 C:0.567114 A:0.432886
chr28 11791122 2 308 C:0.948052 T:0.0519481
chr28 11791647 2 306 T:0.173203 C:0.826797
chr28 11791873 2 304 T:0.0394737 C:0.960526
chr28 11791900 2 294 C:0.751701 T:0.248299
chr28 11793201 2 308 C:0.724026 T:0.275974
chr28 11793581 2 300 C:0.953333 T:0.0466667
chr28 11793605 2 308 G:0.977273 A:0.0227273
chr28 11793750 2 294 A:0.748299 AAG:0.251701
chr28 11793873 2 304 A:0.161184 C:0.838816
chr28 11793901 2 296 AT:0.692568 A:0.307432
chr28 11794282 2 298 T:0.963087 C:0.0369128
chr28 11794300 2 304 G:0.75 T:0.25
chr28 11794318 2 310 G:0.770968 T:0.229032
chr28 11794971 2 294 G:0.97619 A:0.0238095
chr28 11794979 2 292 G:0.157534 A:0.842466
chr28 11795991 2 306 C:0.957516 T:0.0424837

chr28 11796018 3 298 T:0.302013 TG:0.436242
TTG:0.261745
chr28 11796027 2 302 G:0.864238 GGA:0.135762
chr28 11796042 2 302 T:0.940397 C:0.0596026
chr28 11796175 2 304 G:0.950658 A:0.0493421
chr28 11796203 2 308 C:0.977273 T:0.0227273
chr28 11796322 2 310 C:0.725806 G:0.274194
chr28 11796550 2 304 T:0.743421 C:0.256579
chr28 11796653 2 308 G:0.957792 T:0.0422078
chr28 11796680 2 306 A:0.738562 C:0.261438
chr28 11796783 3 272 TA:0.470588 T:0.301471
TAA:0.227941
chr28 11796785 2 294 A:0.982993 T:0.0170068
chr28 11796954 2 304 T:0.967105 TATAA:0.0328947
chr28 11796955 4 306 CTAAA:0.650327 C:0.218954
ATAAA:0.0392157 CTAAATAAA:0.0915033
chr28 11797048 3 296 GAA:0.182432 G:0.581081
GA:0.236486
chr28 11797348 2 304 C:0.940789 T:0.0592105
chr28 11797612 2 310 C:0.993548 G:0.00645161
chr28 11797870 2 306 TA:0.95098 T:0.0490196
chr28 11797886 2 302 C:0.721854 CT:0.278146
chr28 11797924 2 304 C:0.759868 G:0.240132
chr28 11797926 2 304 C:0.759868 G:0.240132
chr28 11797930 3 306 AAG:0.493464 A:0.267974
GAG:0.238562
chr28 11798052 2 296 C:0.716216 T:0.283784
chr28 11798176 2 292 GA:0.972603 G:0.0273973
chr28 11798392 2 292 T:0.722603 C:0.277397
chr28 11798395 2 292 C:0.722603 T:0.277397
chr28 11798517 2 292 AAATG:0.571918 A:0.428082
chr28 11798537 2 298 GAATGAATA:0.939597 G:
0.0604027
chr28 11798545 2 316 A:0.892405 AATG:0.107595
chr28 11798566 2 298 A:0.761745 C:0.238255
chr28 11798574 2 310 A:0.951613 AAG:0.0483871
chr28 11799037 2 312 T:0.724359 C:0.275641
chr28 11799055 2 306 T:0.330065 G:0.669935
chr28 11799202 2 286 C:0.741259 G:0.258741
chr28 11799343 2 266 C:0.725564 T:0.274436
chr28 11799356 2 272 C:0.897059 T:0.102941
chr28 11799383 2 258 C:0.705426 T:0.294574
chr28 11799462 2 286 A:0.167832 G:0.832168
chr28 11799673 2 294 C:0.979592 T:0.0204082
chr28 11799848 2 290 C:0.355172 T:0.644828
chr28 11799928 2 278 C:0.870504 CCT:0.129496
chr28 11799947 2 288 C:0.600694 G:0.399306
chr28 11800055 2 300 T:0.386667 C:0.613333
chr28 11800064 2 296 TC:0.466216 T:0.533784
chr28 11800122 2 292 T:0.428082 C:0.571918
chr28 11800129 2 290 T:0.955172 G:0.0448276
chr28 11800151 2 294 C:0.969388 A:0.0306122
chr28 11800187 2 300 T:0.95 C:0.05
chr28 11800210 2 302 G:0.996689 A:0.00331126

chr28	11800343	4	312	CCTCTCTCTCTCT:0.394231	C:0.125	CCTCT:0.339744	CCTCTCT:0.141026
chr28	11800516	2	296	T:0.817568	A:0.182432		
chr28	11800593	2	306	A:0.408497	C:0.591503		
chr28	11800604	2	304	AG:0.717105	A:0.282895		
chr28	11800720	2	296	A:0.648649	G:0.351351		
chr28	11800826	2	306	G:0.156863	A:0.843137		
chr28	11800827	2	306	T:0.957516	C:0.0424837		
chr28	11800915	2	314	T:0.152866	G:0.847134		
chr28	11801101	2	308	T:0.818182	A:0.181818		
chr28	11801371	2	308	G:0.827922	A:0.172078		
chr28	11801386	2	304	A:0.838816	G:0.161184		
chr28	11801464	2	304	A:0.983553	C:0.0164474		
chr28	11801555	2	310	G:0.974194	A:0.0258065		
chr28	11801669	2	314	C:0.573248	T:0.426752		
chr28	11801788	2	314	G:0.942675	A:0.0573248		
chr28	11802005	2	310	G:0.645161	C:0.354839		
chr28	11802011	2	310	G:0.693548	A:0.306452		
chr28	11802016	2	310	T:0.825806	C:0.174194		
chr28	11802098	2	304	C:0.661184	CT:0.338816		
chr28	11802211	2	306	A:0.95098	T:0.0490196		
chr28	11802315	2	314	GTTTTCTTTCT:0.914013	G: 0.0859873		
chr28	11802650	2	314	A:0.573248	C:0.426752		
chr28	11802676	2	304	C:0.717105	T:0.282895		
chr28	11802821	2	312	T:0.833333	C:0.166667		
chr28	11803773	4	314	CGT:0.480892	C:0.302548	CGTGT:0.178344	CGTGTGTGTGT:0.0382166
chr28	11804481	2	312	T:0.413462	C:0.586538		
chr28	11804513	2	312	C:0.977564	T:0.0224359		
chr28	11804562	2	308	C:0.980519	T:0.0194805		
chr28	11804597	2	308	G:0.977273	A:0.0227273		
chr28	11804796	2	312	G:0.977564	A:0.0224359		
chr28	11804985	2	302	G:0.94702	A:0.0529801		
chr28	11805043	2	314	C:0.818471	G:0.181529		
chr28	11805140	2	310	AC:0.825806	A:0.174194		
chr28	11805141	2	310	C:0.606452	A:0.393548		
chr28	11805286	2	308	A:0.386364	AGAG:0.613636		
chr28	11805554	2	302	C:0.612583	T:0.387417		
chr28	11805609	2	312	G:0.823718	T:0.176282		
chr28	11805896	2	310	T:0.906452	C:0.0935484		
chr28	11805899	2	312	A:0.951923	G:0.0480769		
chr28	11806045	2	310	G:0.835484	T:0.164516		
chr28	11806146	2	302	C:0.407285	CT:0.592715		
chr28	11806176	2	292	CA:0.94863	C:0.0513699		
chr28	11806298	2	302	A:0.410596	G:0.589404		
chr28	11806664	2	308	A:0.785714	T:0.214286		
chr28	11807120	2	304	TACC:0.8125	T:0.1875		
chr28	11807136	3	310	CT:0.764516	C:0.190323	CTT:0.0451613	
chr28	11807147	2	308	A:0.954545	T:0.0454545		
chr28	11807307	2	310	CAA:0.619355	C:0.380645		
chr28	11807387	2	298	G:0.577181	T:0.422819		
chr28	11807555	2	304	A:0.411184	T:0.588816		

chr28	11807626	2	296	A:0.837838	G:0.162162	
chr28	11807707	2	294	C:0.802721	T:0.197279	
chr28	11807769	2	294	G:0.884354	A:0.115646	
chr28	11807828	2	310	A:0.809677	G:0.190323	
chr28	11808195	2	310	T:0.841935	C:0.158065	
chr28	11808348	2	302	C:0.834437	T:0.165563	
chr28	11808481	2	302	T:0.860927	C:0.139073	
chr28	11808638	2	306	C:0.921569	T:0.0784314	
chr28	11808712	2	304	T:0.75	C:0.25	
chr28	11809063	2	310	T:0.4	C:0.6	
chr28	11809072	2	310	T:0.4	C:0.6	
chr28	11809151	2	314	C:0.585987	T:0.414013	
chr28	11809171	2	314	G:0.977707	A:0.022293	
chr28	11809177	2	306	A:0.401961	G:0.598039	
chr28	11809382	2	300	A:0.946667	G:0.0533333	
chr28	11809535	2	316	C:0.908228	T:0.0917722	
chr28	11809603	2	310	ATTC:0.893548	A:0.106452	
chr28	11809604	2	310	TTC:0.964516	T:0.0354839	
chr28	11809605	2	310	TC:0.970968	T:0.0290323	
chr28	11809606	2	310	C:0.658065	CT:0.341935	
chr28	11809614	2	310	T:0.993548	TA:0.00645161	
chr28	11809622	2	310	T:0.596774	C:0.403226	
chr28	11809676	2	294	G:0.598639	A:0.401361	
chr28	11809724	2	290	C:0.603448	T:0.396552	
chr28	11809857	2	314	A:0.573248	G:0.426752	
chr28	11809963	2	304	A:0.585526	G:0.414474	
chr28	11810177	2	304	T:0.838816	A:0.161184	
chr28	11810265	2	312	G:0.833333	A:0.166667	
chr28	11810439	2	312	G:0.977564	C:0.0224359	
chr28	11810719	2	304	T:0.575658	C:0.424342	
chr28	11810788	2	306	T:0.437908	C:0.562092	
chr28	11810820	2	306	C:0	A:1	
chr28	11810822	2	306	T:0	G:1	
chr28	11810869	2	306	C:0.588235	T:0.411765	
chr28	11810984	2	308	C:0.954545	A:0.0454545	
chr28	11811150	2	306	G:0.954248	A:0.0457516	
chr28	11811157	2	304	G:0.769737	A:0.230263	
chr28	11811263	2	312	C:0.580128	T:0.419872	
chr28	11811376	2	302	C:0.748344	T:0.251656	
chr28	11811557	2	318	T:0.965409	G:0.0345912	
chr28	11811873	3	300	CTT:0.51	C:0.266667	CT:0.223333
chr28	11811960	2	294	C:0.829932	T:0.170068	
chr28	11812022	2	308	G:0.837662	T:0.162338	
chr28	11812159	2	310	G:0.96129	A:0.0387097	
chr28	11812218	2	304	T:0.414474	A:0.585526	
chr28	11812393	2	312	G:0.955128	T:0.0448718	
chr28	11812447	2	310	T:0.964516	C:0.0354839	
chr28	11812641	2	300	G:0.74	C:0.26	
chr28	11812831	2	308	G:0.467532	A:0.532468	
chr28	11812881	2	310	A:0.583871	AAT:0.416129	
chr28	11813079	2	310	G:0.974194	A:0.0258065	
chr28	11813123	2	306	G:0.977124	A:0.0228758	
chr28	11813221	2	304	AC:0.970395	A:0.0296053	

chr28	11813276	2	302	T:0.751656	TA:0.248344	
chr28	11813318	2	308	A:0.409091	G:0.590909	
chr28	11813397	2	302	A:0.97351	G:0.0264901	
chr28	11813433	2	300	C:0.453333	T:0.546667	
chr28	11813461	2	294	AAC:0.965986	A:0.0340136	
chr28	11813649	2	290	C:0.97931	T:0.0206897	
chr28	11813676	2	306	T:0.421569	TCGAGGTC:0.578431	
chr28	11813870	2	314	G:0.974522	GT:0.0254777	
chr28	11813872	2	314	G:0.974522	GT:0.0254777	
chr28	11813873	2	314	G:0.974522	GATA:0.0254777	
chr28	11813877	2	314	G:0.974522	GAAGTGAA:0.0254777	
chr28	11813879	2	314	ATTC:0.974522	A:0.0254777	
chr28	11813883	2	314	T:0.974522	G:0.0254777	
chr28	11813898	2	294	GA:0.139456	G:0.860544	
chr28	11813981	2	300	G:0.466667	A:0.533333	
chr28	11813984	2	300	C:0.98	T:0.02	
chr28	11814013	2	302	T:0.172185	C:0.827815	
chr28	11814146	2	304	G:0.967105	GA:0.0328947	
chr28	11814337	2	302	TCTC:0.966887	T:0.0331126	
chr28	11814602	2	306	C:0.934641	CA:0.0653595	
chr28	11814736	2	300	A:0.966667	C:0.0333333	
chr28	11815053	2	306	A:0.957516	G:0.0424837	
chr28	11815772	2	304	T:0.430921	A:0.569079	
chr28	11815788	2	306	G:0.166667	A:0.833333	
chr28	11816506	3	316	A:0.509494	AAC:0.237342	AACACACACAC:0.253165
chr28	11816632	2	312	T:0.650641	C:0.349359	
chr28	11816655	2	306	C:0.647059	T:0.352941	
chr28	11816786	2	302	T:0.698675	C:0.301325	
chr28	11816941	2	308	C:0.996753	T:0.00324675	
chr28	11816942	2	308	A:0.743506	G:0.256494	
chr28	11816992	2	314	G:0.684713	C:0.315287	
chr28	11816998	2	314	C:0.684713	T:0.315287	
chr28	11817018	2	314	A:0.684713	G:0.315287	
chr28	11817080	2	304	C:0.680921	T:0.319079	
chr28	11817154	2	304	A:0.680921	G:0.319079	
chr28	11817388	2	308	A:0.756494	G:0.243506	
chr28	11817437	2	308	T:0.681818	A:0.318182	
chr28	11817467	2	308	T:0.681818	C:0.318182	
chr28	11817485	2	308	C:0.681818	T:0.318182	
chr28	11817814	2	306	C:0.650327	T:0.349673	
chr28	11817846	2	312	CTAATT:0.958333	C:0.0416667	
chr28	11818123	2	310	C:0.687097	T:0.312903	
chr28	11818310	2	298	A:0.939597	G:0.0604027	
chr28	11818731	2	312	C:0.669872	T:0.330128	
chr28	11819259	2	296	T:0.689189	C:0.310811	
chr28	11819408	2	316	A:0.686709	G:0.313291	
chr28	11819651	2	306	G:0.679739	A:0.320261	
chr28	11819773	2	308	G:0.662338	C:0.337662	
chr28	11819835	3	282	CAA:0.195035	C:0.695035	CA:0.109929
chr28	11819852	2	298	A:0.667785	G:0.332215	
chr28	11819948	2	310	A:0.703226	G:0.296774	

chr28	11819970	2	318	GTTAACTTAAGTTAAGTTAACTTAAGGCTTAAGTTAAGTTA:0.163522	G:0.836478	
chr28	11820100	2	300	T:0.836667	C:0.163333	
chr28	11820249	2	300	C:0.796667	T:0.203333	
chr28	11820285	3	314	GATAAATAAATAA:0.340764	G:0.232484	GATAAATAA:0.426752
chr28	11820434	2	302	T:0.682119	C:0.317881	
chr28	11820503	2	292	C:0.791096	T:0.208904	
chr28	11821191	2	300	G:0.78	GA:0.22	
chr28	11821468	2	304	T:0.924342	TG:0.0756579	
chr28	11822074	2	312	A:0.557692	T:0.442308	
chr28	11822106	2	312	C:0.625	T:0.375	
chr28	11822620	2	306	G:0.833333	T:0.166667	
chr28	11823401	2	298	C:0.993289	T:0.00671141	
chr28	11823549	2	304	T:0.542763	C:0.457237	
chr28	11823682	2	298	G:0.154362	A:0.845638	
chr28	11823745	2	310	A:0.593548	G:0.406452	
chr28	11824349	2	304	G:0.9375	C:0.0625	
chr28	11824401	2	310	G:0.132258	A:0.867742	
chr28	11824639	2	306	T:0.928105	G:0.0718954	
chr28	11824717	2	310	G:0.687097	T:0.312903	
chr28	11825067	2	306	G:0.918301	GC:0.0816993	
chr28	11825091	2	314	G:0.933121	A:0.066879	
chr28	11825265	2	308	G:0.477273	A:0.522727	
chr28	11825271	2	306	T:0.794118	C:0.205882	
chr28	11825284	2	308	C:0.477273	A:0.522727	
chr28	11825581	2	314	A:0.671975	G:0.328025	
chr28	11825663	2	302	C:0.450331	A:0.549669	
chr28	11825667	2	298	A:0.520134	T:0.479866	
chr28	11825919	2	312	T:0.996795	C:0.00320513	
chr28	11826023	2	308	G:0.152597	A:0.847403	
chr28	11826264	2	308	C:0.461039	CA:0.538961	
chr28	11826297	2	312	C:0.932692	T:0.0673077	
chr28	11826375	2	312	C:0.461538	A:0.538462	
chr28	11826420	2	298	C:0.147651	T:0.852349	
chr28	11826445	2	298	G:0.47651	C:0.52349	
chr28	11826491	2	302	G:0.665563	A:0.334437	
chr28	11826844	2	308	A:0.525974	G:0.474026	
chr28	11826905	2	314	C:0.770701	CTAAT:0.229299	
chr28	11826973	2	318	T:0.669811	C:0.330189	
chr28	11827081	2	312	CAATAA:0.923077	C:0.0769231	
chr28	11827150	2	304	A:0.973684	G:0.0263158	
chr28	11827449	2	308	T:0.951299	C:0.0487013	
chr28	11827559	2	302	C:0.68543	T:0.31457	
chr28	11827649	2	302	G:0.930464	A:0.0695364	
chr28	11827729	2	316	A:0.933544	G:0.0664557	
chr28	11827775	2	312	C:0.679487	T:0.320513	
chr28	11827790	2	312	G:0.919872	GAT:0.0801282	
chr28	11827919	2	308	A:0.162338	T:0.837662	
chr28	11827970	2	306	T:0.924837	A:0.0751634	
chr28	11827995	2	316	A:0.683544	ATTATATG:0.316456	
chr28	11828110	2	306	C:0.934641	A:0.0653595	
chr28	11828111	2	306	G:0.970588	A:0.0294118	
chr28	11828210	2	314	G:0.799363	A:0.200637	

chr28	11828230	2	308	T:0.928571	TA:0.0714286
chr28	11828240	2	312	T:0.983974	A:0.0160256
chr28	11828364	2	308	G:0.555195	A:0.444805
chr28	11828419	2	312	C:0.541667	T:0.458333
chr28	11828430	2	312	G:0.541667	GT:0.458333
chr28	11828459	2	312	C:0.544872	T:0.455128
chr28	11828495	2	318	C:0.144654	T:0.855346
chr28	11828545	2	312	C:0.599359	T:0.400641
chr28	11828579	2	308	T:0.525974	C:0.474026
chr28	11828585	2	306	GA:0.594771	G:0.405229
chr28	11828601	2	318	T:0.95283	C:0.0471698
chr28	11828692	2	300	G:0.556667	T:0.443333
chr28	11828724	2	260	A:0.257692	G:0.742308
chr28	11828765	2	204	G:0.54902	A:0.45098
chr28	11828766	2	204	A:0.387255	G:0.612745
chr28	11830489	2	104	C:0.605769	T:0.394231
chr28	11830516	2	144	ATT:0.548611	A:0.451389
chr28	11830520	2	144	T:0.548611	TAAA:0.451389
chr28	11830549	2	230	TA:0.278261	T:0.721739
chr28	11830623	2	286	G:0.937063	A:0.0629371
chr28	11830972	2	292	TTC:0.60274	T:0.39726
chr28	11830989	2	304	TC:0.588816	T:0.411184
chr28	11831002	2	302	C:0.645695	T:0.354305
chr28	11831044	2	304	A:0.947368	G:0.0526316
chr28	11831052	2	304	CT:0.947368	C:0.0526316
chr28	11831054	2	290	TA:0.668966	T:0.331034
chr28	11831158	2	300	A:0.166667	AG:0.833333
chr28	11831197	2	298	G:0.489933	A:0.510067
chr28	11831230	2	300	ACT:0.65	A:0.35
chr28	11831478	2	294	A:0.547619	G:0.452381
chr28	11831512	2	306	AT:0.169935	A:0.830065
chr28	11831519	2	308	C:0.555195	T:0.444805
chr28	11831520	2	308	C:0.512987	G:0.487013
chr28	11831564	2	312	A:0.554487	T:0.445513
chr28	11831602	2	300	T:0.953333	A:0.0466667
chr28	11831672	2	314	A:0.535032	G:0.464968
chr28	11831673	2	314	C:0.700637	T:0.299363
chr28	11831689	2	314	ATACC:0.535032	A:0.464968
chr28	11831695	2	314	GGAA:0.535032	G:0.464968
chr28	11831743	2	298	A:0.177852	C:0.822148
chr28	11831811	2	312	T:0.490385	C:0.509615
chr28	11831902	2	314	C:0.477707	T:0.522293
chr28	11831994	2	312	G:0.971154	T:0.0288462
chr28	11832056	2	310	G:0.948387	A:0.0516129
chr28	11832120	2	296	G:0.527027	A:0.472973
chr28	11832161	2	308	C:0.548701	T:0.451299
chr28	11832260	2	316	G:0.487342	A:0.512658
chr28	11832317	2	298	G:0.516779	GA:0.483221
chr28	11832396	2	294	A:0.92517	G:0.0748299
chr28	11832428	2	302	A:0.5	G:0.5
chr28	11832479	2	310	C:0.496774	A:0.503226
chr28	11832562	2	304	G:0.496711	A:0.503289
chr28	11832586	2	302	T:0.513245	C:0.486755
chr28	11832644	2	300	T:0.51	C:0.49

chr28	11832742 5	298	ATTT:0.197987	A:0.0771812
AT:0.302013	ATT:0.174497	ATTTT:0.248322
chr28	11832909 2	310	A:0.5	G:0.5
chr28	11832947 2	310	T:0.945161	TA:0.0548387
chr28	11832948 2	310	A:0.841935	T:0.158065
chr28	11832949 2	310	G:0.512903	A:0.487097
chr28	11833158 2	318	T:0.943396
TTAAAACGTTTAAAAAAAAATAAAAAAAA:0.0566038
chr28	11833277 2	304	C:0.536184	T:0.463816
chr28	11833284 2	308	T:0.993506	C:0.00649351
chr28	11833337 2	310	G:0.516129	A:0.483871
chr28	11833421 2	300	G:0.976667	T:0.0233333
chr28	11833456 2	302	CA:0	C:1
chr28	11833459 2	302	C:0	G:1
chr28	11833491 2	310	G:0.0193548	GAA:0.980645
chr28	11833492 2	310	T:0.0193548	TTAG:0.980645
chr28	11833560 2	320	ATGTGAATCT:0.946875	A:
0.053125
chr28	11833746 2	296	C:0.496622	T:0.503378
chr28	11833798 2	296	T:0.97973	C:0.0202703
chr28	11833836 2	300	T:0.95	C:0.05
chr28	11834109 2	296	C:0.97973	T:0.0202703
chr28	11834218 2	314	C:0.675159	CCAAA:0.324841
chr28	11834221 2	314	C:0.675159	T:0.324841
chr28	11834404 2	304	A:0.5625 G:0.4375
chr28	11834520 2	306	G:0.147059	A:0.852941
chr28	11834541 2	306	G:0.169935	GT:0.830065
chr28	11834686 2	310	G:0.53871	A:0.46129
chr28	11834708 2	306	G:0.944444	A:0.0555556
chr28	11834721 2	308	C:0.149351	T:0.850649
chr28	11834734 2	308	C:0.149351	T:0.850649
chr28	11834783 2	288	G:0.649306	A:0.350694
chr28	11834854 2	294	A:0.97619	G:0.0238095
chr28	11835063 2	306	G:0.297386	A:0.702614
chr28	11835117 2	304	C:0.927632	CA:0.0723684
chr28	11835326 2	306	G:0.150327	A:0.849673
chr28	11835337 3	318	T:0.679245
TAAAAATGGGAAGGTAAAAA:0.27044	TAAAAATGGGAAGGTAAAAAA:0.0503145
chr28	11835457 2	310	G:0.922581	T:0.0774194
chr28	11835484 2	300	T:0.15	A:0.85
chr28	11835514 2	298	C:0.912752	CA:0.0872483
chr28	11835610 2	308	T:0.142857	C:0.857143
chr28	11835757 2	306	C:0.830065	T:0.169935
chr28	11835894 2	302	C:0.142384	T:0.857616
chr28	11835919 2	302	A:0.142384	G:0.857616
chr28	11835996 2	306	T:0.643791	A:0.356209
chr28	11836065 2	308	T:0.951299	C:0.0487013
chr28	11836313 2	312	A:0.833333
ATGAGAAAATATTTTTTTTTTTT:0.166667
chr28	11836403 2	308	C:0.954545	T:0.0454545
chr28	11836409 2	308	A:0.506494	G:0.493506
chr28	11836574 2	312	T:0.935897	G:0.0641026
chr28	11836609 2	308	C:0.646104	A:0.353896
chr28	11836733 2	304	C:0.151316	T:0.848684

chr28	11836800	2	310	T:0.170968	C:0.829032
chr28	11836862	2	316	G:0.161392	A:0.838608
chr28	11837006	2	308	A:0.172078	C:0.827922
chr28	11837042	2	314	A:0.681529	T:0.318471
chr28	11837067	2	314	C:0.681529	G:0.318471
chr28	11837127	2	310	A:0.187097	C:0.812903
chr28	11837136	2	306	C:0.95098	A:0.0490196
chr28	11837245	2	312	T:0.842949	C:0.157051
chr28	11837546	2	310	T:0.670968	C:0.329032
chr28	11837672	2	304	G:0.703947	T:0.296053
chr28	11837742	2	298	A:0.687919	G:0.312081
chr28	11837794	2	302	T:0.539735	A:0.460265
chr28	11837849	2	308	A:0.155844	G:0.844156
chr28	11837878	2	300	T:0.853333	TA:0.146667
chr28	11837879	2	304	T:0.473684	A:0.526316
chr28	11838120	2	306	G:0.69281	A:0.30719
chr28	11838205	2	310	TAATG:0.690323	T:0.309677
chr28	11838398	2	306	T:0.676471	A:0.323529
chr28	11838570	2	314	A:0.175159	G:0.824841
chr28	11838777	2	306	G:0.147059	A:0.852941
chr28	11838809	2	306	ATC:0.95098	A:0.0490196
chr28	11838940	2	306	A:0.156863	G:0.843137
chr28	11839043	2	300	G:0.146667	T:0.853333
chr28	11839070	2	304	A:0.157895	G:0.842105
chr28	11839158	2	306	C:0.156863	T:0.843137
chr28	11839209	2	314	T:0.171975	TAAGA:0.828025
chr28	11839356	2	306	G:0.166667	GA:0.833333
chr28	11839467	2	308	G:0.162338	C:0.837662
chr28	11840040	2	306	T:0.666667	C:0.333333
chr28	11840100	2	300	C:0.836667	A:0.163333
chr28	11840264	2	306	G:0.539216	A:0.460784
chr28	11840358	2	304	T:0.217105	G:0.782895
chr28	11840359	2	304	A:0.217105	G:0.782895
chr28	11840463	2	316	G:0.873418	C:0.126582
chr28	11841017	2	310	AG:0.693548	A:0.306452
chr28	11841310	2	310	T:0.132258	C:0.867742
chr28	11841644	2	308	C:0.918831	T:0.0811688
chr28	11841865	2	302	T:0.52649	C:0.47351
chr28	11841892	2	304	A:0.930921	G:0.0690789
chr28	11841941	2	310	G:0.970968	A:0.0290323
chr28	11841996	2	314	C:0.130573	T:0.869427
chr28	11842065	2	308	T:0.172078	TTATATC:0.827922
chr28	11842081	2	304	A:0.980263	G:0.0197368
chr28	11842281	2	298	TA:0.573826	T:0.426174
chr28	11842318	2	312	C:0.13141	A:0.86859
chr28	11842384	2	298	T:0.979866	G:0.0201342
chr28	11842436	2	306	A:0.140523	G:0.859477
chr28	11842615	2	308	T:0.142857	C:0.857143
chr28	11842801	2	298	C:0.708054	A:0.291946
chr28	11842817	2	296	G:0.841216	A:0.158784
chr28	11842837	2	296	A:0.983108	G:0.0168919
chr28	11842919	2	306	A:0.127451	C:0.872549
chr28	11842933	2	306	T:0.823529	A:0.176471
chr28	11843043	2	302	A:0.688742	G:0.311258

chr28	11843103	2	312	G:0	GA:1		
chr28	11843108	2	312	AT:0	A:1		
chr28	11843111	2	312	G:0	A:1		
chr28	11843114	2	312	AC:0	A:1		
chr28	11843120	2	312	G:0	A:1		
chr28	11843121	2	312	A:0	G:1		
chr28	11843135	2	312	G:0	T:1		
chr28	11843136	2	312	A:0	G:1		
chr28	11843151	2	312	C:0	A:1		
chr28	11843152	2	312	G:0	C:1		
chr28	11843216	2	312	GC:0	G:1		
chr28	11843431	3	300	G:0.606667	GA:0.22	GAA:0.173333	
chr28	11843435	2	302	A:0.837748	G:0.162252		
chr28	11843497	2	310	C:0.46129	T:0.53871		
chr28	11843521	2	310	A:0.845161	G:0.154839		
chr28	11843596	2	306	C:0.79085	T:0.20915		
chr28	11843803	2	284	T:0.816901	C:0.183099		
chr28	11843819	4	308	CCTCTCTCTCTCTCTCTCT:0.175325	C:0.227273	CCTCT:0.311688	CCTCTCTCT:0.285714
chr28	11843936	2	290	G:0.848276	A:0.151724		
chr28	11844118	2	122	C:0.704918	T:0.295082		
chr28	11844333	2	300	C:0.156667	A:0.843333		
chr28	11844361	2	298	AT:0.808725	A:0.191275		
chr28	11844447	2	302	T:0.788079	G:0.211921		
chr28	11844576	2	316	C:0.882911	CAAAA:0.117089		
chr28	11845066	2	230	C:0.656522	T:0.343478		
chr28	11845187	2	214	CA:0.602804	C:0.397196		
chr28	11845202	2	214	C:0.602804	A:0.397196		
chr28	11845333	2	300	A:0.846667	G:0.153333		
chr28	11845820	2	162	T:0.851852	A:0.148148		
chr28	11845839	2	178	A:0.179775	G:0.820225		
chr28	11845849	2	190	T:0.957895	G:0.0421053		
chr28	11845940	2	302	C:0.586093	T:0.413907		
chr28	11846050	2	126	A:0.214286	G:0.785714		
chr28	11846163	2	304	T:0.815789	G:0.184211		
chr28	11846225	2	308	G:0.652597	A:0.347403		
chr28	11846457	2	306	T:0.163399	G:0.836601		
chr28	11846915	2	300	GT:0.843333	G:0.156667		
chr28	11846968	2	308	G:0.451299	A:0.548701		
chr28	11847408	2	304	G:0.121711	A:0.878289		
chr28	11847412	2	302	C:0.440397	T:0.559603		
chr28	11847489	2	302	A:0.625828	G:0.374172		
chr28	11847696	2	306	G:0.614379	A:0.385621		
chr28	11847823	2	298	T:0.694631	G:0.305369		
chr28	11848129	2	298	T:0.758389	C:0.241611		
chr28	11848274	2	314	A:0.83758	G:0.16242		
chr28	11848503	2	308	TTTTC:0.685065	T:0.314935		
chr28	11848581	2	294	A:0.94898	C:0.0510204		
chr28	11849481	2	280	C:0.875	T:0.125		
chr28	11849660	2	304	A:0.983553	T:0.0164474		
chr28	11849668	2	306	A:0.748366	G:0.251634		
chr28	11849831	2	304	A:0.430921	G:0.569079		
chr28	11850180	2	308	T:0.266234	TA:0.733766		

chr28	11850231	2	314	T:0.140127	G:0.859873	
chr28	11850436	2	296	T:0.858108	TA:0.141892	
chr28	11850510	2	306	C:0.826797	G:0.173203	
chr28	11850866	2	310	G:0.980645	T:0.0193548	
chr28	11851134	2	312	G:0.823718	A:0.176282	
chr28	11851149	2	298	CT:0.469799	C:0.530201	
chr28	11851158	2	302	T:0.655629	A:0.344371	
chr28	11851160	2	298	A:0.704698	AT:0.295302	
chr28	11851192	2	276	A:0.518116	AAG:0.481884	
chr28	11851261	2	280	C:0.846429	T:0.153571	
chr28	11851340	2	306	C:0.898693	T:0.101307	
chr28	11851374	2	300	CA:0.943333	C:0.0566667	
chr28	11851486	2	304	G:0.986842	A:0.0131579	
chr28	11851525	2	298	T:0.14094	C:0.85906	
chr28	11851613	2	312	C:0.849359	T:0.150641	
chr28	11851725	2	302	T:0.738411	C:0.261589	
chr28	11851797	2	300	T:0.98	G:0.02	
chr28	11852005	2	306	A:0.892157	T:0.107843	
chr28	11852018	2	308	A:0.282468	G:0.717532	
chr28	11852216	2	294	C:0.877551	T:0.122449	
chr28	11852271	2	302	C:0.52649	A:0.47351	
chr28	11852326	2	306	A:0.130719	G:0.869281	
chr28	11852709	2	308	T:0.279221	C:0.720779	
chr28	11852752	2	308	A:0.909091	G:0.0909091	
chr28	11852785	2	310	T:0.245161	A:0.754839	
chr28	11853025	3	304	CAA:0.161184	C:0.134868	CA:0.703947
chr28	11853136	2	306	C:0.856209	T:0.143791	
chr28	11853296	2	308	C:0.685065	A:0.314935	
chr28	11853440	2	306	T:0.124183	C:0.875817	
chr28	11853526	2	304	A:0.927632	G:0.0723684	
chr28	11853567	2	308	G:0.987013	A:0.012987	
chr28	11853933	2	310	A:0.674194	G:0.325806	
chr28	11854146	2	306	T:0.542484	G:0.457516	
chr28	11854209	2	310	G:0.654839	A:0.345161	
chr28	11854354	2	304	A:0.536184	G:0.463816	
chr28	11854391	2	302	G:0.927152	C:0.0728477	
chr28	11854476	2	298	TA:0.657718	T:0.342282	
chr28	11854506	2	312	C:0.673077	T:0.326923	
chr28	11854642	2	302	A:0.142384	T:0.857616	
chr28	11854673	2	300	T:0.7	A:0.3	
chr28	11854676	2	298	A:0.627517	T:0.372483	
chr28	11854708	2	304	C:0.944079	A:0.0559211	
chr28	11855104	2	314	C:0.83121	T:0.16879	
chr28	11855240	2	320	TA:0.659375	T:0.340625	
chr28	11855243	2	320	CTCTATTTGTATATTTTGACAATCTATTT:0.659375	C:0.340625	
chr28	11855485	2	302	TAA:0.864238	T:0.135762	
chr28	11855489	2	298	AAAAAATAAAATAAAAT:0.916107	A:0.0838926	
chr28	11855491	2	316	A:0.490506	AT:0.509494	
chr28	11855492	2	316	AAAT:0.933544	A:0.0664557	
chr28	11855495	3	316	TAAAATAAAATAAAA:0.360759	T:0.129747	TAAATAAAATAAAA:0.509494

chr28	11855500	3	316	TA:0.667722	TAAA:0.0664557
T:0.265823					
chr28	11855501	2	316	A:0.825949	AAAT:0.174051
chr28	11855509	2	316	A:0.490506	T:0.509494
chr28	11855989	2	304	A:0.894737	G:0.105263
chr28	11856291	2	310	G:0.703226	A:0.296774
chr28	11856359	2	304	C:0.608553	A:0.391447
chr28	11856589	2	294	T:0.972789	C:0.0272109
chr28	11856689	2	300	G:0.656667	A:0.343333
chr28	11856771	2	304	A:0.598684	C:0.401316
chr28	11856801	2	300	C:0.986667	T:0.0133333
chr28	11856828	2	296	C:0.983108	T:0.0168919
chr28	11856850	2	302	G:0.94702	T:0.0529801
chr28	11856984	2	294	C:0.911565	T:0.0884354
chr28	11857174	2	294	GA:0.163265	G:0.836735
chr28	11857197	2	298	G:0.946309	A:0.0536913
chr28	11857306	2	306	T:0.133987	A:0.866013
chr28	11857441	2	310	C:0.151613	T:0.848387
chr28	11857656	2	306	G:0.875817	A:0.124183
chr28	11857712	2	296	G:0.503378	A:0.496622
chr28	11857887	2	304	G:0.786184	T:0.213816
chr28	11857896	2	298	C:0.842282	T:0.157718
chr28	11858000	2	298	A:0.144295	C:0.855705
chr28	11858084	2	306	A:0.859477	G:0.140523
chr28	11858194	2	300	G:0.796667	T:0.203333
chr28	11858610	2	302	T:0.327815	C:0.672185
chr28	11858912	2	308	C:0.980519	T:0.0194805
chr28	11859084	2	304	C:0.819079	A:0.180921
chr28	11859194	2	300	C:0.85 T:0.15	
chr28	11859376	2	308	C:0.808442	T:0.191558
chr28	11859705	2	310	ATTT:0.345161	A:0.654839
chr28	11859712	2	308	C:0.961039	T:0.038961
chr28	11859833	2	282	T:0.343972	TA:0.656028
chr28	11859960	2	296	A:0.692568	T:0.307432
chr28	11860041	2	302	T:0.483444	C:0.516556
chr28	11860201	2	298	CAT:0.828859	C:0.171141
chr28	11860909	2	302	C:0.523179	A:0.476821
chr28	11861144	2	316	G:0.971519	GTAAAA:0.028481
chr28	11861147	2	316	G:0.971519	GA:0.028481
chr28	11861148	2	316	T:0.971519	TAATCTTTTATCTAGG:
0.028481					
chr28	11861292	2	298	T:0.942953	C:0.057047
chr28	11861299	2	296	C:0.962838	CT:0.0371622
chr28	11861520	2	312	C:0.833333	G:0.166667
chr28	11862021	2	292	C:0.136986	T:0.863014
chr28	11862382	2	308	A:0.821429	G:0.178571
chr28	11862419	2	296	G:0.638514	T:0.361486
chr28	11862432	2	284	A:0.711268	C:0.288732
chr28	11862474	2	288	A:0.826389	G:0.173611
chr28	11862665	2	304	GA:0.996711	G:0.00328947
chr28	11862708	2	300	TG:0.803333	T:0.196667
chr28	11862852	2	304	A:0.131579	G:0.868421
chr28	11863064	2	298	G:0.969799	C:0.0302013
chr28	11863087	2	306	G:0.130719	A:0.869281

chr28 11863317 2 308 TCAA:0.853896 T:0.146104
chr28 11863409 5 312 AAG:0.230769 A:0.153846
AAGAGAG:0.169872 AAGAGAGAG:0.333333 AAGAGAGAGAG:0.112179
chr28 11863543 2 304 T:0.151316 G:0.848684
chr28 11863601 2 310 C:0.858065 A:0.141935
chr28 11863735 2 308 C:0.987013 T:0.012987
chr28 11863841 2 302 C:0.480132 T:0.519868
chr28 11863912 2 306 T:0.470588 C:0.529412
chr28 11864152 2 300 A:0.466667 G:0.533333
chr28 11864492 2 312 A:0.823718 G:0.176282
chr28 11864653 2 308 A:0.892857 T:0.107143
chr28 11864859 2 304 C:0.161184 CCT:0.838816
chr28 11865229 2 300 T:0.636667 C:0.363333
chr28 11865376 2 302 C:0.817881 G:0.182119
chr28 11865467 2 258 TAGA:0.70155 T:0.29845
chr28 11865607 4 318 C:0.292453 CTGTGTG:0.166667
CTGTGTGTGTG:0.106918 CTGTGTGTGTGTGTG:0.433962
chr28 11865639 2 316 C:0.563291 G:0.436709
chr28 11865677 2 318 A:0.688679 AAATG:0.311321
chr28 11865679 2 308 ATAAATAAG:0.477273 A:
0.522727
chr28 11865687 2 318 G:0.688679 A:0.311321
chr28 11865692 3 318 CAAAT:0.663522 C:0.0251572
TAAAT:0.311321
chr28 11865704 4 318 T:0.688679 TAAATA:0.110063
TAAATAAATA:0.100629 TAAATAAATAAATA:0.100629
chr28 11865794 2 286 C:0.947552 T:0.0524476
chr28 11866188 2 306 T:0.964052 C:0.0359477
chr28 11866467 2 306 G:0.133987 A:0.866013
chr28 11866651 2 310 C:0.125806 G:0.874194
chr28 11866749 2 280 G:0.253571 GA:0.746429
chr28 11866946 2 240 ATTAAT:0.7875 A:0.2125
chr28 11866955 2 284 A:0.81338 ATTTAT:0.18662
chr28 11867008 2 208 C:0.764423 T:0.235577
chr28 11867265 2 298 C:0.946309 A:0.0536913
chr28 11867606 2 308 C:0.746753 T:0.253247
chr28 11867663 2 302 A:0.854305 C:0.145695
chr28 11867677 2 304 C:0.967105 T:0.0328947
chr28 11868484 2 314 ATATACT:0.936306 A:0.0636943
chr28 11868566 2 302 T:0.715232 C:0.284768
chr28 11868609 2 298 G:0.973154 A:0.0268456
chr28 11868894 2 304 T:0.476974 TG:0.523026
chr28 11868900 2 304 ATC:0.476974 A:0.523026
chr28 11868905 2 304 T:0.476974
TCAGACCATGAGGTATATCAAATTAGATGTGGCA:0.523026
chr28 11869415 2 310 G:0.980645 A:0.0193548
chr28 11869471 2 308 C:0.633117 T:0.366883
chr28 11869604 2 292 C:0.928082 G:0.0719178
chr28 11869666 2 302 A:0.533113 AT:0.466887
chr28 11869801 2 310 CT:0.119355 C:0.880645
chr28 11869826 2 312 C:0.967949 T:0.0320513
chr28 11869857 2 304 A:0.523026 C:0.476974
chr28 11870017 2 300 A:0.776667 AT:0.223333
chr28 11870084 2 302 T:0.536424 C:0.463576

chr28	11870558	2	292	CA:0.140411	C:0.859589		
chr28	11870749	2	296	T:0.837838	TC:0.162162		
chr28	11870751	2	294	C:0.591837	CG:0.408163		
chr28	11870758	4	306	C:0.51634	CAA:0.0620915	CCCAAA:0.183007	CCCCAAA:0.238562
chr28	11870817	2	296	G:0.547297	A:0.452703		
chr28	11870859	2	294	G:0.969388	A:0.0306122		
chr28	11871195	2	318	G:0.946541	A:0.0534591		
chr28	11871288	2	304	A:0.986842	G:0.0131579		
chr28	11871939	2	304	C:0.615132	CAAAA:0.384868		
chr28	11871970	2	306	G:0.53268	A:0.46732		
chr28	11872004	2	312	G:0.125	T:0.875		
chr28	11872102	3	298	GTT:0.35906	G:0.302013	GTTT:0.338926	
chr28	11872114	2	294	T:0.802721	A:0.197279		
chr28	11872115	3	298	T:0.486577	A:0.211409	TAAA:0.302013	
chr28	11872116	2	296	A:0.658784	T:0.341216		
chr28	11872293	4	296	GTT:0.253378	G:0.293919	GT:0.0777027	GTTT:0.375
chr28	11872329	2	302	C:0.615894	G:0.384106		
chr28	11872333	2	304	C:0.950658	T:0.0493421		
chr28	11872600	2	312	G:0.641026	GA:0.358974		
chr28	11873067	2	310	A:0.348387	AGT:0.651613		
chr28	11873070	4	314	A:0.16879	ATAG:0.210191	ATAGTATAGTATAG:0.442675	ATATAGTATAGTATAGTATAG:0.178344
chr28	11873397	2	312	T:0.137821	C:0.862179		
chr28	11873413	2	310	C:0.919355	T:0.0806452		
chr28	11873422	2	312	T:0.137821	C:0.862179		
chr28	11873476	2	302	G:0.976821	A:0.0231788		
chr28	11873785	2	308	A:0.99026	G:0.00974026		
chr28	11873991	2	312	T:0.839744	A:0.160256		
chr28	11874303	2	302	A:0.801325	C:0.198675		
chr28	11874403	2	304	T:0.141447	TC:0.858553		
chr28	11874613	2	296	C:0.719595	T:0.280405		
chr28	11874662	2	296	C:0.138514	G:0.861486		
chr28	11874841	2	308	G:0.62987	A:0.37013		
chr28	11874853	2	304	T:0.973684	C:0.0263158		
chr28	11875008	2	300	CA:0.786667	C:0.213333		
chr28	11875013	2	310	A:0.687097	AAC:0.312903		
chr28	11875034	2	314	AC:0.843949	A:0.156051		
chr28	11875039	2	310	AC:0.919355	A:0.0806452		
chr28	11875040	3	318	C:0.902516	A:0.081761	CAAA:0.0157233	
chr28	11875046	2	314	ACC:0.506369	A:0.493631		
chr28	11875047	2	316	C:0.756329	A:0.243671		
chr28	11875048	3	318	C:0.735849	CAAA:0.091195	CAAAAAAAAA:0.172956	
chr28	11875329	2	310	C:0.625806	CT:0.374194		
chr28	11875353	2	314	G:0.178344	C:0.821656		
chr28	11875472	2	300	G:0.616667	A:0.383333		
chr28	11875746	2	304	G:0.963816	A:0.0361842		
chr28	11875928	2	310	A:0.651613	G:0.348387		
chr28	11876735	2	306	C:0.70915	T:0.29085		

chr28	11876758	2	302	A:0.695364	AT:0.304636	
chr28	11876893	2	296	A:0.432432	AT:0.567568	
chr28	11877045	2	296	T:0.381757	C:0.618243	
chr28	11877356	2	306	T:0.447712	C:0.552288	
chr28	11877566	2	310	C:0.970968	T:0.0290323	
chr28	11877714	2	304	A:0.953947	G:0.0460526	
chr28	11877788	2	302	AT:0.440397	A:0.559603	
chr28	11877919	2	300	G:0.783333	A:0.216667	
chr28	11878099	2	310	C:0.435484	T:0.564516	
chr28	11878140	2	312	T:0.948718	C:0.0512821	
chr28	11878157	2	310	A:0.151613	G:0.848387	
chr28	11878380	2	310	C:0.806452	T:0.193548	
chr28	11878898	2	304	TC:0.634868	T:0.365132	
chr28	11878899	2	304	C:0.822368	T:0.177632	
chr28	11878981	2	302	A:0.94702	G:0.0529801	
chr28	11879372	2	300	G:0.473333	A:0.526667	
chr28	11879468	2	300	T:0	C:1	
chr28	11879520	2	292	C:0	T:1	
chr28	11879527	2	292	C:0	T:1	
chr28	11879532	2	292	C:0	T:1	
chr28	11879539	2	292	TG:0	T:1	
chr28	11879542	2	292	G:0	GT:1	
chr28	11879590	2	284	G:0	T:1	
chr28	11879696	2	212	A:0.962264	G:0.0377358	
chr28	11879839	2	280	TA:0.825	T:0.175	
chr28	11879919	2	306	C:0.816993	T:0.183007	
chr28	11880140	2	312	T:0.439103	G:0.560897	
chr28	11880412	2	306	AG:0	A:1	
chr28	11880549	2	302	AT:0.933775	A:0.0662252	
chr28	11880555	2	304	T:0.430921	G:0.569079	
chr28	11881779	2	288	G:0.774306	A:0.225694	
chr28	11881837	2	296	C:0.72973	T:0.27027	
chr28	11882344	2	308	G:0.737013	T:0.262987	
chr28	11882615	2	300	T:0.693333	C:0.306667	
chr28	11883074	2	306	G:0.957516	C:0.0424837	
chr28	11883409	2	306	G:0.722222	A:0.277778	
chr28	11883866	2	302	G:0.970199	T:0.0298013	
chr28	11883981	2	312	T:0.695513	A:0.304487	
chr28	11884006	2	304	G:0.973684	T:0.0263158	
chr28	11884147	2	312	G:0.807692	A:0.192308	
chr28	11884208	2	312	C:0.945513	T:0.0544872	
chr28	11884563	2	298	A:0.402685	G:0.597315	
chr28	11885008	2	310	G:0.735484	A:0.264516	
chr28	11885048	2	308	C:0.980519	T:0.0194805	
chr28	11885990	2	298	C:0.714765	T:0.285235	
chr28	11886152	2	256	GAT:0.871094	G:0.128906	
chr28	11886155	2	256	C:0.871094	CA:0.128906	
chr28	11886315	2	296	ATTT:0.763514	A:0.236486	
chr28	11886318	3	296	T:0.506757	A:0.256757	TAAAAAAAAAAAAA:0.236486
chr28	11886414	2	298	G:0.687919	A:0.312081	
chr28	11886615	2	310	G:0.412903	T:0.587097	
chr28	11887045	2	306	T:0.477124	TA:0.522876	
chr28	11887409	2	314	T:0.815287	C:0.184713	

chr28	11887582	2	306	G:0.75817	A:0.24183		
chr28	11887973	2	310	C:0.96129	T:0.0387097		
chr28	11888154	2	308	C:0.483766	T:0.516234		
chr28	11888210	2	312	G:0.980769	A:0.0192308		
chr28	11888556	2	298	C:0.812081	G:0.187919		
chr28	11888695	2	306	C:0.941176	A:0.0588235		
chr28	11889398	2	274	C:0.759124	T:0.240876		
chr28	11889609	2	300	G:0.973333	A:0.0266667		
chr28	11890634	2	300	G:0.996667	T:0.00333333		
chr28	11890884	2	312	A:0.932692	G:0.0673077		
chr28	11891477	2	306	C:0.493464	G:0.506536		
chr28	11891523	2	308	A:0.961039	G:0.038961		
chr28	11891537	2	308	C:0.782468	T:0.217532		
chr28	11892124	2	312	G:0.983974	C:0.0160256		
chr28	11892190	2	312	C:0.926282	T:0.0737179		
chr28	11892692	2	314	AG:0.977707	A:0.022293		
chr28	11892694	2	314	C:0.977707	T:0.022293		
chr28	11893064	2	312	A:0.769231	G:0.230769		
chr28	11893084	2	312	G:0.878205	C:0.121795		
chr28	11893095	2	310	C:0.929032	A:0.0709677		
chr28	11893182	2	304	GA:0.444079	G:0.555921		
chr28	11893201	2	306	G:0.470588	A:0.529412		
chr28	11893257	2	304	A:0.473684	G:0.526316		
chr28	11893458	2	302	G:0.798013	C:0.201987		
chr28	11893603	2	306	C:0.918301	T:0.0816993		
chr28	11894628	2	302	C:0.460265	T:0.539735		
chr28	11895309	2	306	T:0.382353	C:0.617647		
chr28	11896364	2	310	C:0.935484	T:0.0645161		
chr28	11897117	2	310	TCCTAAGC:0.751613	T:0.248387		
chr28	11897146	2	306	A:0.843137	G:0.156863		
chr28	11897231	2	304	T:0.496711	G:0.503289		
chr28	11897412	2	296	AT:0.966216	A:0.0337838		
chr28	11897471	2	300	C:0.843333	T:0.156667		
chr28	11897491	2	300	A:0.843333	C:0.156667		
chr28	11897718	2	282	G:0.851064	A:0.148936		
chr28	11897838	2	302	A:0.145695	T:0.854305		
chr28	11898077	2	268	C:0.865672	T:0.134328		
chr28	11898115	2	266	C:0.902256	T:0.0977444		
chr28	11898121	2	310	AATATATATATATATATAT:0.212903	A:0.787097		
chr28	11898593	2	248	ATG:0.774194	A:0.225806		
chr28	11898597	3	300	G:0.62	GTA:0.2	GTATA:0.18	
chr28	11898767	2	306	G:0.784314	A:0.215686		
chr28	11898859	2	296	G:0.831081	A:0.168919		
chr28	11899659	2	310	G:0.774194	A:0.225806		
chr28	11899683	2	304	A:0.9375	G:0.0625		
chr28	11899909	2	302	A:0.956954	T:0.0430464		
chr28	11900076	2	306	G:0.882353	A:0.117647		
chr28	11900354	2	300	A:0.99	T:0.01		
chr28	11900702	2	300	C:0.843333	CT:0.156667		
chr28	11900739	2	300	CT:0.946667	C:0.0533333		
chr28	11900952	4	296	GAAA:0.307432	G:0.121622	GAA:0.243243	GAAAAA:0.327703

chr28	11900971	2	288	A:0.885417	T:0.114583		
chr28	11901038	2	284	T:0.524648	G:0.475352		
chr28	11901103	4	292	C:0.636986	CCT:0.195205	CCTCT:0.0719178	CCTCTCT:0.0958904
chr28	11901139	2	290	AAAT:0.882759	A:0.117241		
chr28	11901142	2	288	T:0.944444	TA:0.0555556		
chr28	11901145	2	296	A:0.935811	ATT:0.0641892		
chr28	11901490	2	304	G:0.957237	C:0.0427632		
chr28	11901529	2	310	G:0.948387	A:0.0516129		
chr28	11901609	2	304	G:0.493421	A:0.506579		
chr28	11901648	2	302	T:0.0728477	C:0.927152		
chr28	11901796	2	314	C:0.490446	T:0.509554		
chr28	11901801	2	314	A:0.156051	T:0.843949		
chr28	11902561	2	306	G:0.683007	A:0.316993		
chr28	11902834	2	310	A:0.154839	G:0.845161		
chr28	11902875	2	318	T:0.767296	TTTTCTTTC:0.232704		
chr28	11903577	2	300	G:0.976667	A:0.0233333		
chr28	11904397	2	302	A:0.572848	G:0.427152		
chr28	11904411	2	306	G:0.732026	GA:0.267974		
chr28	11904423	2	306	T:0.732026	TG:0.267974		
chr28	11904429	2	300	A:0.37	G:0.63		
chr28	11904966	2	300	T:0.76	G:0.24		
chr28	11905046	2	316	A:0.737342	G:0.262658		
chr28	11905088	2	312	A:0.926282	AT:0.0737179		
chr28	11905201	2	294	C:0.119048	G:0.880952		
chr28	11905204	2	294	C:0.748299	T:0.251701		
chr28	11905287	2	300	T:0.41	A:0.59		
chr28	11905538	2	304	G:0.371711	A:0.628289		
chr28	11905562	2	312	C:0.753205	A:0.246795		
chr28	11905567	2	312	G:0.753205	A:0.246795		
chr28	11905695	2	308	T:0.948052	A:0.0519481		
chr28	11905737	2	306	GA:0.947712	G:0.0522876		
chr28	11905896	2	308	AAC:0.75	A:0.25		
chr28	11905908	2	304	C:0.404605	A:0.595395		
chr28	11906048	2	308	G:0.694805	C:0.305195		
chr28	11906064	2	308	T:0.103896	G:0.896104		
chr28	11906235	2	294	C:0.683673	A:0.316327		
chr28	11906252	2	300	A:0.68	T:0.32		
chr28	11906403	2	306	A:0.931373	G:0.0686275		
chr28	11906470	2	314	A:0.684713	G:0.315287		
chr28	11906582	2	302	G:0.950331	C:0.0496689		
chr28	11906587	2	300	A:0.71	G:0.29		
chr28	11906709	2	296	A:0.689189	G:0.310811		
chr28	11906993	2	310	T:0.687097	C:0.312903		
chr28	11907074	2	308	T:0.678571	TC:0.321429		
chr28	11907188	2	298	C:0.959732	T:0.0402685		
chr28	11907338	2	308	AC:0.672078	A:0.327922		
chr28	11907340	2	308	A:0.672078	T:0.327922		
chr28	11907516	2	312	T:0.967949	C:0.0320513		
chr28	11907594	2	302	G:0.417219	A:0.582781		
chr28	11907854	2	300	GAAC:0.123333	G:0.876667		
chr28	11908150	2	310	T:0.870968	A:0.129032		
chr28	11908333	2	312	A:0.294872	G:0.705128		

chr28	11908483	2	308	A:0.701299	C:0.298701	
chr28	11908607	2	310	A:0.796774	G:0.203226	
chr28	11908661	2	304	CT:0.973684	C:0.0263158	
chr28	11908670	2	304	TA:0.786184	T:0.213816	
chr28	11908671	2	302	A:0.844371	T:0.155629	
chr28	11908792	2	296	C:0.787162	T:0.212838	
chr28	11908821	2	302	C:0.794702	G:0.205298	
chr28	11908827	2	302	C:0.794702	T:0.205298	
chr28	11909091	2	298	C:0.791946	T:0.208054	
chr28	11909359	2	302	G:0.81457	GTGAA:0.18543	
chr28	11909966	2	306	TGA:0.614379	T:0.385621	
chr28	11910739	2	318	C:0.562893	CAAATATAAAAAAATATAAAAAAA:0.437107	
chr28	11910742	2	318	C:0.562893	CAAAAAAT:0.437107	
chr28	11910910	2	312	G:0.310897	C:0.689103	
chr28	11911220	2	312	CTG:0.935897	C:0.0641026	
chr28	11911433	2	300	G:0.313333	T:0.686667	
chr28	11911657	2	302	C:0.970199	T:0.0298013	
chr28	11912001	3	290	CTT:0.562069	C:0.0724138	CT:0.365517
chr28	11912002	2	304	T:0.759868	TC:0.240132	
chr28	11912028	2	286	C:0.304196	T:0.695804	
chr28	11912031	2	286	T:0.304196	C:0.695804	
chr28	11912299	2	320	C:0.95625	T:0.04375	
chr28	11912379	2	320	G:0.896875	GTC:0.103125	
chr28	11912395	2	320	C:0.984375	G:0.015625	
chr28	11912418	2	318	TAAA:0.893082	T:0.106918	
chr28	11912629	2	304	A:0.694079	G:0.305921	
chr28	11912867	2	308	T:0.961039	C:0.038961	
chr28	11912928	2	306	A:0.728758	C:0.271242	
chr28	11912964	2	306	T:0.156863	TTGA:0.843137	
chr28	11913368	2	296	T:0.314189	A:0.685811	
chr28	11913502	2	306	T:0.303922	A:0.696078	
chr28	11913526	2	310	C:0.303226	T:0.696774	
chr28	11913869	2	304	C:0.694079	T:0.305921	
chr28	11914247	2	306	A:0.313725	T:0.686275	
chr28	11914547	2	298	CTTT:0.748322	C:0.251678	
chr28	11914566	3	298	TAAG:0.634228	T:0.107383	AAAG:0.258389
chr28	11914575	2	286	T:0.311189	C:0.688811	
chr28	11914582	2	286	T:0.311189	TAGAC:0.688811	
chr28	11914621	2	298	C:0.724832	G:0.275168	
chr28	11914627	2	298	T:0.724832	C:0.275168	
chr28	11914642	2	298	C:0.724832	G:0.275168	
chr28	11914711	2	304	C:0.907895	T:0.0921053	
chr28	11915125	2	300	G:0.273333	A:0.726667	
chr28	11915222	2	314	CTCTTACTCTTAGACCT:0.318471	C:0.681529	
chr28	11915267	2	304	T:0.355263	A:0.644737	
chr28	11915465	2	308	A:0.305195	G:0.694805	
chr28	11915522	2	300	G:0.993333	T:0.00666667	
chr28	11915639	2	300	G:0.266667	A:0.733333	
chr28	11915812	2	310	G:0.290323	A:0.709677	
chr28	11915856	2	304	T:0.766447	A:0.233553	

chr28	11916054	2	280	A:0.296429	G:0.703571	
chr28	11916275	2	278	G:0.97482	A:0.0251799	
chr28	11916371	2	290	T:0.403448	G:0.596552	
chr28	11916624	2	304	C:0.944079	T:0.0559211	
chr28	11916653	2	308	T:0.961039	C:0.038961	
chr28	11916841	2	294	A:0.965986	G:0.0340136	
chr28	11918348	2	164	G:0.542683	A:0.457317	
chr28	11918457	2	262	TA:0.736641	T:0.263359	
chr28	11918523	2	290	G:0.724138	GTT:0.275862	
chr28	11919089	2	306	C:0.892157	T:0.107843	
chr28	11919381	2	270	A:0.374074	G:0.625926	
chr28	11919506	3	312	GGTGTGTGTGT:0.471154	G:0.282051	GGTGTGTGTGTGT:0.246795
chr28	11920706	2	312	CT:0.801282	C:0.198718	
chr28	11921527	2	296	G:0.773649	A:0.226351	
chr28	11922160	2	304	GA:0.990132	G:0.00986842	
chr28	11922375	2	296	A:0.702703	T:0.297297	
chr28	11922483	2	312	C:0.971154	T:0.0288462	
chr28	11922543	2	302	C:0.950331	T:0.0496689	
chr28	11922905	2	304	A:0.967105	G:0.0328947	
chr28	11923553	2	304	G:0.378289	C:0.621711	
chr28	11924130	2	310	A:0.73871	G:0.26129	
chr28	11924200	2	290	G:0.827586	GA:0.172414	
chr28	11924543	3	300	T:0.636667	TA:0.166667	TAA:0.196667
chr28	11924749	2	298	C:0.95302	T:0.0469799	
chr28	11924955	2	304	G:0.960526	C:0.0394737	
chr28	11925141	2	306	C:0.820261	T:0.179739	
chr28	11925145	2	306	C:0.820261	T:0.179739	
chr28	11925403	2	294	T:0.823129	G:0.176871	
chr28	11925465	3	298	TAC:0.33557	T:0.416107	TACAC:0.248322
chr28	11925618	2	298	GTA:0.825503	G:0.174497	
chr28	11926540	2	308	C:0.964286	T:0.0357143	
chr28	11926790	2	292	ATAT:0.808219	A:0.191781	
chr28	11927064	2	300	A:0.94	G:0.06	
chr28	11927120	2	302	C:0.976821	A:0.0231788	
chr28	11927127	2	302	G:0.976821	A:0.0231788	
chr28	11927537	2	302	A:0.89404	T:0.10596	
chr28	11927971	2	304	A:0.766447	G:0.233553	
chr28	11928193	3	304	GA:0.707237	G:0.0953947	GAAAA:0.197368
chr28	11928202	2	304	A:0.572368	G:0.427632	
chr28	11928669	2	306	C:0.993464	T:0.00653595	
chr28	11929228	2	306	A:0.767974	G:0.232026	
chr28	11929501	2	288	A:0.295139	T:0.704861	
chr28	11929565	2	206	T:0.291262	TG:0.708738	
chr28	11929912	2	294	A:0.445578	T:0.554422	
chr28	11930389	2	296	G:0.976351	C:0.0236486	
chr28	11930492	2	290	A:0.72069	G:0.27931	
chr28	11930545	2	286	G:0.143357	GAATA:0.856643	
chr28	11930585	2	294	CT:0.928571	C:0.0714286	
chr28	11930846	2	310	T:0.990323	C:0.00967742	
chr28	11931229	2	302	C:0.983444	T:0.0165563	

chr28 11931810 2 304 A:0.947368 G:0.0526316
chr28 11931814 2 308 C:0.792208 CTAAA:0.207792
chr28 11932298 2 310 G:0.303226 T:0.696774
chr28 11932598 2 306 GTATATATATATATATATATATATATATATATA:
0.535948 G:0.464052
chr28 11932897 2 228 G:0.403509 A:0.596491
chr28 11933330 2 310 A:0.996774 AAAAT:0.00322581
chr28 11933512 2 306 G:0.271242 GT:0.728758
chr28 11933888 2 310 T:0.13871 C:0.86129
chr28 11934128 2 294 C:0.765306 T:0.234694
chr28 11934241 2 306 T:0.70915 TC:0.29085
chr28 11934242 5 316 T:0.449367 C:0.275316
TCTCTCTC:0.129747 TCTCTCTCTCTCTC:0.0537975 TCTCTCTCTCTCTCTC:
0.0917722
chr28 11934544 2 304 CAG:0.743421 C:0.256579
chr28 11934741 2 304 G:0.75 A:0.25
chr28 11935044 2 294 T:0.404762 A:0.595238
chr28 11935286 2 312 C:0.820513 CA:0.179487
chr28 11935773 2 296 CCTCTCTCT:0.317568 C:
0.682432
chr28 11936014 2 290 T:0.941379 TA:0.0586207
chr28 11936934 2 294 T:0.585034 TA:0.414966
chr28 11937093 2 290 G:0.837931 T:0.162069
chr28 11937180 2 300 G:0.793333 A:0.206667
chr28 11937363 2 312 T:0.977564 A:0.0224359
chr28 11937914 2 290 T:0.958621 TAA:0.0413793
chr28 11937922 2 306 A:0.96732 AT:0.0326797
chr28 11937923 2 306 A:0.954248 ATAT:0.0457516
chr28 11937925 3 306 A:0.797386 T:0.0555556
ATAT:0.147059
chr28 11937926 2 306 A:0.882353 AT:0.117647
chr28 11937927 4 306 A:0.261438 T:0.418301
AT:0.24183 ATAT:0.0784314
chr28 11937946 2 274 A:0.346715 G:0.653285
chr28 11938525 3 304 TA:0.5 T:0.398026
TTAAAAAAA:0.101974
chr28 11938606 2 282 G:0.950355 A:0.0496454
chr28 11938703 2 274 ATTTT:0.791971 A:0.208029
chr28 11938709 2 274 T:0.791971 TAAAAAA:0.208029
chr28 11938710 2 276 A:0.963768 T:0.0362319
chr28 11938757 5 310 AAAATAAATAAATAAATAAAT:0.26129
A:0.196774 AAAAT:0.0419355 AAAATAAAT:0.380645
AAAATAAATAAAT:0.119355
chr28 11938798 2 312 AAAT:0.980769 A:0.0192308
chr28 11938805 2 312 TAAATAAATAAAATA:0.980769 T:
0.0192308
chr28 11940234 2 306 C:0.104575 G:0.895425
chr28 11940662 2 308 T:0.948052 A:0.0519481
chr28 11941067 2 258 G:0.953488 A:0.0465116
chr28 11941333 3 278 GA:0.55036 G:0.21223
GAA:0.23741
chr28 11941949 3 302 CT:0.546358 C:0.370861
CTT:0.0827815
chr28 11942027 2 310 T:0.825806 A:0.174194

chr28	11942083	2	308	C:0.808442	CT:0.191558	
chr28	11942208	2	302	A:0.966887	G:0.0331126	
chr28	11942504	2	312	T:0.990385	C:0.00961538	
chr28	11943099	2	302	G:0.933775	A:0.0662252	
chr28	11943182	2	302	T:0.596026	C:0.403974	
chr28	11943470	3	292	C:0.510274	CT:0.386986	CTT:0.10274
chr28	11943775	2	312	T:0.964744	C:0.0352564	
chr28	11943783	2	314	G:0.993631	T:0.00636943	
chr28	11944716	3	318	C:0.789308	CTATT:0.185535	CTATTTATT:0.0251572
chr28	11944733	2	314	TA:0.538217	T:0.461783	
chr28	11944735	2	314	TTTATTTA:0.538217	T:0.461783	
chr28	11945088	2	300	A:0.956667	T:0.0433333	
chr28	11945937	2	302	G:0.993377	A:0.00662252	
chr28	11946538	2	314	G:0.509554	A:0.490446	
chr28	11946751	2	300	T:0.946667	TG:0.0533333	
chr28	11946778	2	304	A:0.805921	G:0.194079	
chr28	11946871	2	302	C:0.950331	T:0.0496689	
chr28	11947351	2	300	C:0.873333	T:0.126667	
chr28	11947671	2	314	C:0.821656	T:0.178344	
chr28	11947690	2	314	T:0.821656	TC:0.178344	
chr28	11948105	2	292	T:0.825342	G:0.174658	
chr28	11948372	2	292	G:0.80137	C:0.19863	
chr28	11948644	2	308	T:0.964286	C:0.0357143	
chr28	11949722	2	302	C:0.761589	T:0.238411	
chr28	11949766	2	304	G:0.388158	A:0.611842	
chr28	11952122	2	304	TA:0.990132	T:0.00986842	
chr28	11952170	2	308	A:0.996753	T:0.00324675	
chr28	11953056	2	310	A:0.841935	G:0.158065	
chr28	11953367	2	300	A:0.966667	G:0.0333333	
chr28	11953661	2	296	T:0	A:1	
chr28	11953692	2	288	AGATAGAT:0.986111	A:0.0138889	
chr28	11953710	2	304	AGAT:0.894737	A:0.105263	
chr28	11953926	2	264	TGGGGG:0	T:1	
chr28	11953931	2	264	G:0	GTCATT:1	
chr28	11953980	2	282	C:0	G:1	
chr28	11954267	2	300	GT:0.473333	G:0.526667	
chr28	11954676	3	316	C:0.509494	CACAT:0.240506	CACACAT:0.25
chr28	11956227	2	304	T:0.865132	TCC:0.134868	
chr28	11957195	2	298	A:0.97651	G:0.0234899	
chr28	11957224	2	292	G:0.684932	A:0.315068	
chr28	11957389	2	282	GTC:0.801418	G:0.198582	
chr28	11957443	2	296	TA:0.949324	T:0.0506757	
chr28	11957444	2	298	A:0.949664	T:0.0503356	
chr28	11958561	2	310	C:0.96129	T:0.0387097	
chr28	11958875	2	306	A:0.281046	G:0.718954	
chr28	11959680	2	308	T:0.0811688	C:0.918831	
chr28	11959744	2	298	C:0.828859	T:0.171141	
chr28	11960152	2	314	C:0.980892	A:0.0191083	
chr28	11960153	2	314	A:0.980892	G:0.0191083	

chr28 11960190 2 306 G:0.539216 C:0.460784
chr28 11960659 2 306 T:0.95098 C:0.0490196
chr28 11960831 2 308 C:0.324675 T:0.675325
chr28 11961189 4 316 TAAAA:0.196203 T:0.376582
TA:0.243671 TAA:0.183544
chr28 11961209 2 308 C:0.844156 T:0.155844
chr28 11961560 2 304 A:0.970395 T:0.0296053
chr28 11961614 2 302 T:0.963576 C:0.0364238
chr28 11962378 2 306 A:0.954248 G:0.0457516
chr28 11962816 2 294 G:0.540816 T:0.459184
chr28 11963058 2 308 A:0.996753 T:0.00324675
chr28 11963604 2 304 G:0.657895 T:0.342105
chr28 11964129 2 316 CCCAACAGT:0.987342 C:
0.0126582
chr28 11964386 3 304 G:0.621711 GA:0.319079
GAA:0.0592105
chr28 11964876 2 306 A:0.797386 G:0.202614
chr28 11965104 2 308 C:0.694805 CA:0.305195
chr28 11966279 2 302 C:0.811258 T:0.188742
chr28 11966965 2 306 T:0.947712 C:0.0522876
chr28 11967211 2 310 G:0.958065 A:0.0419355
chr28 11967480 2 308 C:0.470779 G:0.529221
chr28 11967512 2 316 T:0.674051 C:0.325949
chr28 11967802 2 296 T:0.942568 TA:0.0574324
chr28 11967808 2 296 A:0.131757 AT:0.868243
chr28 11967885 2 300 C:0.793333 T:0.206667
chr28 11968174 2 294 G:0.986395 A:0.0136054
chr28 11968322 2 302 C:0.0860927 T:0.913907
chr28 11968856 4 310 TAAA:0.403226 T:0.0741935
TA:0.383871 TAA:0.13871
chr28 11969669 2 294 T:0.734694 TTC:0.265306
chr28 11970830 3 294 G:0.193878 T:0.697279
GT:0.108844
chr28 11971952 2 308 T:0.12987 A:0.87013
chr28 11972207 2 310 CT:0.709677 C:0.290323
chr28 11972301 2 296 G:0.797297 A:0.202703
chr28 11973488 2 298 T:0.137584 A:0.862416
chr28 11973621 2 312 C:0.961538 T:0.0384615
chr28 11974765 6 314 TTTTA:0.264331 T:0.0700637
TTTTATTTA:0.191083 TTTTATTTATTTA:0.280255
TTTTATTTATTTATTTA:0.0796178 TTTTATTTATTTATTTATTTA:0.11465
chr28 11974832 2 288 C:0.701389 T:0.298611
chr28 11975354 3 316 CCTCTCT:0.64557 C:0.287975
CCTCTCTCT:0.0664557
chr28 11975970 2 308 TAA:0.652597 T:0.347403
chr28 11976288 2 300 C:0.65 T:0.35
chr28 11976388 2 266 C:0.842105 G:0.157895
chr28 11976416 2 282 GA:0.833333 G:0.166667
chr28 11977267 2 296 A:0.976351 T:0.0236486
chr28 11977593 2 292 TG:0.94863 T:0.0513699
chr28 11977594 2 292 G:0.520548 T:0.479452
chr28 11977941 2 280 G:0.657143 GCT:0.342857
chr28 11978498 2 302 C:0.966887 T:0.0331126
chr28 11978698 2 306 C:0.95098 G:0.0490196

chr28	11979036	2	302	C:0	T:1		
chr28	11979037	2	302	T:0	A:1		
chr28	11979045	2	302	G:0	A:1		
chr28	11979095	3	318	C:0	A:0.31761	G: 0.68239	
chr28	11979096	2	318	G:0	A:1		
chr28	11979117	2	318	C:0	A:1		
chr28	11979120	2	318	TG:0	T:1		
chr28	11979122	2	318	T:0	A:1		
chr28	11979124	2	318	T:0	TA:1		
chr28	11979128	2	318	G:0	GA:1		
chr28	11979294	2	278	G:0.658273	A:0.341727		
chr28	11979403	2	308	A:0.987013	T:0.012987		
chr28	11979410	2	306	T:0.418301	TA:0.581699		
chr28	11979466	2	308	A:0.948052	G:0.0519481		
chr28	11979565	2	292	T:0.969178	G:0.0308219		
chr28	11979783	2	304	GA:0.983553	G:0.0164474		
chr28	11979986	2	308	T:0.896104	TA:0.103896		
chr28	11980824	2	308	T:0.853896	A:0.146104		
chr28	11981157	2	296	A:0.831081	G:0.168919		
chr28	11981193	2	302	T:0.354305	C:0.645695		
chr28	11981412	2	272	C:0.849265	A:0.150735		
chr28	11981521	2	248	AT:0.633065	A:0.366935		
chr28	11981630	2	168	G:0.880952	A:0.119048		
chr28	11981868	2	276	T:0.963768	C:0.0362319		
chr28	11981921	2	298	G:0.973154	A:0.0268456		
chr28	11982155	2	306	C:0.98366	T:0.0163399		
chr28	11982863	2	214	TA:0.542056	T:0.457944		
chr28	11982967	2	298	T:0.385906	G:0.614094		
chr28	11983015	2	278	C:0.964029	T:0.0359712		
chr28	11984094	4	300	TA:0.456667	T:0.0633333	TAA:0.22	TAAA:0.26
chr28	11984180	2	296	A:0.85473	T:0.14527		
chr28	11984232	2	268	TA:0.764925	T:0.235075		
chr28	11984843	2	306	T:0.683007	C:0.316993		
chr28	11985322	4	316	T:0.626582	TACACAC:0.167722	TACACACACACAC:0.0759494	TACACACACACACAC:0.129747
chr28	11985341	2	310	A:0.967742	ACACACG:0.0322581		
chr28	11986832	2	304	C:0.493421	T:0.506579		
chr28	11986972	2	314	T:0.468153	G:0.531847		
chr28	11987107	2	304	C:0.677632	T:0.322368		
chr28	11987193	2	310	C:0.980645	CT:0.0193548		
chr28	11987203	3	310	TAA:0.819355	AAA:0.145161	T:0.0354839	
chr28	11987204	2	306	A:0.27451	T:0.72549		
chr28	11987205	3	310	A:0.619355	T:0.345161	ATTT:0.0354839	
chr28	11987394	2	278	G:0.485612	A:0.514388		
chr28	11987565	2	304	C:0.661184	G:0.338816		
chr28	11987567	2	306	G:0.526144	C:0.473856		
chr28	11987569	2	306	T:0.0816993	G:0.918301		
chr28	11987614	3	306	A:0.117647	AC:0.803922	AAC:0.0784314	
chr28	11987738	2	296	G:0.516892	A:0.483108		

chr28	11987792	2	292	C:0.94863	T:0.0513699
chr28	11987831	2	292	CA:0.534247	C:0.465753
chr28	11987838	2	296	AG:0.976351	A:0.0236486
chr28	11987939	2	310	G:0.5	A:0.5
chr28	11988358	2	306	T:0.519608	A:0.480392
chr28	11988666	2	304	C:0.5	T:0.5
chr28	11989396	2	304	C:0.121711	T:0.878289
chr28	11989706	2	312	T:0.778846	C:0.221154
chr28	11989779	2	310	C:0.235484	T:0.764516
chr28	11989837	2	306	G:0.839869	A:0.160131
chr28	11989917	2	308	A:0.243506	G:0.756494
chr28	11990163	2	304	G:0.822368	A:0.177632
chr28	11990325	2	310	T:0.935484	G:0.0645161
chr28	11990723	2	306	TTGAC:0.964052	T:0.0359477
chr28	11990918	2	312	TTGTC:0.948718	T:0.0512821
chr28	11990954	2	308	A:0.954545	T:0.0454545
chr28	11991092	2	298	C:0.755034	G:0.244966
chr28	11991267	2	306	C:0.980392	T:0.0196078
chr28	11991290	2	304	T:0.973684	C:0.0263158
chr28	11991416	2	302	G:0.241722	A:0.758278
chr28	11991771	2	316	C:0.227848	T:0.772152
chr28	11992173	2	298	A:0.0604027	G:0.939597
chr28	11992225	2	292	TA:0.84589	T:0.15411
chr28	11992457	2	310	T:0.783871	C:0.216129
chr28	11992471	2	312	CT:0.955128	C:0.0448718
chr28	11992489	2	300	C:0.96	A:0.04
chr28	11992545	2	308	G:0.993506	A:0.00649351
chr28	11992674	2	298	GA:0.187919	G:0.812081
chr28	11993152	2	308	A:0.0974026	G:0.902597
chr28	11993700	2	306	A:0.0555556	G:0.944444
chr28	11993701	2	308	A:0.951299	G:0.0487013
chr28	11994118	2	316	A:0.981013	T:0.0189873
chr28	11994154	2	316	G:0.724684	A:0.275316
chr28	11995746	2	306	T:0.941176	C:0.0588235
chr28	11995875	2	284	T:0.947183	A:0.0528169
chr28	11996015	2	308	C:0.938312	T:0.0616883
chr28	11996842	2	302	T:0.182119	C:0.817881
chr28	11996881	2	308	C:0.977273	T:0.0227273
chr28	11996935	2	304	G:0.0559211	T:0.944079
chr28	11997298	2	288	G:0.9375	GA:0.0625
chr28	11997605	2	304	C:0.976974	T:0.0230263
chr28	11997790	2	302	G:0.930464	GA:0.0695364
chr28	11997824	2	294	C:0.996599	G:0.00340136
chr28	11998542	2	300	C:0.0566667	CT:0.943333
chr28	11998797	2	298	G:0.0503356	C:0.949664
chr28	11998803	2	298	G:0.902685	A:0.0973154
chr28	11999041	2	308	G:0.951299	A:0.0487013
chr28	11999424	2	306	G:0.977124	T:0.0228758
