# Supplementary material for: Genome-Wide Analyses Suggest Mechanisms Involving Early B-Cell Development in Canine IgA Deficiency
Source: PLoS One. 2015 Jul 30;10(7):e0133844. doi: 10.1371/journal.pone.0133844 (PMC4520476; doi:10.1371/journal.pone.0133844)
Supplement: S17 Table — (PDF) [file pone.0133844.s027.pdf]

Table S17: Coordinates in canfam2.0, hg18 and canfam3 for all IgA associated regions for GRAIL and INRICH analyses

	canfam2.0			hg18			canfam3		
region_id*	chr	start	stop	chr	start	stop	chr	start	stop
gsp1	28	13444314	16073744	10	98864730	102081284	28	10446800	13077479
gsp2	28	21775320	21875320	10	108502194	108635312	28	18773291	18873291
gsp3	28	12856831	12991728	10	98214849	98361838	28	9859408	9994305
gsp4	28	10681718	10801858	10	95146745	95318062	28	7683212	7803352
gsp5	28	8931432	9031432	10	93083749	93209420	28	5933856	6033856
gsp6	4	34328856	34428856	10	84273940	84379019	4	31118913	31218913
gsp7	7	18466863	18566863	1	180325372	180442510	7	15505664	15605664
gsp8	7	19106802	19206802	1	180987985	181085258	7	16146250	16246197
gsp9	7	20699277	20799277	1	182736880	182843787	7	17736665	17836665
gsp10	7	22587896	22687896	1	184854786	184988218	7	19623829	19723829
gsp11	7	25683140	25783140	1	175030853	175136140	7	22714328	22814328
gsp12	8	19014736	19114736	14	37059446	37159929	8	16008011	16108075
gsp13	22	23796999	23896999	13	65129544	65275791	22	20848859	20949206
gsp14	24	44233381	44333381	20	53925151	54050713	24	41251998	41351998
gsp15	35	4931728	5031728	6	1438693	1531669	35	1931709	2031709
gsp16	35	11258142	11358142	6	8286608	8399749	35	8250009	8350115
ggsd1	5	9500347	11174145	11	125720557	127533615	5	6498684	8172621
ggsd6	8	66251422	66867233	14	93721279	94450755	8	63211755	63827575
ggsd7	10	72227420	72329612	2	70765977	70893781	10	69072184	69174348
ggsd8	14	45130092	45246967	7	29151909	29280089	14	42174407	42291282
ggsd9	16	48920635	49337432	4	185504021	185740373	16	45960356	46377153
ggsd11	23	51492483	51592483	3	155505284	155640373	23	48439474	48539474
ggsd12	27	40276517	40483033	12	7926708	8122868	27	37248047	37454652
ggsd13	5	32196812	32296812	11	101759107	101906384	5	29188112	29288253
ggr1	6	21024771	21124771	16	29893801	30037277	6	18011946	18112312
ggr2	21	49116056	49216056	11	24704154	24837668	21	45923013	46023013
ggr3	26	10131206	10865491	12	120221446	121289322	26	7114611	7846707
ggr4	28	40226068	40360530	10	130172844	130315432	28	37228122	37362478
ggr5	29	13811471	14805990	8	61420069	62567586	29	10808328	11803162
ggr6	34	24416647	25600000	3	190391367	191812849	34	21406221	22589723
ggr7	34	44349864	44757483	3	179227787	179685817	34	41343801	41751420
ggr8	34	13267919	13690838	5	2245032	2769961	34	10261397	10685205
glr1	23	27717862	27823499	3	18261917	18389612	23	24665511	24771148
glr2	23	45077859	45194536	3	148089336	148202105	23	42026107	42142835
glr3	30	20807660	20907660	15	50188546	50300642	30	17817239	17917239

** g=gwas region followed by breed.*
