# Supplementary material for: Genome-Wide Analyses Suggest Mechanisms Involving Early B-Cell Development in Canine IgA Deficiency
Source: PLoS One. 2015 Jul 30;10(7):e0133844. doi: 10.1371/journal.pone.0133844 (PMC4520476; doi:10.1371/journal.pone.0133844)
Supplement: S7 Fig — The proportion of variants with variation in 20 GSDs in sliding-windows (11 variants per window, 1 variant overlap) is presented in A. Panel B shows a distinct increase in the degree of genetic differentiation (FST) between dogs and wolves spanning a 75-kb region (windows with FST > 0.67 are coloured in black) within the SLIT1 locus. FST values of two consecutive 50-kb windows reached 0.68 and 0.67, respectively (windows with FST > 0.43 are coloured in grey). Additionally, panel B presents the positions of the top-associated SNPs in SP, black bars representing blocks of fixed variants in GSD (based on panel A) and the positions of CTCF transcription factor binding sites based on the human (hg19) Transcription Factor ChIP-seq from ENCODE V2 and the genes in the region. The corresponding region in human hg19 with the full track from Transcription Factor ChIP-seq from ENCODE V2 is presented in panel C. (PDF) [file pone.0133844.s007.pdf]

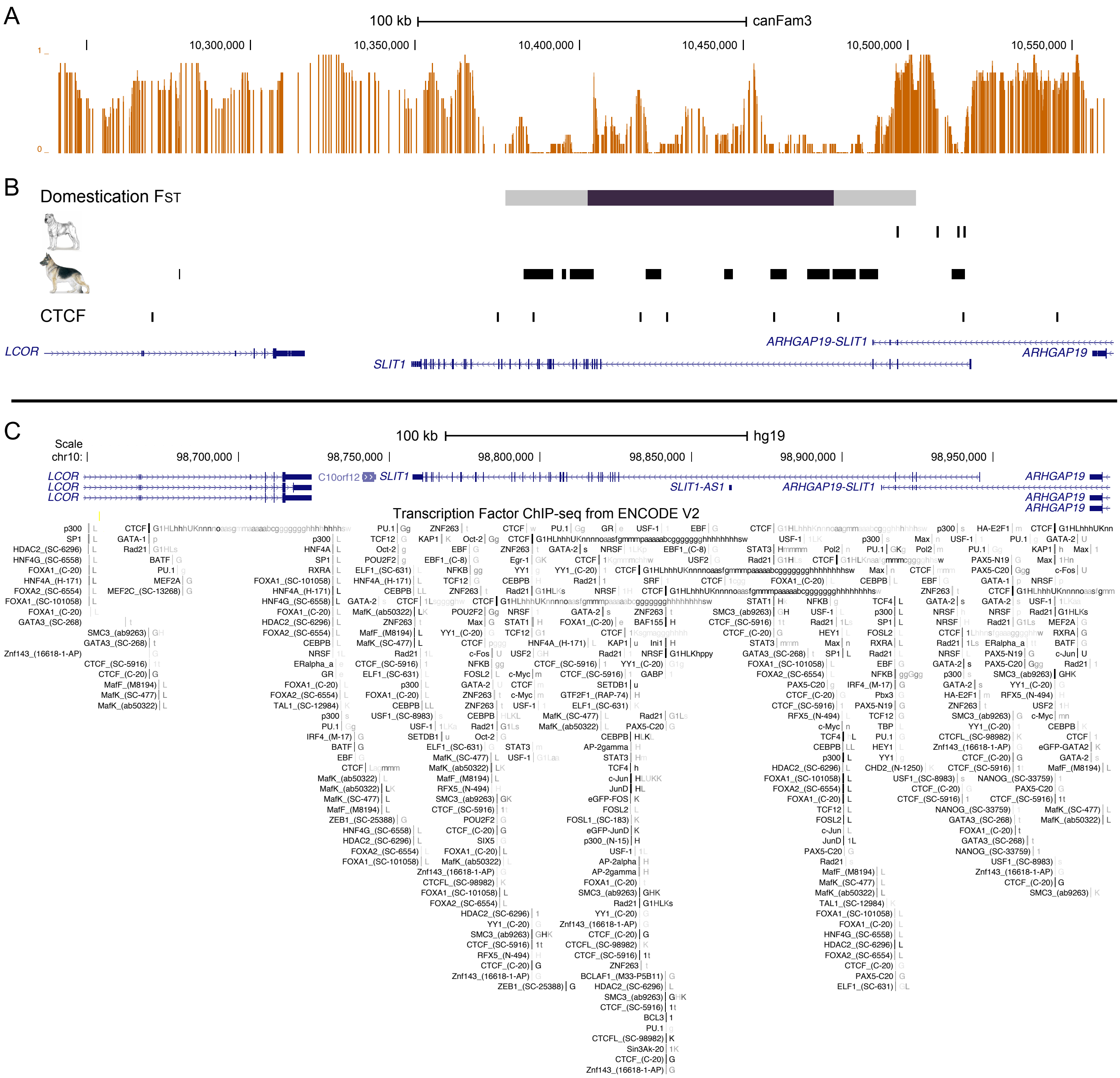
A
100 kb
canFam3
10,300,000
10,350,000
10,400,000
10,450,000
10,500,000
10,550,000
1
0
B
Domestication FST
CTCF
LCOR
ARHGAP19-SLIT1
ARHGAP19
SLIT1
C
100 kb
hg19
Scale
chr10:
98,700,000
98,750,000
98,800,000
98,850,000
98,900,000
98,950,000
LCOR
LCOR
LCOR
C10orf12
SLIT1
SLIT1-AS1
ARHGAP19-SLIT1
ARHGAP19
ARHGAP19
ARHGAP19
Transcription Factor ChIP-seq from ENCODE V2
